# Comprehensive annotation of secondary metabolite biosynthetic genes and gene clusters of *Aspergillus nidulans*, *A. fumigatus*, *A. niger* and *A. oryzae*

**DOI:** 10.1186/1471-2180-13-91

**Published:** 2013-04-26

**Authors:** Diane O Inglis, Jonathan Binkley, Marek S Skrzypek, Martha B Arnaud, Gustavo C Cerqueira, Prachi Shah, Farrell Wymore, Jennifer R Wortman, Gavin Sherlock

**Affiliations:** 1 Department of Genetics, Stanford University Medical School, Stanford, CA 94305-5120, USA; 2 Broad Institute, 7 Cambridge Center, Cambridge, MA 02141, USA

**Keywords:** *Aspergillus*, Gene clusters, Gene Ontology, Genome annotation, Secondary metabolism, Sybil

## Abstract

**Background:**

Secondary metabolite production, a hallmark of filamentous fungi, is an expanding area of research for the *Aspergilli.* These compounds are potent chemicals, ranging from deadly toxins to therapeutic antibiotics to potential anti-cancer drugs. The genome sequences for multiple *Aspergilli* have been determined, and provide a wealth of predictive information about secondary metabolite production. Sequence analysis and gene overexpression strategies have enabled the discovery of novel secondary metabolites and the genes involved in their biosynthesis. The *Aspergillus* Genome Database (AspGD) provides a central repository for gene annotation and protein information for *Aspergillus* species. These annotations include Gene Ontology (GO) terms, phenotype data, gene names and descriptions and they are crucial for interpreting both small- and large-scale data and for aiding in the design of new experiments that further *Aspergillus* research.

**Results:**

We have manually curated Biological Process GO annotations for all genes in AspGD with recorded functions in secondary metabolite production, adding new GO terms that specifically describe each secondary metabolite. We then leveraged these new annotations to predict roles in secondary metabolism for genes lacking experimental characterization. As a starting point for manually annotating *Aspergillus* secondary metabolite gene clusters, we used antiSMASH (antibiotics and Secondary Metabolite Analysis SHell) and SMURF (Secondary Metabolite Unknown Regions Finder) algorithms to identify potential clusters in *A. nidulans*, *A. fumigatus, A. niger* and *A. oryzae*, which we subsequently refined through manual curation.

**Conclusions:**

This set of 266 manually curated secondary metabolite gene clusters will facilitate the investigation of novel *Aspergillus* secondary metabolites.

## Background

Secondary metabolites produced by fungi are a rich source of medically useful compounds because of their pharmaceutical and toxicological properties [1]. While secondary metabolites are not required for an organism’s growth or primary metabolism, they may provide important benefits in its environmental niche. For example, *A. nidulans laeA* mutants defective in the production of secondary metabolites are ingested more readily by the fungivorous arthropod, *Folsomia candida*, suggesting that secondary metabolite production can protect fungi from predation [2].

The *Aspergilli* are producers of a wide variety of secondary metabolites of considerable medical, industrial, agricultural and economic importance. For example, the antibiotic penicillin is produced by *A. nidulans* and the genes involved in the penicillin biosynthetic pathway have been extensively studied [3-5]. Sterigmatocystin (ST), an aflatoxin (AF) precursor, and many of the genes that are involved in its biosynthesis have also been extensively studied in *A. nidulans*[6-10]. AF is a secondary metabolite produced mainly by *Aspergillus* species growing in foodstuffs [11], and it is of both medical and economic importance as contaminated food sources are toxic to humans and animals when ingested. Gliotoxin is an extremely toxic secondary metabolite produced by several *Aspergillus* species during infection [12,13]. The ability of this toxin to modulate the host immune system and induce apoptosis in a variety of cell-types has been most studied in the ubiquitous fungal pathogen, *A. fumigatus*[14,15].

The availability of *Aspergillus* genomic sequences has greatly facilitated the identification of numerous genes involved in the production of other secondary metabolites. Based on the number of predicted secondary metabolite biosynthesis genes and the fact that the expression of many secondary metabolite gene clusters is cryptic [16], meaning that expression is not evident under standard experimental conditions, there appears to be the potential for production of many more secondary metabolites than currently known [17]. Secondary metabolite biosynthetic genes often occur in clusters that tend to be sub-telomerically located and are coordinately regulated under certain laboratory conditions [18-20]. Typically, a secondary metabolite biosynthetic gene cluster contains a gene encoding one of several key “backbone” enzymes of the secondary metabolite biosynthetic process: a polyketide synthase (PKS), a non-ribosomal peptide synthetase (NRPS), a polyketide synthase/non-ribosomal peptide synthetase hybrid (PKS-NRPS), a prenyltransferase known as dimethylallyl tryptophan synthase (DMATS) and/or a diterpene synthase (DTS).

Comparative sequence analysis based on known backbone enzymes has been used to identify potential secondary metabolite biosynthetic gene clusters for subsequent experimental verification. One approach for experimental verification is the deletion of genes with suspected roles in secondary metabolite biosynthesis followed by identification of the specific secondary metabolite profiles of the mutants by thin layer chromatography, NMR or other methods [7,8]. For example, the deletion of *A. fumigatus encA*, which encodes an ortholog of the *A. nidulans* non-reducing PKS (NR-PKS) *mdpG*, followed by analysis of culture extracts using high-performance liquid chromatography (HPLC) enabled the recent identification of endocrocin and its biosynthetic pathway intermediates [21]. Similarly, the deletion of the gene encoding the PKS, *easB*, enabled the identification of the emericellamide biosynthetic pathway of *A. nidulans*[22]. Another approach is the overexpression of predicted transcriptional regulators of secondary metabolism gene clusters with subsequent analysis of the gene expression and secondary metabolite profiles of the resulting strains, which has facilitated the identification of numerous secondary metabolites and the genes responsible for their synthesis [23,24]. For example, overexpression of *laeA* in *A. nidulans*, a global transcriptional regulator of secondary metabolism production, coupled with microarray analysis, facilitated the delineation of the cluster responsible for production of the anti-tumor compound, terrequinone A [18]. Thus, genome sequence analysis, coupled with targeted experimentation, has been a highly effective strategy for identifying novel secondary metabolites and the genes involved in their synthesis.

The *Aspergillus* Genome Database (AspGD; http://www.aspgd.org) is a web-based resource that provides centralized access to gene and protein sequences, analysis tools and manually curated information derived from the published scientific literature for *A. nidulans*, *A. fumigatus*, *A. niger* and *A. oryzae*[25,26]. AspGD curators read the published experimental literature to record information including gene names and synonyms, write free-text descriptions of each gene, record phenotypes and assign terms that describe functional information about genes and proteins using the Gene Ontology (GO; http://www.geneontology.org). These annotations are an important resource for the scientific research community, used both for reference on individual genes of interest as well as for analysis of results from microarray, proteomic experiments, or other screens that produce large lists of genes.

The GO is a structured vocabulary for describing the functions associated with genes products [27]. GO terms describe the activity of a gene product (Molecular Function; MF) within the cell, the biological process (Biological Process; BP) in which a gene product is involved and the location within the cell (Cellular Component; CC) where the gene product is observed [28]. Evidence codes are assigned to GO annotations based on the type of available experimental evidence.

At the start of this project most of the terms needed to describe secondary metabolite biosynthetic genes or regulators of secondary metabolism did not yet exist in the GO. Thus, in order to provide an improved annotation of secondary metabolite biosynthetic genes and their regulatory proteins, we developed new GO terms for secondary metabolite production in collaboration with the GO Consortium, and reannotated the entire set of genes associated with secondary metabolism in AspGD. We then performed a comprehensive analysis of the secondary metabolism biosynthetic genes and their orthologs across the genomes of *A. nidulans*, *A. fumigatus*, *A. niger* and *A. oryzae* and now provide a set of manually annotated secondary metabolite gene clusters*.* We anticipate that these new, more precise annotations will encourage the rapid and efficient experimental verification of novel secondary metabolite biosynthetic gene clusters in *Aspergillus* and the identification of the corresponding secondary metabolites.

## Results

### Identifying genes for reannotation

Many branches of the GO, such as apoptosis and cardiac development [29], have recently been expanded and revised to include new terms that are highly specific to these processes. The secondary metabolism literature has expanded over the last several years, allowing AspGD curators to make annotations to an increasing number of genes with roles in secondary metabolism. During routine curation, it became apparent that hundreds of *Aspergillus* genes that were candidates for annotation to the GO term ‘secondary metabolic process’ had the potential for more granular annotations, since, in many cases, the specific secondary metabolite produced by a gene product is known. At the inception of this project, only terms for ‘aflatoxin biosynthetic process, ’ ‘penicillin biosynthetic process’ and ‘sterigmatocystin biosynthetic process, ’ the 3 most well-studied secondary metabolites to date, were present in the GO (Additional file 1).

Candidate genes for reannotation were identified as those that had pre-existing GO annotations to ‘secondary metabolic process’ or curated mutant phenotypes that impact secondary metabolite production. For example, numerous genes in AspGD are annotated with mutant phenotypes affecting the production of secondary metabolites such as asperthecin [30], austinol and dehydroaustinol [31], emericellin [32], fumiquinazolines [33], orsellinic acid [34], pseurotin A [35], shamixanthones [32,36] and violaceol [37] among others. These genes were then analyzed and a list of new GO terms was generated to annotate these genes more specifically (Table 1 and Additional file 1).

**Table 1:** **Number of *Aspergillus *genes with manual and computational GO annotations to ‘secondary metabolic process**’

**GO term ID**	**GO term name**	**Manual GO annotations**	**A. nidulans IEA annotations**	**A. fumigatus IEA annotations**	**A. niger IEA annotations**	**A. oryzae IEA annotations**	Total IEA annotations*
GO:1900596	(+)-kotanin biosynthetic process	1	1	1	0	1	3
GO:1900581	(17Z)-protosta-17(20),24-dien-3beta-ol biosynthetic process	1	0	0	0	0	0
GO:0045122	aflatoxin biosynthetic process (PT)	1	6	2	0	2	10
GO:1900587	arugosin biosynthetic process	1	0	3	2	1	6
GO:1900554	asperfuranone biosynthetic process	7	0	10	29	23	62
GO:0036184	asperthecin biosynthetic process	5	0	11	10	6	27
GO:1900560	austinol biosynthetic process	15	0	10	13	12	35
GO:1900805	brevianamide F biosynthetic process	1	2	0	4	4	10
GO:1900566	chanoclavine-I biosynthetic process	3	1	0	2	2	5
GO:1900563	dehydroaustinol biosynthetic process	16	0	11	14	13	38
GO:1900599	demethylkotanin biosynthetic process	1	1	1	0	1	2
GO:1900617	emericellamide A biosynthetic process	2	0	3	8	6	17
GO:1900557	emericellamide biosynthetic process	6	0	6	20	12	38
GO:1900575	emodin biosynthetic process	1	0	3	2	1	6
GO:1900602	endocrocin biosynthetic process	4	3	0	3	4	10
GO:0035837	ergot alkaloid biosynthetic process (PT)	5	6	0	4	7	17
GO:1900611	F-9775A biosynthetic process	3	0	0	0	0	0
GO:1900614	F-9775B biosynthetic process	3	0	0	0	0	0
GO:0031171	ferricrocin biosynthetic process	4	2	1	4	4	11
GO:1900809	fumigaclavine C biosynthetic process	2	2	0	1	1	4
GO:1900778	fumiquinazoline A biosynthetic process	2	2	0	2	2	6
GO:2001310	gliotoxin biosynthetic process	11	5	0	5	10	20
GO:0006583	melanin biosynthetic process from tyrosine	1	1	0	1	0	2
GO:1900815	monodictyphenone biosynthetic process	15	0	20	29	23	72
GO:1900551	N',N'',N'''-triacetylfusarinine C biosynthetic process	4	5	0	5	5	15
GO:1900787	naphtho-gamma-pyrone biosynthetic process	2	2	2	0	2	6
GO:1900584	o-orsellinic acid biosynthetic process	4	0	6	18	9	33
GO:1900821	orlandin biosynthetic process	1	1	1	0	1	3
GO:0042318	penicillin biosynthetic process (PT)	17	10	14	14	16	54
GO:0030639	polyketide biosynthetic process (PT)	18	0	0	1	0	1
GO:1900793	shamixanthone biosynthetic process	5	0	10	5	8	23
GO:0019290	siderophore biosynthetic process (PT)	5	3	0	4	3	10
GO:0045461	sterigmatocystin biosynthetic process (PT)	36	0	39	57	50	146
GO:1900605	tensidol A biosynthetic process	1	1	1	0	1	3
GO:1900796	terrequinone A biosynthetic process	1	0	0	0	1	1
GO:1900590	violaceol I biosynthetic process	2	0	1	2	4	7
GO:1900593	violaceol II biosynthetic process	2	0	1	2	4	7

* Numbers are combined for *A. nidulans*, *A. fumigatus*, *A. niger* and *A. oryzae.*

We also used published SMURF (Secondary Metabolite Unknown Regions Finder) predictions [38] to annotate additional candidate gene cluster backbone enzymes (i.e., PKS, NRPS, DMATS). SMURF is highly accurate at predicting most of these cluster backbone enzymes; across the four species of *Aspergillus* analyzed it identified a total of 105 genes as encoding PKS or PKS-like enzymes, 65 genes encoding NRPS or NRPS-like enzymes, 8 genes encoding putative hybrid PKS-NRPS enzymes and 15 DMATS. Note that DTS genes are not predicted by the SMURF algorithm. The AspGD Locus Summary pages now indicate these annotations based on the cluster backbone predictions generated by SMURF and by direct experimental characterization from the secondary metabolism literature.

### Expansion of the secondary metabolism branch of the GO

To improve the accuracy of the AspGD GO annotation in the area of secondary metabolite production, a branch of the GO in which terms were sparse, we worked in collaboration with the GO Consortium to add new, more specific terms to the BP aspect of the ontology, and then used many of these new GO terms to annotate the *Aspergillus* genes that had experimentally determined mutant phenotype data associated with one or more secondary metabolite. We focused on the BP annotations because the relevant processes are well-represented in the experimental literature, whereas experimental data to support CC annotations are relatively sparse in the secondary metabolism literature. Adequate MF terms exist for the PKS and NRPS enzymes, but annotations to them in AspGD are mostly based on computationally determined domain matches and Interpro2GO annotations, or by annotations with Reviewed Computational Analysis (RCA) as the evidence code, meaning that these functions are predicted, rather than directly characterized through experiments.

The new GO annotations that we have added now precisely specify the secondary metabolite produced. For example, *mdpG* is known to influence the production of arugosin, emodin, monodictyphenone, orsinellic acid, shamixanthones and sterigmatocystin in *A. nidulans*. The gene was formerly annotated to the fairly nonspecific parental term ‘secondary metabolic process’ (GO:0019748), but because the secondary metabolites produced by this protein are known and published, it is now annotated to the new and more informative child terms ‘arugosin biosynthetic process’ (GO:1900587), ‘emodin biosynthetic process’ (GO:1900575), ‘monodictyphenone biosynthetic process’ (GO:1900815), ‘o-orsellinic acid biosynthetic process’ (GO:1900584), ‘shamixanthone biosynthetic process’ (GO:1900793) and ‘sterigmatocystin biosynthetic process’ (GO:0045461).

In total, we added 290 new BP terms to the GO for 48 secondary metabolites produced by one or more *Aspergillus* species. There are over 400 *Aspergillus* genes in AspGD that have been manually or computationally annotated to more specific secondary metabolism BP terms, based on over 260 publications (Table 2). A complete list of the GO terms for secondary metabolic process annotations is available in Additional file 1. The addition of new terms is ongoing as new secondary metabolites and their biosynthetic genes are identified and described in the scientific literature. The process of adding new GO terms depends on the elucidation of the structure of the secondary metabolite as the structure is required for new ChEBI (Chemical Entities of Biological Interest; http://www.ebi.ac.uk/chebi/) terms to be assigned, and these chemical compound terms are a prerequisite for GO term assignments involving chemical compounds. These new and improved GO terms provide researchers with valuable clues to aid in the identification of proteins involved in the production of specific classes of *Aspergillus* secondary metabolites.

**Table 2:** GO terms used for secondary metabolism annotations at AspGD

	***A. nidulans***	***A. fumigatus***	***A. niger***	***A. oryzae***
Number of predicted protein-encoding genes	10,287	9,793	13,870	11,896
Number of genes with GO annotations to secondary metabolism	248	171	228	195
Number of genes with manual GO annotations to secondary metabolism*	202	96	81	32
Number of genes with computational GO annotations to secondary metabolism*	58	98	170	166

* or to child terms of ‘secondary metabolic process’ (GO: 0019748).

### Predictive annotation using orthology relationships in conjunction with experimentally-based GO term assignments

Manual curation of the genes of one species can be used to computationally annotate the uncharacterized genes in another species based on orthology relationships. The use of GO to describe gene products facilitates comparative analysis of functions of orthologous genes throughout the tree of life, including orthologous genes within the filamentous fungi. To augment the manual GO curation in AspGD, we leveraged orthology relationships to assign GO annotations to genes that lacked manual annotations of their own but which had an experimentally characterized ortholog in AspGD, the *Saccharomyces* Genome Database (SGD) (http://www.yeastgenome.org) or PomBase (http://www.pombase.org). A total of 492 GO annotations were made to secondary metabolism-related genes in *A. nidulans*, *A. fumigatus*, *A. niger* and *A. oryzae* based on their orthology relationships (Table 3). Files listing these orthology relationships are available for download at http://www.aspergillusgenome.org/download/homology/orthologs/ and the files describing all GO term annotations for each gene product in AspGD are available at http://www.aspergillusgenome.org/download/go/. A list of all genes annotated to the secondary metabolic process branch of the GO and their associated annotations can be obtained through the AspGD Advanced Search Tool (http://www.aspergillusgenome.org/cgi-bin/search/featureSearch).

**Table 3:** **Number of GO annotations for secondary metabolism that were transferred to and between *Aspergillus *species under curation at AspGD**

**From:**	**To *A. nidulans***	**To *A. fumigatus***	**To *A. niger***	**To *A. oryzae***
*S. cerevisiae*	3	1	0	4
*S. pombe*	1	0	0	0
*A. nidulans*	n/a	96	138	131
*A. fumigatus*	53	n/a	47	55
*A. niger*	2	1	n/a	3
*A. oryzae*	4	3	5	n/a

### Manual annotation of computationally predicted gene clusters

Algorithms such as SMURF [38] and antiSMASH (antibiotics and Secondary Metabolite Analysis SHell) [39] can be used to predict fungal secondary metabolite gene clusters. Both of these algorithms are based on the identification of backbone enzymes, usually one or more polyketide synthase (PKS), non-ribosomal peptide synthetase (NRPS), hybrid PKS-NRPS, NRPS-like enzyme or dimethylallyl tryptophan synthase (DMATS), and the use of a training set of experimentally characterized clusters. Adjacent genes are then scanned for the presence of common secondary metabolite gene domains and boundaries are predicted for each cluster. We used the pre-computed gene clusters for *A. nidulans*, *A. fumigatus*, *A. niger* and *A. oryzae* that were identified at the J. Craig Venter Institute (JCVI) with the SMURF algorithm [38]. We also used the antiSMASH algorithm [39] on these genomes to make gene cluster predictions and added 5 additional clusters for *A. nidulans* based on the presence of DTS/ent-kaurene synthase backbone enzymes.

**Figure 1:**
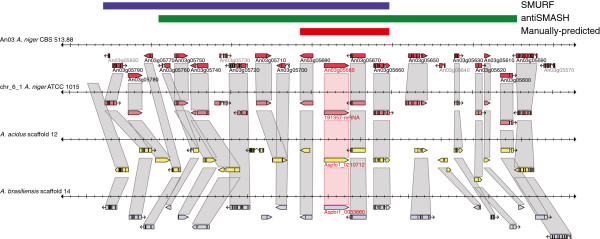
**Genomic context of the predicted An03g05680 cluster of *A. niger *viewed with the Sybil multiple genome browser.** Boundary predictions for *A. niger* CBS 513.88 species identifies predicted clusters in *A. niger* ATCC 1015, *A. acidus* and *A. brasiliensis* by matching orthologous protein clusters in Sybil. The red bar delineates the manually predicted cluster boundary based on cluster synteny between 2 *A. niger* strains and 2 additional *Aspergillus* species. The blue bar indicates the extent of the SMURF cluster prediction and the green bar indicates the antiSMASH-predicted boundaries.

Altogether, a total of 261 non-redundant clusters were predicted by SMURF and antiSMASH: 71 for *A. nidulans*, 39 for *A. fumigatus*, 81 for *A. niger* and 75 for *A. oryzae* (Tables 4, 5, 6, 7). Neither SMURF nor antiSMASH predict DTS-based clusters, so these clusters were manually identified based on their annotations. Because clusters with other types of non-PKS and non-NRPS backbone enzymes were included in the antiSMASH predictions and SMURF only analyzes PKS, NRPKS or DMATS-based clusters, antiSMASH identified more clusters than SMURF in every species except for *A. niger* (Table 8). For clusters identified by both algorithms, there were no cases where both the left and right boundary predictions were the same, although a small number of single boundary predictions did coincide with each other (Tables 4, 5, 6, 7). Both the experimentally and manually (see below) predicted clusters tend to be smaller than the SMURF and antiSMASH algorithms predict, as the algorithms are designed to err on the side of inclusivity while the manual boundaries are designed to provide increased precision of the cluster boundaries through the examination of inter- and intra-cluster genome synteny alignments across multiple *Aspergillus* species. SMURF was previously reported to overpredict boundaries by about 4 genes [38] and we found that antiSMASH performed similarly. Figure 1 shows an example of the disparity between these two prediction programs in cluster boundary determination and how intra- and inter-species cluster synteny data used in our analysis aids in the manual predictions of secondary metabolite gene cluster boundaries (see below).

**Table 4:** ***A. nidulans *secondary metabolite biosynthetic gene clusters determined by SMURF, antiSMASH and by manual annotation or experimental characterization**

***A. nidulans *Cluster Summary**							
	**Backbone enzyme**	**SMURF**	**antiSMASH**	**Manual/experimental**	**Boundary 1**	**Boundary 2**	**Reference**
Asperfuranone (afo) cluster	AN1036	AN1029 - AN11288	AN1026 - AN1040	AN1029 - AN1036	ED	ED	[40]
Asperthecin (apt) cluster	AN6000	AN5989 - AN6007	AN5991 - AN6009	AN6000 - AN6002*	ED, EP	ED, EP	[30]
Aspyridone (asp) cluster	AN8412	AN8398 - AN8415	AN8404 - AN11609	AN8408 - AN8415*	ED	ED	[41]
Austinol (aus) cluster 1	AN9244	AN9243 - AN9251	AN11817 - AN9254	AN9244 - AN9253	ED, FA	ED	[31]
Austinol (aus) cluster 2	AN8383	AN8382 - AN8395	AN8377 - AN8390	AN8379 - AN8384*	ED, ECS	ED, ECS	[31]
Derivative of Benzaldehyde1 (dba) and F9775 hybrid cluster 1	AN7909	-	AN7895 - AN7919	AN7896 - AN7903*	ED	ED, EP	[34,42]
Derivative of Benzaldehyde1 (dba) and F9775 hybrid cluster 2		AN7905 - AN7923	AN7895 - AN7919	AN7907 - AN7916*	ED	ED	[34,42]
Emericellamide (eas) cluster	AN2547	AN2544 - AN2553	AN2538 - AN10326	AN2545 - AN2549*	ED	ED	[22]
inp cluster	AN3496	AN3491 - AN3506	AN3485 - AN3503	AN3490 - AN3496	EP	EP	[43]
ivo cluster	AN10576	AN10576 - AN4649	AN4637 - AN4649	AN10573 - AN10576	EP	EP	This study
Microperfuranone (mic) cluster	AN3396	AN3391 - AN3408	AN3391 - AN3404	AN3394 - AN3396	EP	EP	[16]
Monodictyphenone (mdp) cluster	AN0150	AN0146 - AN0158	AN11846 - AN0156	AN10021 - AN10023*	ED, EP	ED, EP	[16,44]
Penicillin cluster	AN2621	AN2619 - AN2625	AN2616 - AN10329	AN2621 - AN2623*	ED, EP	ED, EP	[16,45]
Nidulanin A (nptA) cluster	AN11080	-	AN8481 - AN8484	AN11082 - AN11080	IGD, FA	ECS	This study
pkb cluster	AN6448	AN6436 - AN6462	AN6438 - AN6455	AN6444 - AN6451	EP	EP	[16]
pkdA cluster	AN0523	AN0520 - AN0533	AN10104 - AN0534	AN0523 - AN0533	ECS	ECS	This study
pkf cluster	AN3230	AN3217 - AN3237	AN3222 - AN3238	AN3225 -AN3230	EP	ECS, EP	This study
pkg cluster	AN7071	AN7061 - AN7075	AN7064 - AN7078	AN10889 - AN7075	EP	ECS, EP	This study
pkh cluster	AN2035	AN2032 - AN2043	AN2029 - AN2042	AN2030 - AN2038	ECS, IGD	EP	[16]
pki cluster	AN3386	AN3380 - AN3386	AN3380 - AN3392	AN3379 - AN3386	ECS	ECS	This study
Sterigmatocystin (stc) cluster	AN7815	AN7806 - AN7824	AN7815 - AN7832	AN7804 - AN7824*	ED	ED	[46]
Terriquinone (tdi) cluster	AN8514	AN8506 - AN8516	AN8508 - AN8522	AN8513 - AN8520*	ED	ED	[47]
xptA-containing cluster	AN6784	AN6770 - AN6791	AN6780 - AN6786	AN6784 - AN6791	FA, EP	FA, EP	FA, [16]
xptB-containing cluster	AN12402	-	AN7996 - AN12431	AN7999 - AN12431	ECS	IGD	This study
AN0016 cluster	AN0016	AN0015 - AN0029	AN10009 - AN0025	AN0015 - AN0029	ECS, IGD	FA	This study
AN0607 (sidC) cluster	AN0607	AN0592 - AN0607	AN0599 - AN0617	AN0607 - AN0609	ECS	ECS, IGD	This study
AN10289 cluster	AN10289	AN10294 - AN2349	AN10294 - AN2347	AN10289 - AN2347	IGD	FA, IGD	This study
AN10297 cluster	AN10297	AN2392 - AN10301	AN2395 - AN10298	AN2396 - AN11337	FA	FA	This study
AN10396 cluster	AN10396	-	AN3372 - AN3379	AN3375 - AN10401	ECS	ECS, IGD	This study
AN10430 cluster	AN10430	AN3608 - AN3612	AN10429 - AN10448	AN3609 - AN3612	FA	FA	This study
AN10486 cluster	AN10486	AN3911 - AN3917	AN10485 - AN3917	AN3911 - AN10486	ECS	ECS	This study
AN11065 cluster	AN11065	-	AN12204 - AN8263	-	n/a	n/a	n/a
AN11191 cluster	AN11191	AN9210 - AN11192	AN9212 - AN12146	AN11191 - AN9220	ECS, EP	EP	[16]
AN11194 cluster	AN11194	AN9223 - AN9236	AN9220 - AN9236	AN8223 - AN9235	EP	EP	[16]
AN12331 cluster	AN12331	AN7836 - AN7837	AN7831 - AN7842	AN7836 - AN7839	FA	ECS	This study
AN1242 cluster	AN1242	AN1242 - AN1243	AN1236 - AN1250	AN1242 - AN1247	ECS, IGD	ECS	This study
AN1594 cluster	AN1594	-	-	AN1592 - AN1599	EP	EP	[16]
AN1680 cluster	AN1680	AN1677 - AN1691	AN1674 - AN1687	AN1678 - AN1681	ECS	ECS	This study
AN1784 cluster	AN1784	AN1778 - AN1787	AN1776 - AN1790	AN1784 - AN1787	EP	EP	[16]
AN1793 cluster	AN1793	-	AN1790 - AN1802	AN1792 - AN1796	FA	ECS	ECS
AN2064 cluster	AN2064	AN2061 - AN2064	AN2057 - AN2068	-	n/a	n/a	n/a
AN2924 cluster	AN2924	AN2921 - AN2925	AN2917 - AN2928	AN2921 - AN2924	EP	EP	[16]
AN3252 cluster	AN3252	-	-	AN3252 - AN3257	EP	EP	[16]
AN3273 cluster	AN3273		AN3267 - AN3285	AN10388 - AN3287	IGD	IGD	This study
AN3612 cluster	AN3612	AN3608 - AN3612	AN10429 - AN10448	AN3605 - AN3612	FA	ECS, FA	This study
AN4827 cluster	AN4827	AN4823 - AN4837	AN11232 - AN4836	AN10600 - AN11232	EP	EP	[16]
AN5318 cluster	AN5318	AN5317 - AN5329	AN5312 - AN5325	AN5314 - AN5318	ECS	ECS	This study
AN5475 cluster	AN5475	-	-	-	n/a	n/a	n/a
AN5610 cluster	AN5610	-	AN5607 - AN5616	AN9473 - AN5610	ECS, IGD	FA	This study
AN6236 cluster	AN6236	AN6236 - AN6238	AN6230 - AN11851	AN6234 - AN6236	ECS, EP	ECS, EP	ECS, [16]
AN6431 cluster	AN6431	AN6429 - AN6434	AN10818 - AN6437	AN6431 - AN6437	ECS, EP	EP	[16]
AN6791 cluster	AN6791	-	AN11527 - AN10846	AN6787 - AN6791	IGD	FA	This study
AN7084 cluster	AN7084	AN7081 - AN7089	AN7077 - AN7089	AN7080 - AN7086	EP	EP	[16]
AN7489 (mirC) cluster	AN7489	AN7485 - AN7493	-	AN7485 - AN7493	ECS	ECS	This study
AN7838 (AN12331) cluster	AN7838	AN7836 - AN7839	-	AN11024 - AN7839	FA	FA	This study
AN7884 cluster	AN7884	AN7873 - AN7884	AN7879 - AN7892	AN7872 - AN7884	ECS, EP	ECS, EP	ECS, [16]
AN8105 cluster	AN8105	AN8103 - AN8113	AN8098 - AN8113	AN8105 - AN8112	ECS, EP	EP	[16]
AN8142 cluster	AN8142	-	AN8133 - AN8147	AN12440 - AN8144	FA	ECS	This study
AN8209 (wA) cluster	AN8209	-	AN8202 - AN8214	AN12404 - AN8209	EP	ECS, EP	ECS, [16]
AN8249 cluster	AN8249	-	AN11063 - AN11069	-	n/a	n/a	n/a
AN8504 cluster	AN8504	-	AN8495 - AN8508	AN8495 - AN8504	ECS	FA	This study
AN8910 cluster	AN8910	AN8901 - AN8910	AN8903 - AN8915	AN8905 - AN8910	ECS	ECS	This study
AN9005 cluster	AN9005	AN9002 - AN9013	AN9000 - AN9013	AN9002 - AN9007	EP	EP	[16]
AN9129 cluster	AN9129	-	-	AN9129 - AN9130	ECS, EP	ECS	This study
AN9226 cluster	AN9226	AN9223 - AN9236	AN9220 - AN9236	AN9223 - AN9234	EP	EP	[16]
AN9291 (AN11820) cluster	AN11820	AN9280 - AN9307	-	AN11820 - AN9294	EP	ECS	This study
AN9314 cluster	AN9314	-	-	AN9313 - AN9314	EP	EP	[16]
No PKS/NRPS backbone 1	n/a	-	AN9184 - AN9194	AN9179 - AN9185	ECS	ECS	This study
No PKS/NRPS backbone 2	n/a	-	AN10325 - AN11348	-	n/a	n/a	n/a
No PKS/NRPS backbone 3	n/a	-	AN0650 - AN0659	AN0653 - AN0660	ECS	ECS	This study
No PKS/NRPS backbone 4	n/a	-	AN0039 - AN10006	AN0042 - AN0044	EP	EP	[16]

* Experimentally characterized, Abbreviations: *ECS*, End of cluster synteny; *IGD*, Increase in intergenic distance; *FA*, Change in functional annotation; *ED*, Experimentally determined; *EP*, Expression Pattern.

**Table 5:** ***A. fumigatus *secondary metabolite biosynthetic gene clusters determined by SMURF, antiSMASH and by manual annotation or experimental characterization**

***A. fumigatus *cluster summary**							
	**Backbone enzyme**	**SMURF**	**antiSMASH**	**Manual/experimental**	**Boundary 1**	**Boundary 2**	**Reference**
Fumigaclavine C (fga) cluster*	Afu2g18040	Afu2g17930 - Afu2g18070	Afu2g17950 - Afu2g18070	Afu2g17960 - Afu2g18060	ED	ED	[48]
Fumitremorgin B (ftm) cluster*	Afu8g00210	Afu8g00280 - Afu8g00100	Afu8g00100 - Afu8g00110	Afu8g00260 - Afu8g00170	ED	ED	[49]
Gliotoxin (gli) cluster*	Afu6g09650	Afu6g09580 - Afu6g09740	Afu6g09520 - Afu6g09745	Afu6g09630 - Afu6g09740	ED, IGD	ED	[50]
Pseurotin A cluster*	Afu8g00540	-	Afu8g00342 - Afu8g00595	Afu8g005340 - Afu8g00570	ED	ED	[35]
Siderophore (sid) cluster*	Afu3g03420	Afu3g03280 - Afu3g03580	Afu3g03270 - Afu3g03490	Afu3g03300 - Afu3g03460	ED	ED	[48]
Afu1g01010 cluster	Afu1g01010	Afu1g00970 - Afu1g01010	Afu1g00970 - Afu1g01030	Afu1g00980 - Afu1g01010	FA	IGD	This study
Afu1g10380 (nrps1) cluster	Afu1g10380	Afu1g10310 - Afu1g10380	Afu1g10310 - Afu1g10420	Afu1g10270 - Afu1g10380	ECS	IGD	This study
Afu1g17200 (sidC) cluster	Afu1g17200	Afu1g17080 - Afu1g17240	Afu1g17090 - Afu1g17270	Afu1g17200 - Afu1g17240	ECS	ECS	This study
Afu1g17740 cluster	Afu1g17740	Afu1g17710 - Afu1g17740	Afu1g17720 - Afu1g17750	Afu1g17710 - Afu1g17740	ECS	ECS	This study
Afu2g01290 cluster	Afu2g01290	Afu2g01170 - Afu2g01400	Afu2g01210 - Afu2g01390	Afu2g01280 - Afu2g01330	ECS	ECS	This study
Afu2g05760 cluster	Afu2g05760	Afu2g05730 - Afu2g05840	-	Afu2g05740 - Afu2g05830	IGD	IGD	This study
Afu2g17600 cluster	Afu2g17600	Afu2g17511 - Afu2g17600	Afu2g17490 - Afu2g17690	Afu2g17480 - Afu2g17600	IGD	IGD	This study
Afu3g01410 cluster	Afu3g01410	Afu3g01400 - Afu3g01560	Afu3g01360 - Afu3g01560	Afu3g01400 - Afu3g01480	IGD	IGD	This study
Afu3g02530 cluster	Afu3g02530	Afu3g02450 - Afu3g02540	Afu3g02450 - Afu3g02650	Afu3g02520 - Afu3g02650	FA	IGD	This study
Afu3g02570 cluster	Afu3g02570	Afu3g02570 - Afu3g02650	Afu3g02450 - Afu3g02650	Afu3g02520 - Afu3g02650	FA	IGD	This study
Afu3g02670 cluster	Afu3g02670	Afu3g02670 - Afu3g02760	Afu3g02670 - Afu3g02760	Afu3g02670 - Afu3g02720	ECS, IGD	ECS, IGD	This study
Afu3g12920 cluster	Afu3g12920	Afu3g12960 - Afu3g12750	Afu3g13020 - Afu3g12820	Afu3g12960 - Afu3g12890	ECS	ECS	This study
Afu3g12930 cluster	Afu3g12930	Afu3g13000 - Afu3g12750	Afu3g13020 - Afu3g12820	Afu3g12960 - Afu3g12890	ECS	ECS	This study
Afu3g13730 cluster	Afu3g13730	Afu3g13730 - Afu3g13600	Afu3g13790 - Afu3g13600	Afu3g13750 - Afu3g13670	IGD	FA	This study
Afu3g14700 cluster	Afu3g14700	Afu3g14880 - Afu3g14690	Afu3g14820 - Afu3g14620	Afu3g14730 - Afu3g14690	ECS	FA	This study
Afu3g15270 cluster	Afu3g15270	Afu3g15290 - Afu3g15250	Afu3g15350 - Afu3g15190	Afu3g15290 - Afu3g15240	ECS, IGD	ECS	This study
Afu4g00210 cluster	Afu4g00210	Afu4g00260 - Afu4g00210	Afu4g00290 - Afu4g00150	Afu4g00260 - Afu4g00200	IGD	ECS	This study
Afu4g14560 cluster	Afu4g14560	Afu4g14730 - Afu4g14420	Afu4g14660 - Afu4g14440	Afu4g14610 - Afu4g14450	ECS	ECS	This study
Afu5g10120 cluster	Afu5g10120	Afu5g10250 - Afu5g09970	Afu5g10240 - Afu5g10010	Afu5g10130 - Afu5g10040	ECS	FA	This study
Afu5g12730 cluster	Afu5g12730	Afu5g12840 - Afu5g12720	Afu5g12830 - Afu5g12680	Afu5g12770 - Afu5g12730	FA	ECS	This study
Afu6g03480 cluster	Afu6g03480	Afu6g03620 - Afu6g03430	Afu6g03550 - Afu6g03400	Afu6g03490 - Afu6g03430	ECS	ECS, IGD	This study
Afu6g08560 cluster	Afu6g08560	Afu6g08540 - Afu6g08560	Afu6g08520 - Afu6g08640	Afu6g08550 - Afu6g08560	ECS	ECS	ECS, IGD
Afu6g12080 cluster	Afu6g12080	Afu6g12040 - Afu6g12160	Afu6g11980 - Afu6g12145	Afu6g12040 - Afu6g12080	ECS	ECS	This study
Afu6g13930 cluster	Afu6g13930	Afu6g13830 - Afu6g14050	Afu6g13820 - Afu6g14030	Afu6g13920 - Afu6g14000	ECS	ECS	This study
Afu7g00170 cluster	Afu7g00170	Afu7g00200 - Afu7g00120	Afu7g00220 - Afu7g00100	Afu7g00190 - Afu7g00120	ECS	ECS, IGD	This study
Afu8g00540 cluster	Afu8g00540	Afu8g00370 - Afu8g00370	Afu8g00490 - Afu8g00310	-	n/a	n/a	This study
Afu8g00620 cluster	Afu8g00620	Afu8g00640 - Afu8g00470	Afu8g00720 - Afu8g00390	-	n/a	n/a	This study
Afu8g01640 cluster	Afu8g01640	Afu8g01640 - Afu8g01640	Afu8g01600 - Afu8g01730	Afu8g01630 - Afu8g01640	ECS, IGD	ECS, IGD	This study
Afu8g02350 cluster	Afu8g02350	Afu8g02460 - Afu8g02350	Afu8g02490 - Afu8g02280	Afu8g02430 - Afu8g02350	ECS, IGD	ECS, IGD	This study
No PKS or NRPS backbone 1	n/a	-	Afu4g11170 - Afu4g11300	-	n/a	n/a	
No PKS or NRPS backbone 2	n/a	-	Afu4g11980 - Afu4g12070	-	n/a	n/a	
No PKS or NRPS backbone 3	n/a	-	Afu5g00100 - Afu5g04130	-	n/a	n/a	
No PKS or NRPS backbone 4	n/a	-	Afu7g00230 -Afu7g00350	Afu5g00100 - Afu5g00135	ECS, IGD	ECS, IGD	This study
No PKS or NRPS backbone 5	n/a	-	Afu7g01180 - Afu7g01270	Afu7g00260 - Afu7g00270	ECS, FA	ECS	This study

* Experimentally characterized; Abbreviations: *ECS*, End of cluster synteny; *IGD*, Increase in intergenic distance; *FA*, Change in functional annotation; *ED*, Experimentally determined (1).

**Table 6:** ***A. niger *secondary metabolite biosynthetic gene clusters determined by SMURF, antiSMASH and by manual annotation or experimental characterization**

***A. niger *cluster summary**						
	**Backbone enzyme**	**SMURF**	**antiSMASH**	**Manual**	**Boundary 1**	**Boundary 2**
An01g01130 cluster	An01g01130	An01g01080 - An01g01150	An01g01050 - An01g01220	An01g01110 - An01g01230	FA	ECS
An01g06930/ An01g06950 cluster	An01g06930	An01g06730 - An01g06950	An01g06770 - An01g07020	An01g06810 - An01g06970	ECS	ECS. FA
An01g11770 cluster	An01g11770	An01g11740 - An01g11900	An01g11690 - An01g11880	An01g11760 - An01g11830	IGD, ECS	ECS, IGD
An02g00210 cluster	An02g00210	An02g00150 - An02g00290	An02g00140 - An02g00320	An02g00210 - An02g00260	ECS	ECS
An02g00450 cluster	An02g00450	An02g00430 - An02g00580	An02g00340 - An02g00540	-	n/a	n/a
An02g00840 cluster	An02g00840	An02g00710 - An02g00860	An02g00740 - An02g00950	An02g00700 - An02g00840	ECS, IGD	ECS. FA
An02g05070 cluster	An02g05070	An02g05050 - An02g05190	An02g04970 - An02g05170	An02g05050 - An02g05170	ECS	ECS
An02g08290 cluster	An02g08290	An02g08130 - An02g08370	An02g08210 - An02g08380	An02g08290 - An02g08310	ECS	ECS
An02g09430 cluster	An02g09430	An02g09420 - An02g09450	An02g09310 - An02g09530	An02g09390 - An02g09430	ECS	ECS
An02g10140 cluster	An02g10140	An02g10110 - An02g10170	An02g10080 - An02g10200	An02g10140 - An02g10200	ECS	FA
An02g14220 cluster	An02g14220	An02g14190 - An02g14240	-	An02g14170 - An02g14240	ECS	ECS
An03g00650 cluster	An03g00650	An03g00580 - An03g00790	An03g00530 - An03g00760	-	n/a	n/a
An03g01820 cluster	An03g01820	An03g01750 - An03g01850	An03g01730 - An03g01930	An03g01790 - An03g01820	ECS	ECS
An03g03520 cluster	An03g03520	An03g03380 - An03g03620	An03g03450 - An03g03600	An03g03490 - An03g03620	ECS	ECS, IGD
An03g05140 cluster	An03g05140	An03g05050 - An03g05270	An03g05040 - An03g05230	An03g05140 - An03g05170	ECS	ECS
An03g05440 cluster	An03g05440	An03g05300 - An03g05500	An03g05330 - An03g05540	An03g05430 - An03g05500	ECS	ECS
An03g05680 cluster	An03g05680	An03g05660 - An03g05800	An03g05600 - An03g05760	An03g05660 - An03g05710	ECS	ECS
An03g06010 cluster	An03g06010	An03g05810 - An03g06020	An03g05910 - An03g06100	An03g05880 - An03g06020	ECS	ECS, IGD
An03g06380 cluster	An03g06380	An03g06370 - An03g06520	An03g06310 - An03g06470	An03g06310 - An03g06440	ECS, IGD	ECS
An04g01150 cluster	An04g01150	An04g01120 - An04g01170	An04g01070 - An04g01230	An04g01080 - An04g01180	ECS	ECS
An04g04340 cluster	An04g04340	An04g04220 - An04g04520	An04g04280 - An04g04500	An04g04330 - An04g04400	ECS	ECS
An04g04380 cluster	An04g04380	An04g04340 - An04g04520	-	An04g06240 - An04g06320	ECS	ECS
An04g06260 cluster	An04g06260	An04g06230 - An04g06290	An04g06180 - An04g06370	An04g06240 - An04g06320	ECS	ECS
An04g09530 cluster	An04g09530	An04g09500 - An04g09650	An04g09420 - An04g09600	An04g09510 - An04g09570	ECS	ECS
An04g10030 cluster	An04g10030	An04g09890 - An04g10090	An04g09900 - An04g10100	-	n/a	n/a
An05g01060 cluster	An05g01060	An05g01020 - An05g01120	An05g00930 - An05g01140	An05g01060 - An05g01120	ECS	ECS
An06g01300 cluster	An06g01300	An06g01290 - An06g01410	An06g01200 - An06g01390	An06g01290 - An06g01320	ECS	ECS
An07g01030 cluster	An07g01030	An07g00900 - An07g01100	An07g00900 - An07g01130	-	n/a	n/a
An07g02560 cluster	An07g02560	An07g02540 - An07g02650	An07g02520 - An07g02620	An07g02510 - An07g02560	ECS	ECS
An08g02310 cluster	An08g02310	An08g02180 - An08g02330	An08g02210 - An08g02360	An08g02170 - An08g02310	ECS	ECS
An08g03790 cluster	An08g03790	An08g03610 - An08g03870	An08g03700 - An08g03880	An08g03730 - An08g03790	ECS	ECS
An08g04820 cluster	An08g04820	An08g04710 - An08g04940	An08g04710 - An08g04900	An08g04790 - An08g04830	ECS	ECS
An08g09220 cluster	An08g09220	An08g09150 - An08g09400	An08g09140 - An08g09360	-	n/a	n/a
An08g10930 cluster	An08g10930	An08g10880 - An08g11000	-	An08g10860 - An08g10930	ECS	ECS
An09g00450 cluster	An09g00450	An09g00400 - An09g00610	An09g00350 - An09g00610	An09g00390 - An09g00450	ECS	ECS
An09g00520 cluster	An09g00520	An09g00390 - An09g00620	-	An09g00520 - An09g00620	ECS	ECS
An09g01290 cluster	An09g01290	An09g01140 - An09g01460	An09g01200 - An09g01390	An09g01260 - An09g01340	ECS	ECS
An09g01690 cluster	An09g01690	An09g01650 - An09g01860	An09g01560 - An09g01800	An09g01630 - An09g01860	ECS	ECS
An09g01860/An09g01930 cluster	An09g01860	An09g01690 - An09g02020	An09g01740 - An09g02020	An09g01790 - An09g01950	ECS, FA	ECS, FA
An09g02100 cluster	An09g02100	An09g01980 - An09g02230	An09g01980 - An09g02220	-	n/a	n/a
An09g05110 cluster	An09g05110	An09g04960 - An09g05130	An09g05010 - An09g05180	An09g05060 - An09g05150	ECS	ECS
An09g05340 cluster	An09g05340	An09g05310 - An09g05360	An09g05270 - An09g05400	An09g05300 - An09g05350	ECS	ECS
An09g05730 cluster	An09g05730	An09g05710 - An09g05750	An09g05655 - An09g05810	An09g05625 - An09g05730	ECS	ECS
An10g00140 cluster	An10g00140	An10g00010 - An10g00230	An10g00050 - An10g00240	An10g00100 - An10g00210	ECS	ECS
An10g00630 cluster	An10g00630	An10g00540 - An10g00700	-	An10g00620 - An10g00700	ECS	ECS
An11g00050 cluster	An11g00050	An11g00030 - An11g00080	An11g00010 - An11g00100	An11g00040 - An11g00080	ECS	ECS
An11g00250 cluster	An11g00250	An11g00130 - An11g00380	An11g00170 - An11g00350	An11g00250 - An11g00300	ECS	ECS
An11g03920 cluster	An11g03920	An11g03750 - An11g04040	An11g03820 - An11g04010	An11g03870 - An11g03940	ECS	ECS
An11g04280 cluster	An11g04280	An11g04140 - An11g04310	An11g04180 - An11g04350	An11g04250 - An11g04320	ECS	ECS
An11g05500 cluster	An11g05500	An11g05470 - An11g05570	An11g05420 - An11g05560	An11g05480 - An11g05530	ECS	ECS, IGD
An11g05570 cluster	An11g05570	An11g05440 - An11g05590	An11g05510 - An11g05660	-	n/a	n/a
An11g05940 cluster	An11g05940	An11g05820 - An11g05960	An11g05860 - An11g06060	An11g05940 - An11g05960	ECS	ECS
An11g05960 cluster	An11g05960	An11g05790 - An11g05980	An11g05860 - An11g06060	An11g05940 - An11g05960	ECS	ECS
An11g06460 cluster	An11g06460	An11g06420 - An11g06490	An11g06350 - An11g06570	An11g06420 - An11g06490	ECS	ECS
An11g07310 cluster	An11g07310	An11g07280 - An11g07350	An11g07210 - An11g07460	An11g07270 - An11g07350	ECS	ECS
An11g09720 cluster	An11g09720	An11g09700 - An11g09740	An11g09620 - An11g09790	-	n/a	n/a
An12g02670 cluster	An12g02670	An12g02650 - An12g02830	An12g02560 - An12g02920	An12g02620 - An12g02750	ECS	ECS
An12g02730 cluster	An12g02730	An12g02670 - An12g02900	-	-	n/a	n/a
An12g02840 cluster	An12g02840	An12g02680 - An12g02880	-	-	n/a	n/a
An12g07070 cluster	An12g07070	An12g07050 - An12g07110	An12g06930 - An12g07140	An12g07060 - An12g07110	ECS	ECS
An12g07230 cluster	An12g07230	An12g07120 - An12g07250	An12g07150 - An12g07330	An12g07220 - An12g07280	ECS	ECS
An12g10090 cluster	An12g10090	An12g09930 - An12g10220	An12g09980 - An12g10190	An12g10000 - An12g10160	ECS	ECS
An12g10860 cluster	An12g10860	An12g10840 - An12g10870	An12g10770 - An12g10960	An12g10790 - An12g10860	ECS	ECS
An13g01840 cluster	An13g01840	An13g01820 - An13g01860	An13g01790 - An13g01880	An13g01810 - An13g01860	ECS	ECS
An13g02430 cluster	An13g02430	An13g02290 - An13g02540	An13g02350 - An13g02600	An13g02390 - An13g02470	ECS	ECS
An13g02460 cluster	An13g02460	An13g02400 - An13g02540	-	An13g02390 - An13g02480	ECS	ECS
An13g02960 cluster	An13g02960	An13g02940 - An13g03090	An13g02830 - An13g03120	-	n/a	n/a
An13g03040 cluster	An13g03040	An13g02940 - An13g03110	An13g02830 - An13g03120	-	n/a	n/a
An14g01910 cluster	An14g01910	An14g01850 - An14g01970	An14g01820 - An14g02010	An14g01910 - An14g01960	ECS	ECS
An14g04850 cluster	An14g04850	An14g04830 - An14g04870	An14g04750 - An14g04940	An14g04830 - An14g04890	ECS	ECS
An15g02130 cluster	An15g02130	An15g02070 - An15g02190	An15g02040 - An15g02230	An15g02130 - An15g02200	ECS	ECS
An15g04140 cluster	An15g04140	An15g04120 - An15g04220	An15g04050 - An15g04250	An15g04130 - An15g04150	ECS	ECS
An15g05090 cluster	An15g05090	An15g05030 - An15g05150	An15g05000 - An15g05180	An15g05090 - An15g05150	ECS	ECS
An15g07530 cluster	An15g07530	An15g07510 - An15g07600	An15g07400 - An15g07640	An15g07530 - An15g07480	ECS	ECS
An15g07910 cluster	An15g07910	An15g07830 - An15g07930	An15g07810 - An15g07930	An15g07890 - An15g07920	ECS	ECS
An15g07920 cluster	An15g07920	An15g07830 - An15g07930	-	-	n/a	n/a
An16g00600 cluster	An16g00600	An16g00520 - An16g00740	An16g00460 - An16g00730	An16g00520 - An16g00600	ECS	ECS
An16g06720 cluster	An16g06720	An16g06570 - An16g06780	An16g06650 - An16g06790	An16g06720 - An16g06750	ECS	ECS
An18g00520 cluster	An18g00520	An18g00350 - An18g00550	An18g00440 - An18g05820	An18g00460 - An18g00530	ECS	ECS

* Experimentally characterized; Abbreviations: *ECS*, End of cluster synteny; *IGD*, Increase in intergenic distance; *FA*, Change in functional annotation; *ED*, Experimentally determined.

**Table 7:** ***A. oryzae *secondary metabolite biosynthetic gene clusters determined by SMURF, antiSMASH and by manual annotation or experimental characterization**

***A. oryzae*cluster summary**						
	**Backbone enzyme**	**SMURF**	**antiSMASH**	**Manual**	**Boundary 1**	**Boundary 2**
Csypyrone B1 cluster	AO090701000566	-	AO090701000569 - AO090701000568	AO090701000561 - AO090701000568	ED	ED
Dipeptidyl Peptidase IV 2 Inhibitor (WYK-1) cluster	AO090001000009	AO090001000031 - AO090001000009	AO090001000019 - AO090001000001	AO090001000019 - AO090001000009	ED	ED
AO090001000043 cluster	AO090001000043	AO090001000051 - AO090001000021	AO090001000055 - AO090001000021	AO090001000051 - AO090001000028	ECS, FA	FA
AO090001000262 cluster	AO090001000262	-	AO090001000253 - AO090001000268	AO090001000260 - AO090001000264	ECS	FA, ECS
AO090001000277 cluster	AO090001000277	AO090001000293 - AO090001000256	AO090001000289 - AO090001000267	AO090001000292 - AO090001000277	ECS	ECS
AO090001000293 cluster	AO090001000293	-	AO090001000757 - AO090001000295	-	n/a	n/a
AO090001000402 cluster	AO090001000402	AO090001000404 - AO090001000390	AO090001000408 - AO090001000390	AO090001000408 - AO090001000390	ECS	ECS
AO090001000506 cluster	AO090001000506	AO090001000506 - AO090001000505	AO090001000512 - AO090001000495	AO090001000506 - AO090001000505	FA	ECS
AO090001000516 cluster	AO090001000516	AO090001000516 - AO090001000515	AO090001000523 - AO090001000512	AO090001000516 - AO090001000515	ECS	ECS
AO090001000768 cluster	AO090001000768	AO090001000704 - AO090001000692	-	AO090001000768 - AO090001000687	ECS	ECS
AO090003000945 cluster	AO090003000945	AO090003000954 - AO090003000945	AO090003000954 - AO090003000936	AO090003000946 - AO090003000941	ECS	ECS
AO090003001097 cluster	AO090003001097	-	AO090003001090 - AO090003001106	AO090003001094 - AO090003001099	ECS	ECS
AO090003001545 cluster	AO090003001545	AO090003001556 - AO090003001541	AO090003001552 - AO090003001535	AO090003001556 - AO090003001537	ECS	ECS
AO090005000688 cluster	AO090005000688	AO090005000688 - AO090005000693	AO090005000681 - AO090005000696	AO090005000687 - AO090005000695	FA	ECS
AO090005000798 cluster	AO090005000798	-	AO090005000791 - AO090005000805	AO090005000796 - AO090005000805	ECS	ECS
AO090005000952 cluster	AO090005000952	AO090005000952 - AO090005000955	AO090005000942 - AO090005000959	AO090005000952 - AO090005000956	ECS	ECS
AO090005000961 cluster	AO090005000961	AO090005000961 - AO090005000968	AO090005000956 - AO090005000970	AO090005000961 - AO090005000968	ECS	ECS
AO090005000993 cluster	AO090005000993	AO090005000990 - AO090005001001	AO090005000986 - AO090005001002	AO090005000990 - AO090005000993	ECS	ECS
AO090005001079 cluster	AO090005001079	AO090005001078 - AO090005001087	AO090005001075 - AO090005001081	-	n/a	n/a
AO090005001551 cluster	AO090005001551	-	AO090005001668 - AO090005001555	-	n/a	n/a
AO090009000052 cluster	AO090009000052	-	AO090009000046 - AO090009000717	AO090009000051 - AO090009000717	IGD	IGD
AO090009000071 cluster	AO090009000071	AO090009000071 - AO090009000068	AO090009000079 - AO090009000065	AO090009000071 - AO090009000066	ECS, IGD	ECS
AO090009000131 cluster	AO090009000131	AO090009000143 - AO090009000131	AO090009000141 - AO090009000122	AO090009000140 - AO090009000131	FA	ECS
AO090010000048 (aoi) cluster*	AO090010000048	AO090010000035 - AO090010000054	AO090010000040 - AO090010000056	AO090010000040 - AO090010000056*	ED	ED
AO090010000082 cluster	AO090010000082	AO090010000070 - AO090010000082	-	AO090010000074 - AO090010000083	ECS	ECS
AO090010000114 cluster	AO090010000114	AO090010000097 - AO090010000114	AO090010000108 - AO090010000122	AO090010000104 - AO090010000115	ECS	ECS
AO090010000204 cluster	AO090010000204	-	AO090010000202 - AO090010000207	AO090010000202 - AO090010000204	ECS	ECS
AO090010000349 cluster	AO090010000349	AO090010000349 - AO090010000350	AO090010000340 - AO090010000353	AO090010000348 - AO090010000349	ECS	ECS
AO090010000404 cluster	AO090010000404	AO090010000390 - AO090010000407	AO090010000388 - AO090010000414	AO090010000390 - AO090010000407	ECS, FA	ECS
AO090010000426 cluster	AO090010000426	AO090010000424 - AO090010000426	AO090010000417 - AO090010000435	AO090010000422 - AO090010000427	IGD	IGD, ECS
AO090010000498 cluster	AO090010000498	AO090010000488 - AO090010000500	AO090010000490 - AO090010000507	-	n/a	n/a
AO090011000015 cluster	AO090011000015	AO090011000023 - AO090011000004	AO090011000022 - AO090011000009	AO090011000957 - AO090011000009	ECS	ECS
AO090011000043 cluster	AO090011000043	AO090011000043 - AO090011000031	AO090011000049 - AO090011000037	AO090011000043 - AO090011000040	ECS	ECS
AO090011000103 cluster	AO090011000103	-	AO090011000098 - AO090011000107	AO090011000099 - AO090011000105	ECS	ECS
AO090011000328 cluster	AO090011000328	AO090011000328 - AO090011000961	AO090011000333 - AO090011000320	AO090011000329 - AO090011000326	ECS, IGD	ECS, IGD
AO090011000408 cluster	AO090011000408	-	AO090011000403 - AO090011000413	-	n/a	n/a
AO090011000738 cluster	AO090011000738	AO090011000744 - AO090011000729	AO090011000743 - AO090011000733	AO090011000740 - AO090011000738	ECS, FA	ECS, FA
AO090012001034 cluster	AO090012001034	AO090012001034 - AO090012000510	AO090012000526 - AO090012000510	AO090012000521 - AO090012001034	IGD, ECS	ECS, IGD
AO090020000159 cluster	AO090020000159	-	AO090020000154 - AO090020000163	AO090020000156 - AO090020000162	ECS	ECS
AO090020000186 cluster	AO090020000186	AO090020000177 - AO090020000192	AO090020000172 - AO090020000191	AO090020000186 - AO090020000188	ECS	ECS
AO090020000194 cluster	AO090020000194	-	AO090020000191 - AO090020000200	AO090020000194 - AO090020000202	ECS	FA, ECS
AO090020000240 cluster	AO090020000240	AO090020000236 - AO090020000241	AO090020000232 - AO090020000247	AO090020000234 - AO090020000241	ECS, IGD	ECS, IGD
AO090020000380 cluster	AO090020000380	AO090020000380 - AO090020000386	AO090020000369 - AO090020000388	AO090020000366 - AO090020000387	ECS, FA	IGD, FA
AO090020000527 cluster	AO090020000527	AO090020000515 - AO090020000535	AO090020000521 - AO090020000531	-	n/a	n/a
AO090023000082 cluster	AO090023000082	AO090023000097 - AO090023000072	AO090023000096 - AO090023000074	AO090023000082 - AO090023000077	ECS	FA
AO090023000444 cluster	AO090023000444	AO090023000450 - AO090023000444	AO090023000450 - AO090023000436	AO090023000447 - AO090023000443	FA	ECS
AO090023000528 cluster	AO090023000528	AO090023000528 - AO090023000523	AO090023000536 - AO090023000521	AO090023000529 - AO090023000523	ECS, IGD	ECS
AO090023000877 cluster	AO090023000877	AO090023000881 - AO090023000875	AO090023000884 - AO090023000869	-	n/a	n/a
AO090026000009 (afl) cluster*	AO090026000009	AO090026000004 - AO090026000029	AO090026000001 - AO090026000015	AO090026000009 - AO090026000021	ED	ED
AO090026000149 cluster	AO090026000157	AO090026000149 - AO090026000157	AO090026000141 - AO090026000157	AO090026000147 - AO090026000157	ECS, IGD	ECS, IGD
AO090026000378 cluster	AO090026000378	AO090026000378 - AO090026000388	AO090026000369 - AO090026000390	AO090026000378 - AO090026000384	ECS	ECS, FA
AO090026000585 cluster	AO090026000585	AO090026000575 - AO090026000585	AO090026000575 - AO090026000591	AO090026000575 - AO090026000586	FA	FA
AO090038000086 cluster	AO090038000086	AO090038000078 - AO090038000086	AO090038000078 - AO090038000092	-	n/a	n/a
AO090038000098 cluster	AO090038000098	-	AO090038000092 - AO090038000101	AO090038000098 - AO090038000100	ECS	ECS
AO090038000149 cluster	AO090038000149	AO090038000144 - AO090038000149	AO090038000139 - AO090038000156	AO090038000145 - AO090038000151	IGD	IGD
AO090038000210 cluster	AO090038000210	-	AO090038000205 - AO090038000220	AO090038000210 - AO090038000214	FA	ECS, IGD
AO090038000390 cluster	AO090038000390	AO090038000390 - AO090038000399	AO090038000385 - AO090038000399	AO090038000390 - AO090038000392	ECS	FA
AO090038000488 cluster	AO090038000488	-	AO090038000480 - AO090038000492	AO090038000486 - AO090038000490	FA	IGD
AO090038000543 cluster	AO090038000543	AO090038000543 - AO090038000557	AO090038000540 - AO090038000558	AO090038000543 - AO090038000550	ECS, IGD	FA
AO090102000166 cluster	AO090102000166	AO090102000166 - AO090102000169	AO090102000157 - AO090102000173	AO090102000165 - AO090102000170	FA	FA
AO090102000322 cluster	AO090102000322	AO090102000316 - AO090102000322	AO090102000318 - AO090102000324	AO090102000317 - AO090102000322	IGD	ECS, IGD
AO090102000338 cluster	AO090102000338	AO090102000329 - AO090102000338	AO090102000331 - AO090102000342	AO090102000336 - AO090102000338	FA	ECS, IGD
AO090102000465 cluster	AO090102000465	AO090102000457 - AO090102000466	AO090102000457 - AO090012001037	AO090102000464 - AO090102000466	FA	ECS, IGD
AO090103000167 cluster	AO090103000167	AO090103000165 - AO090103000179	AO090103000159 - AO090103000177	AO090103000165 - AO090103000170	ECS,IGD	ECS
AO090103000224 cluster	AO090103000224	AO090103000222 - AO090103000227	AO090103000215 - AO090103000231	AO090103000220 - AO090103000226	ECS, FA	ECS
AO090103000355 cluster	AO090103000355	AO090103000355 - AO090103000366	AO090103000347 - AO090103000364	AO090103000352 - AO090103000361	FA	FA
AO090113000209 cluster	AO090113000209	AO090113000209 - AO090113000204	AO090113000208 - AO090113000199	-	n/a	n/a
AO090120000024 cluster	AO090120000024	AO090120000024 - AO090120000020	AO090120000033 - AO090120000013	AO090120000024 - AO090120000022	ECS	FA, ECS
AO090124000040 cluster	AO090124000040	-	AO090124000035 - AO090124000048	AO090124000035 - AO090124000040	ECS, FA	ECS
AO090206000074 cluster	AO090206000074	AO090206000075 - AO090206000074	AO090206000082 - AO090206000067	AO090206000074 - AO090206000072	ECS, IGD	ECS, IGD
AO090701000530 cluster	AO090701000530	AO090701000525 - AO090701000543	AO090701000525 - AO090701000539	AO090701000525 - AO090701000530	ECS, IGD	ECS, IGD
AO090701000600 cluster	AO090701000600	AO090701000600 - AO090701000603	AO090701000912 - AO090701000604	AO090701000597 - AO090701000600	ECS	ECS, IGD
AO090701000826 cluster	AO090701000826	AO090701000826 - AO090701000833	AO090701000817 - AO090701000917	AO090701000824 - AO090701000838	ECS, IGD	ECS
Non-PKS/NRPS cluster 1	n/a	-	AO090120000058 - AO090120000068	-	n/a	n/a
Non-PKS/NRPS cluster 2	n/a	-	AO090113000164 - AO090113000175	-	n/a	n/a

* Experimentally characterized; Abbreviations: *ECS*, End of cluster synteny; *IGD*, Increase in intergenic distance; *FA*, Change in functional annotation; *ED*, Experimentally determined.

**Table 8:** Number of gene clusters predicted by SMURF, antiSMASH, or manual and experimental methods

	**SMURF**	**antiSMASH**	**Manual**	**Experimental**	Total clusters predicted*
*A. nidulans*	49	66	63	9	71
*A. fumigatus*	33	38	29	5	39
*A. niger*	79	70	66	0	81
*A. oryzae*	57	73	62	2	75

* The number for total clusters predicted is the sum total of non-redundant clusters predicted by any method for a species. Manual boundary predictions include those that were experimentally detemined and published, or were made by evaluating published expression profile data (M. Andersen *et al*, 2013), cluster synteny among *Aspergillus* strains/species, changes in functional annotation and/or increases in intergenic distance, in that order of relative weight.

**Figure 2:**
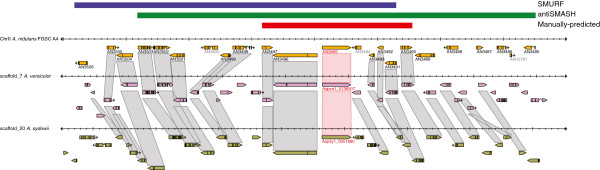
***A. nidulans *AN3497 gene cluster predicted based of gene expression analysis of Andersen et al. 2013.** Red bar indicates manually predicted cluster boundary (AN3490-AN3497) based on expression pattern and aligned with orthologous clusters of *A. versicolor* and *A. sydowii*. Blue bar indicates SMURF boundary prediction (AN3491-AN3506) and green bar indicates the antiSMASH-predicted boundary (AN3485-AN3503).

Andersen *et al.*[16] recently reported another strategy of identifying the extent of secondary metabolite gene cluster boundaries. Their method uses genome-wide microarray expression studies from *A. nidulans* to identify coregulated genes surrounding secondary metabolite gene cluster backbone enzymes. Since secondary metabolite gene clusters often show cryptic expression under many laboratory growth conditions, this study generated expression data from cultures grown on a wide variety of media (to maximize the possibility of expression), and combined these data with previously generated expression data to analyze a superset of 44 expression conditions [16]. Their analysis produced a list of 53 predicted secondary metabolite gene clusters of *A. nidulans*, some of which show clear patterns of coregulated expression while some of the expressed backbone enzymes showed no correlation with adjacent genes. Five of these were DTS-based gene clusters not identified by the SMURF or antiSMASH algorithms. These data have been curated at AspGD and were used as a criterion for our manual cluster boundary predictions (see below). An example of the *inpA*- and *inpB*-containing gene cluster determined by this criterion is shown in Figure 2. The gene clusters of *A. nidulans* with all of the boundary predictions made with ‘expression pattern’ as the primary evidence are listed in Table 4. The total number of boundaries predicted using this criterion is summarized in Table 9.

**Table 9:** Summary of primary criteria used for making manual secondary metabolite gene cluster boundary predictions

	**ED**	**EP**	**ECS**	**FA**	**IGD**
*A. nidulans*	24 (18%)	38 (29%)	47 (36%)	17 (13%)	6 (4%)
*A. fumigatus*	10 (15%)	n/a	39 (57%)	7 (10%)	12 (18%)
*A. niger*	0 (0%)	n/a	129 (98%)	2 (<2%)	1 (<1%)
*A. oryzae*	8 (6%)	n/a	90 (73%)	17 (14%)	9 (7%)

Abbreviations: *ED*, Experimentally determined; *EP*, Published expression pattern (M. Anderson *et al*, 2013); *ECS*, End of cluster synteny; *FA*, Change in functional annotation; *IGD*, Increase in intergenic distance; n/a, not applicable.

**Figure 3:**
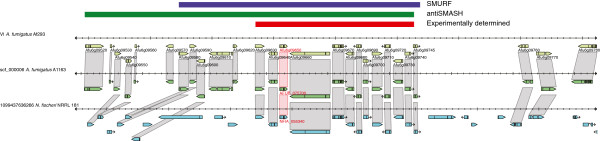
**Conserved cluster synteny between the gliotoxin cluster of *A. fumigatus *and the orthologous cluster of *Neosartorya fischeri*.** The predicted gene cluster is indicated with a red bar. The left border of the Afu6g09650 cluster shows a small increase in intergenic distance while the right border shows a large change in intergenic distance. Both borders are examples of interspecies cluster synteny. Red bar indicates experimentally determined cluster boundary (Afu6g09630 - Afu6g09740). Blue bar indicates SMURF boundary prediction (Afu6g09580 - Afu6g09740) and green bar indicates the antiSMASH-predicted boundary (Afu6g09520 - Afu6g09745).

To generate a high-quality set of candidate secondary metabolite biosynthetic gene clusters, we used SMURF and antiSMASH as the source of cluster predictions, along with manually predicted DTS clusters and then manually refined the gene cluster boundaries. Manual cluster boundary annotations (Tables 4, 5, 6, 7 and Additional files 2, 3, 4, 5) were made based on several criteria: published experimental data (including gene expression studies), synteny between clustered genes among different species indicated by the presence of conserved gene cluster boundaries (Figure 1), functional annotation of predicted genes within and adjacent to clusters and increases in intergenic distance between boundary genes and adjacent genes, which we frequently observed (Figure 3). We determined that gene clusters tend to be conserved between species and that breaks in cluster synteny frequently indicate a cluster boundary. To the best of our knowledge, no gene cluster prediction algorithm or research group has used genomic comparisons between species for large-scale cluster predictions. We used the Sybil viewer [51], which displays alignments of orthologous genes across multiple species in their genomic context, to manually examine potential boundaries and to compare synteny between clusters of different species and/or strains (Figure 1) and the adjacent syntenic regions outside each predicted cluster. The genome sequence is available for two strains each of *A. fumigatus* (Af293 and A1163) and *A. niger* (CBS513.88 and ATCC 1015), which allowed us to consider cluster synteny, which approached 100%, between these strains in addition to the orthology between *Aspergillus* species.

AspGD displays and provides sequence resources for 15 *Aspergillus* genomes and related species. A given genome is typically particularly closely related to that of one or two of the other species; the *A. fumigatus* genome best matches that of *Neosartorya fischeri* (see Sybil syntenic genomic context in Additional file 3), *A. niger* best matches *A. acidus* and *A. brasiliensis* (Additional file 4) and *A. oryzae* best matches *A. flavus* (Additional file 5). Unlike *A. fumigatus*, *A. niger* and *A. oryzae*, *A. nidulans* lacks such a closely related species in AspGD with sufficient synteny to enable routine use of cluster orthology in boundary determination. Therefore, we used other criteria such as published gene expression patterns [16], increases in intergenic distance and changes from secondary metabolism-related gene annotations to non-secondary metabolism-related gene annotations (described below) for making these predictions in *A. nidulans* (Figure 1). The numbers of manually predicted gene clusters in each of these additional species, determined by observing breaks in gene cluster synteny (see Methods), are summarized in Table 9.

In some cases, the functional annotation of the putative gene cluster members was informative in predicting cluster boundaries, especially for *A. nidulans,* which often lacked cluster synteny with other species present in AspGD. In addition to genes encoding the core backbone enzymes, clusters typically include one or more acyl transferase, oxidoreductase, hydrolase, cytochrome P450, transmembrane transporter and a transcription factor. We manually inspected each cluster and the genomic region surrounding it; changes in functional annotations from typical secondary metabolism annotations to annotations atypical of secondary metabolic processes were frequently observed upon traversing a cluster boundary (Additional files 2, 3, 4, 5) and this was used as an additional criterion for boundary prediction, especially in cases where inter- or intra-species clustering or published gene expression data were not available. In some instances, genes with functional annotations unrelated to secondary metabolism are embedded within a cluster. For example, *A. nidulans bglD* (AN7915) encodes a glucosidase present in the F9775 biosynthetic gene cluster (Additional file 2). In a *cclA*Δ strain background in which histone 3 lysine 4 methylation is impaired, the expression of cryptic secondary metabolite clusters, such as F9775, is activated [52]. The activation of *bglD* expression was observed along with other genes in the F9775 cluster and based on this pattern of coregulation, *bglD* is included as a member of this cluster [52]. It is unclear, however, whether *bglD* actually plays a role in F9775 biosynthesis. The gene encoding translation elongation factor 1 gamma, *stcT*, is a member of the ST gene cluster (stc) of *A. nidulans.* Its inclusion in the stc cluster was based on its pattern of coregulation with 24 other genes, some of which have experimentally determined roles in *A. nidulans* ST biosynthesis, or are orthologous to *A. parasiticus* proteins involved in AF production, for which ST is a precursor [46]*.* We also observed a gene, AN2546, that is expressed, and is predicted to encode a glycosylphosphatidylinositol (GPI)-anchored protein [53], located in the emericellamide cluster (Additional file 2); however, an AN2546 deletion strain still produces emericellamide, thus its inclusion in the cluster is based on its genomic location and expression pattern rather than function. These examples indicate that some genes are located within clusters and yet may not contribute to secondary metabolite production. The frequency and significance of unrelated genes that have become incorporated into a secondary metabolism gene cluster remains unclear; experimental verification is needed to further assess these. In cases where the cluster synteny data were compelling, cluster synteny was given higher precedence than functional annotation in the delineation of the cluster boundaries.

Increases in the distance between predicted boundary genes and the gene directly adjacent to a boundary (which we refer to as intergenic distance) were frequently observed. An example with a large intergenic distance at the right boundary is shown in the *A. fumigatus* gliotoxin (gli) cluster (Figure 3). However, we found that more subtle increases in intergenic distance were only somewhat reliable when compared to boundaries with experimental evidence. We therefore only based a cluster boundary prediction on an increase in intergenic distance in a small number of cases where no other data were available (Table 9).

## Discussion

AspGD provides high-quality manual and computational gene structure and function annotations for *A. nidulans*, *A. fumigatus*, *A. niger* and *A. oryzae,* along with sequence analysis and visualization resources for these and additional *Aspergilli* and related species. Among fungal databases, AspGD is the only resource performing comprehensive manual literature curation for *Aspergillus* species. AspGD contains curated data covering the entire corpus of experimental literature for *A. nidulans*, *A. fumigatus*, *A. niger* and *A. oryzae*, with phenotype and GO annotations for every gene described in the literature for these species, including those related to secondary metabolism. The direct, manual curation of genes from the literature forms the basis for the computational annotations at AspGD. This information, collected in a centralized, freely accessible resource, provides an indispensible resource for scientific information for researchers.

During the course of curation, we identified gaps in the set of GO terms that were available in the Biological Process branch of the ontology. To improve the GO annotations for secondary metabolite biosynthetic genes, we added new, more specific BP terms to the GO and used these new terms for direct annotation of *Aspergillus* genes. These terms include the specific secondary metabolite in each GO term name. Because ‘secondary metabolic process’ (GO:0019748) and ‘regulation of secondary metabolite biosynthetic process’ (GO:0043455) map to different branches in the GO hierarchy, complete annotation of transcriptional regulators of secondary metabolite biosynthetic gene clusters, such as *laeA*, requires an additional annotation to the regulatory term that we also added for each secondary metabolite.

GO annotations facilitate predictions of gene function across multiple species and, as part of this project, we used orthology relationships between experimentally characterized *A. nidulans*, *A. fumigatus*, *A. niger* and *A. oryzae* genes to provide orthology-based GO predictions for the unannotated secondary metabolism-related genes in AspGD. The prediction and complete cataloging of these candidate secondary metabolism-related genes will facilitate future experimental studies and, ultimately, the identification of all secondary metabolites and the corresponding secondary metabolism genes in *Aspergillus* and other species.

The SMURF and antiSMASH algorithms are efficient at predicting gene clusters on the basis of the presence of certain canonical backbone enzymes; however, disparities between boundaries predicted by these methods became obvious when the clusters predicted by each method were aligned. While there was an extensive overlap between the two sets of identified clusters, in most cases the cluster boundaries predicted by SMURF and antiSMASH were different, requiring manual refinement.

The data analysis of Andersen *et al.*[16] used a clustering matrix to identify superclusters, defined as clusters with similar expression, independent of chromosomal location, that are predicted to participate in cross-chemistry between clusters to synthesize a single secondary metabolite. They identified seven superclusters of *A. nidulans*. Two known meroterpenoid clusters that exhibit cross-chemistry, and are located on separate chromosomes, are the austinol (aus) clusters involved in the synthesis of austinol and dehydroaustinol [31,37]. The biosynthesis of prenyl xanthones in *A. nidulans* is dependent on three separate gene clusters [36]. This was apparent because the *mdpG* gene cluster was shown to be required for the synthesis of the anthraquinone emodin, monodictyphenone, and related compounds. Emodin and monodictyphenone are precursors of prenyl xanthones and the *mdpG* cluster lacked a prenyltransferase, required for prenyl xanthone synthesis [36]. A search of the *A. nidulans* genome for prenyltransferases that may participate in prenyl xanthone synthesis predicts seven prenyltransferases. Two strains (Δ*xptA* and Δ*xptB*) with mutated prenyltransferase genes at chromosomal locations distant from the *mdpG* cluster, have been described as being defective in prenyl xanthone synthesis. Therefore, while a total of 266 unique clusters were identified in our analysis, published data indicate that some of these clusters may function as superclusters that display cross-chemistry synthesis of a single secondary metabolite or group of related secondary metabolites [16,31,36].

Our manual annotation of secondary metabolite gene clusters in four *Aspergillus* species complements the computational prediction methods for identifying fungal secondary metabolites and the genes responsible for their biosynthesis. Implicit in our interspecies cluster synteny analysis is the prediction of secondary metabolite gene clusters orthologous to those in our curated species. For example, *A. nidulans* gene clusters most closely matched those in *A. versicolor*, thus identifying several new predicted *A. versicolor* gene clusters by orthology and interspecies cluster synteny with the predicted *A. nidulans* clusters (Additional file 2).

## Conclusions

These new curated data, based on both computational analysis and manual evaluation of the *Aspergillus* genomes, provide researchers with a comprehensive set of annotated secondary metabolite gene clusters and a comprehensive functional annotation of the secondary metabolite gene products within AspGD. We anticipate that these new data will promote research in this important and complex area of *Aspergillus* biology.

## Methods

### Generation of new GO terms

The Gene Ontology Consortium requires that any compounds within BP term names in the GO be cataloged in the Chemical Entities of Biological Interest (ChEBI) database (http://www.ebi.ac.uk/chebi/). To enable the creation of the new GO terms, we first requested and were assigned ChEBI identifiers for all secondary metabolites recorded in AspGD. Once ChEBI term identifiers were assigned, the relevant GO terms were requested from the GO Consortium through TermGenie (http://go.termgenie.org/) for biosynthetic process, metabolic process and catabolic process terms for each new secondary metabolic process term and regulation of secondary metabolic process term (Additional file 1).

### Orthologous protein predictions

Jaccard-clustering, which groups together highly similar proteins within a genome of interest, was used to make ortholog predictions between the *Aspergillus* species and is described in detail at http://sybil.sourceforge.net/documentation.html#jaccard. Briefly, the first step of this algorithm identifies highly similar proteins within each genome of interest. The resulting groups (“clusters”) from multiple genomes are themselves grouped in the second step to form orthologous groups (“Jaccard Orthologous Clusters”). The corresponding genes can be subsequently analyzed in their genomic context to visually identify conserved synteny blocks that are displayed in the Sybil genome viewer (aspgd.broadinstitute.org). The ortholog predictions for all AspGD species are available for download at http://www.aspergillusgenome.org/download/homology/orthologs/. Orthologous protein predictions between *Saccharomyces cerevisiae*, *Schizosaccharomyces pombe* and the *Aspergillus* protein sets were made by pair-wise comparisons using the InParanoid software [54]. InParanoid was chosen based on compatibility with the existing ortholog analysis pipeline at AspGD, and comparable accuracy when compared with alternative methods [55]. Stringent cutoffs were used: BLOSUM80 and an InParanoid score of 100% (parameters: -F \“m S\” -M BLOSUM80). The data from this comparison are available for download at (http://www.aspergillusgenome.org/download/homology/).

### Orthology- and domain-based GO transfer

To augment the annotations for all genes, including secondary metabolism related genes, we used manual and domain-based GO annotations to annotate the predicted orthologs that lacked direct experimental characterization. Ortholog predictions for *A. nidulans, A. fumigatus, A. niger* and *A. oryzae* were made based on the characterized proteins of *S. cerevisiae*, *S. pombe* and the other *Aspergillus* species in AspGD. Candidate GO annotations to be used as the basis for these inferences are limited to those with experimental evidence, that is, with evidence codes of IDA (Inferred from Direct Assay), IPI (Inferred from Physical Interaction), IGI (Inferred from Genetic Interaction) or IMP (Inferred from Mutant Phenotype). Annotations that are themselves predicted in *S. cerevisiae, S. pombe* or in *Aspergillus,* either based on sequence similarity or by some other methods, are excluded from this group to avoid transitive propagation of predictions. Also excluded from the predicted annotation set are annotations that are redundant with existing, manually curated annotations or those that assign a related but less specific GO term. The orthology-based GO assignments are given the evidence code IEA (Inferred from Electronic Annotation) and displayed with the source species and name of the gene from which they were derived, along with a hyperlink to the appropriate gene page at AspGD, SGD or PomBase. The new annotations that have been manually assigned or electronically transferred from *S. cerevisiae* and *S. pombe* to *A. nidulans*, *A. fumigatus*, *A. niger* and *A. oryzae*, and between the *Aspergillus* species are summarized in Table 3.

Domain-based GO transfers were assigned to a lower precedence than orthology-based transfers. IprScan predicts InterPro domains based on protein sequences [56]. The Interpro2go mapping file (http://www.ebi.ac.uk/interpro) was used to map GO annotations to genes with the corresponding domain predictions. A domain-based GO prediction was made only if it was not redundant with an existing manually-curated or orthology-based GO term, or one of its parental terms, that was already assigned to an orthologous protein.

Finally, descriptions for genes lacking manual or GO-based annotations were constructed from the manual GO terms assigned to characterized orthologs. GO annotations were included with the following precedence: BP, followed by MF, and then CC. For genes that lacked experimental characterization and characterized orthologs, but had functionally characterized InterPro domains, descriptions were generated from the domain-based GO annotations. The same precedence rules applied as to the descriptions generated using orthology-based GO information. For genes that lacked experimental characterization and characterized orthologs, and without functionally characterized InterPro domains, but had uncharacterized orthologs, the descriptions simply list the orthology relationship because no inferred GO information was available.

### Secondary metabolic gene cluster analysis and annotation

The pre-computed results file (smurf_output_precomputed_08.13.08.zip) was downloaded from the SMURF website (http://jcvi.org/smurf/index.php). Version 1.2.1 of the antiSMASH program [39] was downloaded from (http://antismash.secondarymetabolites.org/) and run locally on the chromosome and/or contig sequences of *A. nidulans* FGSC A4, *A. fumigatus* Af293, *A. niger* CBS 513.88 and *A. oryzae* RIB40. Details of the parameters the antiSMASH program uses to predict boundaries are in described in Medema et al. 1998 [39] and those for SMURF are described in Khaldi et al. 2010 [38]. The secondary metabolic gene clusters predicted by these programs were manually analyzed and annotated using functional data available for each gene in AspGD. Cluster membership was determined based on physical proximity of candidate genes to cluster backbone genes. Adjacent genes were added to the cluster if they had functional annotations common to known secondary metabolism genes. In cases where backbone genes had Jaccard orthologs in other species (see above), we required orthology between all other cluster members. Confirmation of orthology between clusters was facilitated by use of the Sybil multiple genome browser which can be used to evaluate synteny between species. We visually evaluated synteny by examining whether a gene that was putatively in a cluster had orthologs in the other species – where a gene in the species in which the cluster was identified no longer had orthologs in the other species that were adjacent, we inferred a break in synteny. Cluster boundaries were also determined by changes in common functional annotation, or by an increase in intergenic distances. tRNAs and other non-coding RNAs were excluded in cluster boundary analysis. Annotated images of the orthologous gene clusters are included in Additional files 2, 3, 4, 5.

## Competing interests

The authors declare that they have no competing interests.

## Authors’ contributions

DOI, MBA and MSS designed the project, DOI wrote the manuscript, GS, JRW, MBA and MSS edited the manuscript, DOI and MSS analyzed the data, DOI and MSS annotated the data, JB, GC, PS and FW performed bioinformatics analysis of the data. All authors read and approved the final manuscript.

## Supplementary Material

Additional file 1 Contains a table listing all GO terms available from the GO Consortium describing fungal secondary metabolic processes as of December 2012. Click here for file

Additional file 2 **Contains a table listing the manually annotated gene clusters predicted by SMURF and antiSMASH for *A. nidulans.*** Click here for file

Additional file 3 **Contains a table listing manually annotated gene clusters predicted by SMURF and antiSMASH for *A. fumigatus.*** Click here for file

Additional file 4 **A table listing the manually annotated gene clusters predicted by SMURF and antiSMASH for *A. niger.*** Click here for file

Additional file 5 **A table listing manually annotated gene clusters predicted by SMURF and antiSMASH for *A. oryzae.*** Click here for file
