# Supplementary material for: Comprehensive annotation of secondary metabolite biosynthetic genes and gene clusters of Aspergillus nidulans, A. fumigatus, A. niger and A. oryzae
Source: BMC Microbiol. 2013 Apr 26;13:91. doi: 10.1186/1471-2180-13-91 (PMC3689640; doi:10.1186/1471-2180-13-91)
Supplement: Additional file 1 — Contains a table listing all GO terms available from the GO Consortium describing fungal secondary metabolic processes as of December 2012. [file 1471-2180-13-91-S1.doc]

Additional file 1. Metabolic triads of terms for secondary metabolism added to the Biological Process branch of the GO*.

| **GO terms for secondary metabolism** | |
| --- | --- |
| * previously available term | |
|  |  |
| **GOID** | **GO term name** |
| GO:0036146 | cellular response to mycotoxin |
| GO:0046222 | aflatoxin metabolic process* |
| GO:0046223 | aflatoxin catabolic process* |
| GO:0045122 | aflatoxin biosynthetic process* |
| GO:1900177 | regulation of aflatoxin biosynthetic process |
| GO:1900178 | negative regulation of aflatoxin biosynthetic process |
| GO:1900179 | positive regulation of aflatoxin biosynthetic process |
| GO:1900585 | arugosin metabolic process |
| GO:1900586 | arugosin catabolic process |
| GO:1900587 | arugosin biosynthetic process |
| GO:1900626 | regulation of arugosin biosynthetic process |
| GO:1900627 | negative regulation of arugosin biosynthetic process |
| GO:1900628 | positive regulation of arugosin biosynthetic process |
| GO:1900552 | asperfuranone metabolic process |
| GO:1900553 | asperfuranone catabolic process |
| GO:1900554 | asperfuranone biosynthetic process |
| GO:1900637 | regulation of asperfuranone biosynthetic process |
| GO:1900638 | negative regulation of asperfuranone biosynthetic process |
| GO:1900639 | positive regulation of asperfuranone biosynthetic process |
| GO:0036182 | asperthecin metabolic process |
| GO:0036183 | asperthecin catabolic process |
| GO:0036184 | asperthecin biosynthetic process |
| GO:1900379 | regulation of asperthecin biosynthetic process |
| GO:1900380 | negative regulation of asperthecin biosynthetic process |
| GO:1900381 | positive regulation of asperthecin biosynthetic process |
| GO:1901516 | aspyridone A metabolic process |
| GO:1901517 | aspyridone A catabolic process |
| GO:1901518 | aspyridone A biosynthetic process |
| GO:1901519 | aspyridone B metabolic process |
| GO:1901520 | aspyridone B catabolic process |
| GO:1901521 | aspyridone B biosynthetic process |
| GO:1900558 | austinol metabolic process |
| GO:1900559 | austinol catabolic process |
| GO:1900560 | austinol biosynthetic process |
| GO:1900640 | regulation of austinol biosynthetic process |
| GO:1900641 | negative regulation of austinol biosynthetic process |
| GO:1900642 | positive regulation of austinol biosynthetic process |
| GO:1900761 | averantin metabolic process |
| GO:1900762 | averantin catabolic process |
| GO:1900763 | averantin biosynthetic process |
| GO:1900803 | brevianamide F metabolic process |
| GO:1900804 | brevianamide F catabolic process |
| GO:1900805 | brevianamide F biosynthetic process |
| GO:1900858 | regulation of brevianamide F biosynthetic process |
| GO:1900859 | negative regulation of brevianamide F biosynthetic process |
| GO:1900860 | positive regulation of brevianamide F biosynthetic process |
| GO:1900564 | chanoclavine-I metabolic process |
| GO:1900565 | chanoclavine-I catabolic process |
| GO:1900566 | chanoclavine-I biosynthetic process |
| GO:1900643 | regulation of chanoclavine-I biosynthetic process |
| GO:1900644 | negative regulation of chanoclavine-I biosynthetic process |
| GO:1900645 | positive regulation of chanoclavine-I biosynthetic process |
| GO:1900567 | chanoclavine-I aldehyde metabolic process |
| GO:1900568 | chanoclavine-I aldehyde catabolic process |
| GO:1900569 | chanoclavine-I aldehyde biosynthetic process |
| GO:1900646 | regulation of chanoclavine-I aldehyde biosynthetic process |
| GO:1900647 | negative regulation of chanoclavine-I aldehyde biosynthetic process |
| GO:1900648 | positive regulation of chanoclavine-I aldehyde biosynthetic process |
| GO:1900797 | cordyol C metabolic process |
| GO:1900798 | cordyol C catabolic process |
| GO:1900799 | cordyol C biosynthetic process |
| GO:1900861 | regulation of cordyol C biosynthetic process |
| GO:1900862 | negative regulation of cordyol C biosynthetic process |
| GO:1900863 | positive regulation of cordyol C biosynthetic process |
| GO:1900800 | cspyrone B1 metabolic process |
| GO:1900801 | cspyrone B1 catabolic process |
| GO:1900802 | cspyrone B1 biosynthetic process |
| GO:1900561 | dehydroaustinol metabolic process |
| GO:1900562 | dehydroaustinol catabolic process |
| GO:1900563 | dehydroaustinol biosynthetic process |
| GO:1900649 | regulation of dehydroaustinol biosynthetic process |
| GO:1900650 | negative regulation of dehydroaustinol biosynthetic process |
| GO:1900651 | positive regulation of dehydroaustinol biosynthetic process |
| GO:1900597 | demethylkotanin metabolic process |
| GO:1900598 | demethylkotanin catabolic process |
| GO:1900599 | demethylkotanin biosynthetic process |
| GO:1900652 | regulation of demethylkotanin biosynthetic process |
| GO:1900653 | negative regulation of demethylkotanin biosynthetic process |
| GO:1900654 | positive regulation of demethylkotanin biosynthetic process |
| GO:1900570 | diorcinol metabolic process |
| GO:1900571 | diorcinol catabolic process |
| GO:1900572 | diorcinol biosynthetic process |
| GO:1900655 | regulation of diorcinol biosynthetic process |
| GO:1900656 | negative regulation of diorcinol biosynthetic process |
| GO:1900657 | positive regulation of diorcinol biosynthetic process |
| GO:1900555 | emericellamide metabolic process |
| GO:1900556 | emericellamide catabolic process |
| GO:1900557 | emericellamide biosynthetic process |
| GO:1900658 | regulation of emericellamide biosynthetic process |
| GO:1900659 | negative regulation of emericellamide biosynthetic process |
| GO:1900660 | positive regulation of emericellamide biosynthetic process |
| GO:1900615 | emericellamide A metabolic process |
| GO:1900616 | emericellamide A catabolic process |
| GO:1900617 | emericellamide A biosynthetic process |
| GO:1900661 | regulation of emericellamide A biosynthetic process |
| GO:1900662 | negative regulation of emericellamide A biosynthetic process |
| GO:1900663 | positive regulation of emericellamide A biosynthetic process |
| GO:1900764 | emericellin metabolic process |
| GO:1900765 | emericellin catabolic process |
| GO:1900766 | emericellin biosynthetic process |
| GO:1900834 | regulation of emericellin biosynthetic process |
| GO:1900835 | negative regulation of emericellin biosynthetic process |
| GO:1900836 | positive regulation of emericellin biosynthetic process |
| GO:1900573 | emodin metabolic process |
| GO:1900574 | emodin catabolic process |
| GO:1900575 | emodin biosynthetic process |
| GO:1900664 | regulation of emodin biosynthetic process |
| GO:1900665 | negative regulation of emodin biosynthetic process |
| GO:1900666 | positive regulation of emodin biosynthetic process |
| GO:1900600 | endocrocin metabolic process |
| GO:1900601 | endocrocin catabolic process |
| GO:1900602 | endocrocin biosynthetic process |
| GO:1900667 | regulation of endocrocin biosynthetic process |
| GO:1900668 | negative regulation of endocrocin biosynthetic process |
| GO:1900669 | positive regulation of endocrocin biosynthetic process |
| GO:1901539 | ent-pimara-8(14),15-diene metabolic process |
| GO:1901540 | ent-pimara-8(14),15-diene catabolic process |
| GO:1901541 | ent-pimara-8(14),15-diene biosynthetic process |
| GO:1901542 | regulation of ent-pimara-8(14),15-diene biosynthetic process |
| GO:1901543 | negative regulation of ent-pimara-8(14),15-diene biosynthetic process |
| GO:1901544 | positive regulation of ent-pimara-8(14),15-diene biosynthetic process |
| GO:0035836 | ergot alkaloid metabolic process* |
| GO:1900806 | ergot alkaloid catabolic process |
| GO:0035837 | ergot alkaloid biosynthetic process* |
| GO:1900823 | negative regulation of ergot alkaloid biosynthetic process |
| GO:1900824 | positive regulation of ergot alkaloid biosynthetic process |
| GO:1900609 | F-9775A metabolic process |
| GO:1900610 | F-9775A catabolic process |
| GO:1900611 | F-9775A biosynthetic process |
| GO:1900670 | regulation of F-9775A biosynthetic process |
| GO:1900671 | negative regulation of F-9775A biosynthetic process |
| GO:1900672 | positive regulation of F-9775A biosynthetic process |
| GO:1900612 | F-9775B metabolic process |
| GO:1900613 | F-9775B catabolic process |
| GO:1900614 | F-9775B biosynthetic process |
| GO:1900675 | regulation of F-9775B biosynthetic process |
| GO:1900676 | negative regulation of F-9775B biosynthetic process |
| GO:1900677 | positive regulation of F-9775B biosynthetic process |
| GO:0031170 | ferricrocin metabolic process |
| GO:0031171 | ferricrocin biosynthetic process |
| GO:1900678 | regulation of ferricrocin biosynthetic process |
| GO:1900679 | negative regulation of ferricrocin biosynthetic process |
| GO:1900680 | positive regulation of ferricrocin biosynthetic process |
| GO:1900767 | fonsecin metabolic process |
| GO:1900768 | fonsecin catabolic process |
| GO:1900769 | fonsecin biosynthetic process |
| GO:1900807 | fumigaclavine C metabolic process |
| GO:1900808 | fumigaclavine C catabolic process |
| GO:1900809 | fumigaclavine C biosynthetic process |
| GO:1900837 | regulation of fumigaclavine C biosynthetic process |
| GO:1900838 | negative regulation of fumigaclavine C biosynthetic process |
| GO:1900839 | positive regulation of fumigaclavine C biosynthetic process |
| GO:1900770 | fumitremorgin B metabolic process |
| GO:1900771 | fumitremorgin B catabolic process |
| GO:1900772 | fumitremorgin B biosynthetic process |
| GO:1900855 | regulation of fumitremorgin B biosynthetic process |
| GO:1900856 | negative regulation of fumitremorgin B biosynthetic process |
| GO:1900857 | positive regulation of fumitremorgin B biosynthetic process |
| GO:1900773 | fumiquinazoline metabolic process |
| GO:1900774 | fumiquinazoline catabolic process |
| GO:1900775 | fumiquinazoline biosynthetic process |
| GO:1900776 | fumiquinazoline A metabolic process |
| GO:1900777 | fumiquinazoline A catabolic process |
| GO:1900778 | fumiquinazoline A biosynthetic process |
| GO:1900779 | fumiquinazoline C metabolic process |
| GO:1900780 | fumiquinazoline C catabolic process |
| GO:1900781 | fumiquinazoline C biosynthetic process |
| GO:1900782 | fumiquinazoline F metabolic process |
| GO:1900783 | fumiquinazoline F catabolic process |
| GO:1900784 | fumiquinazoline F biosynthetic process |
| GO:1900539 | fumonisin metabolic process |
| GO:1900540 | fumonisin catabolic process |
| GO:1900541 | fumonisin biosynthetic process |
| GO:1900683 | regulation of fumonisin biosynthetic process |
| GO:1900684 | negative regulation of fumonisin biosynthetic process |
| GO:1900685 | positive regulation of fumonisin biosynthetic process |
| GO:1900576 | gerfelin metabolic process |
| GO:1900577 | gerfelin catabolic process |
| GO:1900578 | gerfelin biosynthetic process |
| GO:1900686 | regulation of gerfelin biosynthetic process |
| GO:1900687 | negative regulation of gerfelin biosynthetic process |
| GO:1900688 | positive regulation of gerfelin biosynthetic process |
| GO:2001308 | gliotoxin metabolic process |
| GO:2001309 | gliotoxin catabolic process |
| GO:2001310 | gliotoxin biosynthetic process |
| GO:1900689 | regulation of gliotoxin biosynthetic process |
| GO:1900690 | negative regulation of gliotoxin biosynthetic process |
| GO:1900691 | positive regulation of gliotoxin biosynthetic process |
| GO:1900810 | helvolic acid metabolic process |
| GO:1900811 | helvolic acid catabolic process |
| GO:1900812 | helvolic acid biosynthetic process |
| GO:1900840 | regulation of helvolic acid biosynthetic process |
| GO:1900841 | negative regulation of helvolic acid biosynthetic process |
| GO:1900842 | positive regulation of helvolic acid biosynthetic process |
| GO:1900594 | (+)-kotanin metabolic process |
| GO:1900595 | (+)-kotanin catabolic process |
| GO:1900596 | (+)-kotanin biosynthetic process |
| GO:1900692 | regulation of (+)-kotanin biosynthetic process |
| GO:1900693 | negative regulation of (+)-kotanin biosynthetic process |
| GO:1900694 | positive regulation of (+)-kotanin biosynthetic process |
| GO:0006582 | melanin metabolic process* |
| GO:0046150 | melanin catabolic process* |
| GO:0042438 | melanin biosynthetic process* |
| GO:0048021 | regulation of melanin biosynthetic process* |
| GO:0048022 | negative regulation of melanin biosynthetic process* |
| GO:0048023 | positive regulation of melanin biosynthetic process* |
| GO:1901510 | (-)-microperfuranone metabolic process |
| GO:1901511 | (-)-microperfuranone catabolic process |
| GO:1901512 | (-)-microperfuranone biosynthetic process |
| GO:1900813 | monodictyphenone metabolic process |
| GO:1900814 | monodictyphenone catabolic process |
| GO:1900815 | monodictyphenone biosynthetic process |
| GO:1900843 | regulation of monodictyphenone biosynthetic process |
| GO:1900844 | negative regulation of monodictyphenone biosynthetic process |
| GO:1900845 | positive regulation of monodictyphenone biosynthetic process |
| GO:1900785 | naphtho-gamma-pyrone metabolic process |
| GO:1900786 | naphtho-gamma-pyrone catabolic process |
| GO:1900787 | naphtho-gamma-pyrone biosynthetic process |
| GO:1900846 | regulation of naphtho-gamma-pyrone biosynthetic process |
| GO:1900847 | negative regulation of naphtho-gamma-pyrone biosynthetic process |
| GO:1900848 | positive regulation of naphtho-gamma-pyrone biosynthetic process |
| GO:1900549 | N',N'',N'''-triacetylfusarinine C metabolic process |
| GO:1900550 | N',N'',N'''-triacetylfusarinine C catabolic process |
| GO:1900551 | N',N'',N'''-triacetylfusarinine C biosynthetic process |
| GO:1900695 | regulation of N',N'',N'''-triacetylfusarinine C biosynthetic process |
| GO:1900696 | negative regulation of N',N'',N'''-triacetylfusarinine C biosynthetic process |
| GO:1900697 | positive regulation of N',N'',N'''-triacetylfusarinine C biosynthetic process |
| GO:1900816 | ochratoxin A metabolic process |
| GO:1900817 | ochratoxin A catabolic process |
| GO:1900818 | ochratoxin A biosynthetic process |
| GO:1900819 | orlandin metabolic process |
| GO:1900820 | orlandin catabolic process |
| GO:1900821 | orlandin biosynthetic process |
| GO:1900582 | o-orsellinic acid metabolic process |
| GO:1900583 | o-orsellinic acid catabolic process |
| GO:1900584 | o-orsellinic acid biosynthetic process |
| GO:1900698 | regulation of o-orsellinic acid biosynthetic process |
| GO:1900699 | negative regulation of o-orsellinic acid biosynthetic process |
| GO:1900700 | positive regulation of o-orsellinic acid biosynthetic process |
| GO:0018940 | orcinol metabolic process |
| GO:0042209 | orcinol catabolic process |
| GO:0046197 | orcinol biosynthetic process |
| GO:1900701 | regulation of orcinol biosynthetic process |
| GO:1900702 | negative regulation of orcinol biosynthetic process |
| GO:1900703 | positive regulation of orcinol biosynthetic process |
| GO:0042316 | penicillin metabolic process* |
| GO:0042317 | penicillin catabolic process* |
| GO:0042318 | penicillin biosynthetic process* |
| GO:1900196 | regulation of penicillin biosynthetic process |
| GO:1900197 | negative regulation of penicillin biosynthetic process |
| GO:1900198 | positive regulation of penicillin biosynthetic process |
| GO:0030638 | polyketide metabolic process* |
| GO:0030640 | polyketide catabolic process* |
| GO:0030639 | polyketide biosynthetic process* |
| GO:1900732 | regulation of polyketide biosynthetic process |
| GO:1900733 | negative regulation of polyketide biosynthetic process |
| GO:1900734 | positive regulation of polyketide biosynthetic process |
| GO:1900579 | (17Z)-protosta-17(20),24-dien-3beta-ol metabolic process |
| GO:1900580 | (17Z)-protosta-17(20),24-dien-3beta-ol catabolic process |
| GO:1900581 | (17Z)-protosta-17(20),24-dien-3beta-ol biosynthetic process |
| GO:0019748 | secondary metabolic process* |
| GO:0043455 | regulation of secondary metabolic process* |
| GO:0090487 | secondary metabolite catabolic process |
| GO:0044550 | secondary metabolite biosynthetic process |
| GO:1900376 | regulation of secondary metabolite biosynthetic process |
| GO:1900377 | negative regulation of secondary metabolite biosynthetic process |
| GO:1900378 | positive regulation of secondary metabolite biosynthetic process |
| GO:1900791 | shamixanthone metabolic process |
| GO:1900792 | shamixanthone catabolic process |
| GO:1900793 | shamixanthone biosynthetic process |
| GO:0045460 | sterigmatocystin metabolic process* |
| GO:0045574 | sterigmatocystin catabolic process* |
| GO:0045461 | sterigmatocystin biosynthetic process* |
| GO:0010913 | regulation of sterigmatocystin biosynthetic process |
| GO:1900760 | negative regulation of sterigmatocystin biosynthetic process |
| GO:0010914 | positive regulation of sterigmatocystin biosynthetic process |
| GO:0009237 | siderophore metabolic process* |
| GO:0046215 | siderophore catabolic process* |
| GO:0019290 | siderophore biosynthetic process* |
| GO:1900704 | regulation of siderophore biosynthetic process |
| GO:1900705 | negative regulation of siderophore biosynthetic process |
| GO:1900706 | positive regulation of siderophore biosynthetic process |
| GO:1900603 | tensidol A metabolic process |
| GO:1900604 | tensidol A catabolic process |
| GO:1900605 | tensidol A biosynthetic process |
| GO:1900707 | regulation of tensidol A biosynthetic process |
| GO:1900708 | negative regulation of tensidol A biosynthetic process |
| GO:1900709 | positive regulation of tensidol A biosynthetic process |
| GO:1900606 | tensidol B metabolic process |
| GO:1900607 | tensidol B catabolic process |
| GO:1900608 | tensidol B biosynthetic process |
| GO:1900710 | regulation of tensidol B biosynthetic process |
| GO:1900711 | negative regulation of tensidol B biosynthetic process |
| GO:1900712 | positive regulation of tensidol B biosynthetic process |
| GO:1900794 | terrequinone A metabolic process |
| GO:1900795 | terrequinone A catabolic process |
| GO:1900796 | terrequinone A biosynthetic process |
| GO:1900852 | regulation of terrequinone A biosynthetic process |
| GO:1900853 | negative regulation of terrequinone A biosynthetic process |
| GO:1900854 | positive regulation of terrequinone A biosynthetic process |
| GO:1900588 | violaceol I metabolic process |
| GO:1900589 | violaceol I catabolic process |
| GO:1900590 | violaceol I biosynthetic process |
| GO:1900713 | regulation of violaceol I biosynthetic process |
| GO:1900714 | negative regulation of violaceol I biosynthetic process |
| GO:1900715 | positive regulation of violaceol I biosynthetic process |
| GO:1900591 | violaceol II metabolic process |
| GO:1900592 | violaceol II catabolic process |
| GO:1900593 | violaceol II biosynthetic process |
| GO:1900716 | regulation of violaceol II biosynthetic process |
| GO:1900717 | negative regulation of violaceol II biosynthetic process |
| GO:1900718 | positive regulation of violaceol II biosynthetic process |
| GO:2001307 | xanthone-containing compound biosynthetic process |
| GO:1900183 | regulation of xanthone-containing compound biosynthetic process |
| GO:1900184 | negative regulation of xanthone-containing compound biosynthetic process |
| GO:1900185 | positive regulation of xanthone-containing compound biosynthetic process |

* as of December 2012
