# Supplementary material for: Comprehensive annotation of secondary metabolite biosynthetic genes and gene clusters of Aspergillus nidulans, A. fumigatus, A. niger and A. oryzae
Source: BMC Microbiol. 2013 Apr 26;13:91. doi: 10.1186/1471-2180-13-91 (PMC3689640; doi:10.1186/1471-2180-13-91)
Supplement: Additional file 2 — Contains a table listing the manually annotated gene clusters predicted by SMURF and antiSMASH for A. nidulans. [file 1471-2180-13-91-S2.pdf]

Additional file 2

Comprehensive annotation of secondary metabolite biosynthetic genes and gene clusters of *Aspergillus nidulans*, *A. fumigatus*, *A. niger* and *A. oryzae*

Diane O. Inglis, Jon Binkley, Marek Skrzypek, Martha B. Arnaud, Gustavo Cerqueira, Prach Shah, Farrell Wymore, Jennifer R. Wortman and Gavin Sherlock

Abbreviations: ECS, end of cluster synteny; IGD, increase in intergenic distance; FA, change in functional annotation; ED, experimentally determined, EP, expression profile (data from M. Andersen *et al*, 2013)

Asperfuranone (afo) cluster

Experimentally determined cluster

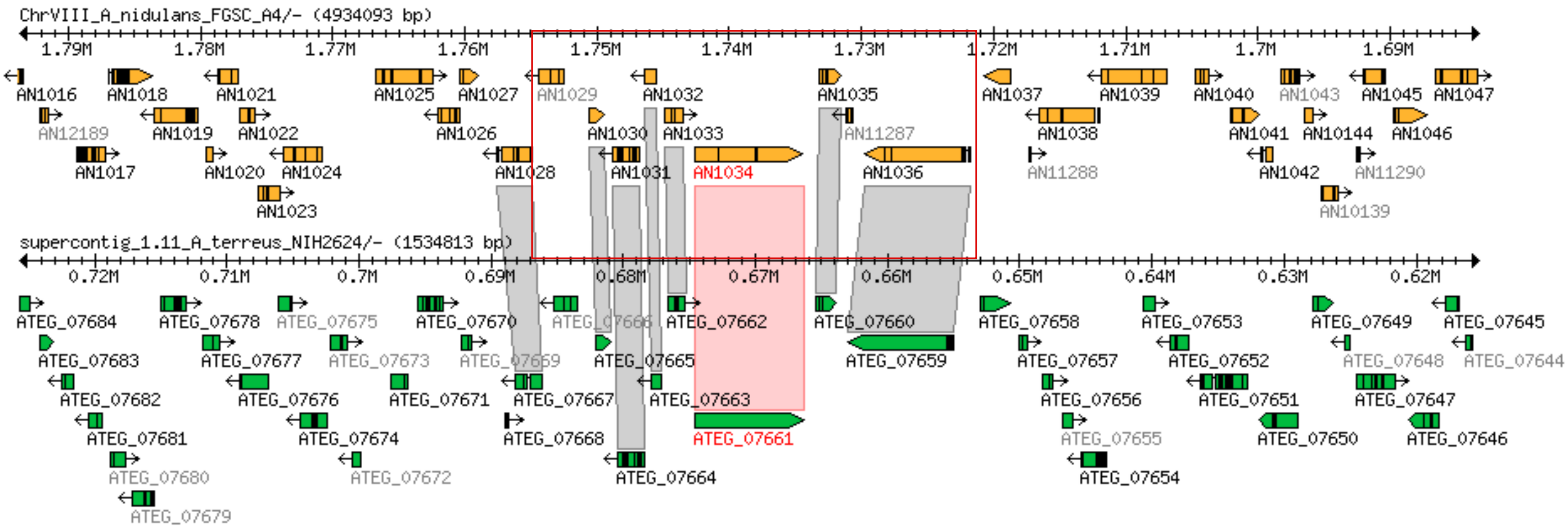

Asperfuranone (afo) cluster

Gene id (SMURF)*	Gene positions	Gene distance	Annotated_gene_function (SMURF)	AspGD gene name	AspGD manual annotation and notes	antiSMASH ID	Gene ID	Note
n/a					Protein of unknown function	prot_ID_1671	AN1026	
n/a					Protein of unknown function	prot_ID_448	AN1027	
n/a					Zn(II)2Cys6 transcription factor	prot_ID_903	AN1028	
AN1029.3	8	1371	conserved hypothetical protein	afoA	Zn(II)2Cys6 domain transcription factor	prot_ID_1382	AN1029	ED
AN1030.3	7	592	conserved hypothetical protein		Oxidoreductase	prot_ID_664	AN1030	
AN1031.3	6	385	conserved hypothetical protein	afoB	Efflux pump	prot_ID_1128	AN1031	
AN1032.3	5	606	hypothetical protein	afoC	Oxidoreductase	prot_ID_1589	AN1032	
AN1033.3	4	850	hypothetical protein	afoD	Salicylate hydroxylase	prot_ID_364	AN1033	
AN1034.3	3	528	polyketide synthase	afoE	Polyketide synthase	prot_ID_820	AN1034	
AN1035.3	2	447	FAD binding domain protein	afoF	FAD/FMN-dependent oxygenase	prot_ID_1293	AN1035	
AN11287.3	1	918	predicted protein		Protein of unknown function	prot_ID_750	AN11287	
**AN1036.3**	0	0	polyketide synthase	afoG	Polyketide synthase	**prot_ID_71**	**AN1036**	ED
AN1037.3	-1	1447	hypothetical protein similar to oleate delta-12 desaturase	odeA	Oleate delta-12 desaturase	prot_ID_524	AN1037	
AN11288.3	-2	1245	predicted protein		Protein of unknown function	prot_ID_1221	AN11288	
n/a					Rab guanyl-nucleotide exchange factor	prot_ID_988	AN1038	
n/a					membrane-tethered transcription factor, putative	prot_ID_1462	AN1039	
n/a					Protein of unknown function	prot_ID_739	AN1040	

Asperthecin (apt) cluster

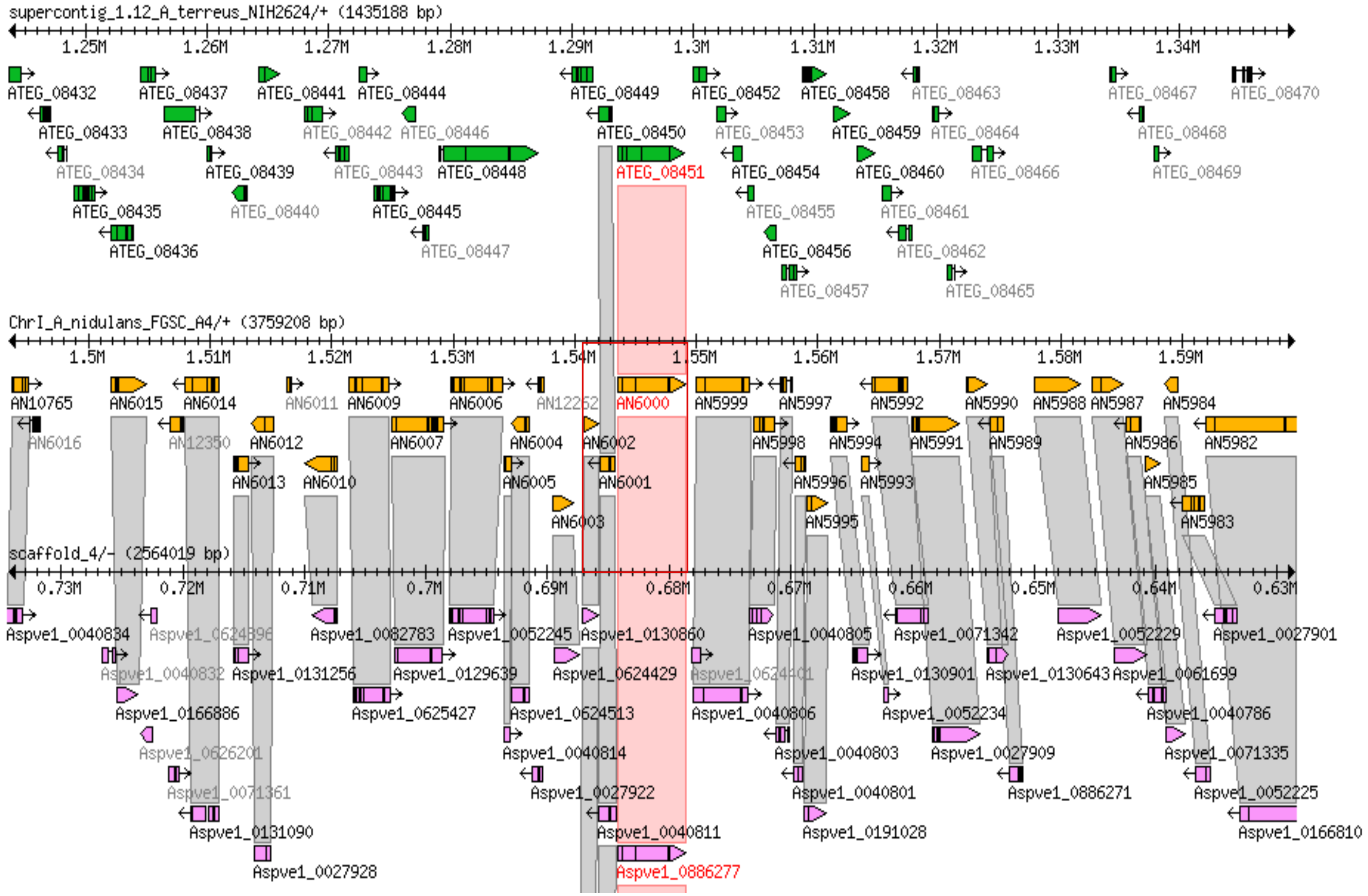

Asperthecin (apt) cluster

Gene id (SMURF)*	Gene positions	Gene distance	Annotated_gene_function (SMURF)	AspGD gene name	AspGD manual annotation and notes	antiSMASH ID	Gene ID	Note
AN5989.3	11	113	conserved hypothetical protein		NAD dependent epimerase/dehydratase	n/a	AN5989	
AN5990.3	10	662	conserved hypothetical protein		Long-chain-fatty-acid-CoA ligase	n/a	AN5990	
AN5991.3	9	400	conserved hypothetical protein	pexA	long-chain-fatty-acid-CoA ligase	prot_ID_370	AN5991	
AN5992.3	8	466	conserved hypothetical protein		DNA replication licensing factor Mcm7	prot_ID_1055	AN5992	
AN5993.3	7	1156	hypothetical protein		Putative adhesin	prot_ID_490	AN5993	
AN5994.3	6	258	conserved hypothetical protein		ATP-dependent NAD(P)H-hydrate dehydratase	prot_ID_1176	AN5994	
AN5995.3	5	242	hypothetical protein		Protein of unknown function	prot_ID_616	AN5995	
AN5996.3	4	96	hypothetical protein		60S acidic ribosomal protein P2	prot_ID_53	AN5996	
AN5997.3	3	395	40S ribosomal protein S15		40S ribosomal protein S15	prot_ID_736	AN5997	
AN5998.3	2	482	conserved hypothetical protein		SET and MYND domain protein	prot_ID_169	AN5998	
AN5999.3	1	1256	conserved hypothetical protein		Carbamoyl-phosphate synthase, large subunit	prot_ID_856	AN5999	
**AN6000.3**	0	0	polyketide synthase	aptA	Polyketide synthase	**prot_ID_873**	**AN6000**	ED
AN6001.3	-1	433	conserved hypothetical protein	aptB	Hydrolase	prot_ID_303	AN6001	
AN6002.3	-2	146	conserved hypothetical protein	aptC	Monooxygenase	prot_ID_991	AN6002	ED
AN6003.3	-3	803	conserved hypothetical protein		Protein of unknown function	prot_ID_428	AN6003	
n/a					Protein of unknown function	prot_ID_1284	AN12262	
AN6004.3	-4	1947	conserved hypothetical protein		actin cytoskeleton protein	prot_ID_1114	AN6004	
AN6005.3	-5	81	conserved hypothetical protein		Protein of unknown function	prot_ID_554	AN6005	
AN6006.3	-6	920	conserved hypothetical protein	kapD	Karyopherin	prot_ID_1235	AN6006	
AN6007.3	-7	547	conserved hypothetical protein		ATP binding, ATP-dependent helicase	prot_ID_676	AN6007	
n/a					AN6008 was merged into AN6009	prot_ID_793	AN6009	

Aspyridone (asp) cluster

Experimentally determined cluster

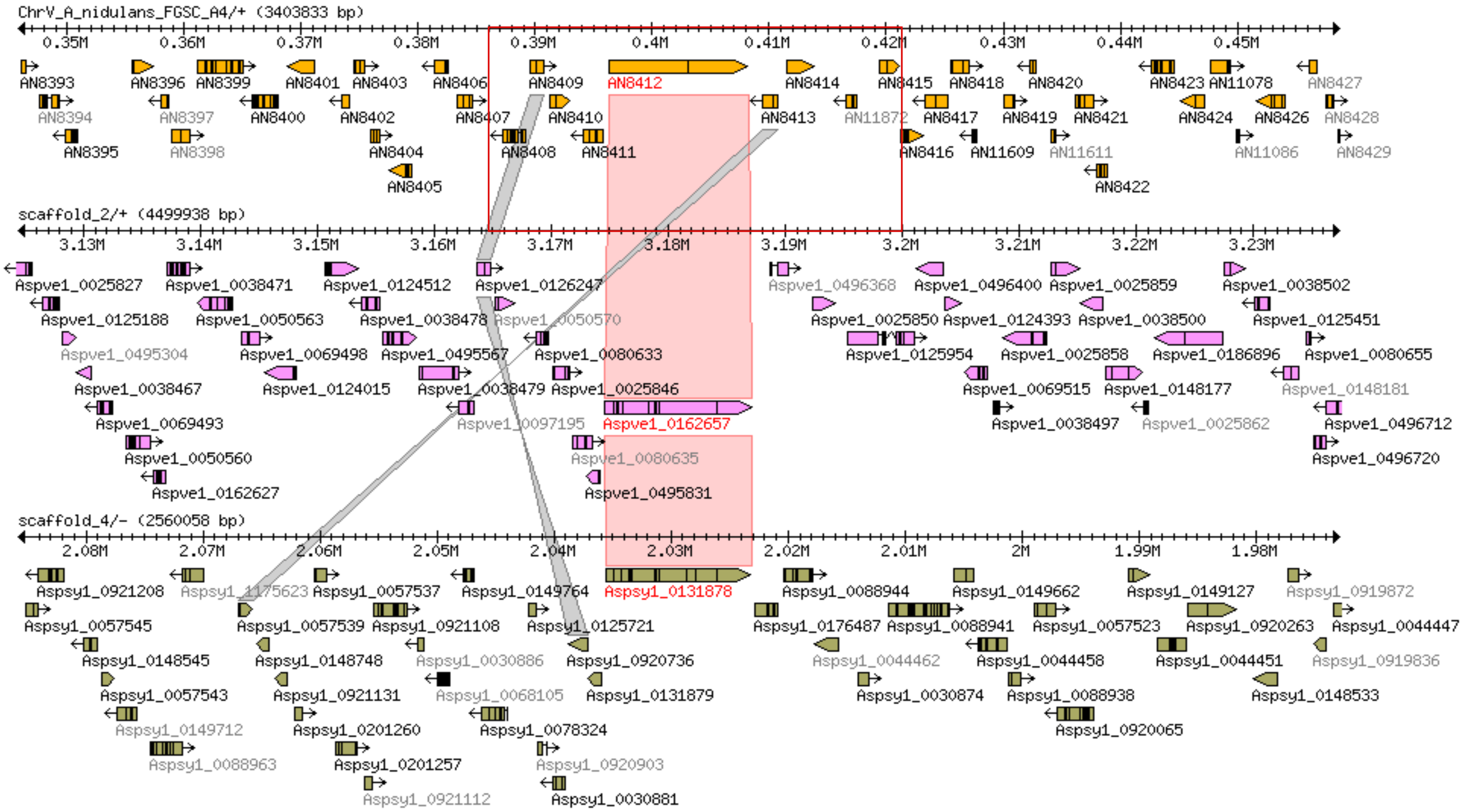

Aspyridone (asp) cluster

Gene id (SMURF)*	Gene positions	Gene distance	Annotated_gene_function (SMURF)	AspGD gene name	AspGD manual annotation and notes	antiSMASH ID	Gene ID	Note
AN8398.3	15	519	conserved hypothetical protein		Oxidoreductase	n/a	AN8398	
AN8399.3	14	917	conserved hypothetical protein		Potassium-transporting ATPase	n/a	AN8399	
AN8400.3	13	749	conserved hypothetical protein		Sugar transporter	n/a	AN8400	
AN8401.3	12	2428	conserved hypothetical protein		Alpha-N-arabinofuranosidase	n/a	AN8401	
AN8402.3	11	414	conserved hypothetical protein		Protein of unknown function	n/a	AN8402	
AN8403.3	10	441	conserved hypothetical protein		Oxidoreductase	n/a	AN8403	
AN8404.3	9	466	conserved hypothetical protein		Protease inhibitor	prot_ID_1032	AN8404	
AN8405.3	8	1805	conserved hypothetical protein		Monooxygenase	prot_ID_572	AN8405	
AN8406.3	7	873	conserved hypothetical protein		Alcohol dehydrogenase	prot_ID_105	AN8406	
AN8407.3	6	1841	conserved hypothetical protein		Protein of unknown function	prot_ID_676	AN8407	
AN11607.3	5	365	predicted protein		Protein of unknown function			
AN8408.3	4	367	cytochrome P450 oxidoreductase	apdB	cytochrome P450	prot_ID_206	AN8408	ED
AN8409.3	3	203	conserved hypothetical protein	apdC	enoyl reductase	prot_ID_776	AN8409	
AN8410.3	2	1116	conserved hypothetical protein	apdD	FAD-dependent monooxygenase	prot_ID_931	AN8410	
AN8411.3	1	476	hypothetical protein	apdE	Cytochrome P450 monooxygenase	prot_ID_476	AN8411	
**AN8412.3**	0	0	hybrid PKS-NRPS	apdA	Hybrid polyketide synthase-nonribosomal peptide synthase (PKS-NRPS)	**prot_ID_1031**	**AN8412**	
AN8413.3	-1	284	conserved hypothetical protein	apdF	Exporter	prot_ID_571	AN8413	
AN8414.3	-2	826	conserved hypothetical protein	apdR	Zn(II)2Cys6 transcription factor	prot_ID_104	AN8414	
AN11608.3	-3	1963	predicted protein		Protein of unknown function	prot_ID_1041	AN11872	
AN8415.3	-4	3186	conserved hypothetical protein	apdG	Acyl-coA dehydrogenase	prot_ID_675	AN8415	ED
n/a				furE	Transporter	prot_ID_204	AN8416	
n/a					Hydrolase	prot_ID_774	AN8417	
n/a					Hydrolase	prot_ID_306	AN8418	
n/a						prot_ID_905	AN11609	

Austinol (aus) cluster 1

Experimentally determined cluster

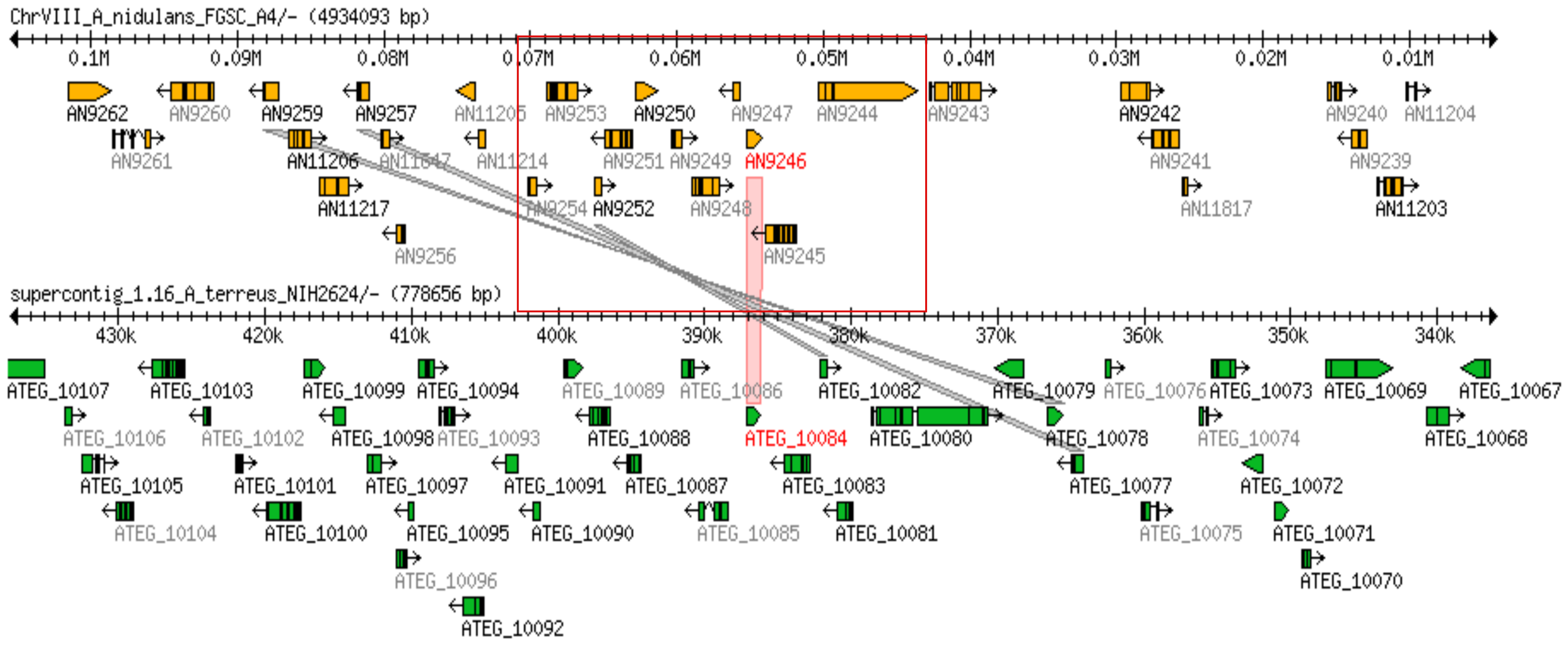

Austinol (aus) AN9244 cluster

Gene id (SMURF)*	Gene positions	Gene distance	Annotated_gene_function (SMURF)	AspGD gene name	AspGD manual annotation and notes	antiSMASH ID	Gene ID	
n/a					Protein of unknown function	prot_ID_1713	AN11817	
n/a					Protein of unknown function	prot_ID_1399	AN9241	
n/a					Protein of unknown function	prot_ID_168	AN9242	
AN9243.3	1	965	NRPS-like enzyme		Protein of unknown function	prot_ID_625	AN9243	
**AN9244.3**	0	0	nonribosomal peptide synthase		Nonribosomal peptide synthase	**prot_ID_1091**	**AN9244**	ED, FA
AN9245.3	-1	1507	fatty acid synthase		3-oxoacyl-[acyl-carrier-protein] reductase	prot_ID_1559	AN9245	
AN9246.3	-2	328	conserved hypothetical protein	ausE	Dioxygenase	prot_ID_332	AN9246	
AN9247.3	-3	361	conserved hypothetical protein	ausF	Protein of unknown function	prot_ID_787	AN9247	
AN9248.3	-4	983	conserved hypothetical protein	CYP650B1 (ausG)	Cytochrome P450	prot_ID_1263	AN9248	
AN9249.3	-5	587	conserved hypothetical protein	ausH	Protein of unknown function	prot_ID_39	AN9249	
AN9250.3	-6	918	conserved hypothetical protein		O-acetyltransferase	prot_ID_1006	AN9250	
AN9251.3	-7	209	conserved hypothetical protein	CYP650A1	Cytochrome P450	prot_ID_1479	AN9251	
n/a					Protein of unknown function	prot_ID_254	AN9252	
n/a				CYP651A1 (ausI)	Cytochrome P450	prot_ID_706	AN9253	ED
n/a					Protein of unknown function	prot_ID_1176	AN9254	

Austinol (aus) cluster 2

Experimentally determined cluster

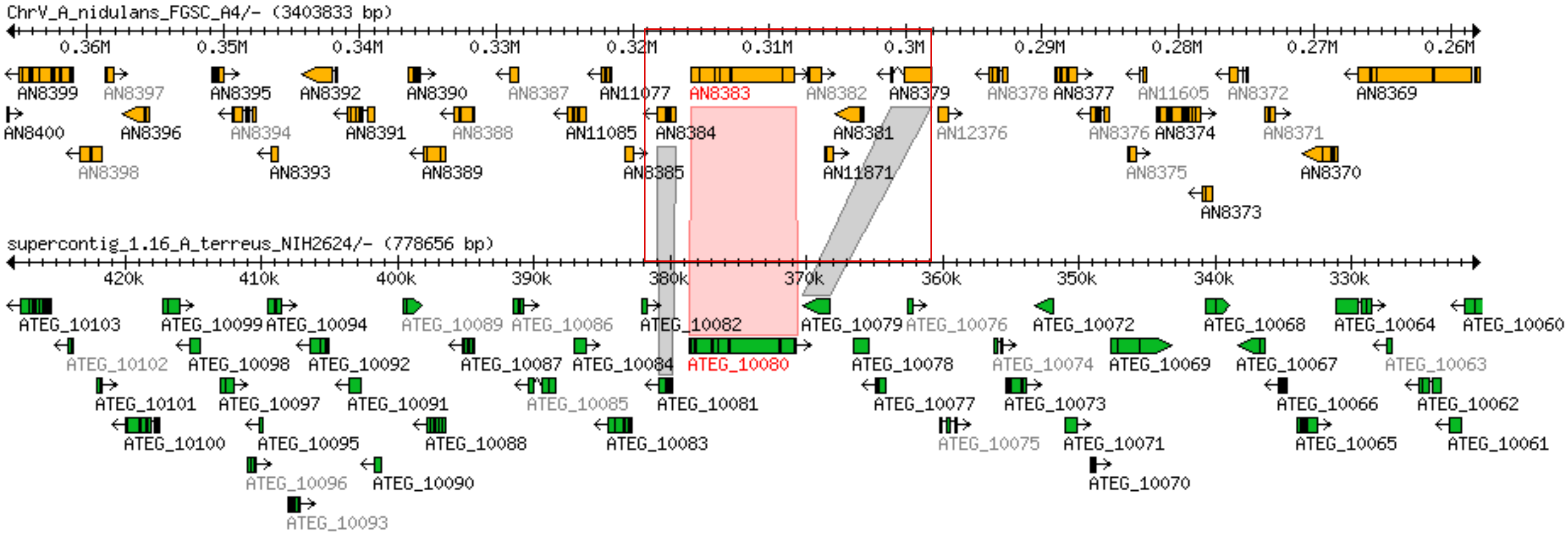

Austinol (aus) cluster

Gene id (SMURF)*	Gene positions	Gene distance	Annotated_gene_function (SMURF)	AspGD gene name	AspGD manual annotation and notes	antiSMASH ID	Gene ID	
n/a					Myb-like transcription factor	prot_ID_161	AN8377	
n/a					Monooxygenase	prot_ID_734	AN8378	
n/a					Oxidoreductase	prot_ID_1052	AN12376	
n/a				ausB	Oxidoreductase	prot_ID_267	AN8379	ED, ECS
n/a				ausC	Protein of unknown function	prot_ID_995	AN8381	
n/a					Protein of unknown function	prot_ID_1056	AN11871	
AN8382.3	1	906	hypothetical protein		Protein of unknown function	prot_ID_540	AN8382	
**AN8383.3**	0	0	polyketide synthase	ausA	Polyketide synthase	**prot_ID_62**	**AN8383**	
AN8384.3	-1	1159	conserved hypothetical protein	ausD	Protein of unknown function	prot_ID_635	AN8384	ED, ECS
AN8385.3	-2	1832	conserved hypothetical protein		Protein of unknown function	prot_ID_159	AN8385	
AN11077.3	-3	955	conserved hypothetical protein		Acetyltransferase	prot_ID_519	AN11077	
AN11085.3	-4	1315	conserved hypothetical protein		D-arabinitol 4-dehydrogenase	prot_ID_518	AN11085	
AN8387.3	-5	3635	conserved hypothetical protein		Protein of unknown function	prot_ID_263	AN8387	
AN8388.3	-6	2608	conserved hypothetical protein		Protein of unknown function	prot_ID_833	AN8388	
AN8389.3	-7	673	conserved hypothetical protein		Predicted acid phosphatase	prot_ID_366	AN8389	
AN8390.3	-8	149	conserved hypothetical protein		GPR1/FUN34/YaaH family member	prot_ID_538	AN8390	
AN8391.3	-9	2505	conserved hypothetical protein		Zn(II)2Cys6 transcription factor	n/a	AN8391	
AN8392.3	-10	840	conserved hypothetical protein		Alpha-glycosidase	n/a	AN8392	
AN8393.3	-11	1834	conserved hypothetical protein		Protein of unknown function	n/a	AN8393	
AN8394.3	-12	1349	conserved hypothetical protein		Transmembrane transporter	n/a	AN8394	
AN8395.3	-13	328	conserved hypothetical protein		Peroxidase	n/a	AN8395	

Combined Derivative of BenzAldehyde1 (dba) and F9775 clusters 1 and 2

Experimentally determined cluster

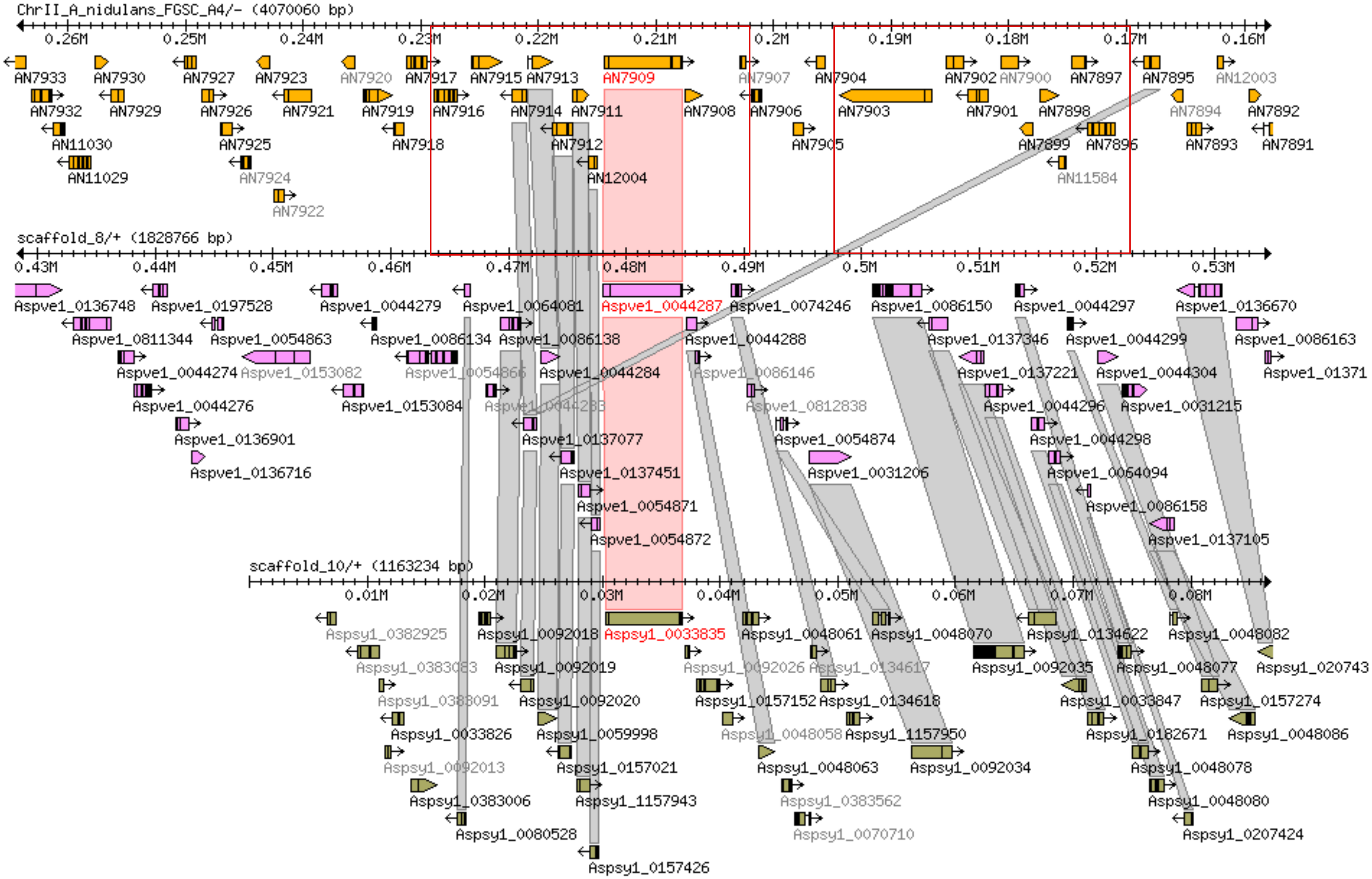

Combined Derivative of BenzAldehyde1 (dba) and F9775 clusters

Gene id (SMURF)*	Gene positions	Gene distance	Annotated_gene_function (SMURF)	AspGD gene name	AspGD manual annotation and notes	antiSMASH ID	Gene ID	
n/a				cipB	oxidoreductase	prot_ID_1082	AN7895	
n/a				dbaA	Zn(II)2Cys6 transcription factor	prot_ID_465	AN7896	ED, cluster 1
n/a				dbaB	FAD-binding monooxygenase	prot_ID_1211	AN7897	
n/a				dbaC	YCII domain protein	prot_ID_983	AN11584	
n/a				dbaD	MFS transporter	prot_ID_594	AN7898	
n/a				dbaE	Esterase/lipase	prot_ID_1344	AN7899	
n/a				dbaF	Oxidoreductase	prot_ID_475	AN7900	
n/a				dbaG	C6 transcription factor	prot_ID_1221	AN7901	
n/a				dbaH	FAD-binding monooxygenase	prot_ID_605	AN7902	
n/a				pkeA/dbaI	Polyketide synthase	prot_ID_5	**AN7903**	ED, EP, cluster 1
n/a					S-adenosylmethionine-dependent methyltransferase	prot_ID_735	AN7904	
AN7905.3	4	2662	conserved hypothetical protein		Phosphogluconate dehydrogenase	prot_ID_127	AN7905	
AN7906.3	3	461	conserved hypothetical protein		Protein of unknown function	prot_ID_867	AN7906	
AN7907.3	2	3221	conserved hypothetical protein		Glyoxylate-bleomycin resistance protein, F9775 secondary metabolite gene cluster	prot_ID_257	AN7907	ED, cluster 2
AN7908.3	1	267	conserved hypothetical protein		Type II fatty acid synthase, F9775 secondary metabolite gene cluster	prot_ID_1005	AN7908	
**AN7909.3**	0	0	polyketide synthase	orsA	Polyketide synthase, F9775 secondary metabolite gene cluster	**prot_ID_387**	**AN7909**	
**n/a**					Protein of unknown function, F9775 secondary metabolite gene cluster	prot_ID_1413	AN12004	
AN7911.3	-1	1512	conserved hypothetical protein	orsB	Amidohydrolase, F9775 secondary metabolite gene cluster	prot_ID_4	AN7911	
AN7912.3	-2	358	conserved hypothetical protein	orsC	Tyrosinase, F9775 secondary metabolite gene cluster	prot_ID_733	AN7912	
AN7913.3	-3	521	conserved hypothetical protein		Protein of unknown function	prot_ID_126	AN7913	
AN7914.3	-4	427	conserved hypothetical protein		Alcohol dehydrogenase, F9775 secondary metabolite gene cluster	prot_ID_865	AN7914	
AN7915.3	-5	909	conserved hypothetical protein		Glucosidase, F9775 secondary metabolite gene cluster	prot_ID_254	AN7915	

AN7916.3	-6	1240	conserved hypothetical protein	Amino acid transporter, F9775 secondary metabolite gene cluster	prot_ID_1003	AN7916	ED, cluster 2
AN7917.3	-7	614	conserved hypothetical protein	oxidoreductase	prot_ID_385	AN7917	
AN7918.3	-8	502	conserved hypothetical protein	Protein of unknown function	prot_ID_1134	AN7918	
AN7919.3	-9	40	conserved hypothetical protein	Zn(II)2Cys6 transcription factor	prot_ID_519	AN7919	
AN7920.3	-10	632	conserved hypothetical protein	Hydrolase	n/a	AN7920	
AN7921.3	-11	2656	conserved hypothetical protein	Zn(II)2Cys6 transcription factor	n/a	AN7921	
AN7922.3	-12	217	conserved hypothetical protein	Riboflavin reductase	n/a	AN7922	
AN7923.3	-13	318	conserved hypothetical protein	Zn(II)2Cys6 transcription factor	n/a	AN7923	

Emericellamide (eas) cluster

Experimentally characterized cluster

AN2546 - uncharacterized cluster member

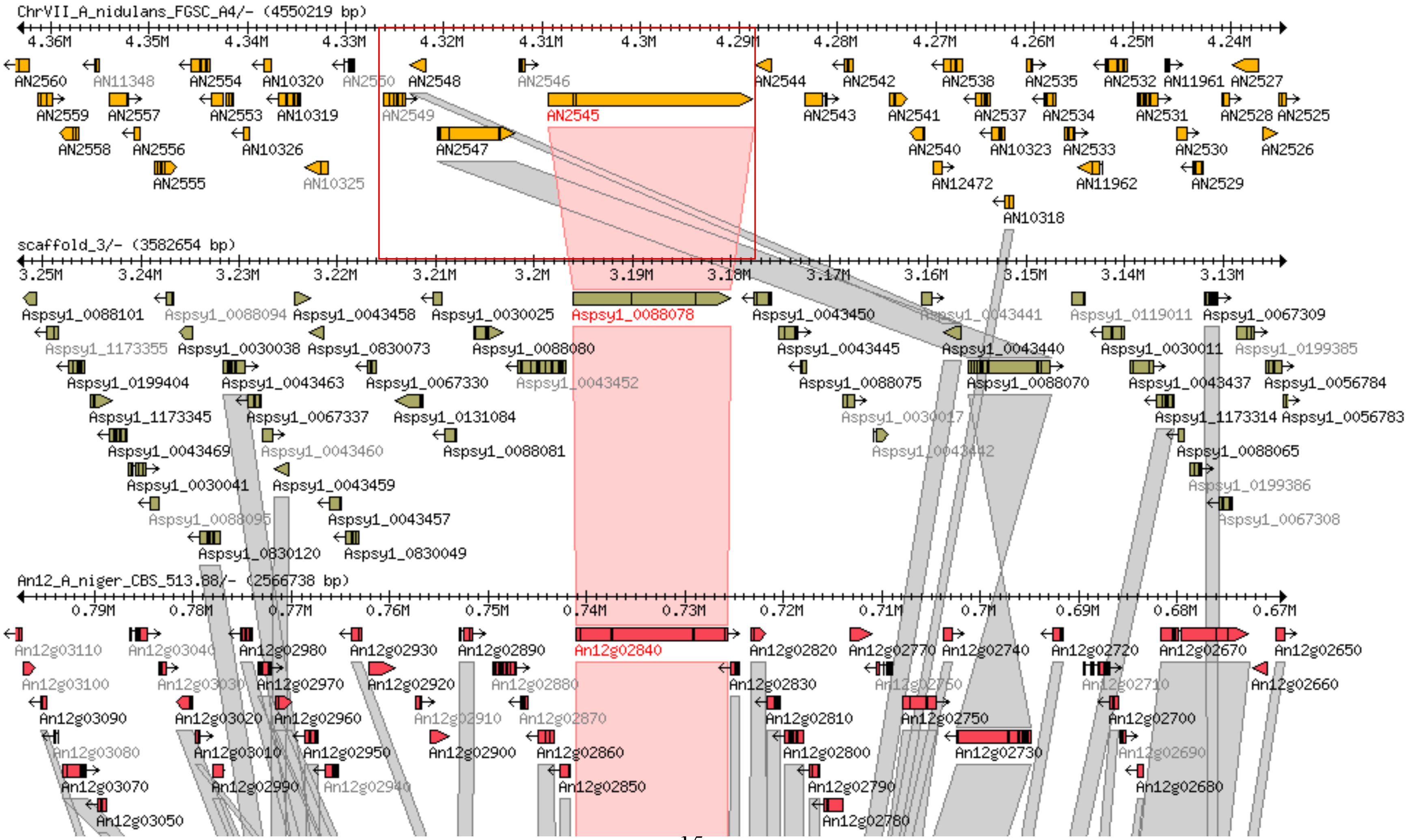

Emericellamide (eas) cluster

Gene id (SMURF)*	Gene positions	Gene distance	Annotated_gene_function (SMURF)	AspGD gene name	AspGD manual annotation and notes	antiSMASH ID	Gene ID	
n/a					Protein of unknown function	prot_ID_306	AN2538	
n/a					AN2539 and AN9518 were merged (and consequently deleted) to create AN12472	prot_ID_1583	AN2539	
					Hydrolase		AN12472	
n/a					Protein of unknown function	prot_ID_1360	AN2540	
n/a					Alpha-N-arabinofuranosidase	prot_ID_690	AN2541	
n/a					Pectate lyase	prot_ID_22	AN2542	
n/a				plyD	Rhamnogalacturonan lyase	prot_ID_836	AN2543	
AN2544.3	3	140	conserved hypothetical protein		Transmembrane transporter	prot_ID_161	AN2544	
AN2545.3	2	2252	nonribosomal peptide synthase	easA	Nonribosomal peptide synthetase	prot_ID_980	AN2545	ED
AN2546.3	1	699	hypothetical protein		Glycosylphosphatidylinositol (GPI)-anchored protein	prot_ID_304	AN2546	
**AN2547.3**	0	0	polyketide synthase	easB	Polyketide synthase	**prot_ID_1120**	**AN2547**	
AN2548.3	-1	957	conserved hypothetical protein	easC	Acyltransferase	prot_ID_452	AN2548	
AN2549.3	-2	473	conserved hypothetical protein	easD	Acyl-CoA ligase	prot_ID_1260	AN2549	ED
AN2550.3	-3	3294	conserved hypothetical protein		Protein of unknown function	prot_ID_21	AN2550	
AN10325.3	-4	1645	conserved hypothetical protein		Tyrosine kinase	prot_ID_212	AN10325	
AN10319.3	-5	530	conserved hypothetical protein		Transmembrane transporter	prot_ID_365	AN10319	
AN10320.3	-6	1169	conserved hypothetical protein		Protein of unknown function	prot_ID_597	AN10320	
AN10326.3	-7	1474	conserved hypothetical protein		Protein of unknown function	prot_ID_1033	AN10326	
AN2553.3	-8	901	conserved hypothetical protein		Zn(II)2Cys6 transcription factor	n/a	AN2553	

inp cluster

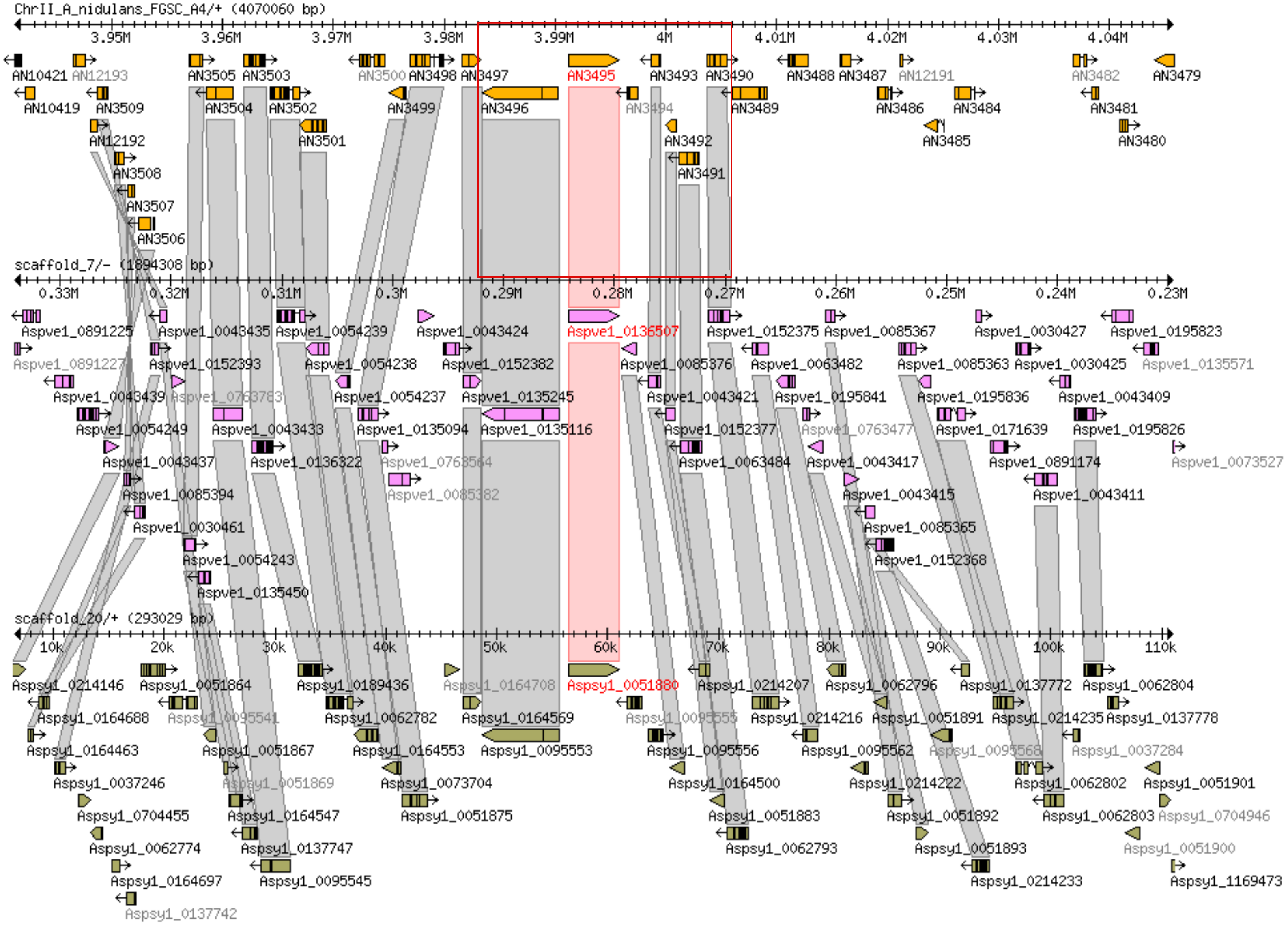
ChrII_A_nidulans_FGSC_A4/+ (4070060 bp)
3.95M
3.96M
3.97M
3.98M
3.99M
4M
4.01M
4.02M
4.03M
4.04M
AN10421
AN12193
AN3505
AN3503
AN3500
AN3498
AN3497
AN3495
AN3493
AN3490
AN3488
AN3487
AN12191
AN3482
AN3479
AN10419
AN3509
AN3504
AN3502
AN3499
AN3496
AN3494
AN3489
AN3486
AN3484
AN3481
AN12192
AN3501
AN3492
AN3485
AN3480
AN3508
AN3491
AN3507
AN3506
scaffold_7/- (1894308 bp)
0.33M
0.32M
0.31M
0.3M
0.29M
0.28M
0.27M
0.26M
0.25M
0.24M
0.23M
Aspve1_0891225
Aspve1_0043435
Aspve1_0054239
Aspve1_0043424
Aspve1_0136507
Aspve1_0152375
Aspve1_0085367
Aspve1_0030427
Aspve1_0195823
Aspve1_0891227
Aspve1_0152393
Aspve1_0054238
Aspve1_0152382
Aspve1_0085376
Aspve1_0063482
Aspve1_0085363
Aspve1_0030425
Aspve1_0135571
Aspve1_0043439
Aspve1_0763783
Aspve1_0054237
Aspve1_0135245
Aspve1_0043421
Aspve1_0195841
Aspve1_0195836
Aspve1_0043409
Aspve1_0054249
Aspve1_0043433
Aspve1_0135094
Aspve1_0135116
Aspve1_0152377
Aspve1_0763477
Aspve1_0171639
Aspve1_0195826
Aspve1_0043437
Aspve1_0136322
Aspve1_0763564
Aspve1_0063484
Aspve1_0043417
Aspve1_0891174
Aspve1_0073527
Aspve1_0085394
Aspve1_0085382
Aspve1_0043415
Aspve1_0043411
Aspve1_0030461
Aspve1_0085365
Aspve1_0054243
Aspve1_0152368
Aspve1_0135450
scaffold_20/+ (293029 bp)
10k
20k
30k
40k
50k
60k
70k
80k
90k
100k
110k
Aspsy1_0214146
Aspsy1_0051864
Aspsy1_0189436
Aspsy1_0164708
Aspsy1_0051880
Aspsy1_0214207
Aspsy1_0062796
Aspsy1_0137772
Aspsy1_0062804
Aspsy1_0164688
Aspsy1_0095541
Aspsy1_0062782
Aspsy1_0164569
Aspsy1_0095555
Aspsy1_0214216
Aspsy1_0051891
Aspsy1_0214235
Aspsy1_0137778
Aspsy1_0164463
Aspsy1_0051867
Aspsy1_0164553
Aspsy1_0095553
Aspsy1_0095556
Aspsy1_0095562
Aspsy1_0095568
Aspsy1_0037284
Aspsy1_0037246
Aspsy1_0051869
Aspsy1_0073704
Aspsy1_0164500
Aspsy1_0214222
Aspsy1_0062802
Aspsy1_0051901
Aspsy1_0704455
Aspsy1_0164547
Aspsy1_0051875
Aspsy1_0051883
Aspsy1_0051892
Aspsy1_0062803
Aspsy1_0704946
Aspsy1_0062774
Aspsy1_0137747
Aspsy1_0062793
Aspsy1_0051893
Aspsy1_0051900
Aspsy1_0164697
Aspsy1_0095545
Aspsy1_0214233
Aspsy1_1169473
Aspsy1_0137742

inp cluster

Gene id (SMURF)*	Gene positions	Gene distance	Annotated_gene_function (SMURF)	AspGD gene name	AspGD manual annotation and notes	antiSMASH ID	Gene ID	
n/a					Protein of unknown function	n/a	AN3484	
n/a					Protein of unknown function	prot_ID_160	AN3485	
n/a					Protein of unknown function	prot_ID_1441	AN12191	
n/a					Protein of unknown function	prot_ID_899	AN3486	
n/a					Protein of unknown function	prot_ID_286	AN3487	
n/a					N,N-dimethylaniline monooxygenase	prot_ID_1033	AN3488	
n/a					Ortholog(s) have endoplasmic reticulum localization	prot_ID_415	AN3489	
n/a					Protein of unknown function	prot_ID_633	AN3490	EP
AN3491.3	5	403	conserved hypothetical protein		Transmembrane transporter	prot_ID_33	AN3491	
AN3492.3	4	618	conserved hypothetical protein	scpR	C2H2 type zinc finger transcription factor	prot_ID_765	AN3492	
AN3493.3	3	1307	conserved hypothetical protein		Threonine-type endopeptidase	prot_ID_157	AN3493	
AN3494.3	2	233	conserved hypothetical protein		Protein of unknown function	prot_ID_898	AN3494	
AN3495.3	1	834	NRPS-like enzyme	inpA	Nonribosomal peptide synthetase	prot_ID_285	AN3495	
**AN3496.3**	0	0	nonribosomal peptide synthase, putatuve	inpB	Nonribosomal peptide synthetase	**prot_ID_1032**	**AN3496**	EP
AN3497.3	-1	27	conserved hypothetical protein	CYP623B2	Cytochrome P450	prot_ID_414	AN3497	
AN3498.3	-2	1672	conserved hypothetical protein		Substrate-specific transmembrane transporter	prot_ID_1159	AN3498	
AN3499.3	-3	432	conserved hypothetical protein		Phosphotransferase	prot_ID_547	AN3499	
AN3500.3	-4	280	conserved hypothetical protein		RNA-directed DNA polymerase	prot_ID_1046	AN3500	
AN3501.3	-5	281	conserved hypothetical protein		Zn(II)2Cys6 transcription factor	prot_ID_427	AN3501	
AN3502.3	-6	149	conserved hypothetical protein		Zn(II)2Cys6 transcription factor	prot_ID_1173	AN3502	
AN3503.3	-7	349	conserved hypothetical protein		Transmembrane transporter	prot_ID_558	AN3503	

AN3504.3	-8	786	conserved hypothetical protein	Alpha-1,4-glucosidase	n/a	AN3504
AN3505.3	-9	306	conserved hypothetical protein	D-alanine-D-alanine ligase	n/a	AN3505
AN3506.3	-10	3177	conserved hypothetical protein	Zn(II)2Cys6 transcription factor	n/a	AN3506

ivo cluster

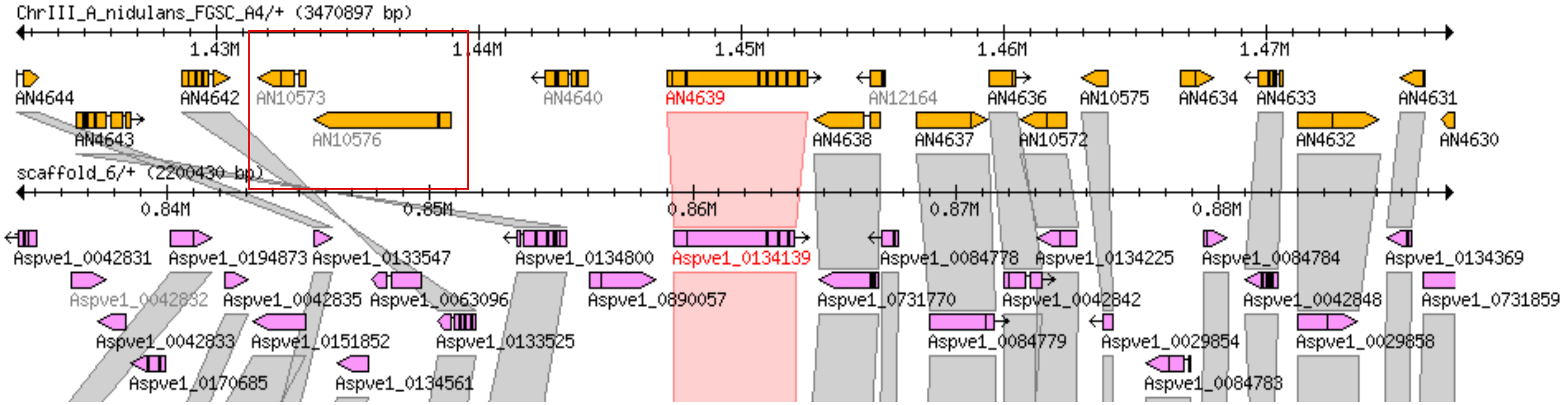
ChrIII_A_nidulans_FGSC_A4/+ (3470897 bp)
1.43M
1.44M
1.45M
1.46M
1.47M
AN4644
AN4642
AN10573
AN4640
AN4639
AN12164
AN4636
AN10575
AN4634
AN4633
AN4631
AN4643
AN10576
AN4638
AN4637
AN10572
AN4632
AN4630
scaffold_6/+ (2200430 bp)
0.84M
0.85M
0.86M
0.87M
0.88M
Aspve1_0042831
Aspve1_0194873
Aspve1_0133547
Aspve1_0134800
Aspve1_0134139
Aspve1_0084778
Aspve1_0134225
Aspve1_0084784
Aspve1_0134369
Aspve1_0042832
Aspve1_0042835
Aspve1_0063096
Aspve1_0890057
Aspve1_0731770
Aspve1_0042842
Aspve1_0042848
Aspve1_0731859
Aspve1_0042833
Aspve1_0151852
Aspve1_0133525
Aspve1_0084779
Aspve1_0029854
Aspve1_0029858
Aspve1_0170685
Aspve1_0134561
Aspve1_0084783

ivo cluster

Gene id (SMURF)*	Gene positions	Gene distance	Annotated_gene_function (SMURF)	AspGD gene name	AspGD manual annotation and notes	antiSMASH ID	Gene ID	
n/a					PA and RING finger domain protein	prot_ID_334	AN4637	
n/a					Protein of unknown function	prot_ID_1259	AN12164	
n/a					Protein of unknown function	prot_ID_974	AN4638	
n/a					TORC1 growth control complex subunit	prot_ID_449	AN4639	
					tRNA-Met(CAU)4	n/a	tM(CAU)4	
n/a					Transmembrane transporter	prot_ID_630	AN4640	
**AN10576.3**	0	0	nonribosomal peptide synthase	ivoA	One module NRPS; conidiophore pigmentation	**prot_ID_570**	**AN10576**	EP
AN10573.3	-1	376	conserved hypothetical protein	ivoC	Cytochrome P450	prot_ID_982	AN10573	EP
AN4642.3	-2	1170	hypothetical protein		Protein of unknown function	prot_ID_739	AN4642	
AN4643.3	-3	2097	conserved hypothetical protein	CYP675A1	Cytochrome P450	prot_ID_221	AN4643	
AN4644.3	-4	1432	conserved hypothetical protein	CYP675A1	Cytochrome P450	prot_ID_853	AN4644	
AN4645.3	-5	1993	conserved hypothetical protein		Protein of unknown function	prot_ID_333	AN4645	
AN4646.3	-6	1	hypothetical protein		Protein of unknown function	prot_ID_973	AN4646	
AN4647.3	-7	407	conserved hypothetical protein		Protein of unknown function	prot_ID_448	AN4647	
AN4648.3	-8	59	conserved hypothetical protein		small nucleolar ribonucleoprotein complex subunit	prot_ID_1086	AN4648	
AN4649.3	-9	806	conserved hypothetical protein		Protein of unknown function	prot_ID_561	AN4649	

Microperfuranone cluster

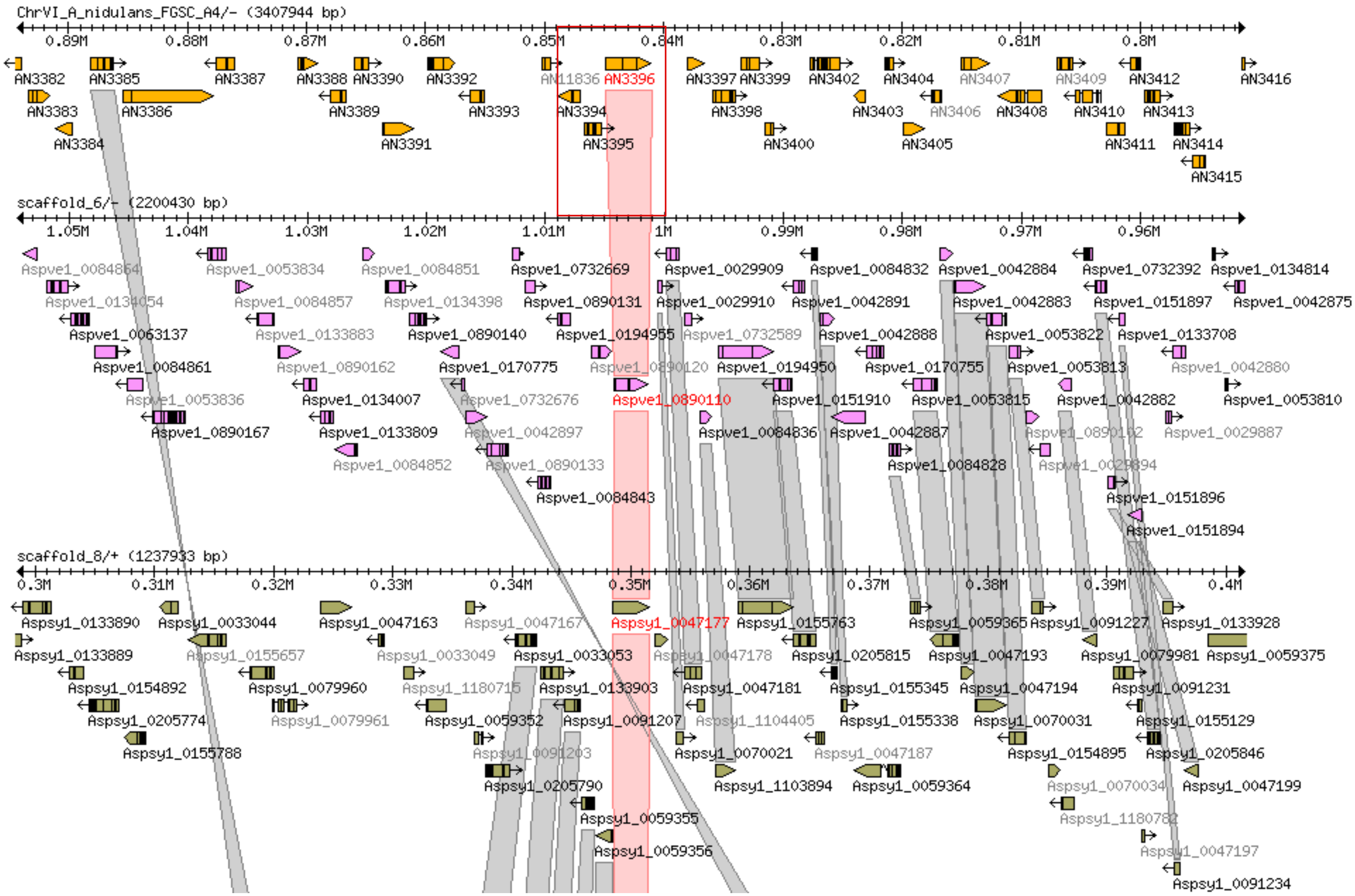

Microperfuranone cluster

Gene id (SMURF)*	Gene positions	Gene distance	Annotated_gene_function (SMURF)	AspGD gene name	AspGD manual annotation and notes	antiSMASH ID	Gene ID	
AN3391.3	6	1039	conserved hypothetical protein		C2H2 and Zn(2)-Cys(6) DNA binding transcription factor	prot_ID_954	AN3391	
AN3392.3	5	1186	conserved hypothetical protein		Monooxygenase	prot_ID_455	AN3392	
AN3393.3	4	3751	hypothetical protein	pepI	Neutral metalloprotease II	prot_ID_1062	AN3393	
AN11393.3	3	2130	predicted protein		Protein of unknown function	prot_ID_1120	AN11836	
AN3394.3	2	434	conserved hypothetical protein	CYP620D1	Cytochrome P450; putative O-methylsterigmatocystin oxidoreductase	prot_ID_553	AN3394	EP
AN3395.3	1	1001	conserved hypothetical protein		Protein of unknown function	prot_ID_67	AN3395	
**AN3396.3**	0	0	NRPS-like enzyme	micA	Non-ribosomal peptide synthase	**prot_ID_663**	**AN3396**	EP
AN11394.3	-1	250	predicted protein		Protein of unknown function			
AN3397.3	-2	2778	conserved hypothetical protein		Protein of unknown function	prot_ID_174	AN3397	
AN3398.3	-3	698	hypothetical protein		Transmembrane transporter	prot_ID_768	AN3398	
AN3399.3	-4	520	conserved hypothetical protein		UDP-N-acetylmuramate dehydrogenase	prot_ID_275	AN3399	
AN3400.3	-5	334	conserved hypothetical protein		Tetrahydroxynaphthalene reductase	prot_ID_668	AN3400	
AN3401.3	-6	665	conserved hypothetical protein		Deleted ORF; DDE1 transposon-related ORF		x	
AN3402.3	-7	1647	hypothetical protein similar to alpha-amylase		Alpha-amylase	prot_ID_774	AN3402	
AN3403.3	-8	1076	conserved hypothetical protein		Protein of unknown function	prot_ID_280	AN3403	
AN3404.3	-9	1667	conserved hypothetical protein		Glycosylphosphatidylinositol (GPI)-anchored protein	prot_ID_885	AN3404	
AN3405.3	-10	773	conserved hypothetical protein		Protein of unknown function	n/a	AN3405	
AN3406.3	-11	703	hypothetical protein		Protein of unknown function	n/a	AN3406	
AN3407.3	-12	1627	conserved hypothetical protein		Serine-type endopeptidase	n/a	AN3407	
AN3408.3	-13	1234	conserved hypothetical protein		Dipeptide transporter	n/a	AN3408	

Monodictyphenone (mdp) cluster

Experimentally determined cluster

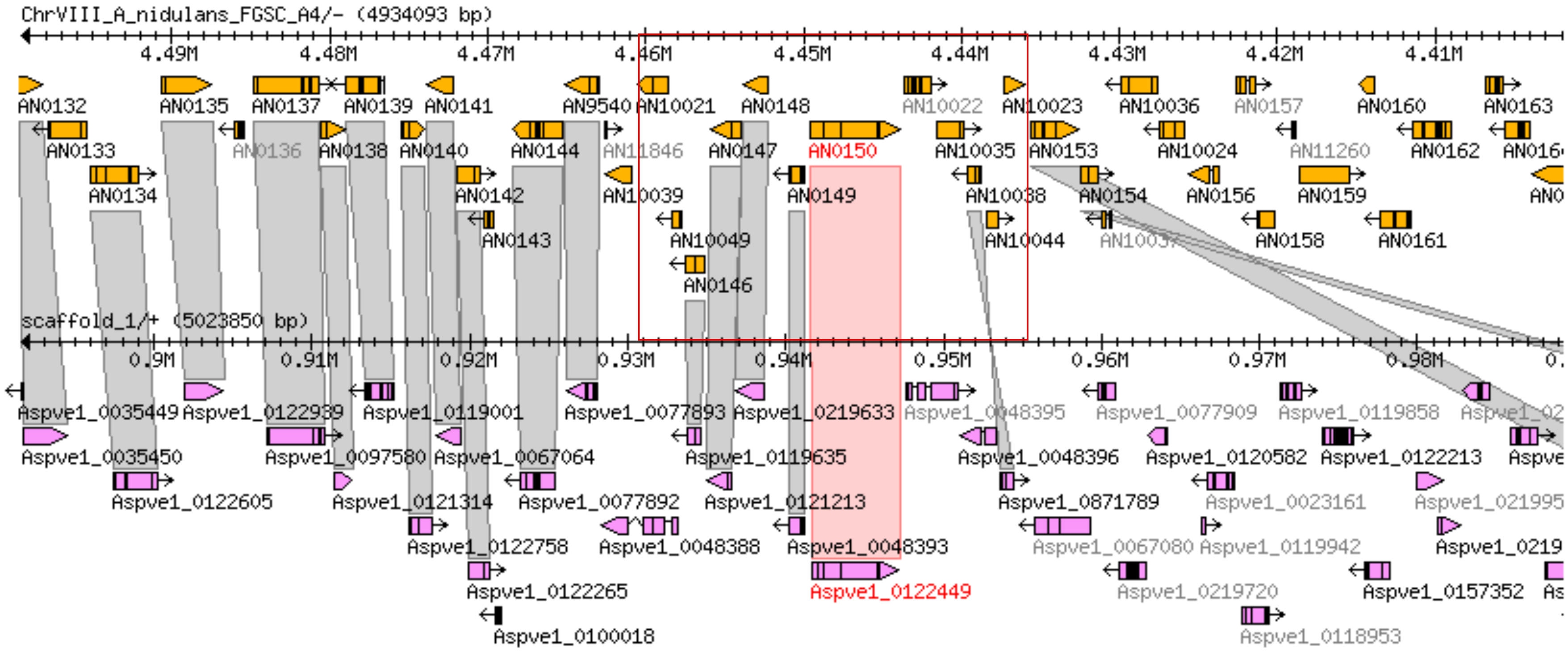

Monodictyphenone (mdp) cluster

Gene id (SMURF)*	Gene positions	Gene distance	Annotated_gene_function (SMURF)	AspGD gene name	AspGD manual annotation and notes	antiSMASH ID	Gene ID	
n/a					Protein of unknown function	prot_ID_1731	AN11846	
n/a					Protein of unknown function	prot_ID_484	AN9540	
n/a					Histidine acid phosphatase	prot_ID_804	AN10039	
n/a				mdpA	Transcription coactivator	prot_ID_401	AN10021	ED
n/a				mdpB	Scytalone dehydratase	prot_ID_886	AN10049	
AN0146.3	4	631	conserved hypothetical protein	mdpC	Versicolorin ketoreductase	prot_ID_916	AN0146	
AN0147.3	3	527	conserved hypothetical protein	mdpD	Flavin-containing monooxygenase	prot_ID_1395	AN0147	
AN0148.3	2	1479	conserved hypothetical protein	mdpE	C6 zinc finger transcription	prot_ID_164	AN0148	
AN0149.3	1	469	conserved hypothetical protein	mdpF	zinc-dependent hydrolase	prot_ID_619	AN0149	
**AN0150.3**	0	0	polyketide synthase	mdpG	Polyketide synthase	**prot_ID_1601**	**AN0150**	
AN10022.3	-1	306	conserved hypothetical protein	mdpH	DUF 1772 superfamily protein	prot_ID_856	AN10022	
AN10035.3	-2	368	conserved hypothetical protein	mdpI	AMP-binding CoA ligase	prot_ID_642	AN10035	
AN10038.3	-3	146	hypothetical protein	mdpJ	Glutathione S transferase	prot_ID_351	AN10038	
AN10044.3	-4	365	conserved hypothetical protein	mdpK	Oxidoreductase	prot_ID_271	AN10044	
AN10023.3	-5	361	conserved hypothetical protein	mdpL	Protein of unknown function	prot_ID_1333	AN10023	ED
AN0153.3	-6	484	hypothetical protein		MYB DNA binding protein	prot_ID_1307	AN0153	
AN0154.3	-7	239	hypothetical protein		TFIID and SAGA complex TAF10 subunit	prot_ID_81	AN0154	
AN10037.3	-8	386	conserved hypothetical protein		Protein of unknown function	prot_ID_1576	AN10037	
AN10036.3	-9	736	conserved hypothetical protein		Transmembrane transporter	prot_ID_1112	AN10036	
AN10024.3	-10	557	conserved hypothetical protein		Monooxygenase	prot_ID_105	AN10024	
AN0156.3	-11	242	conserved hypothetical protein		Inositol tetrakisphosphate 3-kinase	prot_ID_1001	AN0156	
AN0157.3	-12	1490	conserved hypothetical protein		Protein of unknown function	n/a	AN0157	
AN0158.3	-13	117	conserved hypothetical protein		2-alkenal reductase [NAD(P)]	n/a	AN0158	

Penicillin cluster

Experimentally determined cluster

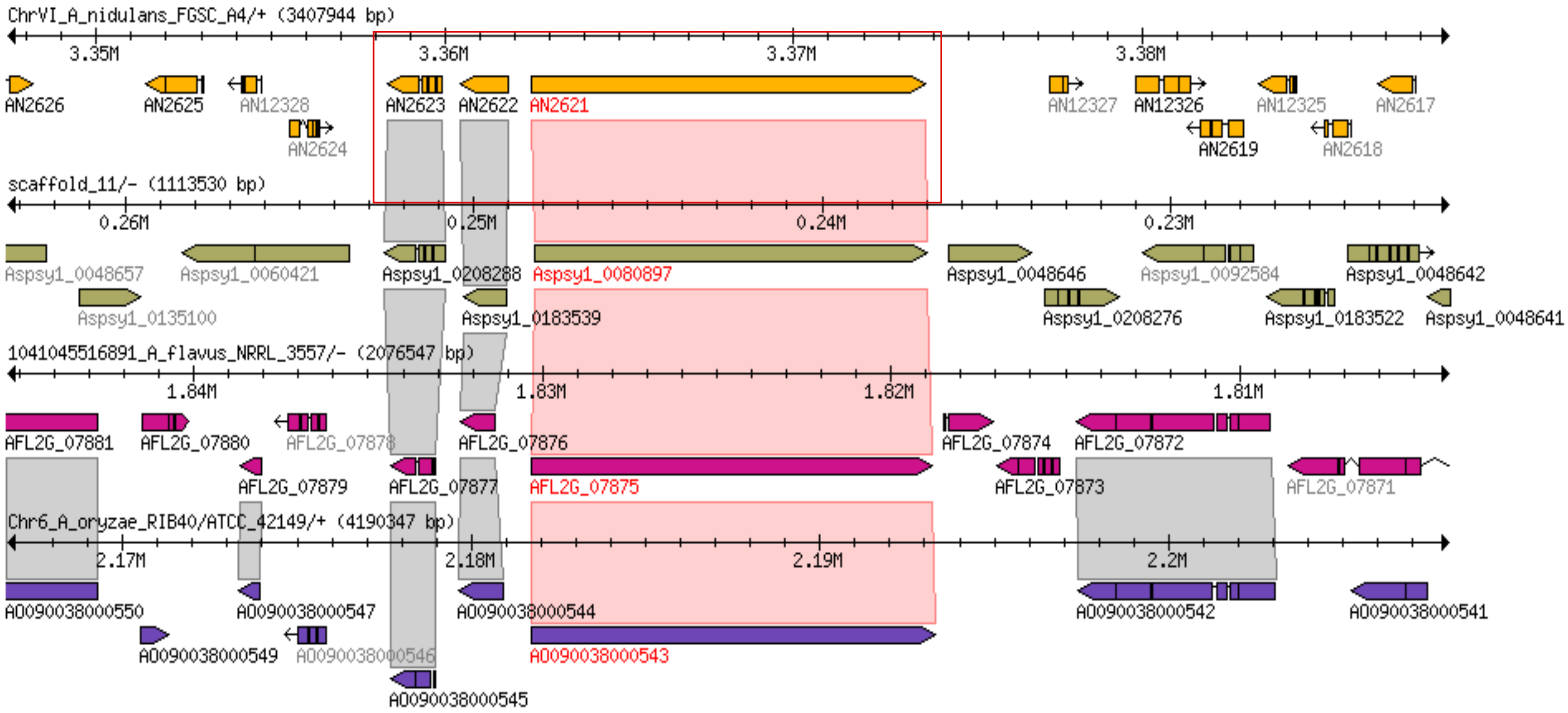

Penicillin cluster

Gene id (SMURF)*	Gene positions	Gene distance	Annotated_gene_function (SMURF)	AspGD gene name	AspGD manual annotation and notes	antiSMASH ID	Gene ID	
n/a					RNA-directed DNA polymerase	prot_ID_101	AN2616	
n/a					Protein of unknown function	prot_ID_694	AN2617	
n/a					Protein of unknown function	prot_ID_207	AN2618	
n/a					Protein of unknown function	prot_ID_1131	AN12325	
AN2619.3	2	243	conserved hypothetical protein		Protein of unknown function	prot_ID_803	AN2619	
n/a					Domain(s) with predicted carbohydrate binding	prot_ID_1159	AN12326	
n/a					Protein of unknown function	prot_ID_1105	AN12327	
**AN2621.3**	0	0	N-(5-amino-5-carboxypentanoyl)-L-cysteinyl-D-valine synthase AcvA	acvA	Delta-(L-alpha-aminoadipyl)-L-cysteinyl-D-valine synthetase	**prot_ID_480**	**AN2621**	ED
AN2622.3	-1	873	isopenicillin N synthetase	aatA	Isopenicillin-N N-acyltransferase	prot_ID_1092	AN2622	
AN2623.3	-2	614	acyl-coenzyme A:6-aminopenicillanic-acid-acyltransferase precursor		Acyl-coenzyme A:6-aminopenicillanic-acid-acyltransferase precursor	prot_ID_581	AN2623	ED
AN2624.3	-3	2161	conserved hypothetical protein		Protein of unknown function	prot_ID_99	AN2624	
AN11354.3	-4	772	hypothetical protein		AN11354 was deleted in current assembly, see AN12328			
n/a					Protein of unknown function	prot_ID_1144	AN12328	
AN2625.3	-5	1274	conserved hypothetical protein		Transmembrane transporter	prot_ID_692	AN2625	
n/a					Protein of unknown function	prot_ID_205	AN2626	
n/a					Mariner transposon-related ORF	prot_ID_384	AN10329	

pkb cluster

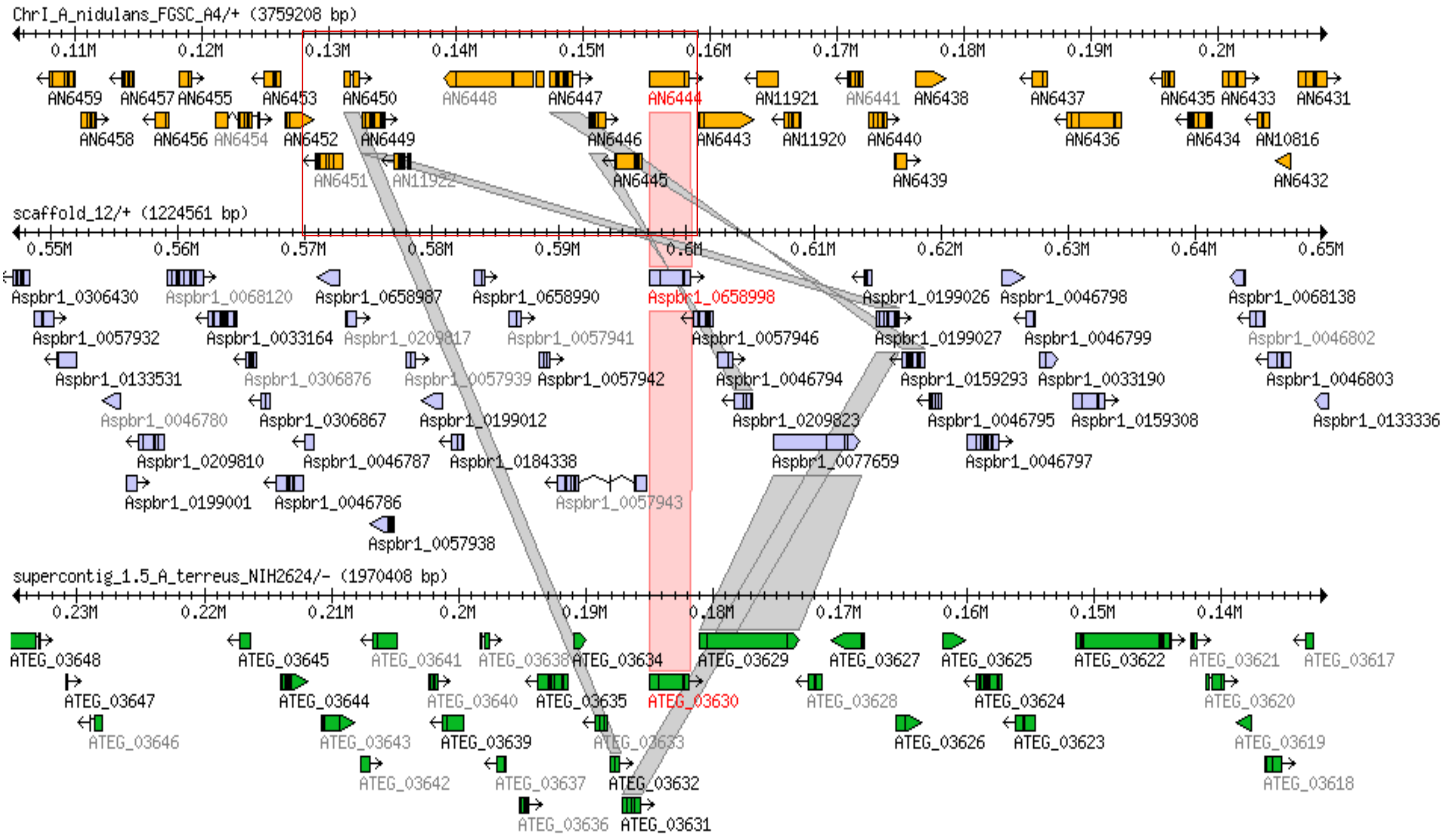

ChrI_A_nidulans_FGSC_A4/+ (3759208 bp)
0.11M 0.12M 0.13M 0.14M 0.15M 0.16M 0.17M 0.18M 0.19M 0.2M
AN6459 AN6457 AN6455 AN6453 AN6450 AN6448 AN6447 AN6444 AN11921 AN6441 AN6438 AN6437 AN6435 AN6433 AN6431
AN6458 AN6456 AN6454 AN6452 AN6449 AN6446 AN6443 AN11920 AN6440 AN6436 AN6434 AN10816
AN6451 AN11922 AN6445 AN6439 AN6432
scaffold_12/+ (1224561 bp)
0.55M 0.56M 0.57M 0.58M 0.59M 0.6M 0.61M 0.62M 0.63M 0.64M 0.65M
Aspbr1_0306430 Aspbr1_0068120 Aspbr1_0658987 Aspbr1_0658990 Aspbr1_0658998 Aspbr1_0199026 Aspbr1_0046798 Aspbr1_0068138
Aspbr1_0057932 Aspbr1_0033164 Aspbr1_0209817 Aspbr1_0057941 Aspbr1_0057946 Aspbr1_0199027 Aspbr1_0046799 Aspbr1_0046802
Aspbr1_0133531 Aspbr1_0306876 Aspbr1_0057939 Aspbr1_0057942 Aspbr1_0046794 Aspbr1_0159293 Aspbr1_0033190 Aspbr1_0046803
Aspbr1_0046780 Aspbr1_0306867 Aspbr1_0199012 Aspbr1_0209823 Aspbr1_0046795 Aspbr1_0159308 Aspbr1_0133336
Aspbr1_0209810 Aspbr1_0046787 Aspbr1_0184338 Aspbr1_0077659 Aspbr1_0046797
Aspbr1_0199001 Aspbr1_0046786 Aspbr1_0057943
Aspbr1_0057938
supercontig_1.5_A_terreus_NIH2624/- (1970408 bp)
0.23M 0.22M 0.21M 0.2M 0.19M 0.18M 0.17M 0.16M 0.15M 0.14M
ATEG_03648 ATEG_03645 ATEG_03641 ATEG_03638 ATEG_03634 ATEG_03629 ATEG_03627 ATEG_03625 ATEG_03622 ATEG_03621 ATEG_03617
ATEG_03647 ATEG_03644 ATEG_03640 ATEG_03635 ATEG_03630 ATEG_03628 ATEG_03624 ATEG_03620
ATEG_03646 ATEG_03643 ATEG_03639 ATEG_03633 ATEG_03626 ATEG_03623 ATEG_03619
ATEG_03642 ATEG_03637 ATEG_03632 ATEG_03618
ATEG_03636 ATEG_03631

pkb cluster

Gene id (SMURF)*	Gene positions	Gene distance	Annotated_gene_function (SMURF)	AspGD gene name	AspGD manual annotation and notes	antiSMASH ID	Gene ID	
AN6436.3	15	1688	hypothetical protein		Transmembrane transporter	n/a		
AN6437.3	14	2435	conserved hypothetical protein		Protein of unknown function	n/a		
AN11508.3	13	3575	predicted protein		Deleted by the Broad	n/a		
AN6438.3	12	558	conserved hypothetical protein		Dipeptidyl-peptidase	prot_ID_6	AN6438	
AN6439.3	11	1202	conserved hypothetical protein		Protein of unknown function	prot_ID_692	AN6439	
AN6440.3	10	583	conserved hypothetical protein		Protein of unknown function	prot_ID_891	AN6440	
AN6441.3	9	2059	conserved hypothetical protein		Protein of unknown function	prot_ID_323	AN6441	
n/a					Oxidoreductase	prot_ID_1298	AN11920	
n/a					Protein of unknown function	prot_ID_1257	AN11921	
AN10817.3	8	957	predicted protein		Deleted ORF	n/a		
AN6442.3	7	85	conserved hypothetical protein		Deleted by the Broad	n/a		
AN6443.3	6	753	conserved hypothetical protein		Oxidoreductase	prot_ID_447	AN6443	
AN6444.3	5	636	NRPS-like enzyme		NRPS-like enzyme	prot_ID_1133	AN6444	EP
AN6445.3	4	828	conserved hypothetical protein		Aryl-alcohol oxidase-related protein	prot_ID_572	AN6445	
AN6446.3	3	325	conserved hypothetical protein		Myb-like transcription factor	prot_ID_5	AN6446	
AN11509.3	2	357	predicted protein		Deleted by the Broad	n/a		
AN6447.3	1	565	conserved hypothetical protein		O-methyltransferase	prot_ID_690	AN6447	
n/a					Protein of unknown function	prot_ID_1310	AN11922	
n/a				pkbA	Putative polyketide synthase; involved in cichorine production	n/a	AN6448	
n/a				*CYP5077A1*	cytochrome P450	n/a	AN6449	
n/a					tetrahydroxynaphthalene reductase	n/a	AN6450	
n/a					transmembrane transporter	n/a	AN6451	EP

pkdA cluster

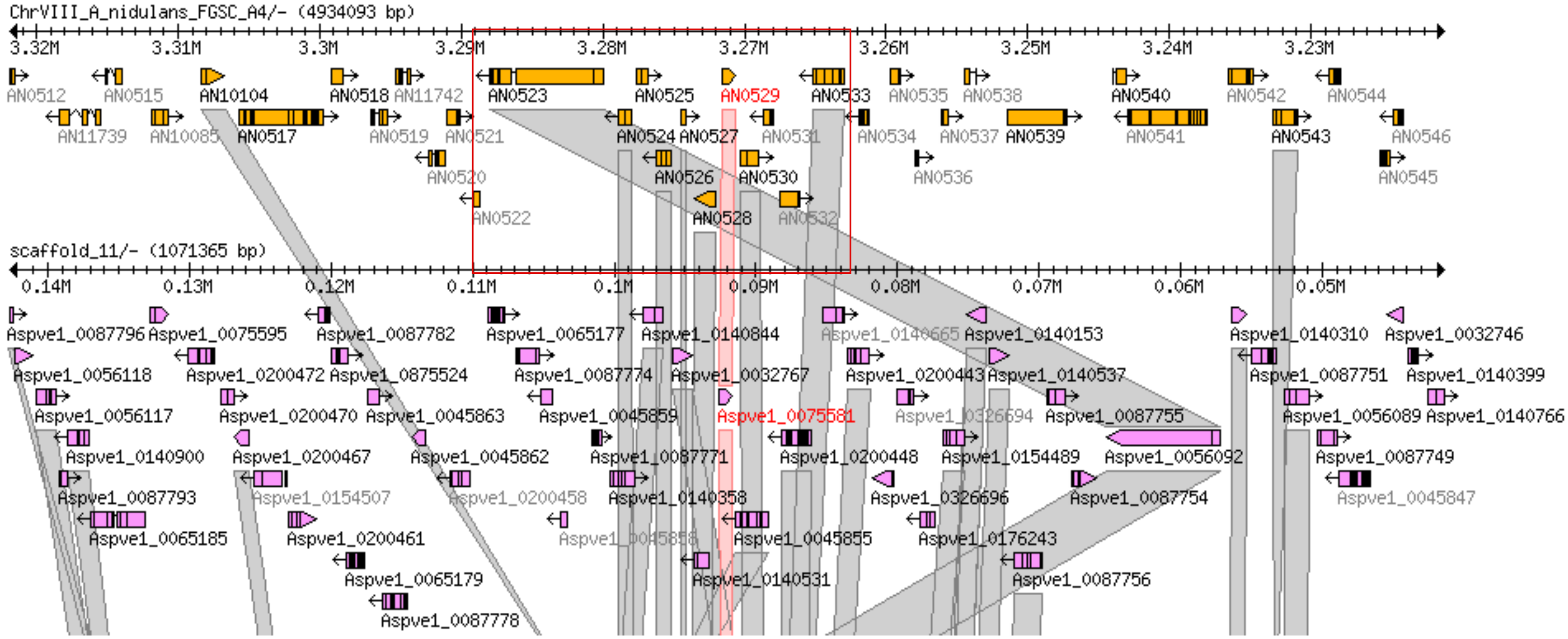

pkdA cluster

Gene id (SMURF)*	Gene positions	Gene distance	Annotated_gene_function (SMURF)	AspGD gene name	AspGD manual annotation and notes	antiSMASH ID	Gene ID	
n/a					Protein of unknown function	prot_ID_911	AN10104	
n/a					Chitinase	prot_ID_1677	AN0517	
n/a					Protein of unknown function	prot_ID_454	AN0518	
n/a					Protein of unknown function	prot_ID_910	AN0519	
n/a					Protein of unknown function	prot_ID_1720	AN11742	
AN0520.3	3	301	conserved hypothetical protein		UDP-N-acetylmuramate dehydrogenase	prot_ID_218	AN0520	
AN0521.3	2	917	conserved hypothetical protein		Oxidoreductase	prot_ID_671	AN0521	
AN0522.3	1	720	hypothetical protein		Protein of unknown function	prot_ID_1135	AN0522	
**AN0523.3**	0	0	polyketide synthase	pkdA	Polyketide synthase	**prot_ID_1596**	**AN0523**	ECS
AN0524.3	-1	1032	conserved hypothetical protein		Protein of unknown function	prot_ID_372	AN0524	
AN0525.3	-2	271	conserved hypothetical protein		Oxidoreductase	prot_ID_827	AN0525	
AN0526.3	-3	497	conserved hypothetical protein		Oxidoreductase	prot_ID_1302	AN0526	
AN0527.3	-4	661	conserved hypothetical protein		histidine acid phosphatase	prot_ID_76	AN0527	
AN0528.3	-5	786	conserved hypothetical protein		Transmembrane transporter	prot_ID_530	AN0528	
AN0529.3	-6	485	conserved hypothetical protein		Protein of unknown function	prot_ID_995	AN0529	
AN0530.3	-7	524	conserved hypothetical protein		Monooxygenase	prot_ID_295	AN0530	
AN0531.3	-8	353	conserved hypothetical protein		Protein of unknown function	prot_ID_747	AN0531	
AN0532.3	-9	563	conserved hypothetical protein		DDE1 transposon-related ORF	prot_ID_1218	AN0532	
AN0533.3	-10	1006	conserved hypothetical protein		Zn(II)2Cys6 transcription factor	prot_ID_1675	AN0533	ECS
n/a					GTPase	prot_ID_452	AN0534	

pkf cluster

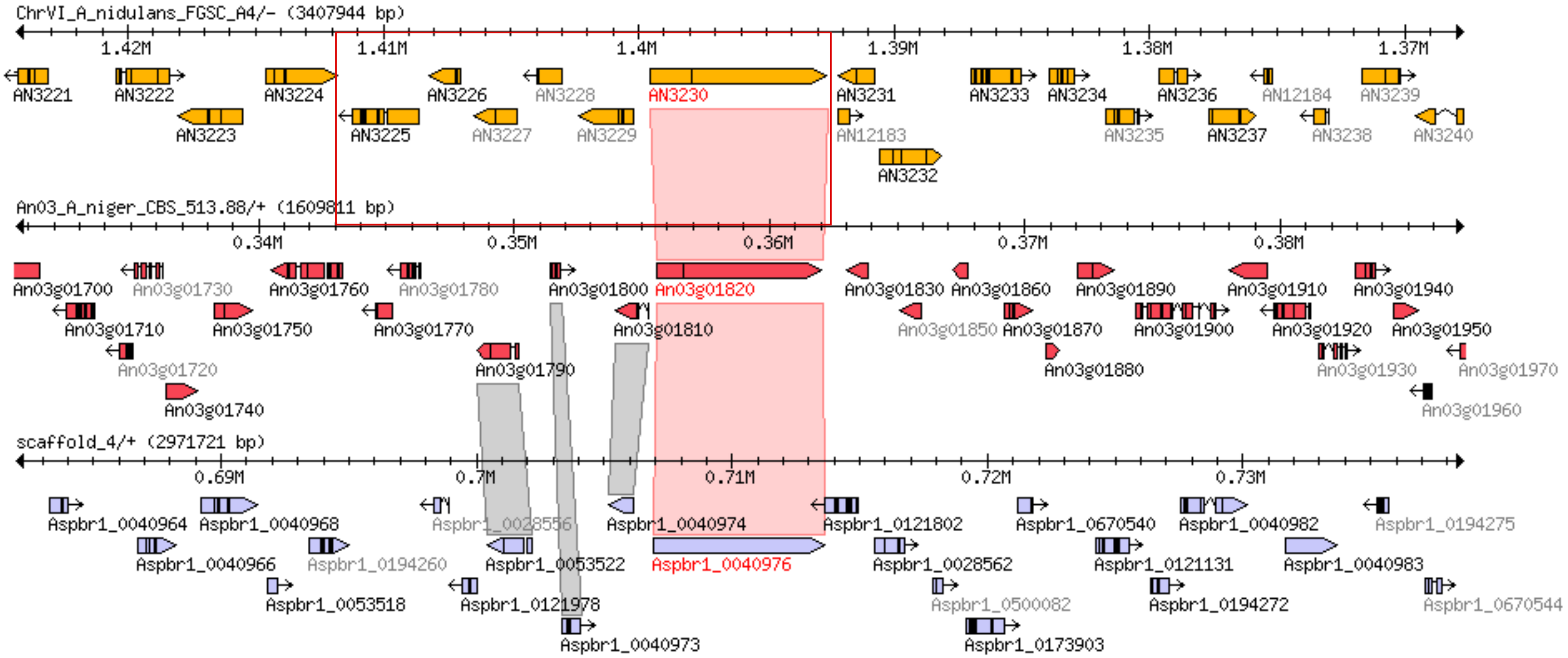
ChrVI_A_nidulans_FGSC_A4/- (3407944 bp)
1.42M
1.41M
1.4M
1.39M
1.38M
1.37M
AN3221
AN3222
AN3223
AN3224
AN3225
AN3226
AN3227
AN3228
AN3229
AN3230
AN3231
AN12183
AN3232
AN3233
AN3234
AN3235
AN3236
AN3237
AN12184
AN3238
AN3239
AN3240
An03_A_niger_CBS_513.88/+ (1609811 bp)
0.34M
0.35M
0.36M
0.37M
0.38M
An03g01700
An03g01710
An03g01720
An03g01730
An03g01740
An03g01750
An03g01760
An03g01770
An03g01780
An03g01790
An03g01800
An03g01810
An03g01820
An03g01830
An03g01850
An03g01860
An03g01870
An03g01880
An03g01890
An03g01900
An03g01910
An03g01920
An03g01930
An03g01940
An03g01950
An03g01960
An03g01970
scaffold_4/+ (2971721 bp)
0.69M
0.7M
0.71M
0.72M
0.73M
Aspbr1_0040964
Aspbr1_0040966
Aspbr1_0040968
Aspbr1_0053518
Aspbr1_0194260
Aspbr1_0028556
Aspbr1_0053522
Aspbr1_0121978
Aspbr1_0040973
Aspbr1_0040974
Aspbr1_0040976
Aspbr1_0121802
Aspbr1_0028562
Aspbr1_0500082
Aspbr1_0173903
Aspbr1_0670540
Aspbr1_0121131
Aspbr1_0194272
Aspbr1_0040982
Aspbr1_0040983
Aspbr1_0194275
Aspbr1_0670544

pkf cluster

Gene id (SMURF)*	Gene positions	Gene distance	Annotated_gene_function (SMURF)	AspGD gene name	AspGD manual annotation and notes	antiSMASH ID	Gene ID	
n/a						n/a		
n/a						n/a		
AN3217.3	13	2312	conserved hypothetical protein		Zn(II)2Cys6 transcription factor	n/a	AN3217	
AN3218.3	12	300	conserved hypothetical protein		Protein of unknown function	n/a	AN3218	
AN3219.3	11	648	hypothetical protein		Protein of unknown function	n/a	AN3219	
AN3220.3	10	795	conserved hypothetical protein		Predicted substrate-specific transmembrane transporter	n/a	AN3220	
AN3221.3	9	2732	conserved hypothetical protein		Transmembrane transporter	n/a	AN3221	
AN3222.3	8	614	conserved hypothetical protein		IMP 5'-nucleotidase	prot_ID_646	AN3222	
AN3223.3	7	661	6-phosphofructokinase	pfkA	6-phosphofructokinase	prot_ID_157	AN3223	
AN3224.3	6	1400	conserved hypothetical protein		RNA polymerase II mediator complex component SRB4	prot_ID_748	AN3224	
AN3225.3	5	677	conserved hypothetical protein		Cytochrome P450	prot_ID_253	AN3225	EP
AN3226.3	4	567	conserved hypothetical protein		HAS domain protein	prot_ID_855	AN3226	
AN3227.3	3	951	conserved hypothetical protein		Monooxygenase	prot_ID_362	AN3227	
AN3228.3	2	741	conserved hypothetical protein		Prenyltransferase	prot_ID_968	AN3228	
AN3229.3	1	922	conserved hypothetical protein		Aryl-alcohol oxidase	prot_ID_466	AN3229	
**AN3230.3**	0	0	polyketide synthase	pkfA	Polyketide synthase	**prot_ID_643**	**AN3230**	ECS, EP
n/a					Protein of unknown function	prot_ID_1124	AN12183	
AN3231.3	-1	380	hypothetical protein		Domain(s) with predicted metal ion transmembrane transporter activity	prot_ID_156	AN3231	
AN3232.3	-2	334	conserved hypothetical protein		Pantothenate transmembrane transporter	prot_ID_747	AN3232	
AN11379.3	-3	292	predicted protein		Deleted ORF	n/a		
AN3233.3	-4	1290	conserved hypothetical protein		Domain(s) with predicted substrate-specific transmembrane transporter activity	prot_ID_252	AN3233	
AN3234.3	-5	1060	conserved hypothetical protein		Oxidoreductase	prot_ID_853	AN3234	
AN3235.3	-6	1280	conserved hypothetical protein		Monooxygenase	prot_ID_360	AN3235	
AN3236.3	-7	792	conserved hypothetical protein		O-methyltransferase	prot_ID_965	AN3236	
AN3237.3	-8	866	conserved hypothetical protein		Protein of unknown function	prot_ID_464	AN3237	

n/a	Protein of unknown function	prot_ID_1137	AN12184
n/a	Protein of unknown function	prot_ID_1073	AN3238

pkg cluster

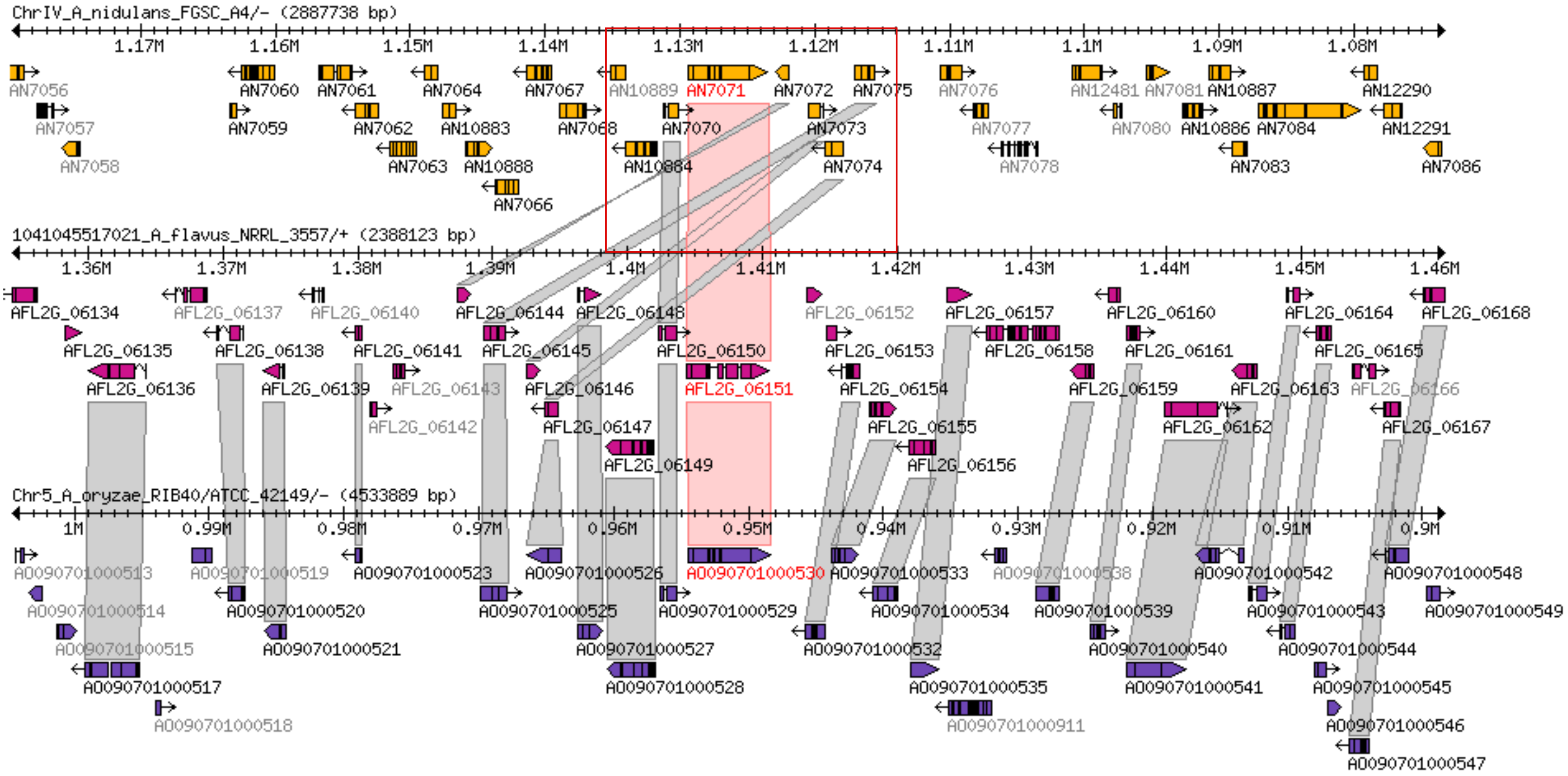
ChrIV_A_nidulans_FGSC_A4/- (2887738 bp)
1.17M
1.16M
1.15M
1.14M
1.13M
1.12M
1.11M
1.1M
1.09M
1.08M
AN7056
AN7060
AN7061
AN7064
AN7067
AN10889
AN7071
AN7072
AN7075
AN7076
AN12481
AN7081
AN10887
AN12290
AN7057
AN7059
AN7062
AN10883
AN7068
AN7070
AN7073
AN7077
AN7080
AN10886
AN7084
AN12291
AN7058
AN7063
AN10888
AN10884
AN7074
AN7078
AN7083
AN7086
AN7066
1041045517021_A_flavus_NRRL_3557/+ (2388123 bp)
1.36M
1.37M
1.38M
1.39M
1.4M
1.41M
1.42M
1.43M
1.44M
1.45M
1.46M
AFL2G_06134
AFL2G_06137
AFL2G_06140
AFL2G_06144
AFL2G_06148
AFL2G_06152
AFL2G_06157
AFL2G_06160
AFL2G_06164
AFL2G_06168
AFL2G_06135
AFL2G_06138
AFL2G_06141
AFL2G_06145
AFL2G_06150
AFL2G_06153
AFL2G_06158
AFL2G_06161
AFL2G_06165
AFL2G_06136
AFL2G_06139
AFL2G_06143
AFL2G_06146
AFL2G_06151
AFL2G_06154
AFL2G_06159
AFL2G_06163
AFL2G_06166
AFL2G_06142
AFL2G_06147
AFL2G_06155
AFL2G_06162
AFL2G_06167
AFL2G_06149
AFL2G_06156
Chr5_A_oryzae_RIB40/ATCC_42149/- (4533889 bp)
1M
0.99M
0.98M
0.97M
0.96M
0.95M
0.94M
0.93M
0.92M
0.91M
0.9M
A0090701000513
A0090701000519
A0090701000523
A0090701000526
A0090701000530
A0090701000533
A0090701000538
A0090701000542
A0090701000548
A0090701000514
A0090701000520
A0090701000525
A0090701000529
A0090701000534
A0090701000539
A0090701000543
A0090701000549
A0090701000515
A0090701000521
A0090701000527
A0090701000532
A0090701000540
A0090701000544
A0090701000517
A0090701000528
A0090701000535
A0090701000541
A0090701000545
A0090701000518
A0090701000911
A0090701000546
A0090701000547

pkg cluster

Gene id (SMURF)*	Gene positions	Gene distance	Annotated_gene_function (SMURF)	AspGD gene name	AspGD manual annotation and notes	antiSMASH ID	Gene ID	
n/a					Protein of unknown function	n/a	AN7059	
n/a					Monophenol monooxygenase	n/a	AN7060	
AN7061.3	12	83	hypothetical protein		Zn(II)2Cys6 transcription factor	n/a	AN7061	
AN7062.3	11	852	conserved hypothetical protein		Protein of unknown function	n/a	AN7062	
AN7063.3	10	532	conserved hypothetical protein		N,N-dimethylaniline monooxygenase	n/a	AN7063	
AN7064.3	9	357	conserved hypothetical protein		Fumarylacetoacetate hydrolase family protein	prot_ID_650	AN7064	
AN10883.3	8	662	hypothetical protein		Protein of unknown function	prot_ID_391	AN10883	
AN10888.3	7	243	hypothetical protein		Monooxygenase	prot_ID_150	AN10888	
AN7066.3	6	557	conserved hypothetical protein	CYP670A1	Cytochrome P450	prot_ID_741	AN7066	
AN7067.3	5	650	conserved hypothetical protein		Domain(s) with predicted substrate-specific transmembrane transporter activity	prot_ID_322	AN7067	
AN7068.3	4	1785	conserved hypothetical protein		Vanillyl alcohol oxidase	prot_ID_839	AN7068	
AN10889.3	3	36	conserved hypothetical protein		O-methyltransferase	prot_ID_656	AN10889	EP
AN10884.3	2	459	conserved hypothetical protein		Oxidoreductase	prot_ID_904	AN10884	
AN7070.3	1	596	conserved hypothetical protein	pkgB	Beta-lactamase-type thioesterase	prot_ID_563	AN7070	
**AN7071.3**	0	0	polyketide synthase	pkgA	Polyketide synthase	**prot_ID_144**	**AN7071**	
AN7072.3	-1	455	conserved hypothetical protein		Zn(II)2Cys6 transcription factor	prot_ID_649	AN7072	
AN7073.3	-2	1428	conserved hypothetical protein		Zn(II)2Cys6 transcription factor	prot_ID_231	AN7073	
AN7074.3	-3	368	conserved hypothetical protein		Oxidoreductase	prot_ID_740	AN7074	
AN7075.3	-4	927	conserved hypothetical protein		UDP-N-acetylmuramate dehydrogenase	prot_ID_321	AN7075	ECS, EP
n/a					Protein of unknown function	prot_ID_837	AN7076	
n/a				acnA	Actinin-like protein	prot_ID_412	AN7077	
n/a					Protein of unknown function	prot_ID_930	AN7078	

pkh cluster

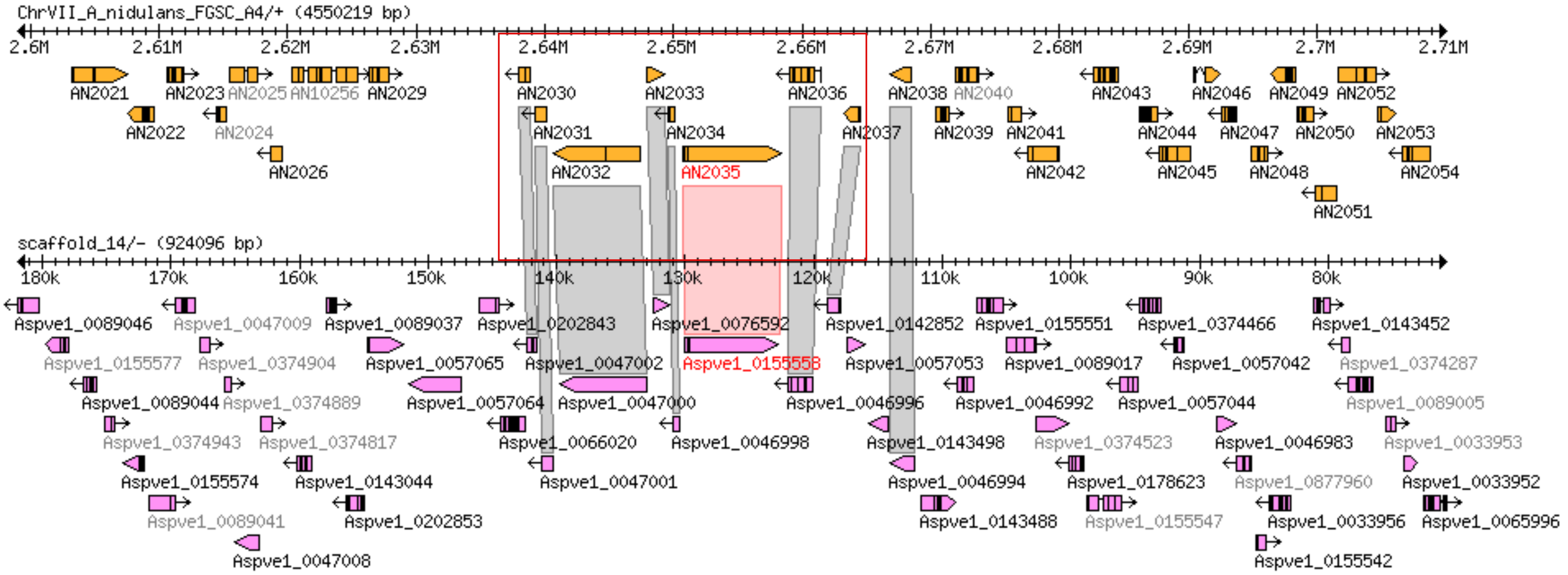

ChrVII_A_nidulans_FGSC_A4/+ (4550219 bp)
2.6M
2.61M
2.62M
2.63M
2.64M
2.65M
2.66M
2.67M
2.68M
2.69M
2.7M
2.71M
AN2021
AN2022
AN2023
AN2024
AN2025
AN10256
AN2026
AN2029
AN2030
AN2031
AN2032
AN2033
AN2034
AN2035
AN2036
AN2037
AN2038
AN2039
AN2040
AN2041
AN2042
AN2043
AN2044
AN2045
AN2046
AN2047
AN2048
AN2049
AN2050
AN2051
AN2052
AN2053
AN2054
scaffold_14/- (924096 bp)
180k
170k
160k
150k
140k
130k
120k
110k
100k
90k
80k
Aspve1_0089046
Aspve1_0155577
Aspve1_0089044
Aspve1_0374943
Aspve1_0155574
Aspve1_0089041
Aspve1_0047008
Aspve1_0047009
Aspve1_0374904
Aspve1_0374889
Aspve1_0374817
Aspve1_0143044
Aspve1_0202853
Aspve1_0089037
Aspve1_0057065
Aspve1_0057064
Aspve1_0202843
Aspve1_0047002
Aspve1_0047000
Aspve1_0066020
Aspve1_0047001
Aspve1_0076592
Aspve1_0155558
Aspve1_0046998
Aspve1_0142852
Aspve1_0057053
Aspve1_0046996
Aspve1_0143498
Aspve1_0046994
Aspve1_0143488
Aspve1_0155551
Aspve1_0089017
Aspve1_0046992
Aspve1_0374523
Aspve1_0178623
Aspve1_0155547
Aspve1_0374466
Aspve1_0057042
Aspve1_0057044
Aspve1_0046983
Aspve1_0877960
Aspve1_0033956
Aspve1_0155542
Aspve1_0143452
Aspve1_0374287
Aspve1_0089005
Aspve1_0033953
Aspve1_0033952
Aspve1_0065996

pkh cluster

Gene id (SMURF)*	Gene positions	Gene distance	Annotated_gene_function (SMURF)	AspGD gene name	AspGD manual annotation and notes	antiSMASH ID	Gene ID	
n/a					F-box protein	prot_ID_1288	AN2029	
n/a					Oxidoreductase	prot_ID_44	AN2030	ECS, IGD
n/a					Protein of unknown function	prot_ID_858	AN2031	
AN2032.3	3	582	polyketide synthase	pkhA	Polyketide synthase	prot_ID_184	AN2032	
AN2033.3	2	383	conserved hypothetical protein		Monooxygenase	prot_ID_1003	AN2033	
AN2034.3	1	542	conserved hypothetical protein		Protein of unknown function	prot_ID_328	AN2034	
**AN2035.3**	0	0	polyketide synthase	pkhB	polyketide synthase	**prot_ID_1143**	**AN2035**	
AN2036.3	-1	692	conserved hypothetical protein		Zn(II)2Cys6 transcription factor	prot_ID_475	AN2036	
AN11323.3	-2	768	predicted protein		AN11323.3 was deleted from the current assembly	prot_ID_1226	AN10256	
AN2037.3	-3	1187	hypothetical protein similar to CipA protein		Oxidoreductase	prot_ID_1286	AN2037	
AN2038.3	-4	2542	conserved hypothetical protein		Oxidoreductase	prot_ID_616	AN2038	EP
AN2039.3	-5	2085	conserved hypothetical protein		Oxidoreductase	prot_ID_1430	AN2039	
AN2040.3	-6	518	conserved hypothetical protein	CYP552A21	Cytochrome P450	prot_ID_181	AN2040	
AN2041.3	-7	2200	conserved hypothetical protein		Protein of unknown function	prot_ID_1001	AN2041	
AN2042.3	-8	446	conserved hypothetical protein		FAD binding domain protein	prot_ID_327	AN2042	
AN2043.3	-9	2733	conserved hypothetical protein		Amino acid transmembrane transporter	n/a	AN2043	

pki cluster

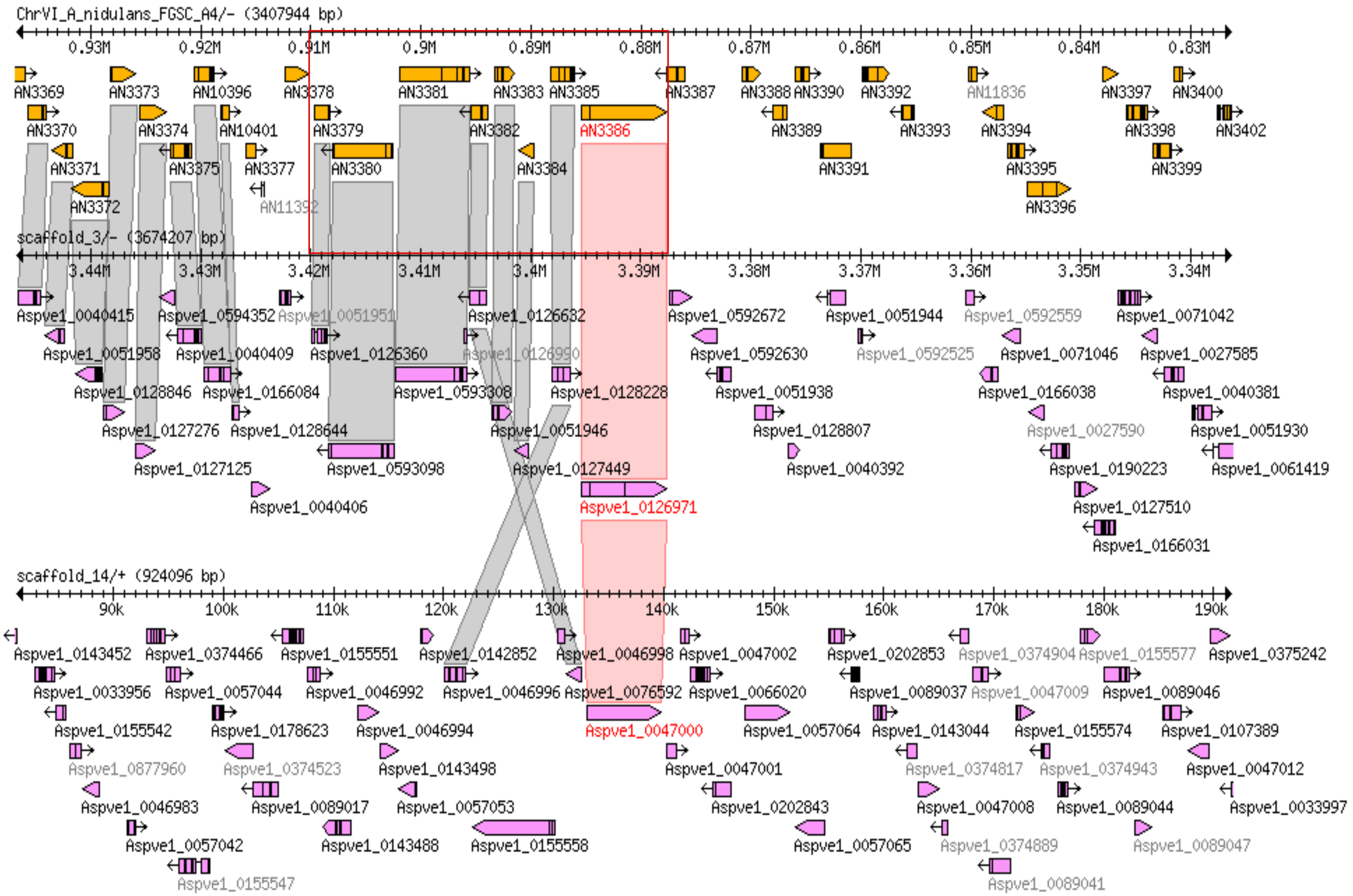
ChrVI_A_nidulans_FGSC_A4/- (3407944 bp)
0.93M
0.92M
0.91M
0.9M
0.89M
0.88M
0.87M
0.86M
0.85M
0.84M
0.83M
AN3369
AN3373
AN10396
AN3378
AN3381
AN3383
AN3385
AN3387
AN3388
AN3390
AN3392
AN11836
AN3397
AN3400
AN3370
AN3374
AN10401
AN3379
AN3382
AN3386
AN3389
AN3393
AN3394
AN3398
AN3402
AN3371
AN3375
AN3377
AN3380
AN3384
AN3391
AN3395
AN3399
AN3372
AN11392
AN3396
scaffold_3/- (3674207 bp)
3.44M
3.43M
3.42M
3.41M
3.4M
3.39M
3.38M
3.37M
3.36M
3.35M
3.34M
Aspve1_0040415
Aspve1_0594352
Aspve1_0051951
Aspve1_0126632
Aspve1_0592672
Aspve1_0051944
Aspve1_0592559
Aspve1_0071042
Aspve1_0051958
Aspve1_0040409
Aspve1_0126360
Aspve1_0126990
Aspve1_0592630
Aspve1_0592525
Aspve1_0071046
Aspve1_0027585
Aspve1_0128846
Aspve1_0166084
Aspve1_0593308
Aspve1_0128228
Aspve1_0051938
Aspve1_0166038
Aspve1_0040381
Aspve1_0127276
Aspve1_0128644
Aspve1_0051946
Aspve1_0128807
Aspve1_0027590
Aspve1_0051930
Aspve1_0127125
Aspve1_0593098
Aspve1_0127449
Aspve1_0040392
Aspve1_0190223
Aspve1_0061419
Aspve1_0040406
Aspve1_0126971
Aspve1_0127510
Aspve1_0166031
scaffold_14/+ (924096 bp)
90k
100k
110k
120k
130k
140k
150k
160k
170k
180k
190k
Aspve1_0143452
Aspve1_0374466
Aspve1_0155551
Aspve1_0142852
Aspve1_0046998
Aspve1_0047002
Aspve1_0202853
Aspve1_0374904
Aspve1_0155577
Aspve1_0375242
Aspve1_0033956
Aspve1_0057044
Aspve1_0046992
Aspve1_0046996
Aspve1_0076592
Aspve1_0066020
Aspve1_0089037
Aspve1_0047009
Aspve1_0089046
Aspve1_0155542
Aspve1_0178623
Aspve1_0046994
Aspve1_0047000
Aspve1_0057064
Aspve1_0143044
Aspve1_0155574
Aspve1_0107389
Aspve1_0877960
Aspve1_0374523
Aspve1_0143498
Aspve1_0047001
Aspve1_0374817
Aspve1_0374943
Aspve1_0047012
Aspve1_0046983
Aspve1_0089017
Aspve1_0057053
Aspve1_0202843
Aspve1_0047008
Aspve1_0089044
Aspve1_0033997
Aspve1_0057042
Aspve1_0143488
Aspve1_0155558
Aspve1_0057065
Aspve1_0374889
Aspve1_0089047
Aspve1_0155547
Aspve1_0089041

pki cluster

Gene id (SMURF)*	Gene positions	Gene distance	Annotated_gene_function (SMURF)	AspGD gene name	AspGD manual annotation and notes	antiSMASH ID	Gene ID	
n/a					Protein of unknown function	n/a	AN3379	ECS
AN3380.3	6	633	fatty acid synthase FasA	pkiB	fatty-acyl-CoA synthase	prot_ID_242	AN3380	
AN3381.3	5	514	conserved hypothetical protein	pkiC	Fatty acid synthetase subunit	prot_ID_843	AN3381	
AN3382.3	4	599	hypothetical protein	salA	Salicylate 1-monooxygenase	prot_ID_351	AN3382	
AN3383.3	3	337	conserved hypothetical protein		Transmembrane transporter	prot_ID_955	AN3383	
AN3384.3	2	1745	conserved hypothetical protein		Trichothecene 3-O-acetyltransferase	prot_ID_456	AN3384	
AN3385.3	1	1000	conserved hypothetical protein		Zn(II)2Cys6 transcription factor	prot_ID_1063	AN3385	
**AN3386.3**	0	0	polyketide synthase	pkiA	Polyketide synthase	**prot_ID_554**	**AN3386**	ECS
n/a				gprD	G-protein coupled receptor	prot_ID_68	AN3387	
n/a				amyF	Apha-amylase	prot_ID_664	AN3388	
n/a				xghA	Xylogalacturonan hydrolase	prot_ID_175	AN3389	
n/a				pmeA	Pectinesterase	prot_ID_349	AN3390	
n/a					C2H2 and Zn(2)-Cys(6) DNA binding transcription factor	prot_ID_954	AN3391	
n/a					Monooxygenase	prot_ID_455	AN3392	

Sterigmatocystin (stc) cluster

Experimentally determined cluster

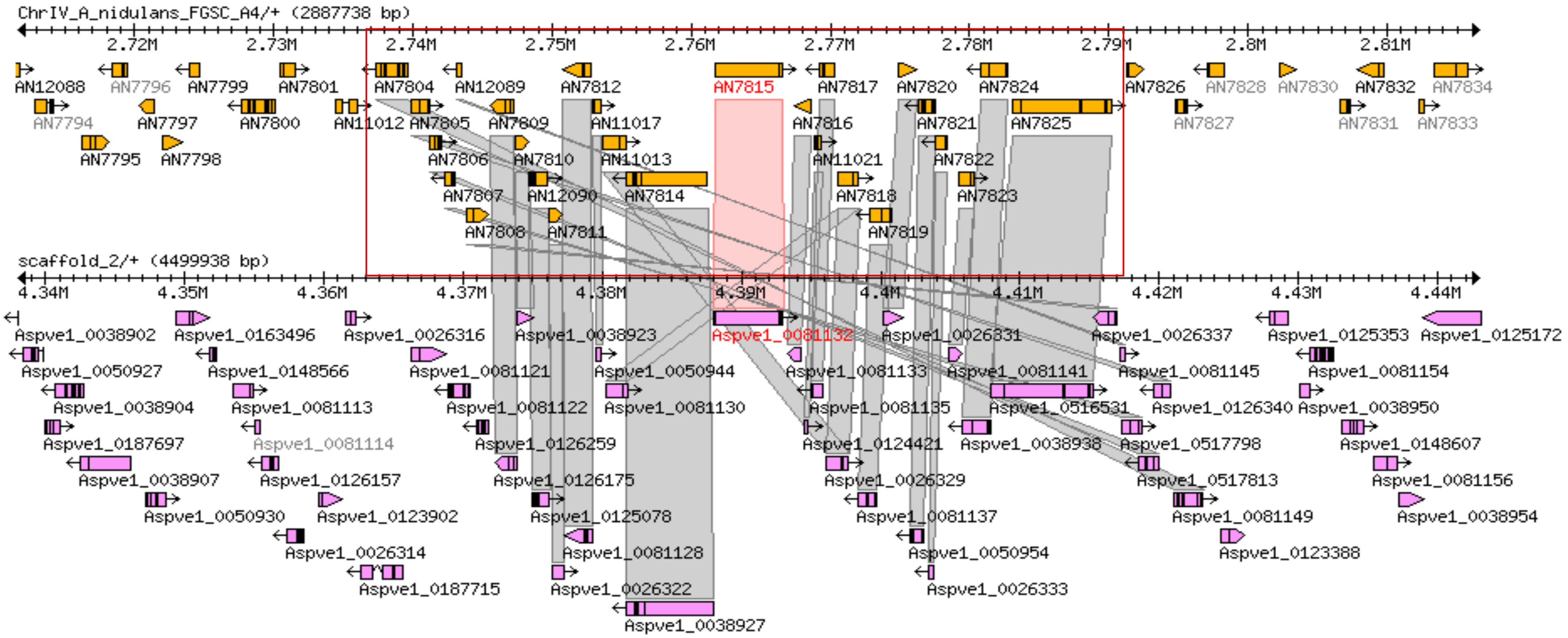

Sterigmatocystin (stc) cluster

Gene id (SMURF)*	Gene positions	Gene distance	Annotated_gene_function (SMURF)	AspGD gene name	AspGD manual annotation and notes	antiSMASH ID	Gene ID	
n/a					Protein of unknown function;	n/a	AN11012	
n/a				stcW	FAD-containing monooxygenase	n/a	AN7804	ED
n/a				stcV	Norsolorinic acid reductase	n/a	AN7805	
AN7806.3	11	199	ketoreductase StcU/VerA	stcU	Versicolorin reductase	n/a	AN7806	
AN7807.3	10	644	sterigmatocystin biosynthesis protein StcT	stcT	Translation elongation factor 1 gamma	n/a	AN7807	
AN7808.3	9	150	sterigmatocystin biosynthesis P450 monooxygenase StcS	stcS	sterigmatocystin biosynthesis CYP59 family P450 monooxygenase	n/a	AN7808	
AN11020.3	8	0	hypothetical protein		ORF not present in AspGD	n/a	AN11020	
AN7809.3	7	160	conserved hypothetical protein		Protein of unknown function	n/a	AN7809	
AN7810.3	6	1201	conserved hypothetical protein	stcQ	Protein of unknown function	n/a	AN7810	
AN7811.3	5	35	conserved hypothetical protein	stcO	Protein of unknown function	n/a	AN7811	
AN7812.3	4	188	conserved hypothetical protein	stcN	Versicolorin B synthase	n/a	AN7812	
AN11017.3	3	93	conserved hypothetical protein		Protein of unknown function	n/a	AN11017	
AN11013.3	2	5	hypothetical protein similar to VerB	stcK	Polyketide synthase/fatty acid synthase beta	n/a	AN11013	
AN7814.3	1	495	sterigmatocystin biosynthesis fatty acid synthase subunit beta	stcL	Polyketide synthase/fatty acid synthase alpha	n/a	AN7814	
**AN7815.3**	0	0	sterigmatocystin biosynthesis fatty acid synthase alpha subunit		Protein of unknown function	**prot_ID_37**	**AN7815**	
AN7816.3	-1	936	conserved hypothetical protein	stcI	Lipase/esterase	prot_ID_547	AN7816	
AN11021.3	-2	151	predicted protein		Protein of unknown function	prot_ID_665	AN11021	
AN7817.3	-3	190	conserved hypothetical protein		Protein of unknown function	prot_ID_125	AN7817	
AN7818.3	-4	176	conserved hypothetical protein	stcF	P450 monooxygenase	prot_ID_634	AN7818	
AN7819.3	-5	930	conserved hypothetical protein		Protein of unknown function	prot_ID_214	AN7819	
AN7820.3	-6	502	sterigmatocystin biosynthesis C6 transcription factor	aflR	Zn(II)2Cys6 transcription factor	prot_ID_361	AN7820	
AN7821.3	-7	192	conserved hypothetical protein	stcE	Norsolorinic acid reductase	prot_ID_876	AN7821	
AN7822.3	-8	314	conserved hypothetical protein		Protein of unknown function	prot_ID_456	AN7822	
AN7823.3	-9	1121	conserved hypothetical protein	stcC	peroxidase	prot_ID_36	AN7823	

AN7825.3	-10	2887	sterigmatocystin polyketide synthase	stcA	Polyketide synthase	prot_ID_123	AN7825	
AN7824.3	-11	134	sterigmatocystin biosynthesis P450 monooxygenase StcB	stcB	P450 monooxygenase	prot_ID_545	AN7824	ED
n/a					Protein of unknown function	prot_ID_632	AN7826	
n/a					Protein of unknown function	prot_ID_213	AN7827	
n/a					Unsaturated rhamnogalacturonan hydrolase	prot_ID_722	AN7828	
n/a					Protein of unknown function	prot_ID_452	AN7830	
n/a					Protein of unknown function	prot_ID_35	AN7831	
n/a					Oxidoreductase	prot_ID_544	AN7832	

Terriquinone (tdi) cluster

Experimentally determined cluster

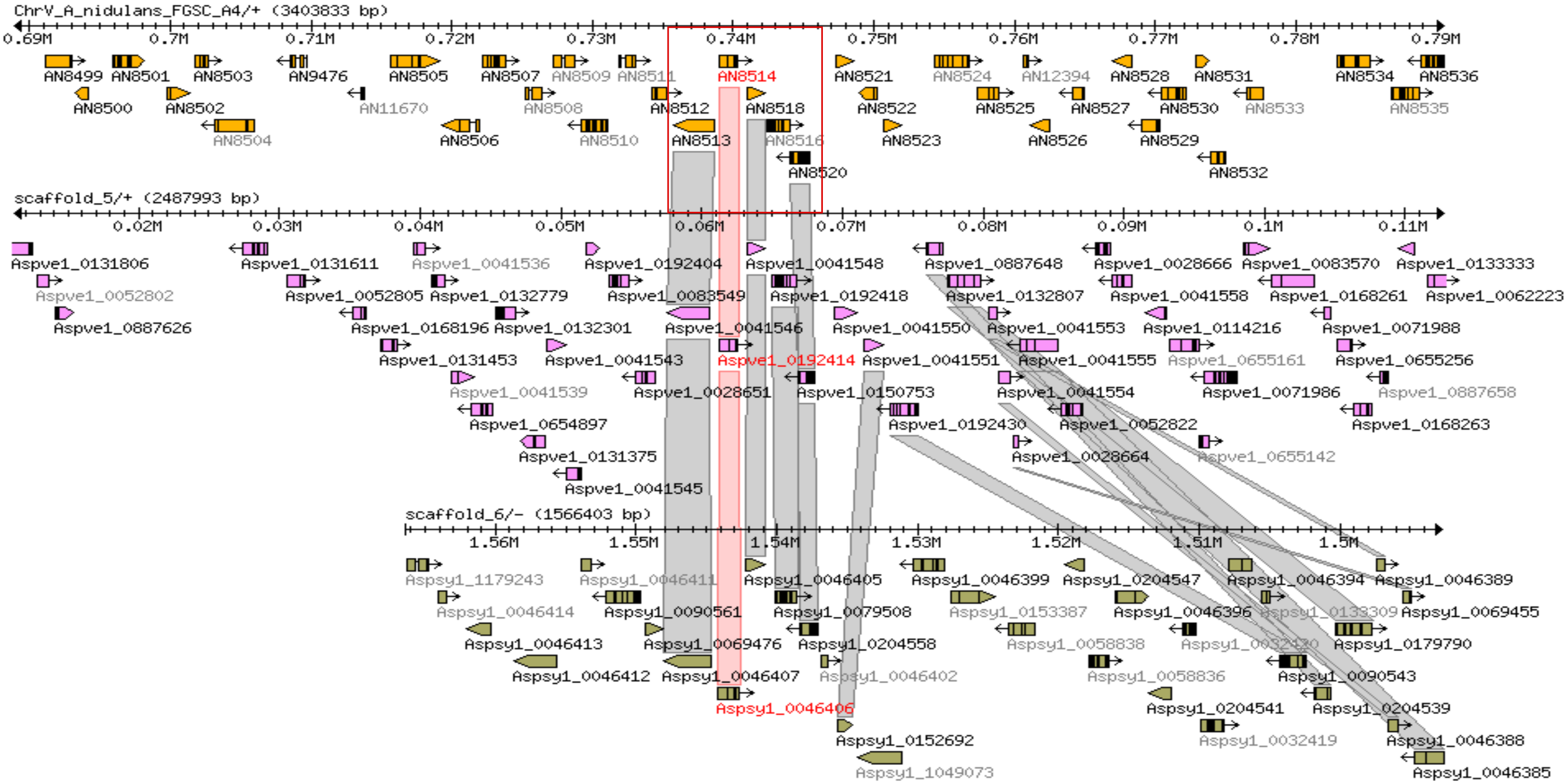

Terriquinone (tdi) cluster

Gene id (SMURF)*	Gene positions	Gene distance	Annotated_gene_function (SMURF)	AspGD gene name	AspGD manual annotation and notes	antiSMASH ID	Gene ID	
AN8506.3	8	306	conserved hypothetical protein		Zn(II)2Cys6 transcription factor	n/a		
AN8507.3	7	1377	conserved hypothetical protein		Transmembrane transporter	n/a		
AN8508.3	6	808	conserved hypothetical protein		Protein of unknown function	prot_ID_374	AN8508	
AN8509.3	5	414	conserved hypothetical protein		Zn(II)2Cys6 transcription factor	prot_ID_929	AN8509	
AN8510.3	4	824	conserved hypothetical protein	CYP686A1P	Cytochrome P450; possibly a pseudogene	prot_ID_66	AN8510	
AN8511.3	3	1034	conserved hypothetical protein		Branched chain amino acid aminotransferase	prot_ID_640	AN8511	
AN8512.3	2	287	conserved hypothetical protein		Protein of unknown function, adjacent to tdi (terrequinone) gene cluster	prot_ID_165	AN8512	
AN8513.3	1	390	NRPS-like enzyme	tdiA	Putative single-module nonribosomal peptide synthetase (NRPS)	prot_ID_737	AN8513	ED
**AN8514.3**	0	0	DMATS type aromatic prenyltransferase	tdiB	Asterriquinone prenyltransferase	**prot_ID_270**	**AN8514**	
AN8515.3	-1	651	conserved hypothetical protein			n/a		
AN8516.3	-2	217	conserved hypothetical protein	tdiD	Aminotransferase	prot_ID_372	AN8516	
n/a				tdiE	Protein of unknown function	n/a		
n/a				tdiC	NADPH-dependent quinone reductase	prot_ID_473	AN8518	
n/a				tdiE	Protein of unknown function	prot_ID_162	AN8520	ED
n/a					Protein of unknown function, adjacent to tdi (terrequinone) gene cluster	prot_ID_735	AN8521	
n/a						prot_ID_268	AN8522	

xptA-containing cluster

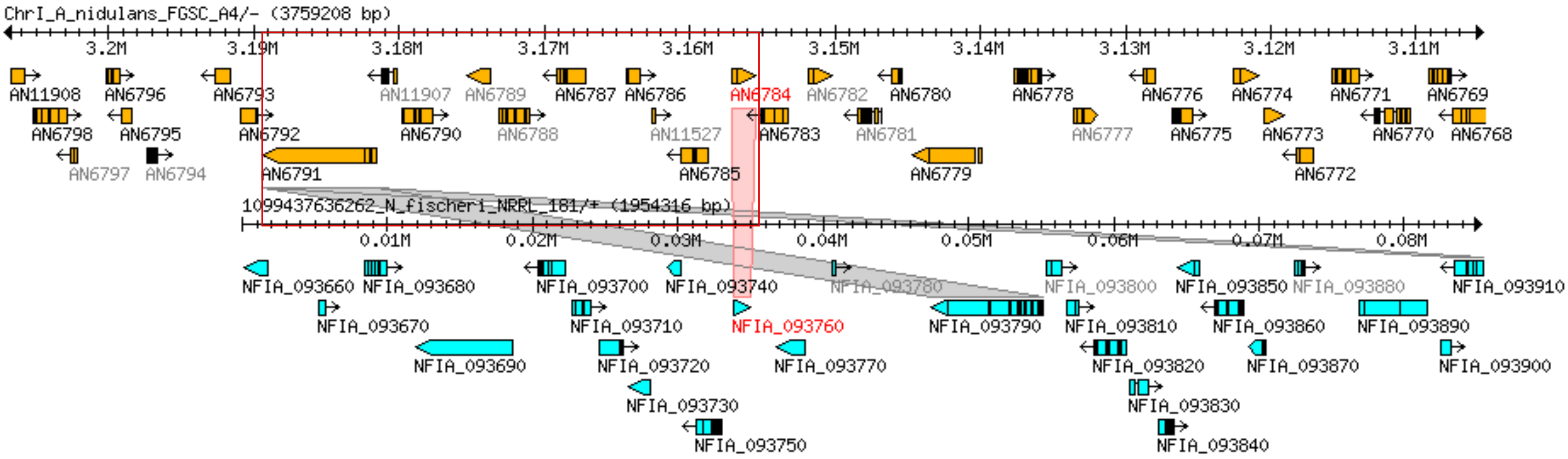

xptA-containing cluster

Gene id (SMURF)*	Gene positions	Gene distance	Annotated_gene_function (SMURF)	AspGD gene name	AspGD manual annotation and notes	antiSMASH ID	Gene ID	
AN6791.3	-8	1801	polyketide synthase, putative		Polyketide synthase	n/a	AN6791	FA, EP
AN6790.3	-7	2598	hypothetical protein		Zn(II)2Cys6 transcription factor	n/a	AN6790	
AN6789.3	-6	1015	conserved hypothetical protein		Protein of unknown function	n/a	AN6789	
AN6788.3	-5	1614	conserved hypothetical protein		Zn(II)2Cys6 transcription factor	n/a	AN6788	
AN6787.3	-4	2709	conserved hypothetical protein	CYP682C1	Cytochrome P450	n/a	AN6787	
AN6786.3	-3	907	hypothetical protein		Beta-1,4-endoglucanase	prot_ID_1076	AN6786	
AN11527.3	-2	1870	predicted protein		Protein of unknown function	prot_ID_1002	AN11527	
AN6785.3	-1	1786	conserved hypothetical protein		Protein of unknown function	prot_ID_391	AN6785	
**AN6784.3**	**0**	0	DMATS type aromatic prenyltransferase, putative	xptA	DMATS) type aromatic prenyltransferase	**prot_ID_954**	**AN6784**	FA, EP
AN6783.3	1	511	conserved hypothetical protein		Nucleobase transmembrane transporter	prot_ID_266	AN6783	
AN6782.3	2	1455	hypothetical protein		Sodium:dicarboxylate symporter	prot_ID_839	AN6782	
AN6781.3	3	1773	hypothetical protein		Protein of unknown function	prot_ID_152	AN6781	
AN6780.3	4	1202	conserved hypothetical protein		Protein of unknown function	prot_ID_720	AN6780	
AN6779.3	5	865	conserved hypothetical protein		ABC superfamily multidrug resistance-associated protein	n/a	AN6779	
AN6778.3	6	2235	conserved hypothetical protein		Transmembrane transporter	n/a	AN6778	
AN6777.3	7	2270	conserved hypothetical protein		F-box protein	n/a	AN6777	
AN6776.3	8	2843	conserved hypothetical protein		Hydrolase	n/a	AN6776	
AN6775.3	9	1227	conserved hypothetical protein	aatB	penicillin biosynthesis protein	n/a	AN6775	
AN6774.3	10	2879	conserved hypothetical protein		Transmembrane transporter	n/a	AN6774	
AN6773.3	11	532	conserved hypothetical protein		Triglyceride lipase	n/a	AN6773	
AN6772.3	12	896	conserved hypothetical protein		Protein of unknown function	n/a	AN6772	
AN6771.3	13	1250	conserved hypothetical protein		Protein of unknown function	n/a	AN6771	
AN6770.3	14	1038	conserved hypothetical protein		Amino acid transmembrane transporter	n/a	AN6770	

xptB-containing cluster

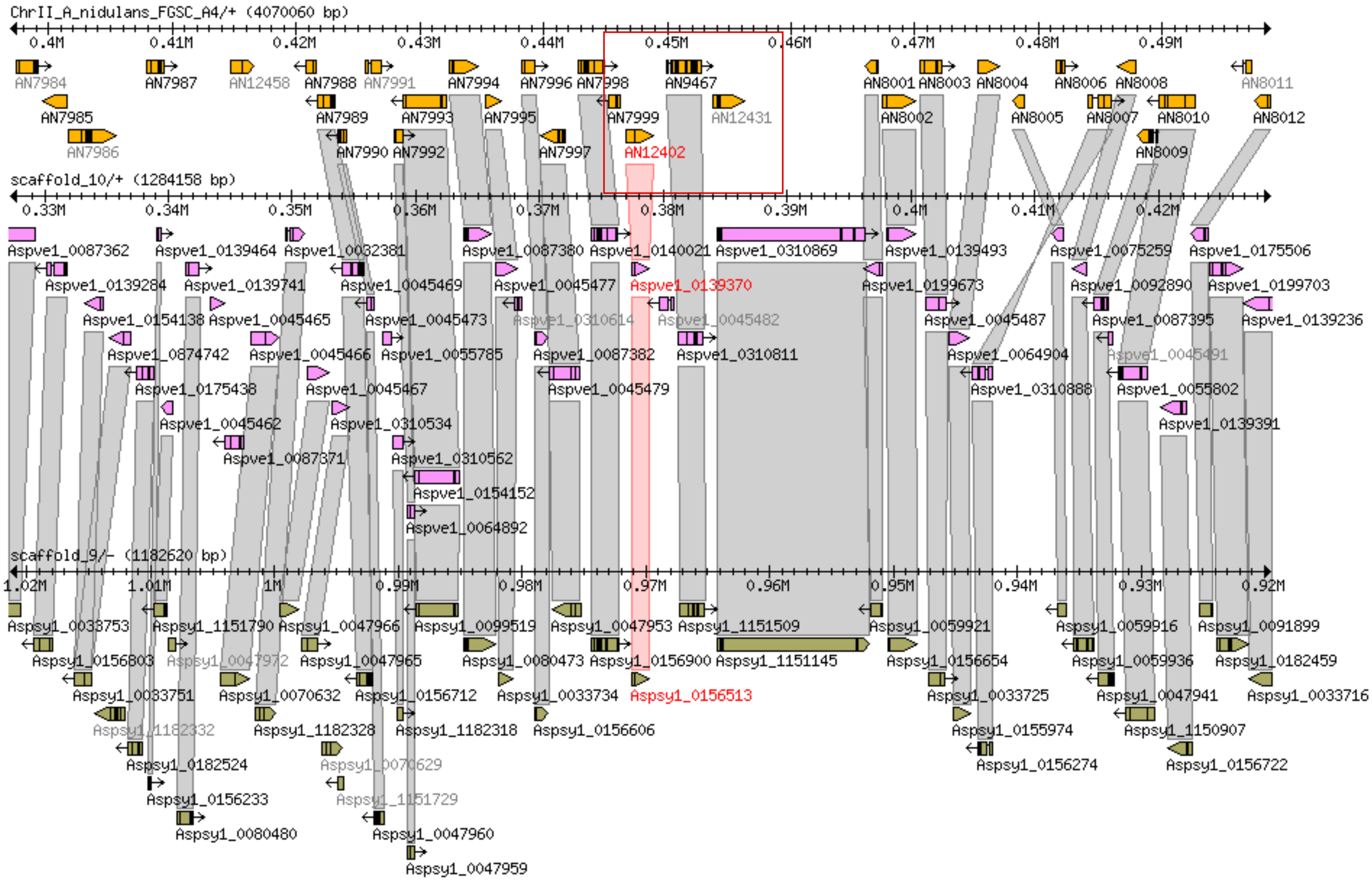

xptB-containing cluster

Gene id (SMURF)*	Gene positions	Gene distance	Annotated_gene_function (SMURF)	AspGD gene name	AspGD manual annotation and notes	antiSMASH ID	Gene ID	
n/a					Protein of unknown function	prot_ID_678	AN7996	
n/a					RNA polymerase III transcription factor subunit	prot_ID_74	AN7997	
n/a					GMC oxidoreductase	prot_ID_804	AN7998	
n/a					Oxidoreductase	prot_ID_202	AN7999	ECS
n/a				xptB	Prenyltransferase	prot_ID_1407	**AN12402**	
n/a					Serine/threonine phosphatase	prot_ID_601	AN9467	
n/a					Protein of unknown function	prot_ID_1451	AN12431	IGD

AN0016 cluster

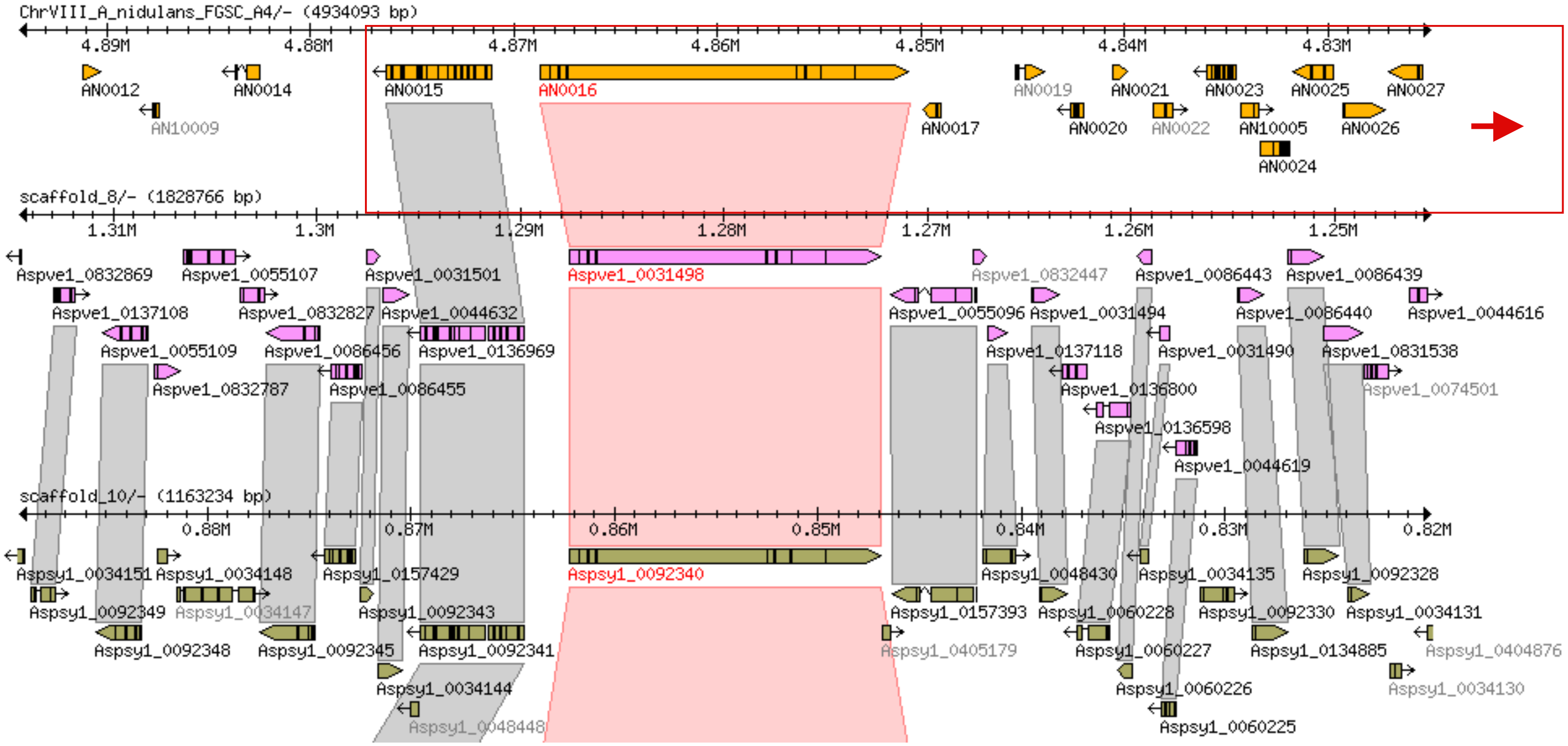

AN0016 cluster

Gene id (SMURF)*	Gene positions	Gene distance	Annotated_gene_function (SMURF)	AspGD gene name	AspGD manual annotation and notes	antiSMASH ID	Gene ID	
n/a					Protein of unknown function	prot_ID_562	AN10009	
n/a					Protein of unknown function	prot_ID_465	AN0014	
AN0015.3	1	2389	conserved hypothetical protein		Transmembrane transporter	prot_ID_922	AN0015	ECS, IGD
**AN0016.3**	0	0	nonribosomal peptide synthase pes1		Nonribosomal peptide synthase	**prot_ID_1400**	**AN0016**	
AN0017.3	-1	632	copper homeostasis protein CutC		Copper ion homeostasis protein	prot_ID_169	AN0017	
AN0019.3	-3	702	conserved hypothetical protein		Protein of unknown function	prot_ID_1093	AN0019	
AN0020.3	-4	1304	conserved hypothetical protein		Protein of unknown function	prot_ID_381	AN0020	
AN0021.3	-5	1452	conserved hypothetical protein		Protein of unknown function	prot_ID_837	AN0021	
AN0022.3	-6	1246	conserved hypothetical protein	aglA	alpha-1,4-galactosidase	prot_ID_1311	AN0022	
AN0023.3	-7	1592	conserved hypothetical protein		Oxidoreductase	prot_ID_85	AN0023	
AN10005.3	-8	290	hypothetical protein		Oxidoreductase	prot_ID_403	AN10005	
AN0024.3	-9	34	conserved hypothetical protein		Oxidoreductase	prot_ID_541	AN0024	
AN0025.3	-10	173	conserved hypothetical protein		N,N-dimethylaniline monooxygenase	prot_ID_1007	AN0025	
AN0026.3	-11	514	conserved hypothetical protein		Zn(II)2Cys6 transcription factor	n/a	AN0026	
AN0027.3	-12	61	conserved hypothetical protein		N,N-dimethylaniline monooxygenase	n/a	AN0027	
AN0028.3	-13	388	conserved hypothetical protein		Hydrolase	n/a	AN0028	
AN0029.3	-14	482	conserved hypothetical protein		Transmembrane transporter	n/a	AN0029	FA

AN0607 (sidC) cluster

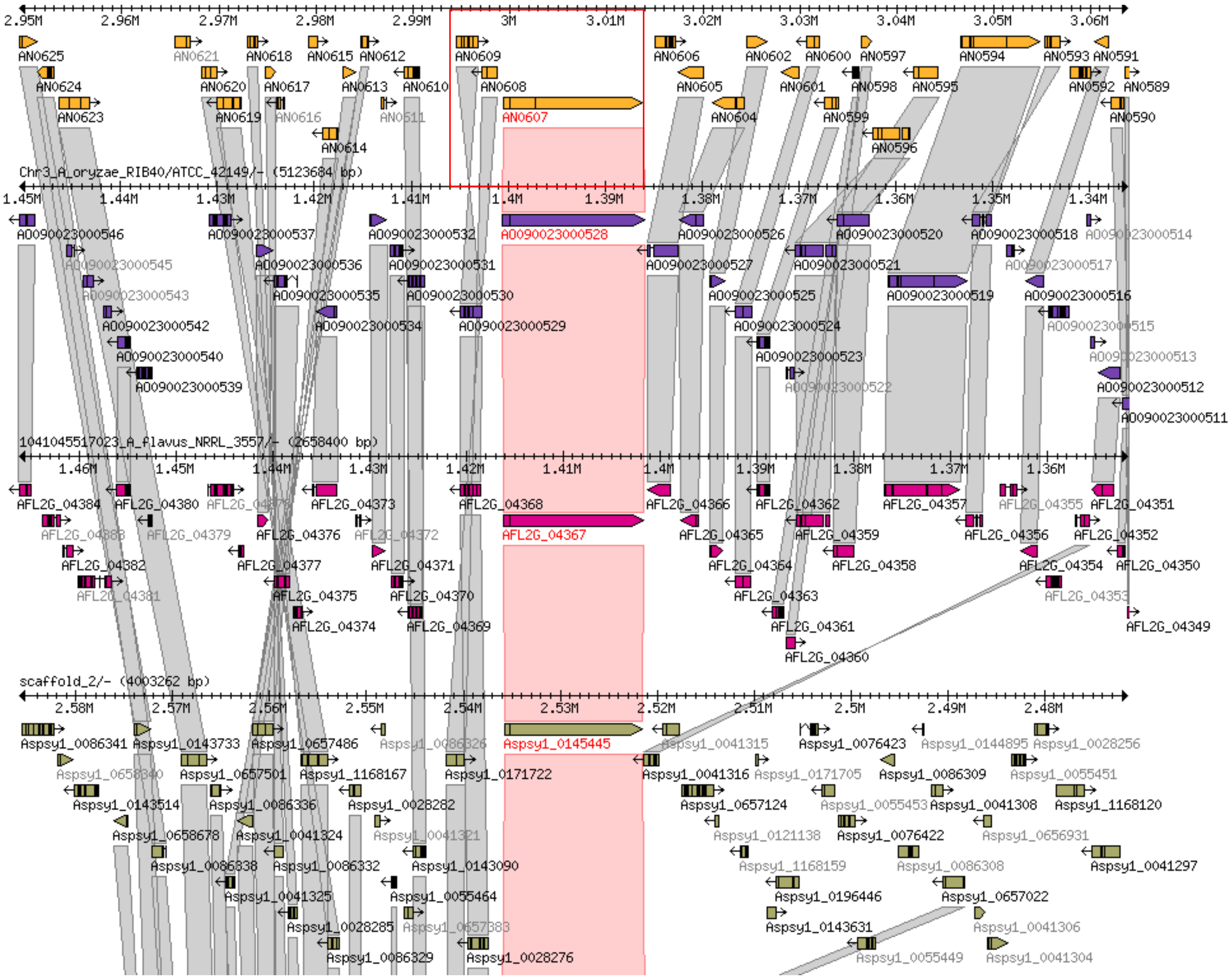

AN0607 (sidC) cluster

Gene id (SMURF)*	Gene positions	Gene distance	Annotated_gene_function (SMURF)	AspGD gene name	AspGD manual annotation and notes	antiSMASH ID	Gene ID	
AN0592.3	14	1007	conserved hypothetical protein		RNA binding protein	n/a	AN0592	
AN0593.3	13	579	conserved hypothetical protein		Dehydrogenase	n/a	AN0593	
AN0594.3	12	2588	conserved hypothetical protein		Cell morphogenesis protein	n/a	AN0594	
AN0595.3	11	596	NADPH--cytochrome P450 reductase		NADPH-cytochrome P450 reductase	n/a	AN0595	
AN0596.3	10	420	conserved hypothetical protein	ddbA	DNA damage binding protein		AN0596	
AN0597.3	9	453	hypothetical protein		Protein of unknown function	n/a	AN0597	
AN0598.3	8	1584	conserved hypothetical protein		Ortholog(s) have role in response to DNA damage stimulus	n/a	AN0598	
AN0599.3	7	425	conserved hypothetical protein		Oxidoreductase	prot_ID_1569	AN0599	
AN0600.3	6	1051	conserved hypothetical protein		Protein of unknown function	prot_ID_1023	AN0600	
AN0601.3	5	1423	conserved hypothetical protein		Transmembrane transporter	prot_ID_1496	AN0601	
AN0602.3	4	1202	conserved hypothetical protein		Protein of unknown function	prot_ID_273	AN0602	
AN0604.3	3	1058	conserved hypothetical protein		Endonuclease	prot_ID_1195	AN0604	
AN0605.3	2	309	conserved hypothetical protein		Protein of unknown function	prot_ID_1650	AN0605	
AN0606.3	1	1226	conserved hypothetical protein	CYP5076A1	Cytochrome P450	prot_ID_431	AN0606	
**AN0607.3**	0	0	nonribosomal peptide siderophore synthase SidC	sidC	Non-ribosomal peptide synthetase, siderophore biosynthesis	**prot_ID_887**	**AN0607**	ECS
n/a					Protein of unknown function	prot_ID_1364	AN0608	
n/a					N',N'',N'''-triacetylfusarinine C biosynthetic protein	prot_ID_134	AN0609	ECS, IGD
n/a					VIC potassium ion channel, beta subunit	prot_ID_1109	AN0610	
n/a					Protein of unknown function	prot_ID_1573	AN0611	
n/a					Protein of unknown function	prot_ID_348	AN0612	
n/a					Protein of unknown function	prot_ID_803	AN0613	
n/a					Transmembrane transporter	prot_ID_1277	AN0614	
n/a					Protein of unknown function	prot_ID_53	AN0615	
n/a					Protein of unknown function	prot_ID_508	AN0616	
n/a					Lyase domain predicted	prot_ID_968	AN0617	

AN10289 cluster

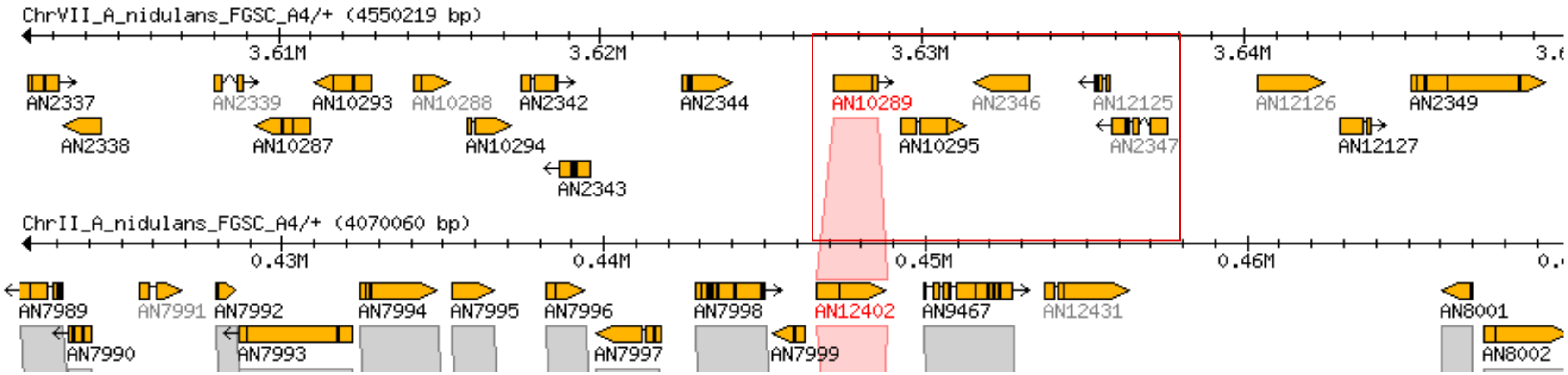

AN10289 cluster

Gene id (SMURF)*	Gene positions	Gene distance	Annotated_gene_function (SMURF)	AspGD gene name	AspGD manual annotation and notes	antiSMASH ID	Gene ID	
n/a					Protein of unknown function	n/a	AN2339	
n/a					Protein of unknown function	n/a	AN10287	
n/a					Protein of unknown function	n/a	AN10293	
n/a					Protein of unknown function	n/a	AN10288	
AN10294.3	4	312	conserved hypothetical protein		Zn(II)2Cys6 transcription factor	prot_ID_162	AN10294	
AN2342.3	3	136	conserved hypothetical protein		Protein of unknown function	prot_ID_1034	AN2342	
AN2343.3	2	2917	conserved hypothetical protein		Nitroreductase	prot_ID_364	AN2343	
AN2344.3	1	3119	conserved hypothetical protein		Transmembrane transporter	prot_ID_1175	AN2344	
**AN10289.3**	0	0	DMATS type aromatic prenyltransferase		DMATS-type aromatic prenyltransferase	**prot_ID_1122**	**AN10289**	IGD
AN10295.3	-1	742	conserved hypothetical protein		B-zip transcription factor	prot_ID_981	AN10295	
AN2346.3	-2	670	conserved hypothetical protein		Protein of unknown function	prot_ID_1323	AN2346	
n/a					Protein of unknown function	prot_ID_1498	AN12125	
AN2347.3	-3	2567	conserved hypothetical protein	CYP504E4P	Cytochrome P450; possibly a pseudogene	prot_ID_650	AN2347	FA, IGD
AN2348.3	-4	2821	conserved hypothetical protein		Deleted ORF	n/a	AN2348	
AN2349.3	-5	1443	hypothetical protein similar to ATP-binding cassette multidrug transport protein ATRC	atrC	ABC transporter of the P-glycoprotein cluster	n/a	AN2349	

AN10297 cluster

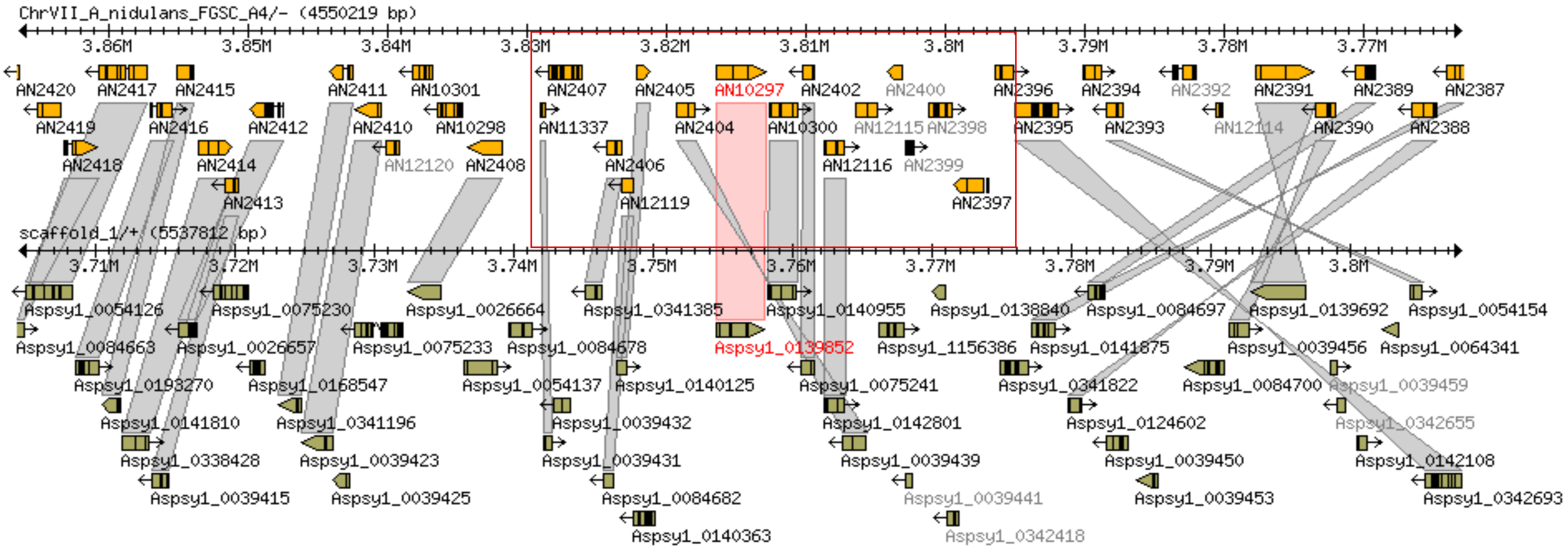

AN10297 cluster

Gene id (SMURF)*	Gene positions	Gene distance	Annotated_gene_function (SMURF)	AspGD gene name	AspGD manual annotation and notes	antiSMASH ID	Gene ID	
AN2392.3	13	2333	conserved hypothetical protein		bHLH transcription factor	n/a		
AN11336.3	12	1051	predicted protein		Deleted by the Broad	n/a		
AN2393.3	11	506	conserved hypothetical protein		Short chain oxidoreductase/dehydrogenase	n/a		
AN2394.3	10	1565	conserved hypothetical protein		Protein of unknown function	n/a		
AN2395.3	9	1167	conserved hypothetical protein		beta-glucuronidase	prot_ID_1215	AN2395	
AN2396.3	8	704	conserved hypothetical protein		Oxidoreductase	prot_ID_543	AN2396	FA
AN2397.3	7	144	conserved hypothetical protein		Protein of unknown function	prot_ID_1361	AN2397	
AN2398.3	6	1196	conserved hypothetical protein		O-methyltransferase	prot_ID_691	AN2398	
AN2399.3	5	281	conserved hypothetical protein		Protein of unknown function	prot_ID_23	AN2399	
AN2400.3	4	525	hypothetical protein		Protein of unknown function	prot_ID_557	AN2400	
AN2401.3	3	664	conserved hypothetical protein		Deleted ORF; AN2401 split into AN12115 and AN12116	n/a		
n/a					Protein of unknown function	prot_ID_1530	AN12115	
n/a					Transmembrane transporter	prot_ID_1543	AN12116	
AN2402.3	2	414	conserved hypothetical protein		Oxidoreductase	prot_ID_704	AN2402	
AN10300.3	1	403	conserved hypothetical protein		Zn(II)2Cys6 transcription factor	prot_ID_316	AN10300	
**AN10297.3**	0	0	NRPS-like enzyme		NAD-binding acyl CoA synthetase	**prot_ID_1121**	**AN10297**	
AN2404.3	-1	1650	conserved hypothetical protein		Protein of unknown function	prot_ID_849	AN2404	
AN2405.3	-2	1952	conserved hypothetical protein		Methyltransferase	prot_ID_171	AN2405	
n/a					Protein of unknown function	prot_ID_1486	AN12119	
AN2406.3	-3	1109	conserved hypothetical protein		N-methyltransferase	prot_ID_992	AN2406	
AN2407.3	-4	1905	conserved hypothetical protein		Domain(s) with predicted role in isoprenoid biosynthetic process	prot_ID_314	AN2407	
AN11337.3	-5	166	predicted protein		Protein of unknown function	prot_ID_861	AN11337	FA
AN2408.3	-6	2805	conserved hypothetical protein		Protein of unknown function	prot_ID_1132	AN2408	
AN10298.3	-7	539	conserved hypothetical protein		3-phosphoserine aminotransferase	prot_ID_453	AN10298	
AN10301.3	-8	428	conserved hypothetical protein		Domain(s) with predicted nucleotide binding activity	n/a	AN10301	

AN10396 cluster

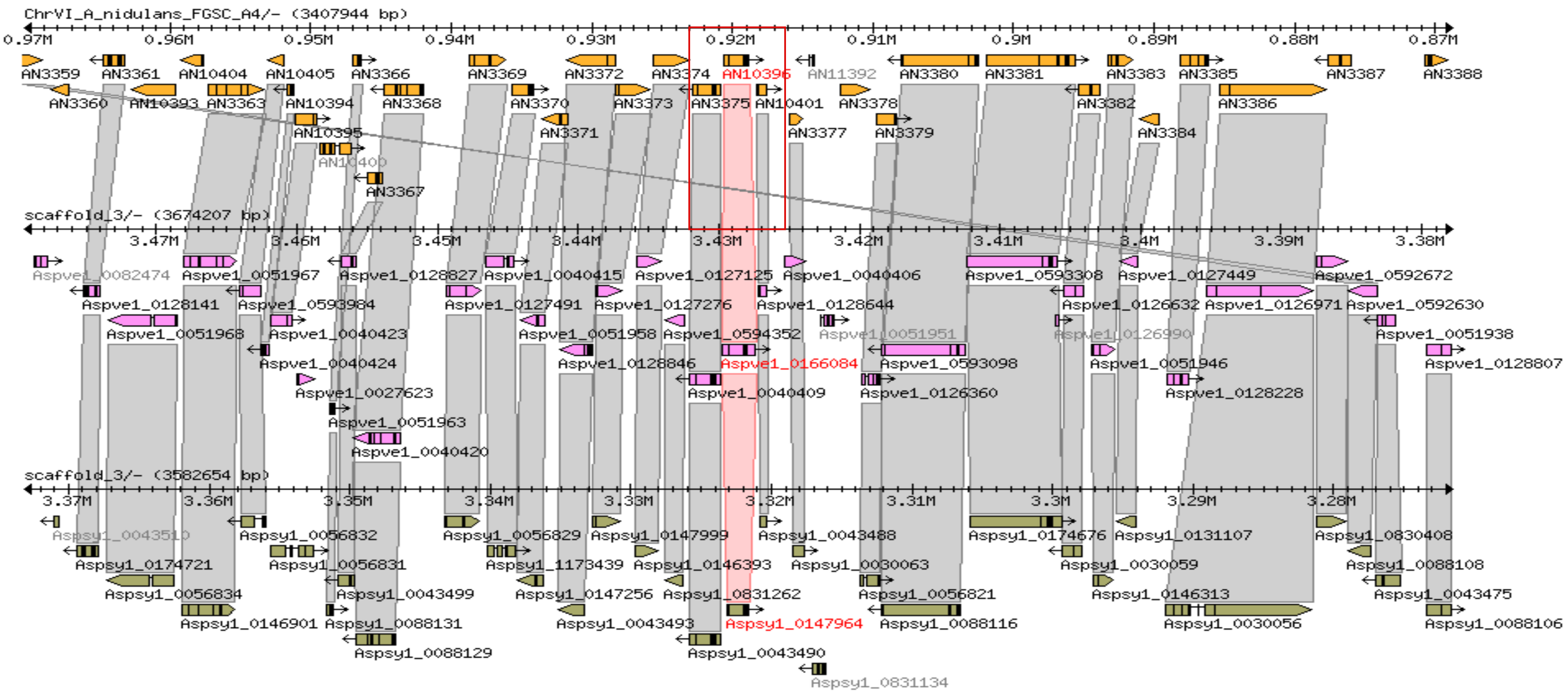

AN10396 cluster

Gene id (SMURF)*	Gene positions	Gene distance	Annotated_gene_function (SMURF)	AspGD gene name	AspGD manual annotation and notes	antiSMASH ID	Gene ID	
n/a					Zn(II)2Cys6 transcription factor	n/a	AN3369	
n/a					Protein of unknown function	n/a	AN3370	
n/a					Protein of unknown function	n/a	AN3371	
n/a				scaA	Campothecin resistance conferring protein rcaA	prot_ID_244	AN3372	
n/a					DNA-directed polymerase kappa	prot_ID_844	AN3373	
n/a					Protein of unknown function	prot_ID_352	AN3374	
n/a					DnaJ domain protein	prot_ID_957	AN3375	ECS
n/a					Farnesyl-diphosphate farnesyltransferase	prot_ID_418	**AN10396**	
n/a					Protein of unknown function	prot_ID_1029	AN10401	ECS, IGD
n/a					Aspartic-type endopeptidase	prot_ID_1064	AN3377	
n/a					Protein of unknown function	prot_ID_454	AN11392	
n/a					homocysteine S-methyltransferase	prot_ID_555	AN3378	
n/a					Protein of unknown function	prot_ID_69	AN3379	

AN10430 cluster

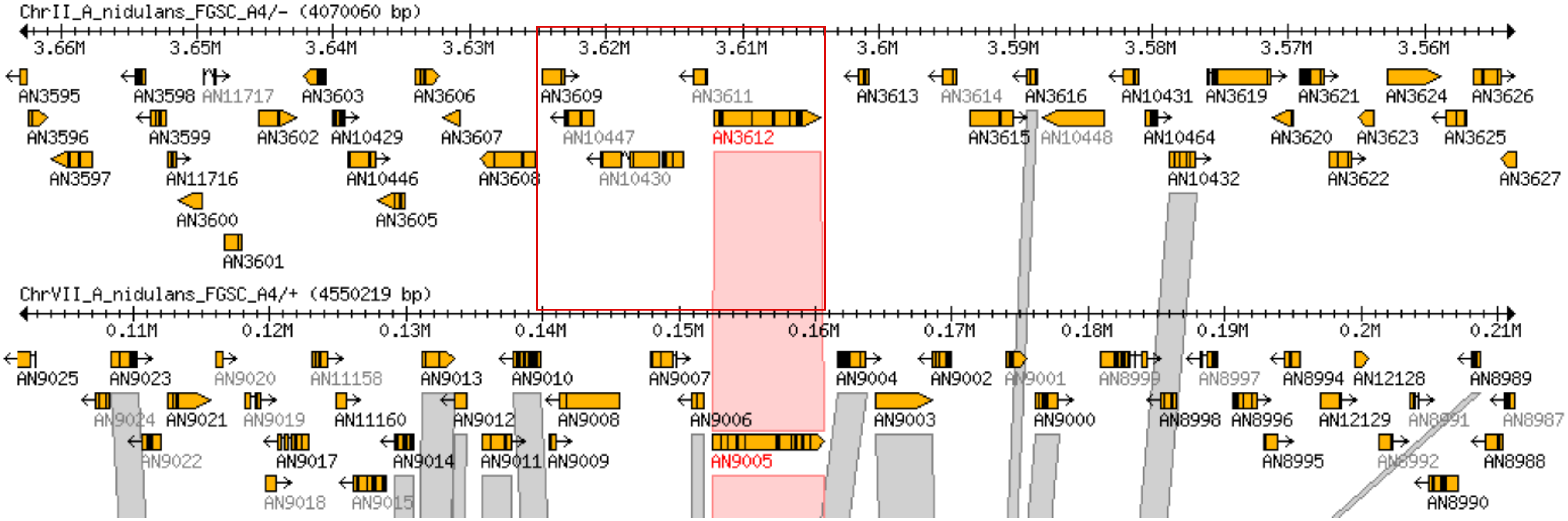

AN10430 cluster

Gene id (SMURF)*	Gene positions	Gene distance	Annotated_gene_function (SMURF)	AspGD gene name	AspGD manual annotation and notes	antiSMASH ID	Gene ID	
n/a					Phosphoserine phosphatase	prot_ID_665	AN10429	
n/a					Phosphoprotein phosphatase	prot_ID_59	AN10446	
n/a					Oxidoreductase	prot_ID_906	AN3605	
n/a					Protein of unknown function	prot_ID_296	AN3606	
n/a				lreB	zinc-finger transcription factor	prot_ID_1042	AN3607	
AN3608.3	3	506	hypothetical protein similar to ABC transporter protein AtrC	atrC	ABC transporter	prot_ID_423	AN3608	
AN3609.3	2	25	conserved hypothetical protein	CYP681A1	Cytochrome P450	prot_ID_1168	AN3609	FA
AN10447.3	1	615	conserved hypothetical protein		Acyl transferase	prot_ID_789	AN10447	
**AN10430.3**	0	0	polyketide synthase		Polyketide synthase	**prot_ID_886**	**AN10430**	
AN3611.3	-1	773	conserved hypothetical protein		Protein of unknown function	prot_ID_769	AN3611	
AN3612.3	-2	425	polyketide synthase		Polyketide synthase	prot_ID_164	AN3612	FA
n/a				xlnA	Endo-1,4-beta-xylanase	prot_ID_903	AN3613	
n/a					Protein of unknown function	prot_ID_292	AN3614	
n/a					Predicted ATP binding, nucleoside-triphosphatase	prot_ID_1039	AN3615	
n/a					Oxidoreductase	prot_ID_421	AN3616	
n/a				tprA	Tetratricopeptide repeat (TPR)-containing protein	prot_ID_186	AN10448	

AN10486 cluster

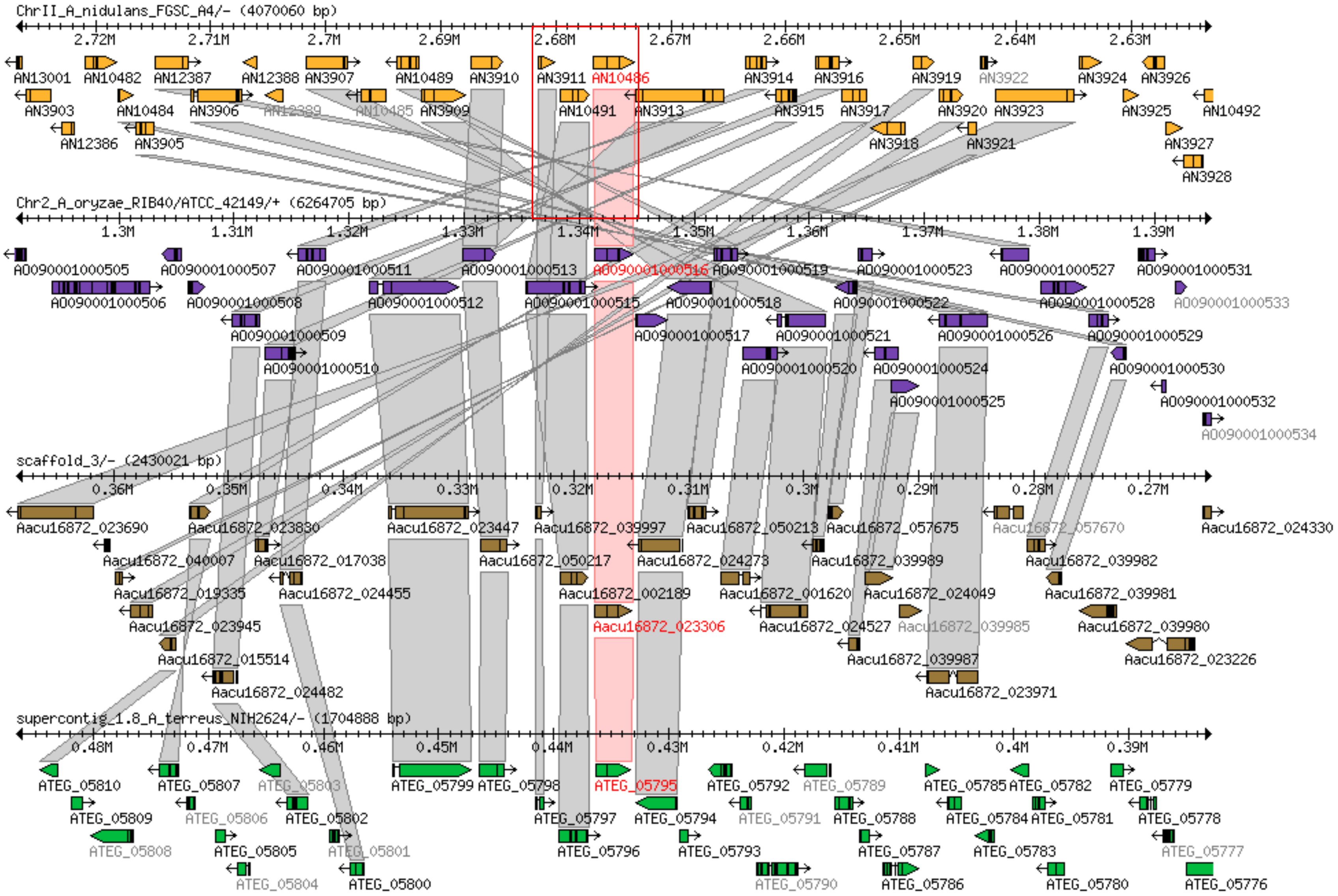

AN10486 cluster

Gene id (SMURF)*	Gene positions	Gene distance	Annotated_gene_function (SMURF)	AspGD gene name	AspGD manual annotation and notes	antiSMASH ID	Gene ID	
n/a					Serine/threonine protein kinase	prot_ID_1180	AN10485	
n/a				FKBP5	FKBP-type peptidyl-prolyl cis-trans isomerase	prot_ID_92	AN10489	
n/a					Deoxycytidyl transferase	prot_ID_451	AN3909	
n/a					Sister chromatid separation protein	prot_ID_670	AN3910	
AN3911.3	2	769	hypothetical protein similar to SndA		Zn(II)2Cys6 transcription factor	prot_ID_67	AN3911	ECS
AN10491.3	1	532	conserved hypothetical protein		Zn(II)2Cys6 transcription factor	prot_ID_1053	AN10491	
**AN10486.3**	0	0	NRPS-like enzyme		NRPS-like enzyme	**prot_ID_566**	**AN10486**	ECS
AN3913.3	-1	673	hypothetical protein similar to adenylate cyclase	cyaA	Adenylate cyclase	prot_ID_192	AN3913	
AN3914.3	-2	2816	conserved hypothetical protein	crhA	Transglycosidase	prot_ID_937	AN3914	
AN10490.3	-3	35	predicted protein		Deleted by the Broad			
AN3915.3	-4	648	conserved hypothetical protein		Transmembrane transporter	prot_ID_321	AN3915	
AN3916.3	-5	2058	conserved hypothetical protein		Gycerol kinase	prot_ID_1070	AN3916	
AN3917.3	-6	195	conserved hypothetical protein	CYP539D1	Putative cytochrome P450	prot_ID_448	AN3917	

AN11065 cluster

No manual prediction made, functional annotations unrelated to secondary metabolism

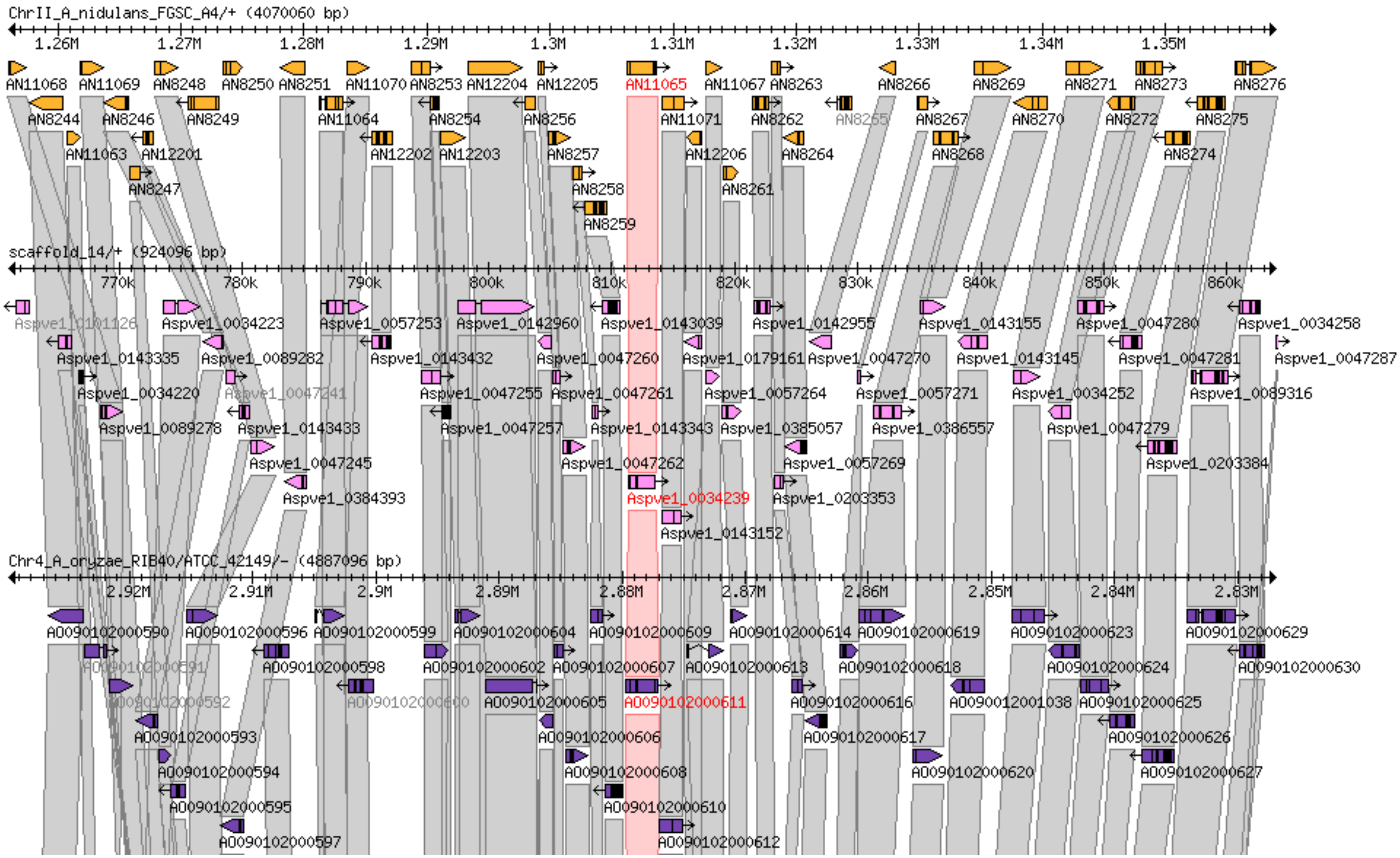

AN11065 cluster

Gene id (SMURF)*	Gene positions	Gene distance	Annotated_gene_function (SMURF)	AspGD gene name	AspGD manual annotation and notes	antiSMASH ID	Gene ID
n/a					Protein of unknown function	prot_ID_1372	AN12204
n/a					50S ribosomal protein L27	prot_ID_335	AN8256
n/a					Protein of unknown function	prot_ID_1416	AN12205
n/a					Scramblase family protein	prot_ID_1080	AN8257
n/a					Ubiquitin-conjugating enzyme	prot_ID_464	AN8258
n/a					Endodeoxyribonuclease	prot_ID_1209	AN8259
n/a					Oxidosqualene-lanosterol cyclase	**prot_ID_389**	**AN11065**
n/a					SGT1 and CS domain protein	prot_ID_256	AN11071
n/a					Protein of unknown function	prot_ID_1439	AN12206
n/a				hstA	histone deacetylase	prot_ID_521	AN11067
n/a				phoA	Cyclin-dependent protein kinase	prot_ID_806	AN8261
n/a				gprH	Secretin-like G-protein coupled receptor	prot_ID_204	AN8262
n/a					Protein of unknown function	prot_ID_947	AN8263

AN11080 cluster

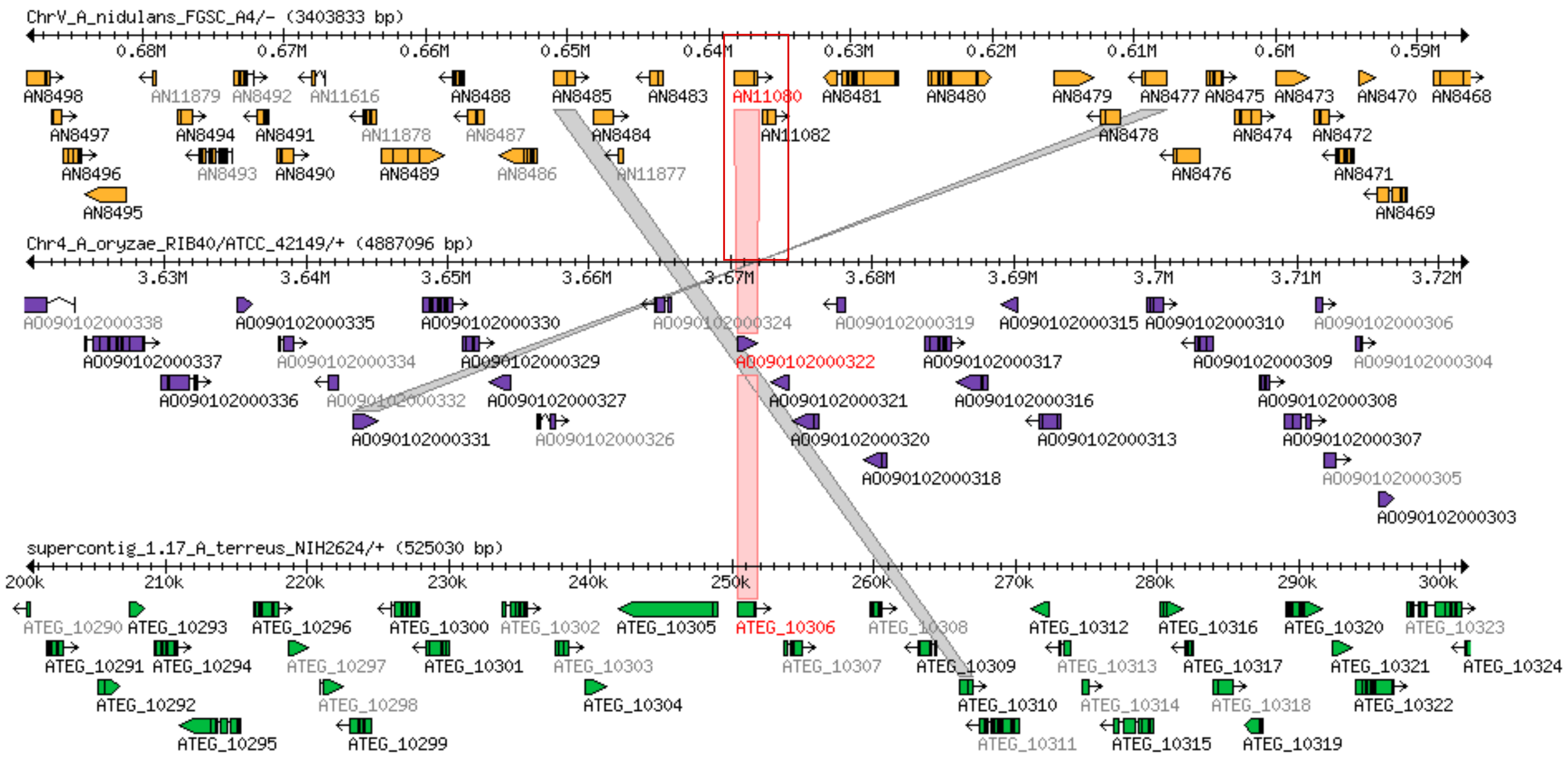

AN11080 cluster

Gene id (SMURF)*	Gene positions	Gene distance	Annotated_gene_function (SMURF)	AspGD gene name	AspGD manual annotation and notes	antiSMASH ID	Gene ID	
n/a					Glucan 1,3-beta-glucosidase			
n/a					Chitinase	prot_ID_121	AN8481	
n/a					Protein of unknown function	prot_ID_877	AN11082	IGD, FA
n/a					DMATS-type aromatic prenyltransferase	prot_ID_782	AN11080	ECS
n/a					Protein of unknown function	prot_ID_224	AN8483	
n/a					Protein of unknown function	prot_ID_1048	AN11877	
n/a					GPI-anchored protein	prot_ID_797	AN8484	

AN11191 Cluster

Adjacent clusters, see AN11194 cluster below

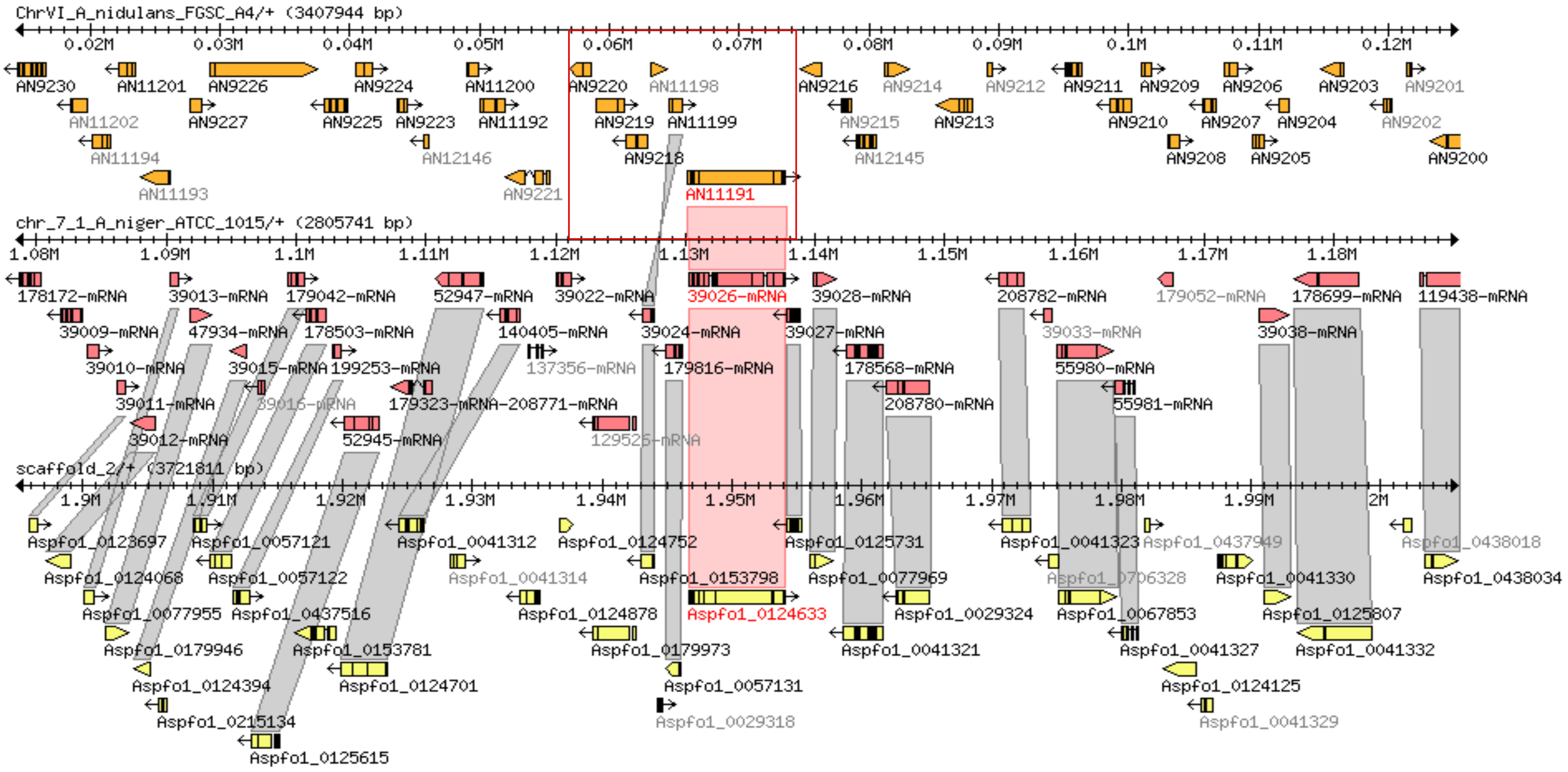

AN11191 Cluster

Gene id (SMURF)*	Gene positions	Gene distance	Annotated_gene_function (SMURF)	AspGD gene name	AspGD manual annotation and notes	antiSMASH ID	Gene ID	
n/a					extracellular guanyl-specific ribonuclease	n/a		
n/a					Protein of unknown function	n/a		
n/a					Protein of unknown function	n/a		
AN9210.3	8	797	conserved hypothetical protein	CYP662A1	Cytochrome P450	n/a		
AN9211.3	7	3682	conserved hypothetical protein		Protein of unknown function	n/a		
AN11645.3	6	1849	predicted protein		Deleted by the Broad	n/a		
AN9212.3	5	1255	hypothetical protein		Protein of unknown function	prot_ID_1018	AN9212	
AN9213.3	4	987	conserved hypothetical protein		Hydrolase	prot_ID_516	AN9213	
AN9214.3	3	2570	conserved hypothetical protein	CYP550B2	Cytochrome P450	prot_ID_27	AN9214	
n/a					Protein of unknown function	prot_ID_1153	AN12145	
AN9215.3	2	1507	hypothetical protein		Protein of unknown function	prot_ID_622	AN9215	
AN9216.3	1	992	conserved hypothetical protein		Protein of unknown function	prot_ID_135	AN9216	
**AN11191.3**	0	0	polyketide synthase		Polyketide synthase	**prot_ID_589**	**AN11191**	ECS, EP
AN11199.3	-1	408	conserved hypothetical protein		Dihydrofolate reductase	prot_ID_1032	AN11199	
AN11198.3	-2	104	conserved hypothetical protein		Oxidoreductase	prot_ID_425	AN11198	
AN9218.3	-3	187	hypothetical protein	CYP540D1	Cytochrome P450	prot_ID_234	AN9218	
AN9219.3	-4	313	conserved hypothetical protein		Transmembrane transporter	prot_ID_835	AN9219	
AN9220.3	-5	673	conserved hypothetical protein		Protein of unknown function	prot_ID_1014	AN9220	EP
AN9221.3	-6	1683	conserved hypothetical protein		Zn(II)2Cys6 transcription factor	prot_ID_511	AN9221	
AN11192.3	-7	48	conserved hypothetical protein	CYP664A1	Cytochrome P450	prot_ID_108	AN11192	
n/a			conserved hypothetical protein		Protein of unknown function	prot_ID_329	AN11200	
n/a					Protein of unknown function	prot_ID_1129	AN12146	
n/a					Demethylsterigmatocystin 6-O-methyltransferase with a predicted role in sterigmatocystin/aflatoxin biosynthesis	n/a	AN9223	
n/a					Domain(s) with predicted monooxygenase activity and role in oxidation-reduction process	n/a	AN9224	
n/a					Putative cytochrome P450	n/a	AN9225	
n/a					Putative nonribosomal peptide	n/a	AN9226	

	synthase (NRPS)		
n/a	Ortholog(s) have dioxygenase activity and role in secondary metabolite biosynthetic process	n/a	AN9227
n/a	Domain(s) with predicted oxygen transporter activity and role in transport	n/a	AN11193
n/a	Domain(s) with predicted oxidoreductase activity and role in carotenoid biosynthetic process, chlorophyll biosynthetic process, oxidation-reduction process, photosynthesis	n/a	AN11201
n/a	Putative dimethyl-allyl-tryptophan synthase (dmats) type aromatic prenyltransferase	n/a	AN11194
n/a	Putative dimethyl-allyl-tryptophan synthase (DMATS) type aromatic prenyltransferase	n/a	AN11202
	Domain(s) with predicted monooxygenase activity and role in oxidation-reduction process	n/a	AN9230

AN11194 cluster

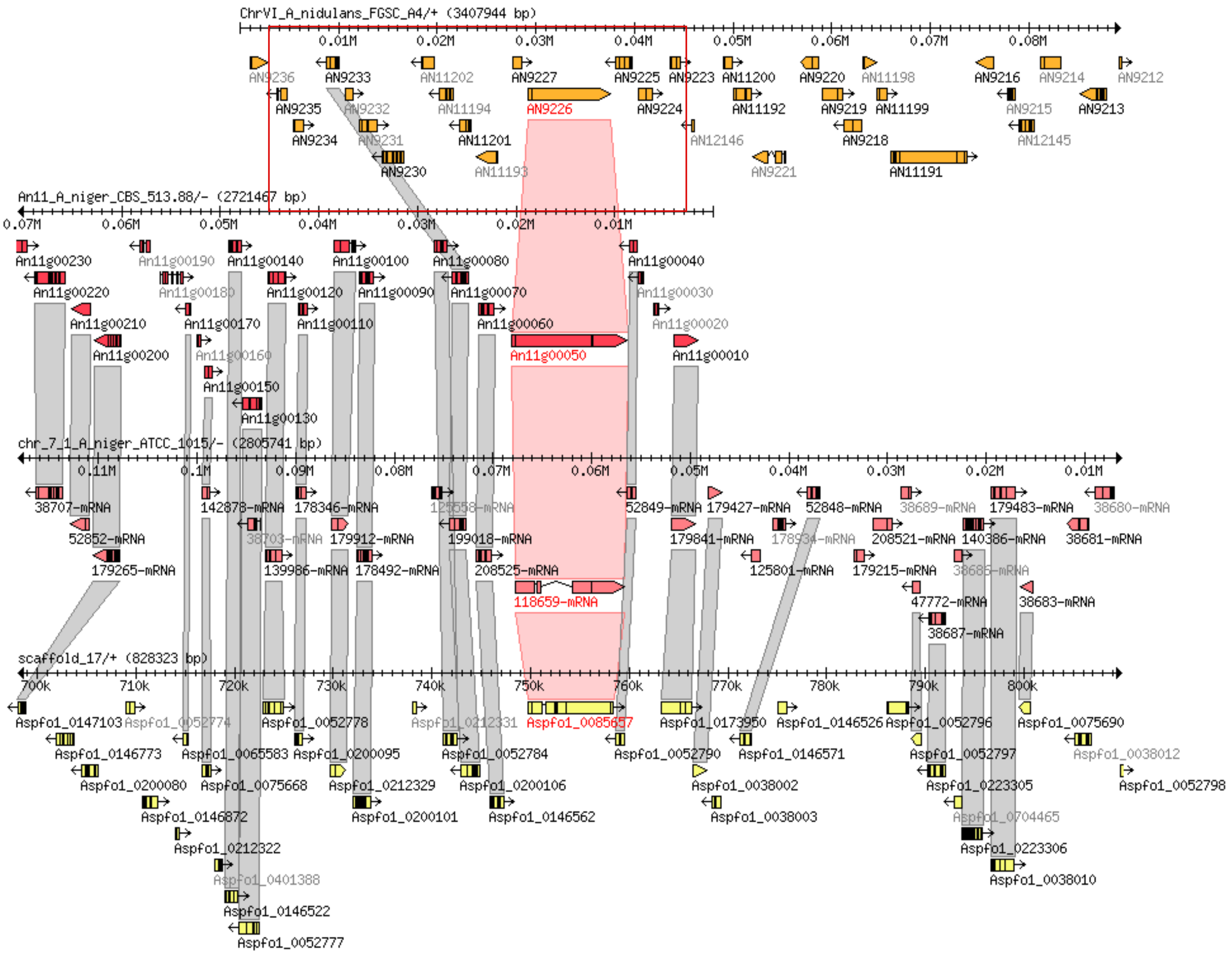

AN11194 cluster

Gene id (SMURF)*	Gene positions	Gene distance	Annotated_gene_function (SMURF)	AspGD gene name	AspGD manual annotation and notes	antiSMASH ID	Gene ID	
n/a					Protein of unknown function	prot_ID_1014	AN9220	
n/a					Zn(II)2Cys6 transcription factor	prot_ID_511	AN9221	
n/a				CYP664A1	Cytochrome P450	prot_ID_108	AN11192	
n/a					Protein of unknown function	prot_ID_329	AN11200	
n/a					Protein of unknown function	prot_ID_1129	AN12146	
AN9223.3	7	1736	conserved hypothetical protein		Demethylsterigmatocystin 6-O-methyltransferase	prot_ID_620	AN9223	EP
AN9224.3	6	633	conserved hypothetical protein		Monooxygenase	prot_ID_133	AN9224	
AN9225.3	5	563	conserved hypothetical protein	CYP679A1	Cytochrome P450	prot_ID_726	AN9225	
**AN9226.3**	4	1045	nonribosomal peptide synthase		Nonribosomal peptide synthase	prot_ID_231	AN9226	
AN9227.3	3	616	conserved hypothetical protein		Dioxygenase	prot_ID_832	AN9227	
AN11193.3	2	429	conserved hypothetical protein		Domain(s) with predicted oxygen transporter activity	prot_ID_698	AN11193	
AN11201.3	1	611	conserved hypothetical protein		Oxidoreductase	prot_ID_933	AN11201	
**AN11194.3**	0	0	DMATS type aromatic prenyltransferase		DMATS-type aromatic prenyltransferase	**prot_ID_210**	**AN11194**	
AN11202.3	-1	363	DMATS type aromatic prenyltransferase		DMATS-type aromatic prenyltransferase	prot_ID_438	AN11202	
AN9230.3	-2	1866	conserved hypothetical protein		Monooxygenase	prot_ID_18	AN9230	
AN9231.3	-3	208	conserved hypothetical protein		UDP-N-acetylmuramate dehydrogenase	prot_ID_617	AN9231	
AN9232.3	-4	666	conserved hypothetical protein		Oxidoreductase	prot_ID_129	AN9232	
AN9233.3	-5	406	conserved hypothetical protein		O-methyltransferase	prot_ID_724	AN9233	
AN9234.3	-6	2320	conserved hypothetical protein		Oxidoreductase	prot_ID_227	AN9234	
AN9235.3	-7	638	conserved hypothetical protein		Isochorismatase family hydrolase	prot_ID_830	AN9235	EP
AN9236.3	-8	895	conserved hypothetical protein		Zn(II)2Cys6 transcription factor	prot_ID_336	AN9236	
n/a					Protein of unknown function	n/a	AN12146	

AN12331 cluster

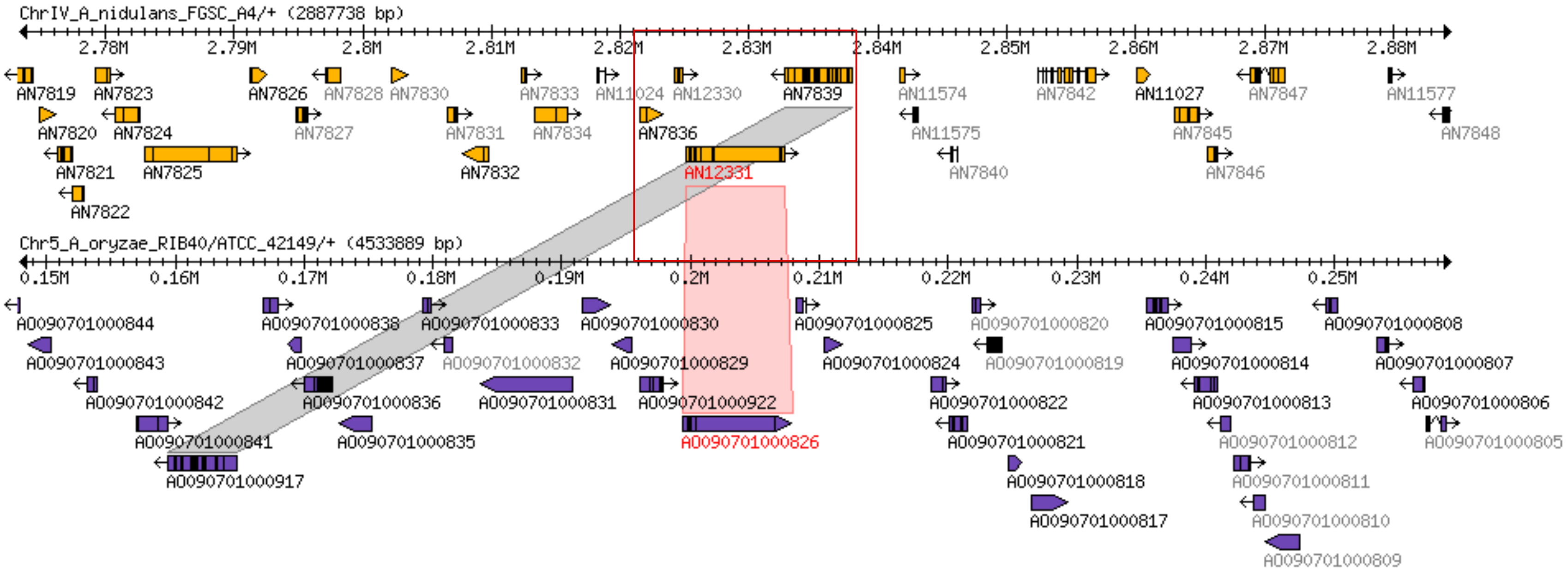

AN12331 cluster

Gene id (SMURF)*	Gene positions	Gene distance	Annotated_gene_function (SMURF)	AspGD gene name	AspGD manual annotation and notes	antiSMASH ID	Gene ID	
n/a					Protein of unknown function	prot_ID_35	AN7831	
n/a					choline dehydrogenase	prot_ID_544	AN7832	
n/a					Protein of unknown function	prot_ID_122	AN7833	
n/a					Protein of unknown function	prot_ID_631	AN7834	
n/a					Transmembrane transporter	prot_ID_337	AN11024	
n/a					Protein of unknown function	prot_ID_720	AN7836	FA
n/a					Protein of unknown function	prot_ID_996	AN12330	
n/a					PKS-like enzyme	prot_ID_957	**AN12331**	
n/a					Transmembrane transporter	prot_ID_392	AN7839	ECS
n/a					Protein of unknown function	prot_ID_593	AN11574	
n/a					Protein of unknown function	prot_ID_175	AN11575	
n/a					Protein of unknown function	prot_ID_541	AN7840	
n/a					Protein of unknown function	prot_ID_630	AN7842	

AN1242 cluster

Highly conserved across numerous species

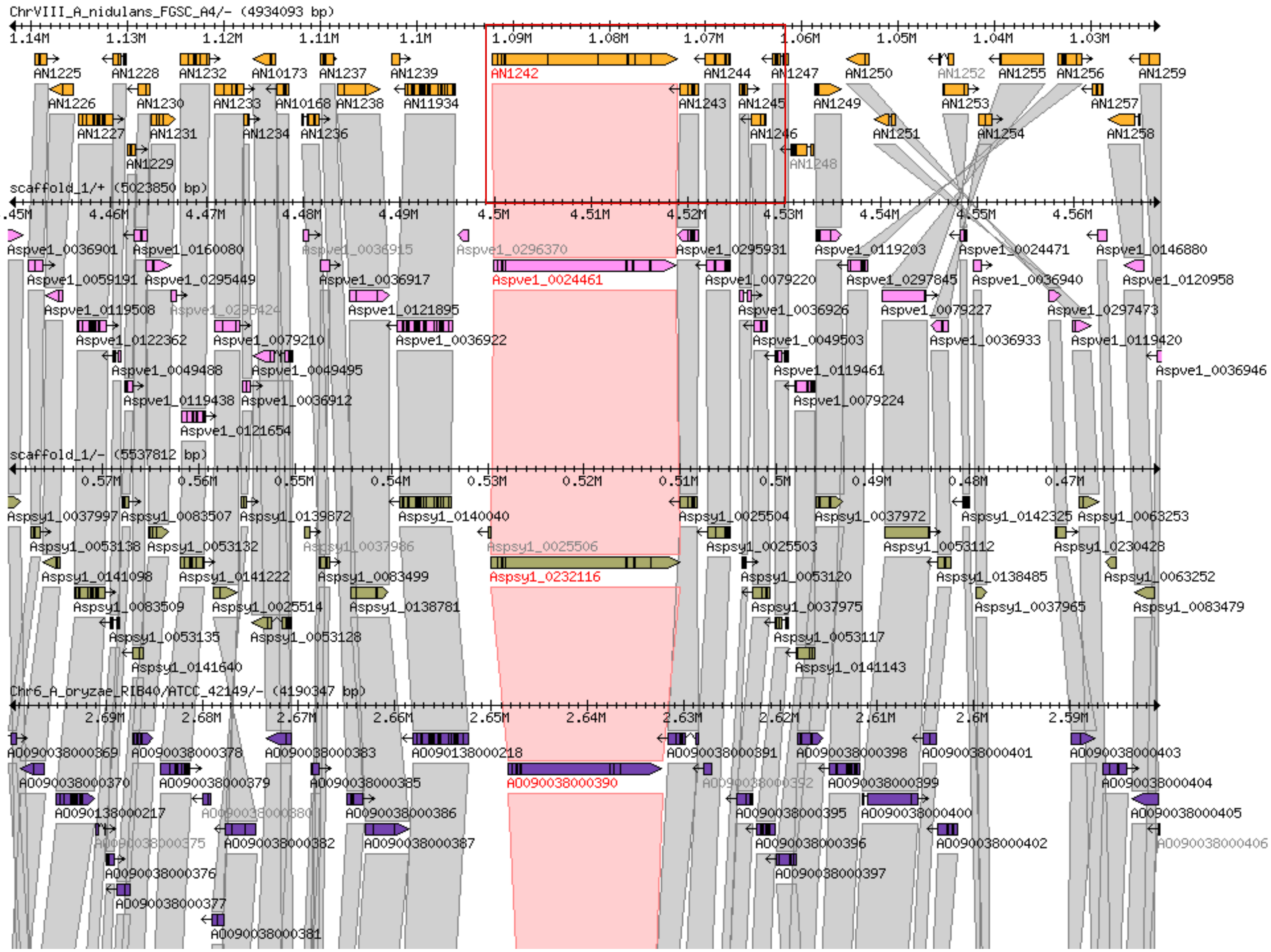

AN1242 cluster

Gene id (SMURF)*	Gene positions	Gene distance	Annotated_gene_function (SMURF)	AspGD gene name	AspGD manual annotation and notes	antiSMASH ID	Gene ID	
n/a					Protein of unknown function	prot_ID_336	AN1236	
n/a				uvsC	DNA damage response protein	prot_ID_791	AN1237	
n/a				nup120	nuclear pore complex protein	prot_ID_1268	AN1238	
n/a					Protein of unknown function	prot_ID_43	AN1239	
n/a					Transmembrane transporter	prot_ID_1703	AN11934	
**AN1242.3**	0	0	nonribosomal peptide synthase		Nonribosomal peptide synthase	**prot_ID_260**	**AN1242**	ECS, IGD
AN1243.3	-1	259	conserved hypothetical protein		Transmembrane transporter	prot_ID_711	AN1243	
n/a					Protein of unknown function	prot_ID_1182	AN1244	
n/a					Protein of unknown function	prot_ID_1637	AN1245	
n/a				pgkA	phosphoglycerate kinase	prot_ID_417	AN1246	
n/a					integral membrane protein	prot_ID_874	AN1247	ECS
n/a					Protein of unknown function	prot_ID_1350	AN1248	
n/a					Formin binding protein	prot_ID_121	AN1249	
n/a					Protein of unknown function	prot_ID_1096	AN1250	

AN1594 (PbcR) cluster

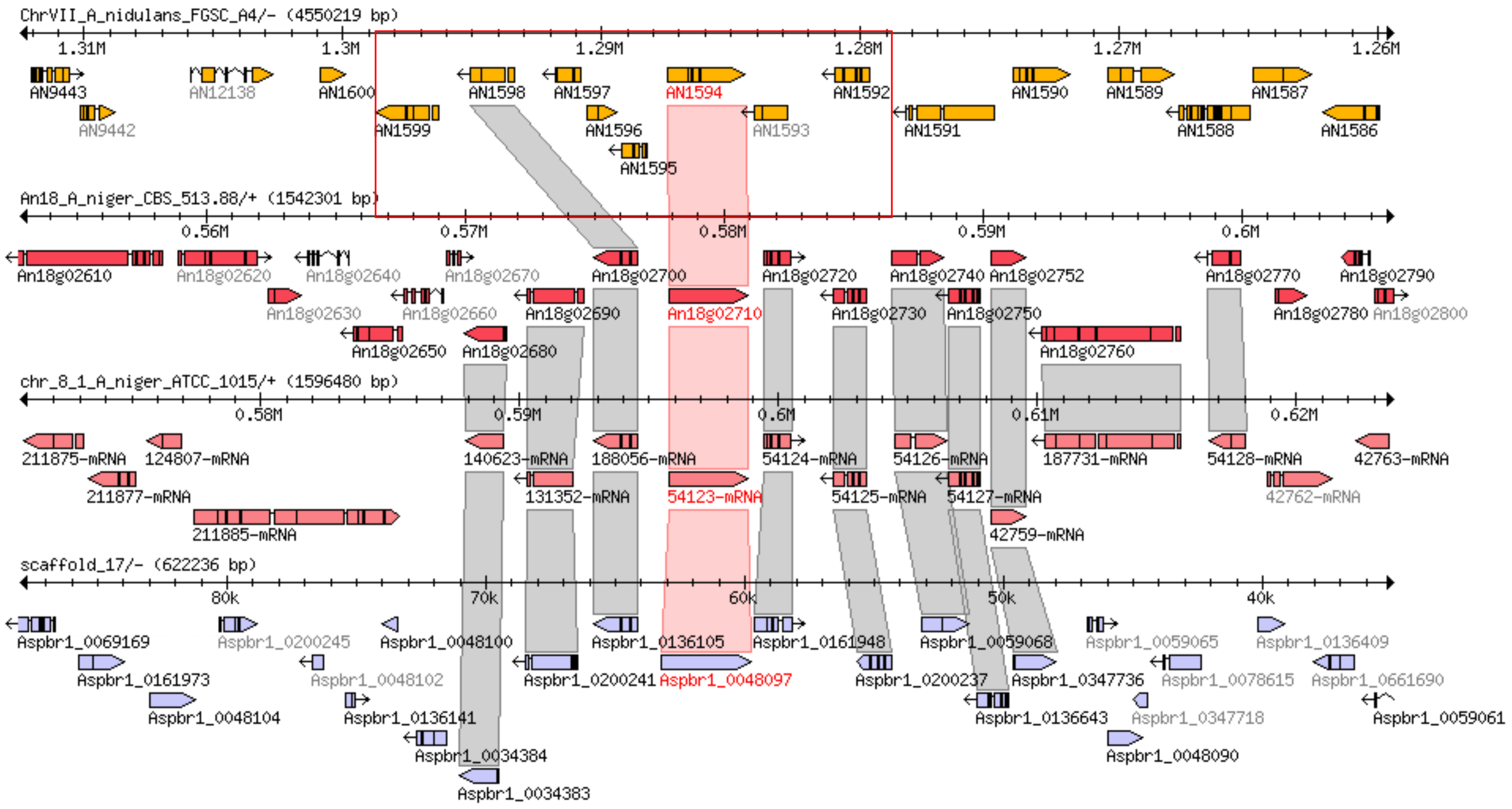

AN1594 (PbcR) cluster

Gene id (SMURF)*	Gene positions	Gene distance	Annotated_gene_function (SMURF)	AspGD gene name	AspGD manual annotation and notes	antiSMASH ID	Gene ID	
n/a					GGPP-synthase	n/a	AN1592	EP
n/a					3-hydroxy-3-methylglutaryl-coenzyme A (HMG-CoA) reductase isozyme	n/a	AN1593	
n/a					Entkaurene synthase	n/a	**AN1594**	
n/a					Elongation factor 1-gamma	n/a	AN1595	
n/a					Short-chain dehydrogenase	n/a	AN1596	
n/a					Protein of unknown function	n/a	AN1597	
n/a				CYP503B1	Cytochrome P450	n/a	AN1598	
n/a				pbcR	Zn(II)(2)Cys(6)-type transcription factor	n/a	AN1599	EP

AN1680 cluster

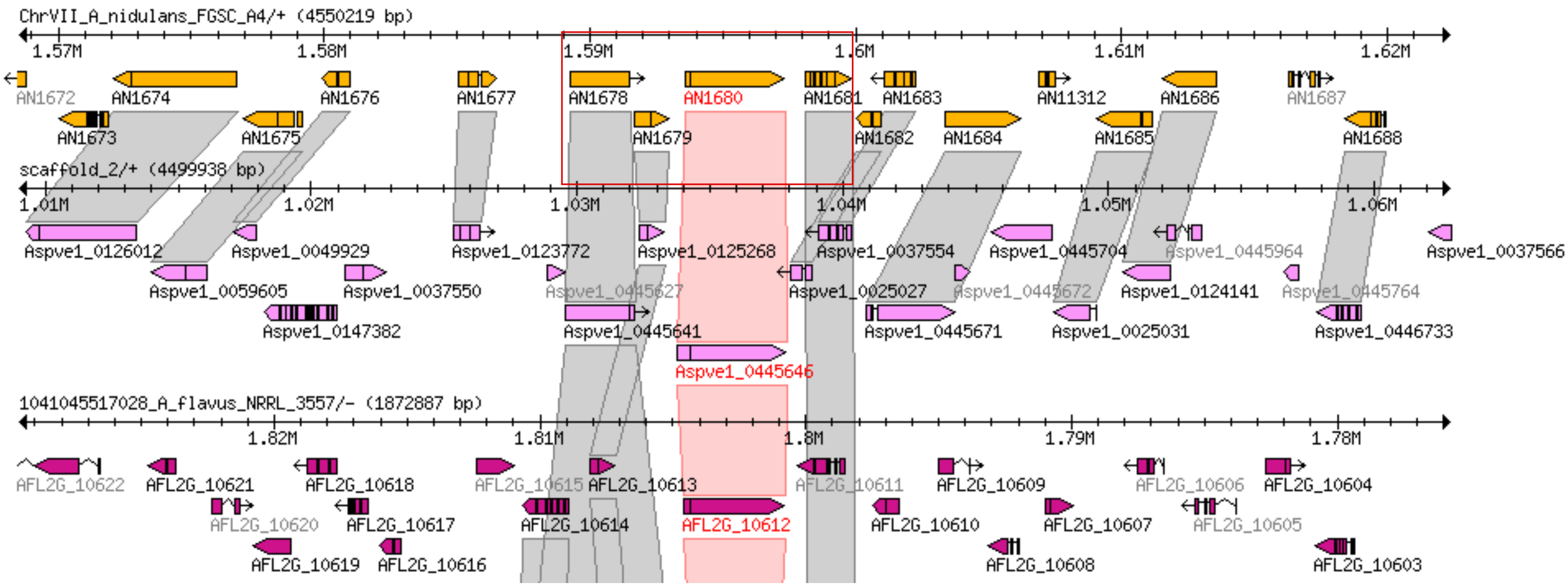

AN1680 Cluster

Gene id (SMURF)*	Gene positions	Gene distance	Annotated_gene_function (SMURF)	AspGD gene name	AspGD manual annotation and notes	antiSMASH ID	Gene ID	
n/a					Domain(s) with predicted DNA binding activity and intracellular localization	prot_ID_478	AN1674	
n/a					Lysophospholipase (phoshopholipase B)	prot_ID_1290	AN1675	
n/a					Oxidoreductase	prot_ID_619	AN1676	
AN1677.3	3	2761	conserved hypothetical protein		Short-chain dehydrogenase	prot_ID_1433	AN1677	
AN1678.3	2	398	conserved hypothetical protein		Transcription factor	prot_ID_764	AN1678	ECS
AN1679.3	1	609	conserved hypothetical protein		Protein of unknown function	prot_ID_95	AN1679	
**AN1680.3**	0	0	NRPS-like enzyme		Nonribosomal peptide synthase (NRPS)-like enzyme	**prot_ID_331**	**AN1680**	
AN1681.3	-1	735	conserved hypothetical protein		Transmembrane transporter	prot_ID_1145	AN1681	ECS
AN1682.3	-2	151	hypothetical protein		Protein of unknown function	prot_ID_476	AN1682	
AN1683.3	-3	192	conserved hypothetical protein		oligosaccharyltransferase delta subunit	prot_ID_1287	AN1683	
AN1684.3	-4	840	conserved hypothetical protein		Protein of unknown function	prot_ID_618	AN1684	
AN11312.3	-5	853	conserved hypothetical protein		Protein of unknown function	prot_ID_964	AN11312	
AN1685.3	-6	1871	3-phytase B precursor		phytase	prot_ID_1432	AN1685	
AN1686.3	-7	903	conserved hypothetical protein		Transcription factor	prot_ID_762	AN1686	
AN1687.3	-8	2110	hypothetical protein		Oxidoreductase	prot_ID_93	AN1687	
AN1688.3	-9	931	conserved hypothetical protein		Protein of unknown function	n/a	AN1688	
AN1689.3	-10	3063	hypothetical protein		Oxidoreductase	n/a	AN1689	
AN1690.3	-11	589	hypothetical protein		Protein of unknown function	n/a	AN1690	
AN1691.3	-12	955	conserved hypothetical protein		Transmembrane transporter	n/a	AN1691	

AN1784 cluster

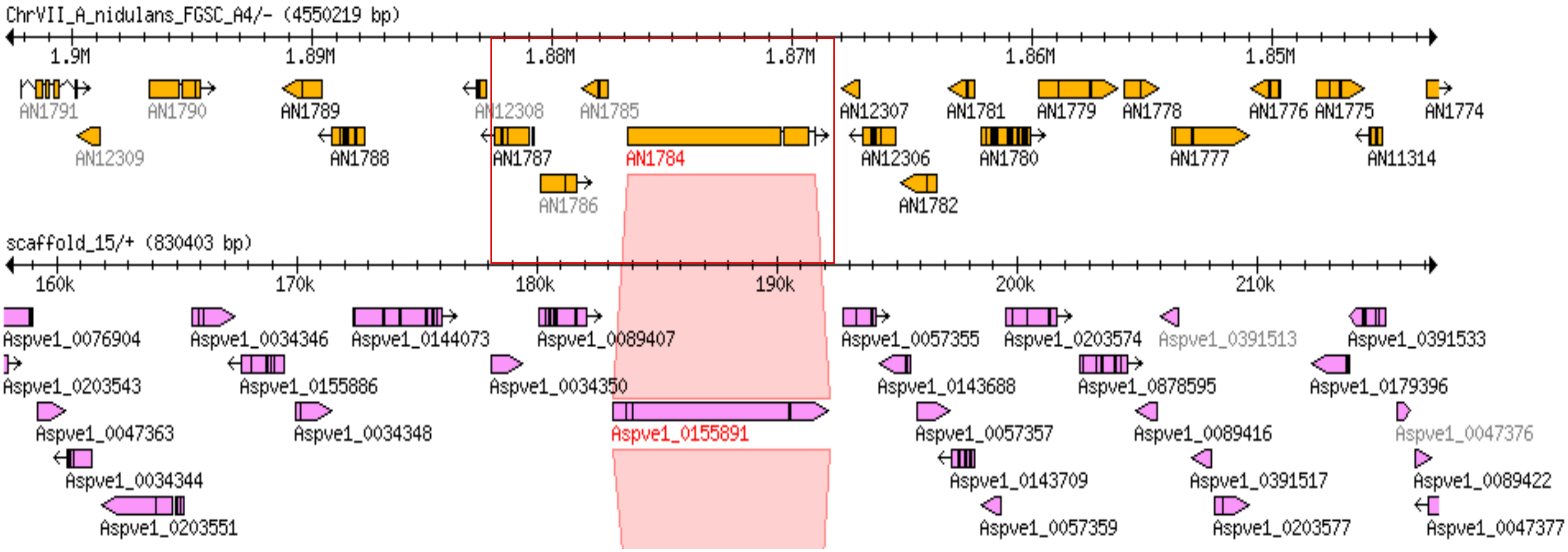

AN1784 cluster

Gene id (SMURF)*	Gene positions	Gene distance	Annotated_gene_function (SMURF)	AspGD gene name	AspGD manual annotation and notes	antiSMASH ID	Gene ID	
n/a					U1 snRNP splicing complex subunit	prot_ID_856	AN1776	
n/a					PHD finger domain protein	prot_ID_180	AN1777	
AN1778.3	6	242	conserved hypothetical protein	pantoB	Ketopentoate hydroxymethyl transferase	prot_ID_1000	AN1778	
AN1779.3	5	399	conserved hypothetical protein		Protein kinase	prot_ID_326	AN1779	
AN1780.3	4	520	conserved hypothetical protein		Aminopeptidase	prot_ID_565	AN1780	
AN1781.3	3	569	conserved hypothetical protein		Oxidoreductase	prot_ID_1381	AN1781	
AN1782.3	2	653	conserved hypothetical protein		Hydrolase	prot_ID_713	AN1782	
n/a					Oxidoreductase	prot_ID_1537	AN12306	
AN1783.3	1	690	conserved hypothetical protein		Protein of unknown function	prot_ID_1512	AN12307	
**AN1784.3**	0	0	polyketide synthase		Polyketide synthase	**prot_ID_855**	**AN1784**	EP
AN1785.3	-1	793	conserved hypothetical protein		Protein of unknown function	prot_ID_178	AN1785	
AN1786.3	-2	127	conserved hypothetical protein		Monooxygenase	prot_ID_998	AN1786	
AN1787.3	-3	272	conserved hypothetical protein		UDP-N-acetylmuramate dehydrogenase	prot_ID_323	AN1787	EP
n/a					Protein of unknown function	prot_ID_1548	AN12308	
n/a					Metal ion transmembrane transporter	prot_ID_1139	AN1788	
n/a					Protein serine/threonine kinase	prot_ID_469	AN1789	
n/a					Protein of unknown function	prot_ID_711	AN1790	

AN1793 cluster

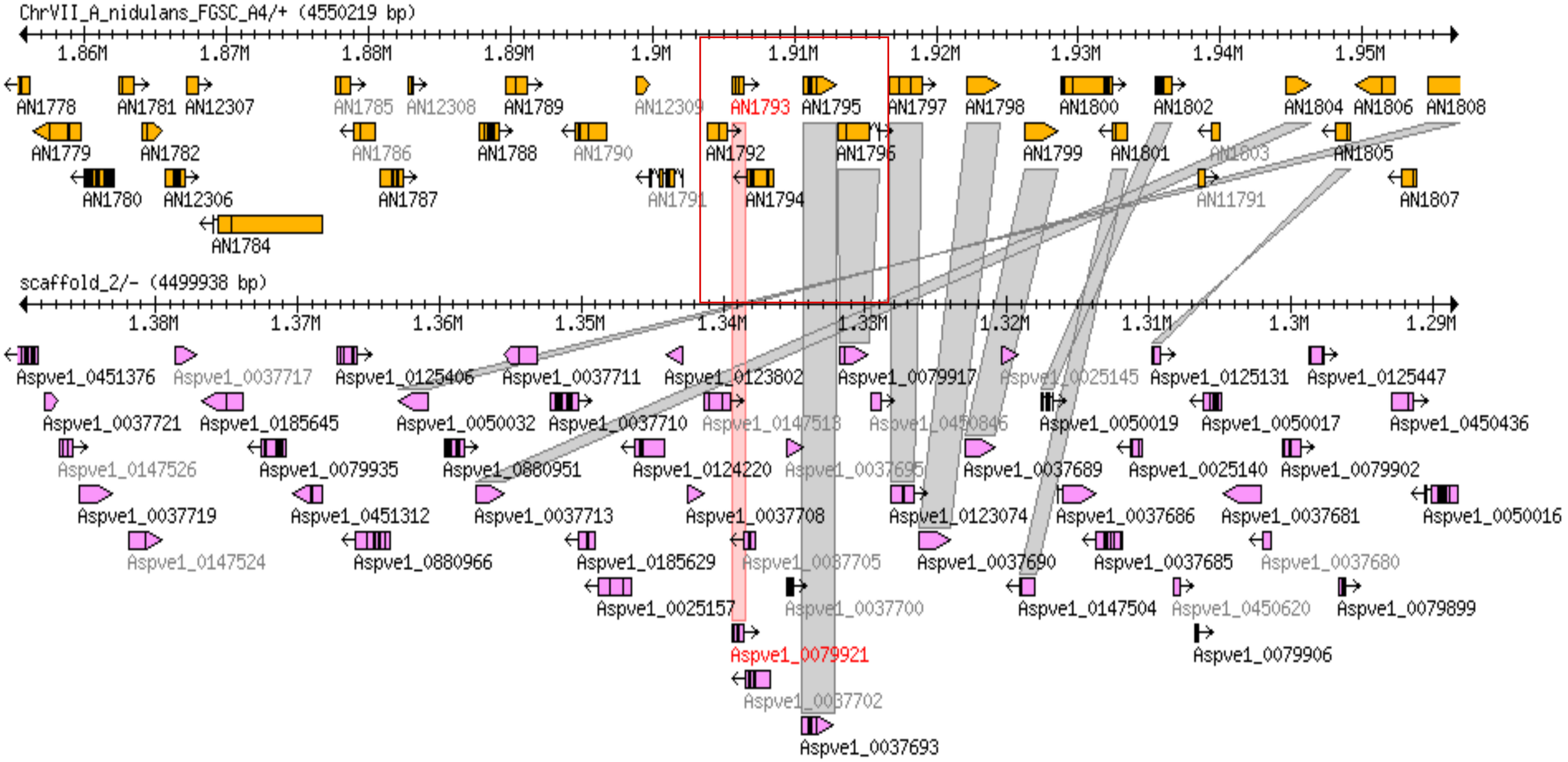

AN1793 cluster

Gene id (SMURF)*	Gene positions	Gene distance	Annotated_gene_function (SMURF)	AspGD gene name	AspGD manual annotation and notes	antiSMASH ID	Gene ID	
n/a					Protein of unknown function	prot_ID_711	AN1790	
n/a					Protein of unknown function	prot_ID_1520	AN12309	
n/a					Protein of unknown function	prot_ID_38	AN1791	
n/a					Hydrolase	prot_ID_853	AN1792	FA
n/a					Terpene synthase	prot_ID_176	**AN1793**	
n/a				CYP682D1	Cytochrome P450	prot_ID_997	AN1794	
n/a					Transmembrane transporter	prot_ID_321	AN1795	
n/a					DNA repair protein	prot_ID_1137	AN1796	ECS
n/a					Sugar transporter	n/a	AN1797	
n/a					Sterol esterase	n/a	AN1798	
n/a					Triglyceride lipase	n/a	AN1799	
n/a					Transmembrane histidine kinase	n/a	AN1800	
n/a					Protein of unknown function	n/a	AN1801	
n/a					Small nuclear ribonucleoprotein	n/a	AN1802	

AN2064 cluster

Possible false positive, functional annotations don't make sense

No manual prediction made

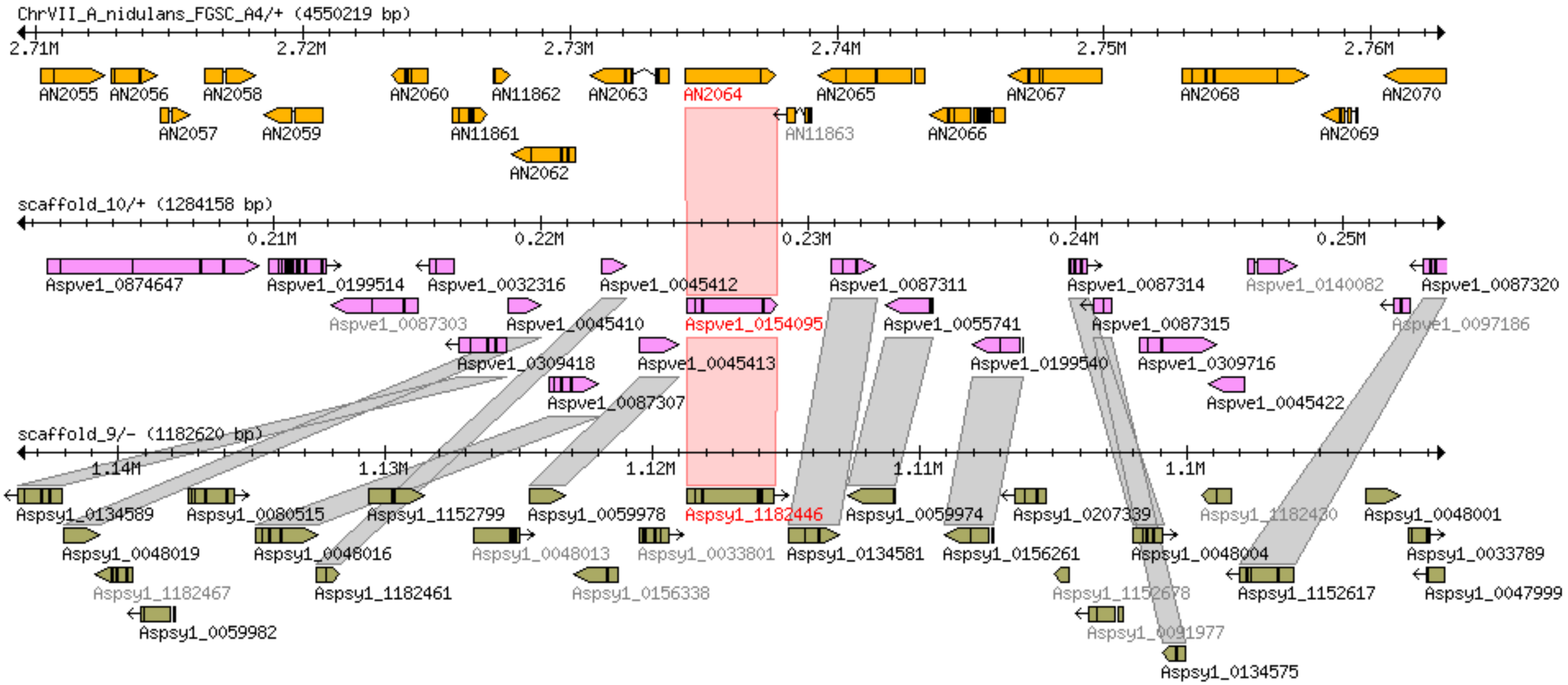

AN2064 cluster

Gene id (SMURF)*	Gene positions	Gene distance	Annotated_gene_function (SMURF)	AspGD gene name	AspGD manual annotation and notes	antiSMASH ID	Gene ID
n/a					50S ribosomal protein L12	prot_ID_86	AN2057
n/a					Protein of unknown function	prot_ID_908	AN2058
n/a				velC	Velvet family protein	prot_ID_232	AN2059
n/a				abxA	Exo-arabinanase	prot_ID_468	AN2060
n/a					oxidoreductase, transferring acyl groups	prot_ID_1485	AN11861
AN2061.3	3	26	conserved hypothetical protein		Deleted ORF		
n/a					Ortholog(s) have TRC complex, nucleus localization	prot_ID_1547	AN11862
AN2062.3	2	442	hypothetical protein similar to bipA	bipA	ER-resident chaperone of the HSP70 family	prot_ID_610	AN2062

AN2924 cluster

Possible false positive prediction

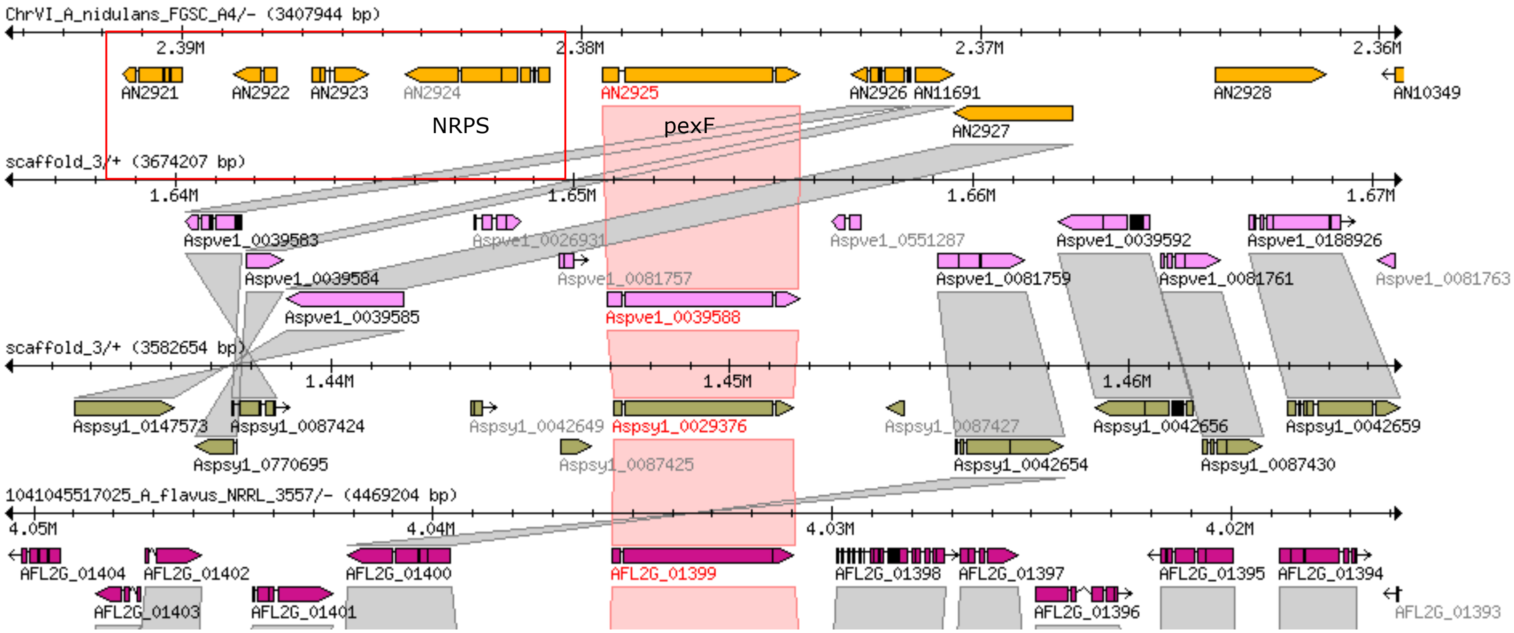

AN2924 cluster

Gene id (SMURF)*	Gene positions	Gene distance	Annotated_gene_function (SMURF)	AspGD gene name	AspGD manual annotation and notes	antiSMASH ID	Gene ID	
n/a					Similar to 26S proteasome regulatory subunit	prot_ID_127	AN2917	
n/a				cct4	Chaperonin complex component	prot_ID_722	AN2918	
n/a				sltA	C2H2 zinc-finger transcription factor	prot_ID_228	AN2919	
n/a				AN2920	Possible pseudogene			
AN2921.3	3	544	conserved hypothetical protein		Domain(s) with predicted nucleotide binding activity	prot_ID_1008	AN2921	EP
AN2922.3	2	1096	conserved hypothetical protein		Protein of unknown function	prot_ID_504	AN2922	
AN2923.3	1	951	conserved hypothetical protein		Protein of unknown function	prot_ID_16	AN2923	
**AN2924.3**	0	0	NRPS-like enzyme		NRPS-like enzyme	**prot_ID_611**	**AN2924**	EP
AN2925.3	-1	1615	conserved hypothetical protein	pexF	peroxisomal import protein	prot_ID_124	AN2925	
n/a				nsa2	60S ribosomal protein	prot_ID_719	AN2926	
n/a					Protein serine/threonine kinase	prot_ID_224	AN2927	
n/a					Protein of unknown function	prot_ID_1107	AN11691	
n/a					Protein of unknown function	prot_ID_827	AN2928	

AN3252 cluster

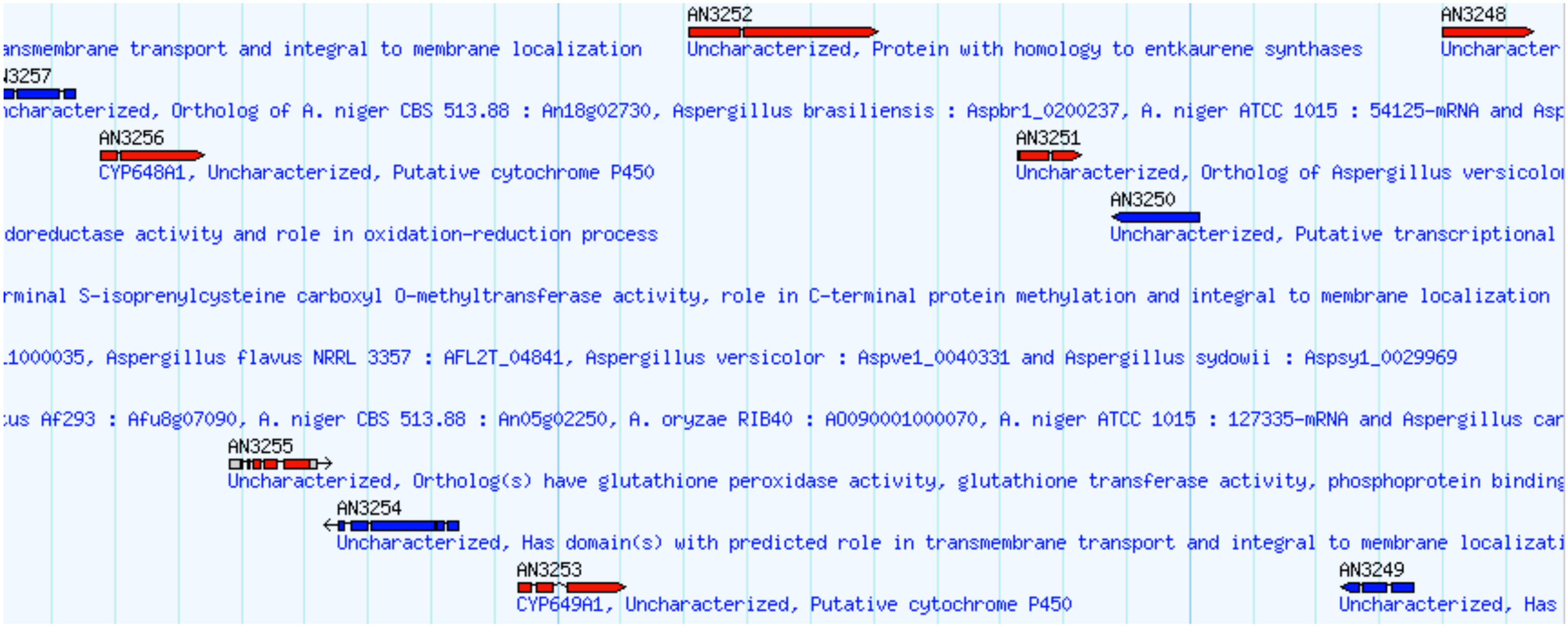

AN3252 cluster

Gene id (SMURF)*	Gene positions	Gene distance	Annotated_gene_function (SMURF)	AspGD gene name	AspGD manual annotation and notes	antiSMASH ID	Gene ID	
n/a					Protein of unknown function	n/a	AN3257	EP
n/a				CYP648A1	cytochrome P450	n/a	AN3256	
n/a					glutathione peroxidase	n/a	AN3255	
n/a					transmembrane transporter	n/a	AN3254	
n/a				*CYP649A1*	cytochrome P450	n/a	AN3253	
n/a					Entkaurene synthase	n/a	**AN3252**	EP

AN3273 cluster

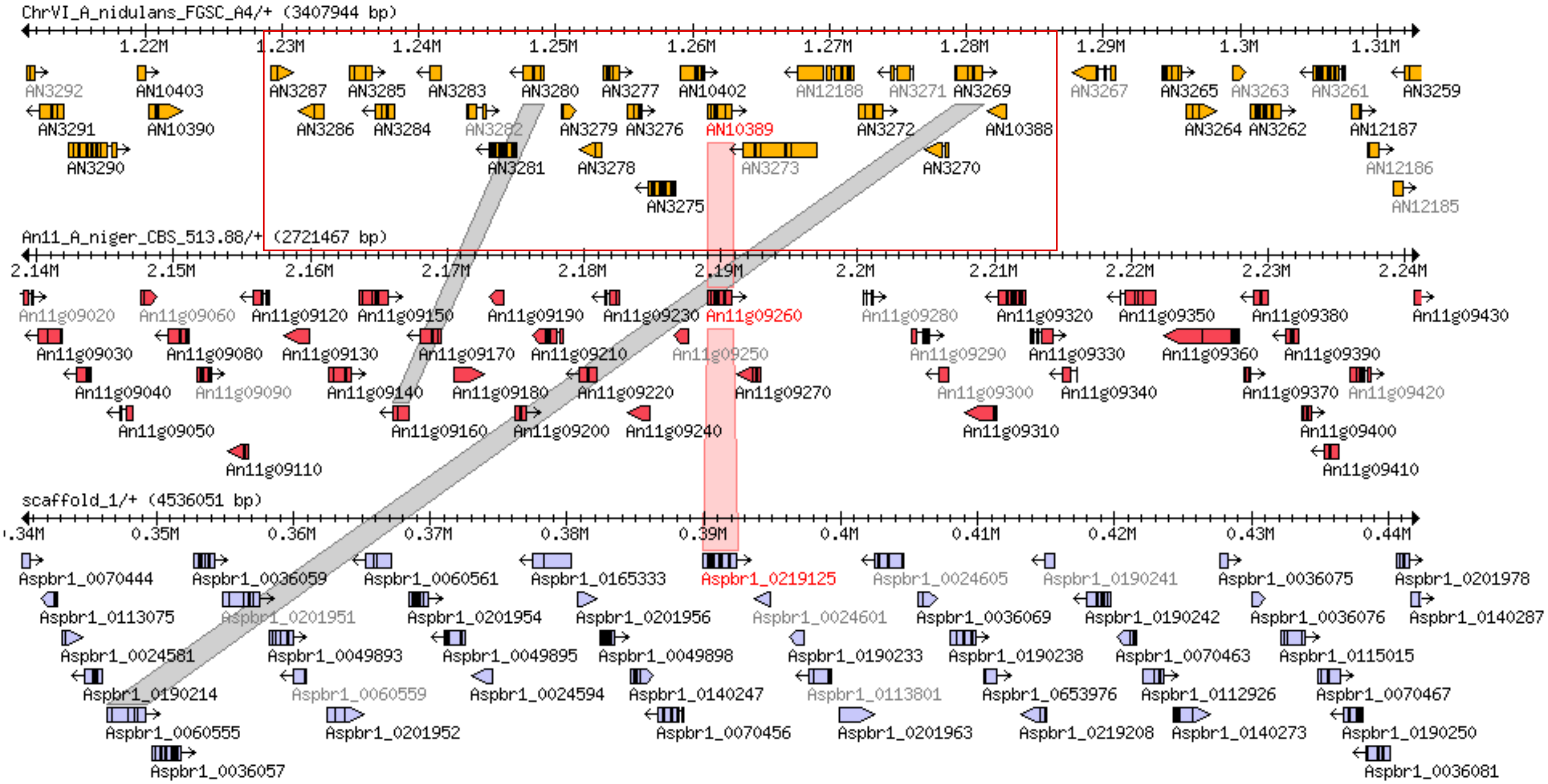

AN3273 cluster

Gene id (SMURF)*	Gene positions	Gene distance	Annotated_gene_function (SMURF)	AspGD gene name	AspGD manual annotation and notes	antiSMASH ID	Gene ID	
n/a					Protein of unknown function	prot_ID_773	AN3267	
n/a					UDP-N-acetylmuramate dehydrogenase	prot_ID_420	AN10388	IGD
n/a				AN10387	possible psuedogene	n/a		
n/a					Zn(II)2Cys6 transcription factor	prot_ID_882	AN3269	
n/a					Transmembrane transporter	prot_ID_1066	AN3270	
n/a					Protein of unknown function	prot_ID_556	AN3271	
n/a				CYP667A1	Cytochrome P450	prot_ID_71	AN3272	
n/a					Transferase	prot_ID_1165	AN12188	
n/a					Polketide synthase	prot_ID_666	**AN3273**	
n/a				CYP654A1	Cytochrome P450	prot_ID_1028	AN10389	
n/a					UDP-N-acetylmuramate dehydrogenase	prot_ID_523	AN10402	
n/a				CYP567E1	Cytochrome P450	prot_ID_771	AN3275	
n/a					Type II fatty acid synthase	prot_ID_278	AN3276	
n/a					Terpene synthase	prot_ID_879	AN3277	
n/a					Protein of unknown function	prot_ID_387	AN3278	
n/a					Oxidoreductase	prot_ID_994	AN3279	
n/a					Zn(II)2Cys6 transcription factor	prot_ID_70	AN3280	
n/a				CYP567D1	Cytochrome P450	prot_ID_665	AN3281	
n/a					Protein of unknown function	prot_ID_177	AN3282	
n/a					Protein of unknown function	prot_ID_770	AN3283	
n/a					Protein of unknown function	prot_ID_277	AN3284	
n/a					Protein of unknown function	prot_ID_877	AN3285	
n/a					Protein of unknown function	n/a	AN3286	
n/a					Transmembrane transporter	n/a	AN3287	IGD

AN3612 cluster

overlaps AN10430 cluster

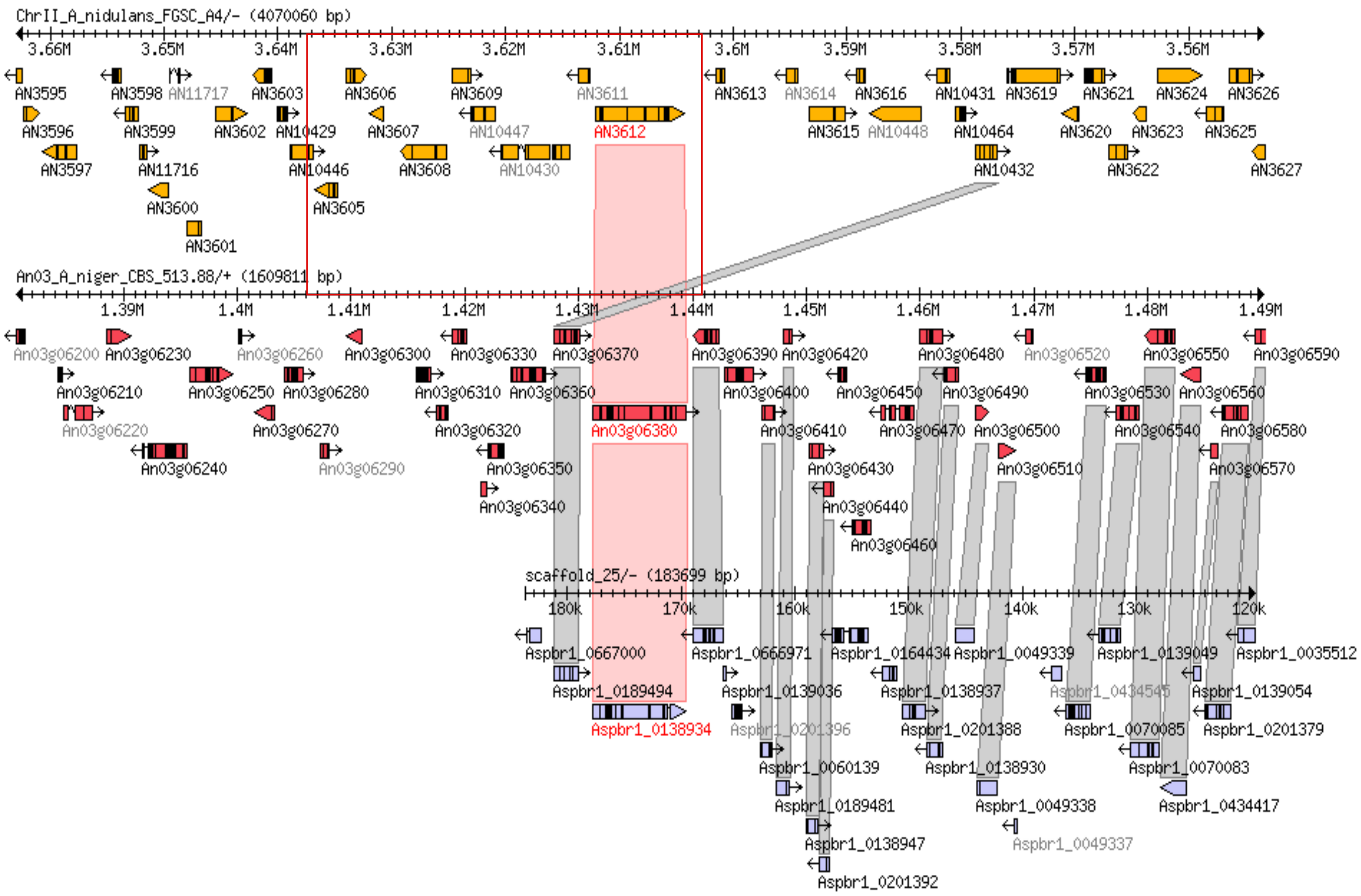

AN3612 cluster (overlaps AN10430 cluster)

Gene id (SMURF)*	Gene positions	Gene distance	Annotated_gene_function (SMURF)	AspGD gene name	AspGD manual annotation and notes	antiSMASH ID	Gene ID	
n/a					Phosphoserine phosphatase	prot_ID_665	AN10429	
n/a					Phosphoprotein phosphatase	prot_ID_59	AN10446	
n/a					Oxidoreductase	prot_ID_906	AN3605	FA
n/a					Protein of unknown function	prot_ID_296	AN3606	
n/a				lreB	Znc-finger transcription factor	prot_ID_1042	AN3607	
AN3608.3	5	506	hypothetical protein similar to ABC transporter protein AtrC	atrC	ABC transporter	prot_ID_423	AN3608	
AN3609.3	4	25	conserved hypothetical protein		Cytochrome P450	prot_ID_1168	AN3609	
AN10447.3	3	615	conserved hypothetical protein		Acyl transferase	prot_ID_789	AN10447	
AN10430.3	2	773	polyketide synthase		Polyketide synthase	prot_ID_886	AN10430	
AN3611.3	1	425	conserved hypothetical protein		Protein of unknown function	prot_ID_769	AN3611	
**AN3612.3**	0	0	polyketide synthase		Polyketide synthase	**prot_ID_164**	**AN3612**	ECS, FA
n/a				xlnA	Endo-1,4-beta-xylanase	prot_ID_903	AN3613	
n/a					Protein of unknown function	prot_ID_292	AN3614	
n/a					Nucleoside-triphosphatase	prot_ID_1039	AN3615	
n/a					Oxidoreductase	prot_ID_421	AN3616	
n/a				tprA	Tetratricopeptide repeat (TPR)-containing protein	prot_ID_186	AN10448	

AN4827 cluster

Functional annotations not related to secondary metabolism

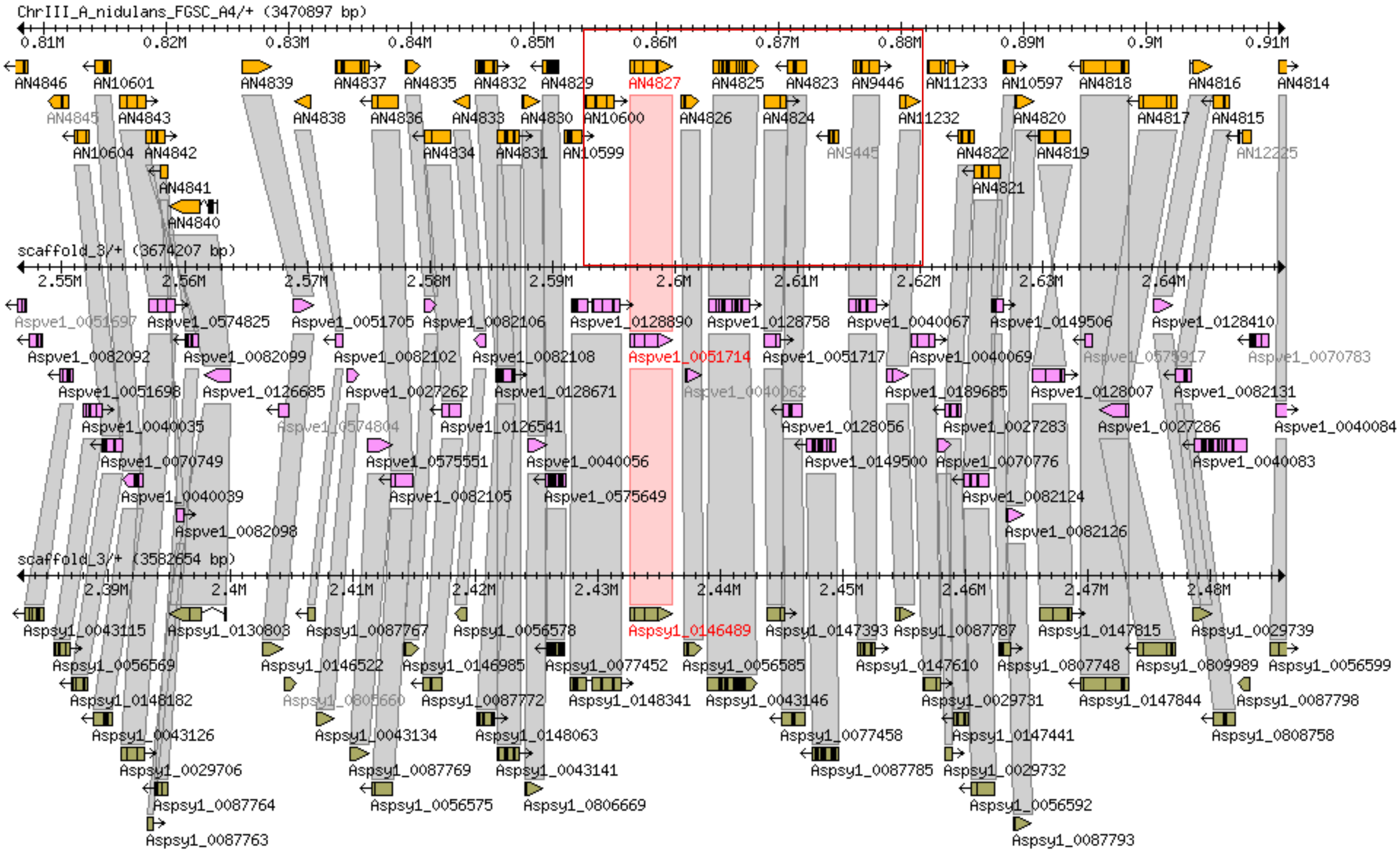

AN4827 cluster

Gene id (SMURF)*	Gene positions	Gene distance	Annotated_gene_function (SMURF)	AspGD gene name	AspGD manual annotation and notes	antiSMASH ID	Gene ID	
n/a					Phosphatidylinositol:UDP-GlcNAc transferase PIG-C	prot_ID_780	AN11232	EP
n/a				panK	Pantothenate kinase	prot_ID_816	AN9446	
n/a					ATP-dependent helicase	prot_ID_187	AN9445	
AN4823.3	4	227	conserved hypothetical protein		Chitin deacetylase	prot_ID_371	AN4823	
AN4824.3	3	635	conserved hypothetical protein		Glycosyl transferase	prot_ID_1008	AN4824	
AN4825.3	2	1077	conserved hypothetical protein		Glucan 1,3-beta-glucosidase	prot_ID_485	AN4825	
AN4826.3	1	785	conserved hypothetical protein		Transmembrane transporter	prot_ID_1120	AN4826	
AN4827.3	0	0	NRPS-like enzyme		NRPS-like enzyme	**prot_ID_590**	**AN4827**	
AN10600.3	-1	1290	hypothetical protein		Zn(II)2Cys6 transcription factor	prot_ID_800	AN10600	EP
AN10599.3	-2	315	hypothetical protein		Mandelate racemase/muconate lactonizing enzyme family protein	prot_ID_384	AN10599	
AN4829.3	-3	519	hypothetical protein		Reductase with a predicted role in carbohydrate metabolism	prot_ID_701	AN4829	
AN4830.3	-4	222	conserved hypothetical protein		Phosphopantothenate-cysteine ligase	prot_ID_892	AN4830	
AN4831.3	-5	413	conserved hypothetical protein		Oxidoreductase	prot_ID_370	AN4831	
AN4832.3	-6	506	conserved hypothetical protein		Protein of unknown function	prot_ID_1007	AN4832	
AN4833.3	-7	632	conserved hypothetical protein		Hydrolase	prot_ID_484	AN4833	
AN11448.3	-8	317	predicted protein		Deleted by the Broad	n/a	AN11448	
AN4834.3	-9	636	conserved hypothetical protein		Protein of unknown function	prot_ID_1119	AN4834	
AN4835.3	-10	319	conserved hypothetical protein		Domain(s) with predicted role in cell division, chromosome segregation	prot_ID_589	AN4835	
AN4836.3	-11	494	conserved hypothetical protein		Ortholog(s) have Golgi apparatus localization	prot_ID_75	AN4836	
**AN4837.3**	-12	559	conserved hypothetical protein		Zn(II)2Cys6 transcription factor	n/a	AN4837	

AN5318 cluster

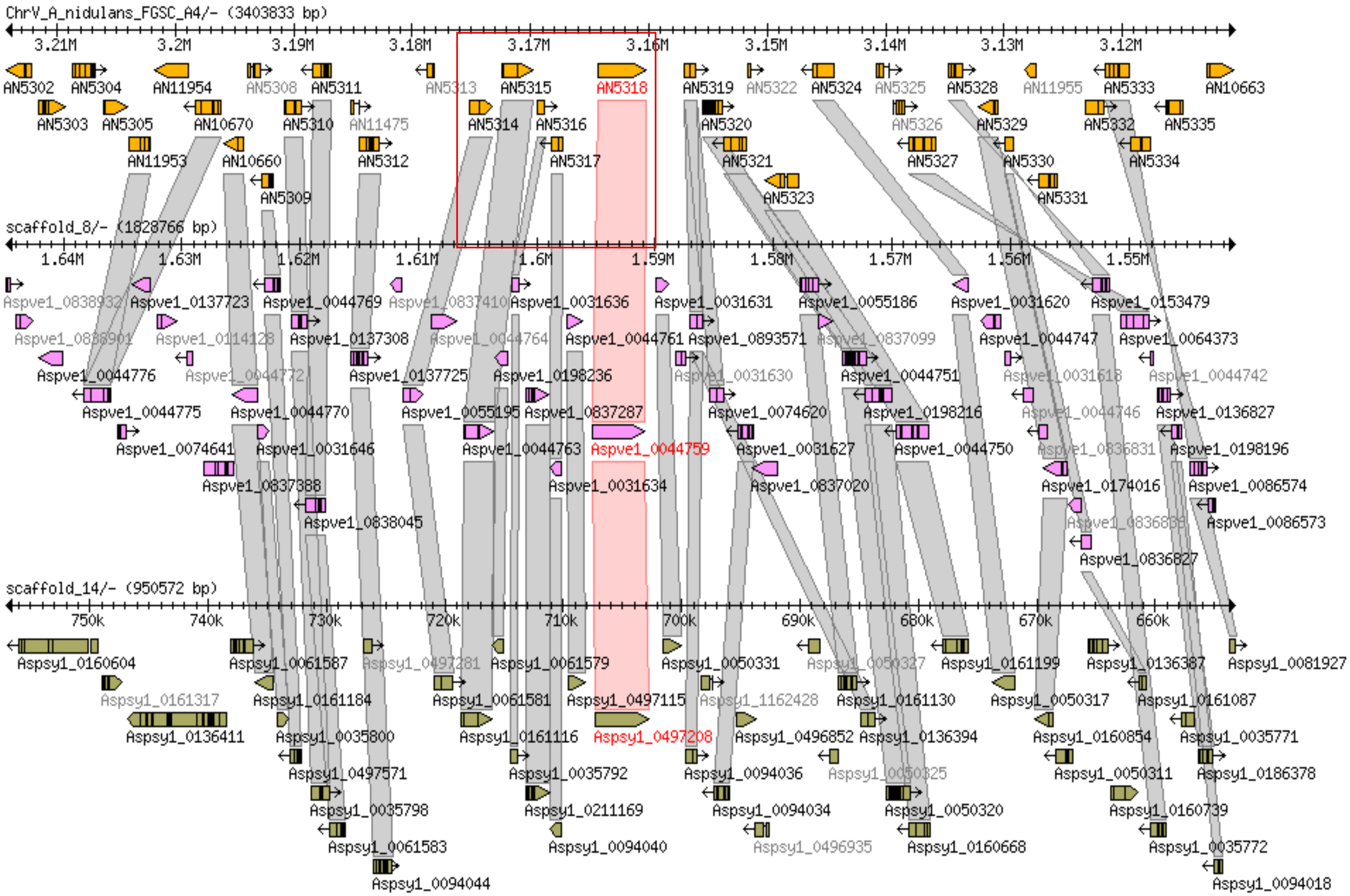

AN5318 cluster

Gene id (SMURF)*	Gene positions	Gene distance	Annotated_gene_function (SMURF)	AspGD gene name	AspGD manual annotation and notes	antiSMASH ID	Gene ID	
n/a					Protein of unknown function	prot_ID_137	AN5312	
n/a					Protein of unknown function	prot_ID_707	AN5313	
n/a					Domain(s) with predicted catalytic activity, pyridoxal phosphate binding	prot_ID_242	AN5314	ECS
n/a					Protein of unknown function	prot_ID_811	AN5315	
n/a					Protein of unknown function	prot_ID_342	AN5316	
AN5317.3	1	2716	conserved hypothetical protein		NmrA-like family protein	prot_ID_903	AN5317	
**AN5318.3**	0	0	NRPS-like enzyme		Non-ribosomal peptide synthetase	**prot_ID_448**	**AN5318**	ECS
AN5319.3	-1	3277	conserved hypothetical protein		Protein of unknown function	prot_ID_1006	AN5319	
AN5320.3	-2	475	conserved hypothetical protein		Hydrolase	prot_ID_134	AN5320	
AN5321.3	-3	105	conserved hypothetical protein		Triacylglycerol lipase	prot_ID_704	AN5321	
AN5322.3	-4	200	conserved hypothetical protein		Protein of unknown function	prot_ID_239	AN5322	
AN5323.3	-5	1143	hypothetical protein		Substrate-specific transmembrane transporter	prot_ID_809	AN5323	
AN5324.3	-6	1860	conserved hypothetical protein	dlpA	Dehydrin-like protein	prot_ID_339	AN5324	
AN5325.3	-7	3548	conserved hypothetical protein		Protein of unknown function	prot_ID_901	AN5325	
AN5326.3	-8	646	conserved hypothetical protein		Protein of unknown function	n/a	AN5326	
AN5327.3	-9	161	conserved hypothetical protein		GTPase	n/a	AN5327	
AN5328.3	-10	1031	conserved hypothetical protein		Oxidoreductase	n/a	AN5328	
AN5329.3	-11	1328	conserved hypothetical protein		Transmembrane transporter	n/a	AN5329	

AN5475 cluster

Possible orphan PKS, no boundary prediction by any method

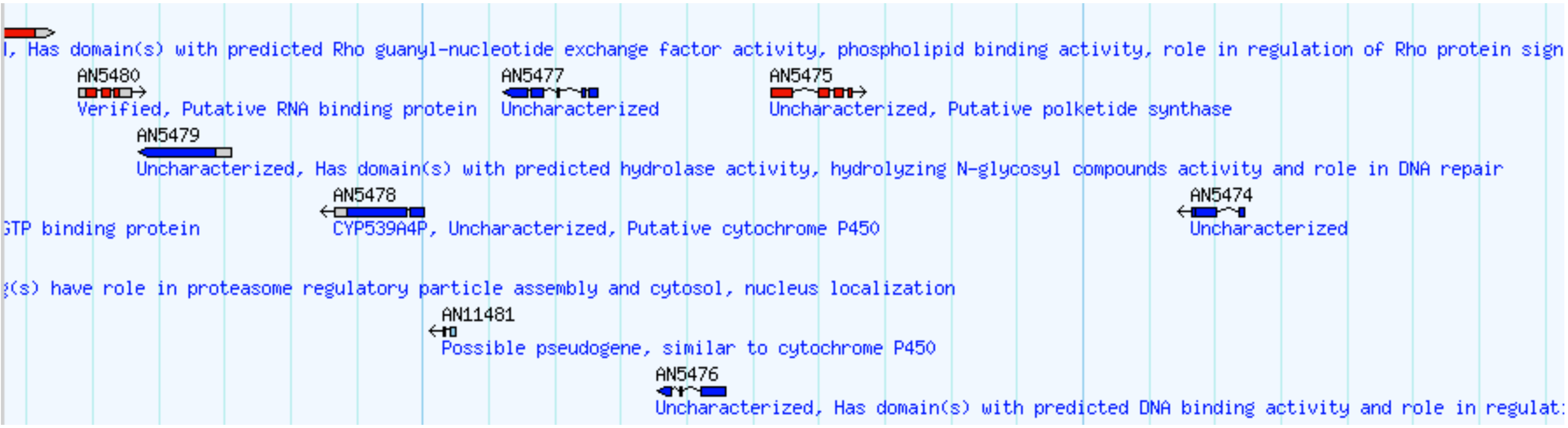

AN5475 cluster

Gene id (SMURF)*	Gene positions	Gene distance	Annotated_gene_function (SMURF)	AspGD gene name	AspGD manual annotation and notes	antiSMASH ID	Gene ID
n/a					Polyketide synthase	n/a	AN5475

AN5610 cluster

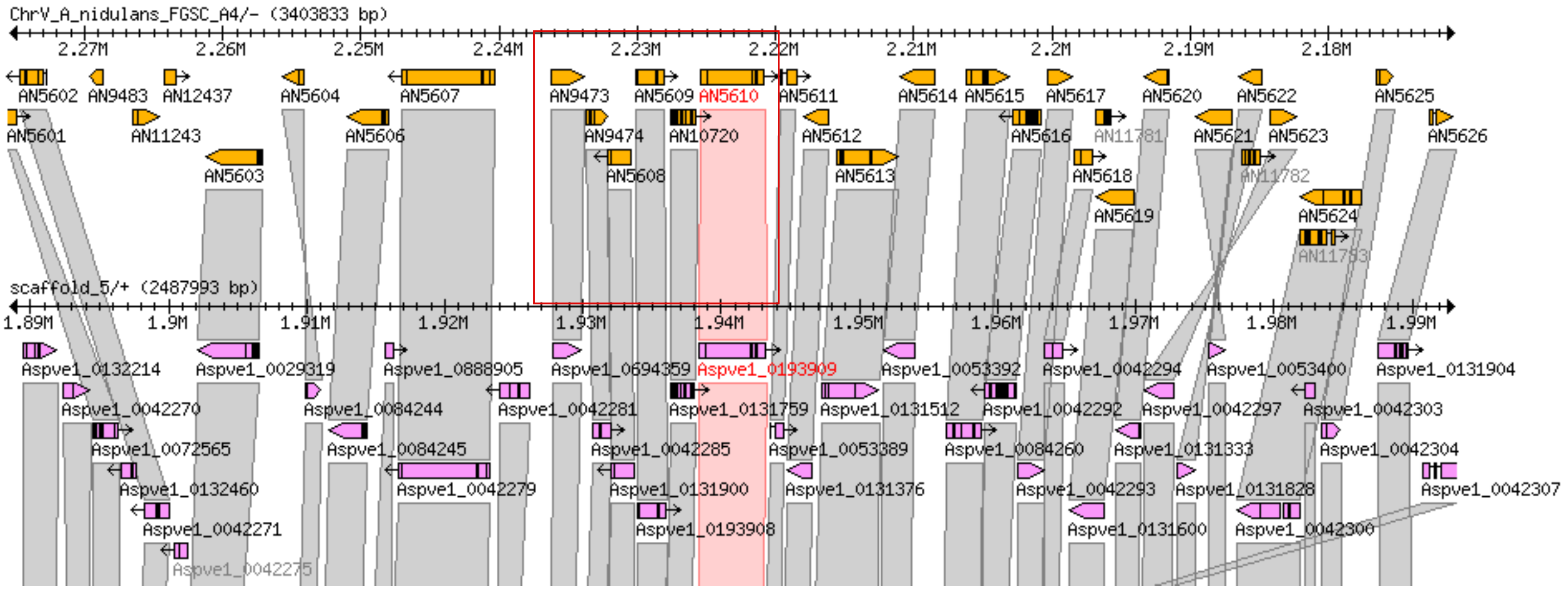

AN5610 cluster

Gene id (SMURF)*	Gene positions	Gene distance	Annotated_gene_function (SMURF)	AspGD gene name	AspGD manual annotation and notes	antiSMASH ID	Gene ID	
n/a					proteasome activator subunit 4	prot_ID_259	AN5607	
n/a					Protein of unknown function	prot_ID_380	AN9473	ECS, IGD
n/a					Aldo-keto reductase	prot_ID_938	AN9474	
n/a					Protein of unknown function	prot_ID_829	AN5608	
n/a					Zn(II)2Cys6 transcription factor	prot_ID_360	AN5609	
n/a					MFS transporter	prot_ID_44	AN10720	
n/a					L-aminoadipate-semialdehyde dehydrogenase	prot_ID_534	AN5610	FA
n/a				canA	Carbonic anhydrase	prot_ID_58	AN5611	
n/a					Protein of unknown function	prot_ID_629	AN5612	
n/a				hxA	Xanthine dehydrogenase	prot_ID_155	AN5613	
n/a					Pheromone-regulated multispanning membrane protein	prot_ID_727	AN5614	
n/a					Protein of unknown function	prot_ID_257	AN5615	
n/a					Kynurenine aminotransferase	prot_ID_828	AN5616	

AN6236 cluster

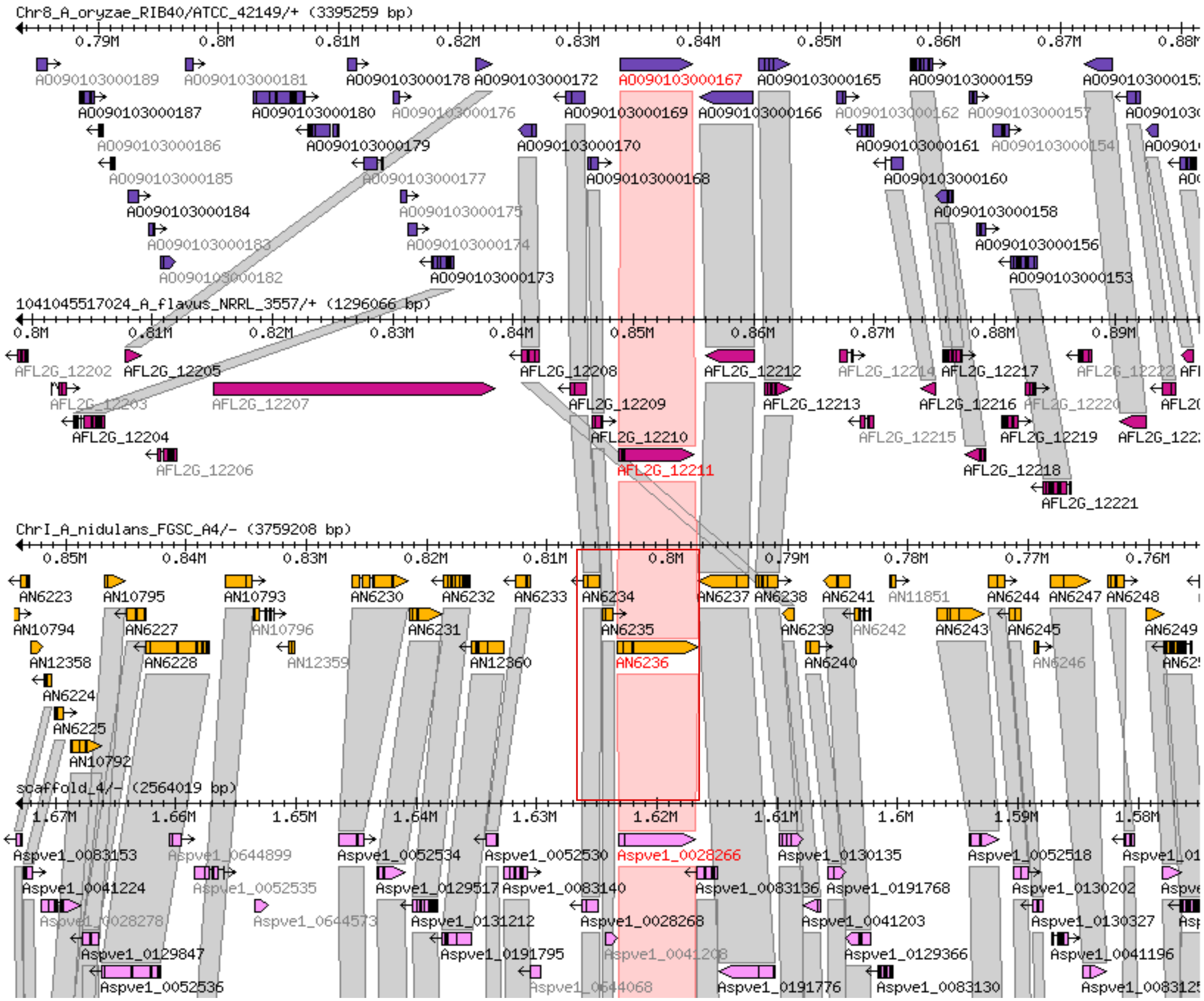

AN6236 cluster

Gene id (SMURF)*	Gene positions	Gene distance	Annotated_gene_function (SMURF)	AspGD gene name	AspGD manual annotation and notes	antiSMASH ID	Gene ID	
n/a				medA	medusa, regulator of conidiophore development	prot_ID_378	AN6230	
n/a				trpB	Tryptophan synthase	prot_ID_1061	AN6231	
n/a				vmaB	F1F0-ATPase complex subunit	prot_ID_500	AN6232	
n/a					Protein of unknown function	prot_ID_1266	AN12360	
n/a					DnaJ domain protein	prot_ID_1182	AN6233	
n/a					siderophore biosynthesis acetylase AceI	prot_ID_625	AN6234	ECS, EP
n/a					Enoyl-CoA hydratase/isomerase family protein	prot_ID_60	AN6235	
**AN6236.3**	0	0	nonribosomal peptide synthetase		Nonribosomal peptide synthetase	**prot_ID_745**	**AN6236**	ECS, EP
AN6237.3	-1	353	hypothetical protein		Transmembrane transporter	prot_ID_178	AN6237	
AN6238.3	-2	358	conserved hypothetical protein		Transmembrane transporter	prot_ID_866	AN6238	
n/a					Protein of unknown function	prot_ID_293	AN6239	
n/a					Protein of unknown function	prot_ID_496	AN6240	
n/a					Protein of unknown function	prot_ID_1179	AN6241	
n/a					Protein of unknown function	prot_ID_622	AN6242	
n/a					Protein of unknown function	prot_ID_1263	AN11851	

AN6431 cluster

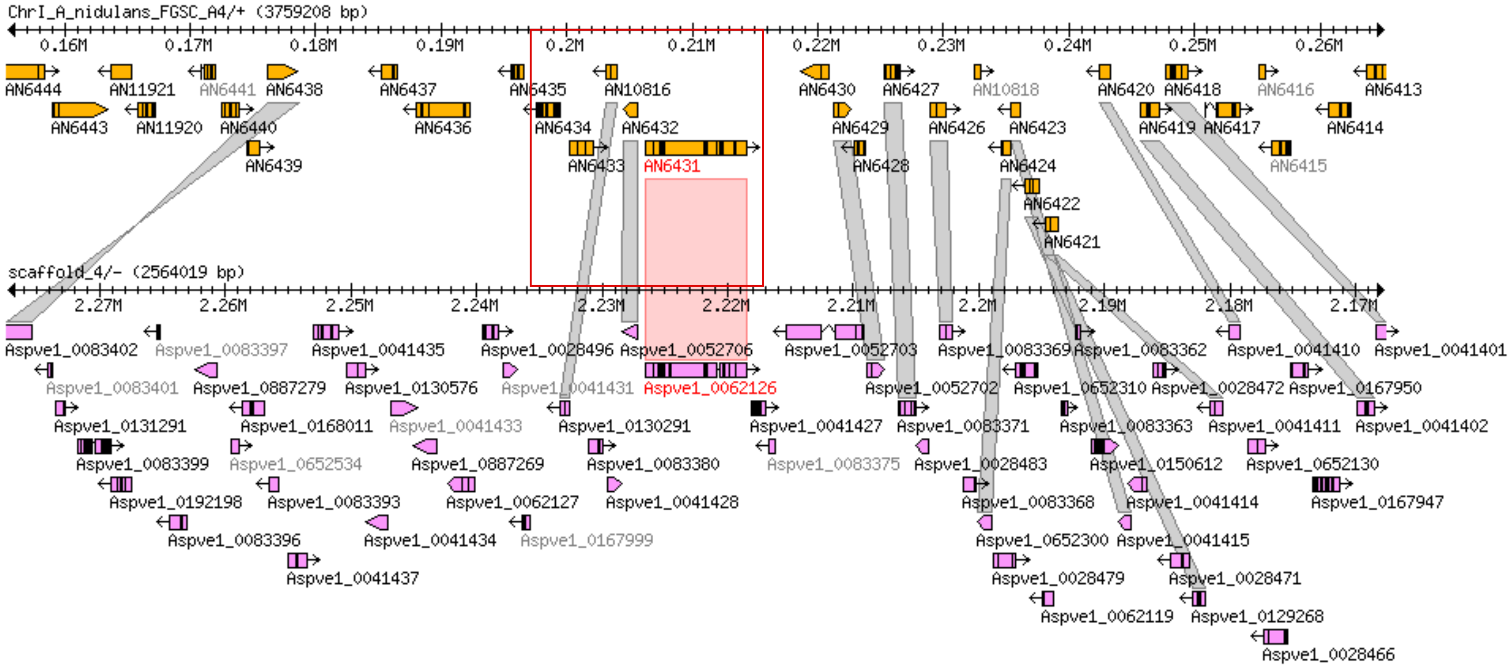

AN6431 cluster

Gene id (SMURF)*	Gene positions	Gene distance	Annotated_gene_function (SMURF)	AspGD gene name	AspGD manual annotation and notes	antiSMASH ID	Gene ID	
n/a					Protein of unknown function	prot_ID_631	AN10818	
n/a					Protein of unknown function	prot_ID_1011	AN6426	
n/a				manC	Endo-beta-(1,4)-mannanase	prot_ID_449	AN6427	
n/a					Beta-1,4-endoglucanase	prot_ID_1135	AN6428	
AN6429.3	2	331	conserved hypothetical protein		Transmembrane transporter	prot_ID_574	AN6429	
AN6430.3	1	1146	C6 transcription factor		Zn(II)2Cys6 transcription factor	prot_ID_773	AN6430	
**AN6431.3**	0	0	polyketide synthase		Polyketide synthase	**prot_ID_204**	**AN6431**	ECS, EP
AN6432.3	-1	581	conserved hypothetical protein		Protein of unknown function	prot_ID_894	AN6432	
AN10816.3	-2	528	hypothetical protein		Protein of unknown function	prot_ID_507	AN10816	
AN6433.3	-3	136	conserved hypothetical protein		Predicted transferase	prot_ID_324	AN6433	
AN6434.3	-4	806	conserved hypothetical protein	CYP672A1	Cytochrome P450	prot_ID_1010	AN6434	EP
n/a					Protein of unknown function	prot_ID_448	AN6435	
n/a					Transmembrane transporter	prot_ID_1134	AN6436	
n/a					Protein of unknown function	prot_ID_573	AN6437	

AN6791 cluster

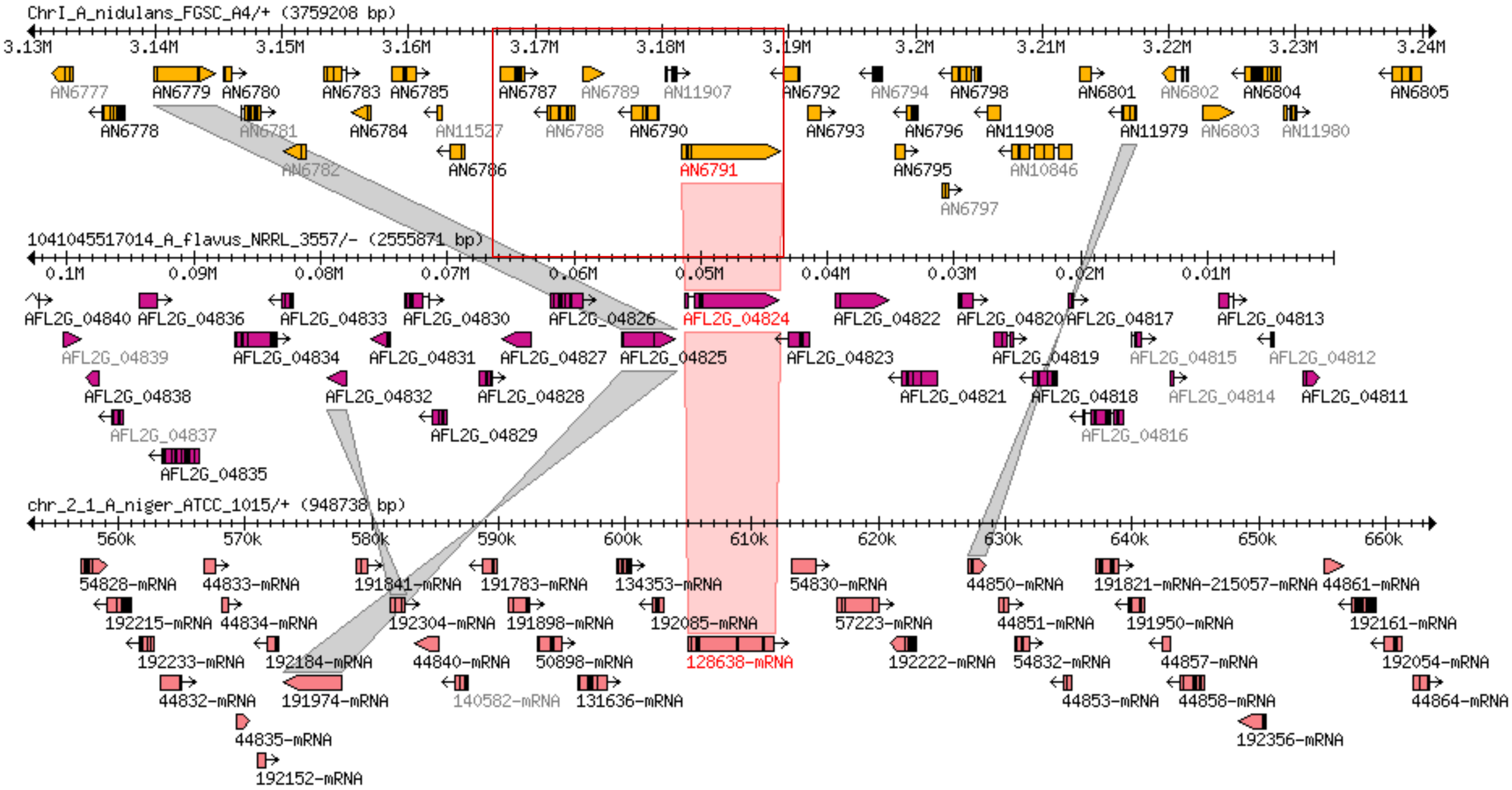

AN6791 cluster

Gene id (SMURF)*	Gene positions	Gene distance	Annotated_gene_function (SMURF)	AspGD gene name	AspGD manual annotation and notes	antiSMASH ID	Gene ID	
n/a					Protein of unknown function	prot_ID_1002	AN11527	
n/a					Beta-1,4-endoglucanase	prot_ID_1076	AN6786	
n/a				CYP682C1	Cytochrome P450	prot_ID_515	AN6787	IGD
n/a					Zn(II)2Cys6 transcription factor	prot_ID_1196	AN6788	
n/a					Protein of unknown function	prot_ID_638	AN6789	
n/a					Protein of unknown function	prot_ID_838	AN6790	
n/a					Protein of unknown function	prot_ID_1313	AN11907	
n/a					Polyketide synthase	prot_ID_265	**AN6791**	FA
n/a					NAD+ dependent glycerol 3-phosphate dehydrogenase	prot_ID_953	AN6792	
n/a					Protein of unknown function	prot_ID_390	AN6793	
n/a					Protein of unknown function	prot_ID_1075	AN6794	
n/a					Protein of unknown function	prot_ID_514	AN6795	
n/a					ThiJ/PfpI family protein	prot_ID_1195	AN6796	
n/a					Protein of unknown function	prot_ID_636	AN6797	
n/a					Protein of unknown function	prot_ID_72	AN6798	
n/a					Hydrolase	prot_ID_1323	AN11908	
n/a						prot_ID_864	AN10846	

AN7084 cluster

Overlaps end of pkg cluster

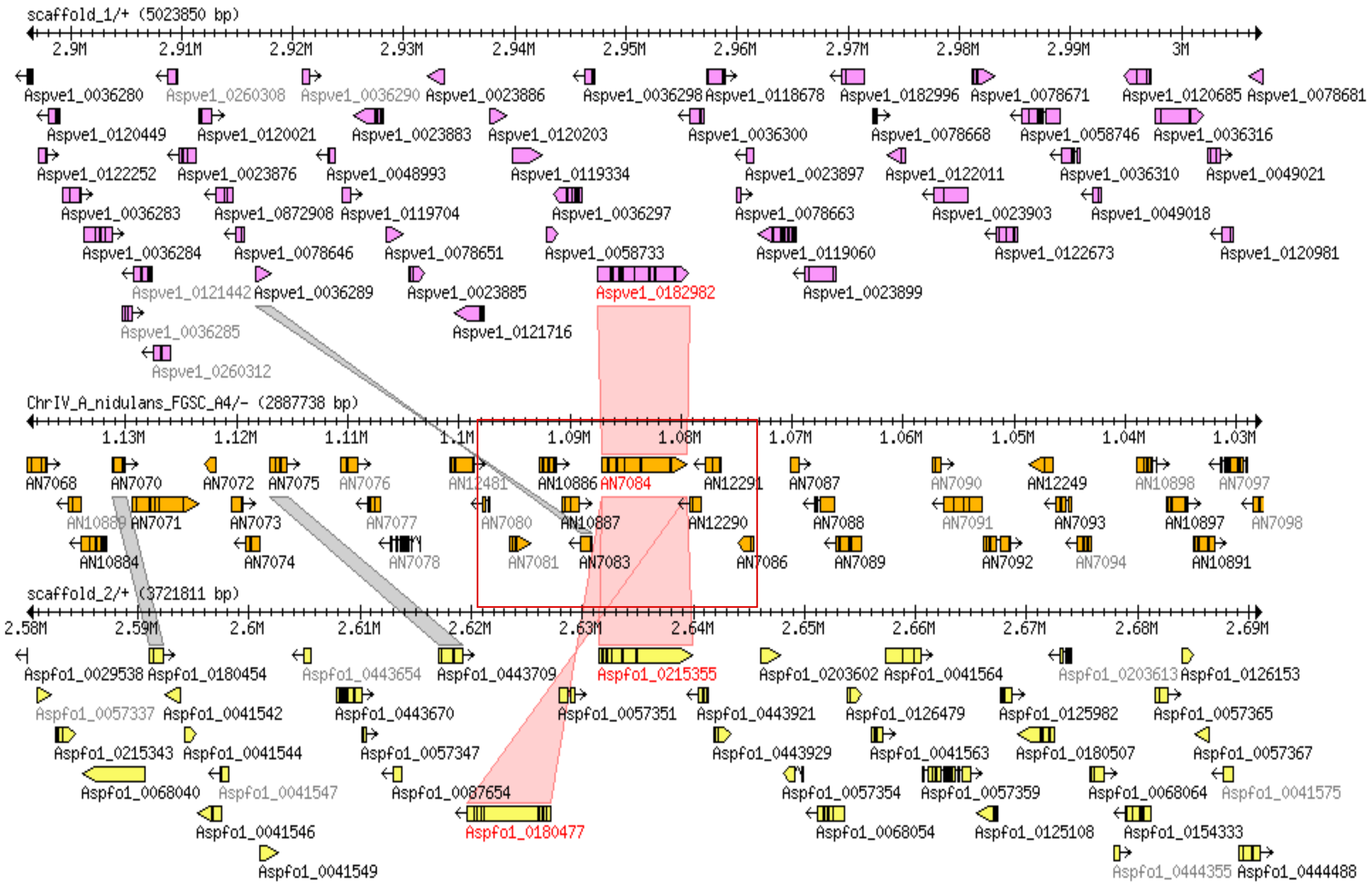

AN7084 cluster

Gene id (SMURF)*	Gene positions	Gene distance	Annotated_gene_function (SMURF)	AspGD gene name	AspGD manual annotation and notes	antiSMASH ID	Gene ID	
n/a				acnA	Actinin-like protein	prot_ID_412	AN7077	
n/a					Protein of unknown function	prot_ID_930	AN7078	
n/a					Protein of unknown function	prot_ID_1004	AN12481	
n/a					Protein of unknown function	prot_ID_648	AN7080	EP
AN7081.3	4	839	conserved hypothetical protein		UDP-N-acetylmuramate dehydrogenase	prot_ID_229	AN7081	
AN10886.3	3	399	conserved hypothetical protein		Protein of unknown function	prot_ID_59	AN10886	
AN10887.3	2	79	conserved hypothetical protein	CYP5095B1	cytochrome P450	prot_ID_568	AN10887	
AN7083.3	1	612	conserved hypothetical protein		Aminopeptidase	prot_ID_319	AN7083	
**AN7084.3**	0	0	PKS-like enzyme		polyketide synthase (PKS)-like enzyme	**prot_ID_836**	**AN7084**	
n/a					Oxidoreductase	prot_ID_977	AN12290	
n/a					Protein of unknown function	prot_ID_999	AN12291	
AN7085.3	-1	239	conserved hypothetical protein		Deleted ORF	n/a		
AN7086.3	-2	1669	conserved hypothetical protein		F-box protein	prot_ID_928	AN7086	EP
AN7087.3	-3	3289	conserved hypothetical protein		Protein of unknown function	prot_ID_502	AN7087	
AN7088.3	-4	1397	conserved hypothetical protein		Transmembrane transporter	prot_ID_81	AN7088	
AN7089.3	-5	146	conserved hypothetical protein		Protein of unknown function	prot_ID_591	AN7089	

AN7489 (mirC) cluster

Annotations not typical of secondary metabolite gene clusters

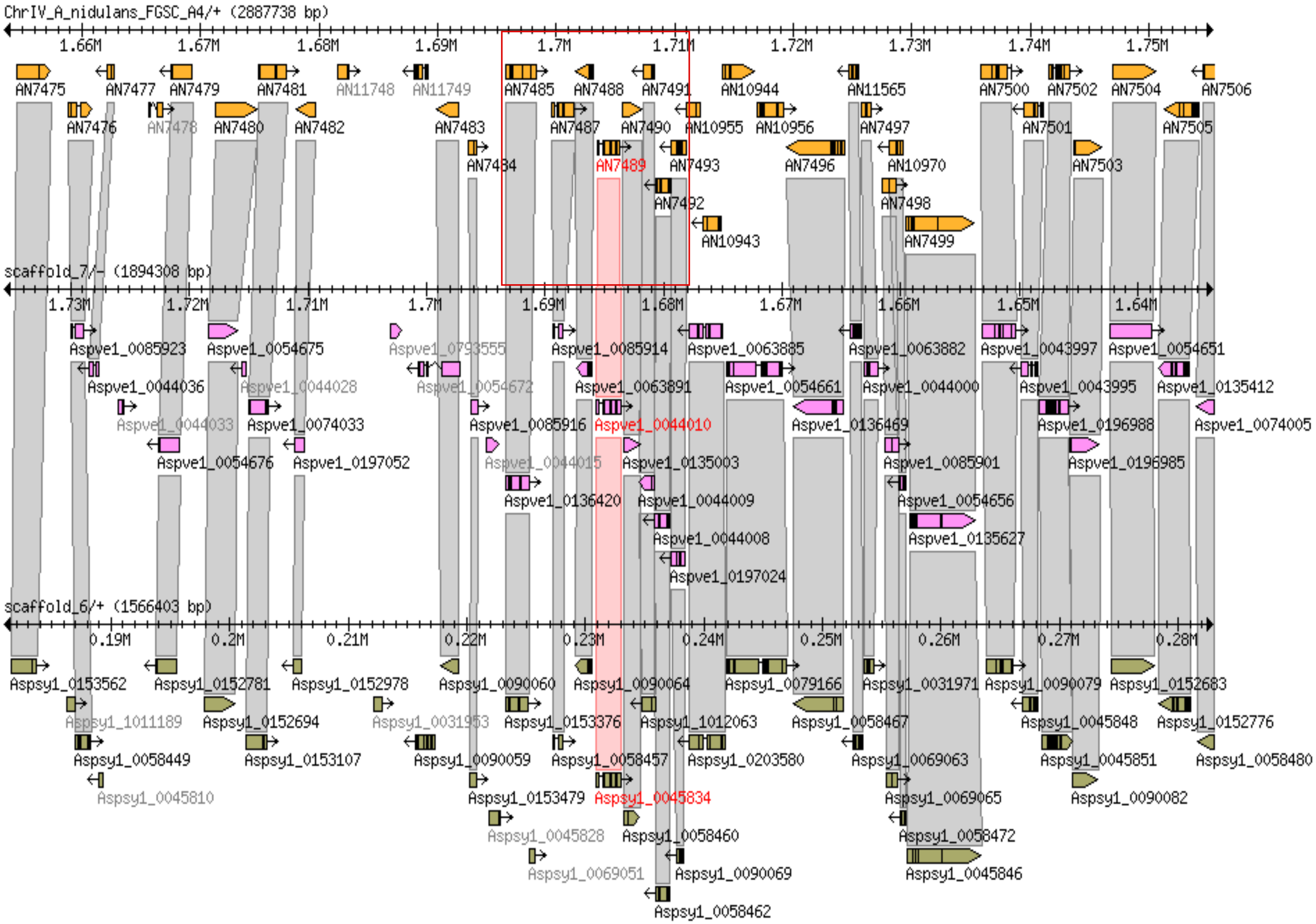

AN7489 (mirC) cluster

Gene id (SMURF)*	Gene positions	Gene distance	Annotated_gene_function (SMURF)	AspGD gene name	AspGD manual annotation and notes	antiSMASH ID	Gene ID	
n/a					Protein of unknown function	n/a	AN11749	
n/a					Protein of unknown function	n/a	AN7483	
n/a					DUF1349 domain protein	n/a	AN7484	
AN7485.3	4	103	siderophore iron transporter mirC	mirC	siderophore iron transporter	n/a	AN7485	ECS
AN7486.3	3	595	predicted protein		Deleted by the Broad	n/a	AN7486	
AN7487.3	2	81	hypothetical protein similar to MODA	modA	Rho GTPase	n/a	AN7487	
AN7488.3	1	598	conserved hypothetical protein		agmatinase	n/a	AN7488	
**AN7489.3**	0	0	mitochondrial 3-oxoacyl-[acyl-carrier-protein]-synthase		mitochondrial 3-oxoacyl-[acyl-carrier-protein]-synthase	n/a	AN7489	
n/a					MT-A70 family protein	n/a	AN7490	
n/a					ubiquitin C-terminal hydrolase	n/a	AN7491	
n/a					mRNA decapping hydrolase	n/a	AN7492	
n/a					Protein of unknown function	n/a	AN7493	ECS

AN7838 (AN12331) cluster

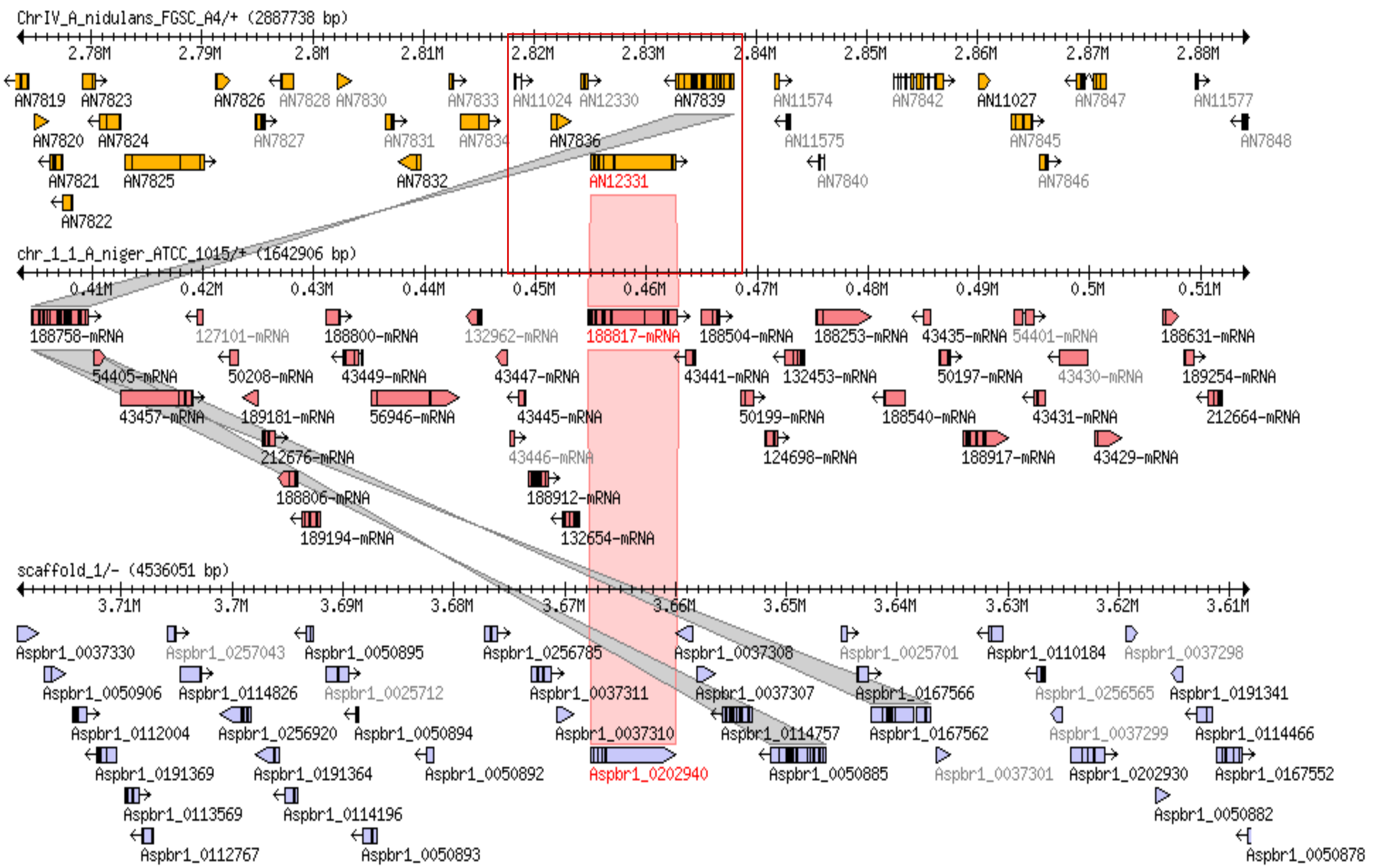

AN7838 (AN12331) cluster

Gene id (SMURF)*	Gene positions	Gene distance	Annotated_gene_function (SMURF)	AspGD gene name	AspGD manual annotation and notes	antiSMASH ID	Gene ID	
n/a					Protein of unknown function	n/a	AN7831	
n/a					Oxidoreductase	n/a	AN7832	
n/a					Protein of unknown function	n/a	AN7833	
n/a					Protein of unknown function	n/a	AN7834	
n/a					Oxidoreductase	n/a	AN11024	FA
AN7836.3	2	1176	conserved hypothetical protein		Protein of unknown function	n/a	AN7836	
AN7837.3	1	225	PKS-like enzyme		Deleted ORF	n/a	AN7837	
					Domain(s) with predicted catalytic activity and role in biosynthetic process		AN12330	
**AN7838.3**	0	0	PKS-like enzyme		Polyketide synthase (PKS)-like enzyme	n/a	AN12331	
AN7839.3	-1	40	conserved hypothetical protein		Transmembrane transporter ATPase	n/a	AN7839	FA
n/a					Protein of unknown function	n/a	AN11574	
n/a					Protein of unknown function	n/a	AN11575	
					Protein of unknown function	n/a	AN7840	

AN7884 cluster

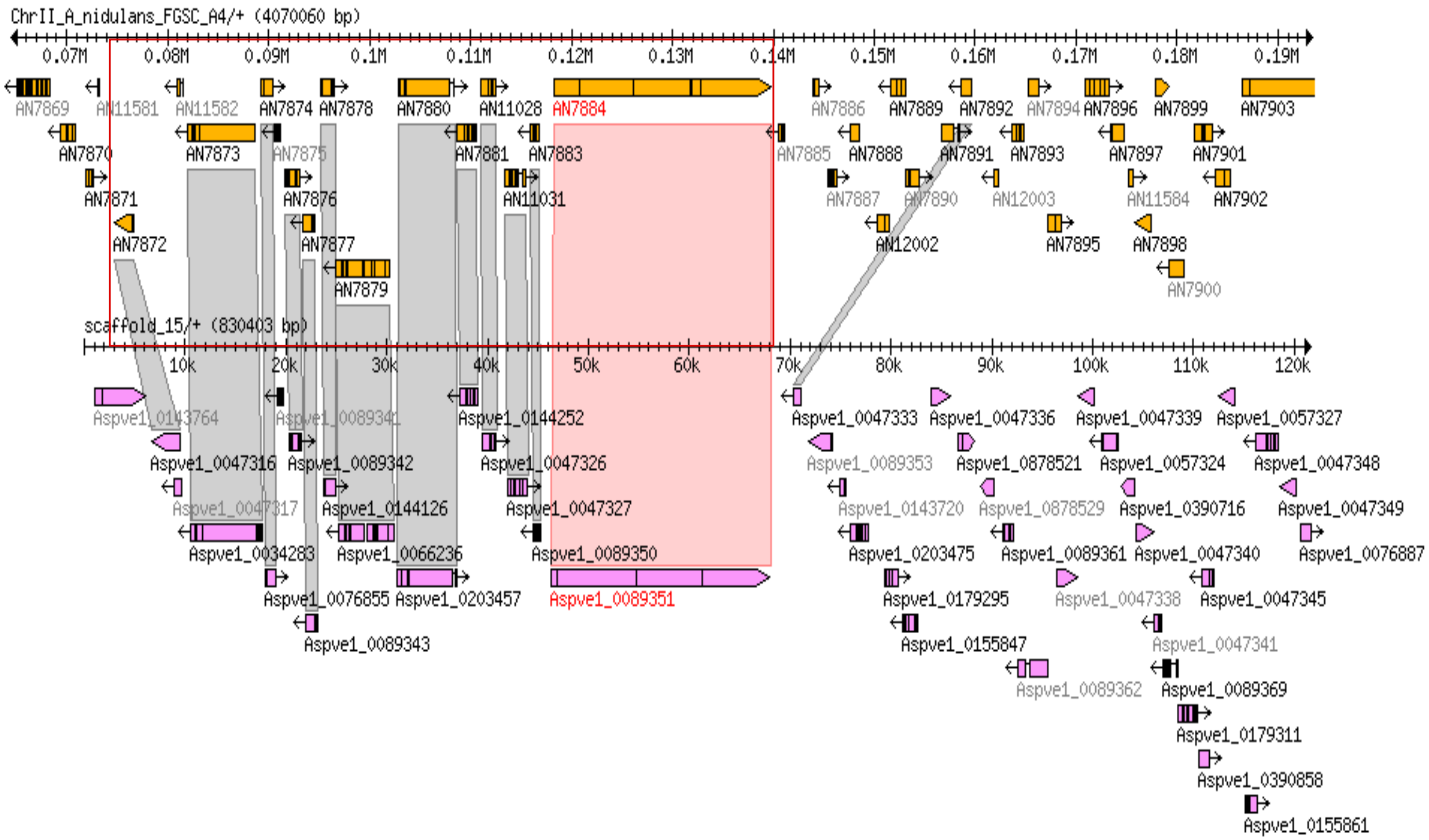

AN7884 cluster

Gene id (SMURF)*	Gene positions	Gene distance	Annotated_gene_function (SMURF)	AspGD gene name	AspGD manual annotation and notes	antiSMASH ID	Gene ID	
n/a					Zn(2)C(6) transcription factor	n/a	AN7872	ECS, EP
n/a					Protein of unknown function	n/a	AN11582	
AN7873.3	12	875	fatty acid synthase beta subunit, putative		Fatty acid synthase beta subunit	n/a	AN7873	
AN7874.3	11	247	conserved hypothetical protein		Protein of unknown function	n/a	AN7874	
AN7875.3	10	625	conserved hypothetical protein		Protein of unknown function	n/a	AN7875	
AN7876.3	9	228	conserved hypothetical protein		Branched chain amino acid aminotransferase	n/a	AN7876	
AN7877.3	8	552	conserved hypothetical protein		RTA1 domain protein	n/a	AN7877	
AN7878.3	7	311	conserved hypothetical protein		Branched chain amino acid aminotransferase	n/a	AN7878	
AN7879.3	6	1102	conserved hypothetical protein		Transmembrane transporter ATPase	prot_ID_1087	AN7879	
AN7880.3	5	99	fatty acid synthase subunit alpha		Fatty-acyl-CoA synthase	prot_ID_1305	AN7880	
AN7881.3	4	662	conserved hypothetical protein	CYP548C1	Cytochrome P450	prot_ID_684	AN7881	
AN11028.3	3	1127	conserved hypothetical protein		Oxidoreductase	prot_ID_743	AN11028	
AN11031.3	2	521	conserved hypothetical protein		Transmembrane transporter	prot_ID_1094	AN11031	
AN7883.3	1	1099	conserved hypothetical protein		Protein of unknown function	prot_ID_811	AN7883	
**AN7884.3**	0	0	nonribosomal peptide synthase		Nonribosomal peptide synthase	**prot_ID_212**	**AN7884**	ECS, EP
n/a					Protein of unknown function	prot_ID_951	AN7885	
n/a					Class V chitinase	prot_ID_339	AN7886	
n/a					Protein of unknown function	prot_ID_1085	AN7887	
n/a					Protein of unknown function	prot_ID_467	AN7888	
n/a					Dioxygenase	prot_ID_1397	AN12002	
n/a					Protein of unknown function	prot_ID_1213	AN7889	
n/a					Glucose oxidase	prot_ID_79	AN7890	
n/a					Beta-1,4-endoglucanase	prot_ID_809	AN7891	
n/a					Small heat-shock protein	prot_ID_209	AN7892	

AN8105 cluster

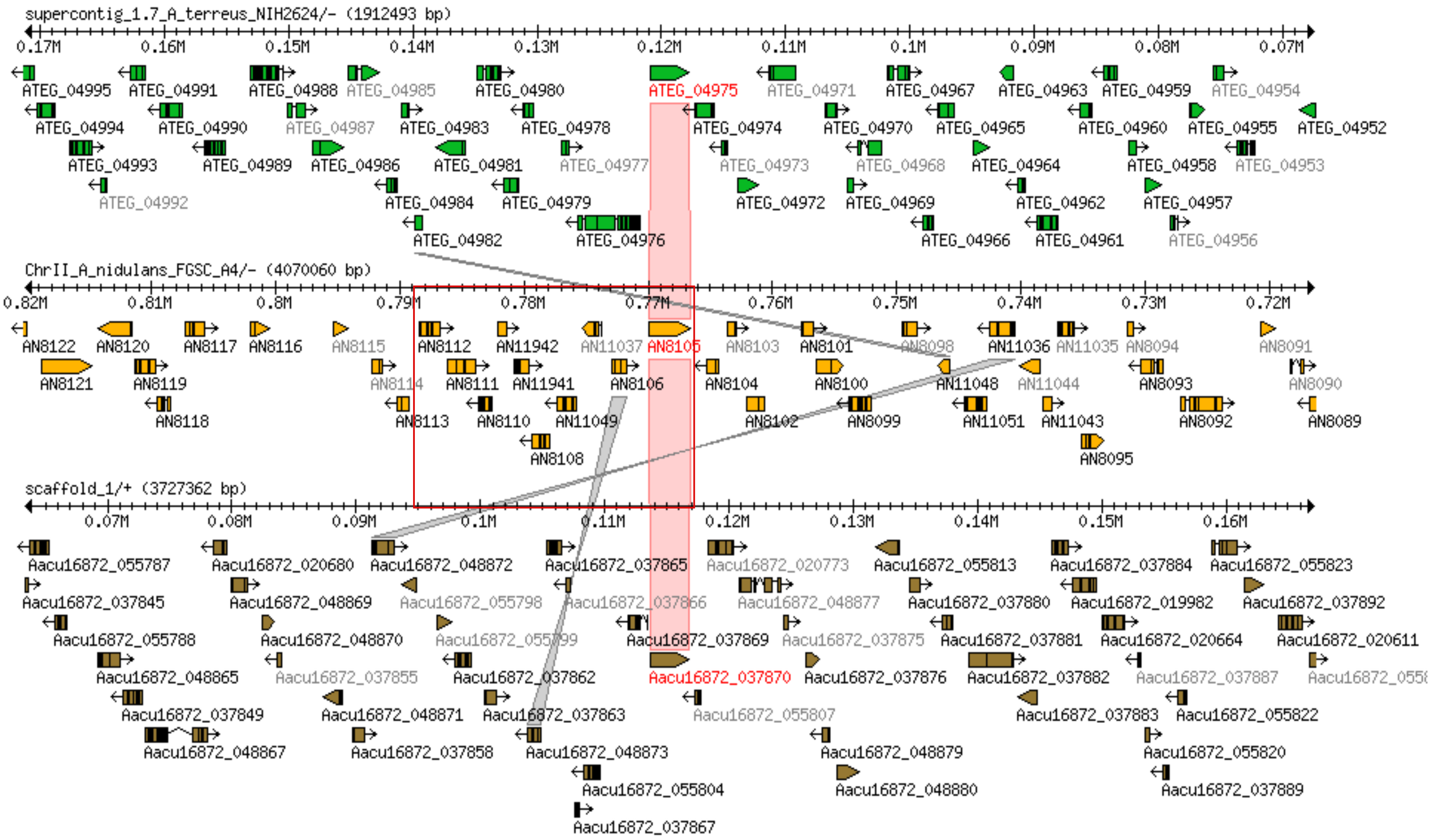

AN8105 cluster

Gene id (SMURF)*	Gene positions	Gene distance	Annotated_gene_function (SMURF)	AspGD gene name	AspGD manual annotation and notes	antiSMASH ID	Gene ID	
n/a					Protein of unknown function	prot_ID_553	AN8098	
n/a					Oxalate decarboxylase	prot_ID_1302	AN8099	
n/a					Solute:hydrogen antiporter	prot_ID_432	AN8100	
n/a					Transcription factor	prot_ID_1178	AN8101	
n/a					Pepsin-like aspartic protease	prot_ID_563	AN8102	
AN8103.3	2	361	conserved hypothetical protein		Zn(II)2Cys6 transcription factor	prot_ID_1315	AN8103	
AN8104.3	1	1361	conserved hypothetical protein		RTA1 domain protein	prot_ID_696	AN8104	
**AN8105.3**	0	0	NRPS-like enzyme		NRPS-like enzyme	**prot_ID_90**	**AN8105**	ECS, EP
AN8106.3	-1	1967	conserved hypothetical protein		Dioxygenase	prot_ID_823	AN8106	
AN11037.3	-2	1133	conserved hypothetical protein		Transmembrane transporter	prot_ID_134	AN11037	
AN11049.3	-3	329	conserved hypothetical protein		Protein of unknown function	prot_ID_391	AN11049	
AN8108.3	-4	806	conserved hypothetical protein		Fumarylacetoacetase	prot_ID_964	AN8108	
AN8109.3	-5	120	conserved hypothetical protein		Deleted ORF			
n/a					L-iditol 2-dehydrogenase	prot_ID_1373	AN11941	
n/a					Predicted ATPase	prot_ID_1385	AN11942	
AN8110.3	-6	247	conserved hypothetical protein		Oxidoreductase	prot_ID_562	AN8110	
AN8111.3	-7	117	conserved hypothetical protein		Zn(II)2Cys6 transcription factor	prot_ID_1314	AN8111	
AN8112.3	-8	457	conserved hypothetical protein		Transmembrane transporter	prot_ID_695	AN8112	EP
AN8113.3	-9	895	conserved hypothetical protein		Oxidoreductase	prot_ID_89	AN8113	
n/a					Protein of unknown function	n/a	AN8114	
n/a					Protein of unknown function	n/a	AN8115	

AN8142 cluster

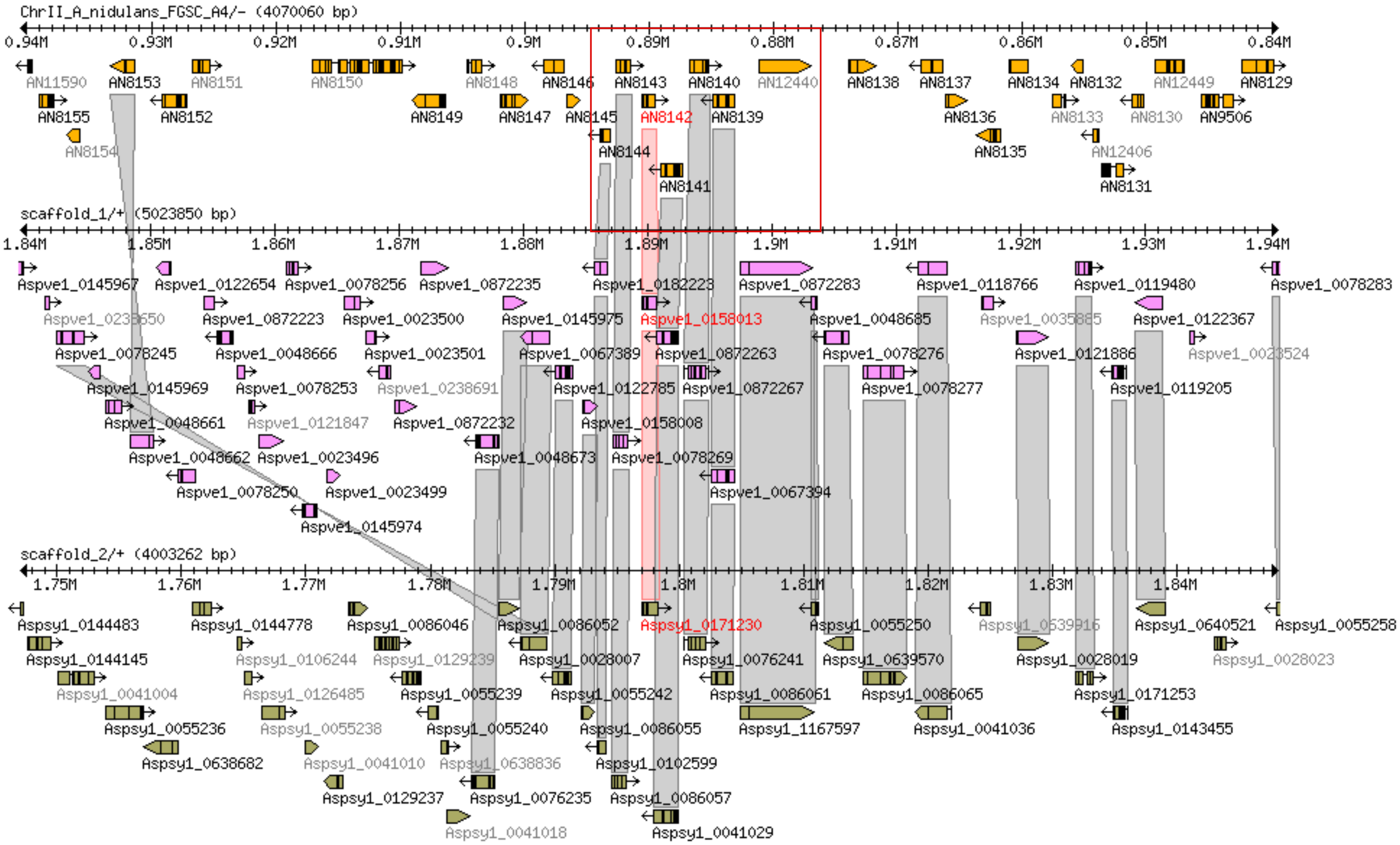

AN8142 cluster

Gene id (SMURF)*	Gene positions	Gene distance	Annotated_gene_function (SMURF)	AspGD gene name	AspGD manual annotation and notes	antiSMASH ID	Gene ID	
n/a					Protein of unknown function	prot_ID_344	AN8133	
n/a					Acyl transferase	prot_ID_1090	AN8134	
n/a					Hexosyl transferase	prot_ID_471	AN8135	
n/a					Protein of unknown function	prot_ID_1219	AN8136	
n/a					Alcohol O-acetyltransferase	prot_ID_600	AN8137	
n/a				aglC	Alpha-galactosidase,	prot_ID_2	AN8138	
n/a					ACP phosphopantetheine attachment site binding, fatty acid biosynthetic process, phosphopantetheine binding, transferase activity	prot_ID_1358	AN12440	FA
n/a				CYP652A1	Cytochrome P450	prot_ID_730	AN8139	
n/a					Monooxygenase	prot_ID_956	AN8140	
n/a				CYP5073A1	Cytochrome P450	prot_ID_342	AN8141	
n/a					UbiA-like prenyltransferase	prot_ID_1089	AN8142	
n/a					Geranylgeranyl pyrophosphate synthetase AtmG	prot_ID_470	AN8143	
n/a					Protein of unknown function	prot_ID_1217	AN8144	ECS
n/a					DUF985 domain protein	prot_ID_599	AN8145	
n/a					Protein of unknown function	prot_ID_1350	AN8146	
n/a					Protein of unknown function	prot_ID_729	AN8147	

AN8209 (wA) cluster

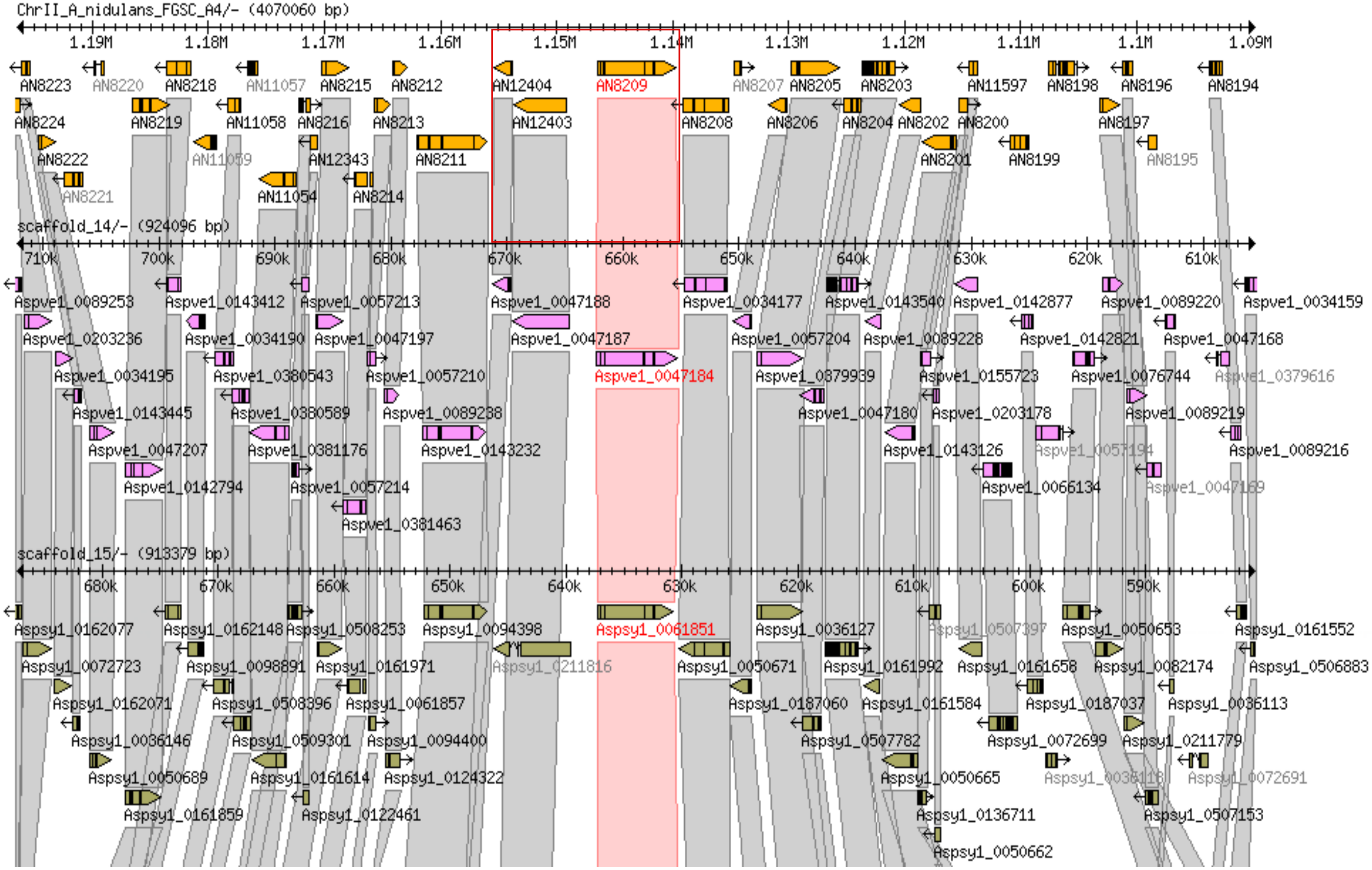

AN8209 cluster

Gene id (SMURF)*	Gene positions	Gene distance	Annotated_gene_function (SMURF)	AspGD gene name	AspGD manual annotation and notes	antiSMASH ID	Gene ID	
n/a					Protein of unknown function	n/a	AN11597	
n/a					Protein of unknown function	n/a	AN8200	
n/a					DNA helicase	n/a	AN8201	
n/a					Protein of unknown function	prot_ID_781	AN8202	
n/a					NAD+ synthase	prot_ID_174	AN8203	
n/a					NAD+ diphosphatase	prot_ID_915	AN8204	
n/a					Protein of unknown function	prot_ID_302	AN8205	
n/a					Protein of unknown function	prot_ID_1050	AN8206	
n/a					Protein of unknown function	prot_ID_430	AN8207	
n/a					Protein of unknown function	prot_ID_1177	AN8208	
n/a				wA	Polyketide synthase, dark green pigment formation	**prot_ID_560**	**AN8209**	ECS, EP
n/a					Deleted; gene split to form AN12403 and AN12404	n/a		
n/a					Protein of unknown function	prot_ID_1360	AN12403	
n/a					Protein of unknown function	prot_ID_1437	AN12404	EP
n/a					Histone demethylase	prot_ID_172	AN8211	
n/a					GIY-YIG catalytic domain containing protein	prot_ID_914	AN8212	
n/a					dTMP kinase	prot_ID_301	AN8213	
n/a					tRNA-guanine transglycosylase family protein	prot_ID_1048	AN8214	

AN8249 cluster

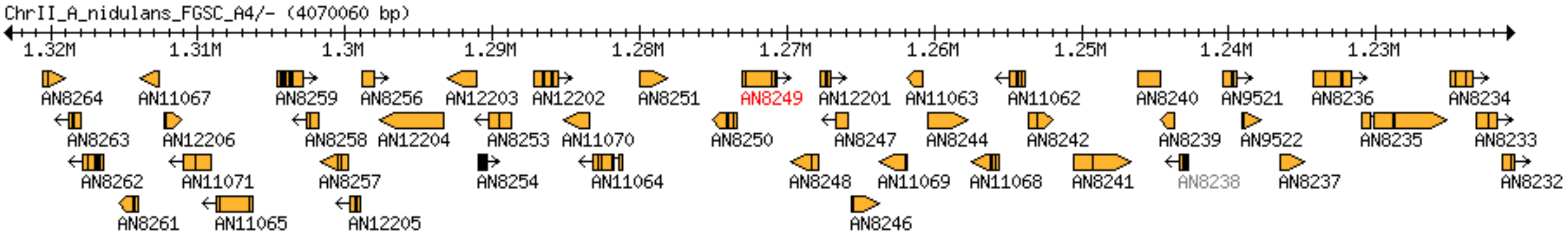

AN8249 cluster

Gene id (SMURF)*	Gene positions	Gene distance	Annotated_gene_function (SMURF)	AspGD gene name	AspGD manual annotation and notes	antiSMASH ID	Gene ID
n/a					Class III chitinase	prot_ID_259	AN11063
n/a				srpA	Signal recognition particle protein	prot_ID_208	AN8246
n/a					DUF1665 domain protein	prot_ID_950	AN8247
n/a					Oxidoreductase	prot_ID_1402	AN12201
n/a					Mitochondrial fusion protein	prot_ID_336	AN8248
n/a					2,3-oxidosqualene-lanosterol cyclase	prot_ID_1083	**AN8249**
n/a				CYP647A1	Cytochrome P450	prot_ID_1299	AN8250
n/a				hapX	bZIP transcription factor	prot_ID_682	AN8251
n/a				agnD	Alpha-1,3-glucanase	prot_ID_1008	AN11064
n/a					Protein of unknown function	prot_ID_653	AN11069

AN8504 cluster

No orthologs for AN8504, used AN8502 to generate cluster image

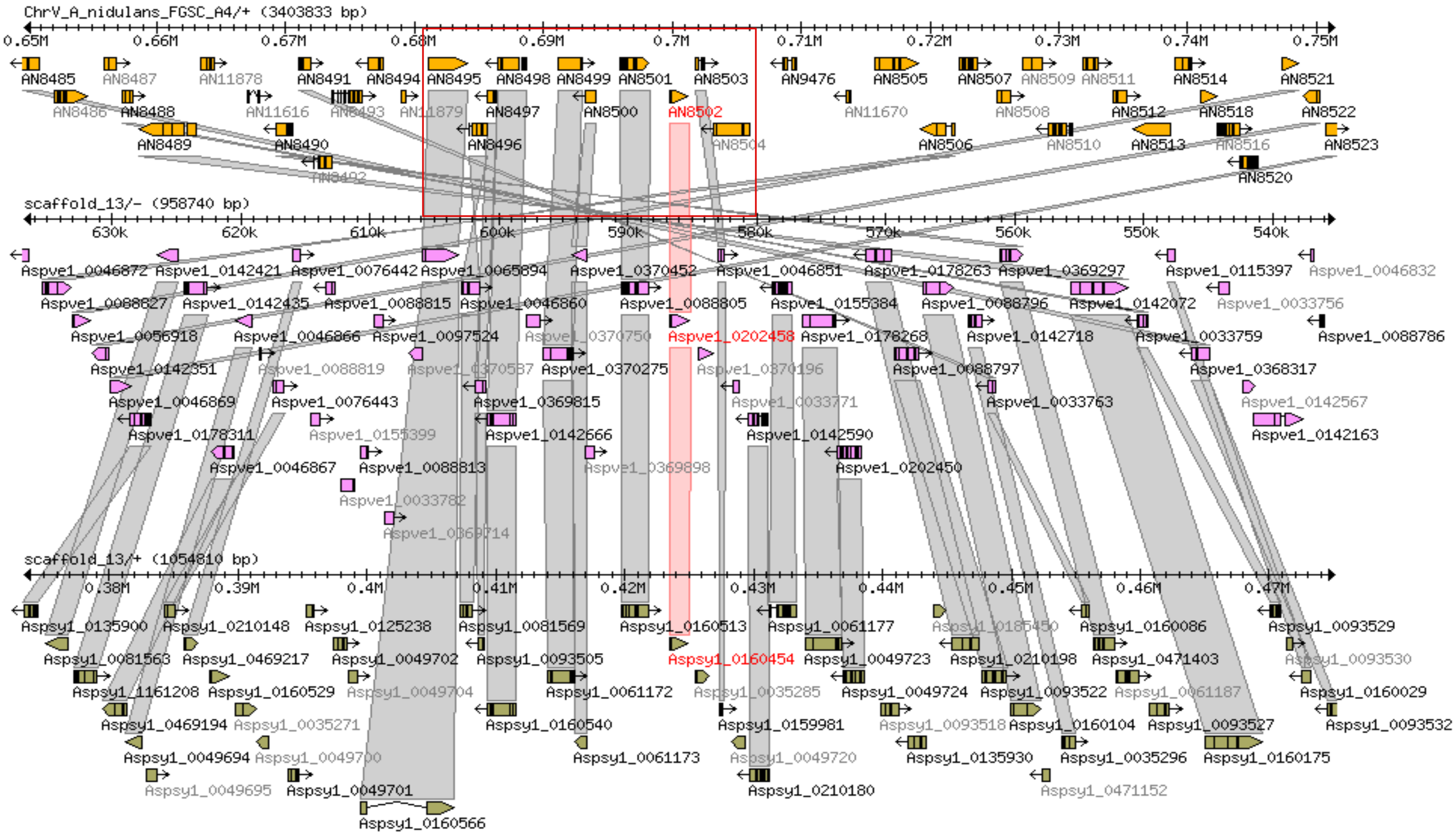

AN8504 cluster

Gene id (SMURF)*	Gene positions	Gene distance	Annotated_gene_function (SMURF)	AspGD gene name	AspGD manual annotation and notes	antiSMASH ID	Gene ID	
n/a					DUF1212 domain membrane protein	prot_ID_432	AN8495	ECS
n/a					Class II aldolase/adducin domain protein	prot_ID_990	AN8496	
n/a					Carbon-sulfur lyase	prot_ID_532	AN8497	
n/a					Proteinase	prot_ID_56	AN8498	
n/a					Siderochrome-iron transporter	prot_ID_628	AN8499	
n/a					Protein of unknown function	prot_ID_1002	AN8500	
n/a					Protein of unknown function	prot_ID_545	AN8501	
n/a					Transmembrane transporter	prot_ID_70	AN8502	
n/a					Protein of unknown function	prot_ID_642	AN8503	
n/a					Nonribosomal peptide synthase	prot_ID_167	**AN8504**	FA
n/a					Protein of unknown function	prot_ID_2	AN9476	
n/a					Protein of unknown function	prot_ID_630	AN11670	
n/a					Protein of unknown function	prot_ID_738	AN8505	
n/a					Zn(II)2Cys6 transcription factor	prot_ID_271	AN8506	
n/a					Transmembrane transporter	prot_ID_838	AN8507	
n/a					Protein of unknown function	prot_ID_374	AN8508	

AN8910 cluster

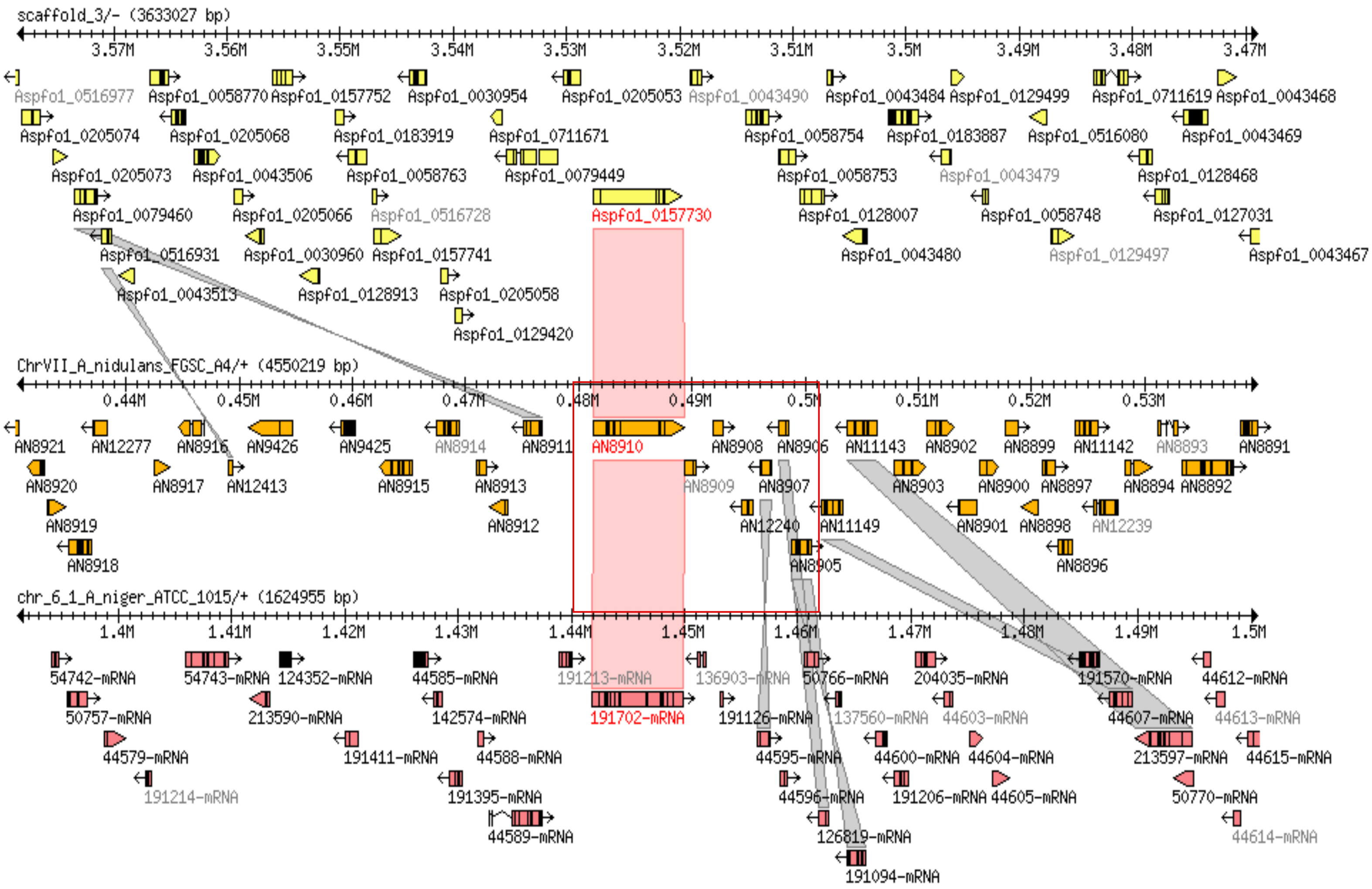

AN8910 cluster

Gene id (SMURF)*	Gene positions	Gene distance	Annotated_gene_function (SMURF)	AspGD gene name	AspGD manual annotation and notes	antiSMASH ID	Gene ID	
AN8901.3	10	316	conserved hypothetical protein			n/a		
AN8902.3	9	428	conserved hypothetical protein			n/a		
AN8903.3	8	2432	conserved hypothetical protein		Peptide transporter	prot_ID_353	AN8903	
AN11143.3	7	363	conserved hypothetical protein	glaA	Glucoamylase	prot_ID_246	AN11143	
AN11149.3	6	868	conserved hypothetical protein		Arylsulfatase	prot_ID_679	AN11149	
AN8905.3	5	285	conserved hypothetical protein		Cytochrome P450	prot_ID_497	AN8905	ECS
AN8906.3	4	502	conserved hypothetical protein		Protein of unknown function	prot_ID_1313	AN8906	
AN8907.3	3	3264	conserved hypothetical protein		C-4 sterol methyl oxidase	prot_ID_640	AN8907	
n/a					Protein of unknown function	prot_ID_1507	AN12240	
AN8908.3	2	1360	conserved hypothetical protein		Protein of unknown function	prot_ID_1455	AN8908	
AN8909.3	1	338	conserved hypothetical protein		Protein of unknown function	prot_ID_788	AN8909	
**AN8910.3**	0	0	polyketide synthase		polyketide synthase	**prot_ID_1021**	**AN8910**	ECS
n/a					Transmembrane transporter	prot_ID_350	AN8911	
n/a					Myo-inositol transporter	prot_ID_1164	AN8912	
n/a					Glycosylphosphatidylinositol (GPI)-anchored protein	prot_ID_494	AN8913	
n/a					Protein of unknown function	prot_ID_1310	AN8914	
n/a					Transmembrane transporter	prot_ID_637	AN8915	

AN9005 cluster

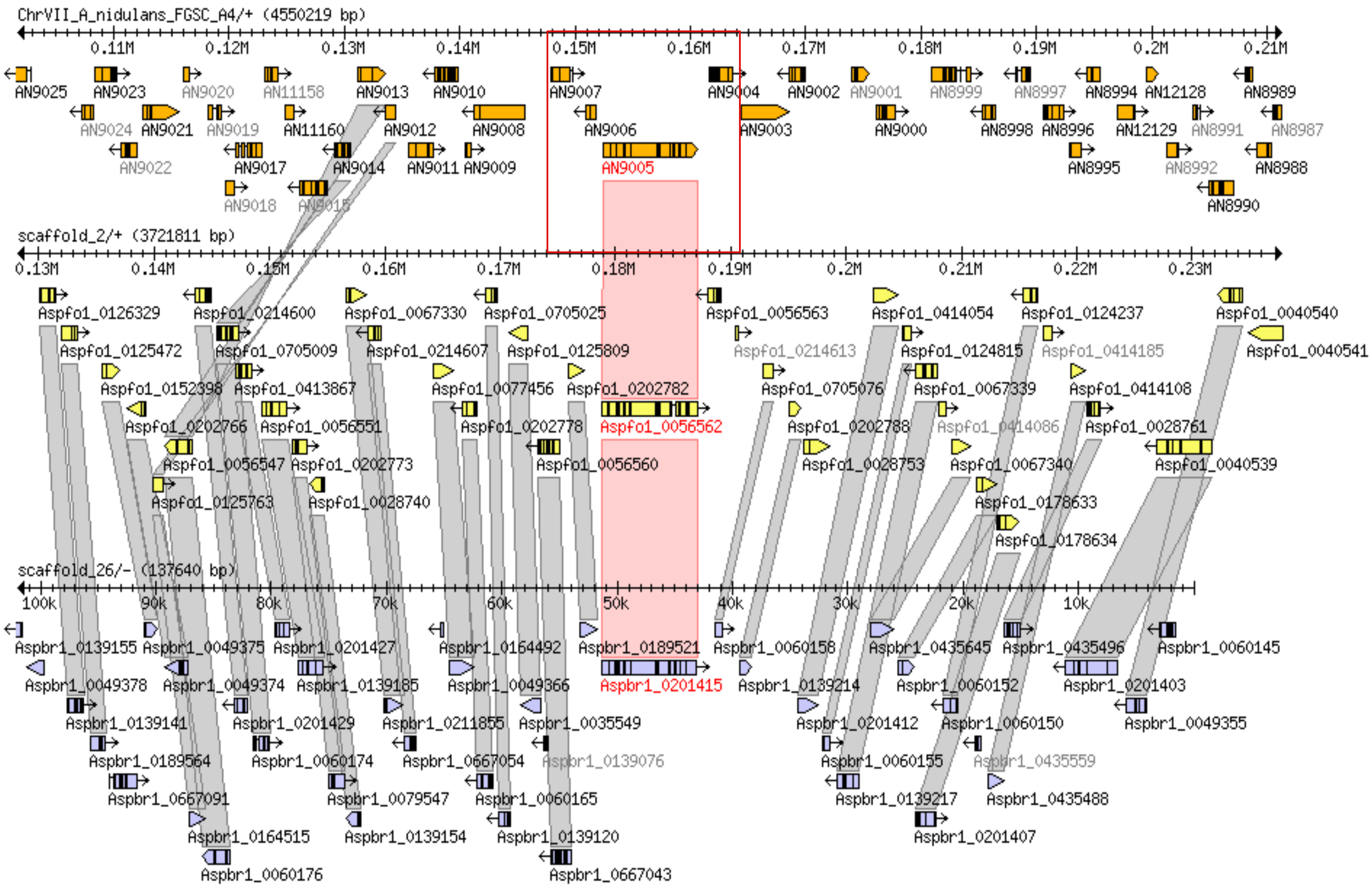

AN9005 cluster

Gene id (SMURF)*	Gene positions	Gene distance	Annotated_gene_function (SMURF)	AspGD gene name	AspGD manual annotation and notes	antiSMASH ID	Gene ID	
n/a					Transmembrane transporter	prot_ID_591	AN9000	
n/a					Protein of unknown function	prot_ID_1408	AN9001	
AN9002.3	3	10	conserved hypothetical protein		Oxidoreductase	prot_ID_737	AN9002	
AN9003.3	2	737	conserved hypothetical protein		Aspartic-type endopeptidase	prot_ID_67	AN9003	
AN9004.3	1	1173	conserved hypothetical protein		Monooxygenase	prot_ID_883	AN9004	EP
**AN9005.3**	0	0	polyketide synthase		Polyketide synthase	prot_ID_208	AN9005	
AN9006.3	-1	739	conserved hypothetical protein		Protein of unknown function	prot_ID_1028	AN9006	
AN9007.3	-2	1425	conserved hypothetical protein	CYP548D1	Cytochrome P450	prot_ID_358	AN9007	EP
AN9008.3	-3	2562	hypothetical protein similar to phytochrome	fphA	Phytochrome photoreceptor	prot_ID_1170	AN9008	
AN9009.3	-4	67	conserved hypothetical protein		Protein of unknown function	prot_ID_503	AN9009	
AN9010.3	-5	665	hypothetical protein		Nicotinamide mononucleotide transmembrane transporter	prot_ID_736	AN9010	
AN9011.3	-6	125	conserved hypothetical protein		Aryl-alcohol oxidase-related protein	prot_ID_66	AN9011	
AN9012.3	-7	1297	conserved hypothetical protein		Lyase	prot_ID_881	AN9012	
AN9013.3	-8	175	conserved hypothetical protein		Zn(II)2Cys6 transcription factor	prot_ID_206	AN9013	

AN9129 Cluster

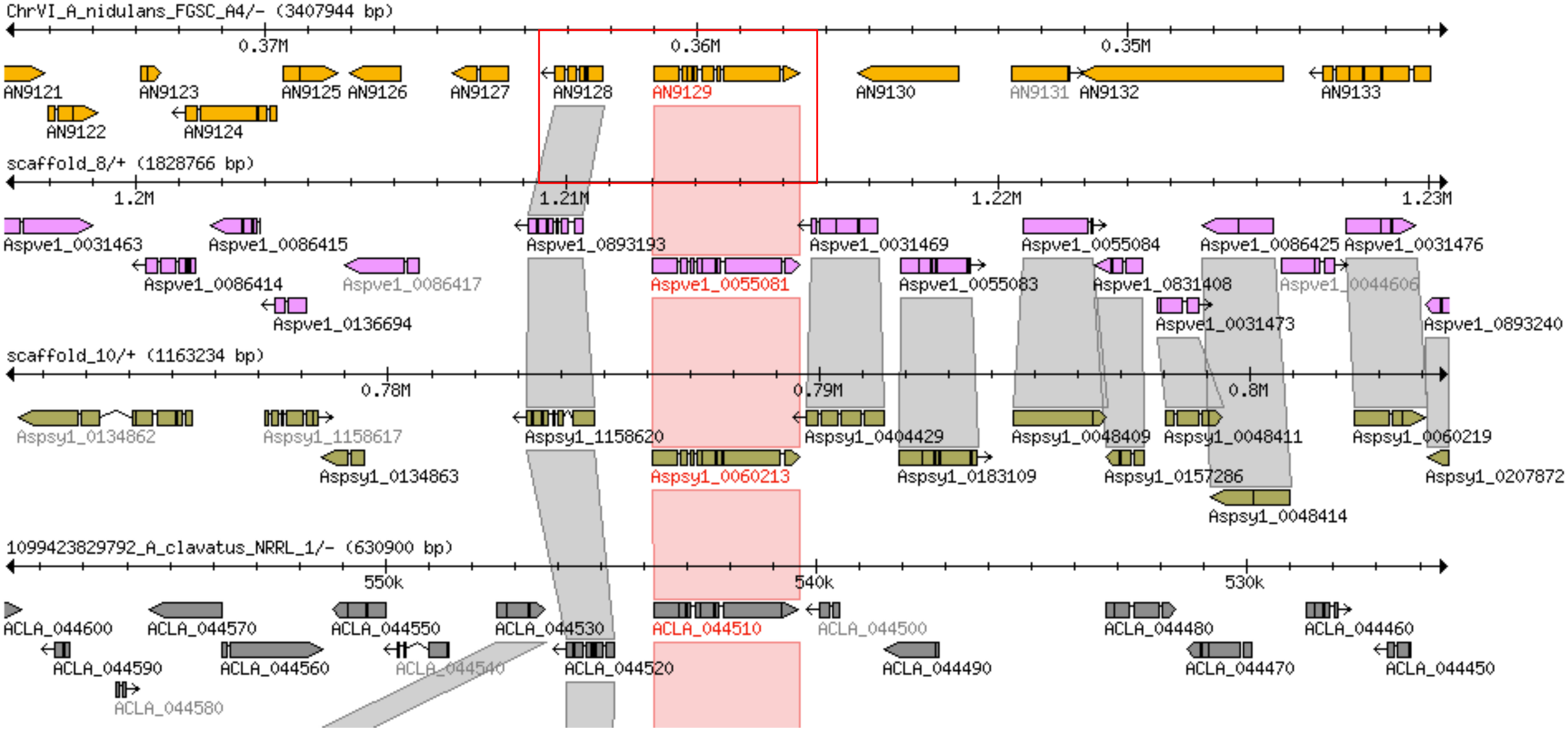

AN9129 cluster

Gene id (SMURF)*	Gene positions	Gene distance	Annotated_gene_function (SMURF)	AspGD gene name	AspGD manual annotation and notes	antiSMASH ID	Gene ID	
n/a					NRPS-like enzyme	n/a	AN9129	ECS, EP
n/a					enoyl-CoA hydratase/isomerase family protein	n/a	AN9130	ECS

AN9226 cluster

Possible overlapping clusters, AN9226 lacks orthologs so AN9227 was used to generate cluster image

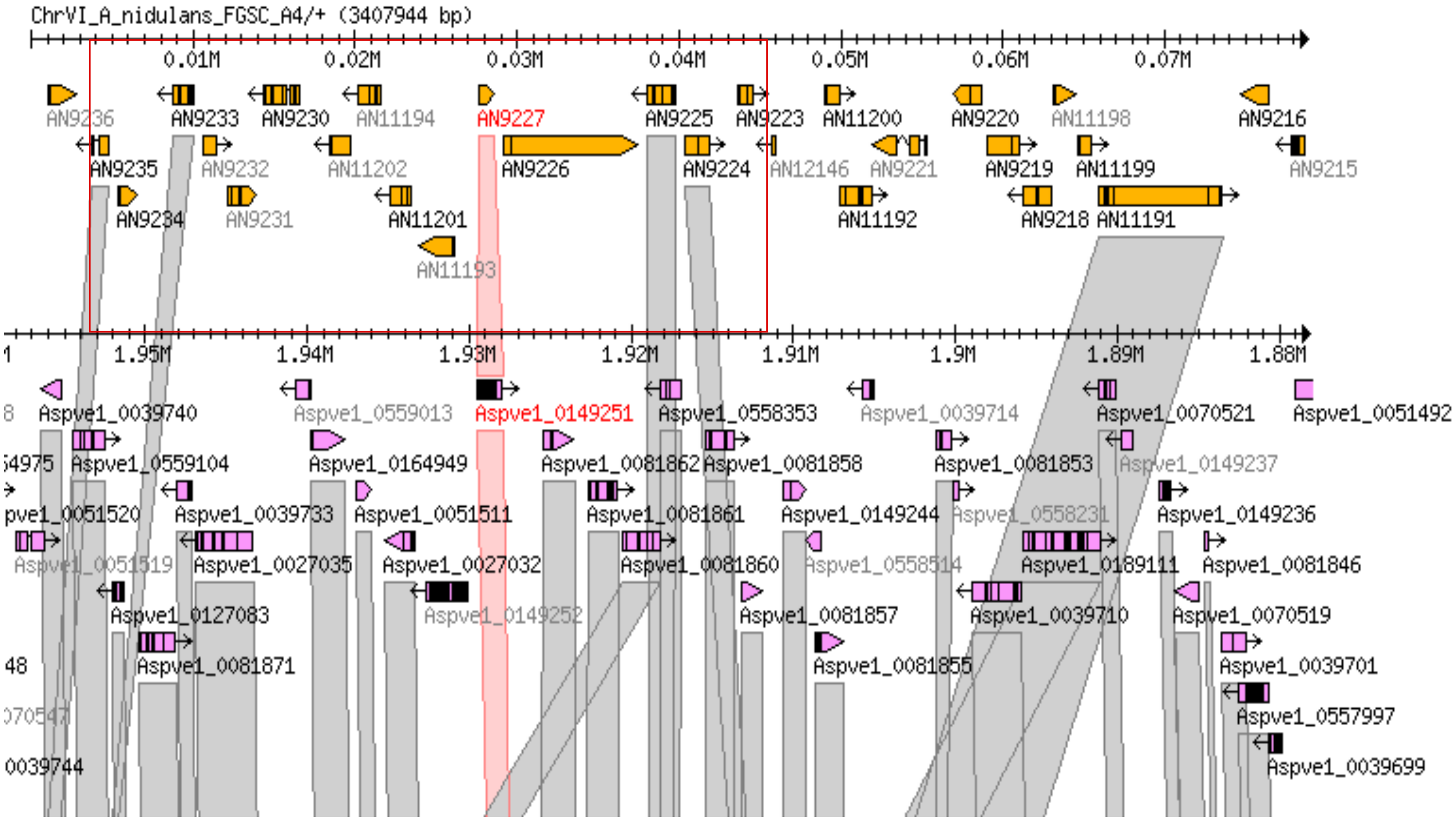

AN9226 cluster

Gene id (SMURF)*	Gene positions	Gene distance	Annotated_gene_function (SMURF)	AspGD gene name	AspGD manual annotation and notes	antiSMASH ID	Gene ID	
					Protein of unknown function	n/a	AN9215	
n/a					Protein of unknown function	n/a	AN9216	
n/a					polyketide synthase	n/a	AN11191	
n/a					Dihydrofolate reductase	n/a	AN11199	
n/a					Oxidoreductase	n/a	AN11198	
n/a					Sterol 14-demethylase	n/a	AN9218	
n/a					Transmembrane transporter	n/a	AN9219	
n/a					Protein of unknown function	prot_ID_1014	AN9220	
n/a					Zn(II)2Cys6 transcription factor	prot_ID_511	AN9221	
n/a				CYP664A1	Cytochrome P450	prot_ID_108	AN11192	
n/a					Protein of unknown function	prot_ID_329	AN11200	
n/a					Protein of unknown function	prot_ID_1129	AN12146	
AN9223.3	3	1736	conserved hypothetical protein		Demethylsterigmatocystin 6-O-methyltransferase	prot_ID_620	AN9223	EP
AN9224.3	2	633	conserved hypothetical protein		Monooxygenase	prot_ID_133	AN9224	
AN9225.3	1	563	conserved hypothetical protein	CYP679A1	Cytochrome P450	prot_ID_726	AN9225	
**AN9226.3**	0	0	nonribosomal peptide synthase		Nonribosomal peptide synthase	**prot_ID_231**	**AN9226**	
AN9227.3	-1	1045	conserved hypothetical protein		Dioxygenase	prot_ID_832	AN9227	
AN11193.3	-2	616	conserved hypothetical protein		Domain(s) with predicted oxygen transporter activity and role in transport	prot_ID_698	AN11193	
AN11201.3	-3	429	conserved hypothetical protein		Oxidoreductase	prot_ID_933	AN11201	
AN11194.3	-4	611	DMATS type aromatic prenyltransferase		DMATS-type aromatic prenyltransferase	prot_ID_210	AN11194	
AN11202.3	-5	363	DMATS type aromatic prenyltransferase		DMATS-type aromatic prenyltransferase	prot_ID_438	AN11202	
AN9230.3	-6	1866	conserved hypothetical protein		Monooxygenase	prot_ID_18	AN9230	
AN9231.3	-7	208	conserved hypothetical protein		UDP-N-acetylmuramate dehydrogenase	prot_ID_617	AN9231	
AN9232.3	-8	666	conserved hypothetical protein		Oxidoreductase	prot_ID_129	AN9232	

AN9233.3	-9	406	conserved hypothetical protein	O-methyltransferase	prot_ID_724	AN9233	
AN9234.3	-10	2320	conserved hypothetical protein	Oxidoreductase	prot_ID_227	AN9234	EP
AN9235.3	-11	638	conserved hypothetical protein	Isochorismatase family hydrolase	prot_ID_830	AN9235	
AN9236.3	-12	895	conserved hypothetical protein	Zn(II)2Cys6 transcription factor	prot_ID_336	AN9236	

AN9291 (AN11820) cluster

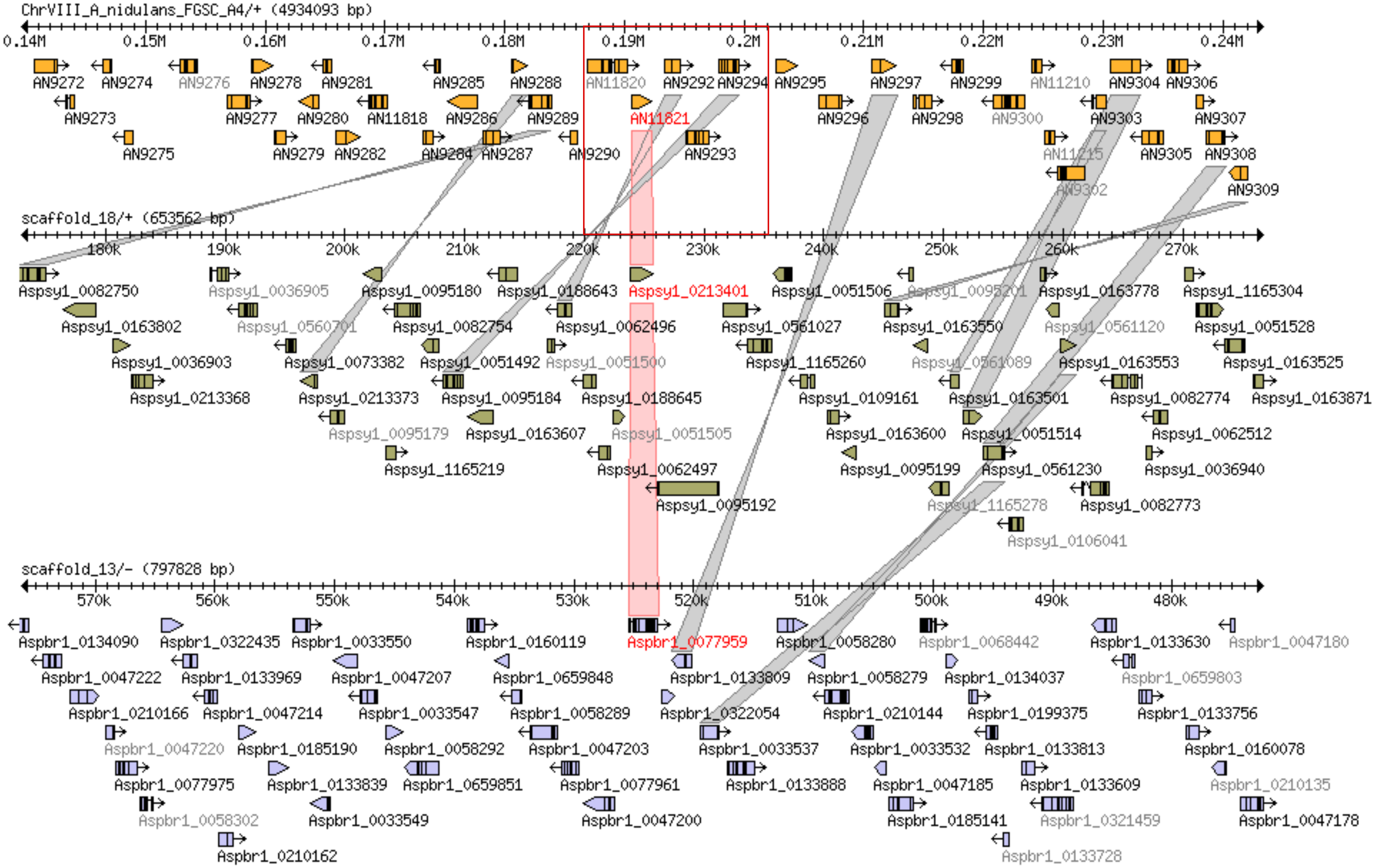

AN9291 cluster

Gene id (SMURF)*	Gene positions	Gene distance	Annotated_gene_function (SMURF)	AspGD gene name	AspGD manual annotation and notes	antiSMASH ID	Gene ID	
AN9280.3	11	354	conserved hypothetical protein		Transmembrane transporter	n/a	AN9280	
AN9281.3	10	364	conserved hypothetical protein		Protein of unknown function	n/a	AN9281	
AN9282.3	9	738	conserved hypothetical protein		N,N-dimethylaniline monooxygenase	n/a	AN9282	
AN9283.3	8	320	conserved hypothetical protein		Possible pseudogene	n/a	AN9283	
AN9284.3	7	130	conserved hypothetical protein		Oxidoreductase	n/a	AN9284	
AN9285.3	6	577	conserved hypothetical protein		Glucose repressible protein ortholog Grg1	n/a	AN9285	
AN9286.3	5	433	conserved hypothetical protein		Alpha-glucuronidase	n/a	AN9286	
AN9287.3	4	949	conserved hypothetical protein		Extracellular GDSL-like lipase/acylhydrolase	n/a	AN9287	
AN9288.3	3	137	conserved hypothetical protein		Protein of unknown function	n/a	AN9288	
AN9289.3	2	1689	conserved hypothetical protein		Transmembrane transporter	n/a	AN9289	
AN9290.3	1	855	conserved hypothetical protein		Protein of unknown function	n/a	AN9290	
**AN9291.3**	0	0	MFS transporter/NRPS-like enzyme		Deleted ORF, see AN11820	n/a	AN9291	
					Nonribosomal peptide sythetase (NRPS)-like enzyme	n/a	**AN11820**	EP
					Transmembrane transporter	n/a	AN11821	
AN9292.3	-1	1168	conserved hypothetical protein		Zn(II)2Cys6 transcription factor	n/a	AN9292	
AN9293.3	-2	435	conserved hypothetical protein		Zn(II)2Cys6 transcription factor	n/a	AN9293	
AN9294.3	-3	830	hypothetical protein		Domain(s) with predicted role in transmembrane transport	n/a	AN9294	ECS
AN9295.3	-4	2999	conserved hypothetical protein		Domain(s) with predicted role in transmembrane transport	n/a	AN9295	
AN9296.3	-5	1950	conserved hypothetical protein	CYP535D1	Cytochrome P450	n/a	AN9296	
AN9297.3	-6	2446	conserved hypothetical protein		Protein of unknown function	n/a	AN9297	
AN9298.3	-7	1797	conserved hypothetical	gmtB	GDP-mannose transporter	n/a	AN9298	

			protein			
AN9299.3	-8	1891	conserved hypothetical protein	Protein of unknown function	n/a	AN9299
AN9300.3	-9	2743	conserved hypothetical protein	Amino acid transmembrane transporter	n/a	AN9300
AN11210.3	-10	953	conserved hypothetical protein	Protein of unknown function	n/a	AN11210
AN11215.3	-11	613	conserved hypothetical protein	Domain(s) with predicted metal ion transmembrane transporter activity	n/a	AN11215
AN9302.3	-12	87	conserved hypothetical protein	Protein serine/threonine kinase	n/a	AN9302
AN9303.3	-13	637	conserved hypothetical protein	Protein of unknown function	n/a	AN9303
AN9304.3	-14	2063	conserved hypothetical protein	Glutathione S-transferase	n/a	AN9304
AN9305.3	-15	214	conserved hypothetical protein	Acyl transferase	n/a	AN9305
AN9306.3	-16	654	hypothetical protein	Protein of unknown function	n/a	AN9306
AN9307.3	-17	427	conserved hypothetical protein	Protein of unknown function	n/a	AN9307

AN9314 cluster

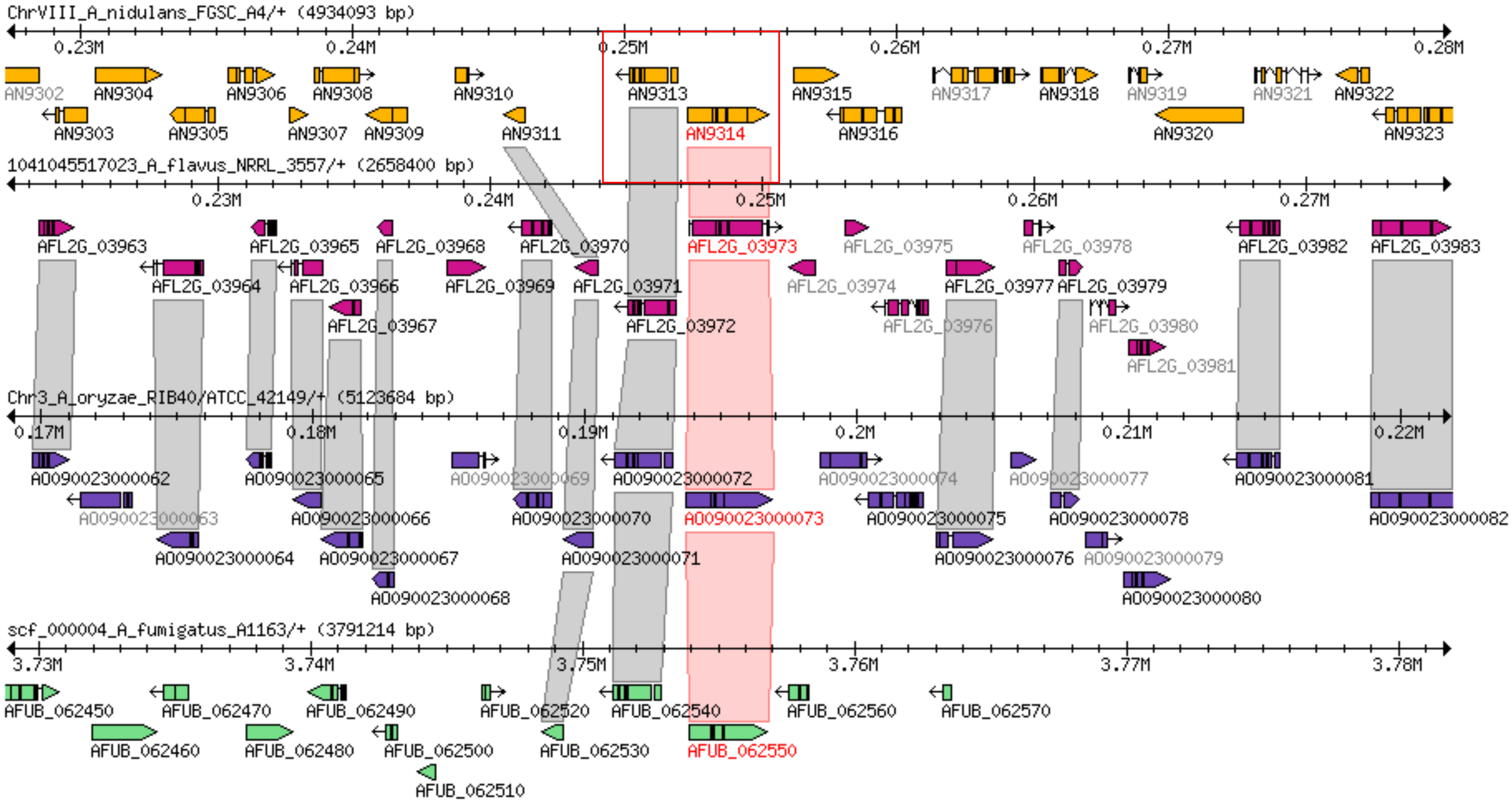

AN9314 cluster

Gene id (SMURF)*	Gene positions	Gene distance	Annotated_gene_function (SMURF)	AspGD gene name	AspGD manual annotation and notes	antiSMASH ID	Gene ID	
n/a					Protein of unknown function	n/a	AN9311	
n/a				*CYP58D1*	Cytochrome P450	n/a	AN9313	EP
n/a					Entkaurene synthase	n/a	**AN9314**	EP

No PKS/NRPS backbone 1

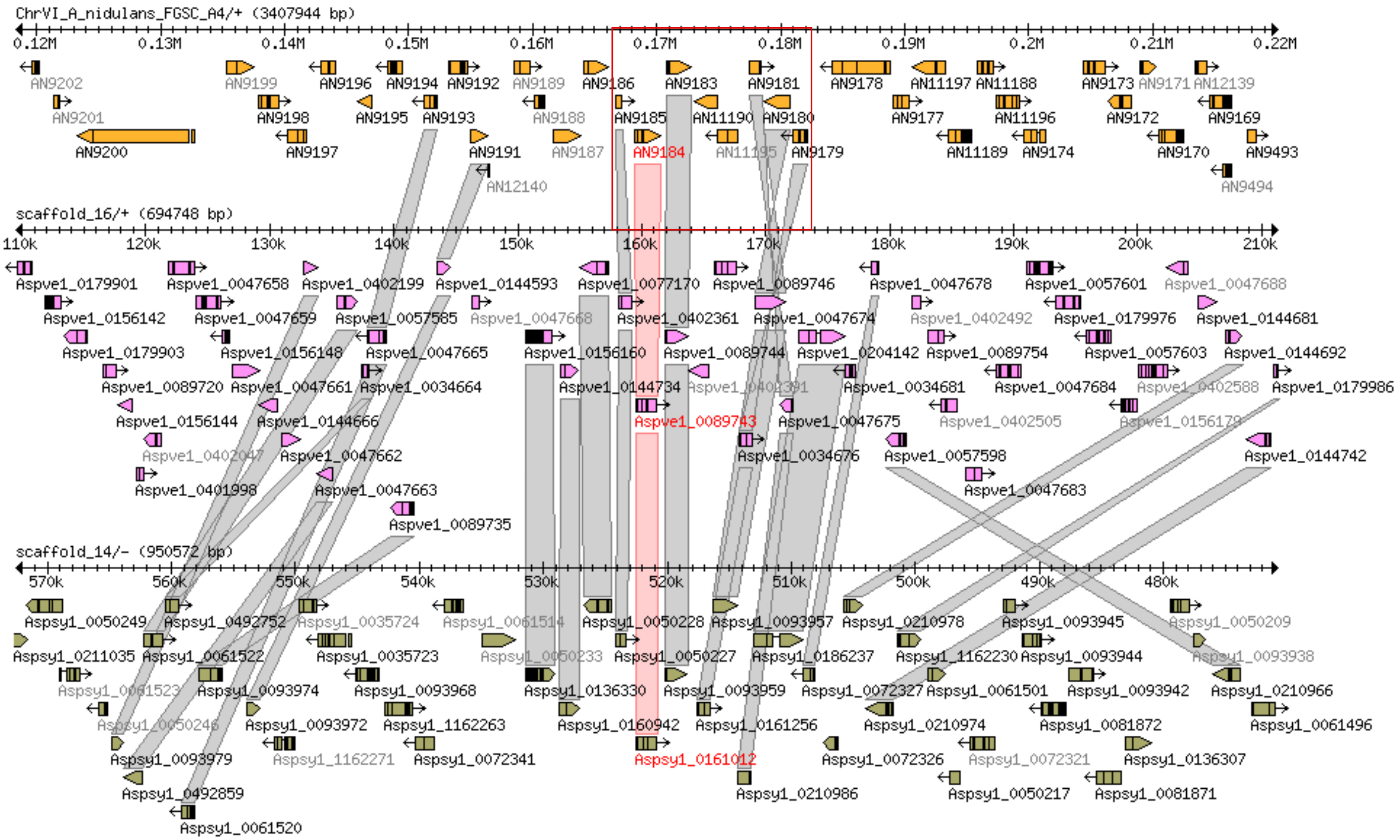

No PKS/NRPS backbone 1

Gene id (SMURF)*	Gene positions	Gene distance	Annotated_gene_function (SMURF)	AspGD gene name	AspGD manual annotation and notes	antiSMASH ID	Gene ID	
n/a					Pyridoxal reductase	n/a	AN9179	ECS
n/a					Transketolase	n/a	AN9180	
n/a					Protein of unknown function	n/a	AN9181	
n/a					C2H2 and Zn(2)-Cys(6) transcription factor	n/a	AN11195	
n/a					Hydrolase	n/a	AN11190	
n/a					Beta-glucosidase	n/a	AN9183	
n/a					Transmembrane transporter	prot_ID_567	AN9184	
n/a					Protein of unknown function	prot_ID_80	AN9185	ECS
n/a					Acid phosphatase	prot_ID_677	AN9186	
n/a					F-box protein	prot_ID_184	AN9187	
n/a					Protein of unknown function	prot_ID_783	AN9188	
n/a					Protein of unknown function	prot_ID_287	AN9189	
n/a					Protein of unknown function	prot_ID_1133	AN12140	
n/a					Protein of unknown function	prot_ID_1075	AN9191	
n/a					Protein of unknown function	prot_ID_564	AN9192	
n/a					Protein of unknown function	prot_ID_78	AN9193	
n/a				cetL	Protein of unknown function	prot_ID_675	AN9194	

No PKS/NRPS backbone 2
No manual prediction made

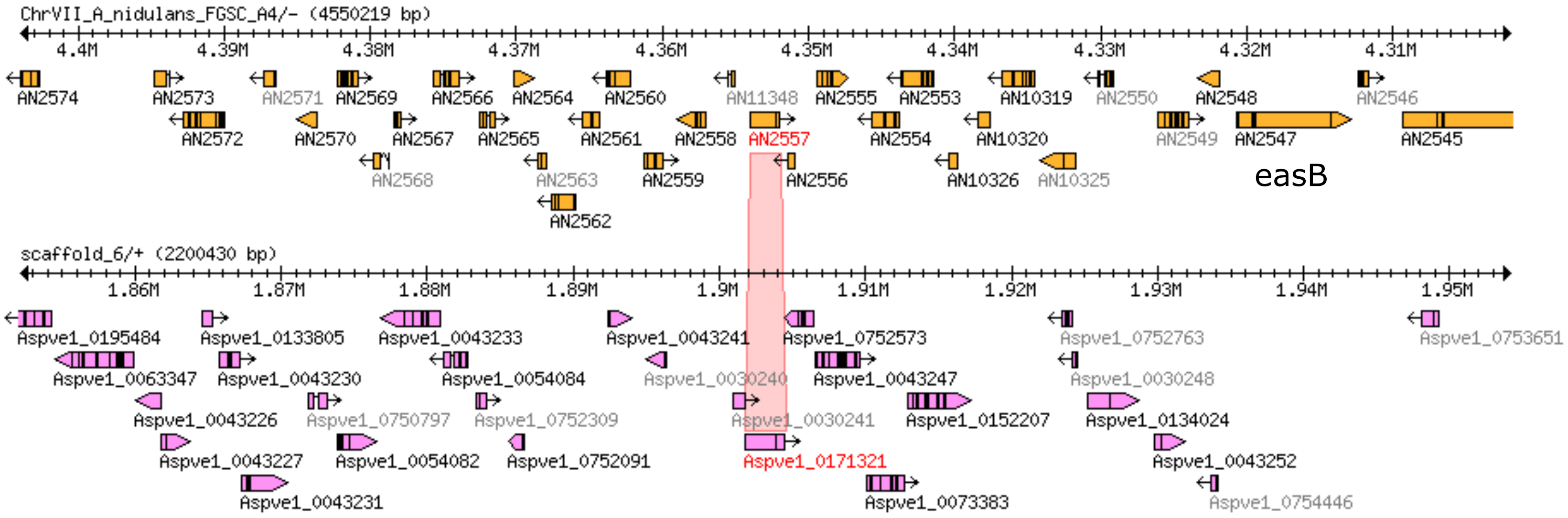

No PKS/NRPS backbone 2

Gene id (SMURF)*	Gene positions	Gene distance	Annotated_gene_function (SMURF)	AspGD gene name	AspGD manual annotation and notes	antiSMASH ID	Gene ID
n/a					Tyrosine kinase	prot_ID_212	AN10325
n/a					Transmembrane transporter	prot_ID_365	AN10319
n/a					Protein of unknown function	prot_ID_597	AN10320
n/a					Protein of unknown function	prot_ID_1033	AN10326
n/a					Protein of unknown function	prot_ID_979	AN2553
n/a					Protein of unknown function	prot_ID_303	AN2554
n/a					Serine carboxypeptidase	prot_ID_1119	AN2555
n/a					Protein of unknown function	prot_ID_451	AN2556
n/a					Methyltransferase	**prot_ID_1259**	**AN2557**
n/a					Protein of unknown function	prot_ID_330	AN11348

No PKS/NRPS backbone 3

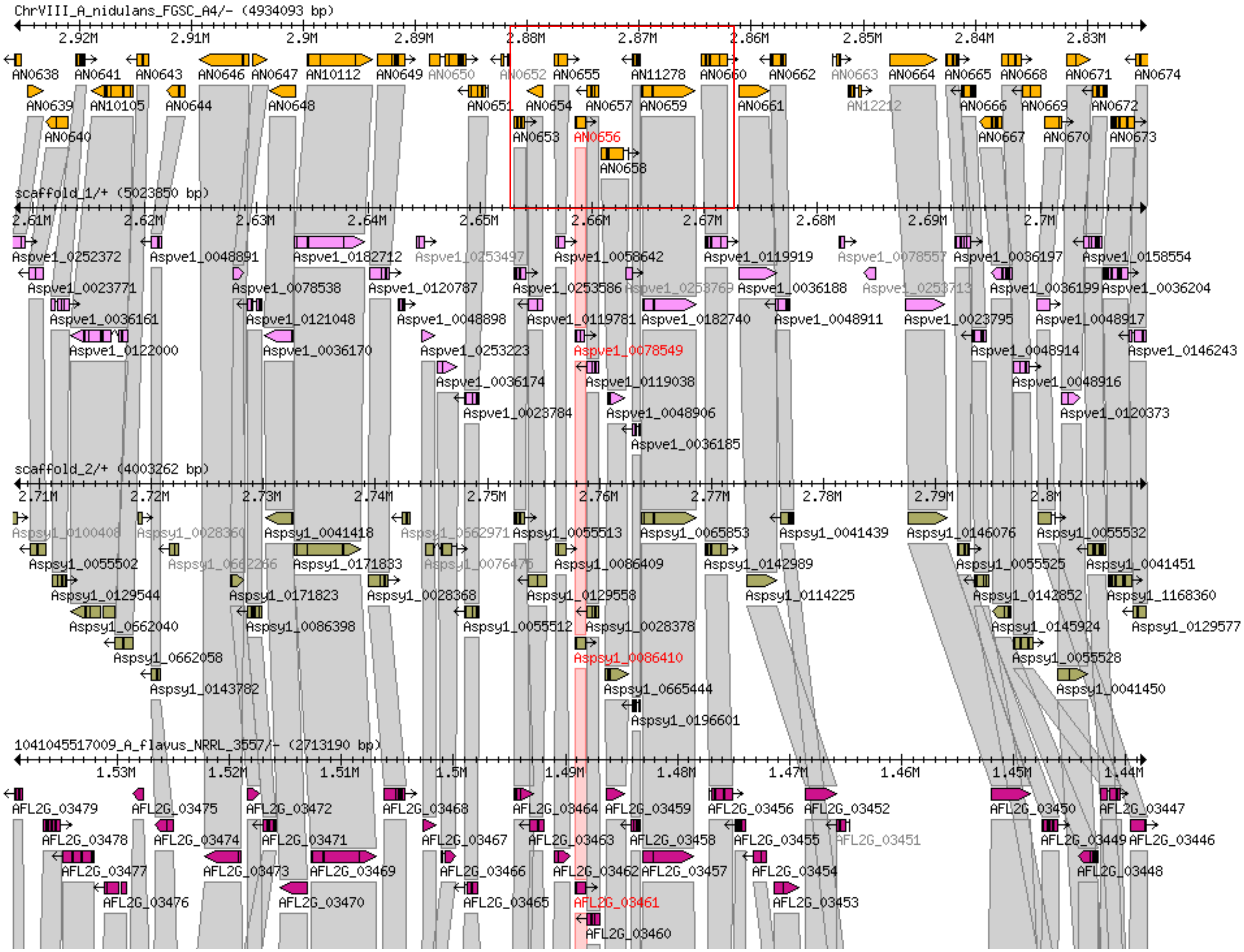

No PKS/NRPS backbone 3

Gene id (SMURF)*	Gene positions	Gene distance	Annotated_gene_function (SMURF)	AspGD gene name	AspGD manual annotation and notes	antiSMASH ID	Gene ID	
n/a					Protein of unknown function	prot_ID_1442	AN0650	
n/a				fadA	Alpha subunit of a heterotrimeric G protein	prot_ID_212	AN0651	
n/a					Protein of unknown function	prot_ID_666	AN0652	
n/a					Zn(II)2Cys6 transcription factor	prot_ID_1130	AN0653	ECS
n/a					Ortholog(s) have farnesyltranstransferase activity, role in terpenoid biosynthetic process and mitochondrion localization	prot_ID_1593	AN0654	
n/a				sepM	serine carboxypeptidase	prot_ID_368	AN0655	
n/a					Has methylase domain involved in ubiquinone/menaquinone biosynthesis	**prot_ID_824**	**AN0656**	
n/a					Mitochondrial outer membrane protein	prot_ID_1299	AN0657	
n/a					Copper-activated transcription factor	prot_ID_73	AN0658	
n/a					Ortholog(s) have role in cristae formation and integral to mitochondrial inner membrane, mitochondrial crista junction localization	prot_ID_1139	AN11278	
n/a					Protein of unknown function	prot_ID_526	AN0659	
n/a					Allantoin transporter; transcript is induced by allantoin and by uric acid and is repressed by ammonium	n/a	AN0660	ECS

No PKS/NRPS backbone 4

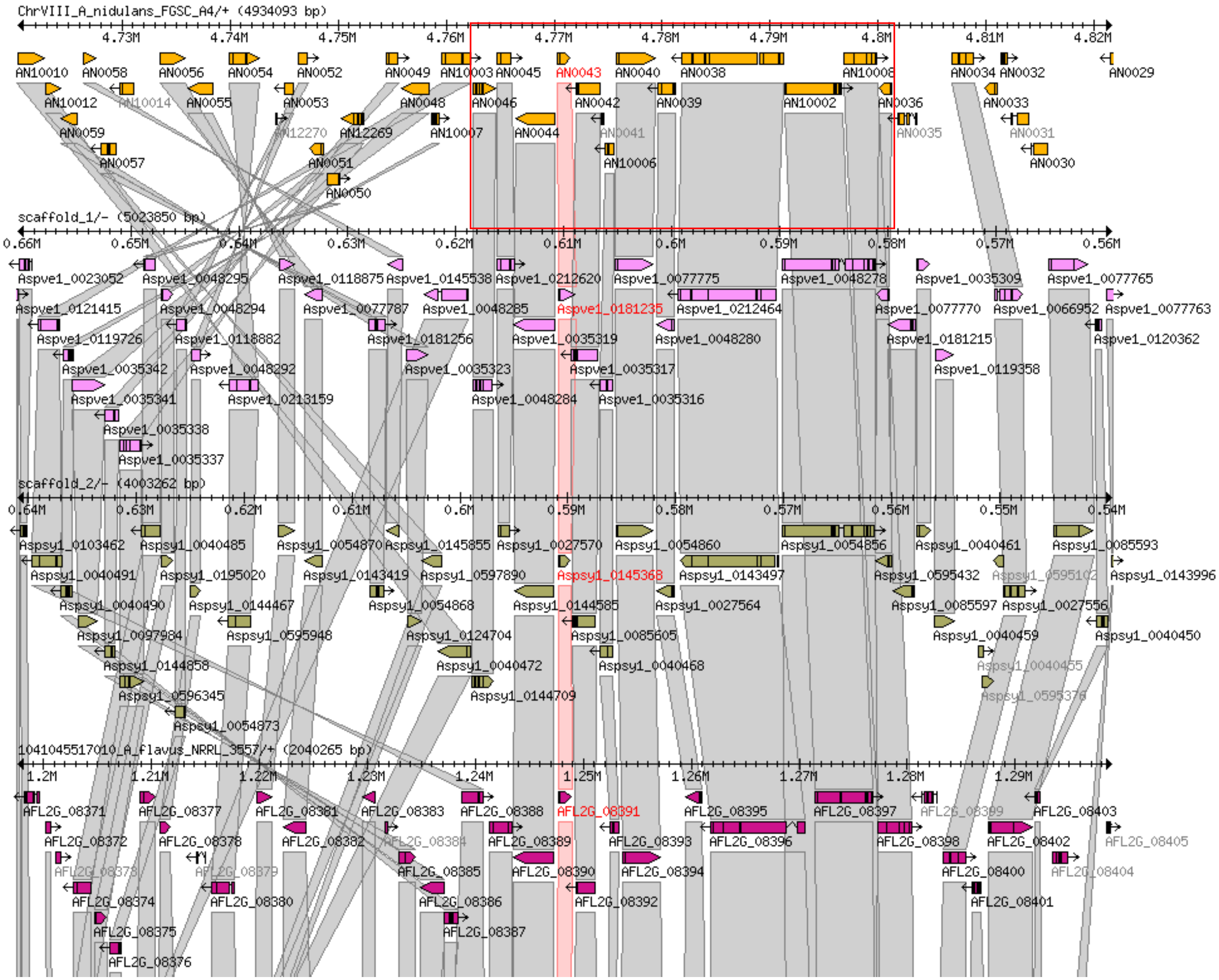

No PKS/NRPS backbone 4

Gene id (SMURF)*	Gene positions	Gene distance	Annotated_gene_function (SMURF)	AspGD gene name	AspGD manual annotation and notes	antiSMASH ID	Gene ID	
n/a					Protein of unknown function	n/a	AN0036	
n/a				culD	Ubiquitin protein ligase	n/a	AN10008	
n/a				nup192	Nuclear pore complex protein	n/a	AN10002	
n/a				atmA	1-phosphatidylinositol-3-kinase	n/a	AN0038	
n/a					Protein of unknown function	prot_ID_1262	AN0039	
n/a					DNA-directed DNA polymerase	prot_ID_539	AN0040	
n/a					Protein of unknown function	prot_ID_1004	AN0041	
n/a					Transmembrane transporter	prot_ID_1477	AN0042	EP
n/a					Transferase	**prot_ID_252**	**AN0043**	
n/a					Single-stranded DNA-dependent ATPase	prot_ID_705	AN0044	EP
n/a					Protein of unknown function	prot_ID_1175	AN0045	
n/a					Histidyl-tRNA synthetase	prot_ID_1632	AN0046	
n/a					GTPase	prot_ID_1168	AN10003	
n/a					Domain(s) with predicted zinc ion binding activity	prot_ID_858	AN10006	
