# Supplementary material for: Comprehensive annotation of secondary metabolite biosynthetic genes and gene clusters of Aspergillus nidulans, A. fumigatus, A. niger and A. oryzae
Source: BMC Microbiol. 2013 Apr 26;13:91. doi: 10.1186/1471-2180-13-91 (PMC3689640; doi:10.1186/1471-2180-13-91)
Supplement: Additional file 3 — Contains a table listing manually annotated gene clusters predicted by SMURF and antiSMASH for A. fumigatus. [file 1471-2180-13-91-S3.pdf]

Additional file 3

Comprehensive annotation of secondary metabolite biosynthetic genes and gene clusters of *Aspergillus nidulans*, *A. fumigatus*, *A. niger* and *A. oryzae*

Diane O. Inglis, Jon Binkley, Marek Skrzypek, Martha B. Arnaud, Gustavo Cerqueira, Prach Shah, Farrell Wymore, Jennifer R. Wortman and Gavin Sherlock

Abbreviations: ECS, end of cluster synteny; IGD, increase in intergenic distance; FA, change in functional annotation; ED, experimentally determined

Fumigaclavine C (fga) cluster
Experimentally determined cluster

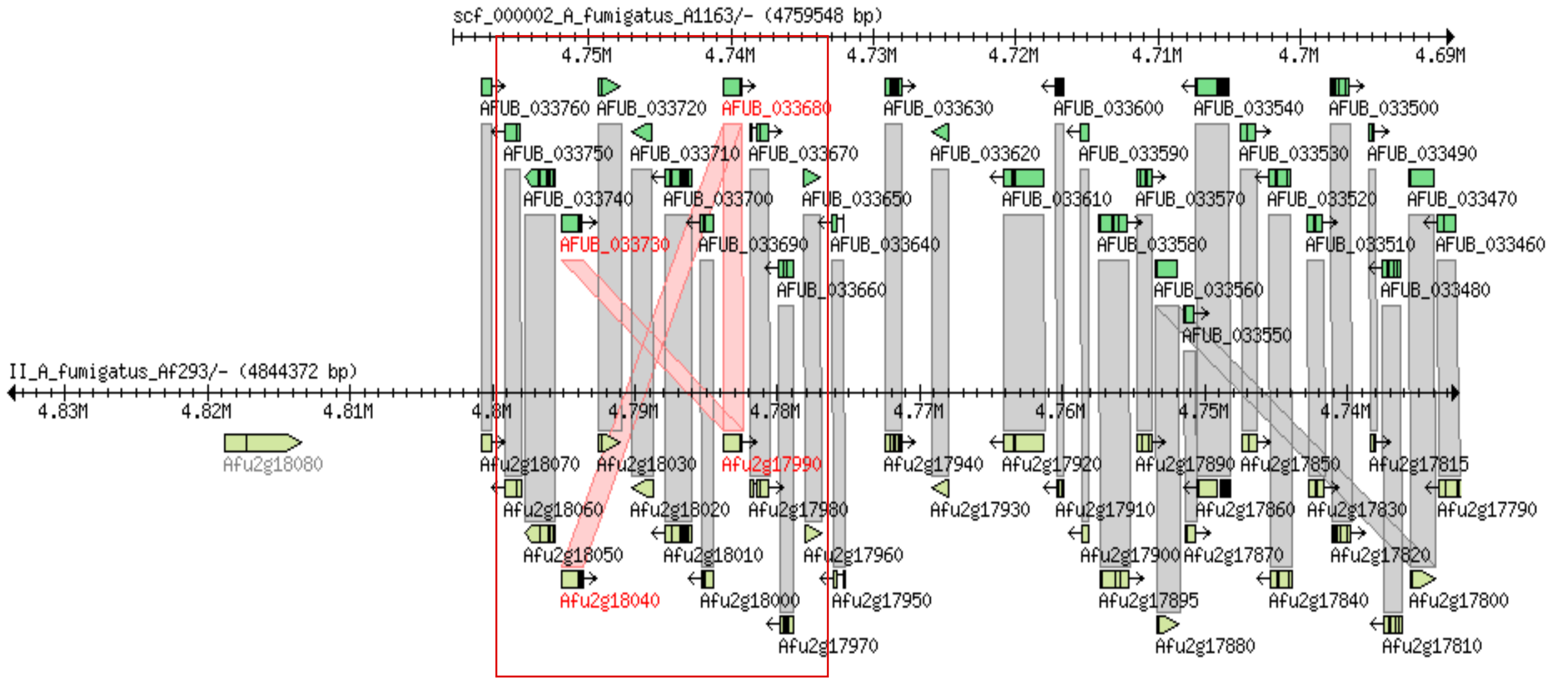

Fumigaclavine C (fga) cluster

Gene id (SMURF)	Gene positions	Gene distance	Annotated_gene_function (SMURF)	AspGD gene name	AspGD supplementary annotation	antiSMASH ID	Gene ID (I)	Gene ID (II)	
AFUA_2G17930	11	2110	integral membrane protein			n/a	Afu2g17930	AFUA_2G17930	
AFUA_2G17940	10	2876	MAK1-like monooxygenase, putative			n/a	Afu2g17940	AFUA_2G17940	
AFUA_2G17950	9	842	conserved hypothetical protein			prot_ID_1588	Afu2g17950	AFUA_2G17950	
AFUA_2G17960	8	788	NADPH dehydrogenase Oye3, putative	fgaOn/a3/easA		prot_ID_1589	Afu2g17960	AFUA_2G17960	ED
AFUA_2G17970	7	706	NmrA-like ergot alkaloid biosynthetic protein A	fgaFS/easG		prot_ID_1590	Afu2g17970	AFUA_2G17970	
AFUA_2G17980	6	530	cytochrome P450 monooxygenase, putative	easK/fgaP450-1		prot_ID_1591	Afu2g17980	AFUA_2G17980	
AFUA_2G17990	5	730	dimethylallyl tryptophan synthase FgaPT1	fgaPT1/easL		**prot_ID_1592**	**Afu2g17990**	**AFUA_2G17990**	
AFUA_2G18000	4	653	short chain dehydrogenase/oxidoreductase Cpox2	fgaDH/easD		prot_ID_1593	Afu2g18000	AFUA_2G18000	
AFUA_2G18010	3	848	cytochrome P450 monooxygenase, putative	easM		prot_ID_1594	Afu2g18010	AFUA_2G18010	
AFUA_2G18020	2	677	O-acetyltransferase, putative	fgaAT/easN		prot_ID_1595	Afu2g18020	AFUA_2G18020	
AFUA_2G18030	1	1073	catalase Cat	fgaCat/easC		prot_ID_1596	Afu2g18030	AFUA_2G18030	
**AFUA_2G18040**	0	0	dimethylallyl tryptophan synthase FgaPT2	dmaW/FgaPT2		**prot_ID_1597**	**Afu2g18040**	**AFUA_2G18040**	
AFUA_2G18050	-1	501	FAD binding oxidoreductase Cpox1	fgaOn/a1/easE		prot_ID_1598	Afu2g18050	AFUA_2G18050	
AFUA_2G18060	-2	257	AdoMet:dimethylallyltryptophan N-methyltransferase EasF	fgaMT/easF		prot_ID_1599	Afu2g18060	AFUA_2G18060	ED
AFUA_2G18070	-3	1001	metalloproteinase, putative			prot_ID_1600	Afu2g18070	AFUA_2G18070	

Fumitremorgin B (ftm) cluster
Experimentally determined cluster

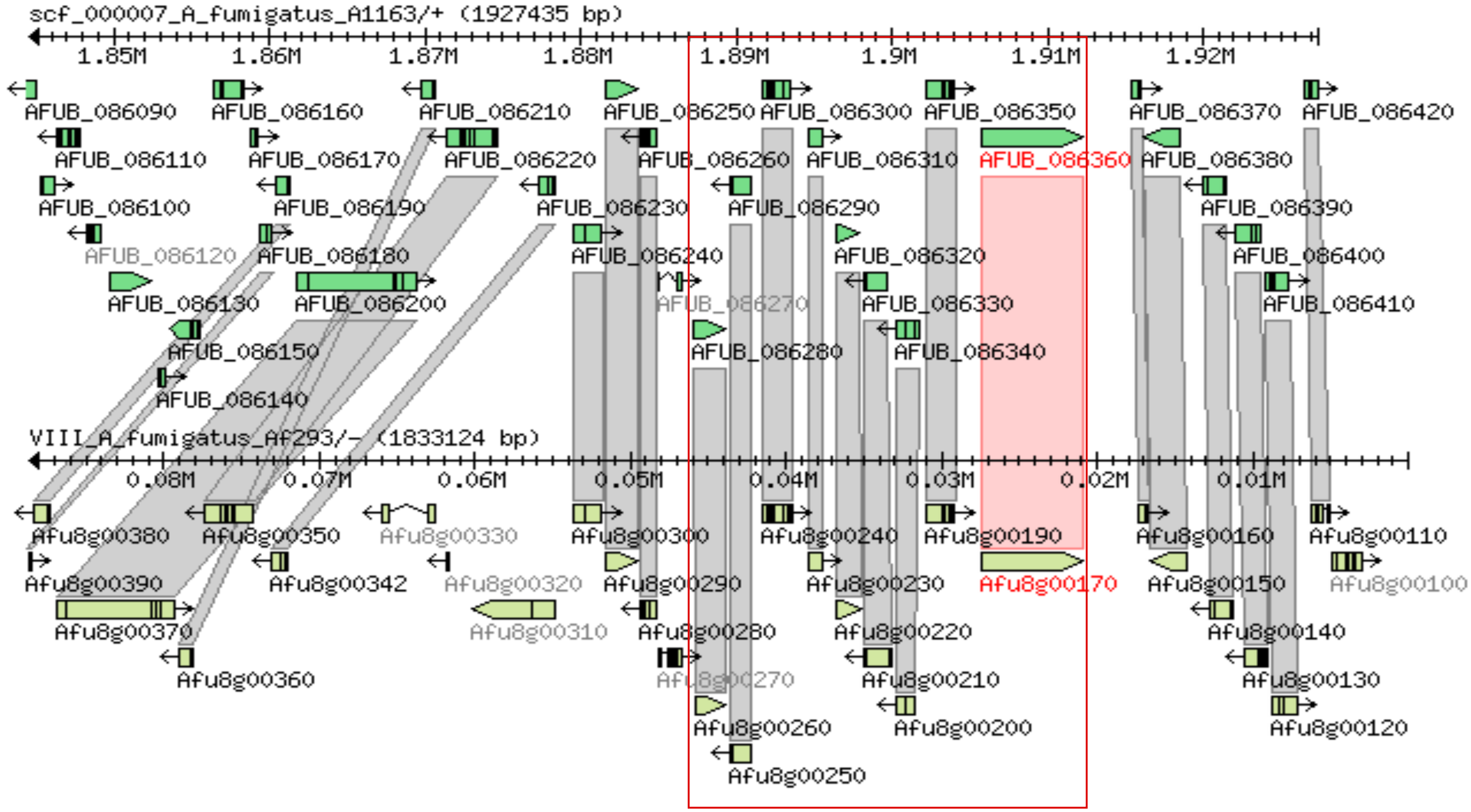

Fumitremorgin B (ftm) cluster

Gene id (SMURF)		Gene distance	Annotated_gene_function (SMURF)	AspGD gene name	AspGD supplementary annotation	antiSMASH ID	Gene ID (I)	Gene ID (II)	
					Aspartate-tRNA ligase	prot_ID_1	Afu8g00100	AFUA_8G00100	
					Oxidoreductase	prot_ID_2	Afu8g00110	AFUA_8G00110	
n/a					Protein of unknown function	prot_ID_20	Afu8g00300	AFUA_8G00300	
n/a					Protein of unknown function	prot_ID_19	Afu8g00290	AFUA_8G00290	
AFUA_8G00280	10	88	short-chain dehydrogenase, putative			prot_ID_18	Afu8g00280	AFUA_8G00280	
AFUA_8G00270	9	790	conserved hypothetical protein			prot_ID_17	Afu8g00270	AFUA_8G00270	
AFUA_8G00260	8	305	F-box domain and ankyrin repeat protein	ftmI		prot_ID_16	Afu8g00260	AFUA_8G00260	ED
**AFUA_8G00250**	7	728	dimethylallyl tryptophan synthase, putative	ftmPT2		prot_ID_15	Afu8g00250	AFUA_8G00250	
AFUA_8G00240	6	1114	cytochrome P450 monooxygenase, putative	ftmG		prot_ID_14	Afu8g00240	AFUA_8G00240	
AFUA_8G00230	5	877	phytanoyl-CoA dioxygenase family protein	ftmF		prot_ID_13	Afu8g00230	AFUA_8G00230	
AFUA_8G00220	4	181	cytochrome P450, putative	ftmE		prot_ID_12	Afu8g00220	AFUA_8G00220	
AFUA_8G00210	3	400	dimethylallyl tryptophan synthase FtmPT1	ftmPT1		**prot_ID_11**	**Afu8g00210**	**AFUA_8G00210**	
AFUA_8G00200	2	751	O-methyltransferase, putative	ftmD		prot_ID_10	Afu8g00200	AFUA_8G00200	
AFUA_8G00190	1	1544	cytochrome P450, putative	ftmC		prot_ID_9	Afu8g00190	AFUA_8G00190	
AFUA_8G00170	0	0	nonribosomal brevianamide peptide synthase FtmA	ftmA		**prot_ID_8**	**Afu8g00170**	**AFUA_8G00170**	ED
AFUA_8G00160	-1	3508	conserved hypothetical protein			prot_ID_7	Afu8g00160	AFUA_8G00160	
AFUA_8G00150	-2	82	conserved hypothetical protein			prot_ID_6	Afu8g00150	AFUA_8G00150	
AFUA_8G00140	-3	1488	MFS transporter, putative			prot_ID_5	Afu8g00140	AFUA_8G00140	
AFUA_8G00130	-4	648	hypothetical protein			prot_ID_4	Afu8g00130	AFUA_8G00130	
AFUA_8G00120	-5	371	hypothetical protein			prot_ID_3	Afu8g00120	AFUA_8G00120	
AFUA_8G00110	-6	970	oxidoreductase, 2OG-Fe(II) oxygenase family, putative			prot_ID_21	Afu8g00110	AFUA_8G00110	
AFUA_8G00100	-7	201	aspartate-tRNA ligase, putative			n/a	Afu8g00100	AFUA_8G00100	

Gliotoxin (gli) cluster

Experimentally determined cluster

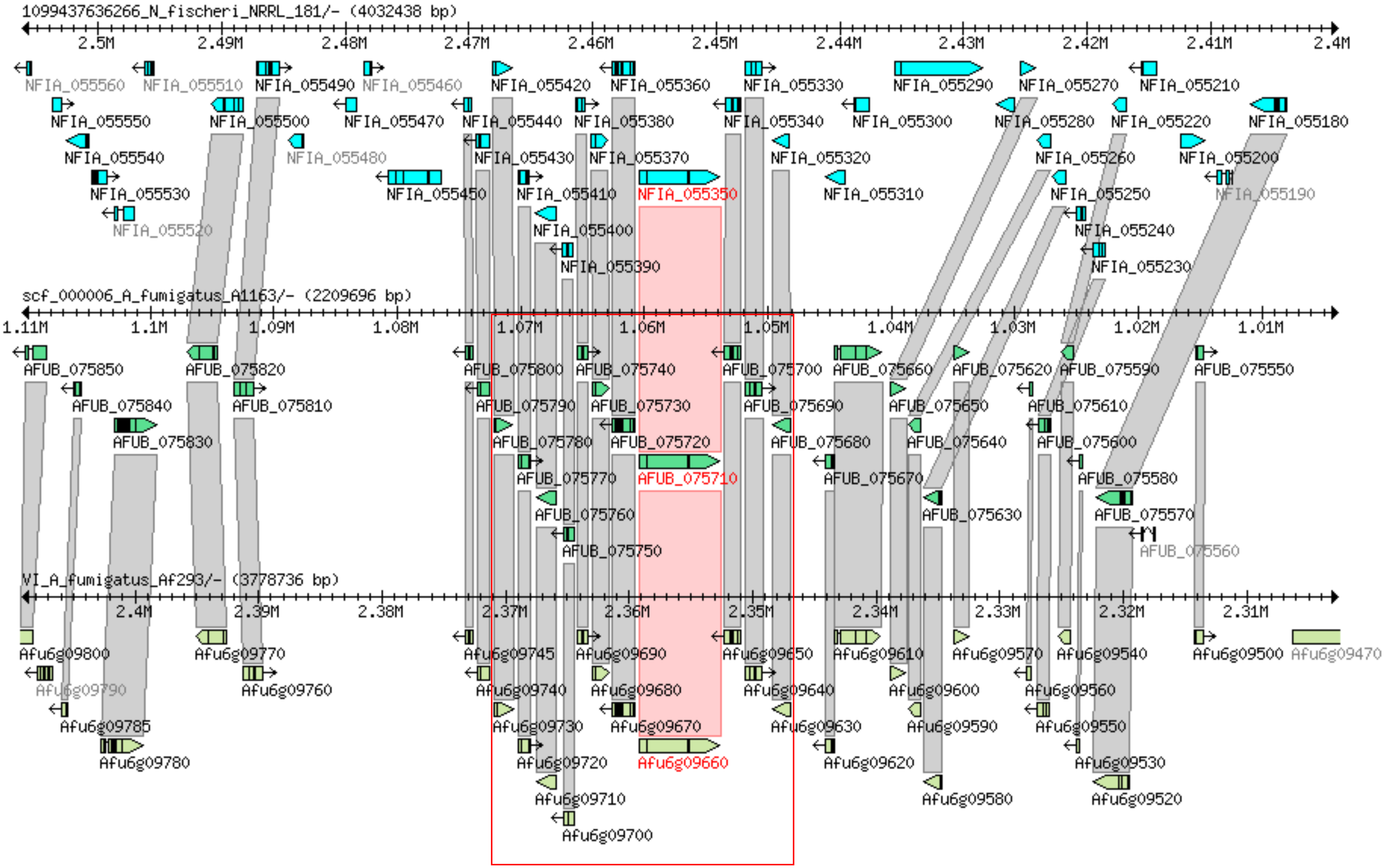

Gliotoxin (gli) cluster

Gene id (SMURF)	Gene positions	Gene distance	Annotated_gene_function (SMURF)	AspGD gene name	AspGD supplementary annotation	antiSMASH ID	Gene ID (I)	Gene ID (II)	
n/a					Putative acetyl hydrolase	prot_ID_722	Afu6g09520	AFUA_6G09520	
n/a					Protein of unknown function	prot_ID_723	Afu6g09530	AFUA_6G09530	
n/a					Endo-1,4 beta-glucanase	prot_ID_724	Afu6g09540	AFUA_6G09540	
n/a					RTA1 domain protein	prot_ID_725	Afu6g09550	AFUA_6G09550	
n/a					Protein of unknown function	prot_ID_726	Afu6g09560	AFUA_6G09560	
n/a					Protein of unknown function	prot_ID_727	Afu6g09570	AFUA_6G09570	
AFUA_6G09580	8	220	C6 finger domain protein, putative			prot_ID_728	Afu6g09580	AFUA_6G09580	
AFUA_6G09590	7	84	zinc alcohol dehydrogenase, putative			prot_ID_729	Afu6g09590	AFUA_6G09590	
AFUA_6G09600	6	712	zinc metallopeptidase, putative			prot_ID_730	Afu6g09600	AFUA_6G09600	
AFUA_6G09610	5	41	nonribosomal peptide synthase, putative			prot_ID_731	Afu6g09610	AFUA_6G09610	
AFUA_6G09620	4	2890	hypothetical protein			prot_ID_732	Afu6g09620	AFUA_6G09620	
AFUA_6G09630	3	720	C6 finger domain protein GliZ	gliZ		prot_ID_733	Afu6g09630	AFUA_6G09630	ED, IGD
AFUA_6G09640	2	343	aminotransferase GliI	gliI		prot_ID_734	Afu6g09640	AFUA_6G09640	
AFUA_6G09650	1	355	membrane dipeptidase GliJ	gliJ		**prot_ID_735**	**Afu6g09650**	**AFUA_6G09650**	
**AFUA_6G09660**	0	0	nonribosomal peptide synthase GliP	gliP		prot_ID_736	Afu6g09660	AFUA_6G09660	
AFUA_6G09670	-1	405	cytochrome P450 oxidoreductase GliC	gliC		prot_ID_737	Afu6g09670	AFUA_6G09670	
AFUA_6G09680	-2	284	O-methyltransferase GliM	gliM		prot_ID_738	Afu6g09680	AFUA_6G09680	
AFUA_6G09690	-3	376	glutathione S-transferase GliG	gliG		prot_ID_739	Afu6g09690	AFUA_6G09690	
AFUA_6G09700	-4	252	gliotoxin biosynthesis protein GliK	gliK		prot_ID_740	Afu6g09700	AFUA_6G09700	
AFUA_6G09710	-5	589	MFS gliotoxin efflux transporter GliA	gliA		prot_ID_741	Afu6g09710	AFUA_6G09710	
AFUA_6G09720	-6	390	methyltransferase GliN	gliN		prot_ID_742	Afu6g09720	AFUA_6G09720	
AFUA_6G09730	-7	454	cytochrome P450 oxidoreductase GliF	gliF		prot_ID_743	Afu6g09730	AFUA_6G09730	
AFUA_6G09740	-8	303	thioredoxin reductase GliT	gliT		prot_ID_744	Afu6g09740	AFUA_6G09740	ED
n/a					conserved hypothetical protein	prot_ID_1210	Afu6g09745	AFUA_6G09745	

Pseurotin A cluster

Experimentally determined cluster

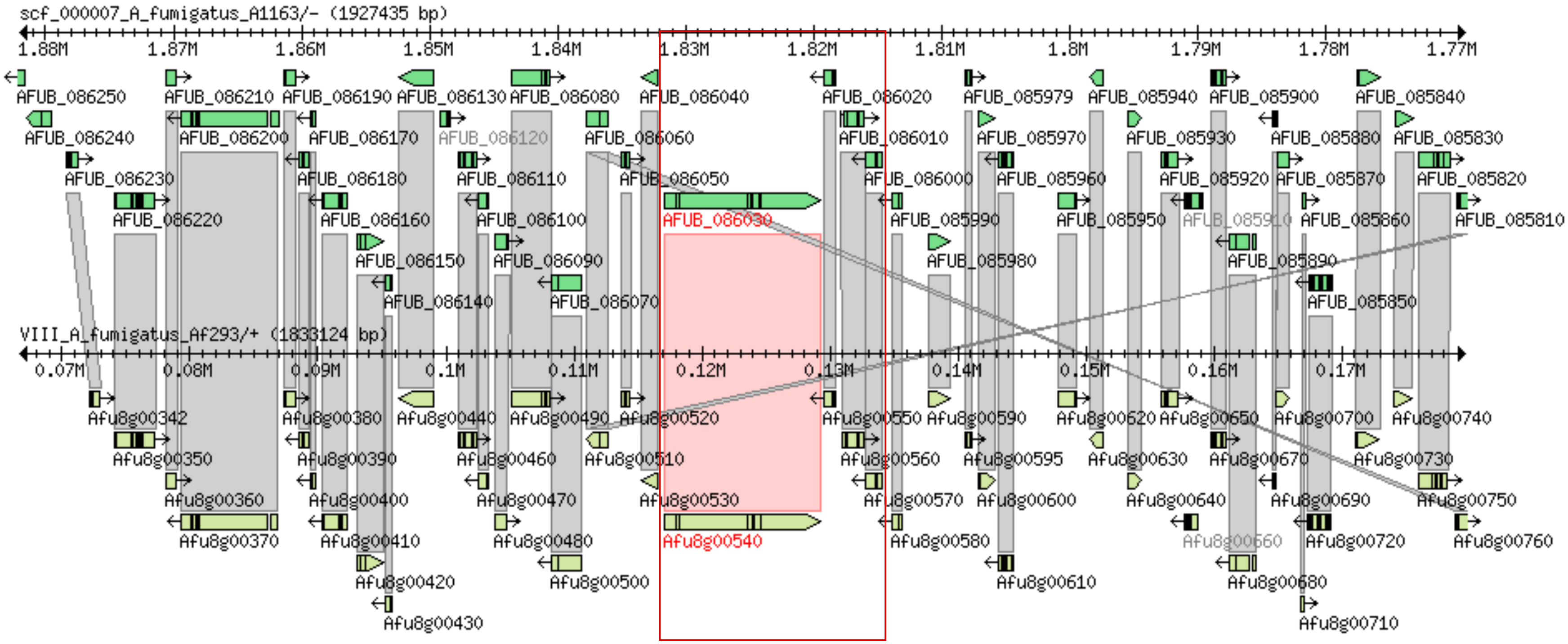

Pseurotin A cluster

Gene id (SMURF)	Gene positions	Gene distance	Annotated_gene_function (SMURF)	AspGD gene name	AspGD supplementary annotation	antiSMASH ID	Gene ID (I)	Gene ID (II)
AFUA_8G00370	-16	483	polyketide synthase, putative				Afu8g00370	AFUA_8G00370
AFUA_8G00380	-15	197	DltD N-terminal domain protein				Afu8g00380	AFUA_8G00380
n/a					Protein of unknown function	prot_ID_607	Afu8g00342	AFUA_8G00342
AFUA_8G00390	-14	87	O-methyltransferase, putative		O-methyltransferase	prot_ID_28	Afu8g00390	AFUA_8G00390
AFUA_8G00400	-13	429	conserved hypothetical protein		Protein of unknown function	prot_ID_29	Afu8g00400	AFUA_8G00400
AFUA_8G00410	-12	878	methionine aminopeptidase, type II, putative		Methionine aminopeptidase type II	prot_ID_30	Afu8g00410	AFUA_8G00410
AFUA_8G00420	-11	176	C6 finger transcription factor, putative	metAP	C6 zinc finger domain protein	prot_ID_31	Afu8g00420	AFUA_8G00420
AFUA_8G00430	-10	445	conserved hypothetical protein		Protein of unknown function	prot_ID_32	Afu8g00430	AFUA_8G00430
AFUA_8G00440	-9	1948	steroid monooxygenase, putative		Putative steroid monooxygenase	prot_ID_33	Afu8g00440	AFUA_8G00440
AFUA_8G00460	-8	133	methionine aminopeptidase, type I, putative		aminopeptidase/metalloexopeptidase	prot_ID_34	Afu8g00460	AFUA_8G00460
AFUA_8G00470	-7	397	conserved hypothetical protein		Protein of unknown function	prot_ID_35	Afu8g00470	AFUA_8G00470
AFUA_8G00480	-6	401	phytanoyl-CoA dioxygenase family protein		Protein of unknown function	prot_ID_36	Afu8g00480	AFUA_8G00480
AFUA_8G00490	-5	48	PKS-like enzyme, putative		Polyketide synthase	prot_ID_37	Afu8g00490	AFUA_8G00490
AFUA_8G00500	-4	409	acetate-CoA ligase, putative		Putative acetate-CoA ligase	prot_ID_38	Afu8g00500	AFUA_8G00500
AFUA_8G00510	-3	1148	cytochrome P450 oxidoreductase OrdA-like, putative		Oxidoreductase	prot_ID_39	Afu8g00510	AFUA_8G00510
AFUA_8G00520	-2	780	integral membrane protein		Prenyltransferase	prot_ID_40	Afu8g00520	AFUA_8G00520
AFUA_8G00530	-1	463	alpha/beta superfamily hydrolase		Alpha/beta hydrolase	prot_ID_41	Afu8g00530	AFUA_8G00530
**AFUA_8G00540**	0	0	hybrid PKS-NRPS enzyme, putative		PKS-NRPS hybrid	**prot_ID_42**	**Afu8g00540**	**AFUA_8G00540**ED
AFUA_8G00550	1	235	methyltransferase SirN-like, putative	nrps14/pesO	methyl transferase	prot_ID_43	Afu8g00550	AFUA_8G00550
AFUA_8G00560	2	539	cytochrome P450 oxidoreductase, putative		Cytochrome P450 monooxygenase	prot_ID_44	Afu8g00560	AFUA_8G00560
AFUA_8G00570	3	89	alpha/beta hydrolase, putative		Hydrolase	prot_ID_45	Afu8g00570	AFUA_8G00570 ED
AFUA_8G00580	4	825	glutathione S-transferase, putative		Elongation factor ElfB	prot_ID_46	Afu8g00580	AFUA_8G00580
AFUA_8G00590	5	2026	fatty acid desaturase, putative	elfB	Oxidoreductase	prot_ID_47	Afu8g00590	AFUA_8G00590
AFUA_8G00595	6	1189	Ham1 family pyrophosphatase, putative		Domain(s) with predicted hydrolase activity	n/a	Afu8g00595	AFUA_8G00595
AFUA_8G00600	7	541	NAD dependent epimerase/dehydratase family protein		Protein of unknown function	prot_ID_48	Afu8g00600	AFUA_8G00600
AFUA_8G00610	8	228	cell surface protein Mas1, putative	mas1	Putative cell surface protein	prot_ID_49	Afu8g00610	AFUA_8G00610
AFUA_8G00620	9	3538	dimethylallyl tryptophan synthase, putative	cdpNPT	DMATS-type N-prenyltransferase	prot_ID_50	Afu8g00620	AFUA_8G00620
n/a					Protein of unknown function	prot_ID_51	Afu8g00630	AFUA_8G00630
n/a					Protein of unknown function	prot_ID_52	Afu8g00640	AFUA_8G00640
n/a					Protein of unknown function	Protein of unknown function	Afu8g00650	AFUA_8G00650
n/a					Protein of unknown function	prot_ID_54	Afu8g00660	AFUA_8G00660

n/a		Protein of unknown function	prot_ID_55	Afu8g00670	AFUA_8G00670
n/a		Protein of unknown function	prot_ID_56	Afu8g00680	AFUA_8G00680
n/a		Protein of unknown function	prot_ID_57	Afu8g00690	AFUA_8G00690
n/a	chi2	Cass III chitinase	prot_ID_58	Afu8g00700	AFUA_8G00700
n/a		Domain(s) with predicted role in defense response, negative regulation of growth	prot_ID_59	Afu8g00710	AFUA_8G00710
n/a		Putative amino acid transporter	prot_ID_60	Afu8g00720	AFUA_8G00720
n/a		Hydrolase	prot_ID_612	Afu8g00595	AFUA_8G00595

Siderophore (sid) cluster

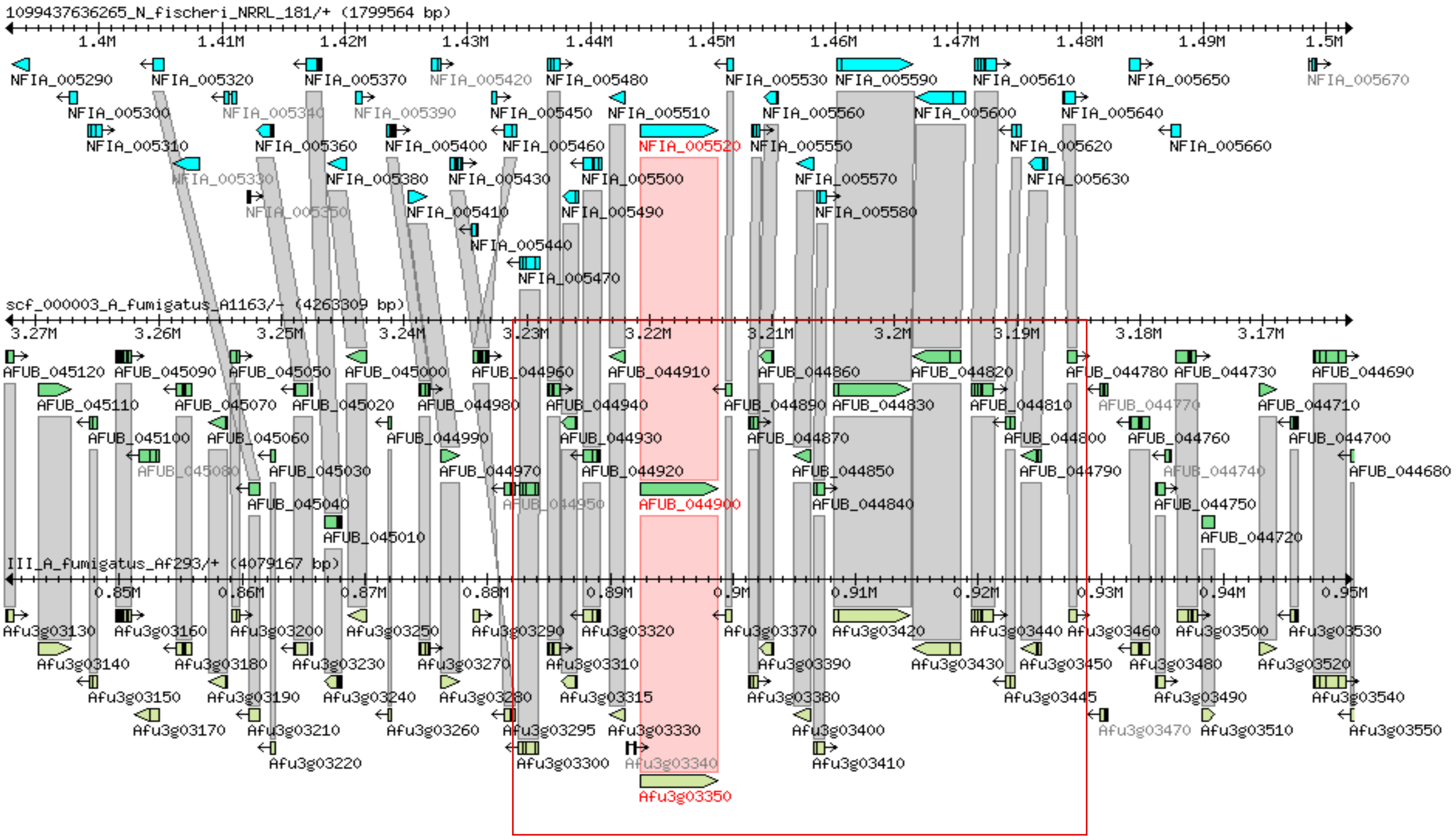

Afu1g17200 (sidC) cluster

Gene id (SMURF)	Gene positions	Gene distance	Annotated_gene_function (SMURF)	AspGD gene name	AspGD supplementary annotation	antiSMASH ID	Gene ID (I)	Gene ID (II)	
n/a						n/a	Afu1g17070	Afu1g17070	
AFUA_1G17080	12	247	acetyltransferase, GNAT family, putative			n/a	Afu1g17080	Afu1g17080	
AFUA_1G17090	11	82	NOL1/NOP2/sun domain protein, putative			prot_ID_1541	Afu1g17090	AFUA_1G17090	
AFUA_1G17100	10	2092	conserved hypothetical protein			prot_ID_1542	Afu1g17100	AFUA_1G17100	
AFUA_1G17110	9	228	long chain fatty alcohol oxidase, putative			prot_ID_1543	Afu1g17110	AFUA_1G17110	
AFUA_1G17120	8	661	translation elongation factor eEF-1B gamma subunit, putative			prot_ID_1544	Afu1g17120	AFUA_1G17120	
AFUA_1G17130	7	54	F-box domain protein			prot_ID_1545	Afu1g17130	AFUA_1G17130	
AFUA_1G17140	6	1204	voltage-gated K+ channel beta subunit (KCNAB), putative			prot_ID_1546	Afu1g17140	AFUA_1G17140	
AFUA_1G17150	5	73	C6 transcription factor, putative			prot_ID_1547	Afu1g17150	AFUA_1G17150	
AFUA_1G17160	4	895	MFS transporter, putative			prot_ID_1548	Afu1g17160	AFUA_1G17160	
AFUA_1G17170	3	89	TfdA family taurine dioxygenase, putative			prot_ID_1549	Afu1g17170	AFUA_1G17170	
AFUA_1G17180	2	1352	pyridine nucleotide-disulphide oxidoreductase AMID-like, putative			prot_ID_1550	Afu1g17180	AFUA_1G17180	
AFUA_1G17190	1	1728	long-chain-fatty-acid-CoA ligase, putative			prot_ID_1551	Afu1g17190	AFUA_1G17190	
**AFUA_1G17200**	0	0	nonribosomal siderophore peptide synthase SidC	sidC		**prot_ID_1552**	**Afu1g17200**	**AFUA_1G17200**	ECS
AFUA_1G17210	-1	1350	conserved hypothetical protein			prot_ID_1553	Afu1g17210	AFUA_1G17210	
AFUA_1G17220	-2	781	extracellular polygalacturonase, putative			prot_ID_1554	Afu1g17220	AFUA_1G17220	
AFUA_1G17230	-3	770	conserved hypothetical protein			prot_ID_1555	Afu1g17230	AFUA_1G17230	
AFUA_1G17240	-4	1006	C6 transcription factor, putative			prot_ID_1556	Afu1g17240	AFUA_1G17240	ECS
n/a				rodB	Conidial cell wall hydrophobin	prot_ID_1557	Afu1g17250	AFUA_1G17250	
n/a					Protein of unknown function	prot_ID_1558	Afu1g17260	AFUA_1G17260	
n/a				fre2	Metalloreductase	prot_ID_1559	Afu1g17270	AFUA_1G17270	

Afu1g01010 cluster

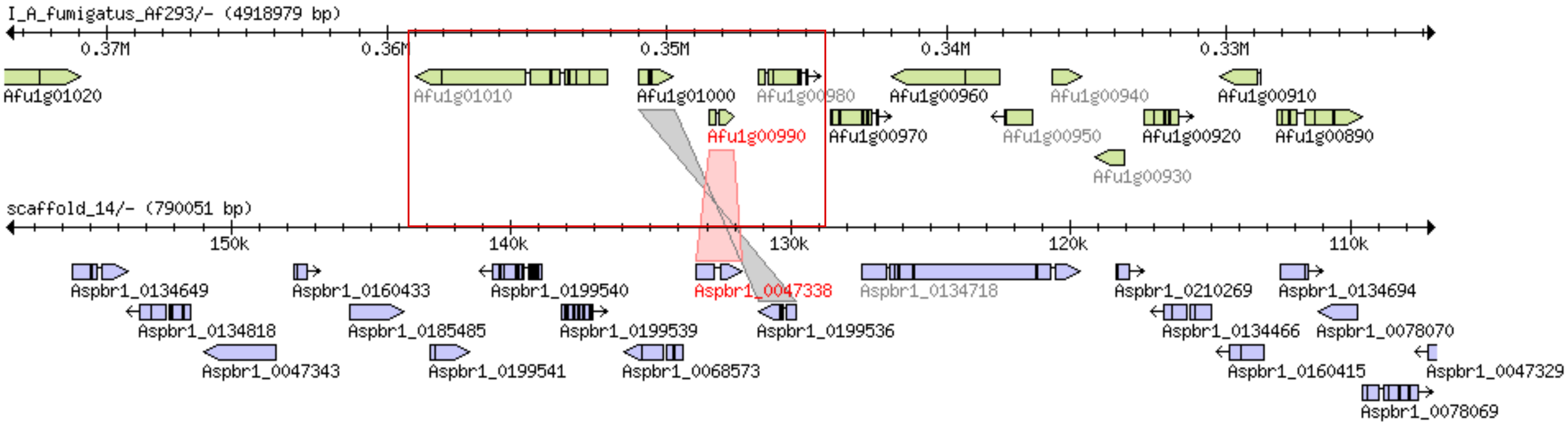

Afu1g01010 cluster

Gene id (SMURF)	Gene positions	Gene distance	Annotated_gene_function (SMURF)	AspGD gene name	AspGD supplementary annotation	antiSMASH ID	Gene ID (I)	Gene ID (II)	
AFUA_1G00970	4	800	MFS monocarboxylate transporter, putative		Transmembrane transporter	prot_ID_77	Afu1g00970	AFUA_1G00970	
AFUA_1G00980	3	918	FAD-dependent oxidase, putative		FAD-dependent oxidase, putative	prot_ID_78	Afu1g00980	AFUA_1G00980	FA
AFUA_1G00990	2	1220	short chain dehydrogenase/reductase family protein		short chain dehydrogenase/reductase family protein	prot_ID_79	Afu1g00990	AFUA_1G00990	
AFUA_1G01000	1	1041	oxidoreductase, 2OG-Fe(II) oxygenase family		oxidoreductase, 2OG-Fe(II) oxygenase family	prot_ID_80	Afu1g01000	AFUA_1G01000	
**AFUA_1G01010**	0	0	polyketide synthase, putative		Polyketide synthase	**prot_ID_81**	**Afu1g01010**	**AFUA_1G01010**	IGD
n/a					Protein of unknown function	prot_ID_72	Afu1g00920	AFUA_1G00920	
n/a					Protein of unknown function	prot_ID_73	Afu1g00930	AFUA_1G00930	
n/a					Mariner Ant1 transposon-related ORF	prot_ID_74	Afu1g00940	AFUA_1G00940	
n/a					Protein of unknown function	prot_ID_75	Afu1g00950	AFUA_1G00950	
n/a					LINE transposon-related ORF	prot_ID_76	Afu1g00960	AFUA_1G00960	
n/a					Protein of unknown function	prot_ID_82	Afu1g01020	AFUA_1G01020	
n/a					Reverse transcriptase	prot_ID_83	Afu1g01030	AFUA_1G01030	

Afu1g10380 (nrps1/pes1) cluster

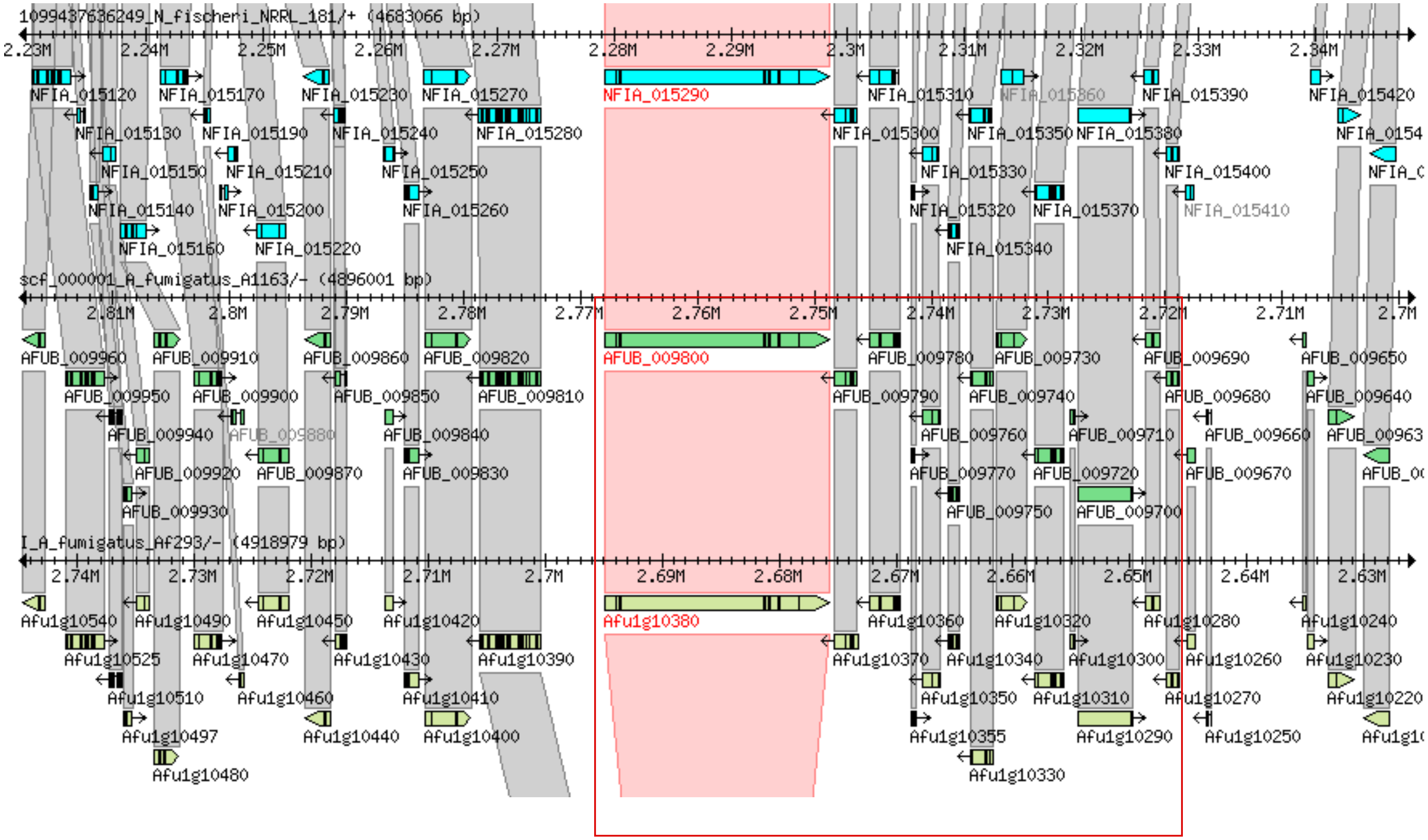

Afu1g10380 (nrps1/pes1) cluster

Gene id (SMURF)	Gene positions	Gene distance	Annotated_gene_function (SMURF)	AspGD gene name	AspGD supplementary annotation	antiSMASH ID	Gene ID (I)	Gene ID (II)	
n/a					oxidoreductase	n/a	Afu1g10270	AFUA_1G10270	ECS
n/a					C2H2 finger domain protein	n/a	Afu1g10280	AFUA_1G10280	
n/a					chromodomain helicase	n/a	Afu1g10290	AFUA_1G10290	
n/a					Protein of unknown function	n/a	Afu1g10300	AFUA_1G10300	
AFUA_1G10310	8	756	RNase L inhibitor of the ABC superfamily, putative			prot_ID_874	Afu1g10310	AFUA_1G10310	
AFUA_1G10320	7	320	formin binding protein (FNB3), putative			prot_ID_875	Afu1g10320	AFUA_1G10320	
AFUA_1G10330	6	916	conserved hypothetical protein			prot_ID_876	Afu1g10330	AFUA_1G10330	
AFUA_1G10340	5	746	integral membrane protein, Mpv17/PMP22 family, putative			prot_ID_877	Afu1g10340	AFUA_1G10340	
AFUA_1G10350	4	548	phosphoglycerate kinase PgkA, putative	pgkA		prot_ID_878	Afu1g10350	AFUA_1G10350	
AFUA_1G10355	3	1006	26 proteasome complex subunit Sem1, putative			prot_ID_1627	Afu1g10355	AFUA_1G10355	
AFUA_1G10360	2	1064	conserved hypothetical protein		Protein of unknown function	prot_ID_879	Afu1g10360	AFUA_1G10360	
AFUA_1G10370	1	487	MFS multidrug transporter, putative		MFS multidrug transporter	prot_ID_880	Afu1g10370	AFUA_1G10370	
**AFUA_1G10380**	0	0	nonribosomal peptide synthase Pes1	nrps1/pes1	Non-ribosomal peptide synthetase	prot_ID_881	Afu1g10380	AFUA_1G10380	IGD
n/a				abcB	ABC multidrug transporter	prot_ID_882	Afu1g10390	AFUA_1G10390	
n/a					Protein of unknown function	prot_ID_883	Afu1g10400	AFUA_1G10400	
n/a					DNA damage repair protein uvsC	prot_ID_884	Afu1g10410	AFUA_1G10410	
n/a					Protein of unknown function	prot_ID_885	Afu1g10420	AFUA_1G10420	

Afu1g17200 (sidC) cluster

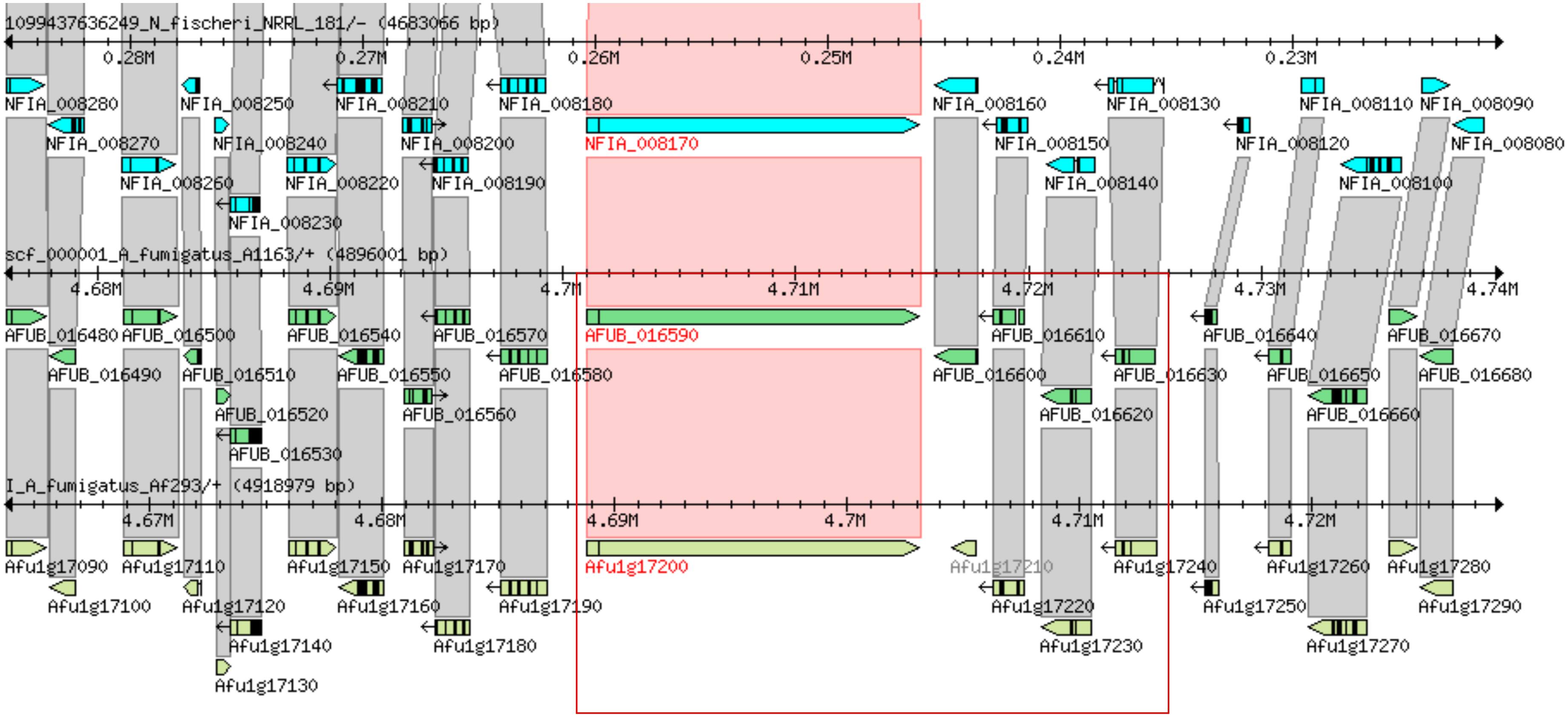

Afu1g17200 (sidC) cluster

Gene id (SMURF)	Gene positions	Gene distance	Annotated_gene_function (SMURF)	AspGD gene name	AspGD supplementary annotation	antiSMASH ID	Gene ID (I)	Gene ID (II)	
n/a						n/a	Afu1g17070	Afu1g17070	
AFUA_1G17080	12	247	acetyltransferase, GNAT family, putative			n/a	Afu1g17080	Afu1g17080	
AFUA_1G17090	11	82	NOL1/NOP2/sun domain protein, putative			prot_ID_1541	Afu1g17090	AFUA_1G17090	
AFUA_1G17100	10	2092	conserved hypothetical protein			prot_ID_1542	Afu1g17100	AFUA_1G17100	
AFUA_1G17110	9	228	long chain fatty alcohol oxidase, putative			prot_ID_1543	Afu1g17110	AFUA_1G17110	
AFUA_1G17120	8	661	translation elongation factor eEF-1B gamma subunit, putative			prot_ID_1544	Afu1g17120	AFUA_1G17120	
AFUA_1G17130	7	54	F-box domain protein			prot_ID_1545	Afu1g17130	AFUA_1G17130	
AFUA_1G17140	6	1204	voltage-gated K+ channel beta subunit (KCNAB), putative			prot_ID_1546	Afu1g17140	AFUA_1G17140	
AFUA_1G17150	5	73	C6 transcription factor, putative			prot_ID_1547	Afu1g17150	AFUA_1G17150	
AFUA_1G17160	4	895	MFS transporter, putative			prot_ID_1548	Afu1g17160	AFUA_1G17160	
AFUA_1G17170	3	89	TfdA family taurine dioxygenase, putative			prot_ID_1549	Afu1g17170	AFUA_1G17170	
AFUA_1G17180	2	1352	pyridine nucleotide-disulphide oxidoreductase AMID-like, putative			prot_ID_1550	Afu1g17180	AFUA_1G17180	
AFUA_1G17190	1	1728	long-chain-fatty-acid-CoA ligase, putative			prot_ID_1551	Afu1g17190	AFUA_1G17190	
**AFUA_1G17200**	0	0	nonribosomal siderophore peptide synthase SidC	sidC		**prot_ID_1552**	**Afu1g17200**	**AFUA_1G17200**	IGD
AFUA_1G17210	-1	1350	conserved hypothetical protein			prot_ID_1553	Afu1g17210	AFUA_1G17210	
AFUA_1G17220	-2	781	extracellular polygalacturonase, putative			prot_ID_1554	Afu1g17220	AFUA_1G17220	
AFUA_1G17230	-3	770	conserved hypothetical protein			prot_ID_1555	Afu1g17230	AFUA_1G17230	
AFUA_1G17240	-4	1006	C6 transcription factor, putative			prot_ID_1556	Afu1g17240	AFUA_1G17240	IGD, FA
n/a				rodB	Conidial cell wall hydrophobin	prot_ID_1557	Afu1g17250	AFUA_1G17250	
n/a					Protein of unknown function	prot_ID_1558	Afu1g17260	AFUA_1G17260	
n/a				fre2	Metalloreductase	prot_ID_1559	Afu1g17270	AFUA_1G17270	

Afu1g17740 cluster

No A1163 orthologous gene cluster

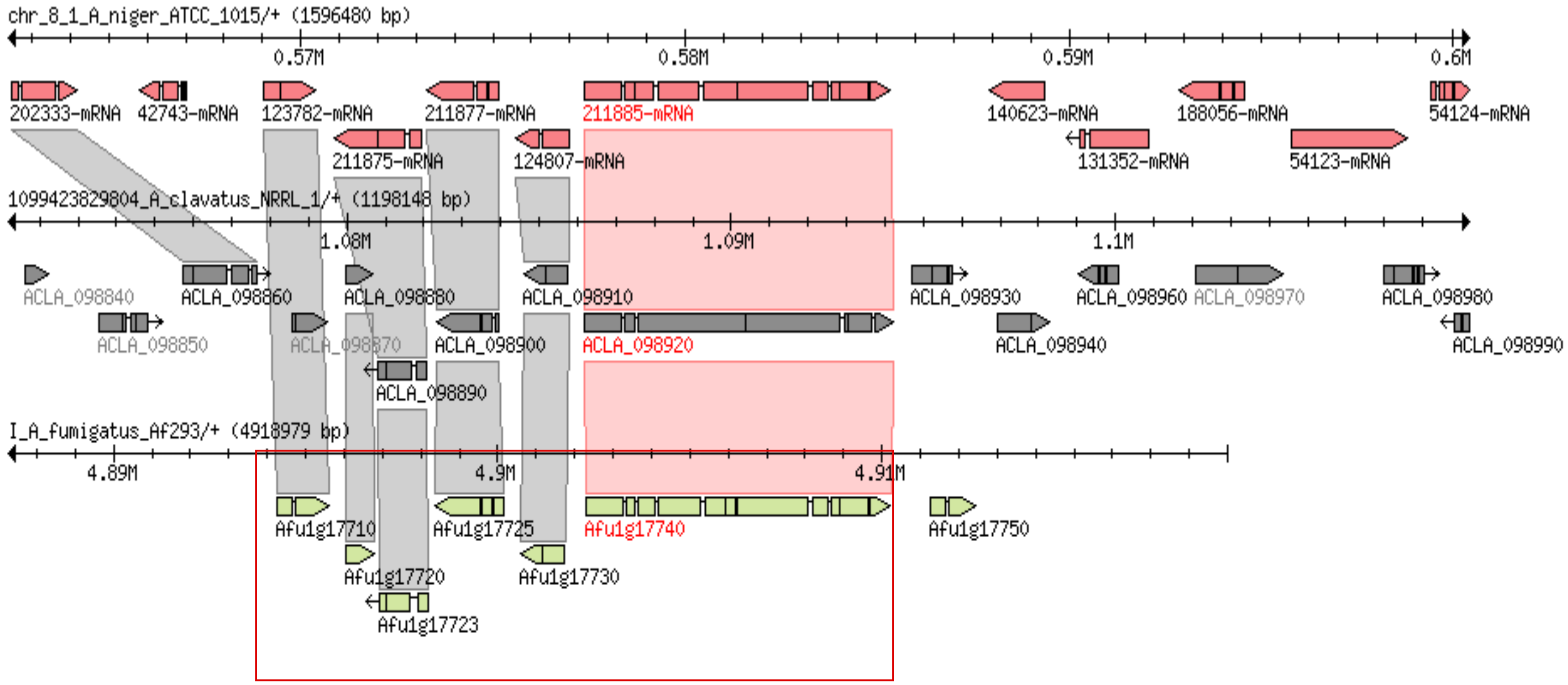

Afu1g17740 cluster

Gene id (SMURF)	Gene positions	Gene distance	Annotated_gene_function (SMURF)	AspGD gene name	AspGD supplementary annotation	antiSMASH ID	Gene ID (I)	Gene ID (II)	
AFUA_1G17710	5	442	C6 transcription factor, putative			n/a	Afu1g17710	AFUA_1G17710	ECS
AFUA_1G17720	4	97	oxidoreductase, putative			prot_ID_1600	Afu1g17720	AFUA_1G17720	
AFUA_1G17723	3	188	aldose 1-epimerase family protein, putative			prot_ID_1612	Afu1g17723	AFUA_1G17723	
AFUA_1G17725	2	439	cytochrome P450 oxidoreductase, putative			prot_ID_1601	Afu1g17725	AFUA_1G17725	
AFUA_1G17730	1	534	zinc binding enoyl reductase, putative			prot_ID_1602	Afu1g17730	AFUA_1G17730	
**AFUA_1G17740**	0	0	polyketide synthase, putative			**prot_ID_1603**	**Afu1g17740**	**AFUA_1G17740**	ECS
n/a					nicotinamide N-methyltransferase, putative	prot_ID_1604	Afu1g17750	AFUA_1G17750	

Afu2g01290 cluster

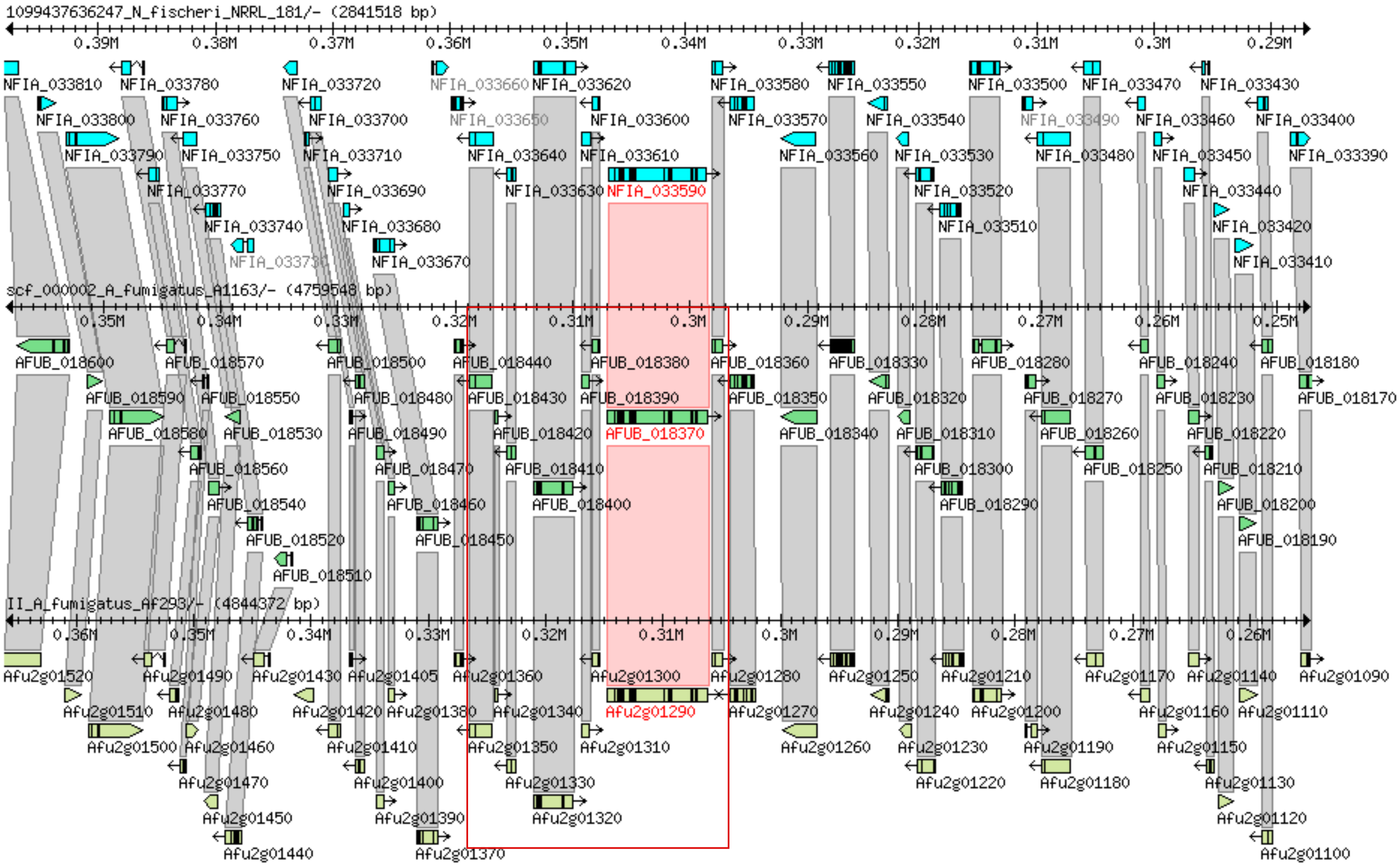

Afu2g01290 cluster

Gene id (SMURF)	Gene positions	Gene distance	Annotated_gene_function (SMURF)	AspGD gene name	AspGD supplementary annotation	antiSMASH ID	Gene ID (I)	Gene ID (II)	
n/a					ATP dependent RNA helicase	prot_ID_109	Afu2g01210	AFUA_2G01210	
n/a					GTP cyclohydrolase II	prot_ID_110	Afu2g01220	AFUA_2G01220	
n/a					Predicted lyase	prot_ID_111	Afu2g01230	AFUA_2G01230	
n/a				invB	Predicted invertase	prot_ID_112	Afu2g01240	AFUA_2G01240	
n/a					Serine peptidase	prot_ID_113	Afu2g01250	AFUA_2G01250	
n/a				srbA	Sterol regulatory element binding protein	prot_ID_114	Afu2g01260	AFUA_2G01260	
n/a					Oxidoreductase	prot_ID_115	Afu2g01270	AFUA_2G01270	
n/a					Oxidoreductase	prot_ID_116	Afu2g01280	AFUA_2G01280	ECS
n/a					Polyketide synthase	**prot_ID_117**	**Afu2g01290**	**AFUA_2G01290**	
n/a					Protein of unknown function	prot_ID_118	Afu2g01300	AFUA_2G01300	
n/a					Protein of unknown function	prot_ID_119	Afu2g01310	AFUA_2G01310	
n/a					P-type ATPase	prot_ID_120	Afu2g01320	AFUA_2G01320	
n/a					Protein of unknown function	prot_ID_121	Afu2g01330	AFUA_2G01330	ECS
n/a					Protein of unknown function	prot_ID_122	Afu2g01340	AFUA_2G01340	
n/a					NAD+/NADH kinase	prot_ID_123	Afu2g01350	AFUA_2G01350	
n/a					Methionine-R-sulfoxide reductase	prot_ID_124	Afu2g01360	AFUA_2G01360	
n/a					Amino acid transmembrane transporter	prot_ID_125	Afu2g01370	AFUA_2G01370	
n/a					GNAT family N-acetyltransferase	prot_ID_126	Afu2g01380	AFUA_2G01380	
n/a					Trans-aconitate 3-methyltransferase	prot_ID_127	Afu2g01390	AFUA_2G01390	

Afu2g05760 cluster

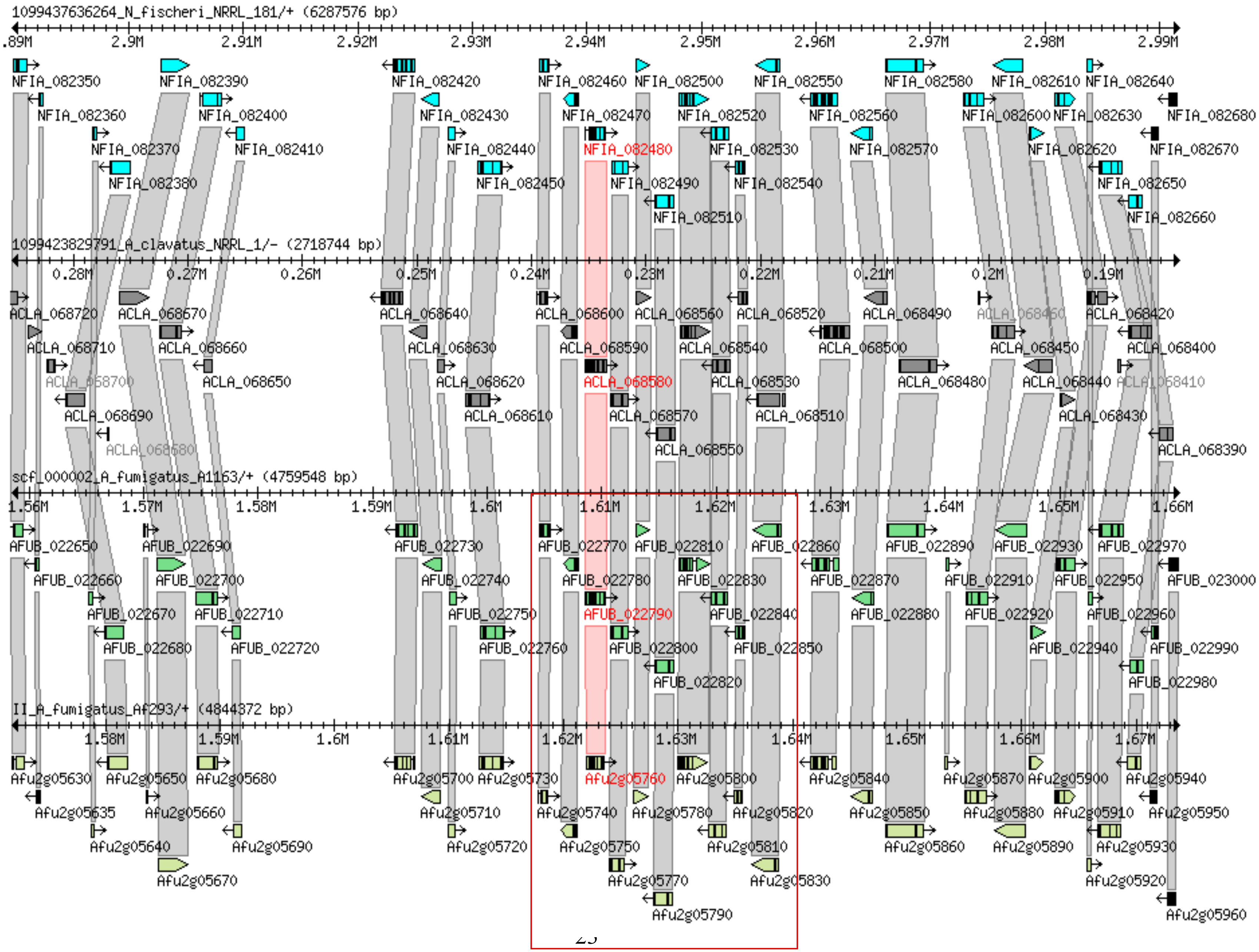

Afu2g05760 cluster

Gene id (SMURF)	Gene positions	Gene distance	Annotated_gene_function (SMURF)	AspGD gene name	AspGD supplementary annotation	antiSMASH ID	Gene ID (I)	Gene ID (II)	
AFUA_2G05730	3	3096	siderochrome-iron transporter MirC			n/a	Afu2g05730	AFUA_2G05730	
AFUA_2G05740	2	1079	Rho GTPase ModA, putative			n/a	Afu2g05740	AFUA_2G05740	IGD
AFUA_2G05750	1	805	agmatinase, putative			n/a	Afu2g05750	AFUA_2G05750	
**AFUA_2G05760**	0	0	beta-ketoacyl synthase (Cem1), putative			n/a	**Afu2g05760**	**AFUA_2G05760**	
AFUA_2G05770	-1	382	SAGA complex component (Sgf73), putative			n/a	Afu2g05770	AFUA_2G05770	
AFUA_2G05780	-2	701	mRNA capping enzyme alpha subunit, putative			n/a	Afu2g05780	AFUA_2G05780	
AFUA_2G05790	-3	418	oligosaccharyl transferase subunit (alpha), putative			n/a	Afu2g05790	AFUA_2G05790	
AFUA_2G05800	-4	433	IdgA domain protein			n/a	Afu2g05800	AFUA_2G05800	
AFUA_2G05810	-5	62	dienelactone hydrolase			n/a	Afu2g05810	AFUA_2G05810	
AFUA_2G05820	-6	638	Riboflavin kinase, putative			n/a	Afu2g05820	AFUA_2G05820	
AFUA_2G05830	-7	819	C6 finger domain protein, putative			n/a	Afu2g05830	AFUA_2G05830	IGD
AFUA_2G05840	-8	2789	MFS multidrug transporter, putative			n/a	Afu2g05840	AFUA_2G05840	

Afu2g17600 cluster

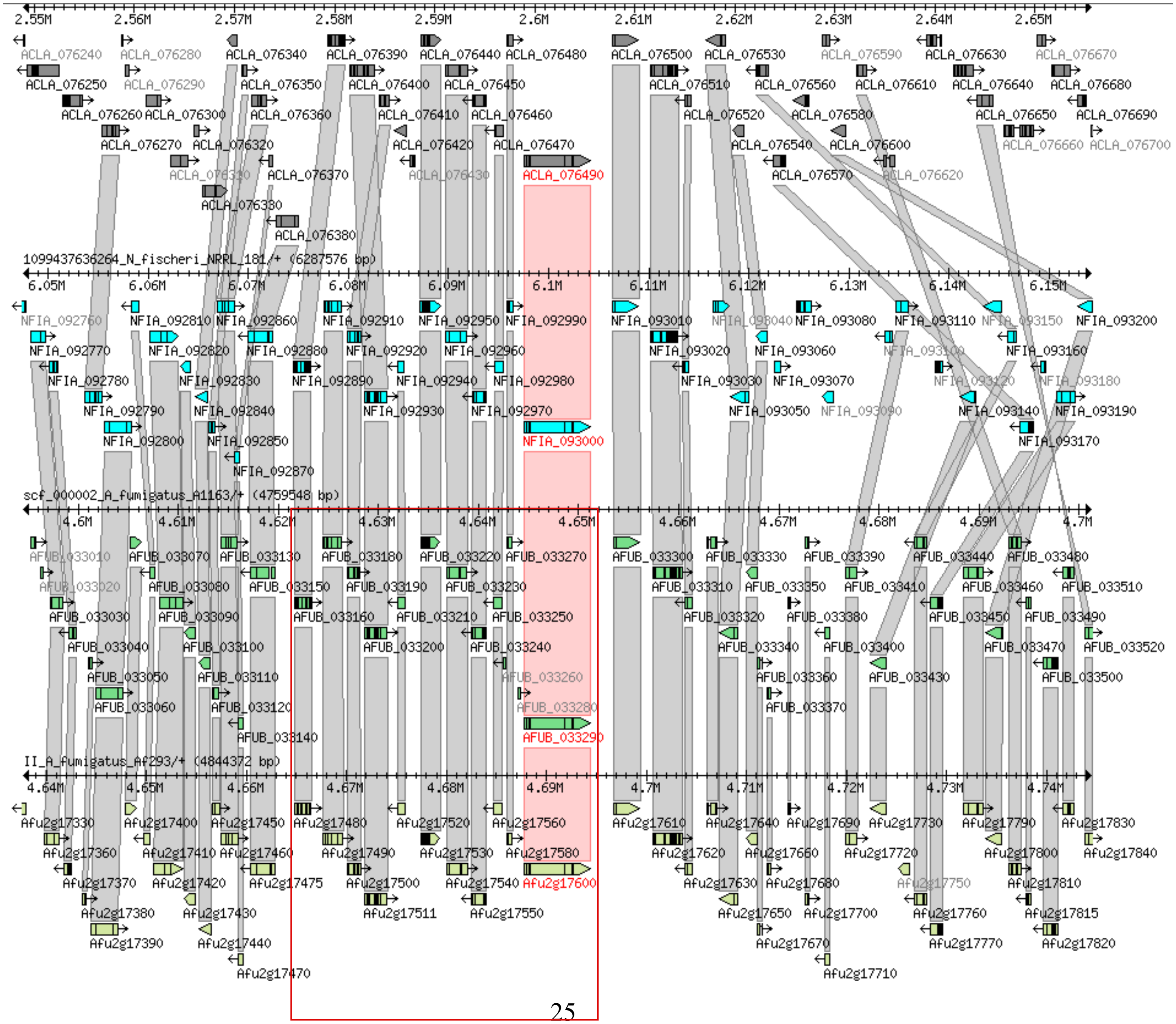

Afu2g17600 cluster

Gene id (SMURF)	Gene positions	Gene distance	Annotated_gene_function (SMURF)	AspGD gene name	AspGD supplementary annotation	antiSMASH ID	Gene ID (I)	Gene ID (II)	
n/a					Protein of unknown function	n/a	Afu2g17480	AFUA_2G17480	IGD
n/a					N,N-dimethylaniline monooxygenase	prot_ID_1545	Afu2g17490	AFUA_2G17490	
n/a					Nitrilase	prot_ID_1546	Afu2g17500	AFUA_2G17500	
AFUA_2G17511	7	1019	C6 finger domain protein, putative						
AFUA_2G17520	6	1578	GNAT family N-acetyltransferase, putative			prot_ID_1547	Afu2g17520	AFUA_2G17520	
AFUA_2G17530	5	506	conidial pigment biosynthesis oxidase Arb2	arb2		prot_ID_1548	Afu2g17530	AFUA_2G17530	
AFUA_2G17540	4	421	conidial pigment biosynthesis oxidase Abr1/brown 1			prot_ID_1549	Afu2g17540	AFUA_2G17540	
AFUA_2G17550	3	837	conidial pigment biosynthesis protein Ayg1	ayg1		prot_ID_1550	Afu2g17550	AFUA_2G17550	
AFUA_2G17560	2	464	conidial pigment biosynthesis 1,3,6,8-tetrahydroxynaphthalene reductase Arp2			prot_ID_1551	Afu2g17560	AFUA_2G17560	
AFUA_2G17580	1	1098	conidial pigment biosynthesis scytalone dehydratase Arp1	arp1		prot_ID_1552	Afu2g17580	AFUA_2G17580	
**AFUA_2G17600**	0	0	conidial pigment polyketide synthase PksP/Alb1	pksP/alb1		**prot_ID_1553**	**Afu2g17600**	**AFUA_2G17600**	IGD
n/a						prot_ID_1554	Afu2g17610	AFUA_2G17610	
n/a					Cellobiose dehydrogenase CDH cytochrome superfamily, secreted	prot_ID_1555	Afu2g17620	AFUA_2G17620	
n/a					Ortholog of the hydrophobic surface binding protein, hsbA	prot_ID_1556	Afu2g17630	AFUA_2G17630	
n/a					Protein of unknown function	prot_ID_1557	Afu2g17640	AFUA_2G17640	
n/a					Protein of unknown function	prot_ID_1558	Afu2g17650	AFUA_2G17650	
n/a					Transmembrane transporter	prot_ID_1559	Afu2g17660	AFUA_2G17660	
n/a					Protein of unknown function	prot_ID_1560	Afu2g17670	AFUA_2G17670	
n/a					Protein of unknown function	prot_ID_1561	Afu2g17680	AFUA_2G17680	
n/a					Protein of unknown function	prot_ID_1562	Afu2g17690	AFUA_2G17690	

Afu3g01410 cluster

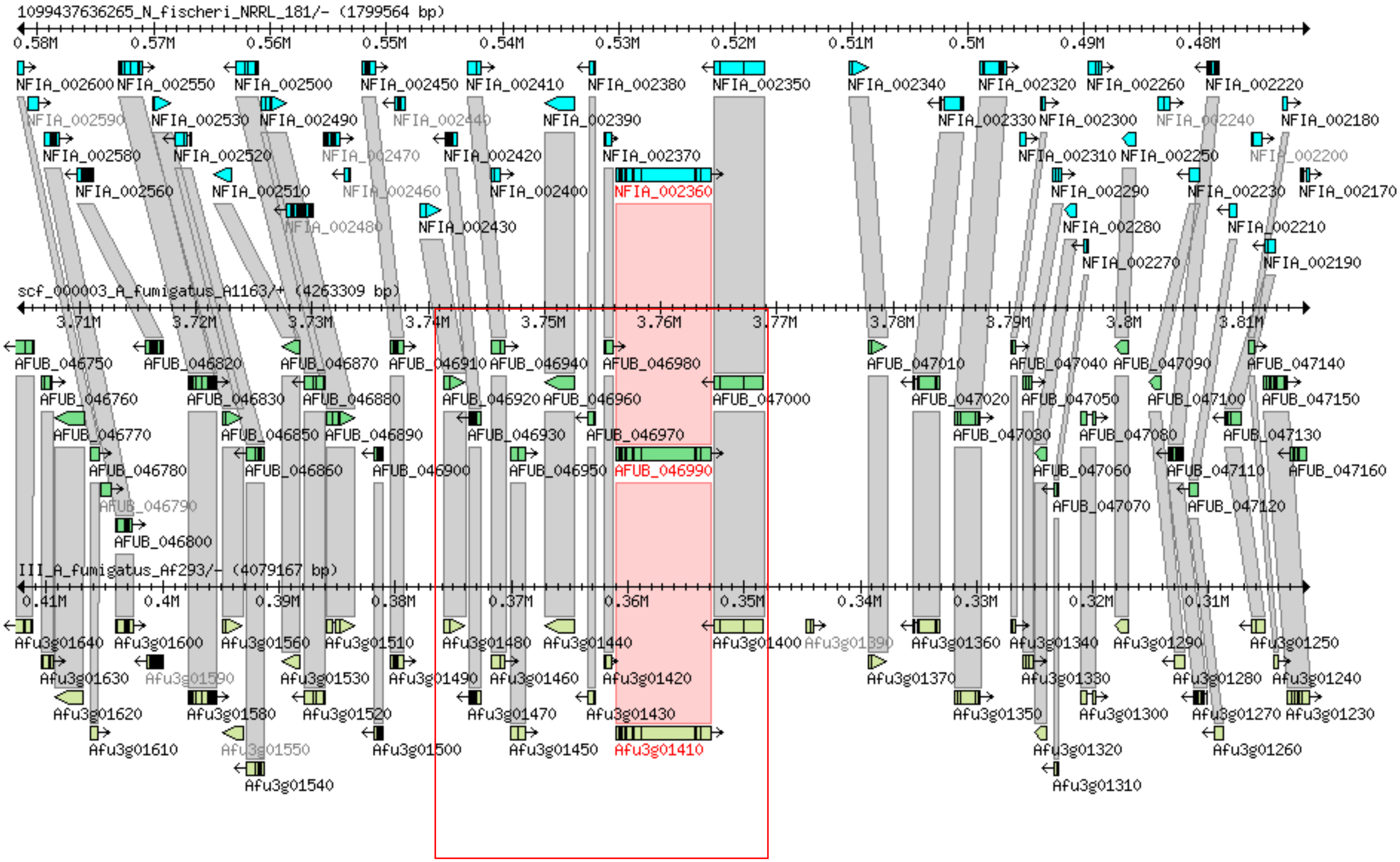

Afu3g01410 cluster

Gene id (SMURF)	Gene positions	Gene distance	Annotated_gene_function (SMURF)	AspGD gene name	AspGD supplementary annotation	antiSMASH ID	Gene ID (I)	Gene ID (II)	
n/a					Transmembrane transporter	prot_ID_119	Afu3g01360	AFUA_3G01360	
n/a					Transmembrane transporter	prot_ID_120	Afu3g01370	AFUA_3G01370	
n/a					Protein of unknown function	prot_ID_121	Afu3g01390	AFUA_3G01390	
AFUA_3G01400	1	235	ABC multidrug transporter, putative			prot_ID_122	Afu3g01400	AFUA_3G01400	IGD
**AFUA_3G01410**	0	0	polyketide synthase, putative			**prot_ID_123**	**Afu3g01410**	**AFUA_3G01410**	
AFUA_3G01420	-1	291	DUF341 family oxidoreductase, putative			prot_ID_124	Afu3g01420	AFUA_3G01420	
AFUA_3G01430	-2	716	benzodiazepine receptor family protein			prot_ID_125	Afu3g01430	AFUA_3G01430	
AFUA_3G01440	-3	1240	DUF1212 domain membrane protein			prot_ID_126	Afu3g01440	AFUA_3G01440	
AFUA_3G01450	-4	1576	3-methyl-2-oxobutanoate dehydrogenase, putative			prot_ID_127	Afu3g01450	AFUA_3G01450	
AFUA_3G01460	-5	388	salicylate hydroxylase, putative			prot_ID_128	Afu3g01460	AFUA_3G01460	
AFUA_3G01470	-6	914	conserved hypothetical protein			prot_ID_129	Afu3g01470	AFUA_3G01470	
AFUA_3G01480	-7	223	conserved hypothetical protein			prot_ID_130	Afu3g01480	AFUA_3G01480	IGD
AFUA_3G01490	-8	3415	alcohol dehydrogenase, putative			prot_ID_131	Afu3g01490	AFUA_3G01490	
AFUA_3G01500	-9	667	integral membrane protein			n/a	Afu3g01500	AFUA_3G01500	
AFUA_3G01510	-10	1580	C6 transcription factor, putative			n/a	Afu3g01510	AFUA_3G01510	
AFUA_3G01520	-11	115	MFS multidrug transporter, putative			n/a	Afu3g01520	AFUA_3G01520	
AFUA_3G01530	-12	408	phosphatidylglycerol specific phospholipase, putative			n/a	Afu3g01530	AFUA_3G01530	
AFUA_3G01540	-13	1642	MFS monocarboxylate transporter, putative			n/a	Afu3g01540	AFUA_3G01540	
AFUA_3G01560	-14	190	amino acid permease (Can1), putative			n/a	Afu3g01560	AFUA_3G01560	

Afu3g02530 cluster

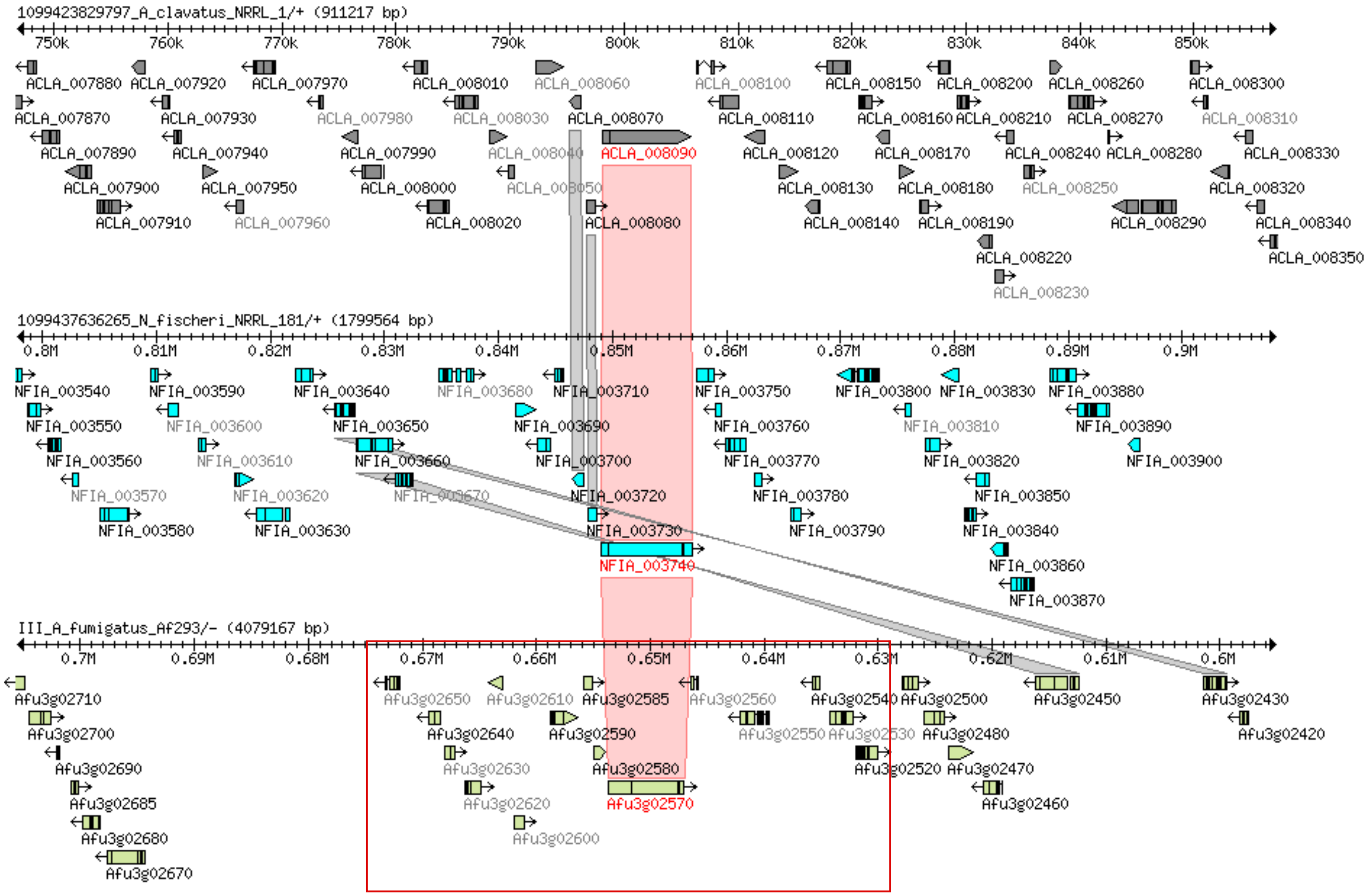

Afu3g02530 cluster

Gene id (SMURF)	Gene positions	Gene distance	Annotated_gene_function (SMURF)	AspGD gene name	AspGD supplementary annotation	antiSMASH ID	Gene ID (I)	Gene ID (II)	
AFUA_3G02450	6	2900	conserved hypothetical protein			prot_ID_220	Afu3g02450	AFUA_3G02450	
AFUA_3G02460	5	895	serine protein kinase, putative			prot_ID_221	Afu3g02460	AFUA_3G02460	
AFUA_3G02470	4	396	amidase family protein			prot_ID_222	Afu3g02470	AFUA_3G02470	
AFUA_3G02480	3	499	C6 transcription factor, putative			prot_ID_223	Afu3g02480	AFUA_3G02480	
AFUA_3G02500	2	2022	protein kinase, putative			prot_ID_224	Afu3g02500	AFUA_3G02500	
AFUA_3G02520	1	48	MFS multidrug transporter, putative			prot_ID_225	Afu3g02520	AFUA_3G02520	FA
**AFUA_3G02530**	0	0	PKS-like enzyme, putative			prot_ID_226	Afu3g02530	AFUA_3G02530	
AFUA_3G02540	-1	867	conserved hypothetical protein			prot_ID_227	Afu3g02540	AFUA_3G02540	
n/a					Polyketide synthase	**prot_ID_228**	**Afu3g02550**	**AFUA_3G02550**	
n/a					Protein of unknown function	prot_ID_229	Afu3g02560	AFUA_3G02560	
n/a					Polyketide synthase	**prot_ID_230**	**Afu3g02570**	**AFUA_3G02570**	
n/a					Oxidoreductase	prot_ID_231	Afu3g02580	AFUA_3G02580	
n/a					Protein of unknown function	prot_ID_232	Afu3g02585	AFUA_3G02585	
n/a					C6 transcription factor	prot_ID_233	Afu3g02590	AFUA_3G02590	
n/a					Protein of unknown function	prot_ID_234	Afu3g02600	AFUA_3G02600	
n/a					Transmembrane transporter	prot_ID_235	Afu3g02610	AFUA_3G02610	
n/a					Monooxygenase	prot_ID_236	Afu3g02620	AFUA_3G02620	
n/a					Protein of unknown function	prot_ID_237	Afu3g02630	AFUA_3G02630	
n/a					Protein of unknown function	prot_ID_238	Afu3g02640	AFUA_3G02640	
n/a					MYB DNA binding domain protein	prot_ID_239	Afu3g02650	AFUA_3G02650	IGD

Afu3g02570 cluster

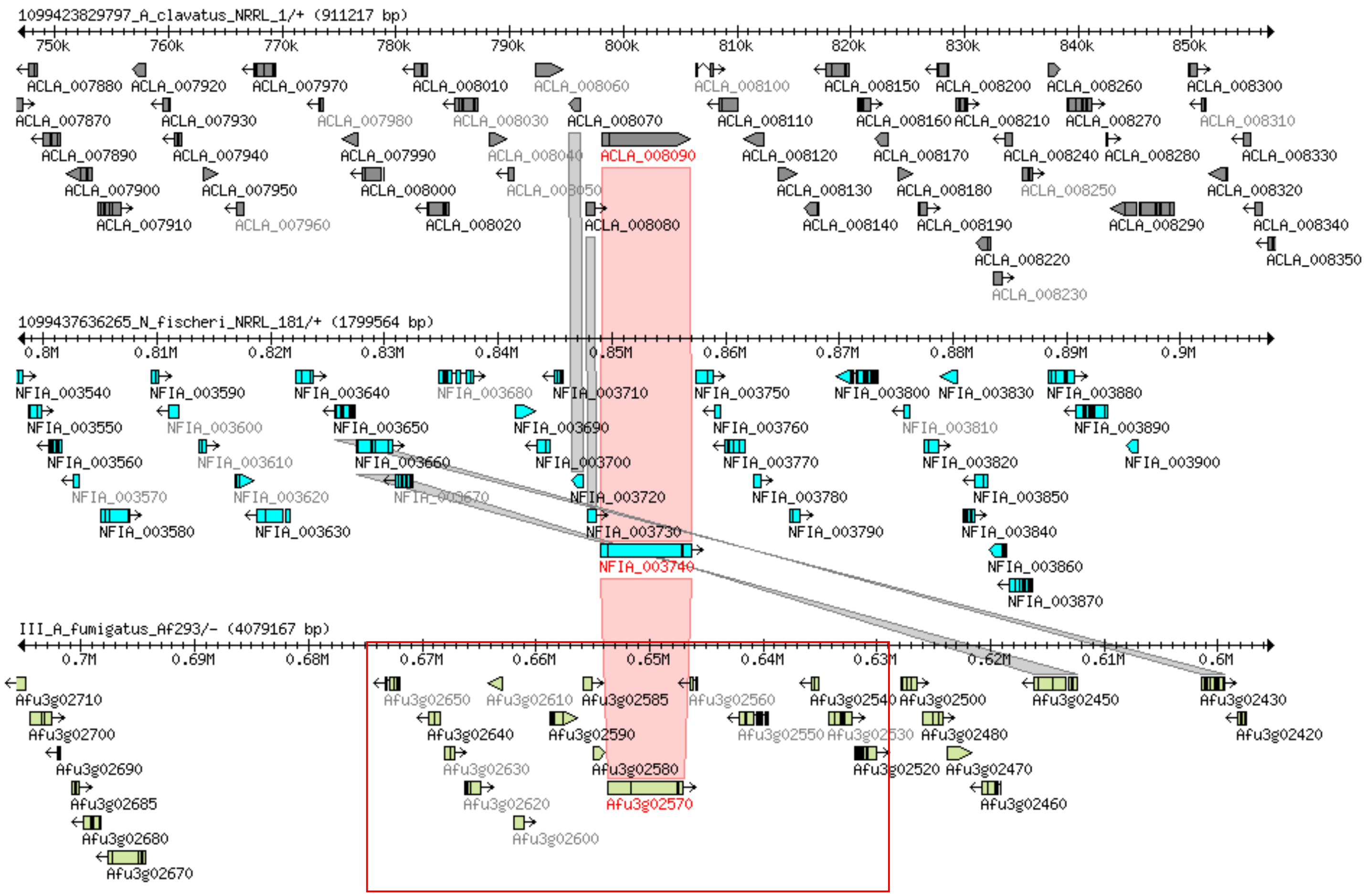

Afu3g02570 cluster

Gene id (SMURF)	Gene positions	Gene distance	Annotated_gene_function (SMURF)	AspGD gene name	AspGD supplementary annotation	antiSMASH ID	Gene ID (I)	Gene ID (II)	
n/a					Protein of unknown function	prot_ID_220	Afu3g02450	AFUA_3G02450	
n/a					Protein serine/threonine kinase	prot_ID_221	Afu3g02460	AFUA_3G02460	
n/a					Carbon-nitrogen ligase	prot_ID_222	Afu3g02470	AFUA_3G02470	
n/a					C6 transcription factor	prot_ID_223	Afu3g02480	AFUA_3G02480	
n/a					Predicted ATP binding, protein kinase	prot_ID_224	Afu3g02500	AFUA_3G02500	
n/a					Transmembrane transporter	prot_ID_225	Afu3g02520	AFUA_3G02520	FA
n/a					polyketide synthase	**prot_ID_226**	**Afu3g02530**	**AFUA_3G02530**	
n/a					Protein of unknown function	prot_ID_227	Afu3g02540	AFUA_3G02540	
n/a					Protein of unknown function	prot_ID_228	Afu3g02550	AFUA_3G02550	
n/a					Deleted ORF	prot_ID_229	Afu3g02560	AFUA_3G02560	
**AFUA_3G02570**	0	0	polyketide synthase, putative			**prot_ID_230**	**Afu3g02570**	**AFUA_3G02570**	
AFUA_3G02580	-1	198	short-chain dehydrogenase/reductase family protein, putative			prot_ID_231	Afu3g02580	AFUA_3G02580	
AFUA_3G02585	-2	218	integral membrane protein			prot_ID_232	Afu3g02585	AFUA_3G02585	
AFUA_3G02590	-3	315	C6 transcription factor, putative			prot_ID_233	Afu3g02590	AFUA_3G02590	
AFUA_3G02600	-4	2323	conserved hypothetical protein			prot_ID_234	Afu3g02600	AFUA_3G02600	
AFUA_3G02610	-5	1097	MFS transporter, putative			prot_ID_235	Afu3g02610	AFUA_3G02610	
AFUA_3G02620	-6	427	phenol hydroxylase, putative			prot_ID_236	Afu3g02620	AFUA_3G02620	
AFUA_3G02630	-7	823	conserved hypothetical protein			prot_ID_237	Afu3g02630	AFUA_3G02630	
AFUA_3G02640	-8	413	nucleoside-diphosphate-sugar epimerase family protein			prot_ID_238	Afu3g02640	AFUA_3G02640	
AFUA_3G02650	-9	2556	MYB DNA binding domain protein			prot_ID_239	Afu3g02650	AFUA_3G02650	IGD

Afu3g02670 cluster

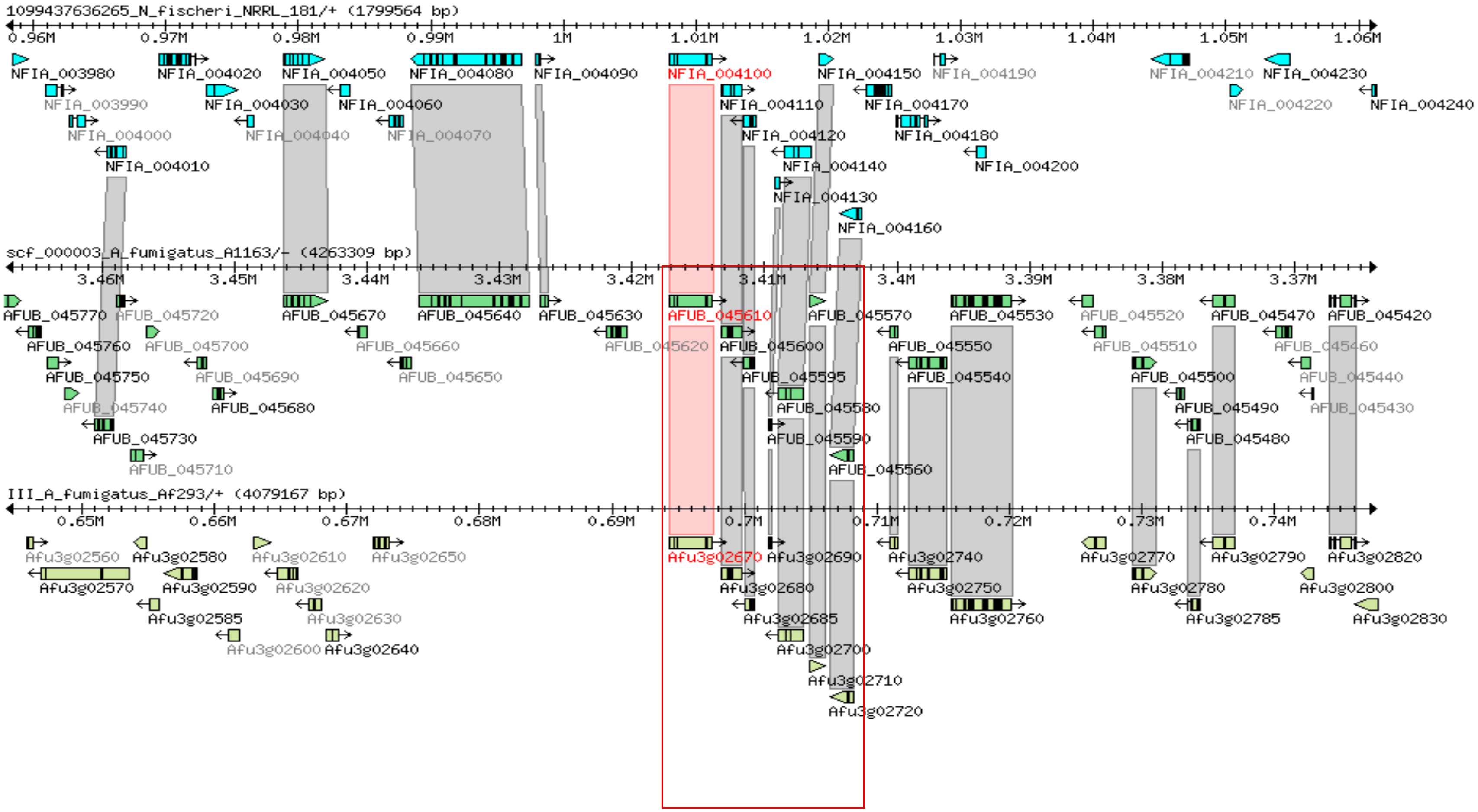

Afu3g02670 cluster

Gene id (SMURF)	Gene positions	Gene distance	Annotated_gene_function (SMURF)	AspGD gene name	AspGD supplementary annotation	antiSMASH ID	Gene ID (I)	Gene ID (II)	
**AFUA_3G02670**	0	0	NRPS-like enzyme, putative			prot_ID_240	Afu3g02670	AFUA_3G02670	ECS, IGD
AFUA_3G02680	-1	575	C6 finger domain protein, putative			prot_ID_1373	Afu3g02685	AFUA_3G02685	
AFUA_3G02685	-2	233	conserved hypothetical protein			prot_ID_241	Afu3g02680	AFUA_3G02680	
AFUA_3G02690	-3	968	conserved hypothetical protein			prot_ID_242	Afu3g02690	AFUA_3G02690	
AFUA_3G02700	-4	401	C6 transcription factor, putative			prot_ID_243	Afu3g02700	AFUA_3G02700	
AFUA_3G02710	-5	385	zinc-binding oxidoreductase, putative			prot_ID_244	Afu3g02710	AFUA_3G02710	
AFUA_3G02720	-6	345	MFS transporter, putative			prot_ID_245	Afu3g02720	AFUA_3G02720	ECS, IGD
AFUA_3G02740	-7	2728	calcium/calmodulin dependent protein kinase, putative			prot_ID_246	Afu3g02740	AFUA_3G02740	
AFUA_3G02750	-8	878	C6 transcription factor, putative			prot_ID_247	Afu3g02750	AFUA_3G02750	
AFUA_3G02760	-9	368	ABC multidrug transporter, putative			prot_ID_248	Afu3g02760	AFUA_3G02760	

Afu3g12920 cluster

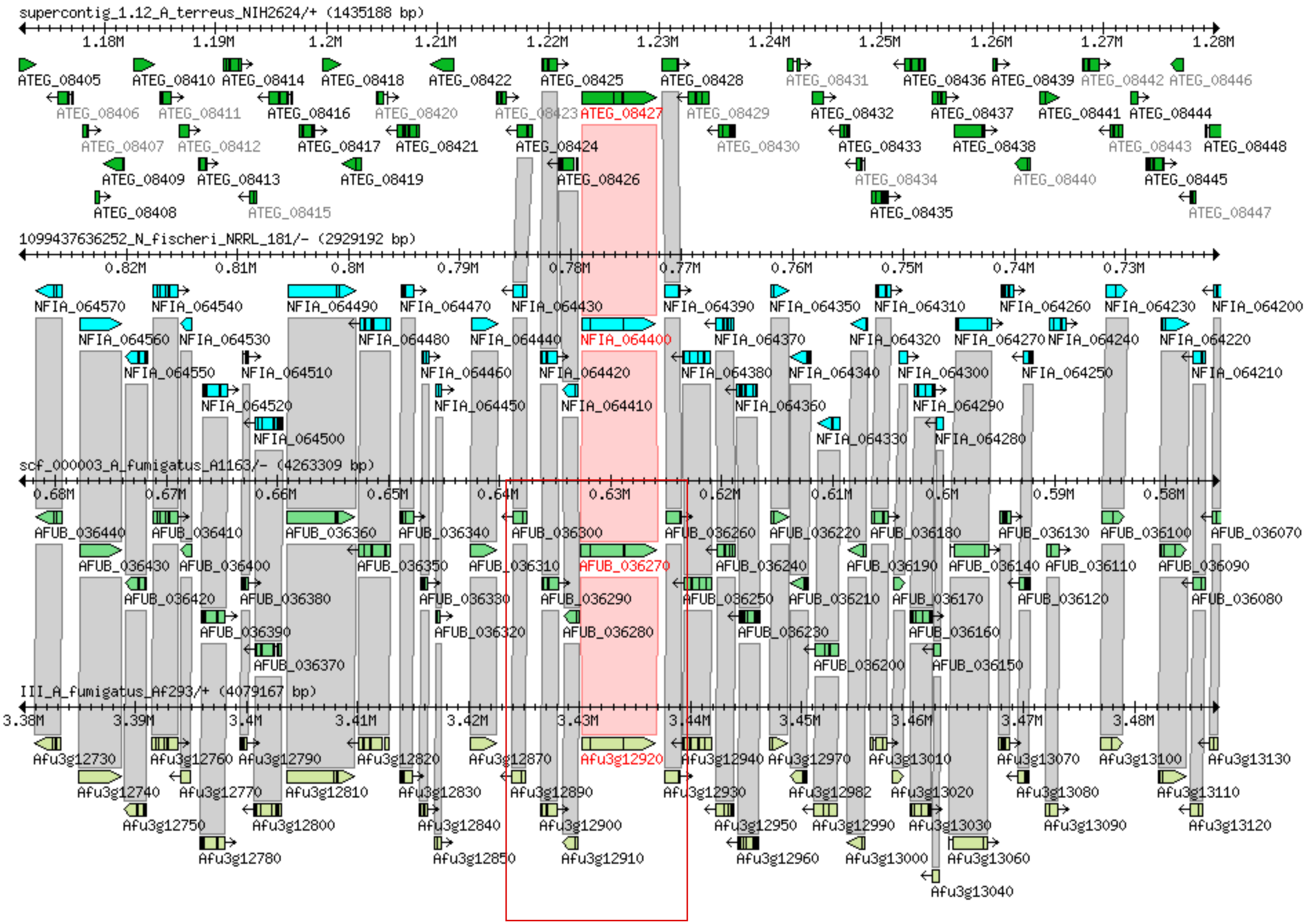

Afu3g12920 cluster

Gene id (SMURF)	Gene positions	Gene distance	Annotated_gene_function (SMURF)	AspGD gene name	AspGD supplementary annotation	antiSMASH ID	Gene ID (I)	Gene ID (II)	
n/a					Protein of unknown function	prot_ID_1122	Afu3g13020	AFUA_3G13020	
n/a					Hydrolase	prot_ID_1121	Afu3g13010	AFUA_3G13010	
n/a					Transmembrane transporter	prot_ID_1120	Afu3g13000	AFUA_3G13000	
n/a					Methyltransferase	prot_ID_1119	Afu3g12990	AFUA_3G12990	
n/a					Protein of unknown function	prot_ID_1359	Afu3g12982	AFUA_3G12982	
n/a					Protein of unknown function	prot_ID_1118	Afu3g12970	AFUA_3G12970	
AFUA_3G12960	4	344	cytochrome P450 monooxigenase GliC-like, putative			prot_ID_1117	Afu3g12960	AFUA_3G12960	ECS
AFUA_3G12950	3	477	FAD binding domain protein			prot_ID_1116	Afu3g12950	AFUA_3G12950	
AFUA_3G12940	2	223	C6 transcription factor, putative			prot_ID_1115	Afu3g12940	AFUA_3G12940	
AFUA_3G12930	1	694	dimethylallyl tryptophan synthase SirD-like, putative	7-dmats		**prot_ID_1114**	**Afu3g12930**	**AFUA_3G12930**	
**AFUA_3G12920**	0	0	nonribosomal peptide synthase GliP-like, putative	nrps5/pesF		**prot_ID_1113**	**Afu3g12920**	**AFUA_3G12920**	
AFUA_3G12910	-1	336	O-methyltransferase GliM-like, putative			prot_ID_1112	Afu3g12910	AFUA_3G12910	
AFUA_3G12900	-2	570	MFS transporter, putative			prot_ID_1111	Afu3g12900	AFUA_3G12900	
AFUA_3G12890	-3	1294	C6 transcription factor GliZ-like, putative			prot_ID_1110	Afu3g12890	AFUA_3G12890	ECS
AFUA_3G12870	-4	1390	GTPase activating protein (Gyp3), putative			prot_ID_1109	Afu3g12870	AFUA_3G12870	
AFUA_3G12850	-5	2601	ubiquitin conjugating enzyme (UbcB), putative			prot_ID_1108	Afu3g12850	AFUA_3G12850	
AFUA_3G12840	-6	604	Signal peptidase I			prot_ID_1107	Afu3g12840	AFUA_3G12840	
AFUA_3G12830	-7	639	RTA1 domain protein, putative			prot_ID_1106	Afu3g12830	AFUA_3G12830	
AFUA_3G12820	-8	984	kinesin family protein			prot_ID_1105	Afu3g12820	AFUA_3G12820	
AFUA_3G12810	-9	301	transcriptional corepressor of histone genes (Hir3), putative			n/a	Afu3g12810	AFUA_3G12810	
AFUA_3G12800	-10	479	clathrin-coated vesiclec protein (Bud7), putative			n/a	Afu3g12800	AFUA_3G12800	
AFUA_3G12790	-11	463	conserved glutamic acid-rich protein			n/a	Afu3g12790	AFUA_3G12790	
AFUA_3G12780	-12	1334	C6 transcription factor, putative			n/a	Afu3g12780	AFUA_3G12780	

AFUA_3G12770	-13	870	nucleoside-diphosphate-sugar epimerase, putative	n/a	Afu3g12770	AFUA_3G12770
AFUA_3G12760	-14	242	conserved hypothetical protein	n/a	Afu3g12760	AFUA_3G12760
AFUA_3G12750	-15	616	DNA repair protein Mus81, putative	n/a	Afu3g12750	AFUA_3G12750

Afu3g12930 cluster

Overlaps cluster above

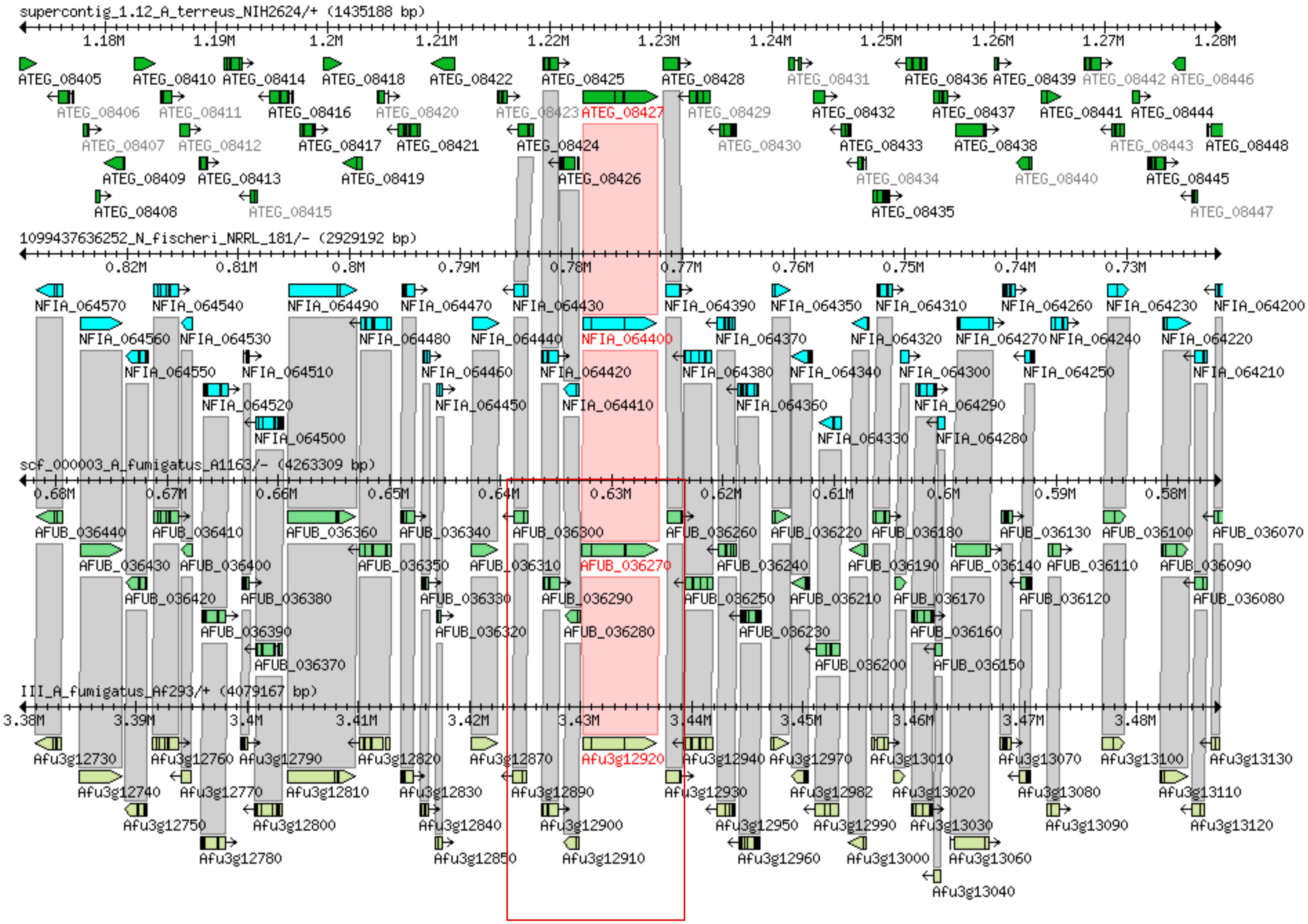

Afu3g12930 cluster

Gene id (SMURF)	Gene positions	Gene distance	Annotated_gene_function (SMURF)	AspGD gene name	AspGD supplementary annotation	antiSMASH ID	Gene ID (I)	Gene ID (II)	
n/a					Protein of unknown function	prot_ID_1122	Afu3g13020	AFUA_3G13020	
n/a					Hydrolase	prot_ID_1121	Afu3g13010	AFUA_3G13010	
AFUA_3G13000	7	900	mitochondrial chaperone BCS1, putative		Transmembrane transporter	prot_ID_1120	Afu3g13000	AFUA_3G13000	
AFUA_3G12990	6	637	conserved proline-rich protein		Methyltransferase	prot_ID_1119	Afu3g12990	AFUA_3G12990	
AFUA_3G12982	5	155	extracellular serine rich protein			prot_ID_1359	Afu3g12982	AFUA_3G12982	
AFUA_3G12970	4	963	DUF580 domain protein Pns1, putative			prot_ID_1118	Afu3g12970	AFUA_3G12970	
AFUA_3G12960	3	344	cytochrome P450 monooxigenase GliC-like, putative			prot_ID_1117	Afu3g12960	AFUA_3G12960	ECS
AFUA_3G12950	2	477	FAD binding domain protein			prot_ID_1116	Afu3g12950	AFUA_3G12950	
AFUA_3G12940	1	223	C6 transcription factor, putative			prot_ID_1115	Afu3g12940	AFUA_3G12940	
**AFUA_3G12930**	0	0	dimethylallyl tryptophan synthase SirD-like, putative	7-dmats		**prot_ID_1114**	**Afu3g12930**	**AFUA_3G12930**	
AFUA_3G12920	-1	694	nonribosomal peptide synthase GliP-like, putative	nrps5/pesF		**prot_ID_1113**	**Afu3g12920**	**AFUA_3G12920**	
AFUA_3G12910	-2	336	O-methyltransferase GliM-like, putative			prot_ID_1112	Afu3g12910	AFUA_3G12910	
AFUA_3G12900	-3	570	MFS transporter, putative			prot_ID_1111	Afu3g12900	AFUA_3G12900	
AFUA_3G12890	-4	1294	C6 transcription factor GliZ-like, putative			prot_ID_1110	Afu3g12890	AFUA_3G12890	ECS
AFUA_3G12870	-5	1390	GTPase activating protein (Gyp3), putative			prot_ID_1109	Afu3g12870	AFUA_3G12870	
AFUA_3G12850	-6	2601	ubiquitin conjugating enzyme (UbcB), putative			prot_ID_1108	Afu3g12850	AFUA_3G12850	
AFUA_3G12840	-7	604	Signal peptidase I			prot_ID_1107	Afu3g12840	AFUA_3G12840	
AFUA_3G12830	-8	639	RTA1 domain protein, putative			prot_ID_1106	Afu3g12830	AFUA_3G12830	
AFUA_3G12820	-9	984	kinesin family protein			prot_ID_1105	Afu3g12820	AFUA_3G12820	
AFUA_3G12810	-10	301	transcriptional corepressor of histone genes (Hir3), putative			n/a	Afu3g12810	AFUA_3G12810	
AFUA_3G12800	-11	479	clathrin-coated vesiclec protein (Bud7), putative			n/a	Afu3g12800	AFUA_3G12800	
AFUA_3G12790	-12	463	conserved glutamic acid-rich protein			n/a	Afu3g12790	AFUA_3G12790	
AFUA_3G12780	-13	1334	C6 transcription factor, putative			n/a	Afu3g12780	AFUA_3G12780	

AFUA_3G12770	-14	870	nucleoside-diphosphate-sugar epimerase, putative	n/a	Afu3g12770	AFUA_3G12770
AFUA_3G12760	-15	242	conserved hypothetical protein	n/a	Afu3g12760	AFUA_3G12760
AFUA_3G12750	-16	616	DNA repair protein Mus81, putative	n/a	Afu3g12750	AFUA_3G12750

Afu3g13730 cluster

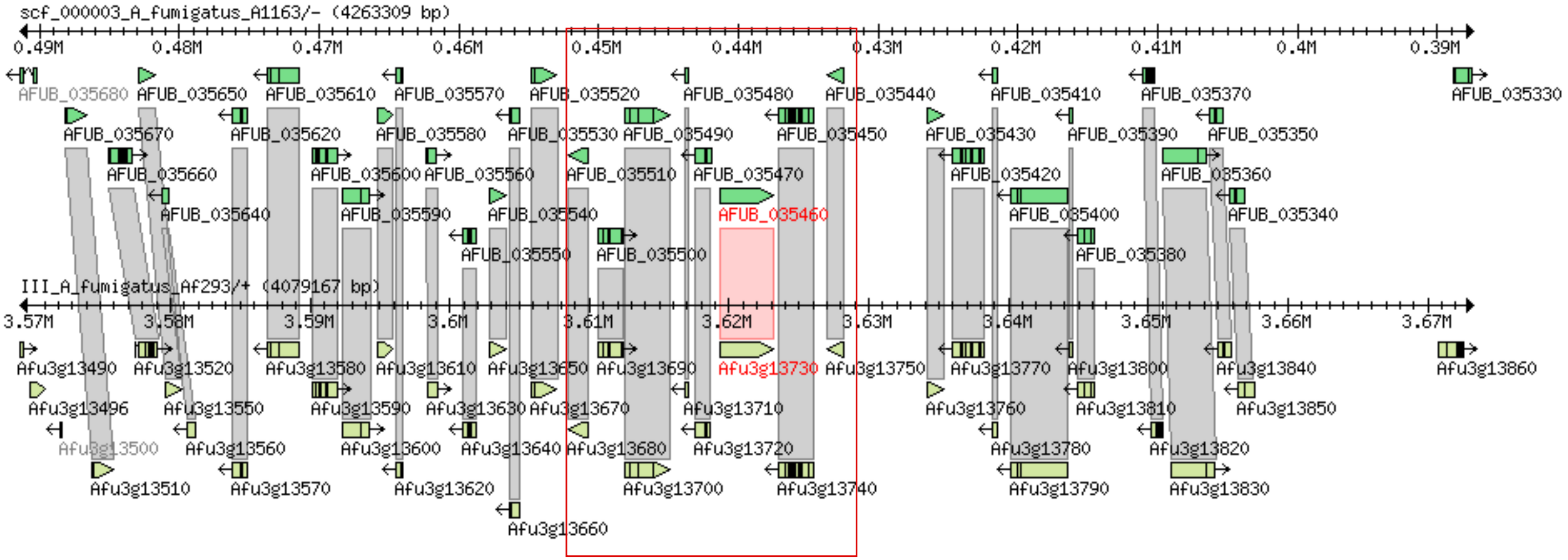

Afu3g13730 cluster

Gene id (SMURF)	Gene positions	Gene distance	Annotated_gene_function (SMURF)	AspGD gene name	AspGD supplementary annotation	antiSMASH ID	Gene ID (I)	Gene ID (II)	
n/a					Transmembrane transporter	prot_ID_1195	Afu3g13790	AFUA_3G13790	
n/a					Predicted adhesin-like protein	prot_ID_1194	Afu3g13780	AFUA_3G13780	
n/a					Vacuolar segregation protein	prot_ID_1193	Afu3g13770	AFUA_3G13770	
n/a					Protein of unknown function	prot_ID_1192	Afu3g13760	AFUA_3G13760	
n/a					Protein of unknown function	prot_ID_1191	Afu3g13750	AFUA_3G13750	IGD
n/a					Hsp70 family protein	prot_ID_1190	Afu3g13740	AFUA_3G13740	
**AFUA_3G13730**	0	0	nonribosomal peptide synthase, putative	nrps6/pesG		**prot_ID_1189**	**Afu3g13730**	**AFUA_3G13730**	
AFUA_3G13720	-1	636	oxidoreductase, 2OG-Fe(II) oxygenase family, putative			prot_ID_1188	Afu3g13720	AFUA_3G13720	
AFUA_3G13710	-2	432	GTP cyclohydrolase I, putative			prot_ID_1187	Afu3g13710	AFUA_3G13710	
AFUA_3G13700	-3	1066	transferase family protein			prot_ID_1186	Afu3g13700	AFUA_3G13700	
AFUA_3G13690	-4	197	pyoverdine/dityrosine biosynthesis family protein, putative			prot_ID_1185	Afu3g13690	AFUA_3G13690	
AFUA_3G13680	-5	747	conserved hypothetical protein			prot_ID_1184	Afu3g13680	AFUA_3G13680	
AFUA_3G13670	-6	791	siderochrome-iron transporter, putative			prot_ID_1183	Afu3g13670	AFUA_3G13670	FA
AFUA_3G13660	-7	938	Ctr copper transporter family protein			prot_ID_1182	Afu3g13660	AFUA_3G13660	
AFUA_3G13650	-8	230	integral membrane protein			prot_ID_1181	Afu3g13650	AFUA_3G13650	
AFUA_3G13640	-9	967	extracellular serine-rich protein, putative			prot_ID_1180	Afu3g13640	AFUA_3G13640	
AFUA_3G13630	-10	1856	conserved hypothetical protein			prot_ID_1179	Afu3g13630	AFUA_3G13630	
AFUA_3G13620	-11	1892	cupin domain protein			prot_ID_1178	Afu3g13620	AFUA_3G13620	
AFUA_3G13610	-12	203	oxidoreductase, zinc-binding, putative			prot_ID_1177	Afu3g13610	AFUA_3G13610	
AFUA_3G13600	-13	488	C6 transcription factor, putative			prot_ID_1176	Afu3g13600	AFUA_3G13600	

Afu3g14700 cluster

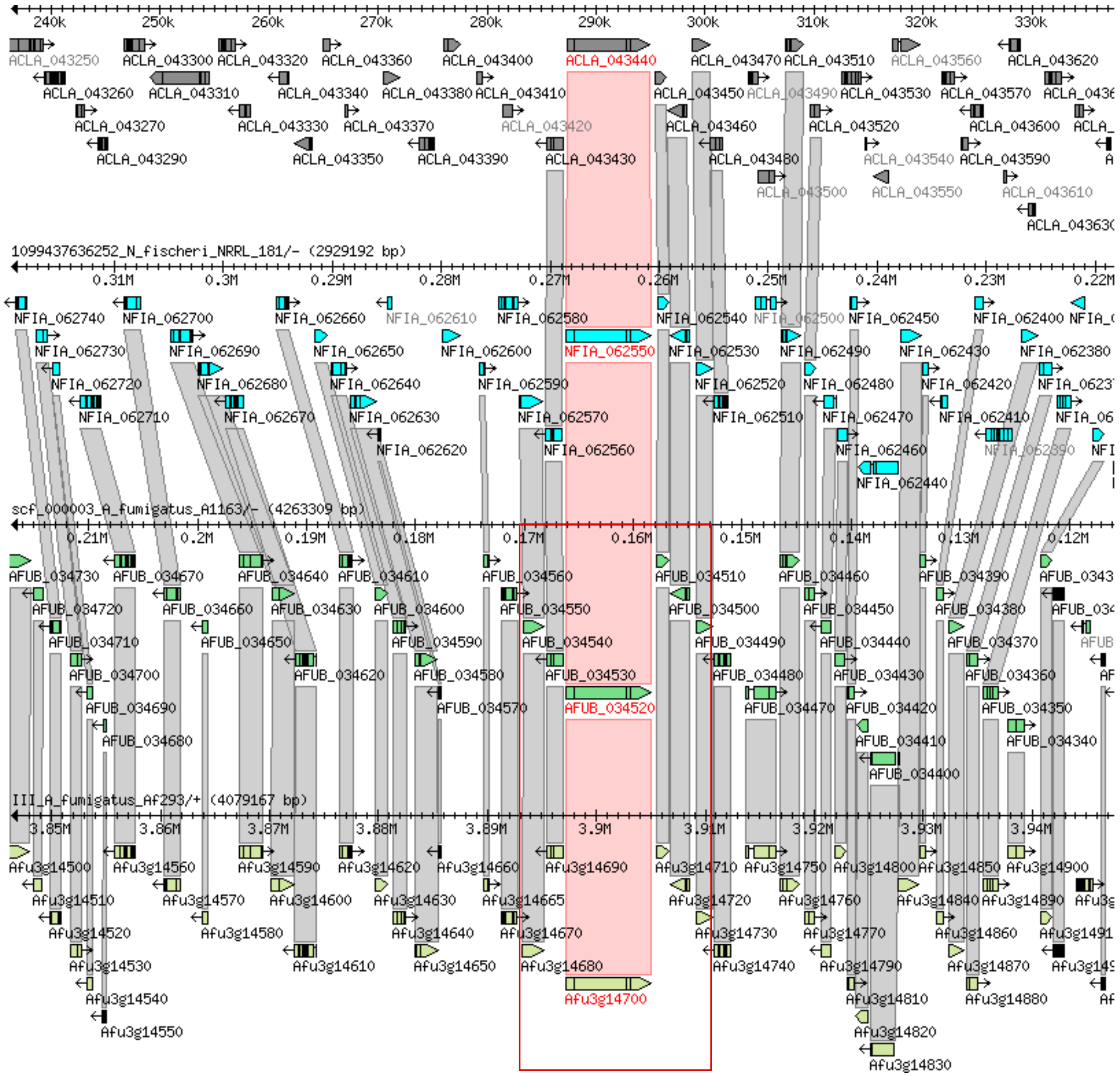

Afu3g14700 cluster

Gene id (SMURF)	Gene positions	Gene distance	Annotated_gene_function (SMURF)	AspGD gene name	AspGD supplementary annotation	antiSMASH ID	Gene ID (I)	Gene ID (II)	
AFUA_3G14880	17	170	oxidoreductase, 2OG-Fe(II) oxygenase family, putative						
AFUA_3G14870	16	456	conserved hypothetical protein						
AFUA_3G14860	15	1033	conserved hypothetical protein						
AFUA_3G14850	14	139	3-dehydroquinate dehydratase, type II						
AFUA_3G14840	13	204	C6 transcription factor, putative						
AFUA_3G14830	12	269	pentafunctional AROM polypeptide, putative						
AFUA_3G14820	11	46	4-hydroxyphenylpyruvate dioxygenase, putative			prot_ID_1293	Afu3g14820	AFUA_3G14820	
AFUA_3G14810	10	202	pathogenesis associated protein Pep2, putative			prot_ID_1292	Afu3g14810	AFUA_3G14810	
AFUA_3G14800	9	427	shikimate 5-dehydrogenase, putative			prot_ID_1291	Afu3g14800	AFUA_3G14800	
AFUA_3G14790	8	455	pentafunctional AROM polypeptide, putative			prot_ID_1290	Afu3g14790	AFUA_3G14790	
AFUA_3G14770	7	464	reductase, putative			prot_ID_1289	Afu3g14770	AFUA_3G14770	
AFUA_3G14760	6	401	cytochrome P450 monooxygenase (Fum15), putative			prot_ID_1288	Afu3g14760	AFUA_3G14760	
AFUA_3G14750	5	1366	fungal specific transcription factor, putative			prot_ID_1287	Afu3g14750	AFUA_3G14750	
AFUA_3G14740	4	121	conserved hypothetical protein			prot_ID_1286	Afu3g14740	AFUA_3G14740	
AFUA_3G14730	3	605	LysR family regulatory protein, putative			prot_ID_1285	Afu3g14730	AFUA_3G14730	ECS
AFUA_3G14720	2	75	MFS transporter, putative			prot_ID_1284	Afu3g14720	AFUA_3G14720	
AFUA_3G14710	1	577	phytanoyl-CoA dioxygenase family protein			prot_ID_1283	Afu3g14710	AFUA_3G14710	
**AFUA_3G14700**	0	0	polyketide synthase, putative			**prot_ID_1282**	**Afu3g14700**	**AFUA_3G14700**	
AFUA_3G14690	-1	320	aminotransferase, putative			prot_ID_1281	Afu3g14690	AFUA_3G14690	FA
n/a				plb3	Secreted lysophospholipase B	prot_ID_1280	Afu3g14680	AFUA_3G14680	
n/a					Transmembrane transporter	prot_ID_1279	Afu3g14670	AFUA_3G14670	
n/a					Protein of unknown function	prot_ID_1278	Afu3g14665	AFUA_3G14665	
n/a					Protein of unknown function	prot_ID_1277	Afu3g14660	AFUA_3G14660	
n/a					Protein of unknown function	prot_ID_1276	Afu3g14650	AFUA_3G14650	

n/a	ssu2	C4-dicarboxylate transporter/malic acidtransport protein	prot_ID_1275	Afu3g14640	AFUA_3G14640
n/a		Oxidoreductase	prot_ID_1274	Afu3g14630	AFUA_3G14630
n/a		Predicted adhesin-like protein; ortholog of A. nidulans abnA	prot_ID_1273	Afu3g14620	AFUA_3G14620

Afu3g15270 cluster

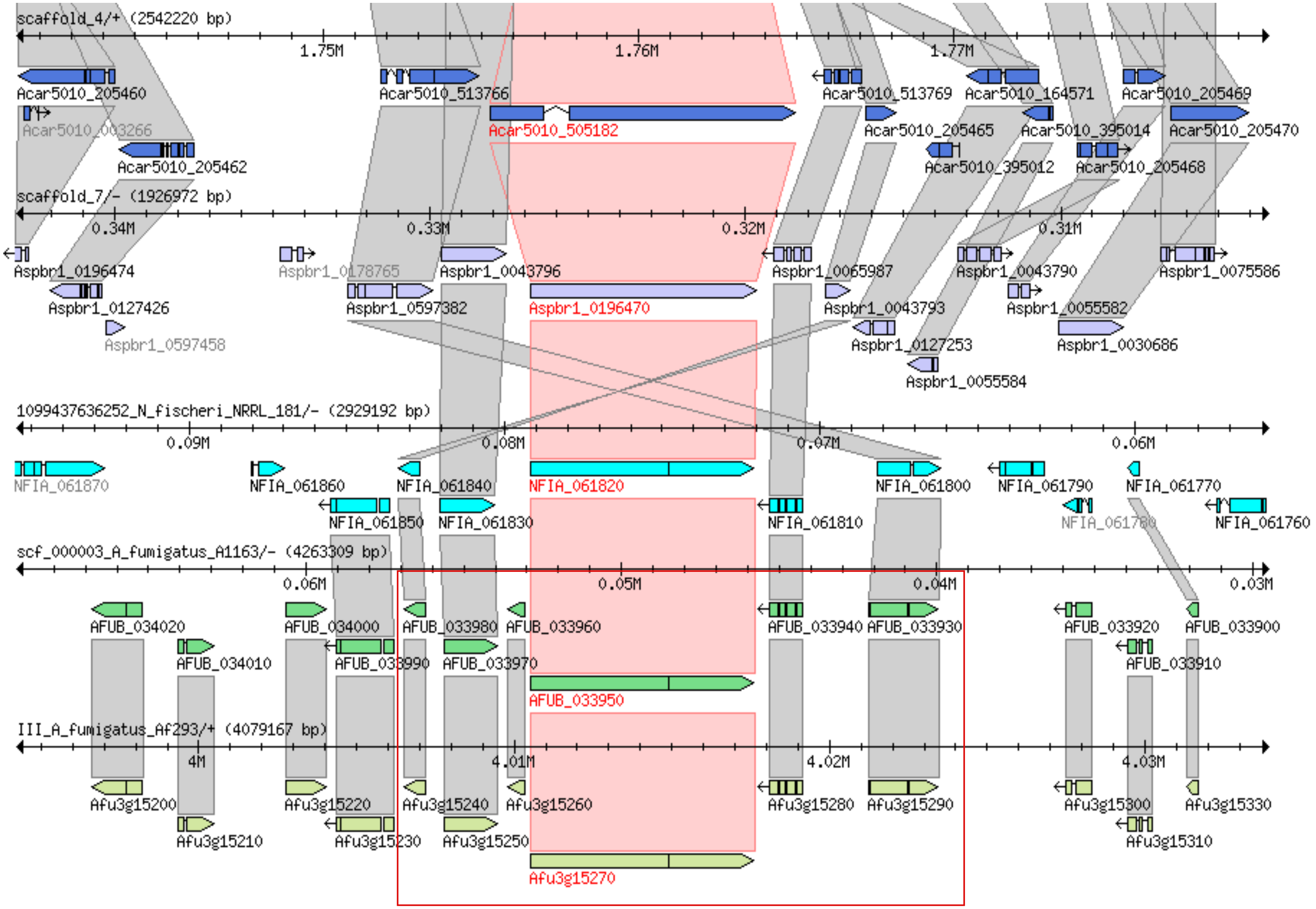

Afu3g15270 cluster

Gene id (SMURF)	Gene positions	Gene distance	Annotated_gene_function (SMURF)	AspGD gene name	AspGD supplementary annotation	antiSMASH ID	Gene ID (I)	Gene ID (II)	
n/a					Oxidoreductase	prot_ID_1342	Afu3g15350	AFUA_3G15350	
n/a					Putative transcription factor	prot_ID_1341	Afu3g15340	AFUA_3G15340	
n/a					Protein of unknown function	prot_ID_1340	Afu3g15330	AFUA_3G15330	
n/a					C-methyl transferase	prot_ID_1339	Afu3g15310	AFUA_3G15310	
n/a					Transmembrane transporter	prot_ID_1338	Afu3g15300	AFUA_3G15300	
AFUA_3G15290	2	2090	C6 transcription factor, putative			prot_ID_1337	Afu3g15290	AFUA_3G15290	ECS, IGD
AFUA_3G15280	1	453	methyltransferase, putative			prot_ID_1336	Afu3g15280	AFUA_3G15280	
**AFUA_3G15270**	0	0	nonribosomal peptide synthase, putative	nrps7/pesH		**prot_ID_1335**	**Afu3g15270**	**AFUA_3G15270**	
AFUA_3G15260	-1	202	hypothetical protein			prot_ID_1334	Afu3g15260	AFUA_3G15260	
AFUA_3G15250	-2	321	MFS drug efflux transporter, putative			prot_ID_1333	Afu3g15250	AFUA_3G15250	
n/a					Carbon-nitrogen ligase	prot_ID_1332	Afu3g15240	AFUA_3G15240	ECS
n/a					Protein of unknown function	prot_ID_1331	Afu3g15230	AFUA_3G15230	
n/a						prot_ID_1330	Afu3g15220	AFUA_3G15220	
n/a						prot_ID_1329	Afu3g15210	AFUA_3G15210	
n/a						prot_ID_1328	Afu3g15200	AFUA_3G15200	
n/a						prot_ID_1327	Afu3g15190	AFUA_3G15190	

Afu4g00210 cluster

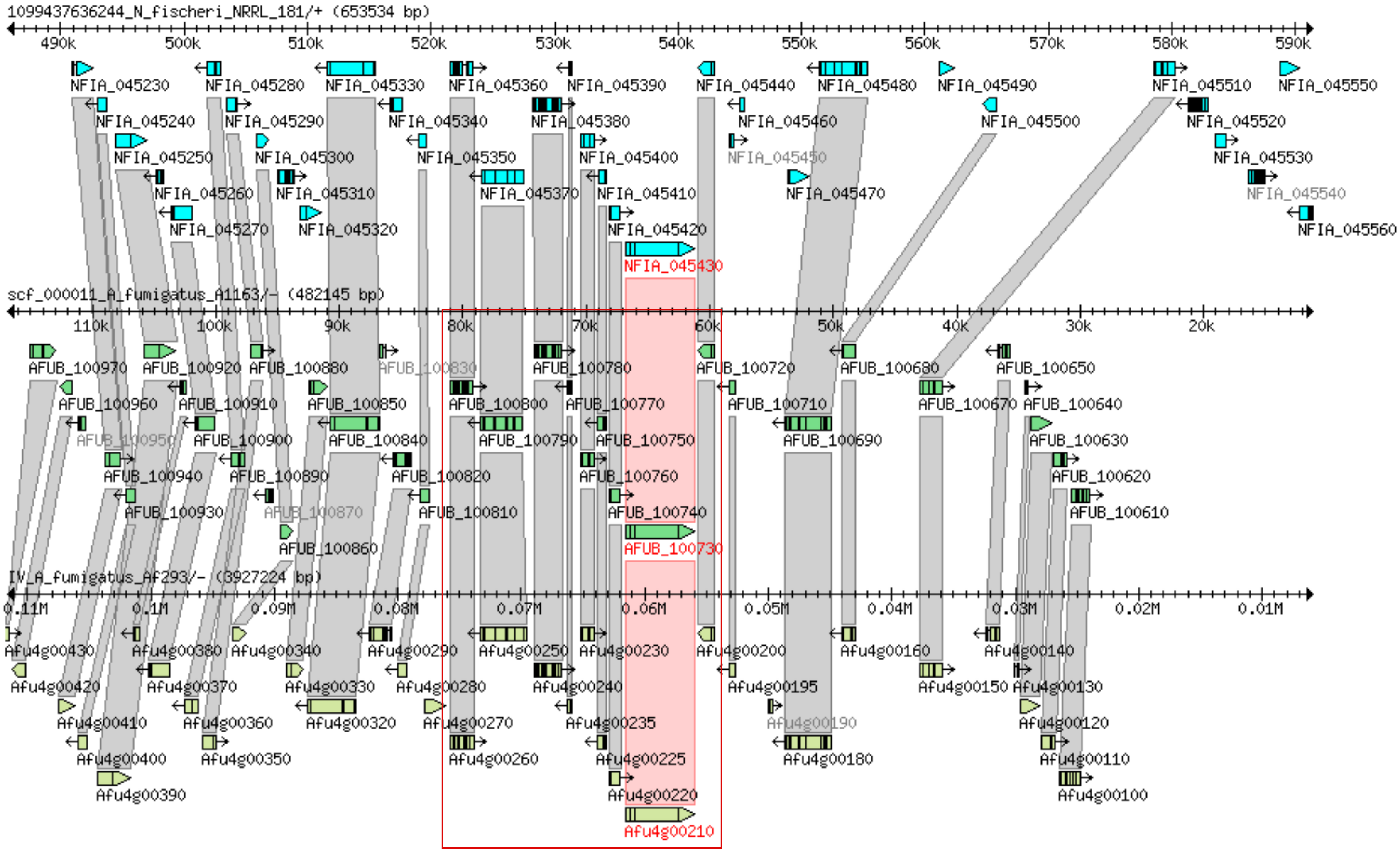

Afu4g00210 cluster

Gene id (SMURF)	Gene positions	Gene distance	Annotated_gene_function (SMURF)	AspGD gene name	AspGD supplementary annotation	antiSMASH ID	Gene ID (I)	Gene ID (II)	
n/a					Succinyl-CoA synthetase beta subunit	prot_ID_19	Afu4g00290	AFUA_4G00290	
n/a					Protein of unknown function	prot_ID_18	Afu4g00280	AFUA_4G00280	
n/a					Protein of unknown function	prot_ID_17	Afu4g00270	AFUA_4G00270	
AFUA_4G00260	7	555	MFS transporter, putative			prot_ID_16	Afu4g00260	AFUA_4G00260	IGD
AFUA_4G00250	6	625	WD40 repeat protein			prot_ID_15	Afu4g00250	AFUA_4G00250	
AFUA_4G00240	5	445	amino acid transporter, putative			prot_ID_14	Afu4g00240	AFUA_4G00240	
AFUA_4G00235	4	675	conserved hypothetical protein			prot_ID_1223	Afu4g00235	AFUA_4G00235	
AFUA_4G00230	3	306	oxidoreductase, 2OG-Fe(II) oxygenase family, putative			prot_ID_13	Afu4g00230	AFUA_4G00230	
AFUA_4G00225	2	313	DUF1772 domain protein			prot_ID_1242	Afu4g00225	AFUA_4G00225	
AFUA_4G00220	1	365	metallo-beta-lactamase domain protein			prot_ID_12	Afu4g00220	AFUA_4G00220	
**AFUA_4G00210**	0	0	polyketide synthase, putative			**prot_ID_11**	**Afu4g00210**	**AFUA_4G00210**	
n/a					F-box domain protein	prot_ID_10	Afu4g00200	AFUA_4G00200	ECS
n/a					Protein of unknown function	prot_ID_1198	Afu4g00195	AFUA_4G00195	
n/a					Protein of unknown function	prot_ID_9	Afu4g00190	AFUA_4G00190	
n/a					Fatty acid 8,11-diol synthase	prot_ID_8	Afu4g00180	AFUA_4G00180	
n/a					Oxidoreductase	prot_ID_7	Afu4g00160	AFUA_4G00160	
n/a					MFS maltose transporter	prot_ID_6	Afu4g00150	AFUA_4G00150	

Afu4g14560 cluster

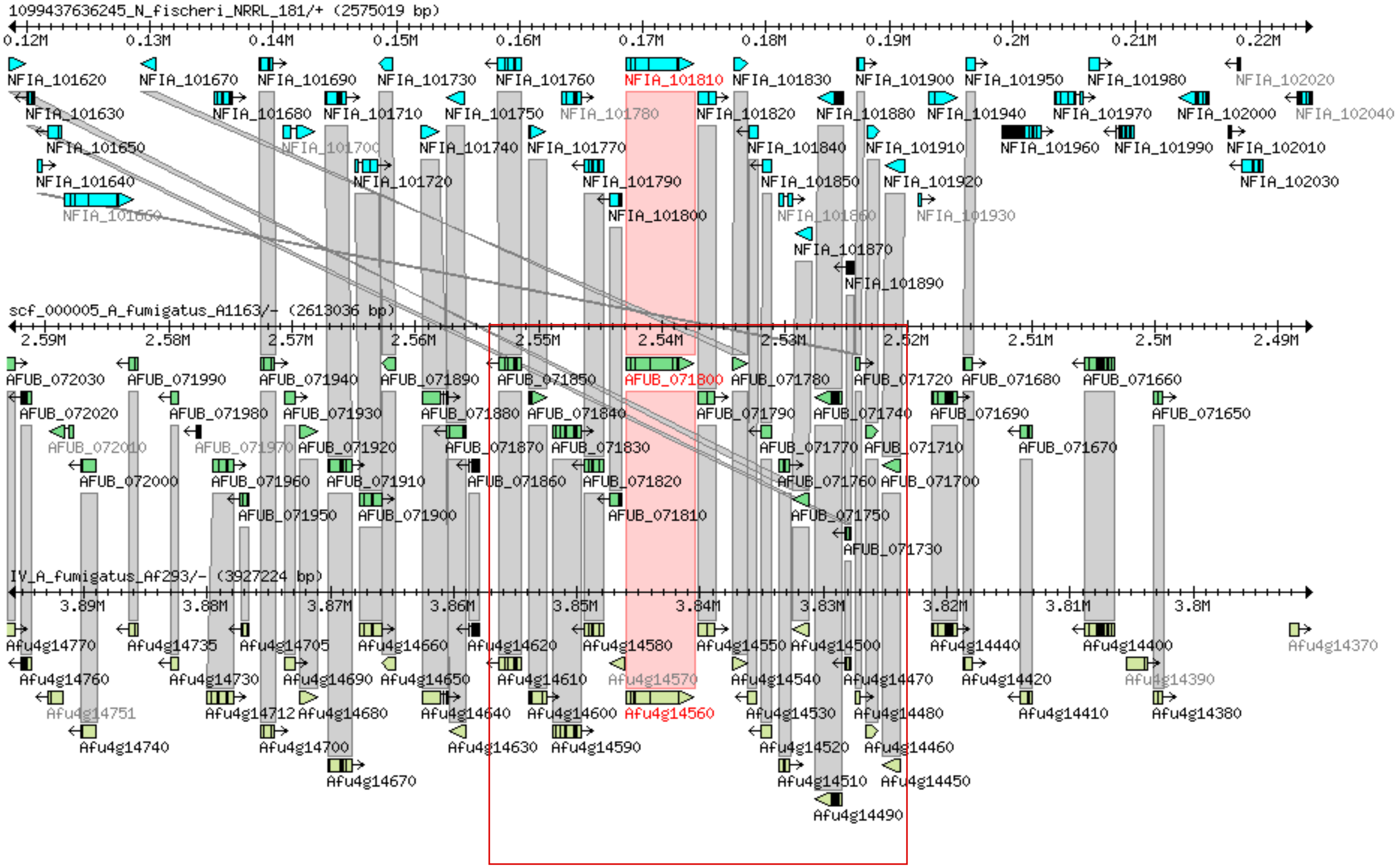

Afu4g14560 cluster

Gene id (SMURF)	Gene positions	Gene distance	Annotated_gene_function (SMURF)	AspGD gene name	AspGD supplementary annotation	antiSMASH ID	Gene ID (I)	Gene ID (II)	
AFUA_4G14730	17	2314	GNAT family acetyltransferase, putative			n/a	Afu4g14730	AFUA_4G14730	
AFUA_4G14712	16	514	C6 transcription factor, putative			n/a	Afu4g14712	AFUA_4G14712	
AFUA_4G14705	15	968	hypothetical protein			n/a	Afu4g14705	AFUA_4G14705	
AFUA_4G14700	14	732	GDSL Lipase/Acylhydrolase family protein			n/a	Afu4g14700	AFUA_4G14700	
AFUA_4G14690	13	352	hypothetical protein			n/a	Afu4g14690	AFUA_4G14690	
AFUA_4G14680	12	828	C6 transcription factor, putative			n/a	Afu4g14680	AFUA_4G14680	
AFUA_4G14670	11	588	MFS quinate transporter, putative			n/a	Afu4g14670	AFUA_4G14670	
AFUA_4G14660	10	31	conserved hypothetical protein			prot_ID_1174	Afu4g14660	AFUA_4G14660	
AFUA_4G14650	9	2222	ribokinase			prot_ID_1173	Afu4g14650	AFUA_4G14650	
AFUA_4G14640	8	66	low affinity iron transporter, putative			prot_ID_1172	Afu4g14640	AFUA_4G14640	
AFUA_4G14630	7	189	FAD binding oxidoreductase, putative			prot_ID_1171	Afu4g14630	AFUA_4G14630	
AFUA_4G14620	6	1527	RING finger protein			prot_ID_1170	Afu4g14620	AFUA_4G14620	
AFUA_4G14610	5	625	MFS sugar transporter, putative			prot_ID_1169	Afu4g14610	AFUA_4G14610	ECS
AFUA_4G14600	4	541	UPF0075 domain protein			prot_ID_1168	Afu4g14600	AFUA_4G14600	
AFUA_4G14590	3	242	C6 transcription factor, putative			prot_ID_1167	Afu4g14590	AFUA_4G14590	
AFUA_4G14580	2	401	O-methyltransferase, putative			prot_ID_1166	Afu4g14580	AFUA_4G14580	
AFUA_4G14570	1	139	metallo-beta-lactamase domain protein, putative			prot_ID_1165	Afu4g1457	AFUA_4G14570	
**AFUA_4G14560**	0	0	polyketide synthase, putative			**prot_ID_1164**	**Afu4g14560**	**AFUA_4G14560**	
AFUA_4G14550	-1	306	O-methyltransferase family protein			prot_ID_1163	Afu4g14550	AFUA_4G14550	
AFUA_4G14540	-2	1372	C6 transcription factor (AflR), putative			prot_ID_1162	Afu4g14540	AFUA_4G14540	
AFUA_4G14530	-3	90	glutathione S-transferase Ure2-like, putative			prot_ID_1161	Afu4g14530	AFUA_4G14530	
AFUA_4G14520	-4	418	NAD-dependent epimerase/dehydratase, putative			prot_ID_1160	Afu4g14520	AFUA_4G14520	
AFUA_4G14510	-5	587	hypothetical protein			prot_ID_1159	Afu4g14510	AFUA_4G14510	
AFUA_4G14500	-6	161	conserved hypothetical protein			prot_ID_1158	Afu4g14500	AFUA_4G14500	
AFUA_4G14490	-7	490	extracellular dihydrogeodin oxidase/laccase, putative			prot_ID_1157	Afu4g14490	AFUA_4G14490	

AFUA_4G14470	-8	243	hypothetical protein		prot_ID_1155	Afu4g14470	AFUA_4G14470	
AFUA_4G14480	-9	275	conserved hypothetical protein		prot_ID_1156	Afu4g14480	AFUA_4G14480	
AFUA_4G14460	-10	354	conserved hypothetical protein		prot_ID_1154	Afu4g14460	AFUA_4G14460	
AFUA_4G14450	-11	399	mannitol dehydrogenase, putative		prot_ID_1153	Afu4g14450	AFUA_4G14450	ECS
AFUA_4G14440	-12	2440	NRPS-like enzyme, putative		prot_ID_1152	Afu4g14440	AFUA_4G14440	Adjacent NRPS
AFUA_4G14420	-13	532	secreted glycosyl hydrolase, putative	Secreted glycosyl hydrolase	n/a	Afu4g14420	AFUA_4G14420	

Afu5g10120 cluster

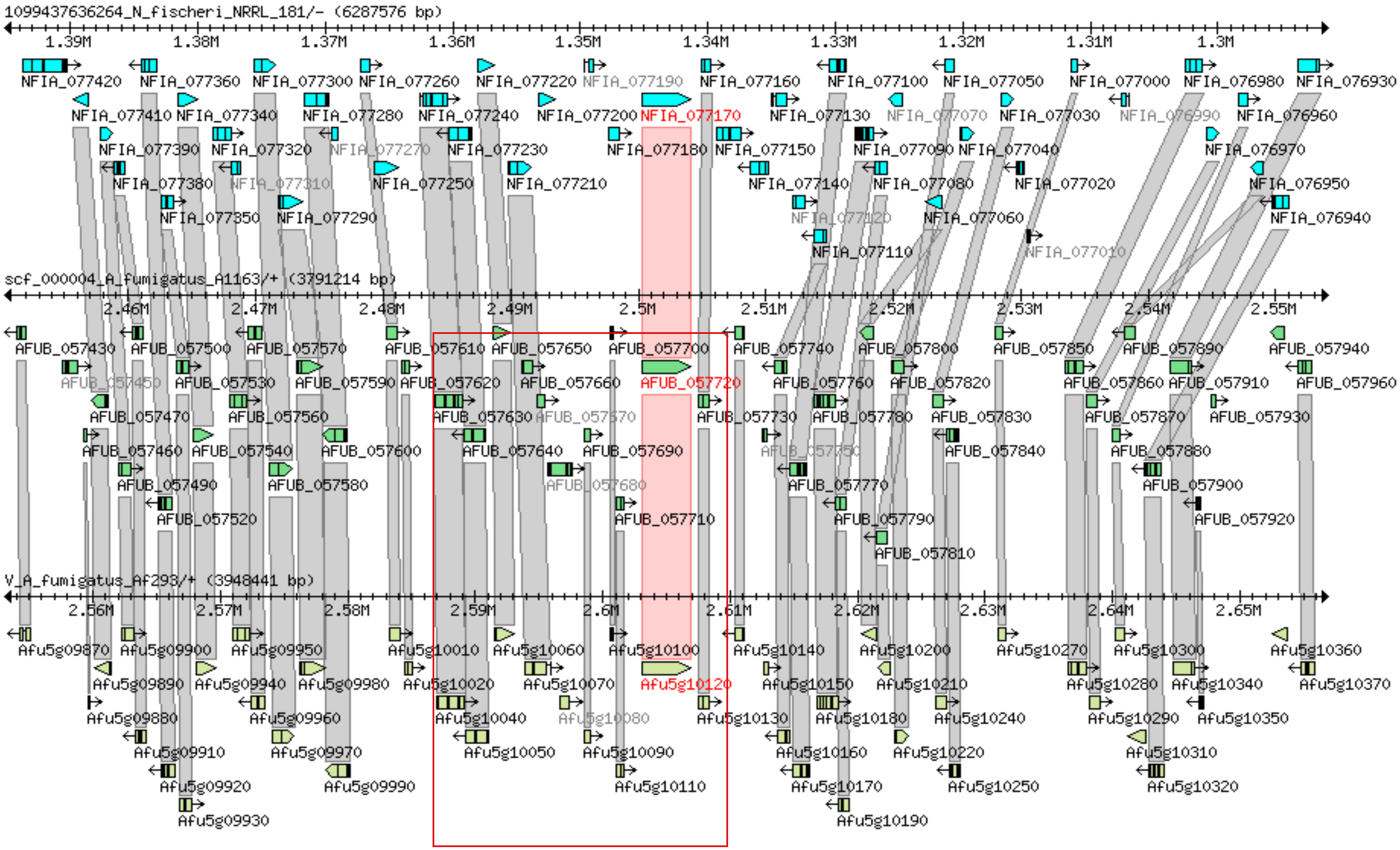

Afu5g10120 cluster

Gene id (SMURF)	Gene positions	Gene distance	Annotated_gene_function (SMURF)	AspGD gene name	AspGD supplementary annotation	antiSMASH ID	Gene ID (I)	Gene ID (II)	
AFUA_5G10250	12	200	conserved hypothetical protein			n/a	Afu5g10250	AFUA_5G10250	
AFUA_5G10240	11	2079	conserved hypothetical protein			prot_ID_873	Afu5g10240	AFUA_5G10240	
AFUA_5G10220	10	407	alcohol dehydrogenase, zinc-containing, putative			prot_ID_872	Afu5g10220	AFUA_5G10220	
AFUA_5G10210	9	153	conserved hypothetical protein			prot_ID_871	Afu5g10210	AFUA_5G10210	
AFUA_5G10200	8	980	hypothetical protein			prot_ID_870	Afu5g10200	AFUA_5G10200	
AFUA_5G10190	7	25	conserved hypothetical protein			prot_ID_869	Afu5g10190	AFUA_5G10190	
AFUA_5G10180	6	636	salicylate hydroxylase, putative			prot_ID_868	Afu5g10180	AFUA_5G10180	
AFUA_5G10170	5	218	pectin lyase			prot_ID_867	Afu5g10170	AFUA_5G10170	
AFUA_5G10160	4	621	NmrA-like family protein			prot_ID_866	Afu5g10160	AFUA_5G10160	
AFUA_5G10150	3	1509	hypothetical protein			prot_ID_865	Afu5g10150	AFUA_5G10150	
AFUA_5G10140	2	1984	MFS multidrug transporter, putative			prot_ID_864	Afu5g10140	AFUA_5G10140	
AFUA_5G10130	1	650	bZIP transcription factor, putative			prot_ID_863	Afu5g10130	AFUA_5G10130	ECS
**AFUA_5G10120**	0	0	NRPS-like enzyme, putative			**prot_ID_862**	**Afu5g10120**	**AFUA_5G10120**	
AFUA_5G10110	-1	1424	conserved hypothetical protein			prot_ID_861	Afu5g10110	AFUA_5G10110	
AFUA_5G10100	-2	177	conserved hypothetical protein			prot_ID_860	Afu5g10100	AFUA_5G10100	
AFUA_5G10090	-3	1526	3-demethylubiquinone-9 3-methyltransferase, putative			prot_ID_859	Afu5g10090	AFUA_5G10090	
AFUA_5G10080	-4	1027	conserved hypothetical protein			prot_ID_858	Afu5g10080	AFUA_5G10080	
AFUA_5G10070	-5	721	3-hydroxyacyl-CoA dehydrogenase, putative			prot_ID_857	Afu5g10070	AFUA_5G10070	
AFUA_5G10060	-6	843	cytochrome b5 reductase, putative			prot_ID_856	Afu5g10060	AFUA_5G10060	
AFUA_5G10050	-7	484	cytochrome P450 monooxygenase, putative			prot_ID_855	Afu5g10050	AFUA_5G10050	
AFUA_5G10040	-8	81	C6 transcription factor, putative			prot_ID_854	Afu5g10040	AFUA_5G10040	FA
AFUA_5G10020	-9	1897	sensor histidine kinase/response regulator, putative			prot_ID_853	Afu5g10020	AFUA_5G10020	
AFUA_5G10010	-10	350	extracellular serine-threonine rich protein			prot_ID_852	Afu5g10010	AFUA_5G10010	
AFUA_5G09990	-11	3165	C6 transcription factor, putative			n/a	Afu5g09990	AFUA_5G09990	
AFUA_5G09980	-12	68	acyl-CoA dehydrogenase, putative			n/a	Afu5g09980	AFUA_5G09980	

AFUA_5G09970	-13	267	67 kDa myosin-cross-reactive antigen family protein	n/a	Afu5g09970	AFUA_5G09970

Afu5g12730 cluster

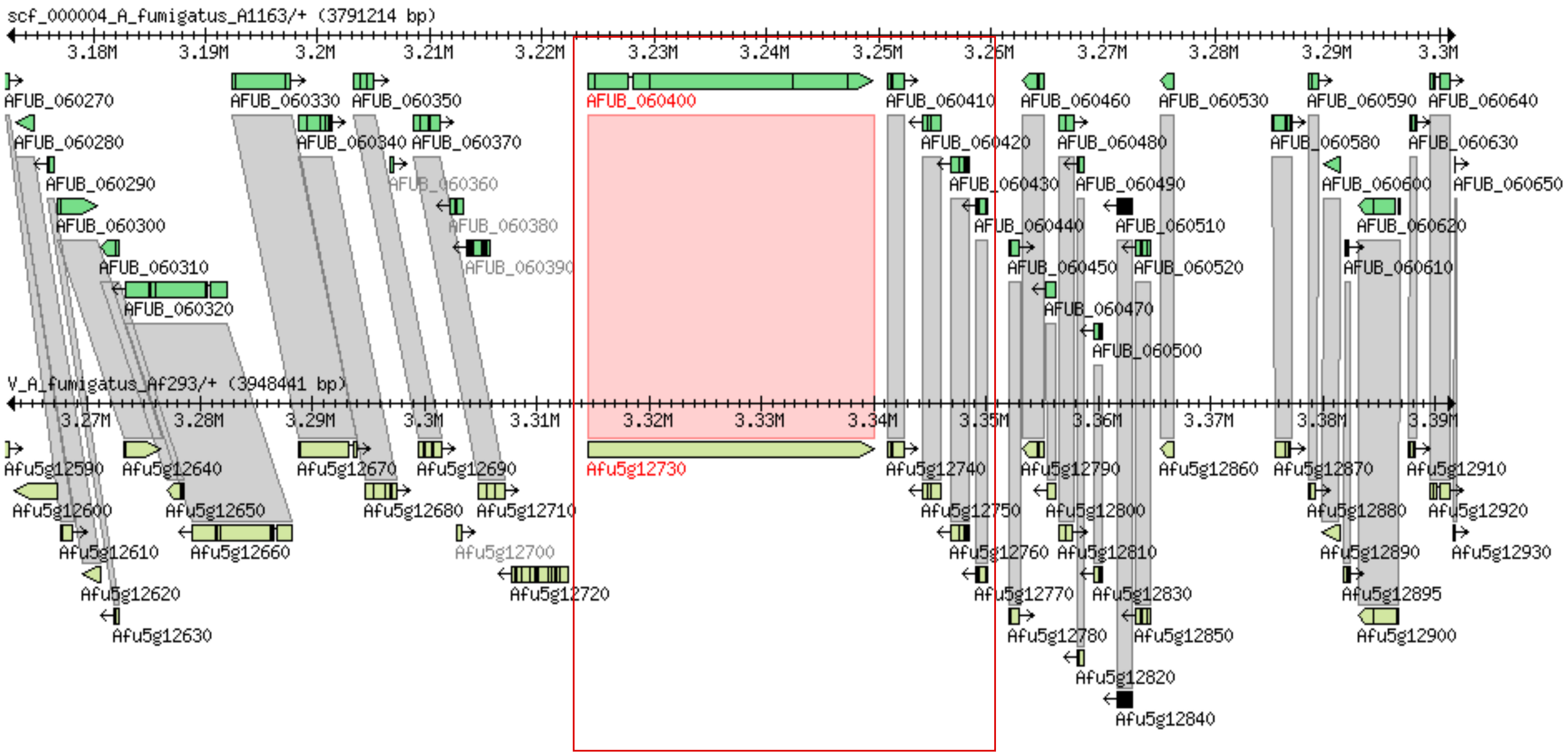

Afu5g12730 cluster

Gene id (SMURF)	Gene positions	Gene distance	Annotated_gene_function (SMURF)	AspGD gene name	AspGD supplementary annotation	antiSMASH ID	Gene ID (I)	Gene ID (II)	
n/a					Protein of unkown function	n/a	Afu5g12860	AFUA_5G12860	
n/a					Cell morphogenesis protein Las1, putative	n/a	Afu5g12850	AFUA_5G12850	
AFUA_5G12840	11	1361	hydroxyacylglutathione hydrolase, putative			n/a	Afu5g12840	AFUA_5G12840	
AFUA_5G12830	10	888	DUF1264 domain protein			n/a	Afu5g12830	AFUA_5G12830	
AFUA_5G12820	9	179	mitochondrial inner membrane protease subunit 1, putative			prot_ID_1113	Afu5g12830	AFUA_5G12830	
AFUA_5G12810	8	405	mitochondrial large ribosomal subunit YmL35, putative			prot_ID_1111	Afu5g12810	AFUA_5G12810	
AFUA_5G12800	7	230	50S ribosomal protein L4			prot_ID_1110	Afu5g12800	AFUA_5G12800	
AFUA_5G12790	6	185	mitochondrial 3-hydroxyisobutyryl-CoA hydrolase, putative			prot_ID_1109	Afu5g12790	AFUA_5G12790	
AFUA_5G12780	5	1936	Kelch repeat protein			prot_ID_1108	Afu5g12780	AFUA_5G12780	
AFUA_5G12770	4	675	metallo-beta-lactamase family protein			prot_ID_1107	Afu5g12770	AFUA_5G12770	FA
AFUA_5G12760	3	968	CCCH zinc finger DNA binding protein			prot_ID_1106	Afu5g12760	AFUA_5G12760	
AFUA_5G12750	2	1540	hypothetical protein			prot_ID_1105	Afu5g12750	AFUA_5G12750	
AFUA_5G12740	1	1153	MFS multidrug transporter, putative			prot_ID_1104	Afu5g12740	AFUA_5G12740	
**AFUA_5G12730**	0	0	nonribosomal peptide synthase, putative	nrps8/pesI		**prot_ID_1103**	**Afu5g12730**	**AFUA_5G12730**	ECS
AFUA_5G12720	-1	1815	ABC multidrug transporter, putative			prot_ID_1102	Afu5g12720	AFUA_5G12720	
n/a						prot_ID_1101	Afu5g12710	AFUA_5G12710	
n/a						prot_ID_1100	Afu5g12700	AFUA_5G12700	
n/a					Dihydroxyacetone kinase	prot_ID_1099	Afu5g12690	AFUA_5G12690	
n/a					Ubiquitin ligase subunit CulD	prot_ID_1098	Afu5g12680	AFUA_5G12680	

Afu6g03480 cluster

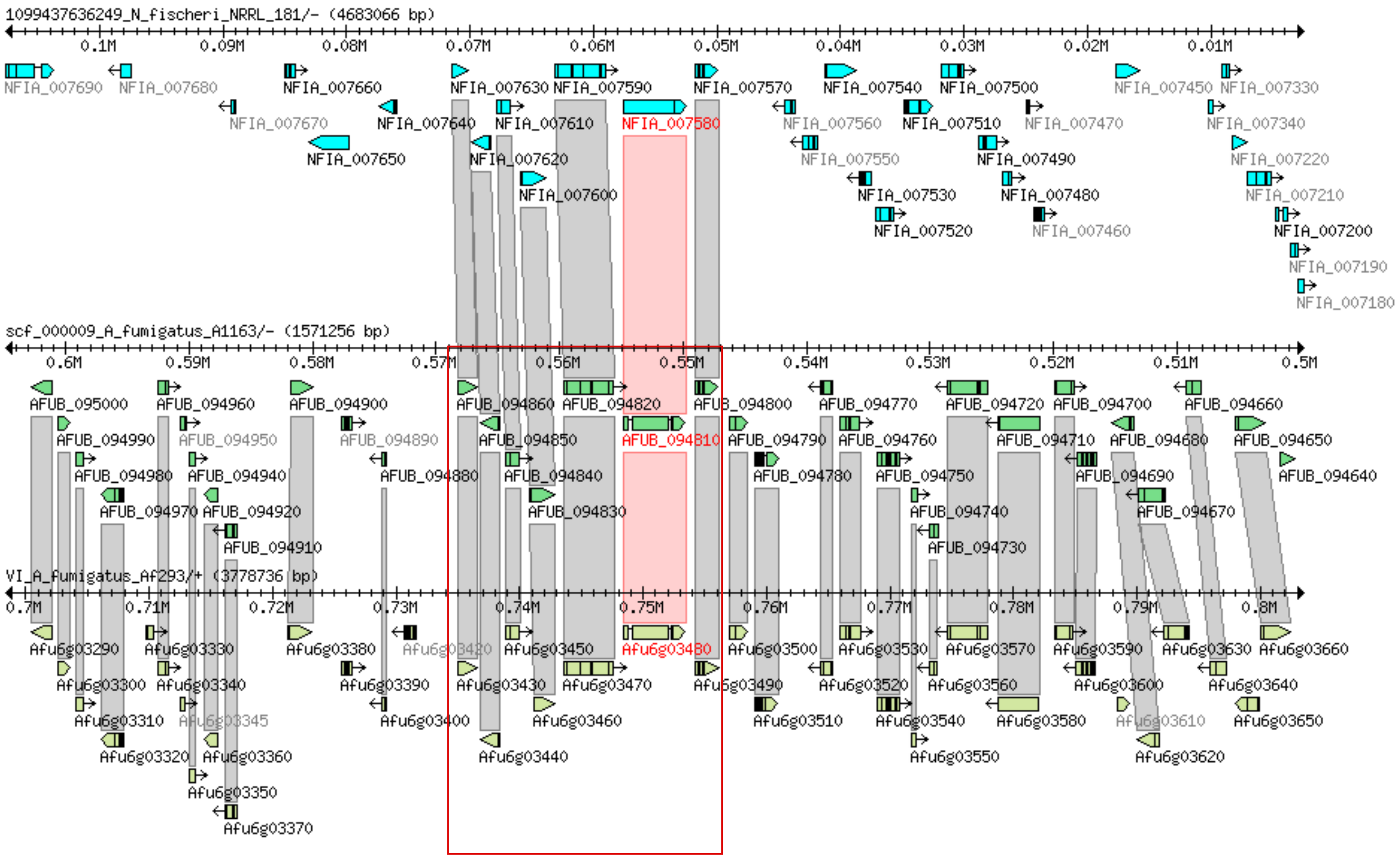

Afu6g03480 cluster

Gene id (SMURF)	Gene positions	Gene distance	Annotated_gene_function (SMURF)	AspGD gene name	AspGD supplementary annotation	antiSMASH ID	Gene ID (I)	Gene ID (II)	
AFUA_6G03620	13	3315	FAD/FMN-containing isoamyl alcohol oxidase MreA			n/a	Afu6g03620	AFUA_6G03620	
AFUA_6G03600	12	214	integral membrane protein (Pth11), putative			n/a	Afu6g03600	AFUA_6G03600	
AFUA_6G03590	11	1201	citrate synthase Cit1, putative			n/a	Afu6g03590	AFUA_6G03590	
AFUA_6G03580	10	830	mRNA-nucleus export ATPase (Elf1), putative			n/a	Afu6g03580	AFUA_6G03580	
AFUA_6G03570	9	791	beta-glucosidase, putative			n/a	Afu6g03570	AFUA_6G03570	
AFUA_6G03560	8	1031	IgE-binding protein, putative			n/a	Afu6g03560	AFUA_6G03560	
AFUA_6G03550	7	945	conserved hypothetical protein			prot_ID_235	Afu6g03550	AFUA_6G03550	
AFUA_6G03540	6	1367	malate synthase AcuE			prot_ID_234	Afu6g03540	AFUA_6G03540	
AFUA_6G03530	5	641	glutamine synthetase, putative			prot_ID_233	Afu6g03530	AFUA_6G03530	
AFUA_6G03520	4	3319	short-chain dehydrogenase/reductase family protein, putative			prot_ID_232	Afu6g03520	AFUA_6G03520	
AFUA_6G03510	3	620	flavin containing polyamine oxidase, putative			prot_ID_231	Afu6g03510	AFUA_6G03510	
AFUA_6G03500	2	803	conserved hypothetical protein			prot_ID_230	Afu6g03500	AFUA_6G03500	
AFUA_6G03490	1	670	phenol 2-monooxygenase, putative			prot_ID_229	Afu6g03490	AFUA_6G03490	ECS
**AFUA_6G03480**	0	0	NRPS-like enzyme, putative			**prot_ID_228**	**Afu6g03480**	**AFUA_6G03480**	
AFUA_6G03470	-1	724	ABC multidrug transporter, putative			prot_ID_227	Afu6g03470	AFUA_6G03470	
AFUA_6G03460	-2	672	conserved hypothetical protein			prot_ID_226	Afu6g03460	AFUA_6G03460	
AFUA_6G03450	-3	1078	N-methyltransferase, putative			prot_ID_225	Afu6g03450	AFUA_6G03450	
AFUA_6G03440	-4	577	fructosyl amino acid oxidase, putative			prot_ID_224	Afu6g03440	AFUA_6G03440	
AFUA_6G03430	-5	217	C6 finger domain protein, putative			prot_ID_223	Afu6g03430	AFUA_6G03430	ECS, IGD
n/a					Trehalase phosphorylase	prot_ID_222	Afu6g03420	AFUA_6G03420	
n/a					Protein of unknown function	prot_ID_221	Afu6g03400	AFUA_6G03400	

Afu6g08560 cluster

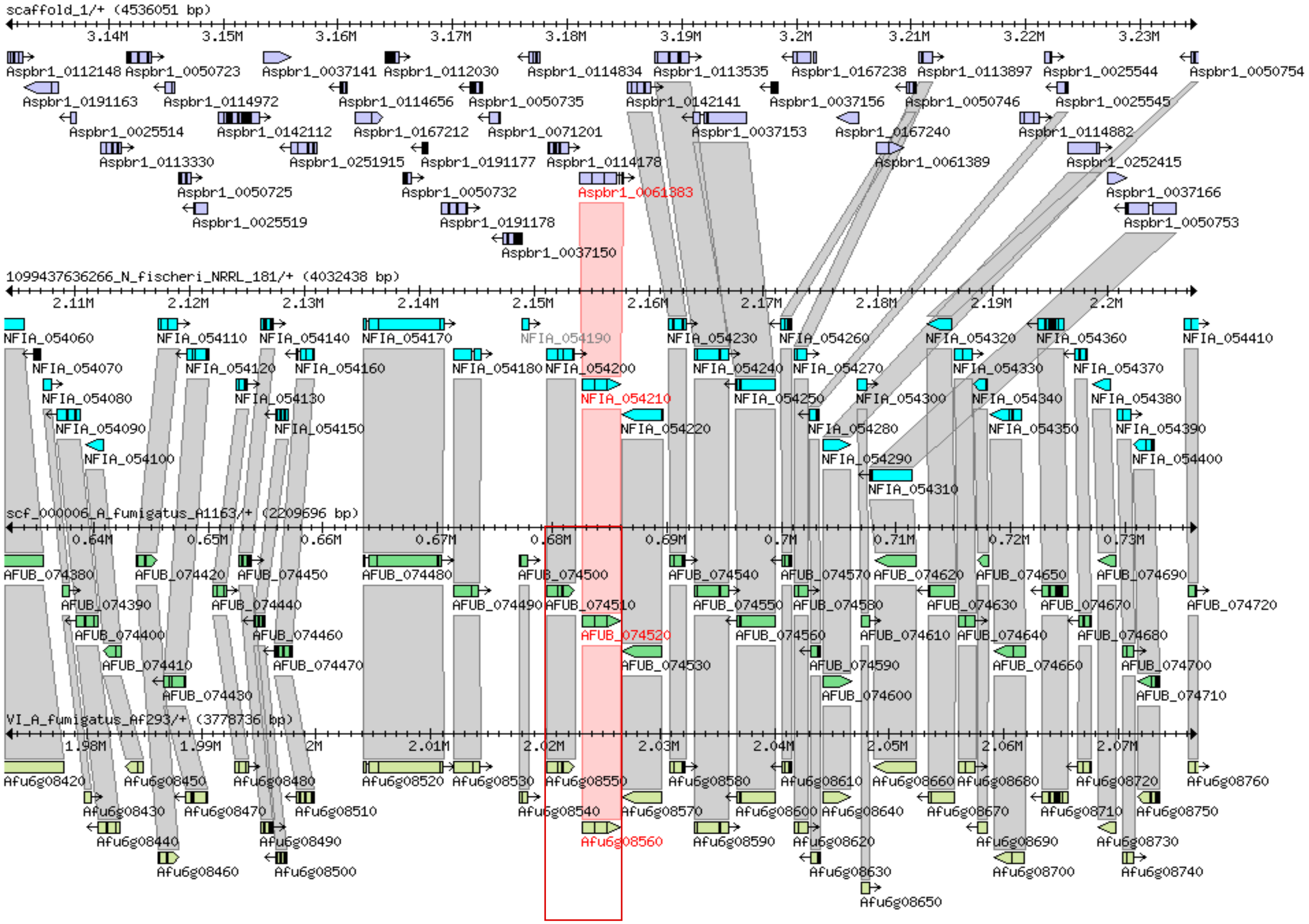

Afu6g08560 cluster

Gene id (SMURF)	Gene positions	Gene distance	Annotated_gene_function (SMURF)	AspGD gene name	AspGD supplementary annotation	antiSMASH ID	Gene ID (I)	Gene ID (II)	
n/a					adenylate cyclase AcyA	prot_ID_628	Afu6g08520	AFUA_6G08520	
n/a					sister chromatid separation protein (Src1), putative	prot_ID_629	Afu6g08530	AFUA_6G08530	
AFUA_6G08540	2	1607	C6 transcription factor SndA, putative			prot_ID_630	Afu6g08540	AFUA_6G08540	
AFUA_6G08550	1	619	C6 transcription factor, putative			prot_ID_631	Afu6g08550	AFUA_6G08550	ECS
**AFUA_6G08560**	0	0	NRPS-like enzyme, putative			**prot_ID_632**	**Afu6g08560**	**AFUA_6G08560**	ECS
n/a					DNA damage repair protein Mus42	prot_ID_633	Afu6g08570	AFUA_6G08570	
n/a				fkbp4	FKBP-type peptidyl-prolyl isomerase	prot_ID_634	Afu6g08580	AFUA_6G08580	
n/a					Protein serine/threonine kinase	prot_ID_635	Afu6g08590	AFUA_6G08590	
n/a					Transcription factor TFIID complex 145 kDasubunit	prot_ID_636	Afu6g08600	AFUA_6G08600	
n/a					RNA methyltransferase	prot_ID_637	Afu6g08610	AFUA_6G08610	
n/a					Protein of unknown function	prot_ID_638	Afu6g08620	AFUA_6G08620	
n/a					Protein of unknown function	prot_ID_639	Afu6g08630	AFUA_6G08630	
n/a					Metalloendopeptidase	prot_ID_640	Afu6g08640	AFUA_6G08640	

Afu6g12080 cluster

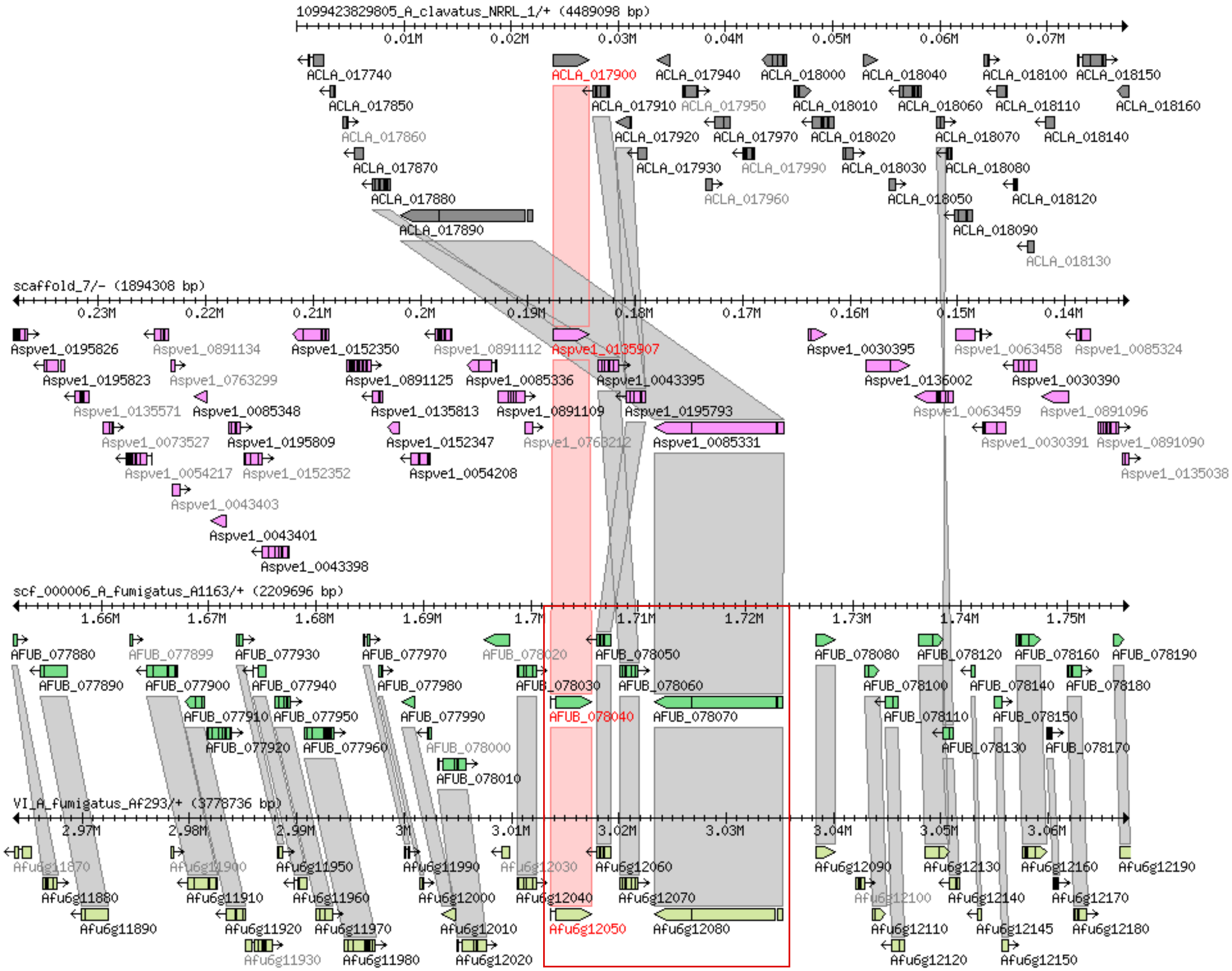

Afu6g12080 cluster

Gene id (SMURF)	Gene positions	Gene distance	Annotated_gene_function (SMURF)	AspGD gene name	AspGD supplementary annotation	antiSMASH ID	Gene ID (I)	Gene ID (II)	
n/a					Protein of unknown function	prot_ID_952	Afu6g11980	AFUA_6G11980	
n/a					Protein of unknown function	prot_ID_953	Afu6g11990	AFUA_6G11990	
n/a					Protein of unknown function	prot_ID_954	Afu6g12000	AFUA_6G12000	
n/a					Hydrolase	prot_ID_955	Afu6g12010	AFUA_6G12010	
n/a					C2H2 finger domain protein	prot_ID_956	Afu6g12020	AFUA_6G12020	
n/a					Rhamnosidase	prot_ID_957	Afu6g12030	AFUA_6G12030	
AFUA_6G12040	4	1252	MFS sugar permease, putative			prot_ID_958	Afu6g12040	AFUA_6G12040	ECS
AFUA_6G12050	3	401	nonribosomal peptide synthase, putative			**prot_ID_959**	**Afu6g12050**	**AFUA_6G12050**	
AFUA_6G12060	2	829	MAK1-like monooxygenase, putative			prot_ID_960	Afu6g12060	AFUA_6G12060	
AFUA_6G12070	1	1390	FAD binding domain protein			prot_ID_961	Afu6g12070	AFUA_6G12070	
**AFUA_6G12080**	0	0	nonribosomal peptide synthase, putative			**prot_ID_962**	**Afu6g12080**	**AFUA_6G12080**	ECS
AFUA_6G12090	-1	3032	HET domain protein			prot_ID_963	Afu6g12090	AFUA_6G12090	
AFUA_6G12100	-2	1903	nitrilase family protein			prot_ID_964	Afu6g12100	AFUA_6G12100	
AFUA_6G12110	-3	610	salicylate synthetase, putative			prot_ID_965	Afu6g12110	AFUA_6G12110	
AFUA_6G12120	-4	425	BNR/Asp-box repeat domain protein			prot_ID_966	Afu6g12120	AFUA_6G12120	
AFUA_6G12130	-5	1972	C6 transcription factor, putative			prot_ID_967	Afu6g12130	AFUA_6G12130	
AFUA_6G12140	-6	41	hypothetical protein			prot_ID_968	Afu6g12140	AFUA_6G12140	
AFUA_6G12145	-7	1771	hypothetical protein			prot_ID_1233	Afu6g12145	AFUA_6G12145	
AFUA_6G12150	-8	1987	bZIP transcription factor (Atf7), putative			n/a	Afu6g12150	AFUA_6G12150	
AFUA_6G12160	-9	1305	C6 transcription factor, putative			n/a	Afu6g12160	AFUA_6G12160	

Afu6g13930 cluster

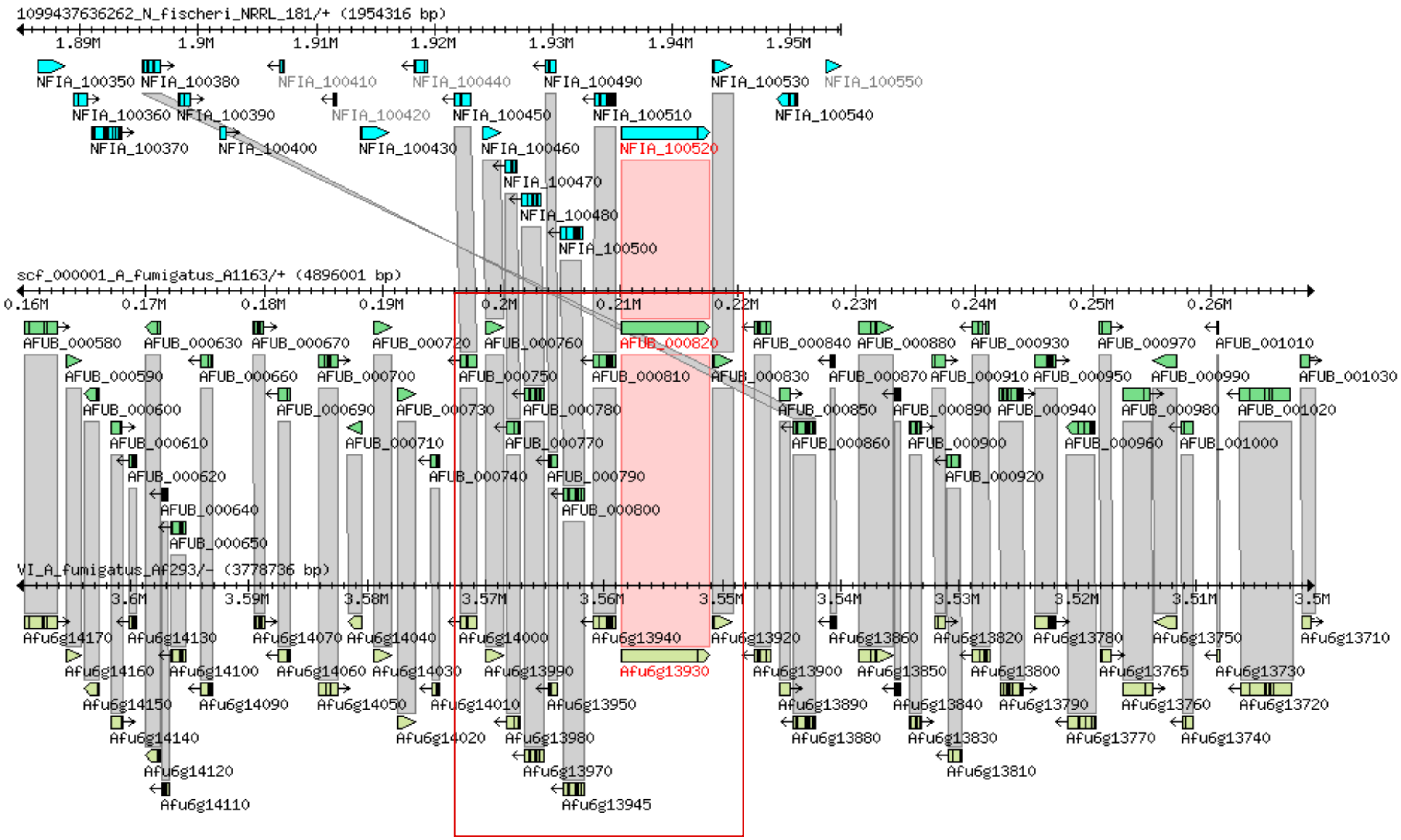

Afu6g13930 cluster

Gene id (SMURF)	Gene positions	Gene distance	Annotated_gene_function (SMURF)	AspGD gene name	AspGD supplementary annotation	antiSMASH ID	Gene ID (I)	Gene ID (II)	
n/a					Putative het locus with a predicted role in heterokaryon incompatibility	prot_ID_1127	Afu6g13820	AFUA_6G13820	
AFUA_6G13830	8	720	oxidoreductase, short chain dehydrogenase/reductase family			prot_ID_1128	Afu6g13830	AFUA_6G13830	
AFUA_6G13840	7	168	conserved hypothetical protein			prot_ID_1129	Afu6g13840	AFUA_6G13840	
AFUA_6G13850	6	1981	GTPase activating protein (Evi5), putative			prot_ID_1130	Afu6g13850	AFUA_6G13850	
AFUA_6G13860	5	1203	conserved hypothetical protein			prot_ID_1131	Afu6g13860	AFUA_6G13860	
AFUA_6G13880	4	170	MFS transporter, putative			prot_ID_1132	Afu6g13880	AFUA_6G13880	
AFUA_6G13890	3	723	conserved hypothetical protein			prot_ID_1133	Afu6g13890	AFUA_6G13890	
AFUA_6G13900	2	1743	conserved hypothetical protein			prot_ID_1134	Afu6g13900	AFUA_6G13900	
AFUA_6G13920	1	240	adenylate-forming enzyme, putative			prot_ID_1135	Afu6g13920	AFUA_6G13920	ECS
**AFUA_6G13930**	0	0	polyketide synthase LovB-like, putative			**prot_ID_1136**	**Afu6g13930**	**AFUA_6G13930**	
AFUA_6G13940	-1	494	cytochrome P450 monooxygenase, putative			prot_ID_1137	Afu6g13940	AFUA_6G13940	
AFUA_6G13945	-2	829	cytochrome P450 monooxygenase, putative			prot_ID_1213	Afu6g13945	AFUA_6G13945	
AFUA_6G13950	-3	478	integral membrane protein			prot_ID_1138	Afu6g13950	AFUA_6G13950	
AFUA_6G13970	-4	366	FAD-dependent monooxygenase (PaxM), putative			prot_ID_1139	Afu6g13970	AFUA_6G13970	
AFUA_6G13980	-5	355	UbiA-like prenyltransferase, putative			prot_ID_1140	Afu6g13980	AFUA_6G13980	
AFUA_6G13990	-6	296	acetyltransferase, putative			prot_ID_1141	Afu6g13990	AFUA_6G13990	
AFUA_6G14000	-7	751	toxin biosynthesis protein Tri7-like , putative			prot_ID_1142	Afu6g14000	AFUA_6G14000	ECS
AFUA_6G14010	-8	1714	GPI anchored protein, putative			prot_ID_1143	Afu6g14010	AFUA_6G14010	
AFUA_6G14020	-9	1347	conserved hypothetical protein			prot_ID_1144	Afu6g14020	AFUA_6G14020	
AFUA_6G14030	-10	509	acyltransferase, putative			prot_ID_1145	Afu6g14030	AFUA_6G14030	
AFUA_6G14040	-11	1034	glycosyl transferase, putative			n/a	Afu6g14040	AFUA_6G14040	
AFUA_6G14050	-12	809	FAD binding domain protein			n/a	Afu6g14050	AFUA_6G14050	

Afu7g00170 cluster

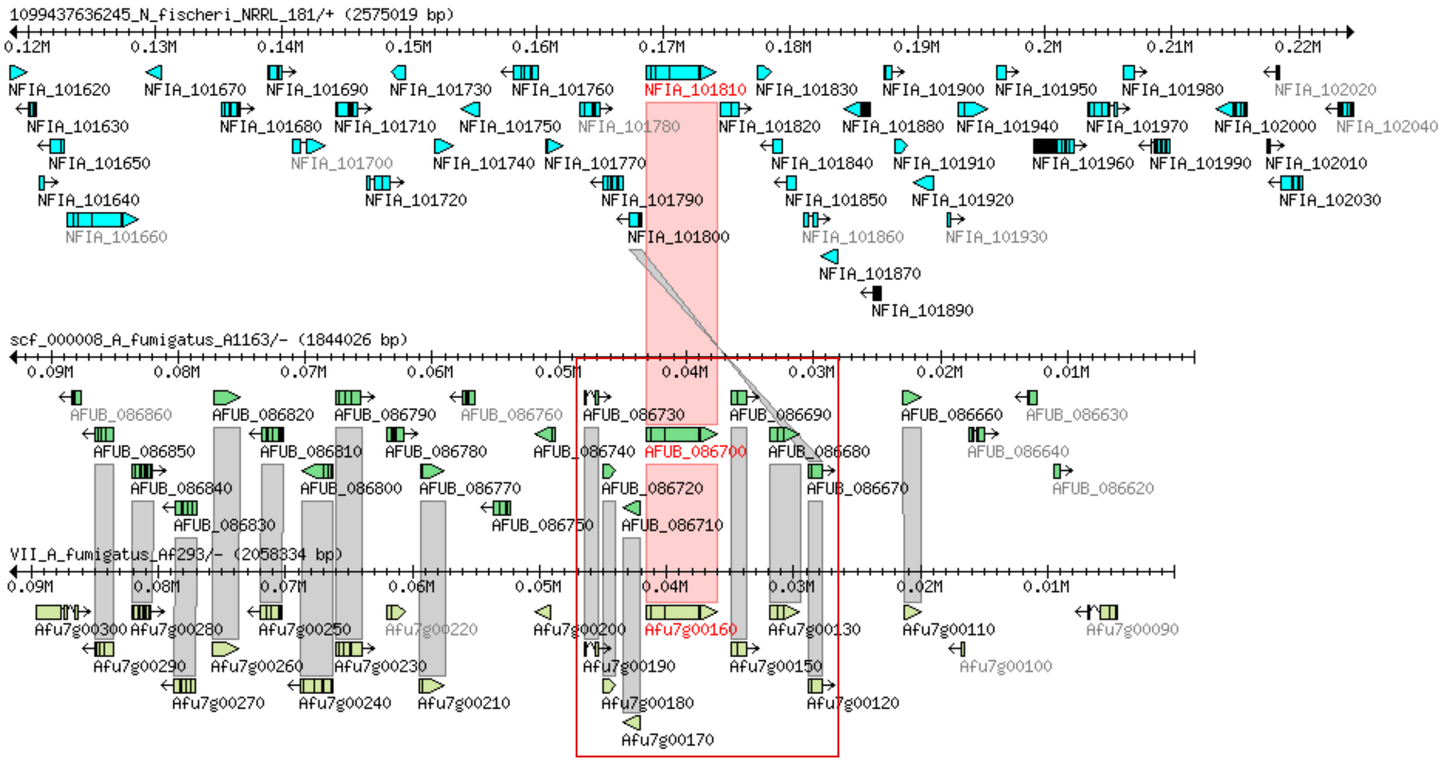

Afu7g00170 cluster

Gene id (SMURF)	Gene positions	Gene distance	Annotated_gene_function (SMURF)	AspGD gene name	AspGD supplementary annotation	antiSMASH ID	Gene ID (I)	Gene ID (II)	
n/a					Transmembrane transporter	prot_ID_13	Afu7g00220	AFUA_7G00220	
n/a					C6 transcription factor	prot_ID_12	Afu7g00210	AFUA_7G00210	
AFUA_7G00200	3	2662	conserved hypothetical protein			prot_ID_11	Afu7g00200	AFUA_7G00200	
AFUA_7G00190	2	387	Protein of unknown function			prot_ID_10	Afu7g00190	AFUA_7G00190	ECS
AFUA_7G00180	1	597	Nucleoside-diphosphate sugar epimerase			prot_ID_9	Afu7g00180	AFUA_7G00180	
**AFUA_7G00170**	0	0	dimethylallyl tryptophan synthase GliD1			**prot_ID_8**	**Afu7g00170**	**AFUA_7G00170**	
AFUA_7G00160	-1	482	polyketide synthase, putative			**prot_ID_7**	**Afu7g00160**	**AFUA_7G00160**	
AFUA_7G00150	-2	1049	FAD-dependent monooxygenase, putative			prot_ID_6	Afu7g00150	AFUA_7G00150	
AFUA_7G00130	-3	1797	C6 transcription factor, putative			prot_ID_5	Afu7g00130	AFUA_7G00130	
AFUA_7G00120	-4	588	metallo-beta-lactamase domain protein			prot_ID_4	Afu7g00120	AFUA_7G00120	ECS, IGD
n/a					Triglyceride lipase	prot_ID_3	Afu7g00110	AFUA_7G00110	
n/a					Protein of unknown function	prot_ID_2	Afu7g00100	AFUA_7G00100	

Afu8g00540 cluster

Possible overlapping or adjacent clusters, no manual prediction made

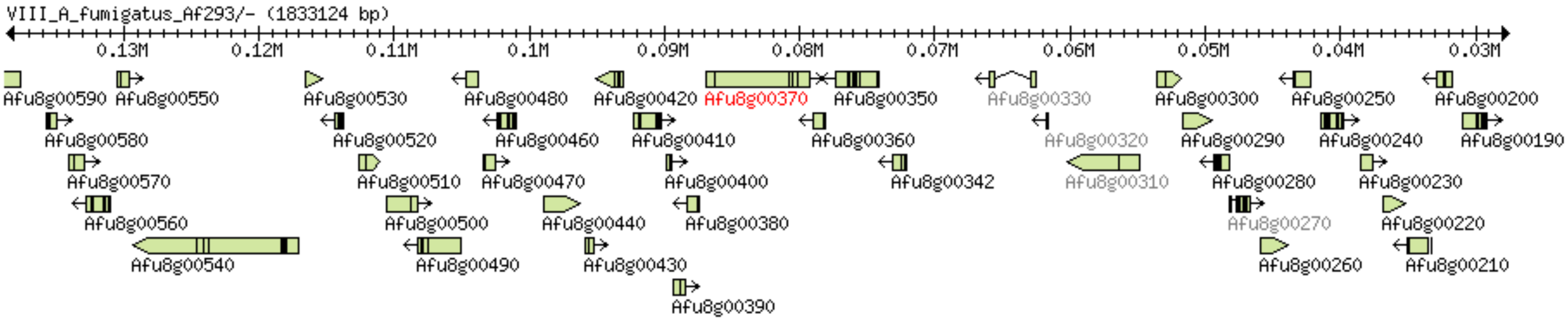

Afu8g00540 cluster

Gene id (SMURF)	Gene positions	Gene distance	Annotated_gene_function (SMURF)	AspGD gene name	AspGD supplementary annotation	antiSMASH ID	Gene ID (I)	Gene ID (II)
AFUA_8G00620	9	3538	dimethylallyl tryptophan synthase, putative			n/a	Afu8g00370	AFUA_8G00620
AFUA_8G00610	8	228	cell surface protein Mas1, putative			n/a	Afu8g00610	AFUA_8G00610
AFUA_8G00600	7	541	NAD dependent epimerase/dehydratase family protein			n/a	Afu8g00600	AFUA_8G00600
AFUA_8G00595	6	1189	Ham1 family pyrophosphatase, putative			n/a	Afu8g00595	AFUA_8G00595
AFUA_8G00590	5	2026	fatty acid desaturase, putative			n/a	Afu8g00590	AFUA_8G00590
AFUA_8G00580	4	825	glutathione S-transferase, putative			n/a	Afu8g00580	AFUA_8G00580
AFUA_8G00570	3	89	alpha/beta hydrolase, putative			n/a	Afu8g00570	AFUA_8G00570
AFUA_8G00560	2	539	cytochrome P450 oxidoreductase, putative			n/a	Afu8g00560	AFUA_8G00560
AFUA_8G00550	1	235	methyltransferase SirN-like, putative			n/a	Afu8g00550	AFUA_8G00550
**AFUA_8G00540**	0	0	hybrid PKS-NRPS enzyme, putative	nrps14		n/a	**Afu8g00540**	**AFUA_8G00540**
AFUA_8G00530	-1	463	alpha/beta superfamily hydrolase			n/a	Afu8g00530	AFUA_8G00530
AFUA_8G00520	-2	780	integral membrane protein			n/a	Afu8g00520	AFUA_8G00520
AFUA_8G00510	-3	1148	cytochrome P450 oxidoreductase OrdA-like, putative			n/a	Afu8g00510	AFUA_8G00510
AFUA_8G00500	-4	409	acetate-CoA ligase, putative			n/a	Afu8g00500	AFUA_8G00500
AFUA_8G00490	-5	48	PKS-like enzyme, putative			prot_ID_37	Afu8g00490	AFUA_8G00490
AFUA_8G00480	-6	401	phytanoyl-CoA dioxygenase family protein			prot_ID_36	Afu8g00480	AFUA_8G00480
AFUA_8G00470	-7	397	conserved hypothetical protein			prot_ID_35	Afu8g00470	AFUA_8G00470
AFUA_8G00460	-8	133	methionine aminopeptidase, type I, putative			prot_ID_34	Afu8g00460	AFUA_8G00460
AFUA_8G00440	-9	1948	steroid monooxygenase, putative			prot_ID_33	Afu8g00440	AFUA_8G00440
AFUA_8G00430	-10	445	conserved hypothetical protein			prot_ID_32	Afu8g00430	AFUA_8G00430
AFUA_8G00420	-11	176	C6 finger transcription factor, putative			prot_ID_31	Afu8g00420	AFUA_8G00420
AFUA_8G00410	-12	878	methionine aminopeptidase, type II, putative			prot_ID_30	Afu8g00410	AFUA_8G00410
AFUA_8G00400	-13	429	conserved hypothetical protein			prot_ID_29	Afu8g00400	AFUA_8G00400
AFUA_8G00390	-14	87	O-methyltransferase, putative			prot_ID_28	Afu8g00390	AFUA_8G00390

AFUA_8G00380	-15	197	DltD N-terminal domain protein		prot_ID_27	Afu8g00380	AFUA_8G00380
AFUA_8G00370	-16	483	polyketide synthase, putative		prot_ID_26	Afu8g00370	AFUA_8G00370
n/a				Protein of unknown function	prot_ID_25	Afu8g00360	AFUA_8G00360
n/a				Protein of unknown function	prot_ID_24	Afu8g00350	AFUA_8G00350
n/a				Endonuclease	prot_ID_23	Afu8g00330	AFUA_8G00330
n/a				Protein of unknown function	prot_ID_22	Afu8g00320	AFUA_8G00320
n/a				Reverse transcriptase, RNaseH	prot_ID_21	Afu8g00310	AFUA_8G00310

Afu8g00620 cluster

No manual prediction made

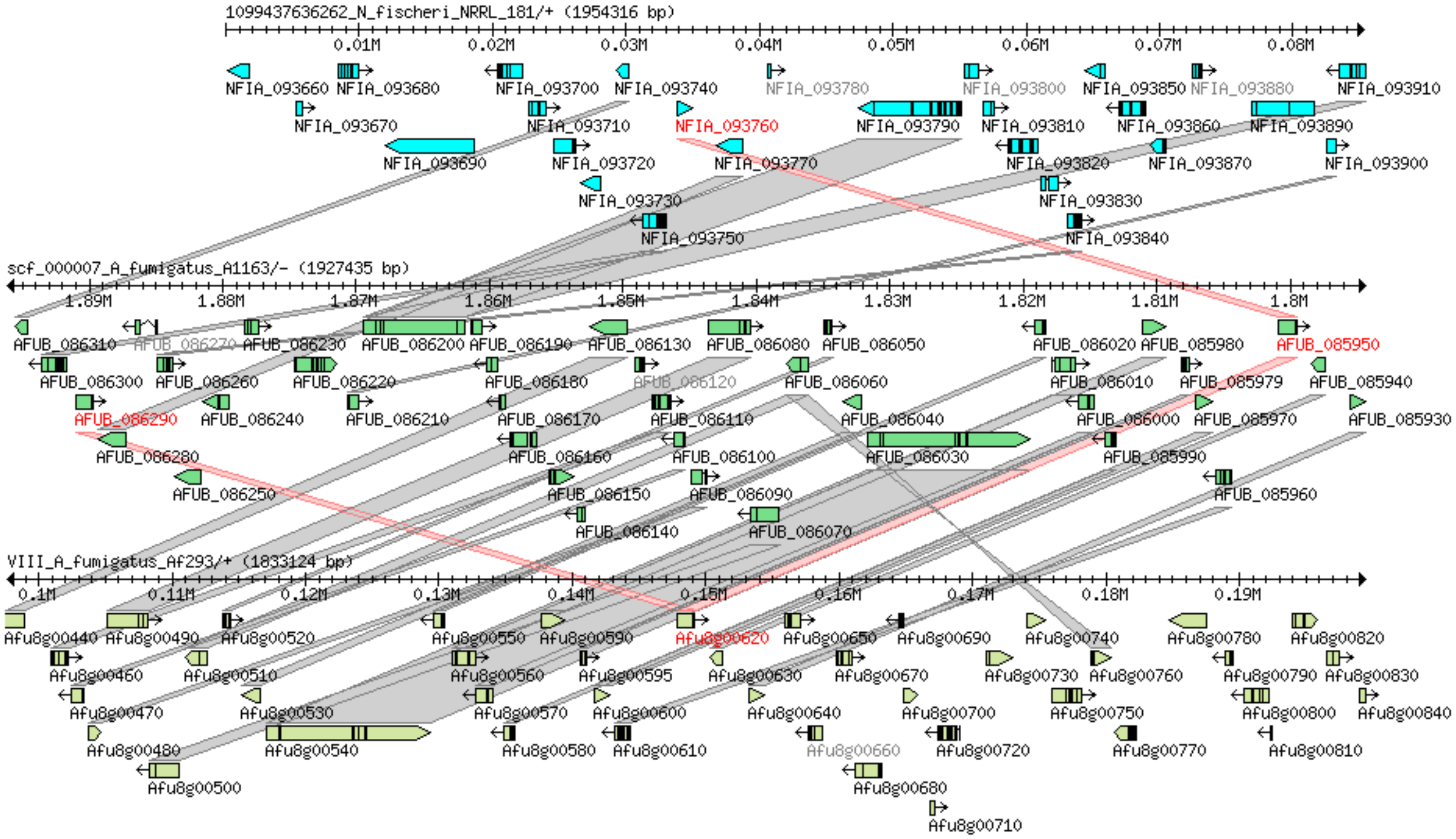

Afu8g00620 cluster

Gene id (SMURF)	Gene positions	Gene distance	Annotated_gene_function (SMURF)	AspGD gene name	AspGD supplementary annotation	antiSMASH ID	Gene ID (I)	Gene ID (II)
					Putative amino acid transporter	prot_ID_60	Afu8g00720	AFUA_8G00720
					Domain(s) with predicted role in defense response, negative regulation of growth	prot_ID_59	Afu8g00710	AFUA_8G00710
n/a					class III chitinase	prot_ID_58	Afu8g00700	AFUA_8G00700
n/a					Protein of unknown function	prot_ID_57	Afu8g00690	AFUA_8G00690
n/a					Protein of unknown function	prot_ID_56	Afu8g00680	AFUA_8G00680
n/a					Protein of unknown function	prot_ID_55	Afu8g00670	AFUA_8G00670
n/a					Protein of unknown function	prot_ID_54	Afu8g00660	AFUA_8G00660
n/a					Protein of unknown function	prot_ID_53	Afu8g00650	AFUA_8G00650
AFUA_8G00640	2	1941	glycosyl transferase, putative		Protein of unknown function	prot_ID_52	Afu8g00640	AFUA_8G00640
AFUA_8G00630	1	1008	conserved hypothetical protein		Protein of unknown function	prot_ID_51	Afu8g00630	AFUA_8G00630
**AFUA_8G00620**	0	0	dimethylallyl tryptophan synthase, putative	cdpNPT	DMATS-type N-prenyltransferase	**prot_ID_50**	**Afu8g00620**	**AFUA_8G00620**
AFUA_8G00610	-1	3538	cell surface protein Mas1, putative	mas1		prot_ID_49	Afu8g00610	AFUA_8G00610
AFUA_8G00600	-2	228	NAD dependent epimerase/dehydratase family protein		Protein of unknown function	prot_ID_48	Afu8g00600	AFUA_8G00600
AFUA_8G00595	-3	541	Ham1 family pyrophosphatase, putative			prot_ID_612	Afu8g00595	AFUA_8G00595
AFUA_8G00590	-4	1189	fatty acid desaturase, putative			prot_ID_47	Afu8g00590	AFUA_8G00590
AFUA_8G00580	-5	2026	glutathione S-transferase, putative			prot_ID_46	Afu8g00580	AFUA_8G00580
AFUA_8G00570	-6	825	alpha/beta hydrolase, putative			prot_ID_45	Afu8g00570	AFUA_8G00570
AFUA_8G00560	-7	89	cytochrome P450 oxidoreductase, putative			prot_ID_44	Afu8g00560	AFUA_8G00560
AFUA_8G00550	-8	539	methyltransferase SirN-like, putative			prot_ID_43	Afu8g00550	AFUA_8G00550
AFUA_8G00540	-9	235	hybrid PKS-NRPS enzyme, putative			**prot_ID_42**	**Afu8g00540**	**AFUA_8G00540**
AFUA_8G00530	-10	463	alpha/beta superfamily hydrolase			prot_ID_41	Afu8g00530	AFUA_8G00530
AFUA_8G00520	-11	780	integral membrane protein			prot_ID_40	Afu8g00520	AFUA_8G00520
AFUA_8G00510	-12	1148	cytochrome P450 oxidoreductase OrdA-like, putative			prot_ID_39	Afu8g00510	AFUA_8G00510
AFUA_8G00500	-13	409	acetate-CoA ligase, putative			prot_ID_38	Afu8g00500	AFUA_8G00500

AFUA_8G00490	-14	48	PKS-like enzyme, putative	prot_ID_37	Afu8g00490	AFUA_8G00490
AFUA_8G00480	-15	401	phytanoyl-CoA dioxygenase family protein	prot_ID_36	Afu8g00480	AFUA_8G00480
AFUA_8G00470	-16	397	conserved hypothetical protein	prot_ID_35	Afu8g00470	AFUA_8G00470
n/a				prot_ID_34	Afu8g00460	AFUA_8G00460
n/a				prot_ID_33	Afu8g00440	AFUA_8G00440
n/a				prot_ID_32	Afu8g00430	AFUA_8G00430
n/a				prot_ID_31	Afu8g00420	AFUA_8G00420
n/a				prot_ID_30	Afu8g00410	AFUA_8G00410
n/a				prot_ID_29	Afu8g00400	AFUA_8G00400
n/a				prot_ID_28	Afu8g00390	AFUA_8G00390

Afu8g01640 cluster

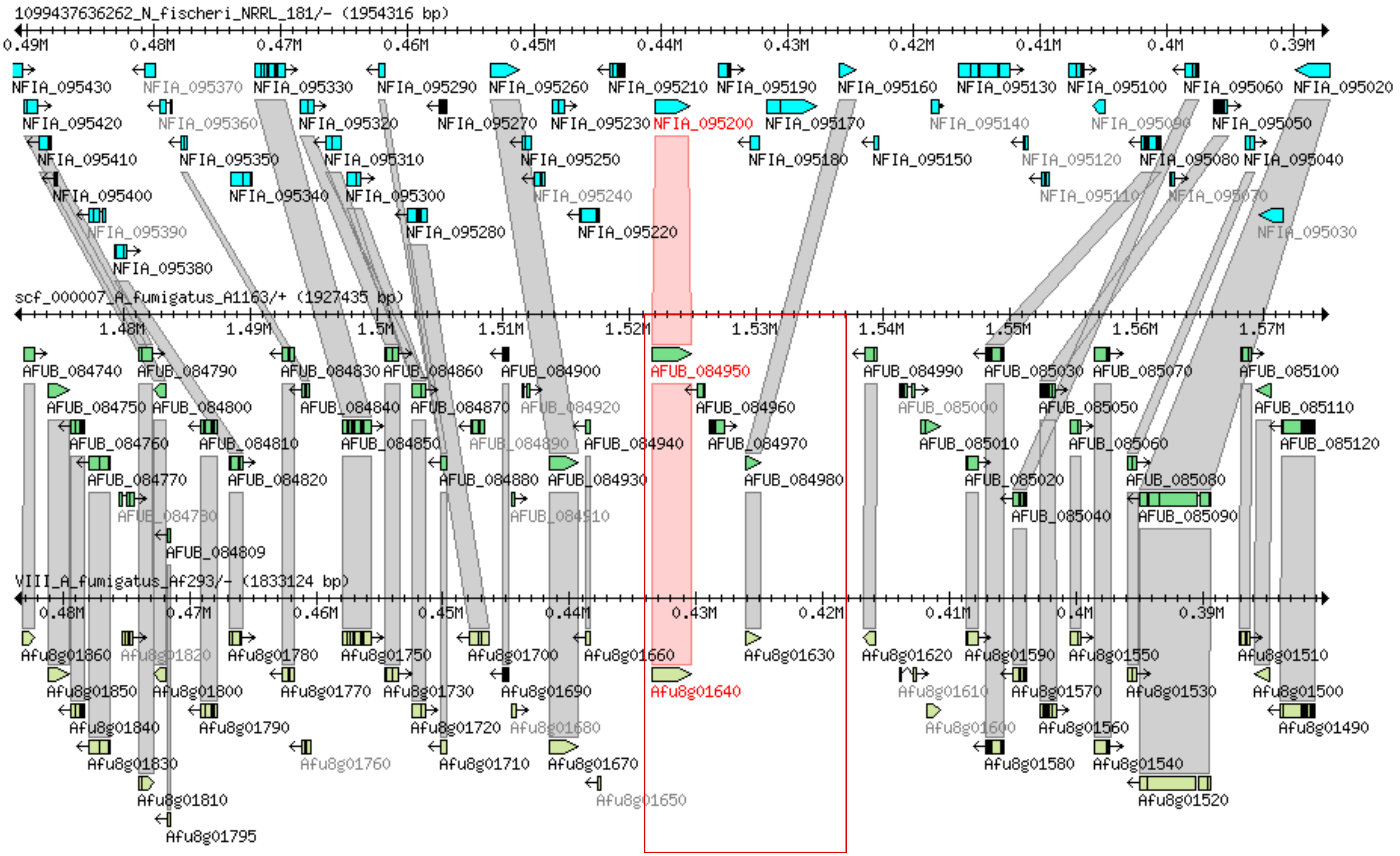

Afu8g01640 cluster

Gene id (SMURF)	Gene positions	Gene distance	Annotated_gene_function (SMURF)	AspGD gene name	AspGD supplementary annotation	antiSMASH ID	Gene ID (I)	Gene ID (II)	
n/a					Protein of unknown function	prot_ID_143	Afu8g01600	AFUA_8G01600	
n/a					Flavin adenine dinucleotide binding, oxidoreductase	prot_ID_146	Afu8g01630	AFUA_8G01630	ECS, IGD
**AFUA_8G01640**	0	0	NRPS-like enzyme, putative		Nonribosomal peptide synthase	**prot_ID_147**	**Afu8g01640**	**AFUA_8G01640**	ECS, IGD
n/a					Protein of unknown function	prot_ID_148	Afu8g01650	AFUA_8G01650	
n/a					Protein of unknown function	prot_ID_149	Afu8g01660	AFUA_8G01660	
n/a					bifunctional catalase-peroxidase	prot_ID_150	Afu8g01670	AFUA_8G01670	
n/a					Protein of unknown function	prot_ID_151	Afu8g01680	AFUA_8G01680	
n/a					Protein of unknown function	prot_ID_152	Afu8g01690	AFUA_8G01690	
n/a					Haloalkane dehalogenase family protein	prot_ID_153	Afu8g01700	AFUA_8G01700	
n/a						prot_ID_154	Afu8g01710	AFUA_8G01710	
n/a						prot_ID_155	Afu8g01720	AFUA_8G01720	
n/a						prot_ID_156	Afu8g01730	AFUA_8G01730	

Afu8g02350 cluster

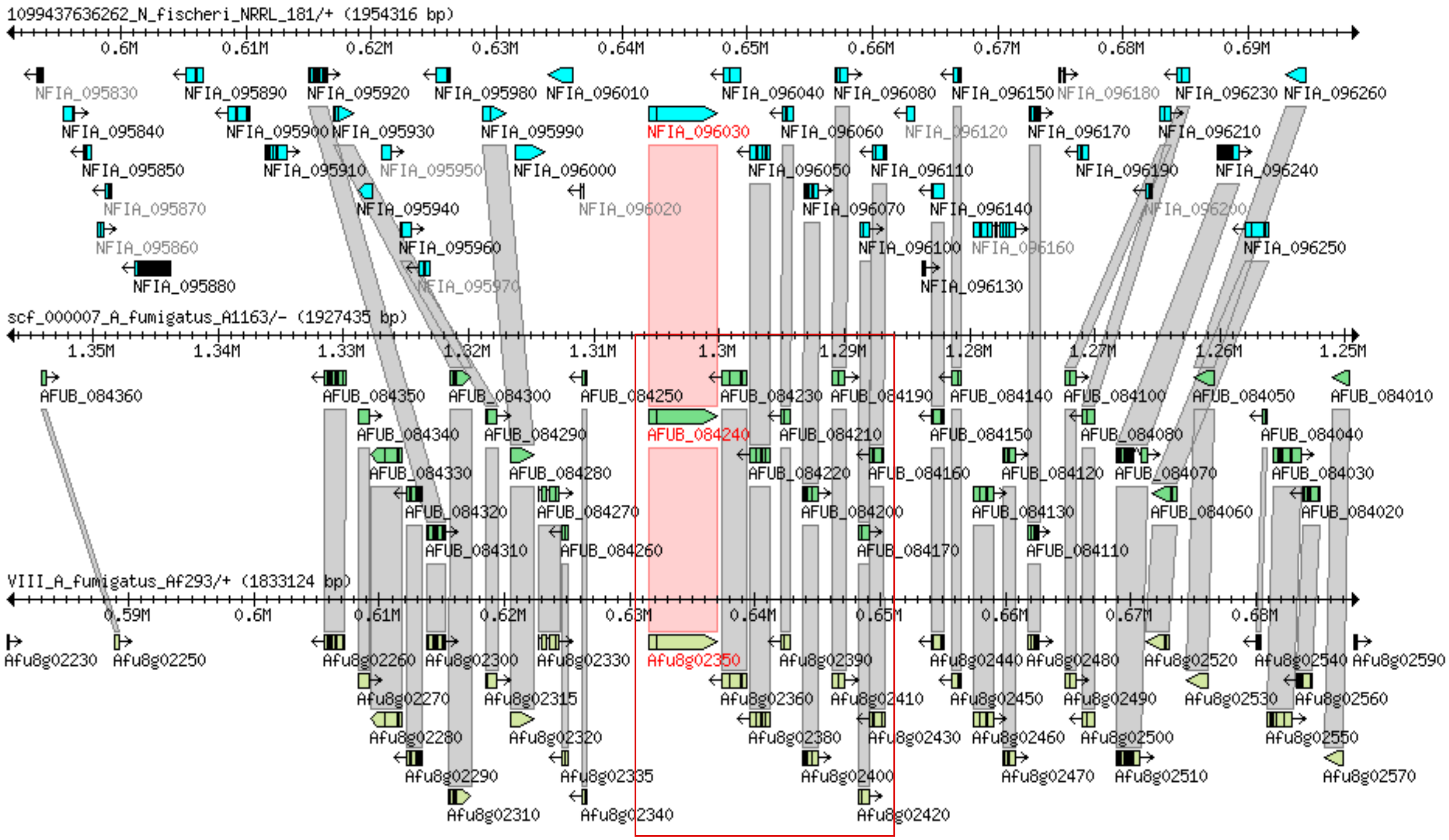

Afu8g02350 cluster

Gene id (SMURF)	Gene positions	Gene distance	Annotated_gene_function (SMURF)	AspGD gene name	AspGD supplementary annotation	antiSMASH ID	Gene ID (I)	Gene ID (II)	
n/a						prot_ID_227	Afu8g02490	AFUA_8G02490	
n/a						prot_ID_226	Afu8g02480	AFUA_8G02480	
n/a						prot_ID_225	Afu8g02470	AFUA_8G02470	
AFUA_8G02460	10	1004	MFS transporter, putative			prot_ID_224	Afu8g02460	AFUA_8G02460	
AFUA_8G02450	9	661	conserved hypothetical protein			prot_ID_223	Afu8g02450	AFUA_8G02450	
AFUA_8G02440	8	3763	C-4 methyl sterol oxidase, putative			prot_ID_222	Afu8g02440	AFUA_8G02440	
AFUA_8G02430	7	44	zinc-binding alcohol dehydrogenase, putative			prot_ID_221	Afu8g02430	AFUA_8G02430	ECS, IGD
AFUA_8G02420	6	911	conserved hypothetical protein			prot_ID_220	Afu8g02420	AFUA_8G02420	
AFUA_8G02410	5	1209	UbiA prenyltransferase family protein			prot_ID_219	Afu8g02410	AFUA_8G02410	
AFUA_8G02400	4	899	geranylgeranyl pyrophosphate synthetase AtmG, putative			prot_ID_218	Afu8g02400	AFUA_8G02400	
AFUA_8G02390	3	897	conserved hypothetical protein			prot_ID_217	Afu8g02390	AFUA_8G02390	
AFUA_8G02380	2	220	FAD-dependent monooxygenase, putative			prot_ID_216	Afu8g02380	AFUA_8G02380	
AFUA_8G02360	1	357	TRI7-like toxin biosynthesis protein, putative			prot_ID_215	Afu8g02360	AFUA_8G02360	
**AFUA_8G02350**	0	0	polyketide synthase, putative		Polyketide synthase	**prot_ID_214**	**Afu8g02350**	**AFUA_8G02350**	ECS, IGD
n/a					Protein of unknown function	prot_ID_213	Afu8g02340	AFUA_8G02340	
n/a					Protein of unknown function	prot_ID_609	Afu8g02335	AFUA_8G02335	
n/a					Protein of unknown function	prot_ID_212	Afu8g02330	AFUA_8G02330	
n/a					Protein of unknown function	prot_ID_211	Afu8g02320	AFUA_8G02320	
n/a					Protein of unknown function	prot_ID_602	Afu8g02315	AFUA_8G02315	
n/a					Oxidoreductase	prot_ID_210	Afu8g02310	AFUA_8G02310	
n/a					FMN binding, oxidoreductase	prot_ID_209	Afu8g02300	AFUA_8G02300	
n/a					Protein of unknown function	prot_ID_208	Afu8g02290	AFUA_8G02290	
n/a					Transcription cofactor	prot_ID_207	Afu8g02280	AFUA_8G02280	

No PKS or NRPS backbone 1
No manual prediction made

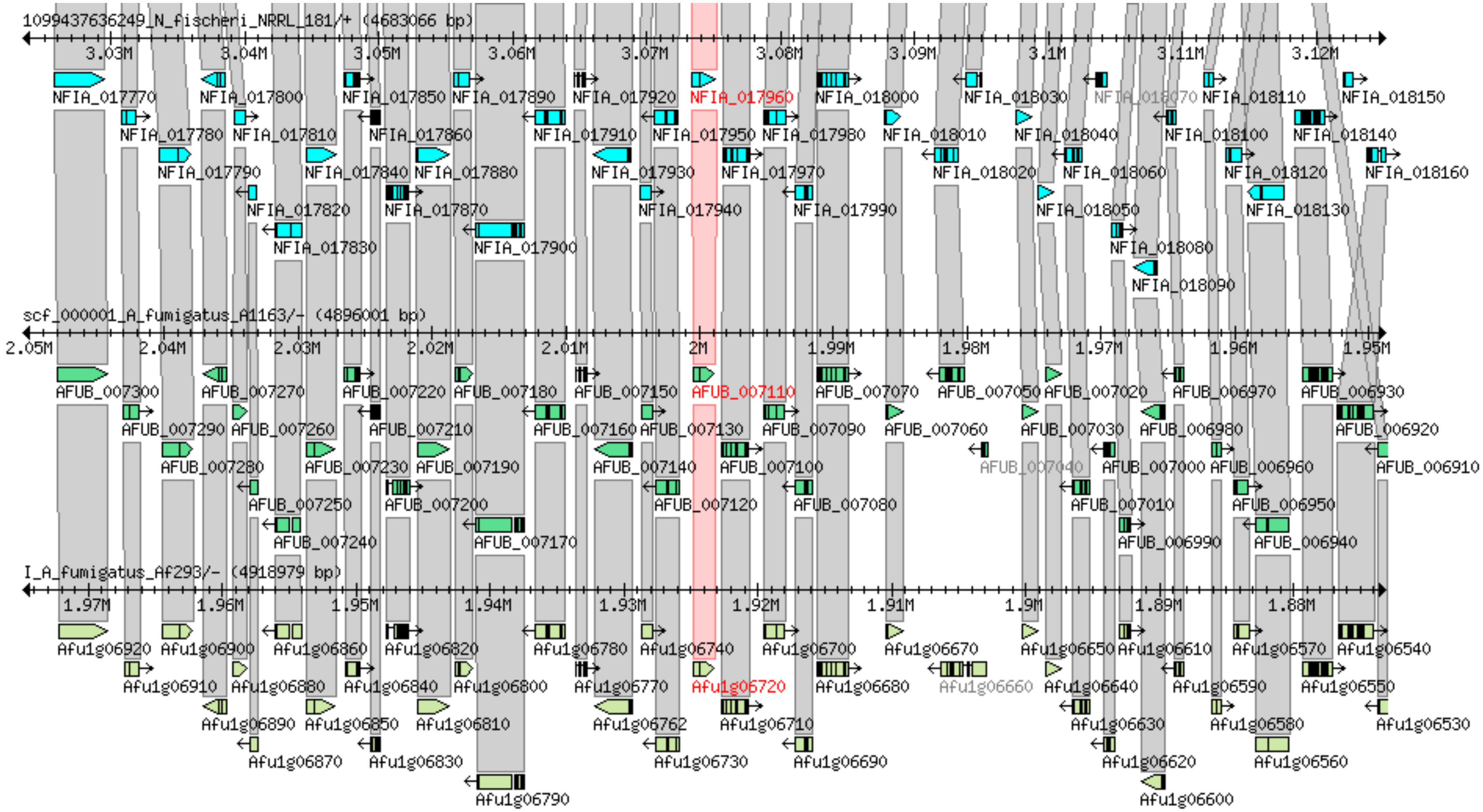

No PKS or NRPS backbone 1

Gene id (SMURF)	Gene positions	Gene distance	Annotated_gene_function (SMURF)	AspGD gene name	AspGD supplementary annotation	antiSMASH ID	Gene ID (I)	Gene ID (II)
n/a					Ortholog(s) have role in maturation of SSU-rRNA	prot_ID_625	Afu1g06690	AFUA_1G06690
n/a				casA	Predicted metacaspase, an aspartate-specific cysteine protease	prot_ID_626	Afu1g06700	AFUA_1G06700
n/a					t-complex protein 1, eta subunit	prot_ID_627	Afu1g06710	AFUA_1G06710
n/a					Histone acetyltransferase	prot_ID_628	Afu1g06720	AFUA_1G06720
n/a					Protein of unknown function	prot_ID_629	Afu1g06730	AFUA_1G06730
n/a					Domain(s) with predicted nucleic acid binding, zinc ion binding activity	prot_ID_630	Afu1g06740	AFUA_1G06740
n/a					Protein of unknown function	prot_ID_1626	Afu1g06762	AFUA_1G06762
n/a					40S ribosomal protein S26	prot_ID_631	Afu1g06770	AFUA_1G06770
n/a					CBS and PB1 domain protein	prot_ID_632	Afu1g06780	AFUA_1G06780
n/a					Importin beta-3 subunit	prot_ID_633	Afu1g06790	AFUA_1G06790

No PKS or NRPS backbone 2

No manual prediction made

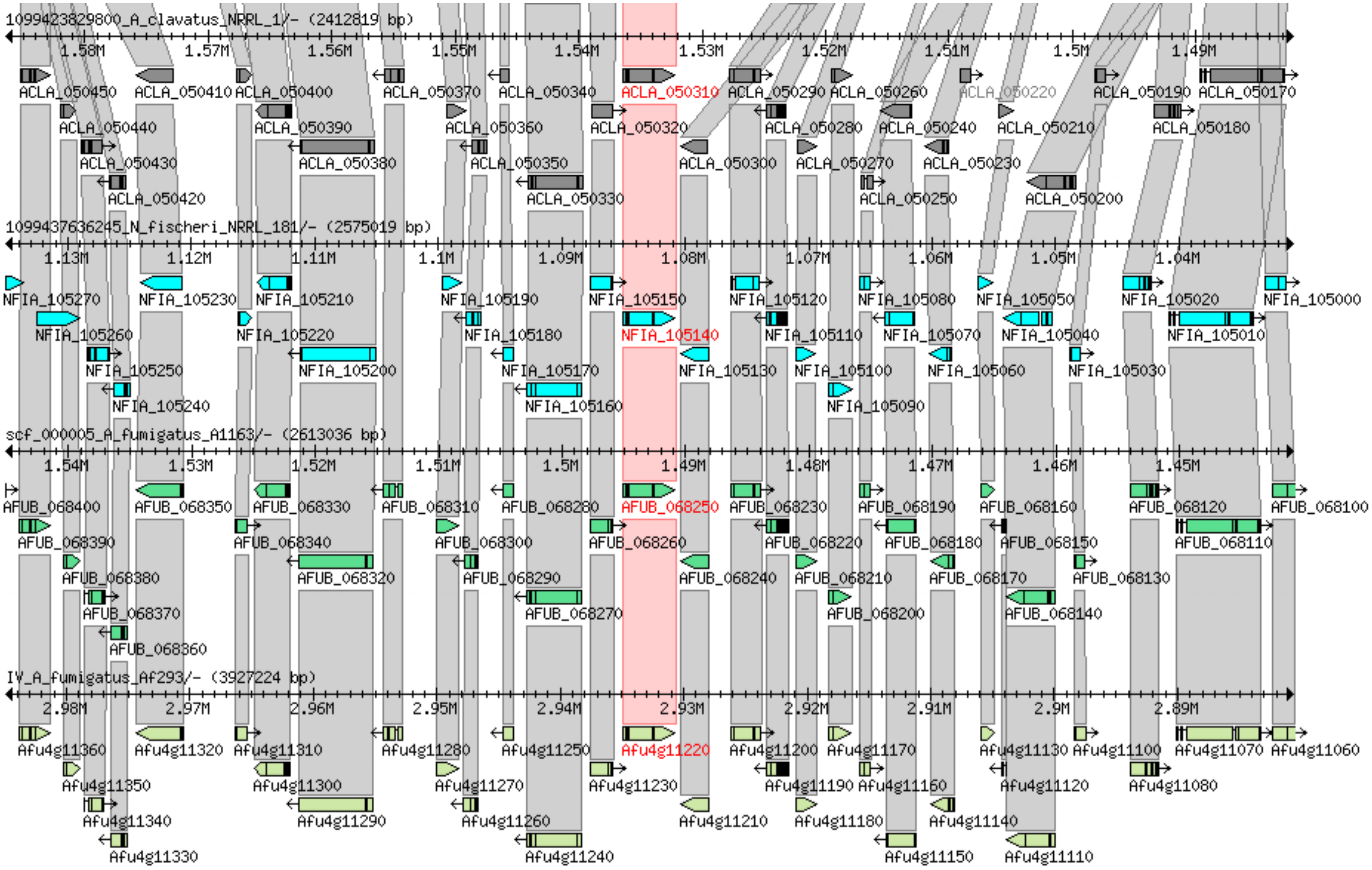

No PKS or NRPS backbone 2

Gene id (SMURF)	Gene positions	Gene distance	Annotated_gene_function (SMURF)	AspGD gene name	AspGD supplementary annotation	antiSMASH ID	Gene ID (I)	Gene ID (II)
n/a					Protein of unknown function	prot_ID_854	Afu4g11170	AFUA_4G11170
n/a					AAA family ATPase	prot_ID_855	Afu4g11180	AFUA_4G11180
n/a					Kynurenine aminotransferase	prot_ID_856	Afu4g11190	AFUA_4G11190
n/a					Protein of unknown function	prot_ID_857	Afu4g11200	AFUA_4G11200
n/a					Ortholog(s) have role in plasma membrane fusion involved in cytogamy, response to pheromone and mating projection tip localization	prot_ID_858	Afu4g11210	AFUA_4G11210
n/a					Ortholog(s) have xanthine dehydrogenase activity and role in purine nucleobase catabolic process	prot_ID_859	Afu4g11220	AFUA_4G11220
n/a					Protein of unknown function	prot_ID_860	Afu4g11230	AFUA_4G11230
n/a				aarA	Alpha-aminoadipate reductase, large subunit	prot_ID_861	Afu4g11240	AFUA_4G11240
n/a				cafA	Carbonic anhydrase	prot_ID_862	Afu4g11250	AFUA_4G11250
n/a					Oxidoreductase	prot_ID_863	Afu4g11260	AFUA_4G11260
n/a					Protein of unknown function	prot_ID_864	Afu4g11270	AFUA_4G11270
n/a					Dolichyl-phosphate-mannose-glycolipid alpha-mannosyltransferase	prot_ID_865	Afu4g11280	AFUA_4G11280
n/a					Peptidase activator activity	prot_ID_866	Afu4g11290	AFUA_4G11290
n/a					Vacuolar ATPase 98 kDa subunit	prot_ID_867	Afu4g11300	AFUA_4G11300

No PKS or NRPS backbone 3
No manual prediction made

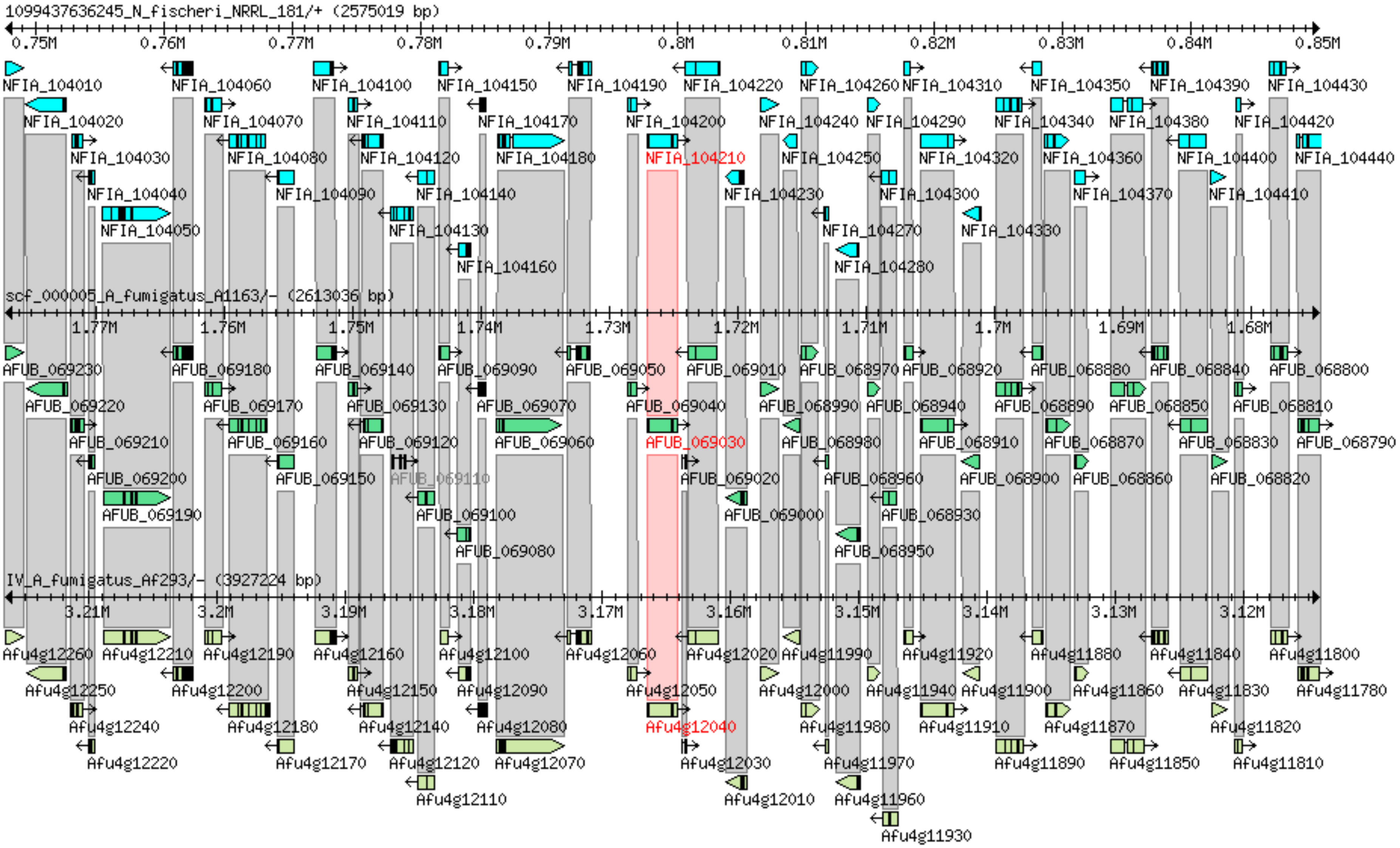

No PKS or NRPS backbone 3

Gene id (SMURF)	Gene positions	Gene distance	Annotated_gene_function (SMURF)	AspGD gene name	AspGD supplementary annotation	antiSMASH ID	Gene ID (I)	Gene ID (II)
n/a					Anthranilate phosphoribosyl transferase	prot_ID_928	Afu4g11980	AFUA_4G11980
n/a					Protein of unknown function	prot_ID_929	Afu4g11990	AFUA_4G11990
n/a					Phosphatidylinositol phospholipase C	prot_ID_930	Afu4g12000	AFUA_4G12000
n/a					2-oxo acid dehydrogenases acyltransferase	prot_ID_931	Afu4g12010	AFUA_4G12010
n/a					Protein of unknown function	prot_ID_932	Afu4g12020	AFUA_4G12020
n/a					Protein of unknown function	prot_ID_933	Afu4g12030	AFUA_4G12030
n/a				erg7B	oxidosqualene:lanosterol cyclase	prot_ID_934	Afu4g12040	AFUA_4G12040
n/a					Thermoresistant gluconokinase	prot_ID_935	Afu4g12050	AFUA_4G12050
n/a					Vacuolar protein sorting-associated protein 26	prot_ID_936	Afu4g12060	AFUA_4G12060
n/a					Ortholog(s) have guanyl-nucleotide exchange factor activity,	prot_ID_937	Afu4g12070	AFUA_4G12070

No PKS or NRPS backbone 4

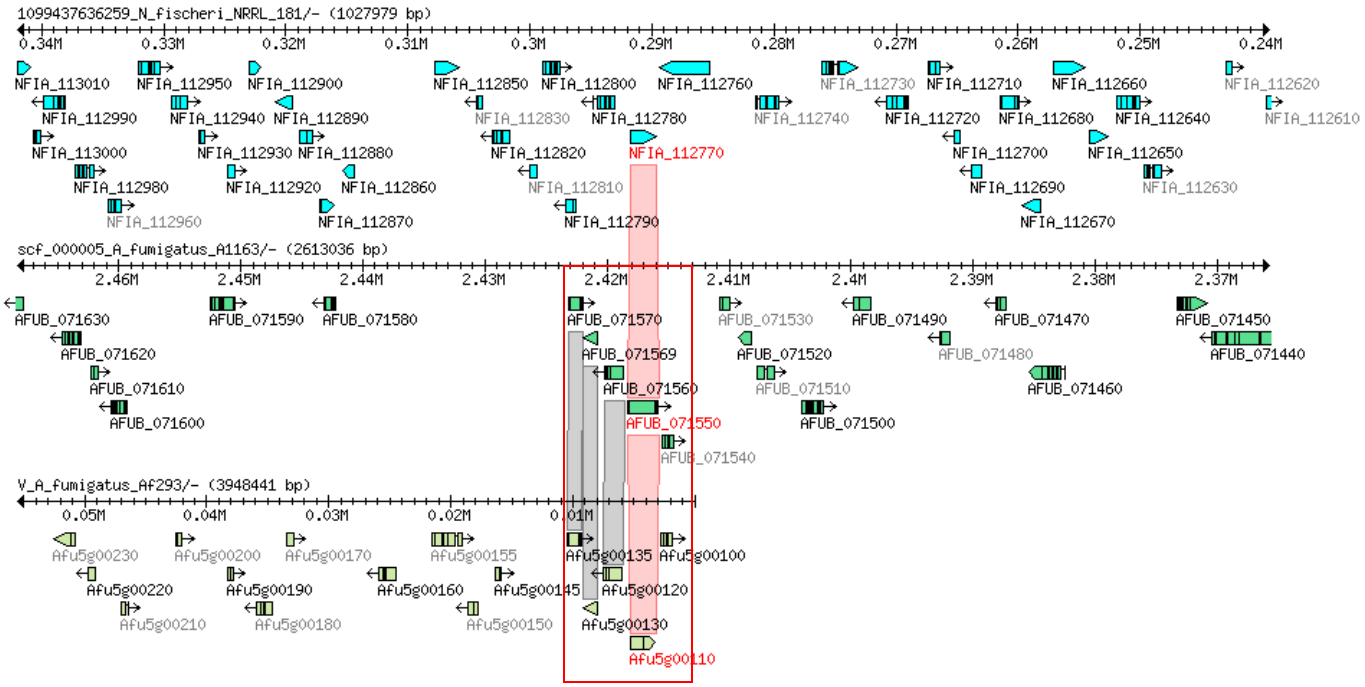

No PKS or NRPS backbone 4

Gene id (SMURF)	Gene positions	Gene distance	Annotated_gene_function (SMURF)	AspGD gene name	AspGD supplementary annotation	antiSMASH ID	Gene ID (I)	Gene ID (II)	
n/a					Protein of unknown function	prot_ID_1	Afu5g00100	AFUA_5G00100	ECS, IGD
n/a					Putative squalene-hopene-cyclase	prot_ID_2	Afu5g00110	AFUA_5G00110	
n/a					Cytochrome P450 oxidoreductase/alkane hydroxylase	prot_ID_3	Afu5g00120	AFUA_5G00120	
n/a					Capsule polysaccharide biosynthesis protein	prot_ID_4	Afu5g00130	AFUA_5G00130	
n/a					Putative transcription factor	prot_ID_1344	Afu5g00135	AFUA_5G00135	ECS, IGD
n/a					DNA damage repair protein (Rad9)	prot_ID_375	Afu5g04020	AFUA_5G04020	
n/a					Domain(s) with predicted structural constituent of ribosome activity, role in translation	prot_ID_376	Afu5g04030	AFUA_5G04030	
n/a					Protein of unknown function	prot_ID_377	Afu5g04040	AFUA_5G04040	
n/a					Scramblase family protein	prot_ID_378	Afu5g04050	AFUA_5G04050	
n/a					Ubiquitin-protein ligase	prot_ID_379	Afu5g04060	AFUA_5G04060	
n/a					Protein with similarity to Spo11	prot_ID_380	Afu5g04070	AFUA_5G04070	
n/a					Oxidosqualene:lanosterol cyclase	prot_ID_381	Afu5g04080	AFUA_5G04080	
n/a					SGT1 and CS domain protein	prot_ID_382	Afu5g04090	AFUA_5G04090	
n/a				gprF	G-protein coupled receptor	prot_ID_383	Afu5g04100	AFUA_5G04100	
n/a					DDE1 transposon-related ORF	prot_ID_384	Afu5g04110	AFUA_5G04110	
n/a					Hydrolase	prot_ID_385	Afu5g04120	AFUA_5G04120	
n/a					Cyclin-dependent protein kinase	prot_ID_386	Afu5g04130	AFUA_5G04130	

No PKS or NRPS backbone 5

Possible cluster based on interspecies synteny indicated

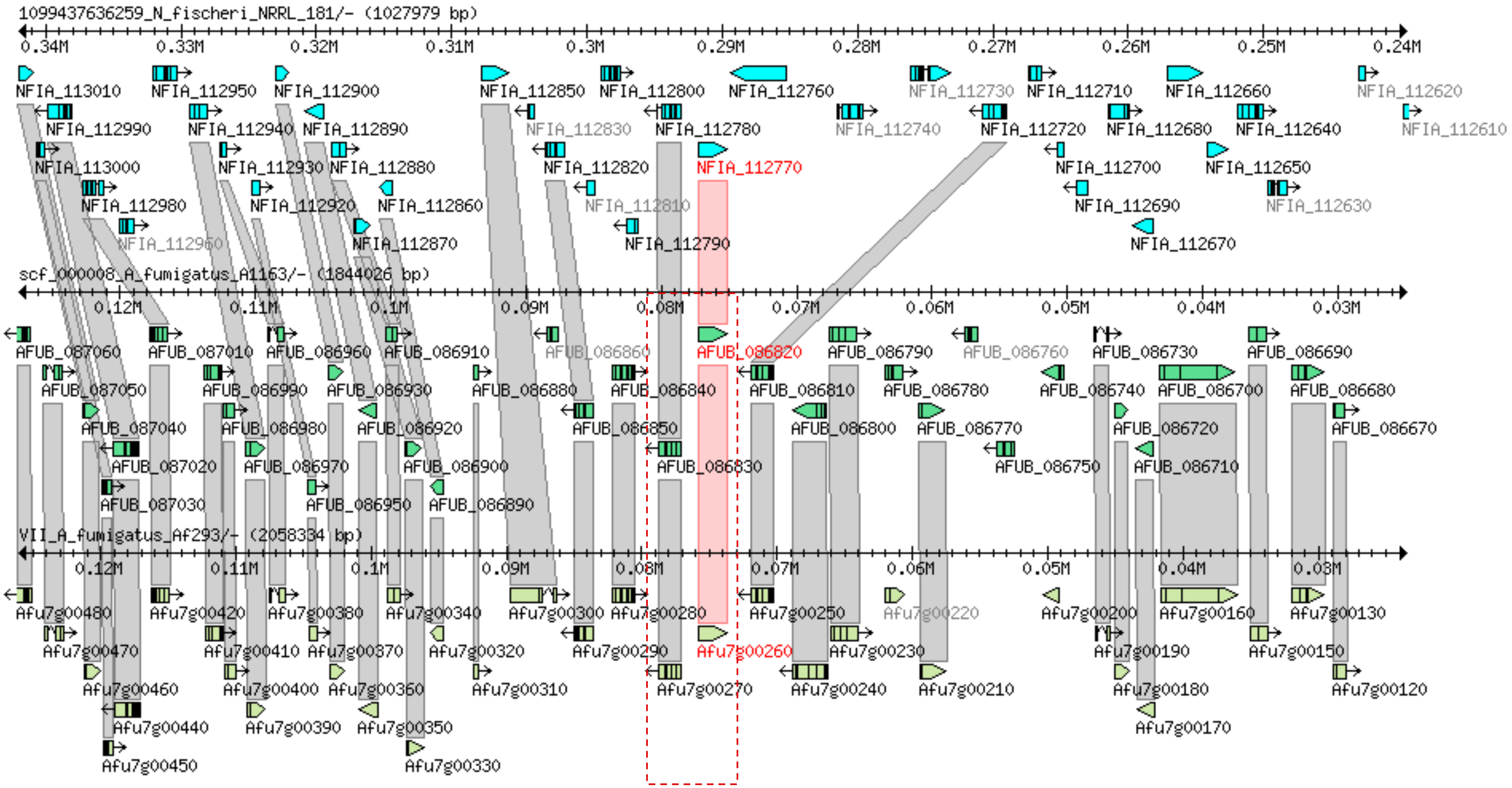

No PKS or NRPS backbone 5

Gene id (SMURF)	Gene positions	Gene distance	Annotated_gene_function (SMURF)	AspGD gene name	AspGD supplementary annotation	antiSMASH ID	Gene ID (I)	Gene ID (II)	
n/a					Transmembrane transporter	prot_ID_14	Afu7g00230	AFUA_7G00230	
n/a					Hydrolase	prot_ID_15	Afu7g00240	AFUA_7G00240	
n/a				tub2	Tubulin beta-2 subunit	prot_ID_16	Afu7g00250	AFUA_7G00250	
n/a					Domain(s) with predicted intramolecular transferase activity and role in hopanoid biosynthetic process	prot_ID_17	Afu7g00260	AFUA_7G00260	ECS, FA
n/a					Protein of unknown function	prot_ID_18	Afu7g00270	AFUA_7G00270	ECS
n/a					Transmembrane transporter	prot_ID_19	Afu7g00280	AFUA_7G00280	
n/a					Protein of unknown function	prot_ID_20	Afu7g00290	AFUA_7G00290	
n/a					Protein of unknown function	prot_ID_21	Afu7g00300	AFUA_7G00300	
n/a					Protein of unknown function	prot_ID_22	Afu7g00310	AFUA_7G00310	
n/a					Protein of unknown function	prot_ID_23	Afu7g00320	AFUA_7G00320	
n/a					Protein of unknown function	prot_ID_24	Afu7g00330	AFUA_7G00330	
n/a					Hydrolase	prot_ID_25	Afu7g00340	AFUA_7G00340	
					NAD-dependent dehydrogenase	prot_ID_26	Afu7g00350	AFUA_7G00350	

No PKS or NRPS backbone 6

Possible cluster based on interspecies synteny indicated

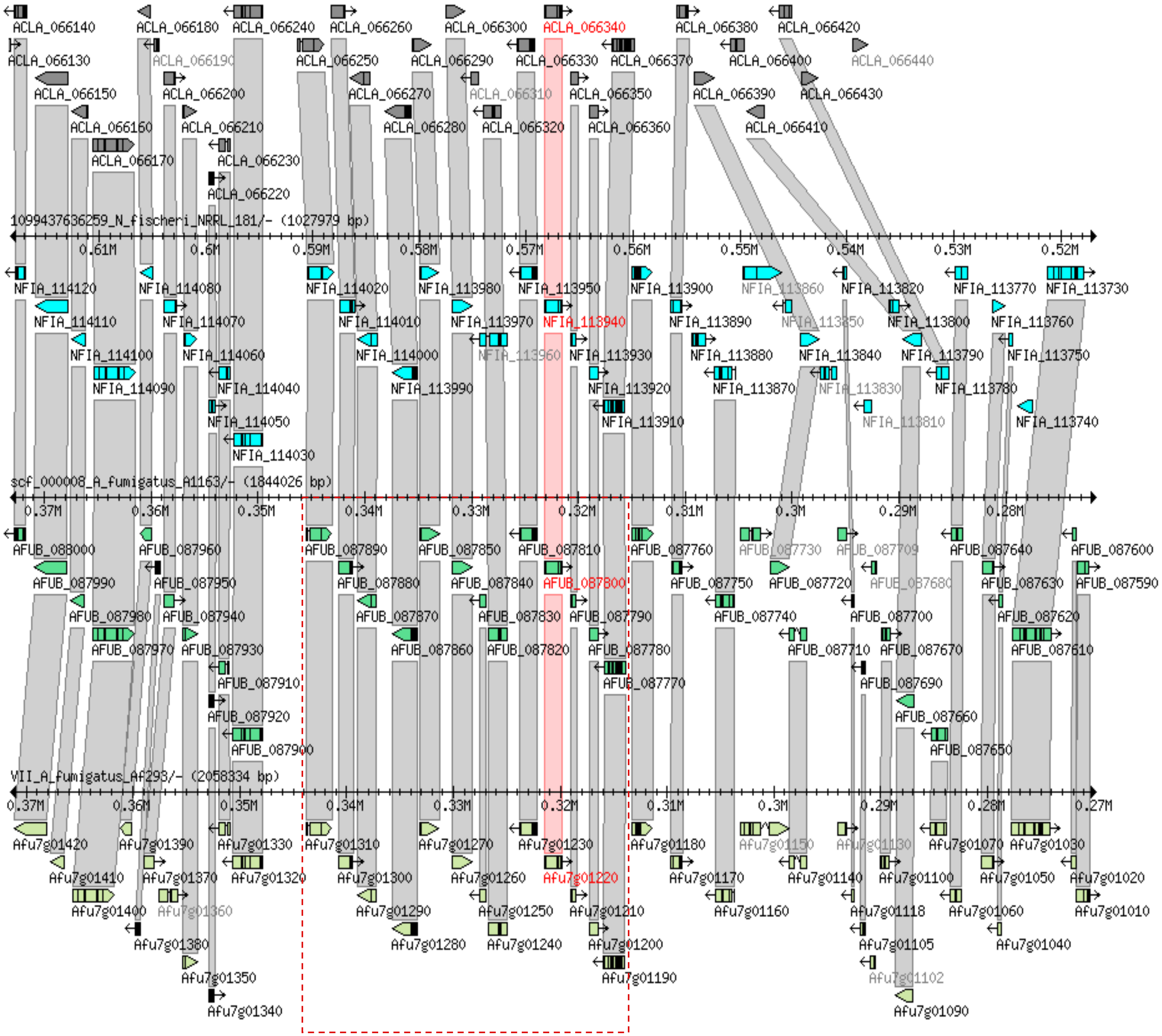

No PKS or NRPS backbone 6

Gene id (SMURF)	Gene positions	Gene distance	Annotated_gene_function (SMURF)	AspGD gene name	AspGD supplementary annotation	antiSMASH ID	Gene ID (I)	Gene ID (II)	
n/a					Protein of unknown function	prot_ID_100	Afu7g01180	AFUA_7G01180	
n/a					Transmembrane transporter	prot_ID_101	Afu7g01190	AFUA_7G01190	ECS
n/a					Aspergillopepsin	prot_ID_102	Afu7g01200	AFUA_7G01200	
n/a					Protein of unknown function	prot_ID_103	Afu7g01210	AFUA_7G01210	
n/a				erg9	squalene synthetase	prot_ID_104	Afu7g01220	AFUA_7G01220	
n/a					Domain(s) with predicted heat shock protein binding	prot_ID_105	Afu7g01230	AFUA_7G01230	
n/a					Domain(s) with predicted acid phosphatase activity	prot_ID_106	Afu7g01240	AFUA_7G01240	
n/a					Protein of unknown function	prot_ID_107	Afu7g01250	AFUA_7G01250	
n/a					Protein of unknown function	prot_ID_108	Afu7g01260	AFUA_7G01260	
n/a					Domain(s) with predicted DNA-directed DNA polymerase activity, damaged DNA binding activity and role in DNA repair	prot_ID_109	Afu7g01270	AFUA_7G01270	
					campothecin resistance conferring protein rcaA	n/a	Afu7g01280	AFUA_7G01280	
					Protein of unknown function	n/a	Afu7g01290	AFUA_7G01290	
					GPI anchor biosynthesis protein	n/a	Afu7g01300	AFUA_7G01300	
					C6 transcription factor	n/a	Afu7g01310	AFUA_7G01310	IGD

Abbreviations: ECS, end of cluster synteny; IGD, increase in intergenic distance; FA, change in functional annotation; ED, experimentally determined
