# Supplementary material for: Comprehensive annotation of secondary metabolite biosynthetic genes and gene clusters of Aspergillus nidulans, A. fumigatus, A. niger and A. oryzae
Source: BMC Microbiol. 2013 Apr 26;13:91. doi: 10.1186/1471-2180-13-91 (PMC3689640; doi:10.1186/1471-2180-13-91)
Supplement: Additional file 4 — A table listing the manually annotated gene clusters predicted by SMURF and antiSMASH for A. niger. [file 1471-2180-13-91-S4.pdf]

Additional file 4

Comprehensive annotation of secondary metabolite biosynthetic genes and gene clusters of *Aspergillus nidulans*, *A. fumigatus*, *A. niger* and *A. oryzae*

Diane O. Inglis, Jon Binkley, Marek Skrzypek, Martha B. Arnaud, Gustavo Cerqueira, Prach Shah, Farrell Wymore, Jennifer R. Wortman and Gavin Sherlock

Abbreviations: ECS, end of cluster synteny; IGD, increase in intergenic distance; FA, change in functional annotation; ED, experimentally determined

An01g01130 cluster

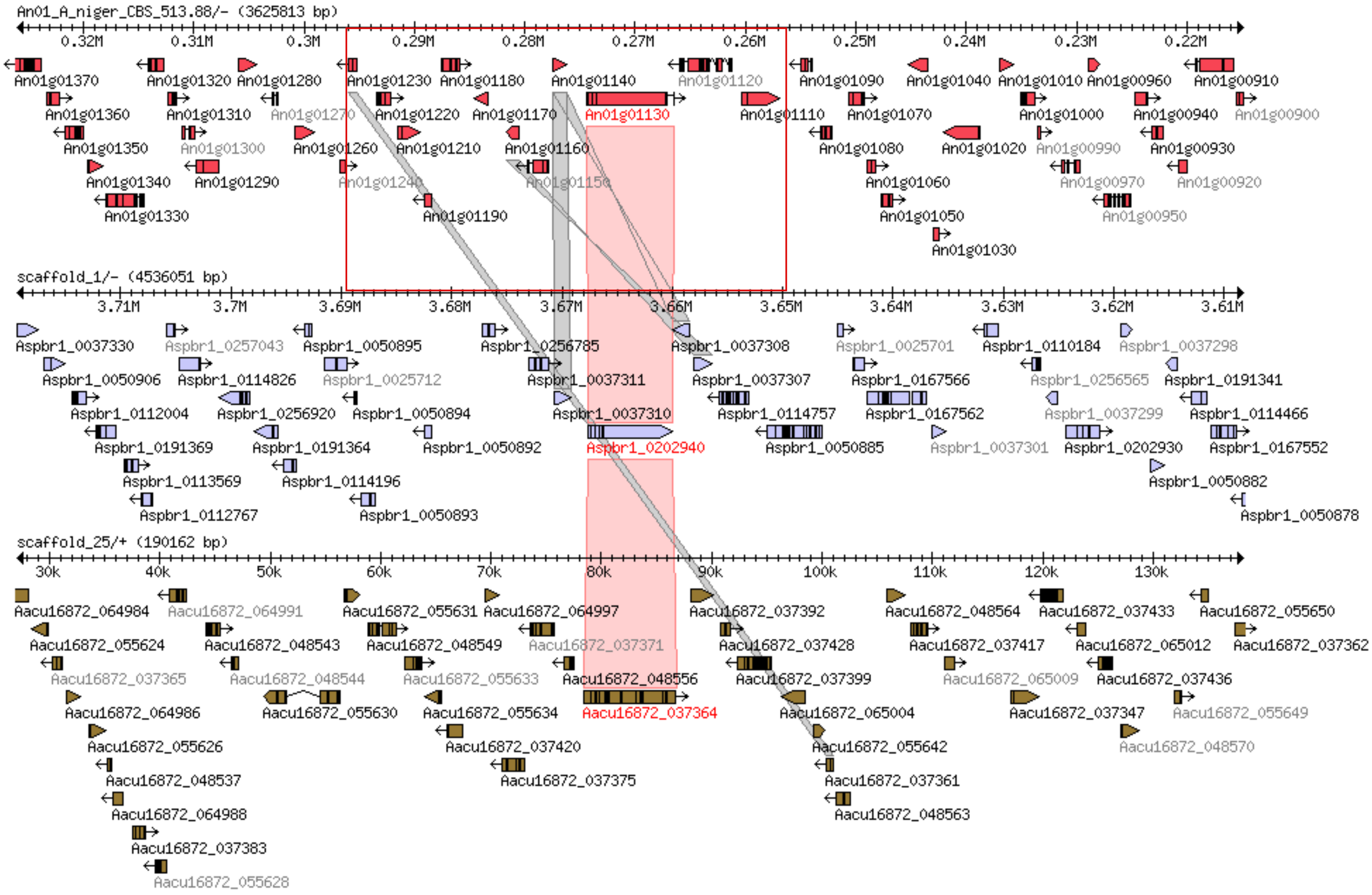

An01g01130 cluster

Gene id (SMURF)	Gene positions	Gene distance	AspGD name	AspGD description line or supplemental annotation	protein ID (antiSMASH)	AspGD ID	
n/a				Protein of unknown function	prot_ID_958	An01g01050	
n/a				Putative splicing factor	prot_ID_1184	An01g01060	
n/a				Oxidoreductase	prot_ID_1058	An01g01070	
An01g01080	4	807		Domain(s) with predicted nucleotide binding, oxidoreductase activity and role in oxidation-reduction process	prot_ID_1314	An01g01080	
An01g01090	3	2034		Similarity to TATA-binding protein-associated phosphoprotein Dr1	prot_ID_1401	An01g01090	
An01g01110	2	807		Domain(s) with predicted hydrolase activity	prot_ID_392	An01g01110	FA
An01g01120	1	516		Protein of unknown function	prot_ID_243	An01g01120	
**An01g01130**	0	0		Polyketide synthase	**prot_ID_1186**	**An01g01130**	
An01g01140	-1	1790		Ortholog of A. nidulans FGSC A4 : AN6764, AN2660, AN7798, A. fumigatus Af293 : Afu8g01960 and A. niger CBS 513.88 : An15g04120, An11g05430, An01g12050, An11g03480	prot_ID_1125	An01g01140	
An01g01150	-2	261		Similar to surface recognition protein PTH11	prot_ID_942	An01g01150	
n/a				Has domain(s) with predicted hydrolase activity and role in metabolic process	prot_ID_849	An01g01160	
n/a				Ortholog of A. nidulans FGSC A4 : AN5419, A. niger CBS 513.88 : An18g02752, A. oryzae RIB40 : AO090012000481, A. niger ATCC 1015 : 206357-mRNA and A. versicolor : Aspve1_0135496	prot_ID_730	An01g01170	
n/a				Has domain(s) with predicted chromate transmembrane transporter activity and role in chromate transport	prot_ID_69	An01g01180	
n/a				Ortholog of A. oryzae RIB40 : AO090701000875, A. brasiliensis : Aspbr1_0654792, Aspergillus flavus NRRL 3357 : AFL2T_03382, AFL2T_06438 and A. versicolor : Aspve1_0130993	prot_ID_304	An01g01190	
n/a				Has domain(s) with predicted serine-type peptidase activity and role in proteolysis	prot_ID_118	An01g01210	
n/a				Ortholog of A. nidulans FGSC A4 : AN11159, A. niger CBS 513.88 : An18g00430, A. brasiliensis : Aspbr1_0058364, A. niger ATCC 1015 : 188035-mRNA and A. clavatus NRRL 1 : ACLA_074330	prot_ID_1089	An01g01220	
n/a				Protein of unknown function	n/a	An01g01230	ECS

An01g06930/ An01g06950 cluster

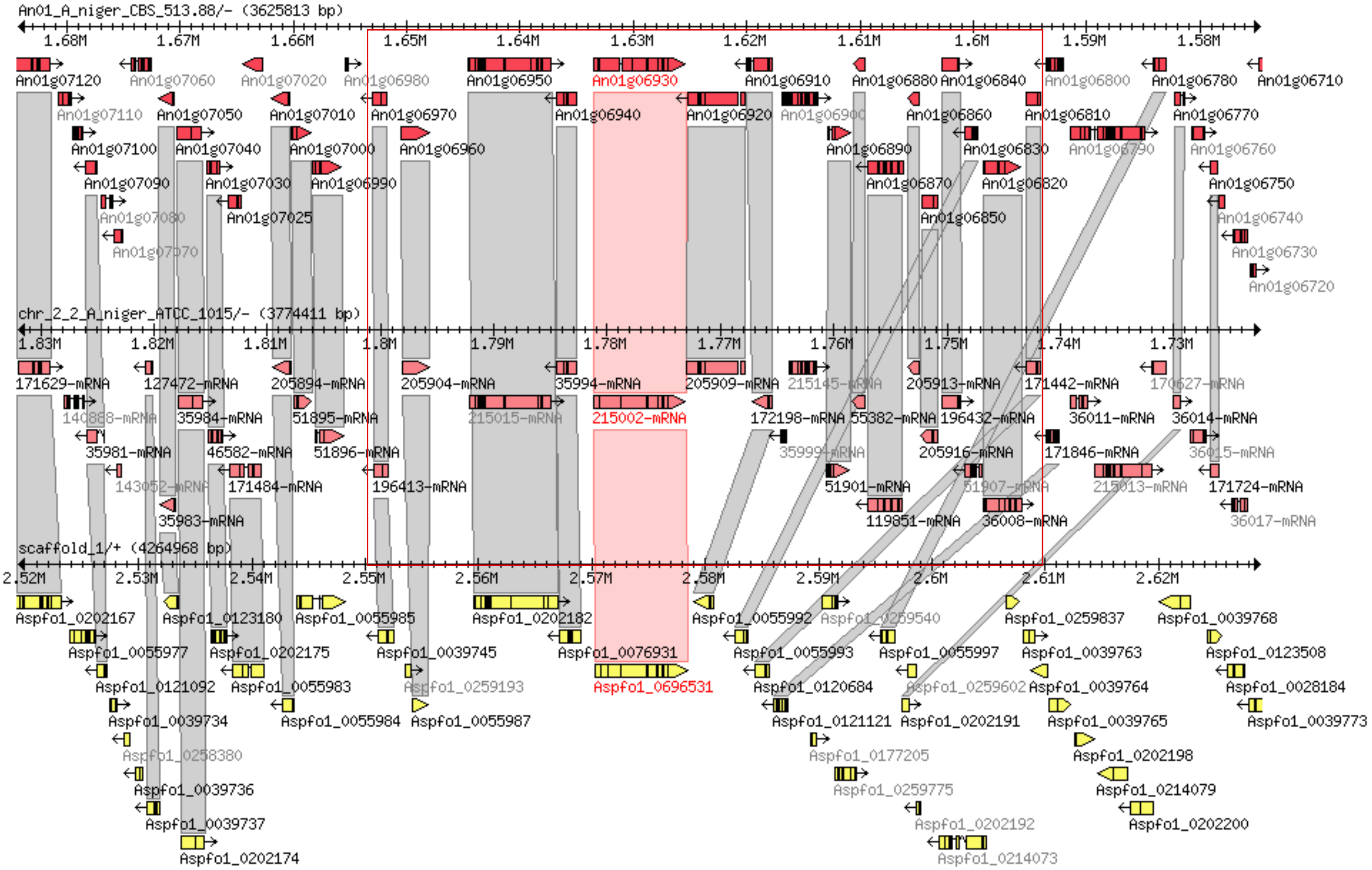

An01g06930 cluster

Gene id (SMURF)	Gene positions	Gene distance	AspGD name	AspGD description line or supplemental annotation	protein ID (antiSMASH)	AspGD ID	
An01g06730	20	721		Protein of unknown function	n/a	An01g06730	
An01g06740	19	142		Protein of unknown function	n/a	An01g06740	
An01g06750	18	424		Domain(s) with predicted N-acetyltransferase activity and role in metabolic process	n/a	An01g06750	
An01g06760	17	632		Protein of unknown function	n/a	An01g06760	
An01g06770	16	736		Ortholog of A. oryzae RIB40 : AO090012000668, A. brasiliensis : Aspbr1_0049222, Aspergillus flavus NRRL 3357 : AFL2T_03545 and A. acidus : Aspfo1_0202191	prot_ID_542	An01g06770	
An01g06780	15	553		Domain(s) with predicted nucleotide binding activity	prot_ID_1315	An01g06780	
An01g06790	14	704		Protein of unknown function	prot_ID_978	An01g06790	
An01g06800	13	434		Domain(s) with predicted hydrolase activity, acting on carbon-nitrogen (but not peptide) bonds, in linear amides activity, role in ceramide metabolic process and integral to membrane localization	prot_ID_1056	An01g06800	
An01g06810	12	362		Domain(s) with predicted 5-methyltetrahydropteroyltriglutamate-homocysteine S-methyltransferase activity and role in methionine biosynthetic process	prot_ID_473	An01g06810	ECS
An01g06820	11	550		Domain(s) with predicted FMN binding, NADPH-hemoprotein reductase activity, aromatase activity, electron carrier activity, heme binding, iron ion binding activity and role in oxidation-reduction process	prot_ID_174	An01g06820	
An01g06830	10	389		3-dehydrosphinganine reductase	prot_ID_687	An01g06830	
An01g06840	9	413		Putative acid-CoA ligase	prot_ID_1211	An01g06840	
An01g06850	8	347		Domain(s) with predicted metal ion binding, oxidoreductase activity and role in oxidation-reduction process	prot_ID_372	An01g06850	
An01g06860	7	615		Ortholog(s) have dioxygenase activity and role in austinol biosynthetic process, dehydroaustinol biosynthetic process, secondary metabolite biosynthetic process	prot_ID_506	An01g06860	
An01g06870	6	319		Domain(s) with predicted pyridoxal phosphate binding, transferase activity, transferring nitrogenous groups activity and role in biosynthetic process	prot_ID_582	An01g06870	
An01g06880	5	342		Domain(s) with predicted catalytic activity, coenzyme binding, nucleotide binding activity and role in cellular metabolic process	prot_ID_449	An01g06880	
An01g06890	4	758		Domain(s) with predicted ACP phosphopantetheine attachment site binding involved in fatty acid biosynthetic	prot_ID_459	An01g06890	

				process activity			
An01g06900	3	985		Domain(s) with predicted DNA binding, sequence-specific DNA binding RNA polymerase II transcription factor activity, sequence-specific DNA binding transcription factor activity, zinc ion binding activity	prot_ID_1399	An01g06900	
An01g06910	2	307		Domain(s) with predicted electron carrier activity, heme binding, iron ion binding, oxidoreductase activity, acting on paired donors, with incorporation or reduction of molecular oxygen activity and role in oxidation-reduction process	prot_ID_749	An01g06910	
An01g06920	1	439		Domain(s) with predicted ATP binding, ATPase activity, coupled to transmembrane movement of substances activity, role in transmembrane transport and integral to membrane localization	prot_ID_943	An01g06920	
**An01g06930**	0	0		Polyketide synthase	**prot_ID_695**	**An01g06930**	
An01g06940	-1	1532		Domain(s) with predicted role in transmembrane transport and integral to membrane localization	prot_ID_1431	An01g06940	
An01g06950	-2	381		Polyketide synthase. Ortholog(s) have role in asperfuranone biosynthetic process, emericellamide biosynthetic process, monodictyphenone biosynthetic process, o-orsellinic acid biosynthetic process, sterigmatocystin biosynthetic process	prot_ID_784	An01g06950	
n/a				Ortholog(s) have role in asperfuranone biosynthetic process, emericellamide biosynthetic process, monodictyphenone biosynthetic process, o-orsellinic acid biosynthetic process, sterigmatocystin biosynthetic process	prot_ID_1001	An01g06960	
n/a			ara1	Ortholog(s) have intracellular localization	prot_ID_905	An01g06970	ECS, FA
n/a				Glycerol dehydrogenase; D-arabinose 1-dehydrogenase [NAD(P)+]; protein levels influenced by presence of starch	prot_ID_37	An01g06980	
n/a				Protein of unknown function	prot_ID_642	An01g06990	
n/a				Ortholog of A. nidulans FGSC A4 : AN10694, A. fumigatus Af293 : Afu4g11740, A. niger ATCC 1015 : 51896-mRNA, A. versicolor : Aspve1_0042220 and A. sydowii : Aspsy1_0149194	prot_ID_354	An01g07000	
n/a				C14 sterol reductase; predicted role in ergosterol synthesis; expression repressed by tunicamycin and DTT; induced by fenpropimorph	prot_ID_1412	An01g07010	
n/a				Has domain(s) with predicted zinc ion binding activity	prot_ID_203	An01g07020	

An01g11770 cluster

No ATCC 1015 orthologous cluster

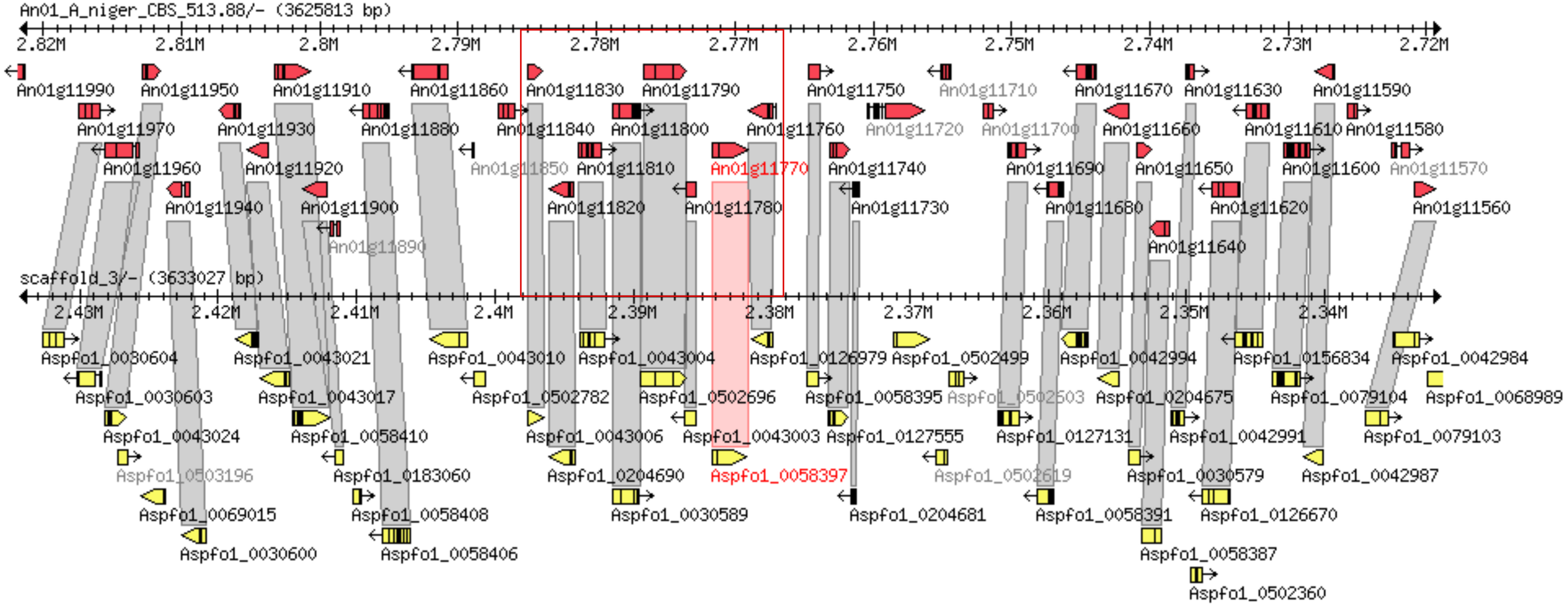

An01g11770 cluster

Gene id (SMURF)	Gene positions	Gene distance	AspGD name	AspGD description line or supplemental annotation	protein ID (antiSMASH)	AspGD ID	
n/a				Ortholog of A. nidulans FGSC A4 : AN8943, A. fumigatus Af293 : Afu2g00900, Afu6g11560, A. niger CBS 513.88 : An05g02420, An03g01300 and A. oryzae RIB40 : AO090113000175, AO090023000422	prot_ID_168	An01g11690	
n/a					prot_ID_693	An01g11700	
n/a				Has domain(s) with predicted transmembrane transporter activity, role in transmembrane transport and integral to membrane localization	prot_ID_798	An01g11710	
n/a				Has domain(s) with predicted FMN binding, iron-sulfur cluster binding, oxidoreductase activity	prot_ID_423	An01g11720	
n/a				Ortholog of A. acidus : Aspfo1_0204681	prot_ID_1350	An01g11730	
An01g11740	3	672		Domain(s) with predicted dipeptidase activity, dipeptidyl-peptidase activity, metalloexopeptidase activity and role in proteolysis	prot_ID_1077	An01g11740	
An01g11750	2	2380		Domain(s) with predicted ATP binding, protein kinase activity and role in protein phosphorylation	prot_ID_303	An01g11750	
An01g11760	1	175		Domain(s) with predicted role in transmembrane transport and integral to membrane localization	prot_ID_1380	An01g11760	IGD, ECS
**An01g11770**	0	0		Domain(s) with predicted ACP phosphopantetheine attachment site binding involved in fatty acid biosynthetic process, ligase activity, phosphopantetheine binding activity	**prot_ID_654**	**An01g11770**	
An01g11780	-1	1165		Domain(s) with predicted hydrolase activity	prot_ID_1296	An01g11780	
An01g11790	-2	65		Ortholog(s) have role in secondary metabolic process	prot_ID_435	An01g11790	
An01g11800	-3	271		Domain(s) with predicted monooxygenase activity and role in oxidation-reduction process	prot_ID_1023	An01g11800	
An01g11810	-4	1030		Domain(s) with predicted hydrolase activity	prot_ID_510	An01g11810	
An01g11820	-5	424		Domain(s) with predicted nucleic acid binding, nucleotide binding activity	prot_ID_929	An01g11820	
An01g11830	-6	509		Ortholog of A. brasiliensis : Aspbr1_0039523 and A. acidus : Aspfo1_0043006	prot_ID_1071	An01g11830	ECS, IGD
An01g11840	-7	908		Ortholog of A. nidulans FGSC A4 : AN7889, AN4657, A. fumigatus Af293 : Afu1g00920, Afu3g06425 and A. niger CBS 513.88 : An07g04250, An05g02120, An12g06960	prot_ID_1270	An01g11840	
An01g11850	-8	1765			prot_ID_836	An01g11850	

An01g11860	-9	666	Domain(s) with predicted DNA binding, sequence-specific DNA binding RNA polymerase II transcription factor activity, sequence-specific DNA binding transcription factor activity, zinc ion binding activity	prot_ID_1177	An01g11860
An01g11880	-10	1642	Domain(s) with predicted electron carrier activity, heme binding, iron ion binding, oxidoreductase activity, acting on paired donors, with incorporation or reduction of molecular oxygen activity and role in oxidation-reduction process	prot_ID_384	An01g11880
An01g11890	-11	1713		n/a	An01g11890
An01g11900	-12	295	Domain(s) with predicted nucleic acid binding, zinc ion binding activity and intracellular localization	n/a	An01g11900

An02g00210 cluster

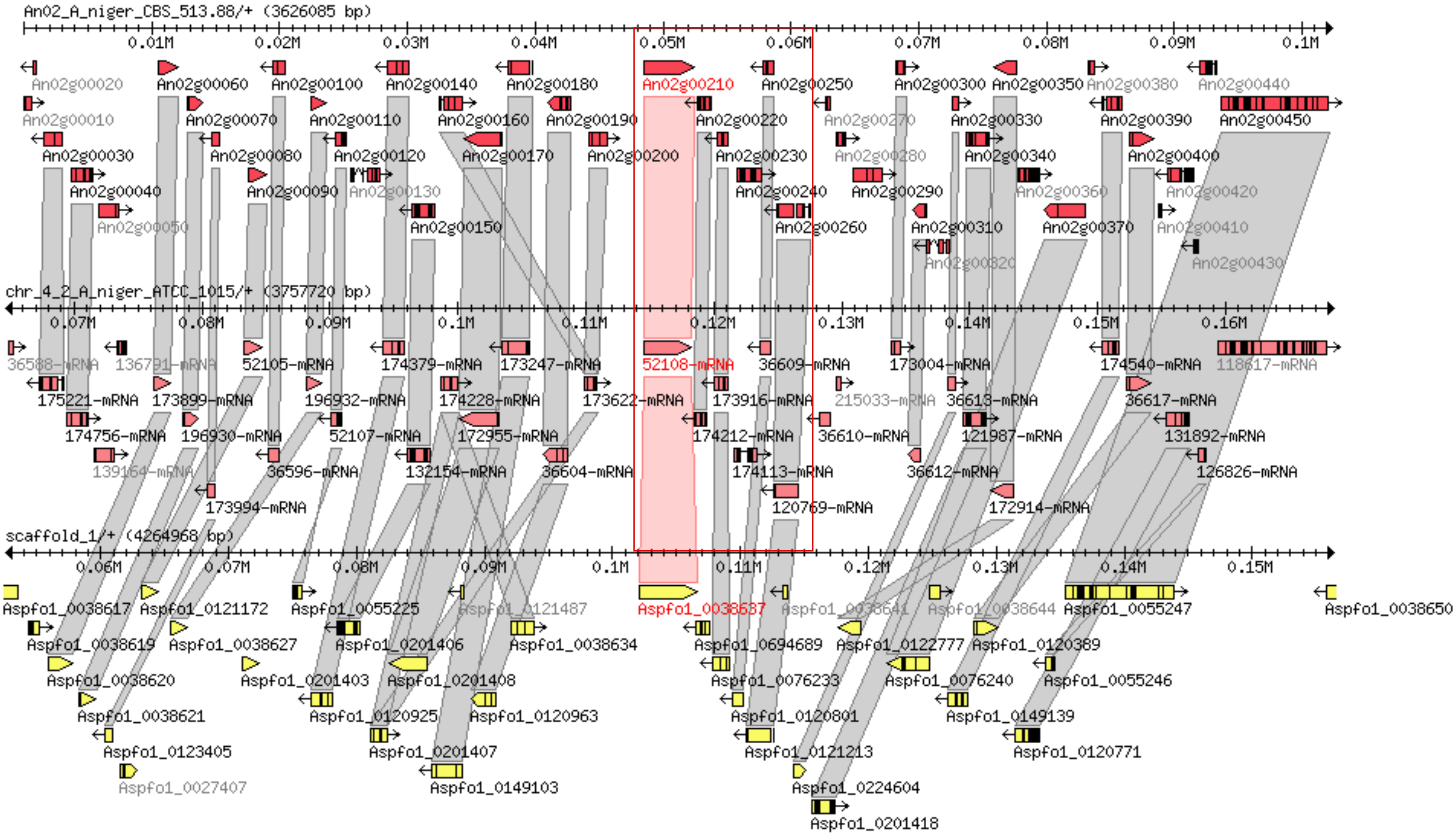

An02g00210 cluster

Gene id (SMURF)	Gene positions	Gene distance	AspGD name	AspGD description line or supplemental annotation	protein ID (antiSMASH)	AspGD ID	
n/a				Xylan-1,4-beta-xylosidase	prot_ID_725	An02g00140	
An02g00150	6	377		Domain(s) with predicted electron carrier activity, heme binding, iron ion binding, oxidoreductase activity, acting on paired donors, with incorporation or reduction of molecular oxygen activity and role in oxidation-reduction process	prot_ID_112	An02g00150	
An02g00160	5	118		Protein with strong similarity to appressorium differentiation protein pth11 of Magnaporthe grisea	prot_ID_783	An02g00160	
An02g00170	4	436		Domain(s) with predicted hydrolase activity	prot_ID_428	An02g00170	
An02g00180	3	1111		Putative phosphate-repressible phosphate permease	prot_ID_673	An02g00180	
An02g00190	2	1379		Protein similar to enantiomer-selective amidase; expression repressed by tunicamycin and DTT	prot_ID_679	An02g00190	
An02g00200	1	2966		Ortholog of A. nidulans FGSC A4 : AN8951, A. niger CBS 513.88 : An02g00160, A. oryzae RIB40 : AO090010000027, AO090011000356 and A. niger ATCC 1015 : 174228-mRNA	prot_ID_106	An02g00200	
**An02g00210**	0	0		NRPS-like protein	**prot_ID_621**	**An02g00210**	ECS
An02g00220	-1	363		Ortholog(s) have tetrahydroxynaphthalene reductase activity, versicolorin reductase activity, role in melanin biosynthetic process, monodictyphenone biosynthetic process, sterigmatocystin biosynthetic process and intracellular localization	prot_ID_1155	An02g00220	
An02g00230	-2	543		Ortholog(s) have dioxygenase activity and role in austinol biosynthetic process, dehydroaustinol biosynthetic process, secondary metabolite biosynthetic process	prot_ID_1019	An02g00230	
An02g00240	-3	675		Domain(s) with predicted amino acid transmembrane transporter activity, role in amino acid transmembrane transport and membrane localization	prot_ID_1107	An02g00240	
An02g00250	-4	56		Domain(s) with predicted deoxyribose-phosphate aldolase activity, role in deoxyribonucleotide catabolic process and cytoplasm localization	prot_ID_686	An02g00250	
An02g00260	-5	246		Ortholog(s) have role in positive regulation of transcription from RNA polymerase II promoter, short-chain fatty acid catabolic process	prot_ID_1032	An02g00260	ECS
An02g00270	-6	1160		Protein of unknown function	prot_ID_820	An02g00270	
An02g00280	-7	482		Domain(s) with predicted role in transmembrane transport and integral to membrane localization	prot_ID_1091	An02g00280	

An02g00290	-8	553	Domain(s) with predicted DNA binding, sequence-specific DNA binding RNA polymerase II transcription factor activity, sequence-specific DNA binding transcription factor activity, zinc ion binding activity	prot_ID_659	An02g00290
n/a			Has domain(s) with predicted DNA binding, sequence-specific DNA binding RNA polymerase II transcription factor activity, sequence-specific DNA binding transcription factor activity, zinc ion binding activity	prot_ID_3	An02g00300
n/a			Has domain(s) with predicted hydrolase activity	prot_ID_651	An02g00310
n/a			Has domain(s) with predicted oxidoreductase activity and role in oxidation-reduction process	prot_ID_827	An02g00320

An02g00450 cluster

No ATCC 1015 ortlogous cluster, no manual prediction made

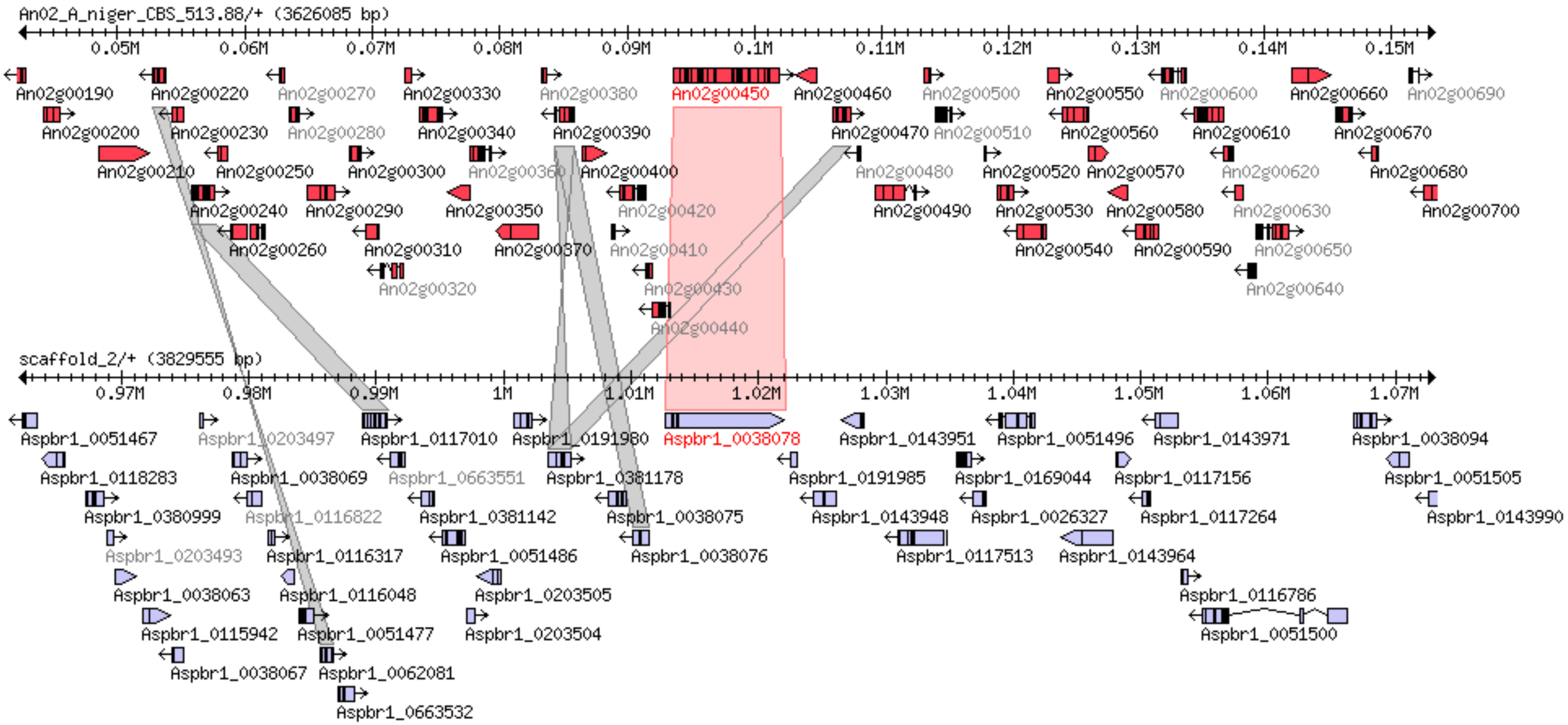

An02g00450 cluster

Gene id (SMURF)	Gene positions	Gene distance	AspGD name	AspGD description line or supplemental annotation	protein ID (antiSMASH)	AspGD ID
n/a				Putative amidase/acetamidase	prot_ID_1183	An02g00340
n/a				Tannin acyl hydrolase with a predicted role in tannic acid degradation	prot_ID_658	An02g00350
n/a					prot_ID_1094	An02g00360
n/a				Ortholog of A. nidulans FGSC A4 : AN9323, A. niger CBS 513.88 : An06g00670, A. oryzae RIB40 : AO090102000655 and A. versicolor : Aspve1_0090061, Aspve1_0142746	prot_ID_325	An02g00370
n/a					prot_ID_1316	An02g00380
n/a				Ortholog of A. nidulans FGSC A4 : AN6413, AN8328, AN3395, AN3241, AN10369, AN1930, AN2386, AN1738, AN9266, AN10886, AN7232, AN7406, AN7774, AN12202	prot_ID_179	An02g00390
n/a				Has domain(s) with predicted choline dehydrogenase activity, flavin adenine dinucleotide binding activity and role in alcohol metabolic process, oxidation-reduction process	prot_ID_1333	An02g00400
n/a					prot_ID_1263	An02g00410
n/a					prot_ID_802	An02g00420
An02g00430	2	102		Protein of unknown function	prot_ID_305	An02g00430
An02g00440	1	257		Protein of unknown function	prot_ID_20	An02g00440
**An02g00450**	0	0		Ortholog(s) have role in asperfuranone biosynthetic process, emericellamide biosynthetic process, monodictyphenone biosynthetic process, o-orsellinic acid biosynthetic process, sterigmatocystin biosynthetic process	**prot_ID_1412**	**An02g00450**
An02g00460	-1	1082		Domain(s) with predicted UDP-N-acetylmuramate dehydrogenase activity, flavin adenine dinucleotide binding activity and role in oxidation-reduction process	prot_ID_1137	An02g00460
An02g00470	-2	1333		Ortholog of A. nidulans FGSC A4 : AN6413, AN8328, AN3395, AN3241, AN10369, AN1930, AN2386, AN1738, AN9266, AN10886, AN7232, AN7406, AN7774, AN12202	prot_ID_212	An02g00470
An02g00480	-3	453		Protein of unknown function	prot_ID_198	An02g00480
An02g00490	-4	1081		Ortholog of A. nidulans FGSC A4 : AN6829, A. fumigatus Af293 : Afu5g12710, A. niger CBS 513.88 : An01g12010, A. niger ATCC 1015 : 46369-mRNA and A. versicolor : Aspve1_0120985, Aspve1_0178343	prot_ID_551	An02g00490
An02g00500	-5	548		Protein of unknown function	prot_ID_514	An02g00500
An02g00510	-6	326		Protein of unknown function	prot_ID_564	An02g00510
An02g00520	-7	2532		Ortholog of A. acidus : Aspfo1_0055254	prot_ID_1055	An02g00520

An02g00530	-8	824	Domain(s) with predicted role in response to stress and integral to membrane localization	prot_ID_1411	An02g00530
An02g00540	-9	238	Domain(s) with predicted DNA binding, sequence-specific DNA binding RNA polymerase II transcription factor activity, sequence-specific DNA binding transcription factor activity, zinc ion binding activity	prot_ID_1480	An02g00540
An02g00550	-10	75	Domain(s) with predicted oxidoreductase activity, acting on paired donors, with incorporation or reduction of molecular oxygen, 2-oxoglutarate as one donor, and incorporation of one atom each of oxygen into both donors activity	n/a	An02g00550
An02g00560	-11	147	Domain(s) with predicted transporter activity, role in transmembrane transport and membrane localization	n/a	An02g00560
An02g00570	-12	17	Domain(s) with predicted hydrolase activity, acting on carbon-nitrogen (but not peptide) bonds activity	n/a	An02g00570
An02g00580	-13	48	Domain(s) with predicted catalytic activity	n/a	An02g00580

An02g00840 cluster

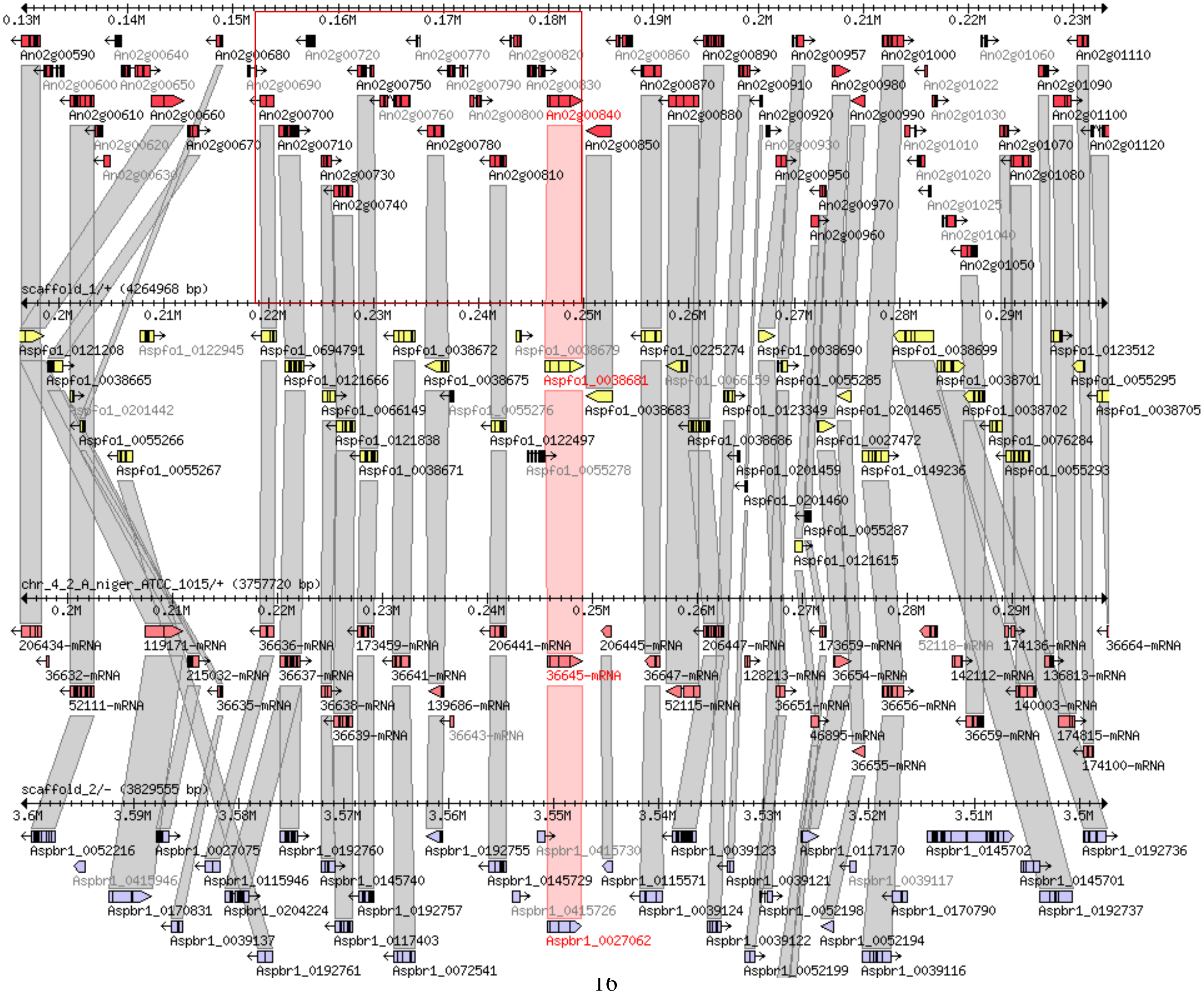

An02g00840 cluster

Gene id (SMURF)	Gene positions	Gene distance	AspGD name	AspGD description line or supplemental annotation	protein ID (antiSMASH)	AspGD ID	
n/a				Protein of unknown function	n/a	An02g00700	ECS, IGD
An02g00710	13	527		Domain(s) with predicted ATP binding activity	n/a	An02g00710	
An02g00720	12	591		Protein of unknown function	n/a	An02g00720	
An02g00730	11	191		Ortholog(s) have triglyceride lipase activity and role in triglyceride catabolic process	n/a	An02g00730	
An02g00740	10	526		Domain(s) with predicted UDP-N-acetylmuramate dehydrogenase activity, flavin adenine dinucleotide binding activity and role in oxidation-reduction process	prot_ID_813	An02g00740	
An02g00750	9	639		Ortholog of A. nidulans FGSC A4 : AN6413, AN8328, AN3395, AN3241, AN10369, AN1930, AN2386, AN1738, AN9266, AN10886, AN7232, AN7406, AN7774, AN12202	prot_ID_306	An02g00750	
An02g00760	8	585		Domain(s) with predicted role in transmembrane transport and integral to membrane localization	prot_ID_167	An02g00760	
An02g00770	7	659		Protein of unknown function	prot_ID_891	An02g00770	
An02g00780	6	276		Ortholog of A. nidulans FGSC A4 : AN1725, A. fumigatus Af293 : Afu2g14400, A. niger CBS 513.88 : An12g01310, A. oryzae RIB40 : AO090012000862, AO090023000064 and A. niger ATCC 1015 : 133532-mRNA	prot_ID_1475	An02g00780	
An02g00790	5	237		Protein of unknown function	prot_ID_56	An02g00790	
An02g00800	4	770		Protein of unknown function	prot_ID_437	An02g00800	
An02g00810	3	385		Domain(s) with predicted O-methyltransferase activity	prot_ID_169	An02g00810	
An02g00820	2	607		Protein of unknown function	prot_ID_931	An02g00820	
An02g00830	1	70		Protein of unknown function	prot_ID_55	An02g00830	
**An02g00840**	0	0		Domain(s) with predicted ACP phosphopantetheine attachment site binding involved in fatty acid biosynthetic process, catalytic activity, nucleotide binding activity	**prot_ID_1343**	**An02g00840**	ECS, FA
An02g00850	-1	610		Glucoamylase (exo-1,4-glucosidase/amyloglucosidase)	prot_ID_1362	An02g00850	
An02g00860	-2	425		Protein of unknown function	prot_ID_11	An02g00860	
n/a				Has domain(s) with predicted protein dimerization activity, sequence-specific DNA binding, sequence-specific DNA binding transcription factor activity and role in regulation of transcription, DNA-dependent	prot_ID_811	An02g00870	

n/a	Has domain(s) with predicted zinc ion binding activity	prot_ID_513	An02g00880
n/a	Phosphoserine transaminase	prot_ID_841	An02g00890
n/a	Has domain(s) with predicted nucleotide binding activity	prot_ID_538	An02g00910
n/a	Ortholog of A. brasiliensis : Aspbr1_0039121, A. acidus : Aspfo1_0201460 and Aspergillus carbonarius ITEM 5010 : Acar5010_001048	prot_ID_344	An02g00920
n/a		prot_ID_1208	An02g00930
n/a	Ortholog of A. versicolor : Aspve1_0031571, A. sydowii : Aspsy1_0048510, Aspergillus terreus NIH2624 : ATET_00686, ATET_05781 and A. niger ATCC 1015 : 36651-mRNA	prot_ID_1409	An02g00950

An02g05070 cluster

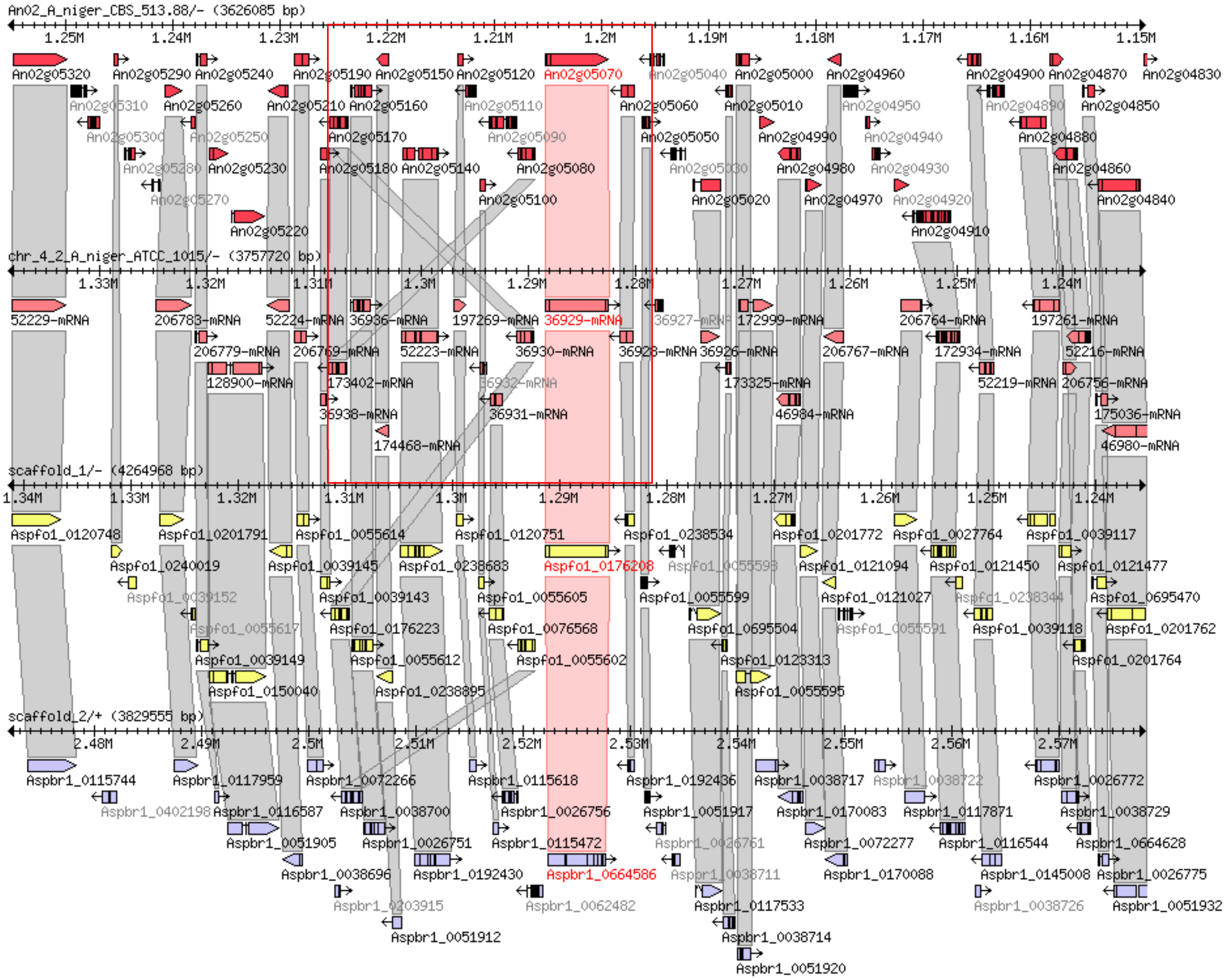

An02g05070 cluster

Gene id (SMURF)	Gene positions	Gene distance	AspGD name	AspGD description line or supplemental annotation	protein ID (antiSMASH)	AspGD ID	
n/a				Ortholog of A. nidulans FGSC A4 : AN6372, A. fumigatus Af293 : Afu2g13990, A. oryzae RIB40 : AO090026000161, A. versicolor : Aspve1_0083251 and A. sydowii : Aspsy1_0045547	prot_ID_699	An02g04970	
n/a				Has domain(s) with predicted ATP binding, ATP-dependent helicase activity, nucleic acid binding activity	prot_ID_1481	An02g04980	
n/a				Ortholog of Aspergillus carbonarius ITEM 5010 : Acar5010_512192	prot_ID_610	An02g04990	
n/a				Has domain(s) with predicted transferase activity, transferring acyl groups activity and role in metabolic process	prot_ID_390	An02g05000	
n/a				Ortholog of A. nidulans FGSC A4 : AN6376, A. oryzae RIB40 : AO090026000166, A. niger ATCC 1015 : 173325-mRNA, A. versicolor : Aspve1_0041315 and A. sydowii : Aspsy1_0058298	prot_ID_772	An02g05010	
n/a				Ortholog of A. nidulans FGSC A4 : AN6377, A. brasiliensis : Aspbr1_0117533, A. niger ATCC 1015 : 36926-mRNA, N. fischeri NRRL 181 : NFIA_089130 and A. clavatus NRRL 1 : ACLA_073670	prot_ID_105	An02g05020	
n/a				Protein of unknown function	prot_ID_1163	An02g05030	
n/a				Protein of unknown function	prot_ID_157	An02g05040	
An02g05050	2	876		Ortholog of A. brasiliensis : Aspbr1_0051917 and A. acidus : Aspfo1_0055599	prot_ID_471	An02g05050	ECS
An02g05060	1	1776		Ortholog of A. nidulans FGSC A4 : AN6378, A. oryzae RIB40 : AO090026000168, A. niger ATCC 1015 : 36928-mRNA, A. versicolor : Aspve1_0052621 and A. sydowii : Aspsy1_0045559	prot_ID_1201	An02g05060	
**An02g05070**	0	0		Domain(s) with predicted ACP phosphopantetheine attachment site binding involved in fatty acid biosynthetic process, ligase activity	**prot_ID_161**	**An02g05070**	
An02g05080	-1	1012		Ortholog(s) have role in monodictyphenone biosynthetic process, secondary metabolic process	prot_ID_1456	An02g05080	
An02g05090	-2	186		Domain(s) with predicted electron carrier activity, heme binding, iron ion binding, monooxygenase activity, oxidoreductase activity, acting on paired donors, with incorporation or reduction of molecular oxygen activity	prot_ID_75	An02g05090	
An02g05100	-3	449		Ortholog of A. fumigatus Af293 : Afu2g13940, A. oryzae RIB40 : AO090026000178, A. brasiliensis : Aspbr1_0115472, N. fischeri NRRL 181 : NFIA_089100 and Aspergillus flavus NRRL 3357 : AFL2T_07068	prot_ID_569	An02g05100	

An02g05110	-4	361	Protein of unknown function	prot_ID_168	An02g05110	
An02g05120	-5	203	Ortholog of A. nidulans FGSC A4 : AN6380, A. fumigatus Af293 : Afu2g13930, A. oryzae RIB40 : AO090026000179, A. niger ATCC 1015 : 197269-mRNA and A. versicolor : Aspve1_0052622	prot_ID_652	An02g05120	
An02g05140	-6	1735	Ortholog of A. oryzae RIB40 : AO090026000180, A. brasiliensis : Aspbr1_0192430, A. niger ATCC 1015 : 52223-mRNA, Aspergillus flavus NRRL 3357 : AFL2T_07066 and A. acidus : Aspfo1_0238683	prot_ID_678	An02g05140	
An02g05150	-7	1293	Domain(s) with predicted cholestenol delta-isomerase activity, role in sterol metabolic process and endoplasmic reticulum, integral to membrane localization	prot_ID_119	An02g05150	
An02g05160	-8	665	Ortholog of A. brasiliensis : Aspbr1_0026751, A. acidus : Aspfo1_0055612, A. versicolor : Aspve1_0028748 and A. sydowii : Aspsy1_0032344	prot_ID_752	An02g05160	
An02g05170	-9	259	Ortholog(s) have role in monodictyphenone biosynthetic process, secondary metabolic process	prot_ID_1350	An02g05170	ECS
An02g05180	-10	238	Ortholog of A. acidus : Aspfo1_0039143 and A. niger ATCC 1015 : 36938-mRNA	n/a	An02g05180	
An02g05190	-11	1281	Domain(s) with predicted nucleic acid binding, zinc ion binding activity	n/a	An02g05190	
n/a			Domain(s) with predicted calcium ion binding, calcium-dependent phospholipid binding activity	n/a	An02g05210	

An02g08290 cluster

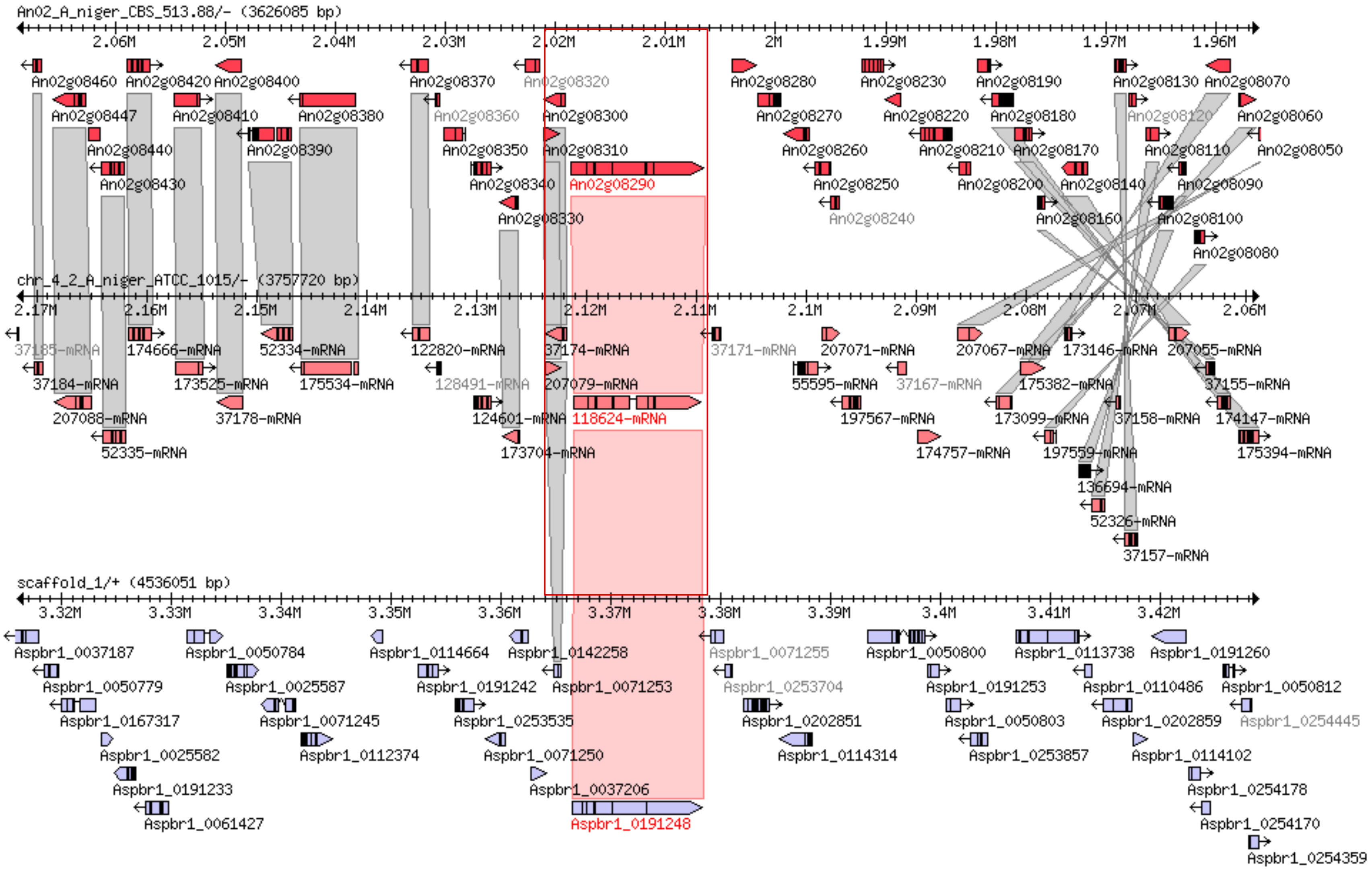

An02g08290 cluster

Gene id (SMURF)	Gene positions	Gene distance	AspGD name	AspGD description line or supplemental annotation	protein ID (antiSMASH)	AspGD ID
An02g08130	15	2827		Ortholog of A. nidulans FGSC A4 : AN2844, A. fumigatus Af293 : Afu3g12255, A. oryzae RIB40 : AO090003000790, A. niger ATCC 1015 : 37157-mRNA and A. versicolor : Aspve1_0080524	n/a	An02g08130
An02g08140	14	1396		Domain(s) with predicted protein tyrosine phosphatase activity, protein tyrosine/serine/threonine phosphatase activity and role in protein dephosphorylation	n/a	An02g08140
An02g08160	13	402		Domain(s) with predicted metal ion binding, phosphoric diester hydrolase activity	n/a	An02g08160
An02g08170	12	767		Ortholog of A. oryzae RIB40 : AO090003000788, A. brasiliensis : Aspbr1_0038368, Aspergillus flavus NRRL 3357 : AFL2T_02231 and A. acidus : Aspfo1_0204450	n/a	An02g08170
An02g08180	11	195		Domain(s) with predicted transmembrane transporter activity, role in transmembrane transport and integral to membrane localization	n/a	An02g08180
An02g08190	10	599		Ortholog of A. brasiliensis : Aspbr1_0389534 and A. acidus : Aspfo1_0493055	n/a	An02g08190
An02g08200	9	660		Domain(s) with predicted nucleotide binding activity	n/a	An02g08200
An02g08210	8	2228		Ortholog of A. nidulans FGSC A4 : AN3356, AN0718, AN5052, A. fumigatus Af293 : Afu1g14460, Afu3g01980, Afu3g12180, Afu8g06010 and A. niger CBS 513.88 : An14g06110, An02g00290, An02g13100, An06g00330	prot_ID_384	An02g08210
An02g08220	7	211		Domain(s) with predicted catalytic activity and role in metabolic process	prot_ID_1059	An02g08220
An02g08230	6	2168		Domain(s) with predicted substrate-specific transmembrane transporter activity, role in transmembrane transport and integral to membrane localization	prot_ID_600	An02g08230
An02g08240	5	65			prot_ID_12	An02g08240
An02g08250	4	705		Domain(s) with predicted cation transmembrane transporter activity, role in cation transport, transmembrane transport and integral to membrane localization	prot_ID_1056	An02g08250
An02g08260	3	883		Domain(s) with predicted sequence-specific DNA binding, sequence-specific DNA binding transcription factor activity, role in regulation of transcription, DNA-dependent and nucleus localization	prot_ID_334	An02g08260
An02g08270	2	989		Domain(s) with predicted oxidoreductase activity and role in oxidation-reduction process	prot_ID_961	An02g08270

An02g08280	1	2921	Ortholog of A. nidulans FGSC A4 : AN5026, A. fumigatus Af293 : Afu3g12140, A. oryzae RIB40 : AO090003000774, A. niger ATCC 1015 : 37144-mRNA and A. versicolor : Aspve1_0051511	prot_ID_856	An02g08280	
**An02g08290**	0	0	Ortholog(s) have role in pseurotin A biosynthetic process	**prot_ID_442**	**An02g08290**	ECS
An02g08300	-1	758	Protein of unknown function; induced by growth on starch and lactate; expression repressed by tunicamycin and DTT	prot_ID_816	An02g08300	
An02g08310	-2	150	Domain(s) with predicted nucleotide binding, oxidoreductase activity, transferase activity, transferring acyl groups other than amino-acyl groups, zinc ion binding activity and role in oxidation-reduction process	prot_ID_379	An02g08310	ECS
An02g08320	-3	449		prot_ID_194	An02g08320	
An02g08330	-4	616	Protein of unknown function; expression repressed by tunicamycin and DTT	prot_ID_1260	An02g08330	
An02g08340	-5	779	Domain(s) with predicted amino acid transmembrane transporter activity, role in amino acid transmembrane transport and membrane localization	prot_ID_501	An02g08340	
An02g08350	-6	406	Ortholog of A. nidulans FGSC A4 : AN8916, A. niger CBS 513.88 : An16g00980, A. niger ATCC 1015 : 41387-mRNA and A. versicolor : Aspve1_0088608	prot_ID_1366	An02g08350	
An02g08360	-7	390		prot_ID_1252	An02g08360	
An02g08370	-8	616	Domain(s) with predicted oxidoreductase activity and role in oxidation-reduction process	prot_ID_1230	An02g08370	
n/a			Has domain(s) with predicted guanyl-nucleotide exchange factor activity, role in regulation of small GTPase mediated signal transduction, small GTPase mediated signal transduction and intracellular localization	prot_ID_1138	An02g08380	

An02g09430 cluster

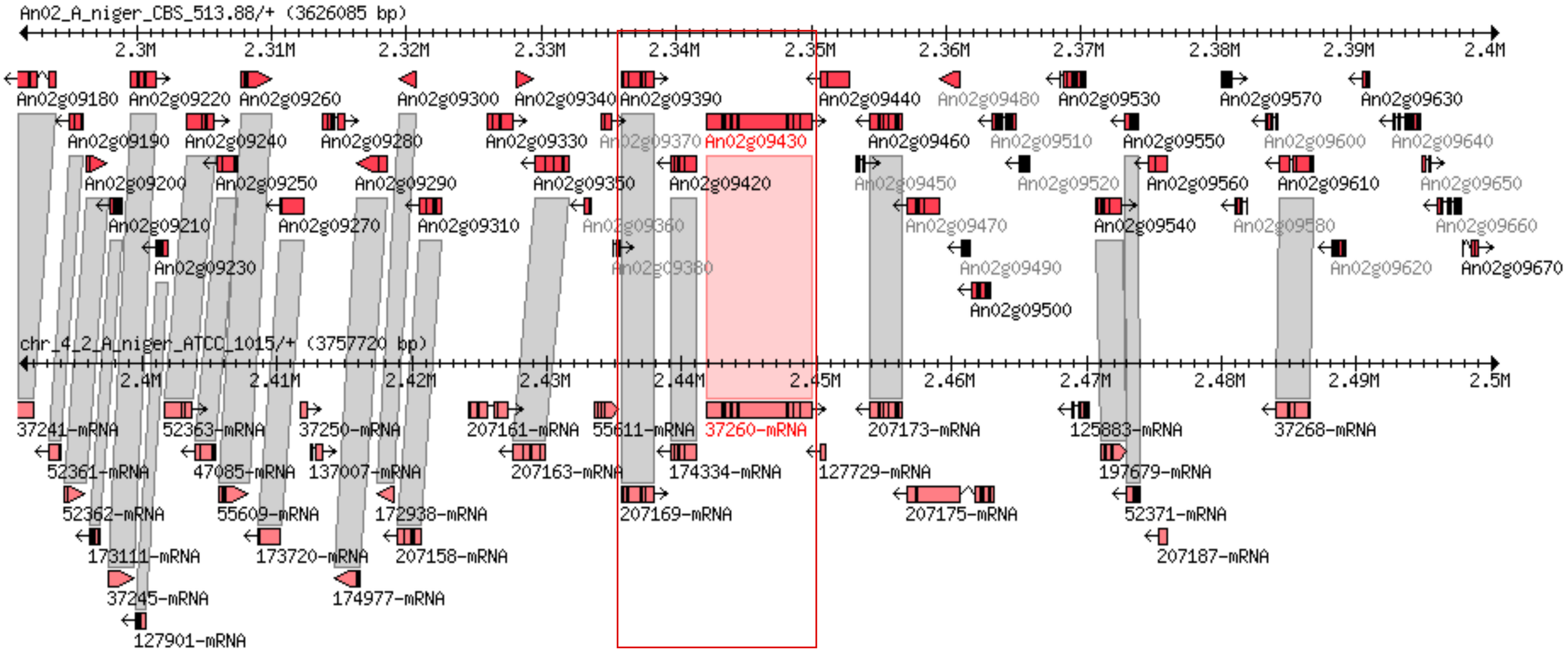

An02g09430 cluster

Gene id (SMURF)	Gene positions	Gene distance	AspGD name	AspGD description line or supplemental annotation	protein ID (antiSMASH)	AspGD ID	
n/a				Has domain(s) with predicted membrane localization	prot_ID_504	An02g09310	
n/a				Ortholog of A. fumigatus Af293 : Afu3g13460, A. oryzae RIB40 : AO090012000779, N. fischeri NRRL 181 : NFIA_063890, Aspergillus flavus NRRL 3357 : AFL2T_03643 and A. clavatus NRRL 1 : ACLA_041690	prot_ID_1095	An02g09330	
n/a				Ortholog of A. nidulans FGSC A4 : AN3158, A. fumigatus Af293 : Afu3g13465, N. fischeri NRRL 181 : NFIA_063880, Aspergillus flavus NRRL 3357 : AFL2T_03645 and A. clavatus NRRL 1 : ACLA_041680	prot_ID_73	An02g09340	
n/a				Has domain(s) with predicted transporter activity, role in transmembrane transport and integral to membrane localization	prot_ID_49	An02g09350	
n/a					prot_ID_76	An02g09360	
n/a				Has domain(s) with predicted translation initiation factor activity, role in translation and eukaryotic translation initiation factor 2 complex localization	prot_ID_374	An02g09370	
n/a					prot_ID_1197	An02g09380	
n/a			yvc1	Calcium activated cation channel with a predicted role in cellular cation homeostasis	prot_ID_1237	An02g09390	ECS
An02g09420	1	785		Domain(s) with predicted electron carrier activity, heme binding, iron ion binding, oxidoreductase activity, acting on paired donors, with incorporation or reduction of molecular oxygen activity and role in oxidation-reduction process	prot_ID_1064	An02g09420	
**An02g09430**	0	0		Domain(s) with predicted ACP phosphopantetheine attachment site binding involved in fatty acid biosynthetic process, nucleotide binding, oxidoreductase activity and transferase activity, more	**prot_ID_507**	**An02g09430**	ECS
An02g09440	-1	631		Ortholog of A. fumigatus Af293 : Afu3g02830, Afu8g02140, A. niger CBS 513.88 : An10g00890, An04g07720, A. oryzae RIB40 : AO090011000172, AO090023000667 and Neosartorya fischeri NRRL 181 : NFIA_001120	prot_ID_646	An02g09440	
An02g09450	-2	541			prot_ID_866	An02g09450	
n/a				Has domain(s) with predicted zinc ion binding activity and intracellular localization	prot_ID_232	An02g09460	
n/a				Potassium- or calcium-transporting ATPase	prot_ID_503	An02g09470	
n/a				Potassium-transporting ATPase	prot_ID_1382	An02g09480	

n/a		prot_ID_542	An02g09490
n/a	Ortholog of A. nidulans FGSC A4 : AN10357, A. fumigatus Af293 : Afu2g15440, Afu3g13810, A. oryzae RIB40 : AO090012000774, AO090005001100 and N. fischeri NRRL 181 : NFIA_063450, NFIA_090680	prot_ID_1244	An02g09500
n/a		prot_ID_1457	An02g09510
n/a		prot_ID_1136	An02g09520
n/a	Protein with similarity to integral membrane protein Pth11 of Magnaporthe grisea	prot_ID_765	An02g09530

An02g10140 cluster

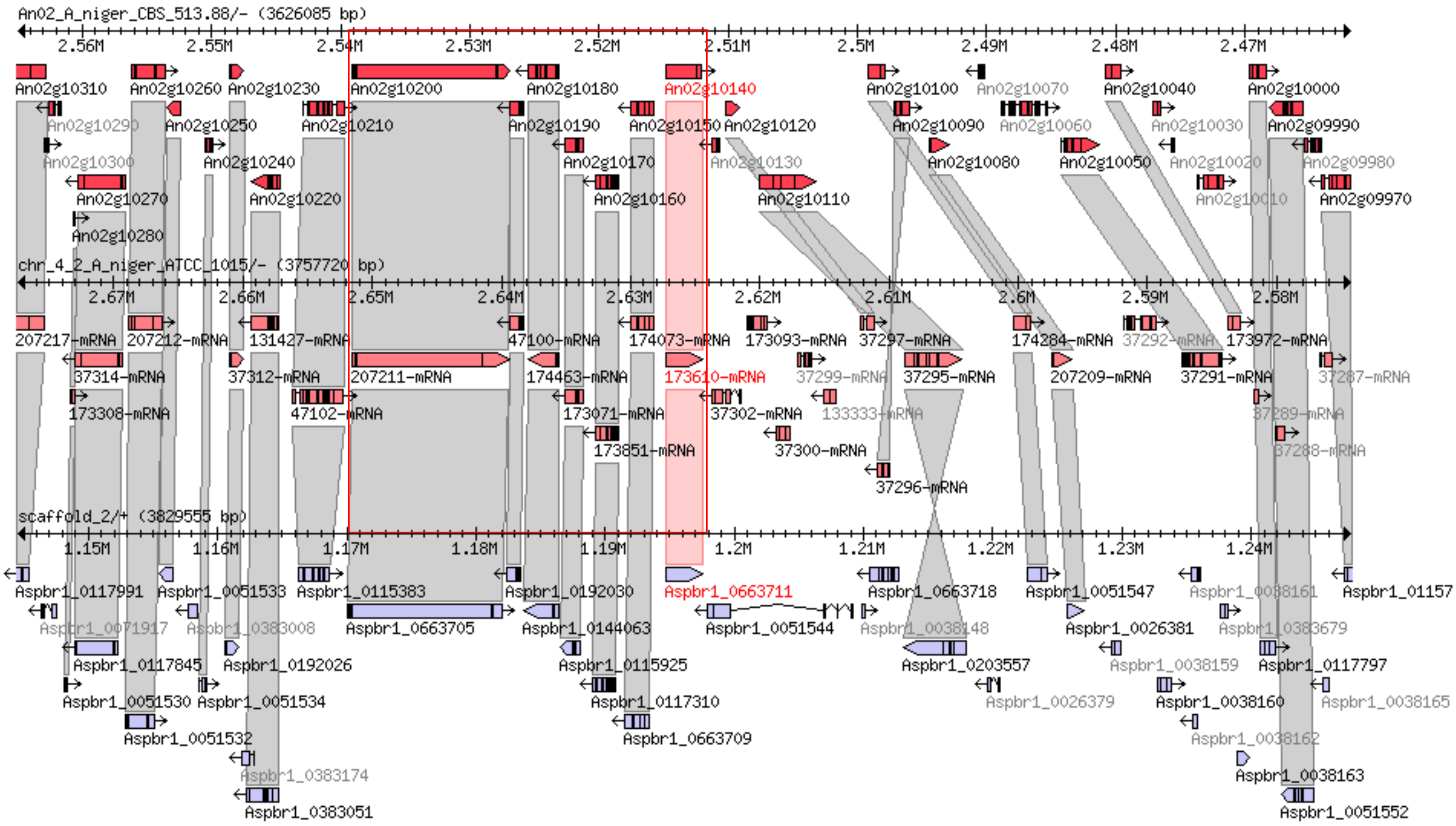

An02g10140 cluster

Gene id (SMURF)	Gene positions	Gene distance	AspGD name	AspGD description line or supplemental annotation	protein ID (antiSMASH)	AspGD ID	
n/a				Has domain(s) with predicted electron carrier activity, heme binding, iron ion binding, oxidoreductase activity, acting on paired donors, with incorporation or reduction of molecular oxygen activity and role in oxidation-reduction process	prot_ID_1469	An02g10080	
n/a				Ortholog of A. niger CBS 513.88 : An08g08360, A. oryzae RIB40 : AO090003001172, A. versicolor : Aspve1_0147312, A. sydowii : Aspsy1_0086600 and Aspergillus terreus NIH2624 : ATET_09649	prot_ID_1360	An02g10090	
n/a				Ortholog of A. nidulans FGSC A4 : AN8003, A. fumigatus Af293 : Afu5g02600, A. niger ATCC 1015 : 174284-mRNA, A. versicolor : Aspve1_0045487 and A. sydowii : Aspsy1_0033725	prot_ID_116	An02g10100	
An02g10110	3	1516		Domain(s) with predicted ATP binding, ATPase activity, coupled to transmembrane movement of substances activity, role in transmembrane transport and integral to membrane localization	prot_ID_351	An02g10110	
An02g10120	2	518		Domain(s) with predicted ATP binding, protein kinase activity and role in protein phosphorylation	prot_ID_38	An02g10120	
An02g10130	1	791		Protein of unknown function	prot_ID_822	An02g10130	
**An02g10140**	0	0		NRPS-like protein similar to nonribosomal peptide synthase	**prot_ID_981**	**An02g10140**	ECS
An02g10150	-1	940		Domain(s) with predicted catalytic activity and role in metabolic process	prot_ID_1405	An02g10150	
An02g10160	-2	841		Domain(s) with predicted pyridoxal phosphate binding, transferase activity, transferring nitrogenous groups activity and role in biosynthetic process	prot_ID_1420	An02g10160	
An02g10170	-3	945		Domain(s) with predicted role in transmembrane transport and integral to membrane localization	prot_ID_582	An02g10170	
n/a				Ortholog of A. nidulans FGSC A4 : AN8002, A. fumigatus Af293 : Afu5g02590, A. oryzae RIB40 : AO090102000374, A. niger ATCC 1015 : 174463-mRNA and A. versicolor : Aspve1_0139493	prot_ID_294	An02g10180	
n/a				Ortholog of A. nidulans FGSC A4 : AN8001, A. oryzae RIB40 : AO090102000373, A. niger ATCC 1015 : 47100-mRNA, A. versicolor : Aspve1_0199673 and A. sydowii : Aspsy1_0059921	prot_ID_964	An02g10190	
n/a				Has domain(s) with predicted phosphotransferase activity, alcohol group as acceptor activity	prot_ID_1353	An02g10200	FA

n/a	Domain(s) with predicted protein phosphatase type 2A regulator activity, role in signal transduction and protein phosphatase type 2A complex localization	n/a	An02g10210

An02g14220 cluster

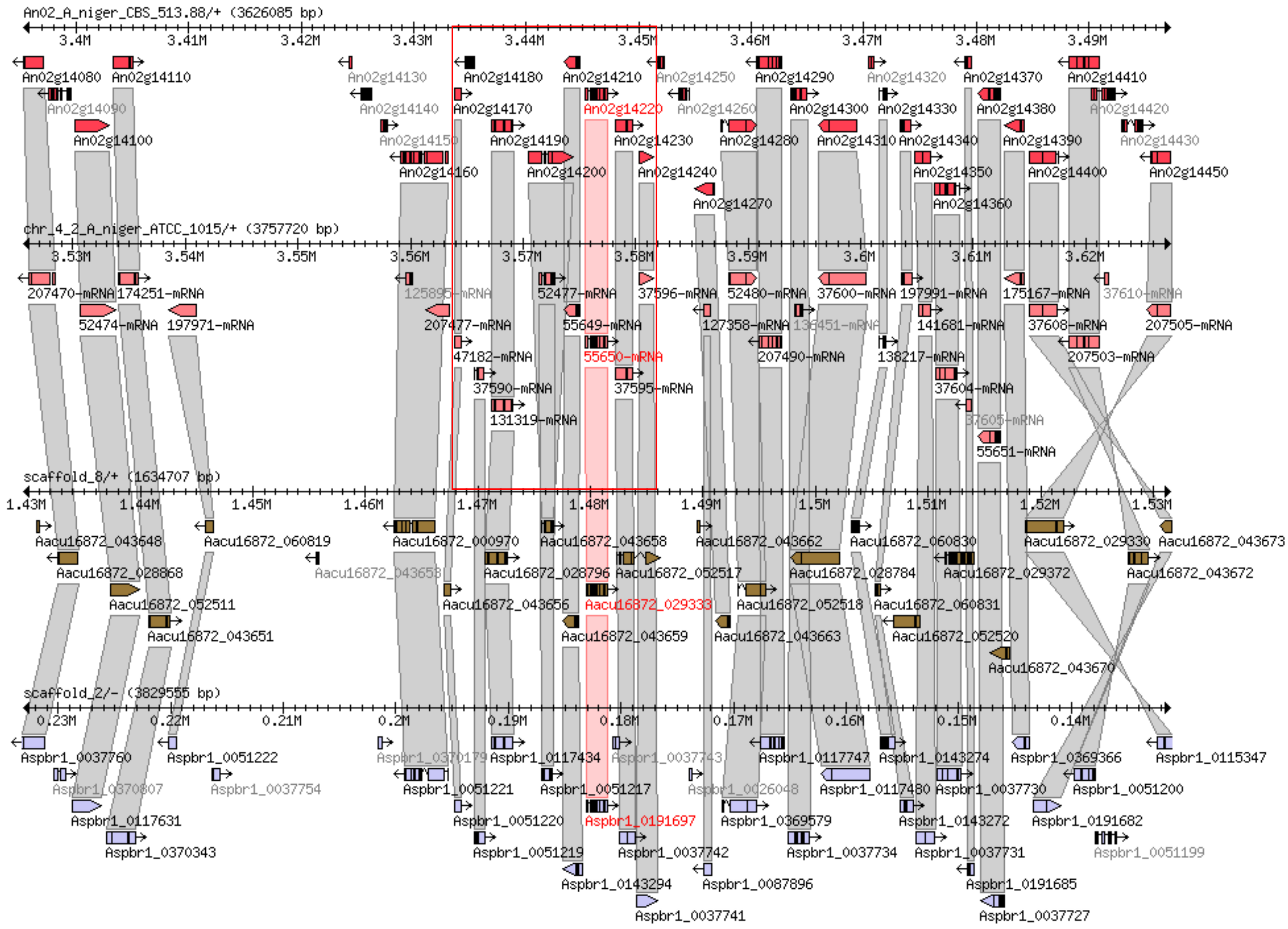

An02g14220 cluster

Gene id (SMURF)	Gene positions	Gene distance	AspGD name	AspGD description line or supplemental annotation	protein ID (antiSMASH)	AspGD ID	
n/a				Ortholog of *A. nidulans FGSC A4* : AN7484, *A. fumigatus Af293* : Afu2g05720, *A. oryzae RIB40* : AO090001000689, *A. niger ATCC 1015* : 47182-mRNA and *Aspergillus versicolor* : Aspve1_0085916	n/a	An02g14170	ECS
n/a				Ortholog of Aspergillus acidus : Aspfo1_0057707	n/a	An02g14180	
An02g14190	3	2426		Domain(s) with predicted role in transmembrane transport and integral to membrane localization	n/a	An02g14190	
An02g14200	2	1044	cftA	Rho GTPase	n/a	An02g14200	
An02g14210	1	575		Domain(s) with predicted hydrolase activity, acting on carbon-nitrogen (but not peptide) bonds, in linear amidines, metal ion binding activity	n/a	An02g14210	
**An02g14220**	0	0		3-oxoacyl-[acyl-carrier protein] synthase	n/a	**An02g14220**	
An02g14230	-1	712		Ortholog of A. nidulans FGSC A4 : AN11747, A. fumigatus Af293 : Afu2g05770, A. brasiliensis : Aspbr1_0037742, A. niger ATCC 1015 : 37595-mRNA and N. fischeri NRRL 181 : NFIA_082490	n/a	An02g14230	
An02g14240	-2	669		Domain(s) with predicted mRNA guanylyltransferase activity, role in 7-methylguanosine mRNA capping and mRNA cap methyltransferase complex localization	n/a	An02g14240	ECS

An03g00650 cluster

No ATCC 1015 matching cluster; no manual prediction made

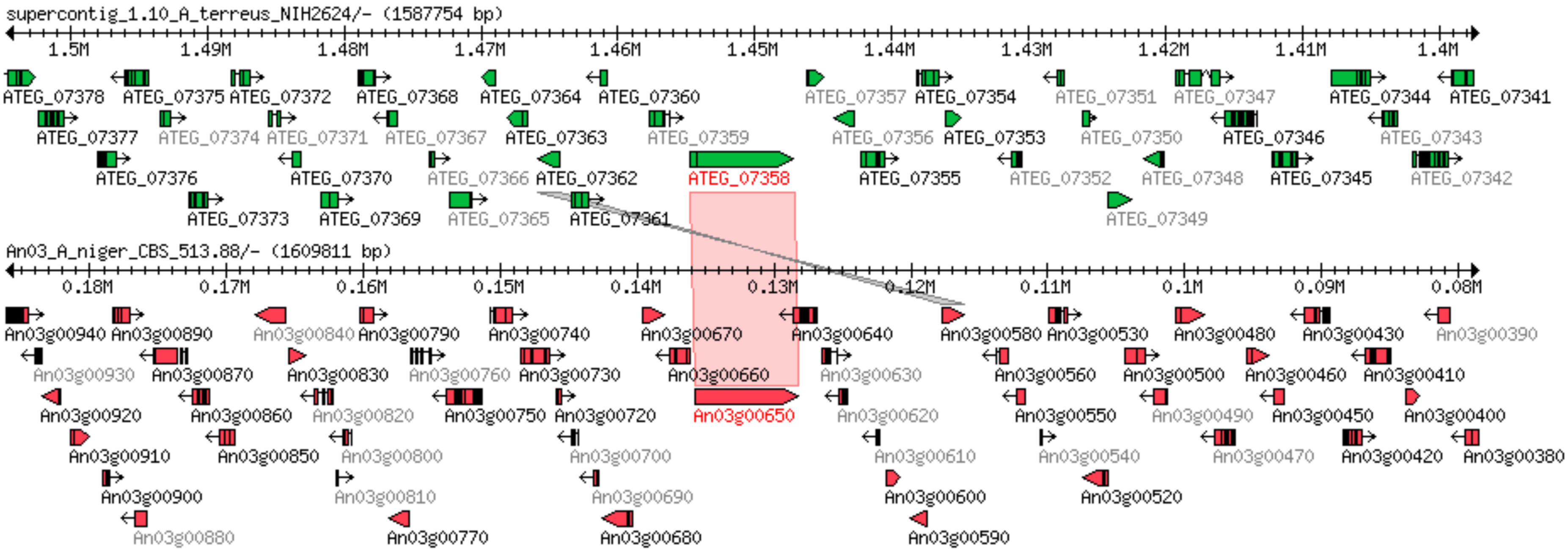

An03g00650 cluster

Gene id (SMURF)	Gene positions	Gene distance	AspGD name	AspGD description line or supplemental annotation	protein ID (antiSMASH)	AspGD ID
n/a				Salicylate hydroxylase with a predicted role in salicylic acid degradation	prot_ID_86	An03g00530
n/a					prot_ID_297	An03g00540
n/a				Ortholog of A. brasiliensis : Aspbr1_0059969 and A. acidus : Aspfo1_0205058	prot_ID_473	An03g00550
n/a				Ortholog of A. nidulans FGSC A4 : AN2026, A. fumigatus Af293 : Afu2g00850, A. oryzae RIB40 : AO090012000015 and A. brasiliensis : Aspbr1_0049054, Aspbr1_0138565	prot_ID_365	An03g00560
An03g00580	7	1158		Domain(s) with predicted electron carrier activity, heme binding, iron ion binding, oxidoreductase activity, acting on paired donors, with incorporation or reduction of molecular oxygen activity and role in oxidation-reduction process	prot_ID_45	An03g00580
An03g00590	6	787		Domain(s) with predicted trichodiene synthase activity and role in sesquiterpenoid biosynthetic process	prot_ID_341	An03g00590
An03g00600	5	473		Domain(s) with predicted ATP binding, protein kinase activity and role in protein phosphorylation	prot_ID_274	An03g00600
An03g00610	4	2045			prot_ID_635	An03g00610
An03g00620	3	60			prot_ID_618	An03g00620
An03g00630	2	378			prot_ID_175	An03g00630
An03g00640	1	256		Ortholog of A. fumigatus Af293 : Afu2g17790, A. brasiliensis : Aspbr1_0032979, N. fischeri NRRL 181 : NFIA_093190, A. acidus : Aspfo1_0058754 and A. fumigatus A1163 : AFUB_033460	prot_ID_434	An03g00640
**An03g00650**	0	0		Putative nonribosomal peptide synthase (NRPS)	**prot_ID_648**	**An03g00650**
An03g00660	-1	494	tauD	Taurine dioxygenase	prot_ID_429	An03g00660
An03g00670	-2	918		Domain(s) with predicted NAD binding, oxidoreductase activity, acting on the CH-OH group of donors, NAD or NADP as acceptor activity and role in glycerol-3-phosphate catabolic process, oxidation-reduction process	prot_ID_171	An03g00670
An03g00680	-3	823		Domain(s) with predicted role in transmembrane transport and integral to membrane localization	prot_ID_153	An03g00680
An03g00690	-4	609			prot_ID_35	An03g00690
An03g00700	-5	1238			prot_ID_352	An03g00700
An03g00720	-6	837		Protein of unknown function; expression induced by tunicamycin and DTT	prot_ID_321	An03g00720
An03g00730	-7	466		Amine oxidase	prot_ID_144	An03g00730

An03g00740	-8	420	Domain(s) with predicted mannan endo-1,6-alpha-mannosidase activity and role in carbohydrate catabolic process	prot_ID_233	An03g00740
An03g00750	-9	393	Domain(s) with predicted DNA binding, sequence-specific DNA binding RNA polymerase II transcription factor activity, sequence-specific DNA binding transcription factor activity, zinc ion binding activity	prot_ID_315	An03g00750
An03g00760	-10	1156		prot_ID_471	An03g00760
An03g00770	-11	329	Protein similar to allergic allergen rAsp f 4 of A. fumigatus; expression repressed by tunicamycin and DTT	n/a	An03g00770
An03g00790	-12	1669	Domain(s) with predicted RNA binding, ribonuclease III activity and role in RNA processing	n/a	An03g00790

An03g01820 cluster

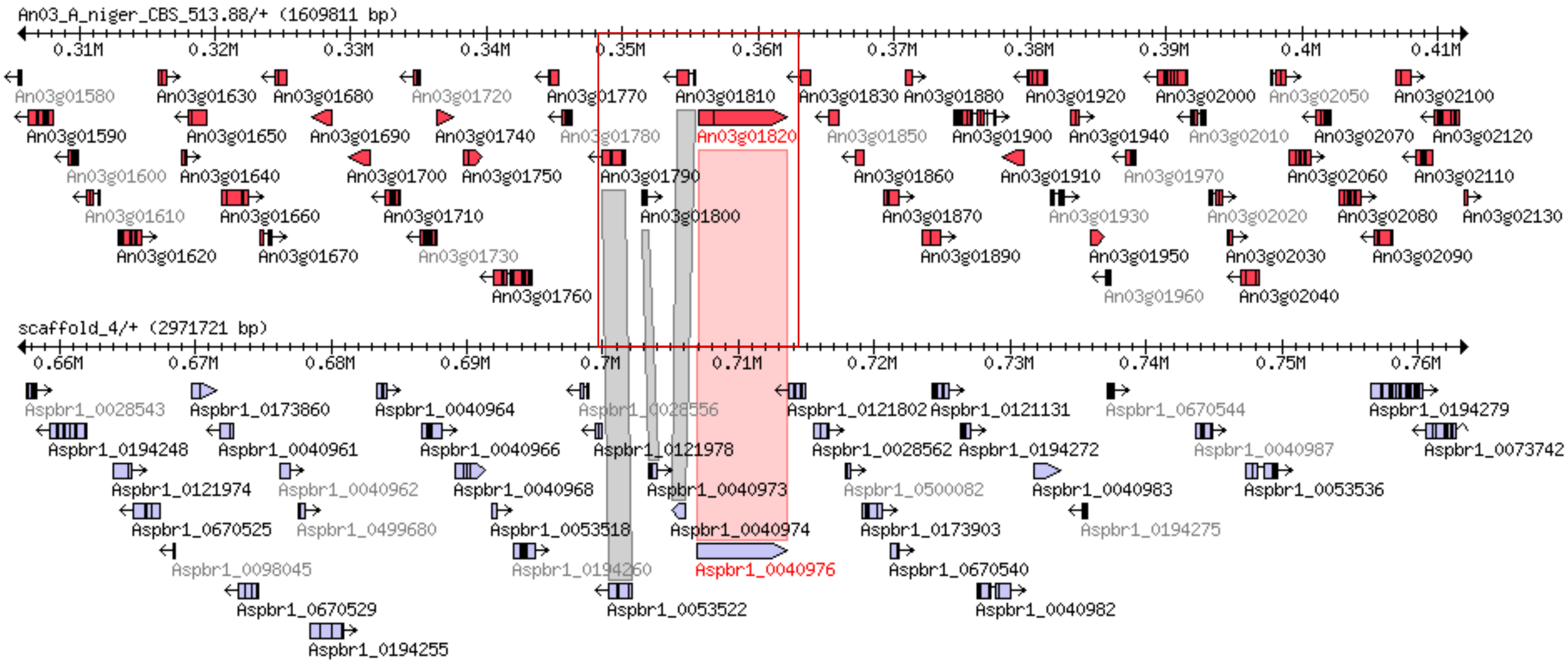

An03g01820 cluster

Gene id (SMURF)	Gene positions	Gene distance	AspGD name	AspGD description line or supplemental annotation	protein ID (antiSMASH)	AspGD ID	
n/a					prot_ID_150	An03g01730	
n/a				Ortholog of A. nidulans FGSC A4 : AN2836, A. niger CBS 513.88 : An16g00290, A. brasiliensis : Aspbr1_0043644, Aspbr1_0049046, Aspbr1_0132878 and A. niger ATCC 1015 : 141246-mRNA, 45726-mRNA	prot_ID_169	An03g01740	
An03g01750	7	677		Domain(s) with predicted substrate-specific transmembrane transporter activity, role in transmembrane transport and integral to membrane localization	prot_ID_370	An03g01750	
An03g01760	6	1264		Domain(s) with predicted DNA binding, sequence-specific DNA binding RNA polymerase II transcription factor activity, sequence-specific DNA binding transcription factor activity, zinc ion binding activity	prot_ID_474	An03g01760	
An03g01770	5	346		Ortholog of A. nidulans FGSC A4 : AN7941, A. fumigatus Af293 : Afu2g12680, Afu7g00370, A. niger ATCC 1015 : 214657-mRNA, A. versicolor : Aspve1_0045447 and A. sydowii : Aspsy1_0156290	prot_ID_257	An03g01770	
An03g01780	4	2225			prot_ID_174	An03g01780	
An03g01790	3	1239		Domain(s) with predicted role in transmembrane transport and integral to membrane localization	prot_ID_219	An03g01790	ECS
An03g01800	2	2120		Ortholog(s) have long-chain fatty acid transporter activity, role in chronological cell aging, fatty acid metabolic process, long-chain fatty acid transport and cytoplasm, extracellular region, nucleus localization	prot_ID_572	An03g01800	
An03g01810	1	325		Ortholog(s) have role in asperfuranone biosynthetic process, secondary metabolic process	prot_ID_573	An03g01810	
**An03g01820**	0	0		Putative melanin polyketide synthase	**prot_ID_420**	**An03g01820**	ECS
An03g01830	-1	968		Ortholog of A. niger CBS 513.88 : An12g05420, An01g01650, A. oryzae RIB40 : AO090701000546, AO090001000314, A. niger ATCC 1015 : 45719-mRNA, 53991-mRNA and A. versicolor : Aspve1_0041648	prot_ID_661	An03g01830	
An03g01850	-2	1265			prot_ID_428	An03g01850	
n/a				Has domain(s) with predicted catalytic activity and role in metabolic process	prot_ID_633	An03g01860	
n/a				Ortholog of A. fumigatus Af293 : Afu5g08440, N. fischeri NRRL 181 : NFIA_078840, A. acidus : Aspfo1_0084088, A. fumigatus A1163 : AFUB_055980 and A. niger ATCC 1015 : 194089-mRNA	prot_ID_318	An03g01870	

n/a	Has domain(s) with predicted acid-amino acid ligase activity and role in post-translational protein modification	prot_ID_408	An03g01880
n/a	Ortholog of A. nidulans FGSC A4 : AN2041, A. niger CBS 513.88 : An16g01460, An16g06600, An11g04970, A. oryzae RIB40 : AO090026000564 and A. niger ATCC 1015 : 39078-mRNA, 41348-mRNA	prot_ID_659	An03g01890
n/a	Ortholog of A. niger ATCC 1015 : 45714-mRNA	prot_ID_368	An03g01900
n/a	Acetylglutamate kinase	prot_ID_65	An03g01910
n/a	Ortholog of A. nidulans FGSC A4 : AN10915, A. niger CBS 513.88 : An04g09630, A. niger ATCC 1015 : 195107-mRNA, 45712-mRNA and A. versicolor : Aspve1_0513563	prot_ID_143	An03g01920
n/a		prot_ID_423	An03g01930

An03g03520 cluster

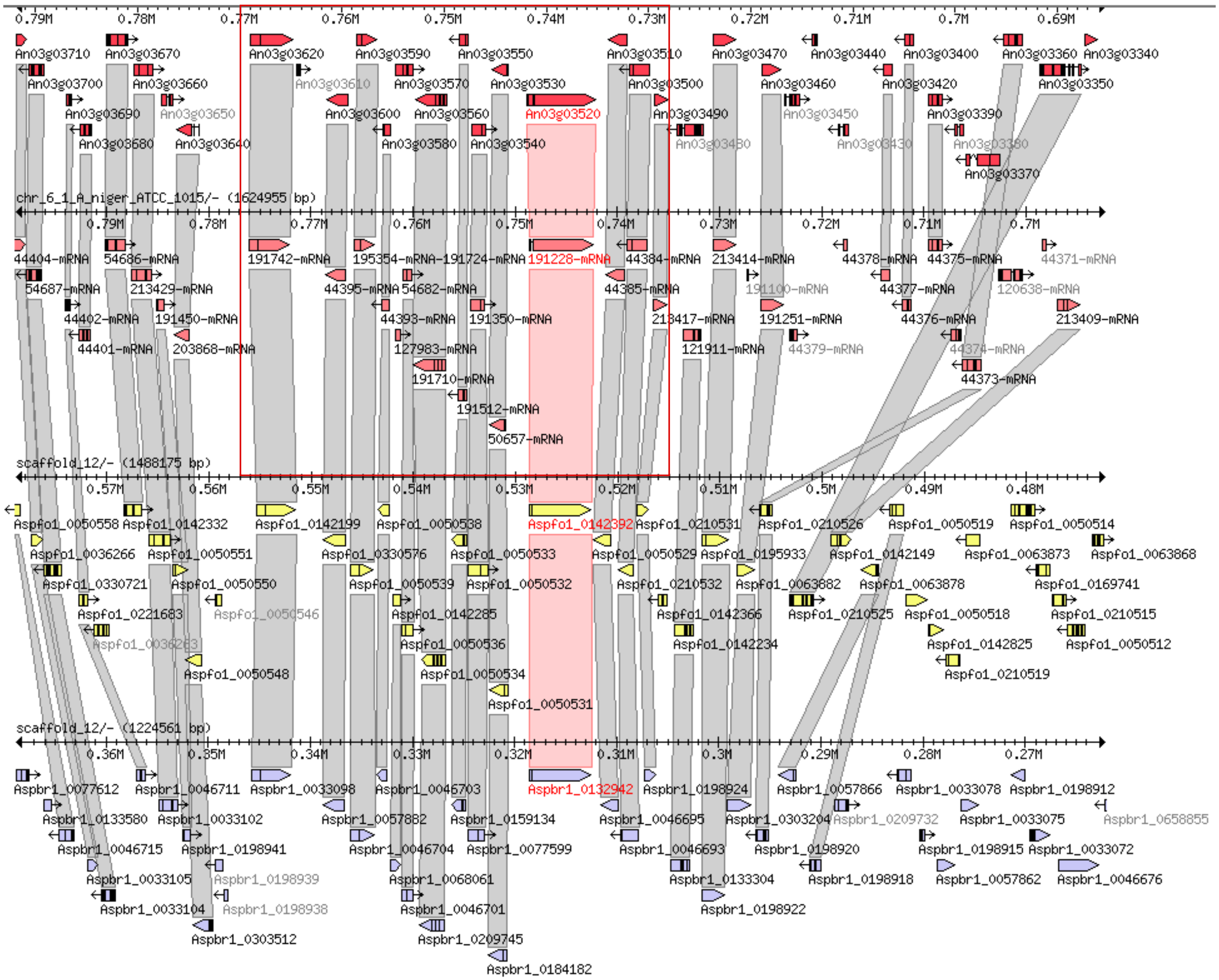

An03g03520 cluster

Gene id (SMURF)	Gene positions	Gene distance	AspGD name	AspGD description line or supplemental annotation	protein ID (antiSMASH)	AspGD ID	
An03g03380	13	1189			n/a	An03g03380	
An03g03390	12	1450		Ortholog of A. niger CBS 513.88 : An09g01480, A. brasiliensis : Aspbr1_0131175, A. niger ATCC 1015 : 43480-mRNA, 44375-mRNA and Aspergillus carbonarius ITEM 5010 : Acar5010_135976	n/a	An03g03390	
An03g03400	11	1224		Ortholog of A. nidulans FGSC A4 : AN8462, A. niger CBS 513.88 : An09g01460, A. brasiliensis : Aspbr1_0131393, A. niger ATCC 1015 : 43481-mRNA, 44376-mRNA and A. acidus : Aspfo1_0194567	n/a	An03g03400	
An03g03420	10	3372		Domain(s) with predicted hydrolase activity	n/a	An03g03420	
An03g03430	9	2152			n/a	An03g03430	
An03g03440	8	1221		Ortholog of A. nidulans FGSC A4 : AN11871, A. fumigatus Af293 : Afu1g11260, Afu6g00270, A. niger CBS 513.88 : An04g04460 and A. niger ATCC 1015 : 190306-mRNA	n/a	An03g03440	
An03g03450	7	355		Domain(s) with predicted nucleotide binding, oxidoreductase activity, zinc ion binding activity and role in oxidation-reduction process	prot_ID_333	An03g03450	
An03g03460	6	2467		Domain(s) with predicted D-arabinono-1,4-lactone oxidase activity, UDP-N-acetylmuramate dehydrogenase activity, flavin adenine dinucleotide binding activity and role in biosynthetic process, oxidation-reduction process	prot_ID_226	An03g03460	
An03g03470	5	1061		Domain(s) with predicted ATP binding, ATPase activity	prot_ID_24	An03g03470	
An03g03480	4	793		Domain(s) with predicted NAD binding, oxidoreductase activity, acting on the CH-OH group of donors, NAD or NADP as acceptor activity and role in oxidation-reduction process	prot_ID_116	An03g03480	
An03g03490	3	649		Ortholog of A. nidulans FGSC A4 : AN2888, A. fumigatus Af293 : Afu3g11580, A. oryzae RIB40 : AO090003000717, A. niger ATCC 1015 : 213417-mRNA and A. versicolor : Aspve1_0556144	prot_ID_499	An03g03490	ECS
An03g03500	2	932		Ortholog of A. nidulans FGSC A4 : AN4162, A. fumigatus Af293 : Afu4g13160, A. oryzae RIB40 : AO090009000265, A. niger ATCC 1015 : 44384-mRNA and A. versicolor : Aspve1_0086858	prot_ID_574	An03g03500	
An03g03510	1	1751		Ortholog of A. nidulans FGSC A4 : AN6634, A. fumigatus Af293 : Afu6g03780, A. oryzae RIB40 : AO090701000171, A. niger ATCC 1015 : 44385-mRNA and A. versicolor : Aspve1_0044304	prot_ID_187	An03g03510	
**An03g03520**	0	0		Ortholog(s) have role in ergosterol biosynthetic process	**prot_ID_302**	**An03g03520**	

An03g03530	-1	1908	Ortholog of A. nidulans FGSC A4 : AN6239, AN6793, A. fumigatus Af293 : Afu3g03390, Afu7g00330, A. oryzae RIB40 : AO090103000170, AO090701000114 and A. niger ATCC 1015 : 50657-mRNA	prot_ID_136	An03g03530	
An03g03540	-2	844	Ortholog(s) have role in ergosterol biosynthetic process, secondary metabolic process, siderophore biosynthetic process	prot_ID_75	An03g03540	
An03g03550	-3	373	Ortholog(s) have role in N',N'',N'''-triacetylfusarinine C biosynthetic process, cellular response to hydrogen peroxide, cellular response to iron ion starvation, ergosterol biosynthetic process, pathogenesis, secondary metabolic process	prot_ID_113	An03g03550	
An03g03560	-4	1262	Ortholog(s) have role in ferric triacetylfusarinine C transport	prot_ID_340	An03g03560	
An03g03570	-5	1229	Domain(s) with predicted N-methyltransferase activity, protein C-terminal S-isoprenylcysteine carboxyl O-methyltransferase activity and role in C-terminal protein methylation, phospholipid metabolic process	prot_ID_299	An03g03570	
An03g03580	-6	489	Ortholog of A. nidulans FGSC A4 : AN7328, A. fumigatus Af293 : Afu2g16550, A. oryzae RIB40 : AO090102000183, A. niger ATCC 1015 : 44393-mRNA and A. versicolor : Aspve1_0050747	prot_ID_488	An03g03580	
An03g03590	-7	886	Domain(s) with predicted N-acetyltransferase activity and role in metabolic process	prot_ID_602	An03g03590	
An03g03600	-8	886	Domain(s) with predicted ATP binding, DNA binding, helicase activity	prot_ID_581	An03g03600	
An03g03610	-9	2579	Protein of unknown function	n/a	An03g03610	
An03g03620	-10	539	Domain(s) with predicted ATP binding, ATPase activity, coupled to transmembrane movement of substances activity, role in transmembrane transport and integral to membrane localization	n/a	An03g03620	ECS, IGS

An03g05140 cluster

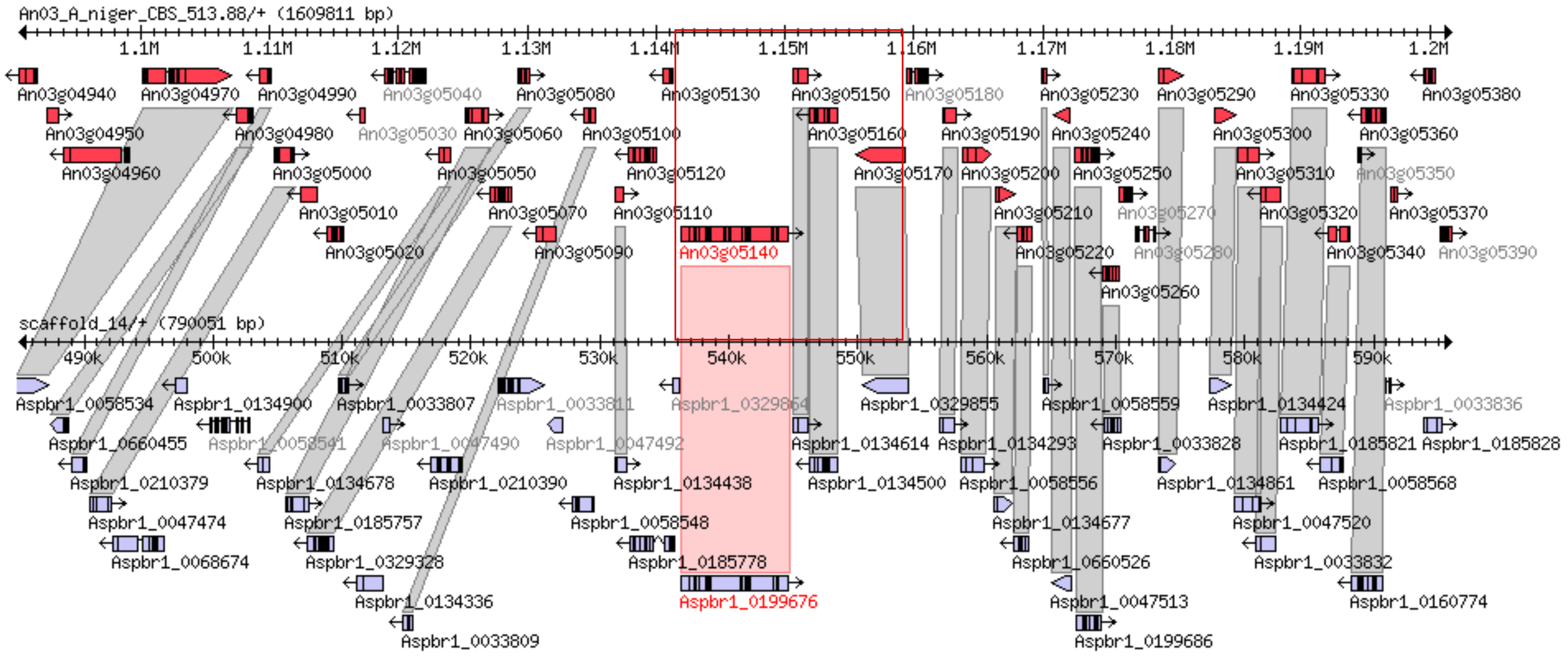

An03g05140 cluster

Gene id (SMURF)	Gene positions	Gene distance	AspGD name	AspGD description line or supplemental annotation	protein ID (antiSMASH)	AspGD ID	
n/a					prot_ID_105	An03g05040	
An03g05050	9	1245		Domain(s) with predicted adenosylhomocysteinase activity, nucleotide binding, oxidoreductase activity and role in oxidation-reduction process	prot_ID_639	An03g05050	
An03g05060	8	153		Domain(s) with predicted amino acid transmembrane transporter activity, role in amino acid transmembrane transport and integral to membrane localization	prot_ID_339	An03g05060	
An03g05070	7	444		Domain(s) with predicted hydrolase activity, acting on carbon-nitrogen (but not peptide) bonds, in linear amidines, metal ion binding activity	prot_ID_663	An03g05070	
An03g05080	6	582		Ortholog of A. nidulans FGSC A4 : AN7668, A. fumigatus Af293 : Afu2g01360, A. oryzae RIB40 : AO090701000421, A. niger ATCC 1015 : 50723-mRNA and A. versicolor : Aspve1_0172224	prot_ID_418	An03g05080	
An03g05090	5	2099		NAD kinase	prot_ID_69	An03g05090	
An03g05100	4	1522		Ortholog(s) have role in exonucleolytic trimming to generate mature 3'-end of 5.8S rRNA from tricistronic rRNA transcript (SSU-rRNA, 5.8S rRNA and LSU-rRNA), more	prot_ID_266	An03g05100	
An03g05110	3	278		Ortholog of A. nidulans FGSC A4 : AN10993, A. fumigatus Af293 : Afu2g01310, A. oryzae RIB40 : AO090701000405, A. niger ATCC 1015 : 50726-mRNA and A. versicolor : Aspve1_0043704	prot_ID_490	An03g05110	
An03g05120	2	466		Domain(s) with predicted role in transmembrane transport and integral to membrane localization	prot_ID_582	An03g05120	
An03g05130	1	583		Ortholog of A. fumigatus Af293 : Afu2g01300, A. versicolor : Aspve1_0036298, A. sydowii : Aspsy1_0086510 and A. niger ATCC 1015 : 126782-mRNA, 126826-mRNA	prot_ID_160	An03g05130	
**An03g05140**	0	0		Putative polyketide synthase	**prot_ID_506**	**An03g05140**	ECS
An03g05150	-1	269		D-mandelate dehydrogenase with a predicted role in mandelate degradation	prot_ID_285	An03g05150	
An03g05160	-2	150		Domain(s) with predicted electron carrier activity, flavin adenine dinucleotide binding, iron ion binding, oxidoreductase activity, role in oxidation-reduction process and integral to membrane localization	prot_ID_619	An03g05160	
An03g05170	-3	2275		Ortholog(s) have sterol response element binding, transcription regulatory region sequence-specific DNA binding activity	prot_ID_657	An03g05170	ECS
An03g05180	-4	184		Protein of unknown function	prot_ID_190	An03g05180	

An03g05190	-5	1003		D-sorbitol dehydrogenase	prot_ID_229	An03g05190
An03g05200	-6	497	protF	Carboxypeptidase Y family secreted protease; expression repressed by tunicamycin and DTT	prot_ID_349	An03g05200
An03g05210	-7	691		Domain(s) with predicted UDP-N-acetylmuramate dehydrogenase activity, flavin adenine dinucleotide binding activity and role in oxidation-reduction process	prot_ID_239	An03g05210
An03g05220	-8	104		Ortholog of A. brasiliensis : Aspbr1_0660526, A. niger ATCC 1015 : 44513-mRNA, A. acidus : Aspfo1_0210672 and Aspergillus carbonarius ITEM 5010 : Acar5010_399425	prot_ID_6	An03g05220
An03g05230	-9	859		Ortholog of A. brasiliensis : Aspbr1_0058559, A. acidus : Aspfo1_0142682 and Aspergillus aculeatus ATCC16872 : Aacu16872_042334	prot_ID_235	An03g05230
An03g05240	-10	487		Domain(s) with predicted nucleotide binding, oxidoreductase activity and role in oxidation-reduction process	n/a	An03g05240
An03g05250	-11	419		Domain(s) with predicted role in transmembrane transport and integral to membrane localization	n/a	An03g05250
An03g05260	-12	122		Ortholog(s) have chitosan binding activity	n/a	An03g05260
An03g05270	-13	23			n/a	An03g05270

An03g05440 cluster

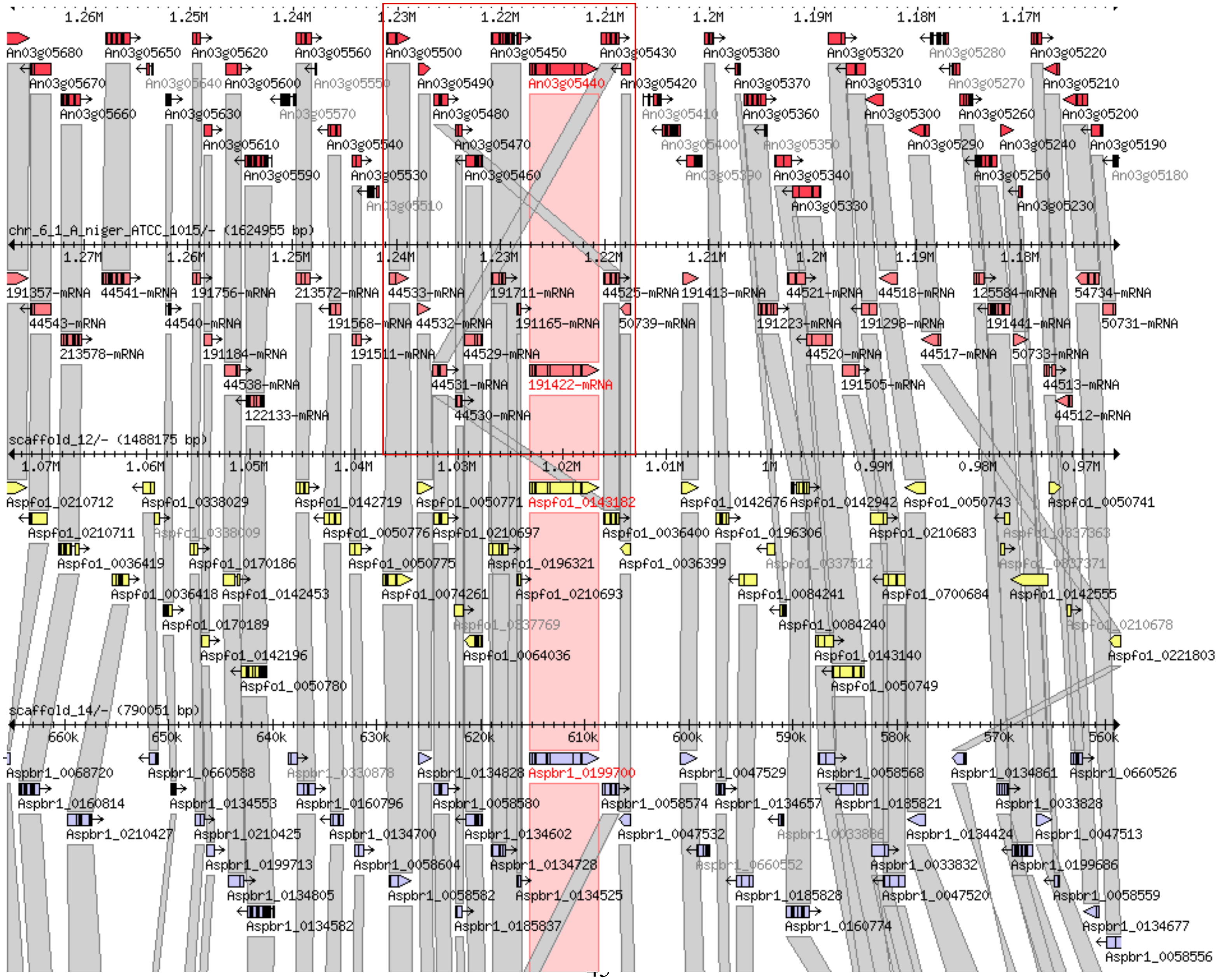

An03g05440 cluster

Gene id (SMURF)	Gene positions	Gene distance	AspGD name	AspGD description line or supplemental annotation	protein ID (antiSMASH)	AspGD ID	
An03g05300	14	88		Tannin acyl hydrolase with a predicted role in tannic acid degradation	n/a	An03g05300	
An03g05310	13	115		Salicylate hydroxylase with a predicted role in salicylic acid degradation	n/a	An03g05310	
An03g05320	12	758		Domain(s) with predicted substrate-specific transmembrane transporter activity, role in transmembrane transport and integral to membrane localization	n/a	An03g05320	
An03g05330	11	40		Putative beta-glucosidase	prot_ID_68	An03g05330	
An03g05340	10	647		Domain(s) with predicted DNA binding, sequence-specific DNA binding RNA polymerase II transcription factor activity, sequence-specific DNA binding transcription factor activity, zinc ion binding activity	prot_ID_389	An03g05340	
An03g05350	9	140		Protein of unknown function	prot_ID_135	An03g05350	
An03g05360	8	443		Ortholog of A. nidulans FGSC A4 : AN2821, A. fumigatus Af293 : Afu2g00180, Afu2g17480, A. niger CBS 513.88 : An04g09420 and A. oryzae RIB40 : AO090102000063, AO090009000716	prot_ID_353	An03g05360	
An03g05370	7	1877		Ortholog of A. acidus : Aspfo1_0084240	prot_ID_590	An03g05370	
An03g05380	6	322		Putative endo-glucanase	prot_ID_173	An03g05380	
An03g05390	5	598		Protein of unknown function	prot_ID_139	An03g05390	
An03g05400	4	24		Protein of unknown function	prot_ID_558	An03g05400	
An03g05410	3	1168		Protein of unknown function	prot_ID_615	An03g05410	
An03g05420	2	256	hNmrr	Protein with a predicted role in nitrogen utilization	prot_ID_595	An03g05420	
An03g05430	1	183		Domain(s) with predicted O-methyltransferase activity	prot_ID_224	An03g05430	ECS
**An03g05440**	0	0		Domain(s) with predicted ACP phosphopantetheine attachment site binding involved in fatty acid biosynthetic process, hydrolase activity, acting on ester bonds, phosphopantetheine binding, transferase activity	**prot_ID_338**	**An03g05440**	
An03g05450	-1	751		Domain(s) with predicted monooxygenase activity and role in oxidation-reduction process	prot_ID_192	An03g05450	
An03g05460	-2	844		Ortholog(s) have catalytic activity and role in sterigmatocystin biosynthetic process	prot_ID_106	An03g05460	
An03g05470	-3	277		Ortholog of A. brasiliensis : Aspbr1_0185837, A. niger ATCC 1015 : 44530-mRNA and Aspergillus carbonarius ITEM 5010 : Acar5010_055781	prot_ID_67	An03g05470	
An03g05480	-4	688		Putative O-methyltransferase	prot_ID_63	An03g05480	

An03g05490	-5	305	Maleylacetate reductase	prot_ID_600	An03g05490	
An03g05500	-6	834	Domain(s) with predicted sequence-specific DNA binding RNA polymerase II transcription factor activity, sequence-specific DNA binding transcription factor activity, zinc ion binding activity	prot_ID_236	An03g05500	ECS
n/a				prot_ID_449	An03g05510	
n/a				prot_ID_381	An03g05530	
n/a				prot_ID_15	An03g05540	

An03g05680 cluster

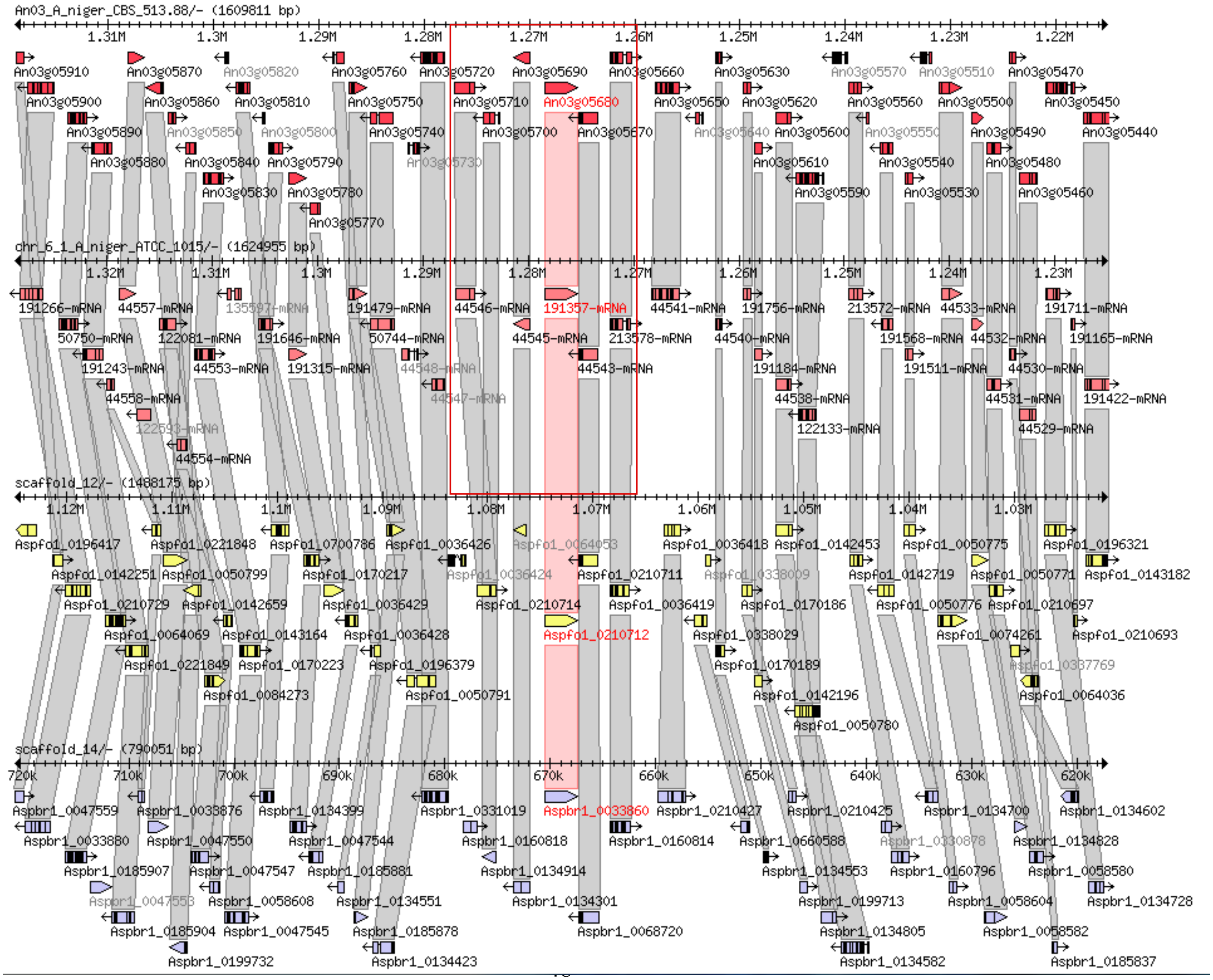

An03g05680 cluster

Gene id (SMURF)	Gene positions	Gene distance	AspGD name	AspGD description line or supplemental annotation	protein ID (antiSMASH)	AspGD ID	
n/a				Ortholog of A. brasiliensis : Aspbr1_0134805, A. acidus : Aspfo1_0142453 and A. niger ATCC 1015 : 44538-mRNA	prot_ID_99	An03g05600	
n/a				Ortholog(s) have intracellular localization	prot_ID_151	An03g05610	
n/a				Ortholog of A. nidulans FGSC A4 : AN2942, AN10320, AN7130, A. fumigatus Af293 : Afu3g15110 and A. niger CBS 513.88 : An15g07840, An12g07260, An03g02580, An01g14840	prot_ID_31	An03g05620	
n/a				Ortholog of A. brasiliensis : Aspbr1_0134553, A. acidus : Aspfo1_0170189, A. niger ATCC 1015 : 44540-mRNA and Aspergillus carbonarius ITEM 5010 : Acar5010_210478	prot_ID_427	An03g05630	
n/a					prot_ID_251	An03g05640	
n/a				Ortholog of A. niger ATCC 1015 : 44541-mRNA	prot_ID_81	An03g05650	
An03g05660	2	1023		Putative catalase	prot_ID_369	An03g05660	ECS
An03g05670	1	215		Ortholog(s) have role in oxidation-reduction process	prot_ID_186	An03g05670	
**An03g05680**	0	0		Protein similar to nonribosomal peptide synthases (NRPS-like)	**prot_ID_385**	**An03g05680**	
An03g05690	-1	1418		Ortholog of A. brasiliensis : Aspbr1_0134301 and A. niger ATCC 1015 : 44545-mRNA	prot_ID_100	An03g05690	
An03g05700	-2	1309		Ortholog of A. brasiliensis : Aspbr1_0134914	prot_ID_658	An03g05700	
An03g05710	-3	660		Ortholog of A. niger ATCC 1015 : 44546-mRNA and A. acidus : Aspfo1_0210714	prot_ID_591	An03g05710	ECS
An03g05720	-4	984		Ortholog of A. nidulans FGSC A4 : AN3519, AN2646, AN0587, AN0866, AN7370 and A. fumigatus Af293 : Afu1g15200, Afu3g03790, Afu4g14040, Afu5g06900, Afu7g08575	prot_ID_629	An03g05720	
An03g05730	-5	155			prot_ID_197	An03g05730	
An03g05740	-6	1441		Ortholog of A. nidulans FGSC A4 : AN8444/celA, A. fumigatus Af293 : Afu3g03620, Afu8g00680, A. oryzae RIB40 : AO090138000033, AO090701000186 and A. niger ATCC 1015 : 50744-mRNA	prot_ID_386	An03g05740	
An03g05750	-7	442		Domain(s) with predicted role in transmembrane transport and integral to membrane localization	prot_ID_441	An03g05750	
An03g05760	-8	554		Domain(s) with predicted protein C-terminal S-isoprenylcysteine carboxyl O-methyltransferase activity, role in C-terminal protein methylation and integral to membrane localization	prot_ID_12	An03g05760	

An03g05770	-9	1189	Domain(s) with predicted transporter activity, role in transport and membrane localization	n/a	An03g05770
An03g05780	-10	288	Ortholog of A. oryzae RIB40 : AO090001000161, AO090020000170, Aspergillus niger ATCC 1015 : 54380-mRNA, A. sydowii : Aspsy1_0214510 and Aspergillus terreus NIH2624 : ATET_02539	n/a	An03g05780
An03g05790	-11	558	Domain(s) with predicted O-methyltransferase activity	n/a	An03g05790
An03g05800	-12	335		n/a	An03g05800

An03g06010 cluster

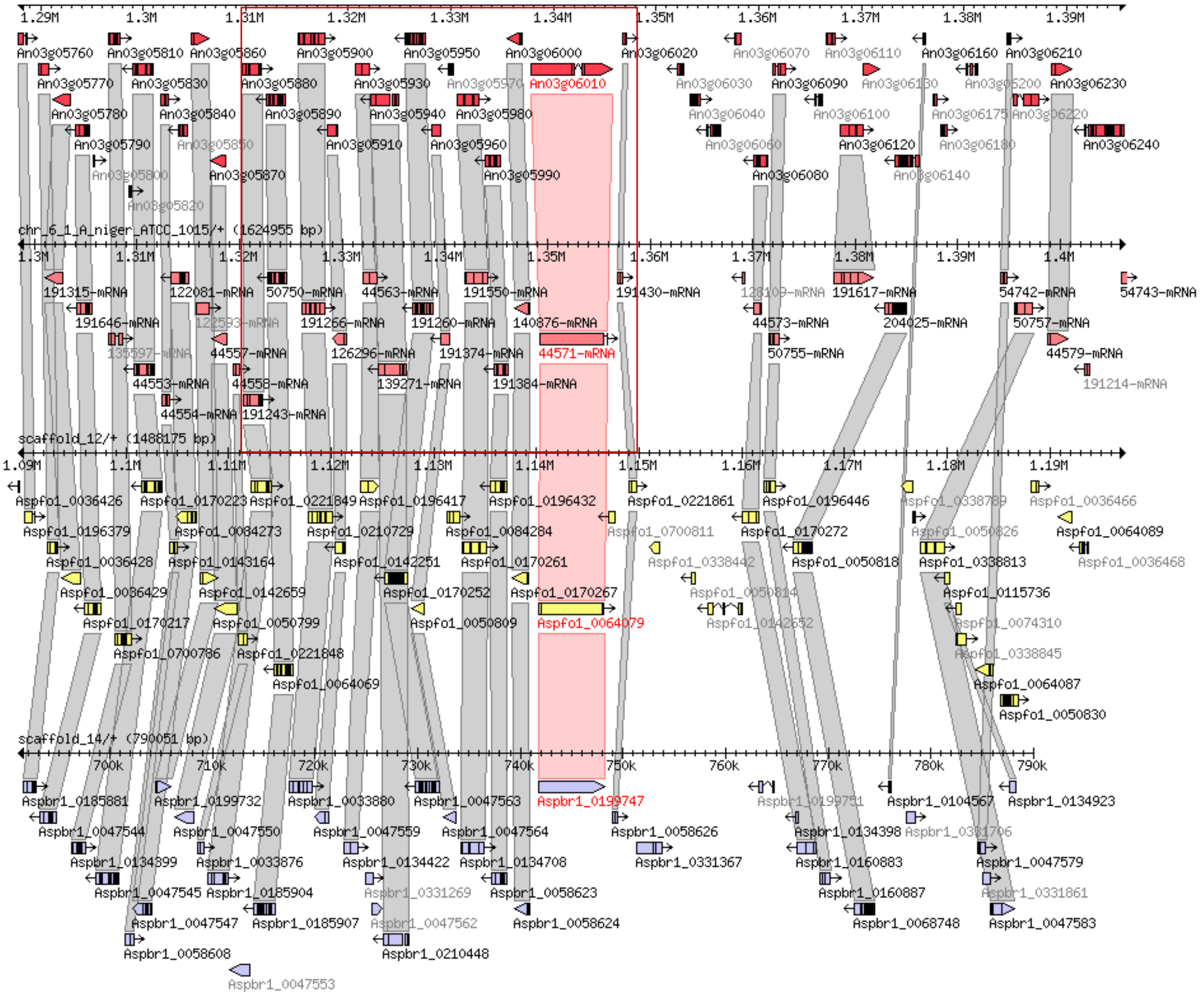

An03g06010 cluster

Gene id (SMURF)	Gene positions	Gene distance	AspGD name	AspGD description line or supplemental annotation	protein ID (antiSMASH)	AspGD ID	
An03g05810	19	719		Ortholog of A. nidulans FGSC A4 : AN6419, AN6946, AN5312, AN5664, AN8984, AN8971, AN9387, AN1317 and A. fumigatus Af293 : Afu5g13725	n/a	An03g05810	
An03g05820	18	55			n/a	An03g05820	
An03g05830	17	770		Domain(s) with predicted role in transmembrane transport and integral to membrane localization	n/a	An03g05830	
An03g05840	16	792		Domain(s) with predicted N-acetyltransferase activity and role in metabolic process	n/a	An03g05840	
An03g05850	15	366		Domain(s) with predicted sequence-specific DNA binding RNA polymerase II transcription factor activity, sequence-specific DNA binding transcription factor activity, zinc ion binding activity	n/a	An03g05850	
An03g05860	14	128		Ortholog of A. nidulans FGSC A4 : AN6383, A. fumigatus Af293 : Afu1g03300, Afu5g00450, Afu6g10780, A. oryzae RIB40 : AO090003001528, AO090023001010 and A. niger ATCC 1015 : 47798-mRNA	n/a	An03g05860	
An03g05870	13	1589		Domain(s) with predicted oxidoreductase activity and role in oxidation-reduction process	n/a	An03g05870	
An03g05880	12	362		Putative amidase/acetamidase	n/a	An03g05880	ECS
An03g05890	11	1318		Domain(s) with predicted role in transmembrane transport and integral to membrane localization	n/a	An03g05890	
An03g05900	10	321		Domain(s) with predicted sequence-specific DNA binding RNA polymerase II transcription factor activity, sequence-specific DNA binding transcription factor activity, zinc ion binding activity	n/a	An03g05900	
An03g05910	9	1677		Ortholog of A. nidulans FGSC A4 : AN12339, A. fumigatus Af293 : Afu8g01800, A. oryzae RIB40 : AO090010000636, A. niger ATCC 1015 : 126296-mRNA and A. versicolor : Aspve1_0046675	prot_ID_388	An03g05910	
An03g05930	8	283		Domain(s) with predicted acid phosphatase activity	prot_ID_342	An03g05930	
An03g05940	7	808	gfaB	Putative glutamine:fructose-6-phosphate amidotransferase; induced by caspofungin	prot_ID_230	An03g05940	
An03g05950	6	667		Domain(s) with predicted role in transmembrane transport and integral to membrane localization	prot_ID_491	An03g05950	
An03g05960	5	689		Domain(s) with predicted catalytic activity	prot_ID_141	An03g05960	
An03g05970	4	317		Protein of unknown function	prot_ID_17	An03g05970	
An03g05980	3	549		Domain(s) with predicted DNA binding, zinc ion binding activity, role in transcription, DNA-dependent and	prot_ID_103	An03g05980	

			nucleus localization			
An03g05990	2	615	Domain(s) with predicted O-methyltransferase activity	prot_ID_267	An03g05990	
An03g06000	1	896	Domain(s) with predicted UDP-N-acetylmuramate dehydrogenase activity, flavin adenine dinucleotide binding activity and role in oxidation-reduction process	prot_ID_237	An03g06000	
**An03g06010**	0	0	Domain(s) with predicted ACP phosphopantetheine attachment site binding involved in fatty acid biosynthetic process, ligase activity, nucleotide binding, phosphopantetheine binding activity	**prot_ID_645**	**An03g06010**	
An03g06020	-1	1076	4-carboxymuconolactone decarboxylase	prot_ID_66	An03g06020	ECS, IGD
n/a				prot_ID_222	An03g06030	
n/a				prot_ID_476	An03g06040	
n/a				prot_ID_260	An03g06060	
n/a				prot_ID_311	An03g06070	
n/a			Has domain(s) with predicted sequence-specific DNA binding RNA polymerase II transcription factor activity, sequence-specific DNA binding transcription factor activity, zinc ion binding activity	prot_ID_278	An03g06080	
n/a			Has domain(s) with predicted role in response to stress and integral to membrane localization	prot_ID_145	An03g06090	
n/a				prot_ID_379	An03g06100	

An03g06380 cluster

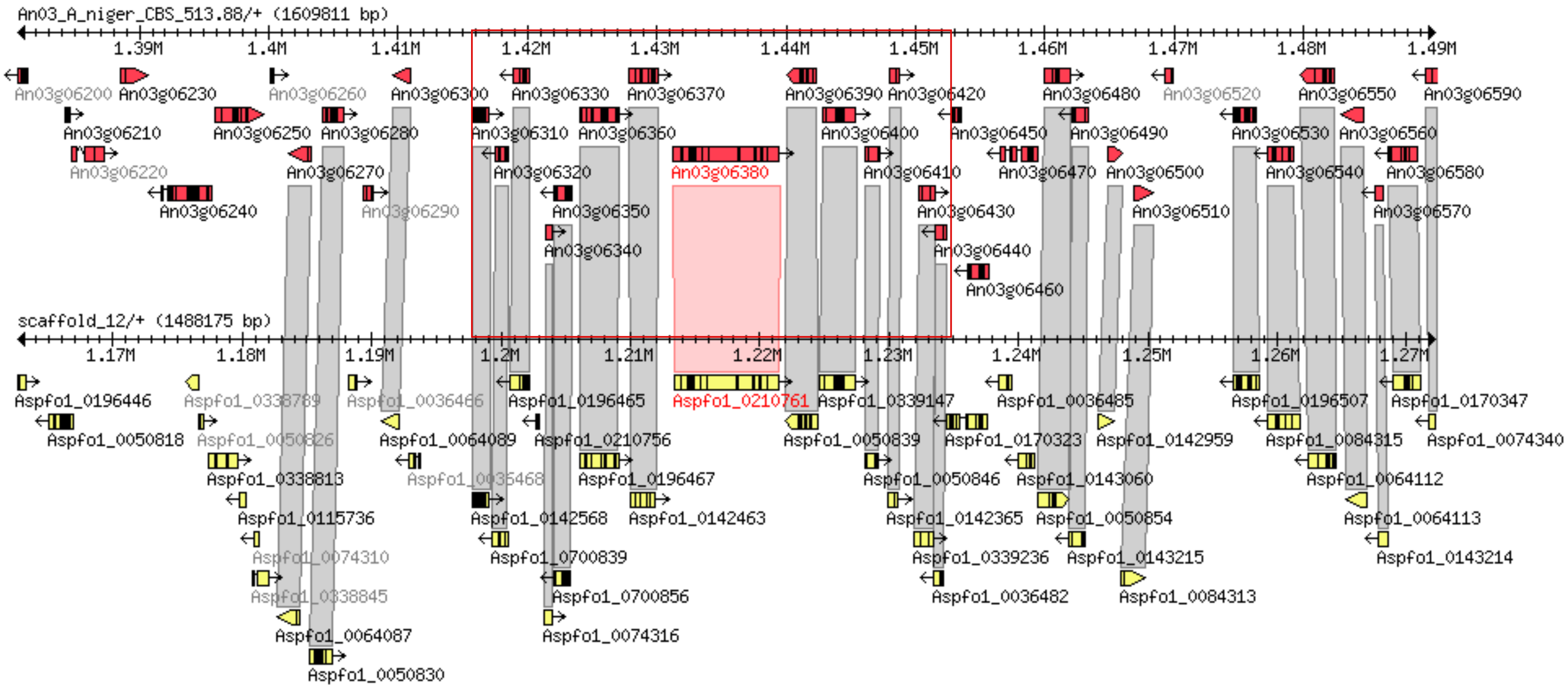

An03g06380 cluster

Gene id (SMURF)	Gene positions	Gene distance	AspGD name	AspGD description line or supplemental annotation	protein ID (antiSMASH)	AspGD ID	
n/a			pmeA	Pectin methylesterase	prot_ID_586	An03g06310	EGS, IGD
n/a				Has domain(s) with predicted catalytic activity and role in metabolic process	prot_ID_414	An03g06320	
n/a				Ortholog of A. nidulans FGSC A4 : AN9032, AN10883, A. fumigatus Af293 : Afu7g06950, Afu8g02470, A. niger CBS 513.88 : An03g00350, An05g02580 and A. oryzae RIB40 : AO090010000660	prot_ID_20	An03g06330	
n/a				Ortholog of A. nidulans FGSC A4 : AN11171, AN7148, A. fumigatus Af293 : Afu4g03595, A. niger CBS 513.88 : An02g01320, An14g01685 and A. oryzae RIB40 : AO090026000213, AO090026000142, AO090023000130	prot_ID_480	An03g06340	
n/a				Ortholog of A. nidulans FGSC A4 : AN4366, A. brasiliensis : Aspbr1_0060292, A. acidus : Aspfo1_0700856, A. versicolor : Aspve1_0752573 and A. sydowii : Aspsy1_0051206	prot_ID_118	An03g06350	
n/a				Putative chitin synthase	prot_ID_567	An03g06360	
An03g06370	1	1069		Domain(s) with predicted DNA binding, zinc ion binding activity, role in transcription, DNA-dependent and nucleus localization	prot_ID_290	An03g06370	
**An03g06380**	0	0		Domain(s) with predicted ACP phosphopantetheine attachment site binding involved in fatty acid biosynthetic process, nucleotide binding, oxidoreductase activity and transferase activity, more	**prot_ID_272**	**An03g06380**	
An03g06390	-1	612		Domain(s) with predicted monooxygenase activity and role in oxidation-reduction process	prot_ID_655	An03g06390	
An03g06400	-2	528		Domain(s) with predicted DNA binding, zinc ion binding activity, role in transcription, DNA-dependent and nucleus localization	prot_ID_51	An03g06400	
An03g06410	-3	777	erg25	Putative C-4 methyl sterol oxidase; induced by fenpropimorph	prot_ID_89	An03g06410	
An03g06420	-4	799		Domain(s) with predicted N-acetyltransferase activity and role in metabolic process	prot_ID_84	An03g06420	
An03g06430	-5	1414		Domain(s) with predicted nucleotide binding, oxidoreductase activity, transferase activity, transferring acyl groups other than amino-acyl groups, zinc ion binding activity and role in oxidation-reduction process	prot_ID_537	An03g06430	
An03g06440	-6	59		Ortholog of A. nidulans FGSC A4 : AN8906, A. fumigatus Af293 : Afu8g02420, A. oryzae RIB40 : AO090010000669, A. niger ATCC 1015 : 126819-mRNA and A. sydowii : Aspsy1_0072834	prot_ID_562	An03g06440	ECS

An03g06450	-7	465	Domain(s) with predicted cell outer membrane, integral to membrane localization	prot_ID_512	An03g06450
An03g06460	-8	445	Domain(s) with predicted electron carrier activity, heme binding, iron ion binding, monooxygenase activity, oxidoreductase activity, acting on paired donors, with incorporation or reduction of molecular oxygen activity	prot_ID_580	An03g06460
An03g06470	-9	927	Ortholog of Aspergillus carbonarius ITEM 5010 : Acar5010_399475	prot_ID_200	An03g06470
An03g06480	-10	525	Ortholog(s) have monooxygenase activity and role in sterigmatocystin biosynthetic process	n/a	An03g06470
An03g06490	-11	127	Ortholog(s) have lipase activity	n/a	An03g06480
An03g06500	-12	1440	Domain(s) with predicted monooxygenase activity and role in oxidation-reduction process	n/a	An03g06490
An03g06510	-13	838	Domain(s) with predicted sequence-specific DNA binding RNA polymerase II transcription factor activity, sequence-specific DNA binding transcription factor activity, zinc ion binding activity	n/a	An03g06500
An03g06520	-14	840		n/a	An03g06510

An04g01150 cluster

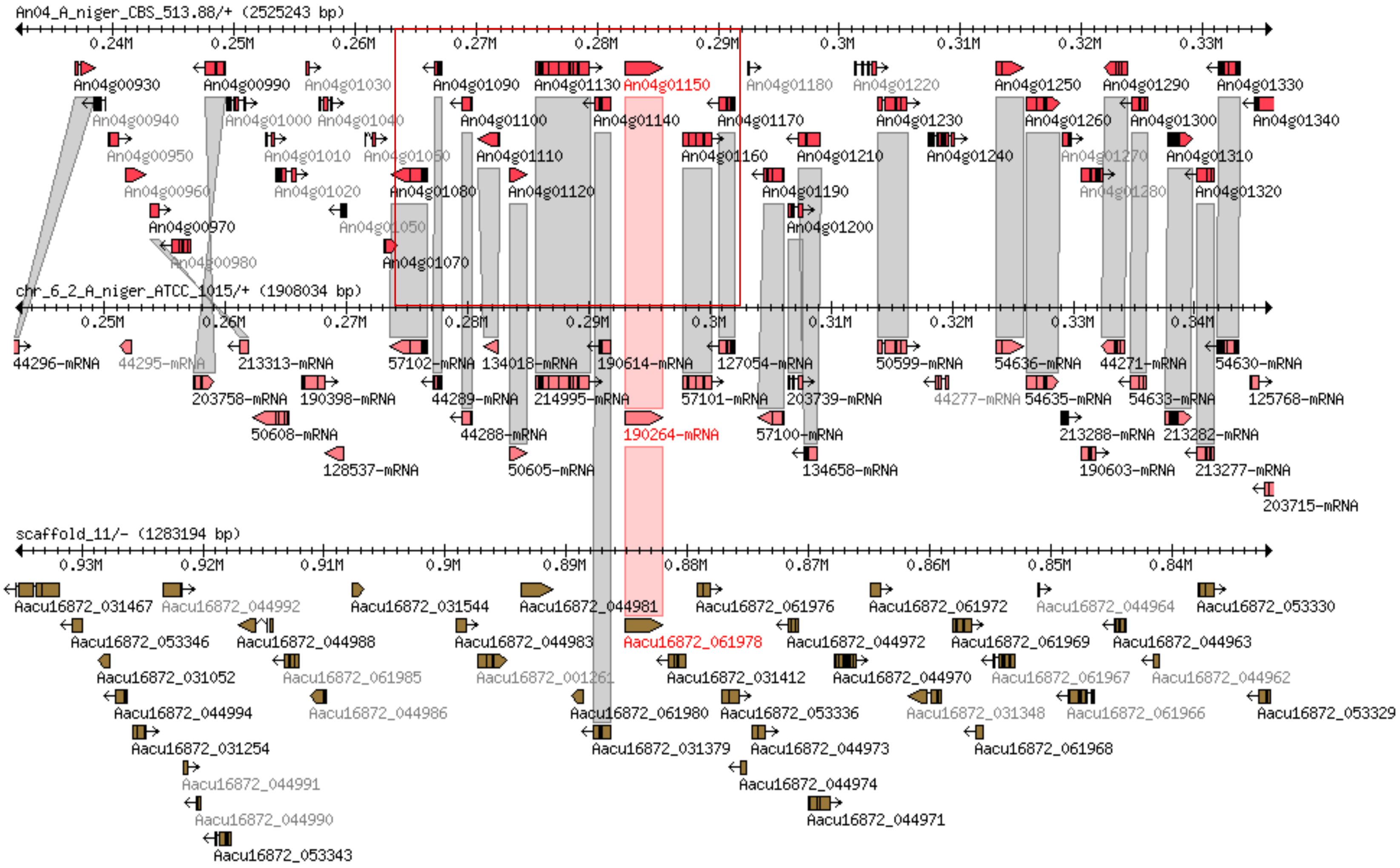

An04g01150 cluster

Gene id (SMURF)	Gene positions	Gene distance	AspGD name	AspGD description line or supplemental annotation	protein ID (antiSMASH)	AspGD ID	
n/a				Ortholog of A. brasiliensis : Aspbr1_0127044, A. acidus : Aspfo1_0044764 and Aspergillus carbonarius ITEM 5010 : Acar5010_002835	prot_ID_168	An04g01070	
n/a				Ribonucleotide reductase	prot_ID_665	An04g01080	ECS
n/a				Ortholog of A. nidulans FGSC A4 : AN4381, A. fumigatus Af293 : Afu4g06700, A. oryzae RIB40 : AO090023000915, A. niger ATCC 1015 : 44289-mRNA and A. versicolor : Aspve1_0043230	prot_ID_731	An04g01090	
n/a				Has domain(s) with predicted nucleoside-triphosphatase activity, nucleotide binding activity	prot_ID_493	An04g01100	
n/a				Ortholog of A. nidulans FGSC A4 : AN4383, A. fumigatus Af293 : Afu4g06730, A. oryzae RIB40 : AO090023000913, A. niger ATCC 1015 : 134018-mRNA and A. versicolor : Aspve1_0043227	prot_ID_862	An04g01110	
An04g01120	3	872		Tetrahydrofolylpolyglutamate synthase	prot_ID_484	An04g01120	
An04g01130	2	345		Ortholog(s) have role in cytokinesis, actomyosin contractile ring assembly	prot_ID_58	An04g01130	
An04g01140	1	1193		8-Amino-7-oxononanoate synthase	prot_ID_261	An04g01140	
**An04g01150**	0	0		Domain(s) with predicted ACP phosphopantetheine attachment site binding involved in fatty acid biosynthetic process, L-aminoadipate-semialdehyde dehydrogenase activity, ligase activity, nucleotide binding activity	**prot_ID_858**	**An04g01150**	
An04g01160	-1	1670		Domain(s) with predicted transferase activity, transferring phosphorus-containing groups activity	prot_ID_458	An04g01160	
An04g01170	-2	1390		Ortholog(s) have role in cellular iron ion homeostasis, iron-sulfur cluster assembly, tRNA wobble uridine modification and mitochondrial matrix localization	prot_ID_969	An04g01170	ECS
n/a				Protein of unknown function	prot_ID_737	An04g01180	
n/a				Ortholog of A. nidulans FGSC A4 : AN4387, A. fumigatus Af293 : Afu4g06780, A. oryzae RIB40 : AO090023000908, A. niger ATCC 1015 : 57100-mRNA and A. versicolor : Aspve1_0750571	prot_ID_402	An04g01190	
n/a				Ortholog(s) have ubiquinol-cytochrome-c reductase activity and role in aerobic respiration, mitochondrial electron transport, ubiquinol to cytochrome c, mitochondrial respiratory chain complex III assembly	prot_ID_855	An04g01200	
n/a				Has domain(s) with predicted catalytic activity and role in base-excision repair	prot_ID_86	An04g01210	

n/a		Protein of unknown function	prot_ID_698	An04g01220
n/a	ecmA	Putative cell wall organization protein	prot_ID_342	An04g01230

An04g04340/An04g04380 cluster

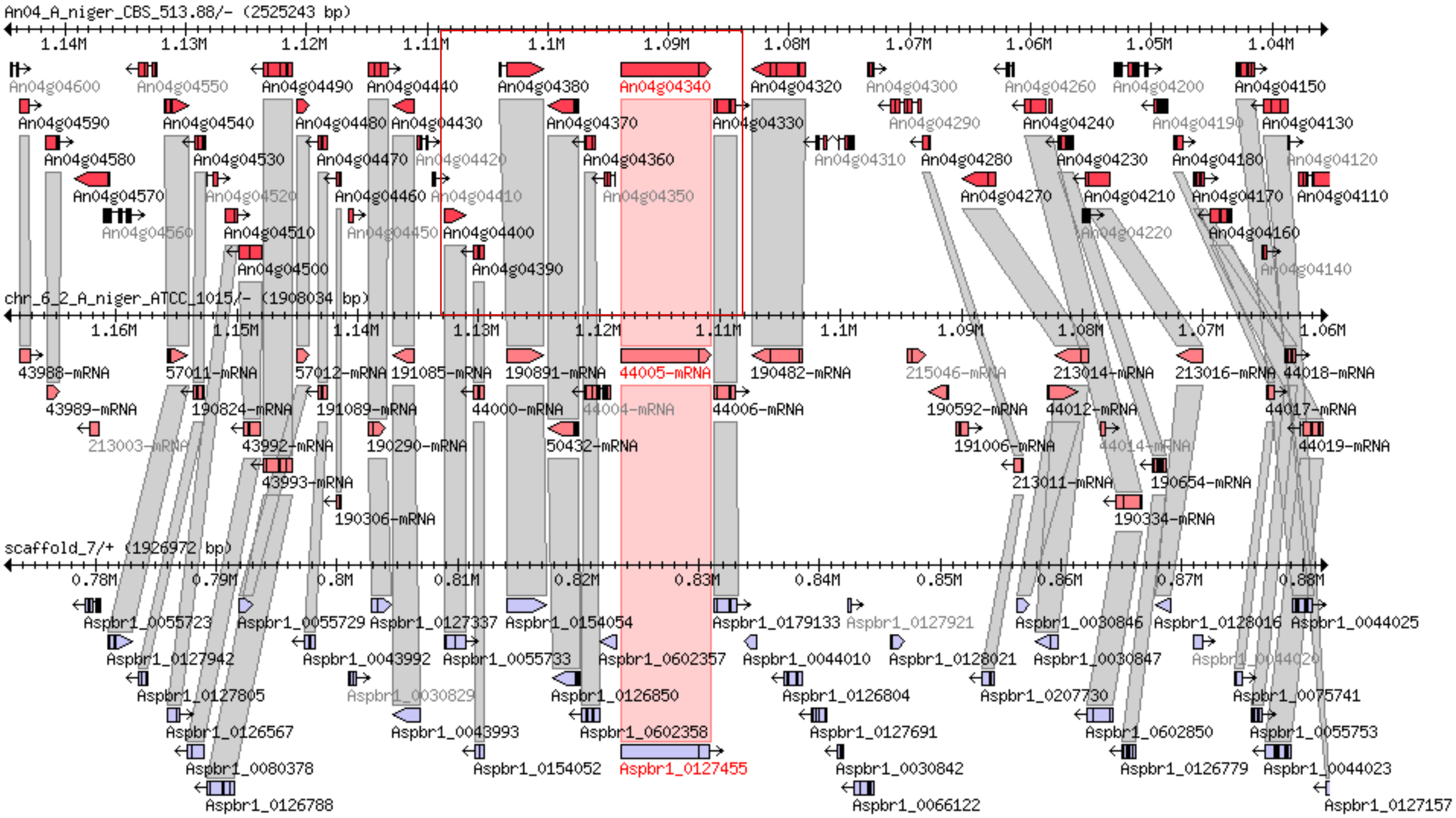

An04g04340 cluster

Gene id (SMURF)	Gene positions	Gene distance	AspGD name	AspGD description line or supplemental annotation	protein ID (antiSMASH)	AspGD ID	
An04g04220	11	701		Protein of unknown function	n/a	An04g04220	
An04g04230	10	664		Ortholog of A. nidulans FGSC A4 : AN1613, A. oryzae RIB40 : AO090023000625, A. niger ATCC 1015 : 190654-mRNA, A. versicolor : Aspve1_0037431 and A. sydowii : Aspsy1_0055705	n/a	An04g04230	
An04g04240	9	984		Domain(s) with predicted transmembrane transporter activity, role in transmembrane transport and integral to membrane localization	n/a	An04g04240	
An04g04260	8	873		Protein of unknown function	n/a	An04g04260	
An04g04270	7	3511		Domain(s) with predicted N,N-dimethylaniline monooxygenase activity, NADP binding, flavin adenine dinucleotide binding activity and role in oxidation-reduction process	n/a	An04g04270	
An04g04280	6	219		Ortholog of A. nidulans FGSC A4 : AN1608, A. fumigatus Af293 : Afu4g09250, A. oryzae RIB40 : AO090023000611, A. niger ATCC 1015 : 213011-mRNA and A. versicolor : Aspve1_0024878, Aspve1_0041545	prot_ID_411	An04g04280	
An04g04290	5	1334		Protein of unknown function	prot_ID_338	An04g04290	
An04g04300	4	1245		Protein of unknown function	prot_ID_225	An04g04300	
An04g04310	3	845		Protein of unknown function	prot_ID_828	An04g04310	
An04g04320	2	1266		Ortholog of A. nidulans FGSC A4 : AN3342, AN10302, AN4609, A. fumigatus Af293 : Afu7g06670, A. niger CBS 513.88 : An16g05670, An04g03250 and A. oryzae RIB40 : AO090011000012	prot_ID_146	An04g04320	
An04g04330	1	289		Ferulate:CoA ligase (AMP-forming)	prot_ID_141	An04g04330	ECS
**An04g04340**	0	0		Domain(s) with predicted ACP phosphopantetheine attachment site binding involved in fatty acid biosynthetic process, nucleotide binding, phosphopantetheine binding, transferase activity and role in biosynthetic process	**prot_ID_445**	**An04g04340**	
An04g04350	-1	465		Protein of unknown function	prot_ID_765	An04g04350	
An04g04360	-2	713		Domain(s) with predicted hydrolase activity, acting on ester bonds activity and role in biosynthetic process	prot_ID_109	An04g04360	
An04g04370	-3	488		Domain(s) with predicted ammonia-lyase activity, role in L-phenylalanine catabolic process, biosynthetic process and cytoplasm localization	prot_ID_653	An04g04370	

An04g04380	-4	659	Domain(s) with predicted ACP phosphopantetheine attachment site binding involved in fatty acid biosynthetic process, L-aminoadipate-semialdehyde dehydrogenase activity, ligase activity, nucleotide binding activity	prot_ID_68	An04g04380	
An04g04390	-5	1402	Domain(s) with predicted catalytic activity and role in metabolic process	prot_ID_474	An04g04390	
An04g04400	-6	770	Ortholog of A. nidulans FGSC A4 : AN3871, A. oryzae RIB40 : AO090001000062, A. brasiliensis : Aspbr1_0055733, N. fischeri NRRL 181 : NFIA_045750 and A. acidus : Aspfo1_0080449	prot_ID_769	An04g04400	ECS
An04g04410	-7	658	Protein of unknown function	prot_ID_749	An04g04410	
An04g04420	-8	340	Protein of unknown function	prot_ID_111	An04g04420	
An04g04430	-9	285	Tannin acyl hydrolase with a predicted role in tannic acid degradation	prot_ID_107	An04g04430	
An04g04440	-10	420	Domain(s) with predicted UDP-N-acetylmuramate dehydrogenase activity, flavin adenine dinucleotide binding activity and role in oxidation-reduction process	prot_ID_67	An04g04440	
An04g04450	-11	1273	Protein of unknown function	prot_ID_1011	An04g04450	
An04g04460	-12	648	Ortholog of A. nidulans FGSC A4 : AN11871, A. fumigatus Af293 : Afu1g11260, Afu6g00270, A. niger CBS 513.88 : An03g03440 and A. niger ATCC 1015 : 190306-mRNA	prot_ID_848	An04g04460	
An04g04470	-13	765	Domain(s) with predicted N-acetyltransferase activity and role in metabolic process	prot_ID_466	An04g04470	
An04g04480	-14	754	Domain(s) with predicted nucleotide binding, oxidoreductase activity, zinc ion binding activity and role in oxidation-reduction process	prot_ID_916	An04g04480	
An04g04490	-15	394	Domain(s) with predicted DNA binding, sequence-specific DNA binding RNA polymerase II transcription factor activity, sequence-specific DNA binding transcription factor activity, zinc ion binding activity	prot_ID_709	An04g04490	
An04g04500	-16	460	Ortholog of A. brasiliensis : Aspbr1_0080378, A. acidus : Aspfo1_0572809, Aspergillus niger ATCC 1015 : 43992-mRNA and Aspergillus terreus NIH2624 : ATET_03625	prot_ID_110	An04g04500	
An04g04510	-17	113	Domain(s) with predicted protein dimerization activity, sequence-specific DNA binding, sequence-specific DNA binding transcription factor activity and role in regulation of transcription, DNA-dependent	n/a	An04g04510	
An04g04520	-18	542	Protein of unknown function	n/a	An04g04520	

An04g06260 cluster

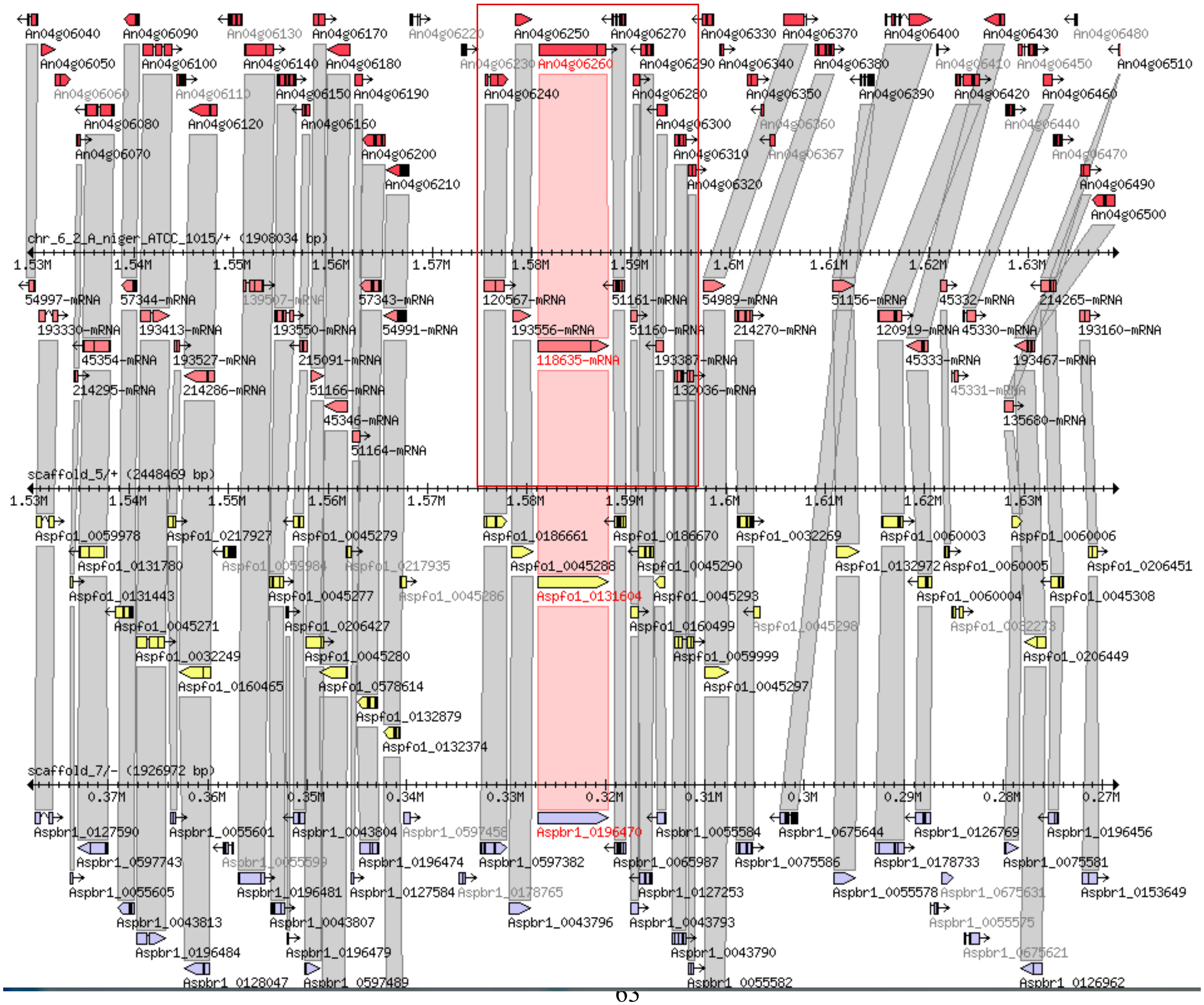

An04g06260 cluster

Gene id (SMURF)	Gene positions	Gene distance	AspGD name	AspGD description line or supplemental annotation	protein ID (antiSMASH)	AspGD ID	
n/a				Ortholog of A. nidulans FGSC A4 : AN1988, A. fumigatus Af293 : Afu4g10490, A. oryzae RIB40 : AO090003001162, A. niger ATCC 1015 : 45346-mRNA and A. versicolor : Aspve1_0025388	prot_ID_1004	An04g06180	
n/a				Ortholog(s) have structural constituent of ribosome activity and mitochondrial large ribosomal subunit localization	prot_ID_812	An04g06190	
n/a				Has domain(s) with predicted carboxy-lyase activity, pyridoxal phosphate binding activity and role in carboxylic acid metabolic process	prot_ID_908	An04g06200	
n/a				Homocitrate synthase	prot_ID_664	An04g06210	
n/a					prot_ID_245	An04g06220	
An04g06230	3	1711			prot_ID_794	An04g06230	
An04g06240	2	820		Ortholog of A. fumigatus Af293 : Afu3g02480, Afu3g15290, Afu4g03670, Afu5g01460 and A. niger CBS 513.88 : An03g02140, An07g05020, An14g01490, An11g08910	prot_ID_33	An04g06240	ECS
An04g06250	1	743		Domain(s) with predicted role in transmembrane transport and integral to membrane localization	prot_ID_522	An04g06250	
**An04g06260**	0	0		Domain(s) with predicted ACP phosphopantetheine attachment site binding involved in fatty acid biosynthetic process, ligase activity, phosphopantetheine binding activity	**prot_ID_529**	**An04g06260**	
An04g06270	-1	590		Ortholog of A. nidulans FGSC A4 : AN3975, AN9193, AN2891 and A. fumigatus Af293 : Afu1g10150, Afu2g04380, Afu3g15150, Afu3g15280, Afu6g03300	prot_ID_533	An04g06270	
An04g06280	-2	643		Ortholog of A. fumigatus Af293 : Afu3g15240, A. brasiliensis : Aspbr1_0031971, Aspbr1_0043793, N. fischeri NRRL 181 : NFIA_005300, NFIA_061840 and Aspergillus flavus NRRL 3357 : AFL2T_06120	prot_ID_217	An04g06280	
An04g06290	-3	178		Domain(s) with predicted DNA binding, sequence-specific DNA binding RNA polymerase II transcription factor activity, sequence-specific DNA binding transcription factor activity, zinc ion binding activity	prot_ID_10	An04g06290	
n/a				Ortholog of A. fumigatus Af293 : Afu4g10450, A. oryzae RIB40 : AO090003001167, A. versicolor : Aspve1_0465452, A. sydowii : Aspsy1_0084433 and Aspergillus terreus NIH2624 : ATET_00905	prot_ID_544	An04g06300	

n/a	Ortholog of A. nidulans FGSC A4 : AN10255, A. fumigatus Af293 : Afu4g10440, A. oryzae RIB40 : AO090003001168, A. versicolor : Aspve1_0037986 and A. sydowii : Aspsy1_0039153	prot_ID_242	An04g06310	
n/a	Has domain(s) with predicted aminoacyl-tRNA hydrolase activity and role in translation	prot_ID_620	An04g06320	ECS
n/a	Has domain(s) with predicted nucleotide binding activity	prot_ID_319	An04g06330	
n/a	Has domain(s) with predicted isomerase activity and role in cellular aromatic compound metabolic process	prot_ID_849	An04g06340	
n/a	Has domain(s) with predicted 3-beta-hydroxy-delta5-steroid dehydrogenase activity, nucleotide binding activity and role in oxidation-reduction process, steroid biosynthetic process	prot_ID_101	An04g06350	
n/a		prot_ID_424	An04g06360	
n/a		prot_ID_9	An04g06367	
n/a	Ortholog of A. nidulans FGSC A4 : AN1992, A. fumigatus Af293 : Afu4g10420, A. oryzae RIB40 : AO090003001170, A. niger ATCC 1015 : 54989-mRNA and A. versicolor : Aspve1_0186037	prot_ID_286	An04g06370	

An04g09530 cluster

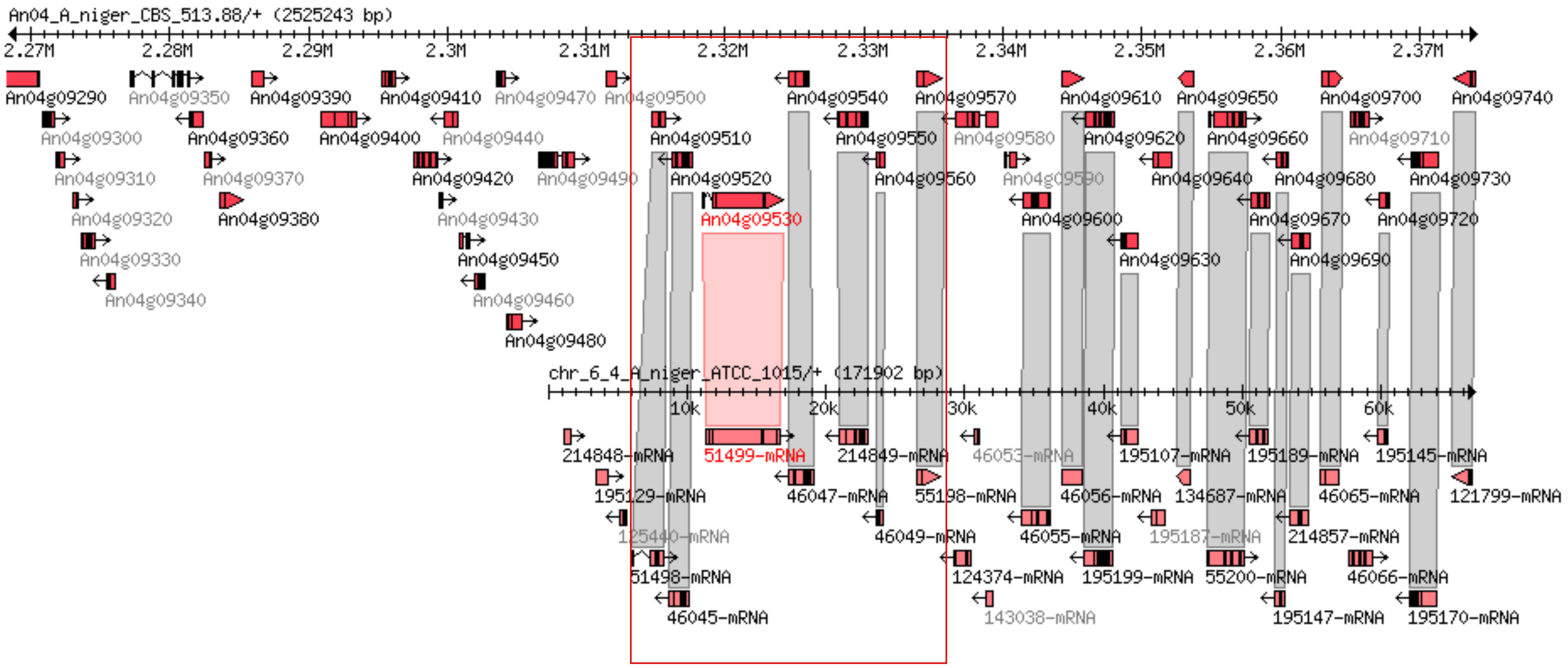

An04g09530 cluster

Gene id (SMURF)	Gene positions	Gene distance	AspGD name	AspGD description line or supplemental annotation	protein ID (antiSMASH)	AspGD ID	
n/a				Ortholog of A. nidulans FGSC A4 : AN2821, A. fumigatus Af293 : Afu2g00180, Afu2g17480, A. niger CBS 513.88 : An03g05360 and A. oryzae RIB40 : AO090102000063, AO090009000716	prot_ID_915	An04g09420	
n/a				Protein of unknown function	prot_ID_730	An04g09430	
n/a				Protein of unknown function	prot_ID_779	An04g09440	
n/a				Ortholog of A. acidus : Aspfo1_0080746	prot_ID_975	An04g09450	
n/a				Protein of unknown function	prot_ID_232	An04g09460	
n/a				Protein of unknown function	prot_ID_800	An04g09470	
n/a				Has domain(s) with predicted nucleotide binding, oxidoreductase activity and role in oxidation-reduction process	prot_ID_367	An04g09480	
n/a				Protein of unknown function	prot_ID_938	An04g09490	
An04g09500	3	2641		Domain(s) with predicted inositol oxygenase activity, iron ion binding activity, role in inositol catabolic process, oxidation-reduction process and cytoplasm localization	prot_ID_608	An04g09500	
An04g09510	2	354		Domain(s) with predicted methyltransferase activity and role in metabolic process	prot_ID_600	An04g09510	ECS
An04g09520	1	813		Putative O-methyltransferase	prot_ID_941	An04g09520	
**An04g09530**	0	0		Ortholog(s) have role in arugosin biosynthetic process, asperthecin biosynthetic process, emodin biosynthetic process, endocrocin biosynthetic process and monodictyphenone biosynthetic process, more	**prot_ID_434**	**An04g09530**	
An04g09540	-1	448		Domain(s) with predicted electron carrier activity, heme binding, iron ion binding, monooxygenase activity, oxidoreductase activity, acting on paired donors, with incorporation or reduction of molecular oxygen activity	prot_ID_572	An04g09540	
An04g09550	-2	2003		Putative 4-hydroxyacetophenone monooxygenase	prot_ID_104	An04g09550	
An04g09560	-3	658		Ortholog(s) have extracellular region localization	prot_ID_408	An04g09560	
An04g09570	-4	2552		Domain(s) with predicted role in transmembrane transport and integral to membrane localization	prot_ID_689	An04g09570	ECS
An04g09580	-5	850		Domain(s) with predicted transferase activity, transferring phosphorus-containing groups activity	prot_ID_476	An04g09580	
An04g09590	-6	598		Protein of unknown function	prot_ID_524	An04g09590	

An04g09600	-7	562	Ortholog of A. oryzae RIB40 : AO090010000288, A. brasiliensis : Aspbr1_0059125, A. niger ATCC 1015 : 46055-mRNA and Aspergillus aculeatus ATCC16872 : Aacu16872_060894	prot_ID_13	An04g09600
An04g09610	-8	859	Ortholog of A. nidulans FGSC A4 : AN3528, A. oryzae RIB40 : AO090113000188, A. brasiliensis : Aspbr1_0162074, A. niger ATCC 1015 : 46056-mRNA and Aspergillus flavus NRRL 3357 : AFL2T_08660	n/a	An04g09610
An04g09620	-9	122	Domain(s) with predicted amino acid transmembrane transporter activity, role in amino acid transmembrane transport and membrane localization	n/a	An04g09620
An04g09630	-10	503	Ortholog of A. nidulans FGSC A4 : AN10915, A. niger CBS 513.88 : An03g01920, A. niger ATCC 1015 : 195107-mRNA, 45712-mRNA and A. versicolor : Aspve1_0513563	n/a	An04g09630
An04g09640	-11	1015	Ortholog of A. nidulans FGSC A4 : AN0196, A. fumigatus Af293 : Afu5g14200, Afu6g13810, Afu8g01720, A. niger CBS 513.88 : An15g02090, A. oryzae RIB40 : AO090003001444 and A. niger ATCC 1015 : 182943-mRNA	n/a	An04g09640
An04g09650	-12	505	Domain(s) with predicted catalytic activity	n/a	An04g09650

An04g10030 cluster
No manual prediction made

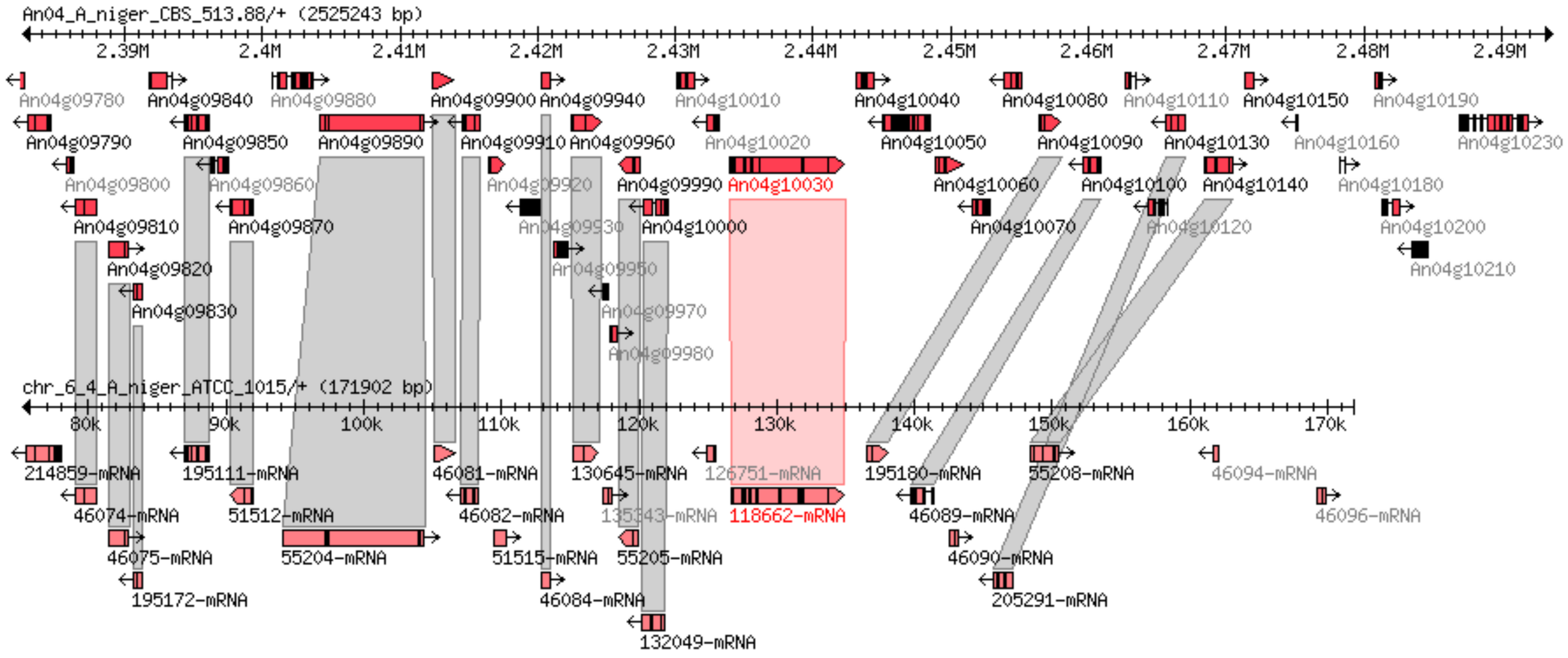

An04g10030 cluster

Gene id (SMURF)	Gene positions	Gene distance	AspGD name	AspGD description line or supplemental annotation	protein ID (antiSMASH)	AspGD ID
An04g09890	14	755	agsA	Putative 1,3-alpha-glucan synthase; induced in the presence of cell wall stress-inducing compounds such as Calcofluor White, SDS, and caspofungin	n/a	An04g09890
An04g09900	13	408		Domain(s) with predicted transferase activity, transferring phosphorus-containing groups activity	prot_ID_201	An04g09900
An04g09910	12	696		Domain(s) with predicted nucleotide binding, oxidoreductase activity, zinc ion binding activity and role in oxidation-reduction process	prot_ID_752	An04g09910
An04g09920	11	989		Domain(s) with predicted nucleotide binding, oxidoreductase activity and role in oxidation-reduction process	prot_ID_897	An04g09920
An04g09930	10	205		Protein of unknown function	prot_ID_355	An04g09930
An04g09940	9	242		Domain(s) with predicted role in cell wall macromolecule catabolic process	prot_ID_401	An04g09940
An04g09950	8	179		Protein of unknown function	prot_ID_571	An04g09950
An04g09960	7	156		Ortholog of A. brasiliensis : Aspbr1_0661759, A. acidus : Aspfo1_0187220 and A. niger ATCC 1015 : 130645-mRNA	prot_ID_776	An04g09960
An04g09970	6	125		Protein of unknown function	prot_ID_74	An04g09970
An04g09980	5	230		Protein of unknown function	prot_ID_554	An04g09980
An04g09990	4	573		Domain(s) with predicted nucleotide binding, oxidoreductase activity and role in oxidation-reduction process	prot_ID_726	An04g09990
An04g10000	3	682		Domain(s) with predicted transporter activity, role in transmembrane transport and integral to membrane localization	prot_ID_209	An04g10000
An04g10010	2	853		Protein of unknown function	prot_ID_22	An04g10010
An04g10020	1	679		Protein of unknown function	prot_ID_112	An04g10020
**An04g10030**	0	0		Putative polyketide synthase (PKS)	**prot_ID_145**	**An04g10030**
An04g10040	-1	774		Putative myo-inositol 2-dehydrogenase	prot_ID_712	An04g10040
An04g10050	-2	609		Domain(s) with predicted DNA binding, sequence-specific DNA binding RNA polymerase II transcription factor activity, sequence-specific DNA binding transcription factor activity, zinc ion binding activity	prot_ID_429	An04g10050
An04g10060	-3	326		Domain(s) with predicted N,N-dimethylaniline monooxygenase activity, NADP binding, flavin adenine dinucleotide binding activity and role in oxidation-reduction process	prot_ID_997	An04g10060
An04g10070	-4	669		Glycerol dehydrogenase	prot_ID_758	An04g10070

An04g10080	-5	929	Ortholog of A. brasiliensis : Aspbr1_0069169, A. acidus : Aspfo1_0070728, A. versicolor : Aspve1_0151102 and A. sydowii : Aspsy1_0057347	prot_ID_329	An04g10080
An04g10090	-6	1212	Domain(s) with predicted substrate-specific transmembrane transporter activity, role in transmembrane transport and integral to membrane localization	prot_ID_375	An04g10090
n/a			Ortholog of A. niger ATCC 1015 : 46089-mRNA	prot_ID_692	An04g10100

An05g01060 cluster

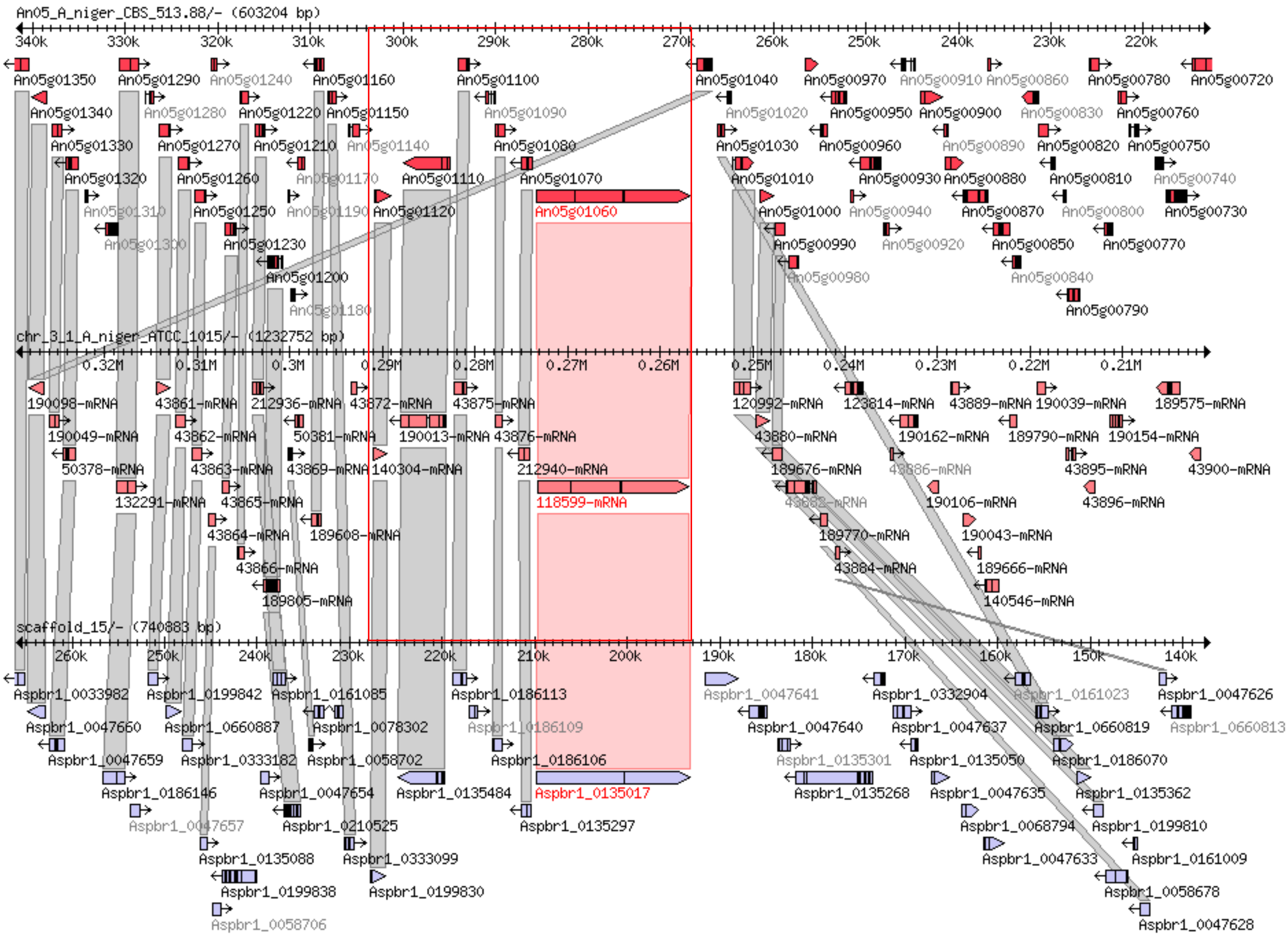

An05g01060 cluster

Gene id (SMURF)	Gene positions	Gene distance	AspGD name	AspGD description line or supplemental annotation	protein ID (antiSMASH)	AspGD ID	
n/a				Protein similar to mitochondrial malic enzyme (NADP-specific); expression repressed by tunicamycin and DTT	prot_ID_26	An05g00930	
n/a					prot_ID_64	An05g00940	
n/a				Domain(s) with predicted FMN binding, oxidoreductase activity and role in oxidation-reduction process	prot_ID_160	An05g00950	
n/a				Domain(s) with predicted rRNA binding activity and intracellular localization	prot_ID_5	An05g00960	
n/a				Ortholog of A. nidulans FGSC A4 : AN6172, A. fumigatus Af293 : Afu2g08340, A. oryzae RIB40 : AO090011000871, A. versicolor : Aspve1_0028126 and A. sydowii : Aspsy1_0151670	prot_ID_149	An05g00970	
n/a				Domain(s) with predicted DNA binding, zinc ion binding activity, role in transcription, DNA-dependent and nucleus localization	prot_ID_217	An05g00980	
n/a				Domain(s) with predicted oxidoreductase activity and role in oxidation-reduction process	prot_ID_201	An05g00990	
n/a				Domain(s) with predicted catalytic activity and role in nucleoside metabolic process	prot_ID_34	An05g01000	
n/a				Domain(s) with predicted phospholipase activity and role in phospholipid catabolic process	prot_ID_196	An05g01010	
An05g01020	3	195			prot_ID_178	An05g01020	
An05g01030	2	622		Ortholog of A. nidulans FGSC A4 : AN9205, A. fumigatus Af293 : Afu5g00100, Afu7g06523, A. niger CBS 513.88 : An16g01380, A. oryzae RIB40 : AO090011000069 and A. niger ATCC 1015 : 41357-mRNA	prot_ID_16	An05g01030	
An05g01040	1	821		Domain(s) with predicted 4 iron, 4 sulfur cluster binding, endonuclease activity and sequence-specific DNA binding RNA polymerase II transcription factor activity, more	prot_ID_181	An05g01040	
**An05g01060**	0	0		Putative nonribosomal peptide synthase (NRPS)	**prot_ID_63**	**An05g01060**	ECS
An05g01070	-1	643		Domain(s) with predicted role in response to stress and integral to membrane localization	prot_ID_14	An05g01070	
An05g01080	-2	1761		Ortholog of A. niger CBS 513.88 : An06g00960, A. oryzae RIB40 : AO090003001547, A. brasiliensis : Aspbr1_0186106, Aspergillus flavus NRRL 3357 : AFL2T_01548 and A. clavatus NRRL 1 : ACLA_003370	prot_ID_224	An05g01080	
An05g01090	-3	137		Questionable ORF	prot_ID_126	An05g01090	

An05g01100	-4	1797	Domain(s) with predicted branched-chain-amino-acid transaminase activity and role in branched-chain amino acid metabolic process	prot_ID_221	An05g01100	
An05g01110	-5	855	Domain(s) with predicted ATP binding, ATPase activity, coupled to transmembrane movement of substances activity, role in transmembrane transport and integral to membrane localization	prot_ID_140	An05g01110	
An05g01120	-6	1415	Domain(s) with predicted electron carrier activity, heme binding, iron ion binding, oxidoreductase activity, acting on paired donors, with incorporation or reduction of molecular oxygen activity and role in oxidation-reduction process	prot_ID_155	An05g01120	ECS
n/a			Protein of unknown function	prot_ID_118	An05g01140	

An06g01300 cluster

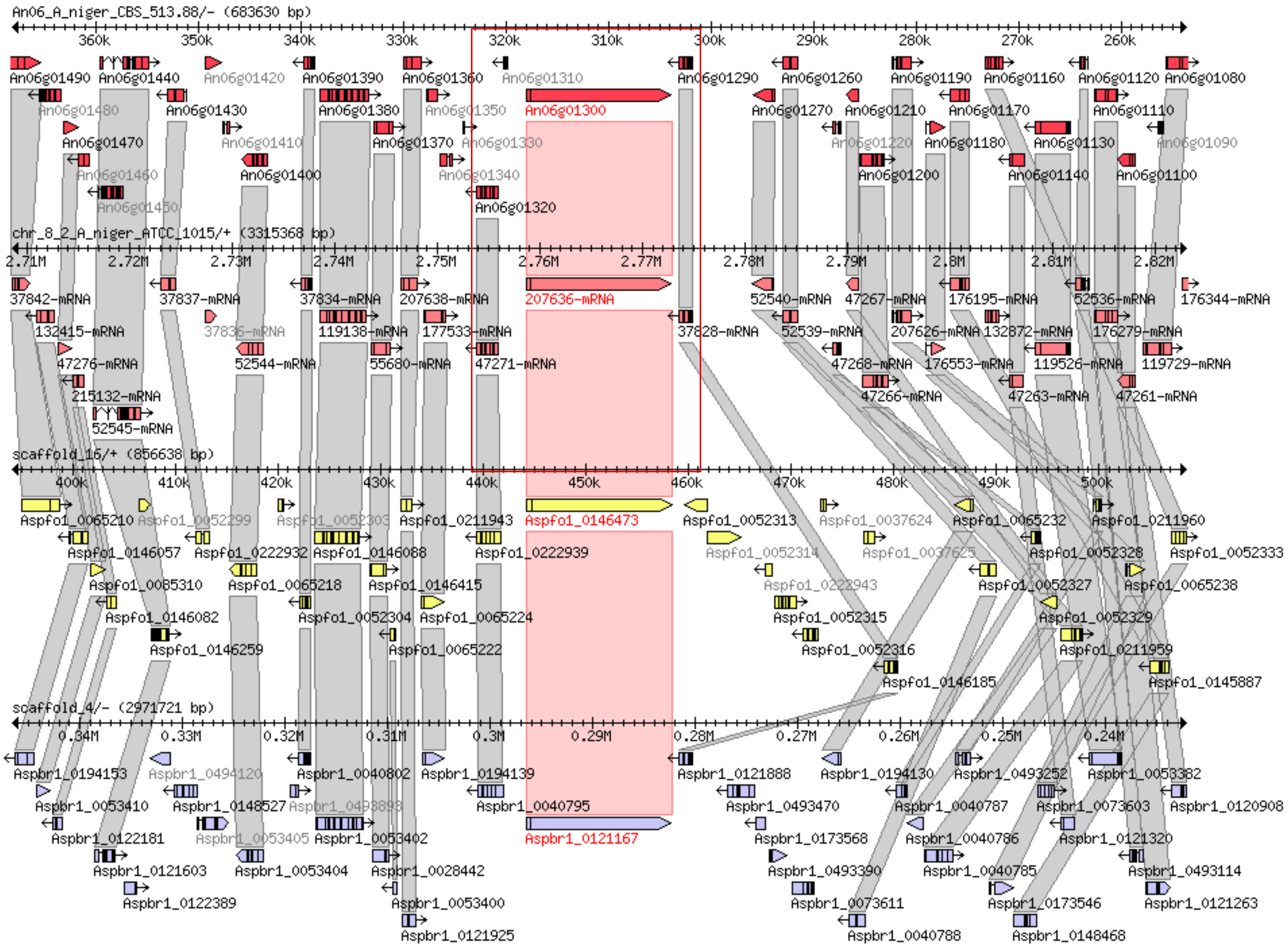

An06g01300 cluster

Gene id (SMURF)	Gene positions	Gene distance	AspGD name	AspGD description line or supplemental annotation	protein ID (antiSMASH)	AspGD ID	
n/a				Conserved endosomal membrane protein; expression enhanced by maltose	prot_ID_220	An06g01200	
n/a				Ortholog of A. nidulans FGSC A4 : AN5340, A. fumigatus Af293 : Afu6g14260/lcp5, A. oryzae RIB40 : AO090103000410, A. niger ATCC 1015 : 47267-mRNA and A. versicolor : Aspve1_0044728	prot_ID_236	An06g01210	
n/a				Protein of unknown function	prot_ID_9	An06g01220	
n/a				Ortholog of A. nidulans FGSC A4 : AN0600, A. fumigatus Af293 : Afu6g10940, A. oryzae RIB40 : AO090023000524, A. niger ATCC 1015 : 52539-mRNA and A. versicolor : Aspve1_0058576	prot_ID_138	An06g01260	
n/a				Protein of unknown function	prot_ID_167	An06g01270	
An06g01290	1	650		Ortholog of A. nidulans FGSC A4 : AN6413, AN8328, AN3395, AN3241, AN10369, AN1930, AN2386, AN1738, AN9266, AN10886, AN7232, AN7406, AN7774, AN12202	prot_ID_213	An06g01290	ECS
**An06g01300**	0	0		Ortholog(s) have role in cellular response to oxidative stress, ferricrocin biosynthetic process, intracellular sequestering of iron ion, iron assimilation	**prot_ID_221**	**An06g01300**	
An06g01310	-1	1836		Protein of unknown function	prot_ID_153	An06g01310	
An06g01320	-2	533		Ortholog(s) have role in N',N'',N'''-triacetylfusarinine C biosynthetic process, cellular response to hydrogen peroxide, cellular response to iron ion starvation and ergosterol biosynthetic process, more	prot_ID_115	An06g01320	ECS
An06g01330	-3	1086		Protein of unknown function	prot_ID_108	An06g01330	
An06g01340	-4	945		Protein of unknown function	prot_ID_223	An06g01340	
An06g01350	-5	103		Protein of unknown function	prot_ID_179	An06g01350	
An06g01360	-6	759		Ortholog of A. nidulans FGSC A4 : AN5668, A. fumigatus Af293 : Afu4g13740, A. oryzae RIB40 : AO090009000197, A. niger ATCC 1015 : 207638-mRNA and A. versicolor : Aspve1_0132964	prot_ID_42	An06g01360	
An06g01370	-7	1237		3-oxoadipate CoA transferase	prot_ID_10	An06g01370	
An06g01380	-8	985		Domain(s) with predicted ubiquitin thiolesterase activity and role in ubiquitin-dependent protein catabolic process	prot_ID_228	An06g01380	
An06g01390	-9	541		NADH-ubiquinone oxidoreductase; respiratory-chain NADH dehydrogenase	prot_ID_80	An06g01390	
An06g01400	-10	3785		Domain(s) with predicted phospholipid binding activity	n/a	An06g01400	
An06g01410	-11	939		Protein of unknown function	n/a	An06g01410	

An07g01030 cluster

No manual prediction made

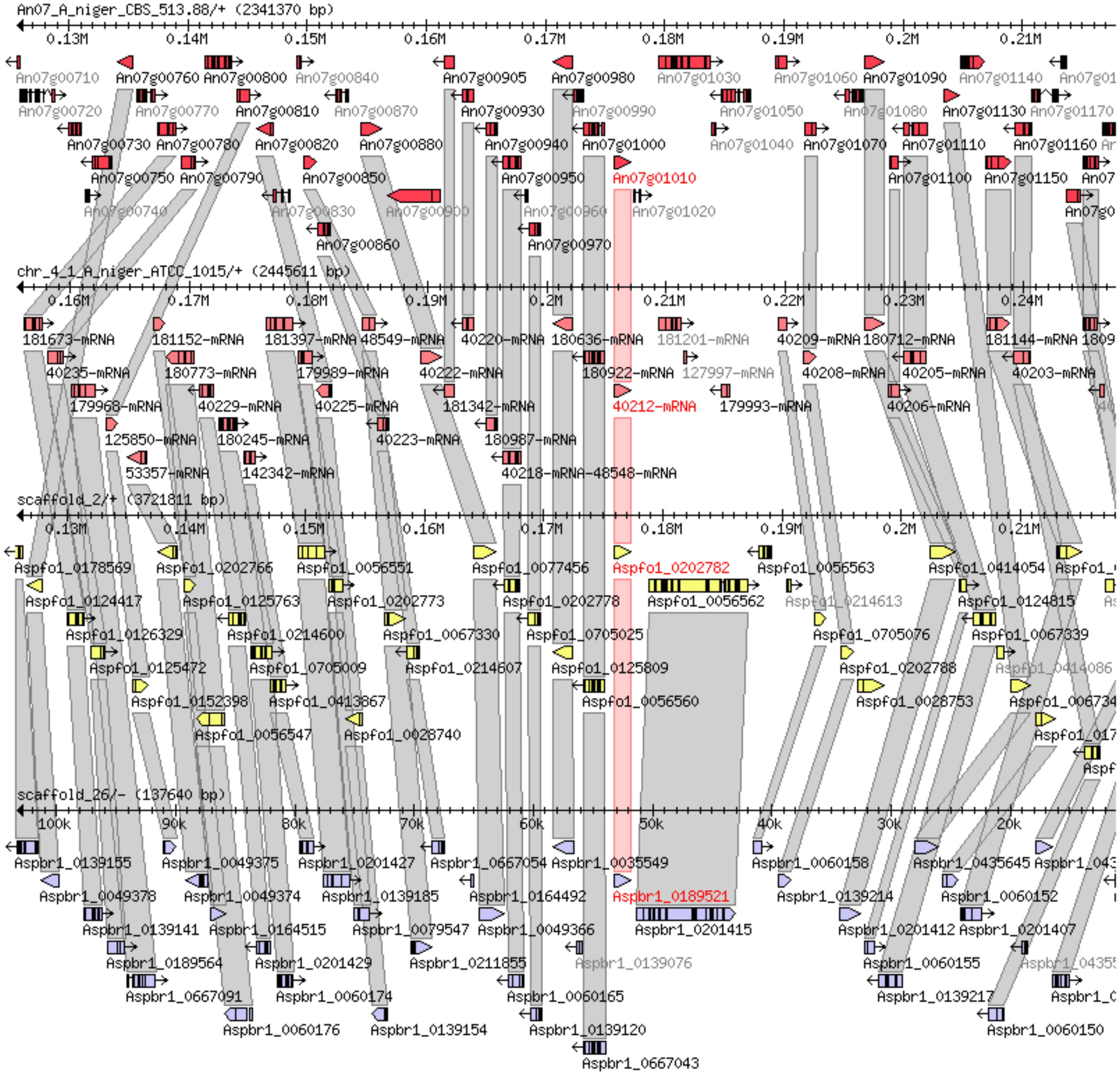

An07g01030 cluster

Gene id (SMURF)	Gene positions	Gene distance	AspGD name	AspGD description line or supplemental annotation	protein ID (antiSMASH)	AspGD ID
An07g00900	12	378		Ortholog(s) have mitochondrion localization	prot_ID_626	An07g00900
An07g00905	11	597		Ortholog of A. nidulans FGSC A4 : AN9483, A. fumigatus Af293 : Afu3g00670, Afu8g01120, A. niger CBS 513.88 : An03g01130, A. oryzae RIB40 : AO090113000089 and A. niger ATCC 1015 : 55131-mRNA	prot_ID_510	An07g00905
An07g00930	10	1036		Domain(s) with predicted hydrolase activity	prot_ID_898	An07g00930
An07g00940	9	603		Ortholog of A. niger ATCC 1015 : 180987-mRNA	prot_ID_524	An07g00940
An07g00950	8	390		Domain(s) with predicted aspartic-type endopeptidase activity and role in proteolysis	prot_ID_261	An07g00950
An07g00960	7	151		Protein of unknown function	prot_ID_885	An07g00960
An07g00970	6	986		Domain(s) with predicted role in response to stress and integral to membrane localization	prot_ID_245	An07g00970
An07g00980	5	30		Domain(s) with predicted role in transmembrane transport and integral to membrane localization	prot_ID_238	An07g00980
An07g00990	4	30		Protein of unknown function	prot_ID_367	An07g00990
An07g01000	3	730		Domain(s) with predicted polygalacturonase activity and role in carbohydrate metabolic process	prot_ID_483	An07g01000
An07g01010	2	253		Domain(s) with predicted sulfuric ester hydrolase activity and role in metabolic process	prot_ID_982	An07g01010
An07g01020	1	1458		Protein of unknown function	prot_ID_317	An07g01020
**An07g01030**	0	0		Domain(s) with predicted transferase activity and role in biosynthetic process	**prot_ID_888**	**An07g01030**
An07g01040	-1	25		Domain(s) with predicted ACP phosphopantetheine attachment site binding involved in fatty acid biosynthetic process activity	prot_ID_969	An07g01040
An07g01050	-2	347		Protein of unknown function	prot_ID_672	An07g01050
An07g01060	-3	2058		Domain(s) with predicted role in cell wall macromolecule catabolic process	prot_ID_361	An07g01060
An07g01070	-4	1422		Domain(s) with predicted ATP binding, protein kinase activity and role in protein phosphorylation	prot_ID_993	An07g01070
An07g01080	-5	2364		Protein of unknown function	prot_ID_557	An07g01080
An07g01090	-6	34		Domain(s) with predicted role in transmembrane transport and integral to membrane localization	prot_ID_74	An07g01090
An07g01100	-7	447		Ortholog of A. nidulans FGSC A4 : AN3299, A. fumigatus Af293 : Afu2g00590, A. oryzae RIB40 : AO090103000485, A. niger ATCC 1015 : 40206-mRNA and A. versicolor :	prot_ID_133	An07g01100

	Aspve1_0027550		
n/a	Domain(s) with predicted transferase activity, transferring hexosyl groups activity	prot_ID_938	An07g01110
n/a	Domain(s) with predicted transferase activity, transferring acyl groups other than amino-acyl groups activity	prot_ID_455	An07g01130

An07g02560 cluster

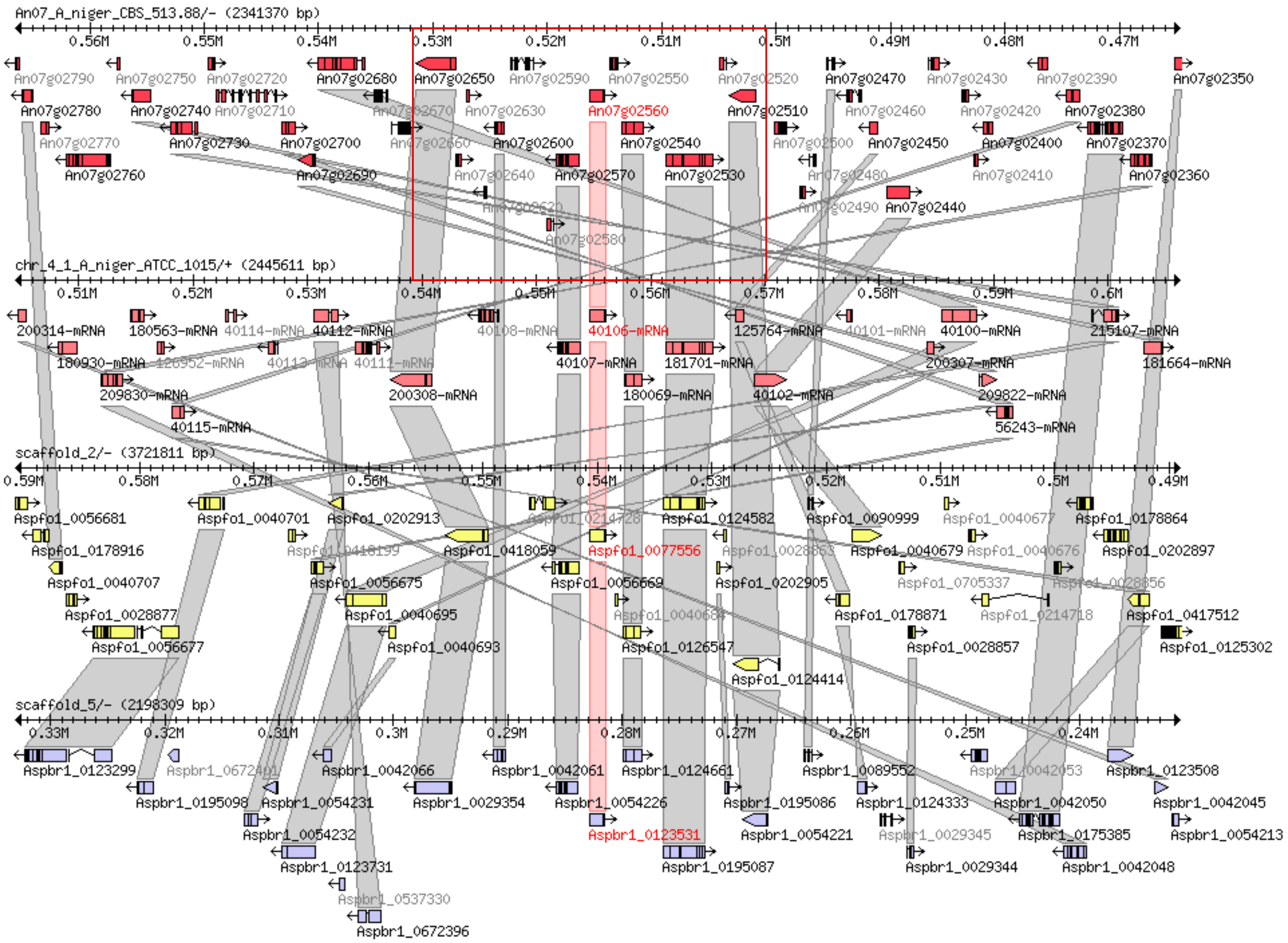

An07g02560 cluster

Gene id (SMURF)	Gene positions	Gene distance	AspGD name	AspGD description line or supplemental annotation	protein ID (antiSMASH)	AspGD ID	
n/a				Protein of unknown function	n/a	An07g02510	ECS
n/a				Protein of unknown function	prot_ID_563	An07g02520	
n/a				Domain(s) with predicted hydrolase activity, acting on ester bonds activity, role in GPI anchor metabolic process, intracellular protein transport and intrinsic to endoplasmic reticulum membrane localization	prot_ID_761	An07g02530	
An07g02540	2	362		Domain(s) with predicted transmembrane transporter activity, role in transmembrane transport and integral to membrane localization	prot_ID_70	An07g02540	
An07g02550	1	356		Weak similarity to DNA dependent ATPase/DNA helicase B	prot_ID_485	An07g02550	
**An07g02560**	0	0		Ortholog(s) have dimethylallyltranstransferase activity, peptidase activity, tryptophan dimethylallyltransferase activity	**prot_ID_651**	**An07g02560**	
An07g02570	-1	954		Domain(s) with predicted electron carrier activity, heme binding, iron ion binding, oxidoreductase activity, acting on paired donors, with incorporation or reduction of molecular oxygen activity and role in oxidation-reduction process	prot_ID_91	An07g02570	
An07g02580	-2	245		Protein of unknown function	prot_ID_75	An07g02580	
An07g02590	-3	1055		Protein of unknown function	prot_ID_837	An07g02590	
An07g02600	-4	649		Ortholog of A. nidulans FGSC A4 : AN6701, A. fumigatus Af293 : Afu7g05650, A. oryzae RIB40 : AO090005000427, A. versicolor : Aspve1_0056558 and A. sydowii : Aspsy1_0034940	prot_ID_998	An07g02600	
An07g02620	-5	694		Protein of unknown function	prot_ID_193	An07g02620	
An07g02630	-6	1189		Protein of unknown function	n/a	An07g02630	
An07g02640	-7	394		Protein of unknown function	n/a	An07g02640	
An07g02650	-8	283		Protein similar to translation elongation factor 3	n/a	An07g02650	ECS

An08g02310 cluster

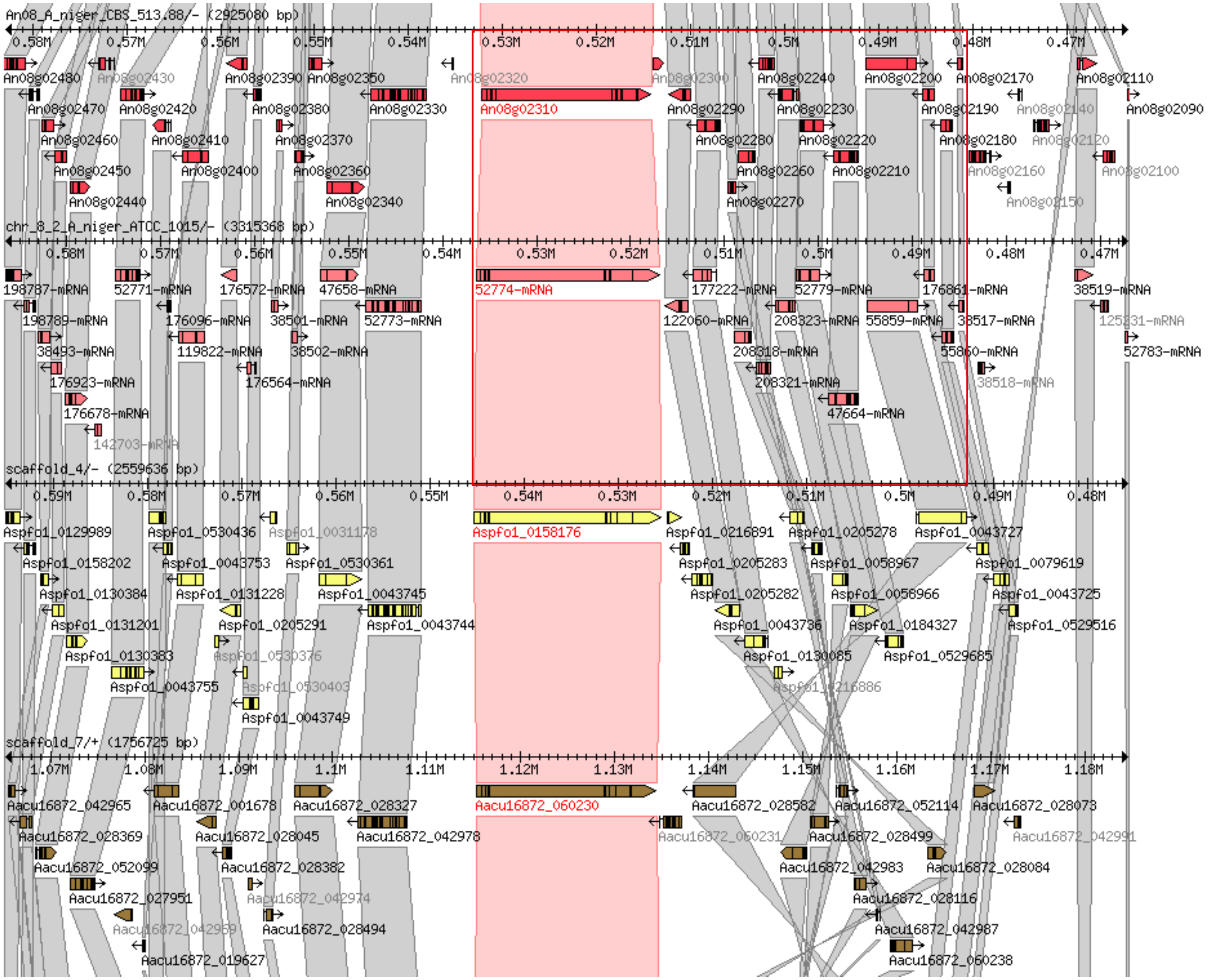

An08g02310 cluster

Gene id (SMURF)	Gene positions	Gene distance	AspGD name	AspGD description line or supplemental annotation	protein ID (antiSMASH)	AspGD ID	
n/a				Protein of unknown funtion	n/a	An08g02170	ECS
An08g02180	12	748		Domain(s) with predicted oxidoreductase activity and role in oxidation-reduction process	n/a	An08g02180	
An08g02190	11	1362		Domain(s) with predicted nucleic acid binding, zinc ion binding activity and intracellular localization	n/a	An08g02190	
An08g02200	10	855		Domain(s) with predicted ATP binding, DNA binding, helicase activity and nucleus localization	n/a	An08g02200	
An08g02210	9	932		Domain(s) with predicted ATP binding, ATPase activity, electron carrier activity, iron-sulfur cluster binding activity	prot_ID_236	An08g02210	
An08g02220	8	345		Ortholog of A. nidulans FGSC A4 : AN1249, A. fumigatus Af293 : Afu1g10320, A. oryzae RIB40 : AO090038000398, A. niger ATCC 1015 : 52779-mRNA and A. versicolor : Aspve1_0119203	prot_ID_341	An08g02220	
An08g02230	7	887		Ortholog of A. fumigatus Af293 : Afu1g10330, A. oryzae RIB40 : AO090038000397, A. niger ATCC 1015 : 208323-mRNA, A. versicolor : Aspve1_0079224 and A. sydowii : Aspsy1_0141143	prot_ID_817	An08g02230	
An08g02240	6	800		Domain(s) with predicted integral to membrane localization	prot_ID_1094	An08g02240	
An08g02260	5	700	pgkA	Phosphoglycerate kinase	prot_ID_1043	An08g02260	
An08g02270	4	929		Ortholog of A. nidulans FGSC A4 : AN1245, A. fumigatus Af293 : Afu1g10355, A. versicolor : Aspve1_0036926, A. sydowii : Aspsy1_0053120 and Aspergillus terreus NIH2624 : ATET_00225	prot_ID_784	An08g02270	
An08g02280	3	920		Ortholog(s) have cytoplasm, nucleus localization	prot_ID_663	An08g02280	
An08g02290	2	1105		Domain(s) with predicted role in transmembrane transport and integral to membrane localization	prot_ID_28	An08g02290	
An08g02300	1	129		Domain(s) with predicted ACP phosphopantetheine attachment site binding involved in fatty acid biosynthetic process activity	prot_ID_1128	An08g02300	
**An08g02310**	0	0		Ortholog(s) have role in cellular response to drug, fumigaclavine C biosynthetic process, fungal-type cell wall organization	**prot_ID_411**	**An08g02310**	ECS
An08g02320	-1	3013		Protein of unknown funtion	prot_ID_881	An08g02320	
An08g02330	-2	2731		Putative ABC multidrug transporter	prot_ID_520	An08g02330	
n/a				Ortholog(s) have nuclear pore, spindle pole body localization	prot_ID_822	An08g02340	

n/a	Ortholog(s) have role in double-strand break repair via homologous recombination, mitotic recombination and nucleus localization	prot_ID_41	An08g02350
n/a	Ortholog of A. nidulans FGSC A4 : AN1236, A. brasiliensis : Aspbr1_0064545, N. fischeri NRRL 181 : NFIA_015250, A. clavatus NRRL 1 : ACLA_025120 and A. acidus : Aspfo1_0530361	prot_ID_1019	An08g02360

An08g03790 cluster

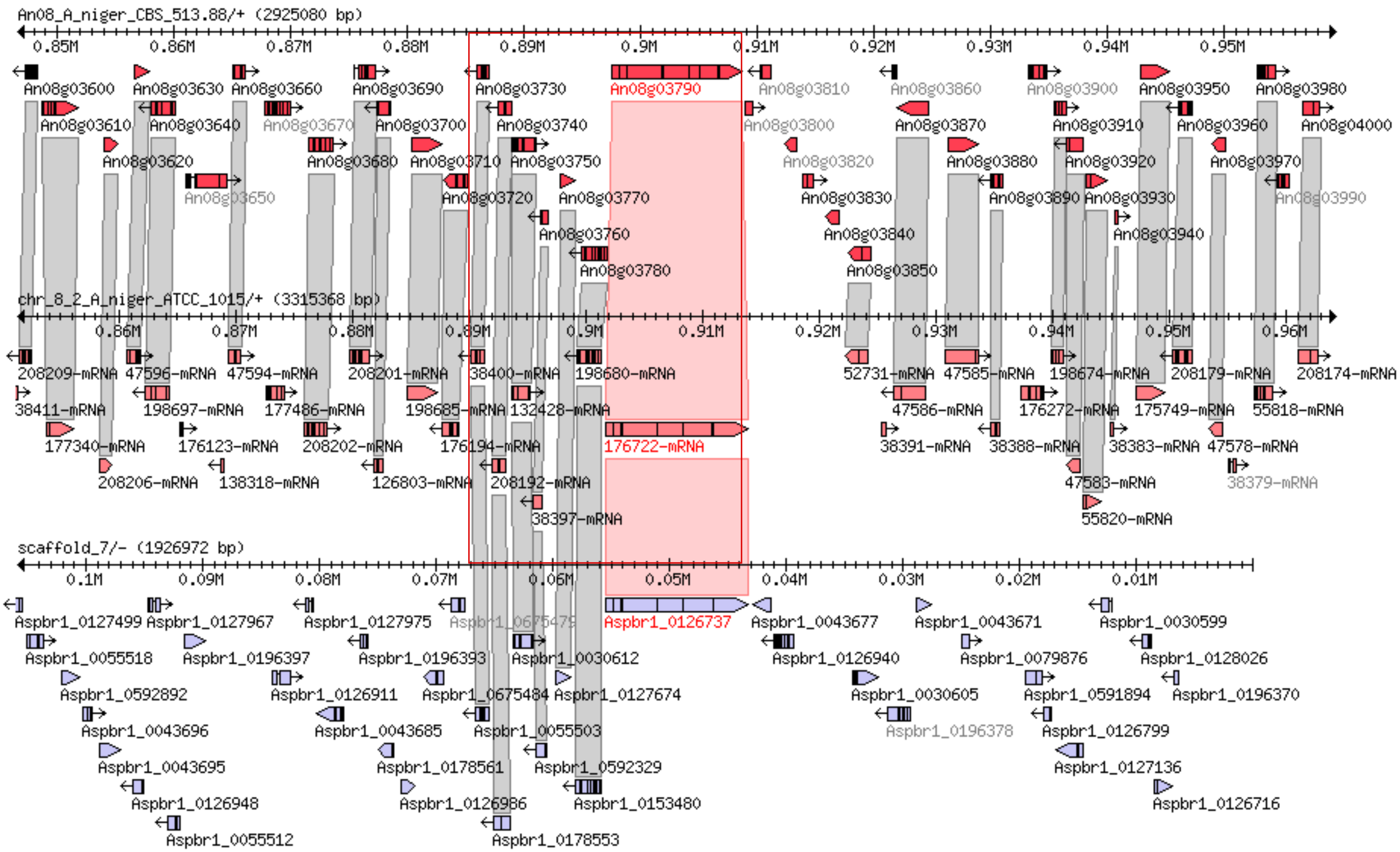

An08g03790 cluster

Gene id (SMURF)	Gene positions	Gene distance	AspGD name	AspGD description line or supplemental annotation	protein ID (antiSMASH)	AspGD ID
An08g03610	18	2328		Domain(s) with predicted hydrolase activity and integral to membrane localization	n/a	An08g03610
An08g03620	17	1497		Ortholog of A. nidulans FGSC A4 : AN1147, A. fumigatus Af293 : Afu1g11500/erg27, A. niger ATCC 1015 : 208206-mRNA, A. versicolor : Aspve1_0049410 and A. sydowii : Aspsy1_0053214	n/a	An08g03620
An08g03630	16	542		Ortholog of A. nidulans FGSC A4 : AN10149, A. fumigatus Af293 : Afu1g11510, A. oryzae RIB40 : AO090038000273, A. niger ATCC 1015 : 47596-mRNA and Aspergillus carbonarius ITEM 5010 : Acar5010_202814	n/a	An08g03630
An08g03640	15	961		Domain(s) with predicted role in transmembrane transport and integral to membrane localization	n/a	An08g03640
An08g03650	14	420		Domain(s) with predicted DNA binding, zinc ion binding activity, role in transcription, DNA-dependent and nucleus localization	n/a	An08g03650
An08g03660	13	1548		Ortholog of A. fumigatus Af293 : Afu1g11540, A. oryzae RIB40 : AO090038000270, A. niger ATCC 1015 : 47594-mRNA, A. versicolor : Aspve1_0119273 and A. sydowii : Aspsy1_0025614	n/a	An08g03660
An08g03670	12	1759		Putative ethanolamine kinase	n/a	An08g03670
An08g03680	11	2133		Ortholog of A. nidulans FGSC A4 : AN1124, A. fumigatus Af293 : Afu1g11720, A. oryzae RIB40 : AO090038000251, A. niger ATCC 1015 : 208202-mRNA and A. versicolor : Aspve1_0288823	n/a	An08g03680
An08g03690	10	517		Putative ADP-ribosylation factor; expression enhanced by maltose	n/a	An08g03690
An08g03700	9	1913		Ortholog of A. nidulans FGSC A4 : AN1127, AN7386, A. fumigatus Af293 : Afu1g11740, A. niger CBS 513.88 : An02g03380, A. oryzae RIB40 : AO090026000507 and A. niger ATCC 1015 : 126803-mRNA	prot_ID_934	An08g03700
An08g03710	8	271		Ortholog of A. nidulans FGSC A4 : AN1128, A. fumigatus Af293 : Afu1g11660, A. oryzae RIB40 : AO090038000255, A. niger ATCC 1015 : 198685-mRNA and A. versicolor : Aspve1_0121473	prot_ID_988	An08g03710
An08g03720	7	794		Domain(s) with predicted DNA binding, zinc ion binding activity, role in transcription, DNA-dependent and nucleus localization	prot_ID_594	An08g03720

An08g03730	6	717	Domain(s) with predicted oxidoreductase activity, acting on paired donors, with incorporation or reduction of molecular oxygen, 2-oxoglutarate as one donor, and incorporation of one atom each of oxygen into both donors activity	prot_ID_36	An08g03730	ECS
An08g03740	5	32	Domain(s) with predicted nucleotide binding, oxidoreductase activity, zinc ion binding activity and role in oxidation-reduction process	prot_ID_605	An08g03740	
An08g03750	4	344	Domain(s) with predicted role in transmembrane transport and integral to membrane localization	prot_ID_67	An08g03750	
An08g03760	3	1083	Protein of unknown function; expression repressed by tunicamycin and DTT	prot_ID_885	An08g03760	
An08g03770	2	595	Domain(s) with predicted sequence-specific DNA binding RNA polymerase II transcription factor activity, sequence-specific DNA binding transcription factor activity, zinc ion binding activity	prot_ID_193	An08g03770	
An08g03780	1	548	Ortholog(s) have catalytic activity and role in sterigmatocystin biosynthetic process	prot_ID_774	An08g03780	
**An08g03790**	0	0	Ortholog(s) have role in pseurotin A biosynthetic process	**prot_ID_426**	**An08g03790**	ECS
An08g03800	-1	197	Domain(s) with predicted nucleotide binding activity	prot_ID_248	An08g03800	
An08g03810	-2	722	Protein of unknown function	prot_ID_518	An08g03810	
An08g03820	-3	1084	Domain(s) with predicted nucleotide binding, oxidoreductase activity, zinc ion binding activity and role in oxidation-reduction process	prot_ID_389	An08g03820	
An08g03830	-4	525	Domain(s) with predicted catalytic activity, coenzyme binding, nucleotide binding activity and role in cellular metabolic process	prot_ID_1137	An08g03830	
An08g03840	-5	948	Ortholog(s) have 3-dehydroshikimate dehydratase activity and role in quinate catabolic process	prot_ID_1100	An08g03840	
An08g03850	-6	856	Ortholog(s) have role in quinate catabolic process	prot_ID_170	An08g03850	
An08g03860	-7	1992	Protein of unknown function	prot_ID_539	An08g03860	
An08g03870	-8	21	Ortholog(s) have sequence-specific DNA binding transcription factor activity and role in positive regulation of transcription from RNA polymerase II promoter, quinate catabolic process	prot_ID_292	An08g03870	
n/a			Ortholog(s) have role in negative regulation of transcription from RNA polymerase II promoter, quinate catabolic process	prot_ID_112	An08g03880	

An08g04820 cluster

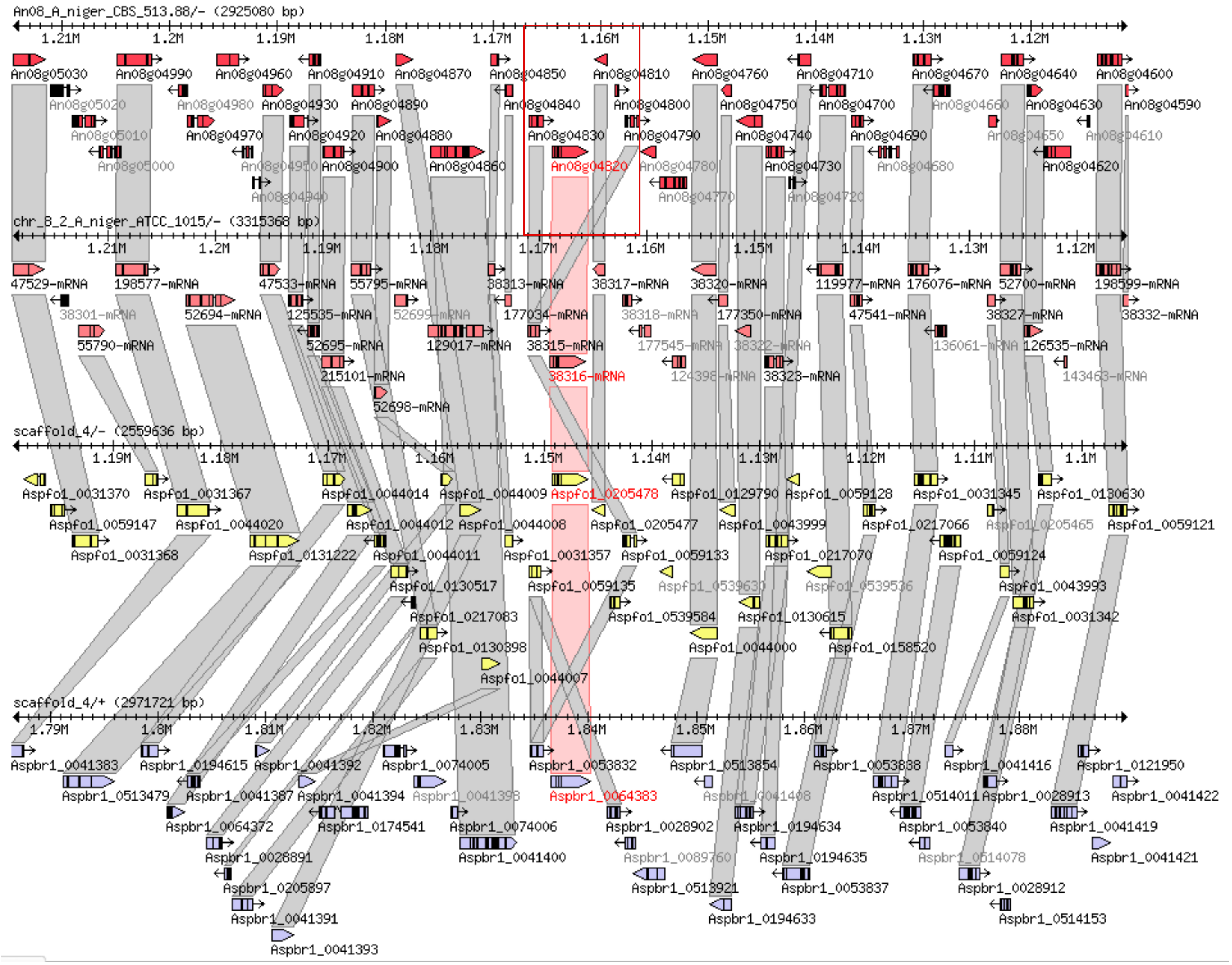

An08g04820 cluster

Gene id (SMURF)	Gene positions	Gene distance	AspGD name	AspGD description line or supplemental annotation	protein ID (antiSMASH)	AspGD ID	
An08g04710	11	139		Ortholog of A. brasiliensis : Aspbr1_0194635	prot_ID_606	An08g04710	
An08g04720	10	421		Protein of unknown function	prot_ID_730	An08g04720	
An08g04730	9	377		Domain(s) with predicted electron carrier activity, oxidoreductase activity and role in oxidation-reduction process	prot_ID_1059	An08g04730	
An08g04740	8	832		Domain(s) with predicted sequence-specific DNA binding RNA polymerase II transcription factor activity, sequence-specific DNA binding transcription factor activity, zinc ion binding activity	prot_ID_1145	An08g04740	
An08g04750	7	276		Domain(s) with predicted proline racemase activity	prot_ID_160	An08g04750	
An08g04760	6	606		Ortholog of A. brasiliensis : Aspbr1_0513854, A. niger ATCC 1015 : 38320-mRNA and A. acidus : Aspfo1_0044000	prot_ID_686	An08g04760	
An08g04770	5	405		Domain(s) with predicted catalytic activity and role in nucleoside metabolic process	prot_ID_557	An08g04770	
An08g04780	4	149		Protein of unknown function	prot_ID_384	An08g04780	
An08g04790	3	560		Ortholog of A. nidulans FGSC A4 : AN6413, AN8328, AN3395, AN3241, AN10369, AN1930, AN2386, AN1738, AN9266, AN10886, AN7232, AN7406, AN7774, AN12202	prot_ID_1149	An08g04790	ECS
An08g04800	2	748		Domain(s) with predicted carbon-sulfur lyase activity and role in metabolic process	prot_ID_154	An08g04800	
An08g04810	1	609		Domain(s) with predicted catalytic activity and role in metabolic process	prot_ID_835	An08g04810	
**An08g04820**	0	0		Protein similar to nonribosomal peptide synthases (NRPS-like)	**prot_ID_1054**	**An08g04820**	
An08g04830	-1	853		Ortholog of A. nidulans FGSC A4 : AN6413, AN8328, AN3395, AN3241, AN10369, AN1930, AN2386, AN1738, AN9266, AN10886, AN7232, AN7406, AN7774, AN12202	prot_ID_943	An08g04830	ECS
An08g04840	-2	1546		Ortholog of A. niger ATCC 1015 : 177034-mRNA	prot_ID_697	An08g04840	
An08g04850	-3	537		Ortholog of A. acidus : Aspfo1_0031357 and A. niger ATCC 1015 : 38313-mRNA	prot_ID_360	An08g04850	
An08g04860	-4	556		Domain(s) with predicted ATP binding, ATPase activity, coupled to transmembrane movement of substances activity, role in transmembrane transport and integral to membrane localization	prot_ID_626	An08g04860	

An08g04870	-5	1590	Ortholog of A. oryzae RIB40 : AO090103000142, A. brasiliensis : Aspbr1_0041393, Aspbr1_0660900, Aspergillus flavus NRRL 3357 : AFL2T_12232 and A. acidus : Aspfo1_0037486, Aspfo1_0044008	prot_ID_217	An08g04870
An08g04880	-6	999	Ortholog of A. nidulans FGSC A4 : AN1066, A. fumigatus Af293 : Afu1g12250, A. oryzae RIB40 : AO090001000304, A. niger ATCC 1015 : 52698-mRNA and A. versicolor : Aspve1_0024252	prot_ID_378	An08g04880
An08g04890	-7	490	Domain(s) with predicted DNA binding activity, role in transcription, DNA-dependent and nucleus localization	prot_ID_910	An08g04890
An08g04900	-8	1118	Ortholog of A. nidulans FGSC A4 : AN1064/nup57, A. fumigatus Af293 : Afu1g12280, A. oryzae RIB40 : AO090001000301, A. niger ATCC 1015 : 215101-mRNA and A. versicolor : Aspve1_0049314	prot_ID_469	An08g04900
An08g04910	-9	632	NADH-ubiquinone oxidoreductase; respiratory-chain NADH dehydrogenase	n/a	An08g04910
An08g04920	-10	281	Domain(s) with predicted fatty-acyl-CoA binding activity	n/a	An08g04920
An08g04930	-11	663	Domain(s) with predicted amino acid transmembrane transporter activity, role in amino acid transmembrane transport and membrane localization	n/a	An08g04930
An08g04940	-12	274	Protein of unknown function	n/a	An08g04940

An08g09220 cluster

No ATCC 1015 orthologous cluster, no manual prediction made

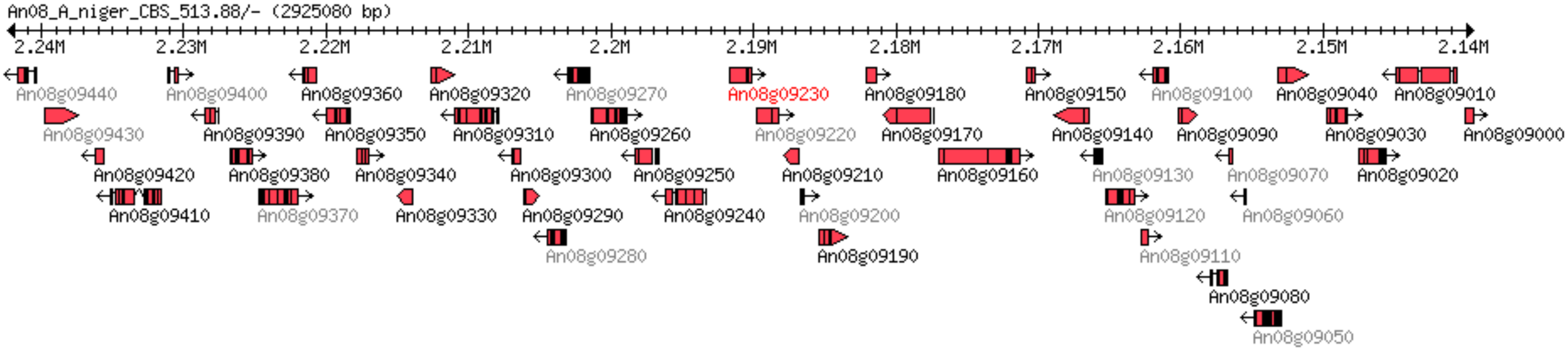

An08g09220 cluster

Gene id (SMURF)	Gene positions	Gene distance	AspGD name	AspGD description line or supplemental annotation	protein ID (antiSMASH)	AspGD ID
n/a				Protein of unknown function	prot_ID_775	An08g09140
An08g09150	7	590		Ortholog of A. acidus : Aspfo1_0130243, A. sydowii : Aspsy1_0041308 and A. niger ATCC 1015 : 38009-mRNA	prot_ID_322	An08g09150
An08g09160	6	277		Ortholog of A. nidulans FGSC A4 : AN5455, A. fumigatus Af293 : Afu6g13370, A. oryzae RIB40 : AO090003000462, A. niger ATCC 1015 : 47358-mRNA and A. versicolor : Aspve1_0132580	prot_ID_1022	An08g09160
An08g09170	5	457		Ortholog of A. nidulans FGSC A4 : AN5456, A. fumigatus Af293 : Afu6g13360, A. oryzae RIB40 : AO090003000461, A. niger ATCC 1015 : 177749-mRNA and A. versicolor : Aspve1_0169365	prot_ID_233	An08g09170
An08g09180	4	1252		Ortholog of A. oryzae RIB40 : AO090023000473, A. versicolor : Aspve1_0045297, A. sydowii : Aspsy1_0046488 and A. niger ATCC 1015 : 38006-mRNA	prot_ID_845	An08g09180
An08g09190	3	995		Ortholog(s) have monooxygenase activity and role in sterigmatocystin biosynthetic process	prot_ID_48	An08g09190
An08g09200	2	78		Protein of unknown function	prot_ID_703	An08g09200
An08g09210	1	268		Domain(s) with predicted isomerase activity, transferase activity and role in queuosine biosynthetic process	prot_ID_402	An08g09210
**An08g09220**	0	0		Protein similar to nonribosomal peptide synthases (NRPS-like)	**prot_ID_12**	**An08g09220**
An08g09230	-1	301		Ortholog(s) have catalytic activity and role in sterigmatocystin biosynthetic process	prot_ID_541	An08g09230
An08g09240	-2	1591		Domain(s) with predicted DNA binding, sequence-specific DNA binding RNA polymerase II transcription factor activity, sequence-specific DNA binding transcription factor activity, zinc ion binding activity	prot_ID_668	An08g09240
An08g09250	-3	499		Domain(s) with predicted role in transmembrane transport and integral to membrane localization	prot_ID_1122	An08g09250
An08g09260	-4	496		Domain(s) with predicted acetoacetate-CoA ligase activity and role in lipid metabolic process	prot_ID_1160	An08g09260
An08g09270	-5	123		Protein of unknown function	prot_ID_932	An08g09270
An08g09280	-6	66		Domain(s) with predicted catalytic activity and role in metabolic process	prot_ID_1168	An08g09280
An08g09290	-7	603		Domain(s) with predicted hydrolase activity, acting on carbon-nitrogen (but not peptide) bonds activity and role in carbohydrate metabolic process	prot_ID_420	An08g09290
An08g09300	-8	225		Lactoylglutathione lyase (glyoxylase I)	prot_ID_492	An08g09300

An08g09310	-9	855	Domain(s) with predicted DNA binding, zinc ion binding activity, role in transcription, DNA-dependent and nucleus localization	prot_ID_459	An08g09310
An08g09320	-10	67	Domain(s) with predicted role in transmembrane transport and integral to membrane localization	prot_ID_4	An08g09320
An08g09330	-11	1268	Ortholog(s) have role in ergot alkaloid biosynthetic process, secondary metabolic process	prot_ID_1010	An08g09330
An08g09340	-12	1832	Ortholog(s) have intracellular localization	prot_ID_873	An08g09340
An08g09350	-13	520	Domain(s) with predicted substrate-specific transmembrane transporter activity, role in transmembrane transport and integral to membrane localization	prot_ID_778	An08g09350
An08g09360	-14	678	Domain(s) with predicted nucleotide binding, oxidoreductase activity and role in oxidation-reduction process	prot_ID_19	An08g09360
An08g09370	-15	219	Domain(s) with predicted DNA binding, sequence-specific DNA binding RNA polymerase II transcription factor activity, sequence-specific DNA binding transcription factor activity, zinc ion binding activity	n/a	An08g09370
An08g09380	-16	453	D-xylulose reductase (xylitol dehydrogenase); sorbitol (glucitol) dehydrogenase (NAD+)	n/a	An08g09380
An08g09390	-17	840	Domain(s) with predicted ATP binding, kinase activity and role in metabolic process	n/a	An08g09390
An08g09400	-18	1766	Protein of unknown function	n/a	An08g09400

An08g10930 cluster

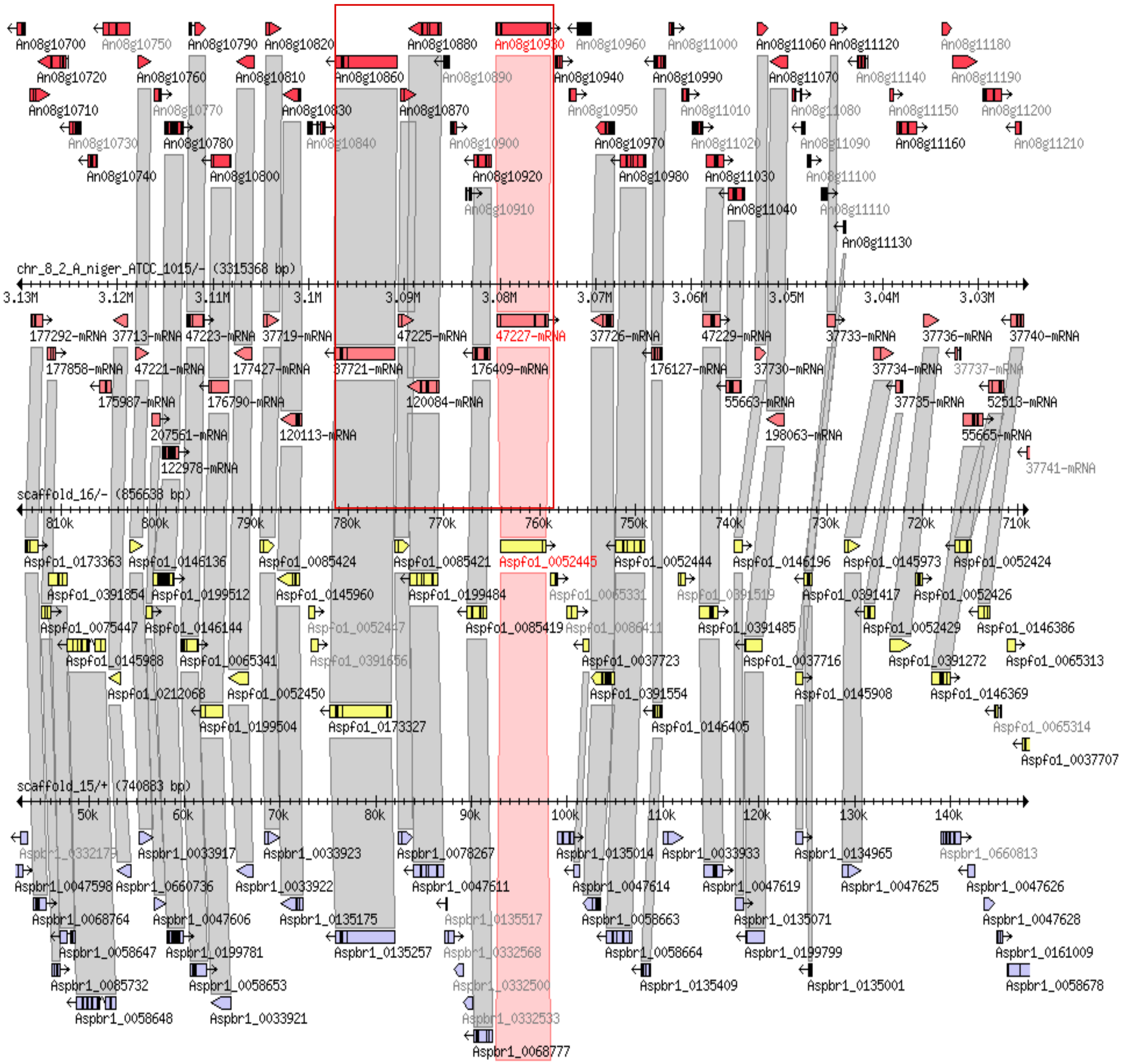

An08g10930 cluster

Gene id (SMURF)	Gene positions	Gene distance	AspGD name	AspGD description line or supplemental annotation	protein ID (antiSMASH)	AspGD ID	
n/a				Putative fatty acid synthase, beta subunit	n/a	An08g10860	ECS
n/a				Has domain(s) with predicted 2 iron, 2 sulfur cluster binding, 2-methylcitrate dehydratase activity and role in propionate catabolic process, 2-methylcitrate cycle	n/a	An08g10870	
An08g10880	5	367		Domain(s) with predicted DNA binding, sequence-specific DNA binding RNA polymerase II transcription factor activity, sequence-specific DNA binding transcription factor activity, zinc ion binding activity	n/a	An08g10880	
An08g10890	4	58		Protein of unknown function	n/a	An08g10890	
An08g10900	3	817		Protein of unknown function	n/a	An08g10900	
An08g10910	2	116		Protein of unknown function	n/a	An08g10910	
An08g10920	1	445		Putative citrate synthase	n/a	An08g10920	
**An08g10930**	0	0		Fatty-acid synthase	n/a	**An08g10930**	ECS
An08g10940	-1	580		Ortholog of A. nidulans FGSC A4 : AN3344/ngn27, A. fumigatus Af293 : Afu5g00720, A. niger CBS 513.88 : An07g02400, An18g00950, A. oryzae RIB40 : AO090038000013 and A. niger ATCC 1015 : 54083-mRNA	n/a	An08g10940	
An08g10950	-2	605			n/a	An08g10950	
An08g10960	-3	85			n/a	An08g10960	
An08g10970	-4	546		Domain(s) with predicted role in transmembrane transport and integral to membrane localization	n/a	An08g10970	
An08g10980	-5	715		Domain(s) with predicted DNA binding, sequence-specific DNA binding RNA polymerase II transcription factor activity, sequence-specific DNA binding transcription factor activity, zinc ion binding activity	n/a	An08g10980	
An08g10990	-6	841		Protein with similarity to dienelactone hydrolase	n/a	An08g10990	
An08g11000	-7	445		Domain(s) with predicted sequence-specific DNA binding RNA polymerase II transcription factor activity, sequence-specific DNA binding transcription factor activity, zinc ion binding activity	n/a	An08g11000	

An09g00450 cluster

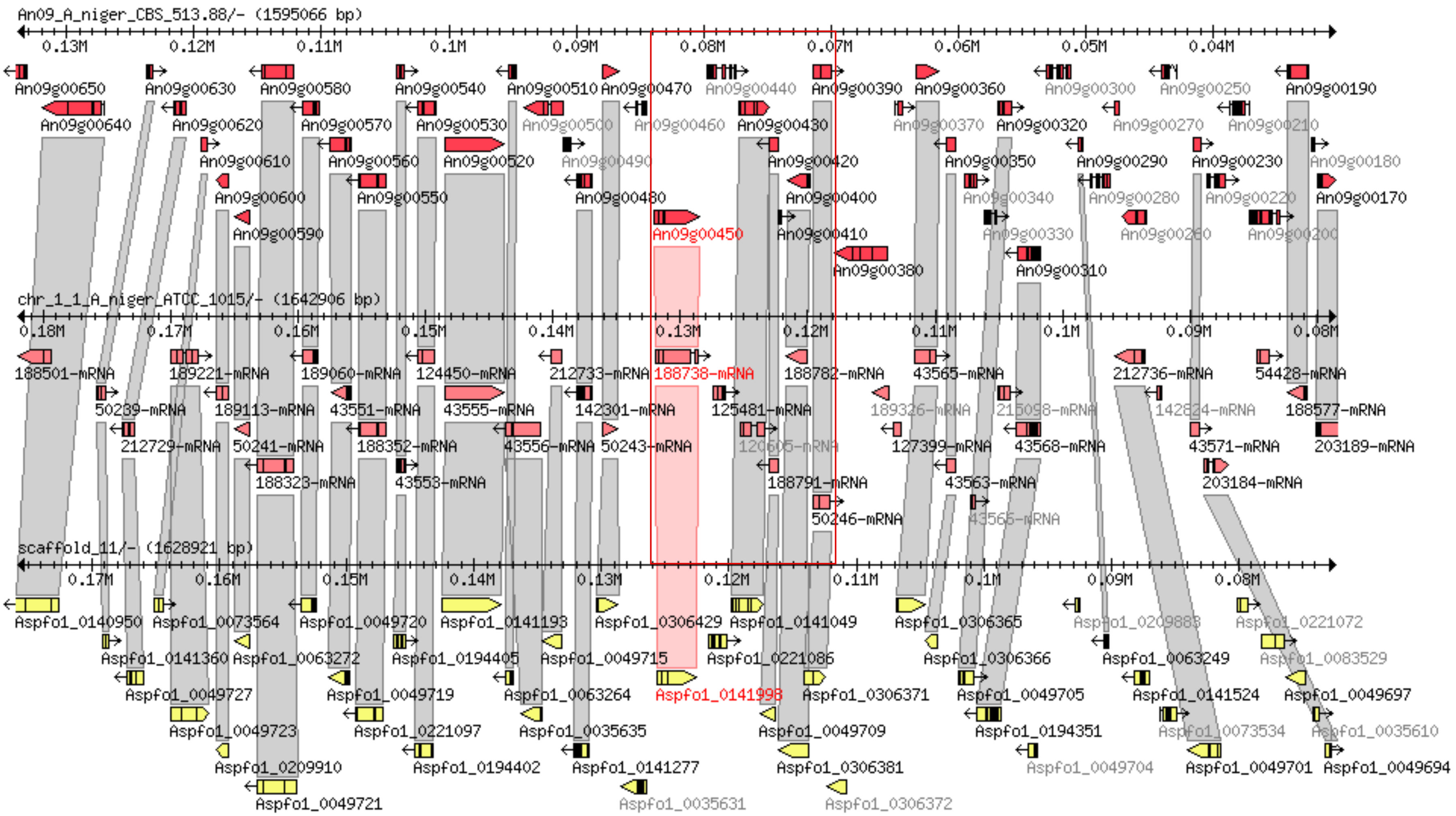

An09g00450 cluster

Gene id (SMURF)	Gene positions	Gene distance	AspGD name	AspGD description line or supplemental annotation	protein ID (antiSMASH)	AspGD ID	
n/a				Ortholog of A. brasiliensis : Aspbr1_0049056, A. acidus : Aspfo1_0306366, A. niger ATCC 1015 : 43563-mRNA and Aspergillus carbonarius ITEM 5010 : Acar5010_131599, Acar5010_131666	prot_ID_309	An09g00350	
n/a				Ortholog of A. nidulans FGSC A4 : AN7985, A. fumigatus Af293 : Afu6g03240, A. oryzae RIB40 : AO090103000145, A. niger ATCC 1015 : 43565-mRNA and A. versicolor : Aspve1_0032365, Aspve1_0072790	prot_ID_414	An09g00360	
n/a				Protein of unknown function	prot_ID_330	An09g00370	
n/a				Domain(s) with predicted ATP binding, protein serine/threonine kinase activity and role in protein phosphorylation	prot_ID_203	An09g00380	
n/a				Domain(s) with predicted oxidoreductase activity and role in oxidation-reduction process	prot_ID_146	An09g00390	ECS
An09g00400	5	277		Domain(s) with predicted amino acid transmembrane transporter activity, role in amino acid transmembrane transport and membrane localization	prot_ID_363	An09g00400	
An09g00410	4	21		Ortholog of A. brasiliensis : Aspbr1_0138124	prot_ID_290	An09g00410	
An09g00420	3	67		Domain(s) with predicted catalytic activity and role in metabolic process	prot_ID_294	An09g00420	
An09g00430	2	144		Domain(s) with predicted DNA binding, sequence-specific DNA binding RNA polymerase II transcription factor activity, sequence-specific DNA binding transcription factor activity, zinc ion binding activity	prot_ID_390	An09g00430	
An09g00440	1	600		Protein of unknown function	prot_ID_213	An09g00440	
**An09g00450**	0	0		Protein similar to nonribosomal peptide synthases (NRPS-like)	**prot_ID_394**	**An09g00450**	ECS
An09g00460	-1	630		Protein of unknown function	prot_ID_429	An09g00460	
An09g00470	-2	1313		Ortholog of A. brasiliensis : Aspbr1_0201039, A. acidus : Aspfo1_0306429, A. versicolor : Aspve1_0363489 and A. niger ATCC 1015 : 50243-mRNA	prot_ID_76	An09g00470	
An09g00480	-3	855		Domain(s) with predicted nucleotide binding activity	prot_ID_10	An09g00480	
An09g00490	-4	372		Protein of unknown function	prot_ID_153	An09g00490	
An09g00500	-5	106		Domain(s) with predicted sequence-specific DNA binding RNA polymerase II transcription factor activity, sequence-specific DNA binding transcription factor activity, zinc ion binding activity	prot_ID_222	An09g00500	

An09g00510	-6	542	Ortholog of A. nidulans FGSC A4 : AN5396, A. niger CBS 513.88 : An01g01630, A. brasiliensis : Aspbr1_0040534, Aspbr1_0188798 and A. niger ATCC 1015 : 52059-mRNA	prot_ID_655	An09g00510
An09g00520	-7	347	Domain(s) with predicted ACP phosphopantetheine attachment site binding involved in fatty acid biosynthetic process, catalytic activity, phosphopantetheine binding activity	prot_ID_372	An09g00520
An09g00530	-8	721	Domain(s) with predicted monooxygenase activity and role in oxidation-reduction process	prot_ID_631	An09g00530
An09g00540	-9	866	Ortholog of A. brasiliensis : Aspbr1_0201030, A. niger ATCC 1015 : 43553-mRNA and A. acidus : Aspfo1_0194405	prot_ID_127	An09g00540
An09g00550	-10	820	Domain(s) with predicted role in transmembrane transport and integral to membrane localization	prot_ID_496	An09g00550
An09g00560	-11	559	Ortholog(s) have role in gliotoxin biosynthetic process	prot_ID_61	An09g00560
An09g00570	-12	1003	Domain(s) with predicted catalytic activity, coenzyme binding, nucleotide binding activity and role in cellular metabolic process	prot_ID_543	An09g00570
An09g00580	-13	994	Domain(s) with predicted DNA binding, sequence-specific DNA binding RNA polymerase II transcription factor activity, sequence-specific DNA binding transcription factor activity, zinc ion binding activity	prot_ID_570	An09g00580
An09g00590	-14	935	Putative salicylate hydroxylase	prot_ID_295	An09g00590
An09g00600	-15	483	Domain(s) with predicted nucleotide binding activity	prot_ID_47	An09g00600
An09g00610	-16	720	Ortholog of A. niger CBS 513.88 : An08g08120, An03g00900, A. oryzae RIB40 : AO090010000695, A. brasiliensis : Aspbr1_0194369, Aspbr1_0196393 and A. niger ATCC 1015 : 45778-mRNA	prot_ID_637	An09g00610

An09g00520 cluster

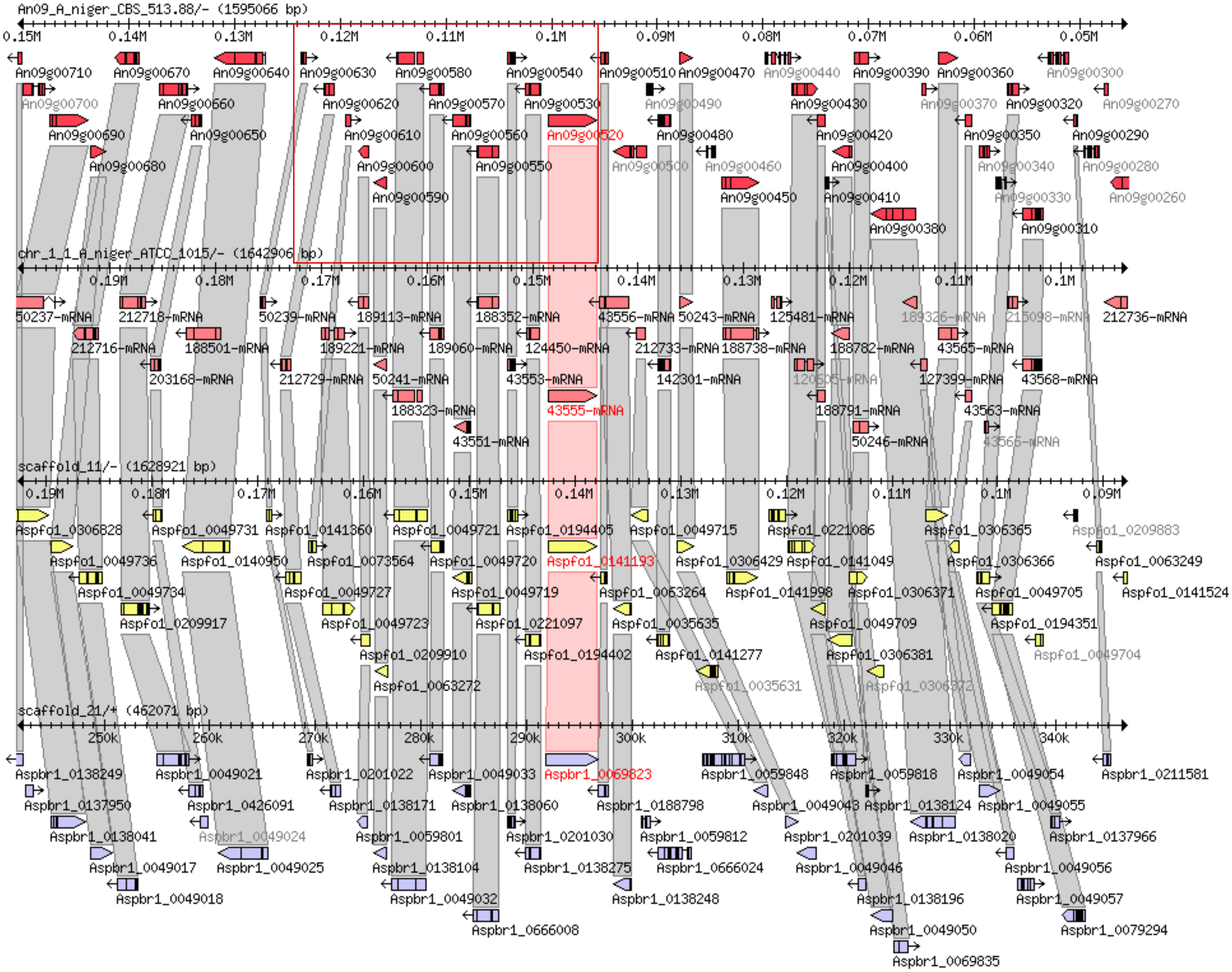

An09g00520 cluster

Gene id (SMURF)	Gene positions	Gene distance	AspGD name	AspGD description line or supplemental annotation	protein ID (antiSMASH)	AspGD ID	
An09g00390	13	236		Domain(s) with predicted oxidoreductase activity and role in oxidation-reduction process	n/a	An09g00390	
An09g00400	12	277		Domain(s) with predicted amino acid transmembrane transporter activity, role in amino acid transmembrane transport and membrane localization	n/a	An09g00400	
An09g00410	11	21		Ortholog of A. brasiliensis : Aspbr1_0138124	n/a	An09g00410	
An09g00420	10	67		Domain(s) with predicted catalytic activity and role in metabolic process	n/a	An09g00420	
An09g00430	9	144		Domain(s) with predicted DNA binding, sequence-specific DNA binding RNA polymerase II transcription factor activity, sequence-specific DNA binding transcription factor activity, zinc ion binding activity	n/a	An09g00430	
An09g00440	8	600			n/a	An09g00440	
An09g00450	7	630		Domain(s) with predicted ACP phosphopantetheine attachment site binding involved in fatty acid biosynthetic process, catalytic activity, nucleotide binding activity	n/a	An09g00450	
An09g00460	6	1313			n/a	An09g00460	
An09g00470	5	855		Ortholog of A. brasiliensis : Aspbr1_0201039, A. acidus : Aspfo1_0306429, A. versicolor : Aspve1_0363489 and A. niger ATCC 1015 : 50243-mRNA	n/a	An09g00470	
An09g00480	4	372		Domain(s) with predicted nucleotide binding activity	n/a	An09g00480	
An09g00490	3	106			n/a	An09g00490	
An09g00500	2	542		Domain(s) with predicted sequence-specific DNA binding RNA polymerase II transcription factor activity, sequence-specific DNA binding transcription factor activity, zinc ion binding activity	n/a	An09g00500	
An09g00510	1	347		Ortholog of A. nidulans FGSC A4 : AN5396, A. niger CBS 513.88 : An01g01630, A. brasiliensis : Aspbr1_0040534, Aspbr1_0188798 and A. niger ATCC 1015 : 52059-mRNA	n/a	An09g00510	
**An09g00520**	0	0		Putative nonribosomal peptide synthase (NRPS)	n/a	**An09g00520**	ECS
An09g00530	-1	721		Domain(s) with predicted monooxygenase activity and role in oxidation-reduction process	n/a	An09g00530	
An09g00540	-2	866		Ortholog of A. brasiliensis : Aspbr1_0201030, A. niger ATCC 1015 : 43553-mRNA and A. acidus : Aspfo1_0194405	n/a	An09g00540	
An09g00550	-3	820		Domain(s) with predicted role in transmembrane transport and integral to membrane localization	n/a	An09g00550	

An09g00560	-4	559		Ortholog(s) have role in gliotoxin biosynthetic process	n/a	An09g00560	
An09g00570	-5	1003		Domain(s) with predicted catalytic activity, coenzyme binding, nucleotide binding activity and role in cellular metabolic process	n/a	An09g00570	
An09g00580	-6	994		Domain(s) with predicted DNA binding, sequence-specific DNA binding RNA polymerase II transcription factor activity, sequence-specific DNA binding transcription factor activity, zinc ion binding activity	n/a	An09g00580	
An09g00590	-7	935		Putative salicylate hydroxylase	n/a	An09g00590	
An09g00600	-8	483		Domain(s) with predicted nucleotide binding activity	n/a	An09g00600	
An09g00610	-9	720		Ortholog of A. niger CBS 513.88 : An08g08120, An03g00900, A. oryzae RIB40 : AO090010000695, A. brasiliensis : Aspbr1_0194369, Aspbr1_0196393 and A. niger ATCC 1015 : 45778-mRNA	n/a	An09g00610	
An09g00620	-10	1092		Domain(s) with predicted nucleotide binding, oxidoreductase activity and role in oxidation-reduction process	n/a	An09g00620	ECS
n/a			cipC	Protein of unknown function	n/a	An09g00630	

An09g01290 cluster

No ATCC 1015 ortholog

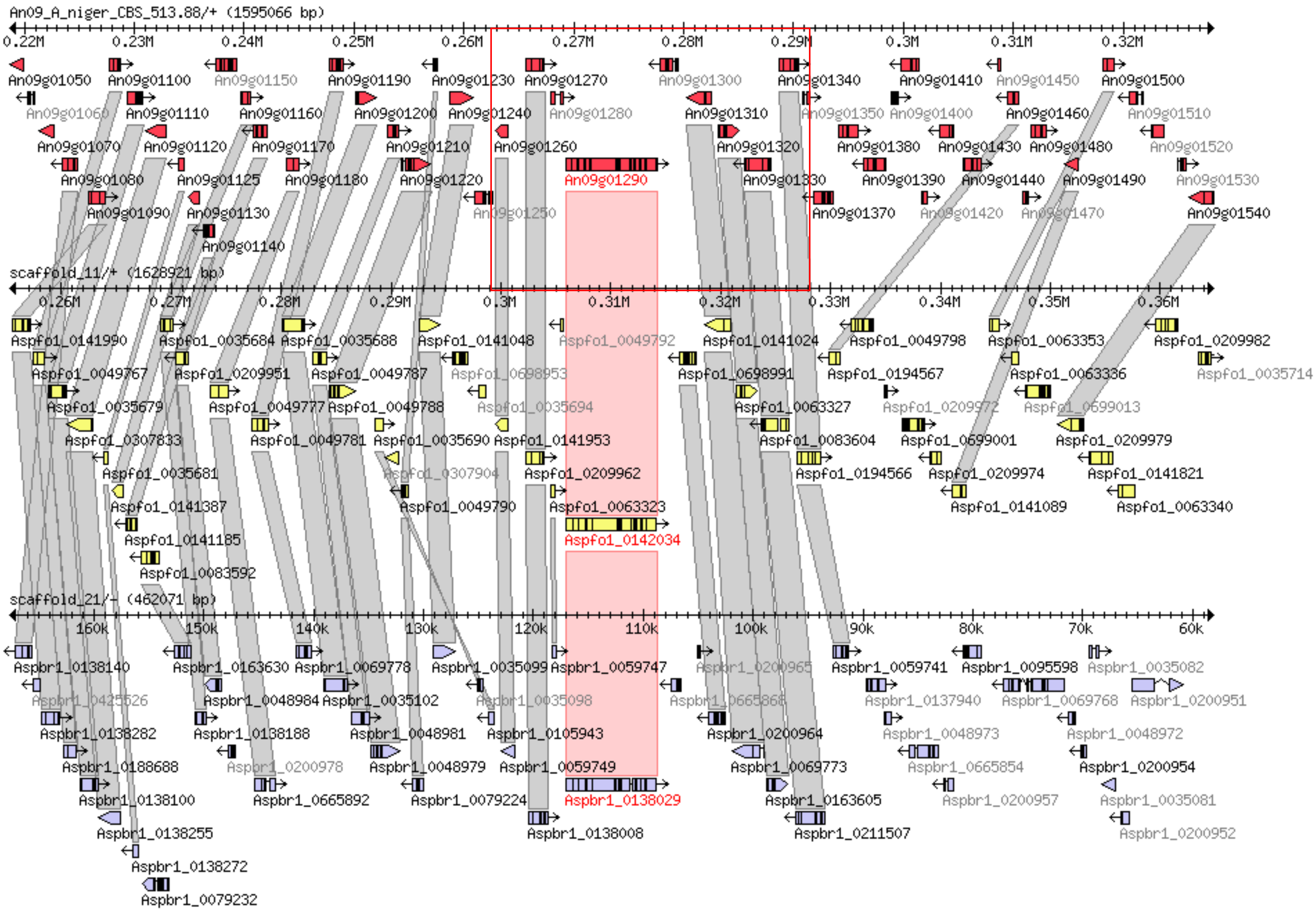

An09g01290 cluster

Gene id (SMURF)	Gene positions	Gene distance	AspGD name	AspGD description line or supplemental annotation	protein ID (antiSMASH)	AspGD ID	
An09g01140	15	114		Domain(s) with predicted hydrolase activity	n/a	An09g01140	
An09g01150	14	632		Domain(s) with predicted nucleotide binding, oxidoreductase activity and role in oxidation-reduction process	n/a	An09g01150	
An09g01160	13	179		Ortholog of A. nidulans FGSC A4 : AN5207, A. fumigatus Af293 : Afu6g07400, A. oryzae RIB40 : AO090005001560, A. niger ATCC 1015 : 125791-mRNA and A. versicolor : Aspve1_0045658	n/a	An09g01160	
An09g01170	12	1825		Domain(s) with predicted oxidoreductase activity, acting on paired donors, with incorporation or reduction of molecular oxygen, 2-oxoglutarate as one donor, and incorporation of one atom each of oxygen into both donors activity	n/a	An09g01170	
An09g01180	11	2729		Ortholog of A. versicolor : Aspve1_0032369, A. sydowii : Aspsy1_0156280, Aspergillus terreus NIH2624 : ATET_04345 and A. niger ATCC 1015 : 43506-mRNA	n/a	An09g01180	
An09g01190	10	1264	abnA	Endo-l,5-alpha-L-arabinase, secreted enzyme involved in degradation of hemicellulosic component L-araban	n/a	An09g01190	
An09g01200	9	1054		Domain(s) with predicted sequence-specific DNA binding RNA polymerase II transcription factor activity, sequence-specific DNA binding transcription factor activity, zinc ion binding activity	prot_ID_379	An09g01200	
An09g01210	8	209		Domain(s) with predicted nucleotide binding, oxidoreductase activity and role in oxidation-reduction process	prot_ID_611	An09g01210	
An09g01220	7	94		Ortholog of A. nidulans FGSC A4 : AN2326, A. fumigatus Af293 : Afu3g14650, A. niger ATCC 1015 : 188883-mRNA, A. versicolor : Aspve1_0050509 and A. sydowii : Aspsy1_0084810	prot_ID_308	An09g01220	
An09g01230	6	1116		Ortholog of A. nidulans FGSC A4 : AN2535, A. fumigatus Af293 : Afu3g14660, A. versicolor : Aspve1_0081353 and A. sydowii : Aspsy1_0087029	prot_ID_135	An09g01230	
An09g01240	5	372		Domain(s) with predicted phospholipase activity and role in phospholipid catabolic process	prot_ID_614	An09g01240	
An09g01250	4	266		Protein of unknown function	prot_ID_679	An09g01250	
An09g01260	3	1633		Domain(s) with predicted nucleotide binding activity	prot_ID_72	An09g01260	ECS
An09g01270	2	504		Domain(s) with predicted electron carrier activity, heme binding, iron ion binding, oxidoreductase activity, acting on paired donors and with incorporation or reduction of molecular oxygen, more	prot_ID_417	An09g01270	

An09g01280	1	271	Questionable ORF	prot_ID_67	An09g01280	
**An09g01290**	0	0	Putative polyketide synthase (PKS)	**prot_ID_4**	**An09g01290**	
An09g01300	-1	218	Protein of unknown function	prot_ID_190	An09g01300	
An09g01310	-2	814	Domain(s) with predicted sequence-specific DNA binding RNA polymerase II transcription factor activity, sequence-specific DNA binding transcription factor activity, zinc ion binding activity	prot_ID_602	An09g01310	
An09g01320	-3	595	Domain(s) with predicted UDP-N-acetylmuramate dehydrogenase activity, flavin adenine dinucleotide binding activity and role in oxidation-reduction process	prot_ID_207	An09g01320	
An09g01330	-4	366	2,3-oxidosqualene-lanosterol cyclase	prot_ID_278	An09g01330	
An09g01340	-5	744	Putative leukotriene B(4) omega-hydroxylase cytochrome P450	prot_ID_628	An09g01340	ECS
An09g01350	-6	458	Protein of unknown function	prot_ID_537	An09g01350	
An09g01370	-7	439	Ortholog of A. nidulans FGSC A4 : AN1643, AN1704, AN7062, A. fumigatus Af293 : Afu2g14410, Afu4g01500 and A. niger CBS 513.88 : An18g01050, An04g02710	prot_ID_553	An09g01370	
An09g01380	-8	568	Domain(s) with predicted UDP-N-acetylmuramate dehydrogenase activity, flavin adenine dinucleotide binding activity and role in oxidation-reduction process	prot_ID_82	An09g01380	
An09g01390	-9	274	Domain(s) with predicted DNA binding, zinc ion binding activity, role in transcription, DNA-dependent and nucleus localization	prot_ID_39	An09g01390	
An09g01400	-10	537		n/a	An09g01400	
An09g01410	-11	118	Domain(s) with predicted electron carrier activity, heme binding, iron ion binding, oxidoreductase activity, acting on paired donors, with incorporation or reduction of molecular oxygen activity and role in oxidation-reduction process	n/a	An09g01410	
An09g01420	-12	213	Domain(s) with predicted cell outer membrane, integral to membrane localization	n/a	An09g01420	
An09g01430	-13	968	Domain(s) with predicted nucleotide binding activity	n/a	An09g01430	
An09g01440	-14	853	Domain(s) with predicted oxidoreductase activity and role in oxidation-reduction process	n/a	An09g01440	
An09g01450	-15	1553		n/a	An09g01450	
An09g01460	-16	599	Ortholog of A. nidulans FGSC A4 : AN8462, A. niger CBS 513.88 : An03g03400, A. brasiliensis : Aspbr1_0131393, A. niger ATCC 1015 : 43481-mRNA, 44376-mRNA and A. acidus : Aspfo1_0194567	n/a	An09g01460	

An09g01690 cluster

NO ATCC 1015 orthologous cluster

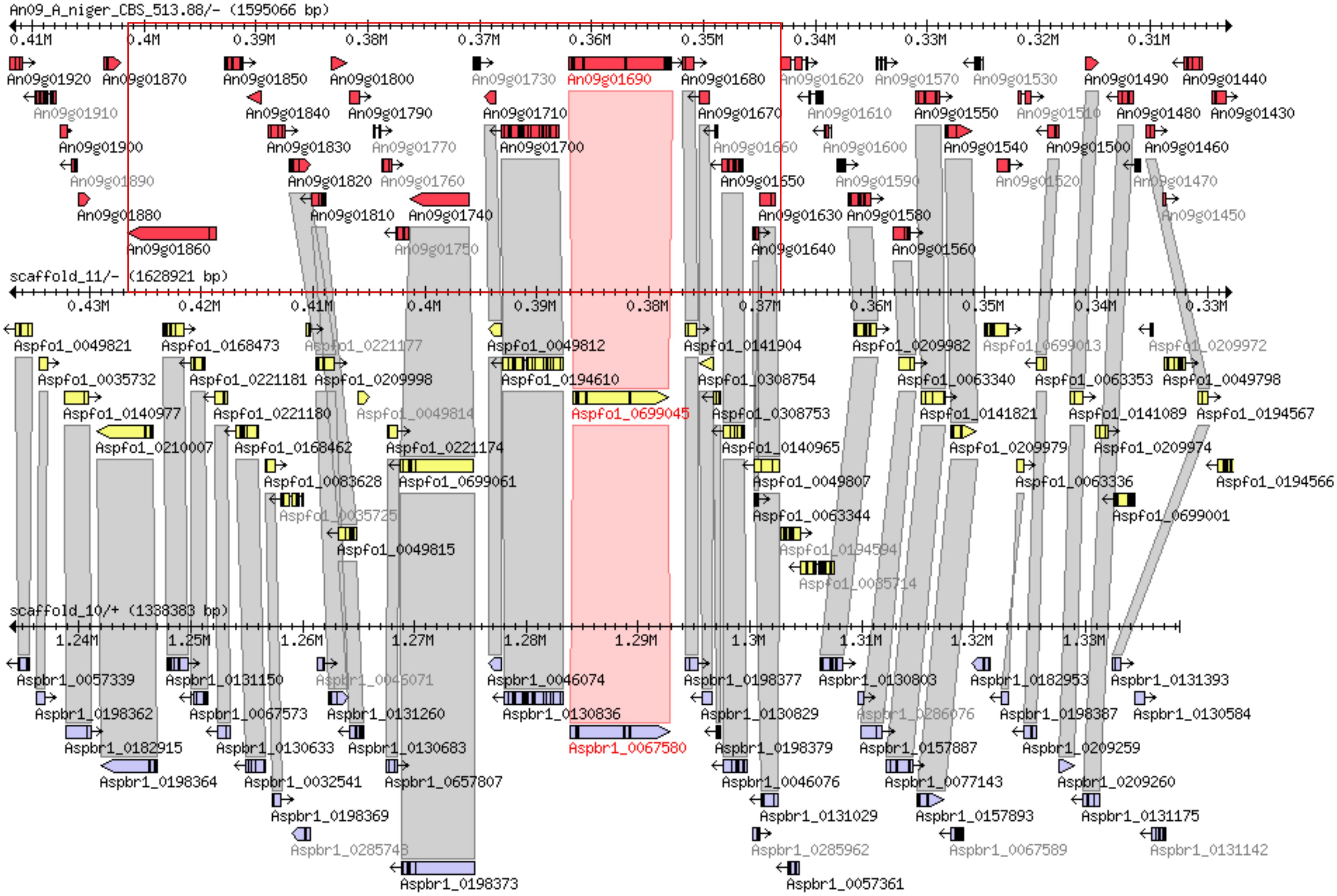

An09g01690 cluster

Gene id (SMURF)	Gene positions	Gene distance	AspGD name	AspGD description line or supplemental annotation	protein ID (antiSMASH)	AspGD ID	
n/a				Domain(s) with predicted transferase activity, transferring glycosyl groups activity and membrane localization	prot_ID_151	An09g01560	
n/a					prot_ID_281	An09g01570	
n/a				Ortholog of A. nidulans FGSC A4 : AN3530, AN2658, A. fumigatus Af293 : Afu1g13760, Afu4g00490, Afu4g14350, A. oryzae RIB40 : AO090166000071 and A. niger ATCC 1015 : 188861-mRNA	prot_ID_248	An09g01580	
n/a				Protein of unknown function	prot_ID_206	An09g01590	
n/a				Protein of unknown function	prot_ID_369	An09g01600	
n/a				Protein of unknown function	prot_ID_6	An09g01610	
n/a				Protein of unknown function	prot_ID_287	An09g01620	
n/a				Domain(s) with predicted acid phosphatase activity	prot_ID_237	An09g01630	ECS
n/a				Ortholog of A. acidus : Aspfo1_0063344	prot_ID_453	An09g01640	
An09g01650	4	400		Domain(s) with predicted role in transmembrane transport and integral to membrane localization	prot_ID_81	An09g01650	
An09g01660	3	530		Ortholog(s) have role in nucleotide-excision repair, phosphorylation of RNA polymerase II C-terminal domain, transcription from RNA polymerase II promoter and core TFIIH complex, holo TFIIH complex localization	prot_ID_438	An09g01660	
An09g01670	2	418		Ortholog of A. brasiliensis : Aspbr1_0130829, A. acidus : Aspfo1_0308754 and Aspergillus carbonarius ITEM 5010 : Acar5010_131745	prot_ID_266	An09g01670	
An09g01680	1	903		Domain(s) with predicted role in response to stress and integral to membrane localization	prot_ID_234	An09g01680	
**An09g01690**	0	0		Putative nonribosomal peptide synthase (NRPS)	**prot_ID_152**	**An09g01690**	
An09g01700	-1	928		Domain(s) with predicted ATP binding, ATPase activity, coupled to transmembrane movement of substances activity, role in transmembrane transport and integral to membrane localization	prot_ID_64	An09g01700	
An09g01710	-2	424		Ortholog of A. nidulans FGSC A4 : AN0788, A. niger CBS 513.88 : An09g02430, An03g05960, An09g00710 and A. oryzae RIB40 : AO090003000250, AO090010000570, AO090023000882, AO090120000022, AO090012000900	prot_ID_565	An09g01710	
An09g01730	-3	404		Protein of unknown function	prot_ID_627	An09g01730	
An09g01740	-4	397		Ortholog(s) have role in fatty acid biosynthetic process, secondary metabolic process	prot_ID_241	An09g01740	

An09g01750	-5	102	Domain(s) with predicted fatty acid synthase activity, role in fatty acid biosynthetic process, oxidation-reduction process and fatty acid synthase complex localization	prot_ID_325	An09g01750	
An09g01760	-6	447	Protein of unknown function	prot_ID_73	An09g01760	
An09g01770	-7	94	Protein of unknown function	prot_ID_393	An09g01770	
An09g01790	-8	1272	Ortholog(s) have role in asperfuranone biosynthetic process, secondary metabolic process	prot_ID_116	An09g01790	
An09g01800	-9	328	Domain(s) with predicted transferase activity, transferring acyl groups other than amino-acyl groups activity	prot_ID_303	An09g01800	
An09g01810	-10	405	Domain(s) with predicted catalytic activity, coenzyme binding, nucleotide binding activity and role in cellular metabolic process	n/a	An09g01810	
An09g01820	-11	209	Ferulate:CoA ligase (AMP-forming)	n/a	An09g01820	
An09g01830	-12	316	Ortholog(s) have role in asperfuranone biosynthetic process, secondary metabolic process	n/a	An09g01830	
An09g01840	-13	553	Salicylate hydroxylase	n/a	An09g01840	
An09g01850	-14	184	3-hydroxybenzoate 4-hydroxylase; benzoate 4-monooxygenase	n/a	An09g01850	
An09g01860	-15	735	Putative polyketide synthase (PKS)	n/a	An09g01860	FA, ECS

An09g01860 cluster

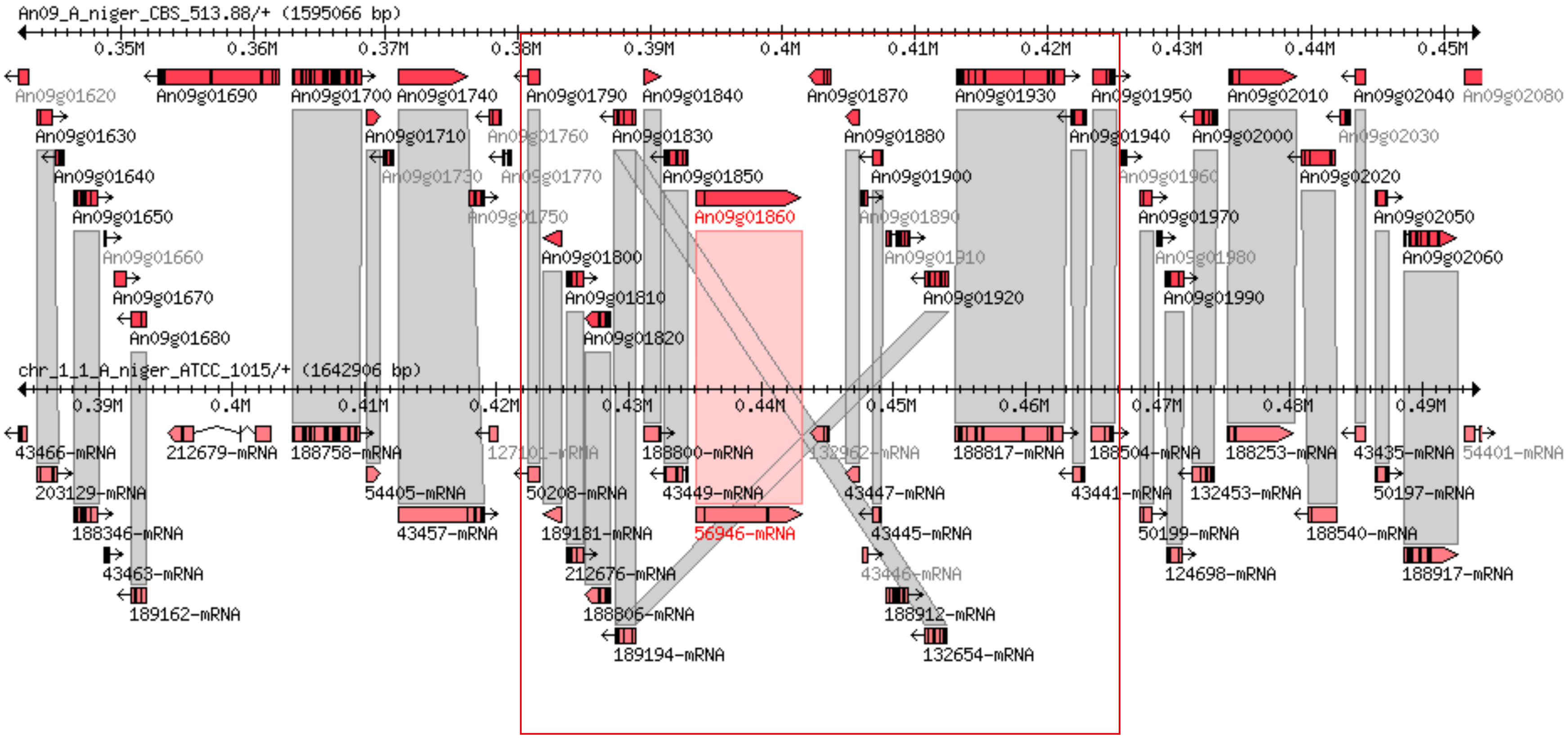

An09g01860 cluster

Gene id (SMURF)	Gene positions	Gene distance	AspGD name	AspGD description line or supplemental annotation	protein ID (antiSMASH)	AspGD ID	
An09g01690	15	928		Domain(s) with predicted ACP phosphopantetheine attachment site binding involved in fatty acid biosynthetic process, ligase activity	n/a	An09g01690	
An09g01700	14	424		Domain(s) with predicted ATP binding, ATPase activity, coupled to transmembrane movement of substances activity, role in transmembrane transport and integral to membrane localization	n/a	An09g01700	
An09g01710	13	404		Ortholog of A. nidulans FGSC A4 : AN0788, A. niger CBS 513.88 : An09g02430, An03g05960, An09g00710 and A. oryzae RIB40 : AO090003000250, AO090010000570, AO090023000882, AO090120000022, AO090012000900	n/a	An09g01710	
An09g01730	12	397		Protein of unknown function	n/a	An09g01730	
An09g01740	11	102		Ortholog(s) have role in fatty acid biosynthetic process, secondary metabolic process	prot_ID_241	An09g01740	
An09g01750	10	447		Domain(s) with predicted fatty acid synthase activity, role in fatty acid biosynthetic process, oxidation-reduction process and fatty acid synthase complex localization	prot_ID_325	An09g01750	
An09g01760	9	94		Protein of unknown function	prot_ID_73	An09g01760	
An09g01770	8	1272		Protein of unknown function	prot_ID_393	An09g01770	
An09g01790	7	328		Ortholog(s) have role in asperfuranone biosynthetic process, secondary metabolic process	prot_ID_116	An09g01790	ECS, FA
An09g01800	6	405		Domain(s) with predicted transferase activity, transferring acyl groups other than amino-acyl groups activity	prot_ID_303	An09g01800	
An09g01810	5	209		Domain(s) with predicted catalytic activity, coenzyme binding, nucleotide binding activity and role in cellular metabolic process	prot_ID_609	An09g01810	
An09g01820	4	316		Ferulate:CoA ligase (AMP-forming)	prot_ID_544	An09g01820	
An09g01830	3	553		Ortholog(s) have role in asperfuranone biosynthetic process, secondary metabolic process	prot_ID_121	An09g01830	
An09g01840	2	184		Salicylate hydroxylase	prot_ID_598	An09g01840	
An09g01850	1	735		3-hydroxybenzoate 4-hydroxylase; benzoate 4-monooxygenase	prot_ID_533	An09g01850	
**An09g01860**	0	0		Putative polyketide synthase (PKS)	**prot_ID_333**	**An09g01860**	
An09g01870	-1	499		Domain(s) with predicted DNA binding, sequence-specific DNA binding RNA polymerase II transcription factor activity, sequence-specific DNA binding transcription factor activity, zinc ion binding activity	prot_ID_356	An09g01870	

An09g01880	-2	1174	Domain(s) with predicted nucleotide binding, oxidoreductase activity, zinc ion binding activity and role in oxidation-reduction process	prot_ID_346	An09g01880	
An09g01890	-3	105	Protein of unknown function	prot_ID_89	An09g01890	
An09g01900	-4	318	Ortholog of A. niger ATCC 1015 : 43445-mRNA	prot_ID_215	An09g01900	
An09g01910	-5	326	Domain(s) with predicted role in transmembrane transport and integral to membrane localization	prot_ID_526	An09g01910	
An09g01920	-6	1019	Ortholog(s) have role in asperfuranone biosynthetic process, secondary metabolic process	prot_ID_426	An09g01920	
An09g01930	-7	630	Ortholog(s) have role in asperfuranone biosynthetic process, emericellamide biosynthetic process, monodictyphenone biosynthetic process, o-orsellinic acid biosynthetic process, sterigmatocystin biosynthetic process	prot_ID_400	An09g01930	
An09g01940	-8	394	Domain(s) with predicted role in response to stress and integral to membrane localization	prot_ID_147	An09g01940	
An09g01950	-9	519	Ortholog(s) have catalytic activity and role in sterigmatocystin biosynthetic process	prot_ID_252	An09g01950	ECS, FA
An09g01960	-10	322	Protein of unknown function	prot_ID_140	An09g01960	
An09g01970	-11	787	Domain(s) with predicted nucleotide binding, oxidoreductase activity and role in oxidation-reduction process	prot_ID_43	An09g01970	
An09g01980	-12	178	Protein of unknown function	prot_ID_42	An09g01980	
An09g01990	-13	272	Branched chain amino acid aminotransferase	prot_ID_269	An09g01990	
An09g02000	-14	814	Domain(s) with predicted electron carrier activity, heme binding, iron ion binding, oxidoreductase activity, acting on paired donors, with incorporation or reduction of molecular oxygen activity and role in oxidation-reduction process	prot_ID_99	An09g02000	
An09g02010	-15	869	Fatty-acid synthase	prot_ID_40	An09g02010	
An09g02020	-16	400	Domain(s) with predicted GTP binding, GTPase activity	prot_ID_607	An09g02020	

An09g02100 cluster

No ATCC 1015 match, no manual prediction made

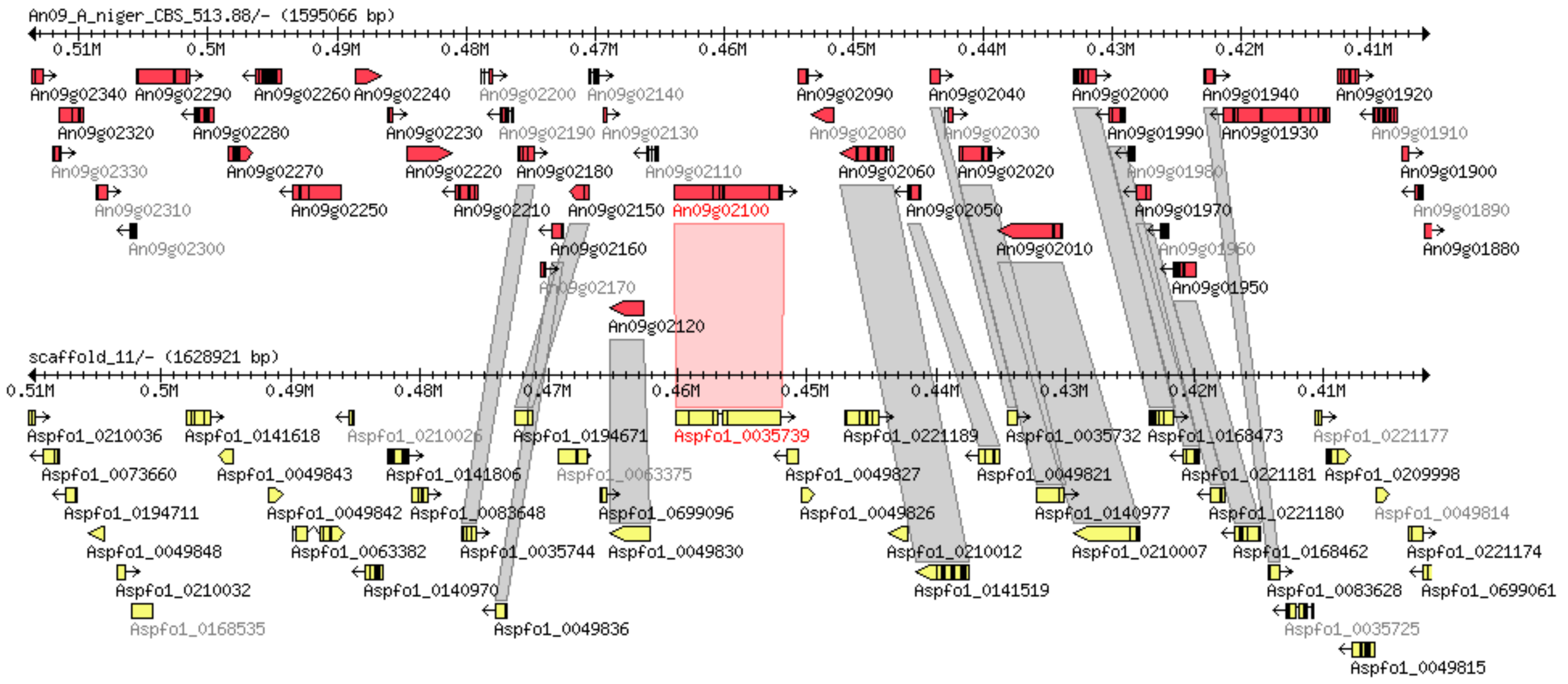

An09g02100 cluster

Gene id (SMURF)	Gene positions	Gene distance	AspGD name	AspGD description line or supplemental annotation	protein ID (antiSMASH)	AspGD ID
An09g01980	11	272			prot_ID_252	An09g01980
An09g01990	10	814		Branched chain amino acid aminotransferase	prot_ID_140	An09g01990
An09g02000	9	869		Domain(s) with predicted electron carrier activity, heme binding, iron ion binding, oxidoreductase activity, acting on paired donors, with incorporation or reduction of molecular oxygen activity and role in oxidation-reduction process	prot_ID_43	
An09g02010	8	400		Fatty-acid synthase	prot_ID_42	An09g02010
An09g02020	7	356		Domain(s) with predicted GTP binding, GTPase activity	prot_ID_269	An09g02020
An09g02030	6	290			prot_ID_99	An09g02030
An09g02040	5	785		Ortholog of A. nidulans FGSC A4 : AN11160, A. oryzae RIB40 : AO090003001445, A. brasiliensis : Aspbr1_0198362, A. niger ATCC 1015 : 43435-mRNA and Aspergillus flavus NRRL 3357 : AFL2T_01636	prot_ID_40	An09g02040
An09g02050	4	1157		Domain(s) with predicted catalytic activity and role in metabolic process	prot_ID_607	An09g02050
An09g02060	3	723		Ortholog of A. nidulans FGSC A4 : AN5037, A. niger ATCC 1015 : 188917-mRNA, A. versicolor : Aspve1_0152207, Aspergillus niger ATCC 1015 : 170720-mRNA and A. sydowii : Aspsy1_0137186	prot_ID_580	An09g02060
An09g02080	2	411		Domain(s) with predicted nucleic acid binding activity	prot_ID_170	An09g02080
An09g02090	1	1087		Ortholog of A. nidulans FGSC A4 : AN7124, A. niger CBS 513.88 : An12g05320, A. oryzae RIB40 : AO090038000612, AO090003001456 and A. brasiliensis : Aspbr1_0044515, Aspbr1_0121978, Aspbr1_0257373	prot_ID_357	An09g02090
**An09g02100**	0	0		Domain(s) with predicted ACP phosphopantetheine attachment site binding involved in fatty acid biosynthetic process, hydrolase activity, acting on ester bonds, nucleotide binding, phosphopantetheine binding, transferase activity	**prot_ID_343**	**An09g02100**
An09g02110	-1	1235			prot_ID_572	An09g02110
An09g02120	-2	262		Domain(s) with predicted oxidoreductase activity, acting on paired donors, with incorporation or reduction of molecular oxygen, 2-oxoglutarate as one donor, and incorporation of one atom each of oxygen into both donors activity	prot_ID_541	An09g02120
An09g02130	-3	278			prot_ID_579	An09g02130
An09g02140	-4	355			prot_ID_666	An09g02140

An09g02150	-5	41	Domain(s) with predicted monooxygenase activity and role in oxidation-reduction process	prot_ID_115	An09g02150
An09g02160	-6	557	Ortholog(s) have rhamnogalacturonan acetylesterase activity and role in pectin catabolic process	prot_ID_420	An09g02160
An09g02170	-7	514		prot_ID_143	An09g02170
An09g02180	-8	703	Triacylglycerol lipase	prot_ID_8	An09g02180
An09g02190	-9	364		prot_ID_536	An09g02190
An09g02200	-10	424		prot_ID_279	An09g02200
An09g02210	-11	272	Domain(s) with predicted role in transmembrane transport and integral to membrane localization	prot_ID_409	An09g02210
An09g02220	-12	322	Domain(s) with predicted DNA-directed DNA polymerase activity, damaged DNA binding, magnesium ion binding activity, role in DNA repair and intracellular localization	prot_ID_486	An09g02220
An09g02230	-13	950	Ortholog of A. nidulans FGSC A4 : AN9338, AN7059, A. niger CBS 513.88 : An12g02100, A. oryzae RIB40 : AO090138000084 and A. niger ATCC 1015 : 50192-mRNA, 50339-mRNA, 50930-mRNA	n/a	An09g02230

An09g05110 cluster

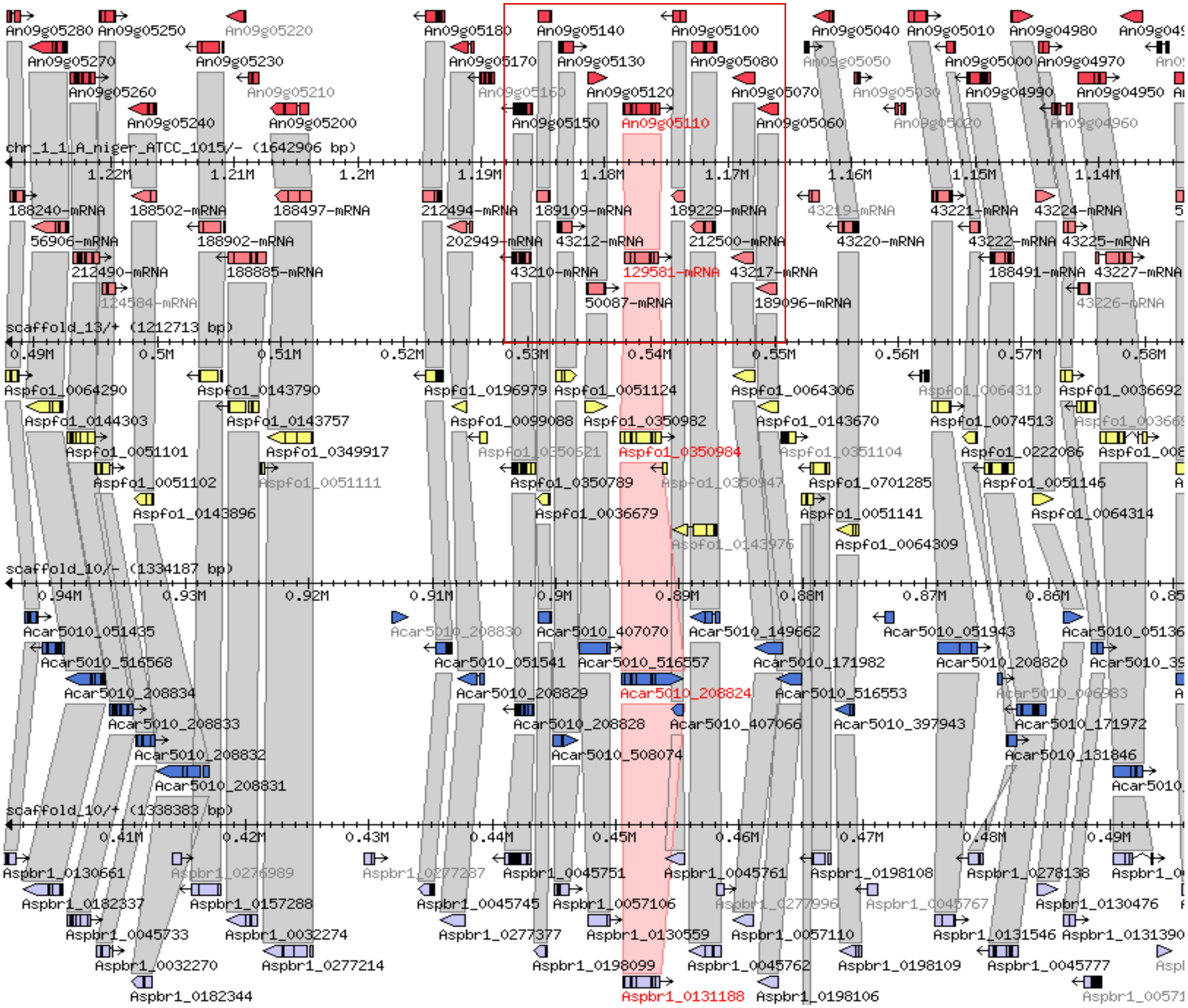

An09g05110 cluster

Gene id (SMURF)	Gene positions	Gene distance	AspGD name	AspGD description line or supplemental annotation	protein ID (antiSMASH)	AspGD ID	
An09g04960	14	95		Domain(s) with predicted ATP binding, protein kinase activity and role in protein phosphorylation	n/a	An09g04960	
An09g04970	13	427		Ortholog of A. nidulans FGSC A4 : AN6420, A. fumigatus Af293 : Afu2g01100, A. oryzae RIB40 : AO090005001149, A. niger ATCC 1015 : 43225-mRNA and A. versicolor : Aspve1_0041410	n/a	An09g04970	
An09g04980	12	1696		Domain(s) with predicted sequence-specific DNA binding RNA polymerase II transcription factor activity, sequence-specific DNA binding transcription factor activity, zinc ion binding activity	n/a	An09g04980	
An09g04990	11	864		Putative amidase/acetamidase	n/a	An09g04990	
An09g05000	10	1391		Ortholog of A. brasiliensis : Aspbr1_0278138, A. acidus : Aspfo1_0222086, A. niger ATCC 1015 : 43222-mRNA and Aspergillus carbonarius ITEM 5010 : Acar5010_131846	n/a	An09g05000	
An09g05010	9	204		Domain(s) with predicted amino acid transmembrane transporter activity, role in amino acid transmembrane transport and membrane localization	prot_ID_161	An09g05010	
An09g05020	8	2902		Protein of unknown function	prot_ID_457	An09g05020	
An09g05030	7	1647		Protein of unknown function	prot_ID_28	An09g05030	
An09g05040	6	306		Ortholog of A. nidulans FGSC A4 : AN8167, A. fumigatus Af293 : Afu5g02950, A. oryzae RIB40 : AO090102000494, A. niger ATCC 1015 : 43220-mRNA and A. versicolor : Aspve1_0143405	prot_ID_198	An09g05040	
An09g05050	5	2048		Protein of unknown function	prot_ID_674	An09g05050	
An09g05060	4	267		Ortholog(s) have role in kojic acid biosynthetic process, positive regulation of kojic acid biosynthetic process, positive regulation of secondary metabolite biosynthetic process, secondary metabolic process	prot_ID_385	An09g05060	ECS
An09g05070	3	1335		Domain(s) with predicted role in transmembrane transport and integral to membrane localization	prot_ID_33	An09g05070	
An09g05080	2	455		Domain(s) with predicted pyridoxal phosphate binding, transferase activity, transferring nitrogenous groups activity and role in biosynthetic process	prot_ID_539	An09g05080	
An09g05100	1	930		Ortholog of A. nidulans FGSC A4 : AN8173, A. fumigatus Af293 : Afu5g03000, A. oryzae RIB40 : AO090102000499, A. niger ATCC 1015 : 189229-mRNA and A. versicolor : Aspve1_0057171	prot_ID_490	An09g05100	

**An09g05110**	0	0	Protein similar to nonribosomal peptide synthases (NRPS-like)	**prot_ID_581**	**An09g05110**	
An09g05120	-1	1346	Triacylglycerol lipase	prot_ID_169	An09g05120	
An09g05130	-2	1070	Domain(s) with predicted oxidoreductase activity and role in metabolic process	prot_ID_268	An09g05130	
n/a			Domain(s) with predicted nucleotide binding activity	prot_ID_588	An09g05140	
n/a			Domain(s) with predicted ferric iron binding, oxidoreductase activity, acting on single donors with incorporation of molecular oxygen, incorporation of two atoms of oxygen activity	prot_ID_515	An09g05150	ECS
n/a			Protein of unknown function	prot_ID_313	An09g05160	
n/a			Domain(s) with predicted viral capsid localization	prot_ID_199	An09g05170	
n/a			Domain(s) with predicted structural constituent of ribosome activity, role in translation and ribosome localization	prot_ID_362	An09g05180	

An09g05340 cluster

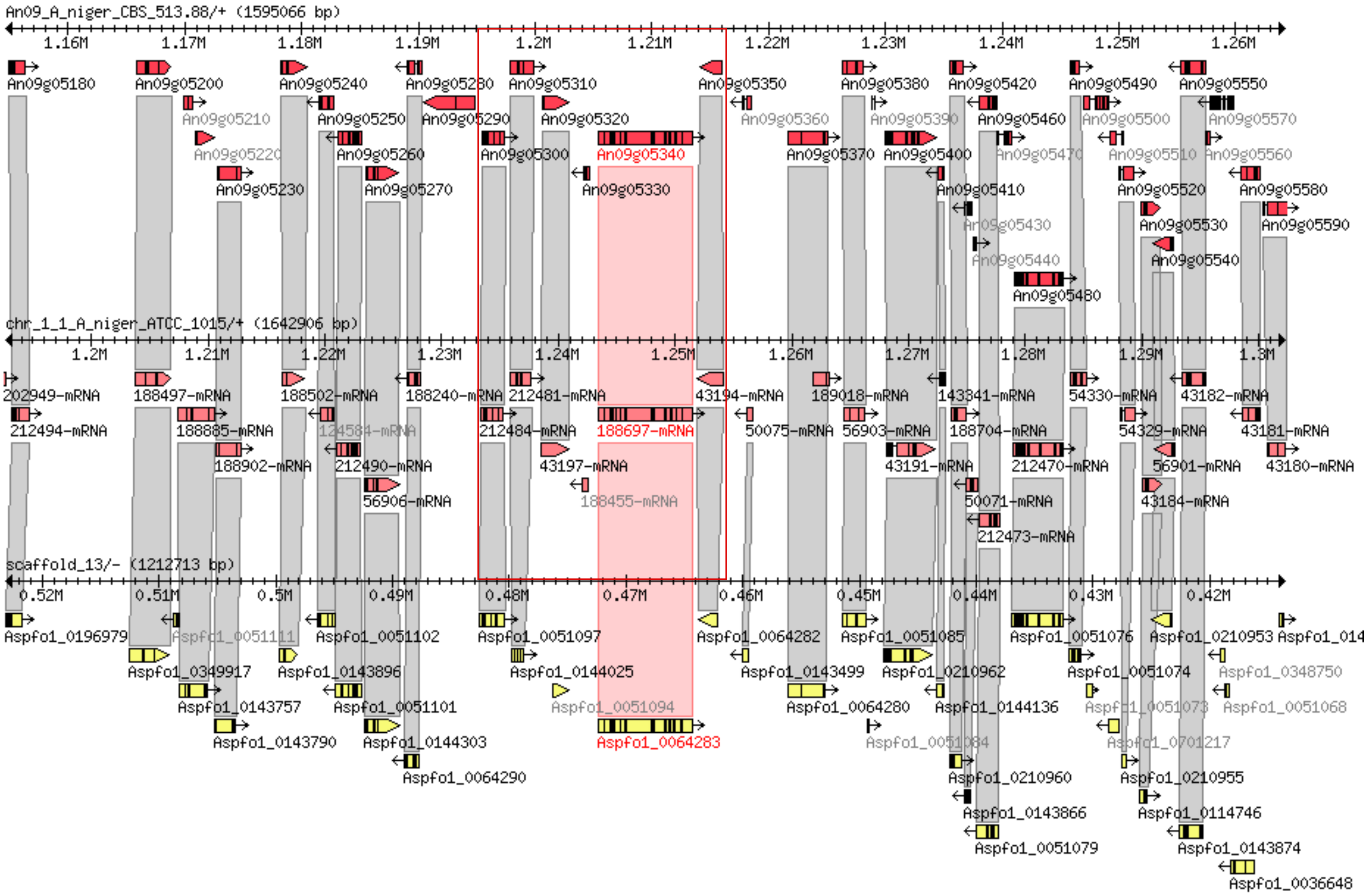

An09g05340 cluster

Gene id (SMURF)	Gene positions	Gene distance	AspGD name	AspGD description line or supplemental annotation	protein ID (antiSMASH)	AspGD ID	
n/a				Ortholog of A. fumigatus Af293 : Afu5g03090, A. oryzae RIB40 : AO090102000512, A. niger ATCC 1015 : 56906-mRNA, A. versicolor : Aspve1_0178942 and A. sydowii : Aspsy1_0136686	prot_ID_488	An09g05270	
n/a				Ortholog of A. nidulans FGSC A4 : AN11053, A. fumigatus Af293 : Afu5g03110, A. niger CBS 513.88 : An01g14890 and A. oryzae RIB40 : AO090701000457, AO090102000514	prot_ID_304	An09g05280	
n/a				Domain(s) with predicted 5'-3' exonuclease activity, nucleic acid binding activity, role in nucleobase-containing compound metabolic process and intracellular localization	prot_ID_596	An09g05290	
n/a				Ortholog of A. nidulans FGSC A4 : AN8187, A. fumigatus Af293 : Afu5g03130, A. oryzae RIB40 : AO090102000516, A. niger ATCC 1015 : 212484-mRNA and A. versicolor : Aspve1_0142812	prot_ID_587	An09g05300	ECS
An09g05310	3	1235		GTP cyclohydrolase I	prot_ID_167	An09g05310	
An09g05320	2	1155		Ortholog of A. fumigatus Af293 : Afu5g03150, A. oryzae RIB40 : AO090102000518, A. niger ATCC 1015 : 43197-mRNA and Aspergillus carbonarius ITEM 5010 : Acar5010_508086	prot_ID_35	An09g05320	
An09g05330	1	694		Ortholog of A. nidulans FGSC A4 : AN9006, A. fumigatus Af293 : Afu3g02540, Afu6g11710, Afu7g08440, Afu5g00145, A. oryzae RIB40 : AO090038000212 and N. fischeri NRRL 181 : NFIA_004090, NFIA_057380	prot_ID_194	An09g05330	
**An09g05340**	0	0		Putative polyketide synthase (PKS)	**prot_ID_597**	**An09g05340**	
An09g05350	-1	558		Ortholog of A. niger CBS 513.88 : An08g08140, An15g05280, An04g02780, A. oryzae RIB40 : AO090010000573 and A. brasiliensis : Aspbr1_0030961, Aspbr1_0045725, Aspbr1_0155263, Aspbr1_0205665	prot_ID_56	An09g05350	ECS
An09g05360	-2	2047		Domain(s) with predicted hydrolase activity and role in dUTP metabolic process	prot_ID_150	An09g05360	
n/a				Domain(s) with predicted ATP binding, protein serine/threonine kinase activity and role in protein phosphorylation	prot_ID_529	An09g05370	
n/a				Domain(s) with predicted translation initiation factor activity, role in translational initiation and cytoplasm localization	prot_ID_186	An09g05380	
n/a					prot_ID_566	An09g05390	

n/a	Domain(s) with predicted cysteine-type peptidase activity and role in proteolysis	prot_ID_293	An09g05400

An09g05730 cluster

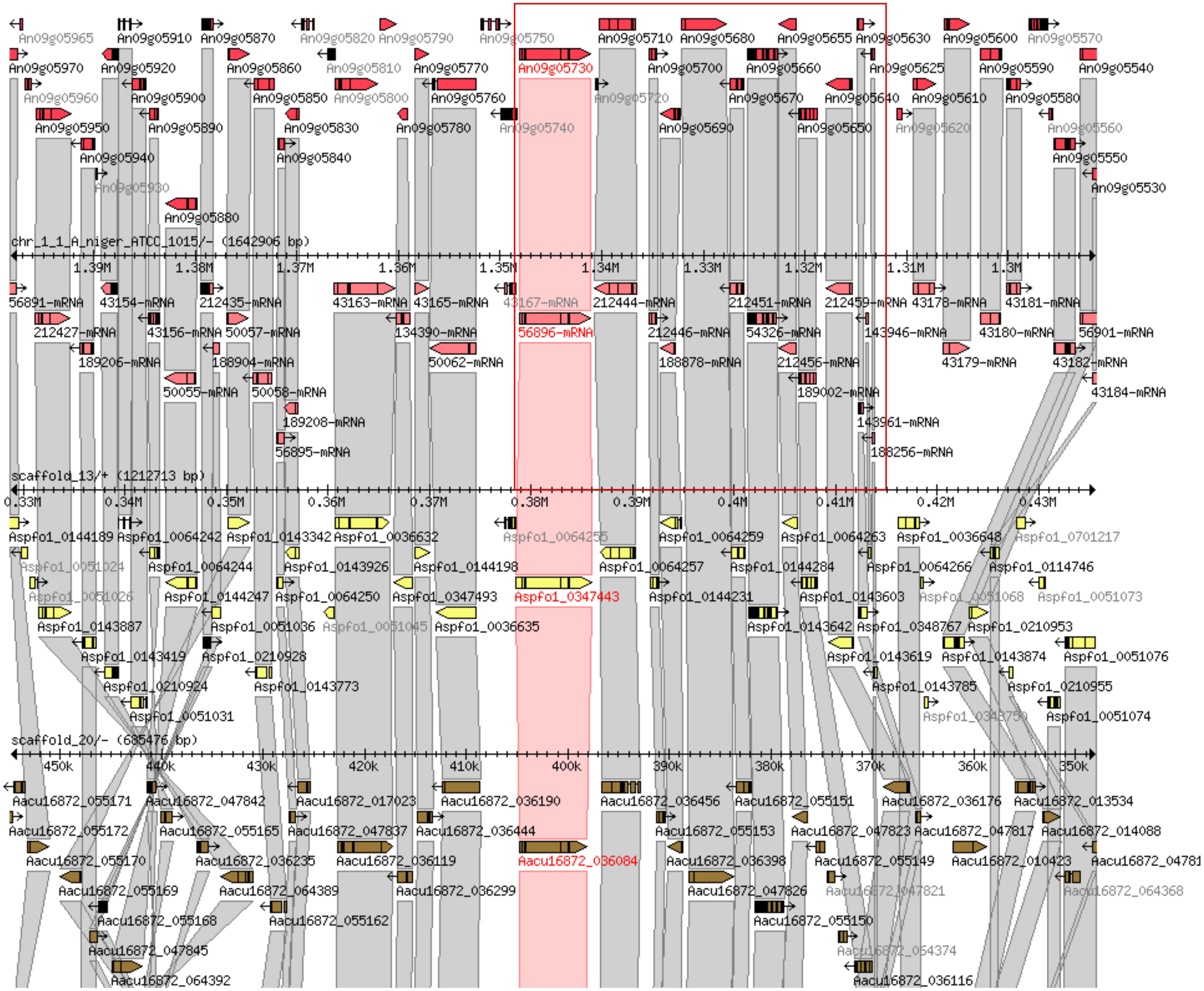

An09g05730 cluster

Gene id (SMURF)	Gene positions	Gene distance	AspGD name	AspGD description line or supplemental annotation	protein ID (antiSMASH)	AspGD ID	
n/a				Protein of unknown function	n/a	An09g05625	ECS
n/a				Protein of unknown function	n/a	An09g05630	
n/a				ATP-dependent DNA helicase	n/a	An09g05640	
n/a				Protein of unknown function	n/a	An09g05650	
n/a				Ortholog of A. nidulans FGSC A4 : AN8202, A. fumigatus Af293 : Afu5g03340, A. oryzae RIB40 : AO090102000539, A. niger ATCC 1015 : 212456-mRNA and A. versicolor : Aspve1_0089228	prot_ID_447	An09g05655	
n/a				NAD synthetase	prot_ID_249	An09g05660	
n/a				NAD pyrophosphatase	prot_ID_647	An09g05670	
n/a				Domain(s) with predicted DNA binding activity, role in telomere maintenance and nuclear chromosome, telomeric region localization	prot_ID_142	An09g05680	
n/a				Domain(s) with predicted N,N-dimethylaniline monooxygenase activity, NADP binding, flavin adenine dinucleotide binding activity and role in oxidation-reduction process	prot_ID_408	An09g05690	
n/a				Domain(s) with predicted metal ion binding, phosphoric diester hydrolase activity	prot_ID_306	An09g05700	
An09g05710	2	170		Domain(s) with predicted nucleus localization	prot_ID_83	An09g05710	
An09g05720	1	775		Protein of unknown function	prot_ID_94	An09g05720	
**An09g05730**	0	0	fwnA	Putative polyketide synthase (PKS) involved in both dihydroxynaphthalene (DHN) melanin and naphtho-gamma-pyrone production; required for wild-type black spore pigmentation; mutants have reduced ornamention on the conidial cell surface	**prot_ID_189**	**An09g05730**	ECS
An09g05740	-1	181		Protein of unknown function	prot_ID_608	An09g05740	
An09g05750	-2	77		Protein of unknown function	prot_ID_205	An09g05750	
n/a				Ortholog of A. nidulans FGSC A4 : AN12403, A. fumigatus Af293 : Afu5g03400, A. oryzae RIB40 : AO090102000549, A. niger ATCC 1015 : 50062-mRNA and A. versicolor : Aspve1_0047187	prot_ID_15	An09g05760	
n/a				Domain(s) with predicted manganese ion binding, pyrophosphatase activity and cytoplasm localization	prot_ID_111	An09g05770	
n/a				Domain(s) with predicted catalytic activity, pyridoxal phosphate binding activity and role in metabolic process	prot_ID_184	An09g05780	

n/a		prot_ID_617	An09g05790
n/a	Domain(s) with predicted DNA binding, oxidoreductase activity, acting on paired donors, with incorporation or reduction of molecular oxygen and 2-oxoglutarate as one donor, more	prot_ID_93	An09g05800
n/a		prot_ID_388	An09g05810

An10g00140 cluster

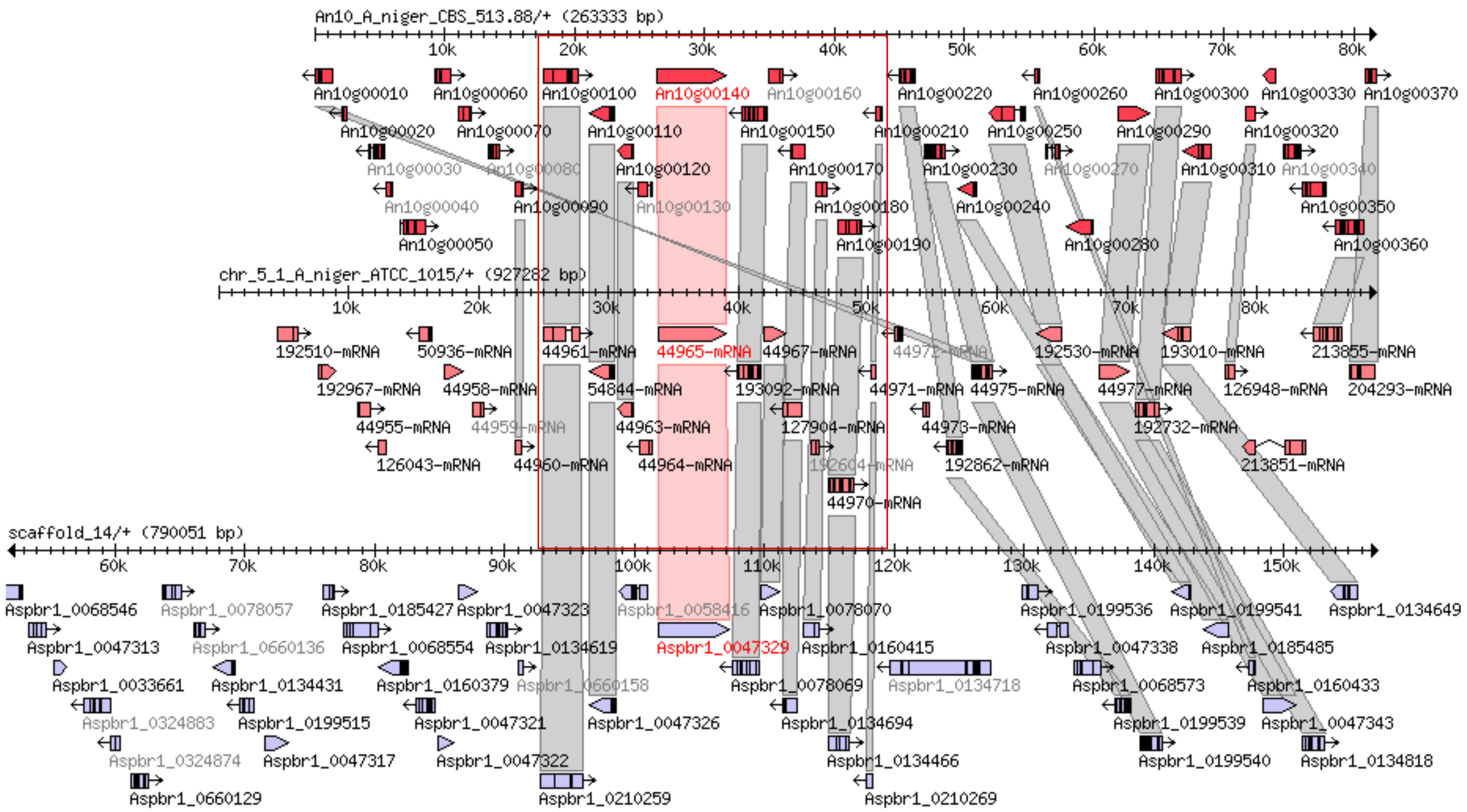

An10g00140 cluster

Gene id (SMURF)	Gene positions	Gene distance	AspGD name	AspGD description line or supplemental annotation	protein ID (antiSMASH)	AspGD ID	
An10g00010	13	686		Putative alcohol dehydrogenase (catalyzed by glycerol dehydrogenase II (NADP+)	n/a	An10g00010	
An10g00020	12	1690		Domain(s) with predicted channel activity, role in cation transport, hemolysis in other organism involved in symbiotic interaction, pore complex assembly and pore complex localization	n/a	An10g00020	
An10g00030	11	120		Domain(s) with predicted hydrolase activity	n/a	An10g00030	
An10g00040	10	521			n/a	An10g00040	
An10g00050	9	795		Domain(s) with predicted role in transmembrane transport and integral to membrane localization	prot_ID_81	An10g00050	
An10g00060	8	487		Domain(s) with predicted sequence-specific DNA binding RNA polymerase II transcription factor activity, sequence-specific DNA binding transcription factor activity, zinc ion binding activity	prot_ID_31	An10g00060	
An10g00070	7	1114		Ortholog(s) have flavin adenine dinucleotide binding activity and role in cellular response to mycotoxin, gliotoxin biosynthetic process	prot_ID_71	An10g00070	
An10g00080	6	1025			prot_ID_78	An10g00080	
An10g00090	5	1501		Ortholog of A. acidus : Aspfo1_0051750 and A. niger ATCC 1015 : 44960-mRNA	prot_ID_62	An10g00090	
An10g00100	4	845		Domain(s) with predicted DNA binding, sequence-specific DNA binding RNA polymerase II transcription factor activity, sequence-specific DNA binding transcription factor activity, zinc ion binding activity	prot_ID_54	An10g00100	ECS
An10g00110	3	295		Domain(s) with predicted electron carrier activity, heme binding, iron ion binding, oxidoreductase activity, acting on paired donors, with incorporation or reduction of molecular oxygen activity and role in oxidation-reduction process	prot_ID_84	An10g00110	
An10g00120	2	467		Domain(s) with predicted catalytic activity and role in metabolic process	prot_ID_99	An10g00120	
An10g00130	1	389		Domain(s) with predicted prenyltransferase activity and integral to membrane localization	prot_ID_103	An10g00130	
**An10g00140**	0	0		Putative polyketide synthase (PKS)	**prot_ID_37**	**An10g00140**	
An10g00150	-1	1228		Domain(s) with predicted electron carrier activity, heme binding, iron ion binding, oxidoreductase activity, acting on paired donors, with incorporation or reduction of molecular oxygen activity and role in oxidation-reduction process	prot_ID_10	An10g00150	

An10g00160	-2	207	Protein of unknown function	prot_ID_45	An10g00160	
An10g00170	-3	574	Domain(s) with predicted nucleotide binding, oxidoreductase activity and role in oxidation-reduction process	prot_ID_91	An10g00170	
An10g00180	-4	802	Ortholog of A. brasiliensis : Aspbr1_0160415, Aspergillus flavus NRRL 3357 : AFL2T_04691, A. clavatus NRRL 1 : ACLA_093650 and A. fumigatus A1163 : AFUB_079730	prot_ID_20	An10g00180	
An10g00190	-5	875	Domain(s) with predicted UDP-N-acetylmuramate dehydrogenase activity, flavin adenine dinucleotide binding activity and role in oxidation-reduction process	prot_ID_87	An10g00190	
An10g00210	-6	970	Ortholog of A. brasiliensis : Aspbr1_0210269, A. clavatus NRRL 1 : ACLA_087630, A. acidus : Aspfo1_0211502, Aspergillus terreus NIH2624 : ATET_01621 and A. niger ATCC 1015 : 44971-mRNA	prot_ID_44	An10g00210	ECS
An10g00220	-7	1350	Ortholog of A. nidulans FGSC A4 : AN9115, A. oryzae RIB40 : AO090010000382, A. brasiliensis : Aspbr1_0199539, A. niger ATCC 1015 : 192862-mRNA and Aspergillus flavus NRRL 3357 : AFL2T_11557	prot_ID_29	An10g00220	
An10g00230	-8	714	Putative formaldehyde dehydrogenase	prot_ID_7	An10g00230	
n/a			Ortholog of A. niger CBS 513.88 : An01g02040, A. brasiliensis : Aspbr1_0040504, Aspbr1_0199541, A. acidus : Aspfo1_0072680, Aspfo1_0144505 and A. versicolor : Aspve1_0087805	prot_ID_1	An10g00240	
n/a			ATP binding, two-component response regulator	n/a	An10g00250	

An10g00630 cluster

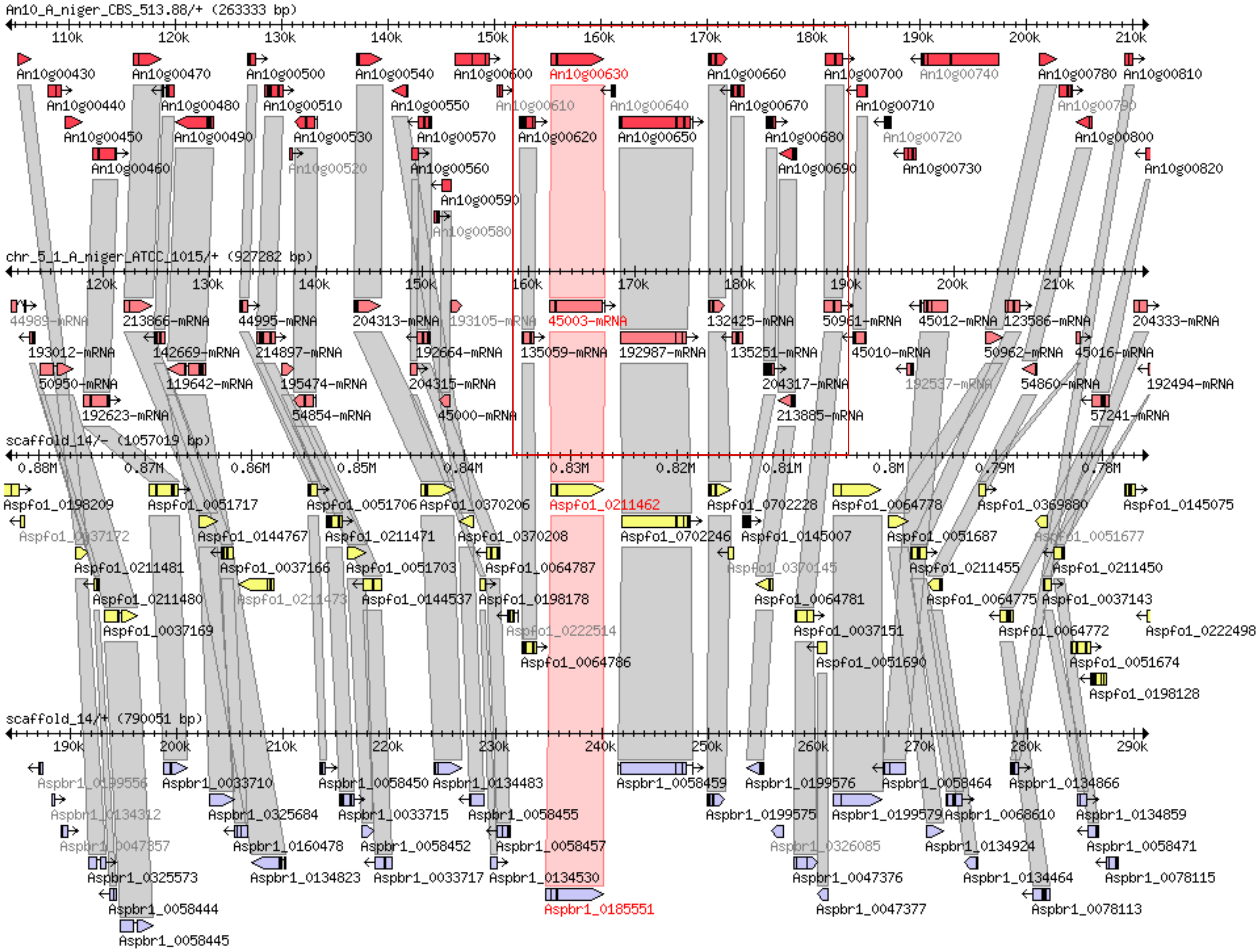

An10g00630 cluster

Gene id (SMURF)	Gene positions	Gene distance	AspGD name	AspGD description line or supplemental annotation	protein ID (antiSMASH)	AspGD ID	
An10g00540	9	906		Domain(s) with predicted zinc ion binding activity	n/a	An10g00540	
An10g00550	8	691		Ortholog of A. brasiliensis : Aspbr1_0058455 and A. acidus : Aspfo1_0370208	n/a	An10g00550	
An10g00560	7	53		Ortholog of A. nidulans FGSC A4 : AN7635/binA, AN11904, A. fumigatus Af293 : Afu3g02216, Afu7g00610, Afu2g00967 and A. oryzae RIB40 : AO090009000537, AO090010000166, AO090701000353	n/a	An10g00560	
An10g00570	6	222		Domain(s) with predicted nucleotide binding, oxidoreductase activity, zinc ion binding activity and role in oxidation-reduction process	n/a	An10g00570	
An10g00580	5	182			n/a	An10g00580	
An10g00590	4	458		Ortholog of A. niger ATCC 1015 : 45000-mRNA	n/a	An10g00590	
An10g00600	3	789		Domain(s) with predicted ADP binding, ATP binding, catalytic activity and role in apoptotic process, defense response, nucleoside metabolic process	n/a	An10g00600	
An10g00610	2	1616			n/a	An10g00610	
An10g00620	1	1375		Domain(s) with predicted branched-chain-amino-acid transaminase activity and role in branched-chain amino acid metabolic process	n/a	An10g00620	ECS
**An10g00630**	0	0		Domain(s) with predicted 3-oxoacyl-[acyl-carrier-protein] reductase (NADPH) activity, fatty-acyl-CoA synthase activity, nucleotide binding activity and role in fatty acid biosynthetic process, oxidation-reduction process	n/a	**An10g00630**	
An10g00640	-1	684		Protein of unknown function	n/a	An10g00640	
An10g00650	-2	442		Domain(s) with predicted enoyl-[acyl-carrier-protein] reductase (NADH) activity, role in fatty acid biosynthetic process, oxidation-reduction process and fatty acid synthase complex localization	n/a	An10g00650	
An10g00660	-3	1474		Domain(s) with predicted electron carrier activity, heme binding, iron ion binding, oxidoreductase activity, acting on paired donors, with incorporation or reduction of molecular oxygen activity and role in oxidation-reduction process	n/a	An10g00660	
An10g00670	-4	369		Ortholog(s) have trans-aconitate 3-methyltransferase activity and cytosol localization	n/a	An10g00670	
An10g00680	-5	2056		Domain(s) with predicted hydrogen ion transmembrane transporter activity, role in ATP hydrolysis coupled proton transport and proton-transporting V-type ATPase, V0	n/a	An10g00680	

			domain localization			
An10g00690	-6	292	Domain(s) with predicted role in transmembrane transport and integral to membrane localization	n/a	An10g00690	
An10g00700	-7	2638	Domain(s) with predicted role in transmembrane transport and integral to membrane localization	n/a	An10g00700	ECS

An11g00050 cluster

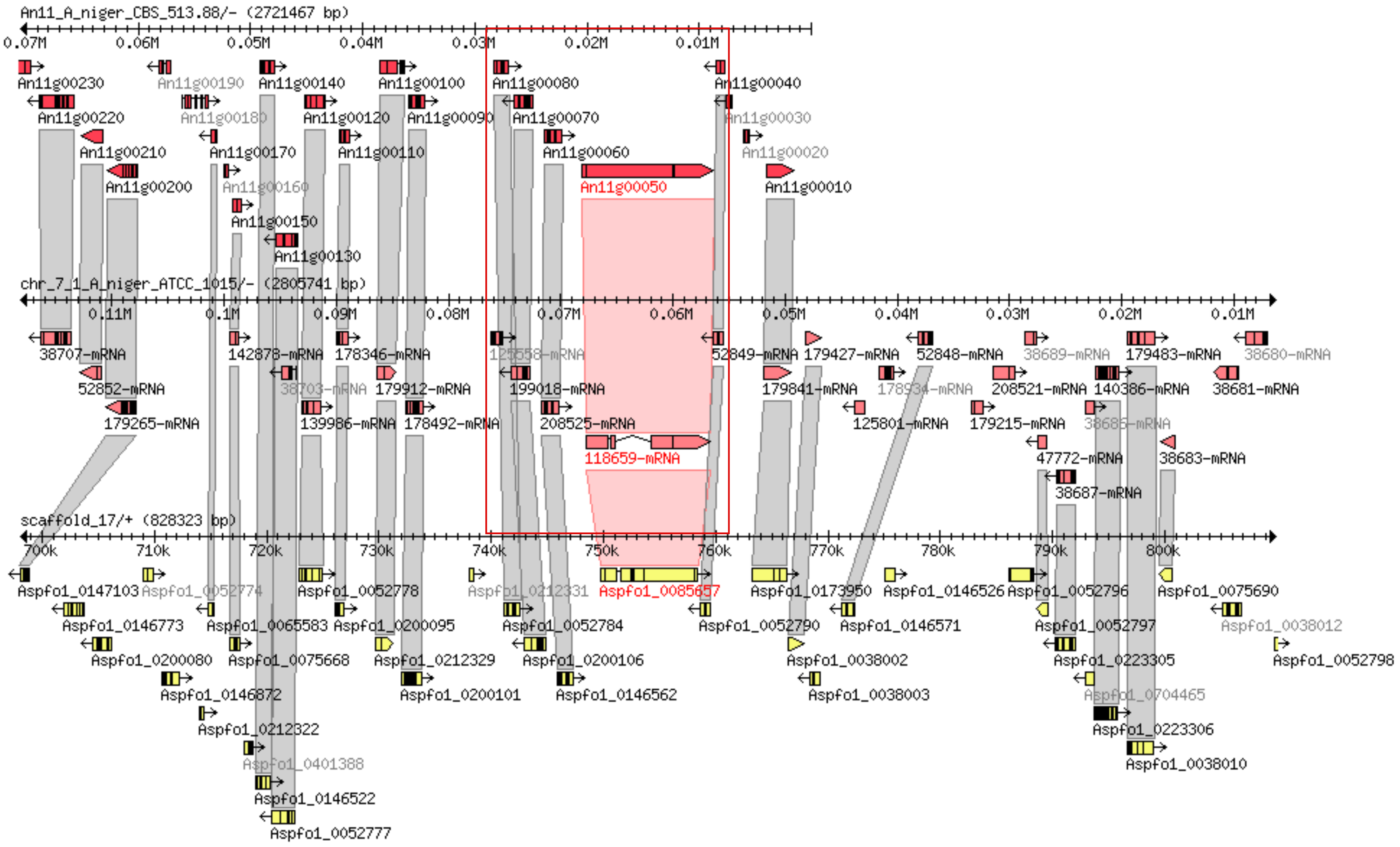

An11g00050 cluster

Gene id (SMURF)	Gene positions	Gene distance	AspGD name	AspGD description line or supplemental annotation	protein ID (antiSMASH)	AspGD ID	
n/a				Domain(s) with predicted GTP binding, GTPase activity	prot_ID_121	An11g00010	
n/a					prot_ID_71	An11g00020	
An11g00030	2	199			prot_ID_799	An11g00030	
An11g00040	1	392		Ortholog of A. nidulans FGSC A4 : AN3008, A. niger CBS 513.88 : An15g05480, A. oryzae RIB40 : AO090011000565, A. niger ATCC 1015 : 52849-mRNA and Aspergillus niger ATCC 1015 : 46090-mRNA	prot_ID_996	An11g00040	ECS
**An11g00050**	0	0		Domain(s) with predicted ACP phosphopantetheine attachment site binding involved in fatty acid biosynthetic process, ligase activity, phosphopantetheine binding activity	**prot_ID_742**	**An11g00050**	
An11g00060	-1	1892		Protein with similarity to integral membrane protein PTH11 of Magnaporthe grisea	prot_ID_1083	An11g00060	
An11g00070	-2	1097		Domain(s) with predicted O-methyltransferase activity	prot_ID_442	An11g00070	
An11g00080	-3	532		Ortholog of A. brasiliensis : Aspbr1_0189328, A. acidus : Aspfo1_0052784, Aspergillus carbonarius ITEM 5010 : Acar5010_163416 and Aspergillus aculeatus ATCC16872 : Aacu16872_048410	prot_ID_814	An11g00080	ECS
n/a				Ortholog of A. nidulans FGSC A4 : AN5943, AN8548, AN8661, AN4642, A. fumigatus Af293 : Afu3g00850, Afu4g08850 and A. niger CBS 513.88 : An02g13470, An03g01430, An12g09260	prot_ID_183	An11g00090	
n/a				Ortholog of A. nidulans FGSC A4 : AN5944, A. oryzae RIB40 : AO090009000720, A. brasiliensis : Aspbr1_0201333, A. niger ATCC 1015 : 179912-mRNA and Aspergillus flavus NRRL 3357 : AFL2T_10172	prot_ID_818	An11g00100	

An11g00250 cluster

No ATCC 1015 orthologous cluster

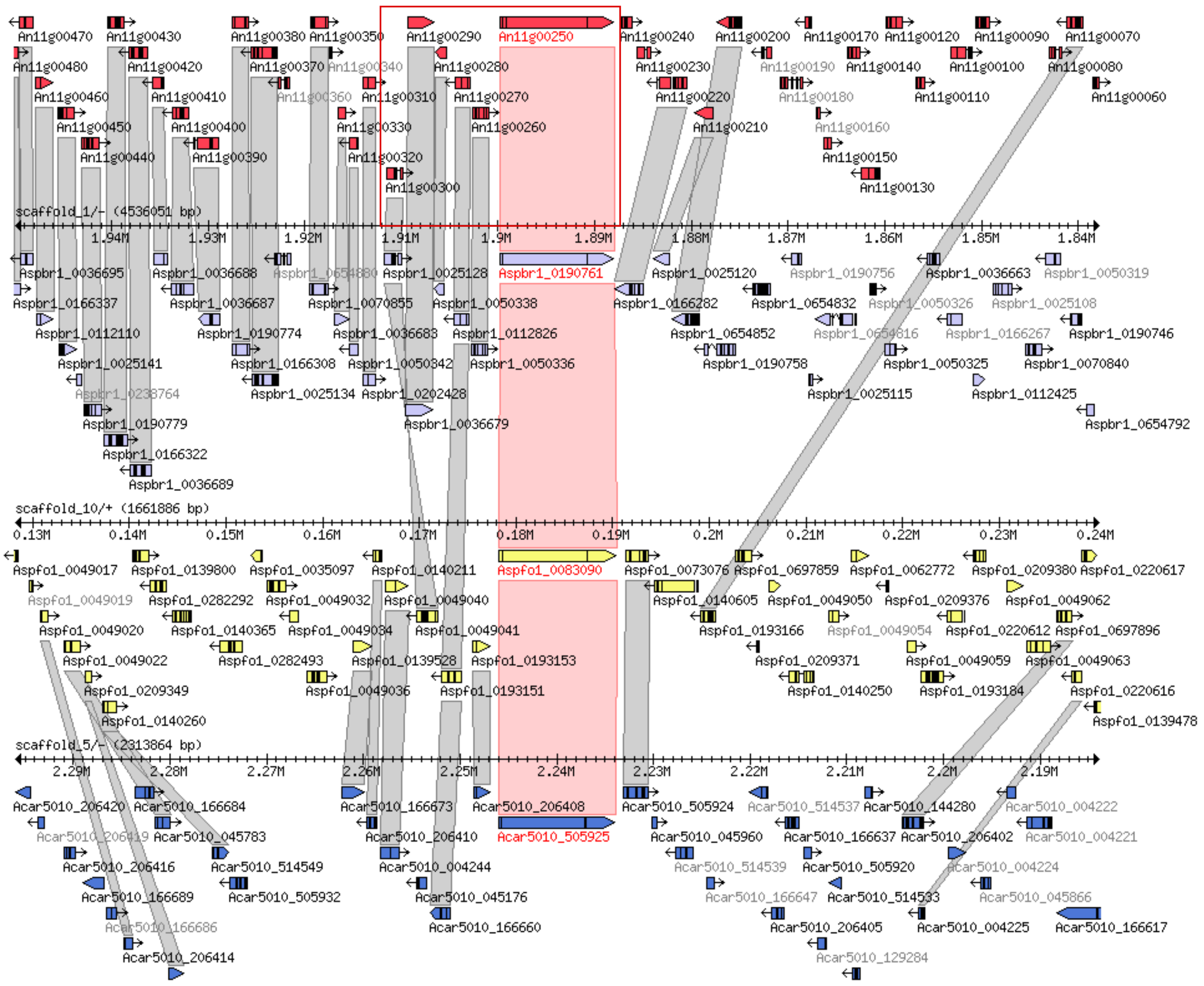

An11g00250 cluster

Gene id (SMURF)	Gene positions	Gene distance	AspGD name	AspGD description line or supplemental annotation	protein ID (antiSMASH)	AspGD ID	
An11g00130	12	116		Ortholog of A. brasiliensis : Aspbr1_0189338, A. acidus : Aspfo1_0052777, A. sydowii : Aspsy1_0079960 and Aspergillus carbonarius ITEM 5010 : Acar5010_163398, Acar5010_208606	n/a	An11g00130	
An11g00140	11	1617		Domain(s) with predicted DNA binding, zinc ion binding activity, role in transcription, DNA-dependent and nucleus localization	n/a	An11g00140	
An11g00150	10	299		Ortholog of A. brasiliensis : Aspbr1_0138759, A. acidus : Aspfo1_0075668, A. niger ATCC 1015 : 142878-mRNA and Aspergillus carbonarius ITEM 5010 : Acar5010_513321	n/a	An11g00150	
An11g00160	9	556			n/a	An11g00160	
An11g00170	8	295		Ortholog of A. niger CBS 513.88 : An01g05460, A. brasiliensis : Aspbr1_0027926, Aspbr1_0070040, A. acidus : Aspfo1_0065583, Aspfo1_0123243 and A. versicolor : Aspve1_0067140	prot_ID_652	An11g00170	
An11g00180	7	947			prot_ID_497	An11g00180	
An11g00190	6	1976		Domain(s) with predicted transmembrane transporter activity, role in transmembrane transport and integral to membrane localization	prot_ID_518	An11g00190	
An11g00200	5	472		Putative beta-glucosidase precursor	prot_ID_572	An11g00200	
An11g00210	4	979		Domain(s) with predicted N,N-dimethylaniline monooxygenase activity, NADP binding, flavin adenine dinucleotide binding activity and role in oxidation-reduction process	prot_ID_681	An11g00210	
An11g00220	3	604		Ortholog(s) have ferric-chelate reductase activity and role in cellular iron ion homeostasis, cellular response to iron ion starvation	prot_ID_840	An11g00220	
An11g00230	2	606		Domain(s) with predicted UDP-N-acetylmuramate dehydrogenase activity, flavin adenine dinucleotide binding activity and role in oxidation-reduction process	prot_ID_758	An11g00230	
An11g00240	1	609		Domain(s) with predicted nucleotide binding activity	prot_ID_388	An11g00240	
**An11g00250**	0	0		Putative polyketide synthase (PKS) - nonribosomal peptide synthase (NRPS) hybrid	**prot_ID_993**	**An11g00250**	ECS
An11g00260	-1	1201		Domain(s) with predicted role in transmembrane transport and integral to membrane localization	prot_ID_549	An11g00260	
An11g00270	-2	263		Protein similar to lanosterol 14 alpha-demethylase	prot_ID_682	An11g00270	
An11g00280	-3	957		Domain(s) with predicted O-methyltransferase activity	prot_ID_281	An11g00280	

An11g00290	-4	210	Putative polyketide synthase (PKS) - nonribosomal peptide synthase (NRPS) hybrid	prot_ID_124	An11g00290	
An11g00300	-5	522	Domain(s) with predicted monooxygenase activity and role in oxidation-reduction process	prot_ID_866	An11g00300	ECS
An11g00310	-6	1156	Domain(s) with predicted aspartic-type endopeptidase activity and role in proteolysis	prot_ID_535	An11g00310	
An11g00320	-7	439	Domain(s) with predicted hydrolase activity, acting on ester bonds activity and role in biosynthetic process	prot_ID_481	An11g00320	
An11g00330	-8	384	Domain(s) with predicted nucleotide binding activity	prot_ID_5	An11g00330	
An11g00340	-9	514	Questionable ORF	prot_ID_258	An11g00340	
An11g00350	-10	222	Domain(s) with predicted UDP-N-acetylmuramate dehydrogenase activity, flavin adenine dinucleotide binding activity and role in oxidation-reduction process	prot_ID_748	An11g00350	
An11g00360	-11	2098		n/a	An11g00360	
An11g00370	-12	52	Domain(s) with predicted DNA binding, sequence-specific DNA binding RNA polymerase II transcription factor activity, sequence-specific DNA binding transcription factor activity, zinc ion binding activity	n/a	An11g00370	
An11g00380	-13	319	Putative efflux pump	n/a	An11g00380	

An11g03920 cluster

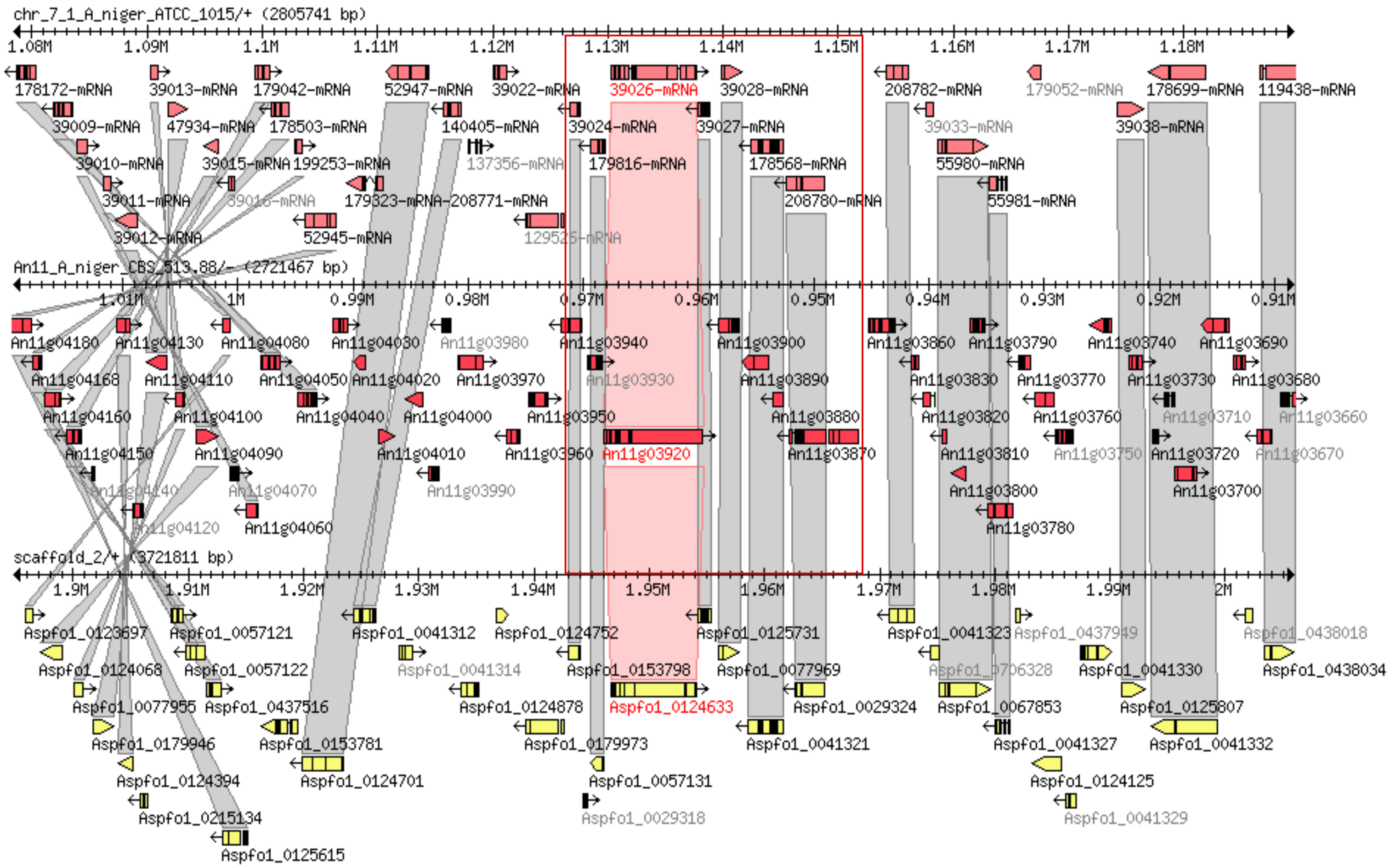

An11g03920 cluster

Gene id (SMURF)	Gene positions	Gene distance	AspGD name	AspGD description line or supplemental annotation	protein ID (antiSMASH)	AspGD ID	
An11g03750	14	112			n/a	An11g03750	
An11g03760	13	546		Ortholog of A. nidulans FGSC A4 : AN1864, A. fumigatus Af293 : Afu2g04535, A. oryzae RIB40 : AO090003000246, A. niger ATCC 1015 : 52941-mRNA and A. versicolor : Aspve1_0050093	n/a	An11g03760	
An11g03770	12	476		Ortholog of A. fumigatus Af293 : Afu2g04533, A. oryzae RIB40 : AO090003000245, A. niger ATCC 1015 : 179547-mRNA, A. versicolor : Aspve1_0125145 and A. sydowii : Aspsy1_0140007	n/a	An11g03770	
An11g03780	11	119		Domain(s) with predicted DNA binding, sequence-specific DNA binding RNA polymerase II transcription factor activity, sequence-specific DNA binding transcription factor activity, zinc ion binding activity	n/a	An11g03780	
An11g03790	10	380		Ortholog of A. nidulans FGSC A4 : AN8471, A. brasiliensis : Aspbr1_0166950, A. niger ATCC 1015 : 179041-mRNA, Aspergillus flavus NRRL 3357 : AFL2T_04215 and A. acidus : Aspfo1_0057088	n/a	An11g03790	
An11g03800	9	396		Domain(s) with predicted monooxygenase activity and role in oxidation-reduction process	n/a	An11g03800	
An11g03810	8	592		Ortholog of A. fumigatus Af293 : Afu3g13620, A. oryzae RIB40 : AO090023000589, A. niger ATCC 1015 : 38979-mRNA and A. versicolor : Aspve1_0045600, Aspve1_0050055	n/a	An11g03810	
An11g03820	7	401		Domain(s) with predicted FMN binding, riboflavin reductase (NADPH) activity and role in oxidation-reduction process	prot_ID_374	An11g03820	
An11g03830	6	1360		Ortholog of A. nidulans FGSC A4 : AN7883, AN4396, A. brasiliensis : Aspbr1_0113618, A. niger ATCC 1015 : 38981-mRNA and A. acidus : Aspfo1_0215105	prot_ID_8	An11g03830	
An11g03860	5	714		Domain(s) with predicted ATP binding, nucleoside-triphosphatase activity	prot_ID_272	An11g03860	
An11g03870	4	412		Ortholog of A. brasiliensis : Aspbr1_0025414, A. acidus : Aspfo1_0125007, A. niger ATCC 1015 : 38983-mRNA and Aspergillus carbonarius ITEM 5010 : Acar5010_204665	prot_ID_16	An11g03870	ECS
An11g03880	3	376		Domain(s) with predicted lyase activity and role in metabolic process	prot_ID_594	An11g03880	
An11g03890	2	277		Domain(s) with predicted DNA binding, sequence-specific DNA binding RNA polymerase II transcription factor activity, sequence-specific DNA binding transcription factor activity, zinc ion binding activity	prot_ID_135	An11g03890	

An11g03900	1	1208		Domain(s) with predicted role in transmembrane transport and integral to membrane localization	prot_ID_341	An11g03900	
**An11g03920**	0	0		Putative diketide synthase; ortholog(s) have role in asperfuranone biosynthetic process, emericellamide biosynthetic process, monodictyphenone biosynthetic process, o-orsellinic acid biosynthetic process, sterigmatocystin biosynthetic process	**prot_ID_187**	**An11g03920**	
An11g03930	-1	104		Putative dihydrofolate reductase	prot_ID_755	An11g03930	
An11g03940	-2	434		Domain(s) with predicted electron carrier activity, heme binding, iron ion binding, oxidoreductase activity, acting on paired donors, with incorporation or reduction of molecular oxygen activity and role in oxidation-reduction process	prot_ID_274	An11g03940	ECS
An11g03950	-3	1028		Ortholog of A. nidulans FGSC A4 : AN8025, A. fumigatus Af293 : Afu1g01450, A. niger CBS 513.88 : An11g08050, An05g01890 and A. oryzae RIB40 : AO090010000064, AO090701000602	prot_ID_709	An11g03950	
An11g03960	-4	728		Ortholog of A. nidulans FGSC A4 : AN6419, AN6946, AN5312, AN5664, AN8984, AN8971, AN9387, AN1317 and A. fumigatus Af293 : Afu5g13725	prot_ID_246	An11g03960	
An11g03970	-5	2281		Domain(s) with predicted ATP binding, nucleoside-triphosphatase activity	prot_ID_482	An11g03970	
An11g03980	-6	744			prot_ID_791	An11g03980	
An11g03990	-7	251			prot_ID_887	An11g03990	
An11g04000	-8	458		Domain(s) with predicted four-way junction helicase activity, nucleotide binding activity and role in DNA recombination, DNA repair	prot_ID_48	An11g04000	
An11g04010	-9	921		Domain(s) with predicted sequence-specific DNA binding RNA polymerase II transcription factor activity, sequence-specific DNA binding transcription factor activity, zinc ion binding activity	prot_ID_477	An11g04010	
An11g04020	-10	1228		Domain(s) with predicted hydrolase activity and role in metabolic process	n/a	An11g04020	
An11g04030	-11	452	pelC	Putative pectin lyase, involved in degradation of pectins	n/a	An11g04030	
An11g04040	-12	1360	pgxA	Putative exopolygalacturonase A; predicted signal peptide secretion sequence	n/a	An11g04040	

An11g04280 cluster

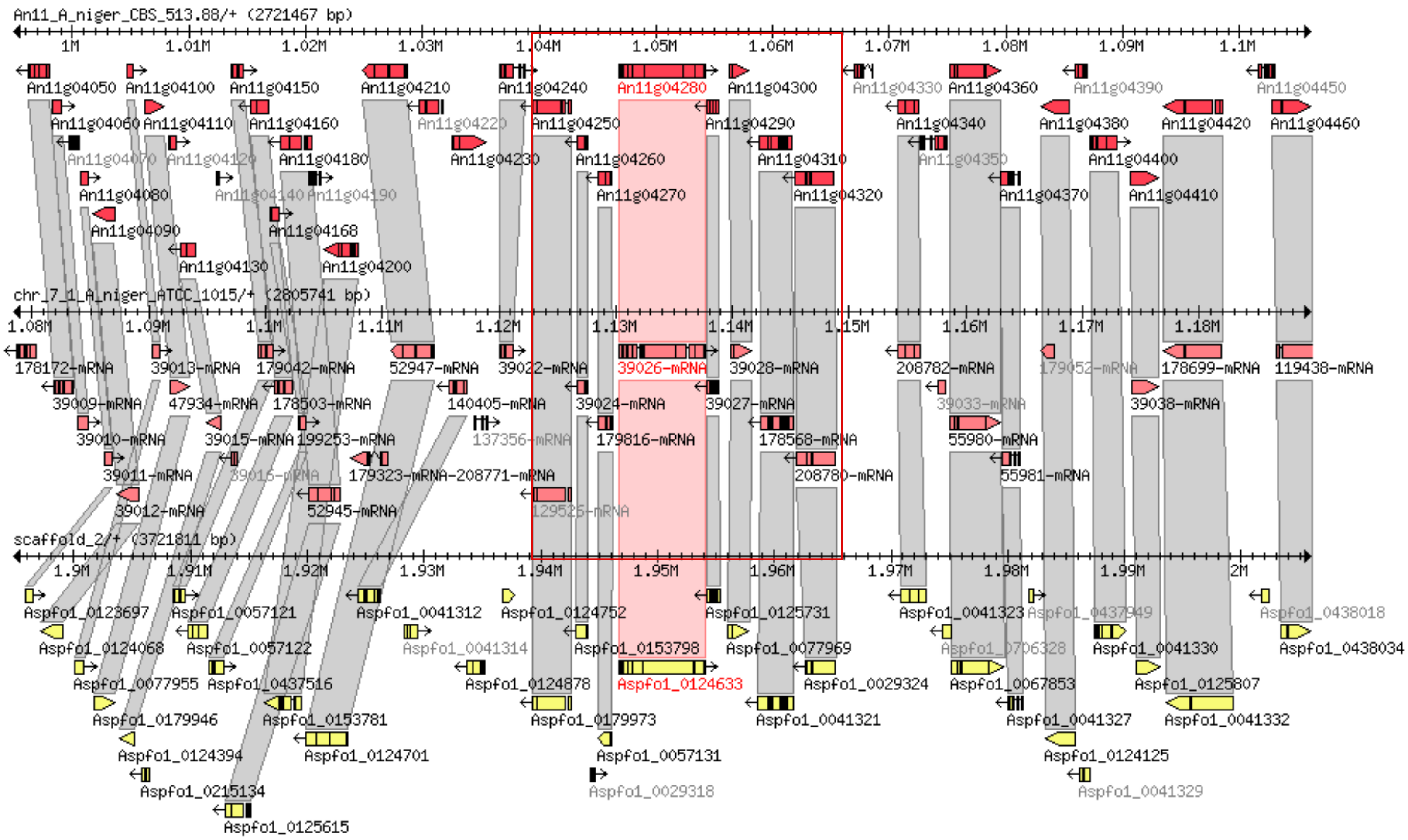

An11g04280 cluster

Gene id (SMURF)	Gene positions	Gene distance	AspGD name	AspGD description line or supplemental annotation	protein ID (antiSMASH)	AspGD ID	
An11g04140	14	875			n/a	An11g04140	
An11g04150	13	420		Putative alcohol dehydrogenase	n/a	An11g04150	
An11g04160	12	540		Ortholog(s) have role in maltose metabolic process, regulation of transcription, DNA-dependent	n/a	An11g04160	
An11g04168	11	288		Ortholog of A. nidulans FGSC A4 : AN11862, A. fumigatus Af293 : Afu2g04610, A. oryzae RIB40 : AO090003000258, A. niger ATCC 1015 : 199253-mRNA and A. sydowii : Aspsy1_0026483	n/a	An11g04168	
An11g04180	10	11	bipA	DnaK-type molecular chaperone; ER chaperone; unfolded protein response (UPR) target gene; expression enhanced by maltose, by heat shock, DTT and by tunicamycin treatment	prot_ID_569	An11g04180	
An11g04190	9	293			prot_ID_155	An11g04190	
An11g04200	8	588		Domain(s) with predicted GTP binding, GTPase activity	prot_ID_797	An11g04200	
An11g04210	7	1079		Domain(s) with predicted exonuclease activity, nucleic acid binding, ubiquitin thiolesterase activity, role in ubiquitin-dependent protein catabolic process and intracellular localization	prot_ID_581	An11g04210	
An11g04220	6	752		Domain(s) with predicted electron carrier activity, heme binding, iron ion binding, oxidoreductase activity, acting on paired donors, with incorporation or reduction of molecular oxygen activity and role in oxidation-reduction process	prot_ID_168	An11g04220	
An11g04230	5	1142		Domain(s) with predicted nucleoside-triphosphatase activity, nucleotide binding activity	prot_ID_718	An11g04230	
An11g04240	4	596		Ortholog of A. nidulans FGSC A4 : AN8532, A. brasiliensis : Aspbr1_0071123, A. niger ATCC 1015 : 39022-mRNA and Aspergillus flavus NRRL 3357 : AFL2T_09181	prot_ID_204	An11g04240	
An11g04250	3	493		L-aminoadipatesemialdehyde dehydrogenase	prot_ID_1077	An11g04250	ECS
An11g04260	2	933		Ortholog of A. nidulans FGSC A4 : AN11199, A. niger CBS 513.88 : An12g02720, A. niger ATCC 1015 : 39024-mRNA, A. versicolor : Aspve1_0053145 and A. sydowii : Aspsy1_0057345	prot_ID_485	An11g04260	
An11g04270	1	576		Domain(s) with predicted nucleotide binding, oxidoreductase activity, zinc ion binding activity and role in oxidation-reduction process	prot_ID_284	An11g04270	

**An11g04280**	0	0	Ortholog(s) have role in asperfuranone biosynthetic process, emericellamide biosynthetic process, monodictyphenone biosynthetic process, o-orsellinic acid biosynthetic process, sterigmatocystin biosynthetic process	**prot_ID_424**	**An11g04280**	
An11g04290	-1	100	Putative alcohol dehydrogenase	prot_ID_331	An11g04290	
An11g04300	-2	900	Ortholog of A. brasiliensis : Aspbr1_0167034, N. fischeri NRRL 181 : NFIA_108050, A. clavatus NRRL 1 : ACLA_047510, A. acidus : Aspfo1_0077969 and A. niger ATCC 1015 : 39028-mRNA	prot_ID_998	An11g04300	
An11g04310	-3	885	Domain(s) with predicted role in intracellular protein transport	prot_ID_1078	An11g04310	
n/a			Domain(s) with predicted ATP binding, protein serine/threonine kinase activity, small GTPase regulator activity and role in actin polymerization or depolymerization, protein complex assembly, protein phosphorylation	prot_ID_62	An11g04320	ECS
n/a				prot_ID_1000	An11g04330	
n/a			Domain(s) with predicted nucleobase transmembrane transporter activity, role in nucleobase transport and membrane localization	prot_ID_641	An11g04340	
n/a				prot_ID_552	An11g04350	

An11g05500 cluster

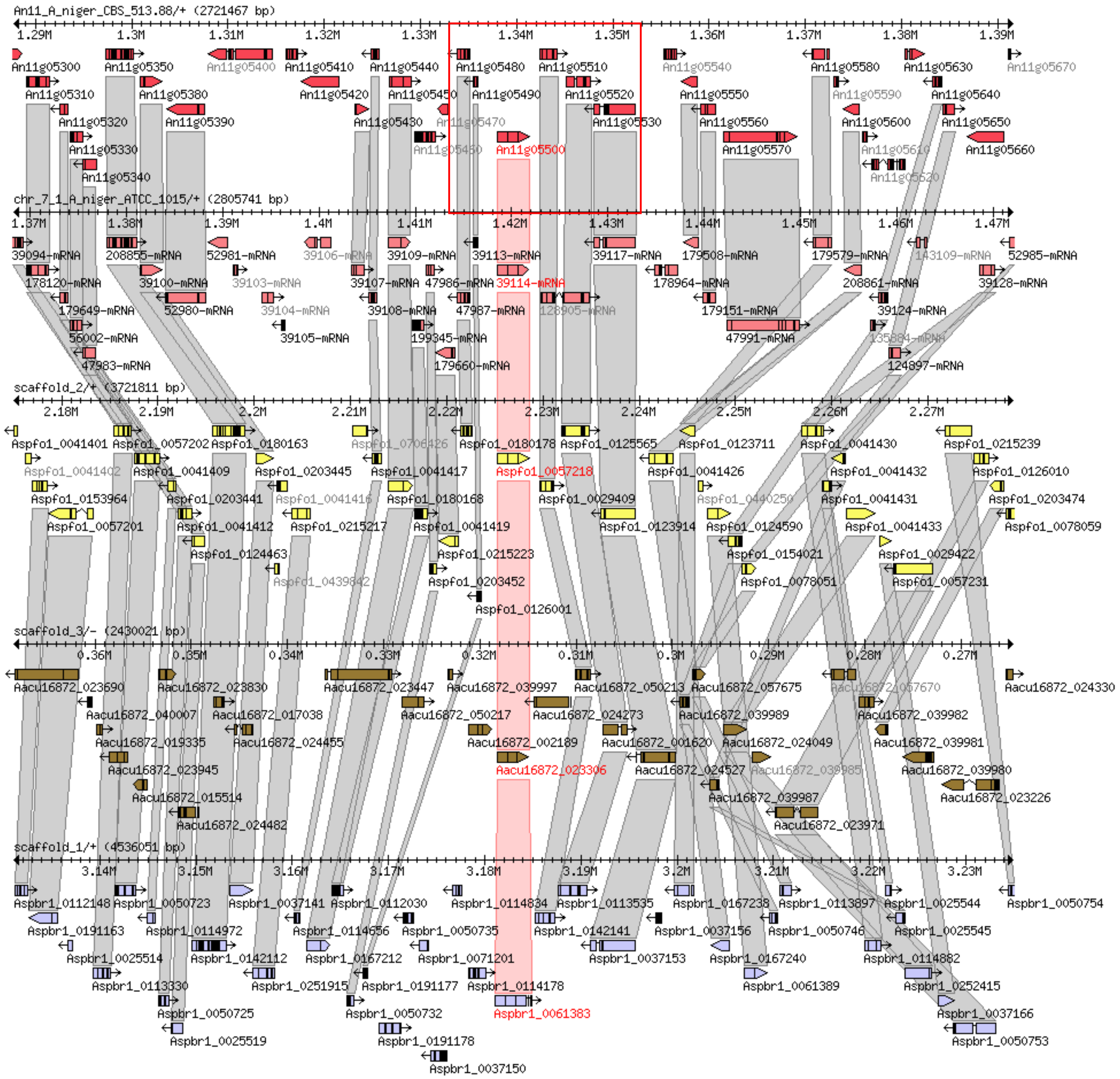

An11g05500 cluster

Gene id (SMURF)	Gene positions	Gene distance	AspGD name	AspGD description line or supplemental annotation	protein ID (antiSMASH)	AspGD ID	
n/a				Ortholog of A. oryzae RIB40 : AO090012000390, AO090009000464, A. brasiliensis : Aspbr1_0130524, N. fischeri NRRL 181 : NFIA_006040 and Aspergillus flavus NRRL 3357 : AFL2T_03297	prot_ID_88	An11g05420	
n/a				Ortholog of A. nidulans FGSC A4 : AN6764, AN2660, AN7798, A. fumigatus Af293 : Afu8g01960 and A. niger CBS 513.88 : An15g04120, An01g12050, An11g03480, An01g01140	prot_ID_72	An11g05430	
n/a				Domain(s) with predicted hydrolase activity	prot_ID_918	An11g05440	
n/a				Ortholog of A. nidulans FGSC A4 : AN10263, A. oryzae RIB40 : AO090023000304, A. niger ATCC 1015 : 39109-mRNA and Aspergillus terreus NIH2624 : ATET_05948	prot_ID_928	An11g05450	
n/a				Domain(s) with predicted actin binding activity, role in actin cytoskeleton organization and F-actin capping protein complex localization	prot_ID_647	An11g05460	
An11g05470	3	994		Protein of unknown function	prot_ID_503	An11g05470	
An11g05480	2	411		Domain(s) with predicted nucleotide binding activity	prot_ID_401	An11g05480	ECS
An11g05490	1	2080		Ortholog of A. brasiliensis : Aspbr1_0191177, A. acidus : Aspfo1_0126001, A. niger ATCC 1015 : 39113-mRNA and Aspergillus carbonarius ITEM 5010 : Acar5010_002287	prot_ID_705	An11g05490	
**An11g05500**	0	0		L-aminoadipatesemialdehyde dehydrogenase	**prot_ID_473**	**An11g05500**	
An11g05510	-1	1267		Domain(s) with predicted role in protein folding	prot_ID_896	An11g05510	
An11g05520	-2	1001		Domain(s) with predicted ATP binding, protein serine/threonine kinase activity and role in protein phosphorylation	prot_ID_39	An11g05520	
An11g05530	-3	1167		Domain(s) with predicted nucleic acid binding, zinc ion binding activity	prot_ID_680	An11g05530	ECS, IGD
An11g05540	-4	2833			prot_ID_817	An11g05540	
An11g05550	-5	450		Domain(s) with predicted role in transmembrane transport and integral to membrane localization	prot_ID_739	An11g05550	
An11g05560	-6	388		8-Amino-7-oxononanoate synthase	prot_ID_282	An11g05560	
An11g05570	-7	947		Domain(s) with predicted ACP phosphopantetheine attachment site binding involved in fatty acid biosynthetic process, nucleotide binding, oxidoreductase activity and transferase activity, more	n/a	An11g05570	

An11g05570 cluster

No manual prediction made

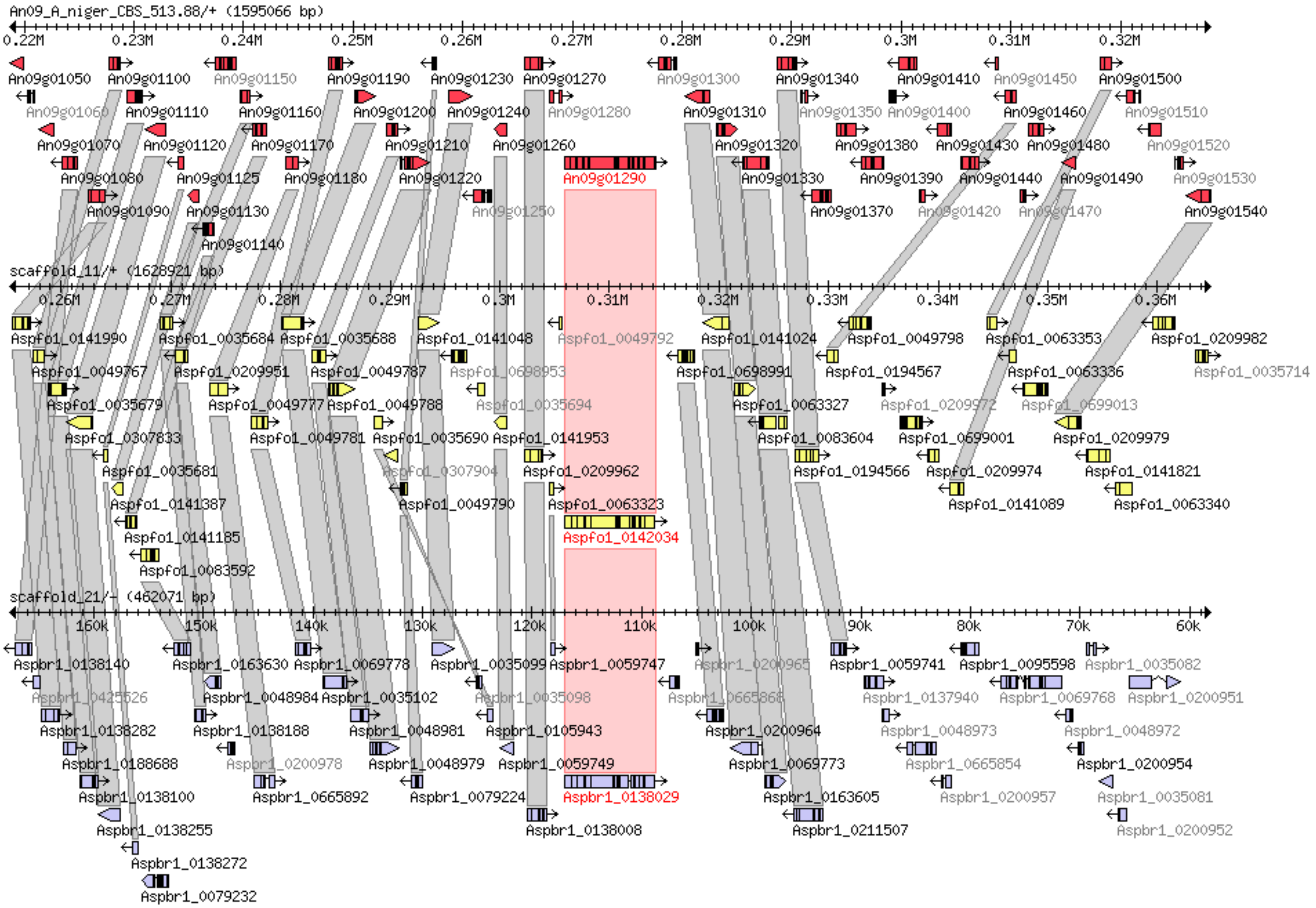

An11g05570 cluster

Gene id (SMURF)	Gene positions	Gene distance	AspGD name	AspGD description line or supplemental annotation	protein ID (antiSMASH)	AspGD ID
An11g05440	13	1190		Domain(s) with predicted hydrolase activity	n/a	An11g05440
An11g05450	12	418		Ortholog of A. nidulans FGSC A4 : AN10263, A. oryzae RIB40 : AO090023000304, A. niger ATCC 1015 : 39109-mRNA and Aspergillus terreus NIH2624 : ATET_05948	n/a	An11g05450
An11g05460	11	108		Domain(s) with predicted actin binding activity, role in actin cytoskeleton organization and F-actin capping protein complex localization	n/a	An11g05460
An11g05470	10	994			n/a	An11g05470
An11g05480	9	411		Domain(s) with predicted nucleotide binding activity	n/a	An11g05480
An11g05490	8	2080		Ortholog of A. brasiliensis : Aspbr1_0191177, A. acidus : Aspfo1_0126001, A. niger ATCC 1015 : 39113-mRNA and Aspergillus carbonarius ITEM 5010 : Acar5010_002287	n/a	An11g05490
An11g05500	7	1267		L-aminoadipatesemialdehyde dehydrogenase	n/a	
An11g05510	6	1001		Domain(s) with predicted role in protein folding	prot_ID_896	An11g05510
An11g05520	5	1167		Domain(s) with predicted ATP binding, protein serine/threonine kinase activity and role in protein phosphorylation	prot_ID_39	An11g05520
An11g05530	4	2833		Domain(s) with predicted nucleic acid binding, zinc ion binding activity	prot_ID_680	An11g05530
An11g05540	3	450			prot_ID_817	An11g05540
An11g05550	2	388		Domain(s) with predicted role in transmembrane transport and integral to membrane localization	prot_ID_739	An11g05550
An11g05560	1	947		8-Amino-7-oxononanoate synthase	prot_ID_282	An11g05560
**An11g05570**	0	0		Domain(s) with predicted ACP phosphopantetheine attachment site binding involved in fatty acid biosynthetic process, nucleotide binding, oxidoreductase activity and transferase activity, more	**prot_ID_143**	**An11g05570**
An11g05580	-1	1376		Domain(s) with predicted choline dehydrogenase activity, flavin adenine dinucleotide binding activity and role in alcohol metabolic process, oxidation-reduction process	prot_ID_729	An11g05580
An11g05590	-2	478			prot_ID_690	An11g05590
n/a				Domain(s) with predicted choline dehydrogenase activity, flavin adenine dinucleotide binding activity and role in alcohol metabolic process, oxidation-reduction process	prot_ID_56	An11g05600
n/a					prot_ID_342	An11g05610
n/a					prot_ID_94	An11g05620

n/a	Ortholog of A. clavatus NRRL 1 : ACLA_044140	prot_ID_1071	An11g05630
n/a	Domain(s) with predicted methyltransferase activity and role in 7-methylguanosine RNA capping, RNA methylation	prot_ID_966	An11g05640
n/a	Ortholog of A. fumigatus Af293 : Afu6g08620, A. oryzae RIB40 : AO090001000523, A. versicolor : Aspve1_0132571, A. sydowii : Aspsy1_0149010 and Aspergillus terreus NIH2624 : ATET_06603	prot_ID_478	An11g05650
n/a	Ortholog of A. nidulans FGSC A4 : AN9003, A. versicolor : Aspve1_0104265 and A. sydowii : Aspsy1_0093599	prot_ID_35	An11g05660

An11g05940/ An11g05960 cluster

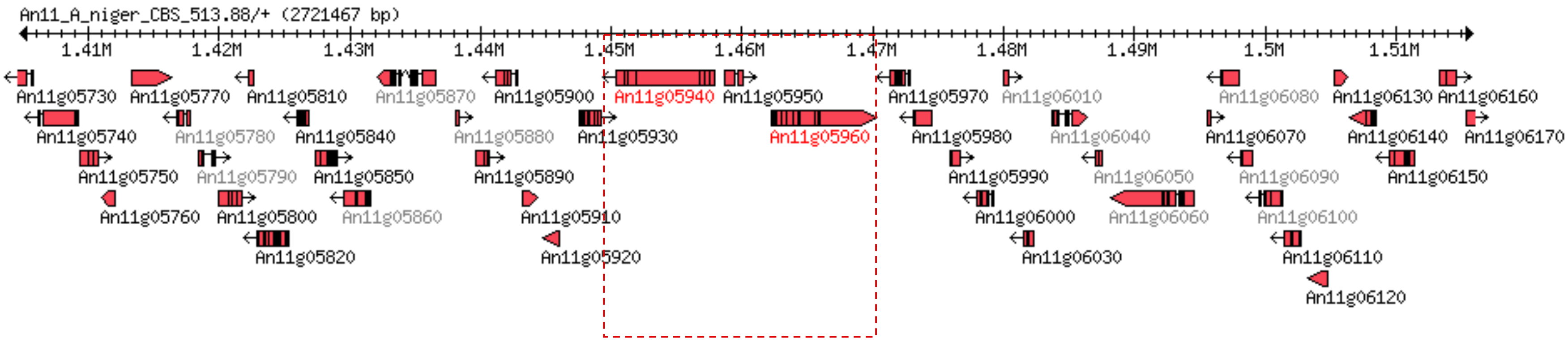

An11g05940/ An11g05960 cluster

Gene id (SMURF)	Gene positions	Gene distance	AspGD name	AspGD description line or supplemental annotation	protein ID (antiSMASH)	AspGD ID	
An11g05820	11	705		Domain(s) with predicted DNA binding, zinc ion binding activity, role in transcription, DNA-dependent and nucleus localization	n/a	An11g05820	
An11g05840	10	497		Domain(s) with predicted nucleotide binding, oxidoreductase activity and role in oxidation-reduction process	n/a	An11g05840	
An11g05850	9	446		Domain(s) with predicted UDP-N-acetylmuramate dehydrogenase activity, flavin adenine dinucleotide binding activity and role in oxidation-reduction process	n/a	An11g05850	
An11g05860	8	491		Domain(s) with predicted cation binding, hydrolase activity, hydrolyzing O-glycosyl compounds activity and role in carbohydrate metabolic process	prot_ID_765	An11g05860	
An11g05870	7	1501			prot_ID_674	An11g05870	
An11g05880	6	1243			prot_ID_403	An11g05880	
An11g05890	5	317		Ortholog of A. brasiliensis : Aspbr1_0191213, A. acidus : Aspfo1_0029439 and A. niger ATCC 1015 : 39144-mRNA	prot_ID_470	An11g05890	
An11g05900	4	445		Ortholog of A. fumigatus Af293 : Afu3g02680, Afu8g01830, A. niger CBS 513.88 : An01g03400 and A. oryzae RIB40 : AO090102000007, AO090003000344, AO090001000755	prot_ID_1027	An11g05900	
An11g05910	3	400		Domain(s) with predicted nucleotide binding, oxidoreductase activity, zinc ion binding activity and role in oxidation-reduction process	prot_ID_173	An11g05910	
An11g05920	2	1502		Domain(s) with predicted hydrolase activity	prot_ID_568	An11g05920	
An11g05930	1	909		Domain(s) with predicted role in transmembrane transport and integral to membrane localization	prot_ID_22	An11g05930	
**An11g05940**	0	0		Putative polyketide synthase (PKS)	**prot_ID_977**	**An11g05940**	ECS
An11g05950	-1	752		Ortholog of A. acidus : Aspfo1_0057351 and Aspergillus terreus NIH2624 : ATET_07281	prot_ID_888	An11g05950	
An11g05960	-2	2140		Ortholog(s) have role in asperfuranone biosynthetic process, emericellamide biosynthetic process, monodictyphenone biosynthetic process, o-orsellinic acid biosynthetic process, sterigmatocystin biosynthetic process	prot_ID_1052	An11g05960	ECS
n/a				Domain(s) with predicted role in cell wall macromolecule catabolic process	prot_ID_1062	An11g05970	

n/a	Ortholog of A. fumigatus Af293 : Afu6g09800, A. brasiliensis : Aspbr1_0167299, N. fischeri NRRL 181 : NFIA_069810, A. acidus : Aspfo1_0041459 and A. versicolor : Aspve1_0656122	prot_ID_812	An11g05980
n/a	Ortholog of A. brasiliensis : Aspbr1_0199302, A. acidus : Aspfo1_0049693, Aspfo1_0125316, A. versicolor : Aspve1_0089146 and A. sydowii : Aspsy1_0050561	prot_ID_810	An11g05990
n/a	Domain(s) with predicted ATP binding, protein kinase activity and role in protein phosphorylation	prot_ID_609	An11g06000
n/a		prot_ID_44	An11g06010
n/a	Ortholog of A. niger ATCC 1015 : 39155-mRNA and Aspergillus aculeatus ATCC16872 : Aacu16872_054439	prot_ID_773	An11g06030
n/a	Domain(s) with predicted phosphotransferase activity, alcohol group as acceptor activity	prot_ID_677	An11g06040
n/a		prot_ID_953	An11g06050
n/a		prot_ID_613	An11g06060

An11g06460 cluster

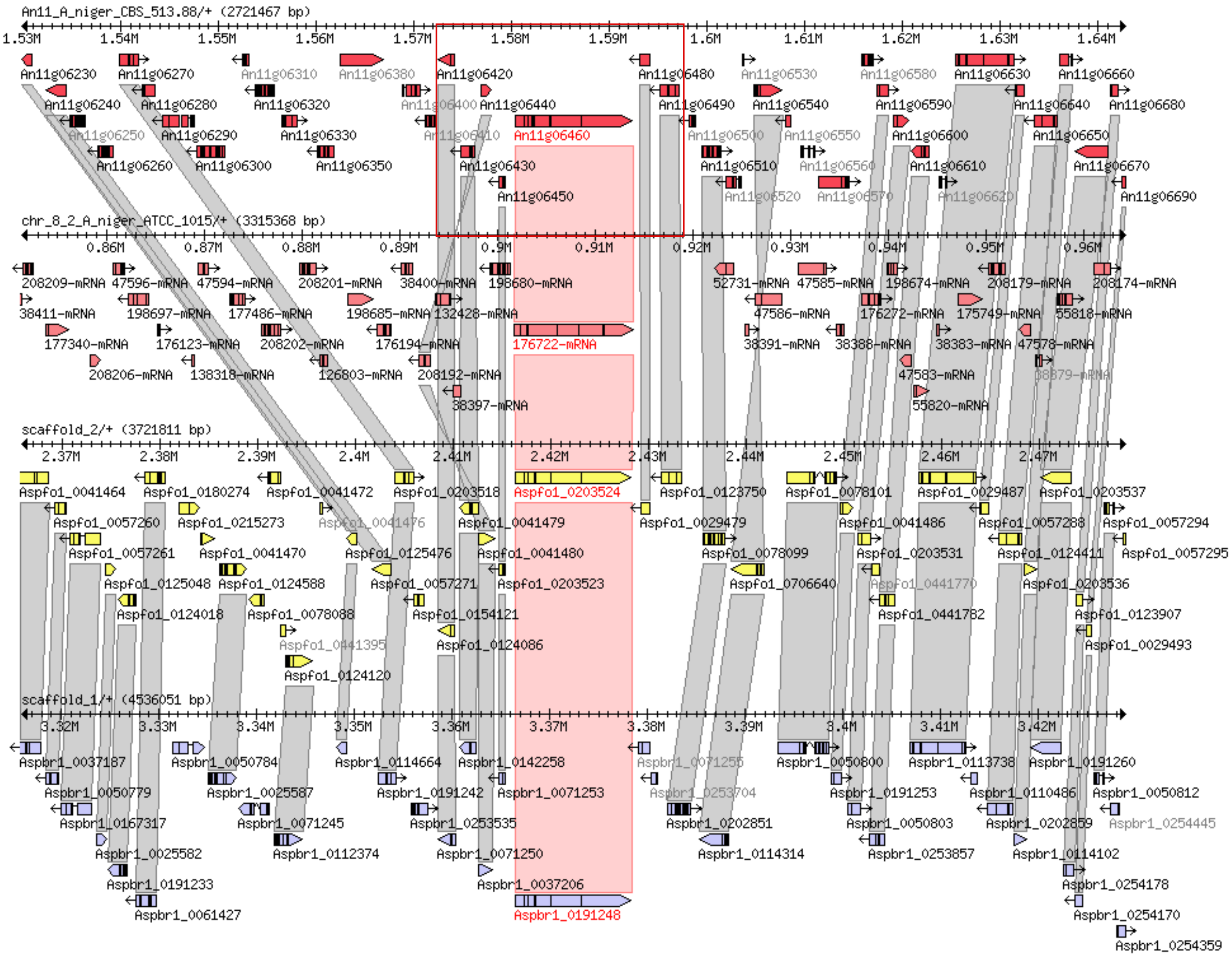

An11g06460 cluster

Gene id (SMURF)	Gene positions	Gene distance	AspGD name	AspGD description line or supplemental annotation	protein ID (antiSMASH)	AspGD ID	
n/a				Putative acid protease	prot_ID_399	An11g06350	
n/a				Ortholog(s) have mitochondrion localization	prot_ID_235	An11g06380	
n/a					prot_ID_573	An11g06400	
n/a					prot_ID_197	An11g06410	
An11g06420	4	718		Domain(s) with predicted role in transmembrane transport and integral to membrane localization	prot_ID_659	An11g06420	ECS
An11g06430	3	622		Ortholog of A. niger CBS 513.88 : An09g02320, A. oryzae RIB40 : AO090001000002, A. brasiliensis : Aspbr1_0062833, Aspbr1_0142258 and Aspergillus flavus NRRL 3357 : AFL2T_04900	prot_ID_247	An11g06430	
An11g06440	2	813		Domain(s) with predicted nucleotide binding, oxidoreductase activity, zinc ion binding activity and role in oxidation-reduction process	prot_ID_592	An11g06440	
An11g06450	1	1027		Ortholog of A. nidulans FGSC A4 : AN1088, A. fumigatus Af293 : Afu4g14470, Afu8g00430, A. niger CBS 513.88 : An07g00070, An07g00010, An02g08300 and A. oryzae RIB40 : AO090001000323	prot_ID_842	An11g06450	
**An11g06460**	0	0		Ortholog(s) have role in pseurotin A biosynthetic process	**prot_ID_449**	**An11g06460**	
An11g06480	-1	730		Ortholog of A. acidus : Aspfo1_0029479 and A. niger ATCC 1015 : 39189-mRNA	prot_ID_308	An11g06480	
An11g06490	-2	1076		Ortholog of A. niger CBS 513.88 : An18g01440, A. oryzae RIB40 : AO090005000944, A. versicolor : Aspve1_0145746, A. sydowii : Aspsy1_0085817 and Aspergillus terreus NIH2624 : ATET_05225	prot_ID_608	An11g06490	ECS
n/a				Protein of unknown function	prot_ID_321	An11g06500	
n/a				Domain(s) with predicted N,N-dimethylaniline monooxygenase activity, NADP binding, flavin adenine dinucleotide binding activity and role in oxidation-reduction process	prot_ID_217	An11g06510	
n/a				Protein of unknown function	prot_ID_949	An11g06520	
n/a				Putative retrotransposable element remnant	prot_ID_623	An11g06530	
n/a			mndA	Beta-mannosidase; glycosyl hydrolase family 2; contains several putative N-glycosylation sites	prot_ID_97	An11g06540	
n/a					prot_ID_639	An11g06550	
n/a					prot_ID_436	An11g06560	
n/a					prot_ID_139	An11g06570	

An11g07310 cluster

No ATCC 1015 orthologous cluster

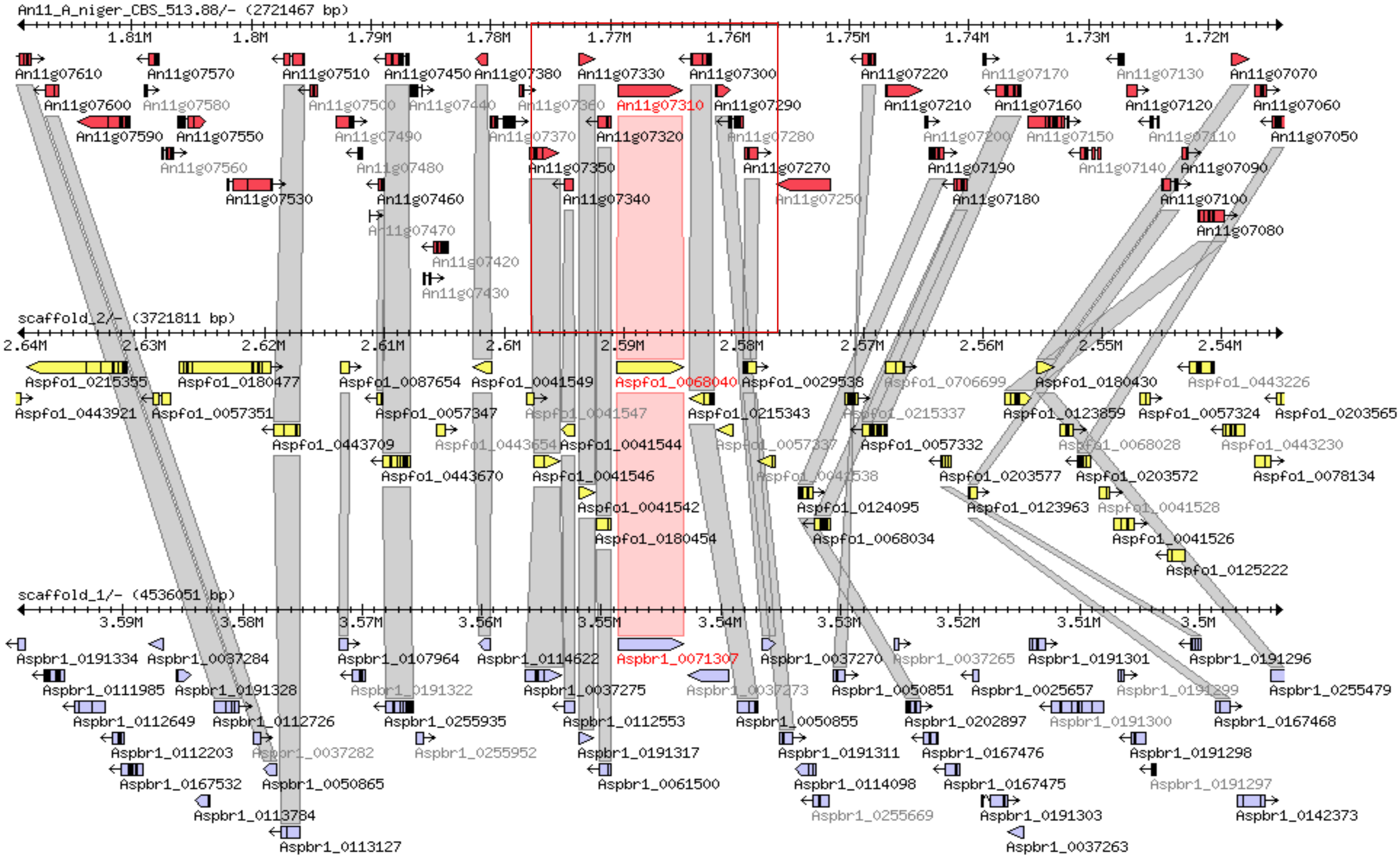

An11g07310 cluster

Gene id (SMURF)	Gene positions	Gene distance	AspGD name	AspGD description line or supplemental annotation	protein ID (antiSMASH)	AspGD ID	
n/a				Putative ankyrin	prot_ID_1081	An11g07210	
n/a				Ortholog of A. nidulans FGSC A4 : AN2023, A. niger CBS 513.88 : An18g00750, An16g04060, A. oryzae RIB40 : AO090001000252, AO090701000624 and A. niger ATCC 1015 : 179170-mRNA, 187809-mRNA, 210569-mRNA	prot_ID_565	An11g07220	
n/a				Ortholog(s) have mitochondrion localization	prot_ID_166	An11g07250	
n/a				Domain(s) with predicted NAD binding, oxidoreductase activity, acting on the CH-OH group of donors, NAD or NADP as acceptor activity and role in oxidation-reduction process	prot_ID_644	An11g07270	ECS
An11g07280	3	117		Transmembrane transporter	prot_ID_614	An11g07280	
An11g07290	2	597		Domain(s) with predicted nucleotidyltransferase activity	prot_ID_532	An11g07290	
An11g07300	1	663		Transmembrane transporter	prot_ID_366	An11g07300	
**An11g07310**	0	0		Polyketide synthase	**prot_ID_99**	**An11g07310**	
An11g07320	-1	640		Hydroxyacylglutathione hydrolase (glyoxylase II)	prot_ID_801	An11g07320	
An11g07330	-2	375		Ortholog(s) have role in asperthecin biosynthetic process, secondary metabolite biosynthetic process	prot_ID_511	An11g07330	
An11g07340	-3	467		Protein similar to O-methyl transferases; expression repressed by tunicamycin and DTT	prot_ID_367	An11g07340	
An11g07350	-4	410		Transcription factor	prot_ID_14	An11g07350	ECS
n/a					prot_ID_563	An11g07360	
n/a					prot_ID_545	An11g07370	
n/a				Domain(s) with predicted catalytic activity and role in biosynthetic process	prot_ID_317	An11g07380	
n/a					prot_ID_738	An11g07420	
n/a					prot_ID_544	An11g07430	
n/a					prot_ID_848	An11g07440	
n/a				Domain(s) with predicted ATP binding, protein serine/threonine kinase activity and role in protein phosphorylation	prot_ID_806	An11g07450	
n/a				Ortholog of A. acidus : Aspfo1_0057347	prot_ID_655	An11g07460	

An11g09720 cluster

Not typical secondary metabolism annotations, no manual prediction made

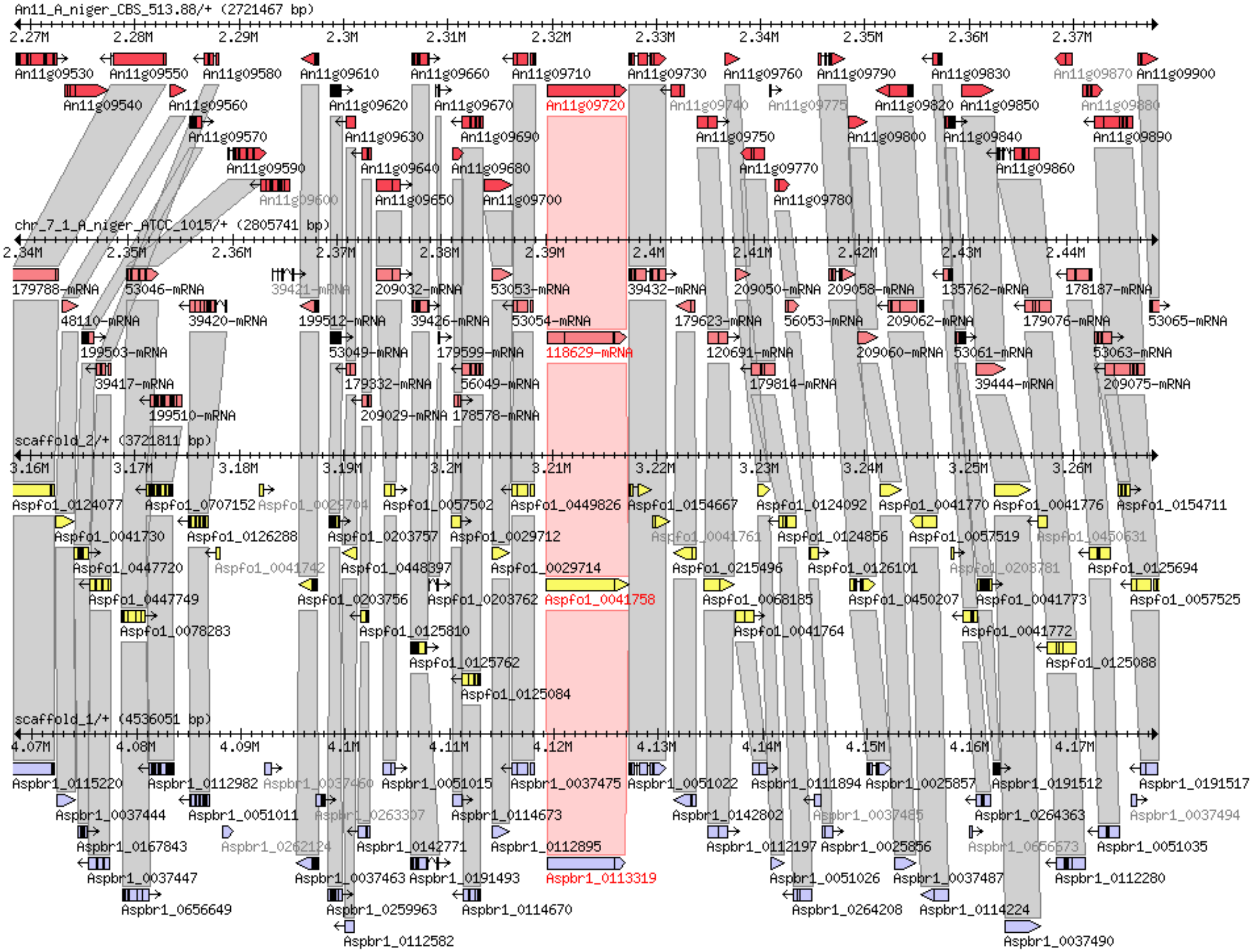

An11g09720 cluster

Gene id (SMURF)	Gene positions	Gene distance	AspGD name	AspGD description line or supplemental annotation	protein ID (antiSMASH)	AspGD ID
					n/a	An11g09610
n/a			rhoC	Rho GTPase	prot_ID_1072	An11g09620
n/a				Domain(s) with predicted octanoyltransferase activity, role in cellular protein modification process, lipoate biosynthetic process and cytoplasm localization	prot_ID_871	An11g09630
n/a				Domain(s) with predicted FMN binding, pyridoxamine-phosphate oxidase activity and role in oxidation-reduction process	prot_ID_734	An11g09640
n/a				Ortholog of A. nidulans FGSC A4 : AN4779, A. fumigatus Af293 : Afu3g06660, A. oryzae RIB40 : AO090020000332, A. niger ATCC 1015 : 209032-mRNA and A. versicolor : Aspve1_0051759	prot_ID_630	An11g09650
n/a				Domain(s) with predicted phosphotransferase activity, for other substituted phosphate groups activity, role in phospholipid biosynthetic process and membrane localization	prot_ID_704	An11g09660
n/a				Ortholog(s) have structural constituent of ribosome activity	prot_ID_119	An11g09670
n/a				Ortholog of A. nidulans FGSC A4 : AN4776, A. fumigatus Af293 : Afu3g06620, A. niger CBS 513.88 : An18g00880, A. oryzae RIB40 : AO090020000337 and A. niger ATCC 1015 : 178578-mRNA	prot_ID_929	An11g09680
n/a				Ortholog of A. nidulans FGSC A4 : AN4775, A. fumigatus Af293 : Afu3g06610, A. oryzae RIB40 : AO090020000338, A. niger ATCC 1015 : 56049-mRNA and A. versicolor : Aspve1_0582820	prot_ID_733	An11g09690
An11g09700	2	301	met1	Siroheme synthase; involved in siroheme biosythesis	prot_ID_992	An11g09700
An11g09710	1	1161		Domain(s) with predicted catalytic activity and role in metabolic process	prot_ID_1036	An11g09710
**An11g09720**	0	0		Polyketide synthase	**prot_ID_707**	**An11g09720**
An11g09730	-1	342		Domain(s) with predicted deoxyribose-phosphate aldolase activity, role in deoxyribonucleotide catabolic process and cytoplasm localization	prot_ID_783	An11g09730
An11g09740	-2	545			prot_ID_837	An11g09740

n/a		Ortholog of A. nidulans FGSC A4 : AN9485, A. fumigatus Af293 : Afu3g06570, A. niger ATCC 1015 : 120691-mRNA, A. versicolor : Aspve1_0027334 and A. sydowii : Aspsy1_0087842	prot_ID_240	An11g09750
n/a		Domain(s) with predicted 4 iron, 4 sulfur cluster binding, lipoate synthase activity and role in lipoate biosynthetic process	prot_ID_15	An11g09760
n/a		Phosphoserine phosphatase	prot_ID_715	An11g09770
n/a			prot_ID_335	An11g09775
n/a		PAPS reductase	prot_ID_551	An11g09780
n/a	sC	ATP sulfurylase	prot_ID_464	An11g09790

An12g02670 cluster

Overlaps An12g02730 cluster

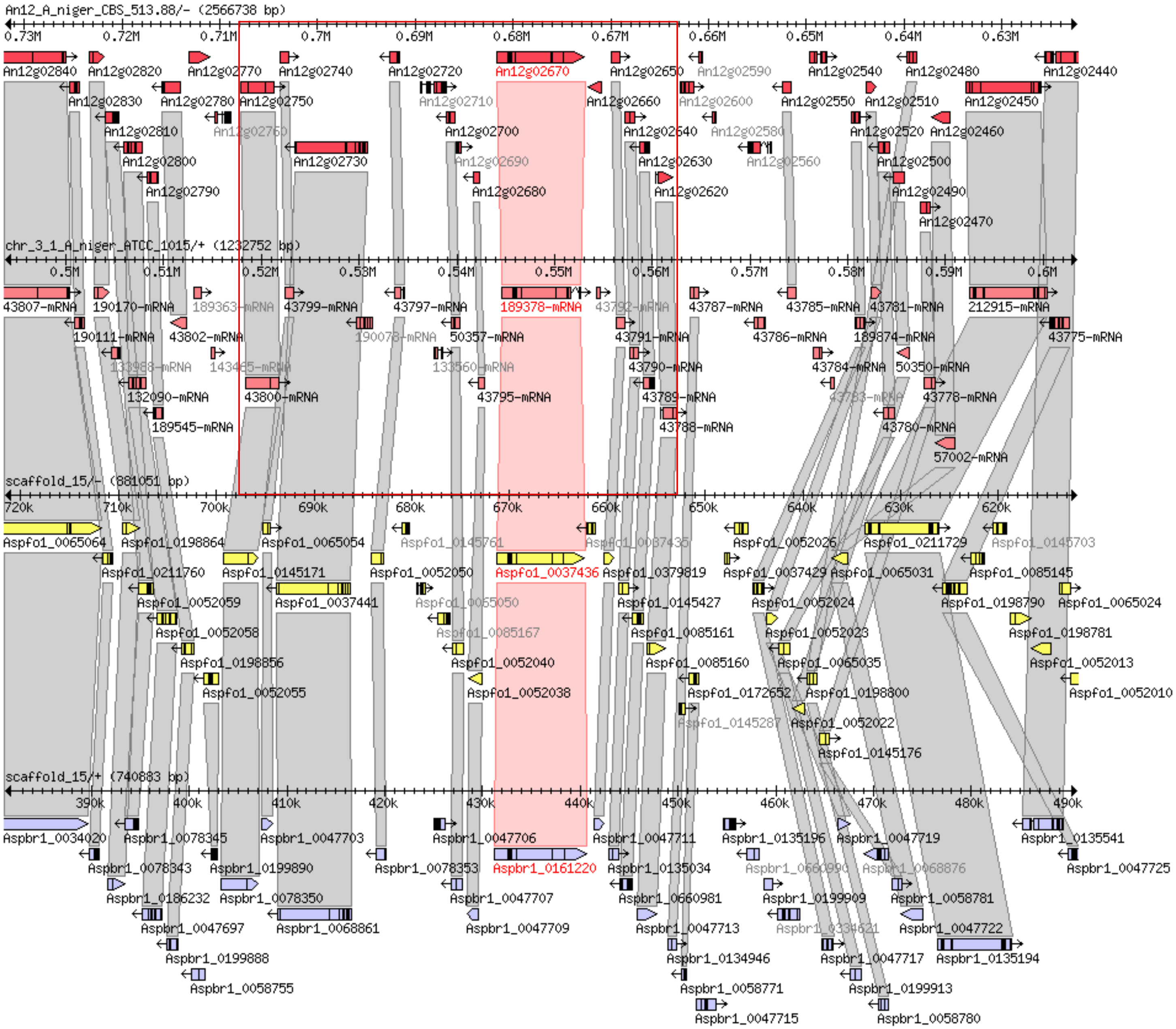

An12g02670 cluster

Gene id (SMURF)	Gene positions	Gene distance	AspGD name	AspGD description line or supplemental annotation	protein ID (antiSMASH)	AspGD ID	
n/a					prot_ID_75	An12g02560	
n/a					prot_ID_396	An12g02580	
n/a					prot_ID_397	An12g02590	
n/a					prot_ID_165	An12g02600	
n/a				Ortholog of A. nidulans FGSC A4 : AN6322, AN0902, A. fumigatus Af293 : Afu1g01340, Afu1g15680, Afu3g01640 and A. niger CBS 513.88 : An01g14020, An08g01460, An11g09210, An11g04710, An05g01040, An05g01340, An04g04080	prot_ID_411	An12g02620	ECS
n/a				Domain(s) with predicted nucleotide binding activity	prot_ID_285	An12g02630	
n/a				Oxidoreductase	prot_ID_242	An12g02640	
An12g02650	2	941		Domain(s) with predicted methyltransferase activity and role in metabolic process	prot_ID_41	An12g02650	
An12g02660	1	247		Ortholog of Aspergillus aculeatus ATCC16872 : Aacu16872_059428	prot_ID_1006	An12g02660	
**An12g02670**	0	0		Multifunctional polyketide synthase	**prot_ID_565**	**An12g02670**	
An12g02680	-1	1828		Ortholog of A. nidulans FGSC A4 : AN11765, A. fumigatus Af293 : Afu3g07625, A. niger CBS 513.88 : An02g12900, An04g10150 and A. oryzae RIB40 : AO090020000130, AO090023000386	prot_ID_600	An12g02680	
An12g02690	-2	1198		Domain(s) with predicted heme binding activity	prot_ID_17	An12g02690	
An12g02700	-3	125		Oxidoreductase	prot_ID_521	An12g02700	
An12g02710	-4	77		Putative transporter	prot_ID_868	An12g02710	
An12g02720	-5	2152		Ortholog of A. nidulans FGSC A4 : AN11199, A. niger CBS 513.88 : An11g04260, A. niger ATCC 1015 : 39024-mRNA, A. versicolor : Aspve1_0053145 and A. sydowii : Aspsy1_0057345	prot_ID_846	An12g02720	
An12g02730	-6	2325		Multifunctional polyketide synthase	prot_ID_734	An12g02730	
An12g02740	-7	535		Ortholog of A. brasiliensis : Aspbr1_0047703, Aspergillus flavus NRRL 3357 : AFL2T_11821, A. acidus : Aspfo1_0065054 and Aspergillus terreus NIH2624 : ATET_02827	prot_ID_975	An12g02740	
An12g02750	-8	475		Putative polyketide synthase	prot_ID_139	An12g02750	ECS
An12g02760	-9	1081		Domain(s) with predicted carbon-sulfur lyase activity and role in metabolic process	prot_ID_1000	An12g02760	
An12g02770	-10	404		Ortholog of Aspergillus terreus NIH2624 : ATET_09243	prot_ID_410	An12g02770	

An12g02780	-11	739	Domain(s) with predicted carbohydrate binding, metallopeptidase activity, zinc ion binding activity and role in cell adhesion, proteolysis	prot_ID_645	An12g02780
An12g02790	-12	347	Domain(s) with predicted nucleotide binding activity	prot_ID_1015	An12g02790
An12g02800	-13	507	Domain(s) with predicted role in transmembrane transport and integral to membrane localization	prot_ID_273	An12g02800
An12g02810	-14	599	Domain(s) with predicted O-methyltransferase activity	prot_ID_21	An12g02810
An12g02820	-15	98	Domain(s) with predicted role in transmembrane transport and integral to membrane localization	prot_ID_175	An12g02820
An12g02830	-16	1107	Ortholog(s) have role in austinol biosynthetic process, dehydroaustinol biosynthetic process, secondary metabolite biosynthetic process	prot_ID_469	An12g02830
n/a			Ortholog(s) have role in brevianamide F biosynthetic process, emericellamide biosynthetic process, pathogenesis, secondary metabolite biosynthetic process, spore germination	prot_ID_804	An12g02840
n/a			Thioredoxin reductase	prot_ID_182	An12g02850
n/a			Domain(s) with predicted role in transmembrane transport and integral to membrane localization	prot_ID_322	An12g02860
n/a				prot_ID_1023	An12g02870
n/a			Domain(s) with predicted DNA binding, sequence-specific DNA binding RNA polymerase II transcription factor activity, sequence-specific DNA binding transcription factor activity, zinc ion binding activity	prot_ID_1017	An12g02880
n/a			Branched chain amino acid aminotransferase	prot_ID_729	An12g02890
n/a			Ortholog of A. niger ATCC 1015 : 54493-mRNA	prot_ID_964	An12g02900
n/a				prot_ID_278	An12g02910
n/a			Putative chitinase	prot_ID_462	An12g02920

An12g02730 cluster

Overlaps An12g02670 cluster

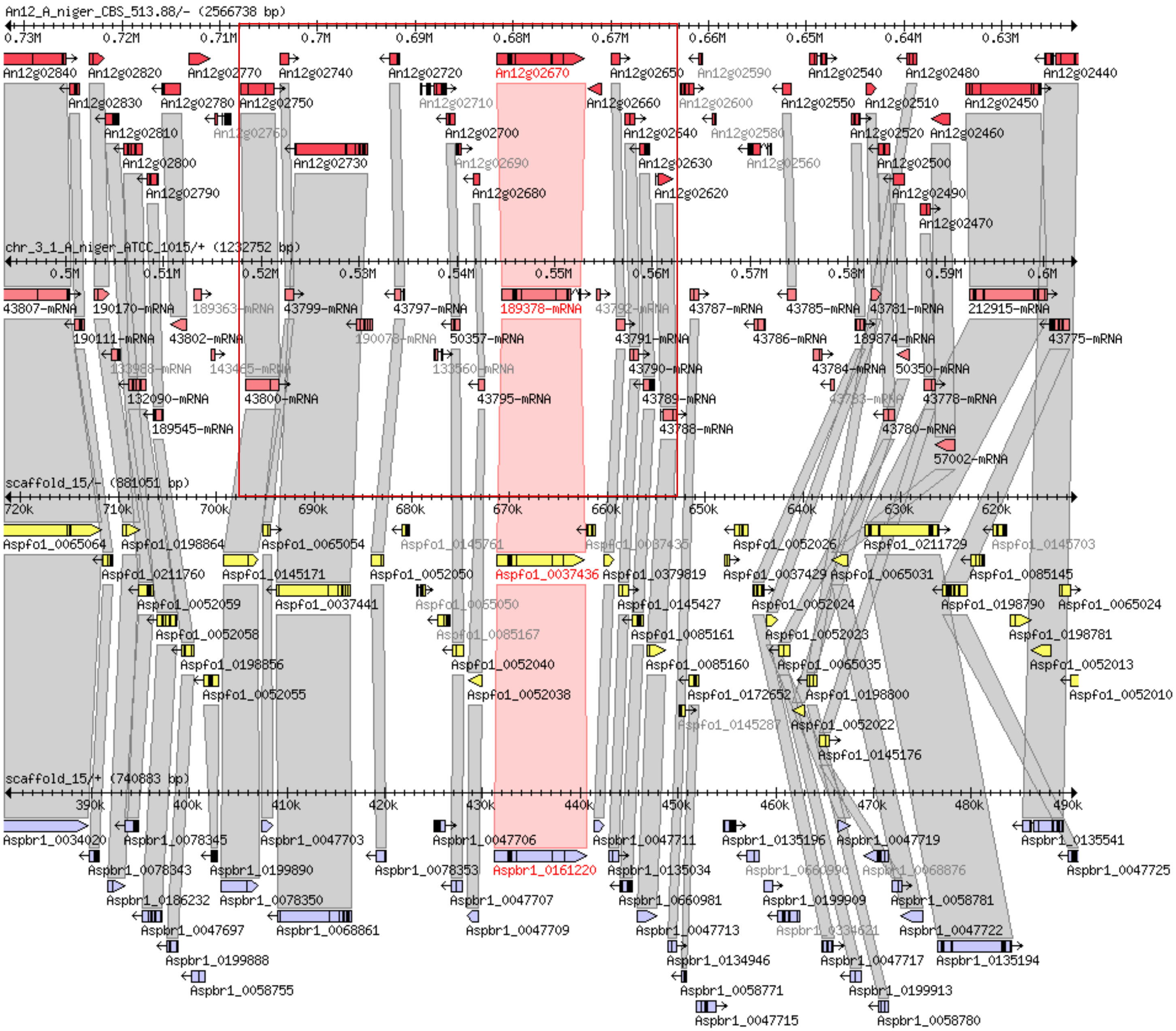

An12g02730 cluster

Gene id (SMURF)	Gene positions	Gene distance	AspGD name	AspGD description line or supplemental annotation	protein ID (antiSMASH)	AspGD ID
An12g02670	6	1828		Domain(s) with predicted ACP phosphopantetheine attachment site binding involved in fatty acid biosynthetic process, L-aminoadipate-semialdehyde dehydrogenase activity and nucleotide binding, more		
An12g02680	5	1198		Ortholog of A. nidulans FGSC A4 : AN11765, A. fumigatus Af293 : Afu3g07625, A. niger CBS 513.88 : An02g12900, An04g10150 and A. oryzae RIB40 : AO090020000130, AO090023000386		
An12g02690	4	125		Domain(s) with predicted heme binding activity		
An12g02700	3	77		Domain(s) with predicted nucleotide binding, oxidoreductase activity and role in oxidation-reduction process		
An12g02710	2	2152				
An12g02720	1	2325		Ortholog of A. nidulans FGSC A4 : AN11199, A. niger CBS 513.88 : An11g04260, A. niger ATCC 1015 : 39024-mRNA, A. versicolor : Aspve1_0053145 and A. sydowii : Aspsy1_0057345		
**An12g02730**	0	0		Ortholog(s) have role in asperfuranone biosynthetic process, emericellamide biosynthetic process, monodictyphenone biosynthetic process, o-orsellinic acid biosynthetic process, sterigmatocystin biosynthetic process		
An12g02740	-1	535		Ortholog of A. brasiliensis : Aspbr1_0047703, Aspergillus flavus NRRL 3357 : AFL2T_11821, A. acidus : Aspfo1_0065054 and Aspergillus terreus NIH2624 : ATET_02827		
An12g02750	-2	475		Domain(s) with predicted ACP phosphopantetheine attachment site binding involved in fatty acid biosynthetic process, catalytic activity and role in metabolic process		
An12g02760	-3	1081		Domain(s) with predicted carbon-sulfur lyase activity and role in metabolic process		
An12g02770	-4	404		Ortholog of Aspergillus terreus NIH2624 : ATET_09243		
An12g02780	-5	739		Domain(s) with predicted carbohydrate binding, metallopeptidase activity, zinc ion binding activity and role in cell adhesion, proteolysis		
An12g02790	-6	347		Domain(s) with predicted nucleotide binding activity		
An12g02800	-7	507		Domain(s) with predicted role in transmembrane transport and integral to membrane localization		
An12g02810	-8	599		Domain(s) with predicted O-methyltransferase activity		
An12g02820	-9	98		Domain(s) with predicted role in transmembrane transport and integral to membrane localization		

An12g02830	-10	1107	Ortholog(s) have role in austinol biosynthetic process, dehydroaustinol biosynthetic process, secondary metabolite biosynthetic process
An12g02840	-11	208	Ortholog(s) have role in brevianamide F biosynthetic process, emericellamide biosynthetic process, pathogenesis, secondary metabolite biosynthetic process, spore germination
An12g02850	-12	516	Thioredoxin reductase
An12g02860	-13	608	Domain(s) with predicted role in transmembrane transport and integral to membrane localization
An12g02870	-14	1024	
An12g02880	-15	231	Domain(s) with predicted DNA binding, sequence-specific DNA binding RNA polymerase II transcription factor activity, sequence-specific DNA binding transcription factor activity, zinc ion binding activity
An12g02890	-16	1981	Branched chain amino acid aminotransferase
An12g02900	-17	1200	Ortholog of A. niger ATCC 1015 : 54493-mRNA

An12g02840 cluster

Overlaps An12g02730 cluster

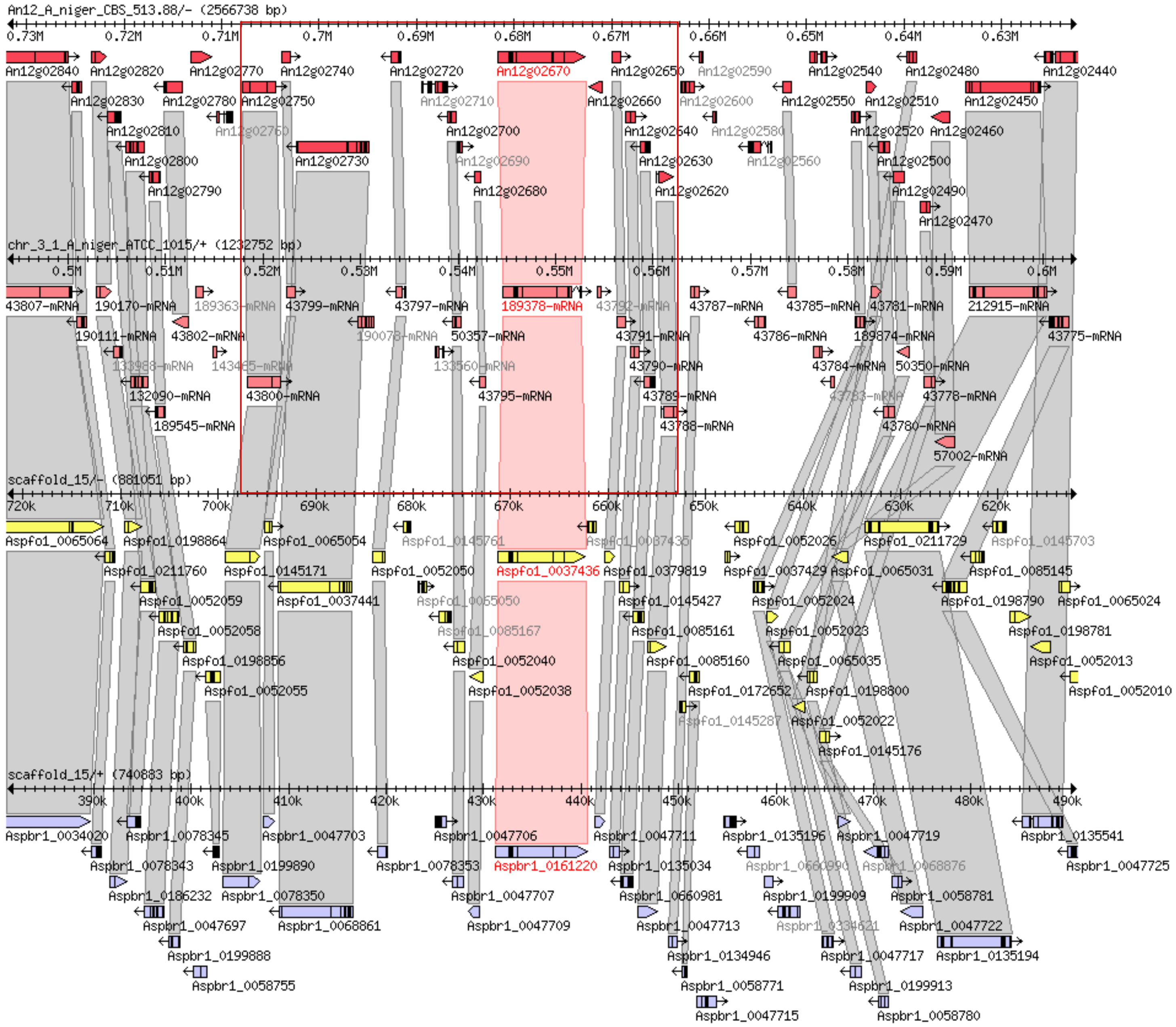

An12g02840 cluster

Gene id (SMURF)	Gene positions	Gene distance	AspGD name	AspGD description line or supplemental annotation	protein ID (antiSMASH)	AspGD ID
An12g02680	16	1198		Ortholog of A. nidulans FGSC A4 : AN11765, A. fumigatus Af293 : Afu3g07625, A. niger CBS 513.88 : An02g12900, An04g10150 and A. oryzae RIB40 : AO090020000130, AO090023000386		
An12g02690	15	125		Domain(s) with predicted heme binding activity		
An12g02700	14	77		Domain(s) with predicted nucleotide binding, oxidoreductase activity and role in oxidation-reduction process		
An12g02710	13	2152				
An12g02720	12	2325		Ortholog of A. nidulans FGSC A4 : AN11199, A. niger CBS 513.88 : An11g04260, A. niger ATCC 1015 : 39024-mRNA, A. versicolor : Aspve1_0053145 and A. sydowii : Aspsy1_0057345		
An12g02730	11	535		Ortholog(s) have role in asperfuranone biosynthetic process, emericellamide biosynthetic process, monodictyphenone biosynthetic process, o-orsellinic acid biosynthetic process, sterigmatocystin biosynthetic process		
An12g02740	10	475		Ortholog of A. brasiliensis : Aspbr1_0047703, Aspergillus flavus NRRL 3357 : AFL2T_11821, A. acidus : Aspfo1_0065054 and Aspergillus terreus NIH2624 : ATET_02827		
An12g02750	9	1081		Domain(s) with predicted ACP phosphopantetheine attachment site binding involved in fatty acid biosynthetic process, catalytic activity and role in metabolic process		
An12g02760	8	404		Domain(s) with predicted carbon-sulfur lyase activity and role in metabolic process		
An12g02770	7	739		Ortholog of Aspergillus terreus NIH2624 : ATET_09243		
An12g02780	6	347		Domain(s) with predicted carbohydrate binding, metallopeptidase activity, zinc ion binding activity and role in cell adhesion, proteolysis		
An12g02790	5	507		Domain(s) with predicted nucleotide binding activity		
An12g02800	4	599		Domain(s) with predicted role in transmembrane transport and integral to membrane localization		
An12g02810	3	98		Domain(s) with predicted O-methyltransferase activity		
An12g02820	2	1107		Domain(s) with predicted role in transmembrane transport and integral to membrane localization		
An12g02830	1	208		Ortholog(s) have role in austinol biosynthetic process, dehydroaustinol biosynthetic process, secondary metabolite biosynthetic process		

**An12g02840**	0	0	Ortholog(s) have role in brevianamide F biosynthetic process, emericellamide biosynthetic process, pathogenesis, secondary metabolite biosynthetic process, spore germination
An12g02850	-1	516	Thioredoxin reductase
An12g02860	-2	608	Domain(s) with predicted role in transmembrane transport and integral to membrane localization
An12g02870	-3	1024	
An12g02880	-4	231	Domain(s) with predicted DNA binding, sequence-specific DNA binding RNA polymerase II transcription factor activity, sequence-specific DNA binding transcription factor activity, zinc ion binding activity

An12g07070 cluster

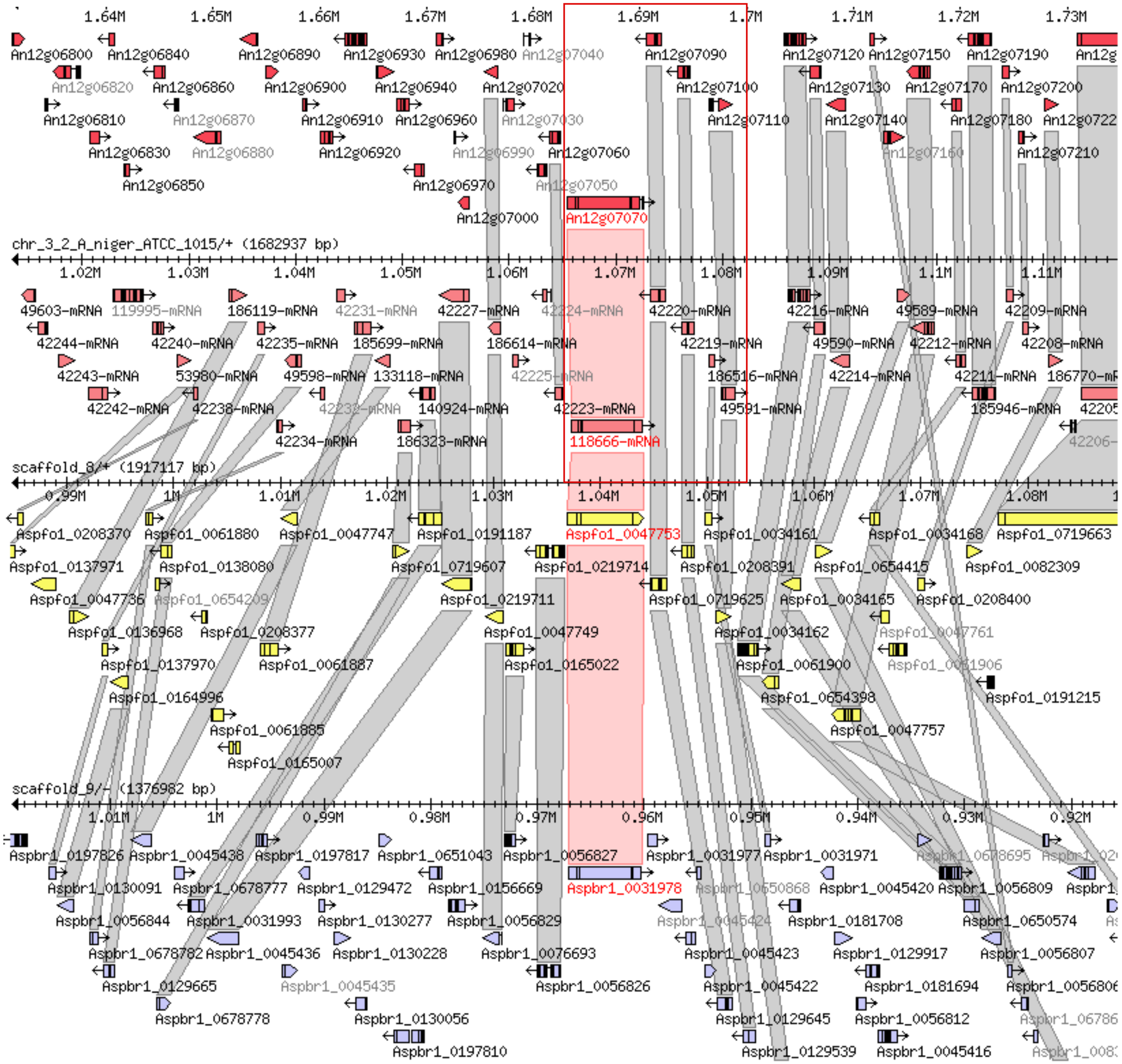

An12g07070 cluster

Gene id (SMURF)	Gene positions	Gene distance	AspGD name	AspGD description line or supplemental annotation	protein ID (antiSMASH)	AspGD ID	
n/a			amyA	Alpha-amylase (1, 4-alpha-D-glucan 4-glucanohydrolase), hydrolyzes the alpha-1,4-glucosidic bonds in glycogen or starch; identical to An05g02100	prot_ID_813	An12g06930	
n/a				Ortholog of A. fumigatus Af293 : Afu1g00550, Afu1g00910, A. niger CBS 513.88 : An05g02110, A. oryzae RIB40 : AO090120000200 and A. brasiliensis : Aspbr1_0139841	prot_ID_1052	An12g06940	
n/a				Ortholog of A. nidulans FGSC A4 : AN7889, AN4657, A. fumigatus Af293 : Afu1g00920, Afu3g06425 and A. niger CBS 513.88 : An07g04250, An05g02120, An01g11840	prot_ID_942	An12g06960	
n/a				Domain(s) with predicted heat shock protein binding, unfolded protein binding activity and role in protein folding	prot_ID_513	An12g06970	
n/a				Ortholog of A. oryzae RIB40 : AO090023000974, AO090023000942 and Aspergillus carbonarius ITEM 5010 : Acar5010_206391	prot_ID_11	An12g06980	
n/a				Putative reverse transcriptase	prot_ID_1036	An12g06990	
n/a				Domain(s) with predicted DNA binding activity and role in DNA integration	prot_ID_584	An12g07000	
n/a				Ortholog of A. nidulans FGSC A4 : AN7534, A. brasiliensis : Aspbr1_0076693, A. niger ATCC 1015 : 186614-mRNA, A. acidus : Aspfo1_0047749 and A. versicolor : Aspve1_0085820	prot_ID_1005	An12g07020	
n/a				Similar to stress response mediator Wsc3	prot_ID_730	An12g07030	
n/a				Questionable ORF	prot_ID_383	An12g07040	
An12g07050	2	251		Weak similarity to dihydrofolate reductase	prot_ID_1035	An12g07050	
An12g07060	1	686		Ortholog of A. niger ATCC 1015 : 42223-mRNA	prot_ID_4	An12g07060	ECS
**An12g07070**	0	0		Putative polyketide synthase	**prot_ID_592**	**An12g07070**	
An12g07090	-1	273		Domain(s) with predicted UDP-N-acetylmuramate dehydrogenase activity, flavin adenine dinucleotide binding activity and role in oxidation-reduction process	prot_ID_1004	An12g07090	
An12g07100	-2	1547		Ortholog of A. brasiliensis : Aspbr1_0129539, N. fischeri NRRL 181 : NFIA_005310, A. acidus : Aspfo1_0208391 and A. versicolor : Aspve1_0165969	prot_ID_777	An12g07100	
An12g07110	-3	1806		Domain(s) with predicted isochorismate synthase activity, oxo-acid-lyase activity and role in biosynthetic process	prot_ID_571	An12g07110	ECS

n/a	Domain(s) with predicted electron carrier activity, heme binding, iron ion binding, oxidoreductase activity, acting on paired donors, with incorporation or reduction of molecular oxygen activity and role in oxidation-reduction process	prot_ID_702	An12g07120
n/a	Domain(s) with predicted nucleotide binding activity	prot_ID_930	An12g07130
n/a	Domain(s) with predicted DNA binding, sequence-specific DNA binding RNA polymerase II transcription factor activity, zinc ion binding activity and role in regulation of transcription, DNA-dependent, transcription, DNA-dependent	prot_ID_34	An12g07140

An12g07230 cluster

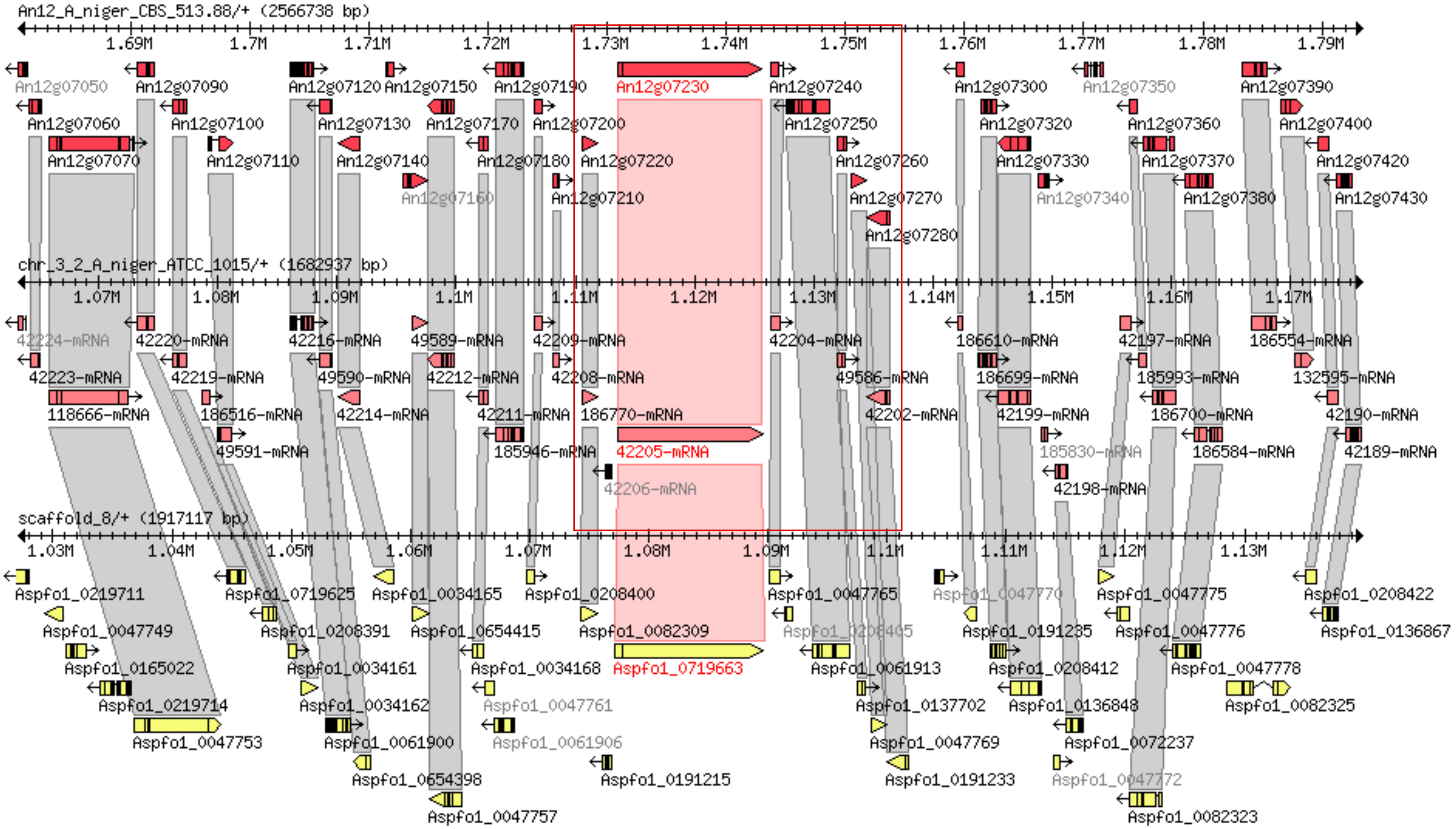

An12g07230 cluster

Gene id (SMURF)	Gene positions	Gene distance	AspGD name	AspGD description line or supplemental annotation	protein ID (antiSMASH)	AspGD ID	
An12g07120	11	372		Domain(s) with predicted electron carrier activity, heme binding, iron ion binding, oxidoreductase activity, acting on paired donors, with incorporation or reduction of molecular oxygen activity and role in oxidation-reduction process	n/a	An12g07120	
An12g07130	10	550		Domain(s) with predicted nucleotide binding activity	n/a	An12g07130	
An12g07140	9	2301		GAL4-type Z(2)-Cys(6) DNA-binding transcription factor	n/a	An12g07140	
An12g07150	8	791		Ortholog of A. oryzae RIB40 : AO090120000038, A. brasiliensis : Aspbr1_0056806, Aspbr1_0669219, Aspergillus flavus NRRL 3357 : AFL2T_08074 and Aspergillus aculeatus ATCC16872 : Aacu16872_047628	prot_ID_563	An12g07150	
An12g07160	7	92			prot_ID_225	An12g07160	
An12g07170	6	2059		Domain(s) with predicted transporter activity, role in transmembrane transport and membrane localization	prot_ID_590	An12g07170	
An12g07180	5	647		Ortholog of A. brasiliensis : Aspbr1_0049679, Aspbr1_0139846, Aspbr1_0181671, Aspbr1_0591537, A. acidus : Aspfo1_0034168 and A. niger ATCC 1015 : 42211-mRNA	prot_ID_855	An12g07180	
An12g07190	4	977		Domain(s) with predicted DNA binding, sequence-specific DNA binding RNA polymerase II transcription factor activity, sequence-specific DNA binding transcription factor activity, zinc ion binding activity	prot_ID_818	An12g07190	
An12g07200	3	936		Ortholog of A. brasiliensis : Aspbr1_0031950, A. acidus : Aspfo1_0208400 and A. niger ATCC 1015 : 42209-mRNA	prot_ID_459	An12g07200	
An12g07210	2	1720		Ortholog of A. niger ATCC 1015 : 42208-mRNA	prot_ID_185	An12g07210	
An12g07220	1	1768		Domain(s) with predicted nucleotide binding activity	prot_ID_742	An12g07220	ECS
**An12g07230**	0	0		Domain(s) with predicted ACP phosphopantetheine attachment site binding involved in fatty acid biosynthetic process, hydrolase activity, acting on ester bonds, ligase activity, phosphopantetheine binding activity	**prot_ID_415**	**An12g07230**	
An12g07240	-1	689		Ortholog of A. nidulans FGSC A4 : AN9208, AN9311, A. fumigatus Af293 : Afu5g14850, A. niger CBS 513.88 : An12g05170, An03g01320 and A. oryzae RIB40 : AO090023000071, AO090026000100	prot_ID_638	An12g07240	
An12g07250	-2	268		Domain(s) with predicted hydrolase activity, acting on carbon-nitrogen (but not peptide) bonds activity	prot_ID_102	An12g07250	
n/a				Ortholog of A. nidulans FGSC A4 : AN2942, AN10320, AN7130, A. fumigatus Af293 : Afu3g15110 and A. niger	prot_ID_518	An12g07260	

	CBS 513.88 : An03g05620, An15g07840, An03g02580, An01g14840			
n/a	Ortholog of A. brasiliensis : Aspbr1_0045398 and A. acidus : Aspfo1_0047769	prot_ID_908	An12g07270	
n/a	Domain(s) with predicted choline dehydrogenase activity, flavin adenine dinucleotide binding activity and role in alcohol metabolic process, oxidation-reduction process	prot_ID_610	An12g07280	ECS
n/a	Ortholog of A. oryzae RIB40 : AO090113000143, A. brasiliensis : Aspbr1_0045397, A. niger ATCC 1015 : 186610-mRNA and Aspergillus flavus NRRL 3357 : AFL2T_08621	prot_ID_221	An12g07300	
n/a	Domain(s) with predicted nucleotide binding activity	prot_ID_310	An12g07320	
n/a	Domain(s) with predicted DNA binding, zinc ion binding activity, role in transcription, DNA-dependent and nucleus localization	prot_ID_254	An12g07330	

An12g10090 cluster

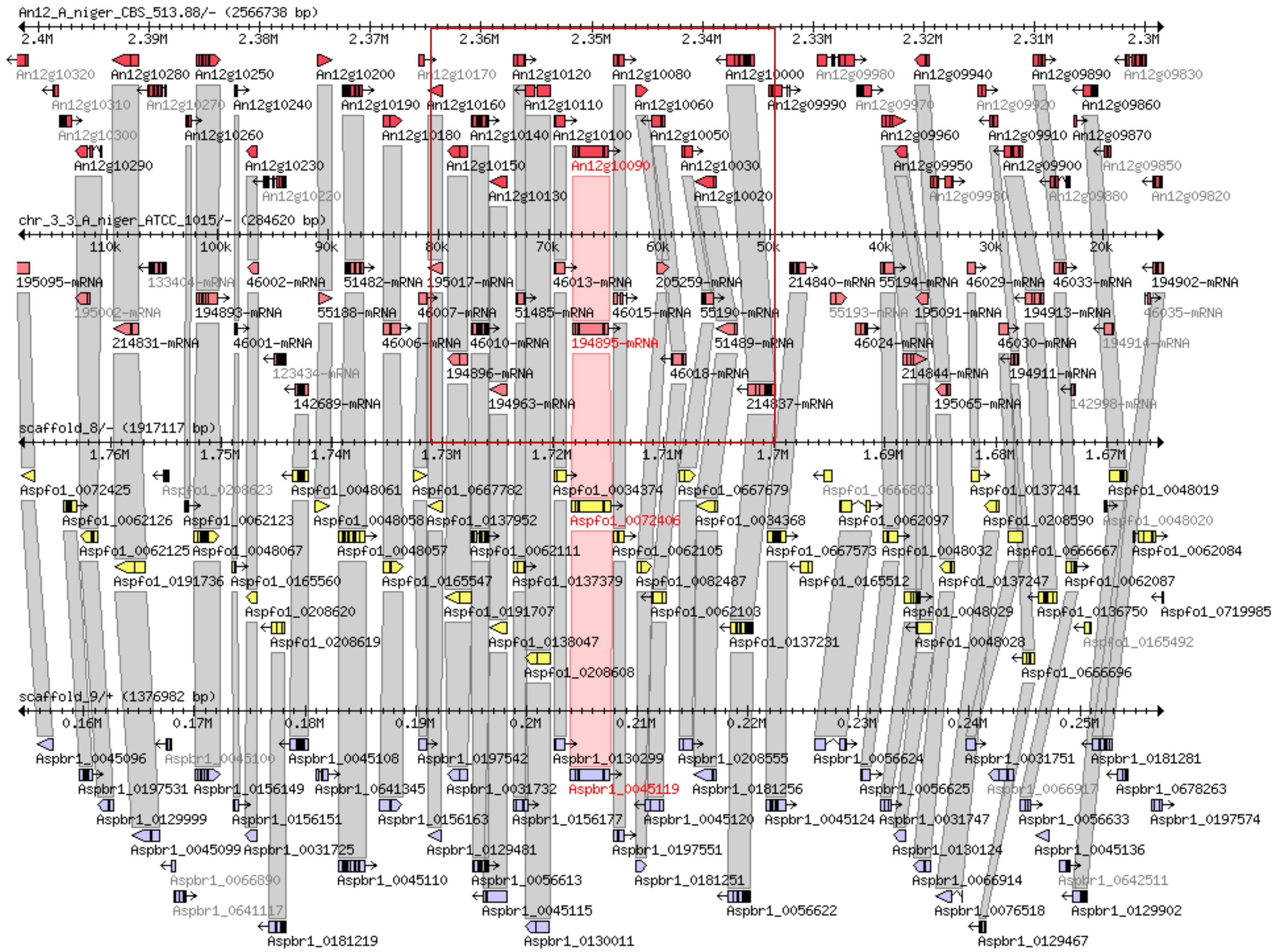

An12g10090 cluster

Gene id (SMURF)	Gene positions	Gene distance	AspGD name	AspGD description line or supplemental annotation	protein ID (antiSMASH)	AspGD ID	
An12g09930	13	146			n/a	An12g09930	
An12g09940	12	725		Stearoyl-CoA 9-desaturase	n/a	An12g09940	
An12g09950	11	69		Putative alcohol dehydrogenase	n/a	An12g09950	
An12g09960	10	916		Ortholog of A. nidulans FGSC A4 : AN5487, AN2619, A. niger CBS 513.88 : An12g02190, A. niger ATCC 1015 : 214844-mRNA, 43756-mRNA and A. versicolor : Aspve1_0121336, Aspve1_0206738	n/a	An12g09960	
An12g09970	9	847			n/a		
An12g09980	8	2523			prot_ID_835	An12g09980	
An12g09990	7	1607		Ortholog of A. fumigatus Af293 : Afu5g14670 and A. fumigatus A1163 : AFUB_062340	prot_ID_844	An12g09990	
An12g10000	6	1127	gabA	Putative GABA permease; expression repressed by tunicamycin and DTT	prot_ID_817	An12g10000	ECS
An12g10020	5	282		Domain(s) with predicted choline dehydrogenase activity, flavin adenine dinucleotide binding activity and role in alcohol metabolic process, oxidation-reduction process	prot_ID_188	An12g10020	
An12g10030	4	1565		Ortholog of A. nidulans FGSC A4 : AN2922, AN1515, AN7536, A. oryzae RIB40 : AO090701000305, AO090102000038, AO090124000043 and A. niger ATCC 1015 : 55190-mRNA	prot_ID_491	An12g10030	
An12g10050	3	350		Domain(s) with predicted catalytic activity and role in metabolic process	prot_ID_137	An12g10050	
An12g10060	2	1060		Domain(s) with predicted nucleotide binding, oxidoreductase activity and role in metabolic process	prot_ID_860	An12g10060	
An12g10080	1	345		Ortholog of A. nidulans FGSC A4 : AN9206, AN1849, A. fumigatus Af293 : Afu4g01210, Afu5g02320, Afu5g09930, A. niger CBS 513.88 : An03g02680, An12g02060 and A. oryzae RIB40 : AO090001000129, AO090010000537	prot_ID_977	An12g10080	
**An12g10090**	0	0		L-aminoadipatesemialdehyde dehydrogenase	**prot_ID_378**	**An12g10090**	
An12g10100	-1	605		Domain(s) with predicted 2-dehydropantoate 2-reductase activity, NADP binding activity, role in oxidation-reduction process, pantothenate biosynthetic process and cytoplasm localization	prot_ID_660	An12g10100	
An12g10110	-2	351		GAL4 zinc binuclear cluster-type transcription factor	prot_ID_970	An12g10110	
An12g10120	-3	73		Domain(s) with predicted metal ion binding activity	prot_ID_980	An12g10120	

An12g10130	-4	609	Domain(s) with predicted amino acid transmembrane transporter activity, role in amino acid transmembrane transport and membrane localization	prot_ID_836	An12g10130	
An12g10140	-5	108	Glycolate oxidase	prot_ID_386	An12g10140	
An12g10150	-6	365	Domain(s) with predicted oxidoreductase activity and role in oxidation-reduction process	prot_ID_786	An12g10150	
An12g10160	-7	486	Domain(s) with predicted transferase activity, transferring glycosyl groups activity and membrane localization	prot_ID_470	An12g10160	ECS
An12g10170	-8	175	Protein of unknown function	prot_ID_227	An12g10170	
An12g10180	-9	1600	Ortholog of A. nidulans FGSC A4 : AN5465, AN2186, AN10116, A. fumigatus Af293 : Afu1g13980, Afu5g02460 and A. niger CBS 513.88 : An08g07120, An02g10330, An12g09760, An07g05030, An05g02480	prot_ID_1053	An12g10180	
An12g10190	-10	1812	Domain(s) with predicted role in transmembrane transport and integral to membrane localization	prot_ID_679	An12g10190	
An12g10200	-11	1193	Putative cell wall protein with internal repeats; induced by caspofungin	n/a	An12g10200	
An12g10210	-12	1000		n/a	An12g10210	
An12g10220	-13	869		n/a	An12g10220	

An12g10860 cluster

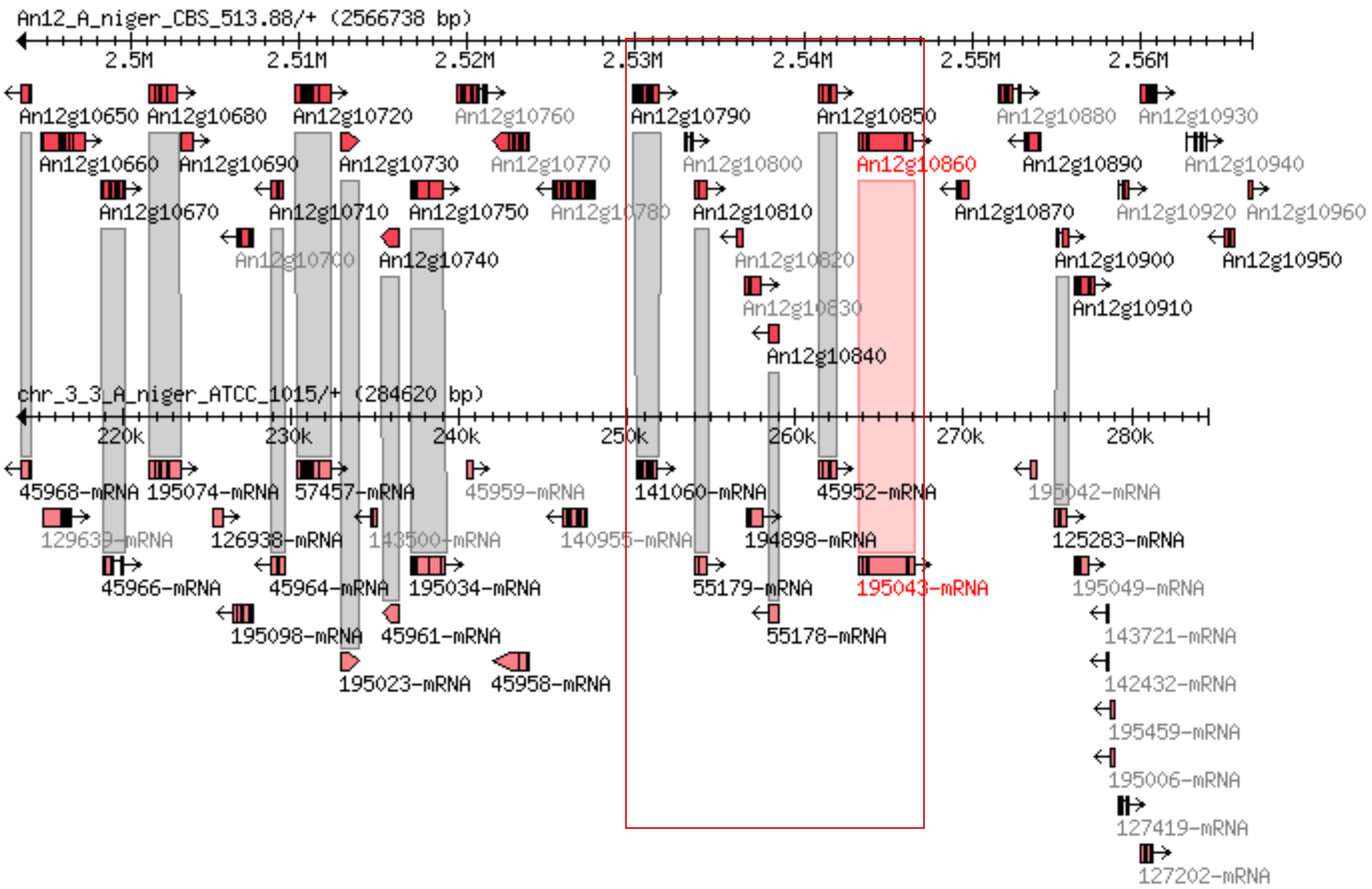

An12g10860 cluster

Gene id (SMURF)	Gene positions	Gene distance	AspGD name	AspGD description line or supplemental annotation	protein ID (antiSMASH)	AspGD ID	
n/a				Domain(s) with predicted choline dehydrogenase activity, flavin adenine dinucleotide binding activity and role in alcohol metabolic process, oxidation-reduction process	prot_ID_918	An12g10770	
n/a				Protein of unknown function	prot_ID_880	An12g10780	
n/a				Domain(s) with predicted peroxidase activity	prot_ID_671	An12g10790	ECS
n/a				Protein of unknown function	prot_ID_45	An12g10800	
n/a				Ortholog of A. brasiliensis : Aspbr1_0210236, A. acidus : Aspfo1_0060989, A. niger ATCC 1015 : 55179-mRNA and Aspergillus carbonarius ITEM 5010 : Acar5010_208649	prot_ID_870	An12g10810	
n/a				Protein of unknown function	prot_ID_449	An12g10820	
n/a				Protein of unknown function	prot_ID_265	An12g10830	
An12g10840	2	2392		Ortholog of A. acidus : Aspfo1_0211322 and A. niger ATCC 1015 : 55178-mRNA	prot_ID_765	An12g10840	
An12g10850	1	1301		Domain(s) with predicted carbon-carbon lyase activity and role in cellular aromatic compound metabolic process	prot_ID_488	An12g10850	
**An12g10860**	0	0		Protein similar to nonribosomal peptide synthases (NRPS-like)	**prot_ID_372**	**An12g10860**	ECS
An12g10870	-1	2502		Domain(s) with predicted N-acetyltransferase activity and role in metabolic process	prot_ID_594	An12g10870	
n/a				Protein of unknown function	prot_ID_915	An12g10880	
n/a				Domain(s) with predicted nucleotide binding, oxidoreductase activity, zinc ion binding activity and role in oxidation-reduction process	prot_ID_454	An12g10890	
n/a				Domain(s) with predicted nucleotide binding, oxidoreductase activity and role in oxidation-reduction process	prot_ID_275	An12g10900	
n/a				Domain(s) with predicted nucleotide binding, oxidoreductase activity, zinc ion binding activity and role in oxidation-reduction process	prot_ID_708	An12g10910	
n/a				Protein of unknown function	prot_ID_787	An12g10920	
n/a				Domain(s) with predicted nucleotide binding activity	prot_ID_58	An12g10930	
n/a				otein of unknown function	prot_ID_543	An12g10940	
n/a				Domain(s) with predicted RNA binding, endoribonuclease activity	prot_ID_757	An12g10950	
n/a				Protein of unknown function	prot_ID_290	An12g10960	

An13g01840 cluster

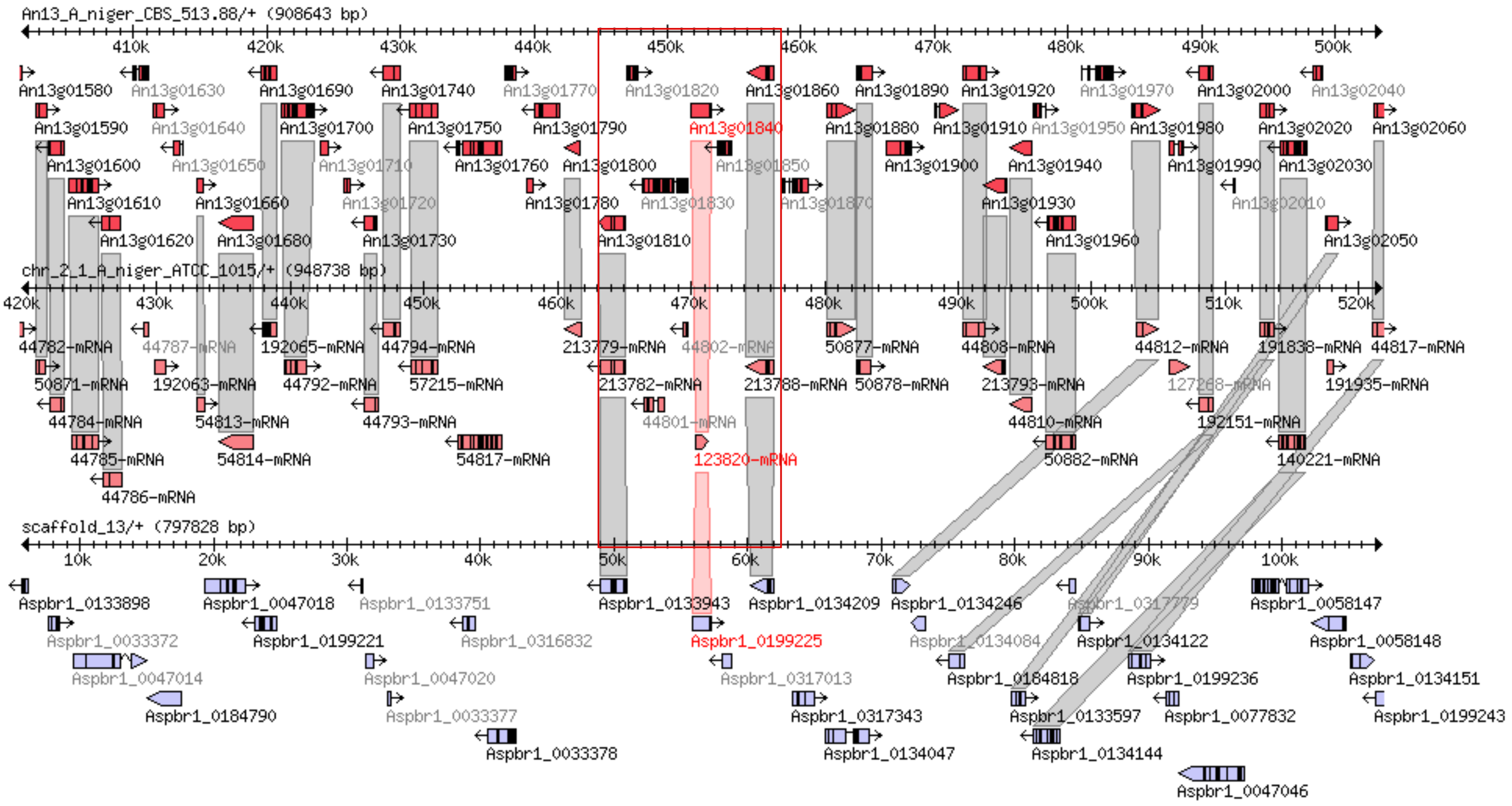

An13g01840 cluster

Gene id (SMURF)	Gene positions	Gene distance	AspGD name	AspGD description line or supplemental annotation	protein ID (antiSMASH)	AspGD ID	
n/a				Ortholog of A. nidulans FGSC A4 : AN9192, A. fumigatus Af293 : Afu3g03910, A. oryzae RIB40 : AO090038000138, N. fischeri NRRL 181 : NFIA_006190 and A. clavatus NRRL 1 : ACLA_060900	prot_ID_88	An13g01790	
n/a				Ortholog of A. fumigatus Af293 : Afu5g00590, A. niger CBS 513.88 : An15g07140, A. versicolor : Aspve1_0046767 and A. sydowii : Aspsy1_0029982, Aspsy1_0040096	prot_ID_220	An13g01800	
n/a				Ortholog(s) have monooxygenase activity and role in sterigmatocystin biosynthetic process	prot_ID_249	An13g01810	ECS
An13g01820	2	251		Protein of unknown function	prot_ID_387	An13g01820	
An13g01830	1	290		Protein of unknown function	prot_ID_3	An13g01830	
**An13g01840**	0	0		Putative dimethylallyl tryptophan synthase (DMAT)	**prot_ID_126**	**An13g01840**	
An13g01850	-1	481		Protein of unknown function	prot_ID_350	An13g01850	
An13g01860	-2	1045		Domain(s) with predicted amino acid transmembrane transporter activity, role in amino acid transmembrane transport and membrane localization	prot_ID_241	An13g01860	ECS
n/a				Protein of unknown function	prot_ID_388	An13g01870	
n/a				Triacylglycerol lipase	prot_ID_347	An13g01880	

An13g02430 cluster

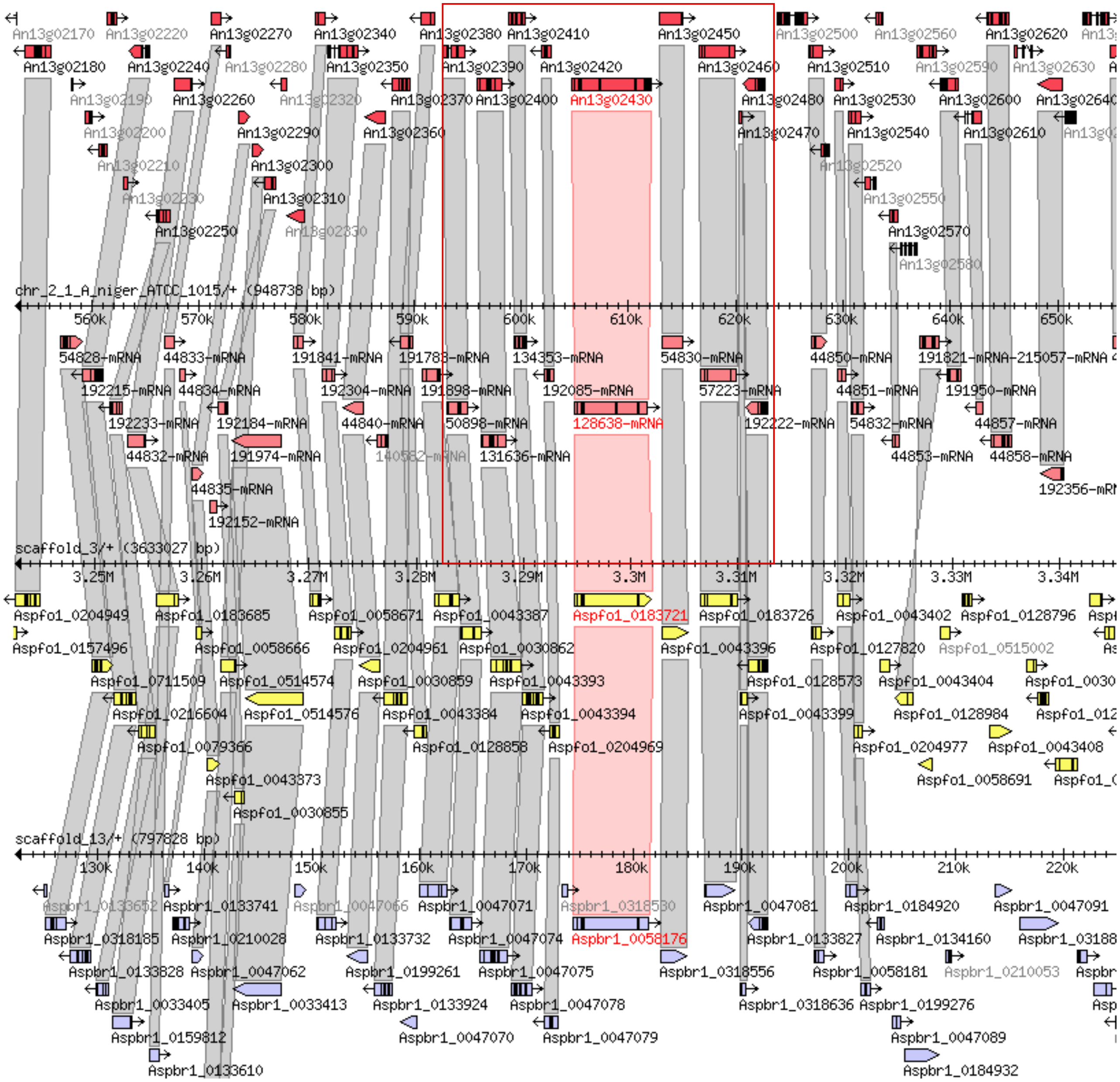

An13g02430 cluster

Gene id (SMURF)	Gene positions	Gene distance	AspGD name	AspGD description line or supplemental annotation	protein ID (antiSMASH)	AspGD ID	
An13g02290	14	276		Ortholog of A. nidulans FGSC A4 : AN2478, A. fumigatus Af293 : Afu6g00240, A. oryzae RIB40 : AO090103000492, A. niger ATCC 1015 : 44835-mRNA and A. versicolor : Aspve1_0140550, Aspve1_0200467	n/a	An13g02290	
An13g02300	13	157		Domain(s) with predicted sequence-specific DNA binding RNA polymerase II transcription factor activity, sequence-specific DNA binding transcription factor activity, zinc ion binding activity	n/a	An13g02300	
An13g02310	12	564		Ribitol dehydrogenase	n/a	An13g02310	
An13g02320	11	104		Vacuolar glutathione S-conjugate ABC transporter	n/a	An13g02320	
An13g02330	10	1006		Domain(s) with predicted ATP binding, ATPase activity, coupled to transmembrane movement of substances activity, role in transmembrane transport and integral to membrane localization	n/a	An13g02330	
An13g02340	9	178		Domain(s) with predicted nucleotide binding, oxidoreductase activity and role in metabolic process	n/a	An13g02340	
An13g02350	8	633		Domain(s) with predicted transporter activity, role in transmembrane transport, water transport and integral to membrane localization	prot_ID_281	An13g02350	
An13g02360	7	634		Ortholog of A. brasiliensis : Aspbr1_0199261, A. acidus : Aspfo1_0030859 and A. niger ATCC 1015 : 44840-mRNA	prot_ID_130	An13g02360	
An13g02370	6	1094		Domain(s) with predicted nucleic acid binding, zinc ion binding activity	prot_ID_65	An13g02370	
An13g02380	5	847		Ortholog of A. nidulans FGSC A4 : AN8523, A. fumigatus Af293 : Afu5g09810, A. niger ATCC 1015 : 191783-mRNA, A. versicolor : Aspve1_0046869 and A. sydowii : Aspsy1_0049687	prot_ID_227	An13g02380	
An13g02390	4	1217		Ortholog(s) have purine nucleobase transmembrane transporter activity, role in hypoxanthine transport and fungal-type vacuole, plasma membrane localization	prot_ID_269	An13g02390	ECS
An13g02400	3	792		Domain(s) with predicted sequence-specific DNA binding RNA polymerase II transcription factor activity, sequence-specific DNA binding transcription factor activity, zinc ion binding activity	prot_ID_373	An13g02400	
An13g02410	2	1574		Domain(s) with predicted metal ion transmembrane transporter activity, role in metal ion transport, transmembrane transport and membrane localization	prot_ID_369	An13g02410	
An13g02420	1	1838		Ortholog of A. brasiliensis : Aspbr1_0047079, Aspergillus flavus NRRL 3357 : AFL2T_11105, A. acidus : Aspfo1_0204969 and Aspergillus terreus NIH2624 :	prot_ID_79	An13g02420	

				ATET_07066			
**An13g02430**	0	0		Multifunctional polyketide synthase; ortholog(s) have role in asperfuranone biosynthetic process, emericellamide biosynthetic process, monodictyphenone biosynthetic process, o-orsellinic acid biosynthetic process, sterigmatocystin biosynthetic process	**prot_ID_311**	**An13g02430**	
An13g02450	-1	720		Domain(s) with predicted catalytic activity	prot_ID_120	An13g02450	
An13g02460	-2	1303		Domain(s) with predicted ACP phosphopantetheine attachment site binding involved in fatty acid biosynthetic process, catalytic activity, nucleotide binding, phosphopantetheine binding activity	prot_ID_255	An13g02460	
An13g02470	-3	428		Ortholog of A. brasiliensis : Aspbr1_0318636, A. acidus : Aspfo1_0043399, Aspergillus carbonarius ITEM 5010 : Acar5010_157459 and Aspergillus aculeatus ATCC16872 : Aacu16872_043764	prot_ID_193	An13g02470	ECS
An13g02480	-4	183		Domain(s) with predicted oxidoreductase activity and role in oxidation-reduction process	prot_ID_78	An13g02480	
An13g02500	-5	1158			prot_ID_323	An13g02500	
An13g02510	-6	167	crhE	Glycosylphosphatidylinositol-anchored chitin transglycosidase	prot_ID_32	An13g02510	
An13g02520	-7	143			prot_ID_375	An13g02520	
An13g02530	-8	541		Domain(s) with predicted carbonate dehydratase activity, zinc ion binding activity and role in one-carbon metabolic process	prot_ID_372	An13g02530	
An13g02540	-9	693		Ortholog of A. fumigatus Af293 : Afu2g17300, A. brasiliensis : Aspbr1_0199276, N. fischeri NRRL 181 : NFIA_092700, A. acidus : Aspfo1_0204977 and A. fumigatus A1163 : AFUB_032950	prot_ID_170	An13g02540	
n/a					prot_ID_304	An13g02550	
n/a					prot_ID_150	An13g02560	
n/a				Ortholog of A. brasiliensis : Aspbr1_0056198, A. acidus : Aspfo1_0074073, A. niger ATCC 1015 : 44853-mRNA and Aspergillus carbonarius ITEM 5010 : Acar5010_133941	prot_ID_234	An13g02570	
n/a					prot_ID_62	An13g02580	
n/a				Domain(s) with predicted transmembrane transporter activity, role in transmembrane transport and integral to membrane localization	prot_ID_81	An13g02590	
n/a				Domain(s) with predicted oxidoreductase activity and role in oxidation-reduction process	prot_ID_94	An13g02600	

An13g02460 cluster

Overlaps An13g02430 cluster

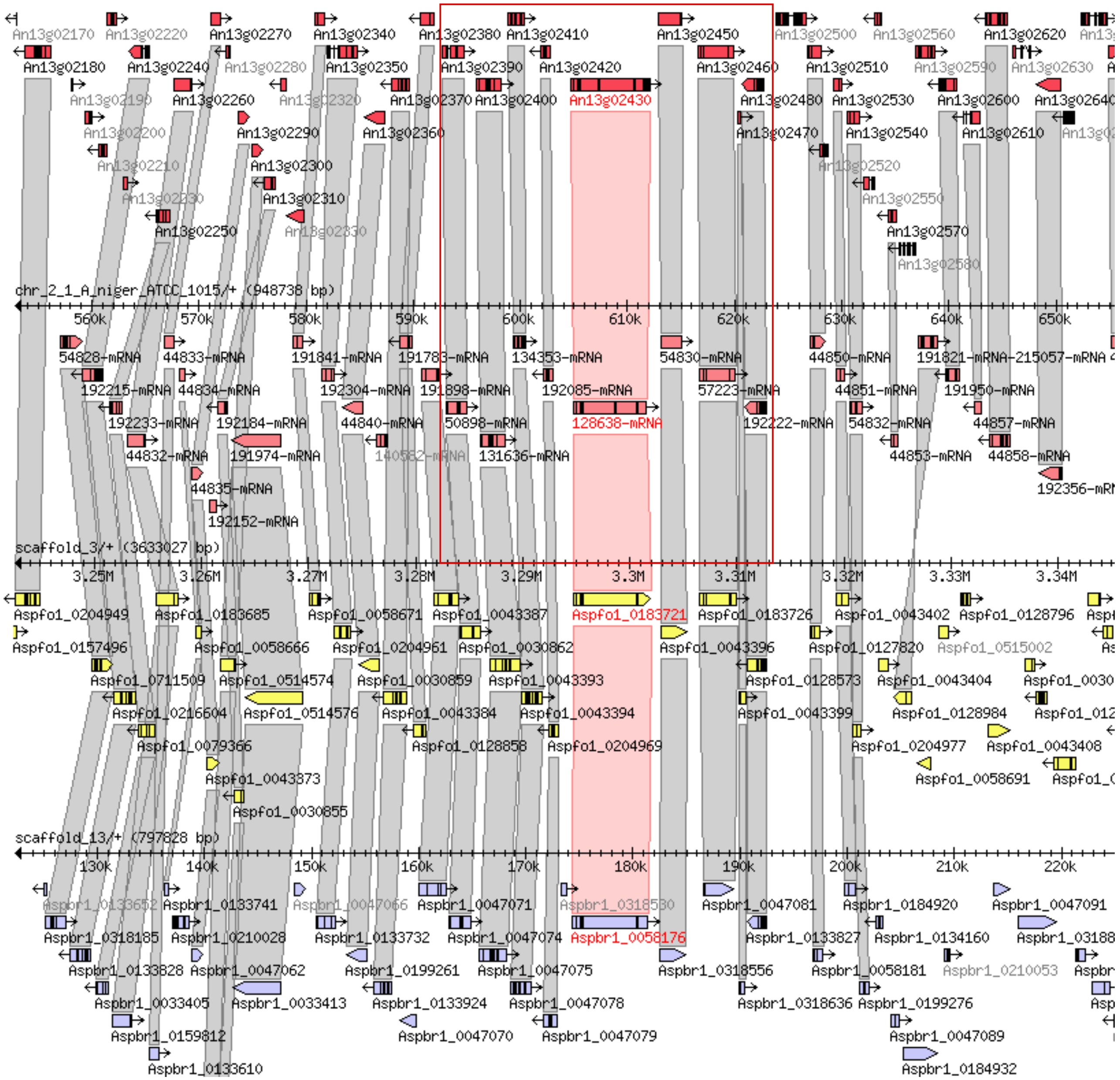

An13g02460 cluster

Gene id (SMURF)	Gene positions	Gene distance	AspGD name	AspGD description line or supplemental annotation		AspGD ID	
n/a				Ortholog(s) have purine nucleobase transmembrane transporter activity, role in hypoxanthine transport and fungal-type vacuole, plasma membrane localization	prot_ID_269	An13g02390	ECS
An13g02400	5	792		Domain(s) with predicted sequence-specific DNA binding RNA polymerase II transcription factor activity, sequence-specific DNA binding transcription factor activity, zinc ion binding activity	prot_ID_373	An13g02400	
An13g02410	4	1574		Domain(s) with predicted metal ion transmembrane transporter activity, role in metal ion transport, transmembrane transport and membrane localization	prot_ID_369	An13g02410	
An13g02420	3	1838		Ortholog of A. brasiliensis : Aspbr1_0047079, Aspergillus flavus NRRL 3357 : AFL2T_11105, A. acidus : Aspfo1_0204969 and Aspergillus terreus NIH2624 : ATET_07066	prot_ID_79	An13g02420	
An13g02430	2	720		Ortholog(s) have role in asperfuranone biosynthetic process, emericellamide biosynthetic process, monodictyphenone biosynthetic process, o-orsellinic acid biosynthetic process, sterigmatocystin biosynthetic process	**prot_ID_311**	**An13g02430**	
An13g02450	1	1303		Domain(s) with predicted catalytic activity	prot_ID_120	An13g02450	
**An13g02460**	0	0		Domain(s) with predicted ACP phosphopantetheine attachment site binding involved in fatty acid biosynthetic process, catalytic activity, nucleotide binding, phosphopantetheine binding activity	prot_ID_255	An13g02460	
An13g02470	-1	428		Ortholog of A. brasiliensis : Aspbr1_0318636, A. acidus : Aspfo1_0043399, Aspergillus carbonarius ITEM 5010 : Acar5010_157459 and Aspergillus aculeatus ATCC16872 : Aacu16872_043764	prot_ID_193	An13g02470	
An13g02480	-2	183		Domain(s) with predicted oxidoreductase activity and role in oxidation-reduction process	prot_ID_78	An13g02480	ECS
An13g02500	-3	1158			prot_ID_323	An13g02500	
An13g02510	-4	167	crhE	Glycosylphosphatidylinositol-anchored chitin transglycosidase	prot_ID_32	An13g02510	
An13g02520	-5	143			prot_ID_375	An13g02520	
An13g02530	-6	541		Domain(s) with predicted carbonate dehydratase activity, zinc ion binding activity and role in one-carbon metabolic process	prot_ID_372	An13g02530	
An13g02540	-7	693		Ortholog of A. fumigatus Af293 : Afu2g17300, A. brasiliensis : Aspbr1_0199276, N. fischeri NRRL 181 : NFIA_092700, A. acidus : Aspfo1_0204977 and A. fumigatus A1163 : AFUB_032950	prot_ID_170	An13g02540	ECS

An13g02960 cluster

No manual prediction made

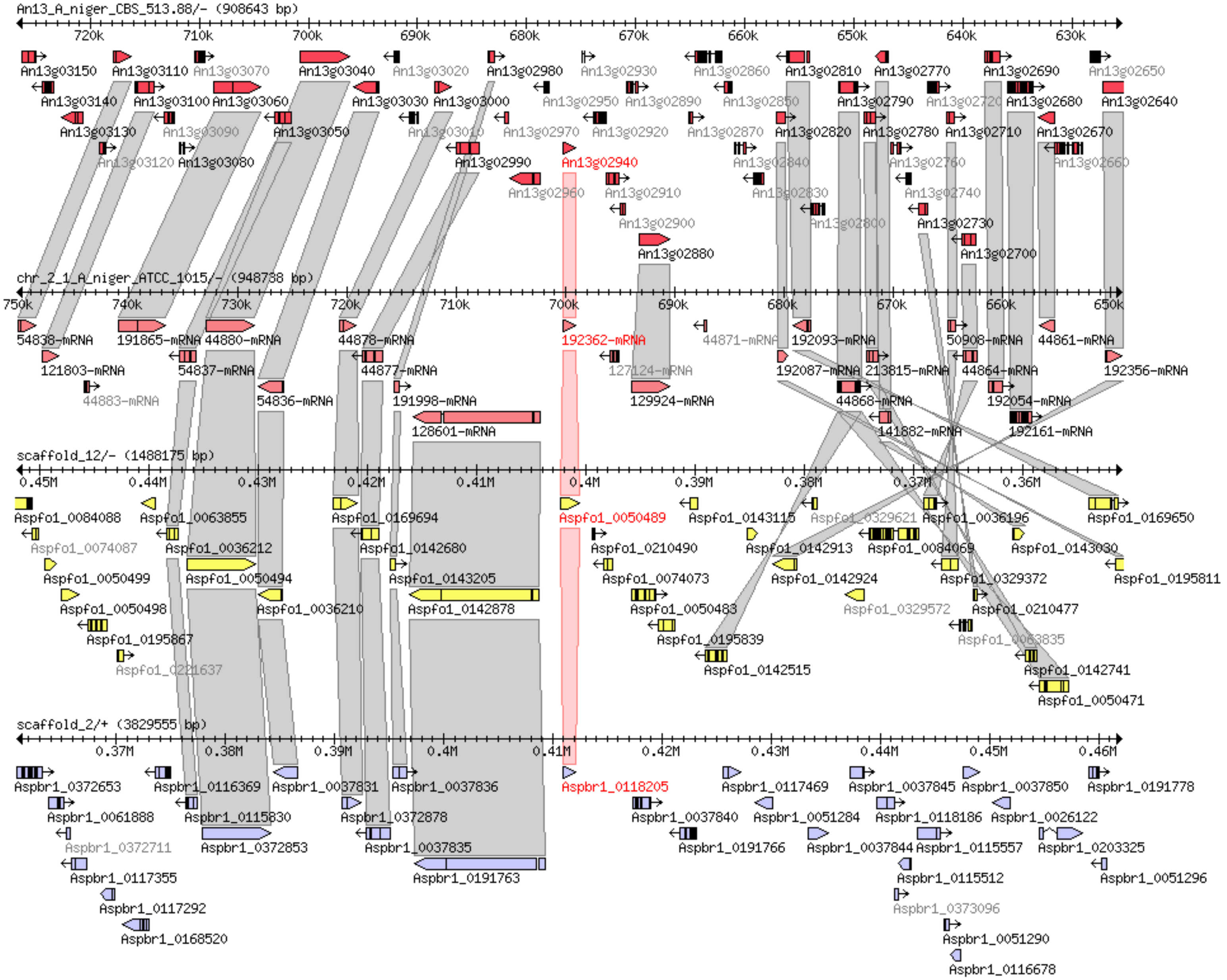

An13g02960 cluster

Gene id (SMURF)	Gene positions	Gene distance	AspGD name	AspGD description line or supplemental annotation	protein ID (antiSMASH)	AspGD ID
n/a				Protein of unknown function	prot_ID_185	An13g02830
n/a				Protein of unknown function	prot_ID_368	An13g02840
n/a				Protein of unknown function	prot_ID_179	An13g02850
n/a				Protein of unknown function	prot_ID_270	An13g02860
n/a				Protein of unknown function	prot_ID_284	An13g02870
n/a				Ortholog of A. nidulans FGSC A4 : AN3479, AN8495, A. fumigatus Af293 : Afu3g01440, Afu4g03340, Afu5g07620, A. oryzae RIB40 : AO090009000050 and A. niger ATCC 1015 : 129924-mRNA	prot_ID_100	An13g02880
n/a				Protein of unknown function	prot_ID_240	An13g02890
n/a				Protein of unknown function	prot_ID_338	An13g02900
n/a				Protein of unknown function	prot_ID_27	An13g02910
n/a				Protein of unknown function	prot_ID_154	An13g02920
n/a				Protein of unknown function	prot_ID_102	An13g02930
An13g02940	2	1329		Putative enoyl reductase	prot_ID_253	An13g02940
An13g02950	1	357		Protein of unknown function	prot_ID_176	An13g02950
**An13g02960**	0	0		Domain(s) with predicted transferase activity and role in biosynthetic process	**prot_ID_2**	**An13g02960**
An13g02970	-1	104		Protein of unknown function	prot_ID_291	An13g02970
An13g02980	-2	889		Ortholog of A. brasiliensis : Aspbr1_0037836, A. acidus : Aspfo1_0143205, A. versicolor : Aspve1_0495831 and A. niger ATCC 1015 : 191998-mRNA	prot_ID_331	An13g02980
An13g02990	-3	1045		Ortholog of A. brasiliensis : Aspbr1_0037835, A. acidus : Aspfo1_0142680 and A. niger ATCC 1015 : 44877-mRNA	prot_ID_266	An13g02990
An13g03000	-4	566		Domain(s) with predicted electron carrier activity, heme binding, iron ion binding, oxidoreductase activity, acting on paired donors and with incorporation or reduction of molecular oxygen, more	prot_ID_144	An13g03000
An13g03010	-5	1453		Protein of unknown function	prot_ID_379	An13g03010
An13g03020	-6	935		Protein of unknown function	prot_ID_327	An13g03020
An13g03030	-7	1377		Domain(s) with predicted sequence-specific DNA binding RNA polymerase II transcription factor activity, sequence-specific DNA binding transcription factor activity, zinc ion binding activity	prot_ID_183	An13g03030
An13g03040	-8	612		Domain(s) with predicted ACP phosphopantetheine attachment site binding involved in fatty acid biosynthetic process, ligase	prot_ID_167	An13g03040

			activity		
An13g03050	-9	1215	Ortholog of A. nidulans FGSC A4 : AN9209, A. fumigatus Af293 : Afu8g01840, Afu3g02685, A. oryzae RIB40 : AO090012000290 and A. niger ATCC 1015 : 190297-mRNA, 42320-mRNA, 54837-mRNA	prot_ID_23	An13g03050
An13g03060	-10	1546	Ortholog(s) have role in drug transmembrane transport	prot_ID_212	An13g03060
An13g03070	-11	676	Protein of unknown function	prot_ID_221	An13g03070
An13g03080	-12	963	Ortholog of A. nidulans FGSC A4 : AN8503, A. brasiliensis : Aspbr1_0044529, Aspergillus flavus NRRL 3357 : AFL2T_10601 and A. acidus : Aspfo1_0328979	prot_ID_168	An13g03080
An13g03090	-13	436	Protein of unknown function	prot_ID_290	An13g03090
n/a			Domain(s) with predicted potassium channel activity, role in potassium ion transmembrane transport and membrane localization	prot_ID_107	An13g03100
n/a			Domain(s) with predicted role in transmembrane transport and integral to membrane localization	prot_ID_283	An13g03110
n/a			Protein of unknown function	prot_ID_145	An13g03120

An13g03040 cluster

Overlaps with An13g02960 cluster , no manual prediction made

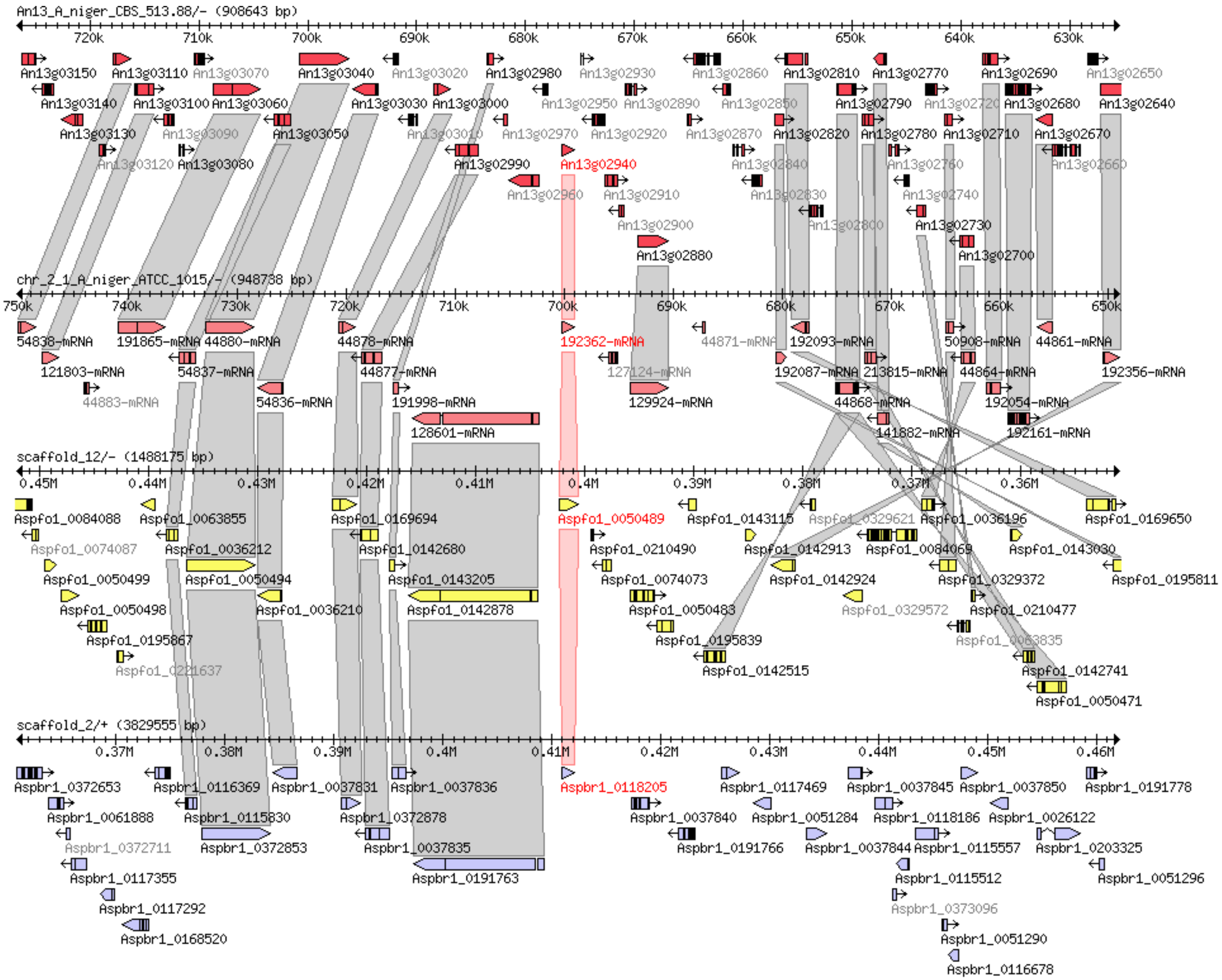

An13g03040 cluster

Gene id (SMURF)	Gene positions	Gene distance	AspGD name	AspGD description line or supplemental annotation	protein ID (antiSMASH)	AspGD ID
An13g02940	10	1329		Putative enoyl reductase	prot_ID_253	An13g02940
An13g02950	9	357			prot_ID_176	An13g02950
An13g02960	8	104		Domain(s) with predicted transferase activity and role in biosynthetic process	**prot_ID_2**	**An13g02960**
An13g02970	7	889			prot_ID_291	An13g02970
An13g02980	6	1045		Ortholog of A. brasiliensis : Aspbr1_0037836, A. acidus : Aspfo1_0143205, A. versicolor : Aspve1_0495831 and A. niger ATCC 1015 : 191998-mRNA	prot_ID_331	An13g02980
An13g02990	5	566		Ortholog of A. brasiliensis : Aspbr1_0037835, A. acidus : Aspfo1_0142680 and A. niger ATCC 1015 : 44877-mRNA	prot_ID_266	An13g02990
An13g03000	4	1453		Domain(s) with predicted electron carrier activity, heme binding, iron ion binding, oxidoreductase activity, acting on paired donors and with incorporation or reduction of molecular oxygen, more	prot_ID_144	An13g03000
An13g03010	3	935			prot_ID_379	An13g03010
An13g03020	2	1377			prot_ID_327	An13g03020
An13g03030	1	612		Domain(s) with predicted sequence-specific DNA binding RNA polymerase II transcription factor activity, sequence-specific DNA binding transcription factor activity, zinc ion binding activity	prot_ID_183	An13g03030
**An13g03040**	0	0		Domain(s) with predicted ACP phosphopantetheine attachment site binding involved in fatty acid biosynthetic process, ligase activity	prot_ID_167	An13g03040
An13g03050	-1	1215		Ortholog of A. nidulans FGSC A4 : AN9209, A. fumigatus Af293 : Afu8g01840, Afu3g02685, A. oryzae RIB40 : AO090012000290 and A. niger ATCC 1015 : 190297-mRNA, 42320-mRNA, 54837-mRNA	prot_ID_23	An13g03050
An13g03060	-2	1546		Ortholog(s) have role in drug transmembrane transport	prot_ID_212	An13g03060
An13g03070	-3	676			prot_ID_221	An13g03070
An13g03080	-4	963		Ortholog of A. nidulans FGSC A4 : AN8503, A. brasiliensis : Aspbr1_0044529, Aspergillus flavus NRRL 3357 : AFL2T_10601 and A. acidus : Aspfo1_0328979	prot_ID_168	An13g03080
An13g03090	-5	436			prot_ID_290	An13g03090
An13g03100	-6	952		Domain(s) with predicted potassium channel activity, role in potassium ion transmembrane transport and membrane localization	prot_ID_107	An13g03100
An13g03110	-7	375		Domain(s) with predicted role in transmembrane transport and integral to membrane localization	prot_ID_283	An13g03110

An14g01910 cluster

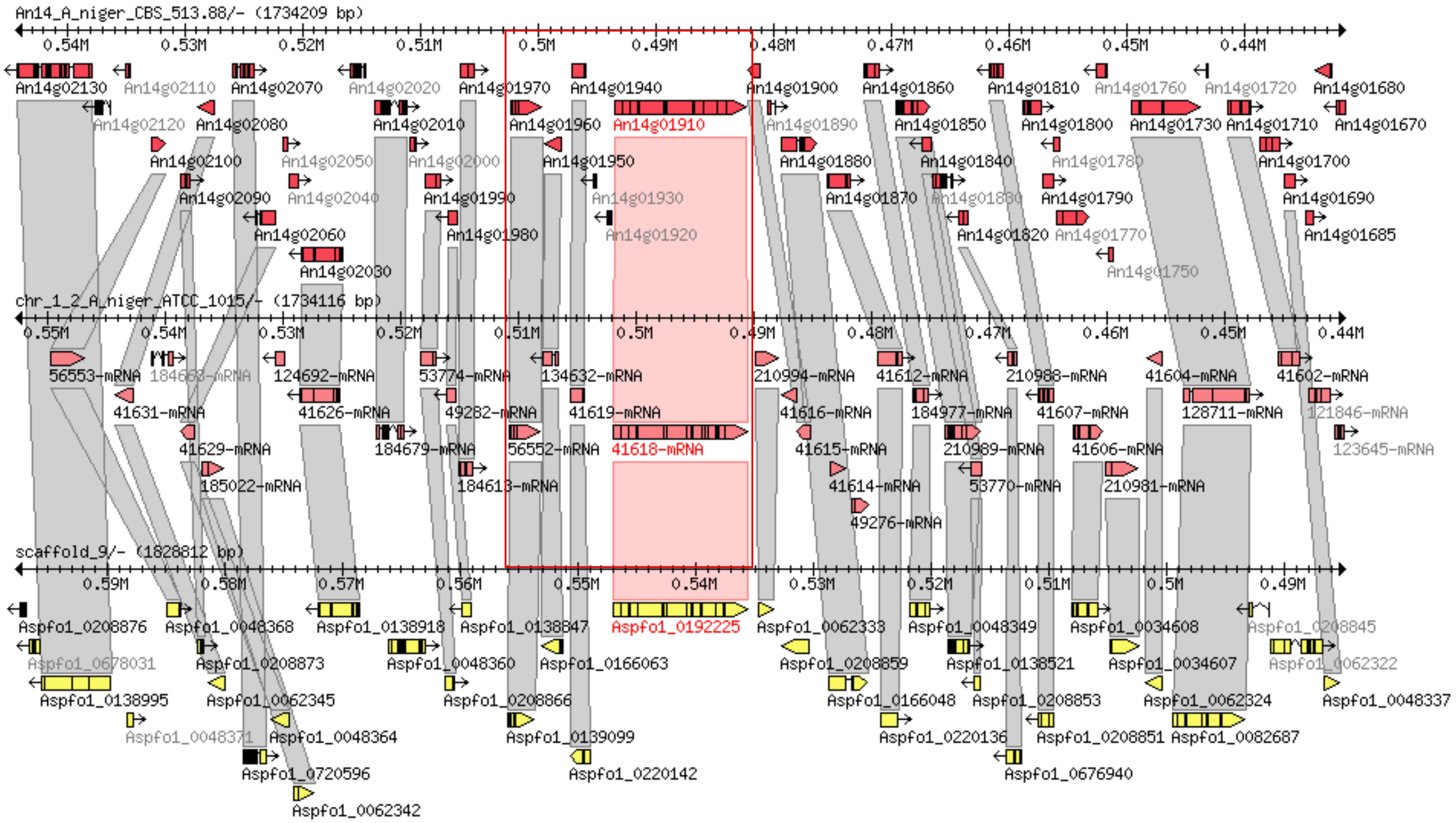

An14g01910 cluster

Gene id (SMURF)	Gene positions	Gene distance	AspGD name	AspGD description line or supplemental annotation	protein ID (antiSMASH)	AspGD ID	
n/a			phiA	Putative cell wall protein similar to A. nidulans phiA; abundantly expressed on d-xylose; induced by caspofungin	prot_ID_354	An14g01820	
n/a					prot_ID_339	An14g01830	
n/a				Ortholog of A. nidulans FGSC A4 : AN3215, AN7166, AN8609, A. fumigatus Af293 : Afu3g14210, Afu4g03360 and A. niger CBS 513.88 : An09g04200	prot_ID_393	An14g01840	
An14g01850	6	1634		Domain(s) with predicted amino acid transmembrane transporter activity, role in amino acid transmembrane transport and membrane localization	prot_ID_557	An14g01850	
An14g01860	5	965		Mitochondrial carrier protein	prot_ID_487	An14g01860	
An14g01870	4	751		Domain(s) with predicted ATP binding, nucleoside-triphosphatase activity	prot_ID_375	An14g01870	
An14g01880	3	503		Phosphatidylserine decarboxylase	prot_ID_143	An14g01880	
An14g01890	2	649			prot_ID_473	An14g01890	
An14g01900	1	151		Ortholog of A. niger ATCC 1015 : 41615-mRNA	prot_ID_71	An14g01900	
**An14g01910**	0	0		Ortholog(s) have role in pseurotin A biosynthetic process	**prot_ID_517**	**An14g01910**	ECS
An14g01920	-1	237		Questionable ORF	prot_ID_358	An14g01920	
An14g01930	-2	959		Questionable ORF	prot_ID_403	An14g01930	
An14g01940	-3	583		Putative enoyl reductase	prot_ID_188	An14g01940	
An14g01950	-4	918		Domain(s) with predicted alcohol O-acetyltransferase activity and role in alcohol metabolic process	prot_ID_490	An14g01950	
An14g01960	-5	480		Ortholog of A. nidulans FGSC A4 : AN7165, A. fumigatus Af293 : Afu4g03330, A. oryzae RIB40 : AO090011000165, A. niger ATCC 1015 : 56552-mRNA and A. versicolor : Aspve1_0140829	prot_ID_565	An14g01960	ECS
An14g01970	-6	2981		Domain(s) with predicted sequence-specific DNA binding RNA polymerase II transcription factor activity, sequence-specific DNA binding transcription factor activity, zinc ion binding activity	prot_ID_691	An14g01970	
n/a				Ortholog(s) have intracellular localization	prot_ID_361	An14g01980	
n/a				Protein of unknown function; expression induced by tunicamycin and DTT	prot_ID_418	An14g01990	
n/a					prot_ID_286	An14g02000	

n/a	Domain(s) with predicted cation transmembrane transporter activity, role in cation transport, transmembrane transport and integral to membrane localization	prot_ID_617	An14g02010

An14g04850 cluster

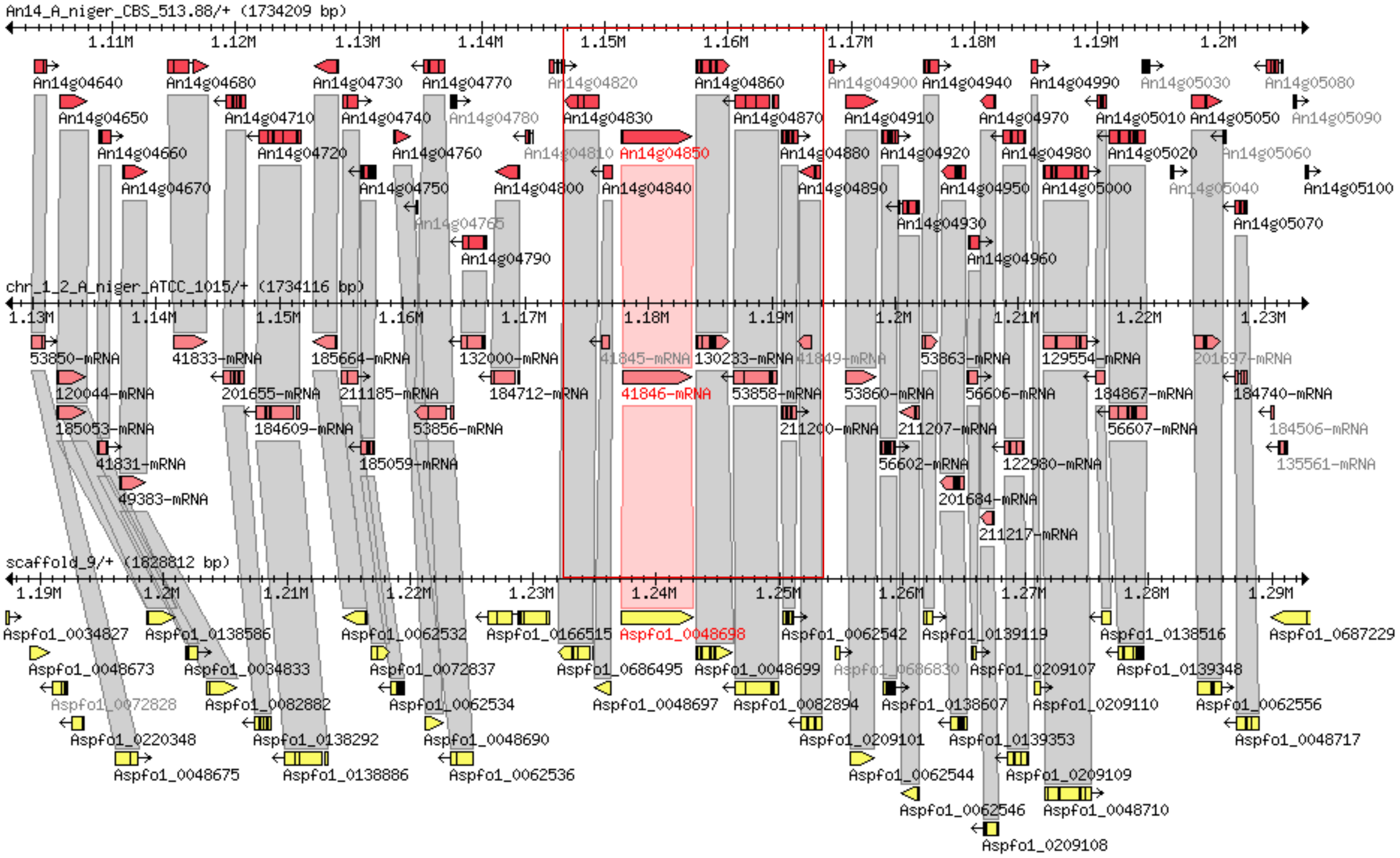

An14g04850 cluster

Gene id (SMURF)	Gene positions	Gene distance	AspGD name	AspGD description line or supplemental annotation	protein ID (antiSMASH)	AspGD ID	
n/a				Ortholog of A. oryzae RIB40 : AO090020000229, A. brasiliensis : Aspbr1_0114295, Aspergillus flavus NRRL 3357 : AFL2T_11065 and A. acidus : Aspfo1_0062534	prot_ID_312	An14g04750	
n/a				Domain(s) with predicted nucleotide binding activity	prot_ID_479	An14g04760	
n/a				Protein of uinknown function	prot_ID_706	An14g04765	
n/a				Domain(s) with predicted protein serine/threonine phosphatase activity, role in protein dephosphorylation and protein serine/threonine phosphatase complex localization	prot_ID_84	An14g04770	
n/a				Protein of uinknown function	prot_ID_680	An14g04780	
n/a				Domain(s) with predicted peptidyl-prolyl cis-trans isomerase activity, ubiquitin-protein ligase activity, role in protein folding, protein ubiquitination and ubiquitin ligase complex localization	prot_ID_626	An14g04790	
n/a				Ortholog(s) have role in cytoplasmic mRNA processing body assembly, deadenylation-independent decapping of nuclear-transcribed mRNA	prot_ID_591	An14g04800	
n/a				Protein of uinknown function	prot_ID_327	An14g04810	
n/a				Protein of uinknown function	prot_ID_660	An14g04820	
An14g04830	2	403		Ortholog(s) have ATP-dependent DNA helicase activity, role in DNA replication, mitochondrial genome maintenance, replication fork progression beyond termination site and nuclear telomeric heterochromatin, replication fork localization	prot_ID_727	An14g04830	ECS
An14g04840	1	798		Ortholog of A. brasiliensis : Aspbr1_0602357, A. acidus : Aspfo1_0045093, Aspfo1_0048697 and Aspergillus terreus NIH2624 : ATET_09618	prot_ID_420	An14g04840	
**An14g04850**	0	0		Putative polyketide synthase (PKS)	**prot_ID_124**	**An14g04850**	
An14g04860	-1	305		Domain(s) with predicted intracellular localization	prot_ID_561	An14g04860	
An14g04870	-2	926		Domain(s) with predicted GTP binding, GTPase activity	prot_ID_15	An14g04870	
n/a				Vacuolar protein-sorting protein	prot_ID_423	An14g04880	
n/a				Domain(s) with predicted ATP binding, protein tyrosine kinase activity and role in protein phosphorylation	prot_ID_278	An14g04890	ECS
n/a				Protein of uinknown function	prot_ID_613	An14g04900	
n/a				Ortholog of A. nidulans FGSC A4 : AN6899, A. oryzae RIB40 : AO090120000492, A. niger ATCC 1015 : 53860-mRNA, A. versicolor : Aspve1_0121406 and A. sydowii :	prot_ID_237	An14g04910	

		Aspsy1_0040365		
n/a	tpiA	Triose-phosphate-isomerase	prot_ID_690	An14g04920
n/a		Ortholog(s) have role in mitochondrion distribution, mitochondrion morphogenesis and mitochondrial membrane localization	prot_ID_368	An14g04930
n/a		Domain(s) with predicted role in ribosome biogenesis and intracellular localization	prot_ID_594	An14g04940

An15g02130 cluster

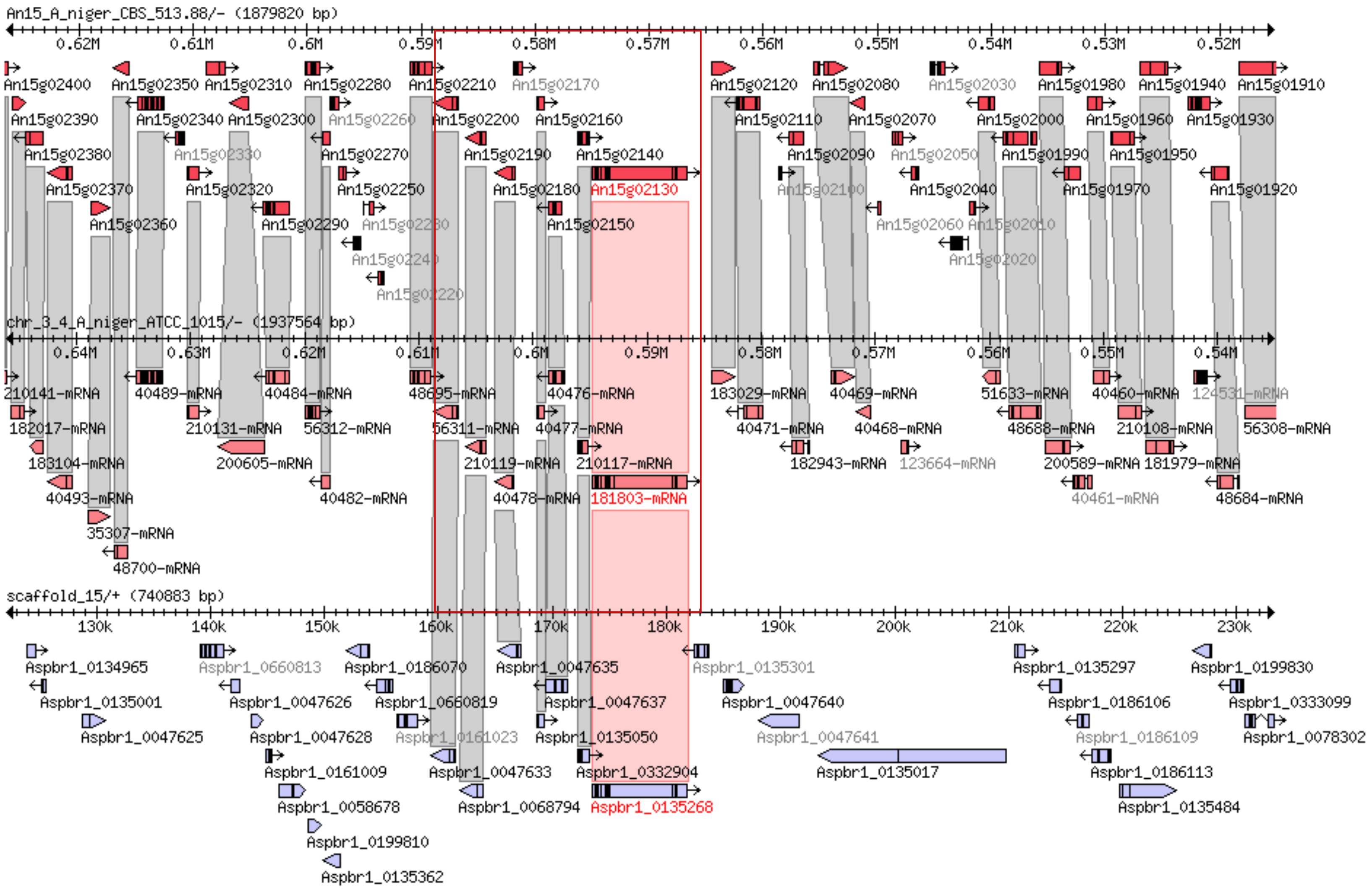

An15g02130 cluster

Gene id (SMURF)	Gene positions	Gene distance	AspGD name	AspGD description line or supplemental annotation	protein ID (antiSMASH)	AspGD ID	
n/a				Ortholog of A. nidulans FGSC A4 : AN6657, A. fumigatus Af293 : Afu6g03400, A. versicolor : Aspve1_0086179 and A. sydowii : Aspsy1_0157063	prot_ID_734	An15g02040	
n/a				Protein of unknown function	prot_ID_619	An15g02050	
n/a					prot_ID_398	An15g02060	
An15g02070	6	154		Domain(s) with predicted monooxygenase activity and role in oxidation-reduction process	prot_ID_69	An15g02070	
An15g02080	5	893		Domain(s) with predicted DNA binding, sequence-specific DNA binding RNA polymerase II transcription factor activity, sequence-specific DNA binding transcription factor activity, zinc ion binding activity	prot_ID_730	An15g02080	
An15g02090	4	493		Ortholog of A. nidulans FGSC A4 : AN0196, A. fumigatus Af293 : Afu5g14200, Afu6g13810, Afu8g01720, A. niger CBS 513.88 : An04g09640, A. oryzae RIB40 : AO090003001444 and A. niger ATCC 1015 : 182943-mRNA	prot_ID_512	An15g02090	
An15g02100	3	1640		Protein of unknown function	prot_ID_424	An15g02100	
An15g02110	2	188		Ortholog of A. acidus : Aspfo1_0081847, A. niger ATCC 1015 : 40471-mRNA and Aspergillus carbonarius ITEM 5010 : Acar5010_400349	prot_ID_764	An15g02110	
An15g02120	1	2104		Ortholog(s) have role in cellular response to drug, conidium formation, pathogenesis, regulation of conidium formation	prot_ID_591	An15g02120	
**An15g02130**	0	0		Putative polyketide synthase (PKS); ortholog(s) have role in asperfuranone biosynthetic process, emericellamide biosynthetic process, monodictyphenone biosynthetic process, o-orsellinic acid biosynthetic process, sterigmatocystin biosynthetic process	**prot_ID_719**	**An15g02130**	ECS
An15g02140	-1	153		Ortholog of A. brasiliensis : Aspbr1_0332904, A. acidus : Aspfo1_0042378 and A. niger ATCC 1015 : 210117-mRNA	prot_ID_96	An15g02140	
An15g02150	-2	1511		Ortholog of A. niger CBS 513.88 : An01g01780, A. oryzae RIB40 : AO090026000150, A. brasiliensis : Aspbr1_0047637 and A. niger ATCC 1015 : 171681-mRNA, 40476-mRNA	prot_ID_682	An15g02150	
An15g02160	-3	285		Domain(s) with predicted peroxiredoxin activity and role in oxidation-reduction process	prot_ID_267	An15g02160	
An15g02170	-4	1105		Protein of unknown function	prot_ID_400	An15g02170	

An15g02180	-5	72		Domain(s) with predicted monooxygenase activity and role in oxidation-reduction process	prot_ID_353	An15g02180	
An15g02190	-6	1084		Domain(s) with predicted role in transmembrane transport and integral to membrane localization	prot_ID_679	An15g02190	
n/a			aod1	Alcohol oxidase; induced by growth on starch and lactate	prot_ID_162	An15g02200	ECS
n/a				Ortholog of A. nidulans FGSC A4 : AN7948, A. niger ATCC 1015 : 48695-mRNA, A. acidus : Aspfo1_0071750 and A. versicolor : Aspve1_0063096	prot_ID_572	An15g02210	
n/a				Protein of unknown function	prot_ID_391	An15g02220	
n/a				Protein of unknown function	prot_ID_651	An15g02230	

An15g04140 cluster

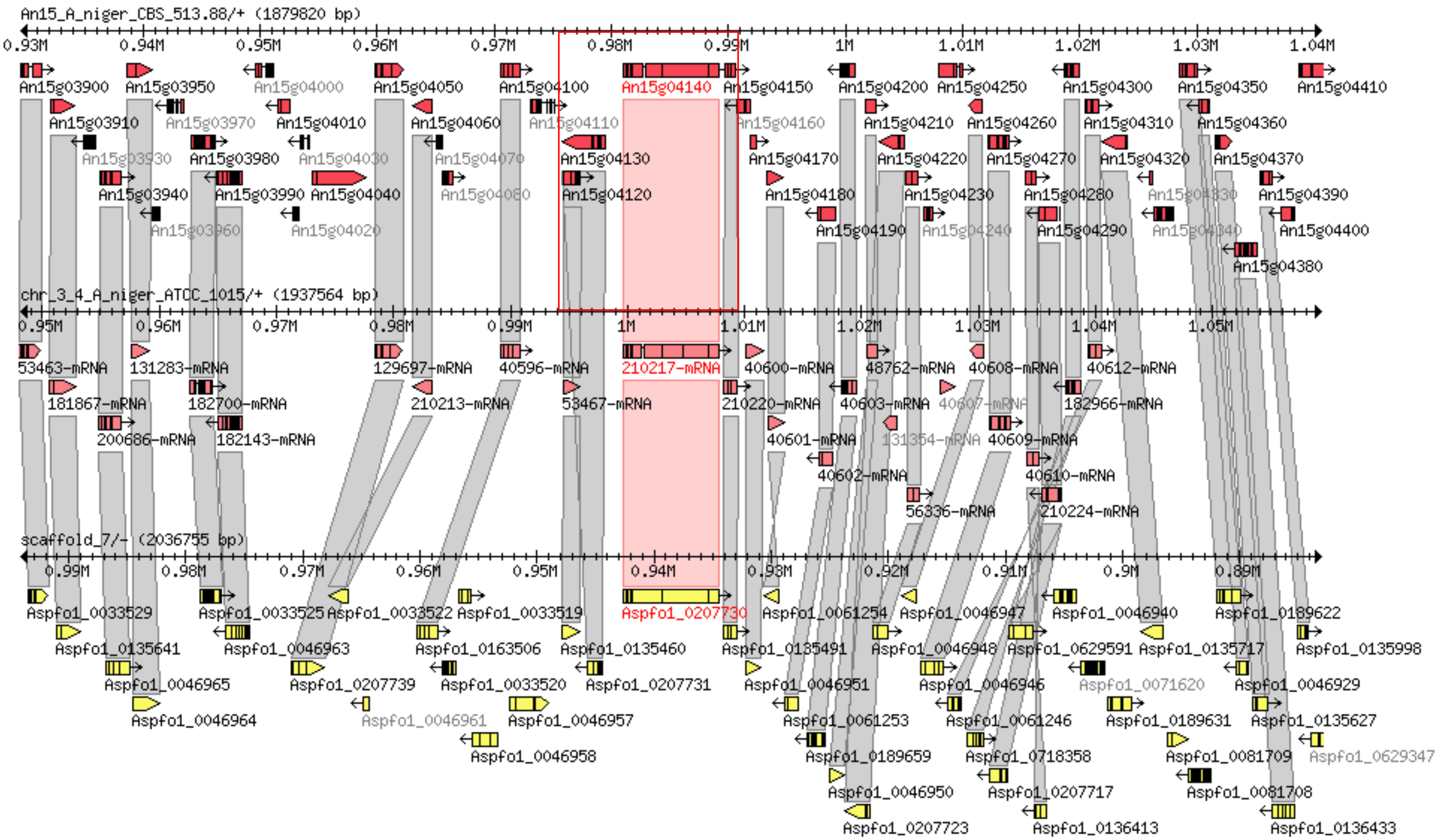

An15g04140 cluster

Gene id (SMURF)	Gene positions	Gene distance	AspGD name	AspGD description line or supplemental annotation	protein ID (antiSMASH)	AspGD ID	
n/a				Domain(s) with predicted solute:hydrogen antiporter activity, role in cation transport, transmembrane transport and integral to membrane localization	prot_ID_42	An15g04050	
n/a				Putative fructose transporter	prot_ID_154	An15g04060	
n/a				Protein of unknown function	prot_ID_282	An15g04070	
n/a				Protein of unknown function	prot_ID_143	An15g04080	
n/a				Domain(s) with predicted oxidoreductase activity, acting on the aldehyde or oxo group of donors, NAD or NADP as acceptor activity and role in oxidation-reduction process	prot_ID_369	An15g04100	
n/a				Protein of unknown function	prot_ID_261	An15g04110	
An15g04120	2	573		Ortholog of A. nidulans FGSC A4 : AN6764, AN2660, AN7798, A. fumigatus Af293 : Afu8g01960 and A. niger CBS 513.88 : An11g05430, An01g12050, An11g03480, An01g01140	prot_ID_35	An15g04120	
An15g04130	1	1877		Ortholog of A. acidus : Aspfo1_0207731	prot_ID_325	An15g04130	ECS
**An15g04140**	0	0		Multifunctional polyketide synthase, ortholog(s) have role in asperfuranone biosynthetic process, emericellamide biosynthetic process, monodictyphenone biosynthetic process, o-orsellinic acid biosynthetic process, sterigmatocystin biosynthetic process	**prot_ID_211**	**An15g04140**	
An15g04150	-1	454		Domain(s) with predicted nucleotide binding, oxidoreductase activity and role in oxidation-reduction process	prot_ID_150	An15g04150	ECS
An15g04160	-2	106		Domain(s) with predicted nucleic acid binding activity	prot_ID_107	An15g04160	
An15g04170	-3	16		Ortholog of A. oryzae RIB40 : AO090026000058	prot_ID_319	An15g04170	
An15g04180	-4	937		Domain(s) with predicted electron carrier activity, heme binding, iron ion binding, oxidoreductase activity, acting on paired donors, with incorporation or reduction of molecular oxygen activity and role in oxidation-reduction process	prot_ID_528	An15g04180	
An15g04190	-5	2974		Ortholog of A. niger CBS 513.88 : An13g03430, A. brasiliensis : Aspbr1_0057462, A. acidus : Aspfo1_0061253, Aspergillus niger ATCC 1015 : 40602-mRNA and A. niger ATCC 1015 : 44906-mRNA	prot_ID_747	An15g04190	
An15g04200	-6	419		Ortholog of A. niger CBS 513.88 : An13g03420, Neosartorya fischeri NRRL 181 : NFIA_002260, A. versicolor : Aspve1_0074246 and A. sydowii : Aspsy1_0134618	prot_ID_495	An15g04200	

An15g04210	-7	843	Domain(s) with predicted oxidoreductase activity and role in oxidation-reduction process	prot_ID_420	An15g04210
An15g04220	-8	251	Domain(s) with predicted DNA binding, zinc ion binding activity, role in transcription, DNA-dependent and nucleus localization	prot_ID_491	An15g04220
n/a			Domain(s) with predicted lyase activity and role in metabolic process	prot_ID_579	An15g04230
n/a			Protein of unknown function	prot_ID_358	An15g04240
n/a			Ortholog of A. brasiliensis : Aspbr1_0287784, A. sydowii : Aspsy1_0041297 and Aspergillus terreus NIH2624 : ATET_06176	prot_ID_212	An15g04250

An15g05090 cluster

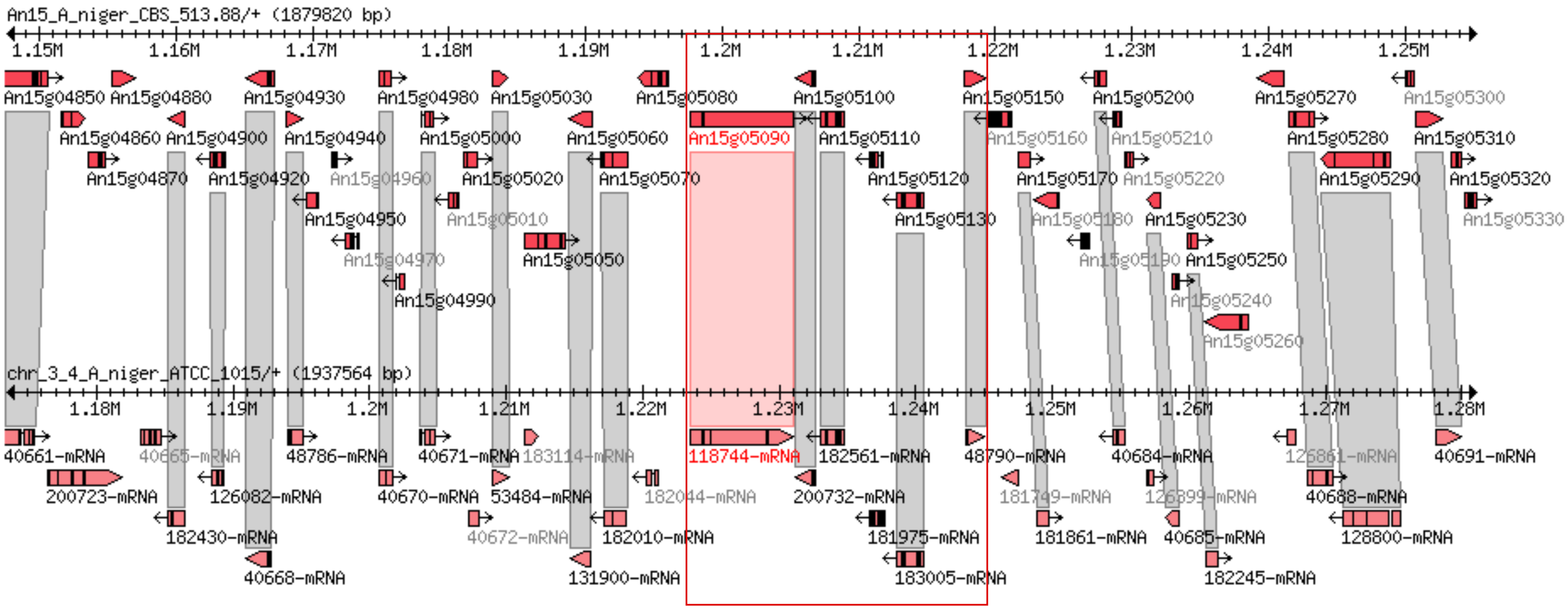

An15g05090 cluster

Gene id (SMURF)	Gene positions	Gene distance	AspGD name	AspGD description line or supplemental annotation	protein ID (antiSMASH)	AspGD ID
n/a				Ortholog of A. niger ATCC 1015 : 40671-mRNA	prot_ID_171	An15g05000
n/a					prot_ID_756	An15g05010
n/a				Ortholog of A. oryzae RIB40 : AO090010000253, AO090010000280 and N. fischeri NRRL 181 : NFIA_057710	prot_ID_351	An15g05020
An15g05030	5	1196		Ortholog of A. nidulans FGSC A4 : AN8528, AN12291, A. fumigatus Af293 : Afu5g09790, A. oryzae RIB40 : AO090010000330 and A. niger ATCC 1015 : 53484-mRNA	prot_ID_262	An15g05030
An15g05050	4	294		Domain(s) with predicted ADP binding, ATP binding, catalytic activity and role in apoptotic process, defense response, nucleoside metabolic process	prot_ID_91	An15g05050
An15g05060	3	700		Domain(s) with predicted role in transmembrane transport and integral to membrane localization	prot_ID_75	An15g05060
An15g05070	2	733		Domain(s) with predicted electron carrier activity, heme binding, iron ion binding, monooxygenase activity, oxidoreductase activity, acting on paired donors, with incorporation or reduction of molecular oxygen activity	prot_ID_371	An15g05070
An15g05080	1	1602		Domain(s) with predicted DNA binding, sequence-specific DNA binding RNA polymerase II transcription factor activity, sequence-specific DNA binding transcription factor activity, zinc ion binding activity	prot_ID_641	An15g05080
**An15g05090**	0	0		Domain(s) with predicted ACP phosphopantetheine attachment site binding involved in fatty acid biosynthetic process, nucleotide binding, oxidoreductase activity and transferase activity, more	**prot_ID_63**	**An15g05090**
An15g05100	-1	83		Domain(s) with predicted sequence-specific DNA binding RNA polymerase II transcription factor activity, sequence-specific DNA binding transcription factor activity, zinc ion binding activity	prot_ID_233	An15g05100
An15g05110	-2	394		Ortholog(s) have catalytic activity and role in steroid metabolic process	prot_ID_94	An15g05110
An15g05120	-3	1773		Domain(s) with predicted hydrolase activity, acting on carbon-nitrogen (but not peptide) bonds, in linear amides activity, role in ceramide metabolic process and integral to membrane localization	prot_ID_773	An15g05120
An15g05130	-4	1051		Domain(s) with predicted arylsulfatase activity and role in phenol-containing compound metabolic process	prot_ID_567	An15g05130
An15g05150	-5	2990		Domain(s) with predicted role in transmembrane transport and integral to membrane localization	prot_ID_106	An15g05150
n/a				Protein of unknown function	prot_ID_737	An15g05160

n/a	Cysteine synthase	prot_ID_515	An15g05170
n/a	Protein of unknown function	prot_ID_183	An15g05180

An15g07530 cluster

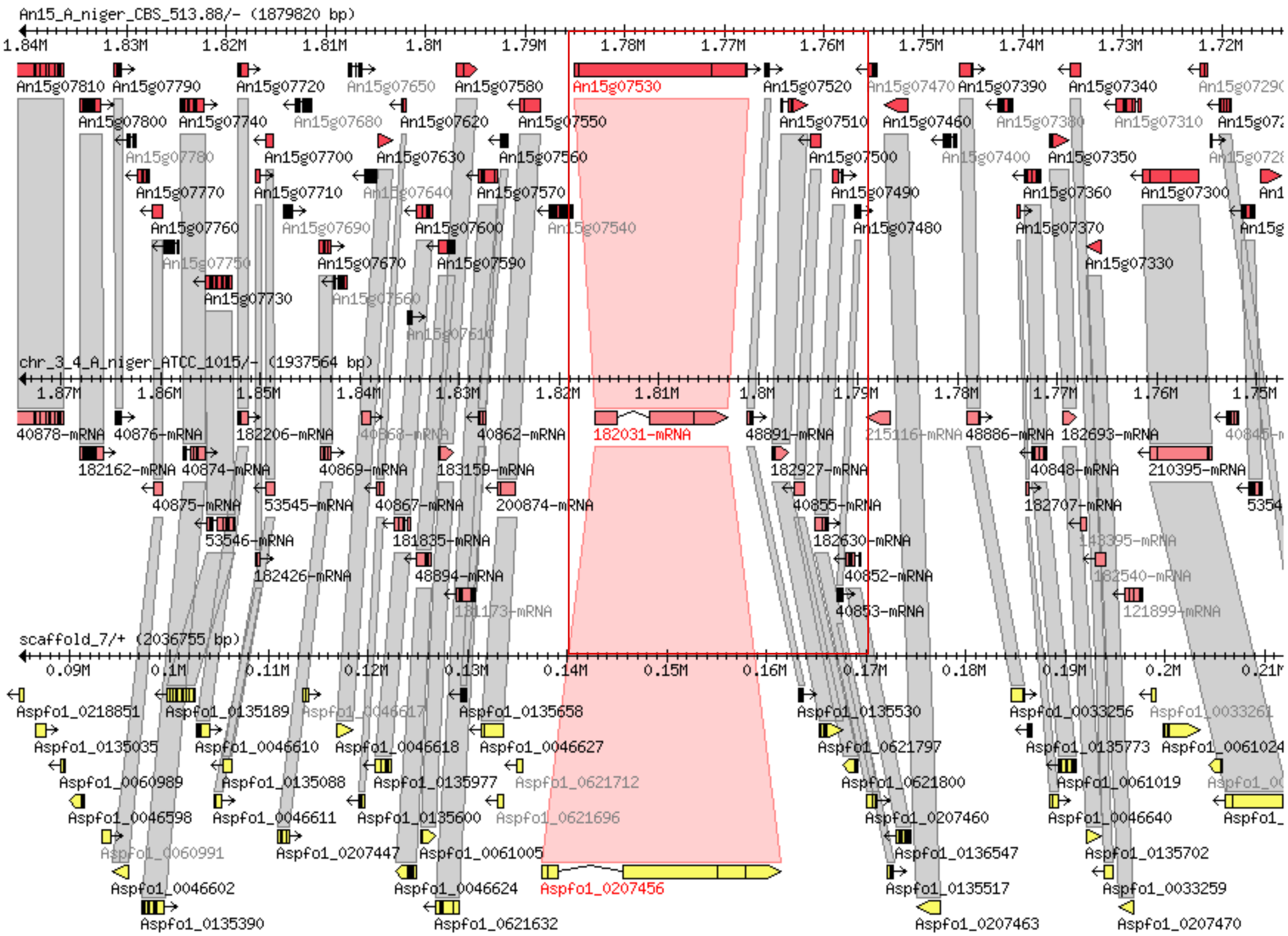

An15g07530 cluster

Gene id (SMURF)	Gene positions	Gene distance	AspGD name	AspGD description line or supplemental annotation	protein ID (antiSMASH)	AspGD ID	
n/a				Protein of unknown function	prot_ID_694	An15g07400	
n/a				Oligopeptide transporter	prot_ID_219	An15g07460	
n/a				Protein of unknown function	prot_ID_265	An15g07470	
n/a				Ortholog of A. nidulans FGSC A4 : AN0020, A. fumigatus Af293 : Afu2g00190, A. niger ATCC 1015 : 40853-mRNA, A. versicolor : Aspve1_0365574 and A. sydowii : Aspsy1_0476409	prot_ID_488	An15g07480	ECS
n/a				Domain(s) with predicted oxidoreductase activity and role in oxidation-reduction process	prot_ID_329	An15g07490	
n/a				Putative ribokinase	prot_ID_402	An15g07500	
An15g07510	2	1030		Domain(s) with predicted transporter activity, role in oligopeptide transport and membrane localization	prot_ID_138	An15g07510	
An15g07520	1	1592		Domain(s) with predicted catalytic activity and role in metabolic process	prot_ID_338	An15g07520	
**An15g07530**	0	0		Putative nonribosomal peptide synthase (NRPS)	**prot_ID_453**	**An15g07530**	ECS
An15g07540	-1	224		Similar to M. grisea PTH11	prot_ID_592	An15g07540	
An15g07550	-2	1164		Ortholog of A. nidulans FGSC A4 : AN11921, A. fumigatus Af293 : Afu8g07200, A. oryzae RIB40 : AO090701000752, A. niger ATCC 1015 : 200874-mRNA and A. versicolor : Aspve1_0131033	prot_ID_506	An15g07550	
An15g07560	-3	1629		Ortholog of A. nidulans FGSC A4 : AN7378, A. fumigatus Af293 : Afu4g14205, A. oryzae RIB40 : AO090103000092, A. niger ATCC 1015 : 40862-mRNA and A. versicolor : Aspve1_0187655	prot_ID_613	An15g07560	
An15g07570	-4	360		Domain(s) with predicted arylsulfatase activity and role in phenol-containing compound metabolic process	prot_ID_660	An15g07570	
An15g07580	-5	214		Domain(s) with predicted oxidoreductase activity and role in oxidation-reduction process	prot_ID_534	An15g07580	
An15g07590	-6	627		Domain(s) with predicted role in transmembrane transport and integral to membrane localization	prot_ID_54	An15g07590	
An15g07600	-7	586		Domain(s) with predicted role in transmembrane transport and integral to membrane localization	prot_ID_563	An15g07600	
n/a				Protein of unknown function	prot_ID_189	An15g07610	

n/a	Ortholog of A. brasiliensis : Aspbr1_0034734, A. acidus : Aspfo1_0135600, A. niger ATCC 1015 : 40867-mRNA and Aspergillus carbonarius ITEM 5010 : Acar5010_209559	prot_ID_309	An15g07620
n/a	Ortholog of A. niger CBS 513.88 : An12g03510, A. acidus : Aspfo1_0046618, A. niger ATCC 1015 : 42488-mRNA and Aspergillus carbonarius ITEM 5010 : Acar5010_053374	prot_ID_247	An15g07630
n/a	Protein of unknown function	prot_ID_134	An15g07640

An15g07910 cluster

Rare A. carbonarius match

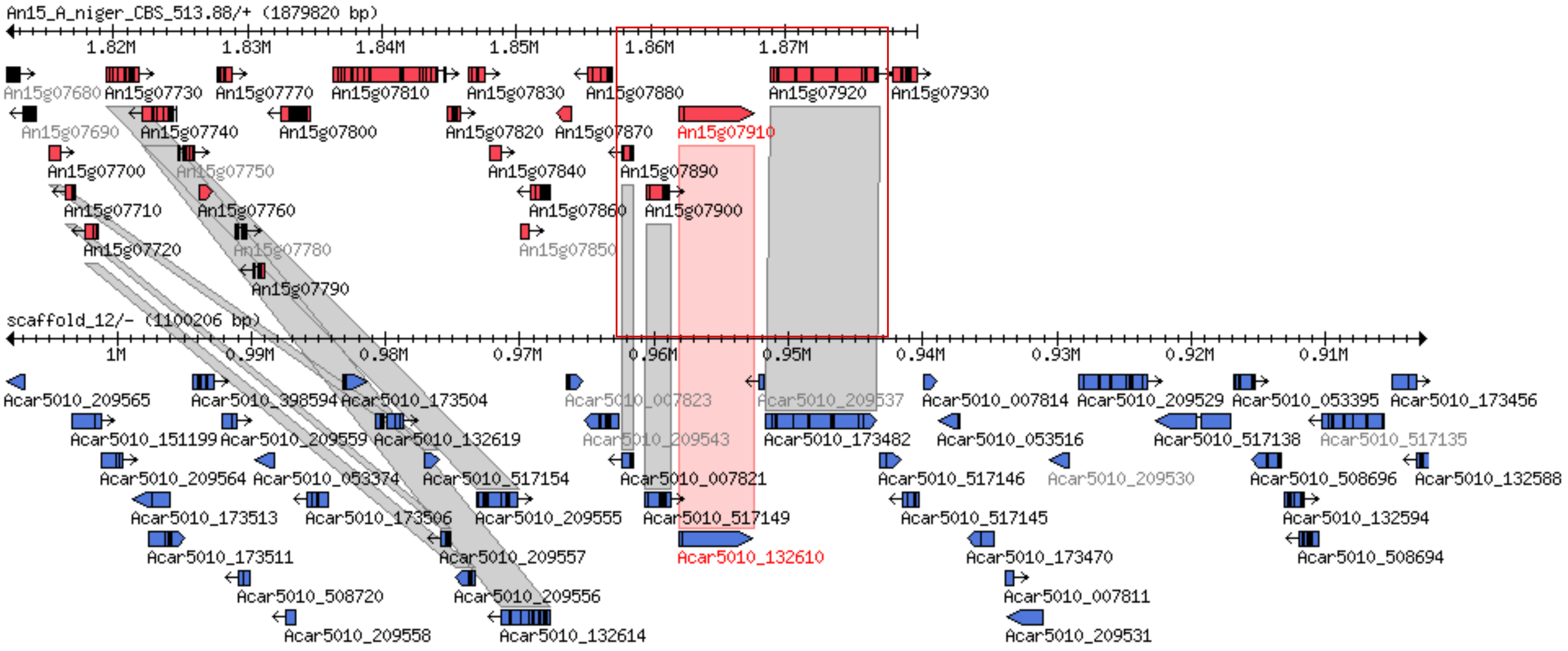

An15g07910 cluster

Gene id (SMURF)	Gene positions	Gene distance	AspGD name	AspGD description line or supplemental annotation	protein ID (antiSMASH)	AspGD ID	
n/a			agsB	Putative alpha-glucan synthase	prot_ID_313	An15g07810	
n/a				Ortholog of A. brasiliensis : Aspbr1_0078933, A. acidus : Aspfo1_0046583, A. niger ATCC 1015 : 53547-mRNA and Aspergillus carbonarius ITEM 5010 : Acar5010_158371	prot_ID_700	An15g07820	
An15g07830	8	355		Domain(s) with predicted hydrolase activity	prot_ID_124	An15g07830	
An15g07840	7	1433		Ortholog of A. nidulans FGSC A4 : AN2942, AN10320, AN7130, A. fumigatus Af293 : Afu3g15110 and A. niger CBS 513.88 : An03g05620, An12g07260, An03g02580, An01g14840	prot_ID_298	An15g07840	
An15g07850	6	55		Protein of unknown function	prot_ID_405	An15g07850	
An15g07860	5	466		Domain(s) with predicted nucleotide binding, oxidoreductase activity and role in oxidation-reduction process	prot_ID_302	An15g07860	
An15g07870	4	1260		Domain(s) with predicted nucleotide binding, oxidoreductase activity, zinc ion binding activity and role in oxidation-reduction process	prot_ID_432	An15g07870	
An15g07880	3	682		Domain(s) with predicted monooxygenase activity and role in oxidation-reduction process	prot_ID_754	An15g07880	
An15g07890	2	1022		Domain(s) with predicted sequence-specific DNA binding, sequence-specific DNA binding transcription factor activity, role in regulation of transcription, DNA-dependent and nucleus localization	prot_ID_59	An15g07890	ECS
An15g07900	1	541		Domain(s) with predicted electron carrier activity, heme binding, iron ion binding, oxidoreductase activity, acting on paired donors, with incorporation or reduction of molecular oxygen activity and role in oxidation-reduction process	prot_ID_383	An15g07900	
**An15g07910**	0	0		Putative nonribosomal peptide synthase (NRPS)	**prot_ID_533**	**An15g07910**	
An15g07920	-1	1121		Protein required for ochratoxin A biosynthesis in black Aspergillus	prot_ID_675	An15g07920	ECS
An15g07930	-2	1032		Domain(s) with predicted heme binding, nitric-oxide synthase activity and role in nitric oxide biosynthetic process, oxidation-reduction process	prot_ID_68	An15g07930	

An15g07920 cluster
No manual prediction made

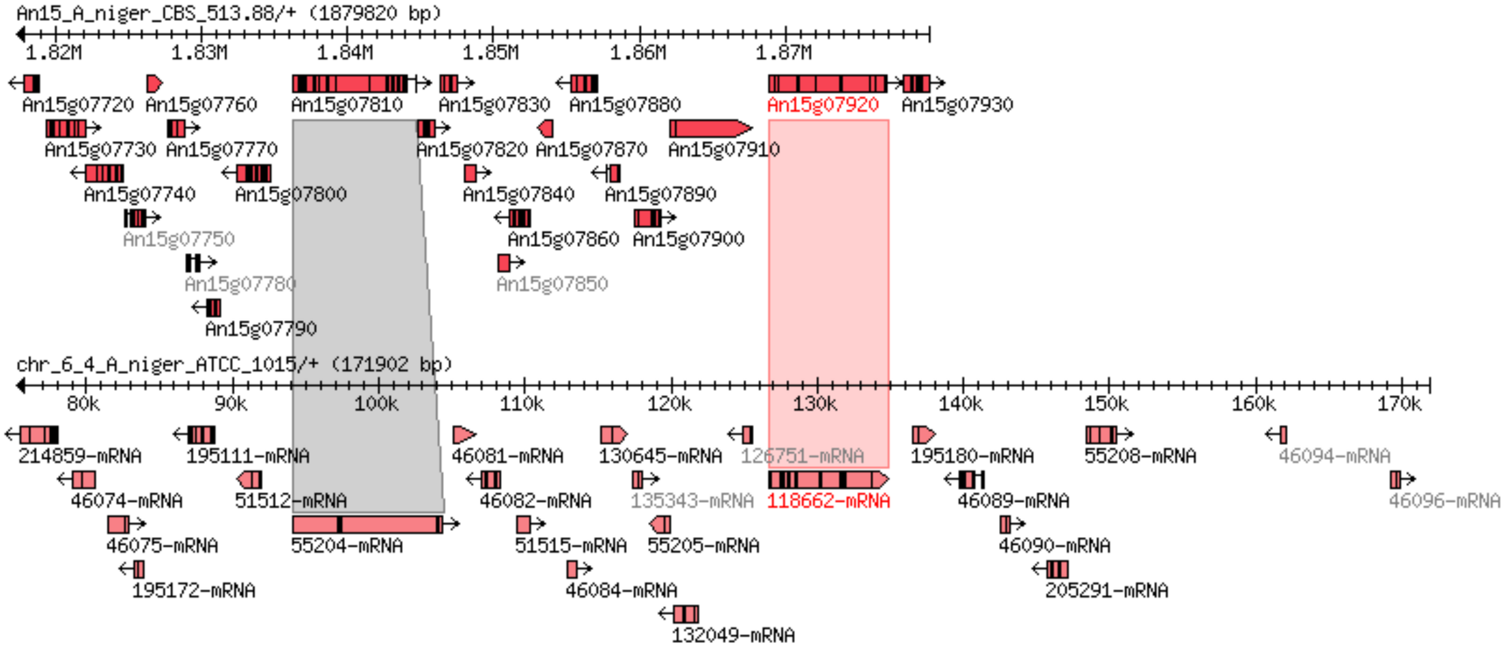

An15g07920 cluster

Gene id (SMURF)	Gene positions	Gene distance	AspGD name	AspGD description line or supplemental annotation	protein ID (antiSMASH)	AspGD ID
An15g07830	9	355		Domain(s) with predicted hydrolase activity	n/a	An15g07830
An15g07840	8	1433		Ortholog of A. nidulans FGSC A4 : AN2942, AN10320, AN7130, A. fumigatus Af293 : Afu3g15110 and A. niger CBS 513.88 : An03g05620, An12g07260, An03g02580, An01g14840	n/a	An15g07840
An15g07850	7	55			n/a	An15g07850
An15g07860	6	466		Domain(s) with predicted nucleotide binding, oxidoreductase activity and role in oxidation-reduction process	n/a	An15g07860
An15g07870	5	1260		Domain(s) with predicted nucleotide binding, oxidoreductase activity, zinc ion binding activity and role in oxidation-reduction process	n/a	An15g07870
An15g07880	4	682		Domain(s) with predicted monooxygenase activity and role in oxidation-reduction process	n/a	An15g07880
An15g07890	3	1022		Domain(s) with predicted sequence-specific DNA binding, sequence-specific DNA binding transcription factor activity, role in regulation of transcription, DNA-dependent and nucleus localization	n/a	An15g07890
An15g07900	2	541		Domain(s) with predicted electron carrier activity, heme binding, iron ion binding, oxidoreductase activity, acting on paired donors, with incorporation or reduction of molecular oxygen activity and role in oxidation-reduction process	n/a	An15g07900
An15g07910	1	1121		Domain(s) with predicted ACP phosphopantetheine attachment site binding involved in fatty acid biosynthetic process, ligase activity, phosphopantetheine binding activity	n/a	An15g07910
**An15g07920**	0	0		Protein required for ochratoxin A biosynthesis in black Aspergillus	n/a	**An15g07920**
An15g07930	-1	1032		Domain(s) with predicted heme binding, nitric-oxide synthase activity and role in nitric oxide biosynthetic process, oxidation-reduction process	n/a	An15g07930

An16g00600 cluster

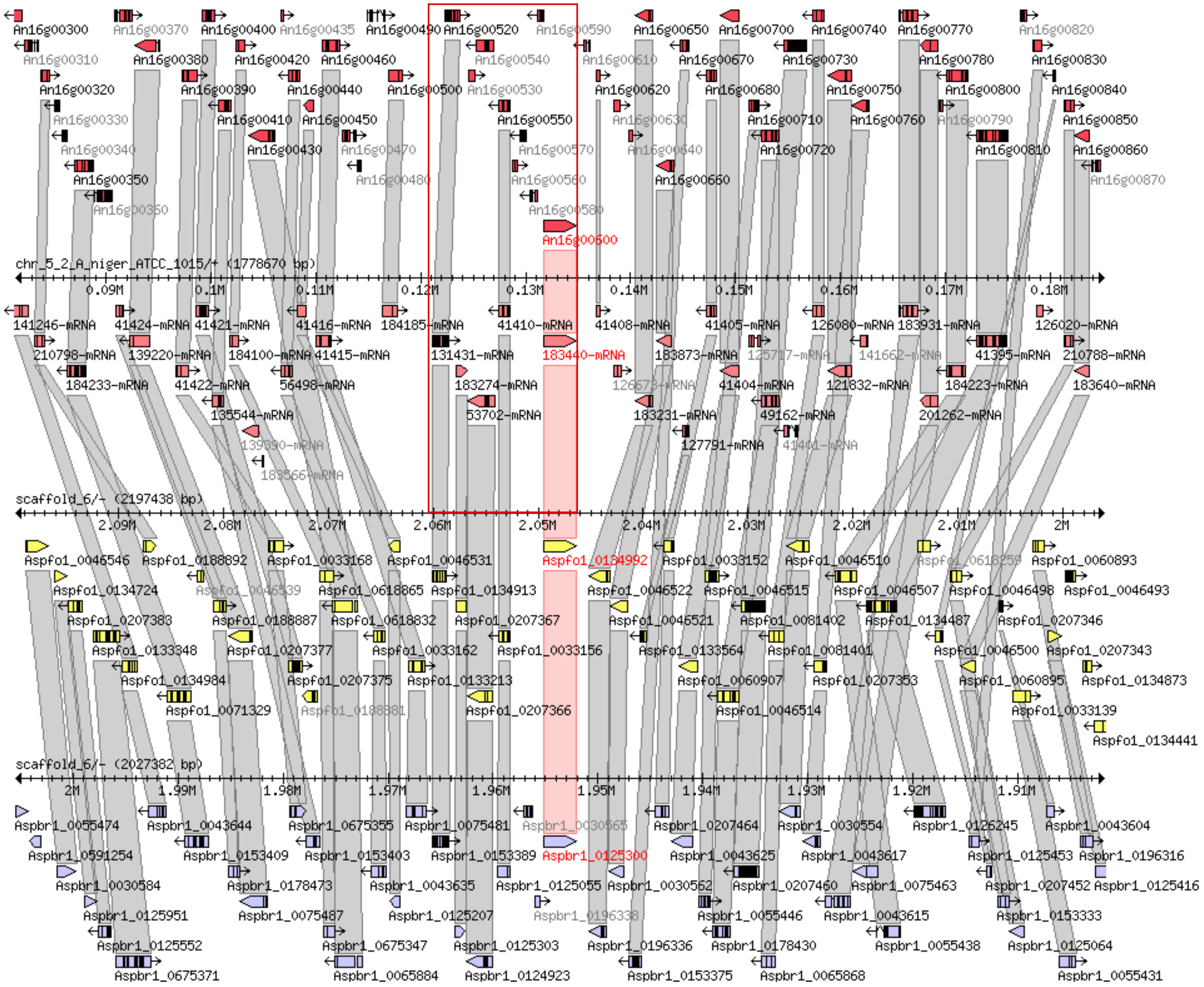

An16g00600 cluster

Gene id (SMURF)	Gene positions	Gene distance	AspGD name	AspGD description line or supplemental annotation	protein ID (antiSMASH)	AspGD ID	
n/a				Domain(s) with predicted role in transmembrane transport and integral to membrane localization	prot_ID_707	An16g00460	
n/a					prot_ID_421	An16g00470	
n/a					prot_ID_457	An16g00480	
n/a				Ortholog of A. acidus : Aspfo1_0126742	prot_ID_777	An16g00490	
n/a				Ortholog of A. fumigatus Af293 : Afu3g10850, A. oryzae RIB40 : AO090102000071, N. fischeri NRRL 181 : NFIA_032600 and Aspergillus flavus NRRL 3357 : AFL2T_05184	prot_ID_415	An16g00500	
An16g00520	8	779		Domain(s) with predicted monooxygenase activity and role in oxidation-reduction process	prot_ID_909	An16g00520	ECS
An16g00530	7	149		Domain(s) with predicted endodeoxyribonuclease activity, producing 5'-phosphomonoesters activity	prot_ID_830	An16g00530	
An16g00540	6	496		Domain(s) with predicted catalytic activity	prot_ID_507	An16g00540	
An16g00550	5	222		Putative benzonitrilase	prot_ID_325	An16g00550	
An16g00560	4	313		Protein of unknown function	prot_ID_562	An16g00560	
An16g00570	3	367		Protein of unknown function	prot_ID_878	An16g00570	
An16g00580	2	37		Protein of unknown function	prot_ID_633	An16g00580	
An16g00590	1	39		Protein of unknown function	prot_ID_655	An16g00590	
**An16g00600**	0	0		Domain(s) with predicted ACP phosphopantetheine attachment site binding involved in fatty acid biosynthetic process, L-aminoadipate-semialdehyde dehydrogenase activity, nucleotide binding activity	**prot_ID_739**	**An16g00600**	**ECS**
An16g00610	-1	761			prot_ID_509	An16g00610	
An16g00620	-2	517		Ortholog of A. niger ATCC 1015 : 41408-mRNA	prot_ID_178	An16g00620	
An16g00630	-3	1297		Domain(s) with predicted peroxidase activity	prot_ID_774	An16g00630	
An16g00640	-4	866			prot_ID_752	An16g00640	
An16g00650	-5	276		Domain(s) with predicted role in transmembrane transport and integral to membrane localization	prot_ID_101	An16g00650	
An16g00660	-6	318		Ortholog of A. nidulans FGSC A4 : AN8162, A. fumigatus Af293 : Afu5g01210, A. oryzae RIB40 : AO090102000383, A. niger ATCC 1015 : 183873-mRNA and A. versicolor : Aspve1_0140072, Aspve1_0155676	prot_ID_294	An16g00660	
An16g00670	-7	535		Ortholog of A. brasiliensis : Aspbr1_0153375, Aspergillus carbonarius ITEM 5010 : Acar5010_206987 and Aspergillus aculeatus ATCC16872 : Aacu16872_042695	prot_ID_398	An16g00670	

An16g00680	-8	1613	Domain(s) with predicted role in response to stress and integral to membrane localization	prot_ID_459	An16g00680
An16g00700	-9	444	Domain(s) with predicted 2 iron, 2 sulfur cluster binding, oxidoreductase activity and role in oxidation-reduction process	prot_ID_640	An16g00700
An16g00710	-10	1014	Domain(s) with predicted calcium ion binding activity	prot_ID_913	An16g00710
An16g00720	-11	161	Domain(s) with predicted role in transmembrane transport and integral to membrane localization	prot_ID_889	An16g00720
An16g00730	-12	481	Domain(s) with predicted role in transmembrane transport and integral to membrane localization	prot_ID_205	An16g00730
An16g00740	-13	545	Domain(s) with predicted nucleotide binding activity	n/a	An16g00740

An16g06720 cluster

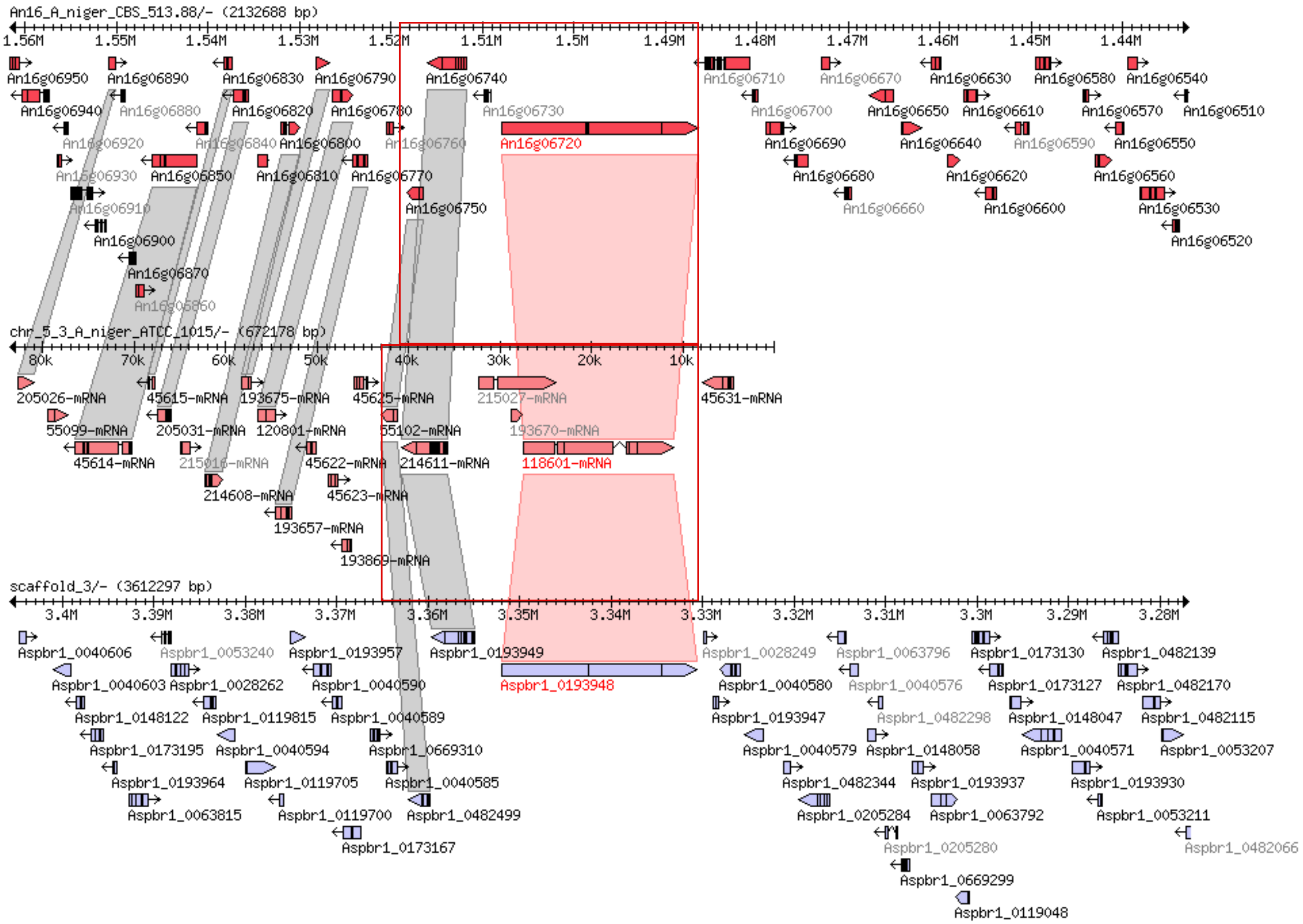

An16g06720 cluster

Gene id (SMURF)	Gene positions	Gene distance	AspGD name	AspGD description line or supplemental annotation	protein ID (antiSMASH)	AspGD ID	
An16g06570	15	3530		Ortholog of A. brasiliensis : Aspbr1_0207064, A. niger ATCC 1015 : 40897-mRNA and Aspergillus carbonarius ITEM 5010 : Acar5010_157234	n/a	An16g06570	
An16g06580	14	742		Domain(s) with predicted substrate-specific transmembrane transporter activity, role in transmembrane transport and integral to membrane localization	n/a	An16g06580	
An16g06590	13	2118		Domain(s) with predicted cation binding, glucosidase activity and role in carbohydrate metabolic process	n/a	An16g06590	
An16g06600	12	803		Putative beta-galactosidase	n/a	An16g06600	
An16g06610	11	456		Domain(s) with predicted substrate-specific transmembrane transporter activity, role in transmembrane transport and integral to membrane localization	n/a	An16g06610	
An16g06620	10	820		1,4-Lactonase (gamma-lactonase)	n/a	An16g06620	
An16g06630	9	830		Domain(s) with predicted nucleotide binding, oxidoreductase activity and role in oxidation-reduction process	n/a	An16g06630	
An16g06640	8	941		Domain(s) with predicted DNA binding, sequence-specific DNA binding RNA polymerase II transcription factor activity, sequence-specific DNA binding transcription factor activity, zinc ion binding activity	n/a	An16g06640	
An16g06650	7	1835		Domain(s) with predicted DNA binding, sequence-specific DNA binding RNA polymerase II transcription factor activity, sequence-specific DNA binding transcription factor activity, zinc ion binding activity	prot_ID_267	An16g06650	
An16g06660	6	1661		Protein of unknown function	prot_ID_458	An16g06660	
An16g06670	5	1458		Protein of unknown function	prot_ID_425	An16g06670	
An16g06680	4	1087		Ortholog of A. oryzae RIB40 : AO090011000713 and Aspergillus flavus NRRL 3357 : AFL2T_05446	prot_ID_168	An16g06680	
An16g06690	3	840		Domain(s) with predicted catalytic activity and role in nucleoside metabolic process	prot_ID_286	An16g06690	
An16g06700	2	98		Protein of unknown function	prot_ID_308	An16g06700	
An16g06710	1	859		Protein of unknown function	prot_ID_198	An16g06710	
**An16g06720**	0	0		Ortholog(s) have role in brevianamide F biosynthetic process, emericellamide biosynthetic process, pathogenesis, secondary metabolite biosynthetic process, spore germination	**prot_ID_855**	**An16g06720**	ECS

An16g06730	-1	1232	Questionable ORF	prot_ID_664	An16g06730	
An16g06740	-2	1917	Similar to cadmium resistance protein; expression repressed by tunicamycin and DTT	prot_ID_620	An16g06740	
An16g06750	-3	652	Protein with similarity to D-stereospecific aminopeptidase; acid protease; expression induced by tunicamycin and DTT	prot_ID_605	An16g06750	ECS
An16g06760	-4	1442	Domain(s) with predicted role in response to stress and integral to membrane localization	prot_ID_553	An16g06760	
An16g06770	-5	2102	Domain(s) with predicted role in transmembrane transport and integral to membrane localization	prot_ID_669	An16g06770	
An16g06780	-6	97	Domain(s) with predicted DNA binding, sequence-specific DNA binding RNA polymerase II transcription factor activity, sequence-specific DNA binding transcription factor activity, zinc ion binding activity	prot_ID_77	An16g06780	
n/a			Ortholog of A. nidulans FGSC A4 : AN2394, A. niger CBS 513.88 : An03g06300, A. oryzae RIB40 : AO090010000576, A. brasiliensis : Aspbr1_0201554 and A. niger ATCC 1015 : 191411-mRNA, 193675-mRNA	prot_ID_496	An16g06790	

An18g00520 cluster

Second rare *A. carbonarius* cluster match

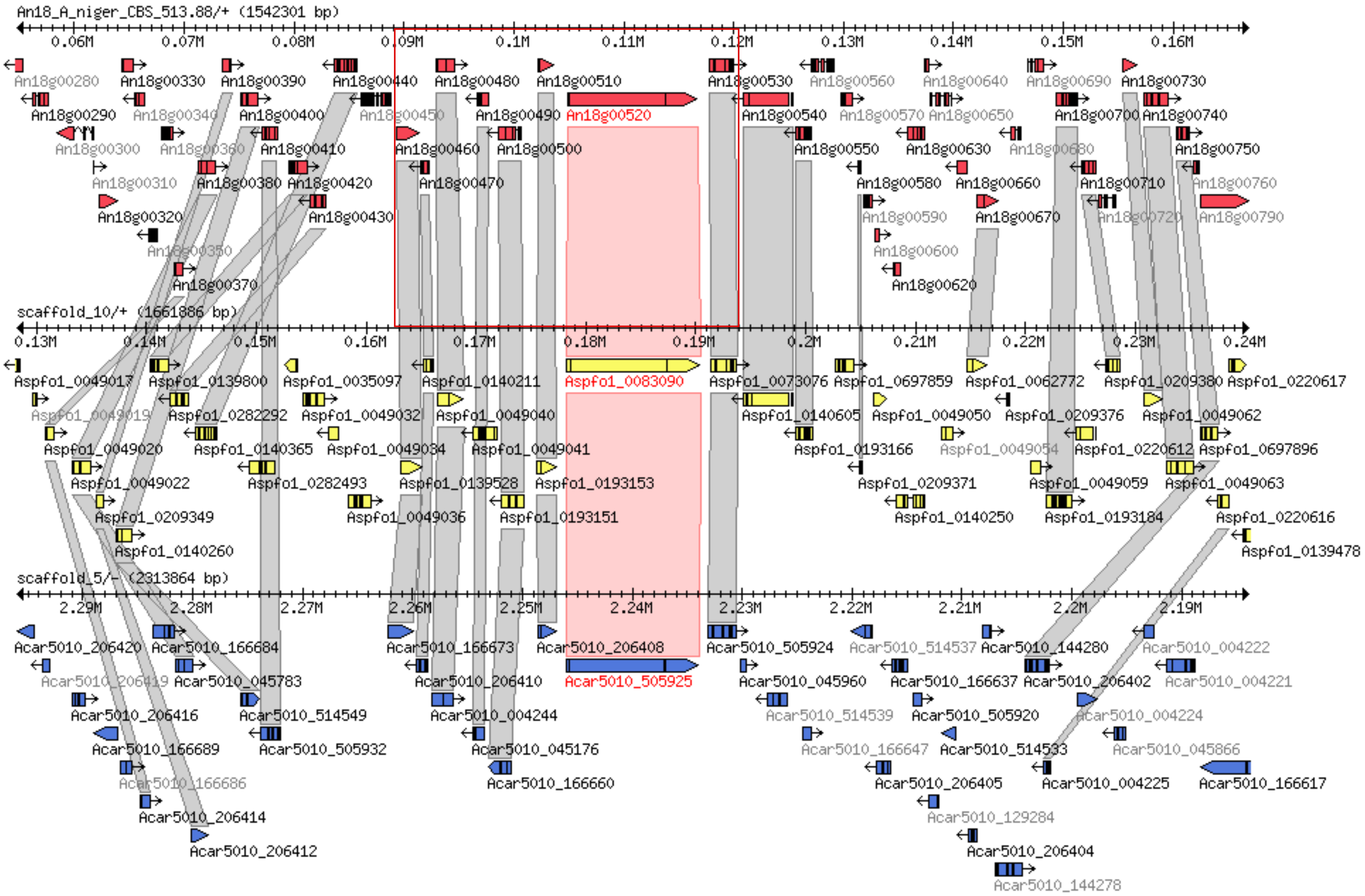

An18g00520 cluster

Gene id (SMURF)	Gene positions	Gene distance	AspGD name	AspGD description line or supplemental annotation	protein ID (antiSMASH)	AspGD ID	
An18g00350	17	356			n/a	An18g00350	
An18g00360	16	193			n/a	An18g00360	
An18g00370	15	1388		Ortholog of A. niger CBS 513.88 : An04g06750, A. brasiliensis : Aspbr1_0033619, Aspbr1_0047824, A. acidus : Aspfo1_0049020 and A. niger ATCC 1015 : 54072-mRNA	n/a	An18g00370	
An18g00380	14	464		Domain(s) with predicted role in transmembrane transport and integral to membrane localization	n/a	An18g00380	
An18g00390	13	848		Ortholog of A. nidulans FGSC A4 : AN3872, AN5090, A. fumigatus Af293 : Afu1g01360, A. niger ATCC 1015 : 127004-mRNA, A. versicolor : Aspve1_0041846 and A. sydowii : Aspsy1_0121091	n/a	An18g00390	
An18g00400	12	232		Putative inorganic phosphate transporter	n/a	An18g00400	
An18g00410	11	1090		Ortholog of A. nidulans FGSC A4 : AN3982, A. oryzae RIB40 : AO090003000863, A. niger ATCC 1015 : 187009-mRNA, Aspergillus flavus NRRL 3357 : AFL2T_02158 and A. versicolor : Aspve1_0188186	n/a	An18g00410	
An18g00420	10	277		Ortholog of A. brasiliensis : Aspbr1_0047280, A. acidus : Aspfo1_0139800, A. niger ATCC 1015 : 123450-mRNA and Aspergillus carbonarius ITEM 5010 : Acar5010_011222	n/a	An18g00420	
An18g00430	9	877		Ortholog of A. nidulans FGSC A4 : AN11159, A. niger CBS 513.88 : An01g01220, A. brasiliensis : Aspbr1_0058364, A. niger ATCC 1015 : 188035-mRNA and A. clavatus NRRL 1 : ACLA_074330	n/a	An18g00430	
An18g00440	8	483		Domain(s) with predicted substrate-specific transmembrane transporter activity, role in transmembrane transport and integral to membrane localization	prot_ID_43	An18g00440	
An18g00450	7	462			prot_ID_658	An18g00450	
An18g00460	6	157		Ortholog of A. nidulans FGSC A4 : AN2818, A. niger CBS 513.88 : An12g03370, An07g00580, A. oryzae RIB40 : AO090103000478, A. niger ATCC 1015 : 180130-mRNA and A. versicolor : Aspve1_0051884	prot_ID_207	An18g00460	ECS
An18g00470	5	535		Domain(s) with predicted hydrolase activity	prot_ID_100	An18g00470	
An18g00480	4	1991		Domain(s) with predicted role in transmembrane transport and integral to membrane localization	prot_ID_53	An18g00480	
An18g00490	3	923		Domain(s) with predicted monooxygenase activity and role in oxidation-reduction process	prot_ID_469	An18g00490	

An18g00500	2	1489	Domain(s) with predicted electron carrier activity, heme binding, iron ion binding, monooxygenase activity, oxidoreductase activity, acting on paired donors, with incorporation or reduction of molecular oxygen activity	prot_ID_132	An18g00500	
An18g00510	1	1327	Domain(s) with predicted UDP-N-acetylmuramate dehydrogenase activity, flavin adenine dinucleotide binding activity and role in oxidation-reduction process	prot_ID_383	An18g00510	
**An18g00520**	0	0	Domain(s) with predicted ACP phosphopantetheine attachment site binding involved in fatty acid biosynthetic process, ligase activity, nucleotide binding and oxidoreductase activity, more	**prot_ID_101**	**An18g00520**	
An18g00530	-1	1078	Domain(s) with predicted choline dehydrogenase activity, flavin adenine dinucleotide binding, nucleotide binding activity and role in alcohol metabolic process, oxidation-reduction process	prot_ID_31	An18g00530	ECS
An18g00540	-2	761	Domain(s) with predicted catalytic activity and role in nucleoside metabolic process	prot_ID_475	An18g00540	
An18g00550	-3	211	Domain(s) with predicted O-methyltransferase activity, protein dimerization activity	prot_ID_153	An18g00550	
n/a				prot_ID_483	An18g00560	
n/a				prot_ID_124	An18g00570	
n/a			Ortholog of A. acidus : Aspfo1_0209371	prot_ID_582	An18g00580	
n/a				prot_ID_136	An18g00590	
n/a				prot_ID_12	An18g00600	
n/a			Domain(s) with predicted transporter activity, role in transport and membrane localization	prot_ID_373	An18g00620	
n/a			Ortholog of Aspergillus carbonarius ITEM 5010 : Acar5010_206398 and Aspergillus aculeatus ATCC16872 : Aacu16872_064631	prot_ID_577	An18g00630	
n/a			Domain(s) with predicted role in intracellular protein transport, vesicle-mediated transport and Golgi apparatus part, clathrin adaptor complex localization	prot_ID_284	An18g05740	
n/a			Ortholog of A. nidulans FGSC A4 : AN5711, A. fumigatus Af293 : Afu1g06690, A. oryzae RIB40 : AO090001000465, A. niger ATCC 1015 : 202631-mRNA and A. versicolor : Aspve1_0053488	prot_ID_640	An18g05750	
n/a			Ortholog(s) have role in response to endoplasmic reticulum stress	prot_ID_317	An18g05760	
n/a			Domain(s) with predicted ATP binding, unfolded protein binding activity and role in protein folding	prot_ID_332	An18g05770	
n/a			Ortholog of A. nidulans FGSC A4 : AN2554, A. fumigatus Af293 : Afu1g06730, A. oryzae RIB40 : AO090001000761, A. niger ATCC 1015 : 42981-mRNA and A. versicolor : Aspve1_0026511	prot_ID_455	An18g05780	

n/a	Domain(s) with predicted nucleic acid binding, zinc ion binding activity	prot_ID_130	An18g05790
n/a	Ortholog of A. nidulans FGSC A4 : AN5714, A. fumigatus Af293 : Afu1g06762, A. oryzae RIB40 : AO090001000477, A. niger ATCC 1015 : 187314-mRNA and A. versicolor : Aspve1_0042424	prot_ID_305	An18g05800
n/a	Protein with similarity to cytoplasmic ribosomal protein of the small subunit S26	prot_ID_44	An18g05810
n/a	Domain(s) with predicted catalytic activity and role in metabolic process	prot_ID_73	An18g05820

Abbreviations: ECS, end of cluster synteny; IGD, increase in intergenic distance; FA, change in functional annotation; ED, experimentally determined
