# Supplementary material for: Comprehensive annotation of secondary metabolite biosynthetic genes and gene clusters of Aspergillus nidulans, A. fumigatus, A. niger and A. oryzae
Source: BMC Microbiol. 2013 Apr 26;13:91. doi: 10.1186/1471-2180-13-91 (PMC3689640; doi:10.1186/1471-2180-13-91)
Supplement: Additional file 5 — A table listing manually annotated gene clusters predicted by SMURF and antiSMASH for A. oryzae. [file 1471-2180-13-91-S5.pdf]

Additional file 5

Comprehensive annotation of secondary metabolite biosynthetic genes and gene clusters of *Aspergillus nidulans*, *A. fumigatus*, *A. niger* and *A. oryzae*

Diane O. Inglis, Jon Binkley, Marek Skrzypek, Martha B. Arnaud, Gustavo Cerqueira, Prach Shah, Farrell Wymore, Jennifer R. Wortman and Gavin Sherlock

Abbreviations: ECS, end of cluster synteny; IGD, increase in intergenic distance; FA, change in functional annotation; ED, experimentally determined

Csypyrone B1 cluster
Experimentally detemined cluster

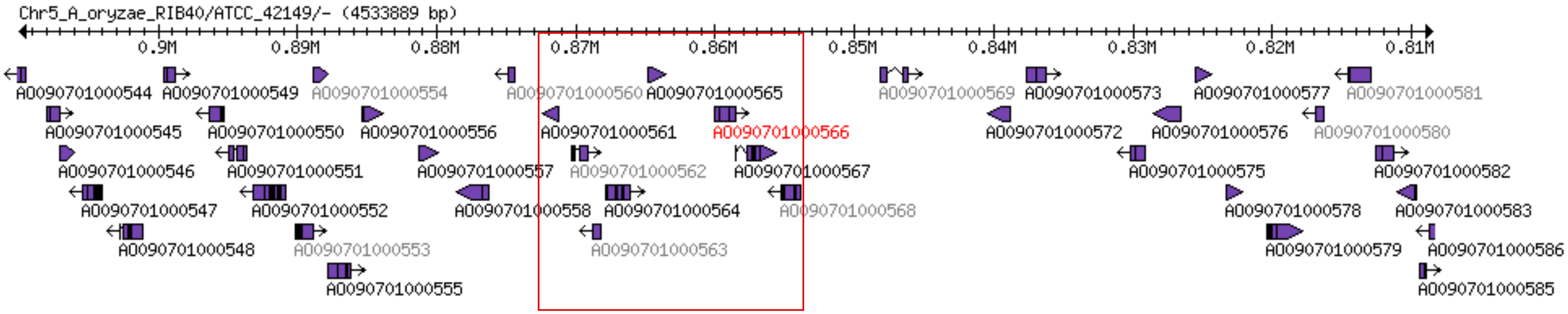

Gene id (SMURF)	Gene positions	Gene distance	Gene Name	Annotated_gene_function	antiSMASH ID	AspGD ID	
n/a				Protein of unknown function	prot_ID_517	AO090701000569	
n/a				Protein of unknown function	prot_ID_32	AO090701000560	
n/a				Putative oxidoreductase	prot_ID_1143	AO090701000561	ED
n/a				Putative dioxygenase	prot_ID_696	AO090701000562	
n/a				Protein of unknown function	prot_ID_268	AO090701000563	
n/a				Putative aminopeptidase or acyl esterase	prot_ID_741	AO090701000564	
n/a				Domain(s) with predicted zinc ion binding activity	prot_ID_585	AO090701000565	
n/a			cysB	Putative type III polyketide synthase; responsible for csypyrone B1 biosynthesis	**prot_ID_1397**	**AO090701000566**	
n/a				Putative transporter	prot_ID_703	AO090701000567	
n/a				Putative TolB protein	prot_ID_1167	AO090701000568	ED

Dipeptidyl Peptidase IV 2 Inhibitor (WYK-1) cluster

Experimentally determined cluster

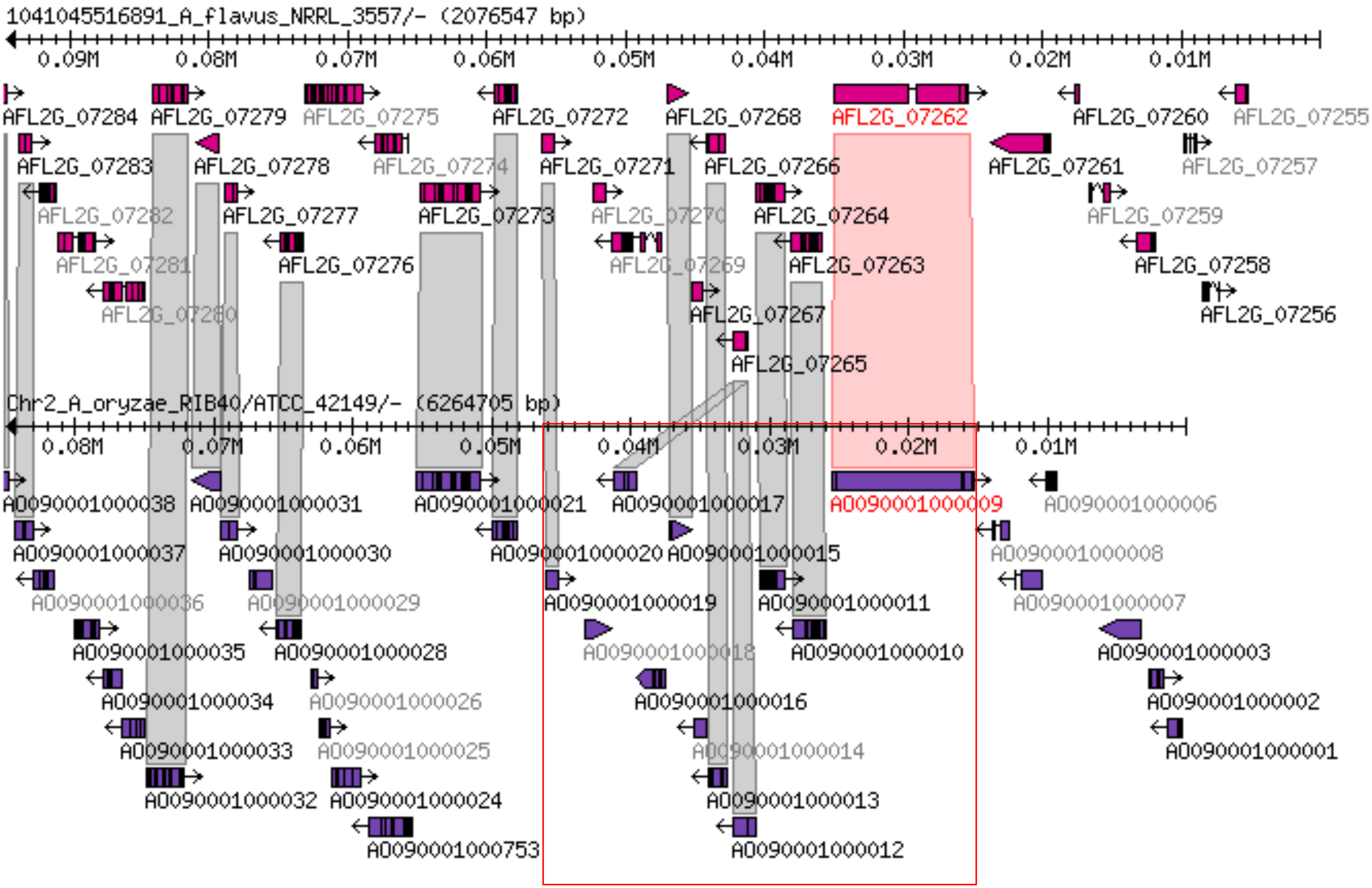

Dipeptidyl Peptidase IV 2 Inhibitor (WYK-1) cluster

Gene id (SMURF)	Gene positions	Gene distance	Gene Name	Annotated_gene_function	antiSMASH ID	AspGD ID	
AO090001000031	-20	467		Domain(s) with predicted role in transmembrane transport and integral to membrane localization	n/a	AO090001000031	
AO090001000030	-19	1305		Ortholog of gliK, involved in gliotoxin biosynthesis	n/a	AO090001000030	
AO090001000029	-18	104		Domain(s) with predicted sequence-specific DNA binding RNA polymerase II transcription factor activity, zinc ion binding activity, role in regulation of transcription, DNA-dependent and nucleus localization	n/a	AO090001000029	
AO090001000028	-17	778		Cytochrome P450 monooxygenase	n/a	AO090001000028	
AO090001000026	-16	131		Domain(s) with predicted electron carrier activity, heme binding, iron ion binding, oxidoreductase activity, acting on paired donors, with incorporation or reduction of molecular oxygen activity and role in oxidation-reduction process	n/a	AO090001000026	
AO090001000025	-15	202		Domain(s) with predicted electron carrier activity, heme binding, iron ion binding, monooxygenase activity, oxidoreductase activity, acting on paired donors, with incorporation or reduction of molecular oxygen activity	n/a	AO090001000025	
AO090001000024	-14	1000		Domain(s) with predicted oxidoreductase activity and role in metabolic process	n/a	AO090001000024	
AO090001000753	-13	468		Domain(s) with predicted DNA binding, sequence-specific DNA binding RNA polymerase II transcription factor activity, zinc ion binding activity and role in regulation of transcription, DNA-dependent, transcription, DNA-dependent	n/a	AO090001000753	
AO090001000021	-12	843		Domain(s) with predicted ATP binding, ATPase activity, coupled to transmembrane movement of substances activity, role in transmembrane transport and integral to membrane localization	n/a	AO090001000021	
AO090001000020	-11	2034		Domain(s) with predicted role in transmembrane transport and integral to membrane localization	n/a	AO090001000020	
AO090001000019	-10	2086	wykD	Haloacid dehalogenase, type II; putative member of the WYK-1 (Dipeptidyl Peptidase IV 2 Inhibitor) gene cluster	prot_ID_1063	AO090001000019	ED
AO090001000018	-9	427	wykR	C6 finger domain protein; putative member of the WYK-1 (Dipeptidyl Peptidase IV 2 Inhibitor) gene cluster	prot_ID_1468	AO090001000018	
AO090001000017	-8	710	wykC	Putative iron / ascorbate dependent oxidoreductase; putative member of the WYK-1 (Dipeptidyl Peptidase IV 2 Inhibitor) gene cluster	prot_ID_932	AO090001000017	
AO090001000016	-7	444		Domain(s) with predicted transporter activity, role in oligopeptide transport and membrane localization	prot_ID_1237	AO090001000016	
AO090001000015	-6	398	wykB	Putative FAD-dependent oxidoreductase; putative member of the WYK-1 (Dipeptidyl Peptidase IV 2 Inhibitor) gene cluster	prot_ID_609	AO090001000015	
AO090001000014	-5	285	wykI	Putative O-methyltransferase; putative member of the WYK-1 (Dipeptidyl Peptidase IV 2 Inhibitor) gene cluster	prot_ID_1030	AO090001000014	

AO090001000013	-4	537	wykH	N-methyltransferase; putative member of the WYK-1 (Dipeptidyl Peptidase IV 2 Inhibitor) gene cluster	prot_ID_64	AO090001000013	
AO090001000012	-3	644	wykG	Putative oxidase; putative member of the WYK-1 (Dipeptidyl Peptidase IV 2 Inhibitor) gene cluster	prot_ID_825	AO090001000012	
AO090001000011	-2	491	wykE	Oligopeptide transporter; putative member of the WYK-1 (Dipeptidyl Peptidase IV 2 Inhibitor) gene cluster	prot_ID_242	AO090001000011	
AO090001000010	-1	430	wykA	Putative phenol hydroxylase; putative member of the WYK-1 (Dipeptidyl Peptidase IV 2 Inhibitor) gene cluster	prot_ID_707	AO090001000010	
**AO090001000009**	0	0	wykN	Non-ribosomal peptide synthase (NRPS) involved in the synthesis of a dipeptidyl peptidase IV 2 inhibitor	prot_ID_140	AO090001000009	ED
n/a				Protein of unknown function	prot_ID_357	AO090001000008	
n/a					prot_ID_986	AO090001000007	
n/a					prot_ID_1583	AO090001000006	
n/a					prot_ID_1543	AO090001000003	
n/a					prot_ID_243	AO090001000002	
n/a					prot_ID_1686	AO090001000001	

AO090001000043 cluster

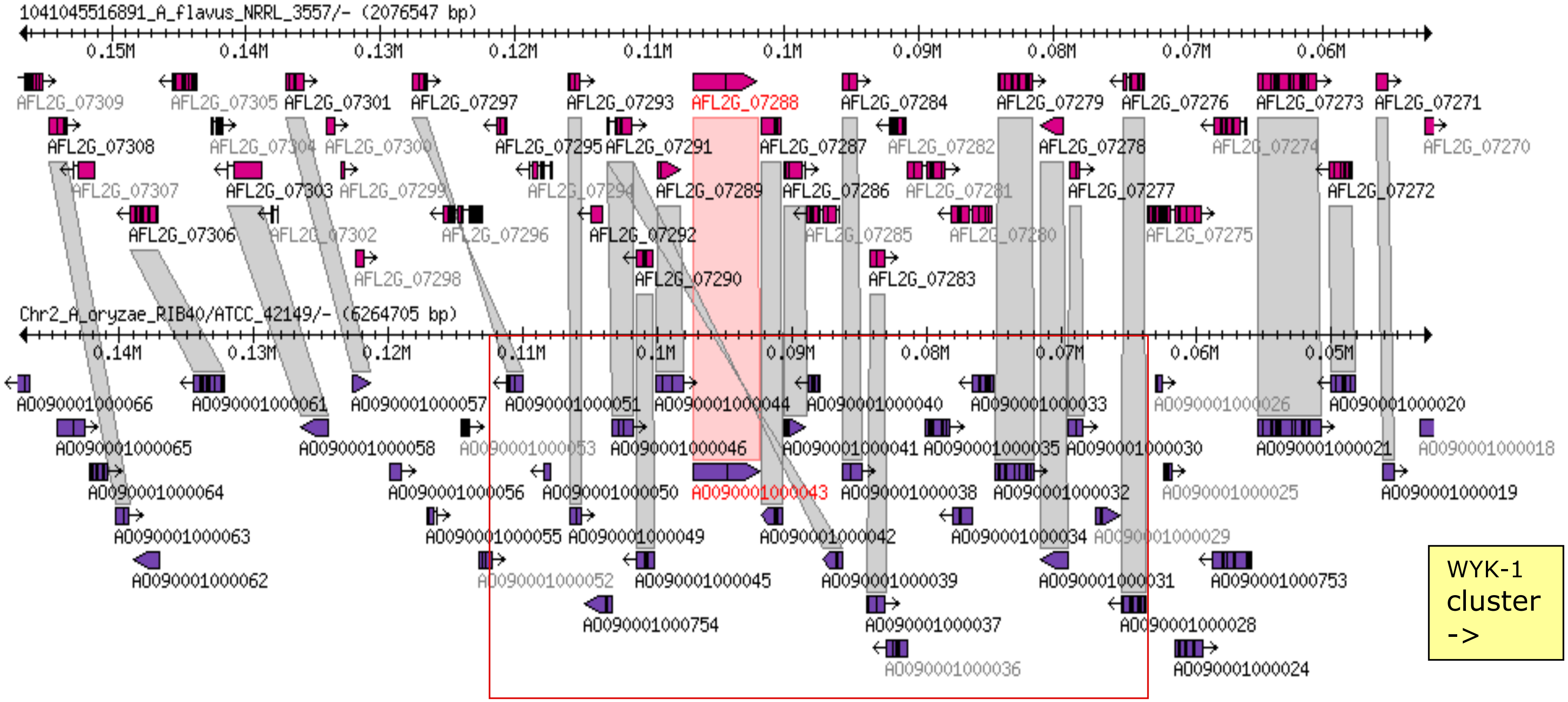

AO090001000043 cluster

Gene id (SMURF)	Gene positions	Gene distance	Gene Name	Annotated_gene_function	antiSMASH ID	AspGD ID	
n/a				Ortholog of A. flavus NRRL 3357 : AFL2T_02252, A. terreus NIH2624 : ATET_07085 and A. carbonarius ITEM 5010 : Acar5010_405538	prot_ID_384	AO090001000055	
n/a				Protein of unknown function	prot_ID_1452	AO090001000053	
n/a				Protein of unknown function	prot_ID_615	AO090001000052	
AO090001000051	-7	1728		Domain(s) with predicted nucleotide binding, oxidoreductase activity and role in oxidation-reduction process	prot_ID_1640	AO090001000051	ECS, FA
AO090001000050	-6	1381		Ortholog of A. niger CBS 513.88 : An05g01820, A. oryzae RIB40 : AO090023000143, A. brasiliensis : Aspbr1_0049118 and A. flavus NRRL 3357 : AFL2T_04033	prot_ID_794	AO090001000050	
AO090001000049	-5	798		Domain(s) with predicted nucleotide binding, oxidoreductase activity and role in oxidation-reduction process	prot_ID_1700	AO090001000049	
AO090001000754	-4	58		Ortholog of Aspergillus clavatus NRRL 1 : ACLA_066670	prot_ID_1972	AO090001000754	
AO090001000046	-3	263		Domain(s) with predicted O-methyltransferase activity	prot_ID_217	AO090001000046	
AO090001000045	-2	248		Domain(s) with predicted nucleotide binding, oxidoreductase activity	prot_ID_1280	AO090001000045	
AO090001000044	-1	805		Domain(s) with predicted nucleobase transmembrane transporter activity, role in nucleobase transport and membrane localization	prot_ID_218	AO090001000044	
**AO090001000043**	0	0		Non-ribosomal peptide synthetase	prot_ID_1283	AO090001000043	
AO090001000042	1	303		Domain(s) with predicted dipeptidase activity, dipeptidyl-peptidase activity, metalloexopeptidase activity and role in proteolysis	prot_ID_423	AO090001000042	
AO090001000041	2	226		Cytochrome P450 monooxygenase	prot_ID_661	AO090001000041	
AO090001000040	3	131		Ortholog(s) have glutathione transferase activity and role in gliotoxin biosynthetic process	prot_ID_1726	AO090001000040	
AO090001000039	4	195		Domain(s) with predicted O-methyltransferase activity, sequence-specific DNA binding transcription factor activity, role in regulation of transcription, DNA-dependent and intracellular localization	prot_ID_138	AO090001000039	
AO090001000038	5	171		Domain(s) with predicted oxidoreductase activity and role in oxidation-reduction process	prot_ID_1265	AO090001000038	
AO090001000037	6	964		Ortholog(s) have flavin adenine dinucleotide binding activity and role in cellular response to mycotoxin, gliotoxin biosynthetic process	prot_ID_291	AO090001000037	
AO090001000036	7	427		Protein of unknown function	prot_ID_1059	AO090001000036	
AO090001000035	8	1576		Cytochrome P450; upregulated by exposure to benomyl	prot_ID_209	AO090001000035	
AO090001000034	9	195		Ortholog of A. clavatus NRRL 1 : ACLA_066780	prot_ID_612	AO090001000034	

AO090001000033	10	155	Ortholog(s) have carbon-sulfur lyase activity and role in gliotoxin biosynthetic process	prot_ID_1797	AO090001000033	
AO090001000032	11	239	Domain(s) with predicted ATP binding, ATPase activity, coupled to transmembrane movement of substances activity, role in transmembrane transport and integral to membrane localization	prot_ID_389	AO090001000032	
AO090001000031	12	601	Domain(s) with predicted role in transmembrane transport and integral to membrane localization	n/a	AO090001000031	
AO090001000030	13	467	Ortholog(s) have role in gliotoxin biosynthetic process	n/a	AO090001000030	
AO090001000029	14	1305	Domain(s) with predicted sequence-specific DNA binding RNA polymerase II transcription factor activity, zinc ion binding activity, role in regulation of transcription, DNA-dependent and nucleus localization	n/a	AO090001000029	
AO090001000028	15	104	Cytochrome P450 monooxygenase	n/a	AO090001000028	FA
AO090001000026	16	778	Domain(s) with predicted electron carrier activity, heme binding, iron ion binding, oxidoreductase activity, acting on paired donors, with incorporation or reduction of molecular oxygen activity and role in oxidation-reduction process	n/a	AO090001000026	
AO090001000025	17	131	Domain(s) with predicted electron carrier activity, heme binding, iron ion binding, monooxygenase activity, oxidoreductase activity, acting on paired donors, with incorporation or reduction of molecular oxygen activity	n/a	AO090001000025	
AO090001000024	18	202	Domain(s) with predicted oxidoreductase activity and role in metabolic process	n/a	AO090001000024	
AO090001000753	19	1000	Domain(s) with predicted DNA binding, sequence-specific DNA binding RNA polymerase II transcription factor activity, zinc ion binding activity and role in regulation of transcription, DNA-dependent, transcription, DNA-dependent	n/a	AO090001000753	
AO090001000021	20	468	Domain(s) with predicted ATP binding, ATPase activity, coupled to transmembrane movement of substances activity, role in transmembrane transport and integral to membrane localization	n/a	AO090001000021	

AO090001000262 cluster

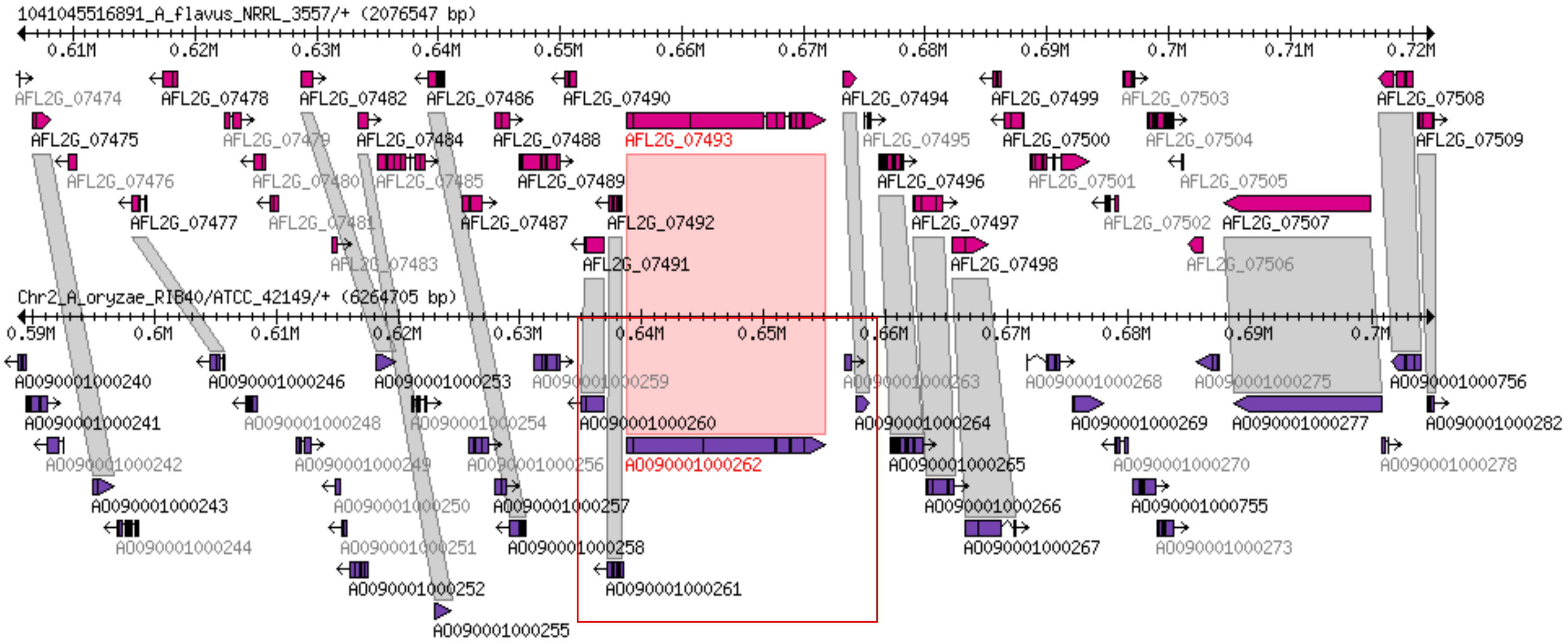

AO090001000262 cluster

Gene id (SMURF)	Gene positions	Gene distance	Gene Name	Annotated_gene_function	antiSMASH ID	AspGD ID	
n/a				Ortholog of A. niger CBS 513.88 : An16g04050, A. oryzae RIB40 : AO090011000252, A. brasiliensis : Aspbr1_0036278 and A. flavus NRRL 3357 : AFL2T_07482	prot_ID_1241	AO090001000253	
n/a				Protein of unknown function	prot_ID_395	AO090001000254	
n/a				Domain(s) with predicted nucleotide binding, oxidoreductase activity, transferase activity, transferring acyl groups other than amino-acyl groups, zinc ion binding activity and role in oxidation-reduction process	prot_ID_1520	AO090001000255	
n/a				Domain(s) with predicted DNA binding, zinc ion binding activity, role in transcription, DNA-dependent and nucleus localization	prot_ID_524	AO090001000256	
n/a				Domain(s) with predicted nucleotide binding, oxidoreductase activity and role in oxidation-reduction process	prot_ID_1773	AO090001000257	
n/a				Domain(s) with predicted catalytic activity and role in cellular amino acid catabolic process	prot_ID_934	AO090001000258	
n/a				Domain(s) with predicted beta-galactosidase activity, cation binding activity and role in carbohydrate metabolic process	prot_ID_449	AO090001000259	
n/a			CYP5099A1	Cytochrome P450 monooxygenase	prot_ID_1065	AO090001000260	ECS
n/a				Domain(s) with predicted oxidoreductase activity, acting on paired donors, with incorporation or reduction of molecular oxygen, 2-oxoglutarate as one donor, and incorporation of one atom each of oxygen into both donors activity	prot_ID_1622	AO090001000261	
n/a				Non-ribosomal peptide synthetase	prot_ID_565	AO090001000262	
n/a				Protein of unknown function	prot_ID_397	AO090001000263	
n/a				Domain(s) with predicted nucleotide binding, oxidoreductase activity, zinc ion binding activity and role in oxidation-reduction process	prot_ID_1195	AO090001000264	FA, ECS
n/a				Domain(s) with predicted role in transmembrane transport and integral to membrane localization	prot_ID_145	AO090001000265	
n/a				Glycoside hydrolase family 3	prot_ID_1165	AO090001000266	
n/a				Protein of unknown function; predicted secretion signal peptide; positively regulated by XlnR; 2 putative XlnR binding sites in the promoter	prot_ID_537	AO090001000267	
n/a				Domain(s) with predicted role in isoprenoid biosynthetic process	prot_ID_1915	AO090001000268	

AO090001000277 cluster

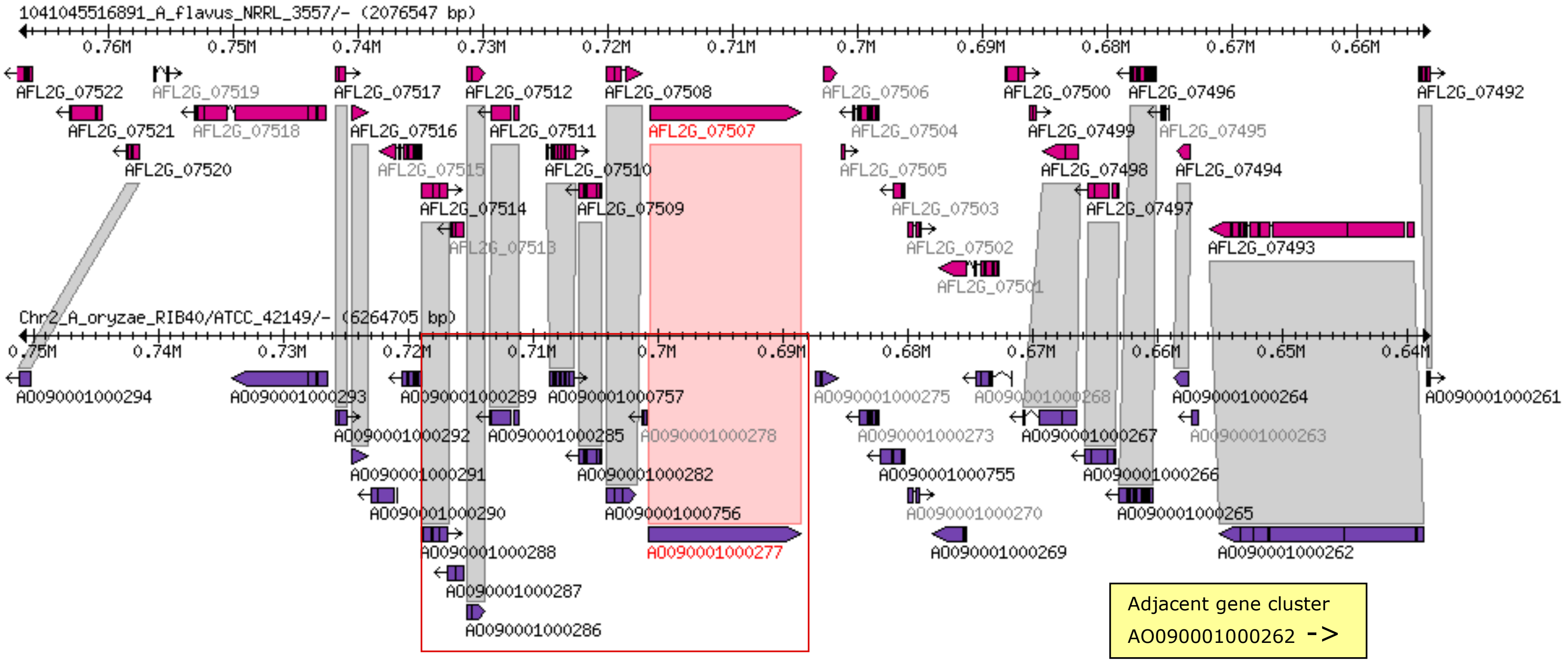

AO090001000277 cluster

Gene id (SMURF)	Gene positions	Gene distance	Gene Name	Annotated_gene_function	antiSMASH ID	AspGD ID	
AO090001000293	-13	768		Polyketide synthase	n/a	AO090001000293	
AO090001000292	-12	515		Protein of unknown function; expression increased in MAT1-1 strain compared to MAT1-2 strain	n/a	AO090001000292	
AO090001000291	-11	971		Ortholog of A. nidulans FGSC A4 : AN2637, A. fumigatus Af293 : Afu2g13650, A. oryzae RIB40 : AO090005001139, AO090026000243 and A. niger ATCC 1015 : 42301-mRNA, 51721-mRNA	n/a	AO090001000291	
AO090001000290	-10	527		Domain(s) with predicted oxidoreductase activity and role in metabolic process		AO090001000290	ECS
AO090001000289	-9	77		Domain(s) with predicted O-methyltransferase activity	prot_ID_606	AO090001000289	
AO090001000288	-8	212		Cytochrome P450 monooxygenase	prot_ID_1451	AO090001000288	
AO090001000287	-7	360		Domain(s) with predicted nucleotide binding, oxidoreductase activity, transferase activity, transferring acyl groups other than amino-acyl groups, zinc ion binding activity and role in oxidation-reduction process	prot_ID_1305	AO090001000287	
AO090001000286	-6	716		Domain(s) with predicted nucleotide binding, oxidoreductase activity and role in metabolic process	prot_ID_4	AO090001000286	
AO090001000285	-5	2506		Domain(s) with predicted DNA binding, zinc ion binding activity, role in transcription, DNA-dependent and nucleus localization	prot_ID_1025	AO090001000285	
AO090001000757	-4	618		Cytochrome P450 monooxygenase	prot_ID_1944	AO090001000757	
AO090001000282	-3	634		Domain(s) with predicted role in transmembrane transport and integral to membrane localization	prot_ID_686	AO090001000282	
AO090001000756	-2	330		Domain(s) with predicted DNA binding, sequence-specific DNA binding RNA polymerase II transcription factor activity, zinc ion binding activity and role in regulation of transcription, DNA-dependent, transcription, DNA-dependent	prot_ID_1965	AO090001000756	
AO090001000278	-1	285		Protein of unknown function	prot_ID_1643	AO090001000278	
**AO090001000277**	0	0		Polyketide synthase	prot_ID_264	AO090001000277	ECS
AO090001000275	1	1307		Domain(s) with predicted DNA binding, zinc ion binding activity, role in transcription, DNA-dependent and nucleus localization	prot_ID_598	AO090001000275	
AO090001000273	2	1823		Ortholog(s) have role in protein targeting to membrane and cytoplasm localization	prot_ID_1134	AO090001000273	
AO090001000755	3	480		Domain(s) with predicted sequence-specific DNA binding RNA polymerase II transcription factor activity, zinc ion binding activity, role in regulation of transcription, DNA-dependent and nucleus localization	prot_ID_1954	AO090001000755	
AO090001000270	4	429		Domain(s) with predicted catalytic activity and role in metabolic process	prot_ID_47	AO090001000270	

AO090001000269	5	1099	Ortholog of A. nidulans FGSC A4 : AN5315, A. niger CBS 513.88 : An01g01450, A. brasiliensis : Aspbr1_0053207, A. niger ATCC 1015 : 36402-mRNA and N. fischeri NRRL 181 : NFIA_044920	prot_ID_1352	AO090001000269
AO090001000268	6	890	Domain(s) with predicted role in isoprenoid biosynthetic process	prot_ID_1915	AO090001000268
AO090001000267	7	932	Protein of unknown function; predicted secretion signal peptide; positively regulated by XlnR; 2 putative XlnR binding sites in the promoter	prot_ID_537	AO090001000267
AO090001000266	8	693	Glycoside hydrolase family 3	n/a	AO090001000266
AO090001000265	9	854	Domain(s) with predicted role in transmembrane transport and integral to membrane localization	n/a	AO090001000265
AO090001000264	10	1753	Domain(s) with predicted nucleotide binding, oxidoreductase activity, zinc ion binding activity and role in oxidation-reduction process	n/a	AO090001000264
AO090001000263	11	412		n/a	AO090001000263
AO090001000262	12	1596	Non-ribosomal peptide synthetase	n/a	AO090001000262
AO090001000261	13	351	Domain(s) with predicted oxidoreductase activity, acting on paired donors, with incorporation or reduction of molecular oxygen, 2-oxoglutarate as one donor, and incorporation of one atom each of oxygen into both donors activity	n/a	AO090001000261
AO090001000260	14	349	Cytochrome P450 monooxygenase	n/a	AO090001000260
AO090001000259	15	2074	Domain(s) with predicted beta-galactosidase activity, cation binding activity and role in carbohydrate metabolic process	n/a	AO090001000259
AO090001000258	16	605	Domain(s) with predicted catalytic activity and role in cellular amino acid catabolic process	n/a	AO090001000258
AO090001000257	17	307	Domain(s) with predicted nucleotide binding, oxidoreductase activity and role in oxidation-reduction process	n/a	AO090001000257
AO090001000256	18	634	Domain(s) with predicted DNA binding, zinc ion binding activity, role in transcription, DNA-dependent and nucleus localization	n/a	AO090001000256

AO090001000402 cluster

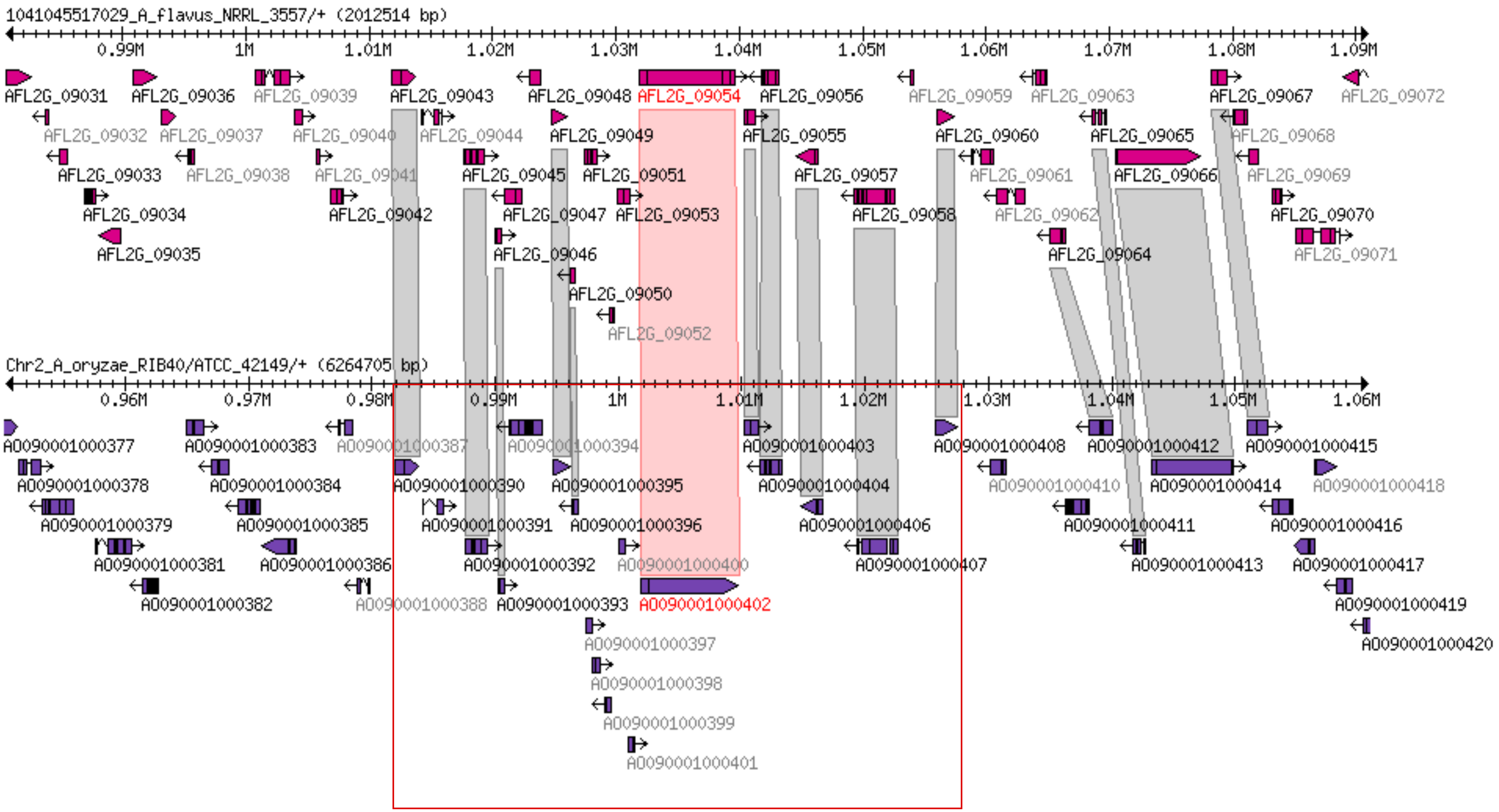

AO090001000402 cluster

Gene id (SMURF)	Gene positions	Gene distance	Gene Name	Annotated_gene_function	antiSMASH ID	AspGD ID	
n/a				Ortholog of *A. flavus NRRL 3357* : AFL2T_09060, AFL2T_09737, *A. versicolor* : Aspve1_0080922 and *A. terreus NIH2624* : ATET_05637	prot_ID_1162	AO090001000408	ECS
n/a				Domain(s) with predicted ADP binding, ATP binding activity and role in apoptotic process, defense response	prot_ID_1827	AO090001000407	
n/a				Ortholog of *A. nidulans FGSC A4* : AN7214, *A. oryzae RIB40* : AO090120000014, AO090103000238, *A. niger ATCC 1015* : 206373-mRNA and *A. versicolor* : Aspve1_0038859	prot_ID_767	AO090001000406	
AO090001000404	-2	370		Domain(s) with predicted role in transmembrane transport and integral to membrane localization	prot_ID_487	AO090001000404	
AO090001000403	-1	1102		Ortholog(s) have cytoplasm, nucleus localization	prot_ID_1401	AO090001000403	
**AO090001000402**	0	0		Polyketide synthase	prot_ID_341	AO090001000402	
AO090001000401	1	578		Protein of unnown function	prot_ID_947	AO090001000401	
AO090001000400	2	174		Domain(s) with predicted nucleotide binding activity	prot_ID_1701	AO090001000400	
AO090001000399	3	694		Domain(s) with predicted nucleotide binding activity	prot_ID_197	AO090001000399	
AO090001000398	4	342		Domain(s) with predicted oxidoreductase activity, acting on paired donors, with incorporation or reduction of molecular oxygen, 2-oxoglutarate as one donor, and incorporation of one atom each of oxygen into both donors activity	prot_ID_1318	AO090001000398	
AO090001000397	5	65			prot_ID_518	AO090001000397	
AO090001000396	6	714		Ortholog of A. nidulans FGSC A4 : AN0527 and A. flavus NRRL 3357 : AFL2T_09050	prot_ID_1579	AO090001000396	
AO090001000395	7	213		Domain(s) with predicted role in transmembrane transport and integral to membrane localization	prot_ID_631	AO090001000395	
AO090001000394	8	877		Domain(s) with predicted monooxygenase activity and role in oxidation-reduction process	prot_ID_1736	AO090001000394	
AO090001000393	9	414		Ortholog of A. nidulans FGSC A4 : AN8308 and A. flavus NRRL 3357 : AFL2T_09046	prot_ID_950	AO090001000393	
AO090001000392	10	766		Domain(s) with predicted DNA binding, sequence-specific DNA binding RNA polymerase II transcription factor activity, zinc ion binding activity and role in regulation of transcription, DNA-dependent, transcription, DNA-dependent	prot_ID_32	AO090001000392	
AO090001000391	11	1847		Ortholog of A. nidulans FGSC A4 : AN7850, A. fumigatus Af293 : Afu4g14030, N. fischeri NRRL 181 : NFIA_102290, A. terreus NIH2624 : ATET_02187 and A. fumigatus A1163 : AFUB_070950	prot_ID_1872	AO090001000391	

AO090001000390	12	203	Putative carnitine transport protein; predominantly expressed in the basal region of hyphae	prot_ID_980	AO090001000390	ECS

AO090001000293 cluster

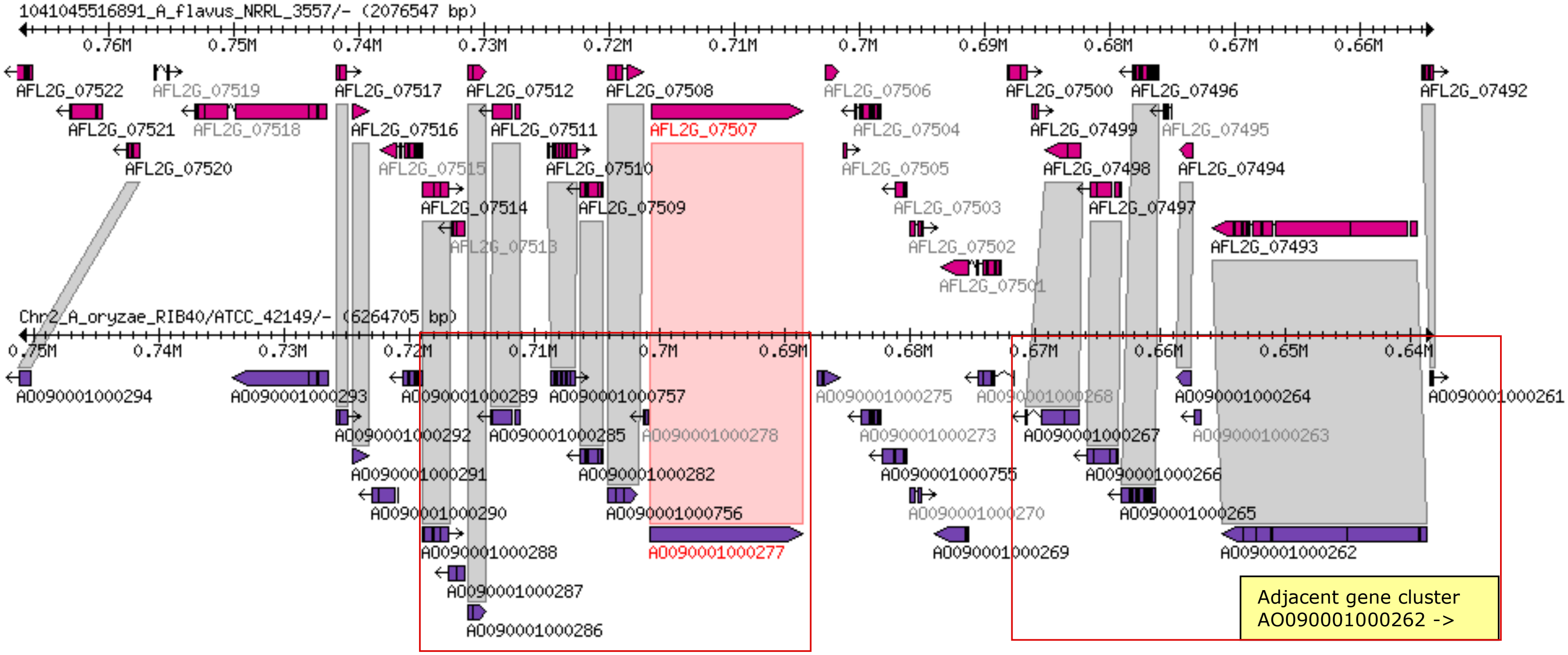

AO090001000293 cluster

Gene id (SMURF)	Gene positions	Gene distance	Gene Name	Annotated_gene_function	antiSMASH ID	AspGD ID	
n/a				Putative polyketide synthase (PKS) - nonribosomal peptide synthase (NRPS) hybrid	n/a	AO090001000277	ECS
n/a					n/a	AO090001000756	
n/a				Domain(s) with predicted role in transmembrane transport and integral to membrane localization	n/a	AO090001000282	
n/a			CYP5286A1	Cytochrome P450 monooxygenase	prot_ID_1944	AO090001000757	
n/a				Domain(s) with predicted DNA binding, zinc ion binding activity, role in transcription, DNA-dependent and nucleus localization	prot_ID_1025	AO090001000285	
n/a				Domain(s) with predicted nucleotide binding, oxidoreductase activity and role in metabolic process	prot_ID_4	AO090001000286	
n/a				Domain(s) with predicted nucleotide binding, oxidoreductase activity, transferase activity, transferring acyl groups other than amino-acyl groups, zinc ion binding activity and role in oxidation-reduction process	prot_ID_1305	AO090001000287	
n/a			CYP655B1	Cytochrome P450 monooxygenase	prot_ID_1451	AO090001000288	ECS
n/a				Domain(s) with predicted O-methyltransferase activity	prot_ID_606	AO090001000289	
n/a				Domain(s) with predicted oxidoreductase activity and role in metabolic process	prot_ID_1243	AO090001000290	
n/a				Ortholog of A. nidulans FGSC A4 : AN2637, A. fumigatus Af293 : Afu2g13650, A. oryzae RIB40 : AO090005001139, AO090026000243 and A. niger ATCC 1015 : 42301-mRNA, 51721-mRNA	prot_ID_396	AO090001000291	
n/a				Protein of unknown function; expression increased in MAT1-1 strain compared to MAT1-2 strain	prot_ID_1512	AO090001000292	
n/a				Polyketide synthase	prot_ID_674	AO090001000293	
n/a				Domain(s) with predicted extracellular region localization	prot_ID_1681	AO090001000294	
n/a				Ortholog(s) have role in acetate catabolic process, alcohol metabolic process, carnitine metabolic process and mitochondrion localization	prot_ID_939	AO090001000295	

AO090001000506 cluster

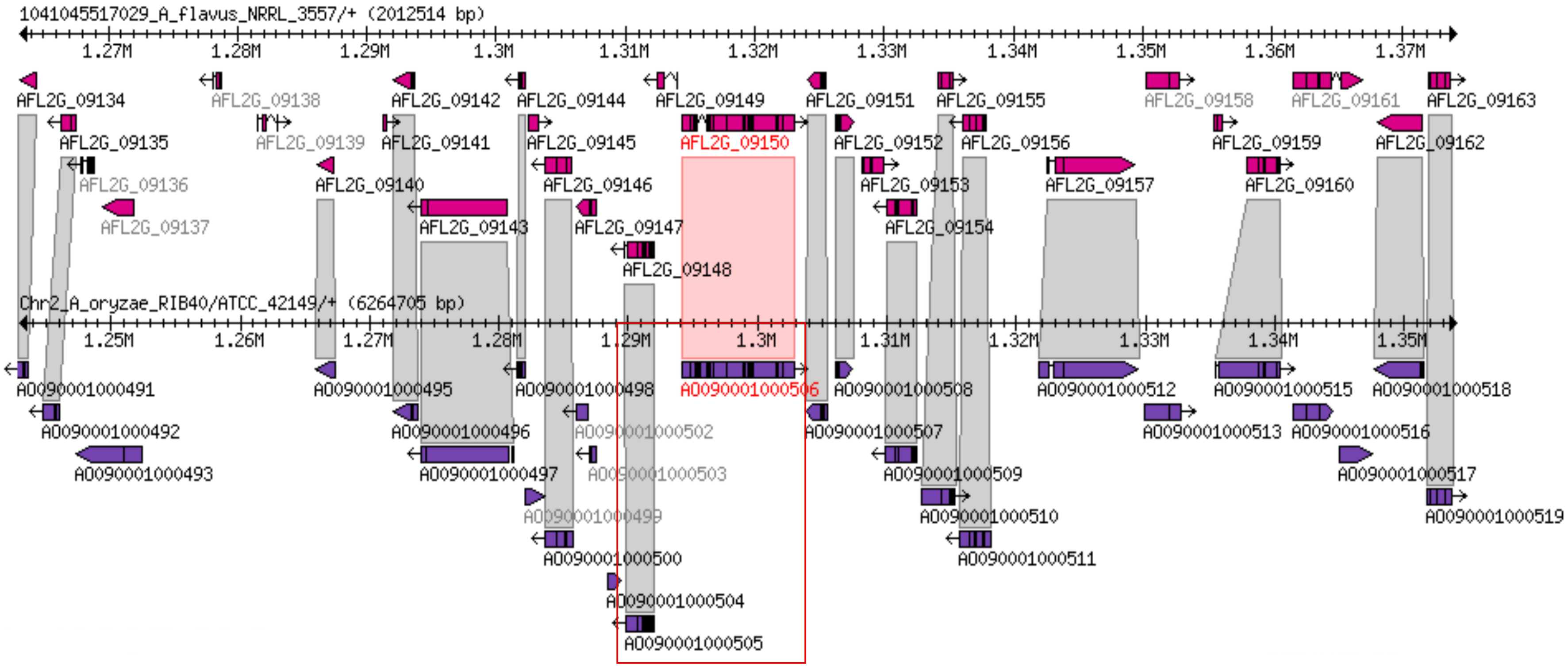

AO090001000506 cluster

Gene id (SMURF)	Gene positions	Gene distance	Gene Name	Annotated_gene_function	antiSMASH ID	AspGD ID	
n/a			cyaA	Adenylate cyclase	prot_ID_296	AO090001000512	
n/a				Domain(s) with predicted hydrolase activity, hydrolyzing O-glycosyl compounds activity, role in carbohydrate metabolic process, cellular cell wall organization and cell wall localization	prot_ID_1366	AO090001000511	
n/a				Domain(s) with predicted transporter activity, role in transmembrane transport, water transport and integral to membrane localization	prot_ID_27	AO090001000510	
n/a				Ortholog(s) have glycerol kinase activity and mitochondrion localization	prot_ID_87	AO090001000509	
n/a				Domain(s) with predicted GTP binding, GTPase activity	prot_ID_1353	AO090001000508	
n/a				Domain(s) with predicted GTP binding, nucleoside-triphosphatase activity	prot_ID_330	AO090001000507	
**AO090001000506**	0	0		Polyketide synthase	prot_ID_1317	AO090001000506	FA
AO090001000505	1	2254		Domain(s) with predicted role in transmembrane transport and integral to membrane localization	prot_ID_444	AO090001000505	ECS
n/a				Domain(s) with predicted phosphoglycolate phosphatase activity and role in metabolic process	prot_ID_1034	AO090001000504	
n/a				Protein of unknown function	prot_ID_180	AO090001000503	
n/a				Protein of unknown function	prot_ID_1724	AO090001000502	
n/a				Ortholog(s) have oxygen-dependent protoporphyrinogen oxidase activity, role in heme biosynthetic process and mitochondrial inner membrane localization	prot_ID_1438	AO090001000500	
n/a				Protein of unknown function	prot_ID_789	AO090001000499	
n/a				Domain(s) with predicted ubiquinol-cytochrome-c reductase activity, role in mitochondrial electron transport, ubiquinol to cytochrome c and mitochondrial envelope localization	prot_ID_266	AO090001000498	
n/a				Ortholog(s) have ubiquitin-protein ligase activity	prot_ID_701	AO090001000497	
n/a				Domain(s) with predicted nucleus localization	n/a	AO090001000496	
n/a				Domain(s) with predicted catalytic activity, cation binding activity and role in carbohydrate metabolic process	n/a	AO090001000495	

AO090001000516 cluster

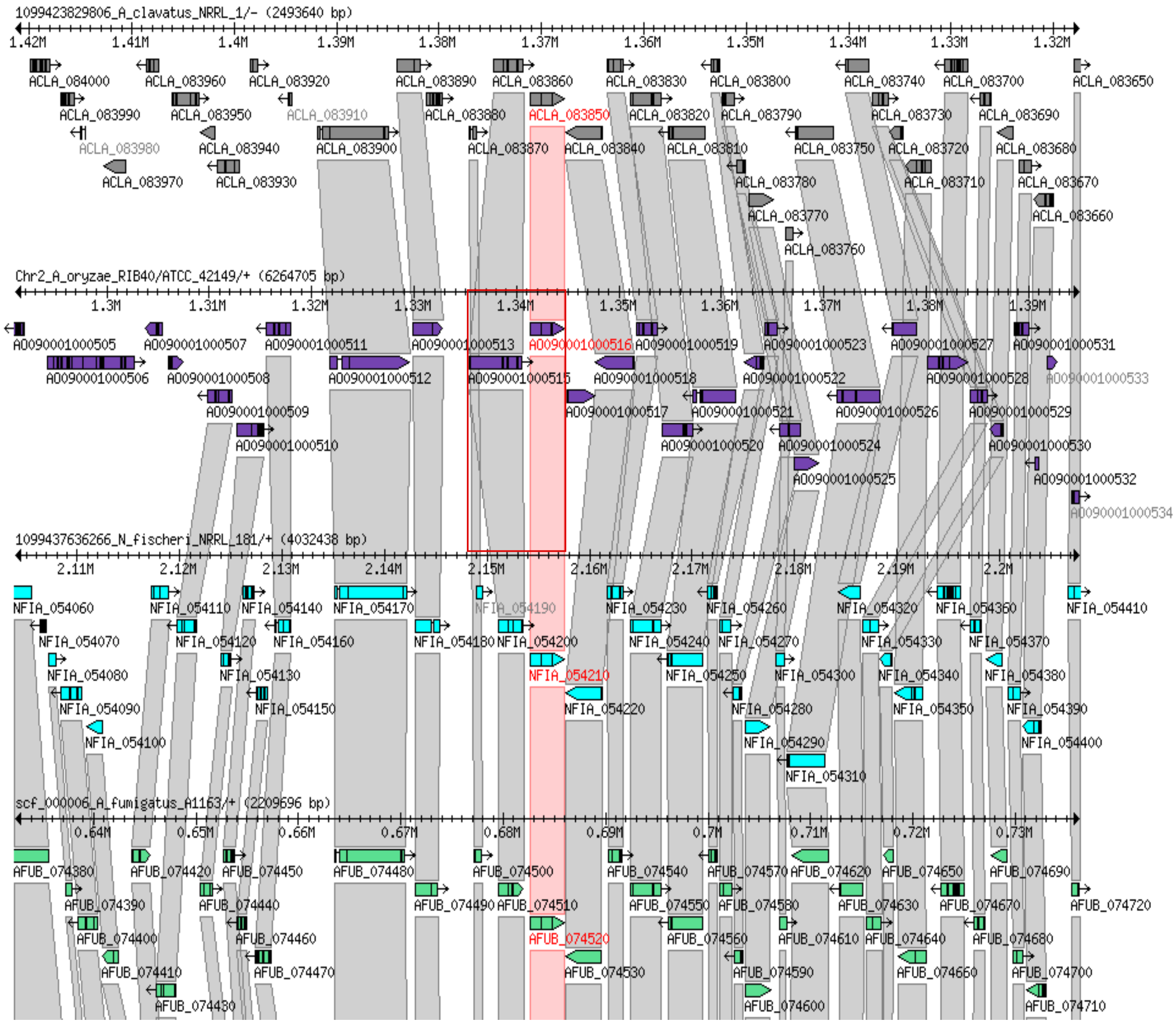

AO090001000516 cluster

Gene id (SMURF)	Gene positions	Gene distance	Gene Name	Annotated_gene_function	antiSMASH ID	AspGD ID	
n/a				golgi reassembly stacking protein GRASP65	prot_ID_319	AO090001000523	
n/a				Ortholog(s) have RNA methyltransferase activity, role in 7-methylguanosine cap hypermethylation, meiosis, nucleologenesis, regulation of telomere maintenance via telomerase and nucleolus localization	prot_ID_1602	AO090001000522	
n/a				Transcription initiation factor TFIID, subunit TAF1	prot_ID_506	AO090001000521	
n/a				Serine/threonine kinase	prot_ID_1815	AO090001000520	
n/a				Ortholog(s) have macrolide binding, peptidyl-prolyl cis-trans isomerase activity	prot_ID_307	AO090001000519	
n/a				Translesion DNA polymerase	prot_ID_1374	AO090001000518	
n/a				Ortholog of A. versicolor : Aspve1_0088708 and A. carbonarius ITEM 5010 : Acar5010_131530	prot_ID_31	AO090001000517	
**AO090001000516**	0	0		Non-ribosomal peptide synthetase	prot_ID_1073	AO090001000516	ECS
AO090001000515	1	1044		Domain(s) with predicted DNA binding, sequence-specific DNA binding RNA polymerase II transcription factor activity, zinc ion binding activity and role in regulation of transcription, DNA-dependent, transcription, DNA-dependent	prot_ID_1908	AO090001000515	ECS
n/a				Ortholog(s) have role in maintenance of rDNA, mitotic sister chromatid segregation and nuclear envelope localization	prot_ID_1628	AO090001000513	
n/a			cyaA	Serine/threonine phosphatase 2C	prot_ID_296	AO090001000512	

AO090001000768 cluster

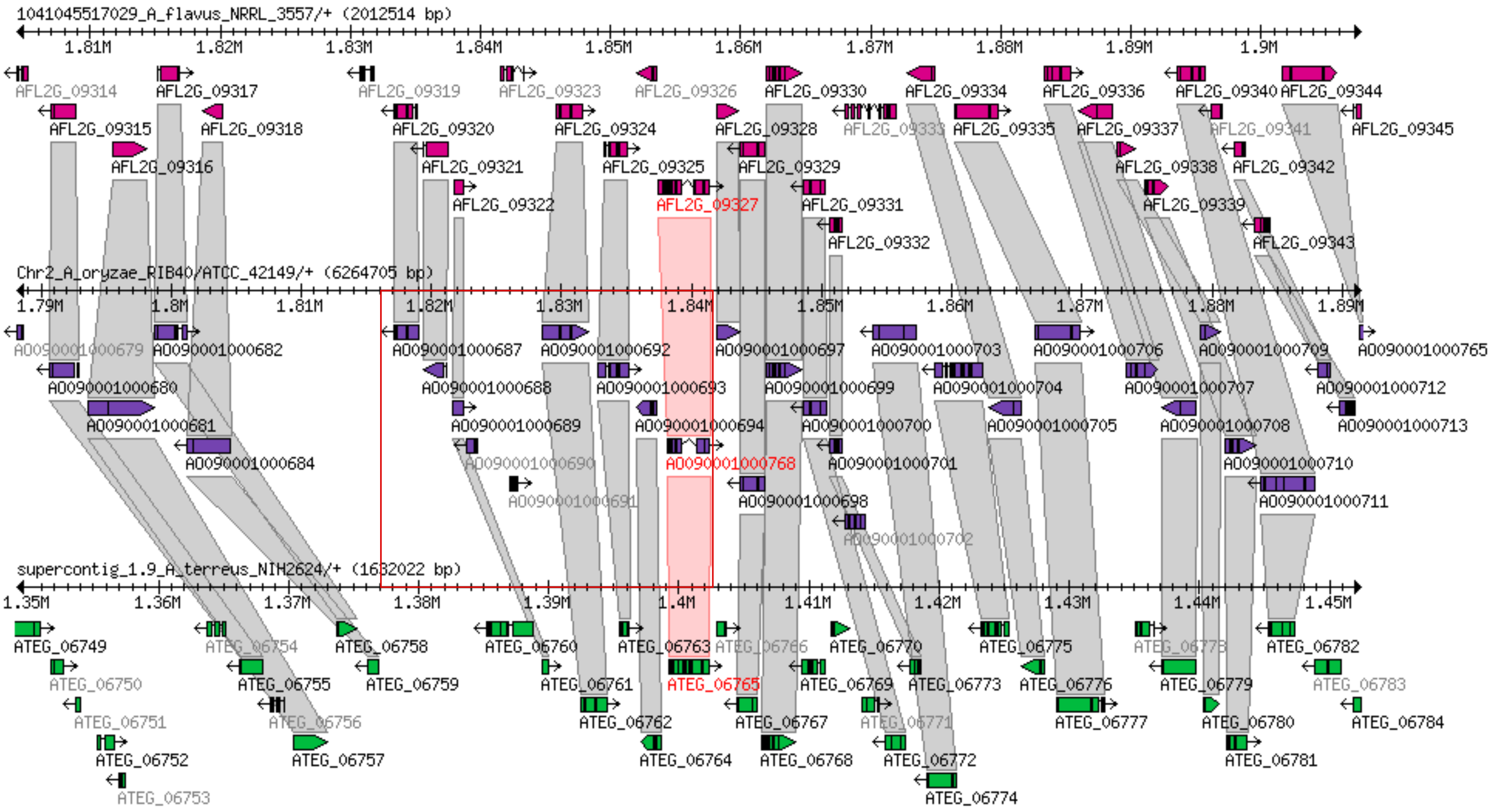

AO090001000768 cluster

Gene id (SMURF)	Gene positions	Gene distance	Gene Name	Annotated_gene_function	antiSMASH ID	AspGD ID	
AO090001000704	-8	2565		Domain(s) with predicted transporter activity, role in transmembrane transport and integral to membrane localization	n/a	AO090001000704	
AO090001000703	-7	1104		Ortholog(s) have RNA polymerase II regulatory region sequence-specific DNA binding activity, role in carbon utilization and cytoplasm, nucleus localization	n/a	AO090001000703	
AO090001000702	-6	276		Domain(s) with predicted catalytic activity and role in metabolic process	n/a	AO090001000702	
AO090001000701	-5	467		Ortholog(s) have riboflavin kinase activity, role in FMN biosynthetic process and mitochondrial inner membrane localization	n/a	AO090001000701	
AO090001000700	-4	118		Ortholog of A. nidulans FGSC A4 : AN7470, A. fumigatus Af293 : Afu2g05810, A. niger CBS 513.88 : An02g14540, A. niger ATCC 1015 : 123724-mRNA and A. versicolor : Aspve1_0135868	n/a	AO090001000700	
AO090001000699	-3	321		Domain(s) with predicted hydrolase activity, acting on glycosyl bonds, phosphotransferase activity, alcohol group as acceptor activity	n/a	AO090001000699	
AO090001000698	-2	412		Ortholog(s) have dolichyl-diphosphooligosaccharide-protein glycotransferase activity, role in protein N-linked glycosylation via asparagine and oligosaccharyltransferase complex localization	n/a	AO090001000698	
AO090001000697	-1	679		Ortholog(s) have mRNA guanylyltransferase activity, role in 7-methylguanosine mRNA capping, positive regulation of transcription from RNA polymerase II promoter and mRNA cap methyltransferase complex localization	n/a	AO090001000697	
**AO090001000768**	0	0		Domain(s) with predicted catalytic activity and role in biosynthetic process	n/a	AO090001000768	ECS
AO090001000694	1	1000		Domain(s) with predicted hydrolase activity, acting on carbon-nitrogen (but not peptide) bonds, in linear amidines, metal ion binding activity	n/a	AO090001000694	
AO090001000693	2	1399		Ortholog(s) have GTPase activity	n/a	AO090001000693	
AO090001000692	3	1840		Domain(s) with predicted role in transmembrane transport and integral to membrane localization	n/a	AO090001000692	
n/a				Protein of unknown function	n/a	AO090001000691	
n/a				Domain(s) with predicted nucleotide binding, oxidoreductase activity, transferase activity, transferring acyl groups other than amino-acyl groups, zinc ion binding activity and role in oxidation-reduction process	n/a	AO090001000690	
n/a				Ortholog of A. nidulans FGSC A4 : AN7484, A. fumigatus Af293 : Afu2g05720, A. niger CBS 513.88 : An02g14170, A. niger ATCC 1015 : 47182-mRNA and A. versicolor : Aspve1_0085916	n/a	AO090001000689	
n/a				Ortholog of A. nidulans FGSC A4 : AN4092, AN7483, A. fumigatus Af293 : Afu2g05710, A. niger ATCC 1015 : 207477-mRNA and A. versicolor : Aspve1_0043503 (2)	n/a	AO090001000688	

n/a	Ortholog of A. fumigatus Af293 : Afu2g05700, N. fischeri NRRL 181 : NFIA_082420, A. flavus NRRL 3357 : AFL2T_09320, A. clavatus NRRL 1 : ACLA_068640 and A. acidus : Aspfo1_0474562	n/a	AO090001000687	ECS

AO090003000945 cluster

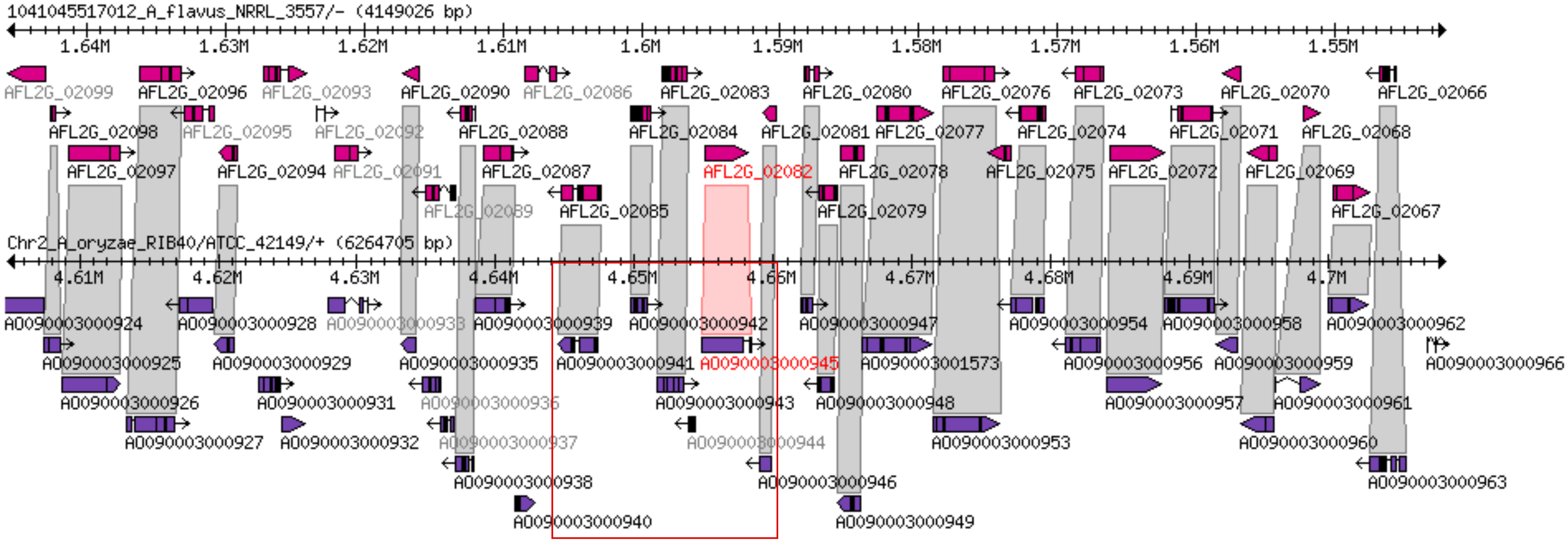

AO090003000945 cluster

Gene id (SMURF)	Gene positions	Gene distance	Gene Name	Annotated_gene_function	antiSMASH ID	AspGD ID	
AO090003000954	-7	2184		Cytochrome P450 monooxygenase	prot_ID_776	AO090003000954	
AO090003000953	-6	617		Ortholog(s) have GTP binding, GTPase activity, glutathione binding, ribosome binding, translation initiation factor activity, role in regulation of translational initiation and cytosolic small ribosomal subunit, mitochondrion localization	prot_ID_1882	AO090003000953	
AO090003001573	-5	1264		Ortholog(s) have role in response to temperature stimulus	prot_ID_1952	AO090003001573	
AO090003000949	-4	334		Ortholog(s) have Rab GTPase activator activity, role in vesicle-mediated transport and intracellular localization	prot_ID_1006	AO090003000949	
AO090003000948	-3	378		Ortholog(s) have Rab GTPase binding activity and Golgi apparatus localization	prot_ID_229	AO090003000948	
AO090003000947	-2	2147		Ortholog(s) have role in protein ubiquitination, ribosome biogenesis and extracellular region, membrane, mitochondrion localization	prot_ID_1220	AO090003000947	
AO090003000946	-1	700		Short chain-type dehydrogenase	prot_ID_1537	AO090003000946	ECS
**AO090003000945**	0	0		Non-ribosomal peptide synthetase	prot_ID_706	AO090003000945	
n/a				Protein of unknown function	prot_ID_1597	AO090003000944	
n/a				Domain(s) with predicted CoA-transferase activity and role in ketone body catabolic process	prot_ID_895	AO090003000943	
n/a				Ortholog of A. nidulans FGSC A4 : AN8945, A. fumigatus Af293 : Afu8g01930, A. oryzae RIB40 : AO090001000531, AO090010000650, A. niger ATCC 1015 : 204025-mRNA and A. versicolor : Aspve1_0057483	prot_ID_1596	AO090003000942	
n/a				Domain(s) with predicted DNA binding, zinc ion binding activity, role in transcription, DNA-dependent and nucleus localization	prot_ID_511	AO090003000941	ECS
n/a				Domain(s) with predicted oxidoreductase activity and role in oxidation-reduction process	prot_ID_1816	AO090003000940	
n/a				Ortholog(s) have role in Golgi to vacuole transport, actin filament-based process, retrograde transport, endosome to Golgi and GARP complex localization	prot_ID_1476	AO090003000939	
n/a			eif-5A	Translation initiation factor 5A; predominantly expressed in the hyphal tip region	prot_ID_1888	AO090003000938	
n/a				Protein of unknown function	prot_ID_813	AO090003000937	
n/a				Protein of unknown function	prot_ID_1689	AO090003000936	
n/a				Ortholog of A. fumigatus Af293 : Afu1g04130, N. fischeri NRRL 181 : NFIA_020600 and A. flavus NRRL 3357 : AFL2T_02090	n/a	AO090003000935	

AO090003001097 cluster

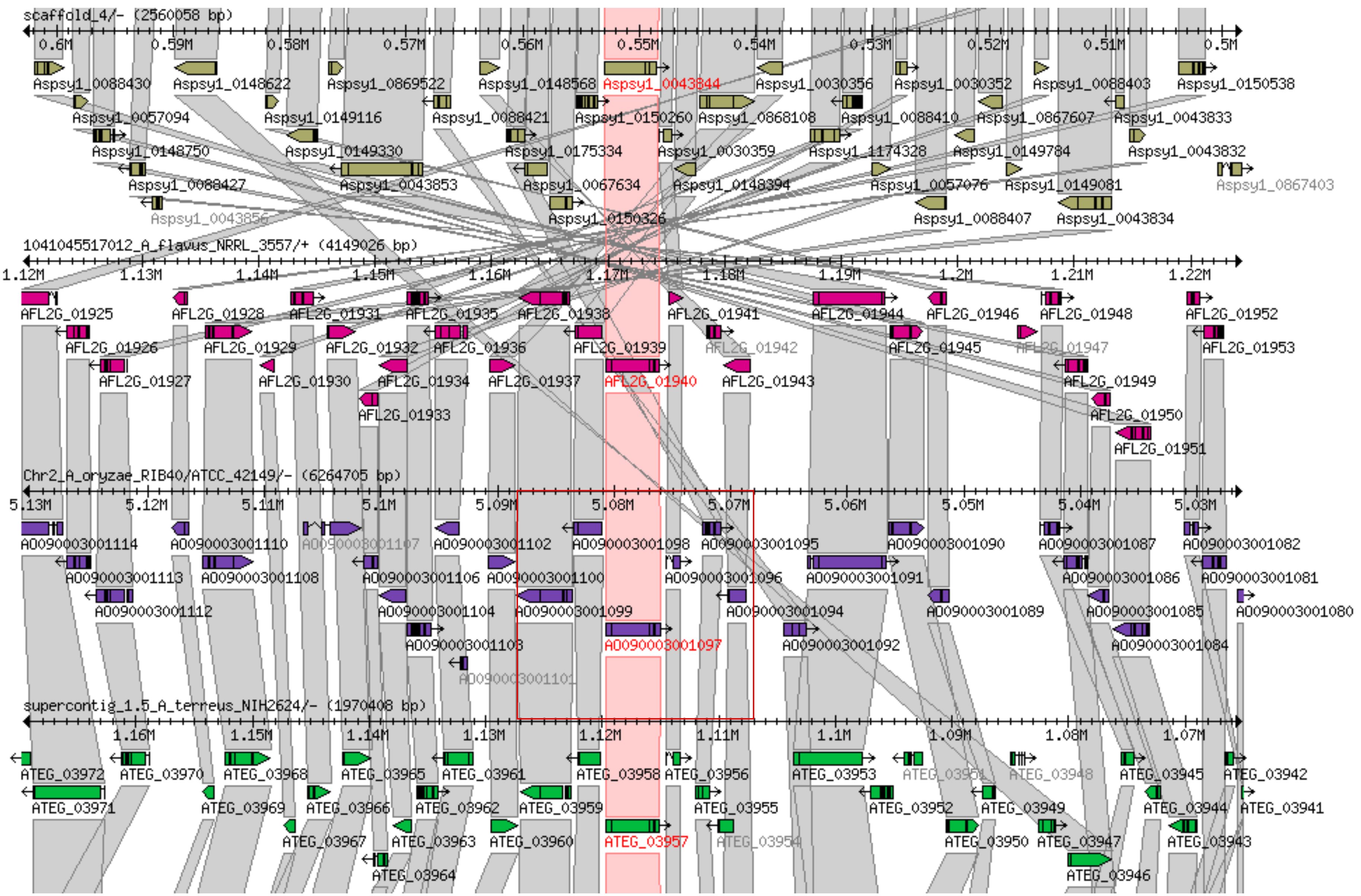

AO090003001097 cluster

Gene id (SMURF)	Gene positions	Gene distance	Gene Name	Annotated_gene_function	antiSMASH ID	AspGD ID	
n/a				Ortholog(s) have proton-transporting ATPase activity, rotational mechanism activity and role in polyphosphate metabolic process, protein complex assembly, proton transport, vacuolar acidification	prot_ID_1229	AO090003001090	
n/a				Ortholog(s) have peptidase activator activity, proteasome binding activity, role in proteasome assembly, regulation of proteasomal protein catabolic process and nucleus, proteasome core complex localization	prot_ID_394	AO090003001091	
n/a				Ortholog(s) have dolichyl-phosphate-mannose-glycolipid alpha-mannosyltransferase activity, role in GPI anchor biosynthetic process and integral to endoplasmic reticulum membrane, mannosyltransferase complex, mitochondrion localization	prot_ID_972	AO090003001092	
n/a				Ortholog of A. nidulans FGSC A4 : AN9473, A. fumigatus Af293 : Afu4g11270, A. niger CBS 513.88 : An04g05360, A. niger ATCC 1015 : 55020-mRNA and A. versicolor : Aspve1_0694359	prot_ID_927	AO090003001094	ECS
n/a				Domain(s) with predicted oxidoreductase activity and role in oxidation-reduction process	prot_ID_1435	AO090003001095	
n/a				Carbonic anhydrase; predominantly expressed in the basal region of hyphae	prot_ID_678	AO090003001096	
n/a				Non-ribosomal peptide synthetase/alpha-aminoadipate reductase	prot_ID_328	AO090003001097	
n/a				Ortholog of A. nidulans FGSC A4 : AN5612, A. fumigatus Af293 : Afu4g11230, A. niger CBS 513.88 : An04g05430, A. niger ATCC 1015 : 51205-mRNA and A. versicolor : Aspve1_0131376	prot_ID_98	AO090003001098	
n/a				Ortholog(s) have xanthine dehydrogenase activity and role in purine nucleobase catabolic process	prot_ID_1149	AO090003001099	ECS
n/a				Ortholog(s) have role in plasma membrane fusion involved in cytogamy, response to pheromone and mating projection tip localization	prot_ID_791	AO090003001100	
n/a				Protein of unknown function	prot_ID_55	AO090003001101	
n/a				Ortholog of A. nidulans FGSC A4 : AN5615, A. fumigatus Af293 : Afu4g11200, A. niger CBS 513.88 : An04g05480, A. niger ATCC 1015 : 45401-mRNA and Aspergillus versicolor : Aspve1_0084260	prot_ID_1913	AO090003001102	
n/a				Ortholog(s) have kynurenine-oxoglutarate transaminase activity, role in kynurenic acid biosynthetic process and mitochondrion localization	prot_ID_792	AO090003001103	
n/a				Ortholog(s) have enzyme activator activity, single-stranded DNA-dependent ATPase activity, role in DNA replication, Okazaki fragment processing, DNA strand renaturation, regulation of DNA repair and cytoplasm, nucleus localization	prot_ID_1572	AO090003001104	

n/a	Ortholog(s) have structural constituent of cytoskeleton activity and role in microtubule nucleation, proteasomal ubiquitin-dependent protein catabolic process, spindle pole body duplication associated with nuclear envelope	prot_ID_50	AO090003001106

AO090003001545 cluster

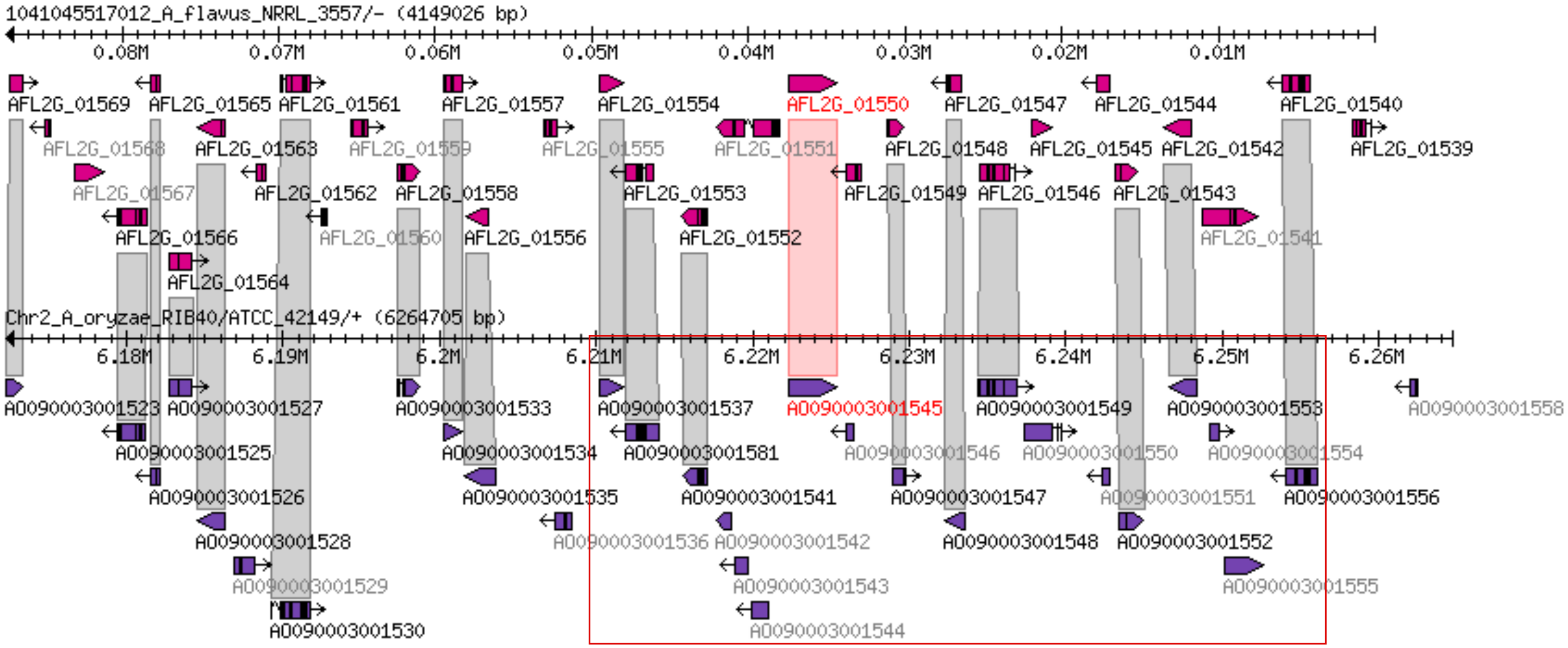

AO090003001545 cluster

Gene id (SMURF)	Gene positions	Gene distance	Gene Name	Annotated_gene_function	antiSMASH ID	AspGD ID	
AO090003001556	-11	1566		Domain(s) with predicted substrate-specific transmembrane transporter activity, role in transmembrane transport and integral to membrane localization	n/a	AO090003001556	ECS
AO090003001555	-10	307		Protein of unknown function	n/a	AO090003001555	
AO090003001554	-9	794		Protein of unknown function	n/a	AO090003001554	
AO090003001553	-8	1539		Ortholog of A. flavus NRRL 3357 : AFL2T_01542	n/a	AO090003001553	
AO090003001552	-7	667		Domain(s) with predicted DNA binding, site-specific DNA-methyltransferase (cytosine-N4-specific) activity, role in N-4 methylation of cytosine, transmembrane transport and integral to membrane localization	prot_ID_1561	AO090003001552	
AO090003001551	-6	2558		Domain(s) with predicted catalytic activity and role in metabolic process	prot_ID_1074	AO090003001551	
AO090003001550	-5	1558		Domain(s) with predicted transferase activity, transferring phosphorus-containing groups activity	prot_ID_343	AO090003001550	
AO090003001549	-4	1196		Domain(s) with predicted transporter activity, role in oligopeptide transport and membrane localization	prot_ID_1482	AO090003001549	
AO090003001548	-3	2868		Ortholog of A. nidulans FGSC A4 : AN5399, A. fumigatus Af293 : Afu2g15280, A. niger CBS 513.88 : An18g02600, A. niger ATCC 1015 : 42737-mRNA and A. versicolor : Aspve1_0045700	prot_ID_845	AO090003001548	
AO090003001547	-2	2442		Ortholog of A. niger CBS 513.88 : An06g00960, An05g01080, A. brasiliensis : Aspbr1_0186106, A. flavus NRRL 3357 : AFL2T_01548 and A. clavatus NRRL 1 : ACLA_003370	prot_ID_1748	AO090003001547	
AO090003001546	-1	543		Protein of unknown function	prot_ID_1128	AO090003001546	
**AO090003001545**	0	0		Non-ribosomal peptide synthetase	prot_ID_1050	AO090003001545	
AO090003001544	1	1161		Domain(s) with predicted electron carrier activity, heme binding, iron ion binding, oxidoreductase activity, acting on paired donors, with incorporation or reduction of molecular oxygen activity and role in oxidation-reduction process	prot_ID_436	AO090003001544	
AO090003001543	2	260		Domain(s) with predicted sequence-specific DNA binding RNA polymerase II transcription factor activity, zinc ion binding activity, role in regulation of transcription, DNA-dependent and nucleus localization	prot_ID_1567	AO090003001543	
AO090003001542	3	323		Protein of unknown function	prot_ID_475	AO090003001542	
AO090003001541	4	601		Monocarboxylate transporter-like protein; predominantly expressed in the basal region of hyphae	prot_ID_773	AO090003001541	
n/a				Ortholog of A. flavus NRRL 3357 : AFL2T_01553	prot_ID_1938	AO090003001581	
n/a				Ortholog of A. flavus NRRL 3357 : AFL2T_01554	prot_ID_1016	AO090003001537	ECS

n/a	Protein of unknown function	prot_ID_210	AO090003001536
n/a	Protein of unknown function	prot_ID_1703	AO090003001535

AO090005000688 cluster

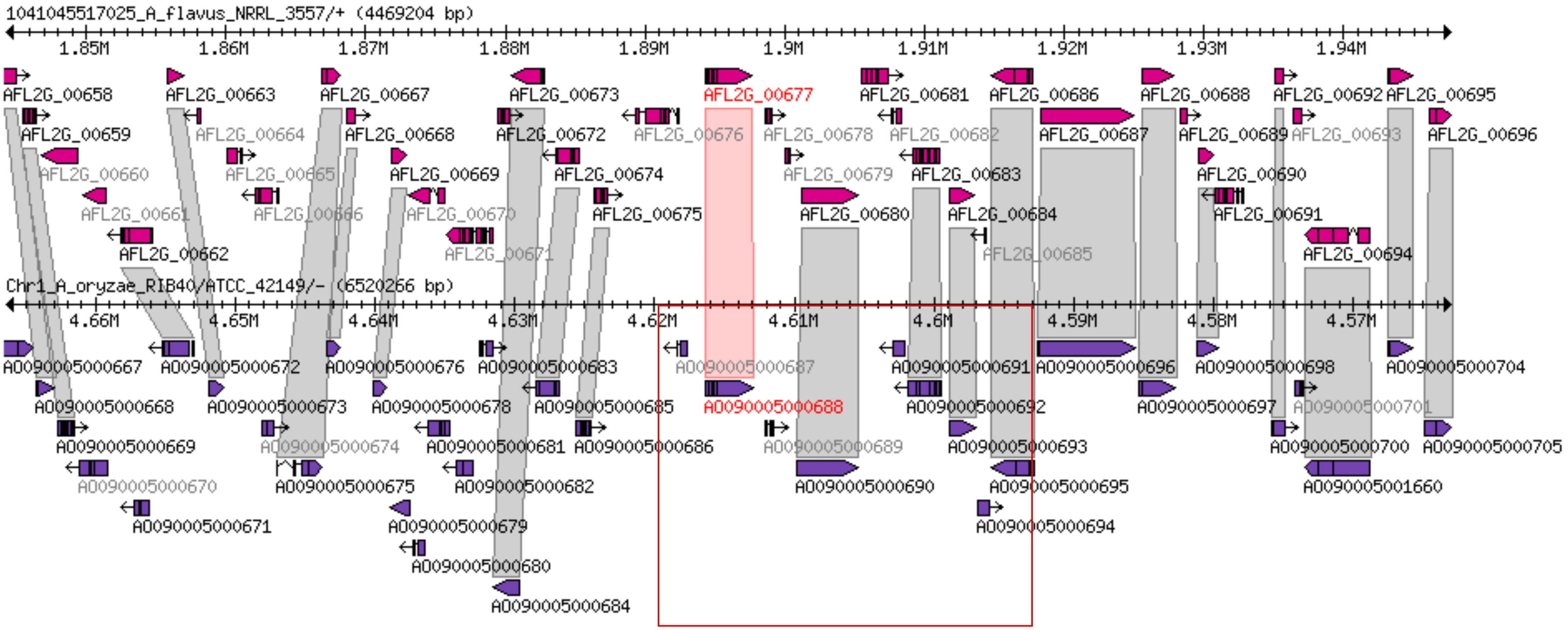

AO090005000688 cluster

Gene id (SMURF)	Gene positions	Gene distance	Gene Name	Annotated_gene_function	antiSMASH ID	AspGD ID	
n/a				Domain(s) with predicted oxidoreductase activity, acting on the aldehyde or oxo group of donors, NAD or NADP as acceptor activity and role in oxidation-reduction process	prot_ID_9	AO090005000681	
n/a				Putative hydrolase	prot_ID_1495	AO090005000682	
n/a				Domain(s) with predicted 3-hydroxyanthranilate 3,4-dioxygenase activity, iron ion binding activity and role in oxidation-reduction process	prot_ID_347	AO090005000683	
n/a				Domain(s) with predicted DNA binding, zinc ion binding activity, role in transcription, DNA-dependent and nucleus localization	prot_ID_1705	AO090005000684	
n/a				Ortholog of A. flavus NRRL 3357 : AFL2T_00674, A. versicolor : Aspve1_0083731 and A. sydowii : Aspsy1_0162677	prot_ID_912	AO090005000685	
n/a				Domain(s) with predicted hydrolase activity	prot_ID_1801	AO090005000686	
n/a				Domain(s) with predicted catalytic activity, nucleotide binding activity and role in biosynthetic process	prot_ID_799	AO090005000687	FA
**AO090005000688**	0	0		Domain(s) with predicted ACP phosphopantetheine attachment site binding involved in fatty acid biosynthetic process, catalytic activity, nucleotide binding, phosphopantetheine binding activity and role in metabolic process	prot_ID_113	AO090005000688	
AO090005000689	1	948		Protein of unknown function	prot_ID_1106	AO090005000689	
AO090005000690	2	1973		Non-ribosomal peptide synthetase	prot_ID_1611	AO090005000690	
AO090005000691	3	2690		Ortholog of A. nidulans FGSC A4 : AN11048, AN5410, A. fumigatus Af293 : Afu3g01150, Afu4g01120, A. niger CBS 513.88 : An12g10330, An11g03520, An02g14500 and A. niger ATCC 1015 : 205254-mRNA	prot_ID_598	AO090005000691	
AO090005000692	4	856		Domain(s) with predicted carbon-nitrogen ligase activity, with glutamine as amido-N-donor, hydrolase activity, acting on carbon-nitrogen (but not peptide) bonds, in linear amides activity	prot_ID_1746	AO090005000692	
AO090005000693	5	912		Domain(s) with predicted role in transmembrane transport and integral to membrane localization	prot_ID_395	AO090005000693	
n/a				Ortholog(s) have ureidoglycolate hydrolase activity	prot_ID_1028	AO090005000694	
n/a				Ortholog(s) have DNA-dependent ATPase activity, dinucleotide insertion or deletion binding, guanine/thymine mispair binding activity, role in mismatch repair, mitochondrial DNA repair and mitochondrion localization	prot_ID_1669	AO090005000695	ECS
n/a				Ortholog(s) have ATP-dependent RNA helicase activity, role in spliceosome conformational change to release U4 (or U4atac) and U1 (or U11) and U4/U6 x U5 tri-snRNP complex, U5 snRNP localization	prot_ID_537	AO090005000696	

AO090005000798 cluster

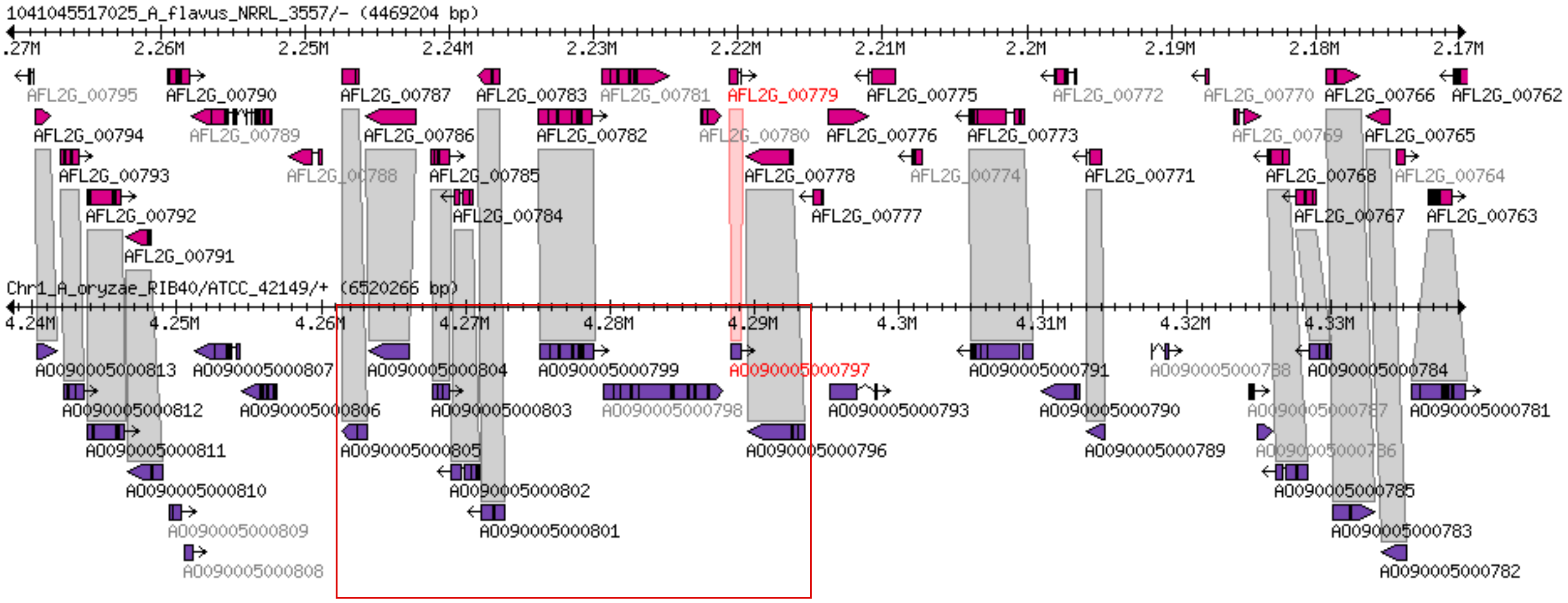

AO090005000798 cluster

Gene id (SMURF)	Gene positions	Gene distance	Gene Name	Annotated_gene_function	antiSMASH ID	AspGD ID	
n/a				Ortholog of A. nidulans FGSC A4 : AN0287, A. fumigatus Af293 : Afu1g02990, A. niger ATCC 1015 : 170766-mRNA, N. fischeri NRRL 181 : NFIA_021640 and A. flavus NRRL 3357 : AFL2T_00773	prot_ID_1851	AO090005000791	
n/a				Ortholog(s) have role in mRNA splicing, via spliceosome and U2-type catalytic step 1 spliceosome, U2-type spliceosomal complex localization	prot_ID_1025	AO090005000793	
n/a				Ortholog of A. nidulans FGSC A4 : AN9448, A. fumigatus Af293 : Afu1g03030, A. niger ATCC 1015 : 51952-mRNA, A. versicolor : Aspve1_0048537 and A. sydowii : Aspsy1_0628822	prot_ID_388	AO090005000796	ECS
n/a				Domain(s) with predicted metal ion binding, phosphoric diester hydrolase activity	prot_ID_156	AO090005000797	
n/a				Polyketide synthase	prot_ID_1842	AO090005000798	
n/a				Domain(s) with predicted ATP binding, calcium-transporting ATPase activity, metal ion binding activity, role in ATP biosynthetic process, calcium ion transmembrane transport and membrane localization	prot_ID_439	AO090005000799	
n/a				Ortholog of A. nidulans FGSC A4 : AN4149, A. flavus NRRL 3357 : AFL2T_00783, A. versicolor : Aspve1_0168074, Aspve1_0655563 and A. sydowii : Aspsy1_0153723	prot_ID_1167	AO090005000801	
n/a				Ortholog of A. nidulans FGSC A4 : AN3415, A. fumigatus Af293 : Afu8g01570, N. fischeri NRRL 181 : NFIA_095060, A. flavus NRRL 3357 : AFL2T_00784 and A. versicolor : Aspve1_0126965	prot_ID_1646	AO090005000802	
n/a				Ortholog of A. nidulans FGSC A4 : AN9450, A. fumigatus Af293 : Afu1g03040, A. niger CBS 513.88 : An01g05230, A. niger ATCC 1015 : 51951-mRNA and A. versicolor : Aspve1_0231478	prot_ID_482	AO090005000803	
n/a				Domain(s) with predicted ATP binding, DNA binding, helicase activity	prot_ID_1925	AO090005000804	
n/a				Ortholog of A. brasiliensis : Aspbr1_0053309, A. flavus NRRL 3357 : AFL2T_00787, A. acidus : Aspfo1_0052424 and A. niger ATCC 1015 : 52513-mRNA	prot_ID_504	AO090005000805	ECS

AO090005000952 cluster

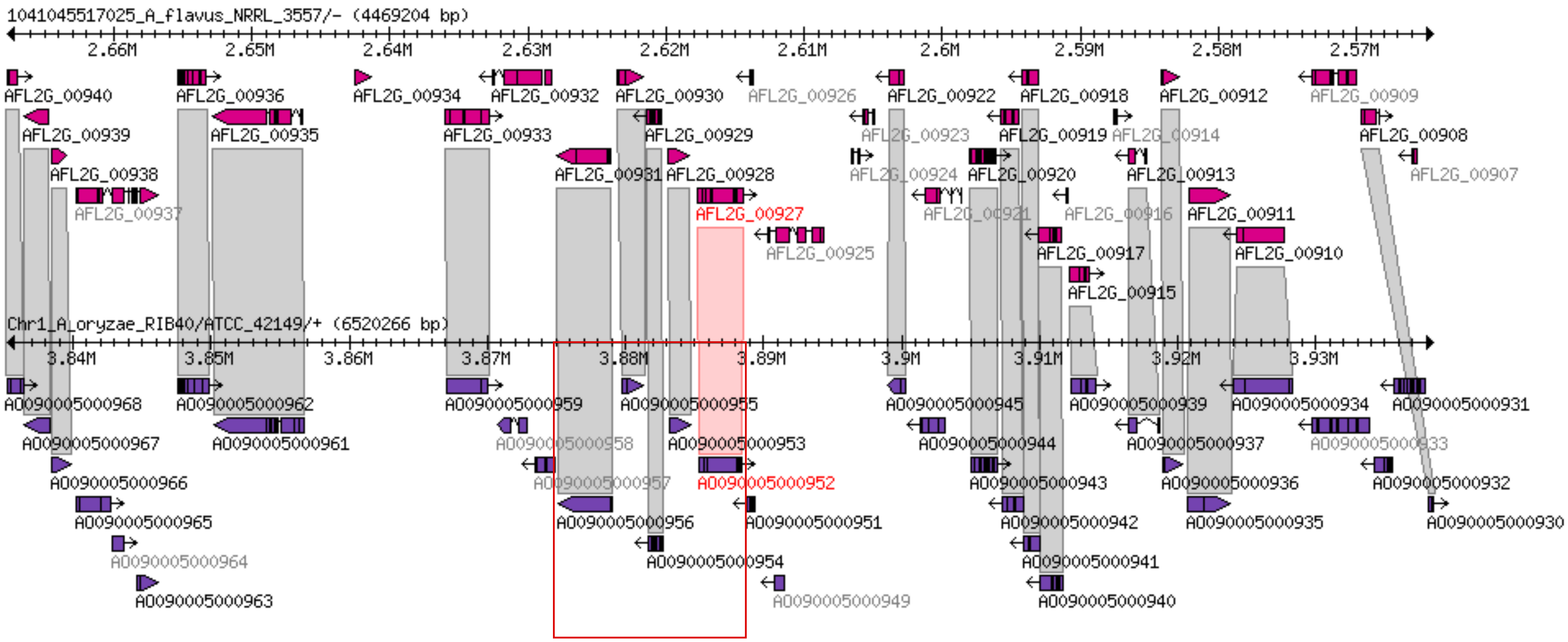

AO090005000952 cluster

Gene id (SMURF)	Gene positions	Gene distance	Gene Name	Annotated_gene_function	antiSMASH ID	AspGD ID	
n/a				Domain(s) with predicted N-acetyltransferase activity and role in metabolic process	prot_ID_166	AO090005000942	
n/a				Domain(s) with predicted N,N-dimethylaniline monooxygenase activity, NADP binding, flavin adenine dinucleotide binding activity and role in oxidation-reduction process	prot_ID_941	AO090005000943	
n/a				Ortholog of A. niger CBS 513.88 : An11g06490, An18g01440, A. brasiliensis : Aspbr1_0210166, A. acidus : Aspfo1_0049126, Aspfo1_0123750 and A.s versicolor : Aspve1_0145746	prot_ID_1810	AO090005000944	
n/a				Ortholog of N. fischeri NRRL 181 : NFIA_115130, A. flavus NRRL 3357 : AFL2T_00922, A. clavatus NRRL 1 : ACLA_065130 and A. versicolor : Aspve1_0057776	prot_ID_703	AO090005000945	
n/a				Protein of unknown function	prot_ID_163	AO090005000949	
n/a				Ortholog of A. nidulans FGSC A4 : AN10907, A. fumigatus Af293 : Afu5g00975, A. brasiliensis : Aspbr1_0198422, A. acidus : Aspfo1_0081662 and A. versicolor : Aspve1_0069870	prot_ID_965	AO090005000951	
**AO090005000952**	0	0		Non-ribosomal peptide synthetase	prot_ID_1511	AO090005000952	ECS
AO090005000953	1	595		Domain(s) with predicted transporter activity, role in transmembrane transport and integral to membrane localization	prot_ID_705	AO090005000953	
AO090005000954	2	502		Domain(s) with predicted aminopeptidase activity and role in proteolysis	prot_ID_368	AO090005000954	
AO090005000955	3	220		Ortholog(s) have sequence-specific DNA binding, sequence-specific DNA binding transcription factor activity	prot_ID_1288	AO090005000955	
n/a				Domain(s) with predicted hydrolase activity	prot_ID_81	AO090005000956	ECS
n/a				Protein of unknown function	prot_ID_1010	AO090005000957	
n/a				Protein of unknown function	prot_ID_183	AO090005000958	
n/a				Ortholog of A. fumigatus Af293 : Afu8g04920, A. niger CBS 513.88 : An12g10470, An18g01890, A. oryzae RIB40 : AO090701000445 and A. niger ATCC 1015 : 45984-mRNA, 54098-mRNA	prot_ID_1512	AO090005000959	

AO090005000961 cluster

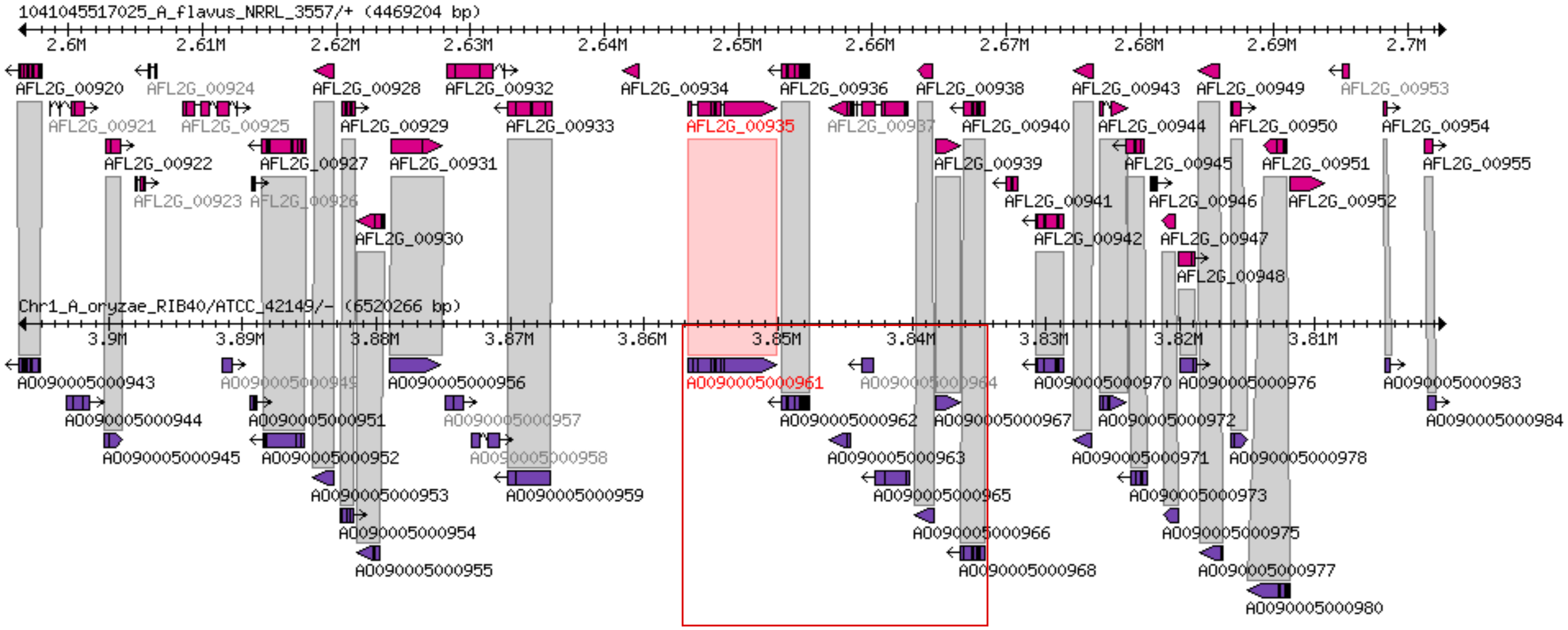

AO090005000961 cluster

Gene id (SMURF)	Gene positions	Gene distance	Gene Name	Annotated_gene_function	antiSMASH ID	AspGD ID	
n/a				Domain(s) with predicted hydrolase activity	prot_ID_81	AO090005000956	
n/a				Protein of unknown function	prot_ID_1010	AO090005000957	
n/a				Protein of unknown function	prot_ID_183	AO090005000958	
n/a				Ortholog of A. fumigatus Af293 : Afu8g04920, A. niger CBS 513.88 : An12g10470, An18g01890, A. oryzae RIB40 : AO090701000445 and A. niger ATCC 1015 : 45984-mRNA, 54098-mRNA	prot_ID_1512	AO090005000959	
**AO090005000961**	0	0		Polyketide synthase	prot_ID_6	AO090005000961	ECS
AO090005000962	1	536		Ortholog(s) have role in cellular response to glucose stimulus and cytoplasm localization	prot_ID_1430	AO090005000962	
AO090005000963	2	1461		Domain(s) with predicted transporter activity, role in transmembrane transport and integral to membrane localization	prot_ID_436	AO090005000963	
AO090005000964	3	905			prot_ID_1271	AO090005000964	
AO090005000965	4	530		Domain(s) with predicted ATP binding, nucleoside-triphosphatase activity	prot_ID_592	AO090005000965	
AO090005000966	5	774		Ortholog of A. flavus NRRL 3357 : AFL2T_00938 and A. versicolor : Aspve1_0390716	prot_ID_1982	AO090005000966	
AO090005000967	6	283		Domain(s) with predicted nucleotide binding, oxidoreductase activity and role in metabolic process	prot_ID_982	AO090005000967	
AO090005000968	7	119		Major facilitator superfamily (MFS) transporter; upregulated by exposure to benomyl	prot_ID_1818	AO090005000968	ECS
				Domain(s) with predicted choline dehydrogenase activity, flavin adenine dinucleotide binding activity and role in alcohol metabolic process, oxidation-reduction process	prot_ID_827	AO090005000970	

AO090005000993 cluster

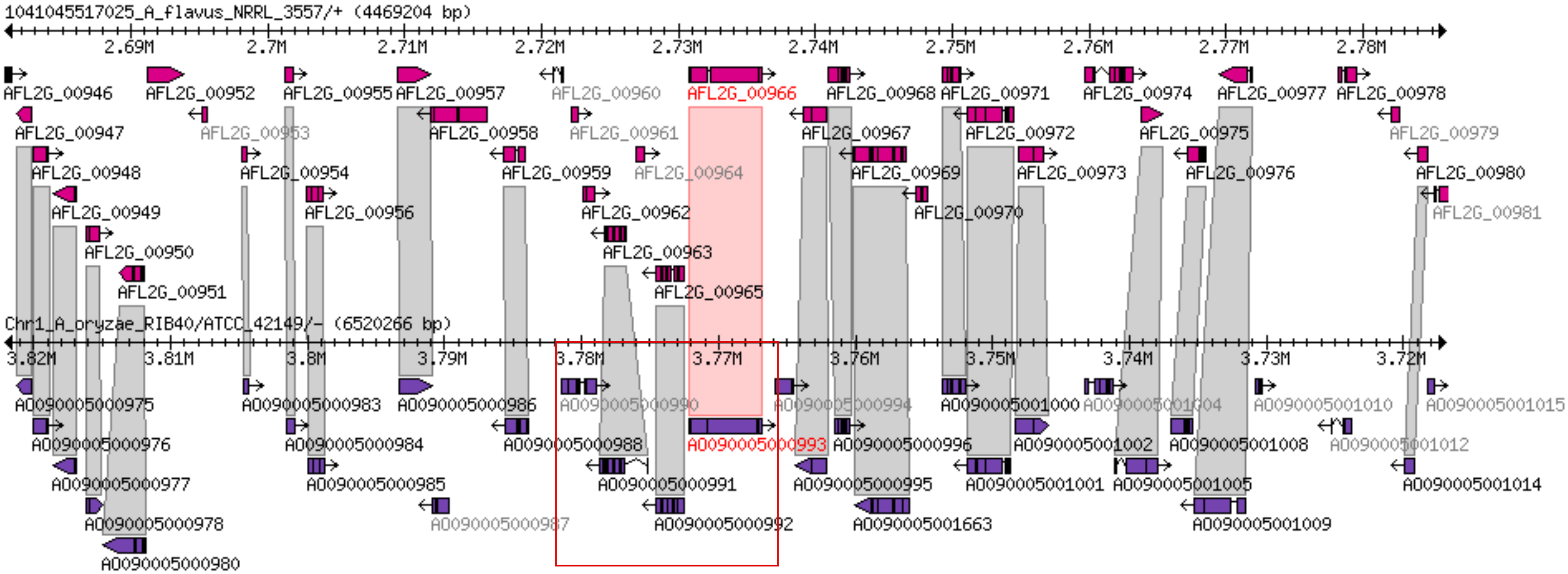

AO090005000993 cluster

Gene id (SMURF)	Gene positions	Gene distance	Gene Name	Annotated_gene_function	antiSMASH ID	AspGD ID	
n/a			xylA	Beta-xylosidase, extracellular enzyme with a role in xylan degradation; key enzyme responsible for dark brown color produced in soy sauce brewing; induced by growth on xylose; positively regulated by XlnR; 3 XlnR binding sites in promoter	prot_ID_747	AO090005000986	
n/a				Domain(s) with predicted ATP binding, ATPase activity	prot_ID_1951	AO090005000987	
n/a				Ortholog of A. nidulans FGSC A4 : AN0638, A. fumigatus Af293 : Afu1g16870, A. niger ATCC 1015 : 170269-mRNA, A. versicolor : Aspve1_0119892 and A. sydowii : Aspsy1_0129541	prot_ID_538	AO090005000988	
AO090005000990	-3	265		Domain(s) with predicted sequence-specific DNA binding RNA polymerase II transcription factor activity, zinc ion binding activity, role in regulation of transcription, DNA-dependent and nucleus localization	prot_ID_1274	AO090005000990	ECS
AO090005000991	-2	697		Domain(s) with predicted transporter activity, role in transmembrane transport and integral to membrane localization	prot_ID_251	AO090005000991	
AO090005000992	-1	407		Domain(s) with predicted catalytic activity and role in metabolic process	prot_ID_1182	AO090005000992	
**AO090005000993**	0	0		Non-ribosomal peptide synthetase	prot_ID_95	AO090005000993	ECS
AO090005000994	1	1140		Protein of unknown function	prot_ID_862	AO090005000994	
AO090005000995	2	678		Ortholog(s) have sphingosine hydroxylase activity, role in ascospore formation, hyphal growth, sphingolipid biosynthetic process and endoplasmic reticulum membrane localization	prot_ID_1966	AO090005000995	
AO090005000996	3	1136		Putative microtubule-binding protein; alternative polyadenylation sites predicted	prot_ID_848	AO090005000996	
AO090005001663	4	557		Domain(s) with predicted 7S RNA binding, DNA binding, sequence-specific DNA binding RNA polymerase II transcription factor activity, zinc ion binding activity	prot_ID_1998	AO090005001663	
AO090005001000	5	2754		Domain(s) with predicted pyridoxal phosphate binding, transaminase activity	prot_ID_1787	AO090005001000	
AO090005001001	6	217		Putative protein factor similar to Ysh1p involved in pre-mRNA 3'-end-processing	prot_ID_977	AO090005001001	
n/a				Domain(s) with predicted role in DNA-dependent transcription, initiation and transcription factor TFIID complex localization	prot_ID_1567	AO090005001002	

AO090005001079 cluster

No manual prediction made

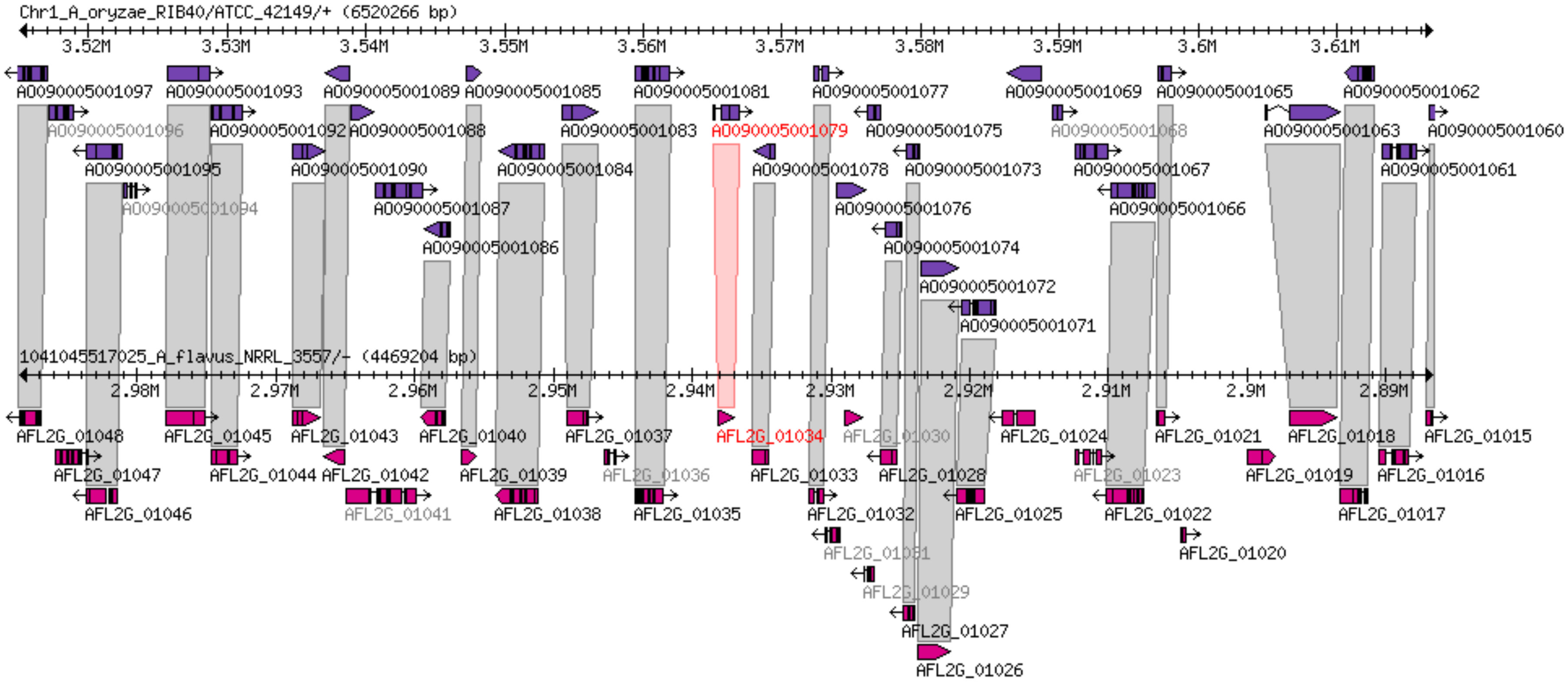

AO090005001079 cluster

Gene id (SMURF)	Gene positions	Gene distance	Gene Name	Annotated_gene_function	antiSMASH ID	AspGD ID
n/a				Ortholog of A. fumigatus Af293 : Afu1g16300, A. niger CBS 513.88 : An04g08050, An02g13120, An01g10860, A. oryzae RIB40 : AO090010000043/aoiC and A. versicolor : Aspve1_0116911	prot_ID_795	AO090005001075
n/a				Ortholog(s) have RNA-dependent ATPase activity	prot_ID_1235	AO090005001076
n/a				Ortholog(s) have proton-transporting ATPase activity, rotational mechanism, structural molecule activity and role in ATP synthesis coupled proton transport, cristae formation, protein oligomerization	prot_ID_1364	AO090005001077
AO090005001078	-1	1239		NAD+-dependent L-arabinitol 4-dehydrogenase; transcriptionally induced by growth on xylose; positively regulated by XlnR; 2 putative XlnR binding sites in the promoter	prot_ID_1735	AO090005001078
**AO090005001079**	0	0		Putative dimethylallyl tryptophan synthase (DMAT)	prot_ID_1102	AO090005001079
AO090005001081	1	3305		Domain(s) with predicted amino acid transmembrane transporter activity, role in amino acid transmembrane transport and membrane localization	prot_ID_330	AO090005001081
AO090005001083	2	3393		Domain(s) with predicted role in cellular metabolic process	n/a	AO090005001083
AO090005001084	3	1865		Alpha-glucosidase	n/a	AO090005001084
AO090005001085	4	1556		Domain(s) with predicted catalytic activity and role in metabolic process	n/a	AO090005001085
AO090005001086	5	1179		Domain(s) with predicted substrate-specific transmembrane transporter activity, role in transmembrane transport and integral to membrane localization	n/a	AO090005001086
AO090005001087	6	367		Domain(s) with predicted DNA binding, sequence-specific DNA binding RNA polymerase II transcription factor activity, zinc ion binding activity and role in regulation of transcription, DNA-dependent, transcription, DNA-dependent	n/a	AO090005001087

AO090005001551 cluster

Probably an orphan PKS, no manual prediction made

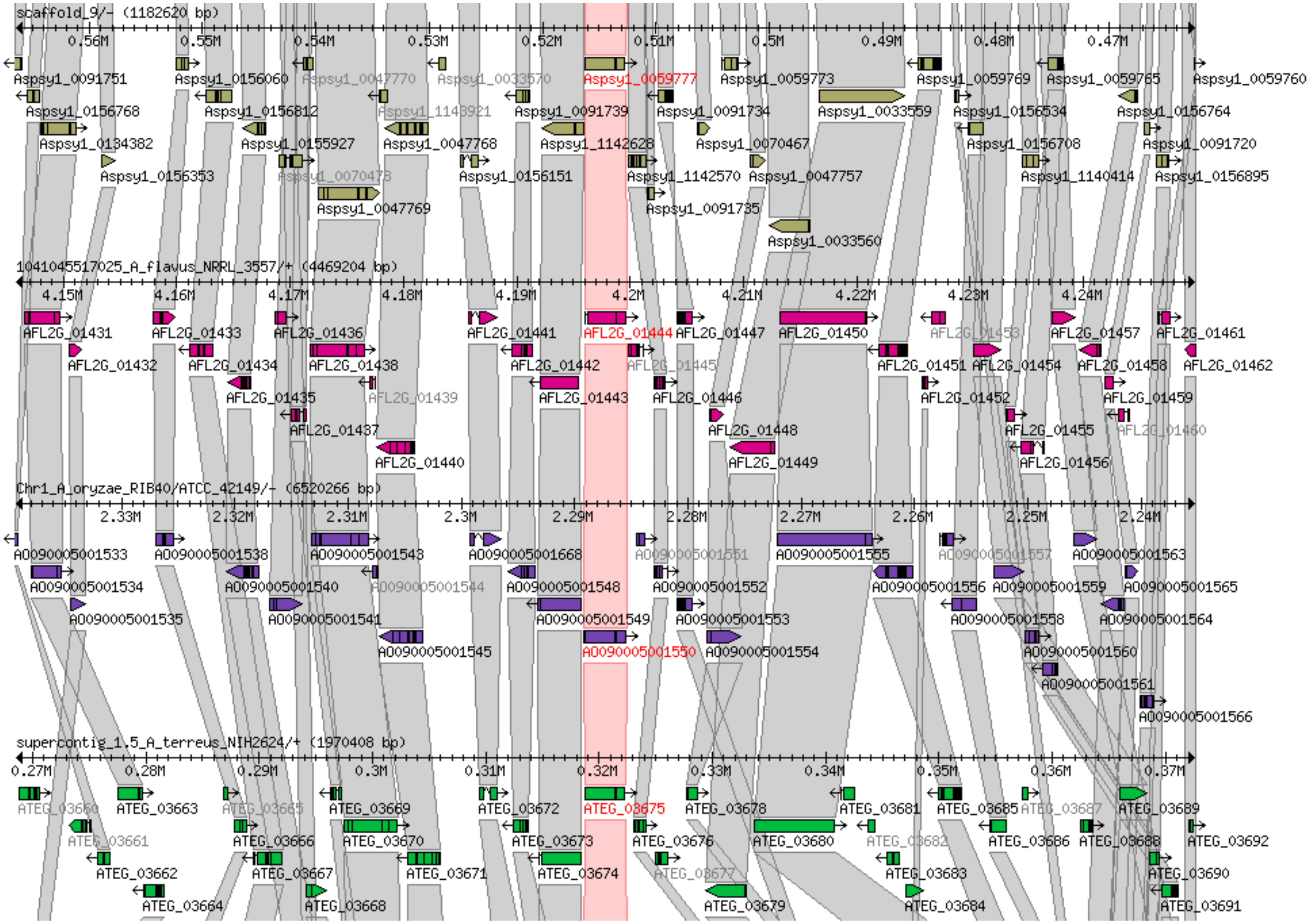

AO090005001551 cluster

Gene id (SMURF)	Gene positions	Gene distance	Gene Name	Annotated_gene_function	antiSMASH ID	AspGD ID
n/a				Domain(s) with predicted protein dimerization activity, sequence-specific DNA binding, sequence-specific DNA binding transcription factor activity and role in regulation of transcription, DNA-dependent	prot_ID_2010	AO090005001668
n/a				Ortholog(s) have lipid binding activity and role in eisosome assembly, endocytosis, negative regulation of protein kinase activity, protein localization, response to heat	prot_ID_1527	AO090005001548
n/a				Domain(s) with predicted endonuclease activity, single-stranded DNA binding activity, role in nucleotide-excision repair and nucleus localization	prot_ID_704	AO090005001549
n/a				Ortholog(s) have role in endocytic recycling and endosome localization	prot_ID_1283	AO090005001550
n/a			csyD	Putative type III polyketide synthase	prot_ID_1779	AO090005001551
n/a				Putative acetylmannan esterase; transcriptionally regulated by ManR	prot_ID_1199	AO090005001552
n/a				Ortholog(s) have cellulase activity and role in xyloglucan catabolic process	prot_ID_7	AO090005001553
n/a				Ortholog(s) have role in fungal-type cell wall organization, protein oligomerization and cellular bud, cytoplasm, membrane raft, plasma membrane localization	prot_ID_1405	AO090005001554
n/a				Ortholog(s) have 1-phosphatidylinositol-3-phosphate 5-kinase activity, phosphatidylinositol-3-phosphate binding activity and role in phosphatidylinositol phosphorylation	prot_ID_1714	AO090005001555

AO090009000052 cluster

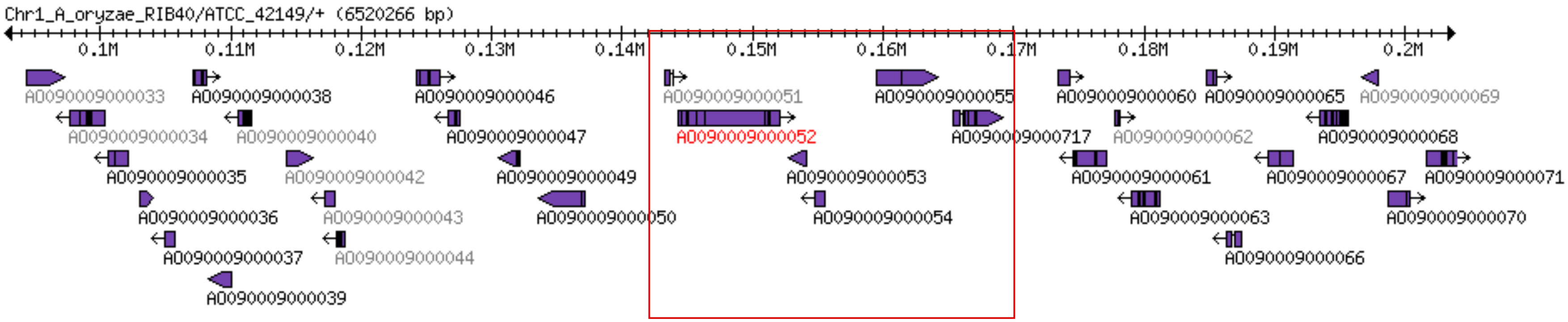

AO090009000052 cluster

Gene id (SMURF)	Gene positions	Gene distance	Gene Name	Annotated_gene_function	antiSMASH ID	AspGD ID	
n/a				Domain(s) with predicted role in transmembrane transport and integral to membrane localization	prot_ID_82	AO090009000046	
n/a				Domain(s) with predicted nucleotide binding, oxidoreductase activity and role in oxidation-reduction process	prot_ID_1481	AO090009000047	
n/a				Domain(s) with predicted role in transmembrane transport and integral to membrane localization	prot_ID_1958	AO090009000049	
n/a				Ortholog of A. nidulans FGSC A4 : AN3479, AN8495, A. fumigatus Af293 : Afu3g01440, Afu4g03340, Afu5g07620, A. niger CBS 513.88 : An13g02880 and A. niger ATCC 1015 : 129924-mRNA	prot_ID_1626	AO090009000050	
n/a				Protein of unknown function	prot_ID_895	AO090009000051	IGD
n/a				Polyketide synthase	prot_ID_1792	AO090009000052	
n/a				Domain(s) with predicted solute:hydrogen antiporter activity, role in cation transport, transmembrane transport and integral to membrane localization	prot_ID_896	AO090009000053	
n/a				Ortholog of Aspergillus flavus NRRL 3357 : AFL2T_10595, A. versicolor : Aspve1_0078217 and A. sydowii : Aspsy1_0027985	prot_ID_1950	AO090009000054	
n/a				Similar to pleiotropic drug resistance proteins, ABC superfamily; upregulated by exposure to benomyl	prot_ID_1301	AO090009000055	
n/a				Domain(s) with predicted sequence-specific DNA binding RNA polymerase II transcription factor activity, zinc ion binding activity, role in regulation of transcription, DNA-dependent and nucleus localization	prot_ID_2012	AO090009000717	IGD

AO090009000071 cluster

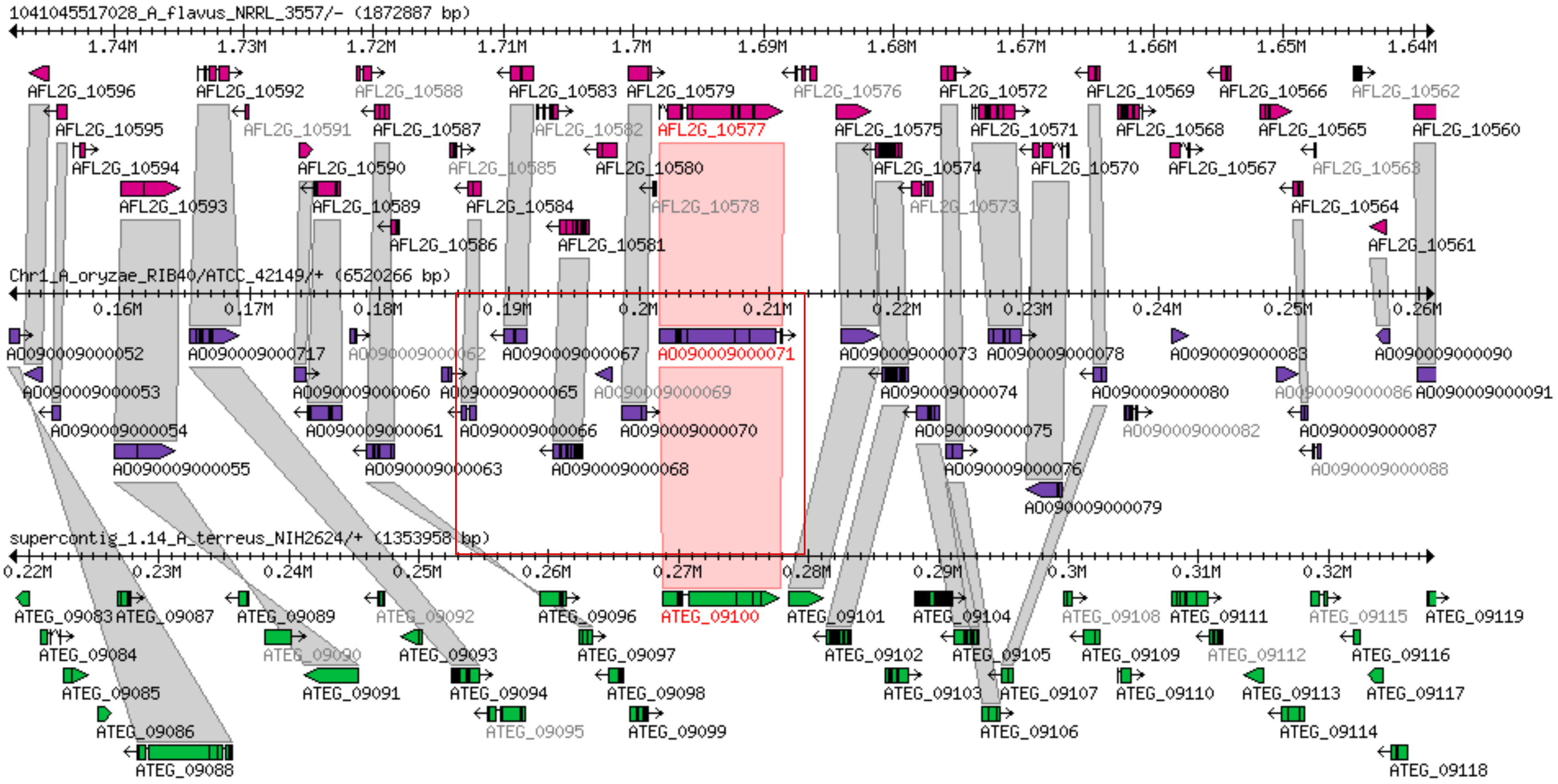

AO090009000071 cluster

Gene id (SMURF)	Gene positions	Gene distance	Gene Name	Annotated_gene_function	antiSMASH ID	AspGD ID	
n/a				Domain(s) with predicted sequence-specific DNA binding RNA polymerase II transcription factor activity, zinc ion binding activity, role in regulation of transcription, DNA-dependent and nucleus localization	prot_ID_769	AO090009000079	
n/a				Domain(s) with predicted oxidoreductase activity, transferase activity and role in biosynthetic process, oxidation-reduction process	prot_ID_1415	AO090009000078	
n/a				Domain(s) with predicted oxidoreductase activity and role in oxidation-reduction process	prot_ID_327	AO090009000076	
n/a				Domain(s) with predicted N,N-dimethylaniline monooxygenase activity, NADP binding, flavin adenine dinucleotide binding activity, role in oxidation-reduction process and intrinsic to endoplasmic reticulum membrane localization	prot_ID_1265	AO090009000075	
n/a				Domain(s) with predicted role in transmembrane transport and integral to membrane localization	prot_ID_112	AO090009000074	
n/a				Domain(s) with predicted RNA binding, RNA-directed DNA polymerase activity and role in RNA-dependent DNA replication	prot_ID_987	AO090009000073	
**AO090009000071**	0	0		Polyketide synthase	prot_ID_1572	AO090009000071	ECS, IGD
AO090009000070	1	1233		Ortholog of A. nidulans FGSC A4 : AN5608, AN4473, A. fumigatus Af293 : Afu4g07720, A. niger CBS 513.88 : An14g07030, An04g02100 and A. oryzae RIB40 : AO090023000800	prot_ID_432	AO090009000070	
AO090009000069	2	832		Protein of unknown function	prot_ID_995	AO090009000069	
AO090009000068	3	1070		Domain(s) with predicted role in transmembrane transport and integral to membrane localization	prot_ID_447	AO090009000068	
n/a				Ortholog(s) have hydroquinone:oxygen oxidoreductase activity and role in melanin biosynthetic process, pigment metabolic process involved in developmental pigmentation	prot_ID_663	AO090009000067	
n/a				Ortholog of A. flavus NRRL 3357 : AFL2T_10584, A. clavatus NRRL 1 : ACLA_064070, A. versicolor : Aspve1_0047683 and A. sydowii : Aspsy1_0188643	prot_ID_222	AO090009000066	ECS
n/a				Domain(s) with predicted aldehyde-lyase activity, zinc ion binding activity and role in carbohydrate metabolic process	prot_ID_1330	AO090009000065	

AO090009000131 cluster

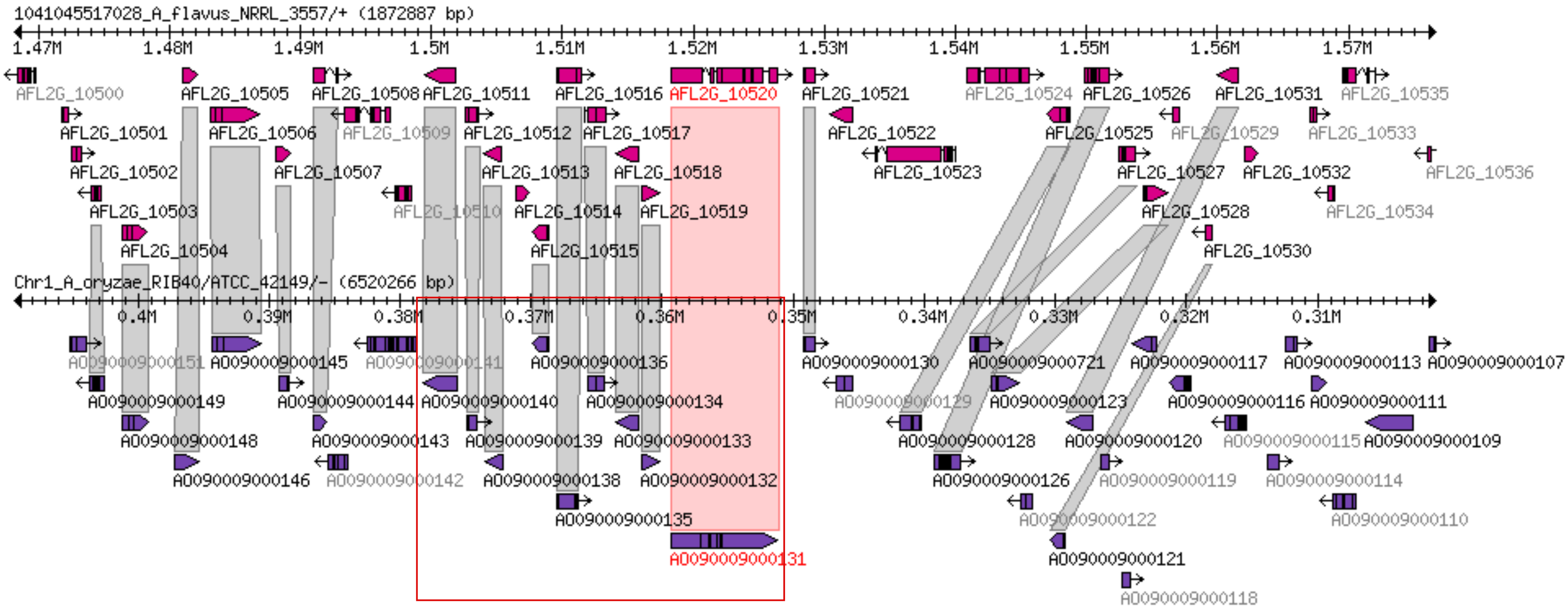

AO090009000131 cluster

Gene id (SMURF)	Gene positions	Gene distance	Gene Name	Annotated_gene_function	antiSMASH ID	AspGD ID	
AO090009000143	-11	130		Domain(s) with predicted hydrolase activity	n/a	AO090009000143	
AO090009000142	-10	1483		Protein of unknown function	n/a	AO090009000142	
AO090009000141	-9	692		Domain(s) with predicted metal ion binding activity, role in transmembrane transport and integral to membrane localization	prot_ID_1860	AO090009000141	
AO090009000140	-8	776		Domain(s) with predicted hydroxymethyl-, formyl- and related transferase activity and role in biosynthetic process	prot_ID_556	AO090009000140	FA
AO090009000139	-7	490		Domain(s) with predicted methylenetetrahydrofolate dehydrogenase (NADP+) activity, nucleotide binding activity and role in folic acid-containing compound biosynthetic process, oxidation-reduction process	prot_ID_55	AO090009000139	
AO090009000138	-6	2450		Domain(s) with predicted role in transmembrane transport and integral to membrane localization	prot_ID_1841	AO090009000138	
AO090009000136	-5	640		Homoserine dehydrogenase	prot_ID_568	AO090009000136	
AO090009000135	-4	748		Domain(s) with predicted oxidoreductase activity, acting on the aldehyde or oxo group of donors, NAD or NADP as acceptor activity and role in oxidation-reduction process	prot_ID_1740	AO090009000135	
AO090009000134	-3	787		Ortholog of A. flavus NRRL 3357 : AFL2T_10517	prot_ID_1295	AO090009000134	
AO090009000133	-2	358		Domain(s) with predicted sequence-specific DNA binding RNA polymerase II transcription factor activity, zinc ion binding activity, role in regulation of transcription, DNA-dependent and nucleus localization	prot_ID_472	AO090009000133	
AO090009000132	-1	854		Ortholog of A. fumigatus Af293 : Afu8g00530, A. niger CBS 513.88 : An07g00020, An13g03240, A. oryzae RIB40 : AO090026000248, AO090102000460 and A. versicolor : Aspve1_0038561, Aspve1_0043401	prot_ID_232	AO090009000132	
**AO090009000131**	0	0		Polyketide synthase	prot_ID_1834	AO090009000131	ECS
n/a				Ortholog of A. flavus NRRL 3357 : AFL2T_10521	prot_ID_755	AO090009000130	
n/a				Protein of unknown function	prot_ID_1867	AO090009000129	
n/a			CYP65AC2	Cytochrome P450 monooxygenase	prot_ID_914	AO090009000128	
n/a				Domain(s) with predicted role in transmembrane transport and integral to membrane localization	prot_ID_1254	AO090009000126	
n/a				Ortholog of A. nidulans FGSC A4 : AN6413, AN8328, AN3395, AN3241, AN10369, AN1930, AN2386, AN1738, AN9266, AN10886, AN7232, AN7406, AN7774, AN12202	prot_ID_1993	AO090009000125.1	
n/a				Domain(s) with predicted catalytic activity, magnesium ion binding, thiamine pyrophosphate binding activity	prot_ID_167	AO090009000123	
n/a				Domain(s) with predicted N-acetyltransferase activity and role in metabolic process	prot_ID_1863	AO090009000122	

AO090010000048 (aoi) cluster
Experimentally determined cluster

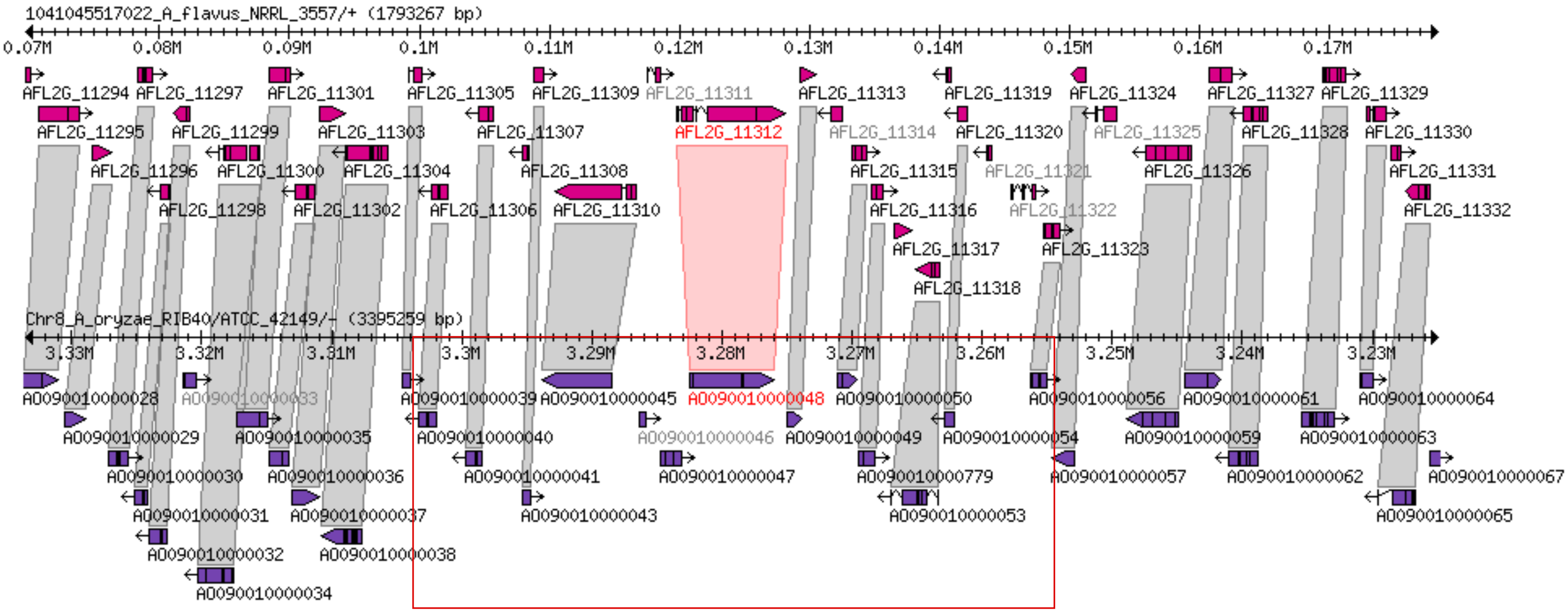

AO090010000048 (aoi) cluster

Gene id (SMURF)	Gene positions	Gene distance	Gene Name	Annotated_gene_function	antiSMASH ID	AspGD ID	
AO090010000035	-11	289		Domain(s) with predicted substrate-specific transmembrane transporter activity, role in transmembrane transport and integral to membrane localization	n/a	AO090010000035	
AO090010000036	-10	362		Domain(s) with predicted role in transmembrane transport and integral to membrane localization	n/a	AO090010000036	
AO090010000037	-9	117		Domain(s) with predicted DNA binding, sequence-specific DNA binding RNA polymerase II transcription factor activity, zinc ion binding activity and role in regulation of transcription, DNA-dependent, transcription, DNA-dependent	n/a	AO090010000037	
AO090010000038	-8	3107		Ortholog of A. flavus NRRL 3357 : AFL2T_11304, A. versicolor : Aspve1_0774606 and A. sydowii : Aspsy1_0057550	n/a	AO090010000038	
AO090010000039	-7	640		Ortholog of A. flavus NRRL 3357 : AFL2T_11305	n/a	AO090010000039	
AO090010000040	-6	2303	aoiA	Member of the aoi gene cluster responsible for polyketide biosynthesis in A. oryzae var. brunneus	prot_ID_40	AO090010000040	ED
AO090010000041	-5	3104	aoiB	Putative alcohol dehydrogenase; member of the aoi gene cluster responsible for polyketide biosynthesis in A. oryzae var. brunneus	prot_ID_650	AO090010000041	
AO090010000043	-4	786	aoiC	Member of the aoi gene cluster responsible for polyketide biosynthesis in A. oryzae var. brunneus	prot_ID_334	AO090010000043	
AO090010000045	-3	2171	aoiD	Member of the aoi gene cluster responsible for polyketide biosynthesis in A. oryzae var. brunneus	prot_ID_748	AO090010000045	
AO090010000046	-2	1125	aoiE	Member of the aoi gene cluster responsible for polyketide biosynthesis in A. oryzae var. brunneus	prot_ID_743	AO090010000046	
AO090010000047	-1	739	aoiF	Putative O-methyltransferase; member of the aoi gene cluster responsible for polyketide biosynthesis in A. oryzae var. brunneus	prot_ID_27	AO090010000047	
**AO090010000048**	0	0	aoiG	Putative iterative polyketide synthase with seven domains; member of the aoi gene cluster responsible for polyketide biosynthesis in A. oryzae var. brunneus	prot_ID_641	AO090010000048	
AO090010000049	1	1014	aoiH	Putative Zn2Cys6 binuclear cluster DNA-binding protein similar to AflR; regulatory member of the aoi gene cluster responsible for polyketide biosynthesis in A. oryzae var. brunneus	prot_ID_453	AO090010000049	
AO090010000050	2	2846	aoiJ	Member of the aoi gene cluster responsible for polyketide biosynthesis in A. oryzae var. brunneus	prot_ID_259	AO090010000050	
AO090010000779	3	424	aoiL	Putative arylamine N-acetyltransferase 2; member of the aoi gene cluster responsible for polyketide biosynthesis in A. oryzae var. brunneus	prot_ID_1083	AO090010000779	
AO090010000053	4	1421	aoiM	Predicted dihydroxy-acid dehydratase; member of the aoi gene cluster responsible for polyketide biosynthesis in A. oryzae var. brunneus	prot_ID_760	AO090010000053	

AO090010000054	5	433	aoiN	Predicted short chain type dehydrogenase; member of the aoi gene cluster responsible for polyketide biosynthesis in A. oryzae var. brunneus	prot_ID_242	AO090010000054	
n/a			aoiO	Predicted O-methyltransferase; member of the aoi gene cluster responsible for polyketide biosynthesis in *A. oryzae var. brunneus*	prot_ID_672	AO090010000056	ED

AO090010000082 cluster

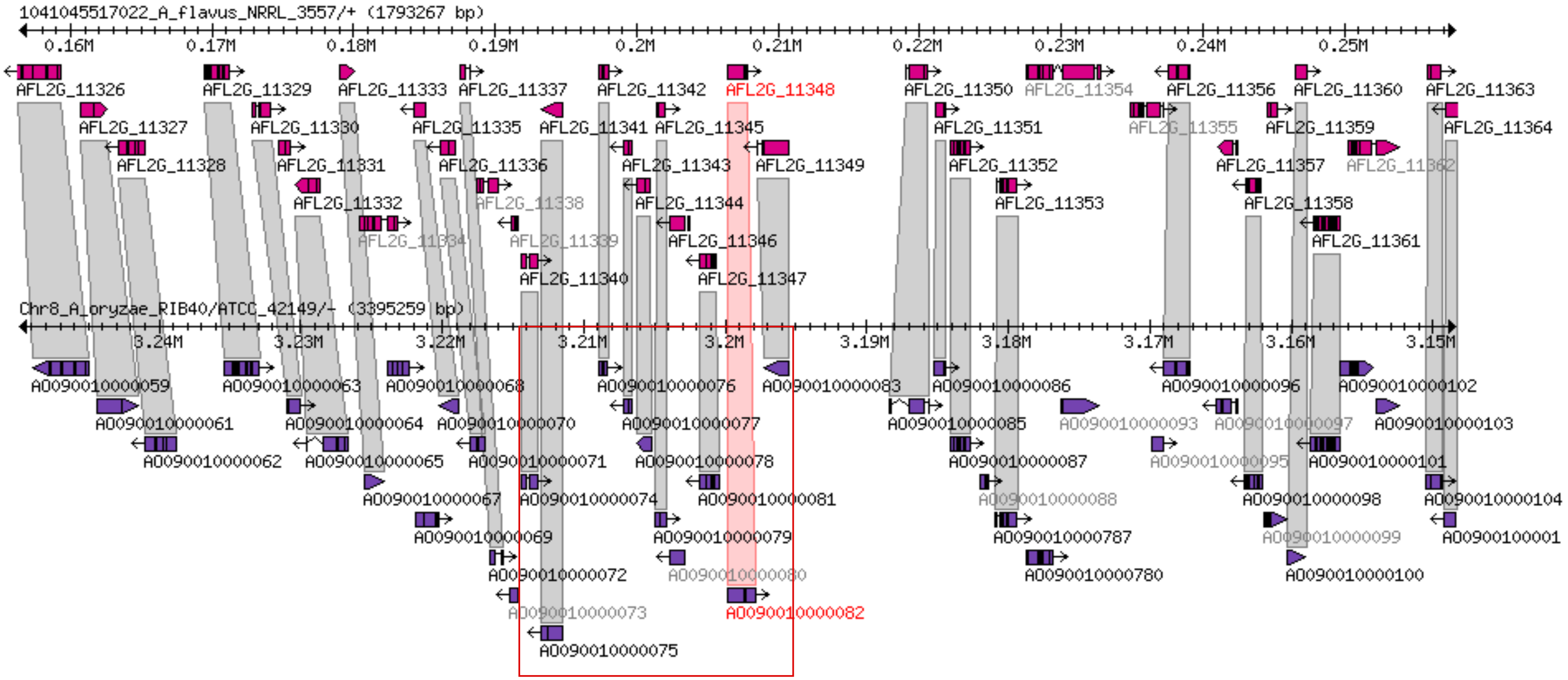

AO090010000082 cluster

Gene id (SMURF)	Gene positions	Gene distance	Gene Name	Annotated_gene_function	antiSMASH ID	AspGD ID	
AO090010000070	-12	1042		Domain(s) with predicted hydrolase activity		AO090010000070	
AO090010000071	-11	365		Domain(s) with predicted catalytic activity, coenzyme binding, nucleotide binding activity and role in cellular metabolic process	n/a	AO090010000071	
AO090010000072	-10	634		Ortholog of A. flavus NRRL 3357 : AFL2T_11337	n/a	AO090010000072	
AO090010000073	-9	305			n/a	AO090010000073	
AO090010000074	-8	184		Domain(s) with predicted oxidoreductase activity, acting on paired donors, with incorporation or reduction of molecular oxygen, 2-oxoglutarate as one donor, and incorporation of one atom each of oxygen into both donors activity	n/a	AO090010000074	ECS
AO090010000075	-7	2515		Cytochrome P450 monooxygenase	n/a	AO090010000075	
AO090010000076	-6	1025		Domain(s) with predicted RNA binding, ribonuclease activity	n/a	AO090010000076	
AO090010000077	-5	408		Domain(s) with predicted lysozyme activity and role in cell wall macromolecule catabolic process, peptidoglycan catabolic process	n/a	AO090010000077	
AO090010000078	-4	404		Domain(s) with predicted nucleotide binding activity	n/a	AO090010000078	
AO090010000079	-3	402		Domain(s) with predicted carbon-sulfur lyase activity and role in metabolic process	n/a	AO090010000079	
AO090010000080	-2	1070		Domain(s) with predicted nucleotide binding activity	n/a	AO090010000080	
AO090010000081	-1	723		Ortholog of A. nidulans FGSC A4 : AN6809, A. fumigatus Af293 : Afu5g00680, A. niger CBS 513.88 : An16g00250, A. niger ATCC 1015 : 210803-mRNA and A. terreus NIH2624 : ATET_09248	n/a	AO090010000081	
**AO090010000082**	0	0		Domain(s) with predicted tryptophan dimethylallyltransferase activity and role in alkaloid metabolic process	n/a	AO090010000082	
n/a				Ortholog of A. flavus NRRL 3357 : AFL2T_11349	n/a	AO090010000083	ECS

AO090010000114 cluster

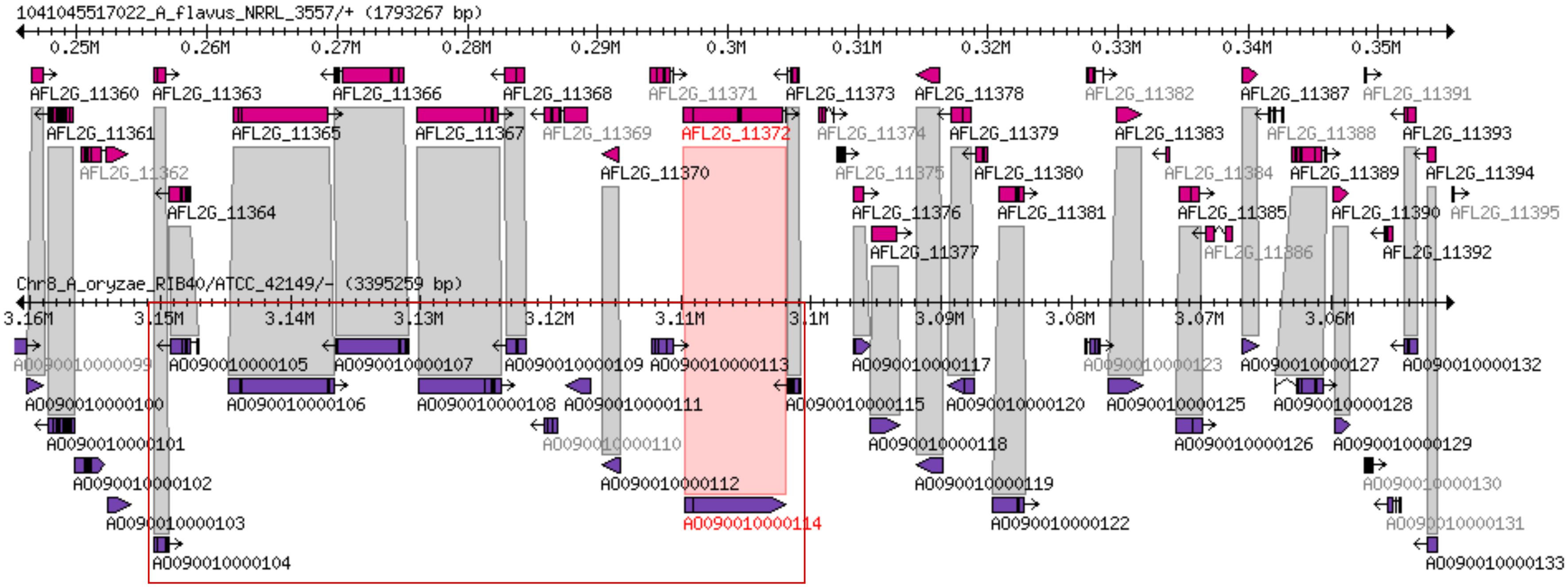

AO090010000114 cluster

Gene id (SMURF)	Gene positions	Gene distance	Gene Name	Annotated_gene_function	antiSMASH ID	AspGD ID	
AO090010000097	-17	585		Domain(s) with predicted sequence-specific DNA binding RNA polymerase II transcription factor activity, zinc ion binding activity, role in regulation of transcription, DNA-dependent and nucleus localization	n/a	AO090010000097	
AO090010000098	-16	217		Domain(s) with predicted nucleotide binding, oxidoreductase activity and role in oxidation-reduction process	n/a	AO090010000098	
AO090010000099	-15	1219			n/a	AO090010000099	
AO090010000100	-14	279		Domain(s) with predicted aspartic-type endopeptidase activity and role in proteolysis	n/a	AO090010000100	
AO090010000101	-13	487		Domain(s) with predicted role in transmembrane transport and integral to membrane localization	n/a	AO090010000101	
AO090010000102	-12	221		Ortholog of A. nidulans FGSC A4 : AN8900, A. fumigatus Af293 : Afu7g01180, A. niger CBS 513.88 : An09g02270, A. niger ATCC 1015 : 189135-mRNA and A. versicolor : Aspve1_0143716	n/a	AO090010000102	
AO090010000103	-11	2408		Ortholog(s) have homocysteine S-methyltransferase activity, role in sulfur amino acid metabolic process and cytoplasm localization	n/a	AO090010000103	
AO090010000104	-10	89		Ortholog of A. nidulans FGSC A4 : AN3379, A. fumigatus Af293 : Afu7g01170, A. niger CBS 513.88 : An12g01970, An01g00420, A. oryzae RIB40 : AO090038000140 and A. niger ATCC 1015 : 50335-mRNA	n/a	AO090010000104	ECS
AO090010000105	-9	2730		Domain(s) with predicted role in transmembrane transport and integral to membrane localization	n/a	AO090010000105	
AO090010000106	-8	392		Ortholog(s) have role in cellular response to drug, growth of symbiont in host, melanin metabolic process, spore germination	n/a	AO090010000106	
AO090010000107	-7	744		Ortholog(s) have role in secondary metabolic process	n/a	AO090010000107	
AO090010000108	-6	393		Ortholog(s) have role in fatty acid biosynthetic process, secondary metabolic process	prot_ID_1019	AO090010000108	
AO090010000109	-5	1460		Ortholog(s) have role in asperfuranone biosynthetic process, pigment biosynthetic process, sexual sporulation resulting in formation of a cellular spore	prot_ID_419	AO090010000109	
AO090010000110	-4	630			prot_ID_872	AO090010000110	
AO090010000111	-3	979		Ortholog of A. fumigatus Af293 : Afu2g09410, N. fischeri NRRL 181 : NFIA_084910, A. clavatus NRRL 1 : ACLA_081200, A. sydowii : Aspsy1_0160093 and A. fumigatus A1163 : AFUB_025270	prot_ID_410	AO090010000111	
AO090010000112	-2	2418		Domain(s) with predicted transferase activity, transferring acyl groups other than amino-acyl groups activity	prot_ID_755	AO090010000112	
AO090010000113	-1	693		Ortholog(s) have role in positive regulation of secondary metabolite biosynthetic process, secondary metabolite biosynthetic process	prot_ID_286	AO090010000113	

**AO090010000114**	0	0		Polyketide synthase	prot_ID_769	AO090010000114	
n/a				Ortholog of A. nidulans FGSC A4 : AN9052, A. oryzae RIB40 : AO090003000760, A.s flavus NRRL 3357 : AFL2T_07295, AFL2T_11373 and A. clavatus NRRL 1 : ACLA_075980	prot_ID_281	AO090010000115	ECS
n/a				Domain(s) with predicted nucleotide binding, oxidoreductase activity and role in oxidation-reduction process	prot_ID_434	AO090010000117	
n/a				Ortholog of A. nidulans FGSC A4 : AN3405, A. niger CBS 513.88 : An12g02110, A. niger ATCC 1015 : 43750-mRNA, A. versicolor : Aspve1_0592672 and A. sydowii : Aspsy1_0830408	prot_ID_522	AO090010000118	
n/a				Ortholog(s) have L-proline transmembrane transporter activity and role in gamma-aminobutyric acid transport, proline transport	prot_ID_39	AO090010000119	
n/a				Ortholog of A. nidulans FGSC A4 : AN10327, A. niger CBS 513.88 : An05g01350, A. brasiliensis : Aspbr1_0033982, A. niger ATCC 1015 : 39005-mRNA and A. flavus NRRL 3357 : AFL2T_11379	prot_ID_120	AO090010000120	
n/a			manG	Mannan endo-1,4-beta-mannosidase; transcriptionally regulated by ManR	prot_ID_367	AO090010000122	

AO090010000204 cluster

No PKS or NRPS backbone

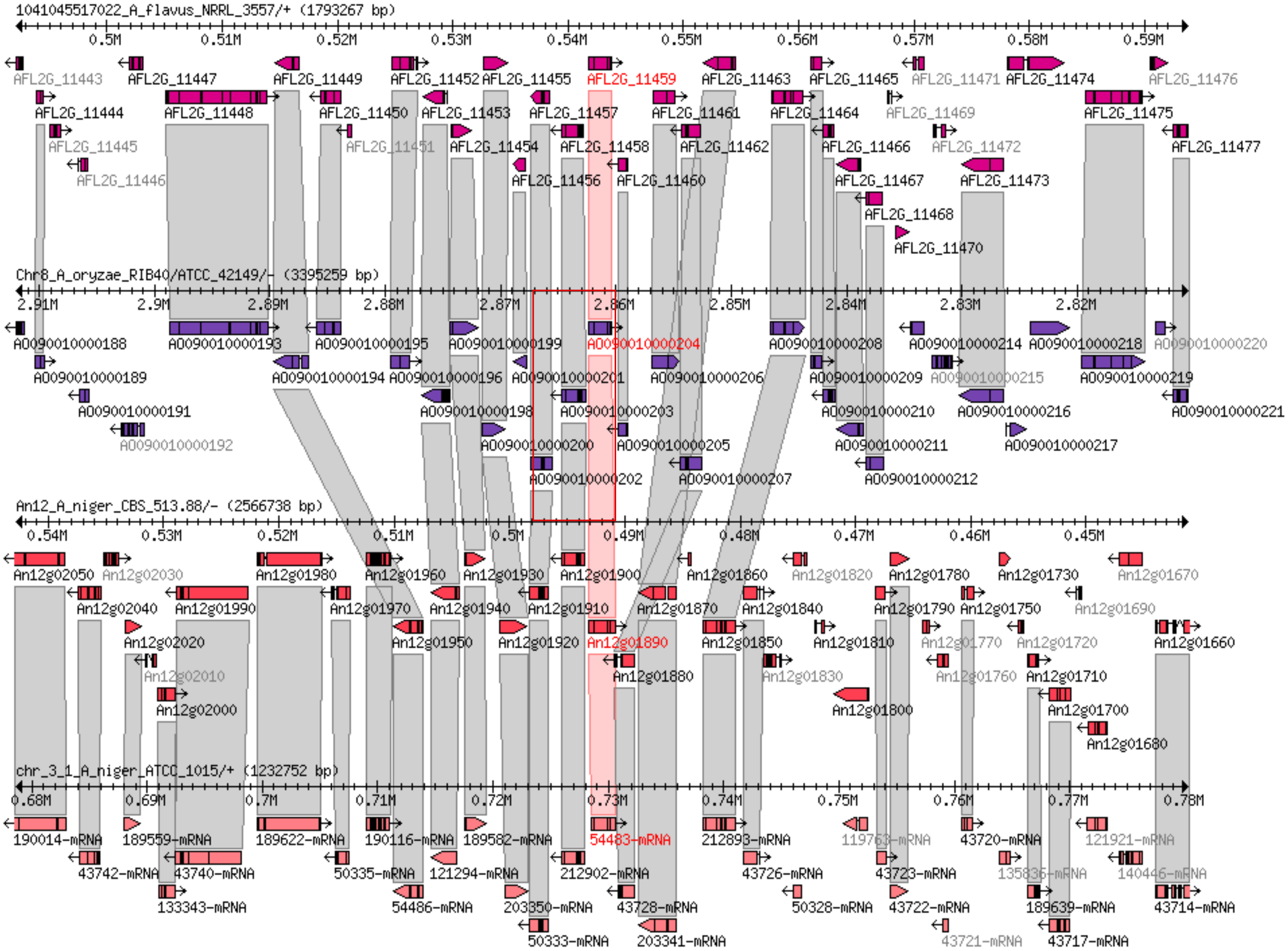

AO090010000204 cluster

Gene id (SMURF)	Gene positions	Gene distance	Gene Name	Annotated_gene_function	antiSMASH ID	AspGD ID	
n/a				Ortholog of A. nidulans FGSC A4 : AN3374, A. fumigatus Af293 : Afu7g01260, A. niger CBS 513.88 : An12g01920, A. niger ATCC 1015 : 203350-mRNA and A. versicolor : Aspve1_0127125	prot_ID_283	AO090010000200	
n/a				Ortholog of A. flavus NRRL 3357 : AFL2T_11456	prot_ID_621	AO090010000201	
n/a				Domain(s) with predicted acid phosphatase activity	prot_ID_476	AO090010000202	ECS
n/a				Domain(s) with predicted heat shock protein binding, unfolded protein binding activity and role in protein folding	prot_ID_795	AO090010000203	
n/a				Ortholog(s) have farnesyl-diphosphate farnesyltransferase activity, squalene synthase activity, role in ergosterol biosynthetic process and endoplasmic reticulum, integral to membrane, mitochondrial outer membrane localization	prot_ID_56	AO090010000204	ECS
n/a				Domain(s) with predicted ubiquitin thiolesterase activity, role in ubiquitin-dependent protein catabolic process and intracellular localization	prot_ID_530	AO090010000205	
n/a				Domain(s) with predicted solute:hydrogen antiporter activity, role in cation transport, transmembrane transport and integral to membrane	prot_ID_578	AO090010000206	
n/a				Ortholog(s) have role in GPI anchor biosynthetic process, fungal-type cell wall organization; reported to overlap the 3' end of the manR transcription factor which has not been identified in AspGD	prot_ID_347	AO090010000207	

AO090010000349 cluster

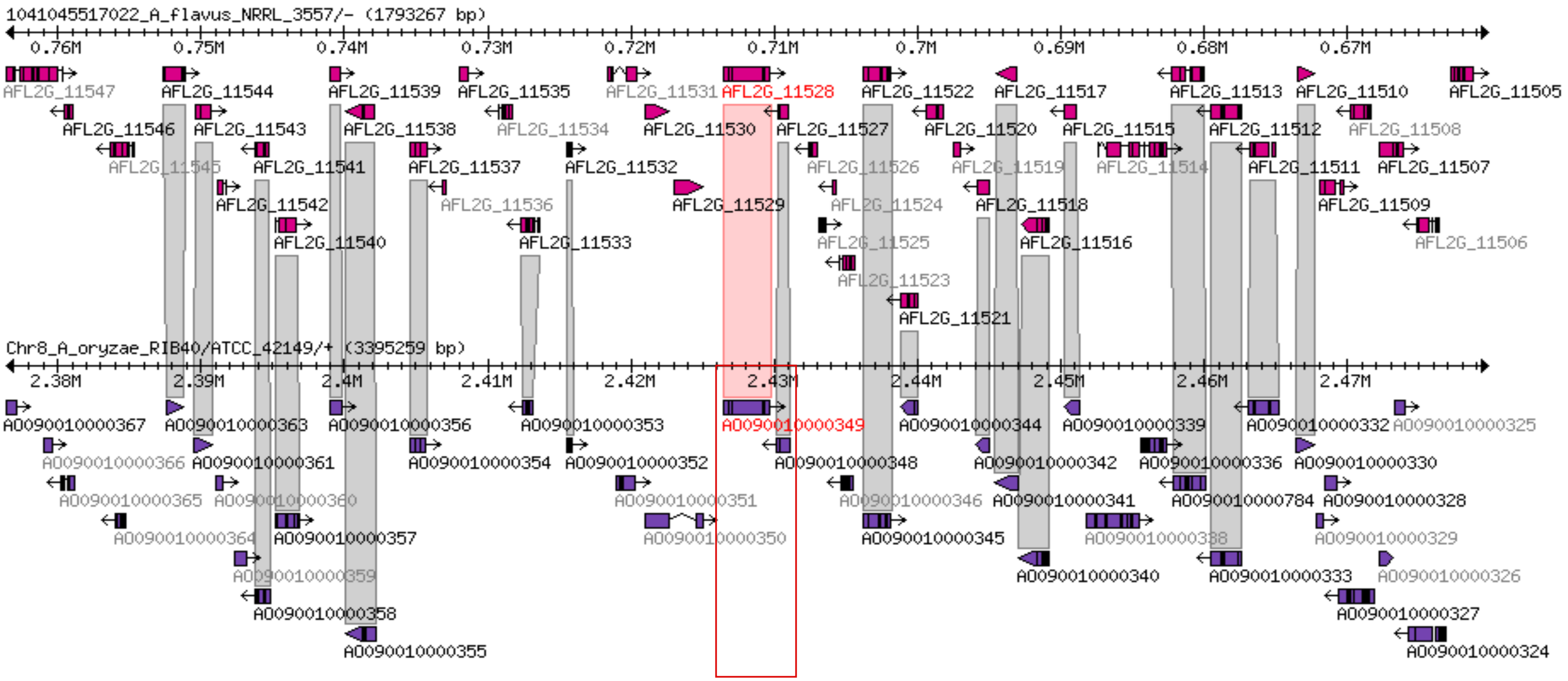

AO090010000349 cluster

Gene id (SMURF)	Gene positions	Gene distance	Gene Name	Annotated_gene_function	antiSMASH ID	AspGD ID	
n/a				Domain(s) with predicted oxidoreductase activity and role in oxidation-reduction process	prot_ID_758	AO090010000340	
n/a				Domain(s) with predicted carboxy-lyase activity, pyridoxal phosphate binding activity and role in cellular amino acid metabolic process	prot_ID_163	AO090010000341	
n/a				Domain(s) with predicted nucleotide binding activity	prot_ID_269	AO090010000342	
n/a				Ortholog of A. brasiliensis : Aspbr1_0148047, A. flavus NRRL 3357 : AFL2T_11521, A. niger ATCC 1015 : 124966-mRNA and A. carbonarius ITEM 5010 : Acar5010_010980	prot_ID_455	AO090010000344	
n/a				Domain(s) with predicted nucleobase transmembrane transporter activity, role in nucleobase transport and membrane localization	prot_ID_916	AO090010000345	
n/a				Protein of unknown function	prot_ID_21	AO090010000346	
n/a				Ortholog of Aspergillus flavus NRRL 3357 : AFL2T_11527	prot_ID_155	AO090010000348	ECS
**AO090010000349**	0	0		Domain(s) with predicted ACP phosphopantetheine attachment site binding involved in fatty acid biosynthetic process, catalytic activity, nucleotide binding, phosphopantetheine binding activity and role in metabolic process	prot_ID_645	AO090010000349	ECS
AO090010000350	1	1444		Domain(s) with predicted role in cell adhesion, transmembrane transport and integral to membrane localization	prot_ID_456	AO090010000350	
n/a				Domain(s) with predicted amino acid transmembrane transporter activity, role in amino acid transmembrane transport and membrane localization	prot_ID_903	AO090010000351	
n/a				Ortholog of A. flavus NRRL 3357 : AFL2T_11532	prot_ID_55	AO090010000352	
n/a				Domain(s) with predicted hydrolase activity, acting on carbon-nitrogen (but not peptide) bonds, in linear amides activity, role in ceramide metabolic process and integral to membrane localization	prot_ID_1045	AO090010000353	

AO090010000404 cluster

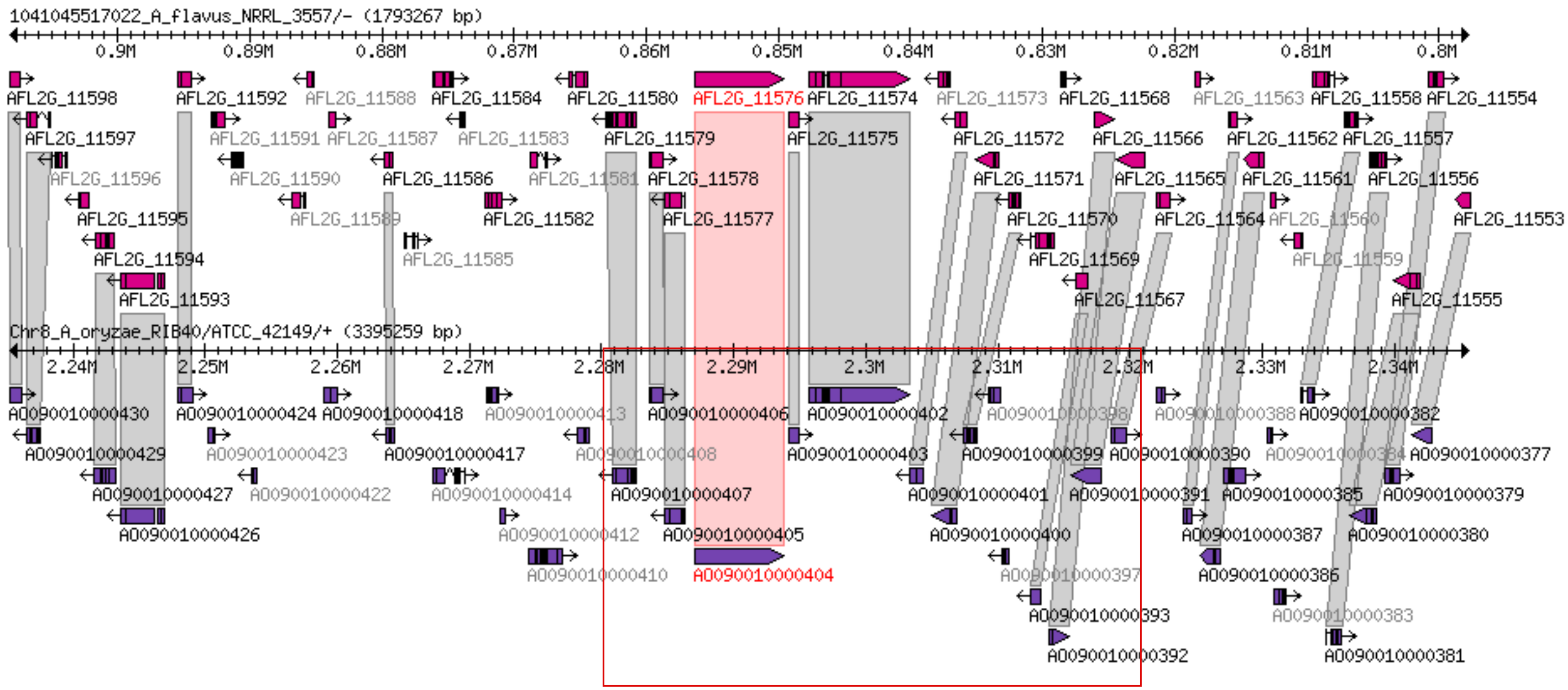

AO090010000404 cluster

Gene id (SMURF)	Gene positions	Gene distance	Gene Name	Annotated_gene_function	antiSMASH ID	AspGD ID	
n/a					prot_ID_568	AO090010000388	
AO090010000390	-11	1043		Domain(s) with predicted nucleotide binding, oxidoreductase activity and role in oxidation-reduction process	prot_ID_495	AO090010000390	ECS, FA
AO090010000391	-10	175		Domain(s) with predicted DNA binding, zinc ion binding activity, role in transcription, DNA-dependent and nucleus localization	prot_ID_820	AO090010000391	
AO090010000392	-9	693		Domain(s) with predicted role in transmembrane transport and integral to membrane localization	prot_ID_651	AO090010000392	
AO090010000393	-8	1711		Domain(s) with predicted carbon-carbon lyase activity and role in cellular aromatic compound metabolic process	prot_ID_960	AO090010000393	
AO090010000397	-7	162		Protein of unknown function	prot_ID_264	AO090010000397	
AO090010000398	-6	969		Domain(s) with predicted oxidoreductase activity, acting on paired donors, with incorporation or reduction of molecular oxygen, 2-oxoglutarate as one donor, and incorporation of one atom each of oxygen into both donors activity	prot_ID_329	AO090010000398	
AO090010000399	-5	825		Domain(s) with predicted ribosome binding, translation elongation factor activity	prot_ID_919	AO090010000399	
AO090010000400	-4	880		Domain(s) with predicted L-ascorbic acid binding, iron ion binding, oxidoreductase activity, acting on paired donors, with incorporation or reduction of molecular oxygen and 2-oxoglutarate as one donor, more	prot_ID_366	AO090010000400	
AO090010000401	-3	51		Ortholog of A. nidulans FGSC A4 : AN2942, AN10320, AN7130, A. fumigatus Af293 : Afu3g15110 and A. niger CBS 513.88 : An03g05620, An15g07840, An12g07260, An03g02580, An01g14840	prot_ID_830	AO090010000401	
AO090010000402	-2	807		Domain(s) with predicted ACP phosphopantetheine attachment site binding involved in fatty acid biosynthetic process, nucleotide binding, oxidoreductase activity and transferase activity, more	prot_ID_537	AO090010000402	
AO090010000403	-1	317		Ortholog(s) have role in asperfuranone biosynthetic process	prot_ID_1034	AO090010000403	
**AO090010000404**	0	0		Polyketide synthase	prot_ID_677	AO090010000404	
AO090010000405	1	723		Ortholog(s) have role in fumiquinazoline A biosynthetic process, monodictyphenone biosynthetic process	prot_ID_50	AO090010000405	
AO090010000406	2	84		Mitochondrial carrier protein	prot_ID_626	AO090010000406	
AO090010000407	3	988		Domain(s) with predicted role in transmembrane transport and integral to membrane localization	prot_ID_10	AO090010000407	ECS
n/a				Protein of unknown function	prot_ID_328	AO090010000408	
n/a				Protein of unknown function	prot_ID_507	AO090010000410	
n/a				Protein of unknown function	prot_ID_523	AO090010000412	
n/a				Protein of unknown function	prot_ID_971	AO090010000413	

n/a	Protein of unknown function	prot_ID_402	AO090010000414

AO090010000426 cluster

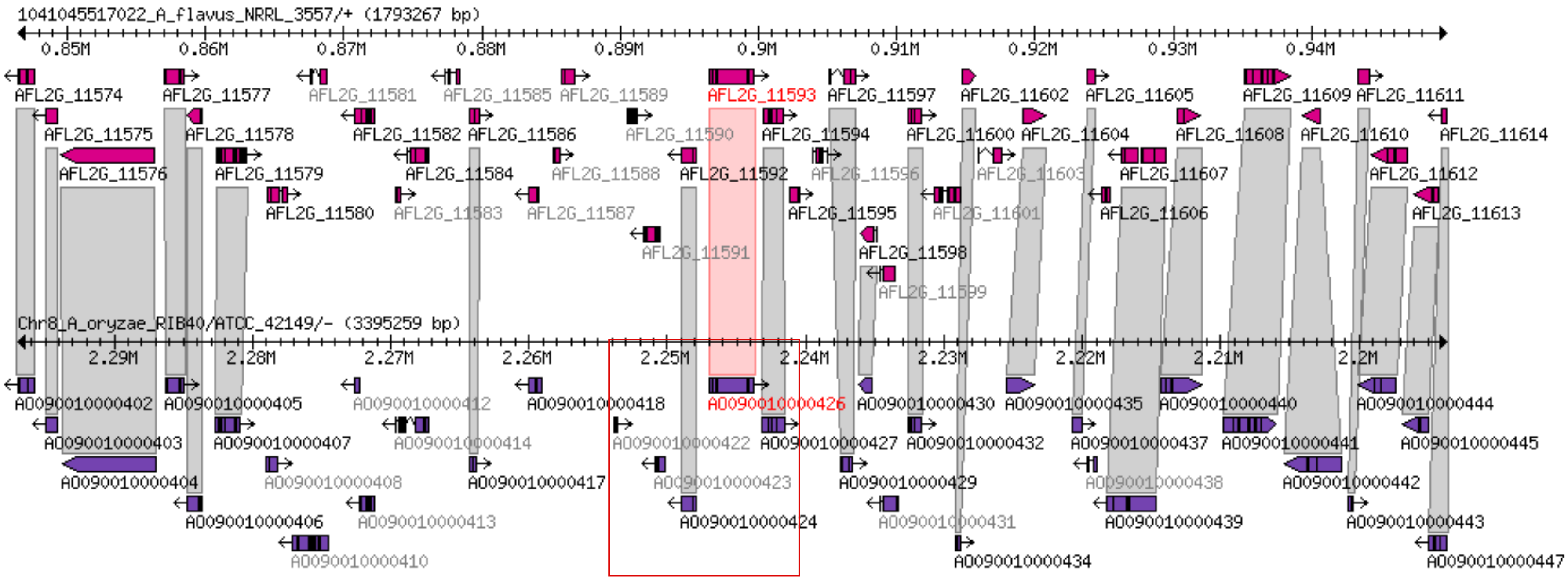

AO090010000426 cluster

Gene id (SMURF)	Gene positions	Gene distance	Gene Name	Annotated_gene_function	antiSMASH ID	AspGD ID	
n/a				Domain(s) with predicted role in cell wall macromolecule catabolic process	prot_ID_692	AO090010000417	
n/a				Ortholog of A. nidulans FGSC A4 : AN3529, AN4144, AN8311, AN4846, A. fumigatus Af293 : Afu1g17260, Afu4g07950, Afu4g09380, Afu6g00560, Afu6g12140, Afu8g01900 and A. niger CBS 513.88 : An16g06170, An06g00470, An11g07840	prot_ID_978	AO090010000418	
n/a				Protein of unknown function	prot_ID_415	AO090010000422	IGD
n/a				Domain(s) with predicted DNA binding, zinc ion binding activity, role in transcription, DNA-dependent and nucleus localization	prot_ID_1000	AO090010000423	
AO090010000424	-1	941		Domain(s) with predicted catalytic activity, coenzyme binding, nucleotide binding activity and role in cellular metabolic process	prot_ID_805	AO090010000424	
**AO090010000426**	0	0		Domain(s) with predicted ACP phosphopantetheine attachment site binding involved in fatty acid biosynthetic process, catalytic activity, nucleotide binding, phosphopantetheine binding activity and role in metabolic process	prot_ID_910	AO090010000426	
n/a				Beta-lactamase	prot_ID_176	AO090010000427	IGD, ECS
n/a				Domain(s) with predicted nucleotide binding, oxidoreductase activity and role in oxidation-reduction process	prot_ID_278	AO090010000429	
n/a				Ortholog of A. flavus NRRL 3357 : AFL2T_11598	prot_ID_465	AO090010000430	
n/a				Protein of unknown function	prot_ID_1053	AO090010000431	
n/a				Domain(s) with predicted role in response to stress and integral to membrane localization (2)	prot_ID_315	AO090010000432	
n/a				Ortholog of A. flavus NRRL 3357 : AFL2T_11602	prot_ID_232	AO090010000434	
n/a				Ortholog of A. nidulans FGSC A4 : AN0213, A. fumigatus Af293 : Afu5g10950, A. niger CBS 513.88 : An14g02320, A. niger ATCC 1015 : 201495-mRNA and A. versicolor : Aspve1_0048464	prot_ID_813	AO090010000435	

AO090010000498 cluster

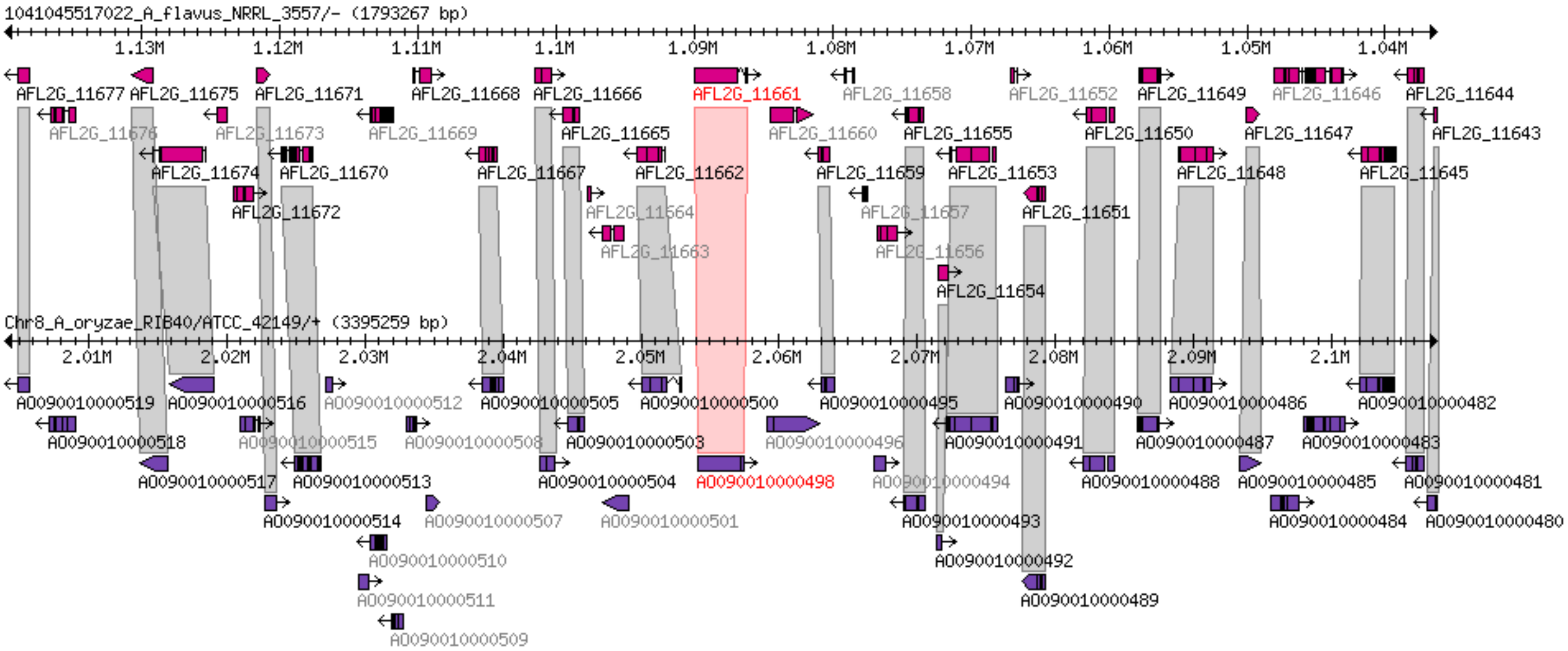

AO090010000498 cluster

Gene id (SMURF)	Gene positions	Gene distance	Gene Name	Annotated_gene_function	antiSMASH ID	AspGD ID
AO090010000488	-9	2966		Domain(s) with predicted substrate-specific transmembrane transporter activity, role in transmembrane transport and integral to membrane localization	n/a	AO090010000488
AO090010000489	-8	127		Ortholog(s) have URM1 activating enzyme activity, nitrate reductase (NADPH) activity, protein adenylyltransferase activity, thiosulfate sulfurtransferase activity	n/a	AO090010000489
AO090010000490	-7	1009		Ortholog of A. fumigatus Af293 : Afu5g10470, N. fischeri NRRL 181 : NFIA_076780, A. clavatus NRRL 1 : ACLA_013500, A. terreus NIH2624 : ATET_07599 and A. fumigatus A1163 : AFUB_058060	prot_ID_542	AO090010000490
AO090010000491	-6	278		Ortholog of A. nidulans FGSC A4 : AN10292, AN7172, A. fumigatus Af293 : Afu5g10460, A. niger CBS 513.88 : An14g02030, An14g04320, A. oryzae RIB40 : AO090011000125 and A. niger ATCC 1015 : 41626-mRNA	prot_ID_992	AO090010000491
AO090010000492	-5	1097		Ortholog of A. nidulans FGSC A4 : AN7961, A. fumigatus Af293 : Afu4g13760, A. versicolor : Aspve1_0045462, A. sydowii : Aspsy1_1151790 and A. terreus NIH2624 : ATET_04942	prot_ID_384	AO090010000492
AO090010000493	-4	1426		Neutral protease II; expression increased in MAT1-1 strain compared to MAT1-2 strain	prot_ID_763	AO090010000493
AO090010000494	-3	2943		Domain(s) with predicted hydrolase activity, hydrolyzing O-glycosyl compounds activity and role in carbohydrate metabolic process	prot_ID_171	AO090010000494
AO090010000495	-2	348		Domain(s) with predicted N-acetyltransferase activity and role in metabolic process	prot_ID_690	AO090010000495
AO090010000496	-1	1861		Histidine kinase-like ATPase, two-component response regulator	prot_ID_90	AO090010000496
**AO090010000498**	0	0		Non-ribosomal peptide synthetase	prot_ID_1068	AO090010000498
AO090010000500	1	1307		Domain(s) with predicted role in transmembrane transport and integral to membrane localization	prot_ID_37	AO090010000500
n/a				Protein of unknown function	prot_ID_660	AO090010000501
n/a				Ortholog(s) have role in cleistothecium development, negative regulation of cleistothecium development	prot_ID_502	AO090010000503
n/a			pel1	Pectin lyase with role in pectin degradation	prot_ID_811	AO090010000504
n/a			sdhB	Succinate dehydrogenase (SDH); mutation confers resistance to the fungicide, carboxin, which interferes with electron transfer from SDH to ubiquinone	prot_ID_345	AO090010000505
n/a				Protein of unknown function	prot_ID_319	AO090010000507

AO090011000015 cluster

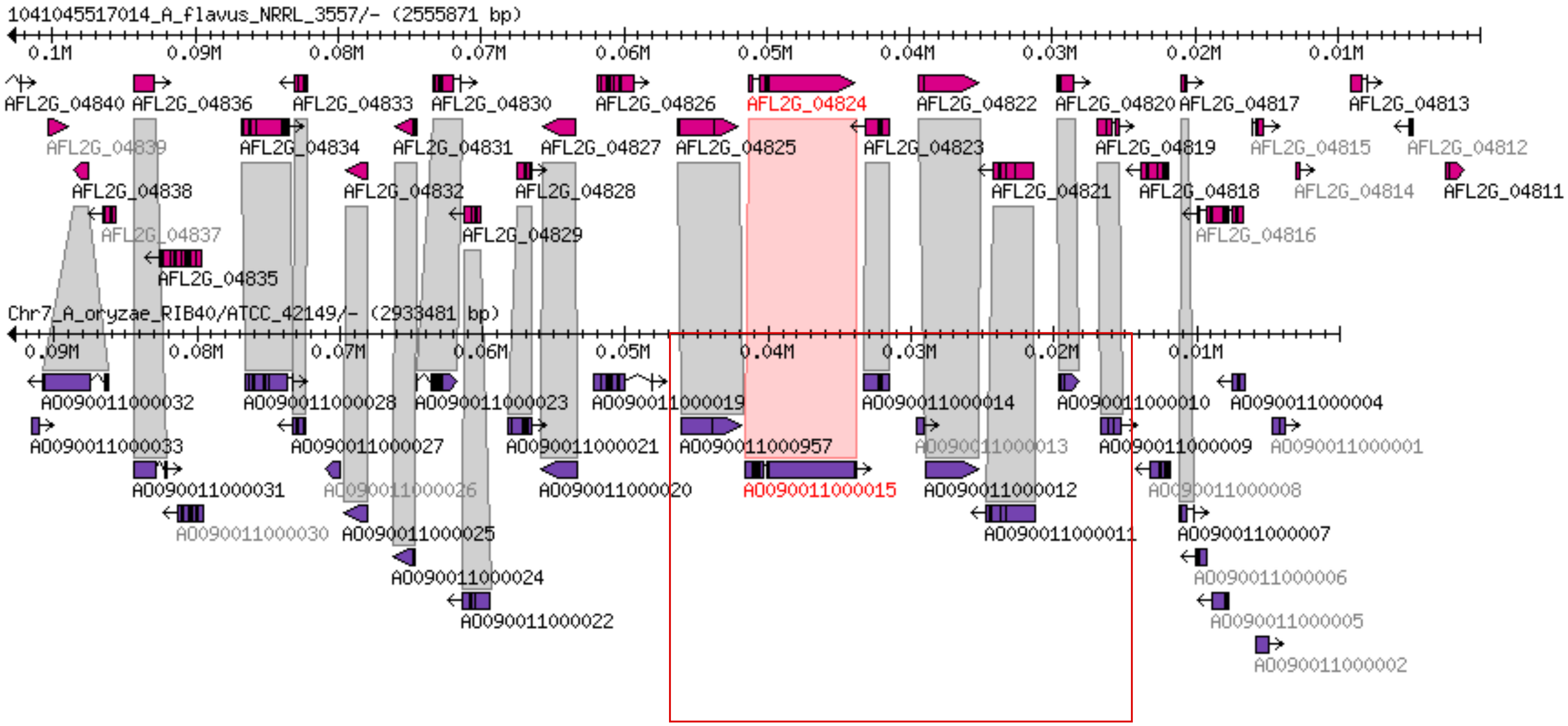

AO090011000015 cluster

Gene id (SMURF)	Gene positions	Gene distance	Gene Name	Annotated_gene_function	antiSMASH ID	AspGD ID	
AO090011000023	-6	492		Domain(s) with predicted role in transmembrane transport and integral to membrane localization	n/a		
AO090011000022	-5	2808		Ortholog(s) have alternative oxidase activity, role in alternative respiration, cellular response to oxidative stress and mitochondrion localization	prot_ID_170	AO090011000022	
AO090011000021	-4	686		Ortholog of A. fumigatus Af293 : Afu1g17050, Afu3g01470, A. niger CBS 513.88 : An03g01670, An10g00970, An13g03140, A. oryzae RIB40 : AO090026000122 and A. niger ATCC 1015 : 194603-mRNA, 45030-mRNA	prot_ID_271	AO090011000021	
AO090011000020	-3	1552		Domain(s) with predicted role in cell adhesion	prot_ID_758	AO090011000020	
AO090011000019	-2	1963		Putative cysteine synthase B	prot_ID_753	AO090011000019	
AO090011000957	-1	668		Domain(s) with predicted ATP binding, ATPase activity, coupled to transmembrane movement of substances activity, role in transmembrane transport and integral to membrane localization	prot_ID_898	AO090011000957	ECS
**AO090011000015**	0	0		Polyketide synthase	prot_ID_52	AO090011000015	
AO090011000014	1	519		Domain(s) with predicted role in transmembrane transport and integral to membrane localization	prot_ID_447	AO090011000014	
AO090011000013	2	2048		Protein of unknown function	prot_ID_879	AO090011000013	
AO090011000012	3	217		Ortholog of A. nidulans FGSC A4 : AN3342, AN10302, AN4609, A. fumigatus Af293 : Afu7g06670, A. niger CBS 513.88 : An04g04320, An16g05670, An04g03250 and A. niger ATCC 1015 : 190482-mRNA, 36572-mRNA, 44094-mRNA	prot_ID_357	AO090011000012	
AO090011000011	4	998		Domain(s) with predicted GTP binding, GTPase activity	prot_ID_390	AO090011000011	
AO090011000010	5	1825		Ortholog of A. fumigatus Af293 : Afu6g11720, A. niger CBS 513.88 : An08g04630, A. brasiliensis : Aspbr1_0028913, A. niger ATCC 1015 : 126535-mRNA and N. fischeri NRRL 181 : NFIA_057390	prot_ID_779	AO090011000010	
AO090011000009	6	1612		Domain(s) with predicted cysteine dioxygenase activity, iron ion binding activity and role in L-cysteine metabolic process, oxidation-reduction process	prot_ID_255	AO090011000009	ECS
AO090011000008	7	2059		Domain(s) with predicted role in transmembrane transport and integral to membrane localization	n/a	AO090011000008	
AO090011000007	8	854		Ortholog of A. nidulans FGSC A4 : AN9185, AN12413, A. brasiliensis : Aspbr1_0035352, A. niger ATCC 1015 : 55148-mRNA and A. flavus NRRL 3357 : AFL2T_04817	n/a	AO090011000007	
AO090011000006	9	90		Protein of unknown function	n/a	AO090011000006	
AO090011000005	10	435		Domain(s) with predicted sequence-specific DNA binding RNA polymerase II transcription factor activity, zinc ion binding activity, role in regulation of transcription, DNA-dependent and nucleus localization	n/a	AO090011000005	

AO090011000004	11	330	Domain(s) with predicted nucleotide binding, oxidoreductase activity and role in oxidation-reduction process	n/a	AO090011000004
n/a			Protein of unknown function	n/a	AO090011000002

AO090011000043 cluster

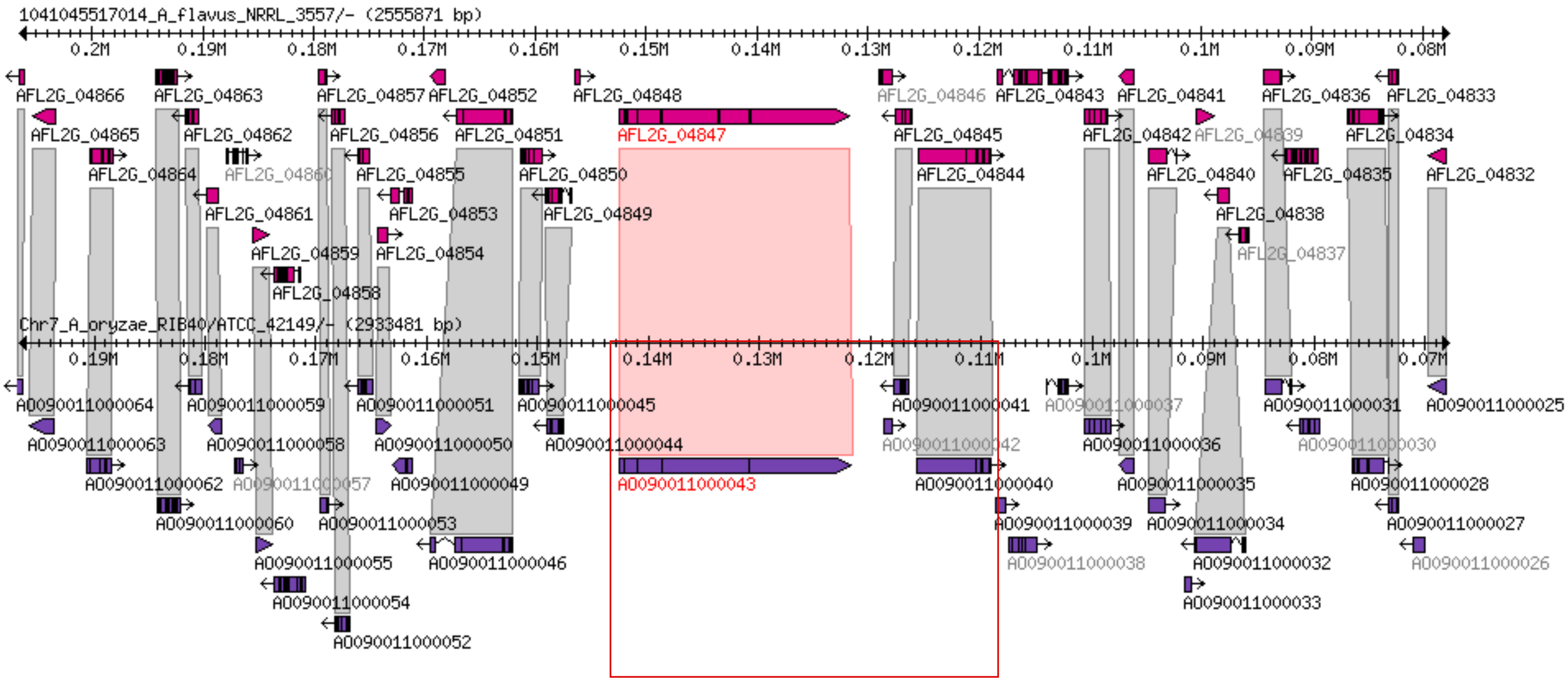

AO090011000043 cluster

Gene id (SMURF)	Gene positions	Gene distance	Gene Name	Annotated_gene_function	antiSMASH ID	AspGD ID	
n/a				Domain(s) with predicted transporter activity, role in transmembrane transport and integral to membrane localization	prot_ID_454	AO090011000049	
n/a				Domain(s) with predicted 3-oxoacyl-[acyl-carrier-protein] reductase (NADPH) activity, aspartic-type endopeptidase activity, fatty-acyl-CoA synthase activity, nucleotide binding activity	prot_ID_706	AO090011000046	
n/a			CYP548H1	Cytochrome P450 monooxygenase	prot_ID_561	AO090011000045	
n/a				Domain(s) with predicted branched-chain-amino-acid transaminase activity and role in branched-chain amino acid metabolic process	prot_ID_183	AO090011000044	
**AO090011000043**	0	0		Non-ribosomal peptide synthetase	prot_ID_2	AO090011000043	ECS
AO090011000042	1	3078		Protein of unknown function	prot_ID_490	AO090011000042	
AO090011000041	2	387		Domain(s) with predicted role in response to stress and integral to membrane localization	prot_ID_370	AO090011000041	
AO090011000040	3	911		Domain(s) with predicted enoyl-[acyl-carrier-protein] reductase (NADH) activity, role in fatty acid biosynthetic process, oxidation-reduction process and fatty acid synthase complex localization	prot_ID_884	AO090011000040	ECS
AO090011000039	4	503		Ortholog of A. nidulans FGSC A4 : AN7874, AN10158, A. fumigatus Af293 : Afu1g12200, Afu3g00570, Afu8g02315, A. niger CBS 513.88 : An08g04520, An06g00280 and A. oryzae RIB40 : AO090001000324, AO090010000032	prot_ID_785	AO090011000039	
AO090011000038	5	402		Domain(s) with predicted ATP binding, ATPase activity, coupled to transmembrane movement of substances activity, role in transmembrane transport and integral to membrane localization	prot_ID_398	AO090011000038	
AO090011000037	6	788		Domain(s) with predicted ATP binding, ATPase activity	prot_ID_654	AO090011000037	
AO090011000036	7	1334		Neutral protease I	n/a	AO090011000036	
AO090011000035	8	909		Ortholog of A. nidulans FGSC A4 : AN3259, A. flavus NRRL 3357 : AFL2T_04841, A. versicolor : Aspve1_0040331 and A. sydowii : Aspsy1_0029969	n/a	AO090011000035	
AO090011000034	9	1270		Domain(s) with predicted amino acid transmembrane transporter activity, role in amino acid transmembrane transport and membrane localization	n/a	AO090011000034	
AO090011000033	10	1704		Ortholog of N. fischeri NRRL 181 : NFIA_108740 and A. carbonarius ITEM 5010 : Acar5010_010490	n/a	AO090011000033	
AO090011000032	11	2406		Domain(s) with predicted metal ion transmembrane transporter activity, role in metal ion transport, transmembrane transport and membrane localization	n/a	AO090011000032	

AO090011000031	12	1854	Domain(s) with predicted amino acid transmembrane transporter activity, role in amino acid transmembrane transport and membrane localization	n/a	AO090011000031

AO090011000103 cluster

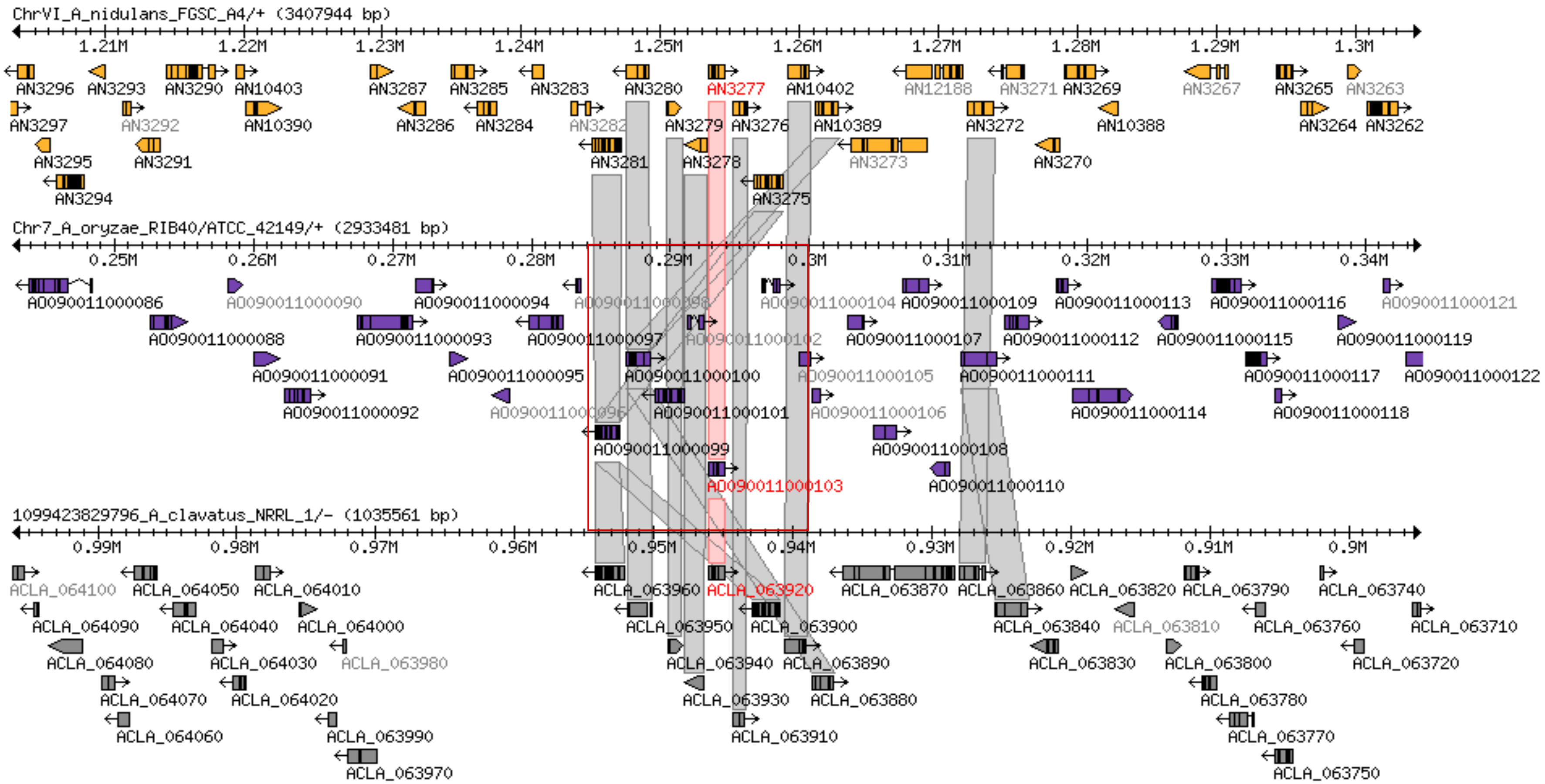

AO090011000103 cluster

Gene id (SMURF)	Gene positions	Gene distance	Gene Name	Annotated_gene_function	antiSMASH ID	AspGD ID	
n/a				Protein of unknown function	prot_ID_814	AO090011000098	
n/a			CYP567D2	Cytochrome P450 monooxygenase	prot_ID_410	AO090011000099	ECS
n/a			CYP654B1	Cytochrome P450 monooxygenase	prot_ID_414	AO090011000100	
n/a			CYP682B2	Cytochrome P450 monooxygenase	prot_ID_448	AO090011000101	
n/a				Domain(s) with predicted nucleotide binding activity	prot_ID_109	AO090011000102	
n/a				Domain(s) with predicted magnesium ion binding, terpene synthase activity	prot_ID_440	AO090011000103	
n/a				Domain(s) with predicted hydrolase activity and role in metabolic process	prot_ID_49	AO090011000104	
n/a				Protein of unknown function	prot_ID_572	AO090011000105	ECS
n/a				Protein of unknown function	prot_ID_187	AO090011000106	
n/a				Ortholog of Aspergillus flavus NRRL 3357 : AFL2T_04903, A. versicolor : Aspve1_0031646 and A. sydowii : Aspsy1_0035800	prot_ID_420	AO090011000107	

AO090011000328 cluster

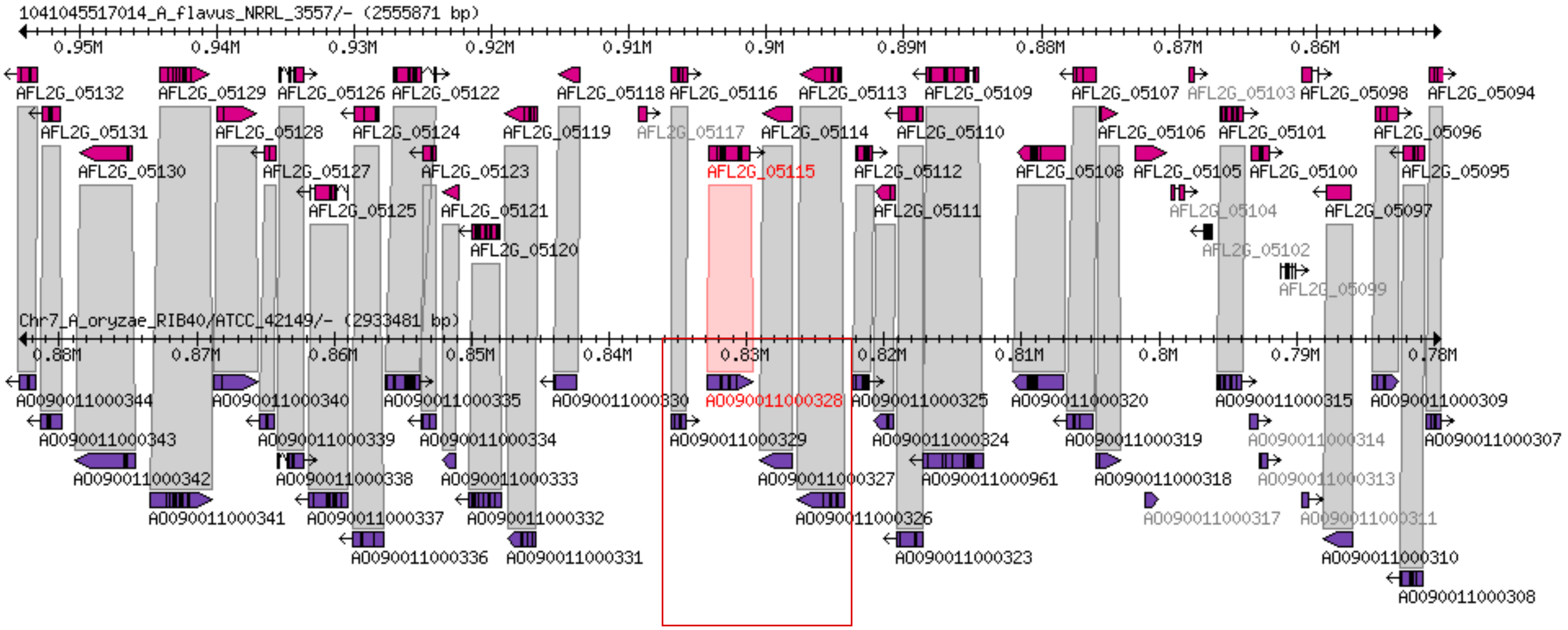

AO090011000328 cluster

Gene id (SMURF)	Gene positions	Gene distance	Gene Name	Annotated_gene_function	antiSMASH ID	AspGD ID	
n/a				Domain(s) with predicted hydrolase activity and role in metabolic process	prot_ID_500	AO090011000333	
n/a				Domain(s) with predicted N,N-dimethylaniline monooxygenase activity, NADP binding, flavin adenine dinucleotide binding activity and role in oxidation-reduction process	prot_ID_143	AO090011000332	
n/a				Ortholog(s) have role in ferric triacetylfusarinine C transport	prot_ID_165	AO090011000331	
n/a				Domain(s) with predicted N,N-dimethylaniline monooxygenase activity, NADP binding, flavin adenine dinucleotide binding activity and role in oxidation-reduction process	prot_ID_653	AO090011000330	
n/a				Ortholog(s) have water channel activity, role in ascospore formation, water transport and plasma membrane localization	prot_ID_608	AO090011000329	
**AO090011000328**	0	0		Protein similar to nonribosomal peptide synthases (NRPS-like)	prot_ID_251	AO090011000328	IGD, ECS
AO090011000327	1	504		Domain(s) with predicted zinc ion binding activity	prot_ID_496	AO090011000327	
AO090011000326	2	677		Ortholog(s) have 3-hydroxyacyl-CoA dehydrogenase activity, enoyl-CoA hydratase activity, role in fatty acid beta-oxidation, very long-chain fatty acid catabolic process and peroxisome localization	prot_ID_144	AO090011000326	ECG, IGD
AO090011000325	3	1022		Ortholog(s) have ATP binding activity and cytoplasm, nucleus localization	prot_ID_332	AO090011000325	
AO090011000324	4	441		Ortholog of A. nidulans FGSC A4 : AN7052, A. fumigatus Af293 : Afu4g03945, A. niger CBS 513.88 : An14g00940 and A. terreus NIH2624 : ATET_02247	prot_ID_768	AO090011000324	
AO090011000323	5	630		Ortholog(s) have cystathionine beta-lyase activity, role in 'de novo' L-methionine biosynthetic process, transsulfuration and peroxisome localization	prot_ID_269	AO090011000323	
AO090011000961	6	525		Cutinase transcription factor 1 alpha	prot_ID_896	AO090011000961	
n/a				Ortholog(s) have mannose-ethanolamine phosphotransferase activity, role in ATP transport, GPI anchor biosynthetic process and endoplasmic reticulum, fungal-type cell wall, fungal-type vacuole localization	prot_ID_86	AO090011000320	

AO090011000408 cluster
No prediction made

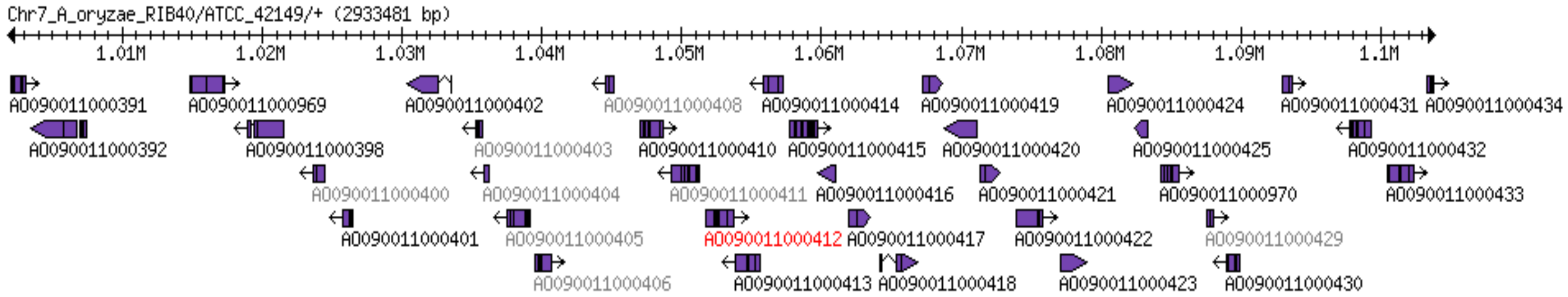

AO090011000408 cluster

Gene id (SMURF)	Gene positions	Gene distance	Gene Name	Annotated_gene_function	antiSMASH ID	AspGD ID
n/a				Protein of unknown function	prot_ID_316	AO090011000403
n/a				Protein of unknown function	prot_ID_722	AO090011000404
n/a				Protein of unknown function	prot_ID_406	AO090011000405
n/a				Domain(s) with predicted role in response to stress and integral to membrane localization	prot_ID_809	AO090011000406
n/a				Domain(s) with predicted magnesium ion binding, terpene synthase activity	prot_ID_200	AO090011000408
n/a				Domain(s) with predicted electron carrier activity, heme binding, iron ion binding, oxidoreductase activity, acting on paired donors, with incorporation or reduction of molecular oxygen activity and role in oxidation-reduction process	prot_ID_77	AO090011000410
n/a			CYP5117A1	Cytochrome P450 monooxygenase; upregulated by exposure to benomyl	prot_ID_552	AO090011000411
n/a			CYP68Q1	Cytochrome P450 monooxygenase; involved in 7-hydroxycoumarin production and in diclofenac conversion; upregulated by exposure to benomyl	prot_ID_195	AO090011000412
n/a				Domain(s) with predicted role in transmembrane transport and integral to membrane localization	prot_ID_122	AO090011000413

AO090011000738 cluster

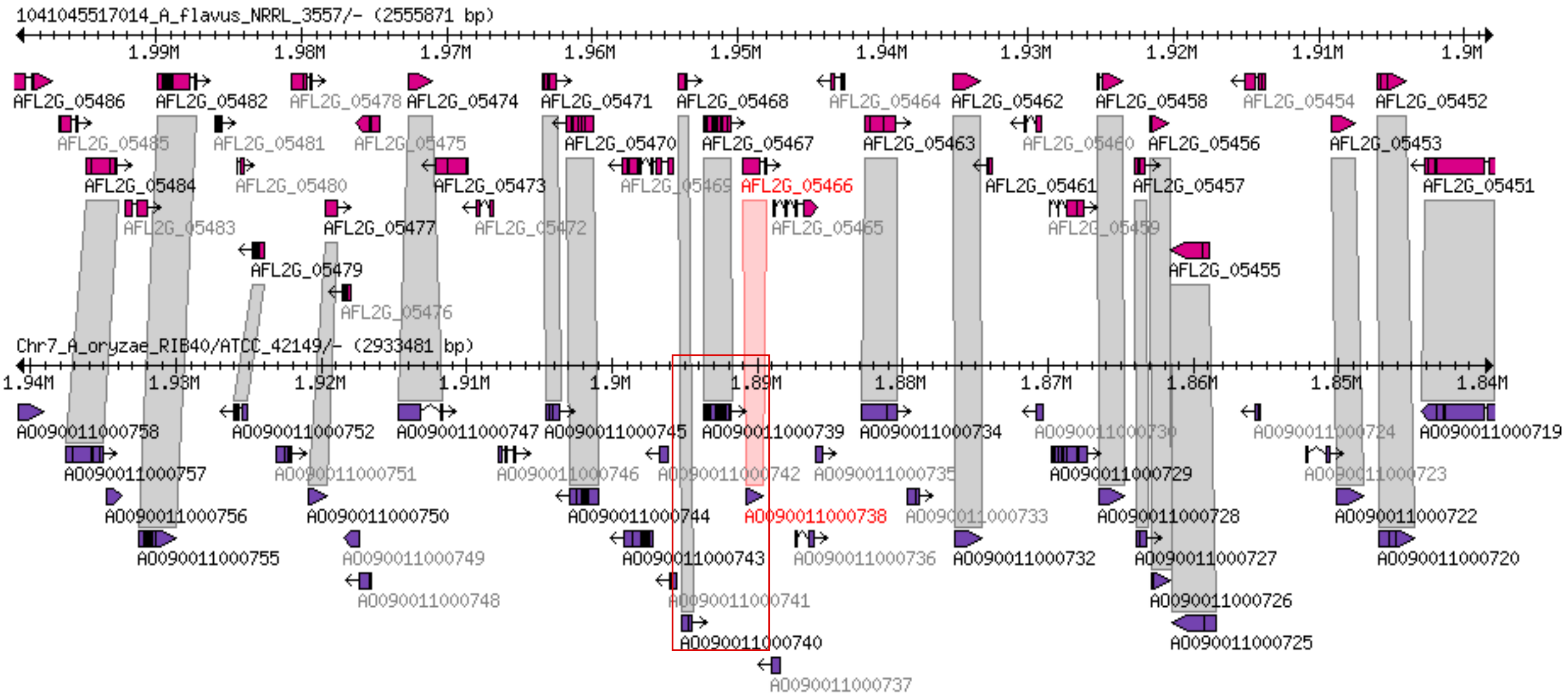

AO090011000738 cluster

Gene id (SMURF)	Gene positions	Gene distance	Gene Name	Annotated_gene_function	antiSMASH ID	AspGD ID	
AO090011000744	-6	1992		Domain(s) with predicted role in transmembrane transport and integral to membrane localization	n/a	AO090011000744	
AO090011000743	-5	477		Cytochrome P450 monooxygenase; expression increased in MAT1-1 strain compared to MAT1-2 strain	prot_ID_13	AO090011000743	
AO090011000742	-4	80		Protein of unknown function	prot_ID_502	AO090011000742	
AO090011000741	-3	363		Protein of unknown function	prot_ID_366	AO090011000741	
AO090011000740	-2	731		Ortholog(s) have role in austinol biosynthetic process, dehydroaustinol biosynthetic process	prot_ID_868	AO090011000740	ECS, FA
AO090011000739	-1	887		Domain(s) with predicted electron carrier activity, heme binding, iron ion binding, oxidoreductase activity, acting on paired donors, with incorporation or reduction of molecular oxygen activity and role in oxidation-reduction process	prot_ID_436	AO090011000739	
**AO090011000738**	0	0		Ortholog(s) have prenyltransferase activity and role in sterigmatocystin biosynthetic process, terrequinone A biosynthetic process	prot_ID_57	AO090011000738	ECS, FA
AO090011000737	1	559		Protein of unknown function	prot_ID_556	AO090011000737	
AO090011000736	2	1130		Protein of unknown function	prot_ID_193	AO090011000736	
AO090011000735	3	127		Domain(s) with predicted ATP binding, helicase activity, role in rRNA processing, snRNA processing, snoRNA processing, tRNA processing, termination of RNA polymerase II transcription and nucleus localization	prot_ID_125	AO090011000735	
AO090011000734	4	3111		Na+/K+ transporter	prot_ID_620	AO090011000734	
AO090011000733	5	668		Protein of unknown function	prot_ID_411	AO090011000733	
AO090011000732	6	2465		Domain(s) with predicted D-amino-acid oxidase activity, nucleotide binding activity and role in oxidation-reduction process	n/a	AO090011000732	
AO090011000730	7	3716		Protein of unknown function	n/a	AO090011000730	
AO090011000729	8	579		Domain(s) with predicted DNA binding, sequence-specific DNA binding RNA polymerase II transcription factor activity, zinc ion binding activity and role in regulation of transcription, DNA-dependent, transcription, DNA-dependent	n/a	AO090011000729	

AO090012001034 cluster

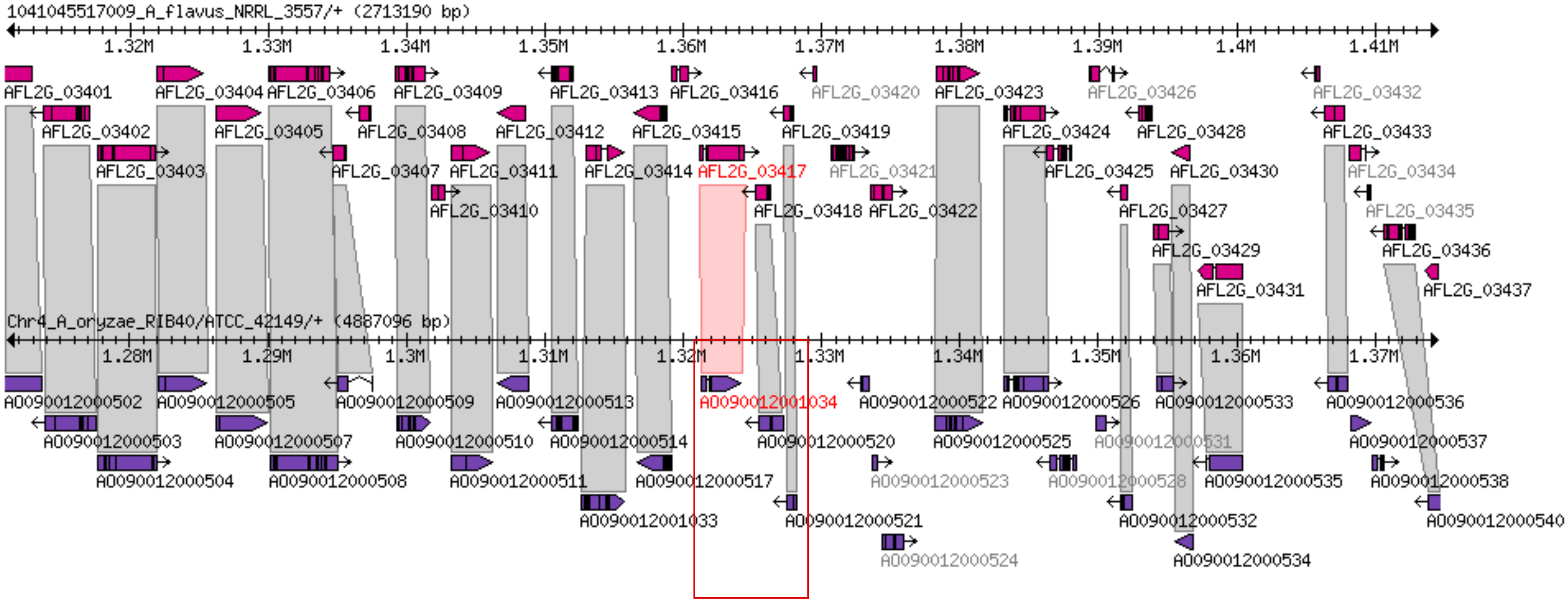

AO090012001034 cluster

Gene id (SMURF)	Gene positions	Gene distance	Gene Name	Annotated_gene_function	antiSMASH ID	AspGD ID	
n/a				Ortholog(s) have transketolase activity, role in pentose-phosphate shunt and intracellular localization	prot_ID_34	AO090012000526	
n/a			facB	Sequence-specific DNA binding transcription factor	prot_ID_853	AO090012000525	
n/a				Protein of unknown function	prot_ID_921	AO090012000524	
n/a				Protein of unknown function	prot_ID_288	AO090012000523	
n/a				Ortholog of A. nidulans FGSC A4 : AN0691, A. fumigatus Af293 : Afu1g13530, A. niger CBS 513.88 : An08g06600, A. versicolor : Aspve1_0036230 and A. sydowii : Aspsy1_0041485	prot_ID_534	AO090012000522	
n/a				Ortholog(s) have intracellular localization	prot_ID_238	AO090012000521	ECS, IGD
n/a				Ortholog(s) have intracellular localization	prot_ID_883	AO090012000520	
**AO090012001034**	0	0		Domain(s) with predicted ACP phosphopantetheine attachment site binding involved in fatty acid biosynthetic process, catalytic activity, nucleotide binding, phosphopantetheine binding activity and role in metabolic process	prot_ID_1493	AO090012001034	ECS, IGD
AO090012000517	1	2395		Ortholog(s) have role in mitotic cell cycle G2/M transition decatenation checkpoint, mitotic cell cycle spindle assembly checkpoint, nucleocytoplasmic transport and kinetochore, nuclear pore, nucleoplasm localization	prot_ID_1399	AO090012000517	
AO090012001033	2	798		Ortholog(s) have role in actin cortical patch assembly, septin cytoskeleton organization and cellular bud neck, cellular bud tip, mating projection base, mating projection tip localization	prot_ID_1489	AO090012001033	
AO090012000514	3	523		Domain(s) with predicted ATP binding, protein serine/threonine kinase activity, protein tyrosine kinase activity and role in protein phosphorylation	prot_ID_887	AO090012000514	
AO090012000513	4	2010		Ortholog of A. nidulans FGSC A4 : AN0698, A. fumigatus Af293 : Afu1g13610, A. niger CBS 513.88 : An08g06690, A. niger ATCC 1015 : 52644-mRNA and A. versicolor : Aspve1_0048949	prot_ID_264	AO090012000513	
AO090012000511	5	825		Domain(s) with predicted role in transmembrane transport	prot_ID_649	AO090012000511	
AO090012000510	6	1869		Domain(s) with predicted monooxygenase activity and role in oxidation-reduction process	prot_ID_1219	AO090012000510	

AO090020000159 cluster
No PKS or NRPS backbone

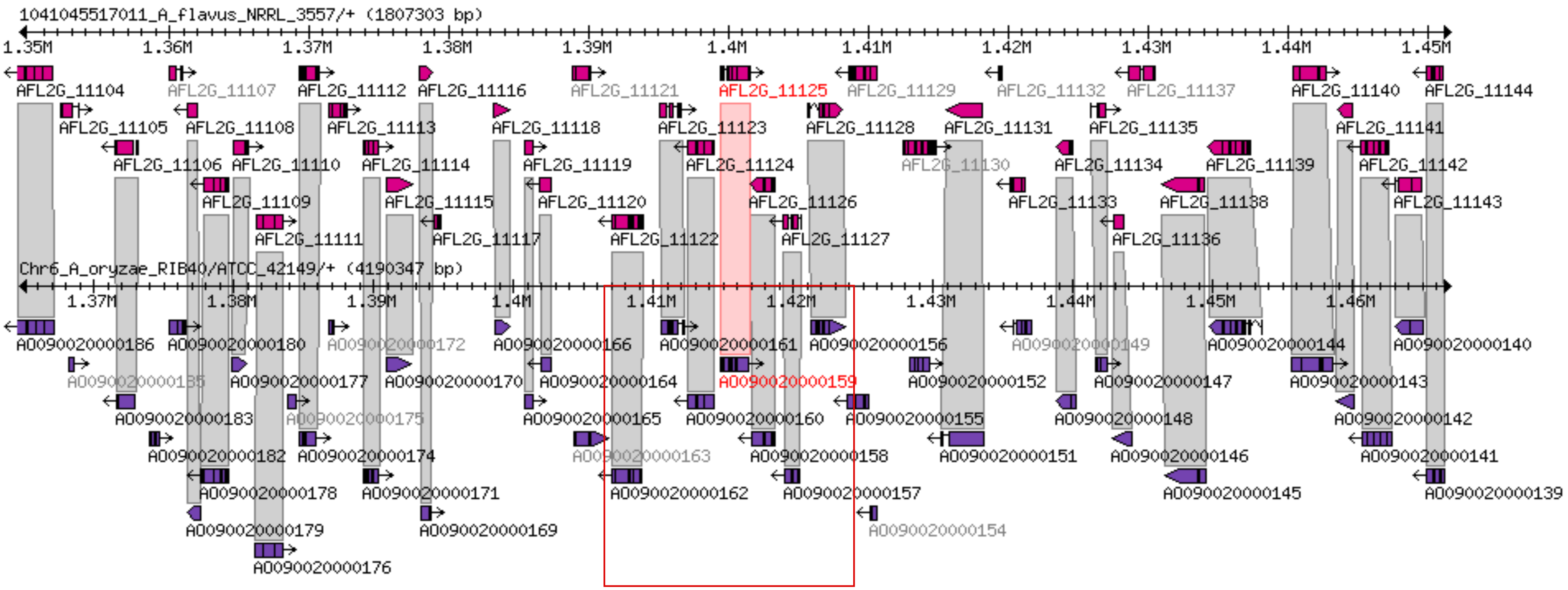

AO090020000159 cluster

Gene id (SMURF)	Gene positions	Gene distance	Gene Name	Annotated_gene_function	antiSMASH ID	AspGD ID	
n/a				Protein of unknown function	prot_ID_278	AO090020000154	
n/a				Domain(s) with predicted oxidoreductase activity and role in oxidation-reduction process	prot_ID_992	AO090020000155	
n/a				Domain(s) with predicted nucleic acid binding, nucleotide binding, zinc ion binding activity and intracellular localization	prot_ID_699	AO090020000156	ECS
n/a				Ortholog of A. flavus NRRL 3357 : AFL2T_11127	prot_ID_19	AO090020000157	
n/a				Domain(s) with predicted oxidoreductase activity and role in carotenoid biosynthetic process, chlorophyll biosynthetic process, oxidation-reduction process, photosynthesis	prot_ID_845	AO090020000158	
n/a				Domain(s) with predicted intramolecular lyase activity, transferase activity and role in biosynthetic process	prot_ID_321	AO090020000159	
n/a				Ortholog of A. niger CBS 513.88 : An12g00880, A. brasiliensis : Aspbr1_0211417, A. flavus NRRL 3357 : AFL2T_11124 and A. acidus : Aspfo1_0211604	prot_ID_246	AO090020000160	
n/a				Ortholog of A. nidulans FGSC A4 : AN6458, A. fumigatus Af293 : Afu3g07420, A. niger CBS 513.88 : An02g10960, An01g01420, An12g05390 and A. oryzae RIB40 : AO090005000921, AO090003001427	prot_ID_800	AO090020000161	
n/a				Domain(s) with predicted UDP-N-acetylmuramate dehydrogenase activity, flavin adenine dinucleotide binding activity and role in oxidation-reduction process	prot_ID_247	AO090020000162	ECS
n/a				Protein of unknown function	prot_ID_798	AO090020000163	

AO090020000186 cluster

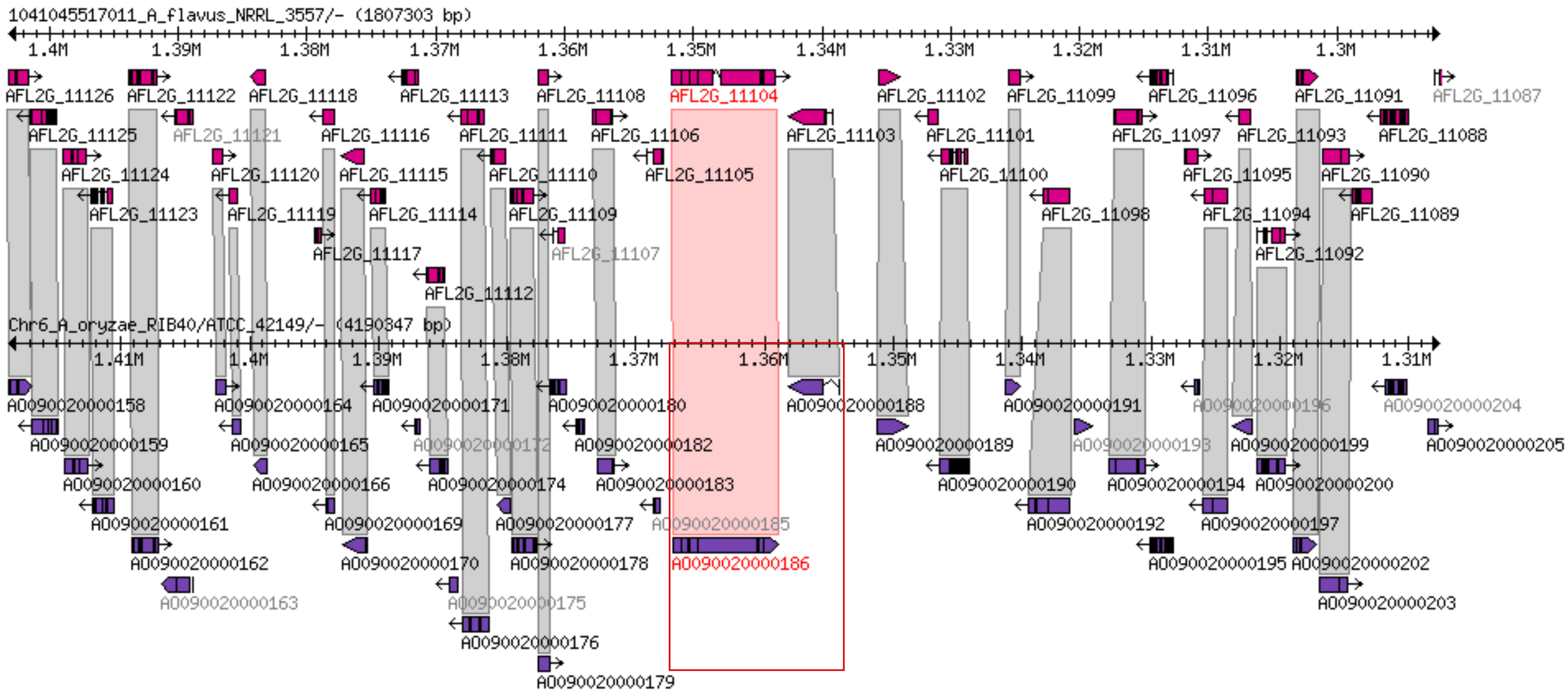

AO090020000186 cluster

Gene id (SMURF)	Gene positions	Gene distance	Gene Name	Annotated_gene_function	antiSMASH ID	AspGD ID	
n/a				Protein of unknown function	prot_ID_917	AO090020000172	
n/a				Domain(s) with predicted role in lipopolysaccharide biosynthetic process	prot_ID_678	AO090020000174	
n/a				Domain(s) with predicted DNA binding, transposase activity and role in DNA integration, regulation of transcription, DNA-dependent, transposition, DNA-mediated	prot_ID_1248	AO090020000175	
n/a				Domain(s) with predicted catalytic activity, cation binding activity and role in carbohydrate metabolic process	prot_ID_375	AO090020000176	
AO090020000177	-7	242		Domain(s) with predicted nucleotide binding, oxidoreductase activity and role in oxidation-reduction process	prot_ID_952	AO090020000177	
AO090020000178	-6	348		Ortholog of A. nidulans FGSC A4 : AN6315, AN9346, A. niger CBS 513.88 : An08g05310 and A. niger ATCC 1015 : 38283-mRNA, 43466-mRNA, 46133-mRNA	prot_ID_233	AO090020000178	
AO090020000179	-5	5		Ortholog(s) have nitrate reductase (NADPH) activity and role in molybdopterin cofactor biosynthetic process	prot_ID_804	AO090020000179	
AO090020000180	-4	841		Ortholog(s) have copper ion binding activity, role in copper ion transport, protein complex assembly and mitochondrial inner membrane localization	prot_ID_820	AO090020000180	
AO090020000182	-3	940		Domain(s) with predicted zinc ion binding activity	prot_ID_506	AO090020000182	
AO090020000183	-2	2970		Domain(s) with predicted UDP-N-acetylmuramate dehydrogenase activity, flavin adenine dinucleotide binding activity and role in oxidation-reduction process	prot_ID_713	AO090020000183	
AO090020000185	-1	988		Protein of unknown function	prot_ID_690	AO090020000185	
**AO090020000186**	0	0		Polyketide synthase	prot_ID_286	AO090020000186	ECS
AO090020000188	1	1370		Ortholog of A. nidulans FGSC A4 : AN4840, A. fumigatus Af293 : Afu3g07350, A. niger CBS 513.88 : An02g13370, A. niger ATCC 1015 : 119945-mRNA and A. versicolor : Aspve1_0126685	prot_ID_1045	AO090020000188	ECS
AO090020000189	2	3054		Ortholog of A. nidulans FGSC A4 : AN4839, A. fumigatus Af293 : Afu3g07340, A. niger CBS 513.88 : An02g13380, A. niger ATCC 1015 : 130550-mRNA and A. versicolor : Aspve1_0051705	prot_ID_327	AO090020000189	
AO090020000190	3	3094		Domain(s) with predicted role in transmembrane transport and integral to membrane localization	prot_ID_461	AO090020000190	
AO090020000191	4	2904		Ortholog of A. nidulans FGSC A4 : AN12127, A. fumigatus Af293 : Afu3g07310, A. niger CBS 513.88 : An02g13400, A. niger ATCC 1015 : 173674-mRNA and A. versicolor : Aspve1_0027262, Aspve1_0040450	prot_ID_1165	AO090020000191	
AO090020000192	5	2010		Domain(s) with predicted acetyl-CoA transporter activity and integral to membrane localization	n/a	AO090020000192	

AO090020000194 cluster

No PKS or NRPS backbone

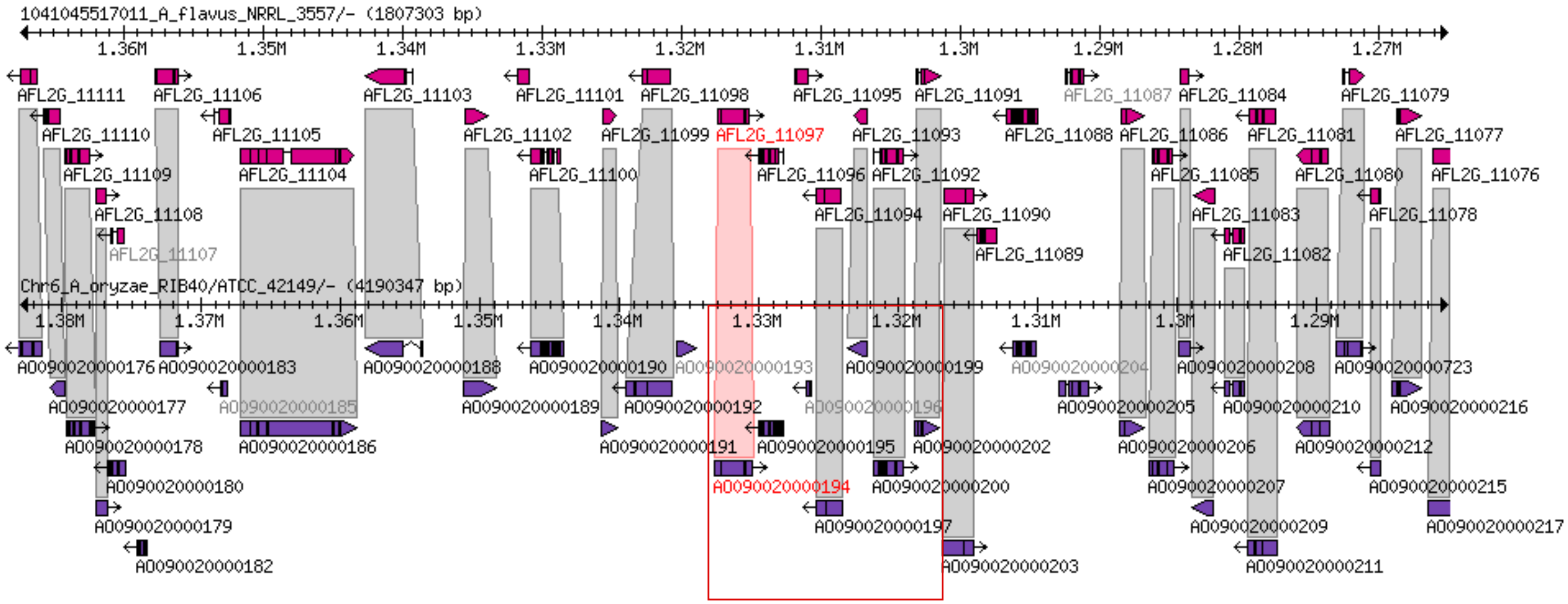

AO090020000194 cluster

Gene id (SMURF)	Gene positions	Gene distance	Gene Name	Annotated_gene_function	antiSMASH ID	AspGD ID	
n/a				Ortholog of A. nidulans FGSC A4 : AN12127, A. fumigatus Af293 : Afu3g07310, A. niger CBS 513.88 : An02g13400, A. niger ATCC 1015 : 173674-mRNA and A. versicolor : Aspve1_0027262, Aspve1_0040450	prot_ID_1165	AO090020000191	
n/a				Domain(s) with predicted acetyl-CoA transporter activity and integral to membrane localization	prot_ID_629	AO090020000192	
n/a				Protein of unknown function	prot_ID_85	AO090020000193	
n/a				Domain(s) with predicted intramolecular transferase activity	prot_ID_815	AO090020000194	ECS
n/a				Putative methyltransferase; upregulated in A. oryzae and A. nidulans under hypoxic growth conditions	prot_ID_267	AO090020000195	
n/a				Protein of unknown function	prot_ID_837	AO090020000196	
n/a				Ortholog of A. nidulans FGSC A4 : AN4834, A. fumigatus Af293 : Afu3g07270, A. niger CBS 513.88 : An02g13430, A. niger ATCC 1015 : 37531-mRNA and A. versicolor : Aspve1_0126541	prot_ID_265	AO090020000197	
n/a				Domain(s) with predicted hydrolase activity and role in metabolic process	prot_ID_438	AO090020000199	
n/a				Ortholog of A. nidulans FGSC A4 : AN4832, A. fumigatus Af293 : Afu3g07240, A. niger CBS 513.88 : An02g13480, A. niger ATCC 1015 : 207429-mRNA and A. versicolor : Aspve1_0128671	prot_ID_1037	AO090020000200	FA, ECS
n/a				Similar to phosphopantothenoylcysteine synthetase/decarboxylase	n/a	AO090020000202	
				Domain(s) with predicted serine-type endopeptidase activity, transferase activity, transferring glycosyl groups activity, role in blood coagulation, proteolysis and extracellular region, membrane localization		AO090020000203	

AO090020000240 cluster

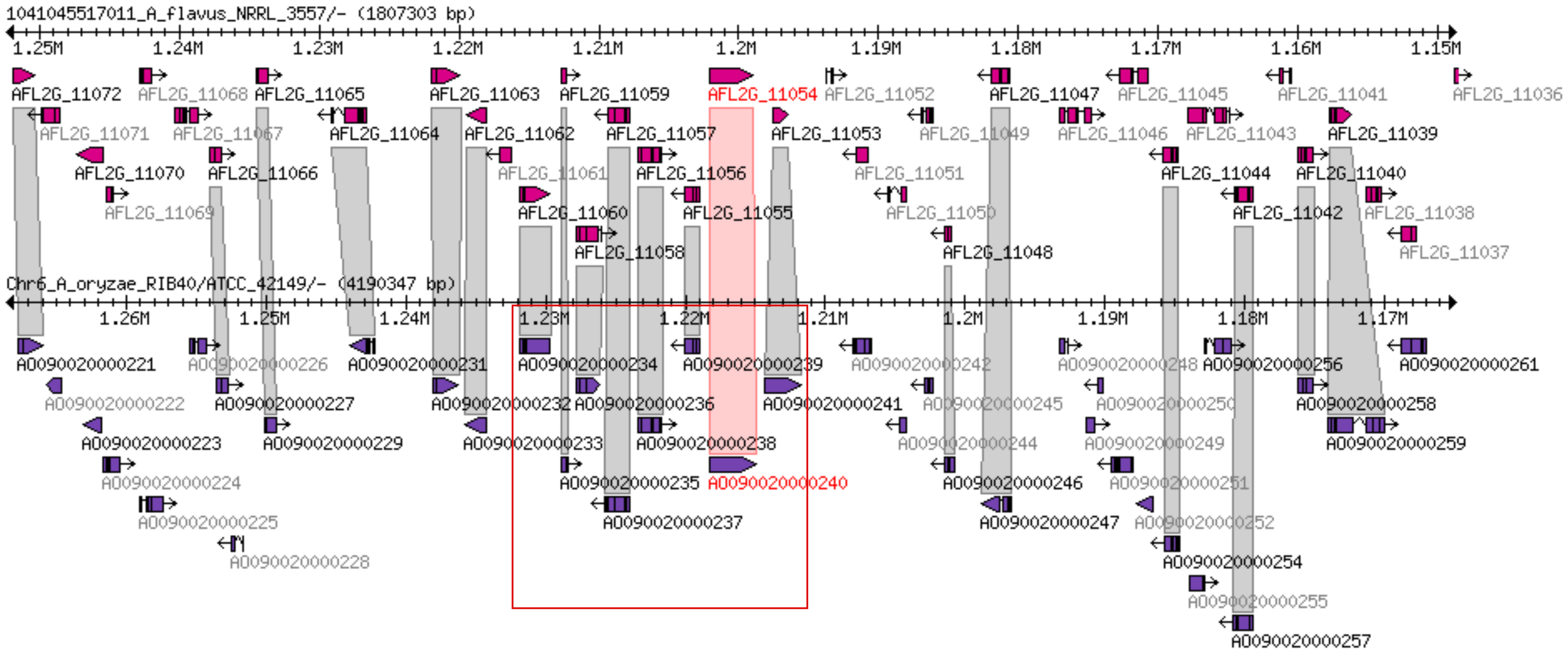

AO090020000240 cluster

Gene id (SMURF)	Gene positions	Gene distance	Gene Name	Annotated_gene_function	antiSMASH ID	AspGD ID	
n/a				Domain(s) with predicted electron carrier activity, molybdenum ion binding, oxidoreductase activity and role in oxidation-reduction process	prot_ID_554	AO090020000232	
n/a				Domain(s) with predicted acid phosphatase activity	prot_ID_79	AO090020000233	
n/a				Domain(s) with predicted role in Mo-molybdopterin cofactor biosynthetic process	prot_ID_466	AO090020000234	ECS, IGD
n/a				Domain(s) with predicted role in sulfur compound metabolic process	prot_ID_1170	AO090020000235	
AO090020000236	-4	357		Domain(s) with predicted transmembrane transporter activity, role in transmembrane transport and integral to membrane localization	prot_ID_387	AO090020000236	
AO090020000237	-3	679		Domain(s) with predicted electron carrier activity, heme binding, iron ion binding, monooxygenase activity, oxidoreductase activity, acting on paired donors, with incorporation or reduction of molecular oxygen activity	prot_ID_980	AO090020000237	
AO090020000238	-2	1529		Cytochrome P450 monooxygenase	prot_ID_117	AO090020000238	
AO090020000239	-1	685		Domain(s) with predicted nucleotide binding activity	prot_ID_797	AO090020000239	
**AO090020000240**	0	0		Non-ribosomal peptide synthetase	prot_ID_628	AO090020000240	
AO090020000241	1	1382		Domain(s) with predicted nucleotide binding, oxidoreductase activity, zinc ion binding activity and role in oxidation-reduction process	prot_ID_87	AO090020000241	ECS, IGD
n/a				Protein of unknown function	prot_ID_1130	AO090020000242	
n/a				Protein of unknown function	prot_ID_997	AO090020000244	
n/a				Domain(s) with predicted carbohydrate binding activity	prot_ID_605	AO090020000245	
n/a				Domain(s) with predicted carbohydrate binding activity	prot_ID_208	AO090020000246	
n/a				Ortholog of A. flavus NRRL 3357 : AFL2T_11047	prot_ID_434	AO090020000247	

AO090020000380 cluster

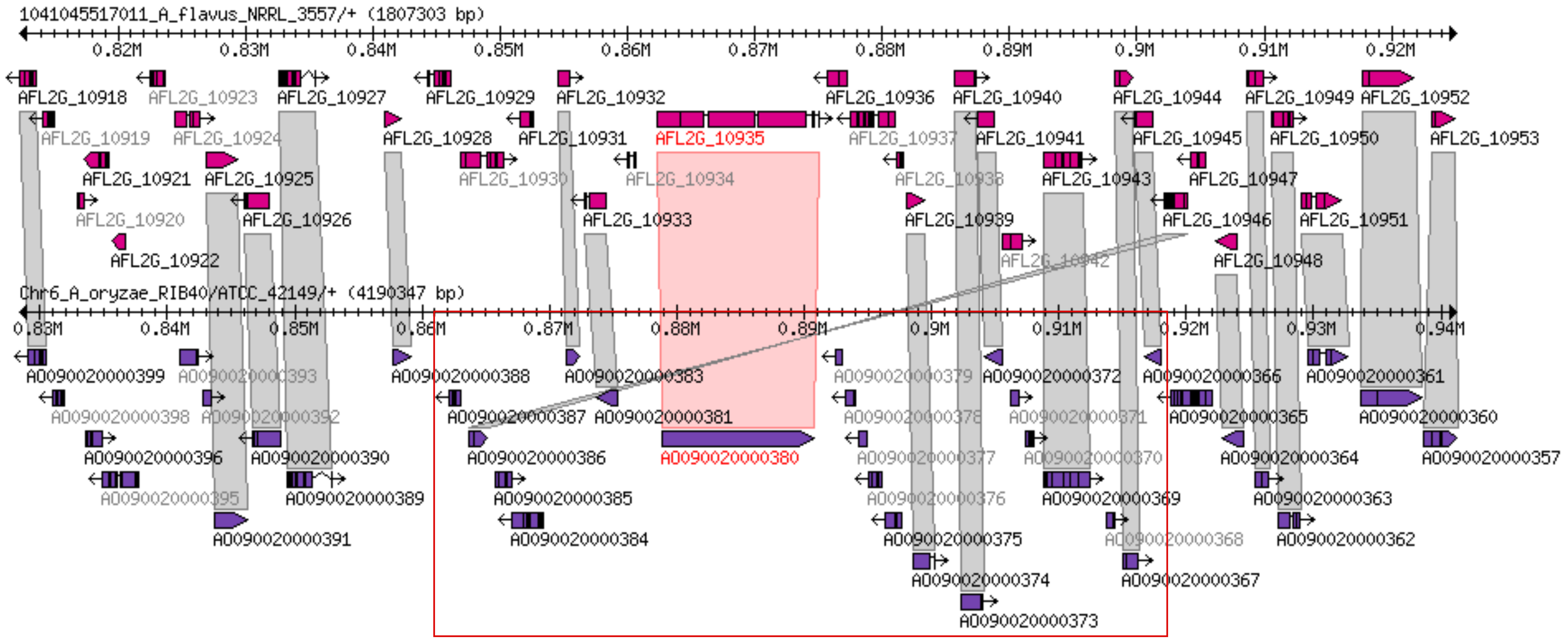

AO090020000380 cluster

Gene id (SMURF)	Gene positions	Gene distance	Gene Name	Annotated_gene_function	antiSMASH ID	AspGD ID	
				Domain(s) with predicted monooxygenase activity and role in oxidation-reduction process		AO090020000366	ECS, FA
n/a				Transmembrane transporter	n/a	AO090020000367	
n/a				Protein of unknown function	n/a	AO090020000368	
n/a				Domain(s) with predicted FMN binding, NADPH-hemoprotein reductase activity, aromatase activity, electron carrier activity, heme binding, iron ion binding activity and role in oxidation-reduction process	prot_ID_912	AO090020000369	
n/a				Protein of unknown function	prot_ID_252	AO090020000370	
n/a				domain(s) with predicted oxidoreductase activity and role in oxidation-reduction process	prot_ID_582	AO090020000371	
n/a				Domain(s) with predicted aldehyde dehydrogenase [NAD(P)+] activity and role in cellular aldehyde metabolic process, oxidation-reduction process	prot_ID_126	AO090020000372	
n/a				Domain(s) with predicted carbon-nitrogen ligase activity, with glutamine as amido-N-donor, hydrolase activity, acting on carbon-nitrogen (but not peptide) bonds, in linear amides activity	prot_ID_1002	AO090020000373	
n/a				Ortholog of A. flavus NRRL 3357 : AFL2T_10939	prot_ID_583	AO090020000374	
n/a				Domain(s) with predicted glutamate-1-semialdehyde 2,1-aminomutase activity, pyridoxal phosphate binding, transaminase activity and role in tetrapyrrole biosynthetic process	prot_ID_795	AO090020000375	
n/a				Protein of unknown function	prot_ID_468	AO090020000376	
n/a				Protein of unknown function	prot_ID_260	AO090020000377	
n/a				Domain(s) with predicted oxidoreductase activity and role in oxidation-reduction process	prot_ID_794	AO090020000378	
n/a				Protein of unknown function	prot_ID_1150	AO090020000379	
**AO090020000380**	0	0		Non-ribosomal peptide synthetase	prot_ID_508	AO090020000380	
AO090020000381	1	3604		Domain(s) with predicted nucleotide binding, oxidoreductase activity and role in oxidation-reduction process	prot_ID_768	AO090020000381	
AO090020000383	2	1492		Domain(s) with predicted catalytic activity, coenzyme binding, nucleotide binding activity and role in cellular metabolic process	prot_ID_791	AO090020000383	
AO090020000384	3	1959		Domain(s) with predicted DNA binding, sequence-specific DNA binding RNA polymerase II transcription factor activity, zinc ion binding activity and role in regulation of transcription, DNA-dependent, transcription, DNA-dependent	prot_ID_241	AO090020000384	
AO090020000385	4	73		Ortholog(s) have nitrilase activity and role in cyanide catabolic process	prot_ID_352	AO090020000385	
AO090020000386	5	593		Domain(s) with predicted role in transmembrane transport and integral to membrane localization	prot_ID_883	AO090020000386	

n/a	Domain(s) with predicted hydrolase activity, acting on carbon-nitrogen (but not peptide) bonds activity and role in nitrogen compound metabolic process	prot_ID_528	AO090020000387	IGD, FA
n/a	Ortholog of A. flavus NRRL 3357 : AFL2T_10928	prot_ID_1067	AO090020000388	

AO090020000527 cluster

No manual prediction made

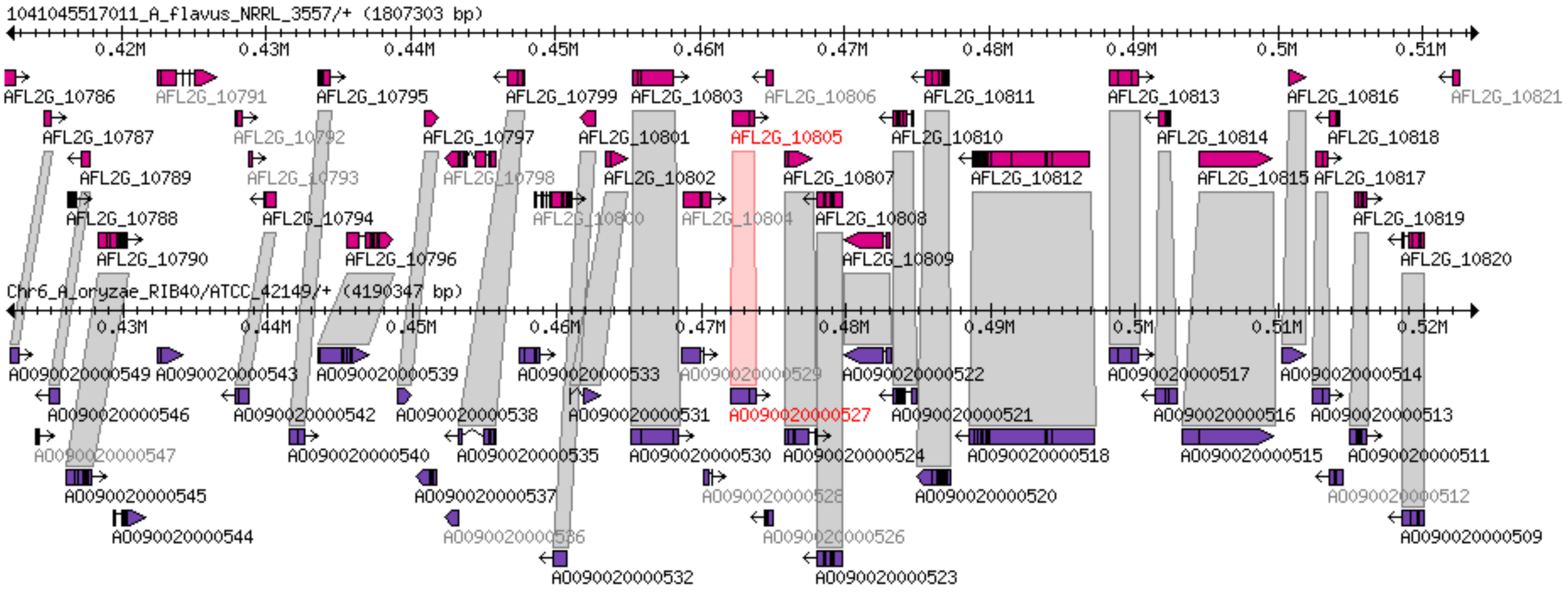

AO090020000527 cluster

Gene id (SMURF)	Gene positions	Gene distance	Gene Name	Annotated_gene_function	antiSMASH ID	AspGD ID
AO090020000515	-10	1978		Ortholog(s) have bile acid-exporting ATPase activity, role in bile acid and bile salt transport and fungal-type vacuole localization	n/a	AO090020000515
AO090020000516	-9	1563		Ortholog(s) have protein homodimerization activity, ubiquitin-protein ligase activity	n/a	AO090020000516
AO090020000517	-8	1597		Ortholog(s) have serine-type endopeptidase activity, role in asexual sporulation resulting in formation of a cellular spore, cellular response to starvation, vacuolar protein catabolic process and intracellular localization	n/a	AO090020000517
AO090020000518	-7	1803		Ortholog(s) have role in fungal-type cell wall organization, intracellular protein transport, response to pH and cytosol, extrinsic to membrane, mitochondrion localization	n/a	AO090020000518
AO090020000520	-6	1097		Ortholog(s) have sedoheptulose-7-phosphate:D-glyceraldehyde-3-phosphate glyceronetransferase activity, role in pentose-phosphate shunt and nucleus localization	n/a	AO090020000520
AO090020000521	-5	605		Ortholog(s) have superoxide dismutase activity	prot_ID_923	AO090020000521
AO090020000522	-4	598		Transcriptional coactivator p100	prot_ID_481	AO090020000522
AO090020000523	-3	51		Ortholog of A. nidulans FGSC A4 : AN0243, A. fumigatus Af293 : Afu5g09260, A. niger CBS 513.88 : An07g03750, A. niger ATCC 1015 : 48464-mRNA and A. versicolor : Aspve1_0078024	prot_ID_1034	AO090020000523
AO090020000524	-2	868		Domain(s) with predicted transporter activity, role in oligopeptide transport and membrane localization	prot_ID_630	AO090020000524
AO090020000526	-1	860		Protein of unknown function	prot_ID_46	AO090020000526
**AO090020000527**	0	0		Ortholog(s) have dimethylallyltranstransferase activity, peptidase activity, tryptophan dimethylallyltransferase activity and role in ergot alkaloid biosynthetic process, protein prenylation, shamixanthone biosynthetic process	prot_ID_606	AO090020000527
AO090020000528	1	1422		Protein of unknown function	prot_ID_188	AO090020000528
AO090020000529	2	17		Domain(s) with predicted catalytic activity and role in nucleoside metabolic process	prot_ID_717	AO090020000529
AO090020000530	3	612		Ortholog(s) have role in protein folding in endoplasmic reticulum and ER membrane protein complex localization	prot_ID_325	AO090020000530
AO090020000531	4	2269		Ortholog of A. nidulans FGSC A4 : AN4729, A. fumigatus Af293 : Afu5g09280, A. niger CBS 513.88 : An07g03730, An14g07330, A. oryzae RIB40 : AO090023000535 and A. niger ATCC 1015 : 180084-mRNA	prot_ID_1230	AO090020000531

AO090020000532	5	135	Ortholog of A. flavus NRRL 3357 : AFL2T_10801	n/a	AO090020000532
AO090020000533	6	814	Ortholog of Aspergillus aculeatus ATCC16872 : Aacu16872_023734	n/a	AO090020000533
AO090020000535	7	1750	Domain(s) with predicted nucleotide binding, oxidoreductase activity and role in oxidation-reduction process	n/a	AO090020000535

AO090023000082 cluster

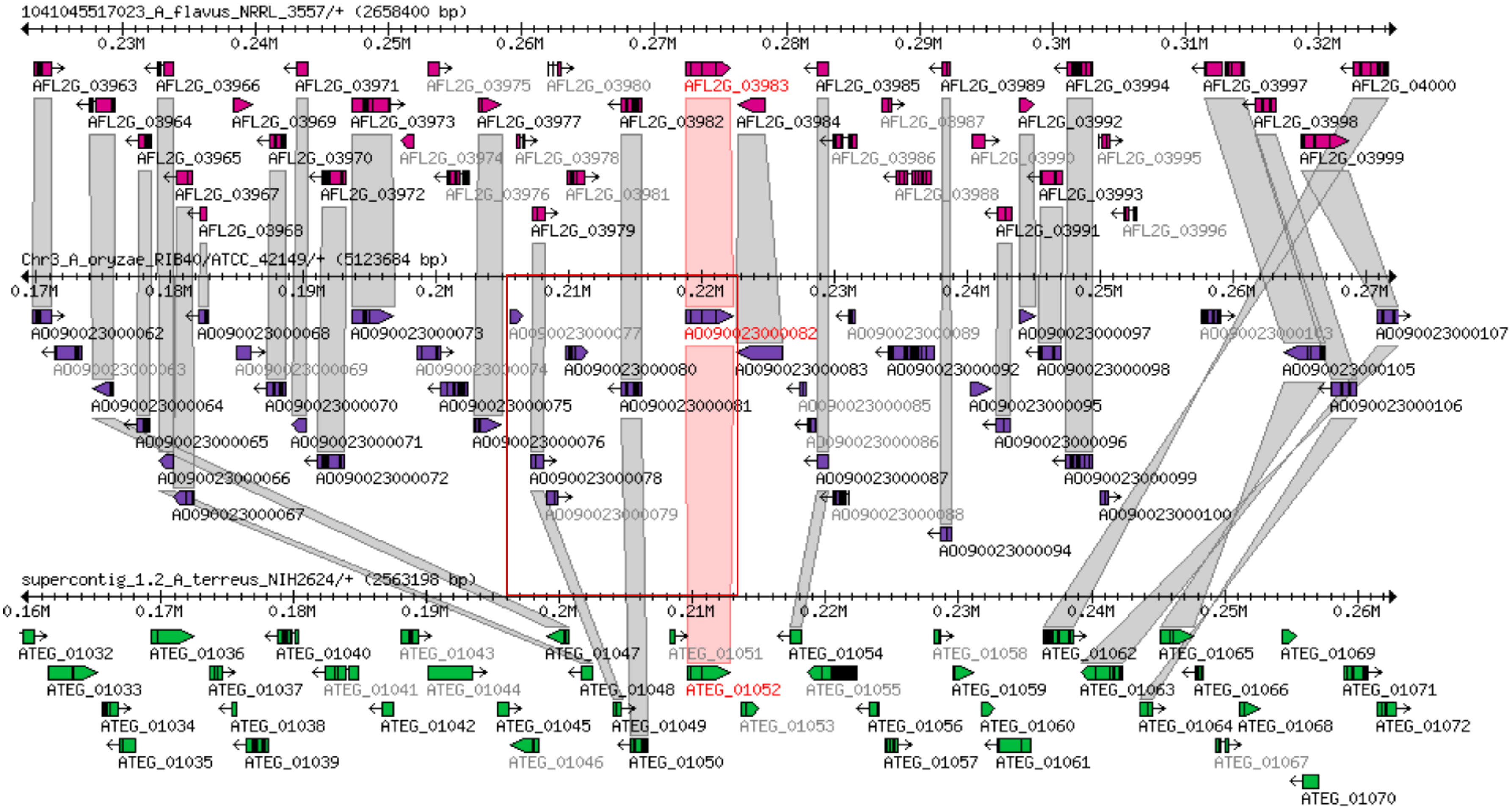

AO090023000082 cluster

Gene id (SMURF)	Gene positions	Gene distance	Gene Name	Annotated_gene_function	antiSMASH ID	AspGD ID	
AO090023000097	-11	792		Domain(s) with predicted hydrolase activity	n/a	AO090023000097	
AO090023000096	-10	369		Domain(s) with predicted catalytic activity, coenzyme binding, nucleotide binding activity and role in cellular metabolic process	prot_ID_689	AO090023000096	
AO090023000095	-9	1677		Domain(s) with predicted carbohydrate binding, catalytic activity and role in carbohydrate catabolic process	prot_ID_1380	AO090023000095	
AO090023000094	-8	889		Ortholog of A. fumigatus Af293 : Afu3g00550, N. fischeri NRRL 181 : NFIA_001160, NFIA_095350, A. flavus NRRL 3357 : AFL2T_03989 and A. versicolor : Aspve1_0148566	prot_ID_473	AO090023000094	
AO090023000092	-7	2993		Domain(s) with predicted role in transmembrane transport	prot_ID_1185	AO090023000092	
AO090023000089	-6	61		Protein of unknown function	prot_ID_775	AO090023000089	
AO090023000088	-5	447		Protein of unknown function	prot_ID_1312	AO090023000088	
AO090023000087	-4	78		Domain(s) with predicted nucleotide binding, oxidoreductase activity and role in oxidation-reduction process	prot_ID_1376	AO090023000087	
AO090023000086	-3	141		Domain(s) with predicted catalytic activity and role in metabolic process	prot_ID_1072	AO090023000086	
AO090023000085	-2	2654		Protein of unknown function	prot_ID_324	AO090023000085	
AO090023000083	-1	568		Ortholog(s) have glucan endo-1,3-beta-glucanase activity, C-3 substituted reducing group, licheninase activity and role in glucan catabolic process	prot_ID_1500	AO090023000083	
**AO090023000082**	0	0		Protein similar to nonribosomal peptide synthases (NRPS-like)	prot_ID_598	AO090023000082	ECS
AO090023000081	1	3375		Domain(s) with predicted O-methyltransferase activity	prot_ID_1289	AO090023000081	
AO090023000080	2	2487		Ortholog of A. niger CBS 513.88 : An09g05120, An12g06560, A. versicolor : Aspve1_0192318, A. sydowii : Aspsy1_1169784 and A. terreus NIH2624 : ATET_02475	prot_ID_566	AO090023000080	
AO090023000079	3	516		Protein of unknown function	prot_ID_707	AO090023000079	
AO090023000078	4	878		Ortholog(s) have intracellular localization	prot_ID_1415	AO090023000078	
AO090023000077	5	715		Domain(s) with predicted N-acetyltransferase activity and role in metabolic process	prot_ID_321	AO090023000077	FA
AO090023000076	6	1062		Domain(s) with predicted beta-galactosidase activity, cation binding activity, role in carbohydrate metabolic process and beta-galactosidase complex localization	prot_ID_1011	AO090023000076	
AO090023000075	7	695		Domain(s) with predicted substrate-specific transmembrane transporter activity, role in transmembrane transport and integral to membrane localization	prot_ID_1406	AO090023000075	
AO090023000074	8	26		Domain(s) with predicted DNA binding, zinc ion binding activity, role in transcription, DNA-dependent and nucleus localization	prot_ID_471	AO090023000074	

AO090023000073	9	2580	Ortholog of A. nidulans FGSC A4 : AN1594, AN9314, A. fumigatus Af293 : Afu5g15060, A. niger CBS 513.88 : An18g02710, A. niger ATCC 1015 : 54123-mRNA and A. versicolor : Aspve1_0137870	n/a	AO090023000073
AO090023000072	10	684	Cytochrome P450 monooxygenase	n/a	AO090023000072

AO090023000444 cluster

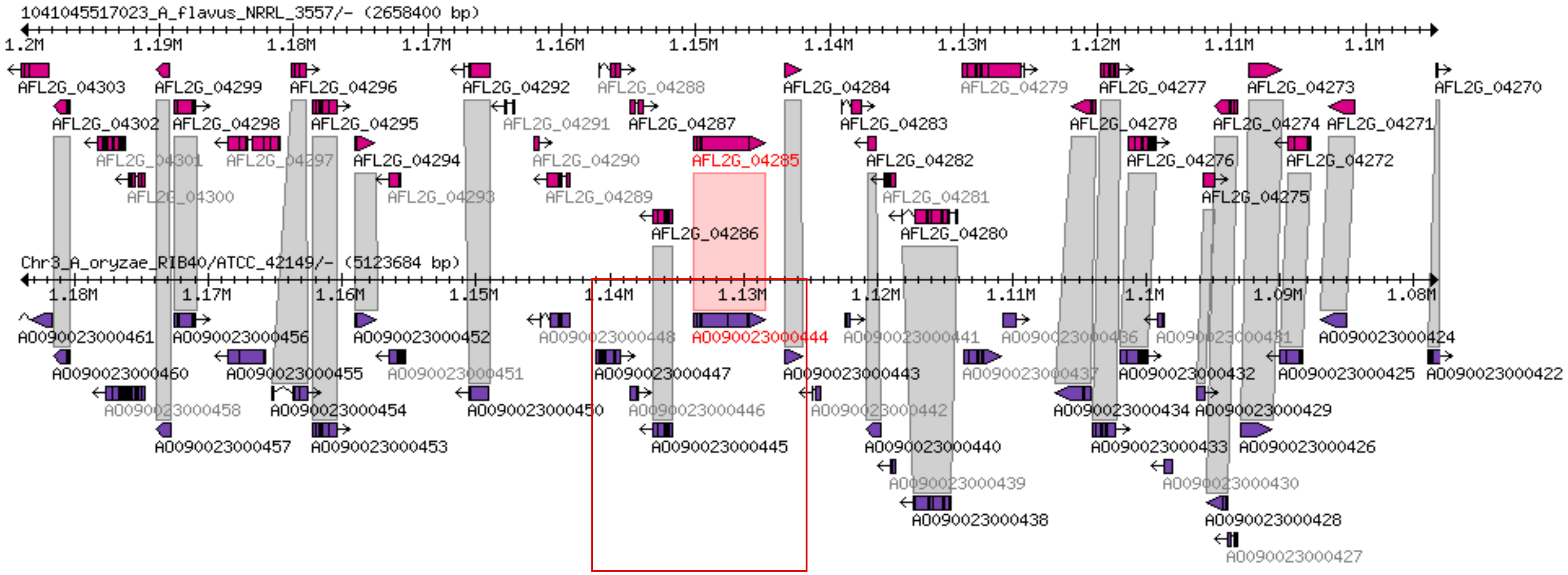

AO090023000444 cluster

Gene id (SMURF)	Gene positions	Gene distance	Gene Name	Annotated_gene_function	antiSMASH ID	AspGD ID	
AO090023000450	-5	3765		Cytochrome P450 monooxygenase; upregulated by exposure to benomyl	prot_ID_184	AO090023000450	
AO090023000448	-4	1955		Domain(s) with predicted acid phosphatase activity	prot_ID_1017	AO090023000448	
AO090023000447	-3	611		Cytochrome P450 monooxygenase	prot_ID_702	AO090023000447	FA
AO090023000446	-2	1061		Domain(s) with predicted methyltransferase activity and role in metabolic process	prot_ID_1439	AO090023000446	
AO090023000445	-1	1529		Domain(s) with predicted O-methyltransferase activity	prot_ID_467	AO090023000445	
**AO090023000444**	0	0		Polyketide synthase	prot_ID_1264	AO090023000444	
n/a				Ortholog(s) have intracellular localization	prot_ID_1099	AO090023000443	ECS
n/a				Domain(s) with predicted catalytic activity, pyridoxal phosphate binding activity	prot_ID_1373	AO090023000442	
n/a				Protein of unknown function	prot_ID_816	AO090023000441	
n/a				Ortholog of A. fumigatus Af293 : Afu2g00980, N. fischeri NRRL 181 : NFIA_033290, A. flavus NRRL 3357 : AFL2T_04282, A. clavatus NRRL 1 : ACLA_003690 and A. versicolor : Aspve1_0043932	prot_ID_388	AO090023000440	
n/a				Protein of unknown function	prot_ID_212	AO090023000439	
n/a				Domain(s) with predicted serine-type peptidase activity and role in proteolysis	prot_ID_1119	AO090023000438	
n/a				Protein of unknown function	prot_ID_24	AO090023000437	
n/a				Protein of unknown function	prot_ID_946	AO090023000436	

AO090023000528 cluster

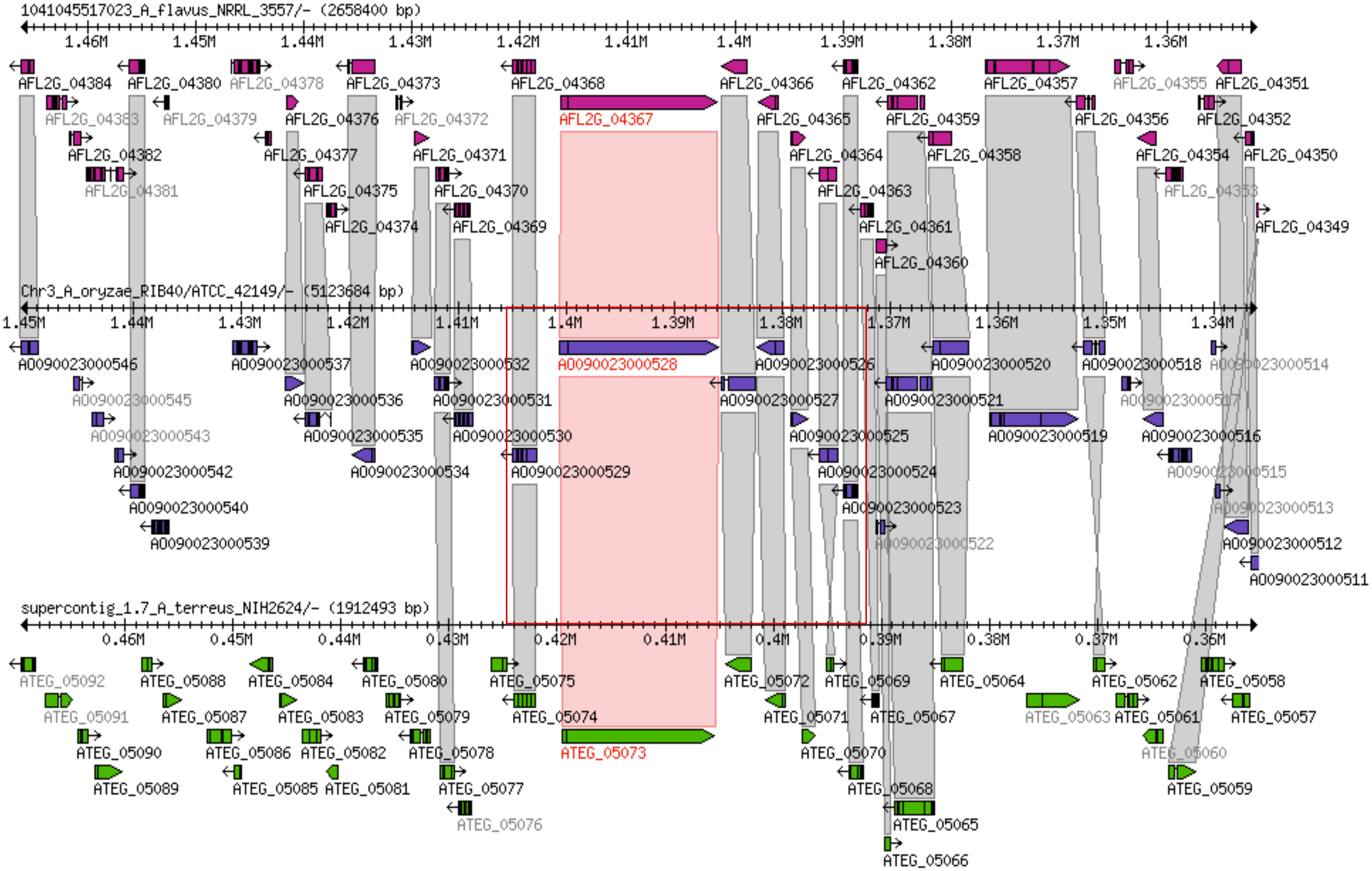

AO090023000528 cluster

Gene id (SMURF)	Gene positions	Gene distance	Gene Name	Annotated_gene_function	antiSMASH ID	AspGD ID	
n/a				Ortholog(s) have zinc ion transmembrane transporter activity and role in response to zinc ion, zinc ion transmembrane transport	prot_ID_710	AO090023000536	
n/a				Ortholog of A. nidulans FGSC A4 : AN4729, A. fumigatus Af293 : Afu5g09280, A. niger CBS 513.88 : An07g03730, An14g07330, A. oryzae RIB40 : AO090020000531 and A. niger ATCC 1015 : 180084-mRNA	prot_ID_1249	AO090023000535	
n/a				Domain(s) with predicted peptidase activity and role in proteolysis	prot_ID_533	AO090023000534	
n/a				Ortholog of A. nidulans FGSC A4 : AN1604/agnE, A. fumigatus Af293 : Afu1g00650, Afu7g08510, Afu8g06030, A. niger CBS 513.88 : An08g09610/agnD, An15g04760 and A. oryzae RIB40 : AO090005000538	prot_ID_717	AO090023000532	
n/a				Domain(s) with predicted oxidoreductase activity and role in oxidation-reduction process	prot_ID_376	AO090023000531	
n/a				Domain(s) with predicted flavin adenine dinucleotide binding, oxidoreductase activity and role in oxidation-reduction process	prot_ID_889	AO090023000530	
n/a				Ortholog(s) have role in N',N'',N'''-triacetylfusarinine C biosynthetic process, cellular response to hydrogen peroxide, cellular response to iron ion starvation and ergosterol biosynthetic process	prot_ID_15	AO090023000529	ECS, IGD
**AO090023000528**	0	0		Putative nonribosomal peptide synthase (NRPS)	prot_ID_901	AO090023000528	
AO090023000527	1	461		Ortholog of A. nidulans FGSC A4 : AN0605, A. fumigatus Af293 : Afu6g10890, A. niger CBS 513.88 : An09g02900, A. niger ATCC 1015 : 54387-mRNA and A. terreus NIH2624 : ATET_05072	prot_ID_680	AO090023000527	
AO090023000526	2	1021		Domain(s) with predicted endonuclease activity and role in nucleotide-excision repair, response to UV	prot_ID_1624	AO090023000526	
AO090023000525	3	1002		Ortholog of A. nidulans FGSC A4 : AN0602, A. niger CBS 513.88 : An15g02350, A. niger ATCC 1015 : 48700-mRNA, A. versicolor : Aspve1_0146150 and A. sydowii : Aspsy1_0055411	prot_ID_1	AO090023000525	
AO090023000524	4	1783		Ortholog of A. nidulans FGSC A4 : AN0600, A. fumigatus Af293 : Afu6g10940, A. niger CBS 513.88 : An06g01260, A. niger ATCC 1015 : 52539-mRNA and A. versicolor : Aspve1_0058576	prot_ID_932	AO090023000524	
AO090023000523	5	1021		Ortholog(s) have (R,R)-butanediol dehydrogenase activity, role in alcohol metabolic process, butanediol biosynthetic process and cytoplasm, nucleus localization	prot_ID_1349	AO090023000523	ECS
n/a				Protein of unknown function	prot_ID_1046	AO090023000522	

n/a	Damage-specific DNA binding complex, subunit DDB1	prot_ID_1285	AO090023000521
n/a	Ortholog(s) have electron carrier activity, role in ergosterol biosynthetic process and mitochondrial outer membrane localization	n/a	AO090023000520

AO090023000877 cluster

No manual prediction made

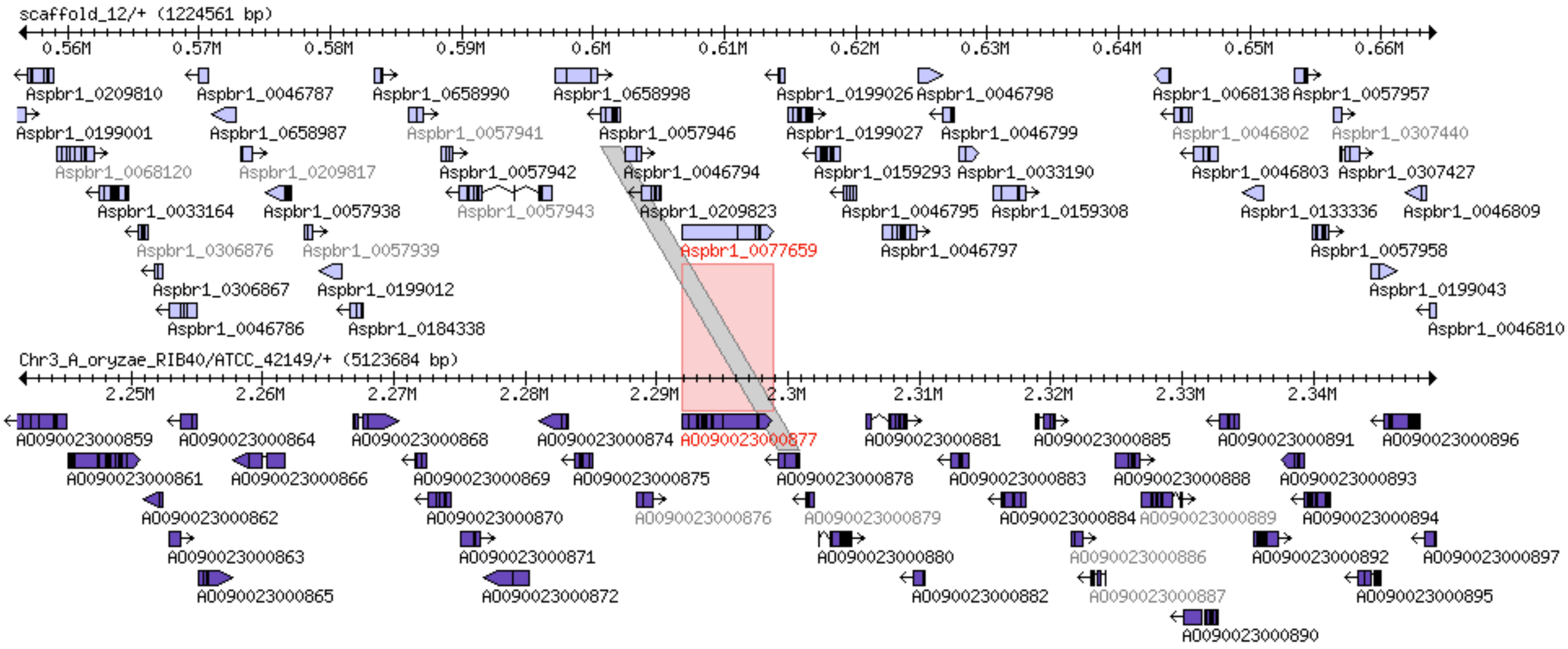

AO090023000877 cluster

Gene id (SMURF)	Gene positions	Gene distance	Gene Name	Annotated_gene_function	antiSMASH ID	AspGD ID
n/a				Domain(s) with predicted FMN binding, heme binding, oxidoreductase activity and role in oxidation-reduction process	prot_ID_1491	AO090023000884
n/a				Domain(s) with predicted nucleic acid binding activity	prot_ID_174	AO090023000883
n/a				Protein of unknown function	prot_ID_1283	AO090023000882
AO090023000881	-4	1150		Domain(s) with predicted role in transmembrane transport and integral to membrane localization	prot_ID_634	AO090023000881
AO090023000880	-3	350		Domain(s) with predicted monooxygenase activity and role in oxidation-reduction process	prot_ID_1350	AO090023000880
AO090023000879	-2	580		Protein of unknown function	prot_ID_909	AO090023000879
AO090023000878	-1	509		Domain(s) with predicted O-methyltransferase activity	prot_ID_222	AO090023000878
**AO090023000877**	0	0		Polyketide synthase	prot_ID_1300	AO090023000877
AO090023000876	1	2157		Domain(s) with predicted sequence-specific DNA binding RNA polymerase II transcription factor activity, zinc ion binding activity, role in regulation of transcription, DNA-dependent and nucleus localization	prot_ID_1410	AO090023000876
AO090023000875	2	3713		Domain(s) with predicted nucleotide binding, oxidoreductase activity and role in oxidation-reduction process	prot_ID_678	AO090023000875
n/a				Tryptophan synthase, beta chain	prot_ID_1540	AO090023000874
n/a				Aspartic-type endopeptidase	prot_ID_915	AO090023000872
n/a				Domain(s) with predicted FMN binding, oxidoreductase activity and role in oxidation-reduction process	prot_ID_250	AO090023000871
n/a				Ortholog of A. nidulans FGSC A4 : AN10552, A. fumigatus Af293 : Afu4g07060, A. niger CBS 513.88 : An04g01460, A. niger ATCC 1015 : 44254-mRNA and A. versicolor : Aspve1_0085132	prot_ID_1305	AO090023000870
n/a				Protein of unknown function	prot_ID_475	AO090023000869

AO090026000009 (afl) cluster

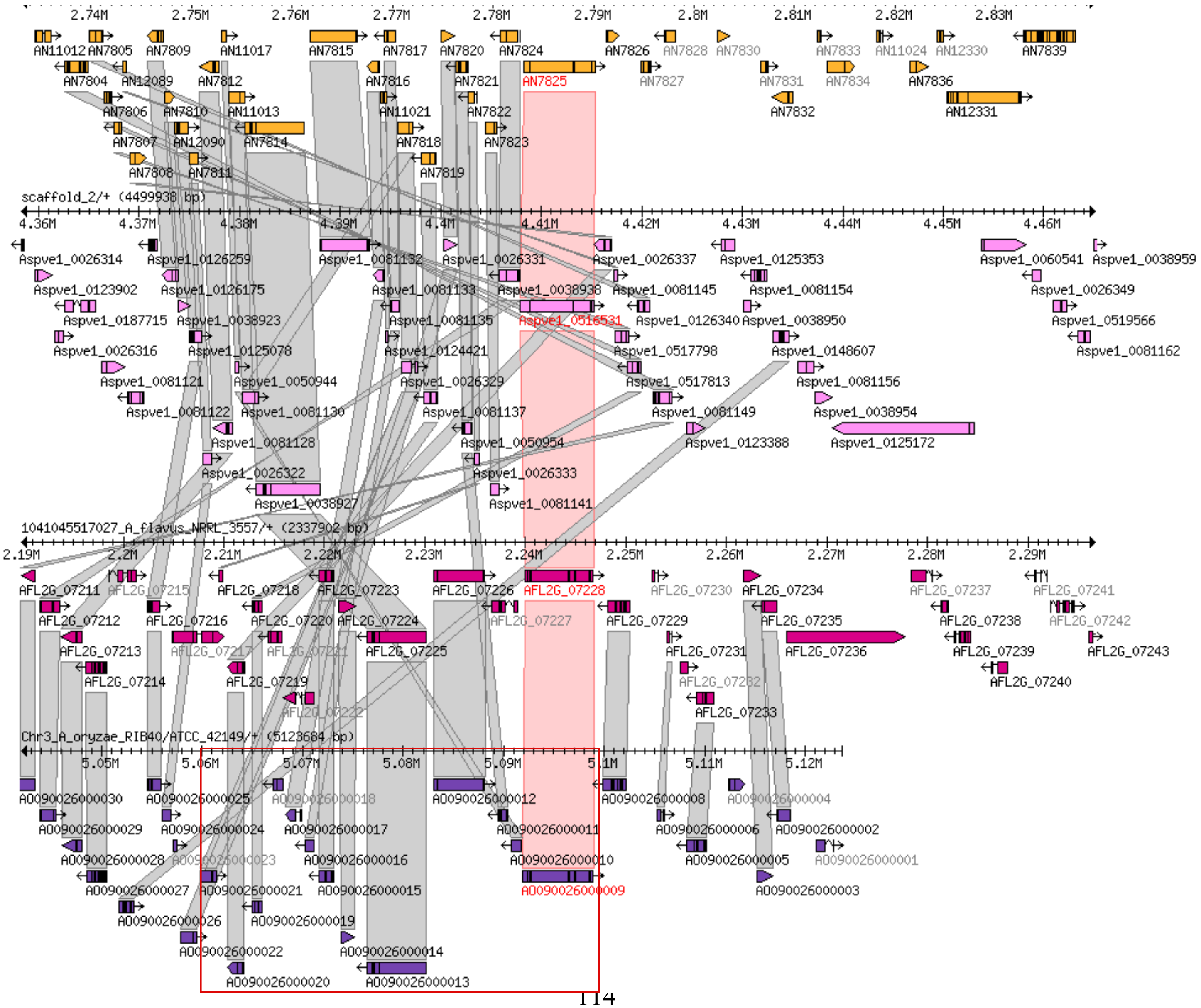

AO090026000009 (afl) cluster

Gene id (SMURF)	Gene positions	Gene distance	Gene Name	Annotated_gene_function	antiSMASH ID	AspGD ID	
n/a			cpaA	Polyketide synthase-nonribosomal peptide synthetase (PKS-NRPS); required for the biosynthesis of cyclopiazonic acid in strain NBRC 4177, RIB40 does not produce cyclopiazonic acid due to a reported truncation in the gene	prot_ID_1035	AO090026000001	
n/a			cpaD	O-dimethylallyltransferase (DMAT); regiospecifically dimethylallylates cAATrp to form beta-cyclopiazonic acid	prot_ID_339	AO090026000002	
n/a				Domain(s) with predicted electron carrier activity, oxidoreductase activity and role in oxidation-reduction process	prot_ID_561	AO090026000003	
AO090026000004	-4	2463		Cytochrome P450 monooxygenase	prot_ID_1303	AO090026000004	
AO090026000005	-3	2341		Domain(s) with predicted role in transmembrane transport and integral to membrane localization	prot_ID_1128	AO090026000005	
AO090026000006	-2	3237		Ortholog of A. flavus NRRL 3357 : AFL2T_07231	prot_ID_215	AO090026000006	
AO090026000008	-1	1341		Domain(s) with predicted role in transmembrane transport and integral to membrane localization; adjacent to the aflatoxin biosynthesis gene cluster	prot_ID_317	AO090026000008	
**AO090026000009**	0	0	pksA	Member of the aflatoxin biosynthesis gene cluster	prot_ID_1605	AO090026000009	ED
AO090026000010	1	750		Member of the aflatoxin biosynthesis gene cluster	prot_ID_347	AO090026000010	
AO090026000011	2	305		Domain(s) with predicted nucleotide binding, oxidoreductase activity and role in oxidation-reduction process; member of the aflatoxin biosynthesis gene cluster	prot_ID_892	AO090026000011	
AO090026000012	3	1315	fas2	Putative 3-oxoacyl-[acyl-carrier-protein] reductase (NADPH); member of the aflatoxin biosynthesis gene cluster	prot_ID_183	AO090026000012	
AO090026000013	4	690	fas1	Putative enoyl-[acyl-carrier-protein] reductase (NADH); member of the aflatoxin biosynthesis gene cluster	prot_ID_579	AO090026000013	
AO090026000014	5	1279	aflR	Putative sequence-specific DNA binding transcription with a predicted role in aflatoxin biosynthesis; member of the aflatoxin biosynthesis gene cluster	prot_ID_1053	AO090026000014	
AO090026000015	6	740	aflJ	Member of the aflatoxin biosynthesis gene cluster	prot_ID_368	AO090026000015	
AO090026000016	7	562	adhA	Domain(s) with predicted nucleotide binding, oxidoreductase activity and role in oxidation-reduction process; member of the aflatoxin biosynthesis gene cluster	n/a	AO090026000016	
AO090026000017	8	407	estA	Putative hydrolase; member of the aflatoxin biosynthesis gene cluster	n/a	AO090026000017	
AO090026000018	9	291	norA	Putative oxidoreductase; member of the aflatoxin biosynthesis gene cluster	n/a	AO090026000018	
AO090026000019	10	1094	ver1	Dehydrogenase/ketoreductase; putative aflatoxin biosynthesis protein; member of the aflatoxin biosynthesis gene cluster	n/a	AO090026000019	
AO090026000020	11	859	CYP59A4	Cytochrome P450 monooxygenase; member of the aflatoxin biosynthesis gene cluster	n/a	AO090026000020	

AO090026000021	12	1052	avnA	Member of the aflatoxin biosynthesis gene cluster	n/a	AO090026000021	ED
AO090026000022	13	368	CYP60B3	Cytochrome P450 monooxygenase; adjacent to the aflatoxin biosynthesis gene cluster	n/a	AO090026000022	
AO090026000023	14	375		Protein of unknown function	n/a	AO090026000023	
AO090026000024	15	205		Ortholog(s) have role in monodictyphenone biosynthetic process	n/a	AO090026000024	
AO090026000025	16	178		Domain(s) with predicted O-methyltransferase activity	n/a	AO090026000025	
AO090026000026	17	1266	omtA	O-methyltransferase	n/a	AO090026000026	
AO090026000027	18	1246	vbs	Domain(s) with predicted electron carrier activity, heme binding, iron ion binding, oxidoreductase activity, acting on paired donors, with incorporation or reduction of molecular oxygen activity and role in oxidation-reduction process	n/a	AO090026000027	
AO090026000028	19	515		Domain(s) with predicted flavin adenine dinucleotide binding, oxidoreductase activity, acting on CH-OH group of donors activity and role in oxidation-reduction process		AO090026000028	
AO090026000029	20	544	CYP62A1	Cytochrome P450 monooxygenase	n/a	AO090026000029	

AO090026000149 cluster

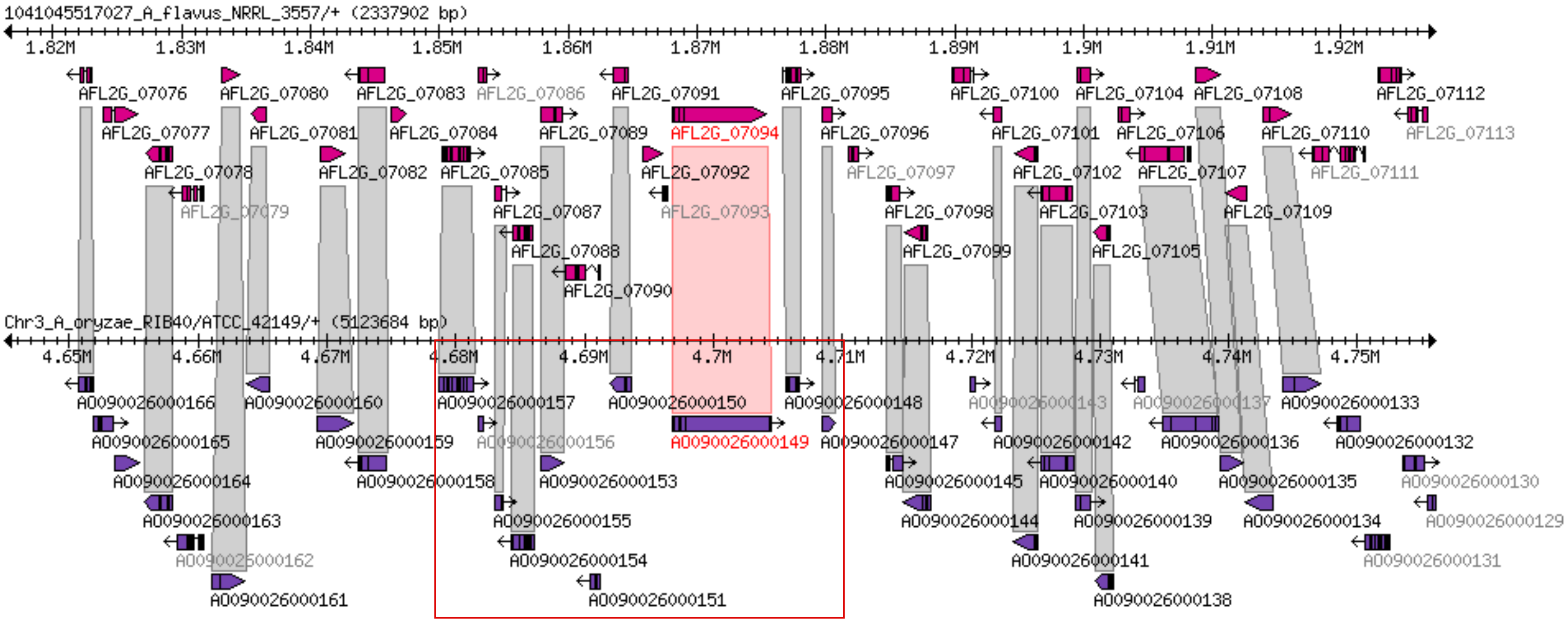

AO090026000149 cluster

Gene id (SMURF)	Gene positions	Gene distance	Gene Name	Annotated_gene_function	antiSMASH ID	AspGD ID	
n/a				Ortholog(s) have GMP synthase (glutamine-hydrolyzing) activity and role in GMP biosynthetic process	prot_ID_170	AO090026000141	
n/a				Ortholog of A. nidulans FGSC A4 : AN11171, AN7148, A. fumigatus Af293 : Afu4g03595, A. niger CBS 513.88 : An02g01320, An14g01685, An03g06340 and A. oryzae RIB40 : AO090026000213, AO090023000130	prot_ID_627	AO090026000142	
n/a				Domain(s) with predicted aldehyde dehydrogenase [NAD(P)+] activity and role in cellular aldehyde metabolic process, oxidation-reduction process	prot_ID_1565	AO090026000143	
n/a				Domain(s) with predicted role in transmembrane transport and integral to membrane localization	prot_ID_741	AO090026000144	
n/a				Domain(s) with predicted oxidoreductase activity and role in metabolic process	prot_ID_1220	AO090026000145	
n/a				Ortholog of A. flavus NRRL 3357 : AFL2T_07096	prot_ID_1163	AO090026000147	ECS, IGD
n/a				Ortholog(s) have feruloyl esterase activity and role in cell wall polysaccharide catabolic process, polysaccharide catabolic process	prot_ID_436	AO090026000148	
**AO090026000149**	0	0		Polyketide synthase	prot_ID_1341	AO090026000149	
AO090026000150	1	3254		Ortholog of A. niger CBS 513.88 : An01g01780, An15g02150, A. brasiliensis : Aspbr1_0047637, A. niger ATCC 1015 : 171681-mRNA, 40476-mRNA and A. flavus NRRL 3357 : AFL2T_07091	prot_ID_102	AO090026000150	
AO090026000151	2	1023		Ortholog of A. niger CBS 513.88 : An09g00960	prot_ID_1596	AO090026000151	
AO090026000153	3	2130		Ortholog(s) have alkyl sulfatase activity, arylsulfatase activity and role in dodecyl sulfate metabolic process	prot_ID_1348	AO090026000153	
AO090026000154	4	553		Domain(s) with predicted role in transmembrane transport and integral to membrane localization	prot_ID_1146	AO090026000154	
AO090026000155	5	576		Ortholog of A. flavus NRRL 3357 : AFL2T_07087	prot_ID_245	AO090026000155	
AO090026000156	6	822		Protein of unknown function	prot_ID_922	AO090026000156	
AO090026000157	7	587		Domain(s) with predicted ATP binding, ATPase activity and membrane localization	prot_ID_9	AO090026000157	ECS, IGD

AO090026000378 cluster

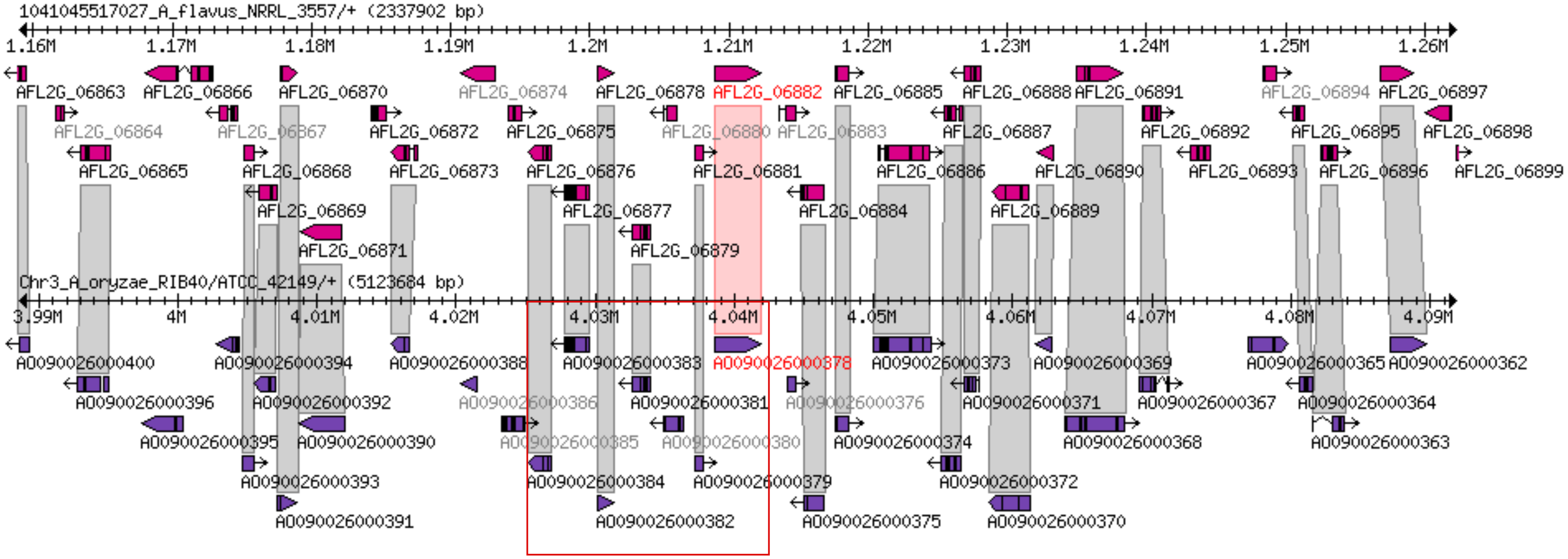

AO090026000378 cluster

Gene id (SMURF)	Gene positions	Gene distance	Gene Name	Annotated_gene_function	antiSMASH ID	AspGD ID	
n/a				Inosine-uridine preferring nucleoside hydrolase	n/a	AO090026000367	
n/a				WD40 repeat protein	n/a	AO090026000368	
n/a				Ortholog of A. nidulans FGSC A4 : AN6290, A. fumigatus Af293 : Afu2g12370, A. niger CBS 513.88 : An02g04480, A. niger ATCC 1015 : 36891-mRNA and A. versicolor : Aspve1_0041143	prot_ID_842	AO090026000369	
n/a				Ortholog of A. nidulans FGSC A4 : AN6289, A. fumigatus Af293 : Afu2g12380, A. niger CBS 513.88 : An02g04500, A. niger ATCC 1015 : 119850-mRNA and A. versicolor : Aspve1_0130464	prot_ID_912	AO090026000370	
n/a				Ortholog(s) have kinase regulator activity, role in cellular calcium ion homeostasis, conidium formation, protein phosphorylation, septin ring assembly and cell septum, cellular bud neck, spindle pole body localization	prot_ID_1463	AO090026000371	
n/a				Ortholog(s) have hydrogen ion transporting ATP synthase activity, rotational mechanism, proton-transporting ATPase activity, rotational mechanism activity and role in ATP synthesis coupled proton transport	prot_ID_1155	AO090026000372	
n/a				Ortholog(s) have cytoplasm localization	prot_ID_153	AO090026000373	
n/a				Ortholog of A. flavus NRRL 3357 : AFL2T_06885 and A. aculeatus ATCC16872 : Aacu16872_064869	prot_ID_1068	AO090026000374	
n/a				Ortholog of A. nidulans FGSC A4 : AN4040, AN7213, A. fumigatus Af293 : Afu5g00960, A. niger CBS 513.88 : An18g01180, An10g00910, An11g01220, An01g11560 and A. oryzae RIB40 : AO090023000615, AO090166000079	prot_ID_403	AO090026000375	
n/a				Protein of unknown function	prot_ID_68	AO090026000376	
**AO090026000378**	0	0		Non-ribosomal peptide synthetase	prot_ID_284	AO090026000378	ECS
AO090026000379	1	929		Ortholog of A. nidulans FGSC A4 : AN7208, A. fumigatus Af293 : Afu6g10080, A. versicolor : Aspve1_0125526 and A. sydowii : Aspsy1_0084577	prot_ID_1135	AO090026000379	
AO090026000380	2	813		Domain(s) with predicted catalytic activity and role in polyamine biosynthetic process	prot_ID_1360	AO090026000380	
AO090026000381	3	933		Ortholog of A. flavus NRRL 3357 : AFL2T_06879	prot_ID_587	AO090026000381	
AO090026000382	4	1310		Domain(s) with predicted trichodiene synthase activity and role in sesquiterpenoid biosynthetic process	prot_ID_255	AO090026000382	
AO090026000383	5	605		Cytochrome P450 monooxygenase	prot_ID_1129	AO090026000383	
AO090026000384	6	861		Domain(s) with predicted electron carrier activity, heme binding, iron ion binding, oxidoreductase activity, acting on paired donors, with incorporation or reduction of molecular oxygen activity and role in oxidation-reduction process	prot_ID_82	AO090026000384	ECS, FA

AO090026000385	7	255	Domain(s) with predicted nucleotide binding activity	prot_ID_1096	AO090026000385
AO090026000386	8	1735	Protein of unknown function	prot_ID_23	AO090026000386
AO090026000388	9	3664	Domain(s) with predicted UDP-N-acetylmuramate dehydrogenase activity, flavin adenine dinucleotide binding activity and role in oxidation-reduction process	prot_ID_206	AO090026000388
n/a			Ortholog of A. nidulans FGSC A4 : AN6284, A. fumigatus Af293 : Afu2g12430, A. niger CBS 513.88 : An02g04620, A. niger ATCC 1015 : 46977-mRNA and A.s versicolor : Aspve1_0641422	prot_ID_152	AO090026000390

AO090026000585 cluster
No matches to clusters in other species

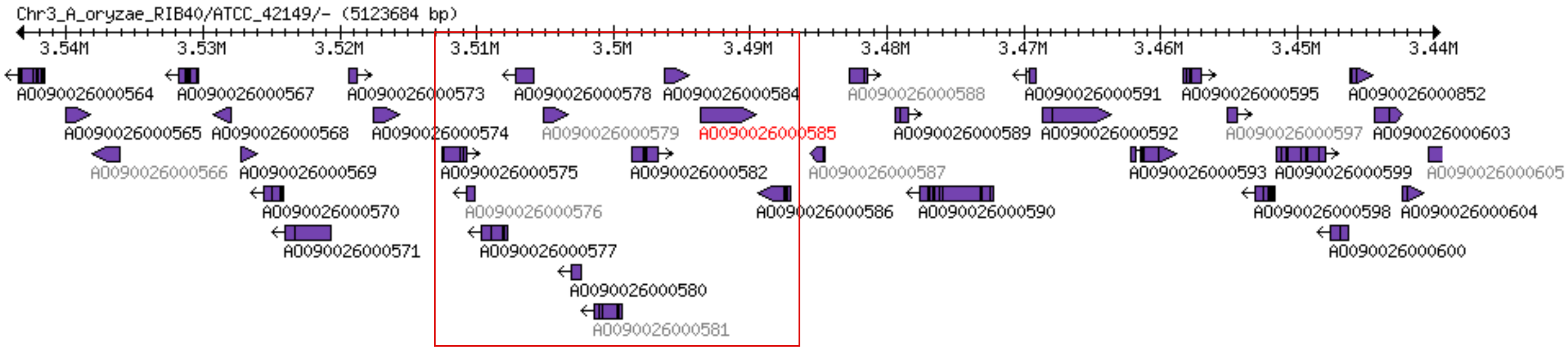

AO090026000585 cluster

Gene id (SMURF)	Gene positions	Gene distance	Gene Name	Annotated_gene_function	antiSMASH ID	AspGD ID	
AO090026000575	-9	61		Cytochrome P450 monooxygenase	prot_ID_447	AO090026000575	FA
AO090026000576	-8	501		Domain(s) with predicted hydrolase activity	prot_ID_182	AO090026000576	
AO090026000577	-7	613		Domain(s) with predicted role in transmembrane transport and integral to membrane localization	prot_ID_658	AO090026000577	
AO090026000578	-6	589		Ortholog(s) have role in alkaloid biosynthetic process, emericellamide A biosynthetic process	prot_ID_8	AO090026000578	
AO090026000579	-5	421		Cytochrome P450 monooxygenase	prot_ID_893	AO090026000579	
AO090026000580	-4	1064		Domain(s) with predicted nucleotide binding, oxidoreductase activity and role in oxidation-reduction process	prot_ID_729	AO090026000580	
AO090026000581	-3	962		Cytochrome P450 monooxygenase	prot_ID_1462	AO090026000581	
AO090026000582	-2	816		Protein of unknown function	prot_ID_501	AO090026000582	
AO090026000584	-1	972		Cytochrome P450 monooxygenase	prot_ID_374	AO090026000584	
**AO090026000585**	0	0		Non-ribosomal peptide synthetase	prot_ID_1065	AO090026000585	
n/a				Predicted phenylalanine ammonia-lyase	prot_ID_193	AO090026000586	FA
n/a				Protein of unknown function	prot_ID_875	AO090026000587	
n/a				Protein of unknown function	prot_ID_677	AO090026000588	
n/a				Protein of unknown function	prot_ID_1433	AO090026000589	
n/a				Guanine nucleotide binding protein MIP1	prot_ID_439	AO090026000590	
n/a				Ortholog of A. flavus NRRL 3357 : AFL2T_06706	prot_ID_1551	AO090026000591	

AO090038000086 cluster

Possible orphan PKS, no manual prediction made

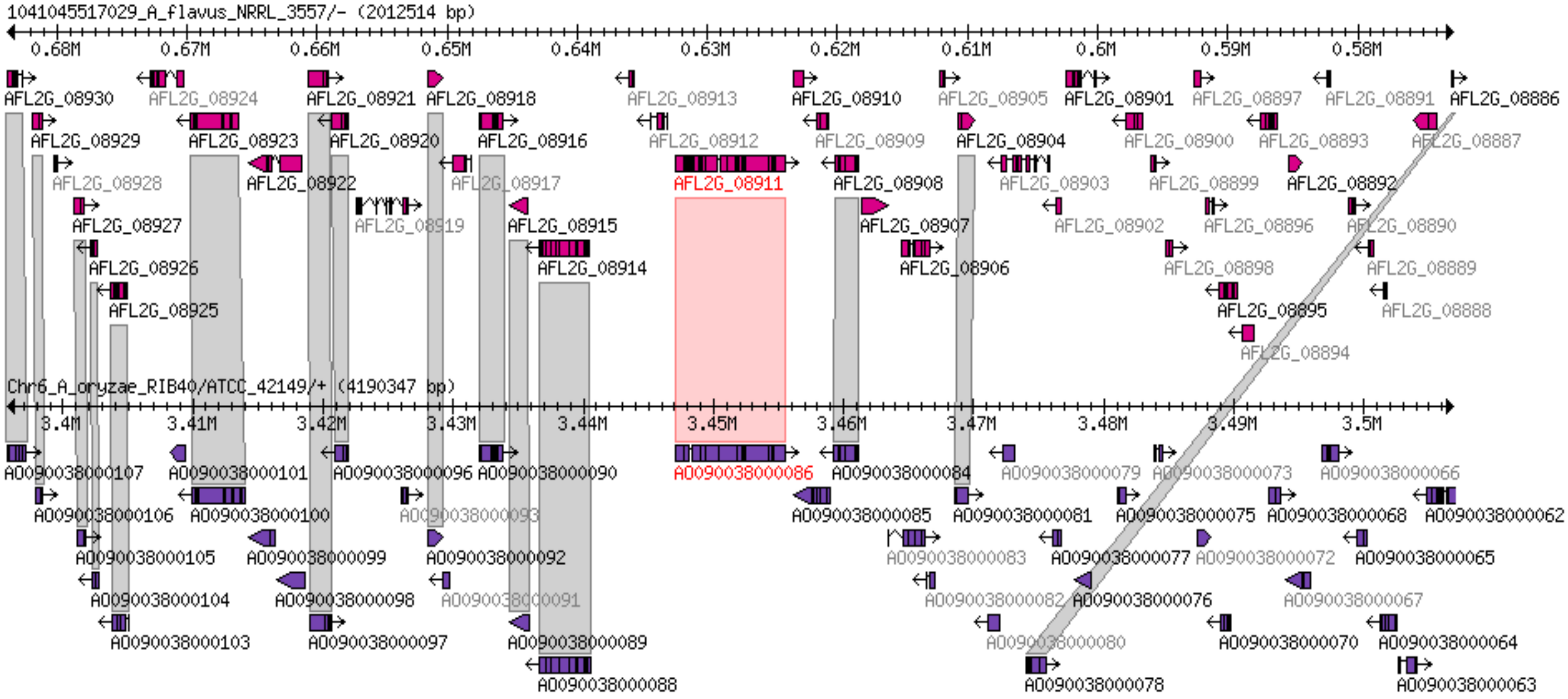

AO090038000086 cluster

Gene id (SMURF)	Gene positions	Gene distance	Gene Name	Annotated_gene_function	antiSMASH ID	AspGD ID
AO090038000078	-8	973		Domain(s) with predicted role in transmembrane transport and integral to membrane localization	prot_ID_138	AO090038000078
AO090038000079	-7	342		Protein of unknown function	prot_ID_737	AO090038000079
AO090038000080	-6	1391		Protein of unknown function	prot_ID_363	AO090038000080
AO090038000081	-5	1538		Domain(s) with predicted oxidoreductase activity and role in carotenoid biosynthetic process, chlorophyll biosynthetic process, oxidation-reduction process, photosynthesis	prot_ID_555	AO090038000081
AO090038000082	-4	67		Domain(s) with predicted transmembrane transporter activity, role in transmembrane transport and integral to membrane localization	prot_ID_1126	AO090038000082
AO090038000083	-3	2260		Domain(s) with predicted UDP-N-acetylmuramate dehydrogenase activity, flavin adenine dinucleotide binding activity and role in oxidation-reduction process	prot_ID_781	AO090038000083
AO090038000084	-2	439		Domain(s) with predicted role in transmembrane transport and integral to membrane localization	prot_ID_48	AO090038000084
AO090038000085	-1	1468		Domain(s) with predicted sequence-specific DNA binding RNA polymerase II transcription factor activity, zinc ion binding activity, role in regulation of transcription, DNA-dependent and nucleus localization	prot_ID_304	AO090038000085
**AO090038000086**	0	0		Polyketide synthase	prot_ID_886	AO090038000086
n/a				Domain(s) with predicted sequence-specific DNA binding RNA polymerase II transcription factor activity, zinc ion binding activity, role in regulation of transcription, DNA-dependent and nucleus localization	prot_ID_304	AO090038000085
n/a				Domain(s) with predicted ATP binding, ATPase activity, coupled to transmembrane movement of ions, phosphorylative mechanism, metal ion binding activity, role in ATP biosynthetic process, cation transport and membrane localization	prot_ID_1027	AO090038000088
n/a				Ortholog of A. nidulans FGSC A4 : AN2957, A. fumigatus Af293 : Afu4g14680, A. niger CBS 513.88 : An02g11270, A. niger ATCC 1015 : 173463-mRNA and A. versicolor : Aspve1_0051391	prot_ID_15	AO090038000089
n/a				Domain(s) with predicted substrate-specific transmembrane transporter activity, role in transmembrane transport and integral to membrane localization	prot_ID_187	AO090038000090
n/a				Protein of unknown function	prot_ID_736	AO090038000091
n/a				Ribokinase	prot_ID_142	AO090038000092

AO090038000098 cluster
No PKS or NRPS backbone

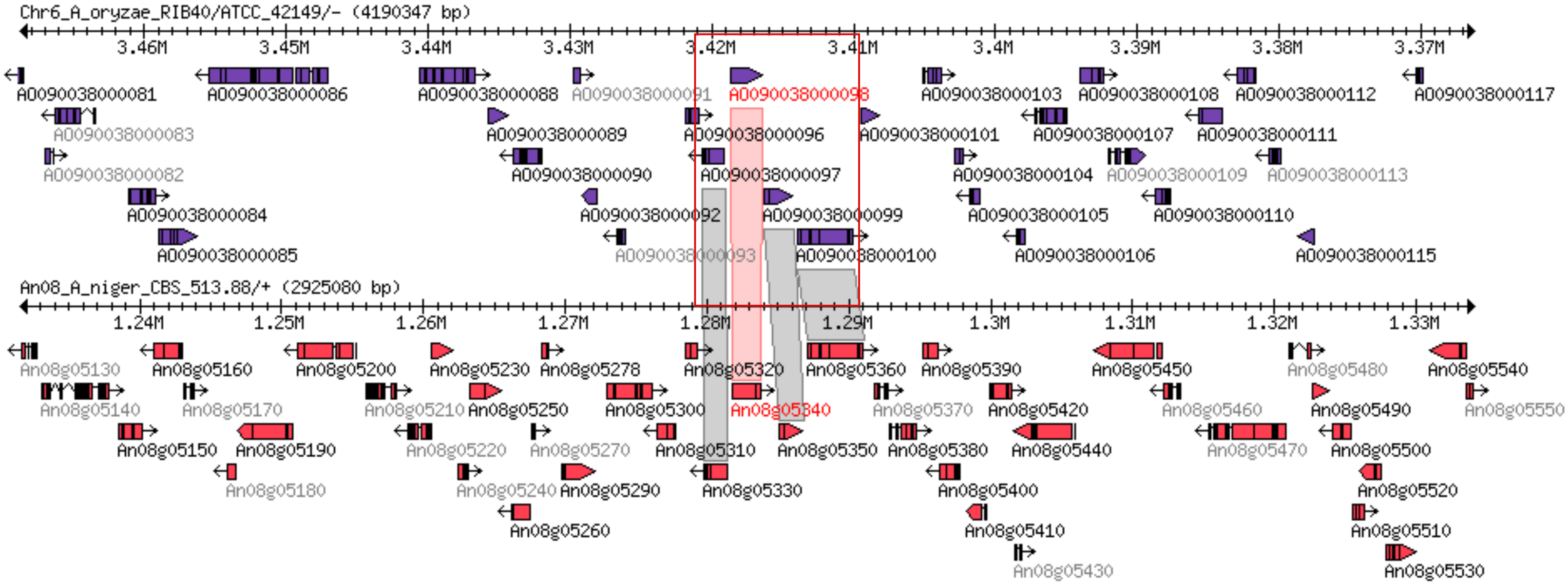

AO090038000098 cluster

Gene id (SMURF)	Gene positions	Gene distance	Gene Name	Annotated_gene_function	antiSMASH ID	AspGD ID	
n/a				Ortholog(s) have cytoplasm, nucleus localization	prot_ID_142	AO090038000092	
n/a				Protein of unknown function	prot_ID_58	AO090038000093	
n/a				Domain(s) with predicted oxidoreductase activity, acting on the CH-CH group of donors activity, role in lipid metabolic process and cytoplasm, integral to membrane localization	prot_ID_1102	AO090038000096	
n/a			CYP5087B1	Cytochrome P450 monooxygenase	prot_ID_1013	AO090038000097	ECS
n/a				Squalene cyclase	prot_ID_932	AO090038000098	
n/a				Domain(s) with predicted transferase activity, transferring acyl groups other than amino-acyl groups activity	prot_ID_213	AO090038000099	
n/a				Domain(s) with predicted ATP binding, ATPase activity, coupled to transmembrane movement of substances activity, role in transmembrane transport and integral to membrane localization	prot_ID_854	AO090038000100	ECS
n/a				Ortholog of A. niger CBS 513.88 : An13g03860, A. brasiliensis : Aspbr1_0128603, A. acidus : Aspfo1_0143176 and A. niger ATCC 1015 : 44928-mRNA	prot_ID_281	AO090038000101	

AO090038000149 cluster

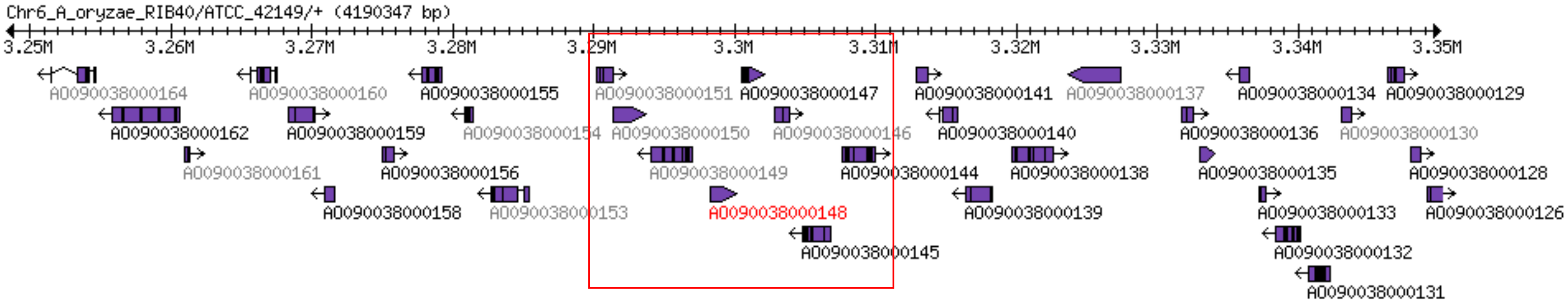

AO090038000149 cluster

Gene id (SMURF)	Gene positions	Gene distance	Gene Name	Annotated_gene_function	antiSMASH ID	AspGD ID	
n/a				Domain(s) with predicted carboxy-lyase activity, magnesium ion binding, thiamine pyrophosphate binding, transferase activity	prot_ID_179	AO090038000139	
n/a				Ortholog of A. nidulans FGSC A4 : AN3379, A. fumigatus Af293 : Afu7g01170, A. niger CBS 513.88 : An12g01970, An01g00420, A. oryzae RIB40 : AO090010000104 and A. niger ATCC 1015 : 50335-mRNA	prot_ID_1069	AO090038000140	
n/a				Domain(s) with predicted role in guanosine tetraphosphate metabolic process	prot_ID_350	AO090038000141	
AO090038000144	-5	781		Ortholog(s) have inorganic phosphate transmembrane transporter activity, manganese ion transmembrane transporter activity and role in manganese ion transport, phosphate ion transport, polyphosphate metabolic process	prot_ID_159	AO090038000144	
AO090038000145	-4	1097		Ortholog of A. flavus NRRL 3357 : AFL2T_08965, Neosartorya fischeri NRRL 181 : NFIA_040790, A. versicolor : Aspve1_0035582 and A. sydowii : Aspsy1_0040722	prot_ID_716	AO090038000145	IGD
AO090038000146	-3	867		Domain(s) with predicted N-acetyltransferase activity and role in metabolic process	prot_ID_544	AO090038000146	
AO090038000147	-2	454		Domain(s) with predicted role in transmembrane transport and integral to membrane localization	prot_ID_1123	AO090038000147	
AO090038000148	-1	1419		Domain(s) with predicted ATP binding, D-alanine-D-alanine ligase activity, metal ion binding activity	prot_ID_67	AO090038000148	
**AO090038000149**	0	0		Non-ribosomal peptide synthetase	prot_ID_1250	AO090038000149	
n/a				Protein of unknown function	prot_ID_1081	AO090038000150	
n/a				Protein of unknown function	prot_ID_383	AO090038000151	IGD
n/a				Protein of unknown function	prot_ID_543	AO090038000153	
n/a				Protein of unknown function	prot_ID_732	AO090038000154	
n/a				Ortholog of A. fumigatus Af293 : Afu8g01790, A. niger CBS 513.88 : An06g00490, A. brasiliensis : Aspbr1_0053332, A. flavus NRRL 3357 : AFL2T_08968 and A. acidus : Aspfo1_0146077	prot_ID_22	AO090038000155	
n/a				Ortholog(s) have IgE binding, ribonuclease activity and role in regulation of cytokine activity	prot_ID_1000	AO090038000156	

AO090038000210 cluster

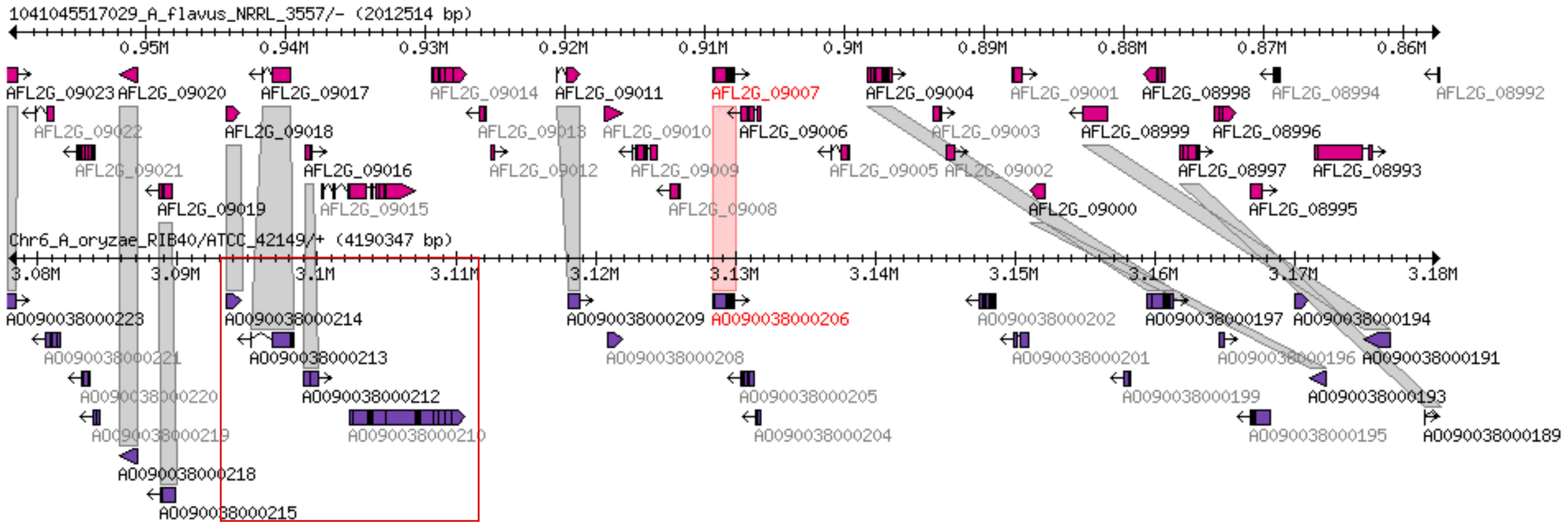

AO090038000210 cluster

Gene id (SMURF)	Gene positions	Gene distance	Gene Name	Annotated_gene_function	antiSMASH ID	AspGD ID	
n/a				Protein of unknown function	prot_ID_651	AO090038000205	
n/a			CYP58F1	Cytochrome P450 monooxygenase; upregulated by exposure to benomyl	prot_ID_136	AO090038000206	
n/a				Protein of unknown function	prot_ID_199	AO090038000208	
n/a				Ortholog of A. flavus NRRL 3357 : AFL2T_09011 and A. aculeatus ATCC16872 : Aacu16872_048001	prot_ID_406	AO090038000209	
n/a				Polyketide synthase	prot_ID_1064	AO090038000210	FA
n/a				Ortholog of A. nidulans FGSC A4 : AN9006, A. fumigatus Af293 : Afu3g02540, Afu6g11710, Afu7g08440, Afu5g00145, A. niger CBS 513.88 : An09g05330 and N. fischeri NRRL 181 : NFIA_004090, NFIA_057380	prot_ID_223	AO090038000212	
n/a				Domain(s) with predicted N,N-dimethylaniline monooxygenase activity, NADP binding, flavin adenine dinucleotide binding activity and role in oxidation-reduction process	prot_ID_774	AO090038000213	
n/a				Domain(s) with predicted triglyceride lipase activity and role in lipid metabolic process	prot_ID_86	AO090038000214	ECS, IGD
n/a				Ortholog of A. flavus NRRL 3357 : AFL2T_09019	prot_ID_1025	AO090038000215	
n/a				domain(s) with predicted coenzyme binding, hydroxymethylglutaryl-CoA reductase (NADPH) activity and role in coenzyme A metabolic process, oxidation-reduction process	prot_ID_366	AO090038000218	
n/a				Protein of unknown function	prot_ID_1254	AO090038000219	
n/a				Protein of unknown function	prot_ID_668	AO090038000220	

AO090038000390 cluster

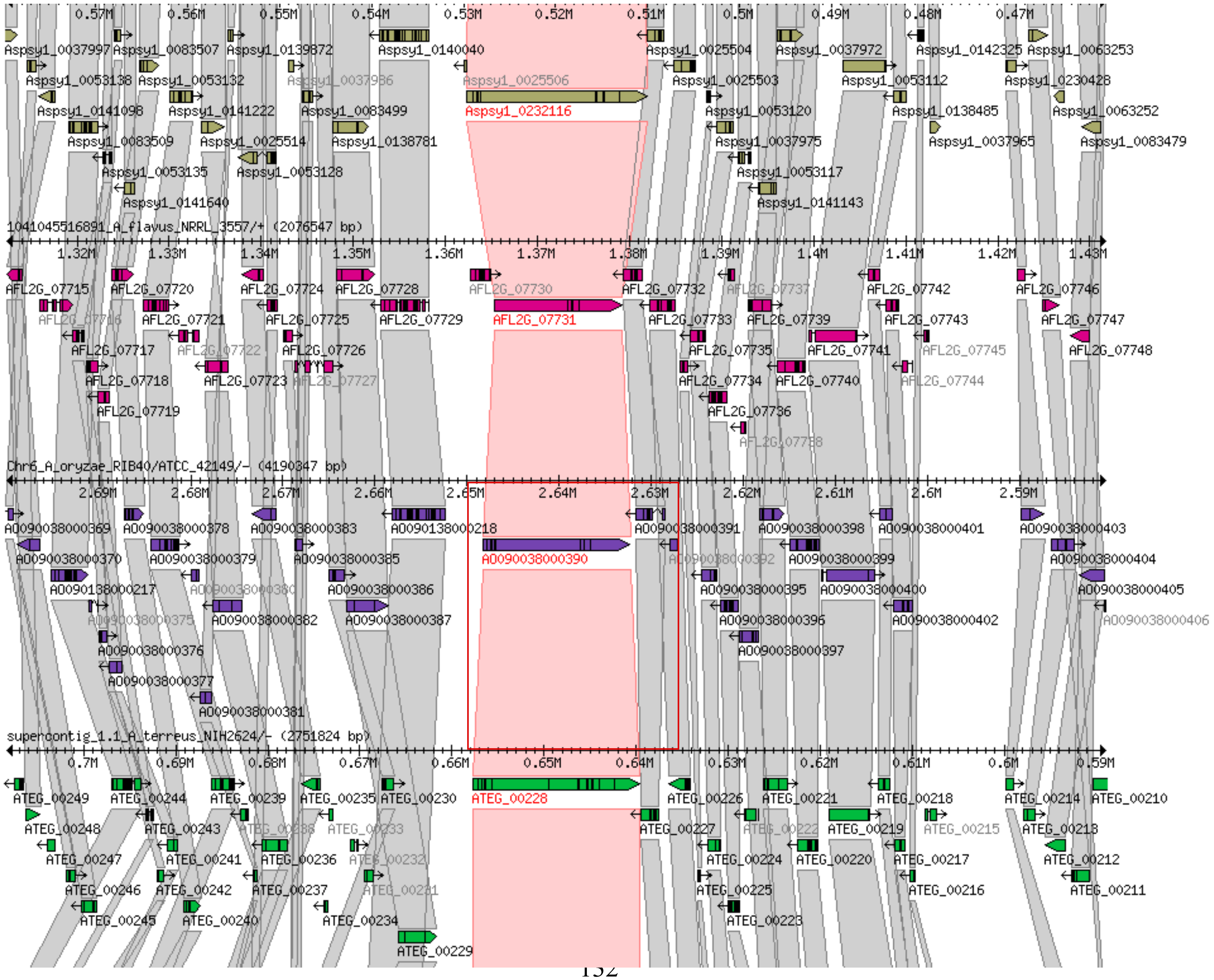

AO090038000390 cluster

Gene id (SMURF)	Gene positions	Gene distance	Annotated_gene_function	antiSMASH ID	AspGD ID	
n/a			Ortholog of A. flavus NRRL 3357 : AFL2T_07726	prot_ID_775	AO090038000385	
n/a			Ortholog(s) have DNA-dependent ATPase activity, recombinase activity, single-stranded DNA binding activity	prot_ID_238	AO090038000386	
n/a			Ortholog(s) have nuclear pore, spindle pole body localization	prot_ID_887	AO090038000387	
n/a			Domain(s) with predicted ATP binding, ATPase activity, coupled to transmembrane movement of substances activity, role in transmembrane transport and integral to membrane localization	prot_ID_1277	AO090138000218	
**AO090038000390**	0	0	Putative non-ribosomal peptide synthetase; alternative polyadenylation sites predicted	prot_ID_1092	AO090038000390	ECS
AO090038000391	1	568	Domain(s) with predicted role in transmembrane transport and integral to membrane localization	prot_ID_995	AO090038000391	
AO090038000392	2	500	Protein of unknown function	prot_ID_449	AO090038000392	FA
AO090038000395	3	2726	Predicted 3-phosphoglycerate kinase with role in glycolysis	prot_ID_97	AO090038000395	
AO090038000396	4	830	Ortholog(s) have fungal-type vacuole localization	prot_ID_816	AO090038000396	
AO090038000397	5	690	Ortholog of A. fumigatus Af293 : Afu1g10330, A. niger CBS 513.88 : An08g02230, A. niger ATCC 1015 : 208323-mRNA, A. versicolor : Aspve1_0079224 and A. sydowii : Aspsy1_0141143	prot_ID_272	AO090038000397	
AO090038000398	6	290	Ortholog(s) have RNA binding activity, role in mRNA splicing, via spliceosome and U1 snRNP, U2-type prespliceosome localization	prot_ID_372	AO090038000398	
AO090038000399	7	1310	Ortholog(s) have iron ion binding activity, role in ribosomal large subunit biogenesis, ribosomal subunit export from nucleus, translational initiation, translational termination and cytosolic ribosome, nucleus localization	prot_ID_909	AO090038000399	

AO090038000488 cluster

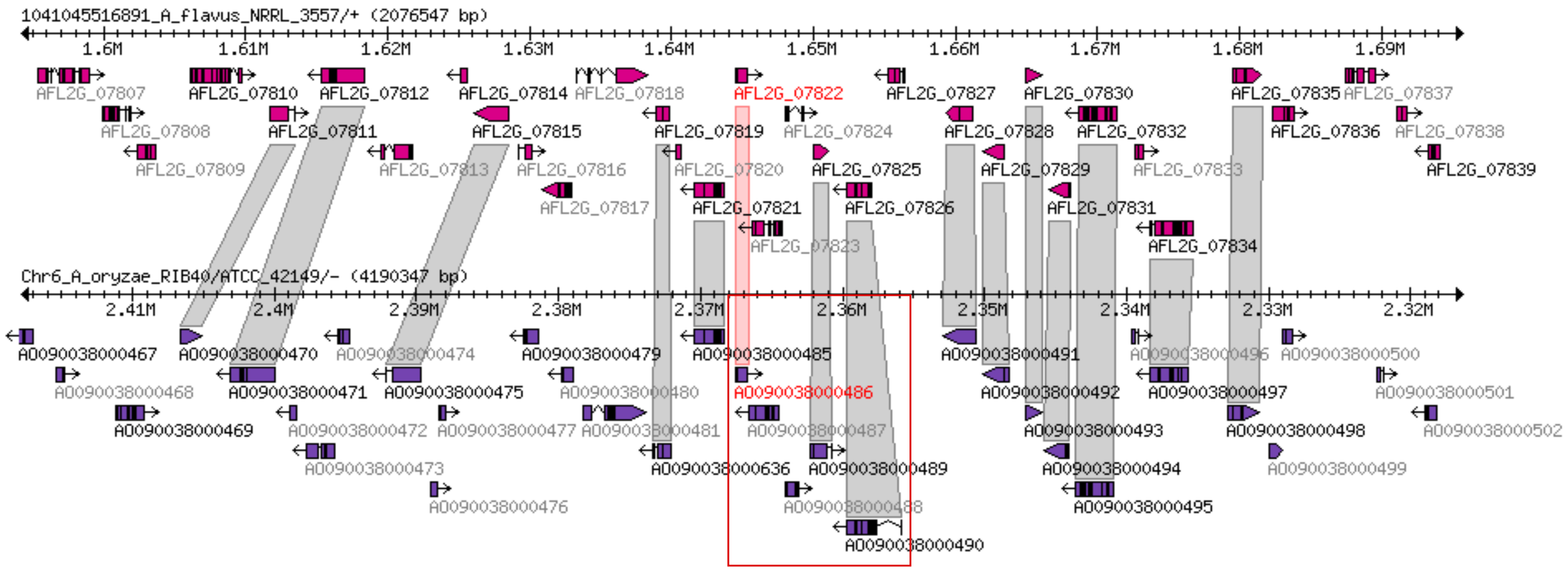

AO090038000488 cluster

Gene id (SMURF)	Gene positions	Gene distance	Gene Name	Annotated_gene_function	antiSMASH ID	AspGD ID	
n/a				Protein of unknown function	prot_ID_703	AO090038000480	
n/a				Protein of unknown function	prot_ID_82	AO090038000481	
n/a				Ortholog of A. niger CBS 513.88 : An07g00200, A. brasiliensis : Aspbr1_0211901, A. flavus NRRL 3357 : AFL2T_07819 and A. acidus : Aspfo1_0152286	prot_ID_1274	AO090038000483.1	
n/a				Ortholog(s) have enterobactin transporter activity and role in iron ion transport	prot_ID_704	AO090038000485	
n/a				Ortholog(s) have hydrolase activity, acting on ester bonds activity, role in iron assimilation and cytoplasm localization	prot_ID_163	AO090038000486	FA
n/a				Domain(s) with predicted catalytic activity and role in metabolic process	prot_ID_848	AO090038000487	
n/a			csyC	Putative type III polyketide synthase	prot_ID_282	AO090038000488	
n/a				Ortholog of A. nidulans FGSC A4 : AN8222, A. flavus NRRL 3357 : AFL2T_07825, A. versicolor : Aspve1_0047207 and A. sydowii : Aspsy1_0050689	prot_ID_922	AO090038000489	
n/a				Domain(s) with predicted amino acid transmembrane transporter activity, role in amino acid transmembrane transport and membrane localization	prot_ID_57	AO090038000490	IGD
n/a				Domain(s) with predicted identical protein binding activity	prot_ID_744	AO090038000491	
n/a				Ortholog of A. fumigatus Af293 : Afu1g01420, A. niger CBS 513.88 : An01g01980, An14g00460, A. oryzae RIB40 : AO090138000165 and A. niger ATCC 1015 : 36356-mRNA, 41492-mRNA	prot_ID_127	AO090038000492	

AO090038000543 cluster

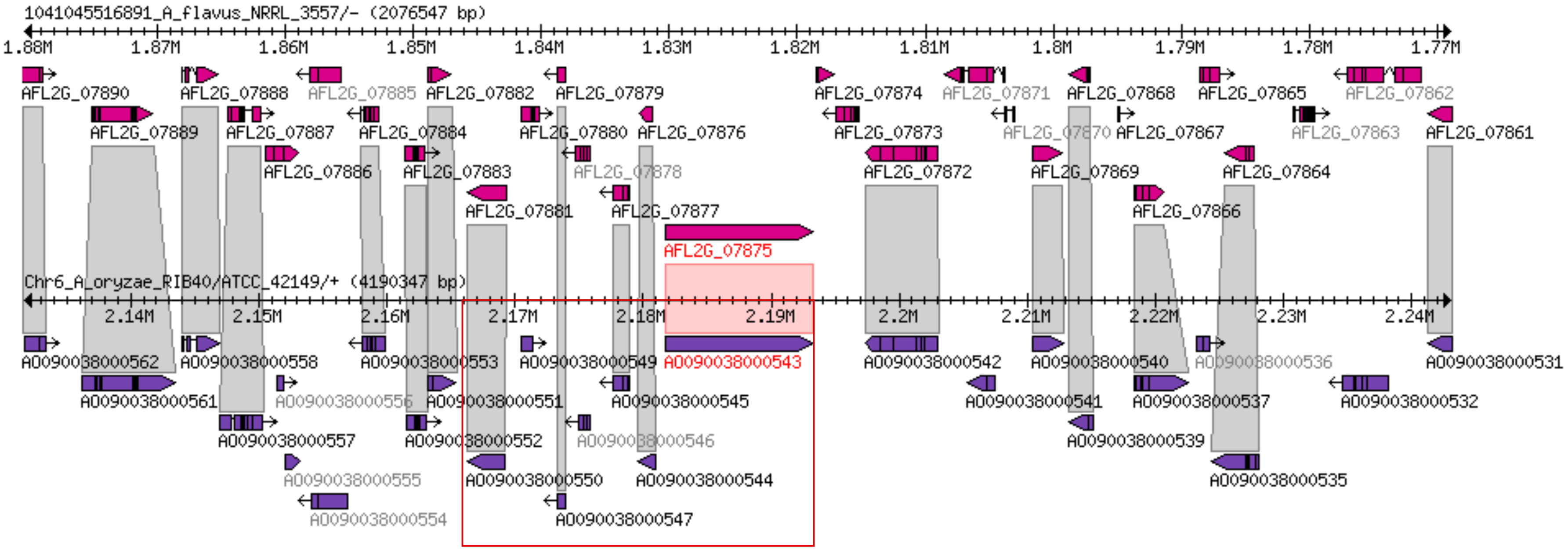

AO090038000543 cluster

Gene id (SMURF)	Gene positions	Gene distance	Gene Name	Annotated_gene_function	antiSMASH ID	AspGD ID	
n/a				Ortholog of A. nidulans FGSC A4 : AN9130, A. fumigatus Af293 : Afu7g01710, A. niger CBS 513.88 : An12g01000, A. niger ATCC 1015 : 43664-mRNA and A. versicolor : Aspve1_0144405 (2)	prot_ID_945	AO090038000540	
n/a				3-hydroxy-3-methylglutaryl-coenzyme A lyase/3 methylglutaconyl coenzyme A hydratase; predominantly expressed in the basal region of hyphae	prot_ID_957	AO090038000541	
n/a				Ortholog of A. nidulans FGSC A4 : AN8562, A. fumigatus Af293 : Afu4g01580, A. niger CBS 513.88 : An12g00890, A. niger ATCC 1015 : 43655-mRNA and A. versicolor : Aspve1_0142203	prot_ID_761	AO090038000542	
**AO090038000543**	0	0		Putative nonribosomal peptide synthase (NRPS)	prot_ID_227	AO090038000543	ECS, IGD
AO090038000544	1	1026		Ortholog(s) have isopenicillin-N synthase activity, manganese ion binding activity and role in penicillin biosynthetic process	prot_ID_670	AO090038000544	
AO090038000545	2	1058		Ortholog(s) have isopenicillin-N N-acyltransferase activity and role in penicillin biosynthetic process	prot_ID_153	AO090038000545	
AO090038000546	3	1855		Protein of unknown function	prot_ID_1061	AO090038000546	
AO090038000547	4	1068		Ortholog of A. flavus NRRL 3357 : AFL2T_07879	prot_ID_512	AO090038000547	
AO090038000549	5	1995		7-keto-8-aminopelargonate synthetase	prot_ID_444	AO090038000549	
AO090038000550	6	1254		Protein similar to nonribosomal peptide synthases (NRPS-like)	prot_ID_1103	AO090038000550	FA
AO090038000551	7	971		Domain(s) with predicted transmembrane transporter activity, role in transmembrane transport and integral to membrane localization	prot_ID_841	AO090038000551	
AO090038000552	8	271		Ortholog(s) have rhamnogalacturonan alpha-L-rhamnopyranosyl-(1->4)-alpha-D-galactopyranosyluronide lyase activity and role in pectin catabolic process	prot_ID_961	AO090038000552	
AO090038000553	9	1896		Ortholog(s) have role in sulfite transport	prot_ID_395	AO090038000553	
AO090038000554	10	1635		Domain(s) with predicted DNA binding activity	prot_ID_1136	AO090038000554	
AO090038000555	11	956		Domain(s) with predicted DNA binding, zinc ion binding activity, role in transcription, DNA-dependent and nucleus localization	prot_ID_577	AO090038000555	
AO090038000556	12	77		Protein of unknown function	prot_ID_667	AO090038000556	
AO090038000557	13	1141		Domain(s) with predicted DNA binding, sequence-specific DNA binding RNA polymerase II transcription factor activity, zinc ion binding activity and role in regulation of transcription, DNA-dependent, transcription, DNA-dependent	prot_ID_149	AO090038000557	
n/a				Ortholog(s) have UTP:glucose-1-phosphate uridylyltransferase activity and role in (1->6)-beta-D-glucan biosynthetic process, UDP-glucose metabolic process, glycogen biosynthetic process, trehalose biosynthetic process	prot_ID_836	AO090038000558	

AO090102000166 cluster

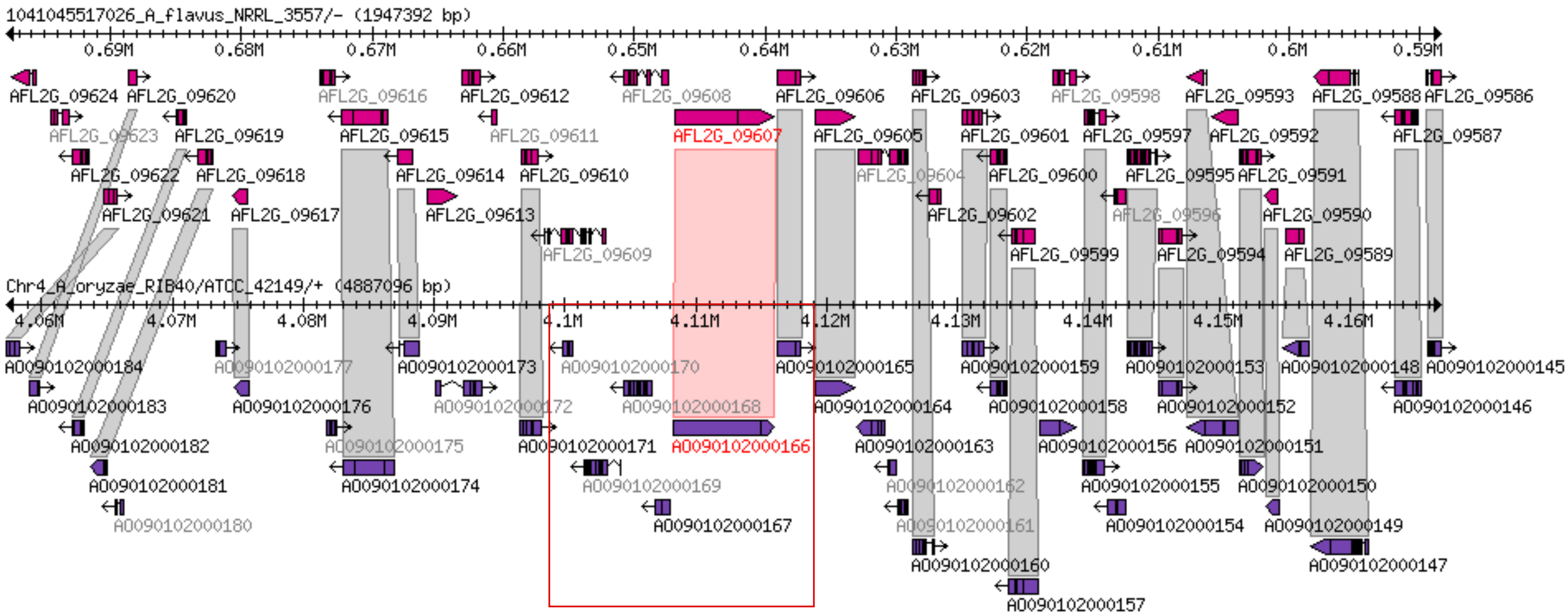

AO090102000166 cluster

Gene id (SMURF)	Gene positions	Gene distance	Gene Name	Annotated_gene_function	antiSMASH ID	AspGD ID	
n/a				Ortholog(s) have tRNA adenylyltransferase activity, role in tRNA 3'-terminal CCA addition and mitochondrial matrix localization	prot_ID_1085	AO090102000157	
n/a				Domain(s) with predicted catalytic activity	prot_ID_265	AO090102000158	
n/a				Domain(s) with predicted catalytic activity and role in trehalose biosynthetic process	prot_ID_855	AO090102000159	
n/a				Domain(s) with predicted calcium ion binding activity	prot_ID_352	AO090102000160	
n/a				Domain(s) with predicted nucleotide binding, oxidoreductase activity	prot_ID_1058	AO090102000161	
n/a				Protein of unknown function	prot_ID_161	AO090102000162	
n/a				Ortholog of A. nidulans FGSC A4 : AN10927, A. fumigatus Af293 : Afu2g16650, A. niger CBS 513.88 : An04g08670 and A. terreus NIH2624 : ATET_02265	prot_ID_124	AO090102000163	
n/a				Ortholog(s) have phosphatidylinositol-3,5-bisphosphate 5-phosphatase activity, role in phosphatidylinositol dephosphorylation and PAS complex, extrinsic to membrane, fungal-type vacuole membrane localization	prot_ID_1318	AO090102000164	
n/a				Domain(s) with predicted catalytic activity and role in metabolic process	prot_ID_721	AO090102000165	FA
**AO090102000166**	0	0		Polyketide synthase	prot_ID_1094	AO090102000166	
AO090102000167	1	615		Ortholog of A. niger CBS 513.88 : An14g03760, A. brasiliensis : Aspbr1_0024828, Aspergillus acidus : Aspfo1_0209024 and A. niger ATCC 1015 : 41772-mRNA	prot_ID_1244	AO090102000167	
AO090102000168	2	828		Cytochrome P450 monooxygenase	prot_ID_474	AO090102000168	
AO090102000169	3	94		Domain(s) with predicted role in transmembrane transport and integral to membrane localization	prot_ID_338	AO090102000169	
n/a				Protein of unknown function	prot_ID_260	AO090102000170	FA
n/a				Domain(s) with predicted mannan endo-1,6-alpha-mannosidase activity and role in carbohydrate catabolic process	prot_ID_873	AO090102000171	
n/a				Acyl-CoA dehydrogenase	prot_ID_67	AO090102000172	
n/a				Domain(s) with predicted oxidoreductase activity and role in oxidation-reduction process	prot_ID_1069	AO090102000173	
n/a				DNA polymerase delta, catalytic subunit	n/a	AO090102000174	

AO090102000322 cluster

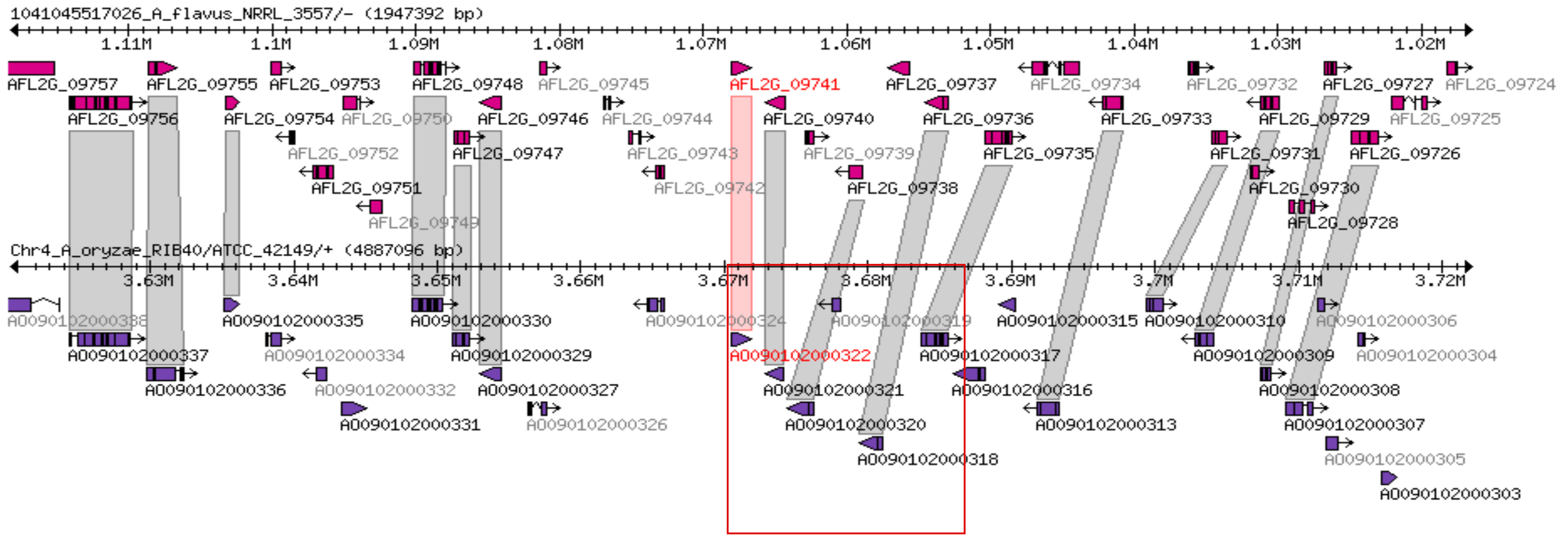

AO090102000322 cluster

Gene id (SMURF)	Gene positions	Gene distance	Gene Name	Annotated_gene_function	antiSMASH ID	AspGD ID	
AO090102000316	-6	1368		Domain(s) with predicted nucleotide binding, oxidoreductase activity, zinc ion binding activity and role in oxidation-reduction process	n/a	AO090102000316	
AO090102000317	-5	2640		Domain(s) with predicted transporter activity, role in transmembrane transport and integral to membrane localization	n/a	AO090102000317	IGD
AO090102000318	-4	1275		Domain(s) with predicted transferase activity, transferring phosphorus-containing groups activity	prot_ID_126	AO090102000318	
AO090102000319	-3	2092		Protein of unknown function	prot_ID_1065	AO090102000319	
AO090102000320	-2	510		Ortholog of A. flavus NRRL 3357 : AFL2T_09738	prot_ID_1158	AO090102000320	
AO090102000321	-1	931		Domain(s) with predicted transferase activity, transferring phosphorus-containing groups activity	prot_ID_522	AO090102000321	
**AO090102000322**	0	0		Dimethylallyl tryptophan synthase (DMAT)	prot_ID_1367	AO090102000322	ECS, IGD
n/a				Protein of unknown function	prot_ID_44	AO090102000324	

AO090102000338 cluster
No match to clusters in other species

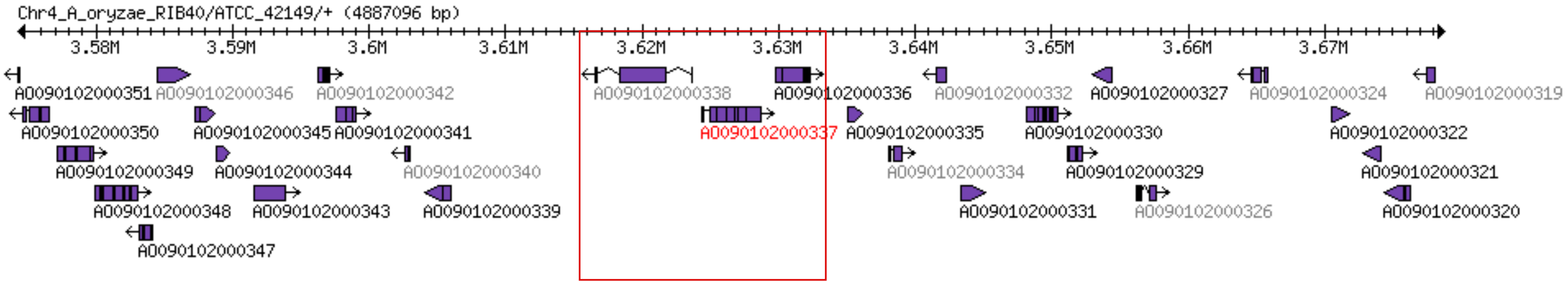

AO090102000338 cluster

Gene id (SMURF)	Gene positions	Gene distance	Gene Name	Annotated_gene_function	antiSMASH ID	AspGD ID	
AO090102000329	-8	794		Domain(s) with predicted nucleotide binding, oxidoreductase activity, zinc ion binding activity and role in oxidation-reduction process	n/a	AO090102000329	
AO090102000330	-7	3197		Cytochrome P450 monooxygenase; involved in 7-hydroxycoumarin production	n/a	AO090102000330	
AO090102000331	-6	1025		Domain(s) with predicted hydrolase activity, hydrolyzing O-glycosyl compounds activity and role in carbohydrate metabolic process	prot_ID_820	AO090102000331	
AO090102000332	-5	2750		Protein of unknown function	prot_ID_568	AO090102000332	
AO090102000334	-4	1952		Protein of unknown function	prot_ID_424	AO090102000334	
AO090102000335	-3	2892		Domain(s) with predicted phospholipase D activity and role in lipid metabolic process	prot_ID_162	AO090102000335	
AO090102000336	-2	1021		Domain(s) with predicted catalytic activity	prot_ID_959	AO090102000336	FA
AO090102000337	-1	622		Domain(s) with predicted ATP binding, ATPase activity, coupled to transmembrane movement of substances activity, role in transmembrane transport and integral to membrane localization	prot_ID_664	AO090102000337	
**AO090102000338**	0	0		Non-ribosomal peptide synthetase	prot_ID_746	AO090102000338	ECS, IGD
n/a				Ortholog(s) have role in unsaturated fatty acid biosynthetic process	prot_ID_390	AO090102000339	
n/a				Protein of unknown function	prot_ID_987	AO090102000340	
n/a				Domain(s) with predicted hydrolase activity, hydrolyzing O-glycosyl compounds activity and role in carbohydrate metabolic process	prot_ID_1137	AO090102000341	
n/a				Protein of unknown function	prot_ID_696	AO090102000342	

AO090102000465 cluster

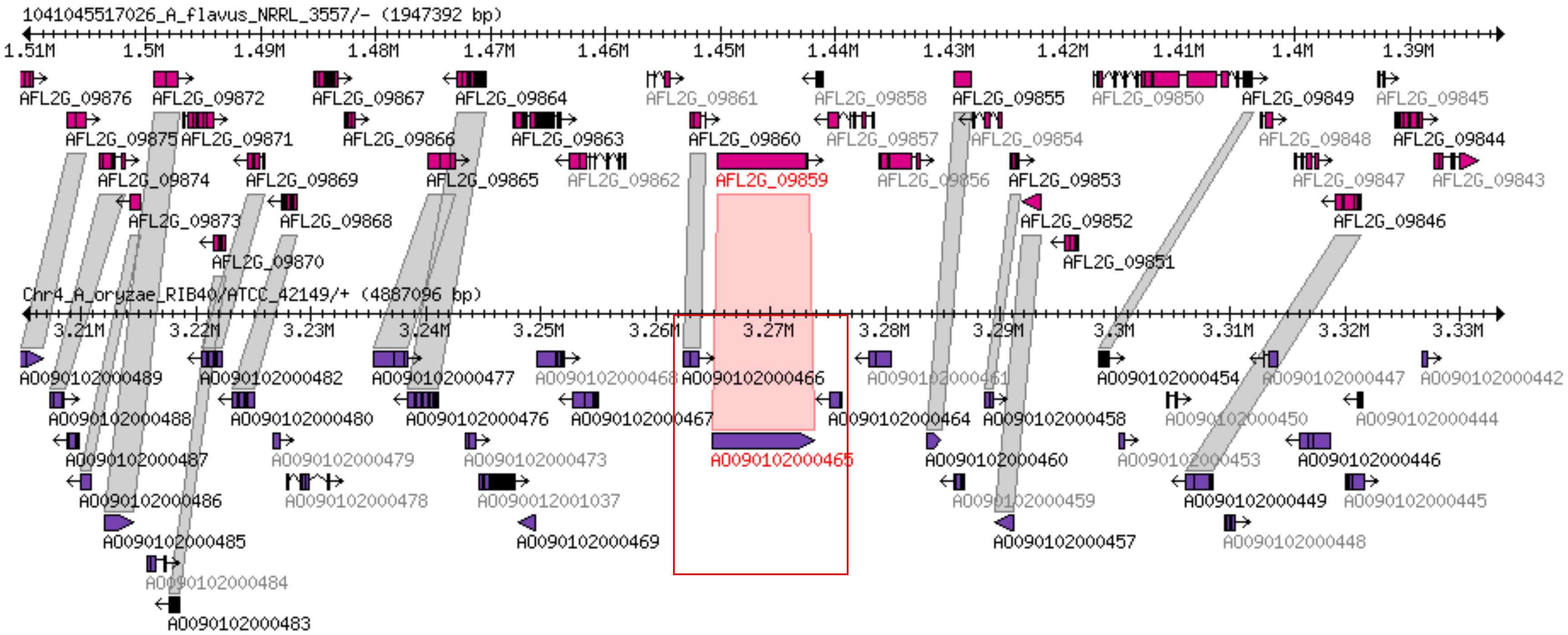

AO090102000465 cluster

Gene id (SMURF)	Gene positions	Gene distance	Gene Name	Annotated_gene_function	antiSMASH ID	AspGD ID	
AO090102000457	-6	86		Domain(s) with predicted UDP-N-acetylmuramate dehydrogenase activity, flavin adenine dinucleotide binding activity and role in oxidation-reduction process	prot_ID_334	AO090102000457	
AO090102000458	-5	1723		Ortholog of A. flavus NRRL 3357 : AFL2T_09853	prot_ID_240	AO090102000458	
AO090102000459	-4	977		Protein of unknown function	prot_ID_1073	AO090102000459	
AO090102000460	-3	3076		Ortholog of A. fumigatus Af293 : Afu8g00530, A. niger CBS 513.88 : An07g00020, An13g03240, A. oryzae RIB40 : AO090009000132, AO090026000248 and A. versicolor : Aspve1_0038561, Aspve1_0043401	prot_ID_1005	AO090102000460	
AO090102000461	-2	2563		Domain(s) with predicted catalytic activity and role in nucleoside metabolic process	prot_ID_357	AO090102000461	
AO090102000464	-1	1215		Domain(s) with predicted oxidoreductase activity and role in oxidation-reduction process	prot_ID_39	AO090102000464	FA
**AO090102000465**	0	0		Non-ribosomal peptide synthetase	prot_ID_827	AO090102000465	
AO090102000466	1	925		Domain(s) with predicted electron carrier activity, heme binding, oxidoreductase activity, acting on paired donors, with incorporation or reduction of molecular oxygen activity and role in oxidation-reduction process	prot_ID_1366	AO090102000466	ECS, IGD
n/a				Ortholog of A. niger CBS 513.88 : An03g03670, A. brasiliensis : Aspbr1_0197056, A. clavatus NRRL 1 : ACLA_097300, A. acidus : Aspfo1_0142332 and A. niger ATCC 1015 : 54686-mRNA	prot_ID_712	AO090102000467	
n/a				Protein of unknown function	prot_ID_512	AO090102000468	
n/a				Ortholog of A. versicolor : Aspve1_0042194 and A. sydowii : Aspsy1_0030438 (2)	prot_ID_1008	AO090102000469	
n/a				Domain(s) with predicted NAD(P)+ transhydrogenase (AB-specific) activity, NADP binding activity, role in oxidation-reduction process and integral to membrane localization	prot_ID_1488	AO090012001037	

AO090103000167 cluster

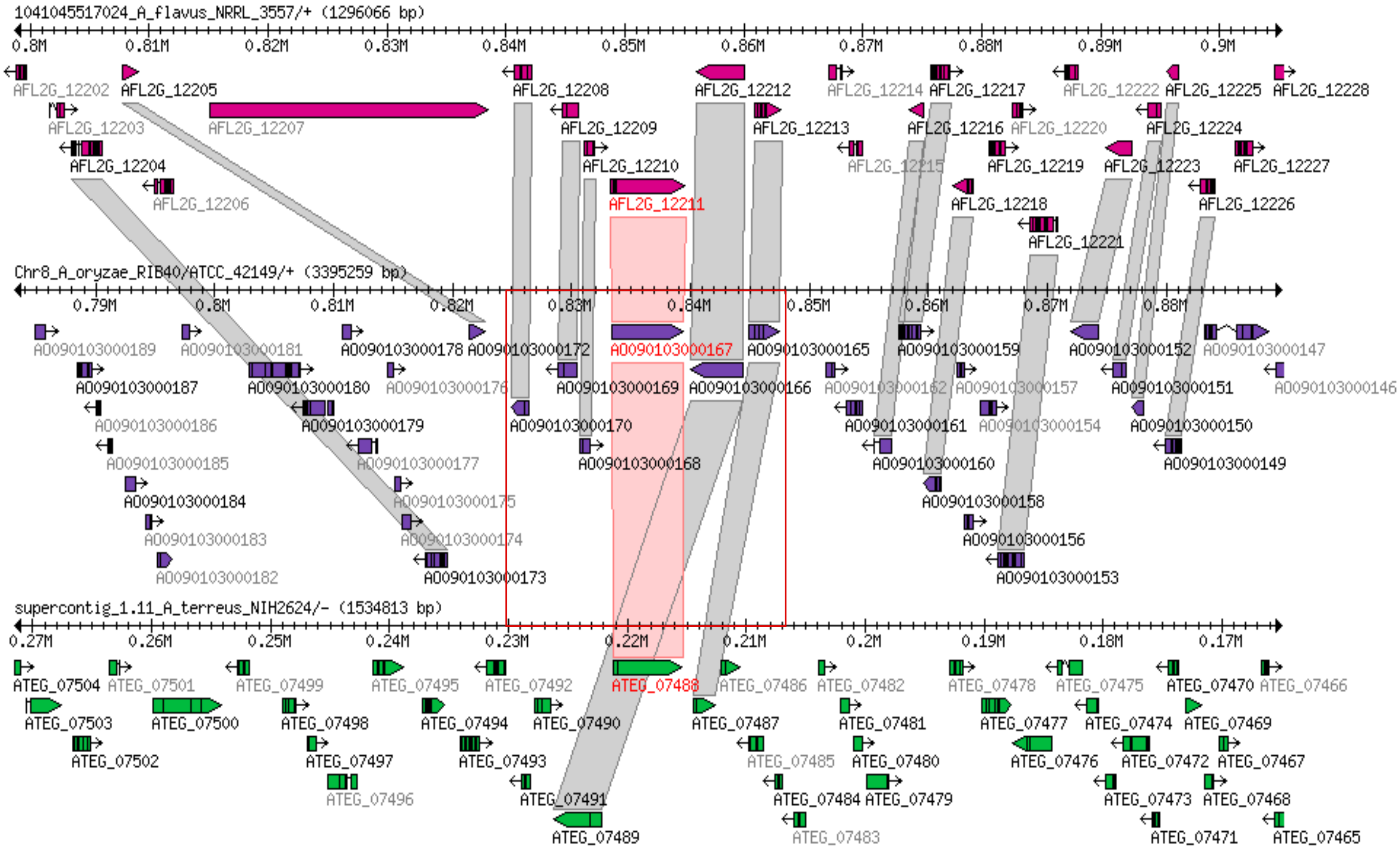

AO090103000167 cluster

Gene id (SMURF)	Gene positions	Gene distance	Gene Name	Annotated_gene_function	antiSMASH ID	AspGD ID	
n/a				Domain(s) with predicted role in transmembrane transport and integral to membrane localization	prot_ID_353	AO090103000159	
n/a				Domain(s) with predicted hydrolase activity	prot_ID_199	AO090103000160	
n/a				Domain(s) with predicted ATP binding, protein kinase activity and role in protein phosphorylation (2)	prot_ID_682	AO090103000161	
				Protein of unknown function	prot_ID_863	AO090103000162	
AO090103000165	-2	999		Ortholog(s) have role in ferric triacetylfusarinine C transport	prot_ID_133	AO090103000165	ECS,IGD
AO090103000166	-1	828		Domain(s) with predicted ATP binding, ATPase activity, coupled to transmembrane movement of substances activity, role in transmembrane transport and integral to membrane localization	prot_ID_246	AO090103000166	
**AO090103000167**	0	0		Non-ribosomal peptide synthetase	prot_ID_711	AO090103000167	
AO090103000168	1	1954		Ortholog(s) have role in N',N'',N'''-triacetylfusarinine C biosynthetic process, cellular response to hydrogen peroxide, cellular response to iron ion starvation, ergosterol biosynthetic process, pathogenesis	prot_ID_878	AO090103000168	
AO090103000169	2	317		Ortholog(s) have role in ergosterol biosynthetic process, siderophore biosynthetic process	prot_ID_300	AO090103000169	
AO090103000170	3	2813		Ortholog of A. nidulans FGSC A4 : AN6239, AN6793, A. fumigatus Af293 : Afu3g03390, Afu7g00330, A. niger CBS 513.88 : An03g03530, A. oryzae RIB40 : AO090701000114 and A. niger ATCC 1015 : 50657-mRNA	prot_ID_642	AO090103000170	ECS
AO090103000172	4	2250		Domain(s) with predicted triglyceride lipase activity and role in lipid catabolic process	prot_ID_1005	AO090103000172	
AO090103000173	5	1882		Domain(s) with predicted electron carrier activity, heme binding, iron ion binding, monooxygenase activity, oxidoreductase activity, acting on paired donors, with incorporation or reduction of molecular oxygen activity	prot_ID_48	AO090103000173	
AO090103000174	6	1194		Protein of unknown function	prot_ID_461	AO090103000174	
AO090103000175	7	123		Protein of unknown function	prot_ID_1050	AO090103000175	
AO090103000176	8	58		Protein of unknown function	prot_ID_306	AO090103000176	
AO090103000177	9	866		Domain(s) with predicted potassium channel activity, role in potassium ion transmembrane transport and membrane localization	prot_ID_840	AO090103000177	
AO090103000178	10	650		Domain(s) with predicted O-methyltransferase activity	n/a	AO090103000178	
AO090103000179	11	718		Domain(s) with predicted DNA binding, sequence-specific DNA binding RNA polymerase II transcription factor activity, zinc ion binding activity and role in regulation of transcription, DNA-dependent, transcription, DNA-dependent	n/a	AO090103000179	

AO090103000224 cluster

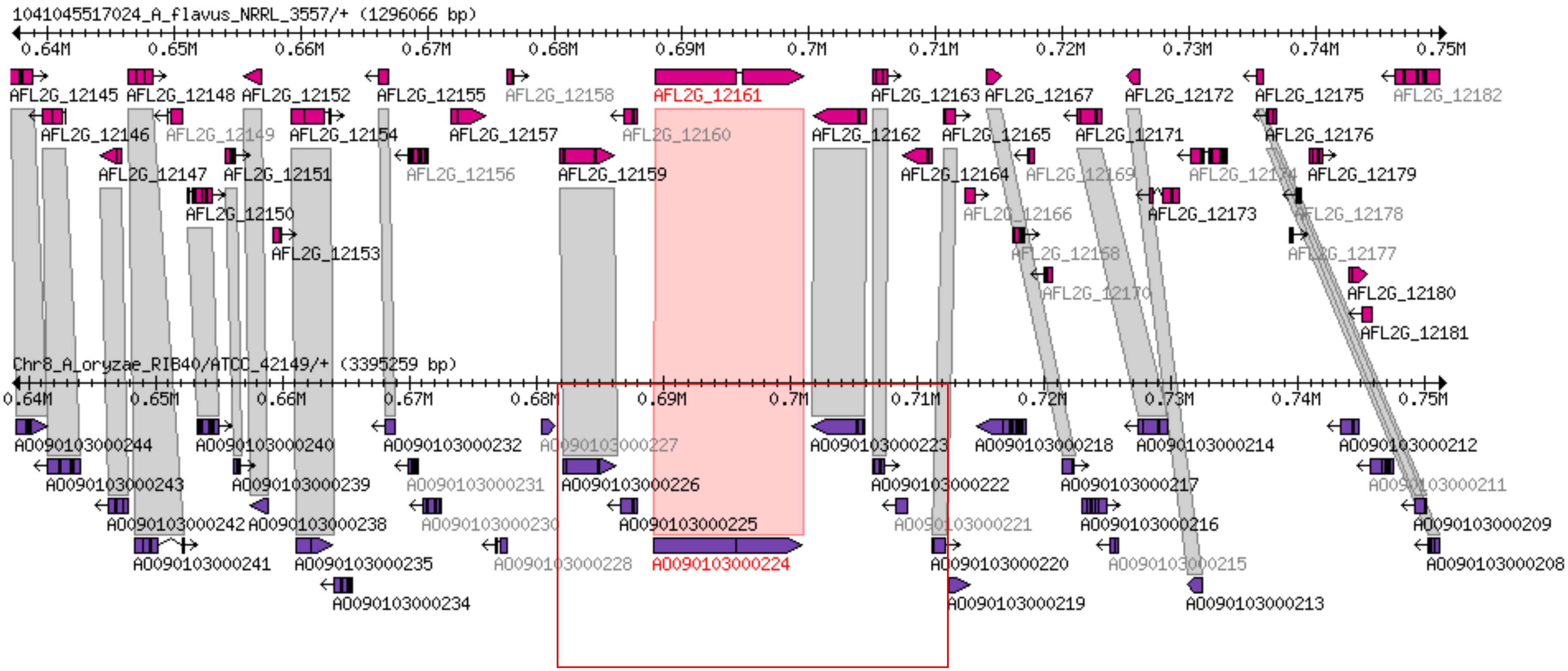

AO090103000224 cluster

Gene id (SMURF)	Gene positions	Gene distance	Gene Name	Annotated_gene_function	antiSMASH ID	AspGD ID	
n/a				Protein of unknown function	prot_ID_949	AO090103000215	
n/a				Domain(s) with predicted role in cell wall macromolecule catabolic process	prot_ID_450	AO090103000216	
n/a				Ortholog of A. flavus NRRL 3357 : AFL2T_12167, A. acidus : Aspfo1_0058137, A. terreus NIH2624 : ATET_09175 and A. carbonarius ITEM 5010 : Acar5010_203830	prot_ID_1057	AO090103000217	
n/a				Domain(s) with predicted cation binding, chitin binding, chitinase activity and role in carbohydrate metabolic process, chitin catabolic process	prot_ID_607	AO090103000218	
n/a				Ortholog of A. sydowii : Aspsy1_0046555	prot_ID_135	AO090103000219	
n/a				Domain(s) with predicted nucleotide binding, oxidoreductase activity, transferase activity, transferring acyl groups other than amino-acyl groups, zinc ion binding activity and role in oxidation-reduction process	prot_ID_973	AO090103000220	ECS, FA
n/a				Protein of unknown function	prot_ID_486	AO090103000221	
AO090103000222	-2	554		Ortholog(s) have role in endocrocin biosynthetic process	prot_ID_931	AO090103000222	
AO090103000223	-1	732		Non-ribosomal peptide synthetase	prot_ID_365	AO090103000223	
**AO090103000224**	0	0		Polyketide synthase	prot_ID_141	AO090103000224	
AO090103000225	1	1360		Isopenicillin N synthase and related dioxygenases family protein	prot_ID_560	AO090103000225	
AO090103000226	2	387		Ortholog(s) have plasma membrane localization	prot_ID_93	AO090103000226	ECS
AO090103000227	3	968		Domain(s) with predicted N-acetyltransferase activity and role in metabolic process	prot_ID_979	AO090103000227	
n/a				Protein of unknown function	prot_ID_503	AO090103000228	
n/a				Protein of unknown function	prot_ID_1036	AO090103000230	
n/a				Protein of unknown function	prot_ID_289	AO090103000231	

AO090103000355 cluster

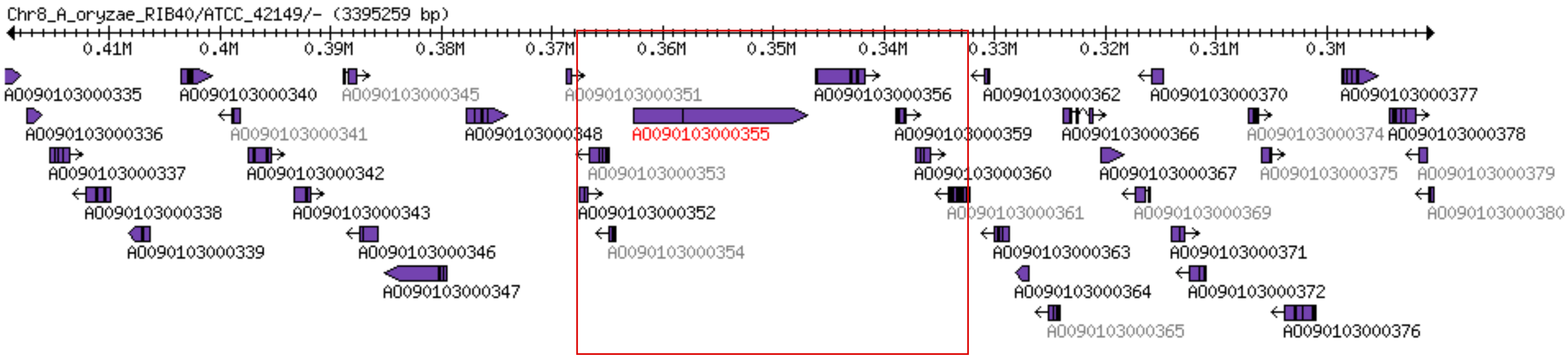

AO090103000355 cluster

Gene id (SMURF)	Gene positions	Gene distance	Gene Name	Annotated_gene_function	antiSMASH ID	AspGD ID	
n/a				Ortholog(s) have DNA topoisomerase (ATP-hydrolyzing) activity	prot_ID_505	AO090103000347	
n/a				Domain(s) with predicted Rab GTPase activator activity, role in regulation of Rab GTPase activity and intracellular localization	prot_ID_94	AO090103000348	
n/a				Protein of unknown function	prot_ID_1061	AO090103000351	
n/a				Ortholog(s) have role in asperfuranone biosynthetic process	prot_ID_474	AO090103000352	FA
n/a				Domain(s) with predicted asparagine synthase (glutamine-hydrolyzing) activity and role in asparagine biosynthetic process	prot_ID_100	AO090103000353	
n/a				Protein of unknown function	prot_ID_583	AO090103000354	
**AO090103000355**	0	0		Non-ribosomal peptide synthetase	prot_ID_24	AO090103000355	
AO090103000356	1	724		Domain(s) with predicted ATP binding, ATPase activity, coupled to transmembrane movement of substances activity, role in transmembrane transport and integral to membrane localization	prot_ID_835	AO090103000356	
AO090103000359	2	2696		Putative reductase	prot_ID_593	AO090103000359	
AO090103000360	3	987		Domain(s) with predicted role in lipid metabolic process	prot_ID_792	AO090103000360	
AO090103000361	4	1541		Domain(s) with predicted N,N-dimethylaniline monooxygenase activity, NADP binding, flavin adenine dinucleotide binding activity and role in oxidation-reduction process	prot_ID_192	AO090103000361	FA
AO090103000362	5	1221		Ortholog of A. nidulans FGSC A4 : AN5401, A. fumigatus Af293 : Afu6g13860, A. niger CBS 513.88 : An08g08500, A. niger ATCC 1015 : 38052-mRNA and A. versicolor : Aspve1_0045294	prot_ID_280	AO090103000362	
AO090103000363	6	445		Ortholog of A. niger CBS 513.88 : An14g02060, A. oryzae RIB40 : AO090012000688, A. niger ATCC 1015 : 41629-mRNA and A. terreus NIH2624 : ATET_08602, ATET_08920	prot_ID_1025	AO090103000363	
AO090103000364	7	676		Ortholog of A. flavus NRRL 3357 : AFL2T_12033 and A. fumigatus A1163 : AFUB_102210	prot_ID_435	AO090103000364	
AO090103000365	8	1796		Protein of unknown function	n/a	AO090103000365	
AO090103000366	9	395		Domain(s) with predicted nucleotide binding, oxidoreductase activity and role in oxidation-reduction process	n/a	AO090103000366	

AO090113000209 cluster
No cluster prediction made, end of genome sequence

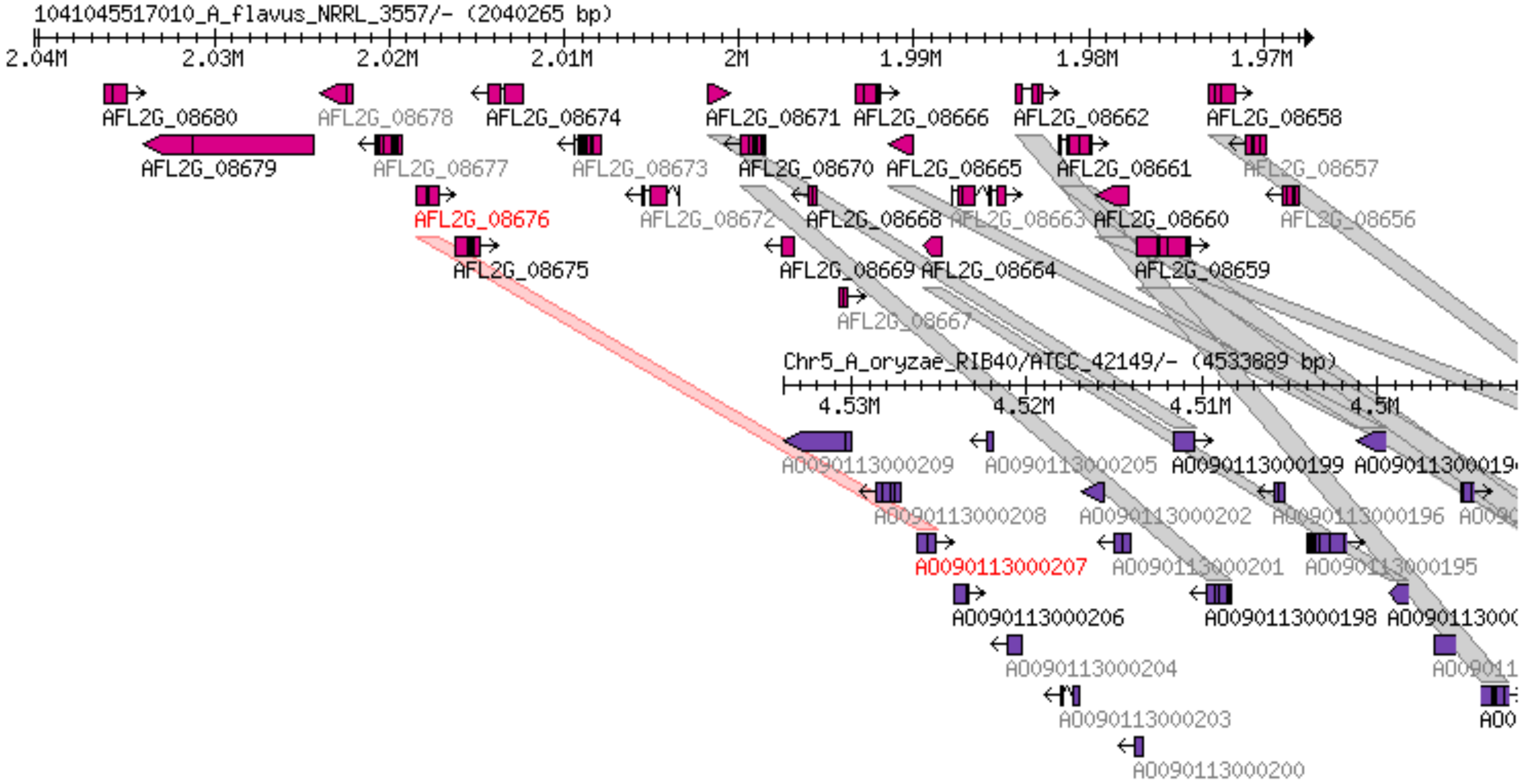

AO090113000209 cluster

Gene id (SMURF)	Gene positions	Gene distance	Gene Name	Annotated_gene_function	antiSMASH ID	AspGD ID
n/a				Domain(s) with predicted role in transmembrane transport and integral to membrane localization	prot_ID_11	AO090113000208
**AO090113000209**	0	0		Polyketide synthase	prot_ID_1441	AO090113000209
AO090113000208	1	1491		Domain(s) with predicted role in transmembrane transport and integral to membrane localization	prot_ID_11	AO090113000208
AO090113000207	2	941		Domain(s) with predicted electron carrier activity, heme binding, iron ion binding, oxidoreductase activity, acting on paired donors, with incorporation or reduction of molecular oxygen activity and role in oxidation-reduction process	prot_ID_793	AO090113000207
AO090113000206	3	945		Domain(s) with predicted monooxygenase activity and role in oxidation-reduction process	prot_ID_209	AO090113000206
AO090113000205	4	1040		Protein of unknown function	prot_ID_1016	AO090113000205
AO090113000204	5	820		Domain(s) with predicted sequence-specific DNA binding RNA polymerase II transcription factor activity, zinc ion binding activity, role in regulation of transcription, DNA-dependent and nucleus localization	prot_ID_1414	AO090113000204
n/a				Protein of unknown function	prot_ID_425	AO090113000203
n/a				Protein of unknown function	prot_ID_715	AO090113000202
n/a				Protein of unknown function	prot_ID_98	AO090113000201
n/a				Domain(s) with predicted ACP phosphopantetheine attachment site binding involved in fatty acid biosynthetic process activity	prot_ID_1002	AO090113000200
n/a				Ortholog(s) have hydrolase activity, acting on glycosyl bonds activity and role in carbohydrate catabolic process	prot_ID_251	AO090113000199

AO090120000024 cluster

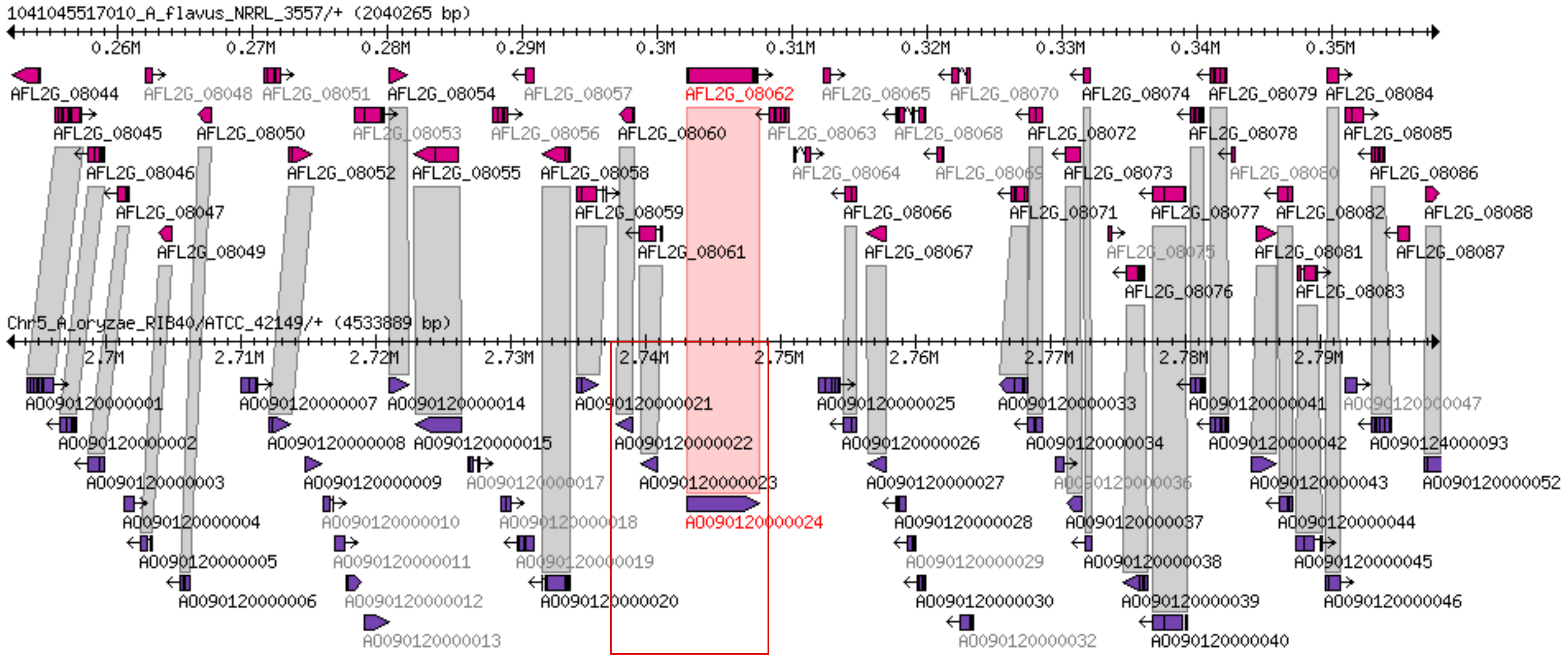

AO090120000024 cluster

Gene id (SMURF)	Gene positions	Gene distance	Gene Name	Annotated_gene_function	antiSMASH ID	AspGD ID	
n/a				Ortholog(s) have role in postreplication repair and Smc5-Smc6 complex, nucleus localization	prot_ID_773	AO090120000033	
n/a				Protein of unknown function	prot_ID_322	AO090120000032	
n/a				Domain(s) with predicted ATP binding, protein kinase activity and role in protein phosphorylation	prot_ID_432	AO090120000030	
n/a				Domain(s) with predicted ATP binding, protein kinase activity and role in protein phosphorylation	prot_ID_1366	AO090120000029	
n/a				Ortholog of A. fumigatus Af293 : Afu1g16350, A. niger CBS 513.88 : An12g03130, A. brasiliensis : Aspbr1_0058727, A. niger ATCC 1015 : 189755-mRNA and N. fischeri NRRL 181 : NFIA_009090	prot_ID_918	AO090120000028	
n/a				Ortholog of A. nidulans FGSC A4 : AN3302, A. niger CBS 513.88 : An01g01010, A. versicolor : Aspve1_0061419 and A. sydowii : Aspsy1_0043459	prot_ID_1191	AO090120000027	
n/a				Xylanase G2, major endo-1,4-beta-xylanase with role in xylan degradation; predicted secretion signal peptide; positively regulated by XlnR; 3 putative XlnR binding sites in promoter; produced in the submerged but not solid culture	prot_ID_95	AO090120000026	
n/a				Putative MFS transporter; positively regulated by XlnR; 1 putative XlnR binding site in the promoter	prot_ID_711	AO090120000025	
**AO090120000024**	0	0		Non-ribosomal peptide synthetase	prot_ID_566	AO090120000024	ECS
AO090120000023	1	2289		Ortholog(s) have dimethylallyltranstransferase activity, peptidase activity, tryptophan dimethylallyltransferase activity and role in ergot alkaloid biosynthetic process, protein prenylation, shamixanthone biosynthetic process	prot_ID_870	AO090120000023	
AO090120000022	2	673		Ortholog of A. nidulans FGSC A4 : AN0788, A. niger CBS 513.88 : An09g02430, An03g05960, An09g00710, An09g01710 and A. oryzae RIB40 : AO090003000250, AO090010000570, AO090023000882, AO090012000900	prot_ID_1248	AO090120000022	FA, ECS
AO090120000021	3	1443		Ortholog(s) have myo-inositol transmembrane transporter activity, role in myo-inositol transport and plasma membrane localization	prot_ID_378	AO090120000021	
AO090120000020	4	513		Domain(s) with predicted sequence-specific DNA binding RNA polymerase II transcription factor activity, zinc ion binding activity, role in regulation of transcription, DNA-dependent and nucleus localization	prot_ID_243	AO090120000020	
n/a				Protein of unknown function	prot_ID_1224	AO090120000019	
n/a				Domain(s) with predicted oxidoreductase activity and role in oxidation-reduction process	prot_ID_1100	AO090120000018	
n/a				Protein of unknown function	prot_ID_626	AO090120000017	
n/a				Ortholog of A. flavus NRRL 3357 : AFL2T_08055	prot_ID_65	AO090120000015	

n/a	Domain(s) with predicted oxidoreductase activity and role in oxidation-reduction process	prot_ID_295	AO090120000014
n/a	Protein of unknown function	prot_ID_926	AO090120000013

AO090124000040 cluster

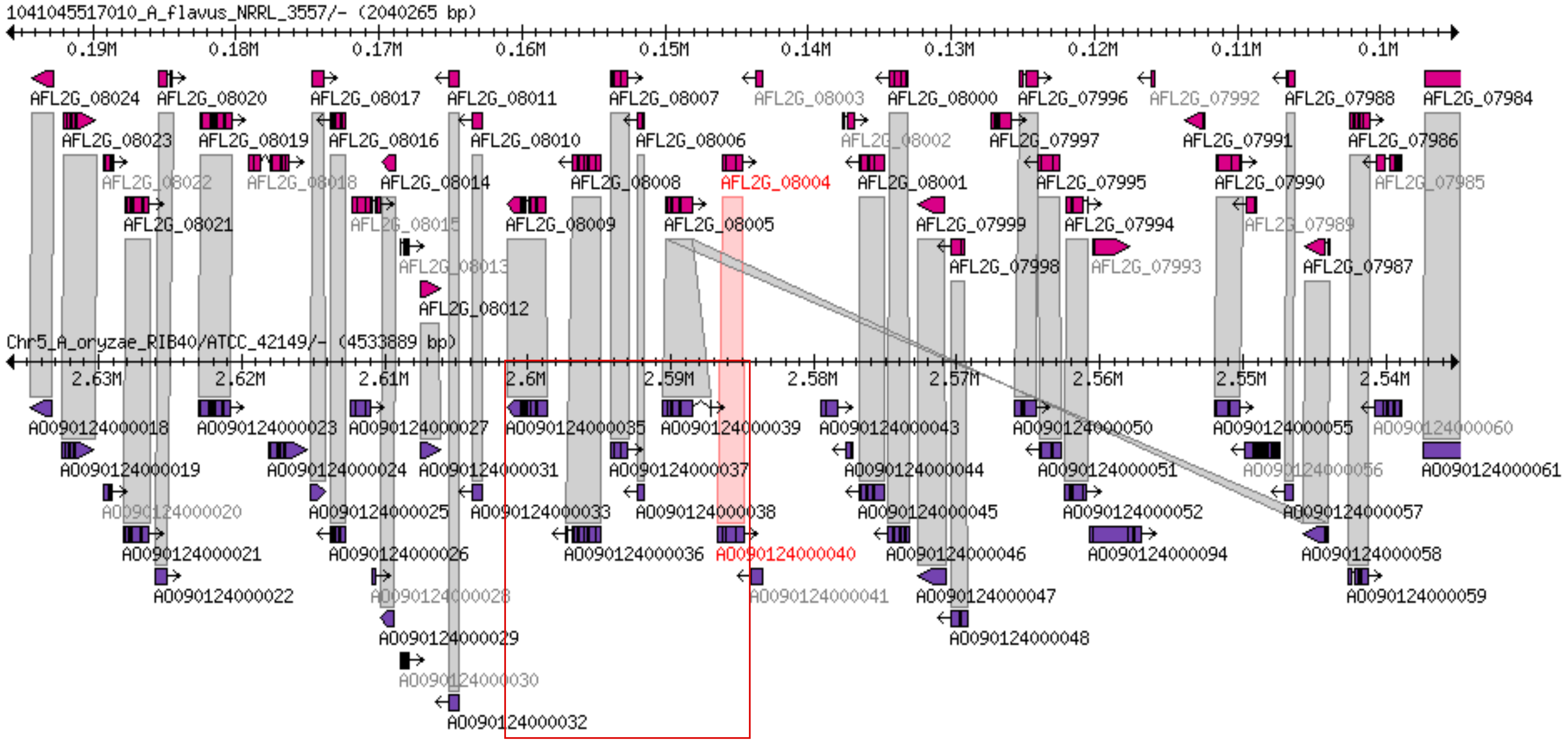

AO090124000040 cluster

Gene id (SMURF)	Gene positions	Gene distance	Gene Name	Annotated_gene_function	antiSMASH ID	AspGD ID	
n/a				Ortholog(s) have ferric-chelate reductase activity, role in cellular response to iron ion starvation, siderophore transport and plasma membrane localization	prot_ID_282	AO090124000035	ECS, FA
n/a				Ortholog(s) have ferroxidase activity, hydroquinone:oxygen oxidoreductase activity, iron ion transmembrane transporter activity	prot_ID_803	AO090124000036	
n/a				Ortholog(s) have iron ion transmembrane transporter activity, role in high-affinity iron ion transport, iron assimilation by reduction and transport and high affinity iron permease complex localization	prot_ID_28	AO090124000037	
n/a				Ortholog of A. flavus NRRL 3357 : AFL2T_08006	prot_ID_335	AO090124000038	
n/a				Domain(s) with predicted amino acid transmembrane transporter activity, role in amino acid transmembrane transport and membrane localization	prot_ID_1362	AO090124000039	
n/a				Type III polyketide synthase, involved in biosynthesis of 3,5-dihydroxybenzoic acid	prot_ID_482	AO090124000040	ECS
n/a				Protein of unknown function	prot_ID_1205	AO090124000041	
n/a				Ortholog of A. nidulans FGSC A4 : AN2922, AN1515, AN7536, A. niger CBS 513.88 : An12g10030, A. oryzae RIB40 : AO090701000305, AO090102000038 and A. niger ATCC 1015 : 55190-mRNA	prot_ID_1050	AO090124000043	
n/a				Ortholog of A. nidulans FGSC A4 : AN12089, A. niger CBS 513.88 : An11g02070, A. oryzae RIB40 : AO090005000192 and A. brasiliensis : Aspbr1_0032040, Aspbr1_0036822, Aspbr1_0439362	prot_ID_655	AO090124000044	
n/a				Ortholog of A. niger CBS 513.88 : An01g00290, A. versicolor : Aspve1_0025846, Aspve1_0086138 and A. sydowii : Aspsy1_0039665, Aspsy1_0046567, Aspsy1_0092019	prot_ID_1088	AO090124000045	
n/a				Has domain(s) with predicted phosphatase activity and role in metabolic process	prot_ID_791	AO090124000046	
n/a				Has domain(s) with predicted carboxy-lyase activity, magnesium ion binding, thiamine pyrophosphate binding activity	prot_ID_1298	AO090124000047	
n/a				Ortholog of A. fumigatus Af293 : Afu3g03950, N. fischeri NRRL 181 : NFIA_006240, Aspergillus flavus NRRL 3357 : AFL2T_07998, A. clavatus NRRL 1 : ACLA_060790 and Aspergillus terreus NIH2624 : ATET_02877	prot_ID_1010	AO090124000048	

AO090206000074 cluster

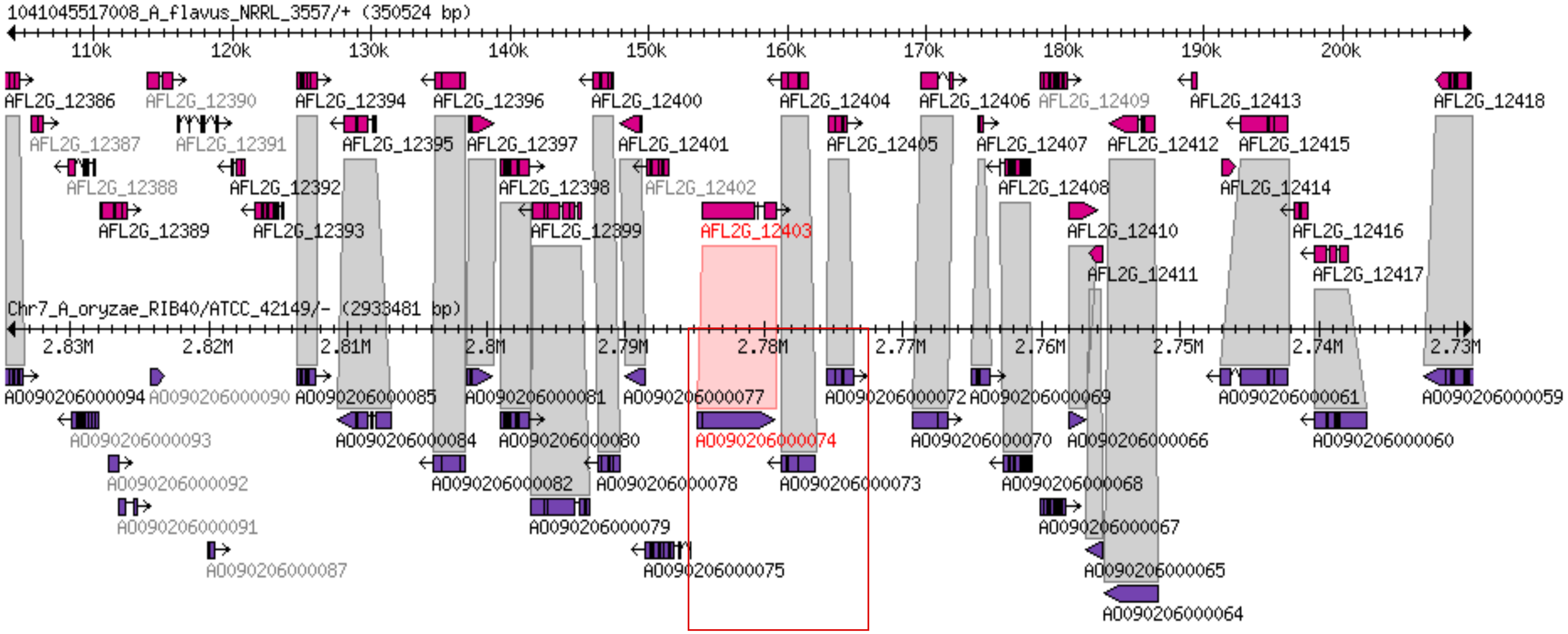

AO090206000074 cluster

Gene id (SMURF)	Gene positions	Gene distance	Gene Name	Annotated_gene_function	antiSMASH ID	AspGD ID	
n/a				Ortholog(s) have role in mRNA splicing, via spliceosome and nucleus, transcriptionally active chromatin localization	prot_ID_727	AO090206000082	
n/a				Ortholog(s) have ubiquitin binding activity, role in negative regulation of transcription from RNA polymerase II promoter by glucose, protein retention in Golgi apparatus, protein targeting to vacuole and ESCRT II complex localization	prot_ID_417	AO090206000081	
n/a				Domain(s) with predicted carbon-nitrogen ligase activity, with glutamine as amido-N-donor, hydrolase activity, acting on carbon-nitrogen (but not peptide) bonds, in linear amides activity	prot_ID_59	AO090206000080	
n/a				Ortholog(s) have chitin synthase activity	prot_ID_619	AO090206000079	
n/a				Ortholog of A. nidulans FGSC A4 : AN7033, A. fumigatus Af293 : Afu4g04190, A. niger CBS 513.88 : An14g00640, A. niger ATCC 1015 : 201359-mRNA and A. versicolor : Aspve1_0075847	prot_ID_234	AO090206000078	
n/a				Domain(s) with predicted role in phosphatidylinositol phosphorylation	prot_ID_775	AO090206000077	
AO090206000075	-1	829		Cytochrome P450 monooxygenase	prot_ID_873	AO090206000075	
**AO090206000074**	0	0		Polyketide synthase	prot_ID_21	AO090206000074	ECS, IGD
n/a				Putative protease	prot_ID_564	AO090206000073	
n/a				Ortholog of A. nidulans FGSC A4 : AN7025, A. fumigatus Af293 : Afu4g04220, A. niger ATCC 1015 : 41507-mRNA, A. versicolor : Aspve1_0088114 and A. sydowii : Aspsy1_0060635	prot_ID_155	AO090206000072	ECS, IGD
n/a				Ortholog of A. nidulans FGSC A4 : AN7026, A. fumigatus Af293 : Afu4g04230, A. niger CBS 513.88 : An14g00600, A. niger ATCC 1015 : 41506-mRNA and A. versicolor : Aspve1_0140914	prot_ID_770	AO090206000070	
n/a				Ortholog of A. nidulans FGSC A4 : AN11541, A. versicolor : Aspve1_0140562, A. sydowii : Aspsy1_0158387 and A. terreus NIH2624 : ATET_10112	prot_ID_474	AO090206000069	
n/a				Domain(s) with predicted role in transmembrane transport and integral to membrane localization	prot_ID_596	AO090206000068	
n/a				Domain(s) with predicted queuine tRNA-ribosyltransferase activity and role in queuosine biosynthetic process	prot_ID_201	AO090206000067	

AO090701000530 cluster

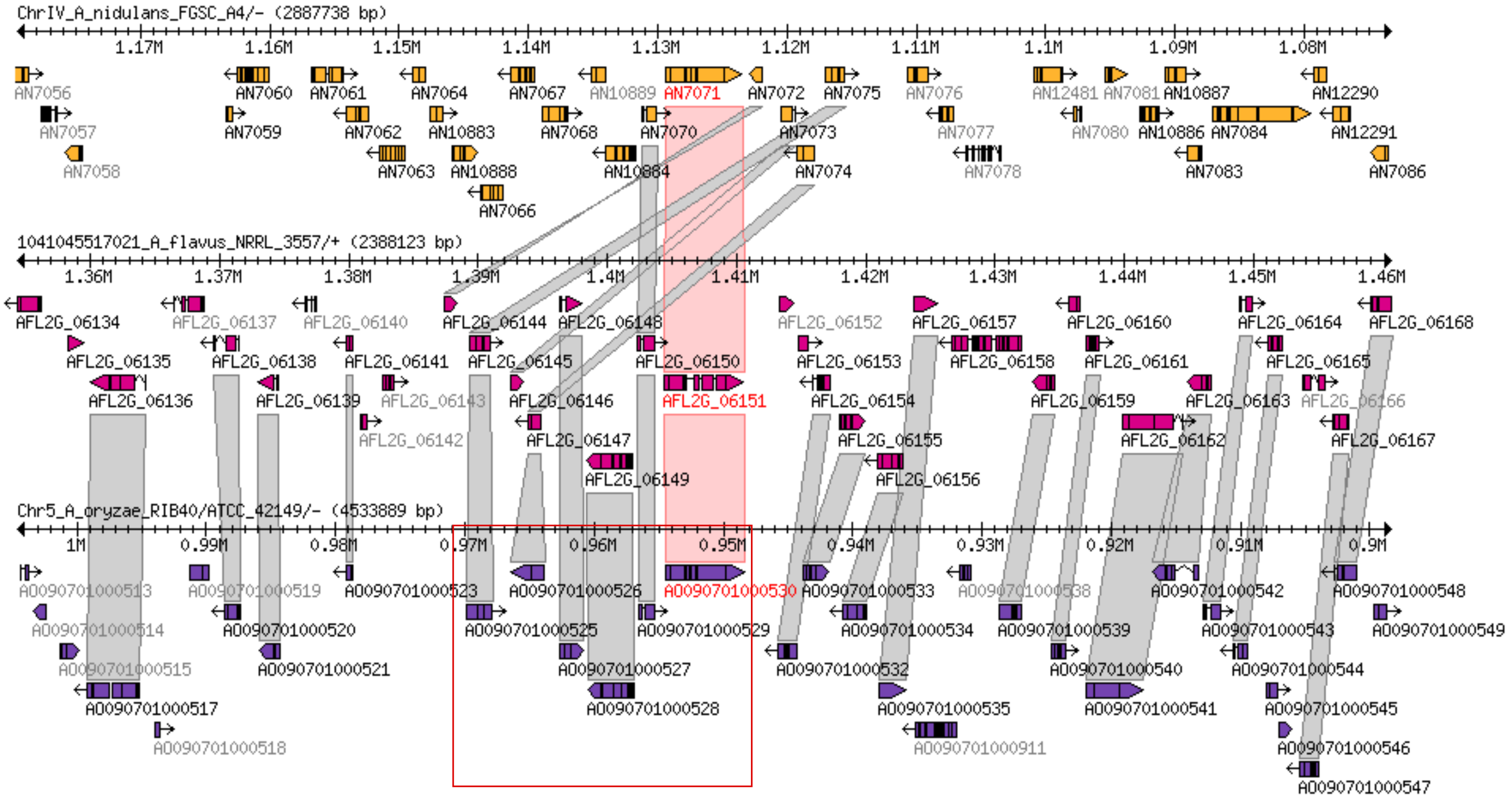

AO090701000530 cluster

Gene id (SMURF)	Gene positions	Gene distance	Gene Name	Annotated_gene_function	antiSMASH ID	AspGD ID	
AO090701000525	-5	2955		Domain(s) with predicted UDP-N-acetylmuramate dehydrogenase activity, flavin adenine dinucleotide binding activity and role in oxidation-reduction process	prot_ID_845	AO090701000525	ECS, IGD
AO090701000526	-4	1489		Domain(s) with predicted nucleotide binding, oxidoreductase activity and role in oxidation-reduction process	prot_ID_644	AO090701000526	
AO090701000527	-3	473		Domain(s) with predicted choline dehydrogenase activity, flavin adenine dinucleotide binding activity and role in alcohol metabolic process, oxidation-reduction process	prot_ID_1070	AO090701000527	
AO090701000528	-2	424		Domain(s) with predicted O-methyltransferase activity, monooxygenase activity and role in oxidation-reduction process	prot_ID_238	AO090701000528	
AO090701000529	-1	776		Ortholog(s) have role in asperthecin biosynthetic process, monodictyphenone biosynthetic process	prot_ID_524	AO090701000529	
**AO090701000530**	0	0		Polyketide synthase	prot_ID_692	AO090701000530	ECS, IGD
AO090701000532	1	2686		Ortholog of A. nidulans FGSC A4 : AN3303, AN3348, AN2044, AN8727, A. fumigatus Af293 : Afu4g10080, Afu7g00420, Afu4g01242 and A. niger CBS 513.88 : An02g09530, An03g00420, An16g01900, An16g06080, An04g06980, An03g00215	prot_ID_474	AO090701000532	
AO090701000533	2	692		Domain(s) with predicted amino acid transmembrane transporter activity, role in amino acid transmembrane transport and integral to membrane localization	prot_ID_1325	AO090701000533	
AO090701000534	3	1389		Domain(s) with predicted role in transmembrane transport and integral to membrane localization	prot_ID_669	AO090701000534	
AO090701000535	4	926		Ortholog of N. fischeri NRRL 181 : NFIA_047410 and A. flavus NRRL 3357 : AFL2T_06157	prot_ID_1174	AO090701000535	
AO090701000911	5	1078		Domain(s) with predicted ATP binding, ATPase activity, coupled to transmembrane movement of substances activity, role in transmembrane transport and integral to membrane localization	prot_ID_1462	AO090701000911	
AO090701000538	6	271		Domain(s) with predicted ATP binding, ATPase activity, coupled to transmembrane movement of substances activity, role in transmembrane transport and integral to membrane localization	prot_ID_1085	AO090701000538	
AO090701000539	7	2108		Ortholog of A. flavus NRRL 3357 : AFL2T_06159	prot_ID_127	AO090701000539	
AO090701000540	8	2328		Ortholog of A. niger CBS 513.88 : An01g05270, A. brasiliensis : Aspbr1_0052930, A. flavus NRRL 3357 : AFL2T_06161 and Neosartorya fischeri NRRL 181 : NFIA_057820	n/a	AO090701000540	
AO090701000541	9	2041		Ortholog of A. fumigatus Af293 : Afu7g00550, A. niger CBS 513.88 : An15g05630, An11g03460, An04g03850, An09g00640, A. oryzae RIB40 : AO090038000448 and A. niger ATCC 1015 : 118837-mRNA	n/a	AO090701000541	

AO090701000542	10	936	Ortholog of A. nidulans FGSC A4 : AN1433, A. fumigatus Af293 : Afu1g01310, Afu8g04210, A. niger CBS 513.88 : An16g08870, An08g11860, An13g01880 and A. oryzae RIB40 : AO090103000036	n/a	AO090701000542
AO090701000543	11	695	60S ribosomal protein L26; predominantly expressed in the hyphal tip region	n/a	AO090701000543

AO090701000600 cluster

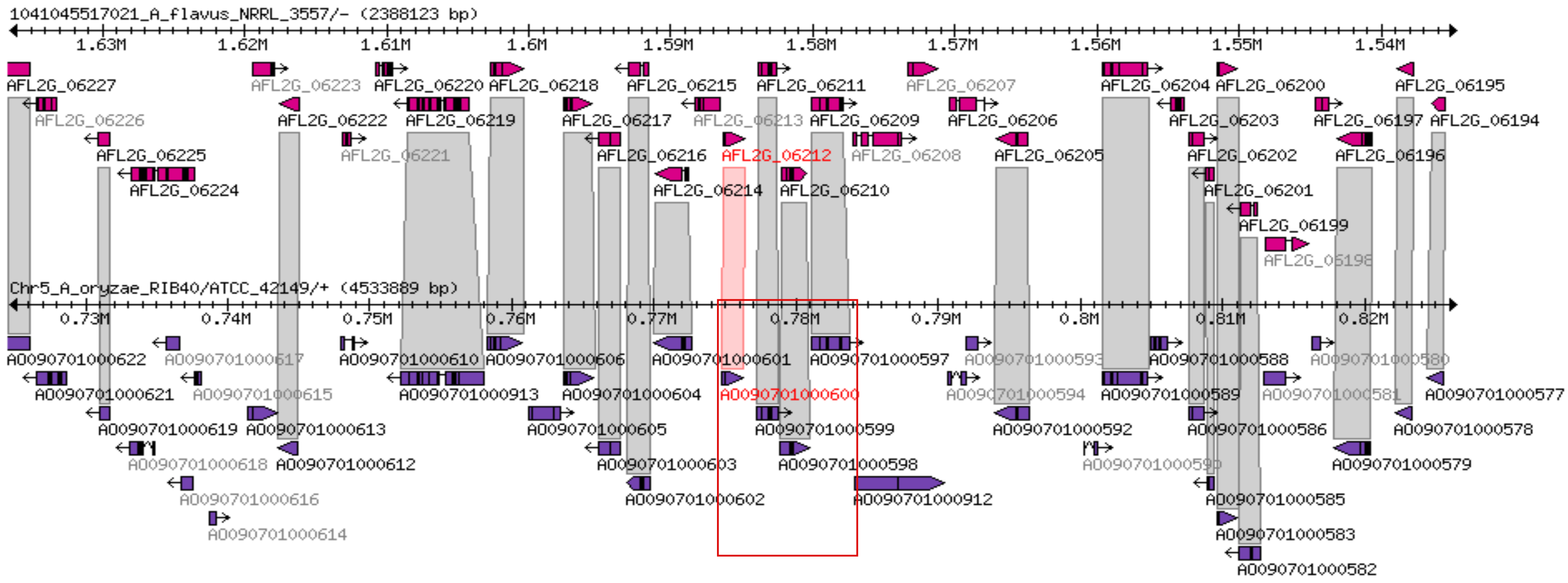

AO090701000600 cluster

Gene id (SMURF)	Gene positions	Gene distance	Gene Name	Annotated_gene_function	antiSMASH ID	AspGD ID	
n/a				Ortholog of A. versicolor : Aspve1_0889539, A. sydowii : Aspsy1_0033360 and A. terreus NIH2624 : ATET_06888	prot_ID_1465	AO090701000912	
n/a				Ortholog(s) have glutamine-tRNA ligase activity, role in glutaminyl-tRNA aminoacylation and cytosol, mitochondrion localization	prot_ID_964	AO090701000597	ECS
n/a				Domain(s) with predicted catalytic activity and role in metabolic process	prot_ID_1282	AO090701000598	
n/a				Ortholog of A. nidulans FGSC A4 : AN6413, AN8328, AN3395, AN3241, AN10369, AN1930, AN2386, AN1738, AN9266, AN10886, AN7232, AN7406, AN7774, AN12202	prot_ID_762	AO090701000599	
**AO090701000600**	0	0		Dimethylallyl tryptophan synthase (DMAT)	prot_ID_1351	AO090701000600	ECS, IGD
AO090701000601	1	2498		Cytochrome P450 monooxygenase; predicted phenylalanine ammonia-lyase	prot_ID_529	AO090701000601	
AO090701000602	2	486		Ortholog of A. nidulans FGSC A4 : AN8025, A. fumigatus Af293 : Afu1g01450, A. niger CBS 513.88 : An11g08050, An05g01890, An11g03950 and A. oryzae RIB40 : AO090010000064	prot_ID_879	AO090701000602	
AO090701000603	3	648		Domain(s) with predicted UDP-N-acetylmuramate dehydrogenase activity, flavin adenine dinucleotide binding activity and role in oxidation-reduction process	prot_ID_1355	AO090701000603	
n/a				ESCRT-I complex component	prot_ID_1044	AO090701000604	

AO090701000826 cluster

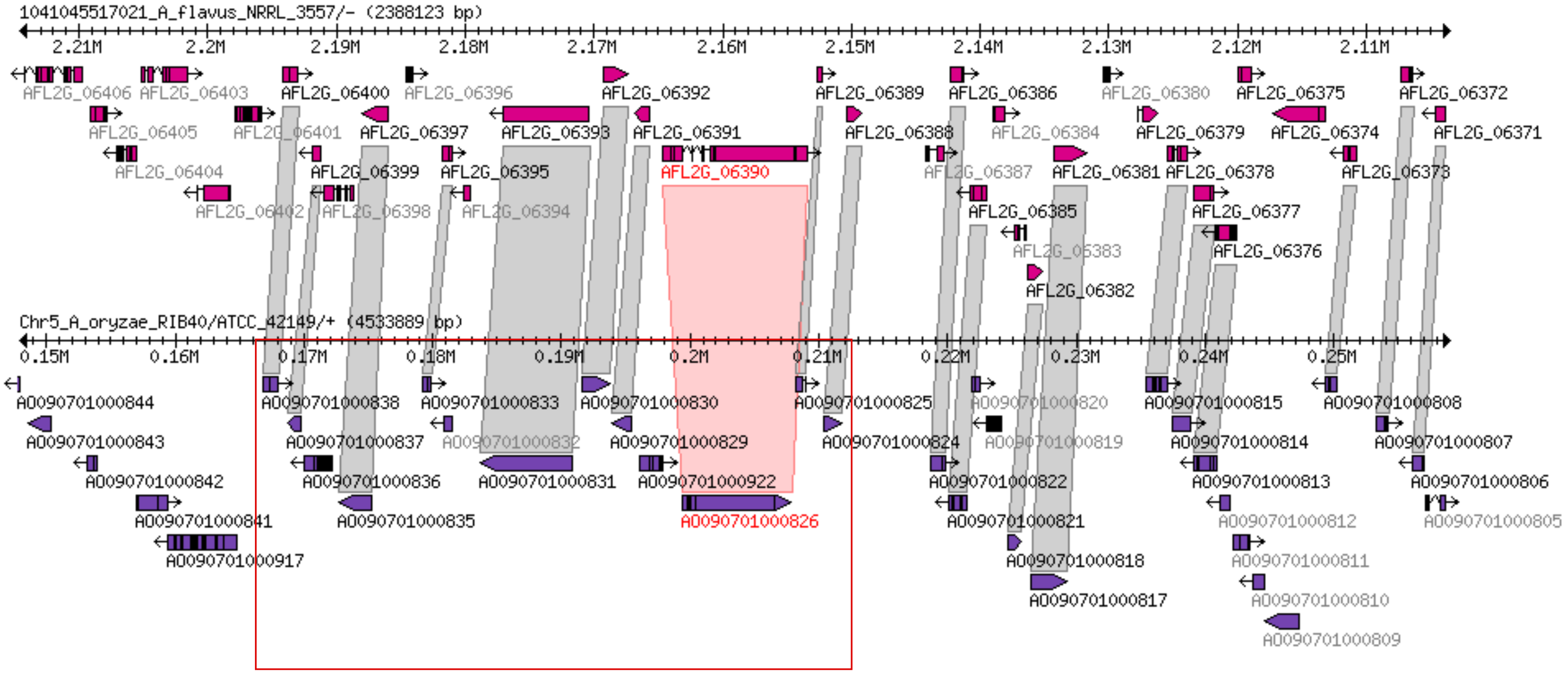

AO090701000826 cluster

Gene id (SMURF)	Gene positions	Gene distance	Gene Name	Annotated_gene_function	antiSMASH ID	AspGD ID	
n/a				Ortholog of A. fumigatus Af293 : Afu1g00220, A. niger CBS 513.88 : An08g08350, A. oryzae RIB40 : AO090138000179 and A. brasiliensis : Aspbr1_0027476, Aspbr1_0502994	prot_ID_1004	AO090701000817	
n/a				Domain(s) with predicted nucleotide binding, oxidoreductase activity, zinc ion binding activity and role in oxidation-reduction process	prot_ID_1022	AO090701000818	
n/a				Domain(s) with predicted oxidoreductase activity and role in oxidation-reduction process	prot_ID_1	AO090701000819	
n/a				Protein of unknown function	prot_ID_1250	AO090701000820	
n/a				domain(s) with predicted nucleotide binding, oxidoreductase activity, transferase activity, transferring acyl groups other than amino-acyl groups, zinc ion binding activity and role in oxidation-reduction process	prot_ID_162	AO090701000821	
n/a				Domain(s) with predicted nucleotide binding, oxidoreductase activity, zinc ion binding activity and role in oxidation-reduction process	prot_ID_1415	AO090701000822	
n/a				Domain(s) with predicted oxidoreductase activity, sequence-specific DNA binding transcription factor activity, role in oxidation-reduction process, regulation of transcription, DNA-dependent and intracellular localization	prot_ID_556	AO090701000824	ECS, IGD
n/a				Ortholog of A. flavus NRRL 3357 : AFL2T_06389	prot_ID_1377	AO090701000825	
**AO090701000826**	0	0		Polyketide synthase; ortholog(s) have role in sterigmatocystin biosynthesis; positively regulated by XlnR; 1 putative XlnR binding site in the promoter	prot_ID_1053	AO090701000826	
AO090701000922	1	1604		Protein of unknown function; positively regulated by XlnR; 6 putative XlnR binding sites in the promoter; ortholog(s) have role in secondary metabolism	prot_ID_1448	AO090701000828.1	
AO090701000829	2	1139		Ortholog(s) have role in asperfuranone biosynthetic process	prot_ID_1066	AO090701000829	
AO090701000830	3	960		Domain(s) with predicted heme binding, iron ion binding, oxidoreductase activity, acting on paired donors, with oxidation of a pair of donors resulting in the reduction of molecular oxygen to two molecules of water activity	prot_ID_308	AO090701000830	
AO090701000831	4	1086		Ortholog(s) have role in asperfuranone biosynthetic process, violaceol I biosynthetic process, violaceol II biosynthetic process	prot_ID_978	AO090701000831	
AO090701000832	5	2392		Protein of unknown function	prot_ID_136	AO090701000832	
AO090701000833	6	796		Domain(s) with predicted nucleotide binding, oxidoreductase activity and role in oxidation-reduction process	prot_ID_745	AO090701000833	
n/a				Domain(s) with predicted transferase activity, transferring acyl groups activity and role in metabolic process	prot_ID_1280	AO090701000835	
n/a				Domain(s) with predicted amino acid transmembrane transporter activity, role in amino acid transmembrane transport and membrane localization	prot_ID_431	AO090701000836	
n/a				Domain(s) with predicted carbon-sulfur lyase activity and role in metabolic	prot_ID_1444	AO090701000837	

	process			
n/a	Domain(s) with predicted hydrolase activity, hydrolyzing O-glycosyl compounds activity and role in carbohydrate metabolic process	prot_ID_936	AO090701000838	ECS
n/a	Domain(s) with predicted ATP binding, ATPase activity, coupled to transmembrane movement of substances activity, role in transmembrane transport and integral to membrane localization	prot_ID_1454	AO090701000917	

Non-PKS/NRPS backbone 1

No manual prediction made

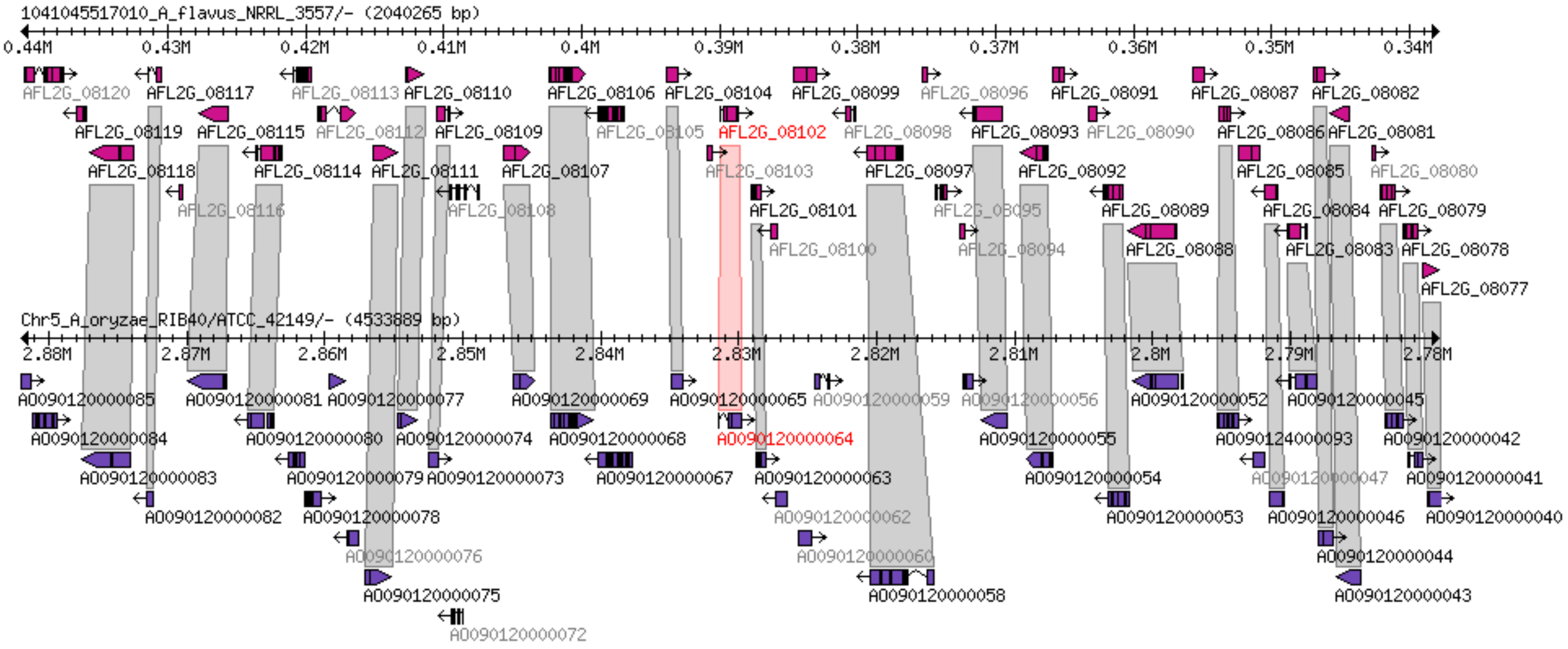

Non-PKS/NRPS cluster 1

Gene id (SMURF)	Gene positions	Gene distance	Gene Name	Annotated_gene_function	antiSMASH ID	AspGD ID
n/a				Domain(s) with predicted catalytic activity	prot_ID_814	AO090120000058
n/a				Protein of unknown function	prot_ID_102	AO090120000059
n/a				Protein of unknown function	prot_ID_712	AO090120000060
n/a				Protein of unknown function	prot_ID_908	AO090120000062
n/a				Domain(s) with predicted electron carrier activity, heme binding, iron ion binding, oxidoreductase activity, acting on paired donors, with incorporation or reduction of molecular oxygen activity and role in oxidation-reduction process	prot_ID_1080	AO090120000063
n/a				Geranylgeranyl pyrophosphate synthase	prot_ID_1164	AO090120000064
n/a				Domain(s) with predicted ATP binding, transferase activity, transferring phosphorus-containing groups activity	prot_ID_336	AO090120000065
n/a			CYP551A2	Cytochrome P450 monooxygenase	prot_ID_1281	AO090120000067
n/a			catB	Catalase with a predicted role in hydrogen peroxide detoxification; expression is induced under hydrogen peroxide treatment; putative heat shock element and a CCAAT-box is involved in high levels of expression	prot_ID_627	AO090120000068

Non-PKS/NRPS backbone 2

No manual predicton made

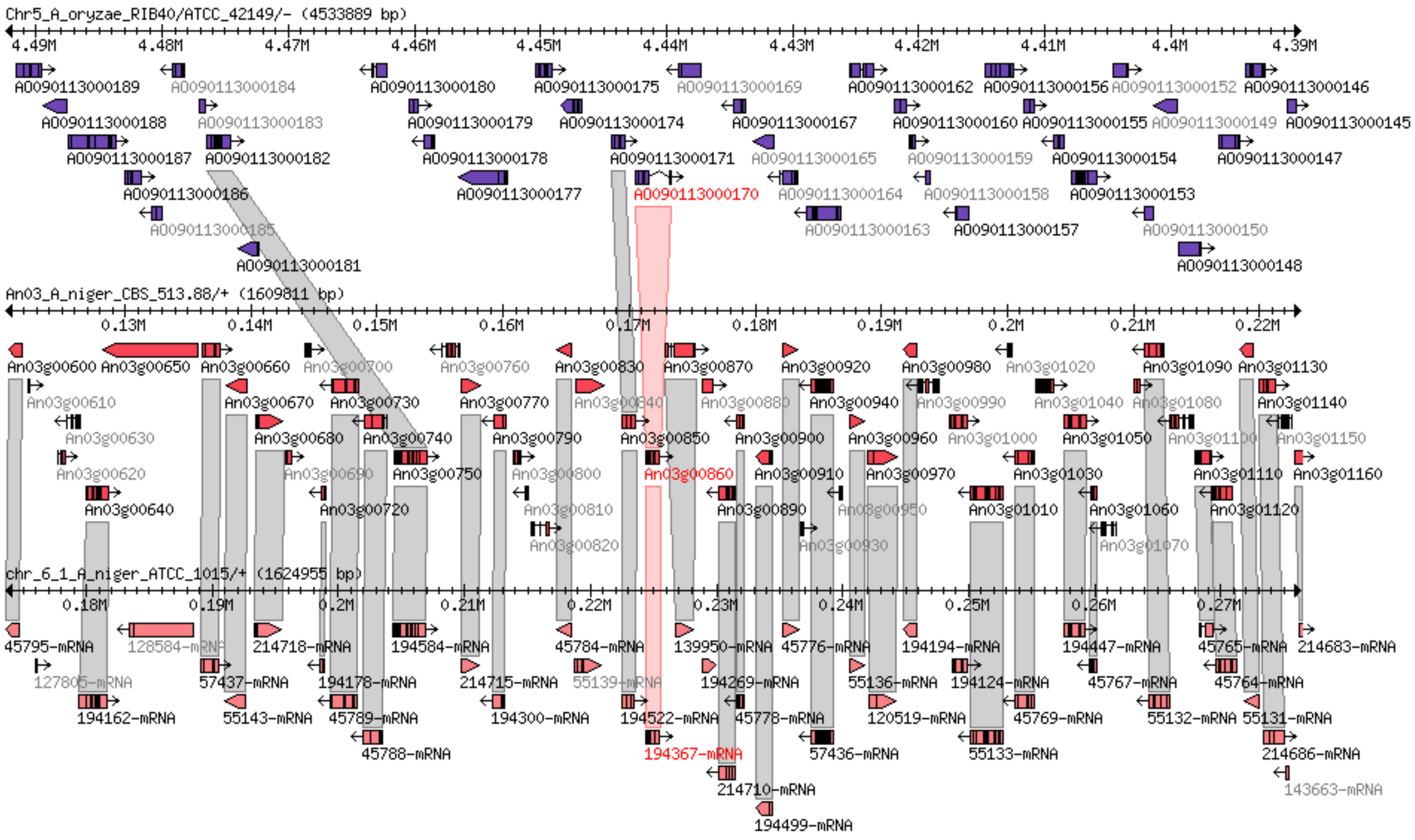

Non-PKS/NRPS cluster 2

Gene id (SMURF)	Gene positions	Gene distance	Gene Name	Annotated_gene_function	antiSMASH ID	AspGD ID
n/a				Domain(s) with predicted sequence-specific DNA binding RNA polymerase II transcription factor activity, zinc ion binding activity, role in regulation of transcription, DNA-dependent and nucleus localization	prot_ID_294	AO090113000164
n/a				Protein of unknown function	prot_ID_927	AO090113000165
n/a				Domain(s) with predicted nucleotide binding activity	prot_ID_765	AO090113000167
n/a				Domain(s) with predicted DNA binding, zinc ion binding activity, role in transcription, DNA-dependent and nucleus localization	prot_ID_403	AO090113000169
n/a				Domain(s) with predicted role in isoprenoid biosynthetic process	prot_ID_408	AO090113000170
n/a				Domain(s) with predicted role in isoprenoid biosynthetic process	prot_ID_246	AO090113000171
n/a				Ortholog(s) have role in austinol biosynthetic process, dehydroaustinol biosynthetic process	prot_ID_853	AO090113000174
n/a				Ortholog of A. nidulans FGSC A4 : AN8943, A. fumigatus Af293 : Afu2g00900, Afu6g11560, A. niger CBS 513.88 : An01g11690, An05g02420, An03g01300 and A. oryzae RIB40 : AO090023000422	prot_ID_45	AO090113000175

Abbreviations: ECS, end of cluster synteny; IGD, increase in intergenic distance; FA, change in functional annotation; ED, experimentally determined
